# Computational discovery of Epstein-Barr virus targeted human genes and signalling pathways

**DOI:** 10.1038/srep30612

**Published:** 2016-07-29

**Authors:** Suyu Mei, Kun Zhang

**Affiliations:** 1 Software College, Shenyang Normal University, Shenyang, 110034, China; 2 Department of Computer Science, Xavier University of Louisiana, New Orleans, LA 70125, USA

## Abstract

Epstein-Barr virus (EBV) plays important roles in the origin and the progression of human carcinomas, e.g. diffuse large B cell tumors, T cell lymphomas, etc. Discovering EBV targeted human genes and signaling pathways is vital to understand EBV tumorigenesis. In this study we propose a noise-tolerant homolog knowledge transfer method to reconstruct functional protein-protein interactions (PPI) networks between Epstein-Barr virus and Homo sapiens. The training set is augmented via homolog instances and the homolog noise is counteracted by support vector machine (SVM). Additionally we propose two methods to define subcellular co-localization (i.e. stringent and relaxed), based on which to further derive physical PPI networks. Computational results show that the proposed method achieves sound performance of cross validation and independent test. In the space of 648,672 EBV-human protein pairs, we obtain 51,485 functional interactions (7.94%), 869 stringent physical PPIs and 46,050 relaxed physical PPIs. Fifty-eight evidences are found from the latest database and recent literature to validate the model. This study reveals that Epstein-Barr virus interferes with normal human cell life, such as cholesterol homeostasis, blood coagulation, EGFR binding, p53 binding, Notch signaling, Hedgehog signaling, etc. The proteome-wide predictions are provided in the supplementary file for further biomedical research.

Virus-host interaction helps virus to hijack host cellular processes for survival and replication within its host. Through interactions with host proteins, virus perturbs and interrupts host signalling pathways to alter key cellular functions^1^. Rapid computational discovery of virus targeted human genes and signaling pathways is of significance to reveal viral pathogenesis and find druggable targets. At present, the majority of computational methods focus on human immunodeficiency virus type 1 (HIV-1)^1^,^2^,^3^,^4^,^5^,^6^,^7^,^8^,^9^, wherein^1^ focuses on predicting activation/inhibition signals and^2^,^3^,^4^,^5^,^6^,^7^,^8^,^9^ focus on prediction protein-protein interactions (PPI) between HIV-1 and human. The reason that HIV-1 is chosen for computational modeling is that HIV-1 is a well-understood virus with the largest experimental virus-host PPI networks. Mei 7 derived 3,638 PPIs as positive training data from HIV-1 database (http://www.ncbi.nlm.nih.gov/projects/RefSeq/HIVInteractions/). Nevertheless, the data size is still much smaller than intra-species PPI network size^10^,^11^,^12^, partly because of small viral genome. Small data poses more challenges from point of view of computational modeling. Among the known viruses, HIV-1 possesses the largest experimental virus-host PPI networks to our knowledge. For the other viruses that possess much smaller experimental virus-host PPI networks, we need to explicitly address special concerns such as augmentation of training data to reduce the risk of model overfitting. To our knowledge, Epstein-Barr virus (EBV) is also a well-studied virus with the second largest experimental virus-host PPI networks after HIV-1, so EBV will be next in line as a model organism for computational modeling.

Epstein-Barr virus (EBV) is the first known human tumor virus that acts as the causative agent of infectious mononucleosis, and plays important roles in the origin or progression of B cell malignancies, e.g. Hodgkinlymphoma, diverse AIDS-associated lymphomas. Nowadays Epstein-Barr virus is also viewed as epithelial tumor virus as well as lymphotropic virus^13^. At present, only 173 EBV-human PPIs are reported in^14^, much smaller than 3,638 HIV-human PPIs. Such a small data puts more challenges on computational modeling. The experimental PPI networks between Epstein-Barr virus and Homo sapiens reveal a limited number of human target genes and signaling pathways. For instances, the interaction of Nur77 with EBNA2 localizes Nur77 to the nucleus and protects cells from Nur77-mediated apoptosis; EBNA3A interaction with RPL4 also regulates programmed cell death; EBV LMP1 is found to interact with TRAF1 protein to link LMP1-mediated B lymphocyte transformation to the signal transduction from TNFR family receptors; and EBNA2 is found to target two signaling pathways that modulate intracellular Ca^2+^ ion levels, etc. This experimental PPI networks can be treated as a reliable training data for computational modeling.

To our knowledge, no computational method has to date been proposed for EBV-human PPI networks reconstruction. The existing computational methods for HIV-human PPI prediction generally focus on integrating multiple feature information (e.g. gene ontology, sequence *k*-mer, gene co-expression, protein structural information, etc.) to improve predictive performance^2^,^3^,^4^,^5^,^6^,^7^,^8^,^9^. Multi-task learning is a sophisticated framework to integrate multiple sources of feature information via parameter optimization^3^,^8^. Data integration is useful to enrich feature information, but meanwhile imposes demanding data constraints on computational model. Once the required feature information for prediction (e.g. gene ontology, structural information) is not available, the trained model cannot work. Mei^7^ introduced homolog knowledge via ensemble learning framework to address this problem. These methods work properly on the moderately-sized HIV-1 data (>3000 PPIs). For extremely small virus-host PPI networks, we need further develop explicit data augmentation methods to reduce the risk of model overfitting.

In this work we aim at discovering Epstein-Barr virus targeted human genes and signaling pathways. In view of the small experimental EBV-human PPI networks, we propose a noise-tolerant homolog knowledge transfer method to explicitly augment the training data. Unlike the probability weighted ensemble learning method that treats homolog knowledge as independent views^7^, we treat homolog knowledge as independent instances, so that the training data are double-sized and the feature information is enriched. However, homolog instances may carry noise from evolutionary divergence. Here we implement homolog knowledge transfer under the learning framework of support vector machine (SVM). SVM is well known for its resistance against noise/outlier via theoretically-sound regularization technique^15^. By conducting GO (gene ontology) enrichment analysis and pathway enrichment analysis, we can easily infer how Epstein-Barr virus interferes with human signaling pathways.

## Data and Methods

### Data and materials

**Figure 1:**
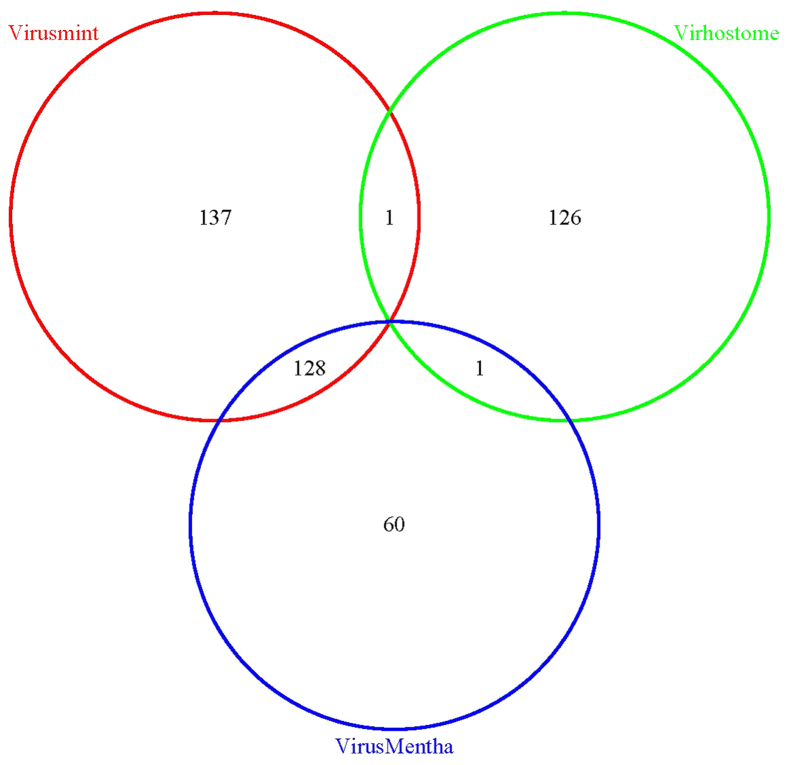
Venn Diagram of data distribution and intersection between Virusmint, Virhostome and VirusMentha.

The experimental PPI networks between Epstein-Barr virus and Homo sapiens are collected from three virus-host PPI databases: VirusMINT^16^ (http://mint.bio.uniroma2.it/virusmint/Welcome.do); Virhostome^17^ (http://interactome.dfci.harvard.edu/V_hostome/index.php); VirusMentha^18^ (http://virusmentha.uniroma2.it/). We remove those obsolete and uncurated proteins by checking against the Uniprot database (http://www.uniprot.org/uniprot/). Those proteins that have no gene names are also removed. As a result, VirusMINT contains 266 PPIs, Virhostome contains 128 PPIs and VirusMentha contains 189 PPIs. The data distribution and intersection between the three data sets are illustrated in Fig. 1. We can see that Virhostome has very small intersections with the other two data sets. Here we use VirusMINT as preliminary training set and use Virhostome as preliminary independent test set to conduct preliminary study. To ensure that the independent test set has no intersection with the training set, we remove from Virhostome those PPIs that are contained in VirusMINT. Furthermore, we remove Virhostome those PPIs whose EBV proteins do not occur in VirusMINT in that the training data do not contain any information about these EBV proteins. Thus the final Virhostome contains 84 interactions. In the end, we further combine VirusMINT and Virhostome to obtain the final training data (denoted as VirusMINT + Virhostome) that contains 350 interactions. Accordingly, we use VirusMentha as the final validation set. Similarly we also remove from VirusMentha those PPIs that are contained in VirusMINT + Virhostome. Thus the final VirusMentha contains 60 PPIs.

The above data are viewed as positive examples. To train a two-class predictive model, we randomly sample the negative examples in the EBV-human protein pair space exclusive of the positive examples. To date how to determine the sampling ratio of negative examples is a controversial issue in computational biology^2^,^3^,^4^,^7^,^8^. In some work, equal size of negative examples is adopted^7^,^12^, while some other work adopts multiple folds of negative examples^3^,^4^. Here we are inclined to adopt 1:1 ratio of negative examples to positive examples for the following reasons: (1) from computation points of view, large ratio of negative examples to positive examples is prone to yield a highly negative class biased model that can hardly recognize true protein-protein interactions; (2) for very small positive training examples, large ratio of negative examples to positive examples could make things much worse, because the limited information contained in positive examples would be overwhelmed by the huge negative examples or even could be neglected; (3) the existing methods that adopt large ratio of negative examples to positive examples seldom provide the bias measure including precision, sensitivity and Matthews correlation coefficient for the small positive class. In the extreme case of highly unbalanced training data, the performance metric accuracy is misleading; (4) we do not know the true ratio of negative examples to positive examples in real world. Actually it is hard to find a direct and interpretable mapping between biological problem and computational problem.

### Multi-instance feature construction

Gene ontology (GO)^19^ has been frequently used to predict protein-protein interactions^2^,^3^,^7^,^8^,^10^,^11^ and is claimed as the most discriminative feature in ref. 20. Nevertheless, the majority of genes/proteins are sparsely annotated with GO terms. In most cases the sparse GO feature vector could only provide very limited information. In some extreme cases that the gene/protein concerned is not annotated at all, the GO feature vector would be null vector. To reduce the risk of null vector and enrich feature information, we depict a gene/protein with two instances, namely target instance and homolog instance. The target instance represents the GO knowledge of the gene/protein itself, while the homolog instance represents the GO knowledge of the homologs. As such, the homolog instance not only enriches the feature information of the target instance but also substitutes the target instance when the gene/protein is not annotated. We extract the homologs from SwissProt^21^ using PSI-BLast^22^ (E-value = 10) against all species. The GO terms are retrieved from GOA^19^. Using *U* to denote the training data, we obtain two sets of GO terms for each protein *i*. One set contains the GO terms of the homologs (denoted as 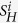), and the other set contains the GO terms of the protein itself (denoted as 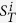). Accordingly, the entire set of GO terms of the training data *U* (denoted as *S*) is defined as follows.


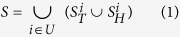


Based on these notations, we formally define the two feature vectors for a protein pair (*i*_1_, *i*_2_) as follows.


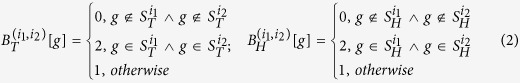


For each GO term *g* ∈ *S*, 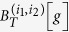 denotes component *g* of the target instance 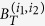 and 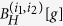 denotes component *g* of the homolog instance 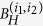. In practical programming implementation, GO term *g* is assigned an integer index. Those GO terms that satisfy *g* *∉* *S* are discarded. Formula (2) indicates that if the protein pair (*i*_1_, *i*_2_) shares the same *GO* term *g*, the corresponding component value in the feature vector 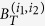 or 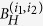 is 2; if neither protein in the protein pair possesses the *GO* term *g,* the value is 0; otherwise, the value is 1. The above definition is symmetrical, i.e., the protein pair (*i*_1_, *i*_2_) and the protein pair (*i*_2_, *i*_1_) have identical feature representation.

### Noise-tolerant homolog knowledge transfer

Homolog knowledge transfer is conducted via homolog instance to serve the purposes of (1) enrichment of the feature information of target instance; (2) substitution for the target instance when the gene/protein is not annotated; (3) augmentation of the training data to reduce the risk of model overfitting. However, the homolog instances may carry noise that results from evolutionary divergence, hence we need to choose a noise-tolerant machine learning framework to implement homolog knowledge transfer. To our knowledge, support vector machine (SVM) is a theoretically well-established machine learning algorithm^15^ that gracefully reduces the adverse effect of noise via regularization technique. For the sake of clarity, here we briefly describe how SVM could explicitly tolerate a certain level of noise. Given training data *x*_*i*_ ∈ *R*^*n*^, *i* = 1, 2, …, *l* and class labels *y* ∈ *R*^*l*^, *y*_*i*_ ∈ {−1, 1}, C-SVM solves the following primal optimization problem:


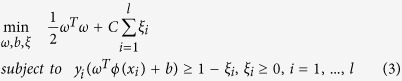


where *ω* represents weight vector, *ϕ*(*x*_*i*_) is mapping function and *C* denotes penalty parameter. Here the slack variables *ξ*_*i*_(≥0, *i* = 1, …, *l*) are introduced to tolerate a certain level of noise, without which, i.e. *ξ*_*i*_ = 0, *i* = 1, …, *l*, C-SVM formulated in formula (3) would be degenerated to a hard-margin SVM. In formula (3), the adverse effect of noise is counteracted by the penalty parameter *C*.

In addition, SVM uses well-known kernel trick to define the inner product between mapping function *ϕ*(*x*) and *ϕ*(*y*), i.e. *k*(*x, y*) = (*ϕ*(*x*)•*ϕ*(*y*)). In the kernel function *k*(*x, y*), there is no need of explicit definition and computation of the mapping function *ϕ*(*x*). Here we adopt *Gaussian* kernel.


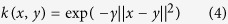


where ||Δ|| denotes the 2-norm of vector Δ, and the hyperparameter *γ* controls the flexibility of *Gaussian* kernel.

Each test protein-protein pair (*i*_1_, *i*_2_) is represented with the target instance 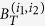
and the homolog instance 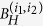, the decision function *f (x*) accordingly yields two outputs, i.e. 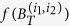 and 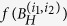. Combining the two outputs, we define the final decision as follows.


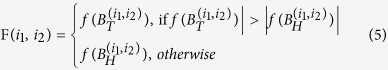


Where |Δ| denotes the absolute value of Δ. Based on the final decision function, we can further determine the final class label for the test protein-protein pair (*i*_1_, *i*_2_) as follows.


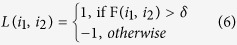


where the threshold *δ* is used to filter out those positive predictions with low confidence.

### Experimental settings and model evaluation

We design three experimental settings to demonstrate the effectiveness of homolog knowledge transfer via homolog instances. The first setting, namely *Single-instance SVM* that represents each protein pair with the target instance alone, is used as the baseline. The second setting, namely *Multi-instance SVM Novel*, is deliberately designed to evaluate the robustness of the model to data unavailability. In this setting, the training data are represented with both target instances and homolog instances, while the test data are represented with only homolog instances. The third setting, namely *Multi-instance SVM*, is designed to evaluate the enrichment of feature information brought about by the homolog instances. In this setting, both the training data and the test data are represented with target instances and homolog instances.

Here we use cross validation and independent test to evaluate the model performance. To reduce the risk of evaluation bias, we simultaneously adopt multiple performance metrics including ROC-AUC (Receiver Operating Characteristic AUC), SE (sensitivity), SP (specificity), MCC (Matthews correlation coefficient), F1 score and Accuracy. Except AUC score, all the other metrics can be derived from confusion matrix. Given confusion matrix *M*, several intermediate variables are defined by formula (7), and then SP_l_, SE_l_ and MCC_l_ for each class label can be calculated by formula (8). Overall accuracy and MCC can be calculated by formula (9),


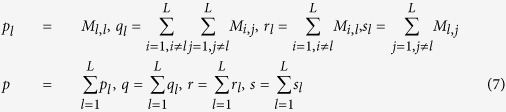



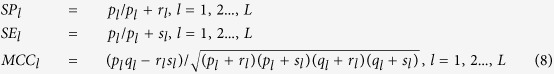



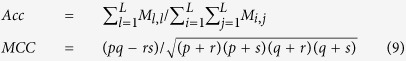


where the element of confusion matrix *M*_*i*_,_*j*_ records the counts that class *i* are classified as class *j*, and *L* denotes the number of class labels. AUC is calculated based on the decision values as defined by formula (5), and F1 score is calculated by formula (10).


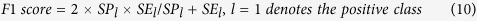


## Results

### Cross validation and independent test

#### Cross validation on the VirusMINT dataset

**Figure 2:**
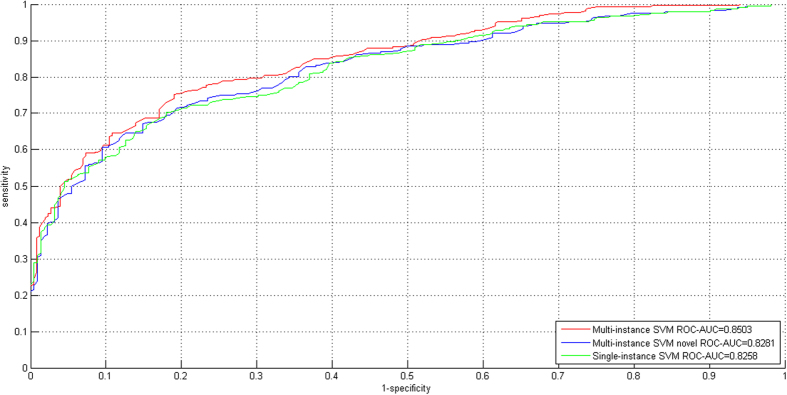
ROC curves for the three experimental settings (i.e. Multi-instance SVM, Multi-instance SVM Novel and Single-instance SVM) on the VirusMINT dataset.

We first evaluate the preliminary feasibility on the VirusMINT dataset. From VirusMINT database^16^, 266 interactions are extracted and are treated as positive data, and the same size of negative data are randomly sampled to train a two-class SVM model. The results of 10-fold cross validation for the three experimental settings are summarized in Table 1, and the corresponding ROC curves are shown in Fig. 2. From the results, we can see that *Multi-instance SVM* achieves the best performance (AUC = 0.8503; Acc = 77.10%; MCC = 0.6139; F1 Score = 0.7736), slightly outperforming *Multi-instance SVM Novel* (AUC = 0.8281; Acc = 75.32%; MCC = 0.5879; F1 Score = 0.7597) and *Single-instance SVM* (AUC = 0.8258; Acc = 73.84%; MCC = 0.5667; F1 Score = 0.7510). The results of *Multi-instance SVM Novel* indicate that the proposed model still works well when the GO knowledge of the gene/protein concerned is not available. Comparing the SP, SE and MCC scores on the positive class and the negative class (see Table 1), we can see that that the proposed model yields little predictive bias.

#### Independent test on the Virhostome dataset

The Virhostome dataset contains 84 interactions. To verify how well the model trained on the VirusMINT dataset generalizes to unseen test data, we further conduct independent test on the Virhostome dataset^17^. The computational result shows that 82.14% of the Virhostome dataset (84 interactions) are correctly recognized. This performance is very promising. At present the independent test performance of the existing methods is not satisfactory. For instances, the semi-supervised multi-task learning method^3^ recognized only 10% experimentally derived HIV-human PPIs. The biological method HT-Y2H recognized only 2.1% HTLV-human PPIs that are derived by other biological experimental methods^23^.

#### Cross validation on the VirusMINT + Virhostome dataset

**Figure 3:**
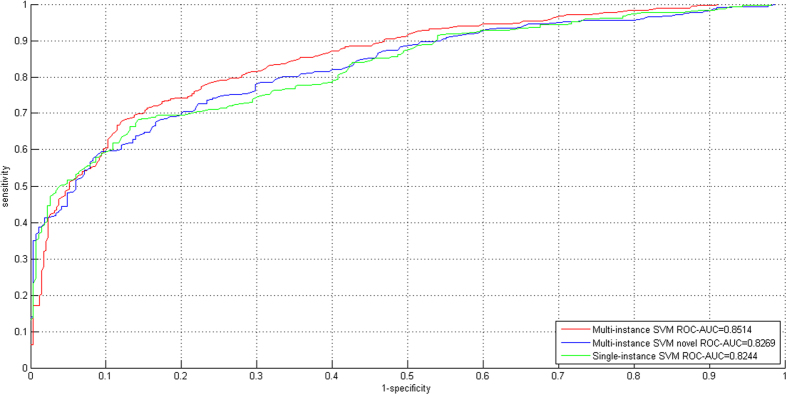
ROC curves for the three experimental settings (i.e. Multi-instance SVM, Multi-instance SVM Novel and Single-instance SVM) on the VirusMINT + Virhostome dataset.

We merge the interactions from VirusMINT and Virhostome databases into the final positive training data (called VirusMINT + Virhostome) that contains 350 examples. To train a two-class SVM model, we also randomly sample 350 negative data (see Supplementary File).The results of 10-fold cross validation for the three experimental settings are provided in Table 2. The ROC curves for 10-fold cross validation are illustrated in Fig. 3. The results in Tables 1 and 2 show that the incorporation of the interactions from Virhostome database does not yield much performance gain. Nevertheless, we still choose the VirusMINT + Virhostome dataset as the final positive training data.

### Proteome-wide reconstruction of EBV-human PPI networks

There are 32 EBV genes/proteins to study in the training data (VirusMINT + Virhostome). The potential human target genes are retrieved from Uniprot (ftp://ftp.uniprot.org/pub/databases/uniprot/current_release/knowledgebase/taxonomic_divisions/uniprot_sprot_human.dat.gz). For each EBV gene/protein, we derive its prediction space by excluding those EBV-human protein pairs that already exist in the training data. Averagely over 20,000 human candidate genes are derived for each EBV gene. The results of the proteome-wide predictions are provided in the Supplementary File. Here we set the threshold *δ* = 0.01 (see formula (6)) to reduce the risk of false positive predictions. Among the 648,672 EBV-human protein pairs, there are 51,485 protein pairs predicted to be interacting (positive), accounting for 7.94% positive rate. Jansen *et al*.^24^ proposed a doctrine that the expected number of negatives (i.e. non-interacting protein pairs) is several orders of magnitude higher than the number of positives (i.e. interacting protein pairs). The 7.94% predicted positive rate is consistent with the doctrine, indicating a low risk of false positive predictions. Nevertheless, 49.64 percent of the 20,334 human proteins are predicted to be targeted by the 32 EBV genes, potentially indicating a certain risk of false positive predictions. It is worth noting that the predicted EBV-human PPIs are functional protein-protein interactions, because we use the three aspects of gene ontology (cellular compartments, molecular functions and biological processes) to depict genes/proteins. If we impose subcellular co-localization on the predicted functional PPIs, we can derive predicted physical PPIs between Epstein-Barr virus and Homo sapiens.

Here we propose two methods to determine whether or not an EBV gene and a human gene are subcellular co-localized. One method is to check whether or not the EBV gene and the human gene are annotated with the same GO term of cellular compartment. The general root GO term GO:0005575 is discarded because it does not provide any useful information. Thus we obtain 869 physical EBV-human PPIs (see the Supplementary file), far less than the predicted 51,485 PPIs. Accordingly, the predicted human target genes add up to 153. This method is reliable to derive physical PPIs, but is too stringent to cover all physical PPIs because the present GO annotations of both EBV genes and human genes are far incomplete. The other method is to relax the criteria of subcellular co-localization. We assume that organelle membrane proteins have large chances to physically contact with the proteins inside or outside the organelle. Under this assumption, we deem as physical interaction any predicted EBV-human PPI that contains EBV membrane protein or human membrane protein. Thus we obtain 46,050 physical EBV-human PPIs (accounting for 7.1% positive rate) and 8,852 human target genes (accounting for 43.53% target rate) (see Supplementary file). This method gains wide coverage of physical EBV-human PPIs, but meanwhile covers those functional EBV-human PPIs whose EBV proteins and human proteins may have no chances of physical contact.

**Figure 4:**
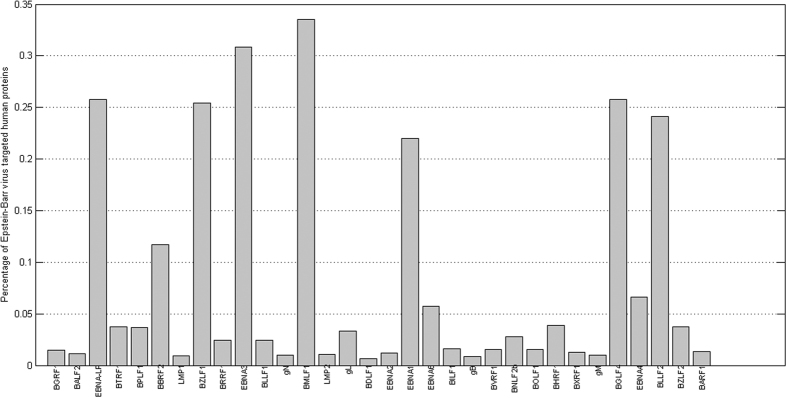
Predicted percentage of Epstein-Barr virus targeted human proteins.

As a whole, the 49.64 percentage of total human genes that the 32 EBV genes seems high, but most of the EBV genes/proteins are predicted to individually interact with less than 5% human genes/proteins (see Fig. 4). Only seven EBV genes/proteins are predicted to interact with more than 20% of the human genes/proteins, including BMFL1 (33.52%), EBNA-LP (25.77%), BZLF1 (25.42%), EBNA3 (30.84%), EBNA1 (21.99%), BGLF4 (25.79%) and BLLF2 (24.11%). Even so, the percentage of human target genes is not high as compared to the existing computational methods of pathogen-host PPI prediction. For instances, 22,651 human genes out of 22,654 human genes are predicted to interact with *Salmonella* genes^25^. HTLV gene is predicted to interact with at least 20% human genes and the highest predicted percentage of human target genes is up to 44.73%^26^. Comparatively, the false positive rate achieved by the proposed method is acceptable.

### Validation against the latest database and recent literature

We further validate the proteome-wide predictions against the latest virus-host database and recent literature. It is not easy to gather supporting evidences in that new evidences are scarce and scattered among thousands of literature. Nevertheless, we still find 58 evidences to support our predictions (see Table 3), including 33 experimental evidences from VirusMentha database^18^ (http://virusmentha.uniroma2.it/) and 25 experimental evidences from recent literature. Take the evidences from recent literature as examples. The interactions {BGLF4, SUMO1} and {BGLF4, SUMO2} have been experimentally verified^27^,^28^. In ref. 27, it has been claimed that SUMO binding by BGLF4 modulates BGLF4 function and affects the efficiency of lytic EBV replication. As regards {BGLF4, Nup62}, it has been claimed that BGLF4 binds to Nup62 and Nup153 to inducereorganization of the nuclear pore complex^29^. In ref. 30, XPC and Cdc20have been identified to predict with BGLF4. As regards {EBNA-LP, ESRRA}, EBNA-LP has been verified to interact with hERR1 (ESRRA) experimentally by yeast two-hybrid library screen, GST pull-down experiments, antibodies & immunoblotting and reporter gene assays, and the interaction involved in EBV-induced transformation affects the expression of hERR1-inducible cellular and viral genes^31^. As regards with {EBNA-LP, RB1}, EBNA-5 protein (EBNA-LP) is reported to form a molecular complex with the retinoblastoma(RB) and p53 tumor suppressor proteins for B-cell transformation^32^. In ref. 33, the following interactions {EBNA-LP, CDKN2A}, {BZLF1, UBN1}, {EBNA1, RPA1}, {EBNA1, TNPO1}, {EBNA3, CTBP1}, {EBNA3, AIP}, {EBNA3, AHR} and {EBNA6, SMN1} were used as training examples for computational modeling. As regards {BZLF1, PARP1}, BZLF1has been experimentally identified to interact with PARP1 to induce repair DNA damage against EBV infection^34^. In ref. 35, BZLF1 is claimed to enhance the ubiquitination and degradation of p53 so as to inhibit the interaction between p53 and MDM2, and thus blocks p53-downstream signaling for efficient viral propagation. In ref. 36, BZLF1 is reported to interact with ZEB1, TP53INP1and NOTCH2. The interaction of Zeb1 with BZLF1 promoter inhibits the lytic cycle inmodel systems, and Notch ligation is experimentally demonstrated to inhibit BZLF1 expression in primary B cell infection. Meanwhile, BZLF1 has also been reported to interact with SUMO1/2/3 in ref. 28. In ref. 37, EBNA1 is experimentally demonstrated to functionally interact with Brd4 in native and heterologous systems to mediate transcriptional activation.

### Comparison with the existing methods on the small *Salmonella* data

The above-described performance estimation of cross validation and independent test has demonstrated the reliability of the proposed method, and the validation against the latest database and recent literature further demonstrates the practical feasibility of the proposed method, we still need to apply the proposed method to other pathogen-host PPI data. Different from the existing methods that reconstruct PPI networks between HIV-1 and Homo sapiens^2^,^3^,^4^,^5^,^6^,^7^,^8^,^9^, the proposed method is especially developed for very small training data.

**Figure 5:**
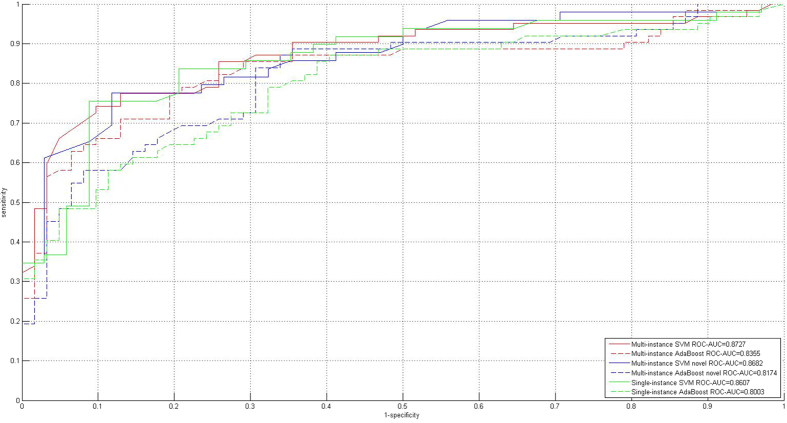
Performance comparison with the existing method on the *Salmonella* dataset.

The size of the PPI networks between *Salmonella* and Homo sapiens is smaller than or approximate to that of the PPI networks between Epstein-Barr virus and Homo sapiens. In ref. 38, a computational method called multi-instance AdaBoost is proposed to exploit 66 PPIs between *Salmonella* and Homo sapiens. This method also augments the training data via homolog instances, but it differently implements noise control under the framework of AdaBoost. We conduct the performance comparison on the same *Salmonella* training data as^38^ and the performance comparison is provided in Table 4 and illustrated in Fig. 5. The computational results show that the proposed method achieves significant performance improvement as compared to the recently advanced multi-instance AdaBoost^38^. The performance improvement is largely brought about by support vector machine (SVM). The results also show that the theoretically-sound SVM outperforms the empirical AdaBoost on the *Salmonella* data in terms of noise tolerance and generalization ability.

HIV-1 is a well-studied virus with the largest experimental virus-host PPI networks, and accordingly computational modeling on the networks has aroused much attention from researchers^2^,^3^,^4^,^5^,^6^,^7^,^8^,^9^. In ref. 9, a training set that contains 3,638 positive examples and 3,638 negative examples is derived to train a probability weighted ensemble transfer learning model. The method proposed in this work is seemingly not applicable to such a large training data because doubling the training data significantly increases the computational complexity on SVM training or even results in computational infeasibility. For the reason, we do not apply the proposed method to the experimental PPI networks between HIV-1 and Homo sapiens.

## Discussions

In recent years, pathogen-host PPI networks reconstruction as a research field of microbial informatics has drawn much attention from computational biologists, e.g. HIV-1^2^,^3^,^4^,^5^,^6^,^7^,^8^,^9^, HTLV^26^, Salmonella^38^, etc. Nourani *et al*.^39^ reviewed a broad range of computational methods for the reconstruction of pathogen-host PPI networks. Discovery of the targeted human genes and signaling pathways is of significance to understand the pathogenesis of Epstein-Barr virus (EBV). Computational reconstruction of proteome-wide protein-protein interaction (PPI) networks between Epstein-Barr virus and Homo sapiens is the first step to achieve this goal. Based on the predicted EBV-human PPI networks, we can infer how Epstein-Barr virus interferes with the normal molecular functions of human genes/proteins and how Epstein-Barr virus blockades human signaling pathways. With this knowledge, it is promising to design or choose proper inhibitors to suppress EBV genes or blockade EBV-human PPIs.

In this work we propose a noise-tolerant homolog knowledge transfer method to discover novel human target genes and signaling pathways, where homolog knowledge is used as independent homolog instances to augment the training data. The homolog instances serve three major purposes: (1) reducing the risk of model overfitting that results from small training data; (2) enriching the feature information of the target instances; (3) substituting the target instances when the knowledge of gene ontology of the gene/protein concerned is not available. The homolog noise that results from evolutionary divergence is counteracted by the regularization technique of support vector machine (SVM).

False positive rate is an important concern of computational reconstruction of protein-protein interaction networks. At present we cannot eliminate false positive predictions completely because the data quality and the computational method are far imperfect. What we are concerned about is how large false positive rate is acceptable. Unfortunately, we do not know the true ratio of positive (interactions) to negative (non-interactions) in the real world, thus we cannot rationally determine the acceptable false positive rate. Nevertheless, we still attempt to evaluate the risk of false positive predictions from the two aspects. The first aspect is the ratio of the predicted positives to the whole space of protein pairs. The proposed method predicts 51,485 functional interactions in the space of 648,672 EBV-human protein pairs (7.94%). If we put the constraint of subcellular co-localization on the predictions, we obtain 869 stringent physical PPIs (EBV gene and human gene are annotated with the same GO term of cellular compartment) and 46,050 relaxed physical PPIs (membrane proteins are assumed to have chances to physically interact with the proteins inside or outside corresponding organelles). Low ratio of positive predictions surely reduces the risk of false positive predictions. The other aspect is the ratio of EBV targeted human genes. In this work, the computational results show that most of the EBV genes/proteins are predicted to individually interact with less than 5% human genes/proteins. Low ratio of EBV targeted human genes also implies low risk of false positive predictions. If the threshold *δ* defined in formula (6) is increased, the two ratios will be decreased to achieve lower risk of false positive predictions.

To reduce the risk of false positive predictions and make the predictions reliable, we need to take into account several major factors for computational modeling, e.g. data size, data quality, data representativeness, computational algorithm, etc. In this work, the data size is increases via homolog instances; the representativeness of negative data is implemented via random sampling in the huge space of protein pairs; the data quality is guaranteed by adopting literature-curated experimental PPI data; and SVM is adopted as the computational framework to reduce the risk of negative homolog knowledge transfer.

The Computational results show that the proposed method achieves satisfactory cross validation and independent test performance. Using the trained model, we have reconstructed the proteome-wide protein-protein interaction networks between Epstein-Barr virus and Homo sapiens, where 33 predictions have been validated against recent VirusMentha database and 25 predictions have been validated against recent literature. To gain more insights, we further conduct GO enrichment and pathway enrichment analysis of predicted proteome-wide EBV-human PPI networks.

### Gene ontology based clustering analysis of EBV-targeted human genes

To cluster the EBV-targeted human proteins that fulfil identical molecular functions, participate in the same biological processes or reside in the same cellular compartments, we use gene ontology term (GO term) as the distance metric for clustering, i.e., the interacting human partners that are annotated with the same GO term are assigned to the same cluster. All the GO terms of human genes/proteins are classified into three major classes, biological processes (P), molecular functions (F) and cellular compartments (C). For each major class, we further consider two scenarios to study the common attack patterns of the 32 EBV proteins: (1) all the 32 EBV proteins are involved in the PPI subnetwork; (2) NOT all the 32 EBV proteins are involved in the PPI subnetwork. The predicted PPI subnetworks are given in the Supplementary File. Here, we take only four predicted PPI subnetwork as examples, interested readers are referred to the Supplementary file for biological cues.

### PPI subnetwork GO:0042632 - cholesterol homeostasis

**Figure 6:**
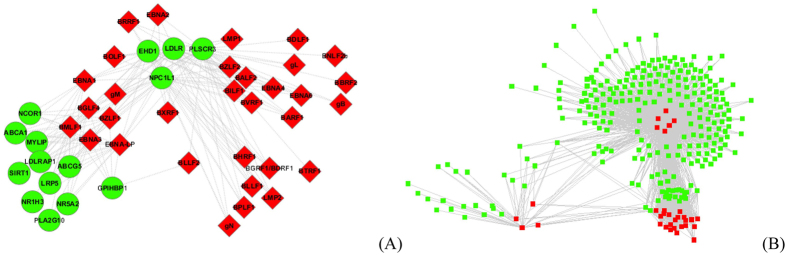
(**A**) The predicted EBV-human PPI sub-networkGO:0042632 (biological processes: cholesterol homeostasis); (**B**) The predicted EBV-human PPI sub-networkGO:0007596 (biological processes: blood coagulation). The red diamond denotes the EBV proteins and the green circle human proteins.

The predicted PPI subnetwork GO:0042632 extracted from the Supplementary file is illustrated in Fig. 6(A). All the human genes/proteins in Fig. 6(A) are involved in the biological processes of cholesterol homeostasis (GO:0042632). As shown in Fig. 6(A), the human protein PLSCR3 is predicted to be targeted by all the 32 EBV proteins. According to UniprotKB (http://www.uniprot.org/uniprot/Q9NRY6), PLSCR3 is claimed to mediate ATP-independent bidirectional transbilayer migration of phospholipids upon binding calcium ions. PLSCR3 also plays a central role in the initiation of fibrin clot formation, the activation of mast cells, the recognition of apoptotic cells and the translocation of cardiolipin from the inner to the outer mitochondrial membrane. From the predicted interactions, we can infer that EBV proteins may interfere with the cholesterol homeostasis and the fibrin clot formation of the host cell. Besides PLSCR3, the other three human proteins {NPC1L1, EHD1, LDLR} are also predicted to be targeted by most of the EBV proteins. NPC1L1 plays important roles in cholesterol biosynthetic process, cholesterol transport and intestinal cholesterol absorption (http://www.uniprot.org/uniprot/Q9UHC9). EHD1 plays roles in cholesterol homeostasis and positive regulation of cholesterol storage and blood coagulation (http://www.uniprot.org/uniprot/Q9H4M9). LDLR plays roles in phospholipid transport, lipoprotein metabolic process and regulation of cholesterol homeostasis (http://www.uniprot.org/uniprot/P01130). In addition, it has been reported the activity of EBV protein LMP2A depends on cholesterol and cholesterol depletion from plasma membrane blocks LMP2A endocytosis, LMP2A phosphorylation and LMP2A ubiquitination, resulting in the accumulation of LMP2A on plasma membrane^40^. These evidences suggest that EBV proteins may interfere with the cholesterol metabolism of host cell and may cause cholesterol related diseases.

### PPI subnetwork GO:0007596 - blood coagulation

The predicted PPI subnetwork GO:0007596 extracted from the Supplementary file is illustrated in Fig. 6(B). All the human proteins in Fig. 6(B) are involved in the biological processes of blood coagulation. Most of the 32 EBV proteins are predicted to target more than 20 human proteins, especially EBNA-LP (224 predicted human partners), EBNA3 (229 predicted human partners), BMLF1 (237 predicted human partners), EBNA1 (199 predicted human partners) and BGLF4 (206 predicted human partners). Among the human partners, PLSCR4 is predicted to be targeted by all the 32 EBV proteins, and the proteins {SPARC, CALU, LRP8, EGF, STIM1, ACTN2, PROC, THBD} are predicted to be targeted by 28 EBV proteins. According to UniprotKB (http://www.uniprot.org/uniprot/P09486), SPARC appears to regulate cell growth through interactions with the extracellular matrix and cytokines, and is involved in the biological processes of blood coagulation, platelet activation/degranulation, heart development, extracellular matrix organization. In ref. 41, it has been reported that a coagulopathy characterized by persistent and extreme elevations in plasma d-dimer and severe life-threatening hemorrhage is associated with hemophagocytic lymphohistiocytosis that is secondary to Epstein-Barr virus-associated T-cell lymphoproliferative disorder.

### PPI subnetwork GO:0005154 - epidermal growth factor receptor binding

**Figure 7:**
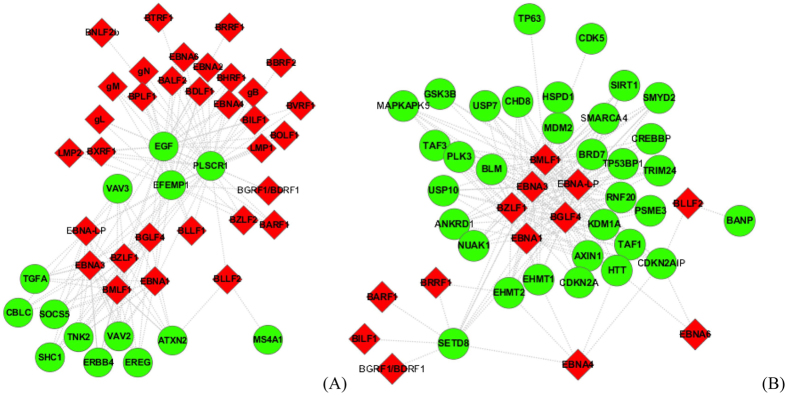
(**A**) The predicted EBV-human PPI sub-networkGO:0005154 (molecular functions: epidermal growth factor receptor binding); (**B**) The predicted EBV-human PPI sub-networkGO:0002039 (molecular functions: p53 binding). The red node denotes EBV proteins and the green node denotes human proteins.

The predicted PPI subnetwork GO:0005154 extracted from the Supplementary file is illustrated in Fig. 7(A). All the human proteins in Fig. 7(A) fulfil the molecular functions of epidermal growth factor receptor binding. Among the 32 EBV proteins, the EBV proteins {EBNA-LP, BZLF1, EBNA3, BMLF1, EBNA1, BGLF4} are predicted to target more than 10 human proteins. Among the predicted human partners, the proteins {EFEMP1, PLSCR1, EGF} are predicted to be targeted by more than 26 EBV proteins. According to UniprotKB (http://www.uniprot.org/uniprot/Q12805), EFEMP1 binds the EGF receptor (EGFR) to induce EGFR autophosphorylation and activation of downstream signaling pathways. In ref. 42, EBV proteinLMP1 is experimentally verified to modulate EGFR promoter activity in an NFkappaB-dependent manner.

### PPI subnetwork GO:0002039-p53 binding

The predicted PPI subnetwork GO:0002039 extracted from the Supplementary file is illustrated in Fig. 7(B). All the predicted human partners in Fig. 7(B) fulfil the molecular functions of p53 binding. The EBV proteins {EBNA-LP, EBNA3, BMLF1, EBNA1, BGLF4} are predicted to interact with more than twenty p53 binding human proteins, wherein SETD8 is predicted to be targeted by 11 EBV proteins. SETD8 is reported to mediate monomethylation of p53/TP53 at ‘Lys-382’ to repress p53/TP53-target genes, and play a negative role in TGF-beta response regulation and a positive role in cell migration (http://www.uniprot.org/uniprot/Q9NQR1). In ref. 43, it has been reported that BZLF1 has numerous effects on p53 posttranslational modification and may inhibit p53 transcriptional function in part through an indirect mechanism involving the suppression of TBP expression.

### EBV targeted human signaling pathways

Pathogens communicate with the host via chains of interactions (referred to as signaling pathways) to subvert the host cellular machinery for its purposes. In ref. 44, pathways analysis shows that a majority of pathways targeted by viral proteins are often used as drug targets. Here we map the predicted human genes/proteins onto the signaling pathways that are curated in NetPath^45^ to derive Epstein-Barr virus targeted human signaling pathways. In NetPath, there are 37 manually curated human cancer/immune signaling pathways. For the sake of simplicity, we merge the 11 sub-types of Interleukin (IL-1 ~ IL-11) into one single signaling pathway, and thus obtain 27 human signaling pathways. Pathway enrichment analysis shows that the 27 signaling pathways are all targeted by Epstein-Barr virus (see Supplementary file). Here we take two signaling pathways as examples and interested readers are referred to the supplementary file for biological cues.

### Notch signaling pathway

**Figure 8:**
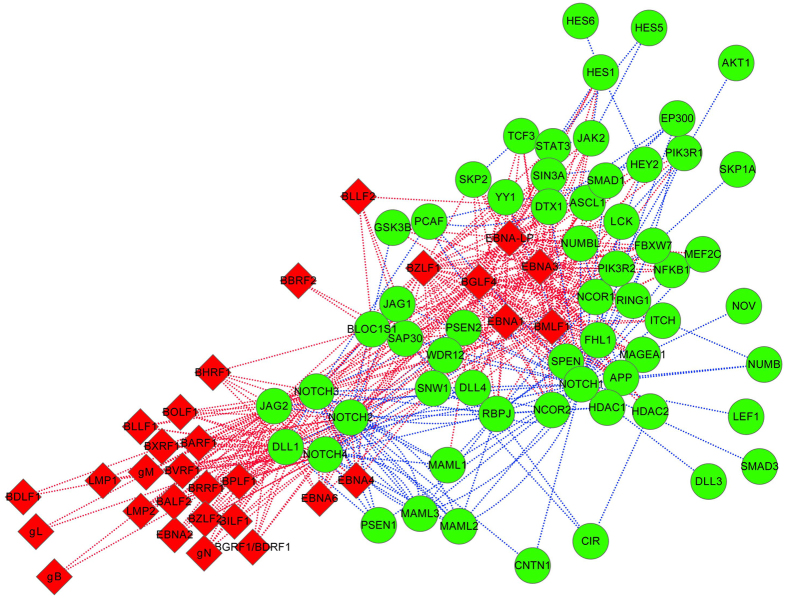
Notch signaling pathway targeted by Epstein-Barr virus. The red diamond denotes EBV proteins and the green circle denotes human proteins. The red dot line denotes the predicted EBV-human PPI and the blue dot line denotes the known interaction in Notch signaling pathway. For the sake of clarity, only the Epstein-Barr virus targeted signaling components of Notch signaling pathway are illustrated.

There are 335 predicted interactions between EBV proteins and the known Notch signaling components. As illustrated in Fig. 8, the signaling components {NOTCH2, NOTCH3, NOTCH4, DLL1, JAG2} are predicted to be targeted by the majority of EBV proteins, and meanwhile the EBV proteins {EBNA-LP, EBNA1, EBNA3, BGLF4, BMLF1, BZLF1} are predicted to target a majority of Notch signaling components. In ref. 46, it has been reported that EBV protein LMP2A causes an elevated mitochondrial fission in gastric and breast cancer cells and LMP2A-mediated Notch pathway is responsible for this enhanced fission.

### Hedgehog signaling pathway

**Figure 9:**
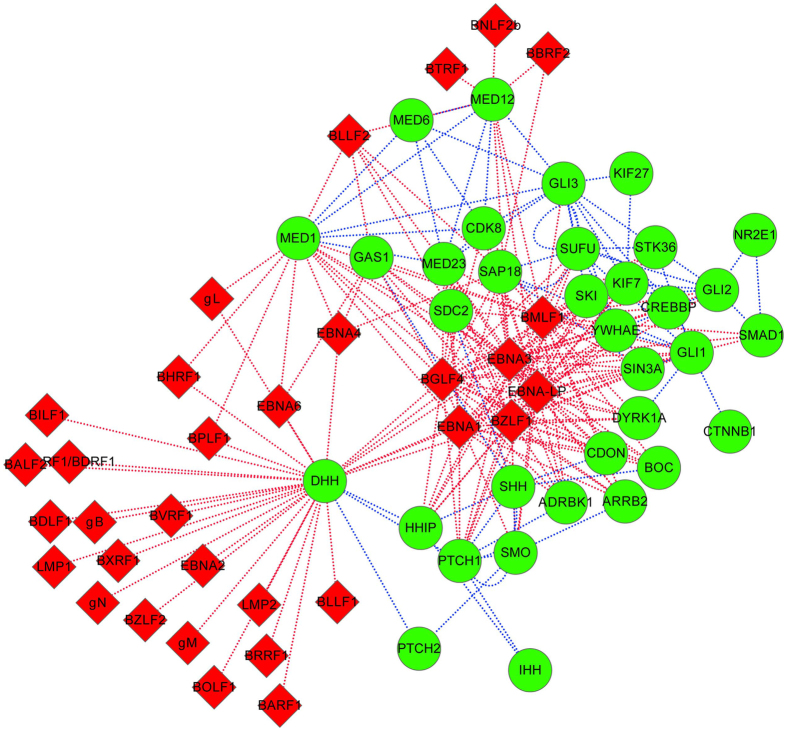
Hedgehog signaling pathway targeted by Epstein-Barr virus. The red diamond denotes EBV proteins and the green circle denotes human proteins. The red dot line denotes the predicted EBV-human PPI and the blue dot line denotes the known interaction in Hedgehog signaling pathway. For the sake of clarity, only the Epstein-Barr virus targeted signaling components of Hedgehog signaling pathway are illustrated.

There are 175 predicted interactions between EBV proteins and the known Hedgehog signaling components. As illustrated in Fig. 9, the signaling component {DHH} is predicted to be targeted by 29 EBV proteins. According to UniprotKB (http://www.uniprot.org/uniprot/O43323), DHH acts as intercellular signal essential for a variety of patterning events during development, e.g. male sex determination, spermatid development, Leydig cell differentiation, etc., and may function as a spermatocyte survival factor in the testes. Among the EBV proteins, {EBNA-LP, EBNA1, EBNA3, BGLF4, BMLF1, BZLF1} are predicted to target a majority of Hedgehog signaling components. In ref. 47, it has been reported that Epstein-Barr virus plays roles in dysregulated Hedgehog signaling pathway in NPC (nasopharyngeal carcinoma) oncogenesis.

## Additional Information

**How to cite this article**: Mei, S. and Zhang, K. Computational discovery of Epstein-Barr virus targeted human genes and signalling pathways. *Sci. Rep.* **6**, 30612; doi: 10.1038/srep30612 (2016).

## Supplementary Material

Supplementary Information

## Figures and Tables

**Table 1:** Ten-fold cross validation performance estimation on the VirusMINT dataset.

	Multi-instance SVM			Multi-instance SVM Novel			Single-instance SVM		
	SP	SE	MCC	SP	SE	MCC	SP	SE	MCC
Positive (interaction)	0.7765	0.7707	0.6153	0.7872	0.7341	0.5928	0.7602	0.7421	0.5735
Negative (non-interaction)	0.7654	0.7713	0.6125	0.7197	0.7748	0.5854	0.7149	0.7342	0.5584
[Acc; MCC]	[77.10%; 0.6139]			[75.32%; 0.5879]			[73.84%; 0.5667]		
[ROC-AUC]	[0.8503]			[0.8281]			[0.8258]		
F1 Score	0.7736			0.7597			0.7510		

**Table 2:** Ten-fold cross validation performance estimation on the VirusMINT + Virhostome dataset.

	Multi-instance SVM			Multi-instance SVM Novel			Single-instance SVM		
	SP	SE	MCC	SP	SE	MCC	SP	SE	MCC
Positive (interaction)	0.7994	0.7400	0.6199	0.8288	0.6698	0.5852	0.7964	0.7013	0.5760
Negative (non-interaction)	0.7580	0.8143	0.6285	0.6779	0.8340	0.5890	0.6865	0.7849	0.5685
[Acc; MCC]	[77.71%; 0.6230]			[74.44%; 0.5753]			[73.93%; 0.5679]		
[ROC-AUC]	[0.8514]			[0.8269]			[0.8243]		
F1 Score	0.7686			0.7409			0.7458		

**Table 3:** Ppredicted interactions validated by VirusMentha database and recent literature.

EBV proteins	VirusMenthavalidation	Literature validation
BGLF4	SUMO1{0.080};SUMO2{0.196}; KAT5{0.103}; XPC{0.111};	SUMO1{0.080}^27^,^28^; SUMO2{0.196}^27^,^28^; Nup62{0.057}^29^; Nup153{0.103}^29^;XPC{0.111}^30^; Cdc20{0.085}^30^;
EBNA-LP	BAG3{0.319};SLC25A5{0.258};EIF2S1{0.286}; HSP90AA1{0.178};NME1{0.175};ATP5A1{0.196}; GCHFR{0.222};CDKN2A{0.029};RPL27A{0.304}; TMED10{0.199};ACTB{0.102};RPL11{0.206}; RBBP4{0.169};PCBP1{0.336}; RBBP7{0.197};RPS27L{0.248};PHB2{0.234}; TMED9{0.267};FKBP14{0.167}; STUB1{0.145}	ESRRA{0.101}^31^; RB1{0.014}^32^; CDKN2A{0.029}^33^
BZLF1	—	PARP1{0.056}^34^; MDM2{0.036}^35^; NOTCH2{0.011}^36^;TP53INP1{0.061}^36^; ZEB1{0.018}^36^; UBN1{0.051}^33^; SUMO1{0.070}^28^; SUMO2{0.186}^28^; SUMO3{0.209}^28^;
EBNA1	IPO5{0.138}; ORC4{0.084}; RPA1{0.045}; PML{0.019}; ORC1{0.081}; KPNB1{0.094}; NAP1L4{0.132}; CDC6{0.050}	Brd4{0.116}^37^; RPA1{0.045}^33^; TNPO1{0.124}^33^;
EBNA3	AHR{0.031}	CTBP1{0.060}^33^; AIP{0.140}^33^; AHR{0.031}^33^;
EBNA6	—	SMN1{0.060}^33^;

The number in the curly braces denotes the confidence level, and the number in the square bracket denotes the literature reference number.

**Table 4:**
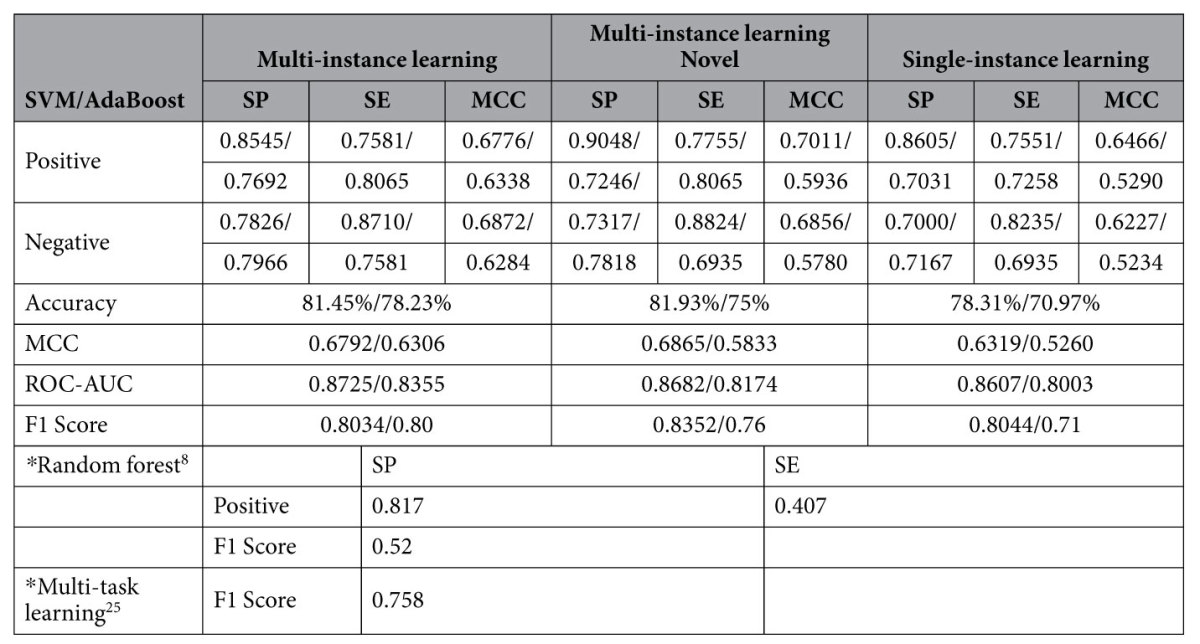
Comparison with the existing methods on the *Salmonella* dataset.

Note: the number before the slash(/) denotes the performance of the proposed method; the number after the slash(/) denotes the performance of the method^38^; *denotes the other existing methods.
