# Supplementary material for: Computational discovery of Epstein-Barr virus targeted human genes and signalling pathways
Source: Sci Rep. 2016 Jul 29;6:30612. doi: 10.1038/srep30612 (PMC4965740; doi:10.1038/srep30612)
Supplement: Supplementary Information [file srep30612-s1.pdf]

Computational discovery of Epstein-Barr virus targeted human genes and signalling pathways

Suyu Mei[①*], Kun Zhang[②#]

① Software College, Shenyang Normal University, Shenyang, 110034, China

② Department of Computer Science, Xavier University of Louisiana, New Orleans, LA 70125, USA

Correspondence: [*]meisygle@gmail.com, [#]kzhang@xula.edu

```
EBNA-LP HNRNPA2B1   +1.0
EBNA-LP SEC16A  +1.0
EBNA-LP CLTC    +1.0
EBNA1   SRPK2   +1.0
EBNA1   CSNK2A1 +1.0
BMLF1   KHDRBS2 +1.0
BRRF1   SNX4    +1.0
Q1HVI2  GRN +1.0
BVRF1   IMMT    +1.0
EBNA-LP PCCB    +1.0
BRRF1   CAP2    +1.0
gL  HOMER3  +1.0
BHRF1   BIK +1.0
EBNA1   MDFI    +1.0
EBNA-LP DNAJB6  +1.0
BILF1   HOMER3  +1.0
EBNA2   CD46    +1.0
EBNA1   USP7    +1.0
EBNA-LP RPS13   +1.0
EBNA4   SIAH1   +1.0
LMP2    LGALS3BP    +1.0
BRRF1   WWP2    +1.0
EBNA-LP FLNC    +1.0
BLLF1   SIAH1   +1.0
BHRF1   LTBP4   +1.0
BHRF1   TNXB    +1.0
EBNA6   RBPMS   +1.0
EBNA-LP RPL18   +1.0
BPLF1   CLK1    +1.0
EBNA2   DYNLL1  +1.0
EBNA-LP RPL10   +1.0
EBNA-LP HSPA5   +1.0
EBNA-LP RPL7    +1.0
BBRF2   GSTA4   +1.0
EBNA-LP HSPA1A  +1.0
EBNA-LP GLUD1   +1.0
EBNA2   CBX3    +1.0
gM  CCDC33  +1.0
EBNA-LP P4HA2   +1.0
BHRF1   MARCO   +1.0
gM  HOMER3  +1.0
BALF2   ELF2    +1.0
BBRF2   PTBP1   +1.0
BRRF1   SH3GLB1 +1.0
BOLF1   SERTAD1 +1.0
EBNA-LP SF3B1   +1.0
BXRF1   FBLN5   +1.0
BDLF1   CCDC14  +1.0
BPLF1   NUCB2   +1.0
EBNA-LP HNRNPK  +1.0
BPLF1   CCHCR1  +1.0
BTRF1   CALCOCO2    +1.0
Q1HVD9  SERTAD1 +1.0
BRRF1   MED4    +1.0
gB  TRIM27  +1.0
EBNA-LP HNRNPD  +1.0
gB  FBLN5   +1.0
EBNA2   SH3GLB1 +1.0
EBNA-LP HNRNPA1 +1.0
BVRF1   TSG101  +1.0
EBNA1   KPNA2   +1.0
BVRF1   RNF31   +1.0
BMLF1   PSME3   +1.0
BVRF1   PSMA3   +1.0
EBNA-LP SSBP1   +1.0
Q1HVI2  PRMT1   +1.0
EBNA-LP RPL7A   +1.0
BVRF1   MMRN2   +1.0
```

```
EBNA-LP NIPSNAP1    +1.0
EBNA6   VWF +1.0
BBRF2   CEP76   +1.0
BRRF1   SEPP1   +1.0
LMP1    ZMYND11 +1.0
gB  LTBP4   +1.0
EBNA-LP SNRPA   +1.0
EBNA1   LNX1    +1.0
BALF2   SIAH1   +1.0
BHRF1   VWF +1.0
BRRF1   TRAF2   +1.0
BZLF1   GPRASP1 +1.0
BRRF1   NTN4    +1.0
EBNA6   FBLN5   +1.0
EBNA-LP MLF2    +1.0
EBNA4   ACTN4   +1.0
BBRF2   NECAB2  +1.0
BRRF1   PHYHD1  +1.0
EBNA1   EBNA1BP2    +1.0
BGLF4   IGHG1   +1.0
gB  TRAF1   +1.0
EBNA4   TRAF4   +1.0
EBNA-LP SLC25A6 +1.0
EBNA-LP SET +1.0
BILF1   GRN +1.0
EBNA-LP CCDC67  +1.0
gB  GRN +1.0
EBNA-LP PCCA    +1.0
EBNA-LP RPL22   +1.0
EBNA-LP PTBP1   +1.0
BALF2   SDCBP2  +1.0
BHRF1   CCDC155 +1.0
EBNA-LP NONO    +1.0
EBNA1   KPNA1   +1.0
LMP1    EGFL7   +1.0
BLLF2   GRN +1.0
EBNA-LP SP100   +1.0
gB  MKRN3   +1.0
BMLF1   RBMX    +1.0
BARF1   ACTN1   +1.0
EBNA-LP RPS18   +1.0
EBNA-LP DDX5    +1.0
EBNA-LP SERBP1  +1.0
EBNA-LP TUBB    +1.0
EBNA4   BANP    +1.0
EBNA-LP SNRPB2  +1.0
EBNA-LP HAX1    +1.0
BILF1   VWF +1.0
BBRF2   PNMA1   +1.0
EBNA6   SEPP1   +1.0
BHRF1   CLEC7A  +1.0
BBRF2   MIPOL1  +1.0
BZLF1   TFG +1.0
Q1HVI2  SLIT3   +1.0
EBNA-LP UBR5    +1.0
EBNA-LP DARS    +1.0
EBNA2   EFEMP2  +1.0
EBNA-LP PRPF8   +1.0
LMP1    UBQLN1  +1.0
EBNA-LP EIF3B   +1.0
EBNA-LP HNRNPR  +1.0
EBNA-LP HNRNPAB +1.0
BSLF1   NFKB1   +1.0
EBNA-LP CLPX    +1.0
BMLF1   TFCP2   +1.0
BZLF1   POGZ    +1.0
BBRF2   TFCP2   +1.0
EBNA-LP DNAJB5  +1.0
```

```
	EBNA-LP	XRCC5	+1.0
	BALF2	TRAF1	+1.0
	EBNA2	GCA	+1.0
	EBNA1	NAP1L1	+1.0
	gB	AP2B1	+1.0
	EBNA1	PICK1	+1.0
	EBNA2	ZMYND11	+1.0
	EBNA-LP	TIMM50	+1.0
	BBRF2	RINT1	+1.0
	EBNA1	KPNA3	+1.0
	EBNA-LP	PSMA3	+1.0
	EBNA2	AES	+1.0
	BZLF1	ENOX2	+1.0
	EBNA-LP	TUBA1B	+1.0
	EBNA-LP	HIST1H1D	+1.0
	BMLF1	UBE2I	+1.0
	BPLF1	CD74	+1.0
	EBNA2	SRI	+1.0
	EBNA1	SRSF1	+1.0
	EBNA2	KRT36	+1.0
	BBRF2	TRAF1	+1.0
	EBNA-LP	RPS9	+1.0
	EBNA-LP	RAD50	+1.0
	BDLF1	CTSC	+1.0
	BHRF1	GRN	+1.0
	LMP1	RABAC1	+1.0
	EBNA-LP	SYNCRIP	+1.0
	EBNA4	ACTN1	+1.0
	BVRF1	EFEMP1	+1.0
	EBNA-LP	RPL8	+1.0
	EBNA-LP	RPL23	+1.0
	EBNA-LP	SF3B2	+1.0
	BLLF1	HOMER3	+1.0
	EBNA-LP	PCBP2	+1.0
	BILF1	LMNB1	+1.0
	EBNA-LP	AHCYL1	+1.0
	BBRF2	NEFL	+1.0
	BBRF2	GOLGA2	+1.0
	EBNA-LP	RFC3	+1.0
	EBNA-LP	CAPZA1	+1.0
	EBNA-LP	TNNT3	+1.0
	EBNA-LP	CDK1	+1.0
	EBNA2	FCHSD1	+1.0
	BPLF1	AP2B1	+1.0
	EBNA-LP	RPL17	+1.0
	EBNA-LP	RPS23	+1.0
	EBNA-LP	SRSF1	+1.0
	EBNA-LP	NCL	+1.0
	gB	VIM	+1.0
	BALF2	USHBP1	+1.0
	EBNA4	GMCL1	+1.0
	BVRF1	NCKIPSD	+1.0
	EBNA-LP	SCYL1	+1.0
	EBNA3	RPL4	+1.0
	EBNA-LP	RPL9	+1.0
	BBRF2	C1orf50	+1.0
	EBNA1	TERF2	+1.0
	EBNA-LP	DHX9	+1.0
	EBNA2	RCBTB1	+1.0
	BVRF1	ACTN1	+1.0
	BALF2	GSTA4	+1.0
	EBNA6	PSMA3	+1.0
	EBNA2	FBLN5	+1.0
	EBNA4	RBPMS	+1.0
	EBNA4	SH3GLB1	+1.0
	EBNA2	CD5L	+1.0
	BHRF1	MIPOL1	+1.0
	EBNA-LP	RCN2	+1.0
```

```
	EBNA-LP	HSPB1	+1.0
	BVRF1	EFEMP2	+1.0
	BILF1	SLIT2	+1.0
	BRRF1	FBLN5	+1.0
	EBNA-LP	GRPEL1	+1.0
	EBNA1	TSG101	+1.0
	BGRF1/BDRF1	GPRASP1	+1.0
	EBNA-LP	RUVBL1	+1.0
	EBNA-LP	IQGAP2	+1.0
	EBNA-LP	DDX17	+1.0
	EBNA-LP	MAGEA1	+1.0
	EBNA-LP	WDR77	+1.0
	BHRF1	TRAF1	+1.0
	EBNA-LP	RPL31	+1.0
	BHRF1	EXOC5	+1.0
	EBNA-LP	ESRRG	+1.0
	EBNA-LP	CSE1L	+1.0
	EBNA-LP	GBAS	+1.0
	BBRF2	ARHGEF10L	+1.0
	BARF1	TMEM66	+1.0
	gB	LAMB1	+1.0
	EBNA-LP	MAGED1	+1.0
	BALF2	MIPOL1	+1.0
	BARF1	PKM	+1.0
	BPLF1	GAPDH	+1.0
	BRRF1	PLSCR1	+1.0
	BPLF1	HBB	+1.0
	EBNA-LP	IARS	+1.0
	EBNA-LP	RPS3A	+1.0
	BPLF1	GRN	+1.0
	EBNA-LP	RPS16	+1.0
	BZLF2	GRN	+1.0
	EBNA-LP	SNRNP200	+1.0
	gB	RBPMS	+1.0
	BPLF1	CERS4	+1.0
	BARF1	SERTAD1	+1.0
	BALF2	NEFL	+1.0
	EBNA-LP	PSMB6	+1.0
	gM	NUCB1	+1.0
	BBRF2	TRIM27	+1.0
	BALF2	ZNF250	+1.0
	BMLF1	TRA2B	+1.0
	gB	PDE6G	+1.0
	LMP1	TRAF1	+1.0
	EBNA-LP	HNRNPCL1	+1.0
	gB	VWF	+1.0
	gB	FAM168B	+1.0
	EBNA-LP	RPL13	+1.0
	BGLF4	IQGAP2	+1.0
	Q1HVI2	HOMER3	+1.0
	EBNA3	NR4A1	+1.0
	EBNA-LP	QARS	+1.0
	BPLF1	RPL3	+1.0
	EBNA-LP	PSMD2	+1.0
	gB	LZTS2	+1.0
	EBNA1	ORC2	+1.0
	EBNA-LP	TRAF4	+1.0
	BHRF1	FN1	+1.0
	gB	USHBP1	+1.0
	gB	TRAF2	+1.0
	EBNA-LP	AKAP8L	+1.0
	EBNA-LP	RPL12	+1.0
	Q1HVI2	VWF	+1.0
	BGRF1/BDRF1	ENOX2	+1.0
	EBNA-LP	SEC13	+1.0
	EBNA-LP	RCN1	+1.0
	EBNA-LP	GCN1L1	+1.0
	BMLF1	DVL2	+1.0
```

```
BMLF1	RBPMS	+1.0
EBNA-LP	HNRNPU	+1.0
EBNA-LP	XPOT	+1.0
gB	FN1	+1.0
BPLF1	IGLL1	+1.0
BDLF3	HOMER3	+1.0
EBNA1	C1QBP	+1.0
EBNA-LP	UBC	+1.0
BBRF2	OPTN	+1.0
LMP1	TRADD	+1.0
BTRF1	PPP1CA	+1.0
BOLF1	PSMA3	+1.0
EBNA-LP	RPS8	+1.0
EBNA-LP	ILF3	+1.0
BBRF2	COPB1	+1.0
EBNA-LP	HIST1H2BC	+1.0
EBNA-LP	SFPQ	+1.0
gB	MID2	+1.0
BRRF1	PARP4	+1.0
BVRF1	PKM	+1.0
EBNA-LP	HSPA8	+1.0
EBNA6	MAGED1	+1.0
BRRF1	EFEMP1	+1.0
BGRF1/BDRF1	GPRASP2	+1.0
gB	SFN	+1.0
BPLF1	HLA-A	+1.0
EBNA-LP	RSL1D1	+1.0
BVRF1	PSME3	+1.0
EBNA4	PSMA3	+1.0
EBNA-LP	BAG2	+1.0
BPLF1	HLA-B	+1.0
BMLF1	MAPRE1	+1.0
EBNA-LP	HNRNPA0	+1.0
EBNA-LP	NUP93	+1.0
EBNA-LP	H1FX	+1.0
EBNA-LP	CAD	+1.0
BMLF1	BEND7	+1.0
EBNA-LP	EPRS	+1.0
BBRF2	USHBP1	+1.0
EBNA-LP	SNRPD2	+1.0
BPLF1	CIR1	+1.0
BHRF1	ACTN1	+1.0
EBNA4	ZYX	+1.0
EBNA-LP	DNAJB1	+1.0
EBNA-LP	RPS2	+1.0
EBNA4	PSME3	+1.0
BALF2	FBLN5	+1.0
gB	GPRASP1	+1.0
BHRF1	BAK1	+1.0
EBNA-LP	RPS6	+1.0
EBNA-LP	RUVBL2	+1.0
EBNA-LP	HSPD1	+1.0
EBNA-LP	PSMC2	+1.0
EBNA-LP	KHDRBS1	+1.0
EBNA-LP	ALDH1B1	+1.0
BBRF2	PICK1	+1.0
gN	HOMER3	+1.0
BRRF1	LZTS2	+1.0
BDLF1	MAPK7	+1.0
EBNA-LP	LRPPRC	+1.0
EBNA-LP	HNRNPM	+1.0
BHRF1	FATE1	+1.0
EBNA-LP	LDHB	+1.0
EBNA2	BLNK	+1.0
EBNA1	KPNA6	+1.0
BBRF2	TTC12	+1.0
BGRF1/BDRF1	TSC22D4	+1.0
BMLF1	RBMY1F	+1.0
```

```
gB  NECAB2  +1.0
BMLF1   SP100   +1.0
BNLF2b  GRN +1.0
gB  SIAH1   +1.0
EBNA3   RBPJ    +1.0
BMLF1   COIL    +1.0
EBNA-LP RPS17   +1.0
BHRF1   ERLIN1  +1.0
EBNA2   PSMA3   +1.0
EBNA1   CSNK2A2 +1.0
```

BARF1	PIK3CD	0.014649
BARF1	SPTA1	0.080431
BARF1	S100A7	0.12218
BARF1	HSPA5	0.070015
BARF1	LDLR	0.058185
BARF1	RABEP1	0.048344
BARF1	PLSCR3	0.18033
BARF1	RPUSD4	0.036127
BARF1	MYH7	0.010368
BARF1	SNAPIN	0.016262
BARF1	ATP5B	0.091401
BARF1	NIN	0.156
BARF1	KCNIP1	0.15012
BARF1	STIM2	0.17798
BARF1	WIPF1	0.018347
BARF1	SPTAN1	0.11038
BARF1	MASP1	0.22863
BARF1	SCUBE3	0.21
BARF1	TH	0.010696
BARF1	PROC	0.19267
BARF1	PDCD6	0.23588
BARF1	S100A9	0.053612
BARF1	ANXA2	0.10249
BARF1	MMP20	0.062311
BARF1	MATN4	0.21701
BARF1	NOTCH3	0.057127
BARF1	RNH1	0.19436
BARF1	CLIP1	0.088777
BARF1	AMOT	0.037373
BARF1	EIF4G2	0.01849
BARF1	THBD	0.13443
BARF1	RNMT	0.041019
BARF1	MAGOH	0.026178
BARF1	EML1	0.21696
BARF1	EPS15	0.085654
BARF1	PCSK2	0.061595
BARF1	PRMT8	0.022498
BARF1	AP3S1	0.087573
BARF1	SDF4	0.16075
BARF1	STAT1	0.025124
BARF1	FAT4	0.20957
BARF1	DTNBP1	0.019997
BARF1	PPEF1	0.11296
BARF1	NELL2	0.052769
BARF1	KAT2A	0.016012
BARF1	MAP1LC3C	0.018262
BARF1	THBS1	0.020574
BARF1	BICD1	0.015862
BARF1	PRMT1	0.036295
BARF1	AP1M1	0.037393
BARF1	TPPP	0.15086
BARF1	SYT11	0.26756
BARF1	HSP90B1	0.11469
BARF1	MACF1	0.14012
BARF1	GCH1	0.05243
BARF1	NKD2	0.21939
BARF1	ATP2B2	0.017472
BARF1	FNBP1L	0.027327
BARF1	FAT1	0.11212
BARF1	S100A11	0.17338
BARF1	NKD1	0.16477
BARF1	EIF1AY	0.022972
BARF1	PPP2R3B	0.10674
BARF1	ACTN4	0.087703
BARF1	EHD1	0.070362
BARF1	S100A13	0.16263
BARF1	MELK	0.061018
BARF1	S100A1	0.12111

BARF1	UTRN	0.084772
BARF1	AQP1	0.012087
BARF1	STAT6	0.022034
BARF1	CALB1	0.15886
BARF1	MASP2	0.21237
BARF1	PARK7	0.052335
BARF1	GAS6	0.013934
BARF1	EFEMP1	0.19709
BARF1	SPARC	0.089614
BARF1	NOTCH4	0.051696
BARF1	PCDH17	0.20957
BARF1	PRDX4	0.01216
BARF1	EIF3B	0.011651
BARF1	REPS1	0.19592
BARF1	NOTCH2	0.021305
BARF1	STIM1	0.098198
BARF1	S100B	0.091888
BARF1	TNNC1	0.19213
BARF1	TMED2	0.016972
BARF1	CETN2	0.16264
BARF1	ANXA6	0.2003
BARF1	ACTN2	0.067393
BARF1	PRSS2	0.18527
BARF1	STAT2	0.02326
BARF1	PITPNM3	0.19041
BARF1	VIL1	0.096224
BARF1	KCNJ4	0.05752
BARF1	CETN1	0.2074
BARF1	VLDLR	0.083972
BARF1	CDH1	0.040664
BARF1	ADAMTS13	0.013528
BARF1	MAP1LC3B	0.051723
BARF1	TTN	0.034704
BARF1	ZDHHC17	0.015295
BARF1	EGF	0.065128
BARF1	LTBP4	0.1552
BARF1	PRMT2	0.021402
BARF1	PLSCR4	0.17559
BARF1	RBM8A	0.024925
BARF1	NOP2	0.030797
BARF1	RHOT1	0.014662
BARF1	SNX9	0.10201
BARF1	RYR2	0.040372
BARF1	PRF1	0.086507
BARF1	IL6ST	0.088582
BARF1	MYO1E	0.052703
BARF1	SETD2	0.029549
BARF1	SNCA	0.010627
BARF1	S100A8	0.076519
BARF1	NID2	0.21193
BARF1	NELL1	0.11139
BARF1	DNER	0.13344
BARF1	PEF1	0.22535
BARF1	MEGF8	0.057424
BARF1	CHP1	0.086468
BARF1	CFTR	0.022142
BARF1	FASN	0.074861
BARF1	GPR98	0.083772
BARF1	MICU1	0.1805
BARF1	CALU	0.1676
BARF1	PPP3CA	0.040175
BARF1	LRP2	0.081724
BARF1	CAPN2	0.032483
BARF1	SLC23A1	0.03595
BARF1	NUCB2	0.13862
BARF1	S100A6	0.14151
BARF1	LRP8	0.14532
BARF1	S100P	0.14551

BARF1	PRDX6	0.037357
BARF1	SETD1B	0.030624
BARF1	RAB11FIP3	0.06782
BARF1	EFCAB4B	0.1586
BARF1	ALKBH8	0.020541
BARF1	DTNA	0.11107
BARF1	REPS2	0.23556
BARF1	PPEF2	0.098821
BARF1	EFEMP2	0.20955
BARF1	ATP2A1	0.064228
BARF1	WLS	0.041118
BARF1	PKD2L1	0.11617
BARF1	EHD3	0.088941
BARF1	THBS2	0.17508
BARF1	CALM1	0.092592
BARF1	EIF4E2	0.03434
BARF1	S100A4	0.22005
BARF1	S100A12	0.10799
BARF1	RCN1	0.23219
BARF1	DPP4	0.02976
BARF1	EIF4E	0.01998
BARF1	DNMT3A	0.01393
BARF1	ACTN3	0.2101
BARF1	ATP5F1	0.039964
BARF1	FBN1	0.19872
BARF1	F10	0.098947
BARF1	CAPN1	0.088108
BARF1	UCKL1	0.019065
BARF1	CELSR3	0.12757
BARF1	N6AMT1	0.046287
BARF1	SETD8	0.012743
BARF1	SIRT4	0.08821
BARF1	TESC	0.13156
BARF1	MICAL1	0.025621
BARF1	SETD1A	0.029191
BARF1	RPH3A	0.098297
BARF1	SRI	0.11112
BARF1	LRP4	0.1171
BARF1	SLIT2	0.039871
BARF1	MATN2	0.1733
BARF1	CIB1	0.15985
BARF1	PPP2R3A	0.14078
BARF1	DLL1	0.15917
BARF1	EHD4	0.055238
BARF1	UPF3B	0.032452
BARF1	MYO6	0.01457
BARF1	PRSS3	0.21621
BARF1	MYL2	0.21951
BARF1	EDEM1	0.11485
BARF1	LRP1	0.05293
BARF1	ADAR	0.016878
BARF1	SNX19	0.14452
BARF1	PDGFRB	0.025764
BARF1	ITSN1	0.044815
BARF1	DST	0.10864
BARF1	MYL6	0.20779
BARF1	COMP	0.14955
BARF1	CCDC47	0.18165
BARF1	ANXA7	0.10146
BARF1	CHP2	0.16948
BARF1	NECAB2	0.23794
BARF1	CANX	0.099784
BARF1	RHOT2	0.015002
BARF1	MMP15	0.048678
BARF1	ANXA11	0.1054
BARF1	PPP3CB	0.045324
BARF1	IQGAP1	0.031098
BARF1	MATN3	0.22743

BARF1	SH3KBP1	0.043003
BARF1	RET	0.083214
BARF1	CDH23	0.071721
BARF1	RAB11FIP4	0.29212
BARF1	SUV39H2	0.032156
BARF1	PLCE1	0.021068
BARF1	CLIC4	0.078459
BARF1	EIF4G1	0.021599
BARF1	SLC22A5	0.010222
BARF1	BNIP2	0.099716
BARF1	JAG2	0.13796
BARF1	EHD2	0.029638
BARF1	CADPS	0.23647
BARF1	PKD2	0.010019
BARF1	CCAR1	0.20142
BARF1	CRELD2	0.20501
BARF1	NBR1	0.022335
BARF1	GSN	0.084747
BARF1	SLC11A2	0.028026
BARF1	EIF4A1	0.020009
BARF1	CABP1	0.046565
BARF1	LETM1	0.21053
BARF1	PIK3CB	0.011891
BARF1	TFB1M	0.047438
BARF1	FAM188A	0.17613
BARF1	AP1B1	0.050626
BARF1	RCN3	0.23
BARF1	PLSCR1	0.095366
BARF1	SLC6A4	0.02386
BARF1	LTBP1	0.21037
BARF1	F2	0.051076
BARF1	SYT1	0.078967
BARF1	PI4K2A	0.046275
BARF1	NCS1	0.19029
BARF1	FBLN5	0.18508
BARF1	DAG1	0.047998
BARF1	DOC2A	0.012672
BARF1	NECAB3	0.14292
BARF1	MLC1	0.02674
BARF1	SNX6	0.058606
BARF1	NPC1L1	0.072769
BARF1	MEGF6	0.21261
BARF1	CALR	0.022522
BARF1	KCNIP2	0.08208
BARF1	PLCG1	0.011852
BARF1	CD93	0.16175
BARF1	GRIN1	0.071173
BARF1	CLINT1	0.031157
BARF1	DHH	0.14521
BARF1	SPHK2	0.042405
BARF1	AP1G2	0.010651
BARF1	MYL6B	0.1787
BARF1	ATP6V0A1	0.040857
BARF1	AP1G1	0.044023
BARF1	CDHR2	0.19188
BARF1	SNX17	0.011735
BARF1	CDH5	0.12501
BARF1	IL6R	0.036951
BARF1	SYT2	0.18963
BARF1	CAPNS1	0.067054
BARF1	TPD52	0.22274
BLLF2	KCT2	0.08834
BLLF2	EPC2	0.024588
BLLF2	CST9	0.015738
BLLF2	VN1R3	0.041316
BLLF2	RRP7A	0.056045
BLLF2	HERPUD1	0.10276
BLLF2	ubl4ab	0.13145

BLLF2	ZNF214	0.016009
BLLF2	DPY19L2P1	0.015516
BLLF2	ZNF804A	0.014966
BLLF2	PIP	0.020798
BLLF2	HID1	0.020499
BLLF2	PIBF1	0.12136
BLLF2	RPL36AL	0.017811
BLLF2	ZNF117	0.015884
BLLF2	ATPIF1	0.11429
BLLF2	BIN2	0.018765
BLLF2	MED1	0.012008
BLLF2	BHLHB9	0.035913
BLLF2	UBR3	0.020454
BLLF2	ZNF394	0.016887
BLLF2	AKAP14	0.1286
BLLF2	POLM	0.01204
BLLF2	G6PD	0.019232
BLLF2	PANK2	0.12807
BLLF2	SEC23B	0.02063
BLLF2	GAGE12I	0.0533
BLLF2	ZNF227	0.018711
BLLF2	PAGE2B	0.16542
BLLF2	ZNF682	0.013755
BLLF2	GDNF	0.037165
BLLF2	PTAR1	0.015041
BLLF2	GOLGA8DP	0.060478
BLLF2	SLC16A12	0.045306
BLLF2	LTV1	0.064163
BLLF2	NRIP2	0.060759
BLLF2	S100A7	0.012052
BLLF2	NANOS1	0.059741
BLLF2	TVP23A	0.072938
BLLF2	TMEM222	0.045489
BLLF2	CD300LF	0.013377
BLLF2	MICALCL	0.038229
BLLF2	G4	0.035586
BLLF2	PTTG2	0.070477
BLLF2	LAMTOR5	0.011179
BLLF2	GSPT2	0.014123
BLLF2	CHMP6	0.02029
BLLF2	SERTAD3	0.1584
BLLF2	PPP1R15B	0.11422
BLLF2	INO80B	0.12959
BLLF2	SHISA4	0.059563
BLLF2	RNMTL1	0.015704
BLLF2	gsg1l	0.018308
BLLF2	CENPW	0.053045
BLLF2	ZNF549	0.015625
BLLF2	DDI1	0.027916
BLLF2	UPF3A	0.14262
BLLF2	JSRP1	0.13943
BLLF2	CSRNP1	0.050116
BLLF2	ZNF721	0.011897
BLLF2	NPAP1	0.082521
BLLF2	RABEP1	0.13753
BLLF2	SKA3	0.04925
BLLF2	VPS54	0.025868
BLLF2	EIF5A	0.010181
BLLF2	CACNA2D1	0.01539
BLLF2	PDSS2	0.017435
BLLF2	MON1A	0.058761
BLLF2	ZNF695	0.013452
BLLF2	SPATS2L	0.042239
BLLF2	FAF1	0.012153
BLLF2	TMPRSS11BNL	0.065048
BLLF2	SLC5A1	0.042551
BLLF2	LRP2BP	0.016127
BLLF2	NUP85	0.015366

BLLF2	C12orf61	0.12444
BLLF2	FAM86B2	0.015735
BLLF2	IP6K2	0.043792
BLLF2	MAP7D3	0.033502
BLLF2	PNMA6D	0.018024
BLLF2	LRRC41	0.015266
BLLF2	MT1A	0.065486
BLLF2	PLSCR3	0.072413
BLLF2	DDX10	0.011433
BLLF2	TCF25	0.074473
BLLF2	CC2D1B	0.012058
BLLF2	N6AMT2	0.019902
BLLF2	PRSS16	0.14555
BLLF2	PLD6	0.010099
BLLF2	OR52N4	0.077843
BLLF2	ASXL3	0.041179
BLLF2	TRAT1	0.029435
BLLF2	FAM150A	0.04694
BLLF2	SLC5A4	0.011347
BLLF2	PLGRKT	0.057743
BLLF2	NRN1	0.070595
BLLF2	DYM	0.087878
BLLF2	ZC3HAV1L	0.11043
BLLF2	TCP10	0.078998
BLLF2	TIMD4	0.017336
BLLF2	STOML3	0.032447
BLLF2	ATP6V0D2	0.031866
BLLF2	VPREB3	0.050086
BLLF2	ORAI3	0.044393
BLLF2	NAP1L3	0.013502
BLLF2	CLC	0.019406
BLLF2	BTBD16	0.022739
BLLF2	C9orf40	0.013398
BLLF2	HSPA12A	0.016682
BLLF2	INTS1	0.042481
BLLF2	XKR7	0.12464
BLLF2	PRRT2	0.019198
BLLF2	OR2T2	0.086959
BLLF2	RPUSD4	0.068412
BLLF2	CMPK2	0.15488
BLLF2	CCDC159	0.076833
BLLF2	ASIC1	0.011846
BLLF2	PRR3	0.020759
BLLF2	ORC2	0.03068
BLLF2	C10orf68	0.093159
BLLF2	CEP85L	0.051836
BLLF2	EBNA1BP2	0.039725
BLLF2	TNIP3	0.015915
BLLF2	ZNF197	0.012141
BLLF2	CCDC42	0.01619
BLLF2	ZNF610	0.010271
BLLF2	SLC4A11	0.01261
BLLF2	TRAPPC2	0.017591
BLLF2	NIP7	0.027627
BLLF2	C16orf70	0.010901
BLLF2	NAT16	0.16314
BLLF2	ANAPC5	0.049863
BLLF2	CCDC162P	0.013726
BLLF2	TSPAN32	0.042793
BLLF2	LIPT2	0.048036
BLLF2	MSL2	0.020649
BLLF2	GOLGA6L1	0.046271
BLLF2	LPIN1	0.01221
BLLF2	SPRR1A	0.079313
BLLF2	WDPCP	0.049256
BLLF2	USHBP1	0.021903
BLLF2	HMGCR	0.013086
BLLF2	LALBA	0.015741

BLLF2	MCM3AP	0.033389
BLLF2	EFCAB5	0.011581
BLLF2	CARD6	0.025478
BLLF2	TARSL2	0.033954
BLLF2	GRHL2	0.010124
BLLF2	FXYD4	0.13089
BLLF2	HIST2H2BE	0.015213
BLLF2	TMEM119	0.058554
BLLF2	TMEM132C	0.097013
BLLF2	ZNF606	0.016917
BLLF2	TEX12	0.05849
BLLF2	ASNA1	0.025889
BLLF2	HKR1	0.016417
BLLF2	KRI1	0.12583
BLLF2	SPATA1	0.023117
BLLF2	CARTPT	0.021612
BLLF2	C4orf32	0.016865
BLLF2	CCDC69	0.040463
BLLF2	MKS1	0.072085
BLLF2	IGFL2	0.18536
BLLF2	RIOK1	0.01156
BLLF2	DTX1	0.016017
BLLF2	BLVRA	0.093373
BLLF2	CCDC109B	0.096441
BLLF2	SPHKAP	0.034187
BLLF2	PXMP2	0.043081
BLLF2	STMN4	0.015563
BLLF2	IRAK1BP1	0.093601
BLLF2	ZPBP2	0.062282
BLLF2	THRAP3	0.057804
BLLF2	MTERFD1	0.083617
BLLF2	VWA3A	0.043778
BLLF2	EIF3J	0.043934
BLLF2	RTN2	0.012103
BLLF2	SLC31A2	0.030935
BLLF2	ZDHHC11	0.012435
BLLF2	ACRC	0.036291
BLLF2	ARSB	0.015933
BLLF2	TAS2R13	0.07066
BLLF2	TTLL12	0.02143
BLLF2	PRDM14	0.013906
BLLF2	GNG10	0.021683
BLLF2	PYCRL	0.020138
BLLF2	B3GAT2	0.012899
BLLF2	EVA1A	0.042517
BLLF2	C10orf62	0.083871
BLLF2	ARV1	0.027467
BLLF2	PRKRIR	0.012324
BLLF2	NOC3L	0.015469
BLLF2	PDF	0.091529
BLLF2	WASH3P	0.017634
BLLF2	AKAP11	0.043916
BLLF2	UNC119B	0.082189
BLLF2	MBTD1	0.010717
BLLF2	ABHD1	0.030277
BLLF2	YMR013W-A	0.098442
BLLF2	SLC39A4	0.027494
BLLF2	CCNB1IP1	0.010001
BLLF2	GFRAL	0.026167
BLLF2	ATG4D	0.052872
BLLF2	TCRA	0.023207
BLLF2	ZNF624	0.011494
BLLF2	BTBD10	0.022244
BLLF2	CASC3	0.15225
BLLF2	CHIC1	0.07041
BLLF2	CYP4F30P	0.016289
BLLF2	NKAIN3	0.10117
BLLF2	CD58	0.016793

BLLF2	AHCTF1	0.020405
BLLF2	MAP2	0.01105
BLLF2	IER3IP1	0.062387
BLLF2	DOPEY1	0.02138
BLLF2	ATP6V1B2	0.014307
BLLF2	TFDP1	0.012927
BLLF2	QARS	0.011795
BLLF2	RPS18	0.017164
BLLF2	ZFP64	0.014046
BLLF2	HBG2	0.10713
BLLF2	TCIRG1	0.013392
BLLF2	CCM2L	0.0494
BLLF2	MAN1B1	0.0577
BLLF2	TRMT6	0.1594
BLLF2	TBCC	0.019588
BLLF2	VWA7	0.11586
BLLF2	LAMP3	0.035555
BLLF2	SLC16A2	0.022773
BLLF2	SPATC1	0.069887
BLLF2	COLGALT2	0.012578
BLLF2	ZNF22	0.01391
BLLF2	MLH1	0.015707
BLLF2	CHRD	0.010771
BLLF2	ZC3H12C	0.034625
BLLF2	BRINP2	0.036406
BLLF2	ATP5B	0.031359
BLLF2	CLDN16	0.016703
BLLF2	NIN	0.14965
BLLF2	PRL	0.010028
BLLF2	NUP93	0.042909
BLLF2	TXNDC17	0.033547
BLLF2	MOCS2	0.051912
BLLF2	LYRM1	0.011036
BLLF2	POLR2G	0.025296
BLLF2	RTN3	0.010062
BLLF2	FGFBP3	0.12025
BLLF2	MTO1	0.17677
BLLF2	TNRC6C	0.046136
BLLF2	ZC3HC1	0.061097
BLLF2	ZNF43	0.013082
BLLF2	NACA2	0.036339
BLLF2	ILDR2	0.075463
BLLF2	PGM3	0.11609
BLLF2	B9D1	0.045536
BLLF2	STIM2	0.01264
BLLF2	MOB3C	0.011211
BLLF2	KIN	0.088744
BLLF2	SRP68	0.023037
BLLF2	HUS1B	0.075382
BLLF2	WBP2	0.09078
BLLF2	NUP107	0.011358
BLLF2	EID2B	0.11663
BLLF2	TMEM2	0.033547
BLLF2	PLBD2	0.048319
BLLF2	TIGD2	0.04209
BLLF2	KCTD12	0.014253
BLLF2	EEFSEC	0.052027
BLLF2	CABYR	0.11266
BLLF2	TRMT1L	0.026723
BLLF2	ERVK6	0.016256
BLLF2	SLAIN2	0.051126
BLLF2	TAS2R50	0.039451
BLLF2	EIF4E3	0.014727
BLLF2	GPAT2	0.024622
BLLF2	FDPS	0.016286
BLLF2	SSSCA1	0.074479
BLLF2	CCDC130	0.041085
BLLF2	BNIPL	0.010799

BLLF2	NUP54	0.058801
BLLF2	MCMDC2	0.039369
BLLF2	CTCFL	0.013322
BLLF2	NUS1	0.019109
BLLF2	ZNF347	0.012141
BLLF2	RPLP1	0.05409
BLLF2	DERL3	0.044974
BLLF2	ZNF696	0.020343
BLLF2	LY6G6F	0.049438
BLLF2	ZNF266	0.012438
BLLF2	CD247	0.010765
BLLF2	MAN1A2	0.03425
BLLF2	SLC23A2	0.053553
BLLF2	ZBTB47	0.024494
BLLF2	FASTKD3	0.022038
BLLF2	SSX6	0.051573
BLLF2	NDUFAF4	0.024708
BLLF2	OTOA	0.033225
BLLF2	THOC5	0.025414
BLLF2	DPY30	0.031855
BLLF2	SLC26A6	0.01159
BLLF2	OR2Z1	0.089905
BLLF2	LRIF1	0.023126
BLLF2	CRYBA4	0.041667
BLLF2	ZNF587B	0.020775
BLLF2	LDB2	0.027039
BLLF2	ANGEL1	0.041244
BLLF2	SLC39A9	0.033565
BLLF2	TMEM30B	0.030381
BLLF2	NUDCD1	0.010903
BLLF2	DDX55	0.010595
BLLF2	PITPNM2	0.023748
BLLF2	C19orf44	0.044733
BLLF2	C15orf40	0.01279
BLLF2	MIA3	0.016338
BLLF2	OR4K14	0.089683
BLLF2	NKAIN1	0.1337
BLLF2	CKM	0.017671
BLLF2	SLAMF7	0.06201
BLLF2	FSIP1	0.10617
BLLF2	SYPL2	0.033909
BLLF2	C14orf1	0.061694
BLLF2	ZNF479	0.020209
BLLF2	COMMD5	0.1092
BLLF2	LGALS7	0.013267
BLLF2	WIPF1	0.048205
BLLF2	AANAT	0.076113
BLLF2	TSGA10IP	0.098928
BLLF2	FTL	0.013851
BLLF2	CD81	0.018015
BLLF2	ST8SIA3	0.025107
BLLF2	TNFAIP8L3	0.034077
BLLF2	SLC30A5	0.012407
BLLF2	MT-ND4	0.079002
BLLF2	GBGT1	0.065562
BLLF2	NBPF15	0.14725
BLLF2	ZNF609	0.015963
BLLF2	ATRIP	0.09946
BLLF2	TBC1D21	0.035997
BLLF2	NDUFAB1	0.062356
BLLF2	CCDC136	0.047935
BLLF2	ZNF134	0.010459
BLLF2	ZNF826P	0.012483
BLLF2	PREP	0.062546
BLLF2	TFCP2L1	0.016262
BLLF2	LAMTOR1	0.044273
BLLF2	DHPS	0.062735
BLLF2	IL12A	0.052235

BLLF2	ARMCX4	0.10366
BLLF2	THAP1	0.041755
BLLF2	COX6B1	0.01762
BLLF2	CLP1	0.016919
BLLF2	POP4	0.12953
BLLF2	C10orf35	0.14207
BLLF2	MCAT	0.022647
BLLF2	CACNG7	0.012447
BLLF2	APOL2	0.059143
BLLF2	LENEP	0.093696
BLLF2	ZNF322	0.015562
BLLF2	TRDC	0.01223
BLLF2	ZNF205	0.018565
BLLF2	NME5	0.015238
BLLF2	CNOT3	0.046349
BLLF2	TM2D3	0.1075
BLLF2	MFAP2	0.1302
BLLF2	TMEM114	0.011703
BLLF2	BCL9	0.075318
BLLF2	TSNAX	0.055546
BLLF2	TADA3	0.028042
BLLF2	ITPKC	0.1025
BLLF2	LSM3	0.036973
BLLF2	DDO	0.013599
BLLF2	PRAMEF9	0.03093
BLLF2	PEA15	0.0164
BLLF2	RPS27L	0.033558
BLLF2	UXT	0.058023
BLLF2	TRAPPC4	0.040034
BLLF2	TAS2R43	0.049325
BLLF2	POLR3D	0.043167
BLLF2	FBXO7	0.070602
BLLF2	WDR93	0.078007
BLLF2	SPBC1539.02	0.053498
BLLF2	LYZL2	0.073565
BLLF2	CEP152	0.039914
BLLF2	OCSTAMP	0.025173
BLLF2	XPO7	0.066465
BLLF2	SPATA31A2	0.071981
BLLF2	H1FNT	0.067577
BLLF2	HEXIM1	0.14618
BLLF2	ATXN10	0.076558
BLLF2	BCMO1	0.010034
BLLF2	TRAPPC6B	0.061914
BLLF2	SC5D	0.020608
BLLF2	COMMD7	0.084735
BLLF2	DUOXA1	0.094009
BLLF2	ECI1	0.0108
BLLF2	ZNF355P	0.019687
BLLF2	ATG4B	0.084362
BLLF2	MAL2	0.066916
BLLF2	EEF1A1	0.011346
BLLF2	ZFAND3	0.031596
BLLF2	ITIH5	0.013358
BLLF2	HIST2H2BD	0.023068
BLLF2	RPS15	0.030666
BLLF2	MEI4	0.072799
BLLF2	FAM187B	0.011942
BLLF2	ATP6V1B1	0.013963
BLLF2	SEC24B	0.011419
BLLF2	GLTSCR1L	0.15703
BLLF2	MED8	0.023432
BLLF2	ZNF891	0.011033
BLLF2	LDOC1	0.016603
BLLF2	HIST1H1A	0.014442
BLLF2	FAM21D	0.082584
BLLF2	TSR2	0.084702
BLLF2	CLDN22	0.028093

BLLF2	MAB21L3	0.017135
BLLF2	PEX19	0.050976
BLLF2	ZNF200	0.013339
BLLF2	TMEM135	0.12446
BLLF2	AUP1	0.011142
BLLF2	SLC34A2	0.12361
BLLF2	SNRPG	0.013798
BLLF2	MUC20	0.12378
BLLF2	HR	0.018039
BLLF2	TECPR1	0.098207
BLLF2	GTPBP1	0.14517
BLLF2	ZNF486	0.014927
BLLF2	SERINC3	0.066262
BLLF2	PMS2P2	0.020081
BLLF2	NPEPL1	0.01782
BLLF2	GOLGA4	0.049226
BLLF2	MALL	0.053961
BLLF2	CHMP4B	0.087046
BLLF2	SELV	0.1346
BLLF2	ZNF320	0.015884
BLLF2	IGKV1-5	0.063187
BLLF2	DKC1	0.064342
BLLF2	CHMP4BP1	0.045165
BLLF2	RPS4Y1	0.05126
BLLF2	BTG4	0.038457
BLLF2	EXOC1	0.057873
BLLF2	ASIC3	0.012575
BLLF2	TMPO	0.064947
BLLF2	PSMD2	0.028317
BLLF2	NR2C2AP	0.01541
BLLF2	smf	0.033783
BLLF2	ATP6V1G2	0.060115
BLLF2	NRIP3	0.060391
BLLF2	MAGEB1	0.016066
BLLF2	ZNF714	0.012142
BLLF2	CDKN1A	0.010801
BLLF2	TMEM132A	0.066796
BLLF2	REEP4	0.015531
BLLF2	PGAM1	0.08885
BLLF2	CAPZB	0.018004
BLLF2	DMRTB1	0.025277
BLLF2	POLG	0.060417
BLLF2	ZSCAN5C	0.028125
BLLF2	MGST3	0.030669
BLLF2	CMTM3	0.086134
BLLF2	PSMF1	0.059016
BLLF2	ZFP30	0.014054
BLLF2	POPDC3	0.034324
BLLF2	THEM4	0.048478
BLLF2	PRAMEF8	0.010179
BLLF2	PRAMEF4	0.034248
BLLF2	C21orf67	0.17504
BLLF2	ZBTB45	0.012285
BLLF2	STOML2	0.019502
BLLF2	UBAP2	0.12409
BLLF2	PKM	0.014648
BLLF2	GFRA2	0.059148
BLLF2	MTRNR2L4	0.10682
BLLF2	ZNF678	0.023185
BLLF2	TNNT1	0.067451
BLLF2	ZNF852	0.015927
BLLF2	MRPS2	0.033158
BLLF2	MFF	0.012571
BLLF2	ZNF766	0.018125
BLLF2	APOBEC3B	0.030937
BLLF2	XKR4	0.12464
BLLF2	C17orf105	0.045268
BLLF2	ANO8	0.060976

BLLF2	MFSD12	0.024591
BLLF2	G4	0.035696
BLLF2	CCK	0.014764
BLLF2	SATL1	0.024723
BLLF2	FAM110D	0.1063
BLLF2	SDR39U1	0.035347
BLLF2	NUMA1	0.028486
BLLF2	MED23	0.014443
BLLF2	PRAMEF1	0.10064
BLLF2	VWA8	0.016662
BLLF2	AGBL5	0.034433
BLLF2	TSPAN19	0.013932
BLLF2	MS4A4E	0.043534
BLLF2	LEPROT	0.018152
BLLF2	EGLN1	0.021532
BLLF2	ZFP14	0.013114
BLLF2	TMEM178A	0.041049
BLLF2	VAC14	0.012421
BLLF2	HSPB11	0.012576
BLLF2	IFRD1	0.058764
BLLF2	ESCO2	0.023836
BLLF2	FSCB	0.044101
BLLF2	TEKT3	0.023137
BLLF2	ZNF628	0.014943
BLLF2	LMF1	0.095921
BLLF2	PPP6R1	0.025472
BLLF2	FRS2	0.011369
BLLF2	SLC13A4	0.030755
BLLF2	PAIP1	0.024496
BLLF2	MORF4L1	0.012463
BLLF2	TBC1D3H	0.013952
BLLF2	ADSS	0.029118
BLLF2	SELPLG	0.15825
BLLF2	PYGO2	0.011627
BLLF2	YTHDF3	0.10348
BLLF2	ZDBF2	0.036596
BLLF2	UBXN7	0.019516
BLLF2	LVP1a	0.10059
BLLF2	NECAB1	0.01767
BLLF2	PLXDC2	0.046883
BLLF2	BLOC1S3	0.1124
BLLF2	AMOT	0.034818
BLLF2	DNAAF2	0.18075
BLLF2	CCDC154	0.031898
BLLF2	ZNF525	0.011194
BLLF2	MAATS1	0.051044
BLLF2	PMM2	0.031679
BLLF2	ALOX5AP	0.035045
BLLF2	HIST1H1C	0.01566
BLLF2	SLC4A1	0.029456
BLLF2	CARNS1	0.022507
BLLF2	PAGE5	0.089625
BLLF2	TOB1	0.043617
BLLF2	kup	0.010589
BLLF2	CLPTM1L	0.09744
BLLF2	SFMBT2	0.012671
BLLF2	NPL	0.065064
BLLF2	BORA	0.074194
BLLF2	PLAC1	0.066973
BLLF2	ZNF317	0.02154
BLLF2	MAN1C1	0.02942
BLLF2	UGCG	0.068693
BLLF2	VPS28	0.023835
BLLF2	EEF1A1P5	0.011059
BLLF2	ZFP57	0.021489
BLLF2	URM1	0.066846
BLLF2	CABS1	0.20758
BLLF2	GPR89A	0.041589

BLLF2	NAPB	0.02508
BLLF2	HHIPL2	0.021187
BLLF2	MAGEA2	0.013768
BLLF2	TRAK2	0.025167
BLLF2	GAGE10	0.090422
BLLF2	SLC16A13	0.060205
BLLF2	CASP8AP2	0.093599
BLLF2	FNTB	0.033427
BLLF2	LINC00474	0.097461
BLLF2	MORC1	0.047469
BLLF2	PTOV1	0.034711
BLLF2	CLDN11	0.02677
BLLF2	CRTC1	0.059806
BLLF2	VEZT	0.18836
BLLF2	SEC62	0.062235
BLLF2	POLR1A	0.010615
BLLF2	TIMM22	0.010717
BLLF2	GNAS	0.046733
BLLF2	BOP	0.010451
BLLF2	DENND1C	0.014078
BLLF2	CLPX	0.08183
BLLF2	FAM117B	0.15564
BLLF2	FAM90A13P	0.01907
BLLF2	GLS	0.014547
BLLF2	HAUS6	0.045814
BLLF2	OR2M3	0.07986
BLLF2	ZNF608	0.061403
BLLF2	EIF4G2	0.032532
BLLF2	LCN1P1	0.012645
BLLF2	LUZP6	0.026485
BLLF2	HELB	0.015007
BLLF2	GLYATL1	0.047653
BLLF2	AKIRIN2	0.1093
BLLF2	KCNK1	0.017293
BLLF2	MT1M	0.015452
BLLF2	ZNF814	0.018774
BLLF2	ZNF223	0.028573
BLLF2	FAM90A8P	0.019086
BLLF2	ORAI1	0.046941
BLLF2	RPRD1A	0.030828
BLLF2	TSFM	0.070247
BLLF2	RAD21	0.018241
BLLF2	LVP1a	0.18936
BLLF2	UCK1	0.013883
BLLF2	ZNF569	0.024153
BLLF2	PHYHIP	0.02348
BLLF2	METTL1	0.10887
BLLF2	MRFAP1	0.14279
BLLF2	MMS19	0.043001
BLLF2	CDC42EP4	0.051072
BLLF2	SYN1	0.037154
BLLF2	RPL11	0.041766
BLLF2	NCBP1	0.018663
BLLF2	ZNF177	0.024724
BLLF2	GNRH2	0.02075
BLLF2	EDC3	0.045903
BLLF2	WFDC3	0.014017
BLLF2	PI3	0.021992
BLLF2	C12orf68	0.044298
BLLF2	NRSN2	0.017149
BLLF2	SAMD4B	0.042054
BLLF2	LYZL4	0.028137
BLLF2	GAGE3	0.029493
BLLF2	ZSCAN5B	0.011707
BLLF2	GDI2	0.016077
BLLF2	GADD45B	0.072964
BLLF2	TMED6	0.019779
BLLF2	MESDC1	0.03115

BLLF2	IFNB1	0.018069
BLLF2	TSPYL6	0.012924
BLLF2	R3HCC1	0.027944
BLLF2	ARSD	0.015208
BLLF2	C10orf91	0.16385
BLLF2	PM20D2	0.034007
BLLF2	CNPPD1	0.015877
BLLF2	PENK	0.048333
BLLF2	KCNJ14	0.014436
BLLF2	DMRTA1	0.024694
BLLF2	IGHV1OR21-1	0.030859
BLLF2	LGALS4	0.020781
BLLF2	NCOA7	0.036003
BLLF2	Gm5153	0.020436
BLLF2	ZNF76	0.010662
BLLF2	TMEM8C	0.018071
BLLF2	MUT	0.064644
BLLF2	GK3P	0.097643
BLLF2	SMARCD1	0.010773
BLLF2	APOBEC3G	0.017663
BLLF2	DYNLT3	0.016816
BLLF2	CLDN14	0.015752
BLLF2	CCDC28A	0.17988
BLLF2	MEMO1	0.043852
BLLF2	ZC3H18	0.077267
BLLF2	HP1BP3	0.011299
BLLF2	RPF2	0.085652
BLLF2	TTC22	0.01601
BLLF2	MRPL46	0.07488
BLLF2	CEP170P1	0.042331
BLLF2	P4HA1	0.044876
BLLF2	ERCC4	0.011406
BLLF2	CYBA	0.029353
BLLF2	ZNF572	0.01766
BLLF2	SSBP2	0.060506
BLLF2	TNFAIP8	0.02779
BLLF2	GNPTG	0.024487
BLLF2	ZNF440	0.024417
BLLF2	TIGAR	0.062271
BLLF2	ZNF490	0.027087
BLLF2	YPEL3	0.036973
BLLF2	LMAN2L	0.055824
BLLF2	LDB1	0.031082
BLLF2	XRCC6	0.02361
BLLF2	TPP2	0.011872
BLLF2	SPEF1	0.086321
BLLF2	NADK2	0.040771
BLLF2	PIK3R5	0.020581
BLLF2	GPIHBP1	0.031057
BLLF2	SF3B5	0.064038
BLLF2	RCAN3	0.027562
BLLF2	ICA1L	0.052515
BLLF2	FAM120C	0.027418
BLLF2	UPF2	0.021487
BLLF2	NECAP1	0.11674
BLLF2	OAZ2	0.11613
BLLF2	RAD21L1	0.014445
BLLF2	LVP1a	0.034644
BLLF2	HIC2	0.010067
BLLF2	ZNF142	0.013435
BLLF2	CNNM2	0.046412
BLLF2	MAGEA9	0.022534
BLLF2	LOXL1	0.030278
BLLF2	MIR17HG	0.17949
BLLF2	CCDC65	0.015093
BLLF2	GPRC5B	0.010187
BLLF2	CNIH1	0.012805
BLLF2	RMDN2	0.022337

BLLF2	SECTM1	0.16085
BLLF2	SERTAD2	0.10879
BLLF2	EIF4ENIF1	0.015059
BLLF2	NME7	0.048196
BLLF2	BRMS1L	0.017587
BLLF2	SAAL1	0.023494
BLLF2	HBE1	0.10713
BLLF2	NREP	0.031792
BLLF2	HORMAD1	0.061835
BLLF2	ZNF461	0.011372
BLLF2	ADCK2	0.020499
BLLF2	PSMB9	0.013505
BLLF2	LGSN	0.015353
BLLF2	ZNF772	0.012673
BLLF2	CP	0.014913
BLLF2	RTFDC1	0.056244
BLLF2	PNPLA5	0.028839
BLLF2	UPK1A	0.015861
BLLF2	ZNF316	0.018786
BLLF2	GID4	0.057032
BLLF2	GTF3A	0.012523
BLLF2	MTRNR2L10	0.10682
BLLF2	CMTM5	0.10972
BLLF2	PTS	0.019267
BLLF2	PPP1R10	0.11674
BLLF2	ST3GAL1	0.013035
BLLF2	EVA1B	0.13365
BLLF2	FAM83F	0.096414
BLLF2	DNAJA4	0.017182
BLLF2	IFITM2	0.017487
BLLF2	LUZP4	0.075615
BLLF2	CREBZF	0.017699
BLLF2	NCR3	0.04106
BLLF2	FAM101A	0.077225
BLLF2	RNASE2	0.014837
BLLF2	SLC5A2	0.015192
BLLF2	SLC24A5	0.012659
BLLF2	ACIN1	0.012258
BLLF2	CKB	0.057743
BLLF2	IFNL3	0.01855
BLLF2	SOAT2	0.015456
BLLF2	NCCRP1	0.082451
BLLF2	CYLC1	0.029738
BLLF2	SNURFL	0.066652
BLLF2	OR5F1	0.085662
BLLF2	OR2T5	0.084698
BLLF2	KIAA0040	0.0208
BLLF2	SPATC1L	0.084334
BLLF2	ANO4	0.039805
BLLF2	G4	0.041759
BLLF2	ZFR	0.01948
BLLF2	RTP4	0.048427
BLLF2	EEF1D	0.031856
BLLF2	TRADD	0.01567
BLLF2	PYGO1	0.012456
BLLF2	PSMD3	0.012032
BLLF2	MTERF	0.046793
BLLF2	ATG13	0.14319
BLLF2	MED19	0.086994
BLLF2	ZDHHC7	0.010168
BLLF2	ANO5	0.026375
BLLF2	PPAPDC2	0.022473
BLLF2	TTC19	0.011157
BLLF2	MT2A	0.050936
BLLF2	ORM1	0.022183
BLLF2	NAIF1	0.052744
BLLF2	CNP	0.013295
BLLF2	IFNA6	0.033273

BLLF2	TCEAL6	0.058208
BLLF2	ZNF528	0.01293
BLLF2	G4	0.022713
BLLF2	CLSPN	0.072971
BLLF2	FAM178A	0.095301
BLLF2	UPK3BL	0.086139
BLLF2	MAFG	0.032608
BLLF2	SPDL1	0.036197
BLLF2	ZSCAN10	0.015997
BLLF2	GOLGA8F	0.074477
BLLF2	RTP1	0.041999
BLLF2	ASTL	0.027438
BLLF2	SPAG7	0.04983
BLLF2	KCNMB3	0.050504
BLLF2	OR51S1	0.087398
BLLF2	MEN1	0.035743
BLLF2	SOBP	0.042284
BLLF2	ZNF806	0.019544
BLLF2	CLDN3	0.02677
BLLF2	CIDEB	0.050395
BLLF2	ZBTB14	0.010293
BLLF2	BEX2	0.13285
BLLF2	SCAMP1	0.011388
BLLF2	SEC23A	0.028071
BLLF2	BCKDHB	0.046339
BLLF2	RNMT	0.030906
BLLF2	C12orf36	0.037897
BLLF2	CTAGE6	0.018636
BLLF2	TBATA	0.13466
BLLF2	TAS2R4	0.064691
BLLF2	AK5	0.011727
BLLF2	FXYD1	0.087069
BLLF2	SCAND2P	0.011945
BLLF2	DDX47	0.022566
BLLF2	TCEB3CL	0.038068
BLLF2	ZNF286B	0.011537
BLLF2	LMAN1L	0.025897
BLLF2	TMEM92	0.092946
BLLF2	Gm5153	0.015978
BLLF2	GAGE2B	0.0533
BLLF2	TOMM22	0.035346
BLLF2	MAGOH	0.010874
BLLF2	GBP1	0.011132
BLLF2	OR2T35	0.10108
BLLF2	SCARA3	0.030023
BLLF2	SRGN	0.10893
BLLF2	CACNA2D2	0.011948
BLLF2	RPUSD3	0.028056
BLLF2	PLIN2	0.020947
BLLF2	TNFAIP1	0.016135
BLLF2	NUDT15	0.012068
BLLF2	AKAP4	0.065111
BLLF2	OTUD6B	0.017319
BLLF2	BRK1	0.061639
BLLF2	C19orf68	0.046212
BLLF2	ACRBP	0.24708
BLLF2	NOL3	0.11034
BLLF2	ZSCAN2	0.016896
BLLF2	FAM83C	0.051942
BLLF2	MAGED1	0.014921
BLLF2	EPPK1	0.015548
BLLF2	IQCK	0.093239
BLLF2	ERVK6	0.014855
BLLF2	ITFG2	0.015911
BLLF2	CXXC5	0.016023
BLLF2	ZNF181	0.013394
BLLF2	ZNF776	0.025248
BLLF2	EXT1	0.011055

BLLF2	IP6K1	0.065236
BLLF2	HAT1	0.078895
BLLF2	TRAPPC3L	0.02089
BLLF2	FAM166A	0.10231
BLLF2	ZNF649	0.021434
BLLF2	NGFRAP1	0.070127
BLLF2	PGS1	0.011448
BLLF2	TLDC1	0.047638
BLLF2	COMMD3	0.057582
BLLF2	ADAD2	0.02818
BLLF2	CDC37L1	0.027283
BLLF2	CDK5R2	0.050133
BLLF2	MAD2L1	0.012912
BLLF2	TAS2R46	0.062545
BLLF2	PRRC2B	0.12934
BLLF2	LZIC	0.011356
BLLF2	XKRY	0.1416
BLLF2	BYSL	0.048779
BLLF2	SPRTN	0.026615
BLLF2	SFSWAP	0.076739
BLLF2	MUM1L1	0.092362
BLLF2	LSM5	0.013583
BLLF2	CTC1	0.01066
BLLF2	SPPL2A	0.049191
BLLF2	CACYBP	0.020328
BLLF2	FAM74A4	0.041725
BLLF2	COBLL1	0.048847
BLLF2	SIAH3	0.020529
BLLF2	UBLCP1	0.031354
BLLF2	RPP14	0.045972
BLLF2	HUS1	0.025068
BLLF2	WHAMM	0.026208
BLLF2	APLF	0.014551
BLLF2	ZNF300P1	0.011551
BLLF2	LZTS1	0.036739
BLLF2	ZNF275	0.01737
BLLF2	OR2B11	0.087261
BLLF2	TCTN2	0.051086
BLLF2	PEX11G	0.11418
BLLF2	OGDHL	0.010269
BLLF2	DISC1	0.019276
BLLF2	IFNA5	0.04109
BLLF2	LTC4S	0.042643
BLLF2	AP3S1	0.020657
BLLF2	DENND6A	0.05795
BLLF2	ZNF818P	0.010442
BLLF2	LYZL6	0.078057
BLLF2	DNAJA1	0.013486
BLLF2	AFF4	0.059853
BLLF2	DNASE1L1	0.1022
BLLF2	MGP	0.035774
BLLF2	TKTL2	0.016868
BLLF2	BCKDHA	0.012611
BLLF2	ZBTB41	0.02013
BLLF2	FBXL22	0.074871
BLLF2	NACAP1	0.032201
BLLF2	KRTAP2-2	0.052528
BLLF2	TAF11	0.073167
BLLF2	SYCE1	0.010202
BLLF2	LAMTOR2	0.048832
BLLF2	DDHD1	0.028943
BLLF2	HIST1H1E	0.01681
BLLF2	CDC45	0.050367
BLLF2	GLB1L	0.01907
BLLF2	YIPF5	0.099377
BLLF2	CHST12	0.017174
BLLF2	UBL5	0.035073
BLLF2	EBAG9	0.058387

BLLF2	NSUN4	0.094727
BLLF2	BEAN1	0.089938
BLLF2	EID3	0.12084
BLLF2	SPACA3	0.063257
BLLF2	HHAT	0.13073
BLLF2	INSIG2	0.01776
BLLF2	SOSTDC1	0.01831
BLLF2	CDCA8	0.022889
BLLF2	GLTP	0.052448
BLLF2	GPATCH1	0.11978
BLLF2	FAM9A	0.097156
BLLF2	SAA4	0.014591
BLLF2	RRN3	0.02664
BLLF2	DTNBP1	0.032059
BLLF2	TAS2R60	0.061704
BLLF2	PNMA2	0.019641
BLLF2	CGB1	0.021509
BLLF2	BPIFB3	0.020313
BLLF2	PLIN3	0.018608
BLLF2	VPS18	0.012475
BLLF2	ZNF621	0.024354
BLLF2	CXorf67	0.16942
BLLF2	TSPAN12	0.0115
BLLF2	NPW	0.059134
BLLF2	RMND1	0.01352
BLLF2	C18orf25	0.056104
BLLF2	ZNF417	0.017221
BLLF2	TPGS1	0.014212
BLLF2	DEFB125	0.027794
BLLF2	A2M	0.013591
BLLF2	FCER1G	0.048266
BLLF2	LARP4B	0.016981
BLLF2	THAP8	0.030578
BLLF2	ISY1	0.10028
BLLF2	CLTB	0.024287
BLLF2	DNAJB12	0.013082
BLLF2	OR2T29	0.084698
BLLF2	C11orf35	0.017494
BLLF2	NAGS	0.071156
BLLF2	SEC11A	0.098525
BLLF2	C16orf86	0.02644
BLLF2	MCC	0.032129
BLLF2	HIST2H2BF	0.015213
BLLF2	ZNF777	0.021402
BLLF2	LAMTOR3	0.013657
BLLF2	ELOVL4	0.092967
BLLF2	FAM188B2	0.17707
BLLF2	OR2I1P	0.087261
BLLF2	GALNT4	0.018718
BLLF2	GTF3C1	0.071232
BLLF2	WTAP	0.070208
BLLF2	EIF1AX	0.12153
BLLF2	ytzL	0.017064
BLLF2	DMAP1	0.076194
BLLF2	DPP3	0.067073
BLLF2	ZSCAN9	0.012748
BLLF2	ZNF551	0.015996
BLLF2	APH1A	0.037359
BLLF2	RSBN1L	0.069723
BLLF2	GG1	0.091434
BLLF2	FXYD6P3	0.065641
BLLF2	CTAG2	0.096998
BLLF2	ZSWIM3	0.073948
BLLF2	AKIP1	0.085627
BLLF2	ZNF263	0.013039
BLLF2	POLR2D	0.044004
BLLF2	SLC26A10	0.011841
BLLF2	ZNF627	0.021902

BLLF2	SF3B2	0.089454
BLLF2	MEI1	0.067791
BLLF2	CXorf23	0.035244
BLLF2	SCGB1D1	0.072399
BLLF2	OR4N2	0.087261
BLLF2	TNNI1	0.028829
BLLF2	NOP58	0.035629
BLLF2	ATP6V1E1	0.037581
BLLF2	SPRR1B	0.079313
BLLF2	TCEB3	0.037241
BLLF2	THEMIS	0.044767
BLLF2	ZNF444	0.032272
BLLF2	AP3M2	0.010574
BLLF2	FLVCR2	0.031708
BLLF2	NUDCD3	0.068237
BLLF2	ZFAND1	0.025628
BLLF2	ZNF646	0.011115
BLLF2	MAGEF1	0.022534
BLLF2	ZNF574	0.015475
BLLF2	ZNF433	0.012141
BLLF2	DIS3	0.010221
BLLF2	AMY2B	0.030931
BLLF2	GSTCD	0.019319
BLLF2	UBR2	0.015078
BLLF2	ZNF175	0.017647
BLLF2	CLUH	0.028125
BLLF2	UNC93A	0.073263
BLLF2	FAM69C	0.1972
BLLF2	G4	0.083419
BLLF2	TBC1D3F	0.012955
BLLF2	PNMA5	0.024269
BLLF2	PANK4	0.015126
BLLF2	ZNF578	0.015243
BLLF2	XKRY2	0.12802
BLLF2	MS4A2	0.038843
BLLF2	POC5	0.021537
BLLF2	ARL2BP	0.040158
BLLF2	SPACA4	0.04415
BLLF2	NRTN	0.024112
BLLF2	EFNB2	0.010883
BLLF2	PFN1	0.018327
BLLF2	POMT1	0.031974
BLLF2	FITM1	0.098622
BLLF2	ZNF75D	0.011529
BLLF2	ATXN3	0.016115
BLLF2	DEDD2	0.10224
BLLF2	CAMSAP1	0.0357
BLLF2	CST9L	0.018109
BLLF2	TAS2R38	0.07066
BLLF2	ZNF623	0.01552
BLLF2	NPIPB5	0.010348
BLLF2	CDC42EP2	0.018745
BLLF2	OR2M5	0.07986
BLLF2	ZNF616	0.012141
BLLF2	METTL2B	0.015087
BLLF2	MSH4	0.021107
BLLF2	KIAA1407	0.056633
BLLF2	TCTEX1D4	0.037899
BLLF2	FAM184B	0.039989
BLLF2	ZFP92	0.017763
BLLF2	TMCC2	0.045411
BLLF2	SEC61A1	0.063011
BLLF2	MAT1A	0.010677
BLLF2	SLC4A8	0.023821
BLLF2	SPACA7	0.086594
BLLF2	TEAD3	0.02038
BLLF2	BET1L	0.010484
BLLF2	ZNF679	0.013157

BLLF2	LPCAT3	0.057459
BLLF2	POLDIP2	0.12897
BLLF2	CCSER1	0.1172
BLLF2	UEVLD	0.015987
BLLF2	CDC42EP5	0.051072
BLLF2	ZNF484	0.0157
BLLF2	ALG1L2	0.010926
BLLF2	ZNF771	0.011348
BLLF2	TOR1A	0.013788
BLLF2	CLTA	0.049288
BLLF2	TCEAL4	0.038032
BLLF2	DYNLT1	0.016587
BLLF2	ZNF846	0.017694
BLLF2	COX6B2	0.017137
BLLF2	CCDC144A	0.012885
BLLF2	BCL2L12	0.065327
BLLF2	CNFN	0.014144
BLLF2	TMEM183B	0.1204
BLLF2	MED26	0.03451
BLLF2	ZMYM1	0.056204
BLLF2	CRP	0.023403
BLLF2	TBC1D16	0.010476
BLLF2	PANX1	0.049632
BLLF2	ZNF729	0.013082
BLLF2	CENPC	0.051253
BLLF2	C1QBP	0.02973
BLLF2	PNMAL2	0.084033
BLLF2	MRPL39	0.025708
BLLF2	DDOST	0.070952
BLLF2	AGR3	0.056538
BLLF2	ECM29	0.026818
BLLF2	NECAP2	0.077301
BLLF2	TADA2A	0.015045
BLLF2	IL17RD	0.076422
BLLF2	PLAGL1	0.020942
BLLF2	GALK1	0.017645
BLLF2	ZFP69B	0.033175
BLLF2	GPR160	0.061984
BLLF2	TXLNG	0.024208
BLLF2	AP4M1	0.013305
BLLF2	ZNF236	0.022049
BLLF2	RASSF8	0.020931
BLLF2	AGR2	0.034693
BLLF2	TMSB15B	0.1054
BLLF2	HBQ1	0.1064
BLLF2	YPR022C	0.1457
BLLF2	PYCR2	0.019575
BLLF2	IRF2BP1	0.024969
BLLF2	ADM	0.022027
BLLF2	LARP4	0.032123
BLLF2	IL22	0.017029
BLLF2	ZNF746	0.02184
BLLF2	GOLGA8CP	0.17823
BLLF2	DBNDD2	0.071459
BLLF2	GRHL1	0.016789
BLLF2	FYB	0.081855
BLLF2	IL17RE	0.12534
BLLF2	CENPT	0.029428
BLLF2	SSBP4	0.021507
BLLF2	CCDC146	0.0769
BLLF2	KCNK12	0.028606
BLLF2	ZNF560	0.010855
BLLF2	CMBL	0.022998
BLLF2	TMEM39B	0.074054
BLLF2	AMPD3	0.013788
BLLF2	Gm5153	0.024354
BLLF2	CCDC150	0.049825
BLLF2	PSME2	0.050072

BLLF2	ZNF669	0.018937
BLLF2	MAD1L1	0.072782
BLLF2	C1orf105	0.14328
BLLF2	PRADC1	0.048576
BLLF2	DCUN1D1	0.03486
BLLF2	STOML1	0.012889
BLLF2	VPREB1	0.10059
BLLF2	MOB3B	0.011211
BLLF2	EIF2B1	0.02714
BLLF2	BCAT1	0.014626
BLLF2	ZNF554	0.018109
BLLF2	ZNF45	0.017526
BLLF2	ADIPOR1	0.01347
BLLF2	ZNF770	0.015721
BLLF2	TMEM184C	0.051189
BLLF2	TMEM192	0.019216
BLLF2	HIST1H2BK	0.016318
BLLF2	PMM1	0.017753
BLLF2	PARPBP	0.075289
BLLF2	FANCA	0.050343
BLLF2	PSME4	0.021421
BLLF2	FIP1L1	0.196
BLLF2	HEXB	0.014428
BLLF2	ATP6V0D1	0.043706
BLLF2	TPD52L1	0.12907
BLLF2	FAM90A5P	0.012963
BLLF2	ZDHHC12	0.010168
BLLF2	ASPHD1	0.07496
BLLF2	ZNF17	0.027138
BLLF2	SPAG17	0.13296
BLLF2	PGBD1	0.021398
BLLF2	ZNHIT2	0.064544
BLLF2	FRG1	0.068303
BLLF2	LIMS3	0.017714
BLLF2	ZBP1	0.043043
BLLF2	ZNF615	0.022901
BLLF2	TC2N	0.014575
BLLF2	ALKBH1	0.038721
BLLF2	NMT2	0.050022
BLLF2	ATPAF2	0.027517
BLLF2	TMEM252	0.057391
BLLF2	ZNF613	0.019884
BLLF2	TBC1D28	0.030736
BLLF2	CCDC66	0.016876
BLLF2	TNFRSF12A	0.034692
BLLF2	POLR3F	0.061465
BLLF2	RHBDD2	0.011655
BLLF2	S100A3	0.017186
BLLF2	CCDC138	0.010697
BLLF2	CLDN6	0.024954
BLLF2	GIMAP2	0.013185
BLLF2	DCST1	0.053557
BLLF2	GLMN	0.072557
BLLF2	OR4F4	0.077525
BLLF2	POMZP3	0.014559
BLLF2	MAGEC2	0.02086
BLLF2	POU2AF1	0.030224
BLLF2	TMEM215	0.020369
BLLF2	CCDC176	0.13202
BLLF2	CASQ2	0.017697
BLLF2	GOLGA6C	0.036322
BLLF2	NUDT13	0.019043
BLLF2	SELT	0.020879
BLLF2	PUS7L	0.040473
BLLF2	HMBS	0.070385
BLLF2	CRTAP	0.044967
BLLF2	ZNF74	0.011639
BLLF2	ZNF783	0.011518

BLLF2	MPV17	0.018481
BLLF2	CTAGE4	0.025836
BLLF2	GLDC	0.028706
BLLF2	PLOD2	0.026311
BLLF2	GCH1	0.067983
BLLF2	C10orf67	0.13291
BLLF2	DAGLB	0.087069
BLLF2	SYT13	0.010546
BLLF2	LINC00597	0.093089
BLLF2	FAM131C	0.023245
BLLF2	ZNF793	0.020528
BLLF2	DYNLRB2	0.023007
BLLF2	SERINC2	0.061958
BLLF2	PRDM11	0.010652
BLLF2	NKD2	0.011665
BLLF2	LGALS8	0.011021
BLLF2	ZNF235	0.019274
BLLF2	UBL3	0.068849
BLLF2	MT1HL1	0.073765
BLLF2	REEP3	0.015077
BLLF2	TRAPPC1	0.054557
BLLF2	KRTAP13-4	0.061321
BLLF2	ZNF311	0.01963
BLLF2	ZNF264	0.013144
BLLF2	CASD1	0.17587
BLLF2	RGMA	0.1033
BLLF2	C2orf88	0.017386
BLLF2	IFT57	0.02608
BLLF2	AP5B1	0.086702
BLLF2	ZNF670	0.017617
BLLF2	ZNF350	0.023309
BLLF2	SPIRE2	0.032233
BLLF2	ZNF398	0.01845
BLLF2	ZNF705A	0.012138
BLLF2	TMEM132D	0.089763
BLLF2	MT-CO2	0.049994
BLLF2	NBPF14	0.14725
BLLF2	KRTAP2-1	0.010898
BLLF2	HORMAD2	0.075735
BLLF2	SCAF1	0.15203
BLLF2	GPAM	0.015766
BLLF2	TMEM130	0.010791
BLLF2	MAGEA12	0.013174
BLLF2	NCOR1P1	0.013103
BLLF2	SLC4A5	0.030044
BLLF2	CD3G	0.011528
BLLF2	TTLL9	0.011795
BLLF2	CHKA	0.061419
BLLF2	BTG2	0.01683
BLLF2	POLR2J2	0.0129
BLLF2	PEX3	0.017006
BLLF2	FAM134A	0.013367
BLLF2	ZNF727	0.013112
BLLF2	MED12L	0.024945
BLLF2	GHITM	0.010674
BLLF2	INCENP	0.043064
BLLF2	BPIFB1	0.015607
BLLF2	C19orf71	0.082772
BLLF2	UCK2	0.010286
BLLF2	JMJD1C	0.017645
BLLF2	ZFP3	0.017247
BLLF2	RNASE9	0.028584
BLLF2	TXLNB	0.033043
BLLF2	SNX29P2	0.048959
BLLF2	TCEB3B	0.044696
BLLF2	SLC31A1	0.030537
BLLF2	RHPN1-AS1	0.11809
BLLF2	ZNF169	0.016611

1497	BLLF2	CDKN2AIP	0.056943
1498	BLLF2	FAM90A20P	0.013324
1499	BLLF2	C6orf118	0.076284
1500	BLLF2	IL17REL	0.026985
1501	BLLF2	NDRG3	0.031885
1502	BLLF2	CCDC74B	0.048971
1503	BLLF2	ZNF248	0.017597
1504	BLLF2	FAM90A14P	0.013324
1505	BLLF2	GIGYF1	0.024067
1506	BLLF2	NXF3	0.066427
1507	BLLF2	SYCP2L	0.084108
1508	BLLF2	H2AFY	0.011154
1509	BLLF2	ZNF48	0.014281
1510	BLLF2	NGLY1	0.010496
1511	BLLF2	IGFL3	0.15193
1512	BLLF2	SEC24D	0.010569
1513	BLLF2	EDAR	0.03449
1514	BLLF2	GORASP1	0.011284
1515	BLLF2	SGCZ	0.045927
1516	BLLF2	TGOLN2	0.10052
1517	BLLF2	MTCP1	0.02712
1518	BLLF2	LINC00471	0.091376
1519	BLLF2	LYRM7	0.017816
1520	BLLF2	AP5M1	0.017318
1521	BLLF2	HABP4	0.093666
1522	BLLF2	LFNG	0.022163
1523	BLLF2	DEFB132	0.10136
1524	BLLF2	CCDC70	0.020828
1525	BLLF2	GPS2	0.14809
1526	BLLF2	CHIT1	0.014784
1527	BLLF2	KLKP1	0.086059
1528	BLLF2	B4GALT3	0.013811
1529	BLLF2	TMC3	0.067548
1530	BLLF2	SSR3	0.054909
1531	BLLF2	ATXN2L	0.04654
1532	BLLF2	SLC12A4	0.012986
1533	BLLF2	PLD4	0.03884
1534	BLLF2	ABRA	0.054202
1535	BLLF2	IGKV1-5	0.020282
1536	BLLF2	CHMP1B	0.029529
1537	BLLF2	ADORA3	0.056739
1538	BLLF2	AKAP2	0.058838
1539	BLLF2	HFE2	0.071688
1540	BLLF2	G4	0.017659
1541	BLLF2	ICA1	0.025051
1542	BLLF2	IDH3B	0.023008
1543	BLLF2	CAB8	0.014365
1544	BLLF2	MSANTD2	0.010155
1545	BLLF2	PRIMPOL	0.032992
1546	BLLF2	ABHD17A	0.032666
1547	BLLF2	CCDC137	0.16112
1548	BLLF2	GPR179	0.10328
1549	BLLF2	TIFAB	0.13933
1550	BLLF2	NCAPG	0.075686
1551	BLLF2	NANOS2	0.045942
1552	BLLF2	ECSIT	0.012871
1553	BLLF2	ERGIC2	0.011161
1554	BLLF2	C15orf54	0.036692
1555	BLLF2	ZNF861P	0.019867
1556	BLLF2	XPO4	0.011255
1557	BLLF2	CATSPERG	0.12346
1558	BLLF2	NUTM2A	0.039737
1559	BLLF2	BCL7C	0.12931
1560	BLLF2	PLAC8L1	0.031224
1561	BLLF2	TAS2R9	0.07066
1562	BLLF2	DENND1B	0.012458
1563	BLLF2	SLC16A6	0.027933
1564	BLLF2	DERL2	0.044974

BLLF2	ZNF415	0.025281
BLLF2	OR14L1P	0.07986
BLLF2	ACOT9	0.078514
BLLF2	HILS1	0.044492
BLLF2	RPS10	0.034761
BLLF2	HIST1H2BO	0.015213
BLLF2	PPP1R2P9	0.07465
BLLF2	JOSD1	0.035968
BLLF2	ENDOD1	0.016336
BLLF2	PTCD2	0.016715
BLLF2	BCDIN3D	0.13226
BLLF2	ZNF830	0.065899
BLLF2	ZSCAN31	0.014364
BLLF2	ST3GAL6	0.020198
BLLF2	CROCCP3	0.033686
BLLF2	NXT2	0.050531
BLLF2	GORAB	0.13825
BLLF2	FAM151B	0.048778
BLLF2	TSPY8	0.018872
BLLF2	MTRNR2L2	0.10682
BLLF2	CRYBB1	0.01613
BLLF2	NSUN7	0.17194
BLLF2	ROPN1	0.028358
BLLF2	MAGEB18	0.023582
BLLF2	GCSAML	0.094697
BLLF2	MED31	0.043805
BLLF2	IQUB	0.015221
BLLF2	NOG	0.026607
BLLF2	VPS25	0.01716
BLLF2	GRHL3	0.015086
BLLF2	CENPB	0.042407
BLLF2	METTL4	0.028519
BLLF2	SPC25	0.010156
BLLF2	PRPSAP1	0.031354
BLLF2	TBRG4	0.082725
BLLF2	OR2V1	0.081986
BLLF2	ZNF514	0.017909
BLLF2	ZNF287	0.018647
BLLF2	ZSCAN18	0.013694
BLLF2	FNIP2	0.216
BLLF2	LYPD6	0.014114
BLLF2	OR4F15	0.079663
BLLF2	MBP	0.036449
BLLF2	TREML1	0.022705
BLLF2	RPP21	0.072272
BLLF2	VPS26B	0.014678
BLLF2	GTPBP3	0.033811
BLLF2	PITPNB	0.015514
BLLF2	STMN2	0.035875
BLLF2	ZNF285	0.016754
BLLF2	NPIPA1	0.06734
BLLF2	FAM47B	0.10482
BLLF2	ZFP2	0.011403
BLLF2	ABO	0.023604
BLLF2	MTDH	0.015932
BLLF2	GXYLT2	0.040667
BLLF2	PRRC2A	0.044778
BLLF2	MBD3L5	0.012412
BLLF2	LSR	0.022316
BLLF2	CD33	0.013492
BLLF2	RDM1	0.056325
BLLF2	SDHAF2	0.058286
BLLF2	MACROD2	0.014224
BLLF2	SUPT20HL2	0.10332
BLLF2	GNG12	0.015901
BLLF2	PPP2R3B	0.012941
BLLF2	TCEB3C	0.054818
BLLF2	MSMB	0.016146

BLLF2	CAPZA2	0.014266
BLLF2	CTNNBIP1	0.052562
BLLF2	EIF5	0.014476
BLLF2	CENPH	0.037106
BLLF2	ENPP5	0.013552
BLLF2	FAM96A	0.020577
BLLF2	SLC5A9	0.024144
BLLF2	SPRYD7	0.028972
BLLF2	GCLC	0.017071
BLLF2	FBXO46	0.12767
BLLF2	TWISTNB	0.036598
BLLF2	ZSCAN22	0.010646
BLLF2	CENPQ	0.038515
BLLF2	SRL	0.052636
BLLF2	TIMM44	0.049142
BLLF2	METAP1D	0.011558
BLLF2	GNG3	0.01521
BLLF2	GTF2E1	0.11145
BLLF2	AP3S2	0.014273
BLLF2	TCEAL8	0.02687
BLLF2	GOLGA2P5	0.11989
BLLF2	EDARADD	0.015885
BLLF2	CTU2	0.034824
BLLF2	OXA1L	0.059012
BLLF2	INTS10	0.085049
BLLF2	NEURL2	0.016169
BLLF2	VPS13B	0.016167
BLLF2	NBPF11	0.027605
BLLF2	AP2S1	0.012282
BLLF2	UTP3	0.058438
BLLF2	ZNF354C	0.018158
BLLF2	CDR2	0.067305
BLLF2	KCTD20	0.018644
BLLF2	RSC1A1	0.058717
BLLF2	LINC00523	0.16942
BLLF2	SLC12A1	0.018412
BLLF2	ZNF781	0.022998
BLLF2	OR2T8	0.07986
BLLF2	TSPAN16	0.027282
BLLF2	SMIM14	0.035963
BLLF2	RABAC1	0.022276
BLLF2	TBC1D3G	0.013952
BLLF2	LIN54	0.024989
BLLF2	LRAT	0.015942
BLLF2	GRINA	0.038079
BLLF2	RHD	0.078785
BLLF2	PLLP	0.028728
BLLF2	EXD1	0.029142
BLLF2	RABGAP1L	0.021339
BLLF2	XBP1	0.023898
BLLF2	ZKSCAN8	0.01458
BLLF2	NEU4	0.015365
BLLF2	HIST1H2BB	0.015213
BLLF2	TEX13A	0.03605
BLLF2	RPS19BP1	0.042698
BLLF2	COMMD1	0.025563
BLLF2	ZNF354A	0.02459
BLLF2	ALKBH5	0.049618
BLLF2	ZSCAN21	0.018441
BLLF2	SACS	0.01727
BLLF2	MAGEB6	0.020868
BLLF2	GEMIN6	0.046493
BLLF2	SLC1A5	0.026235
BLLF2	CSN2	0.17791
BLLF2	EIF2B2	0.014562
BLLF2	EXOSC1	0.059956
BLLF2	SEC61G	0.056277
BLLF2	ZDHHC23	0.010168

BLLF2	IAPP	0.014574
BLLF2	VWA5B1	0.036335
BLLF2	RMI1	0.041874
BLLF2	PIK3R6	0.040705
BLLF2	H2BFS	0.016318
BLLF2	ZNF700	0.019274
BLLF2	FAM69B	0.2045
BLLF2	ZNF471	0.011205
BLLF2	CACNG4	0.048899
BLLF2	ZNF284	0.020075
BLLF2	ZFP1	0.025533
BLLF2	MED18	0.038595
BLLF2	ROM1	0.093307
BLLF2	C9orf152	0.011773
BLLF2	SPATS2	0.039513
BLLF2	SOAT1	0.018483
BLLF2	TMUB2	0.023069
BLLF2	MFSD4	0.037167
BLLF2	G4	0.014549
BLLF2	LAS2	0.025144
BLLF2	ARL14EPL	0.16453
BLLF2	FTHL17	0.011188
BLLF2	NOP14	0.062641
BLLF2	TMED9	0.014897
BLLF2	PLBD1	0.026995
BLLF2	SNRPC	0.044774
BLLF2	YKL023C-A	0.12444
BLLF2	CCDC90B	0.013552
BLLF2	CTAGE9	0.033199
BLLF2	ZNF883	0.019666
BLLF2	IL18	0.014571
BLLF2	STXBP6	0.021182
BLLF2	NICN1	0.017848
BLLF2	PITPNM1	0.023574
BLLF2	GAGE12H	0.0533
BLLF2	TOE1	0.011273
BLLF2	ZNF836	0.011851
BLLF2	OSTM1	0.029179
BLLF2	GNS	0.014603
BLLF2	PALM2	0.051031
BLLF2	OGFR	0.048648
BLLF2	ARFGEF3	0.012385
BLLF2	TAS2R30	0.07066
BLLF2	OGFOD2	0.033036
BLLF2	INTS4L2	0.019145
BLLF2	ATP6V1E2	0.05613
BLLF2	PALM	0.052406
BLLF2	NRDE2	0.047182
BLLF2	SLC34A1	0.13269
BLLF2	HIST1H2BL	0.019924
BLLF2	SLN	0.15555
BLLF2	ZNF561	0.013776
BLLF2	ZIM3	0.012465
BLLF2	OR10P1	0.084698
BLLF2	NOP56	0.035815
BLLF2	PLEKHH3	0.011503
BLLF2	ZNF716	0.013452
BLLF2	BAHD1	0.022638
BLLF2	PGGT1B	0.029645
BLLF2	TEX37	0.028218
BLLF2	UST	0.048041
BLLF2	SPRY2	0.010492
BLLF2	FAM219B	0.012275
BLLF2	CXorf21	0.058352
BLLF2	TBC1D29	0.036853
BLLF2	HIST1H2BH	0.015213
BLLF2	MBD5	0.057564
BLLF2	UGT92A1	0.067086

BLLF2	ELN	0.15741
BLLF2	SWI5	0.014592
BLLF2	LTF	0.02209
BLLF2	DENND6B	0.037634
BLLF2	TMEM233	0.053901
BLLF2	RPL18A	0.012152
BLLF2	KIAA1217	0.032914
BLLF2	NPTXR	0.017423
BLLF2	ORM2	0.04239
BLLF2	TMEM88	0.15564
BLLF2	SAMM50	0.031241
BLLF2	VPS13C	0.070888
BLLF2	FAXDC2	0.023609
BLLF2	EEF2K	0.014126
BLLF2	ZNF41	0.021358
BLLF2	C17orf99	0.021751
BLLF2	EDN1	0.012548
BLLF2	EDEM2	0.017986
BLLF2	XKR3	0.12093
BLLF2	CHGA	0.16545
BLLF2	LVP1a	0.043414
BLLF2	TMEM136	0.058753
BLLF2	FAM127A	0.040435
BLLF2	LCN6	0.01374
BLLF2	EMD	0.045754
BLLF2	UQCRQ	0.042823
BLLF2	C17orf66	0.039776
BLLF2	ECHS1	0.011264
BLLF2	TAS2R42	0.05756
BLLF2	POGK	0.01513
BLLF2	MS4A12	0.01528
BLLF2	TET3	0.087139
BLLF2	MS4A7	0.02686
BLLF2	ZNF33A	0.020823
BLLF2	FAM174B	0.041256
BLLF2	G4	0.035306
BLLF2	SPON2	0.018951
BLLF2	OR10H5	0.077525
BLLF2	R3HCC1L	0.043361
BLLF2	ZNF429	0.01369
BLLF2	HYAL2	0.01079
BLLF2	PIH1D2	0.098658
BLLF2	ZNF662	0.013417
BLLF2	IWS1	0.098175
BLLF2	BTBD17	0.021492
BLLF2	CTLA4	0.011164
BLLF2	ERP29	0.01771
BLLF2	KCNQ1DN	0.01941
BLLF2	APOL5	0.021191
BLLF2	NT5DC3	0.029156
BLLF2	POM121L12	0.074944
BLLF2	TFCP2	0.012586
BLLF2	AKAP5	0.034473
BLLF2	SPINK13	0.014778
BLLF2	PVRIG	0.1024
BLLF2	DAZAP2	0.09711
BLLF2	CPSF2	0.025754
BLLF2	SLC38A9	0.020543
BLLF2	ATP1B3	0.015917
BLLF2	SLC13A2	0.015251
BLLF2	CRYGA	0.046153
BLLF2	ZNF10	0.018272
BLLF2	ZSCAN32	0.018319
BLLF2	GANC	0.010238
BLLF2	RASSF6	0.014526
BLLF2	NDEL1	0.039991
BLLF2	HHLA3	0.035632
BLLF2	NPHS2	0.012731

BLLF2	MLEC	0.036659
BLLF2	PTX3	0.01178
BLLF2	AK9	0.018937
BLLF2	UFD1L	0.036155
BLLF2	SPINK5	0.019421
BLLF2	ABCE1	0.013563
BLLF2	ACOT7	0.031929
BLLF2	EPN3	0.014255
BLLF2	MYRF	0.022176
BLLF2	NUDT21	0.026068
BLLF2	PARK7	0.015772
BLLF2	PRO0255	0.071821
BLLF2	ZNF443	0.012048
BLLF2	TAF6L	0.013569
BLLF2	UNC93B1	0.067303
BLLF2	SUCLA2	0.030902
BLLF2	LINC00472	0.11247
BLLF2	PES1	0.022914
BLLF2	PAQR4	0.025376
BLLF2	PNMA3	0.052934
BLLF2	GPR107	0.013443
BLLF2	SOST	0.051543
BLLF2	OR2AK2	0.070942
BLLF2	MAGEA3	0.013174
BLLF2	C20orf96	0.017946
BLLF2	ZNF224	0.012084
BLLF2	GOLGA6L4	0.11989
BLLF2	RAMP2	0.028038
BLLF2	GOLGA6L6	0.1131
BLLF2	KIAA1958	0.045552
BLLF2	TAS2R41	0.030663
BLLF2	SLAIN1	0.15159
BLLF2	SAA2	0.1015
BLLF2	zag-1	0.019192
BLLF2	PLEKHA8P1	0.041082
BLLF2	TSLP	0.036479
BLLF2	ARL6IP5	0.061037
BLLF2	G4	0.081082
BLLF2	C9orf169	0.033463
BLLF2	DMRTC2	0.027308
BLLF2	EIF3C	0.028645
BLLF2	EIF3M	0.014606
BLLF2	YPL025C	0.052914
BLLF2	ZNF326	0.089467
BLLF2	FBXO32	0.012762
BLLF2	KIAA0226L	0.011382
BLLF2	CLDN5	0.02677
BLLF2	STEAP1	0.052049
BLLF2	MYOZ3	0.064033
BLLF2	YBL100W-C	0.01069
BLLF2	OR4F21	0.07986
BLLF2	DTX2	0.016188
BLLF2	COMMD10	0.01355
BLLF2	NOM1	0.036524
BLLF2	SMYD5	0.063499
BLLF2	GG1	0.076717
BLLF2	FANCL	0.044816
BLLF2	FBXO2	0.012768
BLLF2	CAPZA3	0.022018
BLLF2	FRAT2	0.13069
BLLF2	RFX3	0.01521
BLLF2	ITGB1BP2	0.011048
BLLF2	MDFIC	0.014659
BLLF2	OVOS2	0.014161
BLLF2	ATP1B4	0.024166
BLLF2	APEX1	0.011559
BLLF2	BET1	0.02564
BLLF2	ANXA2R	0.16564

BLLF2	EDDM3B	0.044854
BLLF2	CC2D2B	0.046931
BLLF2	ZNF750	0.065355
BLLF2	ZNF688	0.013856
BLLF2	YML009W-B	0.17226
BLLF2	C14orf178	0.0678
BLLF2	NDUFB6	0.016453
BLLF2	CHML	0.010042
BLLF2	GOLGA8H	0.026859
BLLF2	LINC00116	0.086397
BLLF2	TSPYL1	0.011975
BLLF2	NAGA	0.031085
BLLF2	ERLEC1	0.032394
BLLF2	KIFAP3	0.016275
BLLF2	MED9	0.11845
BLLF2	LGALS13	0.026698
BLLF2	ZNF501	0.015587
BLLF2	DAXX	0.050776
BLLF2	EIF3CL	0.028101
BLLF2	TEX19	0.086297
BLLF2	NIPA1	0.11182
BLLF2	PANX3	0.051226
BLLF2	BID	0.09915
BLLF2	LY6E	0.014097
BLLF2	COMMD2	0.10148
BLLF2	SDPR	0.047034
BLLF2	ZNF202	0.015279
BLLF2	PNMA1	0.023924
BLLF2	CCDC53	0.02266
BLLF2	C1orf168	0.061688
BLLF2	UAP1	0.025106
BLLF2	NIPSNAP3A	0.062206
BLLF2	PCNXL2	0.017099
BLLF2	CIR1	0.058501
BLLF2	YTHDF1	0.075871
BLLF2	RNASE3	0.023404
BLLF2	NRGN	0.067362
BLLF2	FAM179A	0.010182
BLLF2	TCHHL1	0.011189
BLLF2	PIGL	0.10527
BLLF2	HRASLS2	0.024883
BLLF2	MGST2	0.04163
BLLF2	CALCA	0.026873
BLLF2	MRFAP1L1	0.13852
BLLF2	DENND2C	0.014078
BLLF2	SMAGP	0.011656
BLLF2	PLIN4	0.080456
BLLF2	ZNF226	0.019274
BLLF2	TNRC18	0.014543
BLLF2	MFAP5	0.061295
BLLF2	TERT	0.064709
BLLF2	TDRD3	0.018279
BLLF2	ZBTB38	0.012096
BLLF2	ZNF564	0.020067
BLLF2	ZNF862	0.026098
BLLF2	MEAF6	0.050861
BLLF2	CNPY1	0.014776
BLLF2	FAM160A1	0.14303
BLLF2	EXOSC6	0.023753
BLLF2	RCAN2	0.010742
BLLF2	MTX3	0.057605
BLLF2	SLC13A1	0.031637
BLLF2	L2HGDH	0.011279
BLLF2	NASP	0.044951
BLLF2	PIR	0.094073
BLLF2	SRP14	0.015899
BLLF2	VTI1A	0.019034
BLLF2	SPIN1	0.059903

BLLF2	BCAT2	0.023314
BLLF2	ZNF184	0.011494
BLLF2	G4	0.033161
BLLF2	MAFK	0.034844
BLLF2	ZNF337	0.015269
BLLF2	ZNF607	0.011494
BLLF2	WHAMMP3	0.017185
BLLF2	ID2B	0.03758
BLLF2	TNKS1BP1	0.025198
BLLF2	PRAMEF13	0.18496
BLLF2	GET4	0.073437
BLLF2	CCDC85B	0.043314
BLLF2	TGFBR3L	0.015283
BLLF2	PMS2	0.021595
BLLF2	SRRM1	0.18353
BLLF2	ATF7IP2	0.026715
BLLF2	LGALSL	0.019103
BLLF2	GDPD2	0.011676
BLLF2	ZFX	0.010097
BLLF2	PPTC7	0.014982
BLLF2	EMP2	0.021249
BLLF2	NARS	0.037296
BLLF2	ALB	0.041103
BLLF2	RBAK	0.023507
BLLF2	YJR030C	0.088125
BLLF2	TREML4	0.015883
BLLF2	ZNF208	0.011897
BLLF2	PSMD8	0.033935
BLLF2	NAP1L1	0.012924
BLLF2	KIAA1841	0.099598
BLLF2	EAF2	0.067554
BLLF2	SDF2L1	0.029923
BLLF2	STAG3L1	0.068055
BLLF2	C1orf196	0.04432
BLLF2	MGAT2	0.020892
BLLF2	RNF216P1	0.085174
BLLF2	RASSF4	0.052058
BLLF2	PTGES	0.043231
BLLF2	C10orf76	0.0573
BLLF2	DCUN1D2	0.013613
BLLF2	MBD3L4	0.012412
BLLF2	MTRNR2L9	0.10682
BLLF2	Gm5153	0.025502
BLLF2	LINC00476	0.019932
BLLF2	STPG2	0.017044
BLLF2	ZNF705F	0.012489
BLLF2	POLR2K	0.044864
BLLF2	CC2D2A	0.03034
BLLF2	WBSCR23	0.0684
BLLF2	ZMYM5	0.015982
BLLF2	HTN3	0.14883
BLLF2	ARL6IP6	0.10871
BLLF2	PRODH	0.08658
BLLF2	YJEFN3	0.049684
BLLF2	C18orf21	0.049434
BLLF2	MFSD6	0.11182
BLLF2	AURKAIP1	0.027302
BLLF2	kup	0.012333
BLLF2	ZC4H2	0.016875
BLLF2	XAGE2	0.14761
BLLF2	YML009W-B	0.061825
BLLF2	ELOVL3	0.05608
BLLF2	TMEM116	0.011568
BLLF2	SEC23IP	0.014068
BLLF2	PALB2	0.02668
BLLF2	NEIL3	0.018719
BLLF2	NUDT1	0.01632
BLLF2	LRCOL1	0.11873

BLLF2	MAGEB5	0.011196
BLLF2	ZBTB16	0.014838
BLLF2	SAT1	0.02539
BLLF2	PTTG3P	0.049937
BLLF2	DTYMK	0.10426
BLLF2	G4	0.024842
BLLF2	DCTN2	0.013864
BLLF2	PPP1R3D	0.01406
BLLF2	LUZP6	0.046048
BLLF2	GOLGA6L18	0.099402
BLLF2	CD53	0.011442
BLLF2	AFF1	0.059738
BLLF2	MRPL53	0.034157
BLLF2	CD151	0.014204
BLLF2	MDFI	0.06313
BLLF2	YTHDC1	0.10348
BLLF2	ZNF420	0.012141
BLLF2	PTER	0.02862
BLLF2	MIPOL1	0.010911
BLLF2	CSHL1	0.025041
BLLF2	SLC52A3	0.1122
BLLF2	tarbp2	0.018013
BLLF2	SHISA2	0.024587
BLLF2	ZNF215	0.020269
BLLF2	NBPF6	0.03035
BLLF2	TM6SF1	0.070954
BLLF2	TRAPPC8	0.031387
BLLF2	MRPS10	0.13341
BLLF2	GEMIN7	0.091552
BLLF2	TBC1D7	0.016176
BLLF2	SSX3	0.018528
BLLF2	PPP1R1A	0.020122
BLLF2	SEC24C	0.016315
BLLF2	LAMP5	0.071429
BLLF2	VSTM5	0.012952
BLLF2	AMY1A	0.038244
BLLF2	EXOC7	0.012972
BLLF2	DSCR3	0.020973
BLLF2	G4	0.024383
BLLF2	TXNIP	0.080897
BLLF2	SAT2	0.012113
BLLF2	PITPNA	0.045213
BLLF2	TSPAN11	0.010426
BLLF2	ZNF570	0.012692
BLLF2	TIMM17A	0.037722
BLLF2	GAGE2D	0.029493
BLLF2	APRG1	0.14833
BLLF2	ZNF268	0.017
BLLF2	YLPM1	0.016723
BLLF2	BCORP1	0.063333
BLLF2	BRAT1	0.081607
BLLF2	P4HA2	0.047092
BLLF2	TBC1D3	0.011597
BLLF2	FAM160B2	0.032361
BLLF2	MZT2A	0.019063
BLLF2	RNASEH2A	0.13888
BLLF2	GTF3C6	0.09277
BLLF2	WIBG	0.12967
BLLF2	IFT140	0.020539
BLLF2	FAM221B	0.098262
BLLF2	SLC13A5	0.01453
BLLF2	SLCO1A2	0.029211
BLLF2	FRYL	0.027389
BLLF2	PDE6H	0.10012
BLLF2	TRH	0.098046
BLLF2	RIF1	0.10896
BLLF2	DMRTA2	0.027308
BLLF2	RPL18	0.051932

BLLF2	PCK1	0.029439
BLLF2	ERLIN2	0.039739
BLLF2	FLOT2	0.012205
BLLF2	MPTX1	0.036157
BLLF2	ZNF548	0.017271
BLLF2	AMER2	0.11379
BLLF2	APOBEC3C	0.032023
BLLF2	TAF1D	0.022777
BLLF2	EXOSC3	0.024458
BLLF2	STIM1	0.0103
BLLF2	COG4	0.030294
BLLF2	TRAF3IP1	0.1363
BLLF2	PRAMEF7	0.010179
BLLF2	CCDC172	0.019795
BLLF2	ERI3	0.026212
BLLF2	YTHDF2	0.09141
BLLF2	LDOC1L	0.030829
BLLF2	GSS	0.036202
BLLF2	AC3.5	0.065292
BLLF2	SKP1	0.030945
BLLF2	ZNF563	0.013633
BLLF2	ZNF212	0.025373
BLLF2	TSPAN4	0.012484
BLLF2	LINS	0.038582
BLLF2	KHDC3L	0.071749
BLLF2	ZNF585B	0.015248
BLLF2	UPK1B	0.019627
BLLF2	UMPS	0.011587
BLLF2	PRPS1L1	0.024065
BLLF2	OR2J2	0.077525
BLLF2	SAP30	0.10279
BLLF2	ZNF24	0.011638
BLLF2	SHCBP1	0.25217
BLLF2	CRYGC	0.038506
BLLF2	N4BP2L2	0.013539
BLLF2	COX10	0.13225
BLLF2	EFCAB4A	0.02149
BLLF2	TXLNA	0.033304
BLLF2	POLRMT	0.075833
BLLF2	BCAS2	0.021919
BLLF2	C14orf80	0.13773
BLLF2	RAB3IL1	0.024086
BLLF2	NBPF24	0.027605
BLLF2	C1orf94	0.26611
BLLF2	SLC5A7	0.014494
BLLF2	OR10G2	0.091156
BLLF2	REC8	0.010358
BLLF2	SLC16A14	0.024988
BLLF2	C9orf153	0.039234
BLLF2	SSX9	0.013615
BLLF2	WFDC12	0.049017
BLLF2	UBAC2	0.01218
BLLF2	TINF2	0.036861
BLLF2	FBXO44	0.017122
BLLF2	ITM2B	0.043375
BLLF2	FEZ1	0.024744
BLLF2	GNN	0.016995
BLLF2	KARS	0.052603
BLLF2	SMG5	0.010287
BLLF2	TAS2R5	0.028776
BLLF2	ZNF764	0.014598
BLLF2	CCDC51	0.01981
BLLF2	GGA1	0.011653
BLLF2	CWC22	0.023711
BLLF2	NDUFB11	0.014631
BLLF2	MCUR1	0.035709
BLLF2	RNASE4	0.022259
BLLF2	HGD	0.12065

BLLF2	TCAIM	0.017559
BLLF2	TSPAN5	0.010956
BLLF2	OR5L1	0.075258
BLLF2	PRDM5	0.017942
BLLF2	PSRC1	0.075312
BLLF2	REEP1	0.022099
BLLF2	GINM1	0.019058
BLLF2	METTL9	0.032864
BLLF2	BSN	0.1076
BLLF2	MARCH9	0.011896
BLLF2	CERK	0.012942
BLLF2	APOBEC3A	0.033833
BLLF2	C17orf74	0.09406
BLLF2	PRAMEF24	0.010179
BLLF2	C10orf54	0.040603
BLLF2	ANAPC1	0.055577
BLLF2	CT45A5	0.041258
BLLF2	PNRC2	0.15417
BLLF2	CMTM4	0.026425
BLLF2	C2orf78	0.046465
BLLF2	C21orf58	0.043827
BLLF2	RPE65	0.016651
BLLF2	RPL5	0.01207
BLLF2	ZNF234	0.012141
BLLF2	CDCA5	0.072959
BLLF2	HAX1	0.062293
BLLF2	FAM198B	0.066945
BLLF2	THUMPD1	0.01382
BLLF2	GOLGA6A	0.053004
BLLF2	MIA2	0.020213
BLLF2	NDRG4	0.015388
BLLF2	SLCO4A1	0.010877
BLLF2	HEMK1	0.041315
BLLF2	TIGD4	0.012183
BLLF2	FAM210A	0.11687
BLLF2	S100A7L2	0.010438
BLLF2	CXXC11	0.051203
BLLF2	PNLDC1	0.061866
BLLF2	C19orf35	0.1003
BLLF2	SAMD11	0.017785
BLLF2	ZNF550	0.021723
BLLF2	TSPAN10	0.037168
BLLF2	ZNF594	0.017415
BLLF2	ZNF506	0.014684
BLLF2	GAGE6	0.029493
BLLF2	CD44	0.011841
BLLF2	TNFRSF18	0.01651
BLLF2	SMNDC1	0.017318
BLLF2	ANO9	0.067274
BLLF2	ZNF529	0.012843
BLLF2	AATF	0.01779
BLLF2	CNOT1	0.0273
BLLF2	HBZ	0.10325
BLLF2	DDHD2	0.017268
BLLF2	YIR021W-A	0.037598
BLLF2	TACC3	0.017992
BLLF2	LAGE3	0.086421
BLLF2	ZNF534	0.013367
BLLF2	ICOS	0.066514
BLLF2	COMMD9	0.079644
BLLF2	LEMD1	0.027073
BLLF2	DNASE1L2	0.13198
BLLF2	ZNF189	0.01787
BLLF2	UBXN2B	0.03232
BLLF2	MS4A18	0.01193
BLLF2	KCNJ4	0.011702
BLLF2	SYPL1	0.12677
BLLF2	APOBEC1	0.016682

BLLF2	ERLIN1	0.13658
BLLF2	SBSPON	0.013063
BLLF2	ZNF25	0.012311
BLLF2	UBXN11	0.056485
BLLF2	ANGEL2	0.027317
BLLF2	ZNF473	0.019297
BLLF2	AHSA1	0.080112
BLLF2	ZFAND2A	0.012482
BLLF2	CNNM1	0.030968
BLLF2	MIR7-3HG	0.027717
BLLF2	CRB3	0.051987
BLLF2	HRG	0.012447
BLLF2	IL10	0.03054
BLLF2	ORC3	0.016181
BLLF2	FDFT1	0.036168
BLLF2	ITIH2	0.023782
BLLF2	PP632	0.026682
BLLF2	C17orf104	0.020173
BLLF2	ZNF132	0.017
BLLF2	PRO1854	0.029357
BLLF2	ZNF222	0.024306
BLLF2	OR6N2	0.076296
BLLF2	KCNJ9	0.014152
BLLF2	CTAGE3P	0.016642
BLLF2	COPS7A	0.01445
BLLF2	TMCC1	0.014765
BLLF2	GAGE4	0.071229
BLLF2	ZNF75A	0.015132
BLLF2	MPHOSPH10	0.075891
BLLF2	G4	0.01091
BLLF2	PFKFB2	0.014976
BLLF2	AOC2	0.032553
BLLF2	ZNF787	0.018068
BLLF2	ATP5SL	0.016747
BLLF2	ATP1B2	0.013725
BLLF2	NANOGNB	0.014803
BLLF2	ZSWIM7	0.10666
BLLF2	RPAP2	0.030335
BLLF2	C2CD4D	0.033058
BLLF2	HOGA1	0.064257
BLLF2	ZK1128.3	0.043425
BLLF2	RRP1B	0.10364
BLLF2	YDL022C-A	0.023608
BLLF2	PP632	0.025392
BLLF2	STARD3	0.011252
BLLF2	AGTPBP1	0.038696
BLLF2	REEP6	0.02094
BLLF2	SCNM1	0.066871
BLLF2	PHACTR4	0.045572
BLLF2	ZNF343	0.015559
BLLF2	PGK2	0.040401
BLLF2	TBC1D3B	0.010407
BLLF2	PAPSS2	0.010328
BLLF2	NUP98	0.032736
BLLF2	ZNF684	0.021834
BLLF2	SLC1A1	0.025583
BLLF2	MYADM	0.036958
BLLF2	SLC37A3	0.013437
BLLF2	SLC1A2	0.025583
BLLF2	COMMD8	0.030557
BLLF2	PNMAL1	0.044836
BLLF2	RPS13	0.010212
BLLF2	RPL27	0.023705
BLLF2	ZNF121	0.013743
BLLF2	C18orf42	0.010837
BLLF2	NT5DC1	0.015763
BLLF2	IMMP1L	0.011507
BLLF2	METTL22	0.016953

BLLF2	LY6K	0.12689
BLLF2	GLCCI1	0.069273
BLLF2	DCLRE1C	0.012159
BLLF2	ZBTB40	0.015498
BLLF2	SLC30A7	0.013693
BLLF2	ATP5S	0.056259
BLLF2	CEP55	0.029057
BLLF2	NCOA4	0.043188
BLLF2	TSPAN15	0.011257
BLLF2	NUP62	0.012511
BLLF2	PRDM15	0.013681
BLLF2	IFITM5	0.031014
BLLF2	TPT1P8	0.046026
BLLF2	C3orf18	0.023485
BLLF2	GAGE13	0.029493
BLLF2	GALNT15	0.019731
BLLF2	RGS7BP	0.13523
BLLF2	RPA4	0.042416
BLLF2	MED14	0.025584
BLLF2	ZNF761	0.013032
BLLF2	PIK3AP1	0.013915
BLLF2	TRAPPC2L	0.019348
BLLF2	MTRNR2L7	0.10682
BLLF2	AK2	0.0162
BLLF2	GRIFIN	0.017656
BLLF2	ABT1	0.016843
BLLF2	DDI2	0.021693
BLLF2	NDRG2	0.031615
BLLF2	FAM19A3	0.12645
BLLF2	APOBEC3F	0.029072
BLLF2	PSME1	0.060622
BLLF2	ZYG11A	0.013529
BLLF2	CPED1	0.11137
BLLF2	CXorf30	0.10169
BLLF2	CD164L2	0.081816
BLLF2	MUS81	0.049592
BLLF2	APOBEC3D	0.029866
BLLF2	NDST4	0.010186
BLLF2	MLF2	0.096395
BLLF2	CAMSAP3	0.10185
BLLF2	RPL8	0.071576
BLLF2	DACT3	0.024345
BLLF2	Gm5153	0.014704
BLLF2	RPL35	0.03928
BLLF2	ZNF785	0.031687
BLLF2	MIEF1	0.010103
BLLF2	USP53	0.069494
BLLF2	TIMM13	0.016847
BLLF2	OR2L5	0.087045
BLLF2	HIST1H1T	0.013504
BLLF2	ELOVL5	0.010551
BLLF2	BLOC1S6	0.010605
BLLF2	ZNF101	0.021612
BLLF2	HIST1H4G	0.052173
BLLF2	NABP1	0.082829
BLLF2	CCDC132	0.03713
BLLF2	OR4D6	0.077525
BLLF2	MOAP1	0.041392
BLLF2	RPS19	0.061728
BLLF2	GNGT1	0.015648
BLLF2	SDSL	0.058014
BLLF2	TSPY4	0.018872
BLLF2	MSL1	0.096612
BLLF2	NPHP4	0.15757
BLLF2	CRLF2	0.031921
BLLF2	STRIP2	0.072328
BLLF2	ATG4C	0.075402
BLLF2	FAM21B	0.12704

BLLF2	PLSCR4	0.069788
BLLF2	INSIG1	0.030717
BLLF2	DNAJC9-AS1	0.11983
BLLF2	CENPF	0.022801
BLLF2	UBQLNL	0.012011
BLLF2	OR4K13	0.098101
BLLF2	ALG14	0.014871
BLLF2	TMEM30C	0.072735
BLLF2	CCDC152	0.018504
BLLF2	KHDC1L	0.012461
BLLF2	IL26	0.015934
BLLF2	DCP2	0.0116
BLLF2	STAG3L2	0.047809
BLLF2	SCAMP3	0.036502
BLLF2	SYCE3	0.14451
BLLF2	FAM45A	0.13027
BLLF2	KIAA1751	0.014239
BLLF2	FBXO25	0.13451
BLLF2	GGT5	0.034697
BLLF2	ZNF804B	0.019663
BLLF2	TCP10L2	0.078998
BLLF2	VHLL	0.028559
BLLF2	CCDC57	0.010573
BLLF2	GOLGB1	0.079028
BLLF2	SETD3	0.047626
BLLF2	HBA1	0.087769
BLLF2	CYB561D1	0.08025
BLLF2	EVC	0.055541
BLLF2	CAP2	0.014985
BLLF2	C1orf106	0.14644
BLLF2	OR2T34	0.077525
BLLF2	ZC3H11A	0.013987
BLLF2	DNAJB11	0.024353
BLLF2	GOLGA6L3	0.11989
BLLF2	KRTAP12-4	0.018246
BLLF2	WIPF3	0.10006
BLLF2	KRBOX4	0.011793
BLLF2	ZFP64	0.013892
BLLF2	TSPO2	0.022915
BLLF2	C5orf55	0.10807
BLLF2	WWC2-AS2	0.079129
BLLF2	EVA1C	0.030112
BLLF2	SAP18	0.064647
BLLF2	ZNF140	0.015266
BLLF2	ZNF182	0.016033
BLLF2	SYNGR3	0.12121
BLLF2	PPA2	0.011267
BLLF2	CHI3L1	0.01297
BLLF2	CITED2	0.010963
BLLF2	BEST1	0.025695
BLLF2	VPS53	0.042614
BLLF2	TTI1	0.07437
BLLF2	MARCH7	0.012838
BLLF2	CAP1	0.036038
BLLF2	ZNF808	0.012141
BLLF2	SLC9B2	0.052429
BLLF2	UBXN4	0.076316
BLLF2	TKT	0.022157
BLLF2	FBXL18	0.036917
BLLF2	GUCD1	0.024796
BLLF2	KCNK13	0.01684
BLLF2	FAM195B	0.032796
BLLF2	RPP25L	0.10335
BLLF2	CCDC7	0.057491
BLLF2	YFL013W-A	0.077312
BLLF2	HHATL	0.055146
BLLF2	MAGT1	0.031556
BLLF2	CCDC3	0.012366

BLLF2	NDUFAF7	0.10191
BLLF2	TIMP2	0.015797
BLLF2	NBPF20	0.14725
BLLF2	ESF1	0.16847
BLLF2	GAGE1	0.071229
BLLF2	TVP23B	0.026097
BLLF2	ADTRP	0.045088
BLLF2	RGS9BP	0.036636
BLLF2	GREB1	0.017582
BLLF2	OR2T10	0.080961
BLLF2	CUL7	0.014082
BLLF2	BEX4	0.05754
BLLF2	ANO2	0.07255
BLLF2	AK4	0.013307
BLLF2	MTRNR2L3	0.10682
BLLF2	NSUN5	0.058199
BLLF2	KRTAP16-1	0.10635
BLLF2	TMEM45B	0.040694
BLLF2	GAGE12F	0.0533
BLLF2	ZNF366	0.022479
BLLF2	LAMP2	0.029563
BLLF2	TEN1	0.050816
BLLF2	STEAP1B	0.019497
BLLF2	ZNF672	0.017308
BLLF2	OCA2	0.01672
BLLF2	AMPD2	0.017181
BLLF2	Gm5153	0.019666
BLLF2	H1FOO	0.017309
BLLF2	ZNF423	0.014093
BLLF2	MZT1	0.074068
BLLF2	OR13D1	0.072817
BLLF2	CCDC28B	0.025125
BLLF2	CCDC80	0.036683
BLLF2	PGLYRP1	0.015651
BLLF2	VDAC2	0.012759
BLLF2	ZNF697	0.020896
BLLF2	SHARPIN	0.011782
BLLF2	SLC5A10	0.024144
BLLF2	PACS2	0.1419
BLLF2	PRAMEF25	0.015167
BLLF2	AFM	0.052003
BLLF2	EXOC5	0.014449
BLLF2	PCSK9	0.010562
BLLF2	CLDN4	0.022562
BLLF2	SLC51B	0.022987
BLLF2	FAM8A1	0.033818
BLLF2	PGK1	0.041557
BLLF2	BOLA1	0.028362
BLLF2	SHMT1	0.039197
BLLF2	RBM27	0.037097
BLLF2	MTL5	0.048804
BLLF2	CNPY4	0.12727
BLLF2	ZNF496	0.024408
BLLF2	NELFA	0.17422
BLLF2	PRAMEF11	0.017051
BLLF2	PHACTR1	0.06183
BLLF2	TMEM161A	0.013439
BLLF2	GPRIN3	0.034727
BLLF2	DAPL1	0.014224
BLLF2	ZBTB48	0.02149
BLLF2	FAM92B	0.022821
BLLF2	PANX2	0.030795
BLLF2	ZNF79	0.016349
BLLF2	ZNF660	0.023238
BLLF2	ZRANB2	0.01234
BLLF2	UBXN2A	0.078122
BLLF2	C4orf22	0.021002
BLLF2	TCEANC	0.010546

BLLF2	ANO6	0.02818
BLLF2	PHYHD1	0.035889
BLLF2	POLA2	0.034009
BLLF2	DHFRL1	0.057828
BLLF2	APOO	0.087258
BLLF2	PCYT2	0.016912
BLLF2	MED28	0.018547
BLLF2	FUNDC2	0.034801
BLLF2	RIPPLY2	0.036521
BLLF2	KPTN	0.015088
BLLF2	C6orf52	0.01545
BLLF2	TULP3	0.010538
BLLF2	FAM19A4	0.049958
BLLF2	SLC30A9	0.013257
BLLF2	ARRDC3	0.052267
BLLF2	Enho	0.016283
BLLF2	GFRA3	0.013774
BLLF2	IPMK	0.015373
BLLF2	CABLES1	0.026166
BLLF2	CCDC74A	0.048971
BLLF2	GPBP1	0.031142
BLLF2	SERBP1	0.1076
BLLF2	ANP32C	0.010549
BLLF2	TGFBRAP1	0.036243
BLLF2	PRR19	0.072876
BLLF2	ZNF837	0.014966
BLLF2	XRCC4	0.01322
BLLF2	CCDC67	0.011969
BLLF2	STAG3	0.058451
BLLF2	BANP	0.032281
BLLF2	ZNF711	0.013418
BLLF2	RRBP1	0.03142
BLLF2	RPRML	0.050361
BLLF2	ZNF253	0.013023
BLLF2	FAM110C	0.082497
BLLF2	HIST1H2BM	0.015213
BLLF2	SUPT5H	0.011425
BLLF2	ATP6V0A2	0.010748
BLLF2	NRL	0.017102
BLLF2	MT1X	0.065501
BLLF2	ARMC10	0.013764
BLLF2	ZNF888	0.02231
BLLF2	THAP11	0.070724
BLLF2	WBP2NL	0.11207
BLLF2	GAREML	0.15956
BLLF2	MBOAT1	0.06299
BLLF2	PMS2P1	0.020081
BLLF2	SYP	0.019288
BLLF2	BVES	0.034855
BLLF2	ZNF174	0.010653
BLLF2	MIMI	0.12297
BLLF2	LDHC	0.035408
BLLF2	OR2T3	0.077525
BLLF2	TNRC6A	0.051648
BLLF2	TMCO6	0.079059
BLLF2	SLC16A7	0.034067
BLLF2	SLC4A3	0.033657
BLLF2	SPG20	0.03352
BLLF2	TICRR	0.1216
BLLF2	FAM84B	0.011569
BLLF2	SLX1A	0.067797
BLLF2	PHOSPHO2	0.01671
BLLF2	MOSPD1	0.024678
BLLF2	IL27	0.13491
BLLF2	ytzL	0.23096
BLLF2	ERVK6	0.012302
BLLF2	EMID1	0.037069
BLLF2	ERC2-IT1	0.032945

BLLF2	ZNF691	0.022774
BLLF2	WDR96	0.079704
BLLF2	GRAMD1C	0.017866
BLLF2	PYDC2	0.011543
BLLF2	GNL1	0.016174
BLLF2	ZNF665	0.013362
BLLF2	STT3B	0.062158
BLLF2	STARD7	0.020728
BLLF2	OTUD4	0.011403
BLLF2	DAD1	0.071155
BLLF2	ADA	0.011052
BLLF2	FBXO17	0.036078
BLLF2	COL17A1	0.13783
BLLF2	THAP10	0.040627
BLLF2	EIF6	0.047165
BLLF2	UROD	0.071098
BLLF2	CLDN8	0.011729
BLLF2	ZNF2	0.012781
BLLF2	BECN1P1	0.019738
BLLF2	OR4K2	0.087261
BLLF2	TSEN2	0.10191
BLLF2	TMED4	0.012799
BLLF2	SCNN1G	0.012295
BLLF2	C17orf85	0.027169
BLLF2	ZNF568	0.018194
BLLF2	TNRC6B	0.057714
BLLF2	L1TD1	0.011304
BLLF2	C15orf65	0.04685
BLLF2	FAM160B1	0.091475
BLLF2	IL17RA	0.035483
BLLF2	SCAMP4	0.032745
BLLF2	MAPKAP1	0.098583
BLLF2	NUCB1	0.034908
BLLF2	PRIMA1	0.088585
BLLF2	NDUFA5	0.021585
BLLF2	ELOVL6	0.05608
BLLF2	EPDR1	0.017866
BLLF2	SAMD14	0.012196
BLLF2	ZNF577	0.022731
BLLF2	OFD1	0.076499
BLLF2	NEU2	0.033108
BLLF2	YIPF7	0.027303
BLLF2	ZNF18	0.018562
BLLF2	SSX1	0.10234
BLLF2	PCYT1B	0.012274
BLLF2	ZNF331	0.016558
BLLF2	MRPL32	0.071796
BLLF2	ZWINT	0.038414
BLLF2	NAPG	0.032445
BLLF2	CT45A4	0.033289
BLLF2	RBBP8	0.043731
BLLF2	FCF1	0.097118
BLLF2	LGALS16	0.016359
BLLF2	FBXL8	0.19876
BLLF2	SNAPC2	0.029685
BLLF2	STX17	0.026044
BLLF2	SNRPE	0.012895
BLLF2	MBD3L3	0.012412
BLLF2	DENND2D	0.014903
BLLF2	CKS2	0.013375
BLLF2	NBPF21	0.027605
BLLF2	GCHFR	0.032122
BLLF2	TAS2R45	0.045195
BLLF2	MFAP1	0.14535
BLLF2	H1F0	0.012115
BLLF2	BSCL2	0.016571
BLLF2	RAD17	0.010734
BLLF2	OR14J1	0.077525

BLLF2	FBL	0.015991
BLLF2	TTYH1	0.18628
BLLF2	NUPR1L	0.065676
BLLF2	BCL2L13	0.041332
BLLF2	ZNF12	0.023605
BLLF2	C10orf82	0.076986
BLLF2	CCDC8	0.010562
BLLF2	ERICH1-AS1	0.1284
BLLF2	ZNF829	0.01091
BLLF2	CCRN4L	0.039622
BLLF2	CHCHD6	0.12039
BLLF2	C3orf65	0.072229
BLLF2	ZNF480	0.013112
BLLF2	TRAM1L1	0.013336
BLLF2	DRG2	0.063974
BLLF2	ATXN1	0.055746
BLLF2	PELO	0.018012
BLLF2	GAP43	0.017788
BLLF2	OLFML1	0.015701
BLLF2	TTYH2	0.019072
BLLF2	C6orf89	0.028669
BLLF2	BEND7	0.057552
BLLF2	CBY3	0.05513
BLLF2	REEP5	0.019536
BLLF2	MYBBP1A	0.015459
BLLF2	SYNGR1	0.11407
BLLF2	C1orf228	0.01724
BLLF2	VPS52	0.028361
BLLF2	TRAPPC2P1	0.017591
BLLF2	DAP3	0.043223
BLLF2	Enho	0.013842
BLLF2	OR2T1	0.087045
BLLF2	GOLPH3L	0.077301
BLLF2	ORMDL2	0.14095
BLLF2	ZNF626	0.013452
BLLF2	XKR5	0.078034
BLLF2	ZGPAT	0.011089
BLLF2	PRAMEF16	0.022587
BLLF2	ZBTB20	0.012381
BLLF2	RAB3IP	0.017145
BLLF2	DYDC2	0.035536
BLLF2	ZNF840	0.011771
BLLF2	XPO5	0.021053
BLLF2	SS18L1	0.024066
BLLF2	PRPF31	0.017508
BLLF2	ZNF419	0.017439
BLLF2	RPL10	0.044083
BLLF2	LSM14B	0.053969
BLLF2	EP400NL	0.11339
BLLF2	PADI3	0.012903
BLLF2	FAM203B	0.07348
BLLF2	ZNF75CP	0.01533
BLLF2	GAGE7	0.0533
BLLF2	TYW5	0.020413
BLLF2	BCL9L	0.11686
BLLF2	KCNE1	0.034874
BLLF2	PSMB7	0.011177
BLLF2	PRUNE2	0.01458
BLLF2	RANBP17	0.023376
BLLF2	TET1	0.037824
BLLF2	CRIPT	0.02647
BLLF2	ZNF774	0.015841
BLLF2	TGM2	0.032776
BLLF2	RPLP0	0.020178
BLLF2	ST20	0.10156
BLLF2	BAG1	0.033156
BLLF2	BAHCC1	0.013596
BLLF2	CCDC91	0.059992

BLLF2	CITED4	0.01098
BLLF2	QTRTD1	0.12763
BLLF2	LEP	0.012949
BLLF2	LIPT1	0.016272
BLLF2	PPP1R3G	0.029905
BLLF2	YIF1A	0.079001
BLLF2	HEATR5A	0.052637
BLLF2	BEX1	0.14423
BLLF2	TTYH3	0.17536
BLLF2	TECTB	0.01551
BLLF2	PLGLB1	0.060007
BLLF2	LCMT1	0.031036
BLLF2	PSMD13	0.040504
BLLF2	ST5	0.014078
BLLF2	MYADML2	0.048145
BLLF2	EID2	0.095189
BLLF2	ELOVL7	0.05608
BLLF2	FAM184A	0.035982
BLLF2	ZNF441	0.019274
BLLF2	PRPH2	0.03981
BLLF2	PAFAH2	0.015332
BLLF2	PDS5B	0.019525
BLLF2	PCYT1A	0.03166
BLLF2	KRR1	0.0134
BLLF2	OR2AJ1	0.081986
BLLF2	SBSN	0.022917
BLLF2	ZNF383	0.01813
BLLF2	OR2T12	0.07986
BLLF2	OTUD6A	0.033486
BLLF2	MBIP	0.016528
BLLF2	AHSG	0.016834
BLLF2	DDIT4L	0.035423
BLLF2	ARRDC4	0.030131
BLLF2	CNIH4	0.033279
BLLF2	SYN3	0.010331
BLLF2	EIF2S1	0.016997
BLLF2	TAF1C	0.078148
BLLF2	ATP1B1	0.022337
BLLF2	CEP63	0.016893
BLLF2	TICAM1	0.01367
BLLF2	PSMB5	0.013624
BLLF2	ATXN2	0.030219
BLLF2	CD8A	0.018866
BLLF2	SSX2	0.023619
BLLF2	C11orf44	0.052585
BLLF2	LENG8	0.041758
BLLF2	MTUS2	0.037762
BLLF2	CRYL1	0.012312
BLLF2	VWA9	0.032329
BLLF2	UNG	0.13093
BLLF2	MTTP	0.13351
BLLF2	TREM2	0.038135
BLLF2	PYY	0.015734
BLLF2	MYOZ2	0.15296
BLLF2	C16orf72	0.12134
BLLF2	PRODH2	0.026547
BLLF2	TM9SF2	0.037781
BLLF2	UGGT1	0.040845
BLLF2	GADD45G	0.071235
BLLF2	SEC11B	0.018233
BLLF2	HNRNPH2	0.019875
BLLF2	THAP9	0.019585
BLLF2	BEND2	0.054841
BLLF2	TMEM201	0.034394
BLLF2	GALNS	0.013465
BLLF2	MOCS3	0.010602
BLLF2	ZNF732	0.012942
BLLF2	BAG2	0.017498

BLLF2	EIF4B	0.030584
BLLF2	LAPTM4A	0.12739
BLLF2	ZNF416	0.016813
BLLF2	ZNF648	0.016891
BLLF2	TTLL11	0.010826
BLLF2	HPSE2	0.033994
BLLF2	IL6	0.012901
BLLF2	ZNF589	0.020402
BLLF2	VMP1	0.058335
BLLF2	DKKL1	0.13032
BLLF2	CCDC79	0.031772
BLLF2	EXOC3L2	0.050171
BLLF2	PNPLA2	0.02827
BLLF2	UNC119	0.084539
BLLF2	JMJD7	0.012136
BLLF2	SLC52A2	0.1122
BLLF2	KIAA1429	0.0708
BLLF2	POM121L2	0.071318
BLLF2	DGCR8	0.017429
BLLF2	GOLGA6L10	0.099402
BLLF2	POLE2	0.015559
BLLF2	SUPT4H1	0.014901
BLLF2	TSPAN13	0.015148
BLLF2	RIC8B	0.044698
BLLF2	MS4A4A	0.02686
BLLF2	FAM188B	0.17722
BLLF2	PEG3	0.013852
BLLF2	OR2V2	0.081986
BLLF2	ZKSCAN3	0.018982
BLLF2	MISP	0.1306
BLLF2	CCDC180	0.075914
BLLF2	ZNF80	0.019842
BLLF2	RHCG	0.051981
BLLF2	GXYLT1	0.025654
BLLF2	ZNF157	0.016416
BLLF2	ANTXR1	0.042849
BLLF2	ZNF154	0.016088
BLLF2	FAM231B	0.10322
BLLF2	POMT2	0.029124
BLLF2	ZNF492	0.022475
BLLF2	KCNJ12	0.020594
BLLF2	ZNF667	0.011609
BLLF2	ZNF658	0.017
BLLF2	DBN1	0.020476
BLLF2	MSL3	0.014878
BLLF2	AK8	0.018787
BLLF2	LDHAL6A	0.034171
BLLF2	YIPF6	0.066039
BLLF2	ZSCAN12	0.020261
BLLF2	SELO	0.1038
BLLF2	MON1B	0.066118
BLLF2	GPR75	0.013468
BLLF2	ZNF728	0.011684
BLLF2	TIFA	0.03477
BLLF2	FAM27D1	0.074003
BLLF2	ZDHHC15	0.010203
BLLF2	NUCB2	0.012419
BLLF2	SMIM19	0.051963
BLLF2	MGAT1	0.051232
BLLF2	TF	0.013774
BLLF2	EXPH5	0.021119
BLLF2	RAD9A	0.036406
BLLF2	UHRF1BP1L	0.096233
BLLF2	CDKN2A	0.01331
BLLF2	SHANK2-AS3	0.033226
BLLF2	STEAP2	0.01651
BLLF2	UNQ6126/PRO20091	0.071659
BLLF2	H2BFWT	0.016553

BLLF2	OR1K1	0.083407
BLLF2	SESN1	0.02047
BLLF2	NPC2	0.086457
BLLF2	EME2	0.088869
BLLF2	POLR2J3	0.0129
BLLF2	MAGEA11	0.01617
BLLF2	HIN1L	0.012612
BLLF2	TTC26	0.015346
BLLF2	ATXN7L2	0.036584
BLLF2	ZNF786	0.022109
BLLF2	GOLGA6D	0.052161
BLLF2	TIMM17B	0.015618
BLLF2	PSMD5	0.045254
BLLF2	NNT	0.017417
BLLF2	PEX11A	0.10539
BLLF2	APOF	0.037837
BLLF2	MS4A14	0.018733
BLLF2	YGL014C-A	0.12515
BLLF2	GIGYF2	0.024706
BLLF2	AOC1	0.02453
BLLF2	VHL	0.041828
BLLF2	CDCP1	0.043441
BLLF2	SEMG2	0.04573
BLLF2	SMARCD3	0.010234
BLLF2	OAS1	0.049427
BLLF2	NBPF8	0.027605
BLLF2	AFAP1L1	0.017919
BLLF2	RHBG	0.032786
BLLF2	FAM83H	0.032837
BLLF2	IPO13	0.011343
BLLF2	HENMT1	0.017234
BLLF2	EPB42	0.030859
BLLF2	TERF2IP	0.072707
BLLF2	SLC39A5	0.025971
BLLF2	THRSP	0.095072
BLLF2	MED13L	0.031544
BLLF2	IKBKAP	0.020776
BLLF2	NUBP1	0.01236
BLLF2	NT5DC4	0.025689
BLLF2	INSL4	0.076437
BLLF2	UBR4	0.021948
BLLF2	CCDC151	0.022845
BLLF2	PAPOLG	0.024859
BLLF2	ZNF629	0.017634
BLLF2	SYNPO	0.083624
BLLF2	ZMYM3	0.027195
BLLF2	IGFLR1	0.12812
BLLF2	CHST13	0.050034
BLLF2	GGNBP2	0.16349
BLLF2	CT55	0.11335
BLLF2	RFC5	0.013424
BLLF2	PPP6R3	0.06313
BLLF2	JAKMIP2	0.044099
BLLF2	EPG5	0.07841
BLLF2	B3GNTL1	0.035816
BLLF2	SPATA4	0.062299
BLLF2	KIAA1009	0.015154
BLLF2	TTLL4	0.02379
BLLF2	NXT1	0.030814
BLLF2	UPRT	0.054539
BLLF2	ZC3H12D	0.029917
BLLF2	EPC1	0.027089
BLLF2	DGCR14	0.073747
BLLF2	FLG	0.061318
BLLF2	TSPYL2	0.016827
BLLF2	PAQR6	0.01517
BLLF2	ZNF605	0.017414
BLLF2	RILPL1	0.032869

BLLF2	CHMP7	0.054675
BLLF2	PIH1D1	0.033845
BLLF2	VPS29	0.022694
BLLF2	EFNA5	0.010815
BLLF2	PIGC	0.038317
BLLF2	NO66	0.032959
BLLF2	PAQR5	0.018142
BLLF2	NIPAL4	0.1085
BLLF2	ZNF71	0.016033
BLLF2	CMTM2	0.1054
BLLF2	FAM126B	0.11127
BLLF2	KCNJ8	0.018168
BLLF2	ZFP82	0.013394
BLLF2	SLC23A3	0.042843
BLLF2	RRS1	0.053927
BLLF2	DET1	0.021994
BLLF2	FAM131A	0.078837
BLLF2	HOMEZ	0.021399
BLLF2	RABEP2	0.17848
BLLF2	FCAMR	0.026336
BLLF2	LVP1a	0.026485
BLLF2	ZNF835	0.017274
BLLF2	SLC18A1	0.014047
BLLF2	SEL1L3	0.030406
BLLF2	CYB561	0.082545
BLLF2	XPO6	0.035976
BLLF2	SERTAD1	0.12808
BLLF2	SYS1	0.065995
BLLF2	IFI27	0.1124
BLLF2	RWDD4	0.027486
BLLF2	MTRNR2L1	0.10682
BLLF2	INTS4	0.069792
BLLF2	DYNLRB1	0.06498
BLLF2	GYPC	0.026468
BLLF2	EGLN2	0.034954
BLLF2	CHAF1A	0.22754
BLLF2	BTF3	0.017532
BLLF2	KIAA0319L	0.023259
BLLF2	ARSJ	0.014271
BLLF2	SYAP1	0.042957
BLLF2	OR10R2	0.073068
BLLF2	UNQ5830/PRO19650/PRO19816	0.042477
BLLF2	PTCRA	0.051031
BLLF2	SAA1	0.089702
BLLF2	PTGES3L	0.035416
BLLF2	TP53INP1	0.11655
BLLF2	TAS2R39	0.056247
BLLF2	RPS16	0.049063
BLLF2	SERINC4	0.027106
BLLF2	ECH1	0.012699
BLLF2	CD300A	0.011447
BLLF2	ZNF502	0.018212
BLLF2	ZNF699	0.010721
BLLF2	ZNF354B	0.011372
BLLF2	ATXN7L3	0.067245
BLLF2	MNCb-2875	0.041888
BLLF2	ZMYM6	0.031932
BLLF2	CATSPERB	0.12495
BLLF2	FAM127C	0.044712
BLLF2	MCM10	0.060153
BLLF2	ZNF792	0.014054
BLLF2	CBWD7	0.045851
BLLF2	EIF1B	0.06361
BLLF2	PTTG1	0.018952
BLLF2	MUC12	0.047147
BLLF2	SHMT2	0.029786
BLLF2	TBC1D3C	0.013146
BLLF2	CLUAP1	0.1381

BLLF2	NUP160	0.033458
BLLF2	NUP50	0.026315
BLLF2	GPATCH3	0.035258
BLLF2	SLC52A1	0.1122
BLLF2	ADAT3	0.04973
BLLF2	PGLYRP2	0.014994
BLLF2	SYBU	0.03063
BLLF2	LECT1	0.01553
BLLF2	CLCA2	0.020154
BLLF2	C8orf74	0.05388
BLLF2	NOC4L	0.027223
BLLF2	CCDC104	0.030973
BLLF2	YOD1	0.031411
BLLF2	FXYD7	0.020233
BLLF2	ARL6IP1	0.023049
BLLF2	TMEM52	0.053901
BLLF2	BLOC1S4	0.034217
BLLF2	FAIM3	0.040189
BLLF2	THAP3	0.054547
BLLF2	MAB21L2	0.04784
BLLF2	XAGE3	0.16542
BLLF2	C11orf65	0.029395
BLLF2	GGN	0.16577
BLLF2	ZNF256	0.024172
BLLF2	ASAH2B	0.010755
BLLF2	CCDC85A	0.010297
BLLF2	MUC17	0.012459
BLLF2	C1orf111	0.087112
BLLF2	NME6	0.042374
BLLF2	ZNF587	0.017221
BLLF2	BPGM	0.054392
BLLF2	COX18	0.03102
BLLF2	CSF1	0.04343
BLLF2	PMFBP1	0.035709
BLLF2	TCL1B	0.010622
BLLF2	PFDN2	0.050785
BLLF2	AMPD1	0.015525
BLLF2	DLGAP2	0.013157
BLLF2	TSPY2	0.018872
BLLF2	SLC36A3	0.01532
BLLF2	OR2AE1	0.084698
BLLF2	WFDC1	0.061787
BLLF2	GNG5	0.020585
BLLF2	HINT3	0.014268
BLLF2	CCDC87	0.013628
BLLF2	MTERFD3	0.043454
BLLF2	SLC4A9	0.033657
BLLF2	MIS18BP1	0.078967
BLLF2	IL17RB	0.057736
BLLF2	EXOSC9	0.03982
BLLF2	ZNF664	0.01411
BLLF2	TMEM120B	0.046207
BLLF2	G4	0.02086
BLLF2	BEX5	0.067097
BLLF2	PAQR9	0.010354
BLLF2	LAMP1	0.081041
BLLF2	CHGB	0.23749
BLLF2	ZNF788	0.012141
BLLF2	EARS2	0.029197
BLLF2	FAM90A23P	0.012963
BLLF2	ZNF620	0.018383
BLLF2	SLC16A11	0.067898
BLLF2	JMJD8	0.010634
BLLF2	CMTR2	0.046172
BLLF2	IMP3	0.012524
BLLF2	TMEM204	0.17286
BLLF2	TMEM178B	0.031645
BLLF2	ZNF567	0.023779

BLLF2	FH	0.050247
BLLF2	EQTN	0.097178
BLLF2	ZNF233	0.018646
BLLF2	SLC1A7	0.020023
BLLF2	HERPUD2	0.09389
BLLF2	TGM7	0.017337
BLLF2	ZNF34	0.017221
BLLF2	ZNF107	0.011897
BLLF2	VPS36	0.044041
BLLF2	ZC3H13	0.038557
BLLF2	CD3E	0.017326
BLLF2	LGALS9B	0.021753
BLLF2	NT5C3A	0.089133
BLLF2	SART1	0.08238
BLLF2	MT-CO1	0.045901
BLLF2	VPS16	0.025794
BLLF2	MTX1	0.052631
BLLF2	IL9R	0.015241
BLLF2	PRAMEF6	0.016732
BLLF2	MAGED4	0.014458
BLLF2	IBA57	0.083829
BLLF2	EIF3L	0.035809
BLLF2	ATCAY	0.0191
BLLF2	PPA1	0.021103
BLLF2	G4	0.094338
BLLF2	FAM162B	0.10896
BLLF2	MPPED2	0.028833
BLLF2	TMED5	0.017106
BLLF2	TCTN3	0.041052
BLLF2	GINS1	0.037025
BLLF2	DSCR10	0.046719
BLLF2	C5orf38	0.026496
BLLF2	ATXN7L3B	0.053054
BLLF2	UGGT2	0.029405
BLLF2	TUBGCP4	0.018949
BLLF2	MTRNR2L8	0.10682
BLLF2	OR2M7	0.077525
BLLF2	EXOSC4	0.013963
BLLF2	PI4KAP1	0.012268
BLLF2	MT1L	0.065486
BLLF2	SYCP2	0.015939
BLLF2	ABCF3	0.012979
BLLF2	LINC00596	0.032357
BLLF2	SCAMP5	0.036502
BLLF2	PPP1R3E	0.057795
BLLF2	WLS	0.050647
BLLF2	AIM1	0.043308
BLLF2	NOC2L	0.011612
BLLF2	SUPT6H	0.01409
BLLF2	TBC1D17	0.018388
BLLF2	CEP250	0.018544
BLLF2	EIF3D	0.015973
BLLF2	DGCR6L	0.027678
BLLF2	IFT52	0.098043
BLLF2	RPS10P5	0.017467
BLLF2	CT45A3	0.037307
BLLF2	ZNF470	0.011494
BLLF2	TMEM81	0.046991
BLLF2	ZNF726P1	0.010974
BLLF2	LUZP6	0.017699
BLLF2	SCP2D1	0.011671
BLLF2	CD47	0.033299
BLLF2	CHMP3	0.020488
BLLF2	H2AFY2	0.010406
BLLF2	SYNC	0.010455
BLLF2	TIMM8B	0.033099
BLLF2	ZC3H12A	0.028711
BLLF2	CCP110	0.067818

BLLF2	ST3GAL4	0.013035
BLLF2	LSM2	0.011974
BLLF2	CAGE1	0.026529
BLLF2	VWA5A	0.083097
BLLF2	NOMO3	0.025349
BLLF2	SSX4	0.014765
BLLF2	TMEM220	0.011013
BLLF2	LINC00205	0.079441
BLLF2	SLC35B2	0.015358
BLLF2	MLH3	0.031616
BLLF2	FAM46B	0.068611
BLLF2	TCEB1	0.022673
BLLF2	FEZ2	0.063333
BLLF2	NFYA	0.024639
BLLF2	PNAS-138	0.087014
BLLF2	DROSHA	0.018584
BLLF2	ZNF180	0.011639
BLLF2	CD2BP2	0.054366
BLLF2	CD2	0.012507
BLLF2	CLDN20	0.014059
BLLF2	GPN2	0.011322
BLLF2	ACOT8	0.088043
BLLF2	TPPP3	0.081355
BLLF2	SAP130	0.13098
BLLF2	IFNA21	0.020126
BLLF2	MAN2A2	0.017795
BLLF2	NCOA6	0.12421
BLLF2	HTN1	0.12975
BLLF2	CCDC26	0.086275
BLLF2	NSUN5P2	0.068617
BLLF2	ZNF408	0.017213
BLLF2	RPS21	0.013511
BLLF2	GRAMD1A	0.020461
BLLF2	CRYBB3	0.043308
BLLF2	GFOD2	0.015441
BLLF2	MT1F	0.063593
BLLF2	CCDC85C	0.092961
BLLF2	GGT2	0.015156
BLLF2	TXLNG2P	0.05125
BLLF2	TGM1	0.029454
BLLF2	TCEAL2	0.016062
BLLF2	FAM181A	0.031851
BLLF2	CCDC125	0.033408
BLLF2	ZNF160	0.01366
BLLF2	GTPBP10	0.073292
BLLF2	RPL23	0.073418
BLLF2	SUPT20HL1	0.10332
BLLF2	TMEM8B	0.025124
BLLF2	TSPAN9	0.015917
BLLF2	SPATA31A6	0.089472
BLLF2	CAB39L	0.011727
BLLF2	ZNF573	0.012141
BLLF2	SUMF1	0.043879
BLLF2	SLC14A2	0.10439
BLLF2	ECHDC1	0.015697
BLLF2	NFXL1	0.014716
BLLF2	RICTOR	0.022682
BLLF2	C7orf57	0.024099
BLLF2	GBP3	0.015629
BLLF2	LY96	0.067303
BLLF2	ZNF738	0.017539
BLLF2	RPL10L	0.044083
BLLF2	ZNF778	0.022224
BLLF2	ACOT6	0.01021
BLLF2	C22orf43	0.048585
BLLF2	PROK2	0.034516
BLLF2	RIC3	0.033408
BLLF2	MAP7	0.051945

BLLF2	RPL26L1	0.059677
BLLF2	RPS2	0.020898
BLLF2	UTF1	0.028644
BLLF2	IL4R	0.019454
BLLF2	ZDHHC24	0.010719
BLLF2	PTPLA	0.010256
BLLF2	ANTXRL	0.011536
BLLF2	Gm5153	0.020282
BLLF2	FAM166B	0.013411
BLLF2	MAGEC1	0.022534
BLLF2	SAP25	0.10254
BLLF2	EIF4G3	0.013788
BLLF2	ZDHHC4	0.010168
BLLF2	MT1G	0.087702
BLLF2	MID1IP1	0.089757
BLLF2	DLGAP5	0.033531
BLLF2	NUP35	0.014244
BLLF2	AHSP	0.091041
BLLF2	VWA5B2	0.06276
BLLF2	CLDN24	0.028093
BLLF2	KCNJ11	0.014501
BLLF2	UVRAG	0.021281
BLLF2	IP6K3	0.042011
BLLF2	RNASE6	0.019974
BLLF2	PEPD	0.054003
BLLF2	ZNF571	0.011851
BLLF2	PELI2	0.035055
BLLF2	NKAIN2	0.12482
BLLF2	NAF1	0.1253
BLLF2	ZBTB49	0.016552
BLLF2	MLLT3	0.011905
BLLF2	PRAME	0.13851
BLLF2	EIF4E2	0.017694
BLLF2	SLCO5A1	0.021992
BLLF2	ENPP3	0.012175
BLLF2	TAS2R10	0.058873
BLLF2	AFF3	0.036365
BLLF2	UTP14C	0.053799
BLLF2	ZNF20	0.020382
BLLF2	G6PC3	0.042171
BLLF2	TMSB10	0.1054
BLLF2	ZNF304	0.016158
BLLF2	MB	0.058723
BLLF2	DNASE1	0.020348
BLLF2	EXOC6	0.024604
BLLF2	EIF2A	0.011989
BLLF2	AGRP	0.089762
BLLF2	CDIP1	0.015067
BLLF2	ATG12	0.023656
BLLF2	MAP7D2	0.018294
BLLF2	SLC10A1	0.013619
BLLF2	C1GALT1C1	0.018651
BLLF2	PBOV1	0.093125
BLLF2	ZNF702P	0.016231
BLLF2	RPS4Y2	0.0237
BLLF2	OR9K2	0.081986
BLLF2	NAPA	0.026549
BLLF2	ZNF267	0.020413
BLLF2	FAM20B	0.037128
BLLF2	DRAXIN	0.056828
BLLF2	PGRMC2	0.025448
BLLF2	APOL6	0.068424
BLLF2	BZW1	0.021807
BLLF2	FAM149B1	0.070668
BLLF2	ZNF333	0.015273
BLLF2	FTSJ1	0.092272
BLLF2	KCNJ6	0.013039
BLLF2	PDSS1	0.023474

BLLF2	BUD13	0.045248
BLLF2	LRRFIP1	0.055381
BLLF2	PPP2R3C	0.056602
BLLF2	RRP36	0.037219
BLLF2	NAA25	0.019242
BLLF2	MT1H	0.065486
BLLF2	ATG10	0.060522
BLLF2	ZFR2	0.013051
BLLF2	SNRPD2	0.016566
BLLF2	ZNF37A	0.017688
BLLF2	SSX2IP	0.024313
BLLF2	ACD	0.05764
BLLF2	DPP4	0.015701
BLLF2	TAS2R40	0.052995
BLLF2	EIF3E	0.027378
BLLF2	FRG2C	0.09888
BLLF2	EIF4E	0.026145
BLLF2	STAG3L4	0.034299
BLLF2	DSCC1	0.028172
BLLF2	TMEM132E	0.052004
BLLF2	ZNF747	0.011243
BLLF2	BBC3	0.12838
BLLF2	SDC2	0.018985
BLLF2	GPR89C	0.01054
BLLF2	ATP6V1F	0.012478
BLLF2	OAS3	0.043622
BLLF2	DCUN1D3	0.018368
BLLF2	RQCD1	0.095341
BLLF2	NPB	0.13319
BLLF2	LMF2	0.095921
BLLF2	ATP5F1	0.037614
BLLF2	RPL6	0.076891
BLLF2	MIF4GD	0.073882
BLLF2	C19orf12	0.091652
BLLF2	ZNF137P	0.01084
BLLF2	SPAC22F8.05	0.012734
BLLF2	ISYNA1	0.06696
BLLF2	ITPKA	0.09923
BLLF2	RAD1	0.047469
BLLF2	C12orf63	0.040695
BLLF2	SLC16A10	0.045174
BLLF2	SMIM1	0.088415
BLLF2	GPNMB	0.013207
BLLF2	PDYN	0.025882
BLLF2	TAS2R1	0.062538
BLLF2	UCKL1	0.045116
BLLF2	MED15	0.028563
BLLF2	ADAT2	0.011717
BLLF2	DMRT2	0.014356
BLLF2	ZNF3	0.014101
BLLF2	IPO7	0.044041
BLLF2	LEMD2	0.054371
BLLF2	SGCG	0.030728
BLLF2	ZPBP	0.022785
BLLF2	DPM2	0.10129
BLLF2	TPPP2	0.039914
BLLF2	FXYD5	0.022031
BLLF2	NTMT1	0.012773
BLLF2	LETMD1	0.018715
BLLF2	SEC11C	0.077012
BLLF2	FAM46A	0.069863
BLLF2	PPP2R5E	0.013993
BLLF2	PIN4	0.010106
BLLF2	PLGLA	0.060007
BLLF2	TSN	0.070997
BLLF2	SLC29A4	0.017841
BLLF2	LSM14A	0.093562
BLLF2	CDC123	0.045215

BLLF2	FRAT1	0.08831
BLLF2	DGCR6	0.027291
BLLF2	RILP	0.085929
BLLF2	SS18L2	0.033395
BLLF2	GVINP1	0.010561
BLLF2	SDS	0.022697
BLLF2	TSPY3	0.018872
BLLF2	PPIP5K1	0.028888
BLLF2	LMAN1	0.022937
BLLF2	PTGER1	0.014211
BLLF2	DOPEY2	0.025493
BLLF2	UCN3	0.13891
BLLF2	SDHA	0.014723
BLLF2	ZNF597	0.029322
BLLF2	SRFBP1	0.013467
BLLF2	WASF1	0.012628
BLLF2	CTIF	0.030549
BLLF2	GCLM	0.015277
BLLF2	PMS1	0.017261
BLLF2	GNPAT	0.013328
BLLF2	OR4F3	0.07986
BLLF2	ZNF439	0.02135
BLLF2	TRAP1	0.024285
BLLF2	CD3D	0.012158
BLLF2	TCEAL5	0.048702
BLLF2	PEX14	0.058902
BLLF2	GAGE12J	0.029493
BLLF2	JOSD2	0.020513
BLLF2	TMEM41B	0.029913
BLLF2	NIPAL1	0.1085
BLLF2	RPGRIP1	0.023908
BLLF2	SPG21	0.02633
BLLF2	ZFP41	0.016118
BLLF2	OR10AD1	0.077068
BLLF2	SPPL2C	0.027992
BLLF2	POLR2J	0.019467
BLLF2	FAM3A	0.010116
BLLF2	DEHA2C16280g	0.038459
BLLF2	KCNJ13	0.012123
BLLF2	IGFL1	0.14626
BLLF2	ZFP37	0.016196
BLLF2	ZNF259	0.035858
BLLF2	IGBP1	0.068173
BLLF2	POM121	0.11889
BLLF2	TREH	0.027781
BLLF2	KHNYN	0.05627
BLLF2	TNFSF9	0.064518
BLLF2	DIRC2	0.026686
BLLF2	G4	0.029436
BLLF2	COBL	0.072395
BLLF2	GBE1	0.092297
BLLF2	PRNP	0.040398
BLLF2	ZNF619	0.015187
BLLF2	ZNF324	0.014585
BLLF2	GAGE2A	0.0533
BLLF2	NARS2	0.034313
BLLF2	PORCN	0.061426
BLLF2	MS4A15	0.02686
BLLF2	STC2	0.025635
BLLF2	ZSCAN23	0.018723
BLLF2	DEFB129	0.1188
BLLF2	IFNA17	0.013659
BLLF2	ZNF483	0.013394
BLLF2	FAM110B	0.1063
BLLF2	ATXN7L1	0.036584
BLLF2	APOL4	0.011089
BLLF2	ZNF454	0.013405
BLLF2	ZNF230	0.023115

BLLF2	OR2AG2	0.084698
BLLF2	IFNLR1	0.030517
BLLF2	LSM12	0.091756
BLLF2	ZNF544	0.022609
BLLF2	NDNL2	0.01645
BLLF2	ZER1	0.018895
BLLF2	ROPN1L	0.016974
BLLF2	CRYGB	0.020753
BLLF2	UCN	0.048037
BLLF2	TRAPPC9	0.075476
BLLF2	NCDN	0.074246
BLLF2	CRAMP1L	0.036907
BLLF2	GC	0.032677
BLLF2	MSMO1	0.020372
BLLF2	ZNF799	0.011346
BLLF2	MANF	0.017399
BLLF2	ZNF543	0.020718
BLLF2	KIAA0907	0.021082
BLLF2	C12orf57	0.072921
BLLF2	SLC18B1	0.028627
BLLF2	ALKBH2	0.074084
BLLF2	TMEM8A	0.093453
BLLF2	IRF2BPL	0.021707
BLLF2	MCCC2	0.019866
BLLF2	CLUL1	0.015243
BLLF2	ATP6V0B	0.015948
BLLF2	IGFL4	0.17696
BLLF2	CRTC2	0.10458
BLLF2	HIC1	0.012755
BLLF2	ZKSCAN5	0.020139
BLLF2	TAF4	0.014955
BLLF2	FAM228A	0.014904
BLLF2	YAE1D1	0.091099
BLLF2	TMEM64	0.024463
BLLF2	FSCN3	0.013011
BLLF2	ZNF81	0.016095
BLLF2	MTRNR2L6	0.10682
BLLF2	MREG	0.013597
BLLF2	NEURL4	0.015859
BLLF2	CD82	0.012741
BLLF2	CT45A1	0.038807
BLLF2	ERICH1	0.080173
BLLF2	PYHIN1	0.012324
BLLF2	MOB4	0.010448
BLLF2	GIF	0.10729
BLLF2	CTF1	0.01824
BLLF2	POLR2A	0.014946
BLLF2	GGT7	0.034697
BLLF2	SPTSSA	0.014153
BLLF2	NAA30	0.0101
BLLF2	ZNF221	0.01293
BLLF2	DCP1A	0.023939
BLLF2	YARS	0.010545
BLLF2	AMOTL2	0.016217
BLLF2	GATAD2B	0.011025
BLLF2	PPP4R2	0.11523
BLLF2	RTP3	0.035267
BLLF2	STAG3L3	0.047809
BLLF2	G4	0.038787
BLLF2	ZNF823	0.020698
BLLF2	GPRIN1	0.014202
BLLF2	ZNF878	0.01476
BLLF2	ENKUR	0.011279
BLLF2	TMCO1	0.072878
BLLF2	IMMP2L	0.022366
BLLF2	PELI1	0.028673
BLLF2	GUCA2B	0.023033
BLLF2	MTR	0.023552

BLLF2	LIN9	0.028939
BLLF2	LGALS12	0.027737
BLLF2	BVES-AS1	0.062689
BLLF2	SDHD	0.076977
BLLF2	NDUFS5	0.062542
BLLF2	TCEAL1	0.12888
BLLF2	GNL3L	0.011676
BLLF2	MARCKS	0.17265
BLLF2	CACHD1	0.026814
BLLF2	SGCD	0.026617
BLLF2	TMSB4X	0.1228
BLLF2	METTL3	0.013015
BLLF2	AMELY	0.06063
BLLF2	LEPREL2	0.050427
BLLF2	CCDC127	0.026028
BLLF2	FLG2	0.024356
BLLF2	CCDC120	0.090114
BLLF2	LIM2	0.012425
BLLF2	SPATA31E1	0.30742
BLLF2	ZNF385C	0.030446
BLLF2	POLR2L	0.10067
BLLF2	MAGEB16	0.013808
BLLF2	IFNW1	0.03465
BLLF2	C1orf145	0.12673
BLLF2	ZNF701	0.010115
BLLF2	TBC1D5	0.013329
BLLF2	UBP1	0.013161
BLLF2	AKAP9	0.059578
BLLF2	SDC3	0.017481
BLLF2	ASPHD2	0.016651
BLLF2	NUDC	0.062723
BLLF2	SYNE3	0.017511
BLLF2	NYAP2	0.082529
BLLF2	AMY2A	0.03212
BLLF2	ZFP69	0.014226
BLLF2	PANK3	0.12734
BLLF2	OTUD1	0.032171
BLLF2	CPA3	0.011933
BLLF2	TMEM158	0.11192
BLLF2	MAB21L1	0.032244
BLLF2	GPRASP1	0.071878
BLLF2	ZNF124	0.01805
BLLF2	ZNF7	0.015317
BLLF2	VGLL3	0.064674
BLLF2	SFI1	0.1457
BLLF2	C1orf186	0.038205
BLLF2	C2orf71	0.10786
BLLF2	ANO10	0.022073
BLLF2	SLC5A12	0.013969
BLLF2	SCLY	0.014237
BLLF2	DHFR	0.035021
BLLF2	FBLL1	0.015825
BLLF2	SMEK2	0.038401
BLLF2	PELI3	0.031675
BLLF2	OR2T4	0.084698
BLLF2	PRRG4	0.047515
BLLF2	TRDN	0.060189
BLLF2	CD300LB	0.013526
BLLF2	GPR111	0.011988
BLLF2	EIF5A2	0.015992
BLLF2	DSCR9	0.043315
BLLF2	ZNF710	0.019541
BLLF2	OAZ1	0.020321
BLLF2	VGLL2	0.033162
BLLF2	ST6GALNAC4	0.015995
BLLF2	UBXN8	0.05478
BLLF2	APCS	0.043267
BLLF2	MSH5	0.040152

BLLF2	PPP2R3A	0.015753
BLLF2	GSPT1	0.014622
BLLF2	ZNF599	0.018124
BLLF2	AKAP3	0.063699
BLLF2	BAG5	0.028319
BLLF2	GOLGA8I	0.049645
BLLF2	LYRM4	0.071808
BLLF2	EIF2B4	0.014891
BLLF2	RHAG	0.036186
BLLF2	TRABD2A	0.059153
BLLF2	OMP	0.071942
BLLF2	USE1	0.029572
BLLF2	LDHB	0.035408
BLLF2	PEX11B	0.029836
BLLF2	ZNF850	0.012401
BLLF2	PMP22	0.023076
BLLF2	LGALS9	0.021753
BLLF2	MS4A5	0.013303
BLLF2	CD164	0.092099
BLLF2	NFIX	0.014875
BLLF2	TBC1D15	0.010921
BLLF2	AOC3	0.034132
BLLF2	ZNF23	0.017414
BLLF2	ZNF680	0.013452
BLLF2	IFNA16	0.054688
BLLF2	BLOC1S1	0.11868
BLLF2	FAM195A	0.14649
BLLF2	ARPP21	0.022687
BLLF2	PRDM9	0.013289
BLLF2	PGAM5	0.010655
BLLF2	RGAG1	0.036046
BLLF2	GAST	0.066062
BLLF2	TRMT11	0.030443
BLLF2	PGLS	0.012825
BLLF2	EXOC8	0.069185
BLLF2	ZNF425	0.017329
BLLF2	LARGE	0.017243
BLLF2	MCOLN1	0.011666
BLLF2	CGGBP1	0.085582
BLLF2	TIGD1	0.062166
BLLF2	GTSF1	0.17088
BLLF2	FAM134B	0.05259
BLLF2	KCNE1L	0.034399
BLLF2	CDC42SE1	0.027614
BLLF2	SCAPER	0.025424
BLLF2	OBFC1	0.012966
BLLF2	Pom121l2	0.058933
BLLF2	CIDEC	0.034874
BLLF2	ITFG1	0.10678
BLLF2	PNPT1	0.053345
BLLF2	MAP7D1	0.027243
BLLF2	UPF3B	0.14885
BLLF2	CEP89	0.04054
BLLF2	RPL30	0.051399
BLLF2	ATG2A	0.018355
BLLF2	LZTFL1	0.021508
BLLF2	NUPR1	0.095825
BLLF2	ZNF668	0.021054
BLLF2	RHBDL1	0.015746
BLLF2	ZNF260	0.024171
BLLF2	C5orf58	0.13316
BLLF2	PDE6G	0.039553
BLLF2	DSPP	0.034199
BLLF2	WFS1	0.030161
BLLF2	KRBOX1	0.022634
BLLF2	ZNF709	0.012141
BLLF2	DHTKD1	0.017961
BLLF2	LHB	0.055234

BLLF2	DLGAP1	0.03249
BLLF2	CEP112	0.01352
BLLF2	TRAPPC12	0.012459
BLLF2	MFI2	0.021334
BLLF2	ZNF319	0.016157
BLLF2	SEC16A	0.026526
BLLF2	ZNF396	0.011095
BLLF2	ZNF845	0.013362
BLLF2	CRTC3	0.10458
BLLF2	NENF	0.030687
BLLF2	OR10Q1	0.076296
BLLF2	PMAIP1	0.059609
BLLF2	OR13C9	0.089594
BLLF2	SPINK1	0.01185
BLLF2	ZNF658B	0.017
BLLF2	ELL2	0.011004
BLLF2	MTX2	0.014585
BLLF2	RPRD2	0.038467
BLLF2	PPP1R3A	0.036415
BLLF2	OR51J1	0.092776
BLLF2	ZNF84	0.014967
BLLF2	RPL10A	0.026025
BLLF2	DPP7	0.13933
BLLF2	G4	0.015703
BLLF2	CD3EAP	0.063901
BLLF2	CCDC169	0.05489
BLLF2	KIAA0513	0.020922
BLLF2	OVCA2	0.035747
BLLF2	C7orf62	0.069356
BLLF2	SLC16A5	0.035936
BLLF2	RPP30	0.049751
BLLF2	FOPNL	0.033012
BLLF2	ZNF431	0.014927
BLLF2	HIST1H2BJ	0.016318
BLLF2	HIBCH	0.012107
BLLF2	EDEM1	0.012645
BLLF2	TBC1D22A	0.011949
BLLF2	NBPF4	0.03035
BLLF2	MKI67	0.050296
BLLF2	LVP1a	0.10059
BLLF2	DGAT2L7P	0.025018
BLLF2	COL26A1	0.14668
BLLF2	TEX2	0.02172
BLLF2	CKS1B	0.013418
BLLF2	OGFOD3	0.047223
BLLF2	CKLF	0.068801
BLLF2	TIMM23	0.044939
BLLF2	TNFAIP2	0.047379
BLLF2	ANO1	0.05304
BLLF2	MB21D2	0.046631
BLLF2	HBD	0.10713
BLLF2	ZNF286A	0.014527
BLLF2	TIMM8A	0.033118
BLLF2	ARMCX5	0.047461
BLLF2	EFTUD1	0.015427
BLLF2	PSMB11	0.010258
BLLF2	ZNF600	0.013362
BLLF2	VMAC	0.14594
BLLF2	IDH3G	0.01878
BLLF2	ZC3H14	0.012963
BLLF2	SUPT7L	0.082388
BLLF2	C5orf64	0.086972
BLLF2	MEPCE	0.016554
BLLF2	KYNU	0.039791
BLLF2	LSM11	0.11571
BLLF2	DZIP1L	0.029611
BLLF2	DFFA	0.018513
BLLF2	ATP5A1	0.029774

BLLF2	FAM90A27P	0.044722
BLLF2	OIP5	0.037303
BLLF2	SDF2	0.010845
BLLF2	SLC24A4	0.012659
BLLF2	CYB561A3	0.071313
BLLF2	B9D2	0.032249
BLLF2	CGB2	0.021509
BLLF2	SNX19	0.010326
BLLF2	THUMPD2	0.016729
BLLF2	NXNL1	0.014589
BLLF2	MRE11A	0.019525
BLLF2	ZNF705E	0.01892
BLLF2	CLPP	0.080171
BLLF2	CHORDC1	0.018032
BLLF2	SMG6	0.019772
BLLF2	ZNF229	0.019274
BLLF2	APOBEC3H	0.019006
BLLF2	FUT1	0.035485
BLLF2	ATL3	0.01703
BLLF2	ECU09	0.021684
BLLF2	MAT2A	0.010471
BLLF2	SLC51A	0.16719
BLLF2	SPATA2L	0.061133
BLLF2	ADGB	0.024919
BLLF2	IFT46	0.020033
BLLF2	AARD	0.040829
BLLF2	LAPTM5	0.13835
BLLF2	CDCA2	0.084212
BLLF2	MGAT3	0.041074
BLLF2	DACT1	0.024333
BLLF2	SLC4A10	0.01127
BLLF2	ATXN1L	0.086117
BLLF2	YRDC	0.052223
BLLF2	RPS3	0.04309
BLLF2	UTP6	0.023953
BLLF2	ZNF782	0.023549
BLLF2	ZIK1	0.018108
BLLF2	RPUSD1	0.031238
BLLF2	TMEM170A	0.011734
BLLF2	CRISPLD2	0.012348
BLLF2	XKRX	0.13736
BLLF2	PHACTR3	0.06504
BLLF2	WIZ	0.021242
BLLF2	C15orf37	0.025932
BLLF2	BBS1	0.053143
BLLF2	ZNF138	0.011589
BLLF2	BTF3L4	0.0344
BLLF2	RNASE7	0.023925
BLLF2	KCNMB1	0.048698
BLLF2	PRPSAP2	0.025008
BLLF2	SPIDR	0.1138
BLLF2	HMGN1	0.047674
BLLF2	HNRNPF	0.011144
BLLF2	ITPA	0.061976
BLLF2	ELOVL1	0.05608
BLLF2	GK2	0.097643
BLLF2	SLC16A9	0.019793
BLLF2	HIF1AN	0.011095
BLLF2	DARC	0.09946
BLLF2	OR5I1	0.088472
BLLF2	SDHC	0.01294
BLLF2	CSTF3	0.016505
BLLF2	TOR1AIP2	0.060394
BLLF2	OR5L2	0.078589
BLLF2	EXOSC7	0.01331
BLLF2	NSUN2	0.034642
BLLF2	SOGA3	0.017048
BLLF2	ZNF500	0.022197

BLLF2	XAGE5	0.08833
BLLF2	SLC39A7	0.025971
BLLF2	MIEN1	0.014589
BLLF2	ZNF526	0.012294
BLLF2	GOLGA8M	0.013656
BLLF2	DALRD3	0.030168
BLLF2	ZNF91	0.013082
BLLF2	SLC30A3	0.011044
BLLF2	PSAP	0.018826
BLLF2	LYRM9	0.12173
BLLF2	UBAP1	0.02176
BLLF2	CMTM7	0.077491
BLLF2	MUC1	0.15431
BLLF2	TMBIM1	0.027388
BLLF2	RANBP3	0.017656
BLLF2	CTNNA1	0.014828
BLLF2	ZNF705D	0.019705
BLLF2	ZNF324B	0.015806
BLLF2	FAM127B	0.069456
BLLF2	VBP1	0.01667
BLLF2	PLN	0.12048
BLLF2	CDK5R1	0.069616
BLLF2	SUB1	0.10379
BLLF2	APOPT1	0.063367
BLLF2	SPATA31A5	0.055724
BLLF2	HAS2	0.011443
BLLF2	OR2L3	0.09263
BLLF2	OR2M2	0.085924
BLLF2	TMEM176B	0.14176
BLLF2	ZNF239	0.015931
BLLF2	ST8SIA6	0.010397
BLLF2	CXorf40B	0.034409
BLLF2	PPP1R14B	0.048158
BLLF2	IL20RB	0.011407
BLLF2	PAGE1	0.067252
BLLF2	ZNF775	0.02393
BLLF2	SYNGR2	0.10314
BLLF2	TRAPPC6A	0.10889
BLLF2	MPV17L2	0.048961
BLLF2	TARS	0.027169
BLLF2	WFDC9	0.029436
BLLF2	CDC42EP3	0.048992
BLLF2	MAN2C1	0.01898
BLLF2	EIF4EBP2	0.052127
BLLF2	SEL1L2	0.012448
BLLF2	CACNA2D3	0.059466
BLLF2	FAM168B	0.028516
BLLF2	TAS2R19	0.07066
BLLF2	RARS2	0.023325
BLLF2	PDK2	0.013932
BLLF2	DACT2	0.024345
BLLF2	ZNF329	0.022146
BLLF2	PODXL2	0.060919
BLLF2	RFC2	0.011346
BLLF2	TYROBP	0.010456
BLLF2	ZNF418	0.017414
BLLF2	RNASEK	0.018816
BLLF2	MCEMP1	0.038661
BLLF2	RTKN	0.022585
BLLF2	SGD1	0.060584
BLLF2	PRKCDBP	0.036566
BLLF2	ZNF527	0.011142
BLLF2	PDE6D	0.03393
BLLF2	GADD45A	0.070707
BLLF2	BSDC1	0.01367
BLLF2	ORAI2	0.018518
BLLF2	LYZL1	0.071427
BLLF2	IL32	0.095098

BLLF2	DKK1	0.016687
BLLF2	IL17RC	0.12135
BLLF2	ITM2C	0.040124
BLLF2	RNASE8	0.013274
BLLF2	USP54	0.042347
BLLF2	MMAA	0.011422
BLLF2	GOLGA6L19	0.099402
BLLF2	SNRPB	0.011571
BLLF2	PCBD2	0.045142
BLLF2	PPP2R4	0.029115
BLLF2	RTCA	0.033595
BLLF2	LURAP1	0.15863
BLLF2	POPDC2	0.042931
BLLF2	XXYLT1	0.026232
BLLF2	CIDEA	0.025285
BLLF2	SPINK7	0.01137
BLLF2	CMTM6	0.072578
BLLF2	MCMBP	0.012592
BLLF2	TMEM248	0.10812
BLLF2	ZNF271	0.016444
BLLF2	C8orf88	0.050394
BLLF2	RPL12	0.014502
BLLF2	NMRAL1	0.018981
BLLF2	TSC22D1	0.010876
BLLF2	C5orf34	0.050725
BLLF2	METTL14	0.02192
BLLF2	GAS2L2	0.017077
BLLF2	LURAP1L	0.13312
BLLF2	XKR6	0.091992
BLLF2	TMEM33	0.036595
BLLF2	ZNF136	0.014073
BLLF2	HINT2	0.010423
BLLF2	PDHB	0.019566
BLLF2	RPL36AL	0.018542
BLLF2	SPATA31A3	0.055724
BLLF2	MAU2	0.023403
BLLF2	C19orf47	0.038839
BLLF2	C4orf33	0.017358
BLLF2	C12orf50	0.11094
BLLF2	NPTX2	0.040323
BLLF2	C17orf102	0.064345
BLLF2	RMND5B	0.014893
BLLF2	ACRV1	0.012182
BLLF2	IQCG	0.043287
BLLF2	CTAGE8	0.023259
BLLF2	TRGC1	0.017044
BLLF2	KIAA1598	0.057265
BLLF2	HIST1H1D	0.01681
BLLF2	SNX31	0.025902
BLLF2	GGTLC2	0.029477
BLLF2	OR2H1	0.088474
BLLF2	LARP6	0.024385
BLLF2	ZNF583	0.013405
BLLF2	ARHGDIG	0.01309
BLLF2	TMED1	0.034651
BLLF2	PDCD2L	0.065624
BLLF2	TIMP1	0.035853
BLLF2	TOM1L2	0.012846
BLLF2	ZNF391	0.017434
BLLF2	SLC16A3	0.051862
BLLF2	ASIC4	0.012673
BLLF2	RPS5	0.015785
BLLF2	GKN2	0.043579
BLLF2	SPPL3	0.010812
BLLF2	YBX3	0.012019
BLLF2	FAXC	0.013561
BLLF2	B3GALTL	0.0162
BLLF2	SLC39A13	0.025989

BLLF2	MAGEC3	0.010763
BLLF2	YEATS4	0.01001
BLLF2	GYLTL1B	0.010692
BLLF2	MAGEL2	0.022534
BLLF2	PNMA6C	0.018024
BLLF2	UCMA	0.023483
BLLF2	GLI4	0.014137
BLLF2	FAM47C	0.090198
BLLF2	MDC1	0.013455
BLLF2	C10orf111	0.16579
BLLF2	NHP2L1	0.027679
BLLF2	QTRT1	0.13124
BLLF2	AMOTL1	0.068129
BLLF2	ZNF705B	0.01922
BLLF2	SLC35A3	0.011615
BLLF2	PPY	0.016523
BLLF2	DLGAP4	0.022239
BLLF2	ZNF382	0.024934
BLLF2	ZNF717	0.018958
BLLF2	GTPBP2	0.043283
BLLF2	PPP1R1B	0.067712
BLLF2	ZNF718	0.014091
BLLF2	OR2AG1	0.084698
BLLF2	CCHCR1	0.12249
BLLF2	ZNF99	0.013082
BLLF2	OTC	0.041529
BLLF2	SLC26A7	0.011693
BLLF2	ZAR1L	0.12086
BLLF2	PHTF2	0.011848
BLLF2	RASSF2	0.029136
BLLF2	TJAP1	0.12073
BLLF2	DARS	0.063524
BLLF2	UCN2	0.095433
BLLF2	SCHIP1	0.15127
BLLF2	OS9	0.012212
BLLF2	ZFPL1	0.041893
BLLF2	PAGE3	0.06354
BLLF2	LSM7	0.011018
BLLF2	LYPD6B	0.14446
BLLF2	GPR89B	0.041589
BLLF2	PDE12	0.020594
BLLF2	DBF4	0.017907
BLLF2	FBXO10	0.035526
BLLF2	WASH1	0.011385
BLLF2	ZSCAN5A	0.026092
BLLF2	TAF10	0.044591
BLLF2	C17orf80	0.078614
BLLF2	GDPD4	0.010641
BLLF2	AIDA	0.036313
BLLF2	MS4A8	0.02686
BLLF2	GRAMD1B	0.019183
BLLF2	SLC4A7	0.025016
BLLF2	EMP3	0.053323
BLLF2	CHST11	0.038572
BLLF2	SLC16A1	0.020565
BLLF2	CXCL14	0.012336
BLLF2	PATL1	0.054749
BLLF2	PARP10	0.062872
BLLF2	COPG2	0.018701
BLLF2	NSMCE4A	0.018971
BLLF2	ALKBH4	0.069645
BLLF2	FBXL3	0.014015
BLLF2	SLC4A2	0.030186
BLLF2	H1FX	0.018593
BLLF2	PRAMEF5	0.016732
BLLF2	PHOSPHO1	0.010975
BLLF2	NYAP1	0.082529
BLLF2	PCNXL3	0.018945

BLLF2	LHPP	0.047882
BLLF2	MS4A10	0.010933
BLLF2	GOLGA8K	0.021345
BLLF2	PAQR7	0.020651
BLLF2	ZNF562	0.010427
BLLF2	EIF4EBP3	0.026497
BLLF2	TMEM183A	0.1204
BLLF2	CPSF1	0.011646
BLLF2	EFNA3	0.010184
BLLF2	RSAD1	0.014422
BLLF2	HBG1	0.10713
BLLF2	RESP18	0.016524
BLLF2	MAGED2	0.018373
BLLF2	TLX1NB	0.025936
BLLF2	MNS1	0.1678
BLLF2	MS4A3	0.031297
BLLF2	FAM118A	0.028868
BLLF2	FLYWCH2	0.016606
BLLF2	MACC1	0.067694
BLLF2	MAGEH1	0.015446
BLLF2	MSLN	0.010495
BLLF2	RAB11FIP5	0.010509
BLLF2	SERINC5	0.061958
BLLF2	SUZ12	0.020256
BLLF2	LCA10	0.13035
BLLF2	ATP6V1C2	0.021348
BLLF2	BRINP3	0.039011
BLLF2	STATH	0.16865
BLLF2	ARSG	0.011875
BLLF2	NOL6	0.054544
BLLF2	TRAPPC5	0.11776
BLLF2	BTG3	0.031243
BLLF2	TSPAN7	0.011037
BLLF2	CLDN17	0.025631
BLLF2	FAM90A16P	0.019086
BLLF2	KLC3	0.01132
BLLF2	YIPF1	0.018744
BLLF2	PDCD6IP	0.011541
BLLF2	BRICD5	0.051044
BLLF2	ASAH2	0.017665
BLLF2	PGLYRP4	0.011671
BLLF2	RARRES2	0.03376
BLLF2	P4HA3	0.030584
BLLF2	CNGC19	0.17973
BLLF2	C9orf106	0.024161
BLLF2	ENY2	0.057765
BLLF2	LETM2	0.079468
BLLF2	ANO3	0.067274
BLLF2	MNDA	0.015742
BLLF2	RPL4	0.047496
BLLF2	ASXL1	0.028635
BLLF2	MAP1LC3A	0.010741
BLLF2	ATG4A	0.029029
BLLF2	DYDC1	0.020099
BLLF2	CCDC102B	0.014307
BLLF2	SSX7	0.021324
BLLF2	CNRIP1	0.029475
BLLF2	PRPF3	0.065727
BLLF2	ATP6V1H	0.016426
BLLF2	FGFBP2	0.071297
BLLF2	SLCO1B7	0.015671
BLLF2	KDM8	0.011875
BLLF2	ZNF33B	0.020823
BLLF2	PHYH	0.084971
BLLF2	IL17F	0.025449
BLLF2	HLF	0.022254
BLLF2	ZNF35	0.017267
BLLF2	EIF3A	0.042983

BLLF2	TMEM78	0.010792
BLLF2	MGARP	0.026159
BLLF2	RPS4X	0.010823
BLLF2	CLDN15	0.022562
BLLF2	SAP30L	0.078366
BLLF2	PTN	0.046371
BLLF2	TMED8	0.01918
BLLF2	TCERG1L	0.044806
BLLF2	ASIC5	0.01013
BLLF2	IL10RA	0.010625
BLLF2	FAM203A	0.07348
BLLF2	C6orf223	0.17039
BLLF2	C1orf64	0.11791
BLLF2	ELMOD1	0.049054
BLLF2	LDHAL6B	0.035408
BLLF2	ENTPD6	0.012917
BLLF2	MBOAT7	0.064864
BLLF2	REXO2	0.033281
BLLF2	LOX	0.03028
BLLF2	ELP4	0.083298
BLLF2	RCL1	0.017518
BLLF2	VANGL1	0.014929
BLLF2	FRY	0.019377
BLLF2	ATP6V1A	0.013451
BLLF2	NAA35	0.097715
BLLF2	COMMD4	0.058186
BLLF2	PACRGL	0.036673
BLLF2	ENO1	0.015531
BLLF2	NIPA2	0.1085
BLLF2	TMBIM6	0.016703
BLLF2	ZNF780B	0.012141
BLLF2	ORC5	0.02857
BLLF2	OCIAD2	0.10968
BLLF2	PPP1R2P3	0.13785
BLLF2	TCTEX1D1	0.032074
BLLF2	CLU	0.032862
BLLF2	C1orf140	0.11234
BLLF2	FAM69A	0.080484
BLLF2	ATXN7	0.016058
BLLF2	PCCB	0.013966
BLLF2	TAS2R20	0.066642
BLLF2	TDP2	0.011866
BLLF2	YGL006W-A	0.013994
BLLF2	CHCHD3	0.039879
BLLF2	NDUFA12	0.013744
BLLF2	GOLGA6L20	0.053115
BLLF2	SLAMF8	0.011403
BLLF2	LINC00083	0.012708
BLLF2	ZFP90	0.011372
BLLF2	LINC00588	0.11251
BLLF2	RIPPLY1	0.092909
BLLF2	HYPK	0.029223
BLLF2	PSMD11	0.010475
BLLF2	RUNDC1	0.022514
BLLF2	ETNK2	0.06001
BLLF2	WIPF2	0.0662
BLLF2	OR2T27	0.079663
BLLF2	ZNF575	0.020322
BLLF2	NIPSNAP3B	0.018418
BLLF2	DYNLL1	0.011611
BLLF2	HNRNPLL	0.018757
BLLF2	ZSWIM8	0.10471
BLLF2	SLC39A10	0.012764
BLLF2	PGBD5	0.016482
BLLF2	SLC39A8	0.013295
BLLF2	EID1	0.080766
BLLF2	OLA1	0.05537
BLLF2	CEP68	0.020536

BLLF2	ATP6V1D	0.014838
BLLF2	STX6	0.066604
BLLF2	YIF1B	0.082942
BLLF2	SCGB3A2	0.036373
BLLF2	SZRD1	0.039606
BLLF2	IMPG1	0.011233
BLLF2	ZNF689	0.017991
BLLF2	SNW1	0.043977
BLLF2	AC3.5	0.10705
BLLF2	DDX26B	0.12689
BLLF2	ECD	0.038157
BLLF2	FAM20A	0.02361
BLLF2	SGIP1	0.026054
BLLF2	TTR	0.035344
BLLF2	LCHN	0.03047
BLLF2	TGM6	0.016032
BLLF2	CLDN9	0.035403
BLLF2	PPP6R2	0.049278
BLLF2	ITPKB	0.06385
BLLF2	APOBEC2	0.025565
BLLF2	IGKV1-5	0.013084
BLLF2	MIS12	0.014233
BLLF2	FAM45B	0.10423
BLLF2	FAM229B	0.076564
BLLF2	LINC00478	0.028519
BLLF2	ZNF586	0.015884
BLLF2	NGRN	0.026395
BLLF2	RNASE1	0.022796
BLLF2	C2orf81	0.10935
BLLF2	SF1	0.011842
BLLF2	MARCH10	0.01342
BLLF2	KCNJ16	0.018168
BLLF2	PNOC	0.071178
BLLF2	ECM1	0.026943
BLLF2	ZNF816	0.013017
BLLF2	FAM84A	0.046935
BLLF2	TREX2	0.062878
BLLF2	MRPL23	0.02025
BLLF2	ZNF521	0.016897
BLLF2	BAG4	0.056451
BLLF2	DNASE1L3	0.057259
BLLF2	FAM20C	0.024346
BLLF2	CABIN1	0.016269
BLLF2	GPN3	0.016734
BLLF2	INMT	0.015317
BLLF2	SPANXA2-OT1	0.054398
BLLF2	FASTKD2	0.035969
BLLF2	ASIP	0.15947
BLLF2	PUS3	0.14389
BLLF2	CAD	0.013673
BLLF2	IDH3A	0.018324
BLLF2	GRP	0.014462
BLLF2	FAM187A	0.010428
BLLF2	KANSL1L	0.053987
BLLF2	EIF4G1	0.017927
BLLF2	ZNF677	0.019882
BLLF2	CGREF1	0.027695
BLLF2	CACNA2D4	0.068599
BLLF2	SMPD1	0.021465
BLLF2	AKAP8	0.068673
BLLF2	ALPK1	0.032346
BLLF2	ZNF763	0.030302
BLLF2	FAM49B	0.046597
BLLF2	ZNF767	0.063666
BLLF2	GABARAPL2	0.011107
BLLF2	FAM199X	0.041157
BLLF2	RARS	0.023398
BLLF2	ZNF155	0.023662

BLLF2	SLC39A6	0.023428
BLLF2	PRAMEF17	0.022587
BLLF2	NOL12	0.032747
BLLF2	TBCA	0.11419
BLLF2	SARNP	0.018671
BLLF2	DEPDC7	0.013147
BLLF2	VN1R4	0.017496
BLLF2	SPATA31A7	0.055724
BLLF2	HDGFL1	0.012065
BLLF2	SMPX	0.084428
BLLF2	SWT1	0.045328
BLLF2	PAGE4	0.1414
BLLF2	TKTL1	0.029608
BLLF2	FAM90A24P	0.013324
BLLF2	OLAH	0.011086
BLLF2	DDX18	0.011013
BLLF2	TAF1B	0.027175
BLLF2	GAGE12G	0.0533
BLLF2	VIMP	0.053773
BLLF2	FAM117A	0.095736
BLLF2	LRMP	0.060281
BLLF2	LGALS3	0.017391
BLLF2	ZNF32	0.021782
BLLF2	EIF2S3L	0.029998
BLLF2	ADCK3	0.013058
BLLF2	TIPRL	0.094943
BLLF2	FXYD6	0.053669
BLLF2	ZNF251	0.011607
BLLF2	MILR1	0.026602
BLLF2	SPA17	0.01381
BLLF2	MT1E	0.063593
BLLF2	MRPL44	0.025426
BLLF2	EIF1AD	0.014504
BLLF2	BPIFB2	0.043667
BLLF2	TMEM50A	0.037421
BLLF2	CCDC13	0.010862
BLLF2	IFNE	0.011237
BLLF2	FGGY	0.047245
BLLF2	LVP1a	0.010887
BLLF2	SEC22C	0.068644
BLLF2	FAM63B	0.12675
BLLF2	ZNF19	0.015265
BLLF2	SLC1A6	0.026311
BLLF2	COPG1	0.02829
BLLF2	OR12D3	0.077525
BLLF2	ISOC1	0.034695
BLLF2	NBPF16	0.14725
BLLF2	WFDC2	0.025208
BLLF2	CADPS	0.044817
BLLF2	MARCKSL1	0.14465
BLLF2	GBA	0.022174
BLLF2	RSL1D1	0.017673
BLLF2	PRAMEF14	0.11968
BLLF2	CSTB	0.010737
BLLF2	MPV17L	0.036137
BLLF2	NCAPH	0.085686
BLLF2	TRMT10B	0.052582
BLLF2	NIPSNAP1	0.077728
BLLF2	ATG9B	0.10396
BLLF2	NKAIN4	0.061247
BLLF2	C17orf59	0.18692
BLLF2	G4	0.046843
BLLF2	DPPA2	0.093834
BLLF2	IPO9	0.016032
BLLF2	DRC1	0.023541
BLLF2	MTBP	0.03454
BLLF2	ZNF655	0.015928
BLLF2	HIST1H2BD	0.015213

BLLF2	CRYGD	0.047495
BLLF2	NCKAP5	0.061961
BLLF2	ADRM1	0.05067
BLLF2	FAM161B	0.16225
BLLF2	CCAR1	0.13065
BLLF2	ENPP4	0.012379
BLLF2	PFDN4	0.044657
BLLF2	C19orf57	0.082577
BLLF2	EEF2	0.017022
BLLF2	C16orf93	0.061684
BLLF2	ZNF362	0.01242
BLLF2	MED24	0.07288
BLLF2	MT1B	0.065486
BLLF2	FABP5P3	0.012053
BLLF2	FAM3B	0.030634
BLLF2	ZBTB11	0.016097
BLLF2	PRSS23	0.014231
BLLF2	C20orf62	0.028968
BLLF2	C6orf222	0.040109
BLLF2	CCDC171	0.046906
BLLF2	Gm5153	0.02748
BLLF2	CCAR2	0.10499
BLLF2	PLAUR	0.075699
BLLF2	GOSR1	0.012465
BLLF2	FAM178B	0.062571
BLLF2	ZNF250	0.018124
BLLF2	GPHN	0.022467
BLLF2	SCAND1	0.012948
BLLF2	IFI27L2	0.066335
BLLF2	ZNF217	0.018012
BLLF2	OR2L2	0.084698
BLLF2	ZNF213	0.028797
BLLF2	PUS1	0.10147
BLLF2	ENO4	0.08991
BLLF2	COLGALT1	0.040538
BLLF2	GTF2A1	0.058352
BLLF2	PRAF2	0.090505
BLLF2	ZSCAN16	0.01594
BLLF2	COMMD6	0.11184
BLLF2	TAS2R14	0.052783
BLLF2	ZSCAN4	0.015314
BLLF2	SUCLG2	0.034629
BLLF2	GABARAP	0.014859
BLLF2	C1orf195	0.23211
BLLF2	VANGL2	0.016476
BLLF2	CD37	0.013231
BLLF2	FAM90A15P	0.012963
BLLF2	SNX25	0.049714
BLLF2	WFDC5	0.049136
BLLF2	HEPH	0.026573
BLLF2	BLOC1S2	0.021481
BLLF2	MYOZ1	0.084876
BLLF2	TMEM30A	0.022102
BLLF2	CCDC11	0.11706
BLLF2	kup	0.010598
BLLF2	PPP1R1C	0.018075
BLLF2	MUM1	0.04644
BLLF2	TIMELESS	0.01557
BLLF2	IL21	0.029171
BLLF2	IRF2BP2	0.021707
BLLF2	SLC5A11	0.020645
BLLF2	TRMT5	0.013457
BLLF2	NPIPA5	0.019854
BLLF2	ZNF14	0.01787
BLLF2	RASIP1	0.011062
BLLF2	RBBP9	0.1496
BLLF2	IFNA7	0.015487
BLLF2	CNTLN	0.040428

BLLF2	SAGE1	0.022778
BLLF2	MZF1	0.017247
BLLF2	SLC34A3	0.08752
BLLF2	MIS18A	0.032607
BLLF2	TRIP4	0.054844
BLLF2	MOSPD3	0.012295
BLLF2	LEO1	0.023923
BLLF2	CSRP2BP	0.026669
BLLF2	PRR23A	0.041493
BLLF2	PPCDC	0.019537
BLLF2	CCDC22	0.097872
BLLF2	CCDC6	0.010674
BLLF2	MED7	0.047462
BLLF2	TPGS2	0.013018
BLLF2	OR10G3	0.091156
BLLF2	ZNF614	0.016752
BLLF2	TTF1	0.010745
BLLF2	HS1BP3	0.010564
BLLF2	C2orf72	0.11031
BLLF2	OR5M3	0.082214
BLLF2	YBR056C-B	0.065621
BLLF2	Enho	0.01255
BLLF2	PFKFB1	0.025179
BLLF2	EME1	0.072211
BLLF2	CCPG1	0.055246
BLLF2	SCT	0.011315
BLLF2	PPP1R3B	0.011334
BLLF2	TMSB4Y	0.10862
BLLF2	PATL2	0.030815
BLLF2	SENP6	0.012628
BLLF2	SLC3A1	0.010676
BLLF2	SF3A3	0.037243
BLLF2	IFT20	0.012502
BLLF2	ZNF565	0.019072
BLLF2	MAGEA6	0.013174
BLLF2	PFKL	0.014886
BLLF2	C6orf10	0.15334
BLLF2	TRO	0.025747
BLLF2	L3MBTL2	0.010568
BLLF2	CGB	0.022066
BLLF2	TAS2R3	0.064691
BLLF2	SLC5A6	0.012357
BLLF2	ZNF652	0.023622
BLLF2	OR4F6	0.070942
BLLF2	MED27	0.010657
BLLF2	C1orf87	0.034454
BLLF2	CEP57	0.040199
BLLF2	SCIMP	0.16804
BLLF2	TIPIN	0.021233
BLLF2	ZNF879	0.020718
BLLF2	ARMC12	0.045135
BLLF2	NDN	0.024375
BLLF2	ZFP28	0.012549
BLLF2	CACNG5	0.054786
BLLF2	CEP44	0.045476
BLLF2	N4BP2	0.011739
BLLF2	ZNF44	0.011301
BLLF2	SDC1	0.019475
BLLF2	HEPHL1	0.023766
BLLF2	ZNF26	0.018272
BLLF2	TRAM1	0.013207
BLLF2	PGAP2	0.016389
BLLF2	NSUN3	0.036621
BLLF2	DLGAP3	0.029669
BLLF2	DERL1	0.04859
BLLF2	CDCA4	0.023863
BLLF2	FAM96B	0.038195
BLLF2	TRMT112	0.045065

BLLF2	ALG13	0.01657
BLLF2	ZNF611	0.012141
BLLF2	ZNF112	0.016865
BLLF2	NGDN	0.037069
BLLF2	PRKRIP1	0.10825
BLLF2	LUZP2	0.086875
BLLF2	ZNF302	0.010489
BLLF2	PAIP2	0.075414
BLLF2	ZNF135	0.011262
BLLF2	ZSWIM1	0.039875
BLLF2	ZNF28	0.01787
BLLF2	ZNF768	0.021698
BLLF2	MFNG	0.024821
BLLF2	YIPF2	0.090658
BLLF2	EEF1DP3	0.068319
BLLF2	ZNF345	0.02026
BLLF2	SLC12A2	0.027892
BLLF2	SPATA31A1	0.071981
BLLF2	VPS13A	0.04408
BLLF2	C3orf22	0.077922
BLLF2	HPS5	0.047337
BLLF2	EXOC3L1	0.041371
BLLF2	OXR1	0.021152
BLLF2	EXOC3L4	0.057428
BLLF2	TFB2M	0.14811
BLLF2	TBKBP1	0.044041
BLLF2	AK3	0.013917
BLLF2	DKK4	0.037676
BLLF2	CYCB3-1	0.14031
BLLF2	ILF2	0.011021
BLLF2	POT1	0.015216
BLLF2	RRP1	0.072582
BLLF2	RIC8A	0.045487
BLLF2	PPFIA3	0.01419
BLLF2	ZNF833P	0.01686
BLLF2	ZNF334	0.024343
BLLF2	REPIN1	0.016807
BLLF2	GOLGA2	0.018605
BLLF2	B3GNT1	0.087632
BLLF2	PHACTR2	0.034672
BLLF2	SNG1	0.074718
BLLF2	STARD4	0.016579
BLLF2	SPACA5	0.060149
BLLF2	MAGEA5	0.033179
BLLF2	COA7	0.0232
BLLF2	ADSL	0.025741
BLLF2	ZNF432	0.021618
BLLF2	SYCP1	0.020404
BLLF2	PRAP1	0.063045
BLLF2	GYG2	0.042797
BLLF2	PILRA	0.038185
BLLF2	MTRNR2L5	0.10682
BLLF2	EAF1	0.1751
BLLF2	BEND3	0.013731
BLLF2	SCEL	0.025318
BLLF2	SLC4A4	0.033657
BLLF2	ZNF449	0.022949
BLLF2	C22orf26	0.050187
BLLF2	LETM1	0.087111
BLLF2	TSPAN18	0.015222
BLLF2	TNFAIP8L1	0.1206
BLLF2	CRH	0.013564
BLLF2	GGT6	0.062935
BLLF2	RNASE12	0.023404
BLLF2	pkmyt1	0.073881
BLLF2	KCNJ18	0.015531
BLLF2	ARTN	0.037609
BLLF2	IGKV4-1	0.01102

BLLF2	PGBD4	0.036268
BLLF2	SPHK1	0.012803
BLLF2	CRYGN	0.047526
BLLF2	TMEM98	0.12486
BLLF2	KCNE3	0.057315
BLLF2	MRPS7	0.073582
BLLF2	G4	0.068181
BLLF2	MAPRE1	0.012132
BLLF2	ZNF282	0.026912
BLLF2	GZF1	0.01488
BLLF2	FNIP1	0.029724
BLLF2	PMS2CL	0.034876
BLLF2	ARSK	0.010127
BLLF2	LVP1a	0.11612
BLLF2	IFNA1	0.058282
BLLF2	RFC1	0.039639
BLLF2	CMTM8	0.032994
BLLF2	ZNF773	0.012733
BLLF2	G4	0.062196
BLLF2	CTU1	0.077359
BLLF2	MTHFR	0.043092
BLLF2	BCAS3	0.014532
BLLF2	MAD2L1BP	0.069776
BLLF2	TIMM23B	0.030069
BLLF2	PFKFB3	0.020316
BLLF2	KIAA1586	0.018983
BLLF2	FBF1	0.014566
BLLF2	BANF2	0.035021
BLLF2	ZNF491	0.017246
BLLF2	GTF2F1	0.051555
BLLF2	PLA2G15	0.018834
BLLF2	RANBP1	0.01364
BLLF2	EXOG	0.016615
BLLF2	FAM231A	0.39134
BLLF2	MS4A6A	0.034748
BLLF2	NCOA5	0.028215
BLLF2	SPCS3	0.058697
BLLF2	DCUN1D5	0.035513
BLLF2	ALG1	0.12176
BLLF2	C6orf229	0.07732
BLLF2	FAM124A	0.21282
BLLF2	TMEM181	0.082722
BLLF2	SLC1A3	0.026269
BLLF2	HIST2H2BC	0.023068
BLLF2	TCEB3CL2	0.054818
BLLF2	MATR3	0.012563
BLLF2	GPBP1L1	0.054081
BLLF2	CEP164	0.016717
BLLF2	MLLT1	0.017142
BLLF2	SAMD4A	0.034243
BLLF2	TCTE3	0.012315
BLLF2	PATZ1	0.021384
BLLF2	JMY	0.033906
BLLF2	TUSC3	0.041463
BLLF2	HNRNPH3	0.011273
BLLF2	CHST6	0.034294
BLLF2	TMEM71	0.034662
BLLF2	ANAPC4	0.021877
BLLF2	ST6GALNAC5	0.011487
BLLF2	AMER1	0.11706
BLLF2	DNAJC22	0.01358
BLLF2	TRIP11	0.038628
BLLF2	RTF1	0.022145
BLLF2	RWDD3	0.05892
BLLF2	ZNF542	0.015352
BLLF2	NHS	0.029468
BLLF2	LUC7L3	0.013249
BLLF2	SEP15	0.036367

BLLF2	PNRC1	0.14917
BLLF2	ITIH1	0.016652
BLLF2	ZNF876P	0.011129
BLLF2	Gm5153	0.02822
BLLF2	CREG2	0.035244
BLLF2	NPTX1	0.021365
BLLF2	ZSCAN5D	0.016967
BLLF2	CLDN23	0.02374
BLLF2	MAL	0.037281
BLLF2	OOEP	0.12652
BLLF2	LGALS9C	0.021753
BLLF2	FAM188A	0.013181
BLLF2	SPIRE1	0.033919
BLLF2	SMEK1	0.016048
BLLF2	ZP3	0.011984
BLLF2	DUOXA2	0.10973
BLLF2	IPO8	0.01907
BLLF2	MAGEB4	0.020186
BLLF2	USP6NL	0.010242
BLLF2	RASSF10	0.058278
BLLF2	GG1	0.069311
BLLF2	DEFB116	0.034076
BLLF2	CCL28	0.12792
BLLF2	TANGO6	0.019179
BLLF2	ZNF749	0.026467
BLLF2	ZNF8	0.018265
BLLF2	ZNF385D	0.02414
BLLF2	PANK1	0.091235
BLLF2	XPNPEP1	0.026122
BLLF2	EVC2	0.024326
BLLF2	PLSCR1	0.076086
BLLF2	C11orf45	0.020806
BLLF2	FRRS1	0.080422
BLLF2	METTL25	0.057985
BLLF2	CLDN25	0.028093
BLLF2	GAGE5	0.029493
BLLF2	HIST1H2BN	0.015213
BLLF2	R3HDM1	0.016313
BLLF2	OR2M4	0.07986
BLLF2	FXYD2	0.039
BLLF2	FAM63A	0.097284
BLLF2	CHMP2B	0.039756
BLLF2	ORAOV1	0.023804
BLLF2	GNPNAT1	0.11923
BLLF2	SYNE4	0.064545
BLLF2	BCLAF1	0.05209
BLLF2	EFTUD2	0.016788
BLLF2	BLZF1	0.029213
BLLF2	C9orf89	0.10433
BLLF2	BRIX1	0.018967
BLLF2	ZNF585A	0.0149
BLLF2	ZNF497	0.026069
BLLF2	TEKT2	0.010538
BLLF2	NSG2	0.025284
BLLF2	NBPF10	0.042796
BLLF2	SCAMP2	0.034587
BLLF2	NDST1	0.016796
BLLF2	GAGE12B	0.0533
BLLF2	MRGPRF	0.010481
BLLF2	ZNF841	0.017414
BLLF2	SLC12A3	0.026443
BLLF2	ZNF83	0.021403
BLLF2	ZNF584	0.012734
BLLF2	PSENEN	0.050628
BLLF2	ATN1	0.055013
BLLF2	NHEJ1	0.018932
BLLF2	COPRS	0.054429
BLLF2	KDM3A	0.013323

BLLF2	ATG5	0.043723
BLLF2	MRPL42	0.066203
BLLF2	IDI1	0.02142
BLLF2	MED13	0.047027
BLLF2	ADM2	0.023381
BLLF2	NPPA	0.013447
BLLF2	CAMLG	0.071771
BLLF2	NXPE4	0.024982
BLLF2	NKAPL	0.198
BLLF2	INPP1	0.014562
BLLF2	FBXO5	0.010782
BLLF2	IDH1	0.016161
BLLF2	NCKAP5L	0.11907
BLLF2	TPI1	0.01714
BLLF2	DCST2	0.032695
BLLF2	TTLL1	0.010407
BLLF2	SPINK6	0.013369
BLLF2	HCST	0.1856
BLLF2	SCGB3A1	0.1558
BLLF2	SLURP1	0.080082
BLLF2	HIST1H1B	0.017838
BLLF2	ZNF73	0.020347
BLLF2	COX7A1	0.052748
BLLF2	CTCF	0.019848
BLLF2	CCDC153	0.015917
BLLF2	LEPREL4	0.037857
BLLF2	PRR21	0.061589
BLLF2	MED12	0.040899
BLLF2	NAP1L5	0.060101
BLLF2	CCDC115	0.02054
BLLF2	KCNMB2	0.025732
BLLF2	CCDC33	0.033176
BLLF2	MORC4	0.012959
BLLF2	TLDC2	0.1011
BLLF2	ZNF674	0.016095
BLLF2	G4	0.056009
BLLF2	SHROOM1	0.039148
BLLF2	NUBPL	0.011011
BLLF2	TPD52L2	0.069245
BLLF2	C6orf165	0.013695
BLLF2	ASPSCR1	0.072578
BLLF2	EXT2	0.010689
BLLF2	ARL6IP4	0.057873
BLLF2	MSANTD1	0.026839
BLLF2	DPP6	0.012016
BLLF2	CLDN1	0.022497
BLLF2	IFIT2	0.063066
BLLF2	HEATR1	0.011269
BLLF2	SCNN1B	0.020861
BLLF2	TSTD1	0.02067
BLLF2	RMDN1	0.023046
BLLF2	GPR123	0.012508
BLLF2	SMEK3P	0.053715
BLLF2	MIEF2	0.15491
BLLF2	CEP290	0.018815
BLLF2	Enho	0.01255
BLLF2	ZKSCAN4	0.014835
BLLF2	ZNF426	0.013265
BLLF2	NIPAL3	0.1085
BLLF2	RFNG	0.014366
BLLF2	ZNF805	0.020718
BLLF2	TMEM191B	0.056927
BLLF2	PGAM4	0.052841
BLLF2	WRNIP1	0.043084
BLLF2	VGLL1	0.033342
BLLF2	RIMKLB	0.021547
BLLF2	DCT	0.027362
BLLF2	N4BP3	0.054662

BLLF2	CSH1	0.010926
BLLF2	METTL20	0.046327
BLLF2	NOMO1	0.025349
BLLF2	LY86	0.052723
BLLF2	ZNF397	0.011186
BLLF2	TMEM123	0.011138
BLLF2	PRPF8	0.022581
BLLF2	PRAMEF12	0.015057
BLLF2	NOA1	0.011224
BLLF2	OR4F17	0.077525
BLLF2	ZNF582	0.016931
BLLF2	SRXN1	0.018389
BLLF2	C11orf52	0.029453
BLLF2	TBC1D20	0.047274
BLLF2	NPM1	0.013304
BLLF2	NAP1L4	0.011867
BLLF2	NSUN5P1	0.065426
BLLF2	PAQR3	0.020651
BLLF2	SEC16B	0.05482
BLLF2	ZSCAN25	0.016375
BLLF2	SHPK	0.080577
BLLF2	TRMT10A	0.045657
BLLF2	FAM24A	0.1076
BLLF2	OSGEPL1	0.076018
BLLF2	TCAP	0.08091
BLLF2	AGBL1	0.047483
BLLF2	APOBR	0.10088
BLLF2	C12orf71	0.019913
BLLF2	SRRM5	0.033521
BLLF2	CCDC81	0.024896
BLLF2	NMNAT1	0.011194
BLLF2	GPKOW	0.019689
BLLF2	CRBN	0.021547
BLLF2	BUD31	0.014175
BLLF2	DAG1	0.064322
BLLF2	TOMM20	0.014382
BLLF2	MMEL1	0.012533
BLLF2	CACUL1	0.013877
BLLF2	ZNF705G	0.014319
BLLF2	AGTRAP	0.09505
BLLF2	C1orf192	0.062878
BLLF2	MBOAT2	0.034476
BLLF2	TIGD7	0.012435
BLLF2	SMS	0.019025
BLLF2	OTUD5	0.029791
BLLF2	ZNF133	0.024323
BLLF2	MED30	0.01178
BLLF2	HRASLS5	0.01109
BLLF2	CSRNP3	0.084901
BLLF2	HAUS5	0.044414
BLLF2	POM121L1P	0.066477
BLLF2	SUMF2	0.11896
BLLF2	SRM	0.020507
BLLF2	MIDN	0.033904
BLLF2	FBXO6	0.026237
BLLF2	RHCE	0.038611
BLLF2	VPS35	0.025645
BLLF2	DMRT3	0.015958
BLLF2	HRK	0.14241
BLLF2	ASXL2	0.052095
BLLF2	CHCHD4	0.043584
BLLF2	PI4K2B	0.012973
BLLF2	NIPAL2	0.1085
BLLF2	C2orf62	0.021257
BLLF2	NAT8L	0.026716
BLLF2	NME9	0.029313
BLLF2	HAS1	0.018793
BLLF2	ZSCAN20	0.020476

BLLF2	CLCNKA	0.012019
BLLF2	G4	0.010335
BLLF2	COPZ1	0.010854
BLLF2	TNMD	0.067316
BLLF2	SCAI	0.11539
BLLF2	OR2T7	0.087045
BLLF2	AFP	0.018552
BLLF2	SUCO	0.047316
BLLF2	CRYBB2	0.049193
BLLF2	DBF4B	0.017327
BLLF2	NIPBL	0.019115
BLLF2	DCLRE1A	0.018017
BLLF2	ZCCHC17	0.018644
BLLF2	MAGEB2	0.010048
BLLF2	CCDC39	0.023921
BLLF2	ARSH	0.013081
BLLF2	YAL042C-A	0.024701
BLLF2	TAS2R31	0.070501
BLLF2	HS2ST1	0.021699
BLLF2	SNRPF	0.010652
BLLF2	FAM21A	0.10662
BLLF2	BSND	0.097619
BLLF2	BMF	0.044975
BLLF2	C19orf67	0.034004
BLLF2	INTS2	0.02671
BLLF2	C5orf48	0.12646
BLLF2	GNG7	0.01521
BLLF2	ZNF318	0.032807
BLLF2	ETNK1	0.059758
BLLF2	SH3BP5L	0.068733
BLLF2	SENP7	0.01379
BLLF2	CCDC17	0.027643
BLLF2	ESRP2	0.035037
BLLF2	SCGB2B2	0.033863
BLLF2	PILRB	0.027974
BLLF2	CHMP4C	0.035056
BLLF2	FTH1	0.016624
BLLF2	RTP2	0.022588
BLLF2	AKIRIN1	0.078167
BLLF2	RCAN1	0.041139
BLLF2	STMN3	0.031104
BLLF2	PDS5A	0.020673
BLLF2	GNPTAB	0.010856
BLLF2	GOLGA8J	0.01104
BLLF2	TPRX1	0.022842
BLLF2	AKAP8L	0.095842
BLLF2	SYNGR4	0.19351
BLLF2	SNF8	0.025415
BLLF2	NOP9	0.045565
BLLF2	PROM2	0.014283
BLLF2	RASSF9	0.018796
BLLF2	GORASP2	0.015657
BLLF2	MLC1	0.044398
BLLF2	PAPOLB	0.016737
BLLF2	YPEL1	0.033934
BLLF2	IFI35	0.024361
BLLF2	ERVK6	0.01255
BLLF2	NXPE1	0.028455
BLLF2	POLR2H	0.051531
BLLF2	SSBP3	0.07606
BLLF2	RSBN1	0.17531
BLLF2	ZNF358	0.015884
BLLF2	PXMP4	0.069278
BLLF2	STAG1	0.064885
BLLF2	EFNA4	0.01432
BLLF2	CNOT2	0.030229
BLLF2	GLT1D1	0.017689
BLLF2	DMRT1	0.016701

BLLF2	PLD5	0.026588
BLLF2	ZNF784	0.012522
BLLF2	SMIM9	0.075895
BLLF2	GAS2	0.011357
BLLF2	GUK1	0.076727
BLLF2	C19orf82	0.095982
BLLF2	GALK2	0.042094
BLLF2	ZNF707	0.026233
BLLF2	PBXIP1	0.032871
BLLF2	ABHD17C	0.032982
BLLF2	OAS2	0.02444
BLLF2	SUMO4	0.01049
BLLF2	TRMT61B	0.084962
BLLF2	GALNT2	0.031924
BLLF2	USB1	0.060027
BLLF2	CKMT1A	0.03929
BLLF2	OR2H2	0.078589
BLLF2	APH1B	0.045367
BLLF2	SNX6	0.011728
BLLF2	ZNF143	0.01934
BLLF2	MYPOP	0.048396
BLLF2	RFX1	0.011414
BLLF2	ZNF77	0.02203
BLLF2	ATIC	0.11186
BLLF2	CDC73	0.032176
BLLF2	GSE1	0.012679
BLLF2	IDH2	0.011011
BLLF2	ATP6V1G3	0.064113
BLLF2	SECISBP2L	0.12423
BLLF2	NMI	0.02554
BLLF2	CDK5RAP3	0.042839
BLLF2	C15orf62	0.094638
BLLF2	CHMP4A	0.067829
BLLF2	C11orf96	0.073167
BLLF2	CYGB	0.078562
BLLF2	ERRFI1	0.014944
BLLF2	TBCCD1	0.050209
BLLF2	HECA	0.13386
BLLF2	GON4L	0.041013
BLLF2	ZNF547	0.028148
BLLF2	BBS2	0.020379
BLLF2	CDKN2AIPNL	0.1192
BLLF2	RMI2	0.052373
BLLF2	CHMP5	0.05071
BLLF2	GINS4	0.065567
BLLF2	PUS10	0.033006
BLLF2	PHB2	0.015592
BLLF2	INTS4L1	0.010825
BLLF2	DCP1B	0.036983
BLLF2	ZFP62	0.013362
BLLF2	PGAM2	0.052841
BLLF2	FAM92A1	0.026673
BLLF2	ATP6V1G1	0.046718
BLLF2	AKAP7	0.018486
BLLF2	GOLGA6B	0.061779
BLLF2	NACA	0.037202
BLLF2	EGLN3	0.022664
BLLF2	EFNA2	0.014931
BLLF2	ZNF865	0.014175
BLLF2	ARMCX6	0.074472
BLLF2	THAP2	0.010686
BLLF2	EFNB1	0.015049
BLLF2	LYAR	0.013716
BLLF2	AASDHPPT	0.13472
BLLF2	dhrs7cb	0.010449
BLLF2	G4	0.046478
BLLF2	NBEAP1	0.011341
BLLF2	MED17	0.011916

BLLF2	AMER3	0.11379
BLLF2	RSRC1	0.15133
BLLF2	CNNM4	0.043878
BLLF2	MBOAT4	0.080434
BLLF2	DPPA5	0.019372
BLLF2	CHST8	0.023227
BLLF2	LY6G6C	0.087515
BLLF2	HBS1L	0.012846
BLLF2	TSPY10	0.018872
BLLF2	ZSCAN26	0.010573
BLLF2	CNPY3	0.010651
BLLF2	ZNF558	0.014183
BLLF2	EIF2S3	0.03102
BLLF2	RINL	0.049298
BLLF2	ZNF446	0.016717
BLLF2	RSRC2	0.113
BLLF2	ZNF791	0.011346
BLLF2	MRPL11	0.097982
BLLF2	OR10AC1P	0.08847
BLLF2	NUP205	0.060352
BLLF2	ZSCAN29	0.016541
BLLF2	CD19	0.01115
BLLF2	GAS1	0.045591
BLLF2	C16orf45	0.034157
BLLF2	TSPY1	0.019586
BLLF2	GOLGA6L9	0.099402
BLLF2	TGM4	0.015773
BLLF2	TMSB15A	0.1054
BLLF2	ZNF69	0.020358
BLLF2	ASCC2	0.089525
BLLF2	PCBD1	0.023381
BLLF2	DBNDD1	0.018786
BLLF2	NSUN6	0.014748
BLLF2	NCAPG2	0.048637
BLLF2	IFNK	0.058848
BLLF2	ALS2CR11	0.034895
BLLF2	ATF4	0.039175
BLLF2	ZNRD1-AS1	0.027515
BLLF2	MDH2	0.012109
BLLF2	ZSCAN30	0.018145
BLLF2	OR2T11	0.084698
BLLF2	RPUSD2	0.0487
BLLF2	ZNF566	0.012782
BLLF2	ABHD17B	0.056796
BLLF2	SRRT	0.010443
BLLF2	ZNF595	0.01369
BLLF2	UFSP1	0.020381
BLLF2	TBCD	0.031938
BLLF2	LARP1B	0.02473
BLLF2	NME8	0.017414
BLLF2	THEG	0.052449
BLLF2	JAKMIP1	0.018934
BLLF2	SLPI	0.061241
BLLF2	DAO	0.020745
BLLF2	THPO	0.015355
BLLF2	OR2D3	0.084698
BLLF2	ZNF460	0.017687
BLLF2	ESRP1	0.030188
BLLF2	FBRSL1	0.064632
BLLF2	CKMT2	0.01834
BLLF2	CRYBA2	0.046153
BLLF2	REXO1	0.010159
BLLF2	LYZ	0.060667
BLLF2	GPHB5	0.021701
BLLF2	FSCN1	0.05496
BLLF2	STC1	0.067021
BLLF2	ZNF300	0.01526
BLLF2	MOBP	0.064745

BLLF2	MS4A1	0.013187
BLLF2	ZNF737	0.014568
BLLF2	C15orf60	0.092263
BLLF2	RAMP3	0.025193
BLLF2	GLB1L3	0.017795
BLLF2	LRRC14	0.01958
BLLF2	ZNF630	0.016095
BLLF2	RPL3	0.025886
BLLF2	NGB	0.07151
BLLF2	PLAG1	0.01079
BLLF2	ZNF790	0.011898
BLLF2	NDUFA10	0.027458
BLLF2	CHMP2A	0.024531
BLLF2	SNRPN	0.011814
BLLF2	ZKSCAN7	0.014679
BLLF2	ZK1128.3	0.13003
BLLF2	TSPAN33	0.017055
BLLF2	OR2T6	0.087045
BLLF2	LIMS3L	0.017714
BLLF2	MGAT5	0.025829
BLLF2	LYPD3	0.054829
BLLF2	GALP	0.030757
BLLF2	CERCAM	0.01022
BLLF2	CUEDC1	0.046503
BLLF2	FITM2	0.075543
BLLF2	TMEM176A	0.10563
BLLF2	TOR4A	0.013146
BLLF2	HEATR5B	0.10496
BLLF2	OR2J1	0.077525
BLLF2	PDCD4	0.058195
BLLF2	CTAGE15	0.023259
BLLF2	IFITM3	0.024313
BLLF2	ZNHIT1	0.012104
BLLF2	LINC00303	0.024455
BLLF2	CARHSP1	0.039451
BLLF2	CSPP1	0.14247
BLLF2	ZNF552	0.013012
BLLF2	EEF1B2	0.013091
BLLF2	CCDC54	0.021537
BLLF2	MAN1A1	0.02002
BLLF2	ADAT1	0.035671
BLLF2	POM121B	0.082521
BLLF2	SYN2	0.03763
BLLF2	RITA1	0.070398
BLLF2	FAN1	0.07697
BLLF2	CLINT1	0.010624
BLLF2	OAZ3	0.059172
BLLF2	ZNF641	0.029378
BLLF2	C20orf195	0.044264
BLLF2	MRPL54	0.028358
BLLF2	C19orf66	0.097809
BLLF2	NXPH1	0.050559
BLLF2	SLC44A4	0.031104
BLLF2	EIF4E1B	0.020283
BLLF2	NDFIP2	0.047931
BLLF2	ARHGDIB	0.030237
BLLF2	ZNF16	0.016841
BLLF2	IMMT	0.026928
BLLF2	HBM	0.1064
BLLF2	C4orf6	0.075899
BLLF2	M6PR	0.064409
BLLF2	GAGE2E	0.029493
BLLF2	LUC7L2	0.041242
BLLF2	ZNF141	0.013742
BLLF2	MLF1	0.026113
BLLF2	GAS2L1	0.024982
BLLF2	CCDC89	0.067064
BLLF2	FASTKD1	0.018973

BLLF2	UNQ6125/PRO20090	0.13129
BLLF2	SPHK2	0.013934
BLLF2	F13A1	0.020844
BLLF2	RPS20	0.071128
BLLF2	ZNF853	0.01517
BLLF2	VIPAS39	0.058433
BLLF2	RSPH9	0.059257
BLLF2	NPY	0.033305
BLLF2	LDHA	0.035408
BLLF2	ZNF510	0.016481
BLLF2	ZNF341	0.012362
BLLF2	SPATA2	0.1009
BLLF2	PCF11	0.024595
BLLF2	SS18	0.024066
BLLF2	CT45A2	0.037307
BLLF2	ZNF546	0.011851
BLLF2	AICDA	0.019765
BLLF2	ZNF493	0.013082
BLLF2	RPS9	0.018646
BLLF2	RPLP2	0.014047
BLLF2	PLB1	0.0301
BLLF2	PAPOLA	0.040773
BLLF2	N4BP2L1	0.030324
BLLF2	CDC42EP1	0.051072
BLLF2	ZNF720	0.015937
BLLF2	SOGA1	0.062016
BLLF2	OSGEP	0.01327
BLLF2	ROPN1B	0.038203
BLLF2	ZIM2	0.013526
BLLF2	MRPL43	0.042987
BLLF2	FAM231D	0.19084
BLLF2	ZBTB17	0.012456
BLLF2	ESM1	0.018563
BLLF2	GLE1	0.035462
BLLF2	BLMH	0.033753
BLLF2	ANAPC10	0.031314
BLLF2	SAC3D1	0.074039
BLLF2	THAP7	0.023488
BLLF2	SLC1A4	0.026269
BLLF2	SRA1	0.031708
BLLF2	CHMP1A	0.018184
BLLF2	GML	0.13526
BLLF2	MAGEB17	0.01906
BLLF2	XPO1	0.017355
BLLF2	SLC26A3	0.014946
BLLF2	GGPS1	0.010738
BLLF2	TET2	0.056802
BLLF2	ADPRM	0.028815
BLLF2	ATOX1	0.030571
BLLF2	IGKV1-5	0.021749
BLLF2	TMEM99	0.081218
BLLF2	KIAA0087	0.10956
BLLF2	CD68	0.10055
BLLF2	MSANTD3	0.035409
BLLF2	OR4F6	0.092406
BLLF2	ZNF671	0.01776
BLLF2	C11orf72	0.055668
BLLF2	PPP1R3C	0.026232
BLLF2	C9orf171	0.11847
BLLF2	TSACC	0.16147
BLLF2	HIST1H2BC	0.015213
BLLF2	IQCF3	0.024243
BLLF2	LINC00615	0.075192
BLLF2	MARCH5	0.011045
BLLF2	SUPT16H	0.014608
BLLF2	STT3A	0.027999
BLLF2	ZBTB24	0.015662
BLLF2	SLC16A4	0.027453

BLLF2	IGKV4-1	0.015652
BLLF2	OR2D2	0.077525
BLLF2	ESD	0.014181
BLLF2	CCDC94	0.098334
BLLF2	SMIM20	0.015113
BLLF2	MTFP1	0.043877
BLLF2	CNIH2	0.016325
BLLF2	NAA38	0.010296
BLLF2	ZNF70	0.012754
BLLF2	BLOC1S5	0.035147
BLLF2	GNAS	0.035573
BLLF2	MYNN	0.022728
BLLF2	SPTSSB	0.055554
BLLF2	RBBP8NL	0.030643
BLLF2	RGAG4	0.06329
BLLF2	SEC61A2	0.041692
BLLF2	ZNF726	0.013082
BLLF2	NSL1	0.034684
BLLF2	FTMT	0.011024
BLLF2	FAM74A1	0.038219
BLLF2	ZNF445	0.012141
BLLF2	ZNF100	0.02076
BLLF2	ZNF225	0.012141
BLLF2	ATP6V0A1	0.012459
BLLF2	APOL3	0.06166
BLLF2	SLCO3A1	0.014827
BLLF2	ATP5C1	0.038584
BLLF2	C6orf106	0.1021
BLLF2	ORC6	0.044518
BLLF2	TPRKB	0.023251
BLLF2	PPP1R11	0.074625
BLLF2	QRICH1	0.063347
BLLF2	TCEAL3	0.033237
BLLF2	STON1	0.015397
BLLF2	LEPREL1	0.014517
BLLF2	ZNF384	0.010007
BLLF2	TOB2	0.038051
BLLF2	SGSH	0.015933
BLLF2	SCG5	0.15023
BLLF2	TMEM235	0.028199
BLLF2	PRAMEF10	0.056936
BLLF2	TTC23L	0.025237
BLLF2	HSPA9	0.029269
BLLF2	ZNF596	0.014786
BLLF2	NCSTN	0.016783
BLLF2	SLCO2B1	0.02916
BLLF2	GNRH1	0.054384
BLLF2	FAM150B	0.037718
BLLF2	MFSD7	0.060254
BLLF2	PLSCR2	0.072413
BLLF2	UPK3B	0.013624
BLLF2	AFF2	0.061509
BLLF2	TNFSF18	0.088453
BLLF2	DNAJA2	0.016386
BLLF2	MBNL1	0.010582
BLLF2	MED4	0.067524
BLLF2	ZNF844	0.029025
BLLF2	SPEF2	0.046503
BLLF2	ZNF485	0.010709
BLLF2	LUC7L	0.025816
BLLF2	TOMM40L	0.014704
BLLF2	ADSSL1	0.029118
BLLF2	GPI	0.070103
BLLF2	CLDN10	0.010665
BLLF2	TNNI3	0.020342
BLLF2	IFT43	0.021321
BLLF2	ZNF625	0.022911
BLLF2	ZNF517	0.010245

BLLF2	SPINK14	0.01325
BLLF2	DPEP2	0.016566
BLLF2	UBR1	0.041234
BLLF2	IL2RA	0.031487
BLLF2	AIM1L	0.045047
BLLF2	CDR2L	0.030214
BLLF2	REEP2	0.024085
BLLF2	DXO	0.024094
BLLF2	RINT1	0.052792
BLLF2	ZDHHC11B	0.012435
BLLF2	PSMB8	0.01384
BLLF2	SCG3	0.037066
BLLF2	SNX17	0.01396
BLLF2	DFFB	0.027812
BLLF2	UPP2	0.025824
BLLF2	EMG1	0.019244
BLLF2	SMPDL3B	0.019404
BLLF2	HPS6	0.086822
BLLF2	GR6	0.031077
BLLF2	AMD1	0.026488
BLLF2	TP53INP2	0.092703
BLLF2	ZNF165	0.014013
BLLF2	SMN1	0.032634
BLLF2	FSIP2	0.20692
BLLF2	ZKSCAN2	0.014874
BLLF2	ZNF467	0.023603
BLLF2	GTF2E2	0.16227
BLLF2	DAK	0.096329
BLLF2	GFRA4	0.042821
BLLF2	ATXN3L	0.042793
BLLF2	RPL26	0.047272
BLLF2	TRMT61A	0.046072
BLLF2	FAM198A	0.041898
BLLF2	THAP4	0.041358
BLLF2	SLC14A1	0.1353
BLLF2	MINPP1	0.051722
BLLF2	SLC8B1	0.044593
BLLF2	STS	0.014122
BLLF2	SGMS2	0.034864
BLLF2	ZNF146	0.024546
BLLF2	METAP2	0.011789
BLLF2	TPD52	0.034294
BLLF2	POLDIP3	0.02017
BLLF2	INPP4B	0.024297
BLLF2	NUFIP1	0.011259
BLLF2	H2BFM	0.021051
BLLF2	TMEM55B	0.075081
BLLF2	BTNL10	0.015727
BLLF2	SLC16A8	0.074421
BLLF2	LCN12	0.013626
BLLF2	RIPPLY3	0.067552
BLLF2	XRCC5	0.011968
BNLF2b	ubl4ab	0.12232
BNLF2b	AKAP14	0.11049
BNLF2b	MICALCL	0.022266
BNLF2b	PTTG2	0.02526
BNLF2b	CHMP6	0.012519
BNLF2b	UPF3A	0.029812
BNLF2b	RABEP1	0.011122
BNLF2b	CACNA2D1	0.012649
BNLF2b	SPATS2L	0.025252
BNLF2b	C12orf61	0.12014
BNLF2b	IP6K2	0.028304
BNLF2b	PLSCR3	0.057269
BNLF2b	CC2D1B	0.01344
BNLF2b	PRSS16	0.028981
BNLF2b	FAM150A	0.016717
BNLF2b	ZC3HAV1L	0.010341

BNLF2b	CEP85L	0.029836
BNLF2b	NAT16	0.034073
BNLF2b	ANAPC5	0.034935
BNLF2b	BLVRA	0.06236
BNLF2b	IRAK1BP1	0.074793
BNLF2b	ZPBP2	0.029634
BNLF2b	VWA3A	0.049586
BNLF2b	ZDHHC11	0.017893
BNLF2b	ARSB	0.012368
BNLF2b	YMR013W-A	0.046766
BNLF2b	MAN1B1	0.034447
BNLF2b	VWA7	0.073164
BNLF2b	CHRD	0.010656
BNLF2b	ATP5B	0.019221
BNLF2b	NIN	0.096409
BNLF2b	ILDR2	0.074998
BNLF2b	B9D1	0.031689
BNLF2b	STIM2	0.012218
BNLF2b	KIN	0.050772
BNLF2b	BNIPL	0.012742
BNLF2b	LY6G6F	0.055724
BNLF2b	MAN1A2	0.02508
BNLF2b	SLC23A2	0.036827
BNLF2b	NDUFAF4	0.022141
BNLF2b	ANGEL1	0.018763
BNLF2b	PITPNM2	0.021274
BNLF2b	C15orf40	0.011321
BNLF2b	SYPL2	0.031946
BNLF2b	WIPF1	0.046183
BNLF2b	SLC30A5	0.016275
BNLF2b	ATRIP	0.066097
BNLF2b	MCAT	0.015197
BNLF2b	TM2D3	0.099448
BNLF2b	MFAP2	0.050374
BNLF2b	ITPKC	0.050035
BNLF2b	PEA15	0.011848
BNLF2b	WDR93	0.021846
BNLF2b	LYZL2	0.078931
BNLF2b	MEI4	0.0764
BNLF2b	SLC34A2	0.048571
BNLF2b	TECPR1	0.038605
BNLF2b	GTPBP1	0.05331
BNLF2b	CHMP4B	0.0629
BNLF2b	CHMP4BP1	0.024769
BNLF2b	GFRA2	0.054299
BNLF2b	MMRN2	0.011268
BNLF2b	SATL1	0.032281
BNLF2b	PRAMEF1	0.049948
BNLF2b	VWA8	0.017059
BNLF2b	LEPROT	0.012311
BNLF2b	TBC1D3H	0.012574
BNLF2b	AMOT	0.015716
BNLF2b	SLC4A1	0.010864
BNLF2b	UGCG	0.013
BNLF2b	CABS1	0.15253
BNLF2b	NAPB	0.026148
BNLF2b	HAUS6	0.033478
BNLF2b	HELB	0.012508
BNLF2b	AKIRIN2	0.060288
BNLF2b	KCNK1	0.013951
BNLF2b	LVP1a	0.057451
BNLF2b	WFDC3	0.010145
BNLF2b	LYZL4	0.02586
BNLF2b	GADD45B	0.012854
BNLF2b	ARSD	0.011859
BNLF2b	ZC3H18	0.047706
BNLF2b	TIGAR	0.011819
BNLF2b	SF3B5	0.025196

BNLF2b	SECTM1	0.075529
BNLF2b	FAM83F	0.043555
BNLF2b	NCR3	0.036496
BNLF2b	CNP	0.010699
BNLF2b	ASTL	0.014117
BNLF2b	BEX2	0.070221
BNLF2b	NUDT15	0.011978
BNLF2b	BRK1	0.019608
BNLF2b	EPPK1	0.015771
BNLF2b	IP6K1	0.027557
BNLF2b	COMMD3	0.055138
BNLF2b	SPPL2A	0.019076
BNLF2b	CACYBP	0.010593
BNLF2b	UBLCP1	0.017965
BNLF2b	RPP14	0.030338
BNLF2b	LZTS1	0.027266
BNLF2b	AP3S1	0.012913
BNLF2b	LYZL6	0.077563
BNLF2b	MGP	0.030734
BNLF2b	DDHD1	0.023709
BNLF2b	YIPF5	0.05388
BNLF2b	NSUN4	0.055285
BNLF2b	EID3	0.017298
BNLF2b	SPACA3	0.081264
BNLF2b	DTNBP1	0.022569
BNLF2b	CXorf67	0.12983
BNLF2b	CLTB	0.030758
BNLF2b	ELOVL4	0.010836
BNLF2b	FAM188B2	0.11996
BNLF2b	SCGB1D1	0.050618
BNLF2b	TNNI1	0.017666
BNLF2b	AMY2B	0.028058
BNLF2b	GSTCD	0.013814
BNLF2b	FAM69C	0.1376
BNLF2b	TBC1D3F	0.016574
BNLF2b	METTL2B	0.013044
BNLF2b	LPCAT3	0.024407
BNLF2b	CLTA	0.064555
BNLF2b	CRP	0.025393
BNLF2b	AGR2	0.039694
BNLF2b	IRF2BP1	0.011995
BNLF2b	GOLGA8CP	0.12252
BNLF2b	DBNDD2	0.018008
BNLF2b	KCNK12	0.023559
BNLF2b	PRADC1	0.060392
BNLF2b	TMEM184C	0.035514
BNLF2b	TPD52L1	0.10656
BNLF2b	TBC1D28	0.013968
BNLF2b	POLR3F	0.013575
BNLF2b	S100A3	0.013263
BNLF2b	GIMAP2	0.010007
BNLF2b	TMEM215	0.021373
BNLF2b	CCDC176	0.15491
BNLF2b	GCH1	0.051214
BNLF2b	TRAPPC1	0.049812
BNLF2b	KRTAP13-4	0.011623
BNLF2b	CASD1	0.024956
BNLF2b	RNASE9	0.011054
BNLF2b	SNX29P2	0.010075
BNLF2b	CHIT1	0.013583
BNLF2b	B4GALT3	0.011916
BNLF2b	PLD4	0.017998
BNLF2b	ABRA	0.039099
BNLF2b	ADORA3	0.01105
BNLF2b	CCDC137	0.079415
BNLF2b	GPR179	0.09232
BNLF2b	NCAPG	0.051716
BNLF2b	BCL7C	0.065062

BNLF2b	BCDIN3D	0.052135
BNLF2b	ZNF830	0.071988
BNLF2b	NSUN7	0.056102
BNLF2b	MAGEB18	0.021061
BNLF2b	FNIP2	0.11645
BNLF2b	TREML1	0.026058
BNLF2b	RPP21	0.047819
BNLF2b	STMN2	0.025597
BNLF2b	NPIPA1	0.016886
BNLF2b	FAM47B	0.012986
BNLF2b	RDM1	0.035211
BNLF2b	MSMB	0.023297
BNLF2b	CENPH	0.018593
BNLF2b	SRL	0.051817
BNLF2b	TIMM44	0.025962
BNLF2b	AP2S1	0.015309
BNLF2b	SMIM14	0.030199
BNLF2b	TBC1D3G	0.012574
BNLF2b	GEMIN6	0.020737
BNLF2b	SLC1A5	0.019974
BNLF2b	VWA5B1	0.040369
BNLF2b	FAM69B	0.14758
BNLF2b	STXBP6	0.011997
BNLF2b	GNS	0.013167
BNLF2b	OGFR	0.033406
BNLF2b	ATP6V1E2	0.050194
BNLF2b	SLC34A1	0.060997
BNLF2b	MBD5	0.019094
BNLF2b	LTF	0.019138
BNLF2b	C17orf99	0.020225
BNLF2b	CHGA	0.1176
BNLF2b	FAM174B	0.031223
BNLF2b	IWS1	0.059313
BNLF2b	SPINK13	0.015208
BNLF2b	SLC38A9	0.022585
BNLF2b	RASSF6	0.010415
BNLF2b	NDEL1	0.028441
BNLF2b	SPINK5	0.020529
BNLF2b	ANXA2R	0.091771
BNLF2b	LINC00116	0.03586
BNLF2b	LGALS13	0.02109
BNLF2b	COMMD2	0.045507
BNLF2b	SPIN1	0.027633
BNLF2b	PRAMEF13	0.091028
BNLF2b	SRRM1	0.012478
BNLF2b	YJR030C	0.016718
BNLF2b	KIAA1841	0.06368
BNLF2b	PRODH	0.061694
BNLF2b	MFSD6	0.094148
BNLF2b	ZC4H2	0.012888
BNLF2b	SEC23IP	0.011112
BNLF2b	LRCOL1	0.1026
BNLF2b	LAMP5	0.046549
BNLF2b	VSTM5	0.01535
BNLF2b	AMY1A	0.025056
BNLF2b	TXNIP	0.066797
BNLF2b	SAT2	0.010332
BNLF2b	PITPNA	0.037867
BNLF2b	TSPAN11	0.011609
BNLF2b	P4HA2	0.038828
BNLF2b	IFT140	0.016983
BNLF2b	SLCO1A2	0.02921
BNLF2b	PDE6H	0.025104
BNLF2b	MPTX1	0.036183
BNLF2b	EXOSC3	0.017286
BNLF2b	YTHDF2	0.034021
BNLF2b	SKP1	0.01432
BNLF2b	SHCBP1	0.23956

BNLF2b	TMED2	0.010936
BNLF2b	C1orf94	0.15457
BNLF2b	UBAC2	0.010075
BNLF2b	NDUFB11	0.010889
BNLF2b	MCUR1	0.010402
BNLF2b	HGD	0.081831
BNLF2b	BSN	0.057136
BNLF2b	SLCO4A1	0.012484
BNLF2b	HEMK1	0.023662
BNLF2b	FAM210A	0.13164
BNLF2b	DDHD2	0.012781
BNLF2b	DNASE1L2	0.11147
BNLF2b	SYPL1	0.16969
BNLF2b	UBXN11	0.034929
BNLF2b	CRB3	0.032979
BNLF2b	ITIH2	0.03103
BNLF2b	KCNJ9	0.010532
BNLF2b	AOC2	0.019524
BNLF2b	C2CD4D	0.025457
BNLF2b	PGK2	0.012902
BNLF2b	SLC1A1	0.019545
BNLF2b	SLC1A2	0.019545
BNLF2b	COMMD8	0.021143
BNLF2b	TSPAN15	0.010401
BNLF2b	TPT1P8	0.016787
BNLF2b	FAM19A3	0.16762
BNLF2b	CAMSAP3	0.019821
BNLF2b	SDSL	0.057491
BNLF2b	ATG4C	0.017276
BNLF2b	PLSCR4	0.054355
BNLF2b	SETD3	0.010832
BNLF2b	KRTAP12-4	0.01004
BNLF2b	WIPF3	0.10486
BNLF2b	KCNK13	0.014569
BNLF2b	NSUN5	0.060288
BNLF2b	AMPD2	0.015748
BNLF2b	PACS2	0.018521
BNLF2b	AFM	0.038157
BNLF2b	PGK1	0.013185
BNLF2b	RBM27	0.015884
BNLF2b	CNPY4	0.13274
BNLF2b	UBXN2A	0.019262
BNLF2b	APOO	0.016156
BNLF2b	FAM19A4	0.023829
BNLF2b	ARRDC3	0.038107
BNLF2b	MBOAT1	0.012592
BNLF2b	ytzL	0.13646
BNLF2b	EMID1	0.018883
BNLF2b	WDR96	0.044312
BNLF2b	EIF6	0.012221
BNLF2b	TSEN2	0.038532
BNLF2b	NUCB1	0.038329
BNLF2b	PRIMA1	0.075067
BNLF2b	NAPG	0.013176
BNLF2b	FBXL8	0.17086
BNLF2b	TTYH1	0.17167
BNLF2b	C10orf82	0.041532
BNLF2b	CCRN4L	0.036745
BNLF2b	ATXN1	0.026053
BNLF2b	ORMDL2	0.026824
BNLF2b	RAB3IP	0.015398
BNLF2b	TET1	0.013798
BNLF2b	TGM2	0.012279
BNLF2b	BEX1	0.080909
BNLF2b	TTYH3	0.13406
BNLF2b	TECTB	0.017579
BNLF2b	ARRDC4	0.026127
BNLF2b	MTTP	0.010156

BNLF2b	GADD45G	0.02048
BNLF2b	BEND2	0.028807
BNLF2b	GALNS	0.010577
BNLF2b	AKAP6	0.010371
BNLF2b	DGCR8	0.013609
BNLF2b	FAM188B	0.14307
BNLF2b	ANTXR1	0.032322
BNLF2b	KCNJ12	0.015468
BNLF2b	NUCB2	0.013534
BNLF2b	UNQ6126/PRO20091	0.012269
BNLF2b	CDCP1	0.027446
BNLF2b	AFAP1L1	0.020869
BNLF2b	EPB42	0.014575
BNLF2b	INSL4	0.017339
BNLF2b	CT55	0.096956
BNLF2b	B3GNTL1	0.024911
BNLF2b	FLG	0.032585
BNLF2b	CHMP7	0.027507
BNLF2b	KCNJ8	0.013755
BNLF2b	FAM131A	0.017648
BNLF2b	RABEP2	0.17601
BNLF2b	SLC18A1	0.015603
BNLF2b	XPO6	0.020178
BNLF2b	ARSJ	0.01233
BNLF2b	TBC1D3C	0.012084
BNLF2b	GPATCH3	0.023281
BNLF2b	FAIM3	0.013935
BNLF2b	TMEM120B	0.013878
BNLF2b	LAMP1	0.029565
BNLF2b	CHGB	0.22367
BNLF2b	SLC1A7	0.018216
BNLF2b	NT5C3A	0.091453
BNLF2b	IBA57	0.026286
BNLF2b	ATCAY	0.015829
BNLF2b	G4	0.03728
BNLF2b	IFT52	0.063455
BNLF2b	CAGE1	0.02808
BNLF2b	VWA5A	0.067745
BNLF2b	TPPP3	0.066856
BNLF2b	NSUN5P2	0.10425
BNLF2b	TGM1	0.015275
BNLF2b	TMEM8B	0.0239
BNLF2b	GBP3	0.011968
BNLF2b	IL4R	0.017142
BNLF2b	KCNJ11	0.013183
BNLF2b	IP6K3	0.013966
BNLF2b	PEPD	0.048504
BNLF2b	NKAIN2	0.054728
BNLF2b	ENPP3	0.013754
BNLF2b	TAS2R10	0.018128
BNLF2b	EIF2A	0.012677
BNLF2b	FTSJ1	0.014427
BNLF2b	KCNJ6	0.010769
BNLF2b	PPP2R3C	0.039957
BNLF2b	ATG10	0.065605
BNLF2b	EIF4E	0.010183
BNLF2b	ATP5F1	0.031346
BNLF2b	C19orf12	0.019132
BNLF2b	C12orf63	0.011551
BNLF2b	SDS	0.023142
BNLF2b	TCEAL5	0.045759
BNLF2b	RPGRIP1	0.015125
BNLF2b	G4	0.011625
BNLF2b	COBL	0.039879
BNLF2b	DEFB129	0.095261
BNLF2b	LSM12	0.034278
BNLF2b	C12orf57	0.053004
BNLF2b	SLC18B1	0.024656

BNLF2b	TMEM8A	0.079228
BNLF2b	LGALS12	0.01119
BNLF2b	SPATA31E1	0.20888
BNLF2b	SYNE3	0.013352
BNLF2b	AMY2A	0.023146
BNLF2b	PANK3	0.041197
BNLF2b	SFI1	0.088026
BNLF2b	C1orf186	0.022059
BNLF2b	APCS	0.046406
BNLF2b	GOLGA8I	0.031534
BNLF2b	AOC3	0.014354
BNLF2b	CGGBP1	0.056987
BNLF2b	TIGD1	0.014159
BNLF2b	GTSF1	0.15881
BNLF2b	SCAPER	0.021794
BNLF2b	UPF3B	0.010589
BNLF2b	SPINK1	0.013262
BNLF2b	RPRD2	0.011064
BNLF2b	DPP7	0.032008
BNLF2b	CCDC169	0.0193
BNLF2b	OVCA2	0.039441
BNLF2b	COL26A1	0.099026
BNLF2b	LSM11	0.012596
BNLF2b	CHORDC1	0.017258
BNLF2b	ADGB	0.01985
BNLF2b	ATXN1L	0.019828
BNLF2b	C15orf37	0.012633
BNLF2b	SLC16A9	0.017867
BNLF2b	MUC1	0.089121
BNLF2b	CDK5R1	0.054159
BNLF2b	TRAPPC6A	0.047308
BNLF2b	CACNA2D3	0.058677
BNLF2b	GADD45A	0.052443
BNLF2b	LYZL1	0.076632
BNLF2b	SPINK7	0.012673
BNLF2b	CMTM6	0.055126
BNLF2b	TMEM248	0.038199
BNLF2b	GAS2L2	0.019088
BNLF2b	NPTX2	0.048461
BNLF2b	ACRV1	0.015101
BNLF2b	TMED1	0.027765
BNLF2b	TIMP1	0.02626
BNLF2b	FAM47C	0.072007
BNLF2b	C10orf111	0.066699
BNLF2b	AMOTL1	0.011501
BNLF2b	LYPD6B	0.1134
BNLF2b	FBXO10	0.014842
BNLF2b	SLC4A2	0.013706
BNLF2b	MAGED2	0.012292
BNLF2b	MAGEH1	0.016105
BNLF2b	ARSG	0.011012
BNLF2b	YIPF1	0.013511
BNLF2b	BRICD5	0.031362
BNLF2b	LETM2	0.016424
BNLF2b	CNRIP1	0.025483
BNLF2b	SLCO1B7	0.010643
BNLF2b	C1orf64	0.075942
BNLF2b	MBOAT7	0.012866
BNLF2b	NAA35	0.041803
BNLF2b	FAM69A	0.063018
BNLF2b	ETNK2	0.028886
BNLF2b	YIF1B	0.013085
BNLF2b	AC3.5	0.048938
BNLF2b	TTR	0.019055
BNLF2b	TGM6	0.010852
BNLF2b	ITPKB	0.028971
BNLF2b	APOBEC2	0.012202
BNLF2b	MIS12	0.011263

BNLF2b	C2orf81	0.042878
BNLF2b	KCNJ16	0.013755
BNLF2b	ECM1	0.013977
BNLF2b	FAM84A	0.018815
BNLF2b	CGREF1	0.019956
BNLF2b	CACNA2D4	0.067049
BNLF2b	ALPK1	0.019301
BNLF2b	FAM49B	0.052823
BNLF2b	FAM199X	0.0156
BNLF2b	HDGFL1	0.011182
BNLF2b	TIPRL	0.059248
BNLF2b	MILR1	0.027628
BNLF2b	SLC1A6	0.020115
BNLF2b	CADPS	0.06428
BNLF2b	GAS2L3	0.010511
BNLF2b	PRAMEF14	0.097065
BNLF2b	ATG9B	0.035622
BNLF2b	CCAR1	0.11603
BNLF2b	PFDN4	0.018148
BNLF2b	CCDC171	0.013984
BNLF2b	CCAR2	0.079254
BNLF2b	C1orf195	0.16725
BNLF2b	SNX25	0.030366
BNLF2b	WFDC5	0.023791
BNLF2b	IL21	0.021315
BNLF2b	NPIPA5	0.010046
BNLF2b	RASIP1	0.010827
BNLF2b	CNTLN	0.017723
BNLF2b	SLC34A3	0.029135
BNLF2b	LEO1	0.020237
BNLF2b	YBR056C-B	0.03482
BNLF2b	CCPG1	0.058955
BNLF2b	SLC3A1	0.01012
BNLF2b	SF3A3	0.011945
BNLF2b	NSUN3	0.020115
BNLF2b	LUZP2	0.041005
BNLF2b	OXR1	0.013969
BNLF2b	TFB2M	0.073709
BNLF2b	GOLGA2	0.01452
BNLF2b	SNG1	0.012879
BNLF2b	SPACA5	0.062631
BNLF2b	SYCP1	0.012037
BNLF2b	PRAP1	0.04731
BNLF2b	GYG2	0.023701
BNLF2b	PILRA	0.02752
BNLF2b	EAF1	0.093468
BNLF2b	LETM1	0.060061
BNLF2b	KCNJ18	0.01217
BNLF2b	FAM231A	0.23236
BNLF2b	FAM124A	0.049073
BNLF2b	SLC1A3	0.02004
BNLF2b	CEP164	0.015673
BNLF2b	JMY	0.024311
BNLF2b	CHST6	0.033603
BNLF2b	TRIP11	0.024741
BNLF2b	NPTX1	0.017424
BNLF2b	OOEP	0.036187
BNLF2b	FAM188A	0.016104
BNLF2b	PLSCR1	0.055396
BNLF2b	GNPNAT1	0.053046
BNLF2b	COPRS	0.040057
BNLF2b	NKAPL	0.10575
BNLF2b	SPINK6	0.013995
BNLF2b	MED12	0.040074
BNLF2b	TLDC2	0.059904
BNLF2b	TPD52L2	0.058965
BNLF2b	SCNN1B	0.012644
BNLF2b	GPR123	0.014261

BNLF2b	DCT	0.01998
BNLF2b	NSUN5P1	0.078102
BNLF2b	TCAP	0.018399
BNLF2b	DAG1	0.042715
BNLF2b	MMEL1	0.011509
BNLF2b	SUMF2	0.051572
BNLF2b	SCAI	0.024513
BNLF2b	NIPBL	0.020128
BNLF2b	ARSH	0.010576
BNLF2b	C5orf48	0.011409
BNLF2b	ETNK1	0.016804
BNLF2b	PILRB	0.031589
BNLF2b	CHMP4C	0.025258
BNLF2b	AKIRIN1	0.057008
BNLF2b	SYNGR4	0.18734
BNLF2b	IFI35	0.021608
BNLF2b	SMIM9	0.065217
BNLF2b	GAS2	0.014202
BNLF2b	SNX6	0.010248
BNLF2b	CDK5RAP3	0.01828
BNLF2b	CHMP4A	0.046646
BNLF2b	GON4L	0.023985
BNLF2b	CHMP5	0.029002
BNLF2b	FAM92A1	0.020522
BNLF2b	AASDHPPT	0.082598
BNLF2b	MBOAT4	0.011444
BNLF2b	C16orf45	0.016888
BNLF2b	ASCC2	0.037468
BNLF2b	ABHD17B	0.057992
BNLF2b	SLPI	0.034682
BNLF2b	LYZ	0.059329
BNLF2b	FSCN1	0.036669
BNLF2b	LRRC14	0.010992
BNLF2b	NGB	0.017899
BNLF2b	HEATR5B	0.084497
BNLF2b	ADAT1	0.017927
BNLF2b	OAZ3	0.039532
BNLF2b	M6PR	0.011253
BNLF2b	GAS2L1	0.032032
BNLF2b	N4BP2L1	0.033284
BNLF2b	FAM231D	0.092206
BNLF2b	ESM1	0.015625
BNLF2b	ANAPC10	0.030934
BNLF2b	SLC1A4	0.020039
BNLF2b	GML	0.039096
BNLF2b	C11orf72	0.032224
BNLF2b	IQCF3	0.011605
BNLF2b	ESD	0.012297
BNLF2b	BLOC1S5	0.024762
BNLF2b	SLCO3A1	0.01013
BNLF2b	C6orf106	0.054087
BNLF2b	SGSH	0.012368
BNLF2b	MFSD7	0.020201
BNLF2b	PLSCR2	0.057269
BNLF2b	LINC00083	0.010081
BNLF2b	SPINK14	0.012243
BNLF2b	IL2RA	0.026824
BNLF2b	ZDHHC11B	0.017893
BNLF2b	SNX17	0.01773
BNLF2b	TRMT61A	0.014745
BNLF2b	SLC8B1	0.014928
BNLF2b	STS	0.014999
BRRF1	ubl4ab	0.019727
BRRF1	PIK3CD	0.015835
BRRF1	PAGE2B	0.030417
BRRF1	SPTA1	0.081092
BRRF1	S100A7	0.12945
BRRF1	HSPA5	0.069991

BRRF1	LDLR	0.058345
BRRF1	UPF3A	0.020201
BRRF1	RABEP1	0.051229
BRRF1	C12orf61	0.018104
BRRF1	PLSCR3	0.17987
BRRF1	ZC3HAV1L	0.022275
BRRF1	RPUSD4	0.040767
BRRF1	CCDC159	0.014395
BRRF1	C10orf68	0.013901
BRRF1	MYH7	0.012914
BRRF1	SNAPIN	0.018715
BRRF1	KRI1	0.023291
BRRF1	YMR013W-A	0.02013
BRRF1	ATP5B	0.090849
BRRF1	NIN	0.15614
BRRF1	KCNIP1	0.14962
BRRF1	STIM2	0.17745
BRRF1	HUS1B	0.015335
BRRF1	FSIP1	0.021438
BRRF1	WIPF1	0.022289
BRRF1	SPTAN1	0.11053
BRRF1	TSGA10IP	0.016656
BRRF1	MASP1	0.24001
BRRF1	SCUBE3	0.22292
BRRF1	TH	0.010797
BRRF1	SPBC1539.02	0.011989
BRRF1	GLTSCR1L	0.028934
BRRF1	PROC	0.20241
BRRF1	PDCD6	0.23287
BRRF1	S100A9	0.056852
BRRF1	ANXA2	0.10836
BRRF1	MMP20	0.068548
BRRF1	MATN4	0.23015
BRRF1	NOTCH3	0.055201
BRRF1	RNH1	0.19414
BRRF1	UBAP2	0.024269
BRRF1	FAM110D	0.018858
BRRF1	CLIP1	0.088827
BRRF1	AMOT	0.038416
BRRF1	PAGE5	0.017673
BRRF1	GAGE10	0.018514
BRRF1	FAM117B	0.028815
BRRF1	EIF4G2	0.022292
BRRF1	C10orf91	0.024734
BRRF1	CCDC28A	0.027791
BRRF1	GID4	0.010206
BRRF1	FAM83F	0.018859
BRRF1	FAM101A	0.014012
BRRF1	SNURFL	0.010075
BRRF1	THBD	0.13405
BRRF1	BRAF	0.011266
BRRF1	FAM178A	0.016757
BRRF1	RNMT	0.043997
BRRF1	MAGOH	0.02891
BRRF1	EML1	0.2164
BRRF1	IQCK	0.013577
BRRF1	FAM166A	0.016981
BRRF1	MUM1L1	0.018154
BRRF1	EPS15	0.086892
BRRF1	PCSK2	0.063573
BRRF1	PRMT8	0.025342
BRRF1	AP3S1	0.08912
BRRF1	SDF4	0.16075
BRRF1	SPACA3	0.010008
BRRF1	STAT1	0.023207
BRRF1	FAT4	0.20906
BRRF1	DTNBP1	0.021511
BRRF1	PPEF1	0.1149

BRRF1	CXorf67	0.030093
BRRF1	C18orf25	0.010285
BRRF1	NELL2	0.060312
BRRF1	FAM188B2	0.030953
BRRF1	KAT2A	0.01634
BRRF1	SUV420H2	0.012901
BRRF1	MAP1LC3C	0.022638
BRRF1	NUDCD3	0.013553
BRRF1	THBS1	0.021659
BRRF1	BICD1	0.01824
BRRF1	KIAA1407	0.010094
BRRF1	PRMT1	0.038935
BRRF1	CCSER1	0.021131
BRRF1	PNMAL2	0.01518
BRRF1	AP1M1	0.039887
BRRF1	YPR022C	0.020666
BRRF1	TPPP	0.15086
BRRF1	CCDC146	0.010383
BRRF1	SYT11	0.26442
BRRF1	CCDC150	0.011384
BRRF1	C1orf105	0.021071
BRRF1	HSP90B1	0.11412
BRRF1	MACF1	0.13945
BRRF1	CCDC176	0.027199
BRRF1	GCH1	0.052826
BRRF1	C10orf67	0.018624
BRRF1	LINC00597	0.01274
BRRF1	NKD2	0.21702
BRRF1	MT1HL1	0.011331
BRRF1	ATP2B2	0.018165
BRRF1	C19orf71	0.015181
BRRF1	FNBP1L	0.030477
BRRF1	RHPN1-AS1	0.020646
BRRF1	C6orf118	0.011553
BRRF1	FAT1	0.11254
BRRF1	LINC00471	0.014155
BRRF1	S100A11	0.17314
BRRF1	CCDC137	0.029769
BRRF1	TIFAB	0.025487
BRRF1	NKD1	0.16435
BRRF1	FAM151B	0.01052
BRRF1	GCSAML	0.013656
BRRF1	EIF1AY	0.028196
BRRF1	FAM47B	0.016177
BRRF1	SUPT20HL2	0.017294
BRRF1	PPP2R3B	0.1087
BRRF1	ACTN4	0.093283
BRRF1	EHD1	0.071734
BRRF1	LINC00523	0.026052
BRRF1	S100A13	0.17119
BRRF1	MELK	0.062572
BRRF1	S100A1	0.12218
BRRF1	ARL14EPL	0.028439
BRRF1	UTRN	0.086156
BRRF1	SLC34A1	0.018243
BRRF1	CXorf21	0.010883
BRRF1	AQP1	0.012599
BRRF1	STAT6	0.022629
BRRF1	PIH1D2	0.018091
BRRF1	POM121L12	0.013979
BRRF1	CALB1	0.15773
BRRF1	MASP2	0.22351
BRRF1	PARK7	0.052792
BRRF1	LINC00472	0.016444
BRRF1	GOLGA6L4	0.020186
BRRF1	GOLGA6L6	0.019248
BRRF1	KIAA1958	0.011366
BRRF1	SLAIN1	0.029402

BRRF1	ZNF326	0.014658
BRRF1	GAS6	0.014698
BRRF1	ANXA2R	0.024473
BRRF1	YML009W-B	0.025773
BRRF1	C14orf178	0.011458
BRRF1	EIF4A3	0.011932
BRRF1	C1orf168	0.012834
BRRF1	YTHDF1	0.011705
BRRF1	SPARC	0.09468
BRRF1	MRFAP1L1	0.024021
BRRF1	EHMT2	0.011424
BRRF1	NOTCH4	0.049629
BRRF1	FAM160A1	0.025664
BRRF1	PCDH17	0.20906
BRRF1	PRDX4	0.017087
BRRF1	EIF3B	0.015798
BRRF1	YJR030C	0.018149
BRRF1	KIAA1841	0.017638
BRRF1	RNF216P1	0.012237
BRRF1	REPS1	0.19601
BRRF1	XAGE2	0.025719
BRRF1	YML009W-B	0.010936
BRRF1	LRCOL1	0.020696
BRRF1	GOLGA6L18	0.016839
BRRF1	BCORP1	0.011738
BRRF1	WIBG	0.020869
BRRF1	FAM221B	0.017978
BRRF1	NOTCH2	0.021418
BRRF1	STIM1	0.098935
BRRF1	S100B	0.097989
BRRF1	AC3.5	0.011776
BRRF1	TNNC1	0.19086
BRRF1	TMED2	0.019585
BRRF1	C14orf80	0.02387
BRRF1	CETN2	0.16059
BRRF1	ANXA6	0.19955
BRRF1	ACTN2	0.071178
BRRF1	PRSS2	0.19502
BRRF1	STAT2	0.024105
BRRF1	PITPNM3	0.18996
BRRF1	VIL1	0.09559
BRRF1	C19orf35	0.016335
BRRF1	COMMD9	0.012575
BRRF1	KCNJ4	0.059865
BRRF1	CETN1	0.20514
BRRF1	GAGE4	0.013329
BRRF1	ZK1128.3	0.010338
BRRF1	IGFBP2	0.012518
BRRF1	PNMAL1	0.01094
BRRF1	VLDLR	0.083175
BRRF1	CDH1	0.040425
BRRF1	ADAMTS13	0.016941
BRRF1	MAP1LC3B	0.053889
BRRF1	TTN	0.036628
BRRF1	ZDHHC17	0.018255
BRRF1	CXorf30	0.013137
BRRF1	EGF	0.068842
BRRF1	LTBP4	0.16387
BRRF1	PRMT2	0.023363
BRRF1	PLSCR4	0.17528
BRRF1	DNAJC9-AS1	0.018323
BRRF1	RBM8A	0.027201
BRRF1	FAM45A	0.025102
BRRF1	TCP10L2	0.012502
BRRF1	C1orf106	0.027352
BRRF1	NOP2	0.034939
BRRF1	GOLGA6L3	0.020186
BRRF1	RHOT1	0.018084

BRRF1	WWC2-AS2	0.012525
BRRF1	SNX9	0.10201
BRRF1	CCDC7	0.010882
BRRF1	YFL013W-A	0.015265
BRRF1	RYR2	0.040768
BRRF1	PRF1	0.093121
BRRF1	IL6ST	0.092933
BRRF1	MYO1E	0.053408
BRRF1	SETD2	0.028269
BRRF1	PRR19	0.012255
BRRF1	SNCA	0.011147
BRRF1	S100A8	0.080893
BRRF1	NID2	0.22399
BRRF1	GAREML	0.029691
BRRF1	MIMI	0.017065
BRRF1	NELL1	0.11892
BRRF1	ytzL	0.03486
BRRF1	WDR96	0.013348
BRRF1	DNER	0.13345
BRRF1	FAM160B1	0.015392
BRRF1	PEF1	0.22435
BRRF1	NUPR1L	0.012568
BRRF1	MEGF8	0.06159
BRRF1	C10orf82	0.014451
BRRF1	ERICH1-AS1	0.021018
BRRF1	C3orf65	0.011252
BRRF1	CHP1	0.085758
BRRF1	CFTR	0.023355
BRRF1	EP400NL	0.01478
BRRF1	FAM203B	0.015949
BRRF1	FASN	0.074292
BRRF1	HEATR5A	0.010701
BRRF1	GPR98	0.084238
BRRF1	MICU1	0.1802
BRRF1	CALU	0.17761
BRRF1	PPP3CA	0.041132
BRRF1	C16orf72	0.022688
BRRF1	BEND2	0.010273
BRRF1	LRP2	0.081277
BRRF1	CAPN2	0.035599
BRRF1	SLC23A1	0.037388
BRRF1	POM121L2	0.014252
BRRF1	GOLGA6L10	0.016839
BRRF1	FAM188B	0.031125
BRRF1	MISP	0.024412
BRRF1	FAM231B	0.013066
BRRF1	SELO	0.017173
BRRF1	NUCB2	0.14714
BRRF1	UHRF1BP1L	0.019346
BRRF1	UNQ6126/PRO20091	0.011296
BRRF1	YGL014C-A	0.020124
BRRF1	S100A6	0.14092
BRRF1	LRP8	0.15273
BRRF1	CT55	0.016826
BRRF1	S100P	0.14678
BRRF1	PRDX6	0.039853
BRRF1	CHAF1A	0.033389
BRRF1	SETD1B	0.03029
BRRF1	TP53INP1	0.01737
BRRF1	RAB11FIP3	0.069379
BRRF1	EFCAB4B	0.15913
BRRF1	ALKBH8	0.023665
BRRF1	C8orf74	0.011377
BRRF1	XAGE3	0.030417
BRRF1	C1orf111	0.015091
BRRF1	THOC1	0.01147
BRRF1	DTNA	0.11245
BRRF1	REPS2	0.23462

BRRF1	PPEF2	0.099318
BRRF1	EFEMP2	0.22171
BRRF1	ATP2A1	0.064511
BRRF1	WLS	0.044224
BRRF1	LINC00205	0.014816
BRRF1	PKD2L1	0.11613
BRRF1	FAM46B	0.012677
BRRF1	PNAS-138	0.013821
BRRF1	CCDC26	0.012792
BRRF1	EHD3	0.09032
BRRF1	SUPT20HL1	0.017294
BRRF1	VWA5B2	0.013408
BRRF1	THBS2	0.18597
BRRF1	CALM1	0.097351
BRRF1	EIF4E2	0.037929
BRRF1	FLT3	0.010676
BRRF1	S100A4	0.21869
BRRF1	S100A12	0.1144
BRRF1	FAM149B1	0.011614
BRRF1	RCN1	0.23117
BRRF1	DPP4	0.032586
BRRF1	EIF4E	0.022501
BRRF1	DNMT3A	0.016121
BRRF1	ACTN3	0.20857
BRRF1	ATP5F1	0.043108
BRRF1	FBN1	0.20972
BRRF1	F10	0.10549
BRRF1	CAPN1	0.090044
BRRF1	UCKL1	0.022849
BRRF1	FAM46A	0.013611
BRRF1	CELSR3	0.12802
BRRF1	N6AMT1	0.050111
BRRF1	SETD8	0.014782
BRRF1	SIRT4	0.090284
BRRF1	TESC	0.13056
BRRF1	MICAL1	0.028705
BRRF1	LSM12	0.013802
BRRF1	C12orf57	0.010934
BRRF1	SETD1A	0.028956
BRRF1	YAE1D1	0.017207
BRRF1	ERICH1	0.015414
BRRF1	RPH3A	0.09903
BRRF1	SRI	0.1117
BRRF1	LRP4	0.11673
BRRF1	BVES-AS1	0.011146
BRRF1	CCDC120	0.016907
BRRF1	SLIT2	0.041902
BRRF1	C1orf145	0.017247
BRRF1	MATN2	0.18334
BRRF1	CIB1	0.1593
BRRF1	PPP2R3A	0.14139
BRRF1	AKAP3	0.012365
BRRF1	DLL1	0.1666
BRRF1	ACTN1	0.10156
BRRF1	FAM195A	0.027319
BRRF1	EHD4	0.057654
BRRF1	UPF3B	0.035099
BRRF1	MYO6	0.015462
BRRF1	C5orf58	0.017935
BRRF1	PRSS3	0.22747
BRRF1	CCDC169	0.011842
BRRF1	C7orf62	0.011879
BRRF1	MYL2	0.21675
BRRF1	EDEM1	0.11569
BRRF1	LRP1	0.05337
BRRF1	ATP10B	0.01326
BRRF1	ADAR	0.019058
BRRF1	SNX19	0.14566

BRRF1	PDGFRB	0.02565
BRRF1	SPATA2L	0.013086
BRRF1	ITSN1	0.046302
BRRF1	SPIDR	0.02194
BRRF1	DST	0.10725
BRRF1	MYL6	0.20648
BRRF1	COMP	0.15842
BRRF1	CCDC47	0.18131
BRRF1	XAGE5	0.014025
BRRF1	ANXA7	0.10285
BRRF1	CHP2	0.16935
BRRF1	LYRM9	0.020273
BRRF1	FAM127B	0.012995
BRRF1	NECAB2	0.23732
BRRF1	CANX	0.09958
BRRF1	RHOT2	0.018532
BRRF1	SGD1	0.011301
BRRF1	MMP15	0.051422
BRRF1	ANXA11	0.10591
BRRF1	PPP3CB	0.045997
BRRF1	GOLGA6L19	0.016839
BRRF1	C5orf34	0.011412
BRRF1	LURAP1L	0.025103
BRRF1	C12orf50	0.021599
BRRF1	C17orf102	0.013272
BRRF1	IQGAP1	0.0324
BRRF1	MATN3	0.24052
BRRF1	FAM47C	0.017361
BRRF1	SH3KBP1	0.045325
BRRF1	RET	0.082547
BRRF1	PAGE3	0.013771
BRRF1	CDH23	0.072651
BRRF1	LCA10	0.018853
BRRF1	RAB11FIP4	0.28792
BRRF1	CNGC19	0.032075
BRRF1	SUV39H2	0.030999
BRRF1	C6orf223	0.027222
BRRF1	C1orf64	0.0199
BRRF1	C1orf140	0.01824
BRRF1	LINC00588	0.015995
BRRF1	PLCE1	0.022428
BRRF1	AC3.5	0.019116
BRRF1	DDX26B	0.023529
BRRF1	FAM45B	0.019867
BRRF1	FAM229B	0.013745
BRRF1	C2orf81	0.02094
BRRF1	CLIC4	0.078303
BRRF1	EIF4G1	0.024366
BRRF1	SLC22A5	0.012773
BRRF1	FAM49B	0.011003
BRRF1	BNIP2	0.10102
BRRF1	ZNF767	0.012363
BRRF1	JAG2	0.13695
BRRF1	FAM117A	0.015955
BRRF1	EHD2	0.032718
BRRF1	CADPS	0.23398
BRRF1	PKD2	0.010281
BRRF1	C17orf59	0.032124
BRRF1	FAM161B	0.030038
BRRF1	CCAR1	0.17724
BRRF1	C16orf93	0.012971
BRRF1	CCDC171	0.010812
BRRF1	FAM178B	0.012541
BRRF1	C1orf195	0.039545
BRRF1	PIKFYVE	0.010401
BRRF1	CRELD2	0.21739
BRRF1	C2orf72	0.017602
BRRF1	YBR056C-B	0.013615

BRRF1	NBR1	0.02589
BRRF1	GSN	0.089889
BRRF1	SLC11A2	0.027843
BRRF1	AMBRA1	0.012975
BRRF1	C3orf22	0.014721
BRRF1	EIF4H	0.013024
BRRF1	CYCB3-1	0.02126
BRRF1	EIF4A1	0.022624
BRRF1	CABP1	0.049006
BRRF1	LETM1	0.20999
BRRF1	TNFAIP8L1	0.022062
BRRF1	pkmyt1	0.013266
BRRF1	SPHK1	0.010135
BRRF1	PIK3CB	0.01326
BRRF1	FAM231A	0.064494
BRRF1	TFB1M	0.045141
BRRF1	C6orf229	0.011591
BRRF1	FAM124A	0.038204
BRRF1	FAM188A	0.17682
BRRF1	AP1B1	0.052605
BRRF1	RCN3	0.22942
BRRF1	SLC6A4	0.024764
BRRF1	NKAPL	0.02762
BRRF1	NCKAP5L	0.022096
BRRF1	PRR21	0.010123
BRRF1	TLDC2	0.017561
BRRF1	LTBP1	0.22255
BRRF1	F2	0.054289
BRRF1	SMEK3P	0.012283
BRRF1	SYT1	0.079007
BRRF1	CBL	0.010353
BRRF1	NSUN5P1	0.014398
BRRF1	PI4K2A	0.047109
BRRF1	NCS1	0.18819
BRRF1	TCAP	0.015645
BRRF1	DAG1	0.050865
BRRF1	C1orf192	0.011712
BRRF1	DOC2A	0.016791
BRRF1	C5orf48	0.019249
BRRF1	SH3BP5L	0.014314
BRRF1	NECAB3	0.14377
BRRF1	MLC1	0.028783
BRRF1	C19orf82	0.013096
BRRF1	SNX6	0.060675
BRRF1	SECISBP2L	0.020614
BRRF1	C11orf96	0.01137
BRRF1	CDKN2AIPNL	0.025048
BRRF1	NPC1L1	0.074287
BRRF1	MEGF6	0.22542
BRRF1	CALR	0.022526
BRRF1	RSRC2	0.020276
BRRF1	C16orf45	0.010348
BRRF1	GOLGA6L9	0.016839
BRRF1	KCNIP2	0.083092
BRRF1	FBRSL1	0.012173
BRRF1	ZK1128.3	0.021269
BRRF1	CUEDC1	0.010341
BRRF1	PLCG1	0.013125
BRRF1	HEATR5B	0.018024
BRRF1	CD93	0.16142
BRRF1	GRIN1	0.070992
BRRF1	CLINT1	0.034845
BRRF1	C19orf66	0.02084
BRRF1	DHH	0.15332
BRRF1	UNQ6125/PRO20090	0.018438
BRRF1	SPHK2	0.044142
BRRF1	VIPAS39	0.011511
BRRF1	AP1G2	0.014315

BRRF1	FAM231D	0.025126
BRRF1	KIAA0087	0.017123
BRRF1	C11orf72	0.010751
BRRF1	C9orf171	0.019245
BRRF1	LINC00615	0.012943
BRRF1	CCDC94	0.017629
BRRF1	MYL6B	0.17845
BRRF1	RGAG4	0.011781
BRRF1	ATP6V0A1	0.042735
BRRF1	AP1G1	0.04545
BRRF1	CDHR2	0.19139
BRRF1	SNX17	0.014806
BRRF1	CDH5	0.12515
BRRF1	IL6R	0.039886
BRRF1	SYT2	0.18817
BRRF1	CAPNS1	0.070403
BRRF1	GABARAPL1	0.011118
BRRF1	TPD52	0.22129
BTRF1	RRP7A	0.017684
BTRF1	ubl4ab	0.044097
BTRF1	PIBF1	0.015016
BTRF1	ATPIF1	0.021626
BTRF1	AKAP14	0.046831
BTRF1	PANK2	0.02293
BTRF1	MICALCL	0.017199
BTRF1	PTTG2	0.02642
BTRF1	CHMP6	0.010805
BTRF1	UPF3A	0.046096
BTRF1	RABEP1	0.036285
BTRF1	SPATS2L	0.01507
BTRF1	C12orf61	0.050854
BTRF1	IP6K2	0.014315
BTRF1	PLSCR3	0.031932
BTRF1	TCF25	0.017485
BTRF1	PRSS16	0.034831
BTRF1	FAM150A	0.014113
BTRF1	ZC3HAV1L	0.032878
BTRF1	RPUSD4	0.021041
BTRF1	CMPK2	0.022203
BTRF1	CEP85L	0.01813
BTRF1	NAT16	0.031369
BTRF1	ANAPC5	0.020569
BTRF1	WDPCP	0.014447
BTRF1	TEX12	0.010679
BTRF1	IGFL2	0.041124
BTRF1	BLVRA	0.033514
BTRF1	IRAK1BP1	0.039198
BTRF1	ZPBP2	0.019692
BTRF1	VWA3A	0.022418
BTRF1	UNC119B	0.011113
BTRF1	YMR013W-A	0.038411
BTRF1	CASC3	0.02364
BTRF1	CHIC1	0.010737
BTRF1	MAN1B1	0.019096
BTRF1	TRMT6	0.022375
BTRF1	VWA7	0.046529
BTRF1	SPATC1	0.018576
BTRF1	NIN	0.056226
BTRF1	MTO1	0.030616
BTRF1	TNRC6C	0.013765
BTRF1	ILDR2	0.035525
BTRF1	PGM3	0.02119
BTRF1	B9D1	0.016822
BTRF1	KIN	0.028441
BTRF1	PLBD2	0.015305
BTRF1	CABYR	0.026837
BTRF1	LY6G6F	0.024268
BTRF1	MAN1A2	0.013857

BTRF1	SLC23A2	0.022917
BTRF1	NDUFAF4	0.010155
BTRF1	DPY30	0.010905
BTRF1	ANGEL1	0.016714
BTRF1	TMEM30B	0.010824
BTRF1	PITPNM2	0.010379
BTRF1	SYPL2	0.016618
BTRF1	COMMD5	0.018315
BTRF1	WIPF1	0.025686
BTRF1	MT-ND4	0.012201
BTRF1	ATRIP	0.030377
BTRF1	ARMCX4	0.023159
BTRF1	POP4	0.011058
BTRF1	TM2D3	0.045772
BTRF1	MFAP2	0.034387
BTRF1	TSNAX	0.011797
BTRF1	ITPKC	0.030758
BTRF1	TRAPPC4	0.010317
BTRF1	WDR93	0.02688
BTRF1	LYZL2	0.039564
BTRF1	XPO7	0.010962
BTRF1	ATXN10	0.013219
BTRF1	MAL2	0.012363
BTRF1	MEI4	0.030476
BTRF1	SLC34A2	0.033776
BTRF1	TECPR1	0.035039
BTRF1	GTPBP1	0.053922
BTRF1	CHMP4B	0.035137
BTRF1	DKC1	0.015234
BTRF1	CHMP4BP1	0.015684
BTRF1	EXOC1	0.010991
BTRF1	PGAM1	0.017216
BTRF1	UBAP2	0.021784
BTRF1	GFRA2	0.024016
BTRF1	ANO8	0.016615
BTRF1	SATL1	0.013486
BTRF1	PRAMEF1	0.032728
BTRF1	SLC13A4	0.010708
BTRF1	YTHDF3	0.016486
BTRF1	AMOT	0.012886
BTRF1	DNAAF2	0.026505
BTRF1	PAGE5	0.02227
BTRF1	UGCG	0.01816
BTRF1	CABS1	0.075428
BTRF1	NAPB	0.013851
BTRF1	SLC16A13	0.014861
BTRF1	CASP8AP2	0.013512
BTRF1	VEZT	0.03065
BTRF1	HAUS6	0.024104
BTRF1	AKIRIN2	0.038416
BTRF1	RPRD1A	0.013271
BTRF1	LVP1a	0.046913
BTRF1	METTL1	0.01706
BTRF1	LYZL4	0.014059
BTRF1	GADD45B	0.017647
BTRF1	MESDC1	0.011827
BTRF1	NCOA7	0.011962
BTRF1	GK3P	0.011829
BTRF1	ZC3H18	0.028883
BTRF1	MRPL46	0.018887
BTRF1	P4HA1	0.014106
BTRF1	TIGAR	0.020282
BTRF1	SF3B5	0.023879
BTRF1	ICA1L	0.012896
BTRF1	NECAP1	0.024879
BTRF1	SECTM1	0.05374
BTRF1	HORMAD1	0.011525
BTRF1	PPP1R10	0.016974

BTRF1	FAM83F	0.033845
BTRF1	NCR3	0.017776
BTRF1	CKB	0.010464
BTRF1	SPATC1L	0.021552
BTRF1	UPK3BL	0.015164
BTRF1	BEX2	0.039517
BTRF1	AKAP4	0.018375
BTRF1	BRK1	0.015452
BTRF1	ACRBP	0.037217
BTRF1	IP6K1	0.019182
BTRF1	TLDC1	0.015095
BTRF1	COMMD3	0.027545
BTRF1	CDK5R2	0.011917
BTRF1	SFSWAP	0.017863
BTRF1	SPPL2A	0.014327
BTRF1	UBLCP1	0.010473
BTRF1	RPP14	0.019204
BTRF1	LZTS1	0.016509
BTRF1	AP3S1	0.010195
BTRF1	LYZL6	0.040117
BTRF1	MGP	0.015661
BTRF1	DDHD1	0.011899
BTRF1	YIPF5	0.031435
BTRF1	EBAG9	0.010523
BTRF1	NSUN4	0.041728
BTRF1	BEAN1	0.018273
BTRF1	EID3	0.030314
BTRF1	SPACA3	0.037135
BTRF1	HHAT	0.025458
BTRF1	GPATCH1	0.027122
BTRF1	DTNBP1	0.014105
BTRF1	CXorf67	0.061732
BTRF1	CLTB	0.011957
BTRF1	SEC11A	0.02211
BTRF1	ELOVL4	0.023581
BTRF1	FAM188B2	0.064359
BTRF1	GTF3C1	0.010414
BTRF1	EIF1AX	0.022185
BTRF1	DPP3	0.018162
BTRF1	POLR2D	0.01157
BTRF1	SCGB1D1	0.025397
BTRF1	TNNI1	0.012581
BTRF1	NUDCD3	0.013084
BTRF1	AMY2B	0.014115
BTRF1	FAM69C	0.08382
BTRF1	G4	0.013779
BTRF1	LPCAT3	0.019452
BTRF1	CLTA	0.030066
BTRF1	CRP	0.011721
BTRF1	NECAP2	0.022639
BTRF1	AGR2	0.018748
BTRF1	GOLGA8CP	0.068524
BTRF1	DBNDD2	0.022032
BTRF1	KCNK12	0.012804
BTRF1	CCDC150	0.011746
BTRF1	PSME2	0.011533
BTRF1	C1orf105	0.026705
BTRF1	PRADC1	0.024017
BTRF1	TMEM184C	0.018766
BTRF1	FIP1L1	0.031498
BTRF1	TPD52L1	0.026414
BTRF1	ASPHD1	0.017853
BTRF1	FRG1	0.010686
BTRF1	ZBP1	0.013134
BTRF1	ALKBH1	0.012019
BTRF1	NMT2	0.012336
BTRF1	TBC1D28	0.010461
BTRF1	POLR3F	0.010537

BTRF1	TMEM215	0.010787
BTRF1	CCDC176	0.07109
BTRF1	GOLGA6C	0.011598
BTRF1	PUS7L	0.01028
BTRF1	HMBS	0.01111
BTRF1	GCH1	0.024596
BTRF1	TRAPPC1	0.022825
BTRF1	KRTAP13-4	0.013685
BTRF1	CASD1	0.041104
BTRF1	AP5B1	0.019759
BTRF1	CHKA	0.014774
BTRF1	C19orf71	0.013602
BTRF1	SNX29P2	0.01622
BTRF1	NXF3	0.017522
BTRF1	TGOLN2	0.023082
BTRF1	HABP4	0.023437
BTRF1	PLD4	0.015773
BTRF1	ABRA	0.021066
BTRF1	ADORA3	0.017953
BTRF1	AKAP2	0.015405
BTRF1	CCDC137	0.065448
BTRF1	GPR179	0.049604
BTRF1	NCAPG	0.023901
BTRF1	NANOS2	0.011768
BTRF1	BCL7C	0.044684
BTRF1	JOSD1	0.012241
BTRF1	BCDIN3D	0.044938
BTRF1	ZNF830	0.032068
BTRF1	CROCCP3	0.010236
BTRF1	NXT2	0.012427
BTRF1	FAM151B	0.012669
BTRF1	NSUN7	0.063396
BTRF1	MAGEB18	0.011313
BTRF1	TBRG4	0.02208
BTRF1	FNIP2	0.085665
BTRF1	TREML1	0.013222
BTRF1	RPP21	0.028122
BTRF1	STMN2	0.014961
BTRF1	NPIPA1	0.022187
BTRF1	FAM47B	0.021069
BTRF1	RDM1	0.024163
BTRF1	CENPH	0.01205
BTRF1	FBXO46	0.010687
BTRF1	SRL	0.019069
BTRF1	TIMM44	0.013346
BTRF1	GTF2E1	0.026027
BTRF1	UTP3	0.014806
BTRF1	SMIM14	0.014647
BTRF1	LIN54	0.010991
BTRF1	RHD	0.018325
BTRF1	GEMIN6	0.01342
BTRF1	SLC1A5	0.011253
BTRF1	EXOSC1	0.012326
BTRF1	VWA5B1	0.014983
BTRF1	RMI1	0.013225
BTRF1	FAM69B	0.088148
BTRF1	ARL14EPL	0.044583
BTRF1	OGFR	0.016755
BTRF1	ATP6V1E2	0.016387
BTRF1	SLC34A1	0.035924
BTRF1	MBD5	0.019305
BTRF1	C17orf99	0.010368
BTRF1	CHGA	0.072056
BTRF1	C17orf66	0.011761
BTRF1	FAM174B	0.016933
BTRF1	IWS1	0.034806
BTRF1	NT5DC3	0.010574
BTRF1	PVRIG	0.020792

BTRF1	SLC38A9	0.010983
BTRF1	NDEL1	0.016413
BTRF1	SPINK5	0.010587
BTRF1	ACOT7	0.010073
BTRF1	SLAIN1	0.018247
BTRF1	SAA2	0.022792
BTRF1	ZNF326	0.018394
BTRF1	SMYD5	0.01391
BTRF1	ANXA2R	0.048642
BTRF1	LINC00116	0.015948
BTRF1	LGALS13	0.011947
BTRF1	BID	0.018502
BTRF1	COMMD2	0.037234
BTRF1	SDPR	0.010628
BTRF1	NIPSNAP3A	0.012002
BTRF1	SLC13A1	0.010185
BTRF1	PIR	0.011311
BTRF1	SPIN1	0.020165
BTRF1	PRAMEF13	0.059609
BTRF1	SRRM1	0.055445
BTRF1	ALB	0.010402
BTRF1	YJR030C	0.02301
BTRF1	KIAA1841	0.035416
BTRF1	C1orf196	0.014363
BTRF1	PRODH	0.03365
BTRF1	MFSD6	0.043825
BTRF1	LRCOL1	0.05311
BTRF1	YTHDC1	0.016486
BTRF1	NBPF6	0.010783
BTRF1	MRPS10	0.016034
BTRF1	GEMIN7	0.019457
BTRF1	LAMP5	0.029023
BTRF1	AMY1A	0.014325
BTRF1	TXNIP	0.039626
BTRF1	PITPNA	0.017588
BTRF1	APRG1	0.018627
BTRF1	P4HA2	0.020333
BTRF1	GTF3C6	0.015575
BTRF1	WIBG	0.031645
BTRF1	SLCO1A2	0.014957
BTRF1	PDE6H	0.034446
BTRF1	MPTX1	0.018054
BTRF1	ERI3	0.010637
BTRF1	YTHDF2	0.028574
BTRF1	SKP1	0.011536
BTRF1	KHDC3L	0.016967
BTRF1	SHCBP1	0.11721
BTRF1	COX10	0.015649
BTRF1	POLRMT	0.021354
BTRF1	C1orf94	0.078672
BTRF1	WFDC12	0.010775
BTRF1	MCUR1	0.012905
BTRF1	HGD	0.045771
BTRF1	BSN	0.032925
BTRF1	HEMK1	0.018656
BTRF1	FAM210A	0.04222
BTRF1	PNLDC1	0.018868
BTRF1	TSPAN10	0.012088
BTRF1	COMMD9	0.018014
BTRF1	DNASE1L2	0.049216
BTRF1	SYPL1	0.059626
BTRF1	ERLIN1	0.02677
BTRF1	UBXN11	0.021977
BTRF1	AHSA1	0.016114
BTRF1	CRB3	0.020753
BTRF1	ITIH2	0.012268
BTRF1	AOC2	0.013372
BTRF1	C2CD4D	0.013448

BTRF1	RRP1B	0.025984
BTRF1	PGK2	0.011526
BTRF1	NUP98	0.010719
BTRF1	SLC1A1	0.01105
BTRF1	SLC1A2	0.01105
BTRF1	COMMD8	0.011908
BTRF1	LY6K	0.012693
BTRF1	TPT1P8	0.013776
BTRF1	FAM19A3	0.071614
BTRF1	CAMSAP3	0.033308
BTRF1	NABP1	0.015588
BTRF1	CCDC132	0.0103
BTRF1	SDSL	0.024646
BTRF1	ATG4C	0.019693
BTRF1	FAM21B	0.018857
BTRF1	PLSCR4	0.030578
BTRF1	DNAJC9-AS1	0.01493
BTRF1	SETD3	0.01481
BTRF1	CYB561D1	0.018715
BTRF1	WIPF3	0.051391
BTRF1	C5orf55	0.017768
BTRF1	WWC2-AS2	0.010344
BTRF1	SYNGR3	0.021035
BTRF1	RPP25L	0.025059
BTRF1	NDUFAF7	0.02313
BTRF1	ESF1	0.045973
BTRF1	ANO2	0.010634
BTRF1	NSUN5	0.031598
BTRF1	PACS2	0.034056
BTRF1	AFM	0.02076
BTRF1	PGK1	0.011766
BTRF1	RBM27	0.011388
BTRF1	CNPY4	0.056806
BTRF1	NELFA	0.039834
BTRF1	PHACTR1	0.013857
BTRF1	UBXN2A	0.027343
BTRF1	DHFRL1	0.017378
BTRF1	APOO	0.022064
BTRF1	FUNDC2	0.012718
BTRF1	FAM19A4	0.017876
BTRF1	ARRDC3	0.023433
BTRF1	SERBP1	0.015518
BTRF1	MBOAT1	0.0165
BTRF1	SPG20	0.010969
BTRF1	ytzL	0.074946
BTRF1	EMID1	0.014747
BTRF1	WDR96	0.026194
BTRF1	EIF6	0.013958
BTRF1	TSEN2	0.027595
BTRF1	TNRC6B	0.017164
BTRF1	C15orf65	0.012917
BTRF1	MAPKAP1	0.016487
BTRF1	NUCB1	0.015733
BTRF1	PRIMA1	0.034494
BTRF1	OFD1	0.014481
BTRF1	SSX1	0.017622
BTRF1	NAPG	0.013452
BTRF1	FBXL8	0.083475
BTRF1	MFAP1	0.020971
BTRF1	TTYH1	0.068557
BTRF1	C10orf82	0.028147
BTRF1	ERICH1-AS1	0.018246
BTRF1	CCRN4L	0.019
BTRF1	ATXN1	0.022837
BTRF1	SYNGR1	0.019977
BTRF1	ORMDL2	0.035115
BTRF1	DYDC2	0.012686
BTRF1	BCL9L	0.017071

BTRF1	TET1	0.015168
BTRF1	TGM2	0.01036
BTRF1	YIF1A	0.022134
BTRF1	BEX1	0.043871
BTRF1	TTYH3	0.061602
BTRF1	PRPH2	0.012877
BTRF1	ARRDC4	0.01447
BTRF1	VWA9	0.012196
BTRF1	MTTP	0.027536
BTRF1	GADD45G	0.017031
BTRF1	BEND2	0.01922
BTRF1	EXOC3L2	0.011988
BTRF1	UNC119	0.011242
BTRF1	FAM188B	0.06958
BTRF1	RHCG	0.014646
BTRF1	ANTXR1	0.018795
BTRF1	UHRF1BP1L	0.012208
BTRF1	UNQ6126/PRO20091	0.019257
BTRF1	GOLGA6D	0.012976
BTRF1	CDCP1	0.016912
BTRF1	OAS1	0.013507
BTRF1	AFAP1L1	0.010721
BTRF1	INSL4	0.020563
BTRF1	IGFLR1	0.0149
BTRF1	GGNBP2	0.022995
BTRF1	CT55	0.041757
BTRF1	EPG5	0.019094
BTRF1	B3GNTL1	0.015321
BTRF1	NXT1	0.010113
BTRF1	UPRT	0.010776
BTRF1	FLG	0.020547
BTRF1	CHMP7	0.019293
BTRF1	SLC23A3	0.010881
BTRF1	FAM131A	0.020422
BTRF1	RABEP2	0.075122
BTRF1	CYB561	0.019553
BTRF1	XPO6	0.01362
BTRF1	CHAF1A	0.040588
BTRF1	TP53INP1	0.015635
BTRF1	CBWD7	0.010904
BTRF1	GPATCH3	0.013417
BTRF1	YOD1	0.011877
BTRF1	FAIM3	0.013608
BTRF1	MAB21L2	0.012478
BTRF1	GGN	0.026018
BTRF1	PFDN2	0.013534
BTRF1	TMEM120B	0.016326
BTRF1	LAMP1	0.024408
BTRF1	CHGB	0.10868
BTRF1	SLC16A11	0.011711
BTRF1	CMTR2	0.012139
BTRF1	NT5C3A	0.037285
BTRF1	SART1	0.010284
BTRF1	IBA57	0.026738
BTRF1	ATCAY	0.01012
BTRF1	G4	0.029431
BTRF1	WLS	0.011072
BTRF1	IFT52	0.038988
BTRF1	CAGE1	0.013314
BTRF1	VWA5A	0.031833
BTRF1	LINC00205	0.016925
BTRF1	FAM46B	0.010687
BTRF1	TPPP3	0.032015
BTRF1	NSUN5P2	0.043746
BTRF1	TGM1	0.010122
BTRF1	TMEM8B	0.012521
BTRF1	VWA5B2	0.018898
BTRF1	IP6K3	0.012896

6665	BTRF1	PEPD	0.023366
6666	BTRF1	NKAIN2	0.035695
6667	BTRF1	PRAME	0.040956
6668	BTRF1	TAS2R10	0.021853
6669	BTRF1	FTSJ1	0.021242
6670	BTRF1	PPP2R3C	0.021501
6671	BTRF1	ATG10	0.024898
6672	BTRF1	EIF4E	0.010523
6673	BTRF1	BBC3	0.023138
6674	BTRF1	OAS3	0.013328
6675	BTRF1	RQCD1	0.010085
6676	BTRF1	ATP5F1	0.011331
6677	BTRF1	C19orf12	0.026976
6678	BTRF1	C12orf63	0.015697
6679	BTRF1	UCKL1	0.010993
6680	BTRF1	LEMD2	0.012319
6681	BTRF1	SEC11C	0.012984
6682	BTRF1	TSN	0.017199
6683	BTRF1	SDS	0.011492
6684	BTRF1	TCEAL5	0.021886
6685	BTRF1	RPGRIP1	0.010299
6686	BTRF1	POM121	0.025806
6687	BTRF1	COBL	0.015561
6688	BTRF1	GBE1	0.013926
6689	BTRF1	PORCN	0.013643
6690	BTRF1	DEFB129	0.047948
6691	BTRF1	LSM12	0.032995
6692	BTRF1	TRAPPC9	0.010693
6693	BTRF1	C12orf57	0.025112
6694	BTRF1	SLC18B1	0.013884
6695	BTRF1	ALKBH2	0.02575
6696	BTRF1	TMEM8A	0.040367
6697	BTRF1	IGFL4	0.017267
6698	BTRF1	YAE1D1	0.026061
6699	BTRF1	LGALS12	0.01031
6700	BTRF1	AMELY	0.010105
6701	BTRF1	SPATA31E1	0.1146
6702	BTRF1	POLR2L	0.013898
6703	BTRF1	AMY2A	0.013268
6704	BTRF1	PANK3	0.041558
6705	BTRF1	SFI1	0.043529
6706	BTRF1	C1orf186	0.014581
6707	BTRF1	DHFR	0.012317
6708	BTRF1	PRRG4	0.011931
6709	BTRF1	APCS	0.020675
6710	BTRF1	AKAP3	0.020404
6711	BTRF1	GOLGA8I	0.018923
6712	BTRF1	RHAG	0.010014
6713	BTRF1	CD164	0.018629
6714	BTRF1	AOC3	0.012118
6715	BTRF1	EXOC8	0.011703
6716	BTRF1	CGGBP1	0.029645
6717	BTRF1	TIGD1	0.016836
6718	BTRF1	GTSF1	0.073675
6719	BTRF1	SCAPER	0.010859
6720	BTRF1	PNPT1	0.011458
6721	BTRF1	UPF3B	0.043875
6722	BTRF1	C5orf58	0.022902
6723	BTRF1	RPRD2	0.013741
6724	BTRF1	DPP7	0.034486
6725	BTRF1	CCDC169	0.01766
6726	BTRF1	OVCA2	0.019039
6727	BTRF1	NBPF4	0.010783
6728	BTRF1	MKI67	0.012187
6729	BTRF1	COL26A1	0.062664
6730	BTRF1	ARMCX5	0.010946
6731	BTRF1	SUPT7L	0.016516
6732	BTRF1	LSM11	0.033793

BTRF1	CYB561A3	0.017005
BTRF1	CHORDC1	0.010377
BTRF1	SLC51A	0.01206
BTRF1	LAPTM5	0.024705
BTRF1	ATXN1L	0.032173
BTRF1	GK2	0.011829
BTRF1	SLC16A9	0.010296
BTRF1	MUC1	0.048397
BTRF1	CDK5R1	0.030143
BTRF1	SUB1	0.025503
BTRF1	APOPT1	0.013772
BTRF1	SYNGR2	0.019631
BTRF1	TRAPPC6A	0.036063
BTRF1	CACNA2D3	0.027619
BTRF1	GADD45A	0.022687
BTRF1	LYZL1	0.038473
BTRF1	IL17RC	0.013291
BTRF1	CMTM6	0.028843
BTRF1	TMEM248	0.033926
BTRF1	C5orf34	0.012717
BTRF1	GAS2L2	0.010303
BTRF1	NPTX2	0.02113
BTRF1	C17orf102	0.012642
BTRF1	TMED1	0.016139
BTRF1	TIMP1	0.014884
BTRF1	FAM47C	0.03583
BTRF1	C10orf111	0.050035
BTRF1	AMOTL1	0.019233
BTRF1	GTPBP2	0.016956
BTRF1	CCHCR1	0.021878
BTRF1	ZAR1L	0.017375
BTRF1	LYPD6B	0.059845
BTRF1	FBXO10	0.011226
BTRF1	PARP10	0.019047
BTRF1	ALKBH4	0.016683
BTRF1	SLC4A2	0.010109
BTRF1	MNS1	0.036863
BTRF1	LCA10	0.02917
BTRF1	BRINP3	0.011378
BTRF1	NOL6	0.012308
BTRF1	TRAPPC5	0.012098
BTRF1	BRICD5	0.017244
BTRF1	P4HA3	0.011104
BTRF1	CNGC19	0.038229
BTRF1	ENY2	0.013506
BTRF1	LETM2	0.026373
BTRF1	CNRIP1	0.015079
BTRF1	PRPF3	0.012761
BTRF1	C1orf64	0.039592
BTRF1	MBOAT7	0.016898
BTRF1	NAA35	0.03118
BTRF1	FAM69A	0.030277
BTRF1	ETNK2	0.021707
BTRF1	WIPF2	0.011406
BTRF1	YIF1B	0.021443
BTRF1	AC3.5	0.031982
BTRF1	TTR	0.011239
BTRF1	ITPKB	0.019564
BTRF1	C2orf81	0.035687
BTRF1	TREX2	0.015759
BTRF1	DNASE1L3	0.012666
BTRF1	ASIP	0.031054
BTRF1	PUS3	0.038959
BTRF1	CGREF1	0.01153
BTRF1	CACNA2D4	0.03128
BTRF1	AKAP8	0.011147
BTRF1	ALPK1	0.01128
BTRF1	FAM49B	0.023785

BTRF1	FAM199X	0.014329
BTRF1	PAGE4	0.01506
BTRF1	TIPRL	0.032282
BTRF1	MILR1	0.014592
BTRF1	BPIFB2	0.012444
BTRF1	SLC1A6	0.011299
BTRF1	CADPS	0.028134
BTRF1	PRAMEF14	0.054123
BTRF1	ATG9B	0.03222
BTRF1	FAM161B	0.015582
BTRF1	CCAR1	0.056559
BTRF1	PFDN4	0.016412
BTRF1	C16orf93	0.016802
BTRF1	CCDC171	0.015289
BTRF1	CCAR2	0.033408
BTRF1	PUS1	0.011617
BTRF1	COLGALT1	0.011597
BTRF1	C1orf195	0.084001
BTRF1	SNX25	0.019066
BTRF1	WFDC5	0.017182
BTRF1	MUM1	0.010555
BTRF1	IL21	0.01178
BTRF1	RBBP9	0.023877
BTRF1	CNTLN	0.014209
BTRF1	SLC34A3	0.020097
BTRF1	TRIP4	0.012002
BTRF1	LEO1	0.012442
BTRF1	CCDC22	0.030735
BTRF1	YBR056C-B	0.026496
BTRF1	CCPG1	0.025147
BTRF1	SF3A3	0.012677
BTRF1	C6orf10	0.026628
BTRF1	CEP57	0.011873
BTRF1	SCIMP	0.029569
BTRF1	NSUN3	0.017019
BTRF1	PRKRIP1	0.0282
BTRF1	LUZP2	0.031712
BTRF1	TFB2M	0.053795
BTRF1	SNG1	0.018648
BTRF1	SPACA5	0.028783
BTRF1	SYCP1	0.010077
BTRF1	PRAP1	0.023864
BTRF1	GYG2	0.014531
BTRF1	PILRA	0.016194
BTRF1	EAF1	0.062324
BTRF1	LETM1	0.029727
BTRF1	TMEM98	0.035812
BTRF1	CTU1	0.010301
BTRF1	MTHFR	0.014365
BTRF1	MAD2L1BP	0.016563
BTRF1	FAM231A	0.13576
BTRF1	FAM124A	0.056589
BTRF1	TMEM181	0.018232
BTRF1	SLC1A3	0.011275
BTRF1	JMY	0.014123
BTRF1	CHST6	0.016701
BTRF1	TRIP11	0.013677
BTRF1	RWDD3	0.010114
BTRF1	OOEP	0.0432
BTRF1	PANK1	0.014939
BTRF1	PLSCR1	0.032216
BTRF1	FRRS1	0.020926
BTRF1	METTL25	0.011616
BTRF1	GNPNAT1	0.031691
BTRF1	C9orf89	0.015959
BTRF1	COPRS	0.022611
BTRF1	MRPL42	0.010273
BTRF1	CAMLG	0.020978

BTRF1	NKAPL	0.071352
BTRF1	SLURP1	0.027464
BTRF1	PRR21	0.010264
BTRF1	MED12	0.021599
BTRF1	TLDC2	0.038896
BTRF1	TPD52L2	0.02996
BTRF1	IFIT2	0.02124
BTRF1	DCT	0.010332
BTRF1	N4BP3	0.010793
BTRF1	METTL20	0.012519
BTRF1	TBC1D20	0.010554
BTRF1	NSUN5P1	0.034903
BTRF1	SHPK	0.011969
BTRF1	FAM24A	0.022648
BTRF1	TCAP	0.02446
BTRF1	DAG1	0.025475
BTRF1	AGTRAP	0.016635
BTRF1	CSRNP3	0.017557
BTRF1	SUMF2	0.039646
BTRF1	CHCHD4	0.013437
BTRF1	TNMD	0.012708
BTRF1	SCAI	0.032346
BTRF1	NIPBL	0.010533
BTRF1	TAS2R31	0.016211
BTRF1	FAM21A	0.018409
BTRF1	C5orf48	0.039412
BTRF1	ETNK1	0.01968
BTRF1	PILRB	0.014731
BTRF1	CHMP4C	0.015583
BTRF1	AKIRIN1	0.030205
BTRF1	RCAN1	0.011473
BTRF1	AKAP8L	0.021895
BTRF1	SYNGR4	0.094181
BTRF1	MLC1	0.013302
BTRF1	IFI35	0.011557
BTRF1	PXMP4	0.014327
BTRF1	SMIM9	0.03022
BTRF1	GUK1	0.018735
BTRF1	TRMT61B	0.023926
BTRF1	ATIC	0.011698
BTRF1	SECISBP2L	0.018101
BTRF1	CDK5RAP3	0.016132
BTRF1	CHMP4A	0.022733
BTRF1	TBCCD1	0.010748
BTRF1	GON4L	0.015465
BTRF1	CHMP5	0.01886
BTRF1	FAM92A1	0.011722
BTRF1	GOLGA6B	0.013422
BTRF1	AASDHPPT	0.045073
BTRF1	MBOAT4	0.018958
BTRF1	LY6G6C	0.01723
BTRF1	RINL	0.012954
BTRF1	NUP205	0.015577
BTRF1	C16orf45	0.019333
BTRF1	ASCC2	0.03816
BTRF1	ATF4	0.010483
BTRF1	RPUSD2	0.014821
BTRF1	ABHD17B	0.026221
BTRF1	SLPI	0.024472
BTRF1	LYZ	0.029627
BTRF1	FSCN1	0.019679
BTRF1	NGB	0.01941
BTRF1	HEATR5B	0.044957
BTRF1	PDCD4	0.011427
BTRF1	ADAT1	0.014193
BTRF1	OAZ3	0.02145
BTRF1	C19orf66	0.025698
BTRF1	NXPH1	0.013575

BTRF1	C4orf6	0.012673
BTRF1	M6PR	0.022192
BTRF1	GAS2L1	0.015166
BTRF1	VIPAS39	0.013165
BTRF1	N4BP2L1	0.013707
BTRF1	FAM231D	0.053566
BTRF1	ANAPC10	0.014785
BTRF1	SAC3D1	0.018606
BTRF1	SLC1A4	0.011275
BTRF1	GML	0.042525
BTRF1	KIAA0087	0.011887
BTRF1	CD68	0.022275
BTRF1	C11orf72	0.017663
BTRF1	TSACC	0.014418
BTRF1	IQCF3	0.010092
BTRF1	BLOC1S5	0.014017
BTRF1	C6orf106	0.035352
BTRF1	GNRH1	0.012348
BTRF1	MFSD7	0.022121
BTRF1	PLSCR2	0.031932
BTRF1	IL2RA	0.015804
BTRF1	AIM1L	0.01006
BTRF1	TP53INP2	0.011853
BTRF1	FSIP2	0.030486
BTRF1	GTF2E2	0.025057
BTRF1	DAK	0.010759
BTRF1	SLC8B1	0.014379
BTRF1	TPD52	0.010461
BTRF1	SLC16A8	0.012648
BTRF1	RIPPLY3	0.018871
BZLF1	HPS4	0.16097
BZLF1	CBX7	0.1525
BZLF1	ACR	0.1423
BZLF1	ACVR1	0.023369
BZLF1	TAF6	0.022618
BZLF1	GPAA1	0.10587
BZLF1	TRIB3	0.048495
BZLF1	FPR1	0.13103
BZLF1	BRAP	0.12022
BZLF1	SERPINB9	0.12821
BZLF1	SLC25A25	0.052515
BZLF1	MACROD1	0.19527
BZLF1	ITGB5	0.10997
BZLF1	HID1	0.1525
BZLF1	ECT2	0.02699
BZLF1	SHQ1	0.17709
BZLF1	PEX10	0.15157
BZLF1	NLRP2	0.12919
BZLF1	RBL1	0.078774
BZLF1	BIN2	0.22903
BZLF1	BBS10	0.16778
BZLF1	USP2	0.075027
BZLF1	MED1	0.016142
BZLF1	CDC34	0.077963
BZLF1	RGCC	0.03592
BZLF1	EFHC2	0.057416
BZLF1	CDK10	0.077473
BZLF1	EPHA3	0.084056
BZLF1	EFCAB1	0.057416
BZLF1	PIK3CD	0.093643
BZLF1	RAB7A	0.10693
BZLF1	TGFBR2	0.018852
BZLF1	N4BP1	0.17835
BZLF1	CTDNEP1	0.079915
BZLF1	FAM58A	0.10558
BZLF1	PSMD4	0.10128
BZLF1	FZD8	0.06221
BZLF1	PAGE2B	0.011844

BZLF1	PPP5C	0.074207
BZLF1	SLC2A4	0.1095
BZLF1	KEL	0.10395
BZLF1	IFT122	0.20565
BZLF1	NAE1	0.11815
BZLF1	PRPF6	0.17053
BZLF1	SPTA1	0.20259
BZLF1	SEC31A	0.12185
BZLF1	GZMA	0.17122
BZLF1	S100A7	0.26154
BZLF1	IL4	0.030307
BZLF1	NANOS1	0.19433
BZLF1	SLC26A8	0.15375
BZLF1	UBE2H	0.11844
BZLF1	HSPA5	0.14114
BZLF1	LDLR	0.11136
BZLF1	NEDD4	0.041017
BZLF1	LAMTOR5	0.15043
BZLF1	GSPT2	0.1216
BZLF1	SERPINB5	0.16535
BZLF1	SSBP1	0.17271
BZLF1	BIRC6	0.10053
BZLF1	SERTAD3	0.16297
BZLF1	INO80B	0.14187
BZLF1	KLK13	0.19711
BZLF1	AGO2	0.079919
BZLF1	HEPACAM2	0.12981
BZLF1	RNMTL1	0.031621
BZLF1	CENPW	0.14398
BZLF1	UNC13D	0.14312
BZLF1	UPF3A	0.22018
BZLF1	ARHGAP33	0.158
BZLF1	RB1CC1	0.053948
BZLF1	RABEP1	0.23811
BZLF1	SKA3	0.15184
BZLF1	VPS54	0.19518
BZLF1	KIAA1377	0.23617
BZLF1	EIF5A	0.080652
BZLF1	CYS1	0.17767
BZLF1	LIMK1	0.05855
BZLF1	FAF1	0.087546
BZLF1	SLC5A1	0.13353
BZLF1	LRP2BP	0.22903
BZLF1	MAPK8	0.051233
BZLF1	EDF1	0.05203
BZLF1	KCNS1	0.1434
BZLF1	PXN	0.083868
BZLF1	NUP85	0.09266
BZLF1	SOX13	0.056668
BZLF1	CTNND2	0.13027
BZLF1	UBE2D3	0.073979
BZLF1	KRT5	0.13144
BZLF1	HSD17B8	0.13534
BZLF1	BTNL8	0.2221
BZLF1	PLSCR3	0.3631
BZLF1	KREMEN1	0.20332
BZLF1	TCF25	0.056545
BZLF1	NR0B1	0.032925
BZLF1	SNX20	0.18548
BZLF1	BIRC7	0.11583
BZLF1	MCM6	0.062032
BZLF1	FKBP10	0.059302
BZLF1	KRT17	0.10765
BZLF1	MAPKAPK2	0.037816
BZLF1	NOSIP	0.12412
BZLF1	SMURF2	0.070672
BZLF1	DYM	0.16591
BZLF1	ANKIB1	0.18896

BZLF1	ATP6V0D2	0.14096
BZLF1	PTRH2	0.052508
BZLF1	ORAI3	0.2221
BZLF1	CSNK2A1	0.065183
BZLF1	PFKM	0.093063
BZLF1	SPRR2E	0.094134
BZLF1	ELAVL2	0.24605
BZLF1	RPUSD4	0.3381
BZLF1	EMR1	0.018364
BZLF1	CD248	0.058166
BZLF1	ASIC1	0.13132
BZLF1	ORC2	0.12942
BZLF1	PEX16	0.13207
BZLF1	CALN1	0.046998
BZLF1	F9	0.0821
BZLF1	CRB1	0.079989
BZLF1	TRAPPC2	0.095703
BZLF1	NIP7	0.25297
BZLF1	C16orf70	0.20237
BZLF1	USP12	0.16606
BZLF1	DDB2	0.1225
BZLF1	TUBA1B	0.01423
BZLF1	PIP5K1A	0.08567
BZLF1	PEAK1	0.12747
BZLF1	SUN1	0.11645
BZLF1	SFTPD	0.18217
BZLF1	PIP5K1B	0.20153
BZLF1	FAT3	0.047714
BZLF1	TAB3	0.082516
BZLF1	SBF2	0.11529
BZLF1	SLC7A11	0.095003
BZLF1	BNIP1	0.1029
BZLF1	BRMS1	0.13449
BZLF1	FGFR1OP	0.089593
BZLF1	PCDH20	0.049103
BZLF1	DARS2	0.070205
BZLF1	PTPRH	0.031564
BZLF1	SPRR1A	0.094778
BZLF1	CALCOCO2	0.15737
BZLF1	USHBP1	0.21274
BZLF1	CD1B	0.17953
BZLF1	LALBA	0.037133
BZLF1	BRD4	0.12345
BZLF1	EFCAB5	0.057416
BZLF1	TFAP2B	0.021819
BZLF1	PRPF4B	0.09385
BZLF1	MYF5	0.080122
BZLF1	ARHGEF12	0.05703
BZLF1	RNF2	0.07367
BZLF1	NT5C2	0.071829
BZLF1	LLGL2	0.14663
BZLF1	MYH7	0.14901
BZLF1	SORBS3	0.094242
BZLF1	POLD2	0.094305
BZLF1	SNAPIN	0.14038
BZLF1	INTS6	0.17541
BZLF1	STX3	0.10928
BZLF1	PCDH12	0.039246
BZLF1	PDE2A	0.04536
BZLF1	PLXNB1	0.088812
BZLF1	CARTPT	0.10853
BZLF1	HMGB1	0.038908
BZLF1	KCNJ5	0.1861
BZLF1	MLKL	0.088753
BZLF1	MKS1	0.14161
BZLF1	HSPE1	0.14701
BZLF1	MAPK7	0.057116
BZLF1	USP11	0.1463

BZLF1	DTX1	0.084086
BZLF1	ADAMTS5	0.076716
BZLF1	RANBP9	0.13995
BZLF1	SUFU	0.042897
BZLF1	SPHKAP	0.2221
BZLF1	PXMP2	0.18496
BZLF1	SYTL1	0.16926
BZLF1	SOCS7	0.16175
BZLF1	TYRP1	0.1464
BZLF1	ZFPM1	0.026822
BZLF1	UBA1	0.10489
BZLF1	BTBD2	0.21665
BZLF1	EIF3J	0.23414
BZLF1	RTN2	0.17519
BZLF1	RP1	0.11663
BZLF1	CENPJ	0.072007
BZLF1	BCCIP	0.1856
BZLF1	F5	0.14281
BZLF1	IGSF1	0.17412
BZLF1	KCND2	0.090004
BZLF1	PTPN1	0.039059
BZLF1	FKBP1B	0.082506
BZLF1	APLP2	0.075247
BZLF1	C10orf62	0.23617
BZLF1	TERF2	0.075783
BZLF1	RNF167	0.12874
BZLF1	UNC119B	0.20404
BZLF1	RACGAP1	0.038338
BZLF1	RBL2	0.11505
BZLF1	CTBP2	0.11049
BZLF1	JAK3	0.061457
BZLF1	CCNB1IP1	0.13967
BZLF1	ACTA1	0.091723
BZLF1	PSMA1	0.066841
BZLF1	DHX30	0.20506
BZLF1	NLRX1	0.10515
BZLF1	VASP	0.13615
BZLF1	PCDHGA1	0.049103
BZLF1	CABP7	0.046998
BZLF1	CASC3	0.14205
BZLF1	WASF2	0.14272
BZLF1	CD58	0.17798
BZLF1	DUSP1	0.010671
BZLF1	MAP2	0.11898
BZLF1	TBL1XR1	0.08173
BZLF1	ZFYVE27	0.12932
BZLF1	POP7	0.16251
BZLF1	APBB1	0.034251
BZLF1	DNM3	0.026071
BZLF1	PCNT	0.1625
BZLF1	MRPL13	0.18431
BZLF1	U2AF1	0.15567
BZLF1	ARHGDIA	0.13048
BZLF1	PDXP	0.086499
BZLF1	BOC	0.13689
BZLF1	P4HB	0.046536
BZLF1	SNRPA	0.18528
BZLF1	GAREM	0.23617
BZLF1	ELP5	0.1745
BZLF1	ALAS2	0.11275
BZLF1	IGFBP5	0.10512
BZLF1	ZNF675	0.072523
BZLF1	PRKCD	0.020664
BZLF1	DVL2	0.087202
BZLF1	MLH1	0.048267
BZLF1	CHRD	0.11284
BZLF1	BICD2	0.23865
BZLF1	APOB	0.01773

BZLF1	KIF7	0.13866
BZLF1	ADAM9	0.034314
BZLF1	ATP5B	0.1467
BZLF1	NIN	0.3421
BZLF1	UBB	0.02862
BZLF1	TXNDC17	0.18967
BZLF1	ARHGEF1	0.070361
BZLF1	PYCARD	0.021756
BZLF1	MUL1	0.01641
BZLF1	ITCH	0.075971
BZLF1	CEP192	0.16395
BZLF1	SYTL2	0.10816
BZLF1	PGR	0.020247
BZLF1	CARD11	0.096302
BZLF1	LMO2	0.074039
BZLF1	KCNIP1	0.2834
BZLF1	CDC27	0.11491
BZLF1	VPS26A	0.14367
BZLF1	BATF	0.061811
BZLF1	SH3BP5	0.2257
BZLF1	ZC3HC1	0.066583
BZLF1	CDH18	0.046388
BZLF1	AKTIP	0.13776
BZLF1	ANKEF1	0.057416
BZLF1	TNIP1	0.14818
BZLF1	TLE3	0.14912
BZLF1	STIM2	0.34028
BZLF1	FUNDC1	0.1301
BZLF1	FAM109A	0.16289
BZLF1	GP1BA	0.12183
BZLF1	XRN1	0.13392
BZLF1	WBP2	0.22361
BZLF1	CEACAM1	0.16501
BZLF1	NUP107	0.11792
BZLF1	ERBB3	0.055574
BZLF1	ARL11	0.19542
BZLF1	MEGF9	0.052629
BZLF1	SLAIN2	0.22406
BZLF1	SSSCA1	0.20735
BZLF1	CCDC130	0.22358
BZLF1	BNIPL	0.14659
BZLF1	KRT7	0.094185
BZLF1	ZHX2	0.030945
BZLF1	CTCFL	0.088736
BZLF1	RPLP1	0.18101
BZLF1	DERL3	0.17573
BZLF1	KIAA1045	0.03514
BZLF1	SERGEF	0.16089
BZLF1	COG2	0.13423
BZLF1	CD247	0.14659
BZLF1	DSN1	0.14024
BZLF1	ELK1	0.039775
BZLF1	TRAF2	0.0339
BZLF1	TCEA2	0.092542
BZLF1	NKAP	0.12392
BZLF1	FIS1	0.042223
BZLF1	TMED10	0.1187
BZLF1	RALBP1	0.16855
BZLF1	NDUFAF4	0.24141
BZLF1	BUB1B	0.046284
BZLF1	CST5	0.25961
BZLF1	NFKBIB	0.099484
BZLF1	THOC5	0.16212
BZLF1	NAIP	0.15164
BZLF1	DPY30	0.13046
BZLF1	EFCAB14	0.057416
BZLF1	PVRL3	0.15622
BZLF1	LRIF1	0.18862

BZLF1	TNFAIP3	0.01659
BZLF1	PLCB4	0.010043
BZLF1	TNKS	0.064359
BZLF1	TMEM30B	0.21539
BZLF1	PYDC1	0.15022
BZLF1	PITPNM2	0.03197
BZLF1	SOX4	0.015876
BZLF1	NCAPD2	0.14626
BZLF1	MIA3	0.16212
BZLF1	UHRF2	0.074467
BZLF1	CGA	0.12716
BZLF1	PDIA3	0.046914
BZLF1	BIRC3	0.10738
BZLF1	PABPN1	0.12419
BZLF1	TAF3	0.098577
BZLF1	COMMD5	0.19547
BZLF1	ACAN	0.12189
BZLF1	CLASP2	0.063653
BZLF1	NACA	0.1354
BZLF1	JAK2	0.013442
BZLF1	WIPF1	0.23884
BZLF1	SPTAN1	0.24703
BZLF1	NDE1	0.067208
BZLF1	BECN1	0.10953
BZLF1	PTPN3	0.03945
BZLF1	CD81	0.11754
BZLF1	CDC37	0.11436
BZLF1	NOL8	0.20871
BZLF1	PPM1E	0.067625
BZLF1	SPNS1	0.19438
BZLF1	RIMS1	0.11142
BZLF1	ATRIP	0.2025
BZLF1	COPS2	0.15171
BZLF1	NDUFAB1	0.032141
BZLF1	DDR1	0.072881
BZLF1	COL13A1	0.12731
BZLF1	RAD52	0.16167
BZLF1	TCF7	0.024577
BZLF1	LAMTOR1	0.13192
BZLF1	DHPS	0.22547
BZLF1	IL12A	0.065344
BZLF1	SMC3	0.080893
BZLF1	SNX24	0.0118
BZLF1	STAT5A	0.029739
BZLF1	THAP1	0.096002
BZLF1	TUBA3C	0.022139
BZLF1	CDK5RAP2	0.06237
BZLF1	PRX	0.16056
BZLF1	MCRS1	0.13686
BZLF1	POP4	0.19149
BZLF1	FBLN1	0.059907
BZLF1	EDIL3	0.049167
BZLF1	AIMP1	0.13871
BZLF1	MASP1	0.37056
BZLF1	TBL1X	0.039803
BZLF1	AIF1	0.014346
BZLF1	ZNF205	0.11682
BZLF1	INPP5D	0.067508
BZLF1	GCC2	0.10546
BZLF1	SCUBE3	0.44168
BZLF1	KIF13B	0.07925
BZLF1	SLC9A7	0.11899
BZLF1	CNOT3	0.12891
BZLF1	PTPRM	0.036152
BZLF1	BCL9	0.16423
BZLF1	NGFR	0.046568
BZLF1	TADA3	0.029617
BZLF1	STRAP	0.10987

BZLF1	PROZ	0.02232
BZLF1	LSM3	0.16958
BZLF1	DDO	0.13685
BZLF1	PEA15	0.18276
BZLF1	RPS27L	0.13096
BZLF1	RBFOX1	0.20184
BZLF1	AMIGO2	0.16371
BZLF1	MTSS1	0.088733
BZLF1	PSMC3	0.05295
BZLF1	HUWE1	0.11154
BZLF1	PPP1R26	0.1756
BZLF1	UXT	0.096091
BZLF1	TRAPPC4	0.21385
BZLF1	TRIM17	0.14221
BZLF1	HECTD3	0.11462
BZLF1	TH	0.024032
BZLF1	TNFRSF10B	0.1425
BZLF1	SCAP	0.12758
BZLF1	C1QB	0.18501
BZLF1	TRIM13	0.083725
BZLF1	FBXO7	0.16403
BZLF1	HIPK1	0.051364
BZLF1	CAMK2A	0.061892
BZLF1	CEP152	0.073978
BZLF1	APOH	0.094885
BZLF1	HEXIM1	0.14558
BZLF1	PTPRS	0.044715
BZLF1	ATXN10	0.15289
BZLF1	SCN5A	0.061226
BZLF1	GEMIN5	0.20046
BZLF1	COMMD7	0.19672
BZLF1	FHIT	0.077949
BZLF1	ADAM17	0.02832
BZLF1	CSNK1A1	0.041939
BZLF1	ATG4B	0.17722
BZLF1	TERF1	0.059908
BZLF1	MAL2	0.19191
BZLF1	PCDHGB1	0.049103
BZLF1	TDG	0.067658
BZLF1	AGTR1	0.06229
BZLF1	SEC24B	0.10355
BZLF1	ALYREF	0.1554
BZLF1	KPNA1	0.13272
BZLF1	SERINC1	0.12858
BZLF1	AATK	0.12135
BZLF1	GLTSCR1L	0.011469
BZLF1	NCF4	0.12787
BZLF1	MED8	0.1228
BZLF1	ATG16L1	0.18627
BZLF1	USP19	0.10212
BZLF1	RIN3	0.22169
BZLF1	LDOC1	0.19729
BZLF1	FSBP	0.17318
BZLF1	SH3D19	0.1261
BZLF1	TNS4	0.19111
BZLF1	MORF4L2	0.15708
BZLF1	TSR2	0.22901
BZLF1	SAV1	0.13423
BZLF1	EPB41	0.054308
BZLF1	CDH16	0.049103
BZLF1	PDE9A	0.091266
BZLF1	PEX19	0.12642
BZLF1	SYNCRIP	0.11167
BZLF1	NAGK	0.25012
BZLF1	SLC22A6	0.14701
BZLF1	BMP7	0.020762
BZLF1	PPAP2B	0.041267
BZLF1	RPS6	0.031595

BZLF1	TIA1	0.1904
BZLF1	SNRPG	0.13281
BZLF1	TP53RK	0.14496
BZLF1	PCDH18	0.047714
BZLF1	TECPR1	0.18461
BZLF1	RRM2	0.13052
BZLF1	CD300LD	0.2154
BZLF1	KCNJ10	0.077417
BZLF1	CUL4A	0.15694
BZLF1	CD244	0.18631
BZLF1	KCNH1	0.1076
BZLF1	UFC1	0.22211
BZLF1	PAIP2B	0.21536
BZLF1	PTPRN	0.10542
BZLF1	KAT7	0.076345
BZLF1	PML	0.020376
BZLF1	RPGR	0.16833
BZLF1	KBTBD6	0.21685
BZLF1	WDR5	0.11996
BZLF1	GOLGA4	0.14782
BZLF1	MALL	0.16996
BZLF1	CHMP4B	0.17428
BZLF1	TLK2	0.11768
BZLF1	NLRC4	0.073994
BZLF1	PROC	0.32139
BZLF1	PDCD6	0.33409
BZLF1	DKC1	0.18327
BZLF1	USP32	0.045874
BZLF1	OTUD7B	0.11071
BZLF1	COG1	0.15529
BZLF1	S100A9	0.12138
BZLF1	ANXA2	0.2076
BZLF1	TMPO	0.15866
BZLF1	PSMD2	0.093753
BZLF1	MMP20	0.26203
BZLF1	RAET1G	0.19618
BZLF1	DAZL	0.14283
BZLF1	NR2C2AP	0.187
BZLF1	MCM4	0.074902
BZLF1	RTN4	0.05585
BZLF1	RUNX1	0.049153
BZLF1	TOP3A	0.099042
BZLF1	CDKN1A	0.04696
BZLF1	KIF23	0.099359
BZLF1	IL13RA1	0.20858
BZLF1	EZH2	0.10101
BZLF1	MATN4	0.46532
BZLF1	SLX4IP	0.23617
BZLF1	POLG	0.1455
BZLF1	PSMF1	0.094361
BZLF1	FMR1	0.23797
BZLF1	MAP1A	0.057329
BZLF1	NOTCH3	0.16892
BZLF1	DNMT3L	0.12293
BZLF1	VPS72	0.1437
BZLF1	TIMM50	0.07172
BZLF1	CXXC1	0.1045
BZLF1	PVALB	0.061221
BZLF1	RNH1	0.43784
BZLF1	LRRC47	0.21658
BZLF1	FOXRED2	0.25964
BZLF1	PKM	0.08355
BZLF1	ARHGEF6	0.073593
BZLF1	PIK3C2B	0.15211
BZLF1	MFF	0.087905
BZLF1	MMRN2	0.21643
BZLF1	FBXL12	0.21539
BZLF1	THOC7	0.22368

BZLF1	EGFL7	0.036668
BZLF1	POR	0.014626
BZLF1	ASPN	0.066351
BZLF1	MYL1	0.047342
BZLF1	ARL4D	0.10373
BZLF1	NUMA1	0.058198
BZLF1	MED23	0.11748
BZLF1	ADIPOQ	0.018244
BZLF1	TBC1D24	0.16589
BZLF1	HLA-DRB1	0.042526
BZLF1	MED22	0.12535
BZLF1	TGFB3	0.031623
BZLF1	KIR2DL3	0.14321
BZLF1	EGLN1	0.091346
BZLF1	PLXNC1	0.13013
BZLF1	SIAH1	0.060302
BZLF1	FSCB	0.048952
BZLF1	C2CD5	0.053341
BZLF1	SNX27	0.14183
BZLF1	PTPRJ	0.019681
BZLF1	NDUFA9	0.13319
BZLF1	PVRL4	0.17871
BZLF1	PPP6R1	0.17791
BZLF1	FRS2	0.087577
BZLF1	MEF2BNB	0.23593
BZLF1	SPSB2	0.20142
BZLF1	ROBO1	0.077207
BZLF1	PAIP1	0.18282
BZLF1	WRAP53	0.25446
BZLF1	MORF4L1	0.14112
BZLF1	SELPLG	0.13063
BZLF1	CLIP1	0.18537
BZLF1	ATP1A1	0.06696
BZLF1	BBS9	0.17465
BZLF1	YTHDF3	0.23617
BZLF1	HCK	0.053986
BZLF1	USP6	0.12023
BZLF1	TEK	0.039069
BZLF1	TAP2	0.052048
BZLF1	UGP2	0.10276
BZLF1	EXOSC5	0.14842
BZLF1	UBXN7	0.16933
BZLF1	TSHR	0.13954
BZLF1	PLXDC2	0.2221
BZLF1	BLOC1S3	0.11532
BZLF1	AMOT	0.13164
BZLF1	MAATS1	0.22217
BZLF1	TBC1D8	0.02655
BZLF1	ALOX5AP	0.087054
BZLF1	DLK2	0.073374
BZLF1	BHLHE40	0.031462
BZLF1	TTLL6	0.13586
BZLF1	SLC4A1	0.13214
BZLF1	PTK7	0.081068
BZLF1	ADI1	0.12312
BZLF1	PCDHB5	0.044991
BZLF1	SPAG5	0.10218
BZLF1	TOB1	0.18838
BZLF1	BORA	0.12251
BZLF1	WDR12	0.13761
BZLF1	HM13	0.1162
BZLF1	JUNB	0.016659
BZLF1	SLC25A17	0.11318
BZLF1	VPS28	0.16772
BZLF1	URM1	0.19729
BZLF1	NUF2	0.13613
BZLF1	CABS1	0.048272
BZLF1	KLF10	0.022993

BZLF1	MAGEA2	0.1437
BZLF1	TRAK2	0.087439
BZLF1	CASP8AP2	0.11616
BZLF1	MZT2B	0.18902
BZLF1	FNTB	0.12079
BZLF1	RNF7	0.12373
BZLF1	HNRNPR	0.17062
BZLF1	CHFR	0.096217
BZLF1	CLN3	0.029644
BZLF1	CRTC1	0.12504
BZLF1	QKI	0.20459
BZLF1	RTN4R	0.16845
BZLF1	RBM15	0.19125
BZLF1	STXBP2	0.12856
BZLF1	KSR1	0.012
BZLF1	POLR1A	0.11383
BZLF1	EPCAM	0.18647
BZLF1	UMODL1	0.044094
BZLF1	GNL3	0.10959
BZLF1	VPS33B	0.13229
BZLF1	CLPX	0.11513
BZLF1	FAM117B	0.011366
BZLF1	UACA	0.11496
BZLF1	GLS	0.12227
BZLF1	PAK3	0.032077
BZLF1	C5orf30	0.17514
BZLF1	MYL4	0.048053
BZLF1	CD8B	0.14966
BZLF1	EIF4G2	0.23232
BZLF1	TOM1L1	0.15343
BZLF1	RABGGTB	0.21132
BZLF1	COLQ	0.10286
BZLF1	ARHGEF7	0.010452
BZLF1	NPAT	0.050218
BZLF1	ORAI1	0.14249
BZLF1	RAD21	0.1157
BZLF1	MAPT	0.027891
BZLF1	CD80	0.13545
BZLF1	PCDH11Y	0.049103
BZLF1	METTL1	0.19488
BZLF1	MRFAP1	0.19151
BZLF1	MMS19	0.10702
BZLF1	ITGB3	0.037238
BZLF1	INSR	0.016821
BZLF1	TLN2	0.060667
BZLF1	TRABD2B	0.092517
BZLF1	ASB9	0.17654
BZLF1	TRPC3	0.11704
BZLF1	CIZ1	0.13686
BZLF1	CHD8	0.061687
BZLF1	RPL11	0.11919
BZLF1	NLRP1	0.088273
BZLF1	NCBP1	0.096385
BZLF1	EDC3	0.20898
BZLF1	MAD2L2	0.13634
BZLF1	MGRN1	0.085311
BZLF1	CEP76	0.17919
BZLF1	WDR77	0.12065
BZLF1	CRKL	0.15741
BZLF1	USP7	0.070906
BZLF1	PPP2R2B	0.095513
BZLF1	BGLAP	0.042551
BZLF1	TIMP3	0.15469
BZLF1	RPL13	0.18101
BZLF1	ALDH7A1	0.16181
BZLF1	GDI2	0.13638
BZLF1	PTK6	0.062962
BZLF1	ZRSR2	0.14093

BZLF1	CD200R1	0.18472
BZLF1	UHRF1	0.045924
BZLF1	SULT2A1	0.16948
BZLF1	NEBL	0.19877
BZLF1	KLKB1	0.1795
BZLF1	TMF1	0.11851
BZLF1	C10orf91	0.01056
BZLF1	IL1R1	0.15649
BZLF1	LATS2	0.059235
BZLF1	CREB3L3	0.04606
BZLF1	NCOA7	0.14137
BZLF1	PCDHA5	0.046144
BZLF1	RABGAP1	0.14268
BZLF1	SMARCD1	0.14075
BZLF1	SHROOM2	0.097289
BZLF1	ZNF148	0.021477
BZLF1	CCDC28A	0.01109
BZLF1	PCDH11X	0.046215
BZLF1	KBTBD7	0.21685
BZLF1	ERCC4	0.053024
BZLF1	CYBA	0.047279
BZLF1	AKT1S1	0.10878
BZLF1	LTBR	0.14018
BZLF1	ARHGAP8	0.17099
BZLF1	MYOCD	0.032334
BZLF1	UHRF1BP1	0.20074
BZLF1	GH1	0.12133
BZLF1	CTH	0.050878
BZLF1	SHANK2	0.2068
BZLF1	CDH20	0.047672
BZLF1	SLA2	0.19918
BZLF1	CLTC	0.10958
BZLF1	XRCC6	0.012856
BZLF1	GPIHBP1	0.058796
BZLF1	FCGR1A	0.16029
BZLF1	SH3GL1	0.21076
BZLF1	ARVCF	0.17419
BZLF1	CBX2	0.11972
BZLF1	PLEKHA5	0.11694
BZLF1	EIF3F	0.2084
BZLF1	PPP3R2	0.057416
BZLF1	CIITA	0.09932
BZLF1	UPF2	0.21766
BZLF1	CACNB2	0.13762
BZLF1	SIN3B	0.12456
BZLF1	TNPO3	0.1377
BZLF1	CACNA1C	0.1038
BZLF1	SH3RF1	0.10732
BZLF1	TRPM6	0.14186
BZLF1	RAB5A	0.10938
BZLF1	ERBB2IP	0.11247
BZLF1	LPCAT2	0.013422
BZLF1	OPA1	0.068263
BZLF1	PPP2R5D	0.14162
BZLF1	BIN1	0.12069
BZLF1	BCL2L11	0.028729
BZLF1	EIF4ENIF1	0.12984
BZLF1	SYNRG	0.048553
BZLF1	GNAI1	0.052829
BZLF1	HIST2H3A	0.11263
BZLF1	NRIP1	0.047161
BZLF1	PSMB9	0.051916
BZLF1	RBMY1A1	0.23286
BZLF1	EEF1G	0.15571
BZLF1	COL7A1	0.16446
BZLF1	RAG1	0.05216
BZLF1	RRAD	0.16527
BZLF1	STX4	0.096741

BZLF1	LOXL4	0.1654
BZLF1	GFI1	0.070341
BZLF1	SIK3	0.085478
BZLF1	ROBO2	0.091844
BZLF1	CALCA	0.065787
BZLF1	NUP214	0.13835
BZLF1	NAA50	0.26017
BZLF1	STAU1	0.17532
BZLF1	CHAMP1	0.088498
BZLF1	ARHGEF15	0.15623
BZLF1	TPCN1	0.096928
BZLF1	MERTK	0.082782
BZLF1	MRPL20	0.14336
BZLF1	UBL4A	0.19506
BZLF1	CREBZF	0.048777
BZLF1	PELP1	0.176
BZLF1	STAT3	0.016677
BZLF1	ZNF488	0.10672
BZLF1	ACIN1	0.15293
BZLF1	BARD1	0.12278
BZLF1	PPFIA4	0.20894
BZLF1	CKB	0.164
BZLF1	TNFSF12	0.093423
BZLF1	TNS3	0.20333
BZLF1	FAM160A2	0.19812
BZLF1	KMT2C	0.15354
BZLF1	KLHL20	0.12436
BZLF1	RAD23A	0.13182
BZLF1	SMG9	0.16512
BZLF1	HPCAL4	0.065773
BZLF1	VPS4A	0.096279
BZLF1	COCH	0.16039
BZLF1	PSPC1	0.19189
BZLF1	STK40	0.096921
BZLF1	SRPX2	0.13
BZLF1	ORC4	0.10266
BZLF1	THBD	0.26772
BZLF1	GLYCTK	0.25018
BZLF1	ARHGEF4	0.06359
BZLF1	SMAD5	0.020683
BZLF1	KIAA0226	0.17017
BZLF1	DCHS1	0.047639
BZLF1	CREM	0.043201
BZLF1	ZFR	0.19345
BZLF1	RTP4	0.1979
BZLF1	FSHR	0.095864
BZLF1	FANCM	0.113
BZLF1	EEF1D	0.14455
BZLF1	TRADD	0.059513
BZLF1	PYGO1	0.12817
BZLF1	DHX8	0.17067
BZLF1	MTERF	0.13717
BZLF1	ATG13	0.10064
BZLF1	BRAF	0.11813
BZLF1	NPBWR2	0.16562
BZLF1	ARPC2	0.13335
BZLF1	ARFGEF2	0.080639
BZLF1	TTC19	0.16698
BZLF1	BSG	0.10256
BZLF1	MT2A	0.1409
BZLF1	AIG1	0.2221
BZLF1	ORM1	0.21119
BZLF1	HSD17B10	0.089974
BZLF1	RBM5	0.13809
BZLF1	BOP1	0.1157
BZLF1	NOX1	0.059224
BZLF1	GYG1	0.16456
BZLF1	DSC2	0.046371

BZLF1	CAPS	0.028087
BZLF1	CLSPN	0.14509
BZLF1	MLST8	0.16388
BZLF1	WTIP	0.10433
BZLF1	SPTB	0.10342
BZLF1	IL1R2	0.22959
BZLF1	KLF13	0.088234
BZLF1	MSN	0.055154
BZLF1	SPDL1	0.10429
BZLF1	UBE2D4	0.10856
BZLF1	SMARCE1	0.099894
BZLF1	PIGA	0.16429
BZLF1	RPS26	0.11377
BZLF1	MEN1	0.030423
BZLF1	GNMT	0.17685
BZLF1	BRE	0.14787
BZLF1	ZBTB14	0.11335
BZLF1	SCAMP1	0.17327
BZLF1	SEC23A	0.090705
BZLF1	RND1	0.086401
BZLF1	BCKDHB	0.1814
BZLF1	PDLIM7	0.13007
BZLF1	SSH1	0.010827
BZLF1	RNMT	0.25848
BZLF1	TNFRSF21	0.19282
BZLF1	TTF2	0.077372
BZLF1	TOPORS	0.055411
BZLF1	DDX47	0.17587
BZLF1	FREM2	0.026681
BZLF1	GOLM1	0.2087
BZLF1	GNAI2	0.041967
BZLF1	DDX42	0.19117
BZLF1	GAPDHS	0.13522
BZLF1	CDKN1C	0.15718
BZLF1	GRIA1	0.081427
BZLF1	ANKLE2	0.14497
BZLF1	NUP153	0.094869
BZLF1	TOMM22	0.11827
BZLF1	VPS41	0.11736
BZLF1	MAGOH	0.19352
BZLF1	SKI	0.023829
BZLF1	SRGN	0.11147
BZLF1	CEP41	0.14199
BZLF1	RAC1	0.024267
BZLF1	INTS9	0.19122
BZLF1	IPO5	0.14616
BZLF1	VDAC1	0.082961
BZLF1	PRMT5	0.12288
BZLF1	MUC16	0.16768
BZLF1	TNFAIP1	0.12536
BZLF1	CRNN	0.05109
BZLF1	ING4	0.11102
BZLF1	PECAM1	0.14305
BZLF1	EML1	0.43734
BZLF1	GTF2F2	0.075393
BZLF1	CEP170	0.18849
BZLF1	C19orf68	0.23617
BZLF1	NOL3	0.1708
BZLF1	ZWILCH	0.16697
BZLF1	VAV2	0.039493
BZLF1	KIF24	0.16217
BZLF1	MAGED1	0.12504
BZLF1	PTGER4	0.076438
BZLF1	RPRD1B	0.14032
BZLF1	CWC15	0.25271
BZLF1	CALCRL	0.056713
BZLF1	RBPMS	0.17959
BZLF1	DES	0.10938

BZLF1	WDR4	0.17502
BZLF1	PSMC1	0.07467
BZLF1	USP5	0.10179
BZLF1	CITED1	0.020004
BZLF1	HAT1	0.16618
BZLF1	SNCAIP	0.14989
BZLF1	FZD3	0.092917
BZLF1	LIN28A	0.10405
BZLF1	MPDZ	0.16146
BZLF1	NGFRAP1	0.13111
BZLF1	ARID2	0.11736
BZLF1	PGS1	0.024981
BZLF1	TLDC1	0.23617
BZLF1	COMMD3	0.23617
BZLF1	SMAD7	0.022098
BZLF1	SERPINA1	0.15807
BZLF1	NCK1	0.064614
BZLF1	GABBR1	0.051897
BZLF1	MPG	0.040457
BZLF1	CRTAC1	0.067398
BZLF1	SCP2	0.06849
BZLF1	TMEM173	0.012927
BZLF1	MAD2L1	0.091138
BZLF1	PRRC2B	0.23617
BZLF1	CLN6	0.16228
BZLF1	KCNJ3	0.1751
BZLF1	BYSL	0.13679
BZLF1	ERN1	0.021465
BZLF1	SPRTN	0.14121
BZLF1	MYD88	0.04083
BZLF1	LSM5	0.17174
BZLF1	CTC1	0.11175
BZLF1	FBXW8	0.21543
BZLF1	SPPL2A	0.11631
BZLF1	PLS1	0.050763
BZLF1	SYVN1	0.07735
BZLF1	CACYBP	0.16107
BZLF1	INS	0.026801
BZLF1	IRAK4	0.080752
BZLF1	EPS15	0.23004
BZLF1	PLS3	0.054177
BZLF1	MYO5B	0.19086
BZLF1	WWC1	0.11547
BZLF1	HUS1	0.13989
BZLF1	PCSK2	0.23599
BZLF1	CRHR1	0.054126
BZLF1	APLF	0.11587
BZLF1	YY1	0.044096
BZLF1	NKRF	0.16145
BZLF1	ZNF335	0.11682
BZLF1	AURKB	0.030324
BZLF1	RRAGC	0.073802
BZLF1	LZTS1	0.033632
BZLF1	CD48	0.13612
BZLF1	VWCE	0.055781
BZLF1	CLEC3B	0.047698
BZLF1	PRMT8	0.19409
BZLF1	INPP5B	0.13776
BZLF1	ARHGEF16	0.057568
BZLF1	BST2	0.091799
BZLF1	NHLH2	0.11207
BZLF1	PEX11G	0.18025
BZLF1	OGDHL	0.20912
BZLF1	FKBP8	0.09532
BZLF1	DISC1	0.10185
BZLF1	IL33	0.14143
BZLF1	PUM2	0.25814
BZLF1	ESPL1	0.097629

BZLF1	PCGF1	0.12142
BZLF1	CDH8	0.046388
BZLF1	LTC4S	0.07289
BZLF1	AP3S1	0.25604
BZLF1	UHMK1	0.14142
BZLF1	LAT2	0.18509
BZLF1	BUB3	0.10216
BZLF1	RBMY1B	0.2213
BZLF1	SPRR3	0.07748
BZLF1	SDF4	0.31826
BZLF1	KIF14	0.12565
BZLF1	AFF4	0.058017
BZLF1	LEPR	0.18271
BZLF1	ANKHD1	0.26087
BZLF1	MGP	0.042839
BZLF1	BCKDHA	0.19779
BZLF1	TRIM33	0.053885
BZLF1	HBB	0.13165
BZLF1	KIF5B	0.11278
BZLF1	HEG1	0.02884
BZLF1	PRKCQ	0.018271
BZLF1	PHIP	0.12881
BZLF1	TAF11	0.14822
BZLF1	TRPC7	0.11974
BZLF1	LAMTOR2	0.14188
BZLF1	MAZ	0.15249
BZLF1	HIST1H1E	0.1247
BZLF1	WSB1	0.21277
BZLF1	CDC45	0.13433
BZLF1	YAF2	0.11305
BZLF1	MYH9	0.052395
BZLF1	EID3	0.14732
BZLF1	SPACA3	0.18609
BZLF1	PTPLB	0.1254
BZLF1	INSIG2	0.090364
BZLF1	CDCA8	0.11643
BZLF1	STAT1	0.053819
BZLF1	PRKAB1	0.040124
BZLF1	GJA1	0.027793
BZLF1	PSEN2	0.015079
BZLF1	FAT4	0.42347
BZLF1	DTNBP1	0.11842
BZLF1	CUL9	0.13054
BZLF1	PNMA2	0.19264
BZLF1	WWP1	0.087572
BZLF1	PLIN3	0.15168
BZLF1	KCNIP4	0.053118
BZLF1	USP34	0.16542
BZLF1	ZNHIT6	0.16059
BZLF1	VPS18	0.1277
BZLF1	ARHGAP4	0.12066
BZLF1	IFI16	0.046537
BZLF1	PPEF1	0.33442
BZLF1	ZW10	0.066188
BZLF1	GRIN3A	0.075269
BZLF1	CXorf67	0.011202
BZLF1	NPW	0.18374
BZLF1	MAPK11	0.050702
BZLF1	A2M	0.11058
BZLF1	DCTN1	0.13059
BZLF1	FTSJ3	0.24554
BZLF1	ETV1	0.04191
BZLF1	PRKAA2	0.015139
BZLF1	NELL2	0.33181
BZLF1	CALB2	0.071377
BZLF1	PAICS	0.11805
BZLF1	DDX60	0.14981
BZLF1	CLTB	0.17156

BZLF1	PCDHA7	0.046144
BZLF1	CHD4	0.029726
BZLF1	ZC3H10	0.18328
BZLF1	ARRB2	0.053594
BZLF1	PRDX1	0.1491
BZLF1	RHOG	0.085928
BZLF1	TTC3	0.095445
BZLF1	SPON1	0.21435
BZLF1	IRAK3	0.049552
BZLF1	CYFIP1	0.11201
BZLF1	NR2F1	0.028826
BZLF1	ULK2	0.17972
BZLF1	SRSF3	0.19339
BZLF1	PDZK1	0.19916
BZLF1	MCC	0.1533
BZLF1	KAT2B	0.02198
BZLF1	CROCC	0.078783
BZLF1	LAMTOR3	0.16209
BZLF1	ARRB1	0.038971
BZLF1	ELOVL4	0.14319
BZLF1	FAM188B2	0.011596
BZLF1	UBE3A	0.076459
BZLF1	TCF4	0.037858
BZLF1	GTF3C1	0.13129
BZLF1	SMC1A	0.069639
BZLF1	DMAP1	0.12602
BZLF1	RPS27	0.077055
BZLF1	CHEK2	0.022709
BZLF1	STX1A	0.086168
BZLF1	DRD2	0.013865
BZLF1	APH1A	0.082296
BZLF1	GBAS	0.20023
BZLF1	BAZ1B	0.10656
BZLF1	AKIP1	0.19741
BZLF1	PCDHGC5	0.049103
BZLF1	KAT2A	0.10624
BZLF1	COL4A3BP	0.042123
BZLF1	TRPC4	0.11043
BZLF1	KIF5C	0.13094
BZLF1	FZD10	0.085768
BZLF1	PIN1	0.12336
BZLF1	TLX3	0.048898
BZLF1	SUV420H2	0.20557
BZLF1	PCDHGA7	0.049103
BZLF1	HECTD1	0.13827
BZLF1	NME1	0.093004
BZLF1	NOP58	0.1629
BZLF1	MAP1LC3C	0.26069
BZLF1	RFC3	0.096054
BZLF1	ATP6V1E1	0.13846
BZLF1	ATP11B	0.066793
BZLF1	RNF14	0.071152
BZLF1	TARBP2	0.11607
BZLF1	SFR1	0.2118
BZLF1	TAB1	0.072516
BZLF1	RHOA	0.016297
BZLF1	KCNH2	0.081361
BZLF1	KRT20	0.14702
BZLF1	ZNF646	0.11682
BZLF1	MAP4K4	0.1084
BZLF1	DCAF11	0.2289
BZLF1	THBS1	0.043144
BZLF1	RUNX3	0.022209
BZLF1	LMTK2	0.096588
BZLF1	GADD45GIP1	0.17476
BZLF1	DIS3	0.13574
BZLF1	ARR3	0.15678
BZLF1	ABCD3	0.12841

BZLF1	UBR2	0.091562
BZLF1	CMKLR1	0.12428
BZLF1	STK25	0.086817
BZLF1	FANCF	0.19455
BZLF1	SEMG1	0.10338
BZLF1	ICAM3	0.14268
BZLF1	MRPL49	0.18495
BZLF1	ASS1	0.016751
BZLF1	EIF3I	0.23414
BZLF1	ARL2BP	0.17601
BZLF1	UBTF	0.12379
BZLF1	FGF2	0.020041
BZLF1	PFN1	0.099014
BZLF1	DLG3	0.091963
BZLF1	RBCK1	0.024615
BZLF1	DSG2	0.036954
BZLF1	MAP3K12	0.031208
BZLF1	CCNE1	0.039485
BZLF1	RHOC	0.058112
BZLF1	ADRA2B	0.08559
BZLF1	ATXN3	0.083814
BZLF1	EPB41L3	0.083063
BZLF1	CEBPG	0.021538
BZLF1	DEDD2	0.10409
BZLF1	MGMT	0.032382
BZLF1	GIT2	0.10668
BZLF1	SNX33	0.22209
BZLF1	STK24	0.057331
BZLF1	PCDHB2	0.042302
BZLF1	ATG101	0.17013
BZLF1	MIF	0.07804
BZLF1	ISG20L2	0.13162
BZLF1	BICD1	0.13647
BZLF1	AP2M1	0.14221
BZLF1	ING2	0.069719
BZLF1	PIK3CG	0.059705
BZLF1	ADORA1	0.029696
BZLF1	CDC42EP2	0.13195
BZLF1	SF3A1	0.21415
BZLF1	INTS3	0.18835
BZLF1	DSG4	0.037964
BZLF1	TCTEX1D4	0.23617
BZLF1	TMCC2	0.2221
BZLF1	PLG	0.0801
BZLF1	TEAD3	0.0382
BZLF1	NOS2	0.057895
BZLF1	PRMT1	0.21422
BZLF1	USP10	0.11444
BZLF1	LASP1	0.1328
BZLF1	MT-ND1	0.17456
BZLF1	RASSF5	0.17225
BZLF1	CRB2	0.051151
BZLF1	DYNLT1	0.094937
BZLF1	ZMIZ2	0.11255
BZLF1	ALDOB	0.028266
BZLF1	S100Z	0.057416
BZLF1	MED26	0.10938
BZLF1	LRP5	0.072028
BZLF1	HSD17B12	0.093194
BZLF1	DGKQ	0.14026
BZLF1	CRP	0.14472
BZLF1	ARIH2	0.095803
BZLF1	DHX36	0.10453
BZLF1	C1QBP	0.13642
BZLF1	DDOST	0.13831
BZLF1	AGR3	0.27616
BZLF1	AP1M1	0.1858
BZLF1	PUF60	0.13202

BZLF1	GMCL1P1	0.20688
BZLF1	STRA13	0.14008
BZLF1	SNX8	0.18087
BZLF1	MAPK6	0.052424
BZLF1	RPL23A	0.12132
BZLF1	SPOP	0.18939
BZLF1	PRKCZ	0.024565
BZLF1	HDDC2	0.15088
BZLF1	FANCI	0.17662
BZLF1	CYCS	0.044211
BZLF1	SETDB1	0.16056
BZLF1	SEC22B	0.1294
BZLF1	ATP7A	0.01679
BZLF1	AGR2	0.25227
BZLF1	UQCRC2	0.06204
BZLF1	FHL2	0.033304
BZLF1	ADM	0.061821
BZLF1	TPPP	0.32177
BZLF1	ZNF746	0.084775
BZLF1	PRPF4	0.17153
BZLF1	DBNDD2	0.2221
BZLF1	BMP10	0.086023
BZLF1	TBXA2R	0.085025
BZLF1	FYB	0.1122
BZLF1	SYT4	0.10216
BZLF1	IL17RE	0.20889
BZLF1	CENPT	0.11955
BZLF1	RAD51D	0.14363
BZLF1	SYT11	0.4006
BZLF1	CD5	0.15473
BZLF1	SH2D4A	0.17642
BZLF1	ICAM1	0.050563
BZLF1	GZMB	0.15015
BZLF1	GGA2	0.14873
BZLF1	MAD1L1	0.091997
BZLF1	DCUN1D1	0.22213
BZLF1	LPAR1	0.04834
BZLF1	MOB3B	0.20707
BZLF1	MSH6	0.035379
BZLF1	SNRNP200	0.11806
BZLF1	PTCH1	0.030809
BZLF1	EIF2B1	0.10731
BZLF1	PDE3B	0.068697
BZLF1	DRD3	0.043437
BZLF1	CTNNA3	0.08388
BZLF1	IFITM1	0.12915
BZLF1	SWSAP1	0.19415
BZLF1	DDX17	0.13003
BZLF1	RECQL	0.088238
BZLF1	BCS1L	0.13348
BZLF1	SH3PXD2A	0.18389
BZLF1	SFPQ	0.17049
BZLF1	SYK	0.010919
BZLF1	PARPBP	0.17863
BZLF1	FBXL15	0.18367
BZLF1	FANCA	0.17177
BZLF1	EIF2B3	0.15911
BZLF1	TMEM11	0.17999
BZLF1	KDM1A	0.012583
BZLF1	PCDHAC2	0.046144
BZLF1	RBMS1	0.19727
BZLF1	ATP6V0D1	0.11977
BZLF1	TPD52L1	0.16312
BZLF1	WDFY3	0.17741
BZLF1	PSMA4	0.050508
BZLF1	HSP90B1	0.22635
BZLF1	NR2C1	0.040268
BZLF1	PCDH7	0.04754

BZLF1	PIAS1	0.072693
BZLF1	HOXB7	0.041739
BZLF1	TNFSF13B	0.087552
BZLF1	MPP5	0.17546
BZLF1	TRIM2	0.14102
BZLF1	DDAH2	0.14936
BZLF1	ATPAF2	0.21545
BZLF1	MACF1	0.25398
BZLF1	AIFM1	0.083677
BZLF1	CKAP5	0.12742
BZLF1	TNFRSF12A	0.13609
BZLF1	POLR3F	0.10711
BZLF1	S100A3	0.03514
BZLF1	MEIS1	0.033964
BZLF1	WWOX	0.12977
BZLF1	ADORA2A	0.028486
BZLF1	SOS1	0.051464
BZLF1	APOD	0.091931
BZLF1	TXNL4A	0.1799
BZLF1	EVL	0.14179
BZLF1	BOK	0.12311
BZLF1	ALG2	0.13014
BZLF1	GLMN	0.19251
BZLF1	MAGEC2	0.1819
BZLF1	UVSSA	0.2099
BZLF1	PCM1	0.096647
BZLF1	TPSAB1	0.22236
BZLF1	TXNRD2	0.21979
BZLF1	FGF21	0.18756
BZLF1	PCGF3	0.13309
BZLF1	MSR1	0.19718
BZLF1	DUOX2	0.097959
BZLF1	RTN1	0.17512
BZLF1	LARS	0.026564
BZLF1	RBM14	0.10031
BZLF1	OSBPL6	0.15993
BZLF1	STBD1	0.19622
BZLF1	CASQ2	0.049585
BZLF1	MCTP2	0.052239
BZLF1	PRKG1	0.067618
BZLF1	TRPC4AP	0.20764
BZLF1	LPXN	0.098116
BZLF1	UBE2A	0.11309
BZLF1	EPHA7	0.091076
BZLF1	SNTB2	0.17622
BZLF1	CX3CL1	0.056084
BZLF1	PCDHGB4	0.047639
BZLF1	AP5Z1	0.17632
BZLF1	PPIE	0.19385
BZLF1	KLRK1	0.11688
BZLF1	CAST	0.24285
BZLF1	TBC1D25	0.22903
BZLF1	DNAJC7	0.18674
BZLF1	GCH1	0.12268
BZLF1	PTGES3	0.09678
BZLF1	ANKRD1	0.068505
BZLF1	VPS11	0.19492
BZLF1	POLE3	0.13962
BZLF1	SLC9A3	0.13955
BZLF1	OGN	0.15379
BZLF1	HNRNPU	0.12641
BZLF1	OPRD1	0.10521
BZLF1	MDM2	0.036048
BZLF1	NKD2	0.32562
BZLF1	LSAMP	0.20014
BZLF1	PTPN23	0.06107
BZLF1	ADAMTSL2	0.1415
BZLF1	FLNC	0.18269

BZLF1	MAGI3	0.11473
BZLF1	SLAMF1	0.12404
BZLF1	IFT57	0.1547
BZLF1	ADAM23	0.10568
BZLF1	AP5B1	0.21295
BZLF1	TRIM29	0.0539
BZLF1	EIF2B5	0.15502
BZLF1	DENR	0.25383
BZLF1	ZNF350	0.12328
BZLF1	TMEM132D	0.20263
BZLF1	MAPK8IP1	0.054444
BZLF1	FOXN3	0.010935
BZLF1	PTPN4	0.046698
BZLF1	ATP2B2	0.065243
BZLF1	BTG2	0.10878
BZLF1	PEX3	0.12907
BZLF1	GHITM	0.19063
BZLF1	INCENP	0.10511
BZLF1	TBC1D9	0.029191
BZLF1	DOT1L	0.18062
BZLF1	JMJD1C	0.19571
BZLF1	GJD3	0.19105
BZLF1	FNBP1L	0.21948
BZLF1	ITGAV	0.054988
BZLF1	TXLNB	0.22209
BZLF1	SEC13	0.13969
BZLF1	CDH10	0.045044
BZLF1	CDKN2AIP	0.17646
BZLF1	KEAP1	0.14212
BZLF1	EPHA10	0.15083
BZLF1	CNOT6	0.14316
BZLF1	LCK	0.031351
BZLF1	HIPK2	0.027645
BZLF1	PSMC4	0.068776
BZLF1	RAD50	0.039973
BZLF1	FAT1	0.25883
BZLF1	REV1	0.097388
BZLF1	BAZ1A	0.097917
BZLF1	OCRL	0.09385
BZLF1	DYRK1B	0.07899
BZLF1	ABCF1	0.13441
BZLF1	HDAC3	0.023716
BZLF1	FBLN7	0.048621
BZLF1	NXF3	0.23738
BZLF1	H2AFY	0.10747
BZLF1	CCNH	0.01146
BZLF1	NGLY1	0.16994
BZLF1	EDAR	0.13199
BZLF1	MTCH1	0.17249
BZLF1	ERCC8	0.14452
BZLF1	CRELD1	0.054352
BZLF1	ISOC2	0.14322
BZLF1	TGOLN2	0.16404
BZLF1	HABP4	0.1317
BZLF1	XAB2	0.15793
BZLF1	S100A11	0.35883
BZLF1	ITGA5	0.080739
BZLF1	GDF7	0.1223
BZLF1	GPS2	0.18149
BZLF1	SRSF5	0.16667
BZLF1	USH1G	0.14601
BZLF1	SORBS1	0.072269
BZLF1	CHMP1B	0.16737
BZLF1	HFE2	0.15372
BZLF1	IRF3	0.015913
BZLF1	EFCAB6	0.02979
BZLF1	RBBP6	0.10354
BZLF1	MAP3K3	0.082993

BZLF1	TIFAB	0.010714
BZLF1	ESR2	0.029175
BZLF1	TPP1	0.10757
BZLF1	NCAPG	0.16627
BZLF1	NUFIP2	0.25956
BZLF1	ECSIT	0.20436
BZLF1	LRRFIP2	0.21109
BZLF1	AIRE	0.043948
BZLF1	XPO4	0.18424
BZLF1	INIP	0.20153
BZLF1	NKD1	0.33096
BZLF1	NR1H2	0.018787
BZLF1	PPP1R9B	0.092244
BZLF1	PLEKHM2	0.12216
BZLF1	DPM3	0.11981
BZLF1	CYBB	0.086169
BZLF1	CEP70	0.15198
BZLF1	DERL2	0.14493
BZLF1	ACACA	0.10326
BZLF1	SPTBN4	0.042964
BZLF1	STAP1	0.045407
BZLF1	EDA2R	0.13273
BZLF1	PPP6C	0.12778
BZLF1	TRIAP1	0.17993
BZLF1	FASTK	0.15588
BZLF1	RBFOX2	0.13198
BZLF1	GCK	0.054981
BZLF1	TPX2	0.047643
BZLF1	RPS10	0.10366
BZLF1	CD2AP	0.16147
BZLF1	RAP2A	0.095508
BZLF1	CD1A	0.179
BZLF1	SPATA18	0.11933
BZLF1	CBX8	0.14131
BZLF1	PAGR1	0.19426
BZLF1	RNF146	0.10301
BZLF1	IGHG1	0.14848
BZLF1	KIF18A	0.073252
BZLF1	GORAB	0.18683
BZLF1	GABPB1	0.02737
BZLF1	GREM1	0.030986
BZLF1	TSKS	0.10834
BZLF1	INTS12	0.14968
BZLF1	UBE2N	0.050619
BZLF1	MAGEB18	0.23617
BZLF1	MED31	0.13053
BZLF1	MAT2B	0.098369
BZLF1	GEMIN4	0.13963
BZLF1	HMCN1	0.08892
BZLF1	SLC9A3R2	0.095246
BZLF1	KNSTRN	0.11963
BZLF1	ODF2	0.057806
BZLF1	SERPINE1	0.036306
BZLF1	PCBP2	0.13819
BZLF1	SPC25	0.13614
BZLF1	RBM25	0.14991
BZLF1	DACH1	0.019437
BZLF1	PKP1	0.16864
BZLF1	DNAJB5	0.19234
BZLF1	EIF1AY	0.33521
BZLF1	FNIP2	0.17082
BZLF1	DEAF1	0.096416
BZLF1	ARID4B	0.13642
BZLF1	EMR2	0.023368
BZLF1	TREML1	0.18402
BZLF1	MOV10	0.16114
BZLF1	MAGI1	0.13956
BZLF1	PTGIS	0.029036

BZLF1	ARL4A	0.14688
BZLF1	BPTF	0.071947
BZLF1	STMN2	0.14139
BZLF1	CAPS2	0.057416
BZLF1	ARHGAP32	0.077244
BZLF1	MTDH	0.078209
BZLF1	RP9	0.14544
BZLF1	PRRC2A	0.19741
BZLF1	MKKS	0.058392
BZLF1	GMNN	0.12828
BZLF1	PDZD11	0.20714
BZLF1	METTL17	0.22041
BZLF1	MYLPF	0.04461
BZLF1	PSMD10	0.041095
BZLF1	SDHAF2	0.20375
BZLF1	ST13	0.13383
BZLF1	APOA1	0.031437
BZLF1	PSMA5	0.053906
BZLF1	PPP2R3B	0.32512
BZLF1	PEG10	0.12454
BZLF1	INO80	0.089338
BZLF1	CTNNBIP1	0.12253
BZLF1	CENPH	0.092655
BZLF1	HDAC9	0.035524
BZLF1	HEY1	0.022087
BZLF1	ACTN4	0.19844
BZLF1	FBXO46	0.23617
BZLF1	KIAA0141	0.21545
BZLF1	DTX3L	0.13333
BZLF1	EHD1	0.20874
BZLF1	ATL1	0.065113
BZLF1	KIR2DL1	0.18519
BZLF1	RAB25	0.21238
BZLF1	TIMM44	0.13118
BZLF1	PARD6B	0.12337
BZLF1	GTF2E1	0.11091
BZLF1	RAN	0.07453
BZLF1	KIAA0408	0.23617
BZLF1	LILRA4	0.2221
BZLF1	EEA1	0.14037
BZLF1	SUN2	0.096513
BZLF1	SEPT8	0.18395
BZLF1	PINK1	0.018681
BZLF1	FANCG	0.13608
BZLF1	CTU2	0.19692
BZLF1	OXA1L	0.13831
BZLF1	INTS10	0.18099
BZLF1	LPL	0.03922
BZLF1	PKMYT1	0.038414
BZLF1	ERCC6	0.047704
BZLF1	PARVA	0.088598
BZLF1	VCAN	0.031294
BZLF1	CDR2	0.19146
BZLF1	SHFM1	0.215
BZLF1	KRIT1	0.14297
BZLF1	LINC00523	0.011602
BZLF1	RABAC1	0.19867
BZLF1	RUNX2	0.017038
BZLF1	AMFR	0.050515
BZLF1	NEU4	0.10387
BZLF1	INTS8	0.19122
BZLF1	CNOT6L	0.13309
BZLF1	RPS19BP1	0.18683
BZLF1	COMMD1	0.12622
BZLF1	SEPT2	0.067329
BZLF1	RBM4	0.083257
BZLF1	KPNA3	0.13221
BZLF1	ZNRF1	0.16532

BZLF1	GRB7	0.065001
BZLF1	PLCB1	0.012255
BZLF1	ZSCAN21	0.039621
BZLF1	TESK1	0.11218
BZLF1	CDC42BPG	0.01278
BZLF1	CABP4	0.047999
BZLF1	GEMIN6	0.15508
BZLF1	MAPK12	0.048853
BZLF1	MMP2	0.051296
BZLF1	INHA	0.073106
BZLF1	CSN2	0.073562
BZLF1	POLE4	0.14384
BZLF1	EIF2B2	0.15034
BZLF1	RPTN	0.080121
BZLF1	EXOSC1	0.18626
BZLF1	DSP	0.073406
BZLF1	RGS19	0.099721
BZLF1	ZFYVE20	0.10452
BZLF1	UBE2G2	0.10891
BZLF1	RMI1	0.19748
BZLF1	AIP	0.05171
BZLF1	S100A13	0.27966
BZLF1	PIK3R6	0.096114
BZLF1	STUB1	0.080809
BZLF1	MEA1	0.20275
BZLF1	MELK	0.21368
BZLF1	EXO1	0.066655
BZLF1	GEM	0.10821
BZLF1	NEK1	0.083122
BZLF1	HPGDS	0.058156
BZLF1	MED18	0.1292
BZLF1	BNIP3	0.031415
BZLF1	INHBB	0.13552
BZLF1	HSP90AB1	0.086851
BZLF1	CORO1B	0.19517
BZLF1	HTRA3	0.23477
BZLF1	PSMB1	0.053521
BZLF1	S100A1	0.30929
BZLF1	ARL14EPL	0.011194
BZLF1	EIF3H	0.22706
BZLF1	TMED9	0.14564
BZLF1	GPATCH8	0.16351
BZLF1	PRMT6	0.13282
BZLF1	MYL7	0.070814
BZLF1	SNRPC	0.18765
BZLF1	SUMO1	0.069538
BZLF1	PPIA	0.12108
BZLF1	SUMO2	0.18592
BZLF1	USP1	0.13885
BZLF1	ARHGAP24	0.084499
BZLF1	UTRN	0.24635
BZLF1	TRAF3IP3	0.2221
BZLF1	STAM2	0.10942
BZLF1	EGR2	0.045631
BZLF1	UBE2D2	0.10108
BZLF1	EGFL8	0.064418
BZLF1	RAD23B	0.12148
BZLF1	HHIP	0.052619
BZLF1	FANCE	0.20058
BZLF1	GNS	0.03736
BZLF1	PCGF5	0.12946
BZLF1	S100A2	0.055733
BZLF1	POLD4	0.10381
BZLF1	RPL15	0.18663
BZLF1	PALM	0.099337
BZLF1	IDE	0.02048
BZLF1	AP2A2	0.13106
BZLF1	ASH2L	0.10621

BZLF1	DOHH	0.11899
BZLF1	IGF1	0.019573
BZLF1	SLC34A1	0.18581
BZLF1	TRAF3IP2	0.19174
BZLF1	SCUBE2	0.055781
BZLF1	TBC1D2B	0.086597
BZLF1	MCM7	0.080805
BZLF1	TOP2A	0.048773
BZLF1	SGOL2	0.12957
BZLF1	PIK3CA	0.07576
BZLF1	MYL9	0.035984
BZLF1	NOP56	0.21194
BZLF1	KIAA0101	0.17641
BZLF1	TNFRSF1B	0.14433
BZLF1	BAHD1	0.12878
BZLF1	E4F1	0.017498
BZLF1	SPRY2	0.031635
BZLF1	ENG	0.043155
BZLF1	MYO18A	0.13702
BZLF1	ACP1	0.029979
BZLF1	AQP1	0.047509
BZLF1	MAPKAPK3	0.043879
BZLF1	ELN	0.15732
BZLF1	SWI5	0.2118
BZLF1	LTF	0.10409
BZLF1	PPM1B	0.098826
BZLF1	TBC1D10A	0.17396
BZLF1	EFHD1	0.052418
BZLF1	MAP2K3	0.033018
BZLF1	SMC2	0.1149
BZLF1	ZC3HAV1	0.16032
BZLF1	SAMM50	0.12183
BZLF1	FASLG	0.061663
BZLF1	VWF	0.11799
BZLF1	TMBIM4	0.15284
BZLF1	SAP30BP	0.15119
BZLF1	ZFPM2	0.056526
BZLF1	CIB4	0.057416
BZLF1	TSG101	0.066347
BZLF1	CRADD	0.11693
BZLF1	SPTBN1	0.064958
BZLF1	EDEM2	0.015916
BZLF1	KANK1	0.1093
BZLF1	NUP37	0.12175
BZLF1	FLII	0.13684
BZLF1	EMD	0.11085
BZLF1	ECHS1	0.094264
BZLF1	NTRK1	0.047179
BZLF1	RBM17	0.22321
BZLF1	THBS3	0.032401
BZLF1	CUBN	0.023641
BZLF1	PSPH	0.01903
BZLF1	S100A14	0.030763
BZLF1	STAT6	0.13289
BZLF1	MPZL2	0.17972
BZLF1	INVS	0.14488
BZLF1	DTNB	0.014414
BZLF1	USP4	0.13066
BZLF1	RBPJ	0.01544
BZLF1	ATG3	0.1335
BZLF1	PRRG3	0.051176
BZLF1	ULK1	0.04613
BZLF1	SPOCK2	0.068046
BZLF1	HYAL2	0.041115
BZLF1	STAMBP	0.094116
BZLF1	CTLA4	0.17774
BZLF1	SLIT3	0.02273
BZLF1	ARPC5	0.1449

BZLF1	NELFE	0.16268
BZLF1	CLEC1B	0.19113
BZLF1	ATRAID	0.12393
BZLF1	FBXO4	0.1598
BZLF1	HNRNPL	0.15251
BZLF1	TLE2	0.13607
BZLF1	PPP2R2A	0.13685
BZLF1	AKAP5	0.029796
BZLF1	CALB1	0.28624
BZLF1	DAZAP2	0.19146
BZLF1	CPSF2	0.19562
BZLF1	MAP4K1	0.10042
BZLF1	PCGF6	0.033354
BZLF1	PEX5	0.084851
BZLF1	RYK	0.1202
BZLF1	RASSF6	0.21513
BZLF1	NDEL1	0.08117
BZLF1	HHLA3	0.23617
BZLF1	NPHS2	0.18296
BZLF1	DIP2A	0.0983
BZLF1	GAN	0.19375
BZLF1	PDIA4	0.081668
BZLF1	MIER1	0.12297
BZLF1	MAP3K11	0.068111
BZLF1	BAZ2A	0.10947
BZLF1	CAPN11	0.0555
BZLF1	SFTPC	0.13931
BZLF1	UFD1L	0.16981
BZLF1	LIN7A	0.13518
BZLF1	SETMAR	0.12803
BZLF1	VAV3	0.038173
BZLF1	MASP2	0.37304
BZLF1	BATF2	0.064659
BZLF1	NUDT21	0.18414
BZLF1	PCDHA9	0.049103
BZLF1	PARK7	0.14322
BZLF1	RP2	0.095728
BZLF1	FKBP1A	0.10031
BZLF1	TAF6L	0.072213
BZLF1	SLC25A23	0.052515
BZLF1	TRAF7	0.12647
BZLF1	BCL2L10	0.1423
BZLF1	RCN2	0.053649
BZLF1	PRCP	0.21845
BZLF1	SUCLA2	0.064752
BZLF1	TAOK3	0.11262
BZLF1	SSTR2	0.088519
BZLF1	PPP4C	0.092857
BZLF1	HSPA1A	0.05326
BZLF1	TAP1	0.065487
BZLF1	ATG9A	0.21611
BZLF1	CALML4	0.057416
BZLF1	NDUFAF1	0.19276
BZLF1	COG5	0.12066
BZLF1	MAP3K5	0.017563
BZLF1	FRA10AC1	0.20355
BZLF1	PES1	0.11734
BZLF1	CCM2	0.19718
BZLF1	BBIP1	0.19991
BZLF1	TRIM39	0.10402
BZLF1	ABI1	0.073412
BZLF1	PTBP1	0.19228
BZLF1	SOST	0.10274
BZLF1	MAGEA3	0.23617
BZLF1	ZNF224	0.10955
BZLF1	RAMP2	0.067194
BZLF1	TAF13	0.032553
BZLF1	SLAIN1	0.010909

BZLF1	CAPSL	0.055771
BZLF1	RRAS2	0.10329
BZLF1	RAB4A	0.094997
BZLF1	FGF19	0.18094
BZLF1	DDN	0.15594
BZLF1	RAB5B	0.13085
BZLF1	RNF32	0.16192
BZLF1	ARL14	0.25813
BZLF1	LRWD1	0.12354
BZLF1	TXNDC9	0.21642
BZLF1	ARL6	0.068961
BZLF1	EIF3C	0.23414
BZLF1	EIF3M	0.25213
BZLF1	ZNF326	0.17128
BZLF1	MYOZ3	0.22823
BZLF1	KLHL2	0.2174
BZLF1	DTX2	0.11212
BZLF1	COMMD10	0.23617
BZLF1	NOM1	0.22117
BZLF1	ACVR2A	0.024667
BZLF1	GRK5	0.034399
BZLF1	FANCC	0.14321
BZLF1	RFX3	0.033136
BZLF1	ITGB1BP2	0.022877
BZLF1	CENPA	0.059424
BZLF1	MDFIC	0.081893
BZLF1	UBXN6	0.16659
BZLF1	SYNPO2	0.18536
BZLF1	BABAM1	0.17204
BZLF1	IL5RA	0.2
BZLF1	AIF1L	0.039031
BZLF1	LPCAT4	0.036494
BZLF1	CDH11	0.043215
BZLF1	RABL6	0.15749
BZLF1	APEX1	0.062102
BZLF1	ELK3	0.022609
BZLF1	CBLL1	0.1286
BZLF1	PCDHAC1	0.046144
BZLF1	EPPIN	0.19579
BZLF1	GAS6	0.033144
BZLF1	BET1	0.12369
BZLF1	AMPH	0.1799
BZLF1	ABLIM1	0.14131
BZLF1	RANGAP1	0.080948
BZLF1	CIRBP	0.15358
BZLF1	YML009W-B	0.011173
BZLF1	ERLEC1	0.20834
BZLF1	LATS1	0.014621
BZLF1	KIFAP3	0.1423
BZLF1	MED9	0.1292
BZLF1	ABCB1	0.11594
BZLF1	PCDHB6	0.042302
BZLF1	EFEMP1	0.34642
BZLF1	EIF4A3	0.14138
BZLF1	LPAR2	0.17023
BZLF1	ZAP70	0.07191
BZLF1	DAXX	0.057284
BZLF1	IL1A	0.08488
BZLF1	BID	0.061373
BZLF1	COMMD2	0.23617
BZLF1	KANSL1	0.18265
BZLF1	SDPR	0.13486
BZLF1	AGRN	0.065711
BZLF1	MKL1	0.12921
BZLF1	ZNF281	0.017655
BZLF1	TTC37	0.18569
BZLF1	PNMA1	0.16717
BZLF1	APC2	0.14165

BZLF1	CCDC53	0.22498
BZLF1	NIPSNAP3A	0.22256
BZLF1	SPATA13	0.050515
BZLF1	MYO9B	0.13131
BZLF1	C1D	0.14858
BZLF1	BAP1	0.087264
BZLF1	CIR1	0.050751
BZLF1	ULK3	0.086686
BZLF1	TBCB	0.17982
BZLF1	SPARC	0.17918
BZLF1	GTF2H1	0.048364
BZLF1	CREB5	0.043344
BZLF1	TCHHL1	0.057416
BZLF1	EWSR1	0.17622
BZLF1	F2RL1	0.027539
BZLF1	OC90	0.043846
BZLF1	EHMT2	0.1467
BZLF1	PALLD	0.15439
BZLF1	SRSF11	0.20037
BZLF1	NOTCH4	0.13641
BZLF1	RAPGEF3	0.10192
BZLF1	PIK3C3	0.10756
BZLF1	TERT	0.11047
BZLF1	NUTF2	0.19848
BZLF1	ARPC4	0.15561
BZLF1	EXOSC10	0.092486
BZLF1	PCDHB1	0.049103
BZLF1	MEAF6	0.14945
BZLF1	ANG	0.015816
BZLF1	KLHL12	0.24342
BZLF1	TSPYL5	0.13805
BZLF1	PIM1	0.028266
BZLF1	DLX4	0.057083
BZLF1	MBD2	0.096395
BZLF1	MST4	0.031629
BZLF1	FAM160A1	0.010711
BZLF1	TRAIP	0.13331
BZLF1	DFNB31	0.11228
BZLF1	CYTH2	0.10763
BZLF1	CTR9	0.10592
BZLF1	NAA15	0.11131
BZLF1	MATN1	0.22876
BZLF1	NOTCH2NL	0.049691
BZLF1	CA4	0.079011
BZLF1	DEPDC1	0.13687
BZLF1	ID4	0.11035
BZLF1	KIAA1279	0.20237
BZLF1	PCDH17	0.42347
BZLF1	CSNK2A2	0.062471
BZLF1	PIR	0.20376
BZLF1	IGSF8	0.1833
BZLF1	EPHA4	0.061205
BZLF1	CSRNP2	0.046551
BZLF1	SET	0.061064
BZLF1	MCL1	0.092292
BZLF1	PRICKLE1	0.10132
BZLF1	FGFBP1	0.14741
BZLF1	GPR37L1	0.17078
BZLF1	ARHGEF2	0.022611
BZLF1	SLC9A1	0.077618
BZLF1	KITLG	0.097391
BZLF1	DSC3	0.046158
BZLF1	SLC22A3	0.12955
BZLF1	B2M	0.10525
BZLF1	GLRB	0.096652
BZLF1	KCNN4	0.14718
BZLF1	PHB	0.077622
BZLF1	PRDX4	0.25281

BZLF1	CCDC85B	0.132
BZLF1	APC	0.013167
BZLF1	PMS2	0.10712
BZLF1	XK	0.22878
BZLF1	SCAF11	0.13785
BZLF1	EXOC3	0.21329
BZLF1	EIF3B	0.25202
BZLF1	CLGN	0.023112
BZLF1	CIB2	0.057416
BZLF1	LDB3	0.16809
BZLF1	PPM1D	0.06902
BZLF1	KHDRBS3	0.19516
BZLF1	ERBB4	0.025835
BZLF1	EXD3	0.23617
BZLF1	ADH1A	0.14383
BZLF1	SPEN	0.060168
BZLF1	CEP72	0.15978
BZLF1	ALB	0.05963
BZLF1	GGT1	0.13392
BZLF1	NAP1L1	0.14544
BZLF1	SRSF1	0.16269
BZLF1	EAF2	0.1353
BZLF1	CASK	0.072222
BZLF1	ESRRG	0.047136
BZLF1	TARDBP	0.067751
BZLF1	RASSF4	0.22187
BZLF1	REPS1	0.42308
BZLF1	IL15RA	0.20113
BZLF1	PLEK	0.059609
BZLF1	CIRH1A	0.15902
BZLF1	TYRO3	0.079622
BZLF1	RUFY1	0.16533
BZLF1	PEX2	0.11507
BZLF1	PRH1	0.22238
BZLF1	SLC18A2	0.013861
BZLF1	TNFRSF10A	0.13355
BZLF1	POP5	0.14242
BZLF1	TRPS1	0.02819
BZLF1	LRRC26	0.1778
BZLF1	BBS5	0.1532
BZLF1	SIVA1	0.10147
BZLF1	NLGN3	0.086422
BZLF1	GCC1	0.16198
BZLF1	AURKAIP1	0.14888
BZLF1	CYTIP	0.21844
BZLF1	XAGE2	0.010964
BZLF1	USP14	0.12156
BZLF1	PCDHGA9	0.049103
BZLF1	ELOVL3	0.14558
BZLF1	EVI5	0.12055
BZLF1	RASA1	0.038114
BZLF1	PALB2	0.15669
BZLF1	CAND2	0.15802
BZLF1	FABP3	0.19619
BZLF1	CDHR5	0.033684
BZLF1	AHI1	0.066232
BZLF1	MRPS27	0.1722
BZLF1	ZBTB16	0.051062
BZLF1	SAT1	0.19274
BZLF1	DCTN2	0.11361
BZLF1	TBC1D8B	0.029191
BZLF1	TUBGCP3	0.050864
BZLF1	AGO1	0.13264
BZLF1	CD53	0.19631
BZLF1	USP25	0.14135
BZLF1	AFF1	0.067962
BZLF1	SH3GL2	0.11497
BZLF1	TULP1	0.12972

BZLF1	NXF5	0.22678
BZLF1	SNX1	0.13655
BZLF1	CD151	0.17357
BZLF1	MDFI	0.098984
BZLF1	YTHDC1	0.1902
BZLF1	MRPL28	0.18495
BZLF1	KRT16	0.14207
BZLF1	GUCA1C	0.067323
BZLF1	DOCK1	0.12504
BZLF1	CASP10	0.16375
BZLF1	MRPS9	0.15311
BZLF1	GEMIN7	0.15495
BZLF1	TBC1D7	0.21163
BZLF1	CDH7	0.045044
BZLF1	SPI1	0.017032
BZLF1	PPP1R1A	0.20888
BZLF1	SEC24C	0.054992
BZLF1	PRKCSH	0.093011
BZLF1	ZCCHC11	0.12099
BZLF1	TGFBR3	0.038877
BZLF1	ASUN	0.18357
BZLF1	AMY1A	0.11276
BZLF1	GRAP2	0.14724
BZLF1	EXOC7	0.17216
BZLF1	UPF1	0.10599
BZLF1	STX16	0.10828
BZLF1	TXNIP	0.087896
BZLF1	ANKRD28	0.19741
BZLF1	DRD4	0.052773
BZLF1	FGF1	0.073053
BZLF1	ITPR3	0.027258
BZLF1	GAGE2D	0.22903
BZLF1	NEK11	0.072274
BZLF1	ARL13B	0.18382
BZLF1	ASAP1	0.011511
BZLF1	ADAMTS10	0.093256
BZLF1	YLPM1	0.15806
BZLF1	BRAT1	0.19741
BZLF1	SIGIRR	0.16896
BZLF1	TAS2R16	0.1524
BZLF1	ADRA2C	0.098654
BZLF1	PCDHGA2	0.049103
BZLF1	RDX	0.14637
BZLF1	TYR	0.14009
BZLF1	POU3F2	0.03081
BZLF1	ITGB4	0.098765
BZLF1	KAL1	0.14425
BZLF1	ZXDC	0.028952
BZLF1	SSR4	0.093875
BZLF1	DDIT3	0.027567
BZLF1	GTF3C6	0.18778
BZLF1	WIBG	0.24801
BZLF1	ARHGAP22	0.053324
BZLF1	PCDH1	0.041569
BZLF1	SLC40A1	0.11769
BZLF1	FBXW5	0.073139
BZLF1	CFLAR	0.14345
BZLF1	EIF4EBP1	0.10938
BZLF1	MED29	0.15012
BZLF1	CDKN3	0.018694
BZLF1	TAX1BP1	0.15684
BZLF1	NOTCH2	0.092742
BZLF1	LOXL3	0.12948
BZLF1	ERLIN2	0.17045
BZLF1	EFCAB12	0.057416
BZLF1	CDH6	0.024815
BZLF1	FZR1	0.12463
BZLF1	ROR1	0.15552

BZLF1	AMER2	0.2256
BZLF1	MITD1	0.15663
BZLF1	TAF1D	0.15765
BZLF1	SLC4A1AP	0.15423
BZLF1	SIAH2	0.077858
BZLF1	EXOSC3	0.14696
BZLF1	STIM1	0.23397
BZLF1	COG4	0.15148
BZLF1	S100B	0.23026
BZLF1	TRAF3IP1	0.2221
BZLF1	ZAK	0.043392
BZLF1	FZD6	0.069549
BZLF1	KAT6B	0.064585
BZLF1	HMG20B	0.052594
BZLF1	ESRRA	0.026989
BZLF1	SKP1	0.15127
BZLF1	VAMP1	0.20795
BZLF1	BIRC2	0.056249
BZLF1	ZNF212	0.0968
BZLF1	TNNC1	0.3488
BZLF1	DDB1	0.10535
BZLF1	RBMX	0.11349
BZLF1	SAP30	0.12698
BZLF1	ZNF24	0.04997
BZLF1	CHD3	0.052576
BZLF1	ADCYAP1R1	0.050787
BZLF1	CRYGC	0.175
BZLF1	EFCAB4A	0.052632
BZLF1	ERBB2	0.020421
BZLF1	HTATIP2	0.11203
BZLF1	STEAP3	0.13101
BZLF1	TXLNA	0.1939
BZLF1	POLRMT	0.16664
BZLF1	BCAS2	0.17668
BZLF1	TMED2	0.15733
BZLF1	RAB3IL1	0.19162
BZLF1	CCNC	0.046955
BZLF1	C1orf94	0.23617
BZLF1	CASP6	0.066624
BZLF1	VCL	0.080088
BZLF1	PCDHB13	0.044991
BZLF1	CETN2	0.24596
BZLF1	CAMSAP2	0.20887
BZLF1	ANXA6	0.39193
BZLF1	PCDHGC4	0.049103
BZLF1	LCP1	0.03216
BZLF1	DIXDC1	0.089003
BZLF1	SIRPG	0.15628
BZLF1	PA2G4	0.04479
BZLF1	TINF2	0.11207
BZLF1	FBXO44	0.22825
BZLF1	ITM2B	0.056425
BZLF1	ANXA2P2	0.069219
BZLF1	CUL4B	0.1677
BZLF1	KPNB1	0.099363
BZLF1	FEZ1	0.14634
BZLF1	CBX4	0.089305
BZLF1	FKBP9	0.061867
BZLF1	NDC80	0.11683
BZLF1	ATP10D	0.10817
BZLF1	SMG5	0.14988
BZLF1	STK38L	0.029958
BZLF1	ACTN2	0.1281
BZLF1	COL3A1	0.095199
BZLF1	GGA1	0.16725
BZLF1	LRCH4	0.21897
BZLF1	EFNA1	0.10185
BZLF1	ATF5	0.043243

BZLF1	CNOT7	0.030634
BZLF1	SEPN1	0.18685
BZLF1	IFIT3	0.17035
BZLF1	PRSS2	0.32801
BZLF1	STAT2	0.14921
BZLF1	PRDM5	0.078716
BZLF1	FSHB	0.13463
BZLF1	PSRC1	0.12467
BZLF1	REEP1	0.17484
BZLF1	PIFO	0.11902
BZLF1	METTL9	0.23617
BZLF1	CD59	0.16173
BZLF1	NCKAP1L	0.063964
BZLF1	LIMS1	0.14016
BZLF1	ZNF219	0.020002
BZLF1	ACTG1	0.056045
BZLF1	NCKAP1	0.13602
BZLF1	EIF4A2	0.18277
BZLF1	PNRC2	0.14912
BZLF1	PDE4DIP	0.16621
BZLF1	RAB27A	0.064074
BZLF1	SESTD1	0.16554
BZLF1	FKBP4	0.070721
BZLF1	KANK2	0.15856
BZLF1	RPL5	0.11919
BZLF1	TECPR2	0.23617
BZLF1	CDCA5	0.14358
BZLF1	HAX1	0.17926
BZLF1	STAU2	0.20072
BZLF1	SIRT1	0.010036
BZLF1	NOS3	0.020445
BZLF1	PITPNM3	0.37101
BZLF1	SEPSECS	0.12334
BZLF1	VIL1	0.17139
BZLF1	TNIP2	0.041778
BZLF1	ADCY6	0.015441
BZLF1	S100A7L2	0.03514
BZLF1	RNF126	0.18896
BZLF1	EIF5B	0.16561
BZLF1	STX11	0.20453
BZLF1	FSTL3	0.0833
BZLF1	ELK4	0.056984
BZLF1	WEE1	0.050419
BZLF1	PSMA6	0.061132
BZLF1	PCDHA3	0.046144
BZLF1	MUC5B	0.18953
BZLF1	CIB3	0.057416
BZLF1	CCDC59	0.17318
BZLF1	CD44	0.086719
BZLF1	PPID	0.22469
BZLF1	AGL	0.082062
BZLF1	SMNDC1	0.22403
BZLF1	AATF	0.054482
BZLF1	TFF2	0.16366
BZLF1	NEDD9	0.1242
BZLF1	CNOT1	0.1454
BZLF1	RPL14	0.17003
BZLF1	PTPN9	0.059925
BZLF1	ATF7	0.017654
BZLF1	GUCY2C	0.028908
BZLF1	HPCA	0.054177
BZLF1	SMC5	0.13264
BZLF1	ZRANB1	0.095615
BZLF1	DNASE1L2	0.04979
BZLF1	PTPRO	0.032675
BZLF1	KCNJ4	0.22931
BZLF1	ANK3	0.079291
BZLF1	DDX6	0.15787

BZLF1	APOBEC1	0.086909
BZLF1	ERLIN1	0.18681
BZLF1	NLRP3	0.097464
BZLF1	ZNF473	0.11175
BZLF1	RAD9B	0.2044
BZLF1	AHSA1	0.17894
BZLF1	GTF2A2	0.09435
BZLF1	VSIG4	0.2015
BZLF1	TOP1	0.077683
BZLF1	DNMT3B	0.085665
BZLF1	RAC3	0.06569
BZLF1	IGF2R	0.13254
BZLF1	SORCS1	0.19901
BZLF1	PYURF	0.19227
BZLF1	HRG	0.056287
BZLF1	ORC3	0.13652
BZLF1	SCYL3	0.1844
BZLF1	CCL18	0.18014
BZLF1	ARPC3	0.14385
BZLF1	SIT1	0.1573
BZLF1	NUAK1	0.094348
BZLF1	TXN	0.033253
BZLF1	DIABLO	0.14676
BZLF1	C14orf166	0.10317
BZLF1	PIK3R4	0.091803
BZLF1	XRCC1	0.14858
BZLF1	CETN1	0.3321
BZLF1	UBQLN4	0.15685
BZLF1	KCNJ9	0.21183
BZLF1	LPP	0.1289
BZLF1	AKAP12	0.16847
BZLF1	ZBTB25	0.033876
BZLF1	BCOR	0.090056
BZLF1	TTBK2	0.11737
BZLF1	MPHOSPH10	0.11518
BZLF1	MLLT10	0.02892
BZLF1	PFKFB2	0.062662
BZLF1	SGOL1	0.10233
BZLF1	CUL3	0.079485
BZLF1	NTNG1	0.18844
BZLF1	MC1R	0.092783
BZLF1	COG7	0.13846
BZLF1	PROCR	0.16918
BZLF1	ERC2	0.15852
BZLF1	ATP1B2	0.14982
BZLF1	ZSWIM7	0.15284
BZLF1	MAP2K2	0.02426
BZLF1	HLA-A	0.092549
BZLF1	GK	0.17623
BZLF1	GNB2	0.11325
BZLF1	HSPB9	0.19164
BZLF1	PSMC6	0.070564
BZLF1	NDFIP1	0.077257
BZLF1	STK10	0.11503
BZLF1	ANXA13	0.052234
BZLF1	HTRA2	0.096813
BZLF1	NUP98	0.10189
BZLF1	ANK2	0.032068
BZLF1	NFKBIA	0.033158
BZLF1	LAT	0.081085
BZLF1	SOD3	0.09401
BZLF1	SEPT6	0.10511
BZLF1	MYO5A	0.043221
BZLF1	CIC	0.15765
BZLF1	COMMD8	0.23617
BZLF1	IGFBP2	0.15798
BZLF1	DUSP2	0.025217
BZLF1	CCR5	0.073563

BZLF1	RPS13	0.12154
BZLF1	TAF8	0.11552
BZLF1	CRHR2	0.11991
BZLF1	IL1RAPL1	0.10898
BZLF1	HPN	0.10136
BZLF1	VLDLR	0.13584
BZLF1	NUP62	0.05348
BZLF1	INPPL1	0.064377
BZLF1	TRIP13	0.095594
BZLF1	CDH1	0.06911
BZLF1	HTT	0.030618
BZLF1	DNAJB4	0.17095
BZLF1	ADAMTS13	0.16527
BZLF1	BCAP31	0.056072
BZLF1	DIAPH1	0.059711
BZLF1	TEAD1	0.05832
BZLF1	BCL6	0.017096
BZLF1	MAP1LC3B	0.22235
BZLF1	USF2	0.042611
BZLF1	UBA5	0.069264
BZLF1	TTN	0.075825
BZLF1	ZDHHC17	0.179
BZLF1	MICU3	0.054178
BZLF1	CAPNS2	0.05264
BZLF1	ABT1	0.154
BZLF1	NDRG2	0.098471
BZLF1	TWIST2	0.023761
BZLF1	FSTL4	0.055781
BZLF1	POLB	0.053329
BZLF1	ACTR3	0.037902
BZLF1	DCPS	0.084124
BZLF1	TUT1	0.13897
BZLF1	PSMC5	0.056461
BZLF1	NCALD	0.050439
BZLF1	MUS81	0.11512
BZLF1	PRDM16	0.07004
BZLF1	EGF	0.13366
BZLF1	RAPGEF2	0.034129
BZLF1	TFAM	0.057935
BZLF1	TJP1	0.13529
BZLF1	CBX1	0.094536
BZLF1	UIMC1	0.1295
BZLF1	HOMER3	0.1565
BZLF1	AJUBA	0.039161
BZLF1	MIEF1	0.14506
BZLF1	TIRAP	0.059663
BZLF1	PDK3	0.15577
BZLF1	PALM3	0.15847
BZLF1	FYN	0.044381
BZLF1	PRKAB2	0.033
BZLF1	USP26	0.16538
BZLF1	PPP1R16B	0.17125
BZLF1	HIVEP1	0.10672
BZLF1	ELOVL5	0.13432
BZLF1	LTBP4	0.29779
BZLF1	BLOC1S6	0.089007
BZLF1	MOAP1	0.21927
BZLF1	RANBP2	0.078656
BZLF1	PRMT2	0.14773
BZLF1	RPS19	0.13116
BZLF1	DCAF16	0.2289
BZLF1	SMARCAD1	0.095391
BZLF1	CDKN1B	0.054512
BZLF1	NPHP4	0.077657
BZLF1	GNB2L1	0.02263
BZLF1	NUP43	0.12175
BZLF1	AMELX	0.12018
BZLF1	PLSCR4	0.36004

BZLF1	LMNA	0.085789
BZLF1	INSIG1	0.11386
BZLF1	HIRIP3	0.19741
BZLF1	CENPF	0.054708
BZLF1	LIN37	0.22205
BZLF1	MPP7	0.18382
BZLF1	VAMP2	0.13671
BZLF1	RBM8A	0.16858
BZLF1	ALKBH3	0.15503
BZLF1	ZFAND6	0.12348
BZLF1	SHANK1	0.096222
BZLF1	DCP2	0.14626
BZLF1	CCNK	0.10573
BZLF1	FBXL2	0.20628
BZLF1	SCOC	0.1432
BZLF1	LAMB3	0.084129
BZLF1	MBD4	0.093571
BZLF1	RXRB	0.038163
BZLF1	FBXO25	0.17976
BZLF1	KAT8	0.089794
BZLF1	POLD3	0.064684
BZLF1	NID1	0.051729
BZLF1	MEPE	0.21593
BZLF1	GOLGB1	0.16955
BZLF1	HBA1	0.15995
BZLF1	NXF1	0.17926
BZLF1	GMIP	0.14554
BZLF1	C1R	0.10007
BZLF1	TUBG1	0.092328
BZLF1	SLC6A2	0.21877
BZLF1	C1orf106	0.010782
BZLF1	AKR1B10	0.20595
BZLF1	NOP2	0.29812
BZLF1	DNAJC1	0.098884
BZLF1	FCGBP	0.22211
BZLF1	ZC3H11A	0.18328
BZLF1	TSHZ3	0.014162
BZLF1	CASC5	0.074294
BZLF1	SERPINC1	0.14342
BZLF1	FN1	0.095919
BZLF1	ACE	0.014518
BZLF1	SRSF2	0.18303
BZLF1	DMP1	0.011473
BZLF1	RHOT1	0.19848
BZLF1	RPS6KB1	0.012187
BZLF1	DDX39A	0.13388
BZLF1	SAP18	0.13644
BZLF1	SAFB	0.14043
BZLF1	ASB13	0.21937
BZLF1	TEAD4	0.036893
BZLF1	SNX9	0.21332
BZLF1	NLGN4X	0.086061
BZLF1	COL4A3	0.040508
BZLF1	CITED2	0.024794
BZLF1	CSRP3	0.076924
BZLF1	VPS53	0.19463
BZLF1	TTI1	0.23617
BZLF1	NCBP2	0.10578
BZLF1	ZC3H15	0.16478
BZLF1	IFRD2	0.22358
BZLF1	TNPO1	0.13297
BZLF1	LSM1	0.15624
BZLF1	COPS4	0.18165
BZLF1	ABI3	0.19278
BZLF1	UBXN4	0.1566
BZLF1	NLRP12	0.12132
BZLF1	CSF2	0.12714
BZLF1	DNAJC10	0.07255

BZLF1	PARN	0.10868
BZLF1	FAM175A	0.18244
BZLF1	ZCCHC8	0.14433
BZLF1	TRAF3	0.028432
BZLF1	RYR2	0.099765
BZLF1	DLAT	0.18099
BZLF1	ACTR5	0.14291
BZLF1	AXL	0.056064
BZLF1	RAET1E	0.19017
BZLF1	SMC6	0.1462
BZLF1	PARP9	0.26643
BZLF1	OPRM1	0.065301
BZLF1	GTPBP4	0.073919
BZLF1	CRLF1	0.19579
BZLF1	PRF1	0.24444
BZLF1	KIAA1109	0.18092
BZLF1	CACNA1I	0.1645
BZLF1	TFF1	0.16758
BZLF1	PCDHB3	0.042302
BZLF1	RPS6KA4	0.024416
BZLF1	PPP1R13L	0.084625
BZLF1	HSD17B14	0.16124
BZLF1	EXOC4	0.066337
BZLF1	SEPT12	0.12531
BZLF1	CUL7	0.13814
BZLF1	CDC16	0.11792
BZLF1	IL6ST	0.12296
BZLF1	CDC20	0.085673
BZLF1	STRN	0.15352
BZLF1	MYO1E	0.155
BZLF1	SPRY4	0.18273
BZLF1	ZNF366	0.11035
BZLF1	DDX20	0.077948
BZLF1	TEN1	0.1577
BZLF1	UBE2V1	0.066818
BZLF1	TRMT10C	0.2456
BZLF1	OCA2	0.11723
BZLF1	IGF1R	0.044254
BZLF1	PLA2G2C	0.023232
BZLF1	DGKZ	0.11533
BZLF1	SNRPA1	0.16384
BZLF1	LAIR1	0.2154
BZLF1	MICU2	0.055839
BZLF1	F2RL2	0.12912
BZLF1	VEGFB	0.090441
BZLF1	NHLRC1	0.11004
BZLF1	GFER	0.18818
BZLF1	SOCS2	0.12233
BZLF1	ZNF423	0.095476
BZLF1	MZT1	0.18333
BZLF1	IGF2BP1	0.073466
BZLF1	RNF144B	0.1205
BZLF1	PPP1R12C	0.22903
BZLF1	MCFD2	0.044487
BZLF1	CCDC28B	0.22191
BZLF1	MYL12B	0.020292
BZLF1	EIF3K	0.18926
BZLF1	PI4KA	0.20562
BZLF1	CBY1	0.14607
BZLF1	SLC25A24	0.052515
BZLF1	SGCE	0.039696
BZLF1	MAP3K4	0.077479
BZLF1	GPR143	0.10068
BZLF1	CBS	0.024273
BZLF1	TIMM9	0.11822
BZLF1	LNX1	0.12408
BZLF1	PKD2L2	0.061071
BZLF1	VDAC2	0.087876

BZLF1	IL13	0.09703
BZLF1	USP22	0.079924
BZLF1	SHARPIN	0.0944
BZLF1	OLFM1	0.19853
BZLF1	SART3	0.18261
BZLF1	PRPF40A	0.1523
BZLF1	LSM4	0.15637
BZLF1	WNK3	0.10497
BZLF1	FBXO11	0.09218
BZLF1	SNRNP70	0.17656
BZLF1	LDLRAP1	0.081111
BZLF1	EPHB4	0.11573
BZLF1	PCSK9	0.056464
BZLF1	TRA2B	0.22552
BZLF1	DGKI	0.16571
BZLF1	HSPB7	0.174
BZLF1	NOP10	0.14431
BZLF1	ELANE	0.12239
BZLF1	RING1	0.074108
BZLF1	ZNF496	0.036356
BZLF1	NELFA	0.13992
BZLF1	ACVR1B	0.029412
BZLF1	TCEB2	0.12455
BZLF1	TRIM23	0.01173
BZLF1	MYPN	0.16453
BZLF1	BRD3	0.1447
BZLF1	SETD2	0.12968
BZLF1	ZRANB2	0.088837
BZLF1	TRIM32	0.088616
BZLF1	PLD2	0.023643
BZLF1	YWHAH	0.10453
BZLF1	HEMGN	0.1805
BZLF1	PARP4	0.1531
BZLF1	CNEP1R1	0.11931
BZLF1	HNRNPM	0.166
BZLF1	KRT6A	0.14562
BZLF1	KLRF2	0.2073
BZLF1	AKAP17A	0.18643
BZLF1	PCDHA6	0.044854
BZLF1	SERPINB8	0.19105
BZLF1	MED28	0.13749
BZLF1	PLEC	0.13957
BZLF1	FADD	0.078062
BZLF1	TULP3	0.14181
BZLF1	ARRDC3	0.21576
BZLF1	TSC22D4	0.056406
BZLF1	RPS6KA5	0.02085
BZLF1	SERBP1	0.23247
BZLF1	TGFBRAP1	0.16351
BZLF1	FHOD1	0.1453
BZLF1	XRCC4	0.081465
BZLF1	ZFC3H1	0.19364
BZLF1	TP53BP2	0.1305
BZLF1	RNF187	0.10804
BZLF1	E2F3	0.043353
BZLF1	ZNF711	0.10686
BZLF1	PTPN2	0.051455
BZLF1	STIP1	0.15095
BZLF1	MALT1	0.060375
BZLF1	SUPT5H	0.078996
BZLF1	ELMO2	0.059716
BZLF1	ATP6V0A2	0.14057
BZLF1	SNCA	0.023725
BZLF1	FBXO27	0.22133
BZLF1	EMR3	0.02157
BZLF1	CARD10	0.1889
BZLF1	UBE3C	0.11874
BZLF1	RANBP6	0.18996

BZLF1	MCIDAS	0.11577
BZLF1	S100A8	0.15747
BZLF1	THAP11	0.12207
BZLF1	MT-ND3	0.18218
BZLF1	CD70	0.12141
BZLF1	IL2RG	0.14734
BZLF1	NID2	0.4162
BZLF1	GAREML	0.011528
BZLF1	SYP	0.0629
BZLF1	F2R	0.026643
BZLF1	CCNB1	0.035357
BZLF1	CADM1	0.072721
BZLF1	DIS3L	0.19296
BZLF1	NELL1	0.28208
BZLF1	DIRAS3	0.13271
BZLF1	TICRR	0.16652
BZLF1	FAM84B	0.22211
BZLF1	SLX1A	0.1487
BZLF1	ZNF365	0.19177
BZLF1	DEDD	0.12354
BZLF1	ZHX1	0.030238
BZLF1	ANXA1	0.04981
BZLF1	ytzL	0.013356
BZLF1	FAF2	0.2008
BZLF1	CDKN2B	0.056763
BZLF1	PFDN5	0.1288
BZLF1	ZMYND11	0.087267
BZLF1	CLDN7	0.07698
BZLF1	KRAS	0.061336
BZLF1	PTPRE	0.042424
BZLF1	C3	0.092406
BZLF1	PCDHB16	0.044991
BZLF1	PTPRK	0.022869
BZLF1	SNTN	0.054233
BZLF1	SIRT3	0.12464
BZLF1	ADA	0.034652
BZLF1	CRHBP	0.073593
BZLF1	DNER	0.28397
BZLF1	FBXO17	0.22133
BZLF1	COL17A1	0.14961
BZLF1	PRKAR1A	0.095328
BZLF1	EIF6	0.23898
BZLF1	CD72	0.13427
BZLF1	VSNL1	0.057416
BZLF1	RUVBL2	0.052982
BZLF1	FBXL14	0.21751
BZLF1	PCDHGA6	0.049103
BZLF1	CLN5	0.15965
BZLF1	CFL1	0.088134
BZLF1	MBD1	0.046
BZLF1	MKL2	0.13505
BZLF1	SCNN1G	0.15098
BZLF1	PHLDA1	0.19785
BZLF1	NDUFS3	0.14473
BZLF1	POU2F1	0.044038
BZLF1	SLC25A10	0.10246
BZLF1	ATP8A1	0.14699
BZLF1	HMG20A	0.059322
BZLF1	TLX1	0.042295
BZLF1	IL17RA	0.14996
BZLF1	CDK4	0.013438
BZLF1	UBE2V2	0.11692
BZLF1	EFCAB2	0.057416
BZLF1	DSCAM	0.14474
BZLF1	NUCB1	0.054449
BZLF1	SAFB2	0.18667
BZLF1	CYTH1	0.11766
BZLF1	HEYL	0.028507

BZLF1	ELOVL6	0.14773
BZLF1	EPDR1	0.054106
BZLF1	PPARGC1A	0.051889
BZLF1	NRXN1	0.1279
BZLF1	PEF1	0.40897
BZLF1	OFD1	0.11341
BZLF1	CD22	0.18978
BZLF1	RBM15B	0.15651
BZLF1	SHOX	0.051849
BZLF1	NOXA1	0.167
BZLF1	DUX4L9	0.042241
BZLF1	ZWINT	0.12392
BZLF1	NAPG	0.17679
BZLF1	ZFYVE26	0.18206
BZLF1	RBBP8	0.099505
BZLF1	MAX	0.050547
BZLF1	ANXA3	0.037855
BZLF1	FBXL8	0.23617
BZLF1	SNRPE	0.13281
BZLF1	CGN	0.19916
BZLF1	XIAP	0.11529
BZLF1	GCHFR	0.12128
BZLF1	PLA2G16	0.088537
BZLF1	EZR	0.10952
BZLF1	COL16A1	0.17593
BZLF1	PSMA3	0.050508
BZLF1	RAD17	0.12603
BZLF1	FBL	0.23988
BZLF1	TTYH1	0.044871
BZLF1	CNNM3	0.20684
BZLF1	RASGRP2	0.035643
BZLF1	TSSK6	0.078634
BZLF1	MEGF8	0.32375
BZLF1	MCU	0.15013
BZLF1	PDGFB	0.01933
BZLF1	PCDHGB5	0.049103
BZLF1	CHCHD6	0.22944
BZLF1	CDH13	0.021089
BZLF1	RNF168	0.073912
BZLF1	ATXN1	0.11819
BZLF1	FKBP9L	0.046456
BZLF1	GAP43	0.15126
BZLF1	TTYH2	0.18926
BZLF1	BAK1	0.030168
BZLF1	BEND7	0.23617
BZLF1	CHP1	0.14306
BZLF1	PRICKLE3	0.17918
BZLF1	PAF1	0.11242
BZLF1	REEP5	0.20892
BZLF1	ANTXR2	0.15404
BZLF1	MAP4K2	0.059492
BZLF1	NFATC1	0.032686
BZLF1	CYFIP2	0.17743
BZLF1	CSTF2	0.18274
BZLF1	TNK2	0.088426
BZLF1	CAMTA2	0.085252
BZLF1	MAPK3	0.022235
BZLF1	ERAP1	0.092459
BZLF1	UBE2E1	0.077613
BZLF1	CHCHD1	0.1869
BZLF1	APTX	0.051214
BZLF1	VPS52	0.20068
BZLF1	MYL12A	0.058208
BZLF1	CACNG2	0.12034
BZLF1	DAP3	0.082676
BZLF1	ZGPAT	0.032078
BZLF1	RASSF3	0.17431
BZLF1	CFTR	0.096695

BZLF1	RAB3IP	0.14395
BZLF1	SPG7	0.052012
BZLF1	GNAQ	0.028028
BZLF1	CTSH	0.06674
BZLF1	XPO5	0.15652
BZLF1	ABCD2	0.1497
BZLF1	CD86	0.092269
BZLF1	EGFL6	0.055024
BZLF1	KMT2E	0.11888
BZLF1	IMP4	0.14
BZLF1	PRPF31	0.17407
BZLF1	NOMO2	0.18246
BZLF1	SYMPK	0.15566
BZLF1	CUL5	0.093698
BZLF1	CNST	0.21955
BZLF1	COL6A2	0.14205
BZLF1	TRAPPC10	0.19848
BZLF1	LIPE	0.060548
BZLF1	HAP1	0.14866
BZLF1	ATP6V1C1	0.16018
BZLF1	PSMB7	0.053521
BZLF1	AHCYL1	0.16285
BZLF1	TGM2	0.15464
BZLF1	RPLP0	0.14307
BZLF1	NR3C1	0.019831
BZLF1	BAG1	0.10514
BZLF1	ATF7IP	0.14951
BZLF1	MAPK10	0.05683
BZLF1	FASN	0.1361
BZLF1	DLL4	0.026797
BZLF1	SEPT7	0.020252
BZLF1	SMUG1	0.13161
BZLF1	MPZL1	0.093598
BZLF1	BRCA2	0.039569
BZLF1	LCMT1	0.2737
BZLF1	COL1A1	0.038955
BZLF1	EID2	0.11996
BZLF1	ELOVL7	0.15015
BZLF1	RPS3A	0.14716
BZLF1	GPR98	0.20121
BZLF1	PDS5B	0.14107
BZLF1	CRY1	0.039877
BZLF1	NUMBL	0.22027
BZLF1	UBA3	0.046523
BZLF1	ACAA2	0.18503
BZLF1	DLG4	0.016454
BZLF1	LSM6	0.14718
BZLF1	MBIP	0.17904
BZLF1	E2F4	0.012095
BZLF1	MICU1	0.35589
BZLF1	CALU	0.35409
BZLF1	EIF2S1	0.16962
BZLF1	TBRG1	0.16775
BZLF1	ELF2	0.042722
BZLF1	ATP1B1	0.13792
BZLF1	CLPTM1	0.19033
BZLF1	CEP63	0.17857
BZLF1	ZIC3	0.01847
BZLF1	RIPK1	0.021376
BZLF1	TICAM1	0.031521
BZLF1	AP2B1	0.099837
BZLF1	KIRREL3	0.19971
BZLF1	GPSM2	0.11184
BZLF1	PSMB5	0.051495
BZLF1	COIL	0.16235
BZLF1	ATXN2	0.10633
BZLF1	LILRB4	0.14756
BZLF1	PADI2	0.028472

BZLF1	XPC	0.09809
BZLF1	CD8A	0.073415
BZLF1	SSX2	0.16294
BZLF1	LENG8	0.23617
BZLF1	PRKRA	0.16738
BZLF1	KLK8	0.19267
BZLF1	PTPN13	0.03271
BZLF1	PPP3CA	0.12471
BZLF1	STRADB	0.029556
BZLF1	MTUS2	0.20676
BZLF1	ANXA8L2	0.035342
BZLF1	FBLN2	0.06383
BZLF1	BCL2A1	0.20889
BZLF1	HAND2	0.031403
BZLF1	PMEL	0.14826
BZLF1	TACR1	0.046335
BZLF1	SURF4	0.13186
BZLF1	MYOZ2	0.18892
BZLF1	VCP	0.057312
BZLF1	APIP	0.089886
BZLF1	USP28	0.14007
BZLF1	PPP1CA	0.042568
BZLF1	ATF6	0.010686
BZLF1	ITK	0.045746
BZLF1	HOXC13	0.045757
BZLF1	GADD45G	0.15174
BZLF1	BTG1	0.11173
BZLF1	MCTP1	0.052239
BZLF1	CBFB	0.045954
BZLF1	OTUB1	0.13923
BZLF1	PRKX	0.06933
BZLF1	MOCS3	0.099331
BZLF1	CCNG1	0.14063
BZLF1	VSTM2L	0.23617
BZLF1	RFX6	0.10965
BZLF1	ZFYVE28	0.14889
BZLF1	FCGR2C	0.20524
BZLF1	AKAP6	0.081171
BZLF1	BZRAP1	0.20671
BZLF1	EIF4B	0.22273
BZLF1	ANXA10	0.055404
BZLF1	PCDHGB3	0.049103
BZLF1	GLS2	0.13722
BZLF1	RNF40	0.099925
BZLF1	SOX5	0.027851
BZLF1	POP1	0.13903
BZLF1	SELENBP1	0.14489
BZLF1	PVR	0.080481
BZLF1	NCOR2	0.098504
BZLF1	LRP2	0.14789
BZLF1	VMP1	0.13536
BZLF1	CAPN2	0.21949
BZLF1	PAFAH1B3	0.12402
BZLF1	KRT8	0.14201
BZLF1	SLC23A1	0.14428
BZLF1	OCLN	0.042774
BZLF1	SIRPB1	0.19607
BZLF1	CPSF7	0.20005
BZLF1	ABCG5	0.099324
BZLF1	UNC119	0.21489
BZLF1	ABL1	0.024887
BZLF1	DGCR8	0.17846
BZLF1	CLIC1	0.11562
BZLF1	MKRN3	0.10834
BZLF1	TTC1	0.20307
BZLF1	SUPT4H1	0.024054
BZLF1	SREBF2	0.023858
BZLF1	HNRNPC	0.15728

BZLF1	TRIM37	0.091896
BZLF1	GRN	0.13968
BZLF1	PABPC4	0.15541
BZLF1	SLC22A11	0.18096
BZLF1	VPS33A	0.13638
BZLF1	TRIM5	0.082105
BZLF1	ANTXR1	0.13736
BZLF1	TNFRSF11A	0.078247
BZLF1	NPLOC4	0.12189
BZLF1	NAA10	0.19033
BZLF1	RGS2	0.10871
BZLF1	CTSL	0.12082
BZLF1	MON1B	0.23617
BZLF1	TIFA	0.20485
BZLF1	NUCB2	0.28795
BZLF1	TF	0.12891
BZLF1	CBR4	0.20576
BZLF1	RAD9A	0.079286
BZLF1	CBX6	0.15459
BZLF1	MRPL51	0.18431
BZLF1	MSX2	0.015224
BZLF1	CDKN2A	0.033742
BZLF1	PKDREJ	0.047492
BZLF1	PPP1R13B	0.1688
BZLF1	DCC	0.10931
BZLF1	LRSAM1	0.1119
BZLF1	SOX1	0.055826
BZLF1	HELZ2	0.094587
BZLF1	EED	0.12333
BZLF1	ASGR1	0.17348
BZLF1	NPC2	0.11598
BZLF1	HOXA9	0.050751
BZLF1	MAGEA11	0.19741
BZLF1	SH3RF2	0.15017
BZLF1	RAP1GAP	0.10949
BZLF1	USH1C	0.10406
BZLF1	PCDH19	0.049103
BZLF1	RNPS1	0.17125
BZLF1	FAM162A	0.16977
BZLF1	SH3GLB1	0.081267
BZLF1	CDON	0.092987
BZLF1	BCL2L14	0.10001
BZLF1	PINX1	0.081211
BZLF1	PSMD5	0.10649
BZLF1	PEX11A	0.16574
BZLF1	RIPK3	0.039806
BZLF1	S100A6	0.27256
BZLF1	RP1L1	0.20608
BZLF1	EPHB1	0.070976
BZLF1	AOC1	0.041002
BZLF1	STK16	0.060599
BZLF1	VHL	0.067748
BZLF1	FAT2	0.029725
BZLF1	FBXW2	0.20783
BZLF1	PLCG2	0.033506
BZLF1	SMARCD3	0.041213
BZLF1	RNF20	0.072456
BZLF1	ZFP36	0.037611
BZLF1	PPP2R1A	0.043974
BZLF1	IPO13	0.17372
BZLF1	AFG3L2	0.048051
BZLF1	EPB42	0.12553
BZLF1	WDR6	0.18315
BZLF1	DDC	0.098324
BZLF1	TERF2IP	0.062989
BZLF1	SPRED2	0.12977
BZLF1	ORMDL3	0.18681
BZLF1	TLR4	0.011785

BZLF1	PKHD1	0.10282
BZLF1	RNF19B	0.14826
BZLF1	IKBKAP	0.053982
BZLF1	MED11	0.1292
BZLF1	RABGEF1	0.12929
BZLF1	LRP8	0.24801
BZLF1	ZEB1	0.018098
BZLF1	NOX5	0.044823
BZLF1	SYNPO	0.14335
BZLF1	MAVS	0.012566
BZLF1	IGFLR1	0.2154
BZLF1	CASP2	0.088188
BZLF1	RFC5	0.067697
BZLF1	PPP6R3	0.18229
BZLF1	KRT14	0.086906
BZLF1	CBFA2T2	0.029845
BZLF1	GABRG2	0.11189
BZLF1	NUAK2	0.078232
BZLF1	NXT1	0.15151
BZLF1	S100P	0.37438
BZLF1	SNAI1	0.044946
BZLF1	BCL10	0.013214
BZLF1	CARD8	0.17862
BZLF1	PLK3	0.04688
BZLF1	SEMA4D	0.050425
BZLF1	SMURF1	0.072703
BZLF1	SIX5	0.049389
BZLF1	TMEM67	0.14636
BZLF1	WASL	0.051539
BZLF1	LRP1B	0.048402
BZLF1	DCAF7	0.18175
BZLF1	SGTA	0.20261
BZLF1	XPO6	0.16442
BZLF1	TRAK1	0.13006
BZLF1	SERTAD1	0.17971
BZLF1	PCDHGA5	0.049103
BZLF1	RNF103	0.12051
BZLF1	CLASP1	0.065854
BZLF1	DMD	0.080347
BZLF1	E2F2	0.015522
BZLF1	LCA5	0.18127
BZLF1	INTS4	0.19122
BZLF1	DYNLRB1	0.1502
BZLF1	GYPC	0.20241
BZLF1	SEPT1	0.17201
BZLF1	EGLN2	0.12583
BZLF1	PRDX6	0.2071
BZLF1	WT1	0.012451
BZLF1	YWHAZ	0.073044
BZLF1	CHAF1A	0.18065
BZLF1	BTF3	0.17168
BZLF1	KIAA0319L	0.21539
BZLF1	SLC22A1	0.13357
BZLF1	DAZ1	0.20844
BZLF1	SEPT9	0.11541
BZLF1	PPP2R5C	0.11798
BZLF1	TAF9	0.067269
BZLF1	PTCRA	0.21535
BZLF1	SETD1B	0.20536
BZLF1	RIMS2	0.15365
BZLF1	TP53INP1	0.15695
BZLF1	F7	0.02476
BZLF1	ELF4	0.045683
BZLF1	ECH1	0.1181
BZLF1	CPS1	0.038244
BZLF1	C4BPA	0.19925
BZLF1	TAPBP	0.051926
BZLF1	RAPGEF1	0.14314

BZLF1	FEM1B	0.14855
BZLF1	ATXN7L3	0.13102
BZLF1	FNBP1	0.21982
BZLF1	KMT2B	0.068275
BZLF1	MCM10	0.14092
BZLF1	KCNB1	0.14126
BZLF1	CASP9	0.13427
BZLF1	NR4A2	0.023773
BZLF1	VRK1	0.085726
BZLF1	LTN1	0.14982
BZLF1	TEFM	0.11578
BZLF1	MTOR	0.023727
BZLF1	PTTG1	0.10546
BZLF1	PLAU	0.12918
BZLF1	DZIP1	0.12222
BZLF1	CLUAP1	0.20355
BZLF1	MADD	0.15754
BZLF1	NUP160	0.13483
BZLF1	NUP50	0.14537
BZLF1	TRIO	0.02384
BZLF1	RAB11FIP3	0.20846
BZLF1	PROCA1	0.048992
BZLF1	MOB1B	0.12378
BZLF1	EFCAB4B	0.34936
BZLF1	APP	0.013785
BZLF1	CDHR1	0.027553
BZLF1	HPX	0.11277
BZLF1	CLNS1A	0.14056
BZLF1	UCHL1	0.064957
BZLF1	GAB2	0.055419
BZLF1	ALKBH8	0.21821
BZLF1	PARD6G	0.17803
BZLF1	CAMK1	0.036358
BZLF1	SYBU	0.21379
BZLF1	UTP14A	0.17484
BZLF1	PCDHB8	0.049103
BZLF1	NOC4L	0.17095
BZLF1	YOD1	0.13153
BZLF1	GABPA	0.028856
BZLF1	ARL6IP1	0.2418
BZLF1	TMPRSS6	0.19491
BZLF1	BLOC1S4	0.17858
BZLF1	THAP3	0.18277
BZLF1	XAGE3	0.011844
BZLF1	FAM21C	0.18175
BZLF1	S100A7A	0.034181
BZLF1	ZNF256	0.03069
BZLF1	MUC17	0.14795
BZLF1	AP2A1	0.09692
BZLF1	TDGF1	0.048648
BZLF1	ZYX	0.10913
BZLF1	EFCAB3	0.057416
BZLF1	ANKZF1	0.18327
BZLF1	PFDN2	0.15455
BZLF1	PRRG2	0.050958
BZLF1	STK11	0.021775
BZLF1	DLGAP2	0.16704
BZLF1	SMOC2	0.034924
BZLF1	STXBP3	0.10688
BZLF1	TJP3	0.18768
BZLF1	SALL1	0.021015
BZLF1	SERPING1	0.14191
BZLF1	HIP1R	0.13176
BZLF1	THOC1	0.15214
BZLF1	YTHDC2	0.16708
BZLF1	RAD51B	0.13516
BZLF1	HNRNPA2B1	0.16053
BZLF1	CCT2	0.10879

BZLF1	FOSL1	0.026582
BZLF1	SETD6	0.22964
BZLF1	MTPAP	0.13318
BZLF1	CASR	0.13768
BZLF1	YWHAE	0.12322
BZLF1	EDEM3	0.017235
BZLF1	TPO	0.085997
BZLF1	GLA	0.074097
BZLF1	NDUFA13	0.1013
BZLF1	CHGB	0.22236
BZLF1	DTNA	0.30283
BZLF1	REPS2	0.45874
BZLF1	IMP3	0.16412
BZLF1	OPRK1	0.11953
BZLF1	SH3BP4	0.21518
BZLF1	MVP	0.1499
BZLF1	SASS6	0.18444
BZLF1	PPP3R1	0.076807
BZLF1	RRAGB	0.11037
BZLF1	CCDC50	0.22903
BZLF1	CDH24	0.052552
BZLF1	TRIM44	0.18327
BZLF1	ARFGEF1	0.073316
BZLF1	PPEF2	0.23521
BZLF1	EFEMP2	0.42247
BZLF1	DVL3	0.095987
BZLF1	IFNA2	0.13795
BZLF1	MAP4	0.076437
BZLF1	MYLK	0.071399
BZLF1	ZC3H13	0.18328
BZLF1	ALX1	0.050401
BZLF1	SART1	0.14924
BZLF1	MT-CO1	0.076129
BZLF1	AUNIP	0.18802
BZLF1	TANK	0.15782
BZLF1	HERC5	0.10531
BZLF1	DLEU1	0.23617
BZLF1	EIF3L	0.23073
BZLF1	STARD13	0.15998
BZLF1	ATCAY	0.14806
BZLF1	MAP1B	0.019188
BZLF1	HNRNPH1	0.15089
BZLF1	IFI6	0.1702
BZLF1	PFN2	0.16328
BZLF1	USP33	0.080273
BZLF1	DPH3	0.15227
BZLF1	ATP2A1	0.13576
BZLF1	CCS	0.096746
BZLF1	SIK2	0.056707
BZLF1	TCEA1	0.077302
BZLF1	TRRAP	0.13766
BZLF1	FZD4	0.048817
BZLF1	RPA2	0.068721
BZLF1	EXOSC4	0.11529
BZLF1	CSE1L	0.17402
BZLF1	RNF10	0.086579
BZLF1	SCAMP5	0.14502
BZLF1	PADI1	0.028472
BZLF1	HIST3H3	0.083063
BZLF1	CAB39	0.12103
BZLF1	WLS	0.24733
BZLF1	NOC2L	0.1093
BZLF1	ATP6V0A4	0.096149
BZLF1	PPP2R1B	0.22906
BZLF1	ITGA2	0.16081
BZLF1	CEP250	0.069833
BZLF1	EIF3D	0.22706
BZLF1	PDGFRL	0.015051

BZLF1	CXorf40A	0.23617
BZLF1	HNRNPD	0.10442
BZLF1	MYL3	0.044301
BZLF1	AGXT	0.12915
BZLF1	BAG6	0.098305
BZLF1	IL15	0.087045
BZLF1	CHD9	0.092344
BZLF1	CSF2RB	0.16051
BZLF1	RMDN3	0.074808
BZLF1	CD47	0.13647
BZLF1	CHMP3	0.13109
BZLF1	FOS	0.01029
BZLF1	PBK	0.13723
BZLF1	RFC4	0.0702
BZLF1	HOOK2	0.13072
BZLF1	ZC3H12A	0.12037
BZLF1	CCP110	0.1738
BZLF1	SMG7	0.14383
BZLF1	CD200	0.17428
BZLF1	LSM2	0.068096
BZLF1	AP3M1	0.17795
BZLF1	DYNC1I1	0.10458
BZLF1	ZNF703	0.081049
BZLF1	NFYB	0.049505
BZLF1	SH3GL3	0.18443
BZLF1	DMBT1	0.092556
BZLF1	MINK1	0.066552
BZLF1	PKN2	0.040246
BZLF1	MECOM	0.022456
BZLF1	LONP1	0.096084
BZLF1	PDCL3	0.20258
BZLF1	JAM3	0.11179
BZLF1	ASB16	0.21937
BZLF1	MLH3	0.1045
BZLF1	ARHGAP17	0.16037
BZLF1	PKD2L1	0.23426
BZLF1	MOB1A	0.19521
BZLF1	APOL1	0.16707
BZLF1	ADRB3	0.063514
BZLF1	TCEB1	0.11435
BZLF1	VPS4B	0.09212
BZLF1	FEZ2	0.20384
BZLF1	NFYA	0.049708
BZLF1	RNF111	0.076338
BZLF1	DROSHA	0.12385
BZLF1	CD2BP2	0.18346
BZLF1	NINL	0.047255
BZLF1	CD2	0.14501
BZLF1	ACOT8	0.089363
BZLF1	TPPP3	0.056619
BZLF1	NCOA6	0.079625
BZLF1	SMOC1	0.052333
BZLF1	SPPL2B	0.10264
BZLF1	ATR	0.084789
BZLF1	DMPK	0.011446
BZLF1	HTN1	0.20334
BZLF1	TRIM3	0.1419
BZLF1	EDC4	0.11055
BZLF1	PAX9	0.14179
BZLF1	ZNF408	0.0968
BZLF1	BTN3A1	0.18606
BZLF1	BIK	0.1296
BZLF1	PLEKHA1	0.059241
BZLF1	DAB2	0.081939
BZLF1	OR2L13	0.16229
BZLF1	HGS	0.091813
BZLF1	ARHGEF11	0.043318
BZLF1	SLC9A3R1	0.061754

BZLF1	TOPBP1	0.13128
BZLF1	BOLL	0.21351
BZLF1	MYLK2	0.028728
BZLF1	RYBP	0.096952
BZLF1	EHD3	0.24812
BZLF1	TUBB4A	0.01348
BZLF1	DYRK1A	0.040459
BZLF1	TGM1	0.21499
BZLF1	JAK1	0.098006
BZLF1	IGF2	0.084477
BZLF1	HSPA2	0.15213
BZLF1	FAM64A	0.23029
BZLF1	KIAA2018	0.024612
BZLF1	OCIAD1	0.21121
BZLF1	BCR	0.082221
BZLF1	SRCIN1	0.082887
BZLF1	TMEM8B	0.14544
BZLF1	SLC14A2	0.18342
BZLF1	ZZEF1	0.022415
BZLF1	AGER	0.097577
BZLF1	RICTOR	0.1442
BZLF1	MYL10	0.055839
BZLF1	PDGFRA	0.022954
BZLF1	LY96	0.11847
BZLF1	UBE2M	0.10719
BZLF1	NF2	0.058999
BZLF1	MICALL1	0.17775
BZLF1	CSK	0.078459
BZLF1	SMAD2	0.015618
BZLF1	RPS2	0.14323
BZLF1	UTF1	0.12862
BZLF1	EXOSC8	0.17524
BZLF1	IL4R	0.13227
BZLF1	NR3C2	0.05392
BZLF1	KRT15	0.16368
BZLF1	DUT	0.1233
BZLF1	NTN1	0.15351
BZLF1	ZXDA	0.029603
BZLF1	MAGEC1	0.23617
BZLF1	POU2F3	0.033703
BZLF1	P4HTM	0.046906
BZLF1	EFCAB11	0.057416
BZLF1	MT1G	0.095804
BZLF1	DLGAP5	0.11954
BZLF1	ATRX	0.093893
BZLF1	FKBP2	0.18659
BZLF1	TMEM102	0.17908
BZLF1	CDC26	0.15145
BZLF1	RNF6	0.085552
BZLF1	TAF9B	0.095471
BZLF1	DTL	0.13092
BZLF1	UVRAG	0.16993
BZLF1	COPB1	0.077387
BZLF1	KIF6	0.16217
BZLF1	THBS2	0.39077
BZLF1	OIT3	0.02943
BZLF1	EPS15L1	0.020124
BZLF1	PELI2	0.14782
BZLF1	NELFB	0.14121
BZLF1	FAP	0.13076
BZLF1	SLIT1	0.011475
BZLF1	NAF1	0.17548
BZLF1	MLLT3	0.14464
BZLF1	PRAME	0.12387
BZLF1	CALM1	0.16116
BZLF1	CRYAB	0.069988
BZLF1	SMARCA5	0.045675
BZLF1	ASAP2	0.026705

BZLF1	EIF4E2	0.27193
BZLF1	PROM1	0.13382
BZLF1	STAMBPL1	0.1818
BZLF1	MT-RNR2	0.1199
BZLF1	ACVR2B	0.026405
BZLF1	DBNL	0.12978
BZLF1	FGF3	0.13645
BZLF1	E2F1	0.015955
BZLF1	STRN4	0.2231
BZLF1	AVPR1A	0.074627
BZLF1	RRP8	0.14901
BZLF1	HPRT1	0.062944
BZLF1	YWHAG	0.15276
BZLF1	NPM3	0.18015
BZLF1	DNASE1	0.13998
BZLF1	RNF216	0.098361
BZLF1	MAGI2	0.067841
BZLF1	EPOR	0.14188
BZLF1	STRIP1	0.17995
BZLF1	EIF2A	0.12671
BZLF1	KCTD5	0.17357
BZLF1	CASP1	0.068445
BZLF1	ZRANB3	0.050676
BZLF1	GLUD1	0.11091
BZLF1	TFAP2C	0.013545
BZLF1	FLT3	0.093354
BZLF1	EXOC2	0.19843
BZLF1	ATG12	0.18531
BZLF1	RARG	0.015656
BZLF1	KIF1B	0.17048
BZLF1	CAV3	0.042363
BZLF1	SMARCB1	0.079043
BZLF1	CDHR3	0.049103
BZLF1	YEATS2	0.15939
BZLF1	KPNA5	0.17921
BZLF1	ARHGAP10	0.06269
BZLF1	S100A4	0.40887
BZLF1	ERCC5	0.089962
BZLF1	ABCG2	0.15613
BZLF1	GUCA1B	0.065721
BZLF1	NAPA	0.13405
BZLF1	TNPO2	0.17371
BZLF1	COL4A1	0.14967
BZLF1	DNAJC21	0.17361
BZLF1	S100A12	0.23087
BZLF1	FYCO1	0.23926
BZLF1	RGS16	0.2353
BZLF1	WNT1	0.035369
BZLF1	RAB9B	0.17266
BZLF1	C19orf80	0.22904
BZLF1	RCN1	0.44598
BZLF1	NUDT14	0.2082
BZLF1	PHC2	0.12816
BZLF1	MTMR2	0.028969
BZLF1	BMPR2	0.026615
BZLF1	IKBKE	0.065857
BZLF1	DDX39B	0.11995
BZLF1	WNT3A	0.017412
BZLF1	BAZ2B	0.12409
BZLF1	CASP8	0.030817
BZLF1	NEFL	0.11226
BZLF1	KCNJ6	0.17312
BZLF1	MYH2	0.12288
BZLF1	FKBP7	0.059302
BZLF1	MZB1	0.18001
BZLF1	LRRFIP1	0.103
BZLF1	PPP2R3C	0.029125
BZLF1	TNFRSF6B	0.2036

BZLF1	SMAD6	0.034391
BZLF1	TROAP	0.22154
BZLF1	MT1H	0.13681
BZLF1	RAPGEF4	0.070438
BZLF1	PSIP1	0.12404
BZLF1	SNRPD2	0.10454
BZLF1	SSX2IP	0.19823
BZLF1	ACD	0.090083
BZLF1	DPP4	0.12761
BZLF1	RERE	0.046423
BZLF1	SPSB4	0.21937
BZLF1	EIF3E	0.16582
BZLF1	EIF4E	0.16924
BZLF1	DSCC1	0.14013
BZLF1	RAMP1	0.092214
BZLF1	BBC3	0.065238
BZLF1	HSPBP1	0.19433
BZLF1	SDC2	0.10046
BZLF1	AK6	0.24505
BZLF1	HRAS	0.021145
BZLF1	EMILIN2	0.21112
BZLF1	SDIM1	0.22173
BZLF1	AIM2	0.070192
BZLF1	CLTCL1	0.16696
BZLF1	NPHP1	0.09863
BZLF1	CHD7	0.03879
BZLF1	SLC15A2	0.15404
BZLF1	DNMT3A	0.13301
BZLF1	ATP6V1F	0.13601
BZLF1	OAS3	0.12647
BZLF1	INHBA	0.037452
BZLF1	RPS23	0.1099
BZLF1	RQCD1	0.13571
BZLF1	PAG1	0.17931
BZLF1	NPB	0.18944
BZLF1	PIM3	0.10428
BZLF1	ACTN3	0.37525
BZLF1	ANXA4	0.050521
BZLF1	ATP5F1	0.21375
BZLF1	FBN1	0.3753
BZLF1	NR4A1	0.023826
BZLF1	RNF182	0.10602
BZLF1	MIF4GD	0.20396
BZLF1	MAP4K3	0.11086
BZLF1	TNNI2	0.16376
BZLF1	PDPK1	0.015732
BZLF1	RAD1	0.10854
BZLF1	ATG14	0.1968
BZLF1	F10	0.24725
BZLF1	PTK2B	0.012523
BZLF1	CAPN1	0.28261
BZLF1	PAX3	0.022964
BZLF1	UCKL1	0.23085
BZLF1	PLXDC1	0.15464
BZLF1	HSPB8	0.078376
BZLF1	MED15	0.11651
BZLF1	DSG1	0.028357
BZLF1	CEACAM6	0.19422
BZLF1	GSTZ1	0.20589
BZLF1	TLE1	0.074471
BZLF1	SKIL	0.075547
BZLF1	LILRB3	0.18038
BZLF1	ZNF3	0.015653
BZLF1	ZNF346	0.16484
BZLF1	RPSA	0.062468
BZLF1	SRPK1	0.051026
BZLF1	IPO7	0.13137
BZLF1	TRIM24	0.011617

10201	BZLF1	CFL2	0.20288
10202	BZLF1	ACAA1	0.11823
10203	BZLF1	DPM2	0.12934
10204	BZLF1	GHRHR	0.067865
10205	BZLF1	CX3CR1	0.086258
10206	BZLF1	PCDHA8	0.046144
10207	BZLF1	PSMG2	0.17497
10208	BZLF1	NDOR1	0.12488
10209	BZLF1	PPM1A	0.02416
10210	BZLF1	LETMD1	0.14953
10211	BZLF1	SYNM	0.14269
10212	BZLF1	PPP2R5E	0.13326
10213	BZLF1	SCGN	0.12683
10214	BZLF1	SAE1	0.054099
10215	BZLF1	TSN	0.20165
10216	BZLF1	VPS51	0.1774
10217	BZLF1	CELSR3	0.29303
10218	BZLF1	IARS	0.049478
10219	BZLF1	CAND1	0.14703
10220	BZLF1	FCHSD2	0.23617
10221	BZLF1	CATSPER1	0.11768
10222	BZLF1	RILP	0.19747
10223	BZLF1	ING5	0.1018
10224	BZLF1	SENP2	0.1184
10225	BZLF1	RCOR3	0.15235
10226	BZLF1	MAPKAPK5	0.056651
10227	BZLF1	LMAN1	0.060065
10228	BZLF1	UBE2B	0.06014
10229	BZLF1	MRPL48	0.18495
10230	BZLF1	SDHA	0.19415
10231	BZLF1	NR2C2	0.023576
10232	BZLF1	WASF1	0.12183
10233	BZLF1	CTIF	0.21923
10234	BZLF1	RNF115	0.14136
10235	BZLF1	SYNJ2	0.12827
10236	BZLF1	KIAA1524	0.20889
10237	BZLF1	ZNRF3	0.10873
10238	BZLF1	TCEAL5	0.17318
10239	BZLF1	PEX14	0.087717
10240	BZLF1	CACNA1A	0.033855
10241	BZLF1	AGO3	0.17395
10242	BZLF1	RPGRIP1	0.17811
10243	BZLF1	SPOCK1	0.076578
10244	BZLF1	N6AMT1	0.29913
10245	BZLF1	POLR2E	0.060517
10246	BZLF1	S100A5	0.021788
10247	BZLF1	POLR2J	0.070067
10248	BZLF1	CD4	0.049904
10249	BZLF1	ARF1	0.052891
10250	BZLF1	GEMIN8	0.17734
10251	BZLF1	CERS2	0.014727
10252	BZLF1	IGFL1	0.22238
10253	BZLF1	PEX13	0.16039
10254	BZLF1	EMR4P	0.022491
10255	BZLF1	IGBP1	0.11544
10256	BZLF1	MEOX2	0.042535
10257	BZLF1	TFAP4	0.07393
10258	BZLF1	UBAP2L	0.2221
10259	BZLF1	SETD8	0.17171
10260	BZLF1	MAGEA4	0.21685
10261	BZLF1	TAGLN2	0.20858
10262	BZLF1	SIRT4	0.30317
10263	BZLF1	FYTTD1	0.26914
10264	BZLF1	DNAJB6	0.15359
10265	BZLF1	PCDHA4	0.046144
10266	BZLF1	CDH15	0.036818
10267	BZLF1	FAM110A	0.19389
10268	BZLF1	LDHD	0.20262

BZLF1	PRNP	0.048302
BZLF1	TESC	0.223
BZLF1	CLIC3	0.15234
BZLF1	CHD1	0.080298
BZLF1	RBP4	0.092223
BZLF1	HOXB9	0.039003
BZLF1	SETD7	0.19039
BZLF1	FAM161A	0.27455
BZLF1	ATPAF1	0.22217
BZLF1	PARD3	0.087569
BZLF1	FMNL1	0.13806
BZLF1	UBE2Q1	0.12811
BZLF1	IFNLR1	0.19642
BZLF1	EFHB	0.057416
BZLF1	LGI1	0.11337
BZLF1	NME2	0.047864
BZLF1	KIAA0319	0.16778
BZLF1	CRY2	0.061528
BZLF1	ARL2	0.077643
BZLF1	EYS	0.083093
BZLF1	MICAL1	0.21015
BZLF1	NDNL2	0.17062
BZLF1	ROPN1L	0.20097
BZLF1	OBSCN	0.059008
BZLF1	UCN	0.10341
BZLF1	PCDHGB7	0.049103
BZLF1	RPA3	0.090748
BZLF1	FGFR3	0.038281
BZLF1	RWDD1	0.23617
BZLF1	TFPT	0.14454
BZLF1	SMPDL3A	0.17306
BZLF1	RHO	0.094752
BZLF1	LIG3	0.060728
BZLF1	HSPB6	0.20378
BZLF1	ADAMTS4	0.095888
BZLF1	PJA1	0.15063
BZLF1	AHNAK	0.19734
BZLF1	CCNF	0.15957
BZLF1	HOOK3	0.11637
BZLF1	SETD1A	0.19882
BZLF1	GLB1	0.084962
BZLF1	SMARCC2	0.10287
BZLF1	HIC1	0.012031
BZLF1	FCN3	0.15533
BZLF1	PPFIA1	0.2082
BZLF1	TAF4	0.025699
BZLF1	PPP4R4	0.22922
BZLF1	DCLRE1B	0.11286
BZLF1	OSBPL1A	0.093989
BZLF1	GOPC	0.096482
BZLF1	CD82	0.20871
BZLF1	TNFRSF1A	0.070261
BZLF1	DZIP3	0.19071
BZLF1	RALA	0.096499
BZLF1	CD226	0.083173
BZLF1	MOB4	0.17119
BZLF1	BIN3	0.16737
BZLF1	DRG1	0.098936
BZLF1	POLR2A	0.066431
BZLF1	RPH3A	0.23437
BZLF1	SPTSSA	0.14352
BZLF1	DCP1A	0.10909
BZLF1	ADAP1	0.014554
BZLF1	AMOTL2	0.23112
BZLF1	SMARCA1	0.073161
BZLF1	SRI	0.25334
BZLF1	SELL	0.13992
BZLF1	OCM	0.03514

10337	BZLF1	PPP4R2	0.14823
10338	BZLF1	RTP3	0.1979
10339	BZLF1	LRP4	0.23082
10340	BZLF1	DPPA4	0.16798
10341	BZLF1	KAT6A	0.055783
10342	BZLF1	PMF1	0.10693
10343	BZLF1	BDKRB2	0.081951
10344	BZLF1	KPNA2	0.14009
10345	BZLF1	EVI5L	0.17659
10346	BZLF1	NEK2	0.032865
10347	BZLF1	RAD54L	0.10538
10348	BZLF1	TSC2	0.047867
10349	BZLF1	SLC7A8	0.041219
10350	BZLF1	PSG5	0.22204
10351	BZLF1	PIK3R2	0.10736
10352	BZLF1	MAP3K14	0.035502
10353	BZLF1	BMX	0.04596
10354	BZLF1	INADL	0.17244
10355	BZLF1	SEL1L	0.18969
10356	BZLF1	GNL3L	0.1168
10357	BZLF1	PTPN12	0.049908
10358	BZLF1	PLA2G12B	0.027487
10359	BZLF1	FEN1	0.032807
10360	BZLF1	TMSB4X	0.13348
10361	BZLF1	PCDHB12	0.046144
10362	BZLF1	NONO	0.15679
10363	BZLF1	PLK1S1	0.11143
10364	BZLF1	CUL2	0.14362
10365	BZLF1	CCT3	0.1293
10366	BZLF1	SRSF7	0.16132
10367	BZLF1	CABP5	0.051272
10368	BZLF1	SLIT2	0.072386
10369	BZLF1	PKD1	0.020315
10370	BZLF1	HERC2	0.078536
10371	BZLF1	TBC1D5	0.15185
10372	BZLF1	SNED1	0.054106
10373	BZLF1	AKAP9	0.086171
10374	BZLF1	MIB1	0.12161
10375	BZLF1	PASK	0.090733
10376	BZLF1	BLM	0.027095
10377	BZLF1	ARNT	0.024408
10378	BZLF1	SYNE3	0.13542
10379	BZLF1	DOCK4	0.12053
10380	BZLF1	NCLN	0.12701
10381	BZLF1	HNRNPUL1	0.15975
10382	BZLF1	MAGEE1	0.16271
10383	BZLF1	RAD51AP1	0.22819
10384	BZLF1	CNPY2	0.20281
10385	BZLF1	NSRP1	0.19202
10386	BZLF1	SFI1	0.18005
10387	BZLF1	RPP38	0.16251
10388	BZLF1	MATN2	0.35107
10389	BZLF1	BCAR1	0.049158
10390	BZLF1	PSMD9	0.088595
10391	BZLF1	AXIN1	0.019059
10392	BZLF1	SIRT6	0.13682
10393	BZLF1	INTS7	0.19864
10394	BZLF1	STK4	0.01496
10395	BZLF1	GPD2	0.033362
10396	BZLF1	PCDHA10	0.044854
10397	BZLF1	CIB1	0.31435
10398	BZLF1	IRAK2	0.0306
10399	BZLF1	MAPK14	0.013104
10400	BZLF1	UBR5	0.1103
10401	BZLF1	PRRG4	0.048314
10402	BZLF1	ANKRD44	0.23617
10403	BZLF1	KIF13A	0.076198
10404	BZLF1	FNTA	0.16833

BZLF1	EIF5A2	0.13251
BZLF1	MAPRE3	0.17917
BZLF1	CLCN3	0.10757
BZLF1	DNAJB2	0.16343
BZLF1	DNMT1	0.1131
BZLF1	PCDHA11	0.046144
BZLF1	HDAC10	0.068537
BZLF1	PCDHB7	0.047639
BZLF1	TNK1	0.13952
BZLF1	PHF8	0.095545
BZLF1	GYS1	0.073833
BZLF1	PPP2R3A	0.33227
BZLF1	GSPT1	0.12237
BZLF1	DSC1	0.058018
BZLF1	AKAP3	0.24571
BZLF1	PRPF19	0.11961
BZLF1	EIF2B4	0.15
BZLF1	NCF1	0.02466
BZLF1	PSMC2	0.064462
BZLF1	TSSK4	0.07594
BZLF1	USE1	0.14743
BZLF1	LAPTM4B	0.21302
BZLF1	DLL1	0.23349
BZLF1	TAF2	0.097748
BZLF1	ACTN1	0.19903
BZLF1	CABP2	0.04811
BZLF1	VDR	0.028948
BZLF1	PEX11B	0.18083
BZLF1	PMP22	0.19253
BZLF1	RAB6A	0.12209
BZLF1	C11orf54	0.15671
BZLF1	STK38	0.029278
BZLF1	TNKS2	0.097143
BZLF1	RCHY1	0.072412
BZLF1	CCND1	0.031958
BZLF1	CD164	0.13493
BZLF1	EPRS	0.075577
BZLF1	TBC1D15	0.16365
BZLF1	AOC3	0.088495
BZLF1	FUS	0.16181
BZLF1	BLOC1S1	0.14095
BZLF1	FAM195A	0.010919
BZLF1	DCTN3	0.055527
BZLF1	NPHP3	0.10238
BZLF1	USP46	0.17662
BZLF1	UBD	0.13078
BZLF1	MYH10	0.069863
BZLF1	CDT1	0.12848
BZLF1	SYNE1	0.11937
BZLF1	FBXO3	0.1911
BZLF1	LRP6	0.013736
BZLF1	EXOC8	0.06043
BZLF1	KNTC1	0.10287
BZLF1	NCL	0.14005
BZLF1	ITGA1	0.13588
BZLF1	SVEP1	0.048532
BZLF1	NOS1	0.086312
BZLF1	EHD4	0.22676
BZLF1	APBA3	0.15104
BZLF1	RYR3	0.024226
BZLF1	SCUBE1	0.017195
BZLF1	PCDHB14	0.044991
BZLF1	HNRNPK	0.094666
BZLF1	LCAT	0.14874
BZLF1	OBFC1	0.068492
BZLF1	HJURP	0.090992
BZLF1	TWIST1	0.022061
BZLF1	E2F5	0.0166

BZLF1	CSNK1E	0.049258
BZLF1	FAM124B	0.23617
BZLF1	MEF2A	0.026242
BZLF1	PNPT1	0.081102
BZLF1	NPRL2	0.13465
BZLF1	UPF3B	0.20461
BZLF1	AKAP13	0.046295
BZLF1	ATG2A	0.23617
BZLF1	ZC3H7B	0.13687
BZLF1	LZTFL1	0.20339
BZLF1	TLN1	0.021079
BZLF1	BTRC	0.075978
BZLF1	PCDHGA11	0.049103
BZLF1	RHBDL1	0.13761
BZLF1	HNF1B	0.021176
BZLF1	UBE2D1	0.070102
BZLF1	GCKR	0.12053
BZLF1	MYO6	0.06828
BZLF1	LCMT2	0.29133
BZLF1	GATAD2A	0.03686
BZLF1	USP8	0.086189
BZLF1	DSPP	0.066884
BZLF1	FIG4	0.083842
BZLF1	TNFSF14	0.10051
BZLF1	CDC6	0.058374
BZLF1	CDH3	0.070337
BZLF1	PLAT	0.13662
BZLF1	TAX1BP3	0.15383
BZLF1	SNX4	0.19206
BZLF1	NEGR1	0.187
BZLF1	HTR6	0.14307
BZLF1	CUEDC2	0.19264
BZLF1	SEPT11	0.15811
BZLF1	DLGAP1	0.16747
BZLF1	CCND3	0.014064
BZLF1	MFI2	0.13071
BZLF1	FLNA	0.039707
BZLF1	HIST1H4A	0.067063
BZLF1	CTTNBP2NL	0.22787
BZLF1	RAI2	0.21688
BZLF1	SERPINF2	0.11466
BZLF1	FBXL5	0.16134
BZLF1	SGSM3	0.15977
BZLF1	DUOX1	0.10789
BZLF1	F3	0.045759
BZLF1	CECR2	0.16836
BZLF1	PMAIP1	0.047085
BZLF1	SPINK1	0.1966
BZLF1	ABCD1	0.10661
BZLF1	CHAC1	0.15997
BZLF1	DDX3X	0.0184
BZLF1	PCDHA2	0.046144
BZLF1	SH3GLB2	0.16619
BZLF1	CDK16	0.065281
BZLF1	RASEF	0.026314
BZLF1	DPP7	0.010114
BZLF1	PRSS3	0.3757
BZLF1	ANXA8	0.033435
BZLF1	RPS6KC1	0.10405
BZLF1	MAP1S	0.070554
BZLF1	BTK	0.016007
BZLF1	CLEC4G	0.2153
BZLF1	PPIL3	0.21036
BZLF1	CALML5	0.045684
BZLF1	LRRC4C	0.21539
BZLF1	MYL2	0.33548
BZLF1	BAIAP2	0.084187
BZLF1	KAT5	0.090713

10541	BZLF1	PIWIL1	0.17542
10542	BZLF1	ALOX12	0.094139
10543	BZLF1	EDEM1	0.28618
10544	BZLF1	LRP1	0.13007
10545	BZLF1	UFL1	0.13991
10546	BZLF1	APBA2	0.16863
10547	BZLF1	DHDDS	0.2461
10548	BZLF1	MKI67	0.1078
10549	BZLF1	JADE1	0.11911
10550	BZLF1	MTF1	0.016204
10551	BZLF1	FGFR1	0.014827
10552	BZLF1	GEMIN2	0.15043
10553	BZLF1	SELE	0.099407
10554	BZLF1	TONSL	0.15053
10555	BZLF1	CACNB4	0.07614
10556	BZLF1	RBM12B	0.28326
10557	BZLF1	CKS1B	0.169
10558	BZLF1	ATP10B	0.21618
10559	BZLF1	TIMM23	0.15683
10560	BZLF1	DICER1	0.028453
10561	BZLF1	HADHB	0.067994
10562	BZLF1	APPL1	0.054846
10563	BZLF1	CCDC92	0.21453
10564	BZLF1	VPRBP	0.1728
10565	BZLF1	SENP5	0.16588
10566	BZLF1	SUV39H1	0.11823
10567	BZLF1	COPS6	0.16575
10568	BZLF1	SNX2	0.16009
10569	BZLF1	MAP2K5	0.072645
10570	BZLF1	RNF11	0.10979
10571	BZLF1	ITGAM	0.10488
10572	BZLF1	CDH17	0.062722
10573	BZLF1	CDK11B	0.059526
10574	BZLF1	EFCAB9	0.057416
10575	BZLF1	PEX1	0.071802
10576	BZLF1	NUDT18	0.18626
10577	BZLF1	EXOSC2	0.18479
10578	BZLF1	ERG	0.023536
10579	BZLF1	ZNF622	0.11849
10580	BZLF1	PRCC	0.14602
10581	BZLF1	NFS1	0.12828
10582	BZLF1	EEF1E1	0.14734
10583	BZLF1	FANCD2	0.18293
10584	BZLF1	TNNC2	0.048831
10585	BZLF1	GNA12	0.080939
10586	BZLF1	ZC3H14	0.20297
10587	BZLF1	LSM11	0.16304
10588	BZLF1	BUB1	0.046086
10589	BZLF1	RIT1	0.16523
10590	BZLF1	KCNA4	0.1487
10591	BZLF1	RIOK3	0.16446
10592	BZLF1	KISS1	0.21561
10593	BZLF1	DMC1	0.088519
10594	BZLF1	ATP5A1	0.11812
10595	BZLF1	RAB21	0.16007
10596	BZLF1	SGPL1	0.031611
10597	BZLF1	ADAR	0.14695
10598	BZLF1	OIP5	0.13887
10599	BZLF1	GAD1	0.09244
10600	BZLF1	WDTC1	0.11373
10601	BZLF1	SNX19	0.35903
10602	BZLF1	MAP2K4	0.074939
10603	BZLF1	ASPH	0.10952
10604	BZLF1	CREB1	0.011857
10605	BZLF1	LIN28B	0.14428
10606	BZLF1	TUBGCP2	0.1187
10607	BZLF1	HIP1	0.062406
10608	BZLF1	MBD3	0.11948

BZLF1	ITGA4	0.13165
BZLF1	FSTL1	0.036979
BZLF1	SRC	0.022812
BZLF1	MRE11A	0.073766
BZLF1	USP47	0.13567
BZLF1	CALR3	0.017357
BZLF1	CLPP	0.24294
BZLF1	CHORDC1	0.15532
BZLF1	SMG6	0.070502
BZLF1	ID1	0.035295
BZLF1	TRPC1	0.10438
BZLF1	TOM1	0.17794
BZLF1	PITPNC1	0.22654
BZLF1	PDGFRB	0.048993
BZLF1	NDUFB10	0.19294
BZLF1	KIF2C	0.068237
BZLF1	CACNA1B	0.11829
BZLF1	SEPT5	0.027923
BZLF1	PAX8	0.012997
BZLF1	APPBP2	0.19476
BZLF1	C1QTNF3	0.13626
BZLF1	GAD2	0.099628
BZLF1	WNK1	0.11272
BZLF1	DACT1	0.089442
BZLF1	PCNA	0.025049
BZLF1	DDRGK1	0.22949
BZLF1	EPO	0.070696
BZLF1	CD274	0.16761
BZLF1	ITSN1	0.16688
BZLF1	ATP7B	0.043557
BZLF1	PTPRD	0.01641
BZLF1	KDM4A	0.10829
BZLF1	TRAM2	0.20673
BZLF1	CEBPB	0.023803
BZLF1	BRCC3	0.10263
BZLF1	BBS1	0.1734
BZLF1	SLC22A4	0.14032
BZLF1	ETV7	0.054087
BZLF1	HNRNPF	0.15635
BZLF1	CCDC141	0.23617
BZLF1	PCDHGA12	0.049103
BZLF1	ELOVL1	0.13847
BZLF1	PRDX3	0.070252
BZLF1	DST	0.16188
BZLF1	HIF1AN	0.093578
BZLF1	COL18A1	0.06398
BZLF1	OGG1	0.056605
BZLF1	MYL6	0.3813
BZLF1	COMP	0.31323
BZLF1	RNF31	0.096036
BZLF1	EIF3G	0.23351
BZLF1	MLLT6	0.14543
BZLF1	CCDC47	0.37177
BZLF1	SNAP47	0.20174
BZLF1	EXOSC7	0.16974
BZLF1	LIMD1	0.067326
BZLF1	MX1	0.08382
BZLF1	SATB1	0.036039
BZLF1	RIN1	0.18155
BZLF1	RCOR1	0.096472
BZLF1	ANXA7	0.283
BZLF1	SON	0.1461
BZLF1	MAP3K7CL	0.19786
BZLF1	RNF139	0.097208
BZLF1	PRC1	0.03046
BZLF1	CDH26	0.049103
BZLF1	FRK	0.11949
BZLF1	CHP2	0.34144

BZLF1	ADNP	0.021271
BZLF1	TOX3	0.14315
BZLF1	SPG11	0.22771
BZLF1	PSMA7	0.041732
BZLF1	ZFP36L1	0.024647
BZLF1	CD97	0.024897
BZLF1	ABCC2	0.17649
BZLF1	PTPRB	0.043099
BZLF1	POM121C	0.16326
BZLF1	MUC1	0.10937
BZLF1	RANBP3	0.14839
BZLF1	CTNNA1	0.053519
BZLF1	VBP1	0.12238
BZLF1	MRVI1	0.14752
BZLF1	UBE2L6	0.15226
BZLF1	ZHX3	0.041784
BZLF1	RSPO1	0.12065
BZLF1	GOLPH3	0.16253
BZLF1	PLN	0.13934
BZLF1	CDK5R1	0.051092
BZLF1	SUB1	0.12321
BZLF1	ANP32A	0.12872
BZLF1	LTBP2	0.04294
BZLF1	PPM1F	0.050913
BZLF1	AKT3	0.021938
BZLF1	TEAD2	0.060824
BZLF1	PLOD3	0.13328
BZLF1	TOLLIP	0.17361
BZLF1	EIF4EBP2	0.17312
BZLF1	RUVBL1	0.065121
BZLF1	LRP12	0.17159
BZLF1	NDUFS7	0.036177
BZLF1	NECAB2	0.47996
BZLF1	ELF3	0.03531
BZLF1	OTX2	0.010367
BZLF1	OGT	0.07401
BZLF1	GTF3C4	0.17387
BZLF1	PBRM1	0.14179
BZLF1	NCOR1	0.026452
BZLF1	APITD1	0.095814
BZLF1	HGF	0.082688
BZLF1	FAM103A1	0.23592
BZLF1	DNAJB9	0.15261
BZLF1	AVEN	0.20258
BZLF1	SLBP	0.14547
BZLF1	USH2A	0.12385
BZLF1	PHF2	0.11351
BZLF1	PODXL2	0.1957
BZLF1	APLP1	0.03466
BZLF1	FBP1	0.045884
BZLF1	RFC2	0.072302
BZLF1	TYROBP	0.097037
BZLF1	CANX	0.20092
BZLF1	HNF1A	0.017649
BZLF1	RHOT2	0.20466
BZLF1	DOCK9	0.090405
BZLF1	RTKN	0.10561
BZLF1	EYA3	0.020925
BZLF1	KLHL3	0.089381
BZLF1	EHMT1	0.16799
BZLF1	KDR	0.02489
BZLF1	SNAP23	0.1704
BZLF1	MMP15	0.23762
BZLF1	MKNK1	0.078149
BZLF1	TTC8	0.18007
BZLF1	PDE6D	0.26152
BZLF1	ASCC3	0.10467
BZLF1	CCNDBP1	0.19149

BZLF1	PPP2R5B	0.12904
BZLF1	GADD45A	0.14433
BZLF1	PLCB3	0.028392
BZLF1	SNX15	0.01221
BZLF1	LCP2	0.18225
BZLF1	ORAI2	0.2221
BZLF1	SUSD1	0.054178
BZLF1	APBA1	0.11929
BZLF1	DKK1	0.080194
BZLF1	ANXA11	0.24869
BZLF1	SPINK9	0.20268
BZLF1	ITM2C	0.14917
BZLF1	USP54	0.22154
BZLF1	PPP3CB	0.12214
BZLF1	TAB2	0.067488
BZLF1	SNRPB	0.10746
BZLF1	KRT19	0.13848
BZLF1	PCBD2	0.10908
BZLF1	S100G	0.053198
BZLF1	PRKAA1	0.011844
BZLF1	ATG7	0.090126
BZLF1	MDM4	0.098487
BZLF1	SCYL2	0.075007
BZLF1	NCKIPSD	0.21642
BZLF1	SPINK7	0.20902
BZLF1	NQO1	0.16996
BZLF1	NFATC2	0.020319
BZLF1	ACSS1	0.048388
BZLF1	ACACB	0.11988
BZLF1	RBM39	0.16585
BZLF1	LURAP1L	0.010274
BZLF1	IGF2BP3	0.15334
BZLF1	TRIM27	0.051806
BZLF1	CSPG5	0.068503
BZLF1	MC3R	0.1372
BZLF1	PAX2	0.018227
BZLF1	CNOT4	0.11055
BZLF1	CDK12	0.015036
BZLF1	DLG2	0.11886
BZLF1	ZNF136	0.10857
BZLF1	MAST1	0.073689
BZLF1	MAU2	0.17355
BZLF1	SH3PXD2B	0.11572
BZLF1	UBN1	0.05108
BZLF1	C12orf50	0.010062
BZLF1	IQGAP1	0.13003
BZLF1	FAAP24	0.14284
BZLF1	HIRA	0.059405
BZLF1	ATP6V0C	0.12475
BZLF1	STAP2	0.053974
BZLF1	EPAS1	0.012126
BZLF1	CCNA2	0.074764
BZLF1	FZD1	0.041407
BZLF1	ELAVL1	0.10735
BZLF1	RAD51	0.046385
BZLF1	BMI1	0.052148
BZLF1	OPTN	0.15738
BZLF1	TMED1	0.12542
BZLF1	C19orf60	0.23617
BZLF1	RAB9A	0.066111
BZLF1	TIMP1	0.10949
BZLF1	MATN3	0.45266
BZLF1	SKA1	0.11495
BZLF1	EFCAB8	0.057416
BZLF1	ATL2	0.079381
BZLF1	NR2F2	0.011157
BZLF1	RAP1A	0.09493
BZLF1	MAST2	0.06156

BZLF1	SUPT20H	0.18553
BZLF1	SH3BP2	0.11528
BZLF1	YEATS4	0.073889
BZLF1	SFN	0.062502
BZLF1	FBN3	0.069724
BZLF1	G0S2	0.18227
BZLF1	RPL27A	0.18663
BZLF1	MDC1	0.1614
BZLF1	PGRMC1	0.16358
BZLF1	NHP2L1	0.18368
BZLF1	SH3KBP1	0.20921
BZLF1	AMOTL1	0.21057
BZLF1	MYLIP	0.095107
BZLF1	TENC1	0.10347
BZLF1	STK3	0.040022
BZLF1	DLGAP4	0.17633
BZLF1	TOMM70A	0.13344
BZLF1	TCERG1	0.14864
BZLF1	PCDH8	0.035819
BZLF1	UBE4A	0.18722
BZLF1	GTF3C5	0.18029
BZLF1	LRRK2	0.020793
BZLF1	TSC1	0.062532
BZLF1	CPNE6	0.066806
BZLF1	CD276	0.1113
BZLF1	RNF41	0.078082
BZLF1	BASP1	0.078848
BZLF1	RASSF2	0.18547
BZLF1	TJAP1	0.19323
BZLF1	ETV6	0.054289
BZLF1	DARS	0.11992
BZLF1	SCHIP1	0.19134
BZLF1	RET	0.1476
BZLF1	OS9	0.17923
BZLF1	ARHGAP26	0.065606
BZLF1	COL1A2	0.094052
BZLF1	LSM7	0.14778
BZLF1	SRY	0.049283
BZLF1	JAM2	0.17811
BZLF1	MAFB	0.040807
BZLF1	ALOX5	0.09464
BZLF1	SOD1	0.021806
BZLF1	WASH1	0.15557
BZLF1	SPRED1	0.045942
BZLF1	PARP1	0.055802
BZLF1	TAF10	0.019782
BZLF1	CLCF1	0.13783
BZLF1	CHEK1	0.05981
BZLF1	FCRL4	0.2154
BZLF1	SF3B4	0.21348
BZLF1	EPS8	0.20264
BZLF1	PATL1	0.13718
BZLF1	TRAF5	0.10289
BZLF1	PARP10	0.20753
BZLF1	WBP11	0.15349
BZLF1	NSMCE4A	0.19933
BZLF1	SNTA1	0.10929
BZLF1	ATP11A	0.10841
BZLF1	IRF2	0.016516
BZLF1	CDH22	0.049103
BZLF1	THBS4	0.037917
BZLF1	FBXL3	0.1614
BZLF1	DDX1	0.072559
BZLF1	MC4R	0.12252
BZLF1	CDHR4	0.049103
BZLF1	CHD1L	0.14286
BZLF1	RPP25	0.2114
BZLF1	SLC3A2	0.098322

BZLF1	UBQLN1	0.1332
BZLF1	USP13	0.080677
BZLF1	SLC6A3	0.096661
BZLF1	CALCR	0.078285
BZLF1	PTGFRN	0.18681
BZLF1	EIF4EBP3	0.17811
BZLF1	PTPN11	0.02857
BZLF1	CPSF1	0.19066
BZLF1	FLVCR1	0.13918
BZLF1	MS4A3	0.20264
BZLF1	HOXA5	0.013815
BZLF1	HCFC1	0.035027
BZLF1	FLNB	0.13142
BZLF1	TMEM66	0.16896
BZLF1	PICK1	0.070544
BZLF1	MSLN	0.17779
BZLF1	RAB11FIP5	0.080299
BZLF1	SUZ12	0.10056
BZLF1	CDH23	0.1979
BZLF1	KIT	0.025204
BZLF1	STATH	0.17934
BZLF1	UBC	0.027861
BZLF1	RAB11FIP4	0.37909
BZLF1	RLF	0.097246
BZLF1	KRT18	0.097011
BZLF1	FGF13	0.08399
BZLF1	NTM	0.17644
BZLF1	PDCD6IP	0.15845
BZLF1	GAB1	0.077031
BZLF1	HSFX1	0.056615
BZLF1	RARRES2	0.15911
BZLF1	CNGC19	0.01207
BZLF1	HSPB1	0.023512
BZLF1	BMPR1A	0.02387
BZLF1	MYCBPAP	0.15498
BZLF1	MEF2D	0.050748
BZLF1	PSMA2	0.05351
BZLF1	ASXL1	0.10211
BZLF1	APBB2	0.068979
BZLF1	TP53BP1	0.074795
BZLF1	MAP1LC3A	0.15407
BZLF1	UBXN1	0.13486
BZLF1	PRPF3	0.18487
BZLF1	ATP6V1H	0.1382
BZLF1	CDH12	0.045044
BZLF1	PHYH	0.070399
BZLF1	JMJD6	0.081409
BZLF1	RRAGA	0.102
BZLF1	HSPB2	0.16968
BZLF1	PIGT	0.13551
BZLF1	EIF3A	0.10634
BZLF1	MGARP	0.11413
BZLF1	NFKB2	0.024827
BZLF1	UBE2I	0.02658
BZLF1	CASP7	0.084082
BZLF1	H2AFX	0.085726
BZLF1	RHOU	0.045576
BZLF1	SSRP1	0.13676
BZLF1	SUMO3	0.20889
BZLF1	TLR2	0.026878
BZLF1	ATF3	0.038466
BZLF1	SOS2	0.065413
BZLF1	SYNE2	0.086063
BZLF1	BCL2L2	0.17899
BZLF1	SUV39H2	0.16366
BZLF1	IL10RA	0.18885
BZLF1	CLIC5	0.096903
BZLF1	CTBP1	0.010515

BZLF1	UBE2F	0.12131
BZLF1	C6orf223	0.011257
BZLF1	AGTR2	0.072968
BZLF1	IFIH1	0.070317
BZLF1	IER3	0.13986
BZLF1	CNKSR1	0.10248
BZLF1	LDHAL6B	0.18965
BZLF1	MAGEA1	0.15257
BZLF1	MBOAT7	0.19133
BZLF1	FOCAD	0.20949
BZLF1	LOX	0.12007
BZLF1	ELP4	0.12132
BZLF1	ELTD1	0.030367
BZLF1	CAV2	0.069654
BZLF1	FXR2	0.2931
BZLF1	POLR2B	0.069413
BZLF1	VANGL1	0.21539
BZLF1	MPP1	0.16704
BZLF1	TRIP6	0.12232
BZLF1	PKP4	0.12643
BZLF1	COMMD4	0.22903
BZLF1	AGGF1	0.16591
BZLF1	ORC5	0.10775
BZLF1	CLU	0.043561
BZLF1	PPIB	0.18798
BZLF1	PRRG1	0.050958
BZLF1	NRG1	0.027698
BZLF1	A1CF	0.14298
BZLF1	NIFK	0.18844
BZLF1	ATXN7	0.13898
BZLF1	UBA6	0.096462
BZLF1	TDP2	0.11002
BZLF1	SUDS3	0.17525
BZLF1	CHCHD3	0.14606
BZLF1	PRKACB	0.024729
BZLF1	MLANA	0.17744
BZLF1	LPHN1	0.12936
BZLF1	BRINP1	0.19476
BZLF1	WIPF2	0.21539
BZLF1	SNX5	0.016811
BZLF1	MCM2	0.046827
BZLF1	PLCE1	0.109
BZLF1	CD163	0.18893
BZLF1	IL10RB	0.18748
BZLF1	DYNLL1	0.089236
BZLF1	PIDD	0.18895
BZLF1	ADAMTSL4	0.12678
BZLF1	MYOT	0.17274
BZLF1	PDCD1	0.17332
BZLF1	HNRNPLL	0.19531
BZLF1	SDCCAG8	0.16842
BZLF1	IL12B	0.024673
BZLF1	WDR48	0.13257
BZLF1	PDCD11	0.1791
BZLF1	G3BP1	0.13684
BZLF1	RASSF1	0.077369
BZLF1	NELFCD	0.14925
BZLF1	KHDRBS1	0.14565
BZLF1	EID1	0.12211
BZLF1	PCDHB4	0.038452
BZLF1	IGHMBP2	0.027328
BZLF1	SNX3	0.19114
BZLF1	OLA1	0.12983
BZLF1	ATP6V1D	0.13839
BZLF1	TDP1	0.15719
BZLF1	ABL2	0.079197
BZLF1	DOCK3	0.20243
BZLF1	STX6	0.12004

BZLF1 MAK 0.0321
BZLF1 CYLD 0.033214
BZLF1 GP6 0.16539
BZLF1 SNW1 0.11622
BZLF1 PPL 0.14721
BZLF1 KLK3 0.2512
BZLF1 SGIP1 0.075781
BZLF1 WAC 0.14275
BZLF1 TTR 0.19769
BZLF1 ATP2B4 0.12562
BZLF1 BBS7 0.1658
BZLF1 CRYAA 0.090159
BZLF1 NEDD4L 0.081349
BZLF1 PCDH9 0.045167
BZLF1 PPP6R2 0.18336
BZLF1 HPCAL1 0.057416
BZLF1 MIS12 0.14049
BZLF1 ARF4 0.11846
BZLF1 ZNF274 0.03855
BZLF1 DAPK1 0.11097
BZLF1 SOX6 0.017872
BZLF1 ITGA3 0.16382
BZLF1 COG3 0.11503
BZLF1 TNIK 0.060683
BZLF1 BRD7 0.059909
BZLF1 RNASE1 0.19385
BZLF1 NSF 0.087786
BZLF1 RBBP7 0.099278
BZLF1 PTRF 0.11115
BZLF1 ARIH1 0.14444
BZLF1 ECM1 0.1055
BZLF1 KLC2 0.1649
BZLF1 BEGAIN 0.2221
BZLF1 ARF6 0.066269
BZLF1 PAAF1 0.20969
BZLF1 SNX21 0.0118
BZLF1 FLCN 0.18548
BZLF1 KISS1R 0.10976
BZLF1 DNASE1L3 0.030042
BZLF1 RIPK2 0.014764
BZLF1 SNRPD3 0.10532
BZLF1 CLEC2A 0.21919
BZLF1 CTSB 0.043871
BZLF1 ILKAP 0.097419
BZLF1 CLIC4 0.15292
BZLF1 RAB14 0.033483
BZLF1 STX12 0.13451
BZLF1 SAMHD1 0.14089
BZLF1 ACTB 0.030918
BZLF1 EIF4G1 0.17449
BZLF1 SLC22A5 0.14478
BZLF1 KIF3A 0.091071
BZLF1 SCNN1A 0.15722
BZLF1 CGREF1 0.046168
BZLF1 CHAF1B 0.11778
BZLF1 RUSC1 0.15536
BZLF1 NPNT 0.053048
BZLF1 PEX12 0.14296
BZLF1 FLOT1 0.14691
BZLF1 BNIP2 0.27574
BZLF1 GABARAPL2 0.1776
BZLF1 PPIH 0.17267
BZLF1 RARS 0.046953
BZLF1 PRKDC 0.028899
BZLF1 JAG2 0.24424
BZLF1 RGR 0.16496
BZLF1 MYRIP 0.16523
BZLF1 CTTN 0.19532

BZLF1	C1QA	0.19433
BZLF1	PPP1R16A	0.22211
BZLF1	EFCAB7	0.057416
BZLF1	TACC1	0.12949
BZLF1	CREB3	0.012808
BZLF1	LTBP3	0.043196
BZLF1	PLK4	0.083668
BZLF1	SKIV2L2	0.10978
BZLF1	ADRB2	0.031465
BZLF1	DDX18	0.19155
BZLF1	ATP2B1	0.14183
BZLF1	POLR1B	0.10213
BZLF1	VIMP	0.082592
BZLF1	KMT2D	0.12665
BZLF1	CEP97	0.21195
BZLF1	PTPRU	0.058887
BZLF1	TPCN2	0.034102
BZLF1	RUNX1T1	0.035843
BZLF1	CENPI	0.10468
BZLF1	SHC3	0.17208
BZLF1	LGALS3	0.14208
BZLF1	FCGR2B	0.18617
BZLF1	IST1	0.28967
BZLF1	MAP4K5	0.10434
BZLF1	FCHO2	0.15408
BZLF1	TIPRL	0.22183
BZLF1	SKP2	0.12847
BZLF1	PLAA	0.20879
BZLF1	NR1H4	0.010711
BZLF1	DUSP15	0.024886
BZLF1	MIB2	0.12
BZLF1	EHD2	0.21554
BZLF1	PVRL2	0.080507
BZLF1	HS3ST3A1	0.035519
BZLF1	COPS5	0.13056
BZLF1	RNF135	0.14936
BZLF1	CCNE2	0.065691
BZLF1	C5AR2	0.15713
BZLF1	CADPS	0.3563
BZLF1	JUND	0.032097
BZLF1	ABCA1	0.024629
BZLF1	MARK2	0.063098
BZLF1	CNTROB	0.17164
BZLF1	CDC42SE2	0.013837
BZLF1	HEXIM2	0.14548
BZLF1	HRNR	0.038449
BZLF1	PKD2	0.027671
BZLF1	NCAPH	0.17195
BZLF1	CREBRF	0.052323
BZLF1	AURKA	0.01376
BZLF1	PCDHGB2	0.049103
BZLF1	BBS4	0.038283
BZLF1	PDZD7	0.19151
BZLF1	C17orf59	0.012352
BZLF1	PPP2CB	0.044668
BZLF1	DLC1	0.06877
BZLF1	STXBP4	0.024985
BZLF1	IPO9	0.16467
BZLF1	ANXA8L1	0.035342
BZLF1	PDIA6	0.06568
BZLF1	HDAC5	0.021241
BZLF1	TRPC6	0.1161
BZLF1	ZNF655	0.10121
BZLF1	ETF1	0.19811
BZLF1	DCSTAMP	0.10668
BZLF1	EMILIN1	0.17261
BZLF1	CRYGD	0.19011
BZLF1	ADRM1	0.14129

BZLF1	KLK6	0.14923
BZLF1	FAM161B	0.011189
BZLF1	CCAR1	0.31898
BZLF1	PFDN4	0.14609
BZLF1	C19orf57	0.21682
BZLF1	LPCAT1	0.01066
BZLF1	HIST1H3A	0.10605
BZLF1	CCNA1	0.075566
BZLF1	MYCN	0.037379
BZLF1	NKX2-1	0.01141
BZLF1	SPARCL1	0.063567
BZLF1	PDZD3	0.11388
BZLF1	CD40	0.048341
BZLF1	ENPP1	0.045859
BZLF1	PPP1R12A	0.079271
BZLF1	ERC1	0.087187
BZLF1	CTNND1	0.070895
BZLF1	PICALM	0.051309
BZLF1	PLEKHG6	0.06088
BZLF1	GUCA1A	0.071599
BZLF1	SEMA7A	0.14092
BZLF1	CRMP1	0.042418
BZLF1	LITAF	0.13345
BZLF1	TGFA	0.094608
BZLF1	IQCB1	0.2458
BZLF1	CCAR2	0.20454
BZLF1	CACTIN	0.135
BZLF1	PLAUR	0.097806
BZLF1	UTP20	0.1466
BZLF1	DVL1	0.039896
BZLF1	GINS2	0.17469
BZLF1	WDR66	0.057416
BZLF1	LOXL2	0.046231
BZLF1	CASP3	0.052248
BZLF1	SHC1	0.044394
BZLF1	MITF	0.028023
BZLF1	ZNF217	0.016444
BZLF1	MPHOSPH6	0.2162
BZLF1	GTF2A1	0.097904
BZLF1	NLGN4Y	0.15433
BZLF1	SLC2A2	0.014071
BZLF1	ELF1	0.056795
BZLF1	COPS3	0.17881
BZLF1	ZMYND8	0.14544
BZLF1	COMMD6	0.17825
BZLF1	LONRF3	0.17405
BZLF1	GABARAP	0.1443
BZLF1	WWP2	0.050706
BZLF1	C1orf195	0.014661
BZLF1	TGFB1I1	0.088841
BZLF1	PIKFYVE	0.13206
BZLF1	MEX3B	0.044908
BZLF1	FBXW9	0.23617
BZLF1	CD37	0.20276
BZLF1	STAM	0.15524
BZLF1	GSK3A	0.03234
BZLF1	CBLB	0.14143
BZLF1	CRELD2	0.43799
BZLF1	PPP2R5A	0.090877
BZLF1	BLOC1S2	0.10958
BZLF1	MYOZ1	0.18197
BZLF1	TMEM30A	0.20299
BZLF1	PRUNE	0.16373
BZLF1	NCOA3	0.09374
BZLF1	MUM1	0.20662
BZLF1	TIMELESS	0.13443
BZLF1	NEK9	0.018542
BZLF1	OSBPL3	0.15993

BZLF1	NIF3L1	0.15897
BZLF1	MZF1	0.040265
BZLF1	BANF1	0.14607
BZLF1	KXD1	0.22093
BZLF1	CPSF6	0.1682
BZLF1	MAST3	0.10731
BZLF1	FKBP14	0.055889
BZLF1	TOX4	0.1707
BZLF1	ARHGAP44	0.18185
BZLF1	ORC1	0.092355
BZLF1	LEO1	0.13101
BZLF1	CSRP2BP	0.12702
BZLF1	RORA	0.027564
BZLF1	ARL14EP	0.22903
BZLF1	NSMCE1	0.12977
BZLF1	MED7	0.11378
BZLF1	WAPAL	0.097965
BZLF1	TNFSF10	0.082946
BZLF1	GP1BB	0.16848
BZLF1	REL	0.048538
BZLF1	PCDHB10	0.044991
BZLF1	BLCAP	0.20892
BZLF1	AIMP2	0.14884
BZLF1	APOA1BP	0.18294
BZLF1	RPS7	0.12817
BZLF1	LILRB2	0.11163
BZLF1	PFKFB1	0.090706
BZLF1	URI1	0.093854
BZLF1	EME1	0.14763
BZLF1	APPL2	0.074345
BZLF1	RBM26	0.22126
BZLF1	PSMG1	0.21579
BZLF1	SEC61B	0.1095
BZLF1	KIF2A	0.085656
BZLF1	PATL2	0.18507
BZLF1	IL7	0.10683
BZLF1	SEPT14	0.17735
BZLF1	NBR1	0.21235
BZLF1	SENP6	0.14319
BZLF1	EFCAB13	0.029807
BZLF1	HFE	0.1563
BZLF1	DPM1	0.12237
BZLF1	SF3A3	0.12387
BZLF1	IFT20	0.16144
BZLF1	GHR	0.021838
BZLF1	FBXW7	0.042453
BZLF1	MAGEA6	0.21685
BZLF1	RAB24	0.16668
BZLF1	RAB8A	0.12141
BZLF1	PFKL	0.10943
BZLF1	CXADR	0.040974
BZLF1	RAB11FIP2	0.10579
BZLF1	TRO	0.16368
BZLF1	ATP11C	0.10817
BZLF1	CD27	0.13429
BZLF1	LYST	0.10378
BZLF1	CASQ1	0.037561
BZLF1	ZNF652	0.11682
BZLF1	C1orf87	0.057416
BZLF1	CEP57	0.11054
BZLF1	SCIMP	0.18026
BZLF1	TIPIN	0.14338
BZLF1	CHIC2	0.23585
BZLF1	BNIP3L	0.056733
BZLF1	AES	0.099076
BZLF1	STX18	0.14232
BZLF1	VAMP7	0.089555
BZLF1	N4BP2	0.16524

BZLF1	SDC1	0.066765
BZLF1	TRAM1	0.14812
BZLF1	DCAF8	0.2289
BZLF1	DNM1L	0.019851
BZLF1	DLGAP3	0.15176
BZLF1	USP9X	0.12271
BZLF1	PDIA2	0.11795
BZLF1	DERL1	0.12428
BZLF1	RSF1	0.081046
BZLF1	SMG8	0.18686
BZLF1	GSN	0.18711
BZLF1	RELB	0.056704
BZLF1	SLC11A2	0.04338
BZLF1	FAM96B	0.14807
BZLF1	TRMT112	0.27883
BZLF1	NUP133	0.10733
BZLF1	PCDHGA4	0.048194
BZLF1	PAIP2	0.19881
BZLF1	NABP2	0.16218
BZLF1	GCSAM	0.19061
BZLF1	AMBRA1	0.24675
BZLF1	ADCY4	0.059648
BZLF1	MET	0.032048
BZLF1	TREML2	0.19728
BZLF1	PKP2	0.11383
BZLF1	VPS13A	0.18286
BZLF1	BMP2	0.010474
BZLF1	MME	0.032101
BZLF1	CEBPA	0.019404
BZLF1	CSNK1D	0.047651
BZLF1	HADHA	0.071331
BZLF1	PCDHGA3	0.049103
BZLF1	TBKBP1	0.23549
BZLF1	PPP1R27	0.20943
BZLF1	EIF4H	0.22157
BZLF1	HK1	0.12678
BZLF1	BPY2	0.20166
BZLF1	TELO2	0.16612
BZLF1	FRMPD2	0.18883
BZLF1	CCNB3	0.077284
BZLF1	PVRL1	0.11549
BZLF1	IGFBP3	0.097509
BZLF1	CYCB3-1	0.010632
BZLF1	ASCL1	0.011545
BZLF1	UBA2	0.014423
BZLF1	IGFBP7	0.18479
BZLF1	S100A16	0.031292
BZLF1	BLNK	0.16767
BZLF1	RPGRIP1L	0.10164
BZLF1	ILF2	0.14884
BZLF1	POT1	0.086488
BZLF1	GDI1	0.16045
BZLF1	PPFIA3	0.20894
BZLF1	BMPR1B	0.03498
BZLF1	PAM	0.08462
BZLF1	CNTRL	0.17894
BZLF1	CDK13	0.013939
BZLF1	GOLGA2	0.20976
BZLF1	PHACTR2	0.2212
BZLF1	TMEM97	0.14722
BZLF1	TRPC5	0.13553
BZLF1	SP100	0.070382
BZLF1	EIF4A1	0.17577
BZLF1	TDRD7	0.17731
BZLF1	MRPS12	0.14209
BZLF1	CD24	0.051841
BZLF1	CABP1	0.21739
BZLF1	RHNO1	0.18707

BZLF1	DDX5	0.074606
BZLF1	PALD1	0.22256
BZLF1	RBBP5	0.14259
BZLF1	PILRA	0.1846
BZLF1	YBX1	0.038552
BZLF1	EAF1	0.17818
BZLF1	AKAP1	0.064644
BZLF1	SCEL	0.15936
BZLF1	SLC4A4	0.19183
BZLF1	PKN3	0.081854
BZLF1	ARF5	0.12014
BZLF1	LETM1	0.42409
BZLF1	CRH	0.085992
BZLF1	EIF2AK2	0.059949
BZLF1	FSTL5	0.055781
BZLF1	NDRG1	0.10927
BZLF1	CINP	0.19957
BZLF1	TPR	0.094462
BZLF1	NEFM	0.14106
BZLF1	LEMD3	0.1089
BZLF1	BAG3	0.17309
BZLF1	SPHK1	0.092511
BZLF1	PACSIN3	0.16673
BZLF1	VAPA	0.050027
BZLF1	SCARF2	0.19577
BZLF1	MAPRE1	0.10563
BZLF1	CATSPER2	0.11734
BZLF1	GCA	0.069585
BZLF1	FNIP1	0.22188
BZLF1	TCTEX1D2	0.23617
BZLF1	VRK2	0.05687
BZLF1	SMG1	0.098514
BZLF1	ARAF	0.068514
BZLF1	SRSF10	0.15571
BZLF1	GPC3	0.10384
BZLF1	LRRC3	0.2221
BZLF1	RFC1	0.0533
BZLF1	PPP4R1	0.17605
BZLF1	TSSK2	0.06645
BZLF1	CUTA	0.20282
BZLF1	ELL	0.14239
BZLF1	CTU1	0.2316
BZLF1	AKT2	0.010658
BZLF1	PIAS4	0.082616
BZLF1	BCAS3	0.18943
BZLF1	ANGPT2	0.050139
BZLF1	MAD2L1BP	0.16716
BZLF1	ACTR1B	0.15637
BZLF1	PIK3CB	0.096508
BZLF1	RHOB	0.041405
BZLF1	EFHD2	0.074712
BZLF1	CYP4F2	0.026842
BZLF1	FBF1	0.16357
BZLF1	L3MBTL1	0.013911
BZLF1	MRPL12	0.23416
BZLF1	NFYC	0.034442
BZLF1	GTF2F1	0.048931
BZLF1	PKN1	0.026699
BZLF1	IL1RL1	0.12953
BZLF1	NSMAF	0.18844
BZLF1	NCAN	0.017548
BZLF1	FAM231A	0.018954
BZLF1	TFB1M	0.21379
BZLF1	NCOA5	0.17318
BZLF1	ACHE	0.049604
BZLF1	DSG3	0.041703
BZLF1	FAM124A	0.013239
BZLF1	YAP1	0.058227

BZLF1	MATR3	0.10908
BZLF1	PCDHB18	0.049103
BZLF1	PPP2CA	0.038896
BZLF1	CEP164	0.16296
BZLF1	MLLT1	0.14037
BZLF1	RCC1	0.10697
BZLF1	PABPC1	0.10197
BZLF1	PLEKHF2	0.097856
BZLF1	JMY	0.13582
BZLF1	SNTB1	0.1023
BZLF1	AMER1	0.18852
BZLF1	TRIP11	0.12819
BZLF1	RTF1	0.09167
BZLF1	RWDD3	0.19741
BZLF1	MT3	0.033242
BZLF1	LUC7L3	0.15238
BZLF1	MCM5	0.094323
BZLF1	CD28	0.098876
BZLF1	CTDSP1	0.089097
BZLF1	HLA-DRB1	0.038932
BZLF1	PNRC1	0.17318
BZLF1	ITIH1	0.048316
BZLF1	PRTN3	0.20614
BZLF1	MAL	0.18751
BZLF1	FAM188A	0.41104
BZLF1	ZP3	0.043382
BZLF1	BCKDK	0.10005
BZLF1	MCF2	0.08397
BZLF1	UMOD	0.066802
BZLF1	C6	0.16069
BZLF1	HDHD3	0.19526
BZLF1	IPO8	0.16087
BZLF1	RNF43	0.08115
BZLF1	STX2	0.1101
BZLF1	RHBDL3	0.14662
BZLF1	AP1B1	0.20095
BZLF1	CCNT1	0.051726
BZLF1	GAPDH	0.11786
BZLF1	NRD1	0.012979
BZLF1	GRB10	0.096807
BZLF1	NBN	0.03891
BZLF1	RCN3	0.46475
BZLF1	MGST1	0.066054
BZLF1	RNF5	0.10797
BZLF1	PLSCR1	0.1946
BZLF1	CETN3	0.083018
BZLF1	SNX7	0.0118
BZLF1	CTNNBL1	0.17414
BZLF1	KDM2B	0.093756
BZLF1	FDXR	0.12249
BZLF1	FLT1	0.027895
BZLF1	BCLAF1	0.12193
BZLF1	EFTUD2	0.12041
BZLF1	SERPINE2	0.03701
BZLF1	YY1AP1	0.1589
BZLF1	NF1	0.026736
BZLF1	F8	0.16118
BZLF1	FBXO45	0.17781
BZLF1	KIF21A	0.16217
BZLF1	SCAMP2	0.11304
BZLF1	PCDH10	0.049103
BZLF1	GIT1	0.1182
BZLF1	GRIN2B	0.013604
BZLF1	SLC6A4	0.088156
BZLF1	CDH19	0.047672
BZLF1	VEGFC	0.091889
BZLF1	CLK2	0.093733
BZLF1	SLC12A3	0.17511

BZLF1	SNAP25	0.050656
BZLF1	DDX23	0.10563
BZLF1	LSM10	0.15364
BZLF1	SUPV3L1	0.063452
BZLF1	PSENEN	0.12166
BZLF1	ATN1	0.13065
BZLF1	LRPPRC	0.13554
BZLF1	VAPB	0.05577
BZLF1	NHEJ1	0.14606
BZLF1	STAT5B	0.022813
BZLF1	COPRS	0.15917
BZLF1	RPA1	0.051082
BZLF1	HSPG2	0.079365
BZLF1	CELSR2	0.020198
BZLF1	SMARCA4	0.056597
BZLF1	SGCA	0.053822
BZLF1	ATG5	0.14419
BZLF1	ACTL6A	0.091806
BZLF1	PAXIP1	0.12952
BZLF1	EGFR	0.015089
BZLF1	ACTR8	0.16947
BZLF1	CAMLG	0.19763
BZLF1	SELP	0.067842
BZLF1	FBXO5	0.040792
BZLF1	PTGDR	0.18181
BZLF1	APAF1	0.098989
BZLF1	SIKE1	0.17492
BZLF1	CTCF	0.019
BZLF1	HSPD1	0.030127
BZLF1	PPFIA2	0.21537
BZLF1	PCDHGA8	0.049103
BZLF1	MST1R	0.10216
BZLF1	SCIN	0.035904
BZLF1	CCDC33	0.23617
BZLF1	NPC1	0.078791
BZLF1	SYTL5	0.17798
BZLF1	HINFP	0.02379
BZLF1	MORC4	0.16567
BZLF1	MPLKIP	0.18271
BZLF1	PLP2	0.18177
BZLF1	RAB11A	0.096241
BZLF1	LTBP1	0.42166
BZLF1	KIF5A	0.12337
BZLF1	PEX6	0.15673
BZLF1	SMARCC1	0.1127
BZLF1	TPD52L2	0.23321
BZLF1	ASPSCR1	0.15386
BZLF1	ARL6IP4	0.1974
BZLF1	F2	0.12223
BZLF1	CELF1	0.14815
BZLF1	RHOXF2	0.06051
BZLF1	WDYHV1	0.15641
BZLF1	SYT7	0.10285
BZLF1	FAAP20	0.14597
BZLF1	DLG1	0.011574
BZLF1	THOC2	0.23687
BZLF1	PLEKHA2	0.092136
BZLF1	EREG	0.070383
BZLF1	IFIT2	0.17143
BZLF1	HEATR1	0.13587
BZLF1	SCNN1B	0.15108
BZLF1	SMYD2	0.13501
BZLF1	ITPR1	0.068071
BZLF1	VAMP3	0.14301
BZLF1	WIPI2	0.13847
BZLF1	CDH9	0.04378
BZLF1	SPAST	0.079262
BZLF1	PCDHB9	0.044991

BZLF1	RRAS	0.10531
BZLF1	MIEF2	0.14505
BZLF1	FST	0.1063
BZLF1	CEP290	0.10329
BZLF1	KDM5B	0.029217
BZLF1	NOXO1	0.11727
BZLF1	SYT1	0.15746
BZLF1	NSMCE2	0.1069
BZLF1	MRPS16	0.16273
BZLF1	RND2	0.18176
BZLF1	HMOX2	0.11085
BZLF1	WRNIP1	0.12835
BZLF1	TCOF1	0.19508
BZLF1	CBL	0.13905
BZLF1	NEK3	0.10255
BZLF1	CORO1A	0.081341
BZLF1	PHF12	0.10787
BZLF1	SLX4	0.15803
BZLF1	ANKRD27	0.19064
BZLF1	EPHA2	0.047483
BZLF1	MYC	0.010385
BZLF1	ARFRP1	0.11785
BZLF1	UCHL5	0.084646
BZLF1	PRPF8	0.18202
BZLF1	VIM	0.034448
BZLF1	NOA1	0.11706
BZLF1	CTAG1A	0.22903
BZLF1	PEX26	0.16425
BZLF1	WARS	0.092206
BZLF1	TIMM10	0.11387
BZLF1	SLC25A13	0.042505
BZLF1	KLRC2	0.1909
BZLF1	NPM1	0.02064
BZLF1	GDF2	0.082915
BZLF1	NAP1L4	0.14341
BZLF1	MYCBP	0.14947
BZLF1	PI4K2A	0.14612
BZLF1	GRB2	0.015688
BZLF1	ADNP2	0.024047
BZLF1	ZNF639	0.011233
BZLF1	NCS1	0.30131
BZLF1	TCAP	0.1329
BZLF1	HPSE	0.081382
BZLF1	CDK2	0.020431
BZLF1	MBL2	0.1067
BZLF1	NCK2	0.11105
BZLF1	IKZF3	0.026361
BZLF1	NMNAT1	0.16142
BZLF1	GPKOW	0.18125
BZLF1	FBLN5	0.34623
BZLF1	MPP6	0.2154
BZLF1	ARHGAP1	0.10614
BZLF1	CRBN	0.14639
BZLF1	HSCB	0.19294
BZLF1	BIRC5	0.035842
BZLF1	DAG1	0.10037
BZLF1	AP5S1	0.16468
BZLF1	MCM3	0.06016
BZLF1	TOMM20	0.098711
BZLF1	GLTSCR1	0.23617
BZLF1	OTOF	0.05477
BZLF1	AGTRAP	0.21621
BZLF1	ID2	0.044845
BZLF1	ZNF133	0.032412
BZLF1	PACS1	0.17663
BZLF1	DOC2A	0.20902
BZLF1	MED30	0.16301
BZLF1	RHOBTB3	0.17305

BZLF1	XRCC3	0.13769
BZLF1	F11	0.18099
BZLF1	SRGAP2	0.096331
BZLF1	CALCOCO1	0.10121
BZLF1	FBXO6	0.18133
BZLF1	VPS35	0.14333
BZLF1	HRK	0.16364
BZLF1	ADAM22	0.11232
BZLF1	PCDHA12	0.049103
BZLF1	TBC1D9B	0.027662
BZLF1	PTPRT	0.028703
BZLF1	SIGLEC8	0.19651
BZLF1	SNRNP40	0.15403
BZLF1	ARID1A	0.088041
BZLF1	SCNN1D	0.19745
BZLF1	STON2	0.17424
BZLF1	SNX22	0.0118
BZLF1	CENPE	0.064204
BZLF1	HSPA8	0.020701
BZLF1	PLK2	0.066494
BZLF1	FBXW11	0.11371
BZLF1	SCAI	0.14902
BZLF1	KCNMB4	0.15173
BZLF1	NIPBL	0.085735
BZLF1	RPL7A	0.17551
BZLF1	KMT2A	0.046436
BZLF1	UBE2C	0.082828
BZLF1	SERPINA3	0.14058
BZLF1	ADRBK1	0.027718
BZLF1	DOK1	0.075483
BZLF1	IL7R	0.10494
BZLF1	MYL5	0.04829
BZLF1	TPM4	0.027682
BZLF1	NDUFAF3	0.16921
BZLF1	DYX1C1	0.1721
BZLF1	BMF	0.11411
BZLF1	INTS2	0.16457
BZLF1	RAB22A	0.11374
BZLF1	MYO7A	0.073425
BZLF1	PIAS2	0.07492
BZLF1	MUC2	0.21313
BZLF1	SPTLC1	0.10679
BZLF1	NLK	0.041983
BZLF1	ITGA6	0.1484
BZLF1	PILRB	0.2023
BZLF1	CTNNA2	0.076475
BZLF1	DEPTOR	0.19691
BZLF1	FTH1	0.13183
BZLF1	NOD1	0.039855
BZLF1	RCAN1	0.045795
BZLF1	DGKB	0.069937
BZLF1	ZFYVE9	0.20935
BZLF1	PDS5A	0.17873
BZLF1	TIGIT	0.14506
BZLF1	CARS	0.063592
BZLF1	RNF8	0.066501
BZLF1	DPEP1	0.076402
BZLF1	RFX5	0.058062
BZLF1	C8orf48	0.23617
BZLF1	NPHS1	0.12471
BZLF1	TAL1	0.011261
BZLF1	NECAB3	0.34722
BZLF1	AKAP8L	0.1479
BZLF1	SNX12	0.23801
BZLF1	ATM	0.03994
BZLF1	TCHH	0.038461
BZLF1	RASSF9	0.18259
BZLF1	GORASP2	0.16632

BZLF1	KIR2DL4	0.18513
BZLF1	MLC1	0.15122
BZLF1	LBP	0.068472
BZLF1	TRIM16	0.10156
BZLF1	IFI35	0.19837
BZLF1	EDA	0.04922
BZLF1	PXMP4	0.20268
BZLF1	ASGR2	0.17009
BZLF1	STAG1	0.14021
BZLF1	EFNA4	0.25207
BZLF1	SIN3A	0.012839
BZLF1	DYNC1H1	0.11463
BZLF1	GRM7	0.020576
BZLF1	CNTNAP1	0.091784
BZLF1	PBXIP1	0.12714
BZLF1	RRAGD	0.14692
BZLF1	TRMT61B	0.25973
BZLF1	CCR1	0.090031
BZLF1	SDC4	0.12819
BZLF1	LONP2	0.10916
BZLF1	HSPA14	0.16955
BZLF1	PLXNB2	0.14854
BZLF1	APH1B	0.17225
BZLF1	SNX6	0.22052
BZLF1	APOA5	0.067901
BZLF1	KRT1	0.11489
BZLF1	ZNF143	0.091933
BZLF1	BRCA1	0.017696
BZLF1	RFX1	0.14827
BZLF1	CDK20	0.050479
BZLF1	KRT81	0.16448
BZLF1	ERGIC1	0.13523
BZLF1	RALB	0.1064
BZLF1	CDC73	0.09886
BZLF1	GSE1	0.23617
BZLF1	NCOA1	0.066922
BZLF1	FGA	0.14777
BZLF1	RASGRP1	0.013026
BZLF1	HLTF	0.055429
BZLF1	DUSP7	0.020638
BZLF1	PDE4A	0.079591
BZLF1	ZFYVE1	0.11462
BZLF1	IFNAR2	0.082902
BZLF1	NMI	0.18083
BZLF1	CDK5RAP3	0.099404
BZLF1	PCDHGB6	0.049103
BZLF1	WWTR1	0.083851
BZLF1	PCDHB11	0.042302
BZLF1	ASF1A	0.11215
BZLF1	ERRFI1	0.042778
BZLF1	C19orf25	0.23617
BZLF1	IRF7	0.013306
BZLF1	MAPK9	0.054672
BZLF1	BBS2	0.10585
BZLF1	APOA2	0.029497
BZLF1	XPA	0.097914
BZLF1	CHMP5	0.20355
BZLF1	EIF2S2	0.23494
BZLF1	ZFYVE16	0.06938
BZLF1	NMRK2	0.22517
BZLF1	PHB2	0.12482
BZLF1	DCP1B	0.10605
BZLF1	MYB	0.041766
BZLF1	SNRPD1	0.14607
BZLF1	SOCS1	0.11225
BZLF1	C1S	0.081526
BZLF1	NPBWR1	0.15582
BZLF1	SNCB	0.027098

BZLF1	EGLN3	0.11472
BZLF1	NPC1L1	0.23341
BZLF1	PCGF2	0.033479
BZLF1	DNMBP	0.1611
BZLF1	PRAM1	0.25653
BZLF1	EFNB1	0.1521
BZLF1	NT5C3B	0.16786
BZLF1	CDYL2	0.15684
BZLF1	AASDHPPT	0.18568
BZLF1	FHL1	0.10453
BZLF1	GGA3	0.15329
BZLF1	UTP11L	0.15337
BZLF1	RSRC1	0.17042
BZLF1	GLRA1	0.05856
BZLF1	AQP5	0.17274
BZLF1	ARL4C	0.095834
BZLF1	LOR	0.065814
BZLF1	NR0B2	0.053839
BZLF1	ARHGAP21	0.15282
BZLF1	ARL3	0.036304
BZLF1	MEGF6	0.45263
BZLF1	MED20	0.099669
BZLF1	RPS6KA1	0.023524
BZLF1	STAG2	0.13234
BZLF1	EIF2S3	0.16948
BZLF1	NFRKB	0.14936
BZLF1	LAX1	0.092501
BZLF1	CALR	0.042224
BZLF1	JPH2	0.12856
BZLF1	BTBD1	0.20112
BZLF1	SOCS5	0.19009
BZLF1	EMX2	0.026669
BZLF1	MRPL11	0.1907
BZLF1	EGR1	0.023025
BZLF1	MC2R	0.12188
BZLF1	NUP205	0.15073
BZLF1	RAB33A	0.13625
BZLF1	DHX9	0.08206
BZLF1	HOXA10	0.041327
BZLF1	HSP90AA1	0.095004
BZLF1	TAF1	0.037053
BZLF1	GAS1	0.052866
BZLF1	TSPY1	0.1264
BZLF1	TNFRSF14	0.14031
BZLF1	LYL1	0.13638
BZLF1	NUB1	0.17838
BZLF1	NFKBIL1	0.17172
BZLF1	CBX5	0.081371
BZLF1	ZFAND2B	0.1837
BZLF1	F12	0.14424
BZLF1	PCBD1	0.068844
BZLF1	NRBP1	0.06353
BZLF1	SLC25A12	0.028978
BZLF1	ATF4	0.029845
BZLF1	PCNP	0.18942
BZLF1	ATP6AP2	0.1552
BZLF1	PCDHA1	0.044854
BZLF1	ANK1	0.040216
BZLF1	PCBP1	0.18098
BZLF1	HSF1	0.04333
BZLF1	UNC5B	0.1597
BZLF1	SCRIB	0.10044
BZLF1	RPTOR	0.057741
BZLF1	SRRT	0.12793
BZLF1	SSTR3	0.11565
BZLF1	DGKG	0.065559
BZLF1	KHDRBS2	0.20277
BZLF1	MMP9	0.033424

11833	BZLF1	PLXNB3	0.17035
11834	BZLF1	KCNIP2	0.21282
11835	BZLF1	TBCD	0.14532
11836	BZLF1	THEG	0.1806
11837	BZLF1	SLPI	0.17079
11838	BZLF1	PDLIM4	0.18896
11839	BZLF1	DAO	0.1008
11840	BZLF1	PCDHA13	0.049103
11841	BZLF1	KIF3B	0.10238
11842	BZLF1	PRSS8	0.20844
11843	BZLF1	FSCN1	0.16167
11844	BZLF1	TRAF1	0.14249
11845	BZLF1	CALML3	0.057416
11846	BZLF1	POLG2	0.11693
11847	BZLF1	ITGB1BP1	0.18407
11848	BZLF1	MED25	0.090329
11849	BZLF1	RAMP3	0.12288
11850	BZLF1	AGO4	0.17395
11851	BZLF1	HPS1	0.16428
11852	BZLF1	CRK	0.12349
11853	BZLF1	RFWD2	0.10369
11854	BZLF1	SPOCK3	0.051603
11855	BZLF1	PIAS3	0.078948
11856	BZLF1	CDH4	0.032162
11857	BZLF1	TNS1	0.18531
11858	BZLF1	ITGA2B	0.12102
11859	BZLF1	PAFAH1B1	0.019865
11860	BZLF1	MAPK13	0.098671
11861	BZLF1	ADAM12	0.063928
11862	BZLF1	UBA52	0.020537
11863	BZLF1	INO80E	0.17099
11864	BZLF1	KCNIP3	0.018861
11865	BZLF1	SNRPN	0.19879
11866	BZLF1	CDK14	0.0443
11867	BZLF1	PARD6A	0.089342
11868	BZLF1	SMARCA2	0.043553
11869	BZLF1	PLCG1	0.079757
11870	BZLF1	FAM109B	0.16289
11871	BZLF1	ITGAX	0.15997
11872	BZLF1	PDCD4	0.20701
11873	BZLF1	PPP1R32	0.22359
11874	BZLF1	PPP1R8	0.14118
11875	BZLF1	NOTCH1	0.012155
11876	BZLF1	PCLO	0.037285
11877	BZLF1	IL8	0.10065
11878	BZLF1	FBN2	0.057392
11879	BZLF1	ZNHIT1	0.20707
11880	BZLF1	TRAF4	0.038075
11881	BZLF1	EYA2	0.027367
11882	BZLF1	ERCC1	0.036002
11883	BZLF1	PSME3	0.071641
11884	BZLF1	ELOVL2	0.13915
11885	BZLF1	EFCAB10	0.053924
11886	BZLF1	CD93	0.30962
11887	BZLF1	MUC7	0.20215
11888	BZLF1	SRR	0.034095
11889	BZLF1	EEF1B2	0.1655
11890	BZLF1	PPP1R2	0.19671
11891	BZLF1	TEX11	0.17393
11892	BZLF1	LMX1B	0.037026
11893	BZLF1	NLRC5	0.097902
11894	BZLF1	DRD1	0.017064
11895	BZLF1	RITA1	0.15083
11896	BZLF1	GRIN1	0.13789
11897	BZLF1	CST3	0.055459
11898	BZLF1	FAN1	0.13462
11899	BZLF1	CLINT1	0.25587
11900	BZLF1	IRF5	0.013957

BZLF1	EIF2AK3	0.039385
BZLF1	ZBTB21	0.10999
BZLF1	MARK4	0.090026
BZLF1	BBS12	0.18564
BZLF1	DHH	0.28718
BZLF1	SLC25A4	0.086576
BZLF1	SPATA21	0.057416
BZLF1	NDFIP2	0.10882
BZLF1	IK	0.18582
BZLF1	NCOA2	0.034591
BZLF1	CRIPAK	0.17324
BZLF1	PIGS	0.15757
BZLF1	SIRT2	0.031619
BZLF1	IMMT	0.18578
BZLF1	UBE2L3	0.057949
BZLF1	IL5	0.15131
BZLF1	TOMM7	0.12181
BZLF1	RRM1	0.14055
BZLF1	ILK	0.016395
BZLF1	FLT4	0.057372
BZLF1	MLF1	0.13748
BZLF1	STRN3	0.061766
BZLF1	RAP1B	0.085518
BZLF1	IKBKG	0.049637
BZLF1	SPHK2	0.18834
BZLF1	MARK3	0.11761
BZLF1	NR2F6	0.031698
BZLF1	VIPAS39	0.17539
BZLF1	ADAM15	0.11502
BZLF1	PRKCB	0.034522
BZLF1	PLD3	0.083436
BZLF1	PCDHGA10	0.049103
BZLF1	ADAM8	0.070903
BZLF1	TRIP12	0.16475
BZLF1	CSTF1	0.21177
BZLF1	SKA2	0.12188
BZLF1	U2AF2	0.17569
BZLF1	MAP3K8	0.067484
BZLF1	NOSTRIN	0.22556
BZLF1	TCL1A	0.13548
BZLF1	ELMO1	0.089411
BZLF1	SS18	0.11956
BZLF1	U2SURP	0.23522
BZLF1	AICDA	0.064196
BZLF1	RPS9	0.10212
BZLF1	DCHS2	0.049103
BZLF1	SNX18	0.18857
BZLF1	TNFRSF13B	0.18659
BZLF1	SBDS	0.07682
BZLF1	CDC42EP1	0.15527
BZLF1	ADRB1	0.037796
BZLF1	RPL28	0.18101
BZLF1	SIRT7	0.098994
BZLF1	AP1G2	0.18469
BZLF1	PREX1	0.043792
BZLF1	ATP2C2	0.1125
BZLF1	PCDHGC3	0.047639
BZLF1	PDCD10	0.14172
BZLF1	MRM1	0.031621
BZLF1	FAM231D	0.011322
BZLF1	USP44	0.13041
BZLF1	ZBTB17	0.026083
BZLF1	GFI1B	0.044227
BZLF1	CCBE1	0.040177
BZLF1	BLMH	0.13037
BZLF1	IRAK1	0.038499
BZLF1	CXCR4	0.063153
BZLF1	USP37	0.074244

BZLF1	ARFIP2	0.1575
BZLF1	SAC3D1	0.17931
BZLF1	MMS22L	0.18274
BZLF1	MTMR9	0.19584
BZLF1	COL4A2	0.14819
BZLF1	PKD1L2	0.050218
BZLF1	CHMP1A	0.098157
BZLF1	PPM1G	0.072932
BZLF1	XPO1	0.10794
BZLF1	LBR	0.14536
BZLF1	ATP8B1	0.14059
BZLF1	TJP2	0.16947
BZLF1	NR1I2	0.036728
BZLF1	ITM2A	0.2221
BZLF1	RBBP4	0.085316
BZLF1	YWHAQ	0.19532
BZLF1	HOOK1	0.12208
BZLF1	INPP5K	0.029667
BZLF1	PPP1R3C	0.13339
BZLF1	ATP10A	0.091616
BZLF1	KLK5	0.22331
BZLF1	MARCH5	0.061731
BZLF1	PPP1R15A	0.188
BZLF1	STT3A	0.19662
BZLF1	ZBTB24	0.11682
BZLF1	TCF12	0.031946
BZLF1	SPC24	0.14439
BZLF1	NCF2	0.043767
BZLF1	CCL3	0.032906
BZLF1	MYL6B	0.36991
BZLF1	PCK2	0.11987
BZLF1	CNIH2	0.12109
BZLF1	GLI1	0.033629
BZLF1	GIPC1	0.07492
BZLF1	RAD51C	0.10666
BZLF1	NAA38	0.18142
BZLF1	IGF2BP2	0.15225
BZLF1	YWHAB	0.072712
BZLF1	SLC25A5	0.14871
BZLF1	GNA13	0.063487
BZLF1	BLOC1S5	0.17737
BZLF1	TRIM21	0.086216
BZLF1	SPTSSB	0.14352
BZLF1	TAF15	0.16613
BZLF1	RBX1	0.077561
BZLF1	TNNI3K	0.074527
BZLF1	WDR82	0.16738
BZLF1	RAB3A	0.054217
BZLF1	NSL1	0.13275
BZLF1	HMGB2	0.011345
BZLF1	RHOH	0.14923
BZLF1	TCHP	0.17047
BZLF1	ATP6V0A1	0.17415
BZLF1	BRPF3	0.14704
BZLF1	GTF2I	0.05768
BZLF1	CPSF3	0.12748
BZLF1	KCNMA1	0.083713
BZLF1	ORC6	0.14109
BZLF1	CELSR1	0.023829
BZLF1	UBQLN2	0.18569
BZLF1	PLRG1	0.17101
BZLF1	CUL1	0.10894
BZLF1	SCG5	0.16385
BZLF1	CD9	0.12497
BZLF1	DHX58	0.087201
BZLF1	MYO9A	0.11188
BZLF1	SP3	0.052045
BZLF1	POLI	0.094328

BZLF1	SLC24A1	0.19132
BZLF1	DR1	0.087131
BZLF1	TBK1	0.05062
BZLF1	HSPA9	0.10719
BZLF1	NCSTN	0.071908
BZLF1	PLSCR2	0.049691
BZLF1	GSTP1	0.078944
BZLF1	MMP14	0.067231
BZLF1	PIGK	0.089286
BZLF1	CDC5L	0.16587
BZLF1	MBNL1	0.13569
BZLF1	MED4	0.14149
BZLF1	RHBDD1	0.12885
BZLF1	TIE1	0.12894
BZLF1	FABP5	0.19445
BZLF1	HRC	0.048828
BZLF1	AP1G1	0.15994
BZLF1	HMCN2	0.0802
BZLF1	CDHR2	0.38641
BZLF1	FAS	0.029301
BZLF1	APOE	0.010737
BZLF1	TUBB2B	0.034943
BZLF1	WAS	0.13033
BZLF1	ALDOC	0.091317
BZLF1	SNRPB2	0.15621
BZLF1	TNNI3	0.060258
BZLF1	ILF3	0.11719
BZLF1	WDR61	0.12941
BZLF1	SULT2B1	0.12747
BZLF1	PAWR	0.10973
BZLF1	ASIC2	0.1728
BZLF1	CBX3	0.086142
BZLF1	RINT1	0.17398
BZLF1	SHANK3	0.079836
BZLF1	IKBIP	0.1849
BZLF1	RCVRN	0.075233
BZLF1	AGFG1	0.157
BZLF1	INTS5	0.17988
BZLF1	C17orf62	0.2221
BZLF1	SNX17	0.16958
BZLF1	CDH5	0.27362
BZLF1	DDX58	0.047913
BZLF1	ARID1B	0.10444
BZLF1	IL6R	0.089205
BZLF1	HS3ST5	0.15398
BZLF1	TP53INP2	0.19302
BZLF1	ZNF165	0.040754
BZLF1	SEMA6A	0.13679
BZLF1	ZMYND10	0.18327
BZLF1	COTL1	0.18267
BZLF1	SMN1	0.19686
BZLF1	SNIP1	0.13581
BZLF1	SYT2	0.3175
BZLF1	CCNY	0.064745
BZLF1	CAPNS1	0.30579
BZLF1	GTF2E2	0.13776
BZLF1	KCNG3	0.15369
BZLF1	TRIM41	0.12269
BZLF1	MPZL3	0.20208
BZLF1	GABARAPL1	0.2224
BZLF1	GTF2B	0.092717
BZLF1	ANGPTL4	0.11389
BZLF1	WDR16	0.18336
BZLF1	IFIT1	0.1869
BZLF1	PCDHB15	0.046144
BZLF1	RAD18	0.07455
BZLF1	DNM1	0.013852
BZLF1	OCM2	0.057416

BZLF1	SIPA1	0.11202
BZLF1	TPD52	0.40629
BZLF1	MC5R	0.15446
BZLF1	RIPK4	0.10805
BZLF1	POLDIP3	0.20572
BZLF1	IRS2	0.025488
BZLF1	ARID5B	0.081594
BZLF1	NUFIP1	0.13928
BZLF1	KLC1	0.18212
BZLF1	MRPS31	0.16698
BZLF1	FRMPD4	0.23744
BZLF1	LIG4	0.035489
BZLF1	KLHDC3	0.19706
BZLF1	XRCC5	0.034073
BPLF1	ubl4ab	0.079148
BPLF1	MED1	0.010966
BPLF1	GAGE12I	0.037608
BPLF1	PAGE2B	0.16083
BPLF1	SPTA1	0.04984
BPLF1	S100A7	0.084597
BPLF1	HSPA5	0.045888
BPLF1	LDLR	0.036455
BPLF1	UPF3A	0.091945
BPLF1	RABEP1	0.10795
BPLF1	C12orf61	0.070379
BPLF1	FAM86B2	0.013807
BPLF1	PNMA6D	0.022172
BPLF1	PLSCR3	0.11757
BPLF1	ZC3HAV1L	0.090908
BPLF1	BTBD16	0.019077
BPLF1	C9orf40	0.013917
BPLF1	RPUSD4	0.053393
BPLF1	CCDC159	0.061848
BPLF1	C10orf68	0.069593
BPLF1	CCDC42	0.011553
BPLF1	CCDC162P	0.0144
BPLF1	GOLGA6L1	0.039054
BPLF1	KRI1	0.11586
BPLF1	SPATA1	0.020415
BPLF1	CCDC69	0.035494
BPLF1	YMR013W-A	0.072113
BPLF1	CASC3	0.10632
BPLF1	CYP4F30P	0.011758
BPLF1	ATP5B	0.059095
BPLF1	NIN	0.12211
BPLF1	KCNIP1	0.099549
BPLF1	STIM2	0.11754
BPLF1	HUS1B	0.064346
BPLF1	RPLP1	0.044209
BPLF1	PSMG3	0.010523
BPLF1	ANGEL1	0.032586
BPLF1	C19orf44	0.033646
BPLF1	C15orf40	0.010173
BPLF1	FSIP1	0.10964
BPLF1	WIPF1	0.047391
BPLF1	SPTAN1	0.070781
BPLF1	TSGA10IP	0.095939
BPLF1	TNFAIP8L3	0.024434
BPLF1	DHPS	0.053086
BPLF1	POP4	0.091293
BPLF1	MASP1	0.13908
BPLF1	SCUBE3	0.14596
BPLF1	SPBC1539.02	0.043782
BPLF1	GLTSCR1L	0.13501
BPLF1	MAB21L3	0.014302
BPLF1	PROC	0.11683
BPLF1	PDCD6	0.1628
BPLF1	DKC1	0.042837

BPLF1	S100A9	0.037076
BPLF1	ANXA2	0.072344
BPLF1	MMP20	0.02793
BPLF1	smf	0.022208
BPLF1	MAGEB1	0.015359
BPLF1	MATN4	0.15008
BPLF1	NOTCH3	0.034836
BPLF1	RNH1	0.12352
BPLF1	UBAP2	0.11126
BPLF1	C17orf105	0.045991
BPLF1	FAM110D	0.087659
BPLF1	CLIP1	0.056582
BPLF1	BLOC1S3	0.091779
BPLF1	AMOT	0.022555
BPLF1	CCDC154	0.025466
BPLF1	PAGE5	0.079562
BPLF1	GAGE10	0.094536
BPLF1	BOP	0.01005
BPLF1	FAM117B	0.15132
BPLF1	EIF4G2	0.025156
BPLF1	PHYHIP	0.022195
BPLF1	METTL1	0.097571
BPLF1	GAGE3	0.0202
BPLF1	MESDC1	0.023811
BPLF1	C10orf91	0.13409
BPLF1	CCDC28A	0.15496
BPLF1	TTC22	0.014885
BPLF1	FAM120C	0.024507
BPLF1	CCDC65	0.015056
BPLF1	GID4	0.038769
BPLF1	FAM83F	0.080334
BPLF1	FAM101A	0.071841
BPLF1	SNURFL	0.059948
BPLF1	THBD	0.088194
BPLF1	KIAA0040	0.024661
BPLF1	FAM178A	0.095545
BPLF1	MEN1	0.029234
BPLF1	RNMT	0.021404
BPLF1	C12orf36	0.023652
BPLF1	MAGOH	0.010048
BPLF1	EML1	0.14119
BPLF1	OTUD6B	0.014131
BPLF1	NOL3	0.091528
BPLF1	FAM83C	0.04964
BPLF1	IQCK	0.082278
BPLF1	ITFG2	0.013254
BPLF1	FAM166A	0.10068
BPLF1	MUM1L1	0.084576
BPLF1	FAM74A4	0.031695
BPLF1	EPS15	0.051781
BPLF1	PCSK2	0.026421
BPLF1	AP3S1	0.050249
BPLF1	SDF4	0.10561
BPLF1	NACAP1	0.023716
BPLF1	TAF11	0.068991
BPLF1	SPACA3	0.051745
BPLF1	STAT1	0.014433
BPLF1	FAT4	0.13634
BPLF1	DTNBP1	0.022667
BPLF1	PPEF1	0.061096
BPLF1	CXorf67	0.11584
BPLF1	C18orf25	0.049618
BPLF1	NELL2	0.018262
BPLF1	C11orf35	0.016074
BPLF1	C16orf86	0.025095
BPLF1	FAM188B2	0.1457
BPLF1	NUDCD3	0.057017
BPLF1	THBS1	0.015111

BPLF1	MAGEF1	0.020705
BPLF1	KIAA1407	0.041967
BPLF1	FAM184B	0.03066
BPLF1	PRMT1	0.012847
BPLF1	CCSER1	0.10481
BPLF1	CCDC144A	0.010693
BPLF1	PNMAL2	0.072377
BPLF1	AP1M1	0.014147
BPLF1	YPR022C	0.099217
BPLF1	TPPP	0.097066
BPLF1	CCDC146	0.064933
BPLF1	SYT11	0.18459
BPLF1	CCDC150	0.035489
BPLF1	C1orf105	0.1044
BPLF1	HSP90B1	0.075262
BPLF1	MACF1	0.093669
BPLF1	CCDC66	0.01409
BPLF1	CCDC176	0.089774
BPLF1	GCH1	0.043911
BPLF1	C10orf67	0.11034
BPLF1	LINC00597	0.060663
BPLF1	FAM131C	0.020698
BPLF1	ZBTB8OS	0.010741
BPLF1	PRDM11	0.010237
BPLF1	NKD2	0.15184
BPLF1	MT1HL1	0.055705
BPLF1	C2orf88	0.016765
BPLF1	NCOR1P1	0.011922
BPLF1	C19orf71	0.051816
BPLF1	SNX29P2	0.036087
BPLF1	RHPN1-AS1	0.095517
BPLF1	C6orf118	0.054151
BPLF1	IL17REL	0.019792
BPLF1	CCDC74B	0.046306
BPLF1	FAT1	0.070859
BPLF1	LINC00471	0.065999
BPLF1	S100A11	0.11234
BPLF1	CAB8	0.010568
BPLF1	MSANTD2	0.010425
BPLF1	CCDC137	0.14018
BPLF1	TIFAB	0.13603
BPLF1	C15orf54	0.035415
BPLF1	NUTM2A	0.035524
BPLF1	NKD1	0.10752
BPLF1	PLAC8L1	0.026496
BPLF1	CROCCP3	0.023126
BPLF1	FAM151B	0.043431
BPLF1	GCSAML	0.072606
BPLF1	FNIP2	0.18788
BPLF1	FAM47B	0.051713
BPLF1	MBD3L5	0.011827
BPLF1	SUPT20HL2	0.073968
BPLF1	PPP2R3B	0.061393
BPLF1	ACTN4	0.060466
BPLF1	SPRYD7	0.019266
BPLF1	EHD1	0.04076
BPLF1	LINC00523	0.13775
BPLF1	RABGAP1L	0.016441
BPLF1	S100A13	0.11797
BPLF1	MELK	0.032247
BPLF1	C9orf152	0.016497
BPLF1	S100A1	0.074047
BPLF1	ARL14EPL	0.12836
BPLF1	UTRN	0.04917
BPLF1	GAGE12H	0.037608
BPLF1	INTS4L2	0.017242
BPLF1	SLC34A1	0.097098
BPLF1	FAM219B	0.012435

BPLF1	CXorf21	0.043275
BPLF1	ELN	0.11995
BPLF1	FAM127A	0.034711
BPLF1	C17orf66	0.021737
BPLF1	PIH1D2	0.083672
BPLF1	POM121L12	0.056951
BPLF1	CALB1	0.1066
BPLF1	NDEL1	0.022822
BPLF1	MASP2	0.12731
BPLF1	PARK7	0.026889
BPLF1	LINC00472	0.094936
BPLF1	C20orf96	0.017383
BPLF1	GOLGA6L4	0.10417
BPLF1	GOLGA6L6	0.098277
BPLF1	KIAA1958	0.052675
BPLF1	SLAIN1	0.13651
BPLF1	C9orf169	0.028259
BPLF1	YPL025C	0.038834
BPLF1	ZNF326	0.074241
BPLF1	KIAA0226L	0.015158
BPLF1	ANXA2R	0.12048
BPLF1	CC2D2B	0.032149
BPLF1	YML009W-B	0.11628
BPLF1	C14orf178	0.041352
BPLF1	EFEMP1	0.1408
BPLF1	C1orf168	0.060248
BPLF1	YTHDF1	0.05906
BPLF1	SPARC	0.063561
BPLF1	MRFAP1L1	0.11294
BPLF1	NOTCH4	0.033093
BPLF1	CNPY1	0.01472
BPLF1	FAM160A1	0.12303
BPLF1	PCDH17	0.13634
BPLF1	PIR	0.073891
BPLF1	WHAMMP3	0.012874
BPLF1	YJR030C	0.065353
BPLF1	KIAA1841	0.068458
BPLF1	C1orf196	0.042145
BPLF1	RNF216P1	0.063346
BPLF1	REPS1	0.12531
BPLF1	DCUN1D2	0.011877
BPLF1	LINC00476	0.015768
BPLF1	STPG2	0.016165
BPLF1	YJEFN3	0.040443
BPLF1	C18orf21	0.04609
BPLF1	XAGE2	0.11264
BPLF1	YML009W-B	0.040059
BPLF1	LRCOL1	0.11002
BPLF1	GOLGA6L18	0.087875
BPLF1	TXNIP	0.075163
BPLF1	BCORP1	0.059627
BPLF1	FAM160B2	0.026482
BPLF1	WIBG	0.10084
BPLF1	FAM221B	0.080103
BPLF1	NOTCH2	0.010935
BPLF1	MPTX1	0.026798
BPLF1	AMER2	0.10264
BPLF1	STIM1	0.061675
BPLF1	S100B	0.061113
BPLF1	CCDC172	0.016011
BPLF1	LDOC1L	0.028663
BPLF1	AC3.5	0.058848
BPLF1	LINS	0.032829
BPLF1	TNNC1	0.12822
BPLF1	C14orf80	0.12443
BPLF1	NBPF24	0.023693
BPLF1	C1orf94	0.1707
BPLF1	CETN2	0.11216

BPLF1	ANXA6	0.13138
BPLF1	C9orf153	0.028038
BPLF1	ACTN2	0.047924
BPLF1	PRSS2	0.11107
BPLF1	CT45A5	0.033653
BPLF1	C2orf78	0.045853
BPLF1	C21orf58	0.042578
BPLF1	PITPNM3	0.12492
BPLF1	VIL1	0.06494
BPLF1	C19orf35	0.085104
BPLF1	GAGE6	0.0202
BPLF1	YIR021W-A	0.025752
BPLF1	COMMD9	0.062413
BPLF1	KCNJ4	0.028079
BPLF1	ANGEL2	0.020941
BPLF1	MIR7-3HG	0.016077
BPLF1	PP632	0.019872
BPLF1	C17orf104	0.032612
BPLF1	CETN1	0.14126
BPLF1	PRO1854	0.028697
BPLF1	GAGE4	0.065266
BPLF1	ATP5SL	0.014219
BPLF1	C2CD4D	0.02623
BPLF1	ZK1128.3	0.042048
BPLF1	YDL022C-A	0.016529
BPLF1	PP632	0.022233
BPLF1	PNMAL1	0.04871
BPLF1	C18orf42	0.014058
BPLF1	VLDLR	0.058436
BPLF1	CDH1	0.02839
BPLF1	GAGE13	0.0202
BPLF1	MAP1LC3B	0.02414
BPLF1	TTN	0.024443
BPLF1	ZYG11A	0.010522
BPLF1	CXorf30	0.068412
BPLF1	EGF	0.046147
BPLF1	LTBP4	0.1103
BPLF1	CCDC132	0.031069
BPLF1	RPS19	0.049299
BPLF1	PLSCR4	0.11403
BPLF1	DNAJC9-AS1	0.068253
BPLF1	UBQLNL	0.010767
BPLF1	CCDC152	0.015231
BPLF1	KHDC1L	0.010425
BPLF1	FAM45A	0.11853
BPLF1	KIAA1751	0.016992
BPLF1	TCP10L2	0.050959
BPLF1	HBA1	0.062655
BPLF1	C1orf106	0.13332
BPLF1	GOLGA6L3	0.10417
BPLF1	WWC2-AS2	0.061966
BPLF1	SNX9	0.065662
BPLF1	FBXL18	0.026185
BPLF1	GUCD1	0.026282
BPLF1	FAM195B	0.028833
BPLF1	CCDC7	0.048863
BPLF1	YFL013W-A	0.059066
BPLF1	RYR2	0.025812
BPLF1	PRF1	0.055549
BPLF1	IL6ST	0.066767
BPLF1	MYO1E	0.030649
BPLF1	GAGE12F	0.037608
BPLF1	NELFA	0.12794
BPLF1	GPRIN3	0.035785
BPLF1	FAM92B	0.014947
BPLF1	SETD2	0.012016
BPLF1	C4orf22	0.018484
BPLF1	C6orf52	0.010546

BPLF1	CCDC74A	0.046306
BPLF1	PRR19	0.053845
BPLF1	CCDC67	0.010451
BPLF1	S100A8	0.053823
BPLF1	NID2	0.14962
BPLF1	GAREML	0.15494
BPLF1	MIMI	0.090905
BPLF1	NELL1	0.074091
BPLF1	ytzL	0.17863
BPLF1	WDR96	0.05186
BPLF1	DNER	0.08601
BPLF1	COL17A1	0.10451
BPLF1	EIF6	0.033352
BPLF1	L1TD1	0.010943
BPLF1	C15orf65	0.032888
BPLF1	FAM160B1	0.073479
BPLF1	SAMD14	0.012412
BPLF1	PEF1	0.14819
BPLF1	CT45A4	0.025984
BPLF1	FBXL8	0.17346
BPLF1	NUPR1L	0.057252
BPLF1	MEGF8	0.020167
BPLF1	C10orf82	0.047196
BPLF1	ERICH1-AS1	0.10807
BPLF1	C3orf65	0.065519
BPLF1	CHP1	0.059491
BPLF1	CBY3	0.051144
BPLF1	C1orf228	0.019635
BPLF1	DYDC2	0.029811
BPLF1	EP400NL	0.082711
BPLF1	FAM203B	0.07086
BPLF1	FASN	0.046334
BPLF1	HEATR5A	0.044082
BPLF1	GPR98	0.052727
BPLF1	OTUD6A	0.025345
BPLF1	MICU1	0.1183
BPLF1	CALU	0.11652
BPLF1	PPP3CA	0.021672
BPLF1	VWA9	0.026937
BPLF1	MYOZ2	0.11614
BPLF1	C16orf72	0.10508
BPLF1	BEND2	0.037969
BPLF1	EIF4B	0.023368
BPLF1	LRP2	0.055012
BPLF1	SLC23A1	0.018193
BPLF1	UNC119	0.056303
BPLF1	JMJD7	0.010566
BPLF1	POM121L2	0.054682
BPLF1	GOLGA6L10	0.087875
BPLF1	FAM188B	0.15367
BPLF1	MISP	0.12726
BPLF1	FAM231B	0.068996
BPLF1	SELO	0.095529
BPLF1	ZNF728	0.011724
BPLF1	FAM27D1	0.06969
BPLF1	UHRF1BP1L	0.081889
BPLF1	SHANK2-AS3	0.025222
BPLF1	UNQ6126/PRO20091	0.044047
BPLF1	ATXN7L2	0.031942
BPLF1	PEX11A	0.096318
BPLF1	YGL014C-A	0.10875
BPLF1	S100A6	0.092957
BPLF1	LRP8	0.10007
BPLF1	CCDC151	0.016722
BPLF1	CT55	0.081525
BPLF1	S100P	0.088536
BPLF1	RWDD4	0.027858
BPLF1	PRDX6	0.011813

BPLF1	CHAF1A	0.16798
BPLF1	PTGES3L	0.032997
BPLF1	TP53INP1	0.092737
BPLF1	MNCb-2875	0.025132
BPLF1	FAM127C	0.037791
BPLF1	RAB11FIP3	0.039157
BPLF1	EFCAB4B	0.10116
BPLF1	C8orf74	0.039222
BPLF1	CCDC104	0.025463
BPLF1	XAGE3	0.16083
BPLF1	C11orf65	0.019913
BPLF1	C1orf111	0.078859
BPLF1	CCDC87	0.017471
BPLF1	CHGB	0.19328
BPLF1	DTNA	0.065767
BPLF1	REPS2	0.15448
BPLF1	PPEF2	0.058212
BPLF1	EFEMP2	0.14703
BPLF1	MAGED4	0.013164
BPLF1	ATP2A1	0.039082
BPLF1	ATXN7L3B	0.043634
BPLF1	WLS	0.038534
BPLF1	CT45A3	0.028975
BPLF1	CAGE1	0.018968
BPLF1	LINC00205	0.060124
BPLF1	PKD2L1	0.076081
BPLF1	FAM46B	0.07408
BPLF1	PNAS-138	0.075559
BPLF1	NCOA6	0.094891
BPLF1	CCDC26	0.056489
BPLF1	EHD3	0.052531
BPLF1	FAM181A	0.027256
BPLF1	SUPT20HL1	0.073968
BPLF1	C7orf57	0.023194
BPLF1	C22orf43	0.042858
BPLF1	FAM166B	0.014225
BPLF1	VWA5B2	0.04385
BPLF1	THBS2	0.12001
BPLF1	PRAME	0.12951
BPLF1	CALM1	0.067503
BPLF1	EIF4E2	0.01217
BPLF1	MAP7D2	0.017728
BPLF1	S100A4	0.14632
BPLF1	S100A12	0.07488
BPLF1	FAM149B1	0.059888
BPLF1	RCN1	0.15286
BPLF1	DPP4	0.01386
BPLF1	EIF4E	0.020835
BPLF1	STAG3L4	0.028239
BPLF1	ACTN3	0.14064
BPLF1	ATP5F1	0.02391
BPLF1	FBN1	0.14153
BPLF1	SPAC22F8.05	0.010996
BPLF1	C12orf63	0.033663
BPLF1	F10	0.055141
BPLF1	CAPN1	0.039809
BPLF1	UCKL1	0.0424
BPLF1	FAM46A	0.061074
BPLF1	CELSR3	0.080564
BPLF1	RILP	0.069672
BPLF1	SS18L2	0.025717
BPLF1	GAGE12J	0.0202
BPLF1	N6AMT1	0.013794
BPLF1	DEHA2C16280g	0.041174
BPLF1	KHNYN	0.042503
BPLF1	SIRT4	0.043143
BPLF1	TESC	0.089426
BPLF1	GAGE2A	0.037608

BPLF1	ATXN7L1	0.031942
BPLF1	LSM12	0.070109
BPLF1	KIAA0907	0.017696
BPLF1	C12orf57	0.044306
BPLF1	FAM228A	0.012955
BPLF1	YAE1D1	0.075884
BPLF1	CT45A1	0.030198
BPLF1	ERICH1	0.064844
BPLF1	RPH3A	0.061202
BPLF1	SRI	0.070545
BPLF1	LRP4	0.07714
BPLF1	BVES-AS1	0.042734
BPLF1	CCDC127	0.030992
BPLF1	CCDC120	0.073205
BPLF1	MAGEB16	0.013146
BPLF1	SLIT2	0.029335
BPLF1	C1orf145	0.08347
BPLF1	OTUD1	0.022329
BPLF1	MATN2	0.12168
BPLF1	CIB1	0.10486
BPLF1	PPP2R3A	0.088267
BPLF1	AKAP3	0.052703
BPLF1	DLL1	0.11886
BPLF1	ACTN1	0.067408
BPLF1	BLOC1S1	0.097206
BPLF1	FAM195A	0.14243
BPLF1	RGAG1	0.029189
BPLF1	PNMA6A	0.011117
BPLF1	EHD4	0.027261
BPLF1	PNPT1	0.038132
BPLF1	UPF3B	0.10852
BPLF1	C5orf58	0.08206
BPLF1	PRSS3	0.12987
BPLF1	CCDC169	0.038376
BPLF1	C7orf62	0.060796
BPLF1	MYL2	0.1515
BPLF1	EDEM1	0.070962
BPLF1	LRP1	0.033296
BPLF1	MB21D2	0.047848
BPLF1	ARMCX5	0.032096
BPLF1	ATP5A1	0.021135
BPLF1	SNX19	0.087772
BPLF1	CLPP	0.057029
BPLF1	PDGFRB	0.017789
BPLF1	SPATA2L	0.049234
BPLF1	ITSN1	0.023983
BPLF1	C15orf37	0.012345
BPLF1	BTF3L4	0.030568
BPLF1	SPIDR	0.097669
BPLF1	DST	0.075762
BPLF1	MYL6	0.13829
BPLF1	COMP	0.10425
BPLF1	CCDC47	0.11786
BPLF1	XAGE5	0.082769
BPLF1	ANXA7	0.060324
BPLF1	CHP2	0.11056
BPLF1	LYRM9	0.10431
BPLF1	MUC1	0.099477
BPLF1	FAM127B	0.060833
BPLF1	CDK5R1	0.050871
BPLF1	CXorf40B	0.024788
BPLF1	NECAB2	0.15471
BPLF1	CANX	0.065489
BPLF1	SGD1	0.057618
BPLF1	MMP15	0.017899
BPLF1	FBXO34	0.010062
BPLF1	BSDC1	0.013729
BPLF1	ANXA11	0.06624

BPLF1	PPP3CB	0.025806
BPLF1	GOLGA6L19	0.087875
BPLF1	C8orf88	0.044894
BPLF1	C5orf34	0.044587
BPLF1	LURAP1L	0.12954
BPLF1	C19orf47	0.034107
BPLF1	C4orf33	0.01839
BPLF1	C12orf50	0.10207
BPLF1	C17orf102	0.057805
BPLF1	IQGAP1	0.015977
BPLF1	RMND5B	0.013198
BPLF1	IQCG	0.03037
BPLF1	MATN3	0.15997
BPLF1	PNMA6C	0.022172
BPLF1	FAM47C	0.062029
BPLF1	SH3KBP1	0.018109
BPLF1	AMOTL1	0.054174
BPLF1	RET	0.056214
BPLF1	PAGE3	0.054555
BPLF1	MAGED2	0.014969
BPLF1	TLX1NB	0.020247
BPLF1	CDH23	0.043234
BPLF1	LCA10	0.090905
BPLF1	RAB11FIP4	0.1921
BPLF1	CNGC19	0.14162
BPLF1	C9orf106	0.015916
BPLF1	DYDC1	0.017741
BPLF1	CCDC102B	0.010847
BPLF1	CNRIP1	0.025804
BPLF1	TCERG1L	0.042925
BPLF1	SUV39H2	0.011347
BPLF1	C6orf223	0.13889
BPLF1	C1orf64	0.071064
BPLF1	PACRGL	0.023825
BPLF1	TCTEX1D1	0.025063
BPLF1	C1orf140	0.091757
BPLF1	YGL006W-A	0.014059
BPLF1	GOLGA6L20	0.046327
BPLF1	LINC00588	0.084723
BPLF1	HYPK	0.022837
BPLF1	RUNDC1	0.019022
BPLF1	NIPSNAP3B	0.018216
BPLF1	SZRD1	0.040051
BPLF1	AC3.5	0.083359
BPLF1	DDX26B	0.11678
BPLF1	LCHN	0.031852
BPLF1	FAM45B	0.10072
BPLF1	FAM229B	0.0599
BPLF1	LINC00478	0.019265
BPLF1	C2orf81	0.10104
BPLF1	FAM84A	0.036733
BPLF1	SPANXA2-OT1	0.043333
BPLF1	CLIC4	0.051462
BPLF1	EIF4G1	0.012626
BPLF1	FAM49B	0.034276
BPLF1	BNIP2	0.059539
BPLF1	ZNF767	0.057278
BPLF1	FAM199X	0.0365
BPLF1	JAG2	0.093014
BPLF1	HDGFL1	0.010489
BPLF1	SWT1	0.038274
BPLF1	GAGE12G	0.037608
BPLF1	FAM117A	0.08887
BPLF1	CADPS	0.16324
BPLF1	C17orf59	0.17094
BPLF1	DRC1	0.030263
BPLF1	FAM161B	0.1532
BPLF1	CCAR1	0.13462

BPLF1	C16orf93	0.05507
BPLF1	C20orf62	0.022482
BPLF1	C6orf222	0.033008
BPLF1	CCDC171	0.035095
BPLF1	CCAR2	0.067592
BPLF1	PLAUR	0.061437
BPLF1	FAM178B	0.060132
BPLF1	C1orf195	0.17947
BPLF1	CRELD2	0.14196
BPLF1	NPIPA5	0.014536
BPLF1	PRR23A	0.051151
BPLF1	C2orf72	0.10819
BPLF1	YBR056C-B	0.051172
BPLF1	SCIMP	0.1335
BPLF1	ARMC12	0.040366
BPLF1	GSN	0.058741
BPLF1	SLC11A2	0.020037
BPLF1	C3orf22	0.077307
BPLF1	EXOC3L4	0.051169
BPLF1	CYCB3-1	0.10727
BPLF1	COA7	0.021881
BPLF1	CABP1	0.021377
BPLF1	BEND3	0.010545
BPLF1	C22orf26	0.058321
BPLF1	LETM1	0.13706
BPLF1	TNFAIP8L1	0.11712
BPLF1	pkmyt1	0.063668
BPLF1	PGBD4	0.02429
BPLF1	SPHK1	0.01069
BPLF1	CRYGN	0.044772
BPLF1	CTU1	0.070081
BPLF1	FAM231A	0.28315
BPLF1	TFB1M	0.018597
BPLF1	C6orf229	0.059171
BPLF1	FAM124A	0.19717
BPLF1	AMER1	0.1056
BPLF1	FAM188A	0.11036
BPLF1	MAGEB4	0.01671
BPLF1	AP1B1	0.025236
BPLF1	RCN3	0.14951
BPLF1	PLSCR1	0.06676
BPLF1	GAGE5	0.0202
BPLF1	GAGE12B	0.037608
BPLF1	SLC6A4	0.013142
BPLF1	NKAPL	0.14374
BPLF1	NCKAP5L	0.1023
BPLF1	CCDC153	0.01098
BPLF1	PRR21	0.039106
BPLF1	TLDC2	0.099696
BPLF1	LTBP1	0.14769
BPLF1	C6orf165	0.010957
BPLF1	MSANTD1	0.026246
BPLF1	F2	0.029461
BPLF1	SMEK3P	0.046611
BPLF1	SYT1	0.052345
BPLF1	C11orf52	0.030454
BPLF1	NSUN5P1	0.053377
BPLF1	PI4K2A	0.026123
BPLF1	NCS1	0.13032
BPLF1	TCAP	0.071622
BPLF1	C12orf71	0.018769
BPLF1	SRRM5	0.023769
BPLF1	CCDC81	0.026009
BPLF1	FBLN5	0.1322
BPLF1	DAG1	0.042144
BPLF1	AGTRAP	0.07038
BPLF1	C1orf192	0.059356
BPLF1	HRK	0.11583

BPLF1	C2orf62	0.021472
BPLF1	YAL042C-A	0.019732
BPLF1	C19orf67	0.028413
BPLF1	C5orf48	0.098846
BPLF1	SH3BP5L	0.079348
BPLF1	CCDC17	0.022122
BPLF1	NECAB3	0.088706
BPLF1	MLC1	0.02164
BPLF1	C19orf82	0.069634
BPLF1	SUMO4	0.010383
BPLF1	TRMT61B	0.058627
BPLF1	SNX6	0.029889
BPLF1	SECISBP2L	0.09792
BPLF1	C11orf96	0.073988
BPLF1	CDKN2AIPNL	0.11424
BPLF1	INTS4L1	0.010807
BPLF1	FAM92A1	0.017311
BPLF1	NACA	0.028679
BPLF1	NPC1L1	0.0401
BPLF1	AASDHPPT	0.097913
BPLF1	RSRC1	0.11518
BPLF1	MEGF6	0.14732
BPLF1	CALR	0.016339
BPLF1	RSRC2	0.10395
BPLF1	C16orf45	0.03186
BPLF1	GOLGA6L9	0.087875
BPLF1	ALS2CR11	0.027662
BPLF1	ZNRD1-AS1	0.022581
BPLF1	KCNIP2	0.050319
BPLF1	FBRSL1	0.054002
BPLF1	LRRC14	0.017327
BPLF1	ZK1128.3	0.10678
BPLF1	CUEDC1	0.052338
BPLF1	HEATR5B	0.08572
BPLF1	CTAGE15	0.018896
BPLF1	LINC00303	0.019691
BPLF1	CD93	0.099187
BPLF1	CCDC54	0.017849
BPLF1	GRIN1	0.047759
BPLF1	CLINT1	0.01085
BPLF1	C20orf195	0.032553
BPLF1	C19orf66	0.092397
BPLF1	DHH	0.085527
BPLF1	GAGE2E	0.0202
BPLF1	UNQ6125/PRO20090	0.095116
BPLF1	SPHK2	0.01973
BPLF1	VIPAS39	0.041572
BPLF1	CT45A2	0.028975
BPLF1	N4BP2L1	0.023678
BPLF1	FAM231D	0.13128
BPLF1	MAGEB17	0.016749
BPLF1	KIAA0087	0.07258
BPLF1	C11orf72	0.038404
BPLF1	C9orf171	0.085466
BPLF1	IQCF3	0.019384
BPLF1	LINC00615	0.06614
BPLF1	CCDC94	0.091493
BPLF1	MYL6B	0.11571
BPLF1	RBBP8NL	0.030421
BPLF1	RGAG4	0.04937
BPLF1	ATP6V0A1	0.019486
BPLF1	SCG5	0.11499
BPLF1	TTC23L	0.019941
BPLF1	AP1G1	0.023338
BPLF1	CDHR2	0.12503
BPLF1	CDR2L	0.023708
BPLF1	SNX17	0.01261
BPLF1	CDH5	0.080127

BPLF1	GR6	0.026111
BPLF1	IL6R	0.024598
BPLF1	TP53INP2	0.069598
BPLF1	SYT2	0.12842
BPLF1	CAPNS1	0.029111
BPLF1	GTF2E2	0.1207
BPLF1	TPD52	0.14832
BALF2	ubl4ab	0.038772
BALF2	SPTA1	0.043453
BALF2	S100A7	0.07488
BALF2	HSPA5	0.043699
BALF2	LDLR	0.035705
BALF2	RABEP1	0.010553
BALF2	C12orf61	0.037509
BALF2	PLSCR3	0.11075
BALF2	ZC3HAV1L	0.011743
BALF2	YMR013W-A	0.026063
BALF2	ATP5B	0.063193
BALF2	NIN	0.091678
BALF2	KCNIP1	0.095393
BALF2	STIM2	0.11235
BALF2	SPTAN1	0.06422
BALF2	MASP1	0.15746
BALF2	SCUBE3	0.13825
BALF2	PROC	0.14072
BALF2	PDCD6	0.16356
BALF2	S100A9	0.032343
BALF2	ANXA2	0.069178
BALF2	MMP20	0.018625
BALF2	MATN4	0.14137
BALF2	NOTCH3	0.026975
BALF2	RNH1	0.1143
BALF2	CLIP1	0.048865
BALF2	AMOT	0.014282
BALF2	FAM83F	0.019926
BALF2	THBD	0.083335
BALF2	EML1	0.13301
BALF2	EPS15	0.042794
BALF2	PCSK2	0.020076
BALF2	AP3S1	0.040558
BALF2	SDF4	0.10023
BALF2	STAT1	0.011362
BALF2	FAT4	0.12834
BALF2	DTNBP1	0.011825
BALF2	PPEF1	0.048182
BALF2	CXorf67	0.047097
BALF2	FAM188B2	0.049851
BALF2	THBS1	0.014195
BALF2	TPPP	0.088697
BALF2	SYT11	0.17334
BALF2	HSP90B1	0.073232
BALF2	MACF1	0.084554
BALF2	CCDC176	0.064352
BALF2	GCH1	0.028018
BALF2	NKD2	0.15281
BALF2	FAT1	0.063874
BALF2	S100A11	0.10498
BALF2	CCDC137	0.036728
BALF2	NKD1	0.1014
BALF2	PPP2R3B	0.048496
BALF2	ACTN4	0.056085
BALF2	EHD1	0.03347
BALF2	S100A13	0.11006
BALF2	MELK	0.025092
BALF2	S100A1	0.059316
BALF2	UTRN	0.037148
BALF2	SLC34A1	0.028573
BALF2	CALB1	0.10353

BALF2	MASP2	0.14186
BALF2	PARK7	0.027642
BALF2	ANXA2R	0.033194
BALF2	EFEMP1	0.13737
BALF2	SPARC	0.060914
BALF2	NOTCH4	0.02747
BALF2	PCDH17	0.12834
BALF2	YJR030C	0.012303
BALF2	KIAA1841	0.021439
BALF2	REPS1	0.11401
BALF2	LRCOL1	0.037951
BALF2	MPTX1	0.01344
BALF2	STIM1	0.054999
BALF2	S100B	0.050549
BALF2	TNNC1	0.12407
BALF2	CETN2	0.11301
BALF2	ANXA6	0.12478
BALF2	ACTN2	0.045876
BALF2	PRSS2	0.1235
BALF2	PITPNM3	0.11097
BALF2	VIL1	0.062961
BALF2	KCNJ4	0.016634
BALF2	CETN1	0.14094
BALF2	C2CD4D	0.010502
BALF2	VLDLR	0.057484
BALF2	CDH1	0.027785
BALF2	MAP1LC3B	0.011896
BALF2	TTN	0.021963
BALF2	EGF	0.041457
BALF2	LTBP4	0.10673
BALF2	PLSCR4	0.10682
BALF2	SNX9	0.061327
BALF2	RYR2	0.022103
BALF2	PRF1	0.043561
BALF2	IL6ST	0.067417
BALF2	MYO1E	0.025222
BALF2	S100A8	0.04819
BALF2	NID2	0.14421
BALF2	NELL1	0.066093
BALF2	ytzL	0.052691
BALF2	WDR96	0.016911
BALF2	DNER	0.079638
BALF2	PEF1	0.14143
BALF2	C10orf82	0.014942
BALF2	CHP1	0.058728
BALF2	FASN	0.049132
BALF2	GPR98	0.046949
BALF2	MICU1	0.11212
BALF2	CALU	0.11015
BALF2	PPP3CA	0.015731
BALF2	BEND2	0.011389
BALF2	LRP2	0.048527
BALF2	FAM188B	0.048437
BALF2	NUCB2	0.083904
BALF2	S100A6	0.08313
BALF2	LRP8	0.10553
BALF2	CT55	0.03314
BALF2	S100P	0.076631
BALF2	RAB11FIP3	0.030617
BALF2	EFCAB4B	0.092749
BALF2	DTNA	0.051482
BALF2	REPS2	0.14692
BALF2	PPEF2	0.05158
BALF2	EFEMP2	0.14074
BALF2	ATP2A1	0.037201
BALF2	PKD2L1	0.071505
BALF2	EHD3	0.044554
BALF2	THBS2	0.11169

12989	BALF2	CALM1	0.066739
12990	BALF2	S100A4	0.14115
12991	BALF2	S100A12	0.066296
12992	BALF2	RCN1	0.14589
12993	BALF2	ACTN3	0.13666
12994	BALF2	ATP5F1	0.010987
12995	BALF2	FBN1	0.13764
12996	BALF2	F10	0.059765
12997	BALF2	CAPN1	0.037836
12998	BALF2	CELSR3	0.072712
12999	BALF2	SIRT4	0.031499
13000	BALF2	TESC	0.087969
13001	BALF2	LSM12	0.016988
13002	BALF2	C12orf57	0.023637
13003	BALF2	RPH3A	0.051155
13004	BALF2	SRI	0.063008
13005	BALF2	LRP4	0.07309
13006	BALF2	SLIT2	0.028625
13007	BALF2	MATN2	0.11637
13008	BALF2	CIB1	0.09948
13009	BALF2	PPP2R3A	0.079193
13010	BALF2	DLL1	0.12113
13011	BALF2	ACTN1	0.064056
13012	BALF2	EHD4	0.016614
13013	BALF2	UPF3B	0.016235
13014	BALF2	PRSS3	0.14506
13015	BALF2	MYL2	0.15196
13016	BALF2	EDEM1	0.062204
13017	BALF2	LRP1	0.02664
13018	BALF2	SNX19	0.07718
13019	BALF2	PDGFRB	0.017094
13020	BALF2	ITSN1	0.015344
13021	BALF2	DST	0.076249
13022	BALF2	MYL6	0.13353
13023	BALF2	COMP	0.098733
13024	BALF2	CCDC47	0.11061
13025	BALF2	ANXA7	0.050011
13026	BALF2	CHP2	0.10418
13027	BALF2	NECAB2	0.14572
13028	BALF2	CANX	0.057493
13029	BALF2	ANXA11	0.05924
13030	BALF2	PPP3CB	0.020716
13031	BALF2	MATN3	0.15363
13032	BALF2	FAM47C	0.024577
13033	BALF2	RET	0.055549
13034	BALF2	CDH23	0.035457
13035	BALF2	RAB11FIP4	0.20807
13036	BALF2	CNRIP1	0.010788
13037	BALF2	C1orf64	0.031099
13038	BALF2	AC3.5	0.016974
13039	BALF2	C2orf81	0.01918
13040	BALF2	FAM84A	0.010755
13041	BALF2	CLIC4	0.048758
13042	BALF2	FAM49B	0.017489
13043	BALF2	BNIP2	0.04958
13044	BALF2	JAG2	0.090507
13045	BALF2	CADPS	0.15332
13046	BALF2	CCAR1	0.13064
13047	BALF2	C1orf195	0.068189
13048	BALF2	CRELD2	0.13385
13049	BALF2	YBR056C-B	0.017083
13050	BALF2	GSN	0.051109
13051	BALF2	SLC11A2	0.019887
13052	BALF2	LETM1	0.12914
13053	BALF2	FAM231A	0.08451
13054	BALF2	FAM124A	0.025437
13055	BALF2	FAM188A	0.099152
13056	BALF2	AP1B1	0.015522

BALF2	RCN3	0.14075
BALF2	PLSCR1	0.058636
BALF2	NKAPL	0.043358
BALF2	TLDC2	0.021649
BALF2	LTBP1	0.14146
BALF2	F2	0.030983
BALF2	SYT1	0.045922
BALF2	NSUN5P1	0.029251
BALF2	NCS1	0.12956
BALF2	DAG1	0.030803
BALF2	C5orf48	0.016335
BALF2	NECAB3	0.0785
BALF2	SNX6	0.01946
BALF2	FAM92A1	0.010071
BALF2	NPC1L1	0.030292
BALF2	MEGF6	0.13905
BALF2	CALR	0.013987
BALF2	C16orf45	0.010189
BALF2	KCNIP2	0.043265
BALF2	HEATR5B	0.030378
BALF2	CD93	0.098888
BALF2	GRIN1	0.045252
BALF2	DHH	0.099774
BALF2	SPHK2	0.01114
BALF2	N4BP2L1	0.011138
BALF2	FAM231D	0.036182
BALF2	C11orf72	0.011071
BALF2	MYL6B	0.10816
BALF2	ATP6V0A1	0.010579
BALF2	AP1G1	0.016048
BALF2	CDHR2	0.1178
BALF2	CDH5	0.072603
BALF2	IL6R	0.02075
BALF2	SYT2	0.11781
BALF2	CAPNS1	0.012436
BALF2	TPD52	0.14338
BHRF1	ubl4ab	0.067228
BHRF1	MED1	0.012432
BHRF1	GAGE12I	0.061401
BHRF1	PAGE2B	0.18173
BHRF1	SPTA1	0.051695
BHRF1	S100A7	0.095078
BHRF1	HSPA5	0.05161
BHRF1	LDLR	0.037921
BHRF1	UPF3A	0.10139
BHRF1	RABEP1	0.10371
BHRF1	C12orf61	0.056824
BHRF1	FAM86B2	0.010865
BHRF1	PNMA6D	0.036107
BHRF1	PLSCR3	0.11676
BHRF1	ZC3HAV1L	0.090795
BHRF1	BTBD16	0.014271
BHRF1	C9orf40	0.02217
BHRF1	RPUSD4	0.038389
BHRF1	CCDC159	0.079361
BHRF1	C10orf68	0.068698
BHRF1	CCDC42	0.011411
BHRF1	CCDC162P	0.020413
BHRF1	GOLGA6L1	0.050213
BHRF1	KRI1	0.14504
BHRF1	SPATA1	0.024328
BHRF1	CCDC69	0.061393
BHRF1	YMR013W-A	0.076123
BHRF1	CASC3	0.12725
BHRF1	CYP4F30P	0.014406
BHRF1	ATP5B	0.066645
BHRF1	NIN	0.11373
BHRF1	C5orf52	0.013333

BHRF1	KCNIP1	0.10467
BHRF1	STIM2	0.11681
BHRF1	HUS1B	0.078618
BHRF1	C1orf222	0.017447
BHRF1	RPLP1	0.054528
BHRF1	PSMG3	0.012866
BHRF1	ANGEL1	0.029744
BHRF1	C19orf44	0.033784
BHRF1	FSIP1	0.1074
BHRF1	WIPF1	0.049955
BHRF1	SPTAN1	0.070321
BHRF1	TSGA10IP	0.099495
BHRF1	TNFAIP8L3	0.02237
BHRF1	LAMTOR1	0.038943
BHRF1	MASP1	0.18881
BHRF1	SCUBE3	0.16377
BHRF1	SPBC1539.02	0.060976
BHRF1	HEXIM1	0.13525
BHRF1	C2orf43	0.022878
BHRF1	GLTSCR1L	0.17391
BHRF1	MAB21L3	0.02422
BHRF1	PRR15L	0.024887
BHRF1	PROC	0.15839
BHRF1	PDCD6	0.16198
BHRF1	S100A9	0.036969
BHRF1	ANXA2	0.080948
BHRF1	MMP20	0.03676
BHRF1	smf	0.023023
BHRF1	MAGEB1	0.013937
BHRF1	MATN4	0.16837
BHRF1	NOTCH3	0.03411
BHRF1	RNH1	0.13903
BHRF1	UBAP2	0.12603
BHRF1	C17orf105	0.044034
BHRF1	RRP7B	0.017609
BHRF1	FAM110D	0.12646
BHRF1	SELPLG	0.14545
BHRF1	CLIP1	0.06053
BHRF1	BLOC1S3	0.10504
BHRF1	AMOT	0.027299
BHRF1	CCDC154	0.025697
BHRF1	PAGE5	0.078876
BHRF1	C20orf196	0.0164
BHRF1	GAGE10	0.11079
BHRF1	BOP	0.011235
BHRF1	FAM117B	0.17096
BHRF1	EIF4G2	0.029399
BHRF1	PHYHIP	0.020129
BHRF1	GAGE3	0.033027
BHRF1	MESDC1	0.027597
BHRF1	C10orf91	0.1556
BHRF1	CCDC28A	0.17536
BHRF1	TTC22	0.016517
BHRF1	FAM120C	0.029523
BHRF1	CCDC65	0.018746
BHRF1	GID4	0.051177
BHRF1	FAM83F	0.082737
BHRF1	FAM101A	0.091371
BHRF1	SNURFL	0.065186
BHRF1	THBD	0.087572
BHRF1	KIAA0040	0.039909
BHRF1	ENTHD1	0.01414
BHRF1	FAM178A	0.08402
BHRF1	MEN1	0.03309
BHRF1	RNMT	0.025394
BHRF1	C12orf36	0.02525
BHRF1	MAGOH	0.01004
BHRF1	EML1	0.15837

BHRF1	OTUD6B	0.018954
BHRF1	NOL3	0.10619
BHRF1	FAM83C	0.061782
BHRF1	IQCK	0.086813
BHRF1	ITFG2	0.016737
BHRF1	FAM166A	0.11659
BHRF1	MUM1L1	0.10245
BHRF1	FAM74A4	0.028792
BHRF1	EPS15	0.054259
BHRF1	PCSK2	0.032462
BHRF1	AP3S1	0.05321
BHRF1	SDF4	0.11837
BHRF1	NACAP1	0.028606
BHRF1	TAF11	0.083628
BHRF1	SPACA3	0.048758
BHRF1	STAT1	0.015601
BHRF1	FAT4	0.1354
BHRF1	DTNBP1	0.020993
BHRF1	SDE2	0.014405
BHRF1	PPEF1	0.072674
BHRF1	CXorf67	0.12107
BHRF1	C18orf25	0.062588
BHRF1	NELL2	0.020602
BHRF1	THEGL	0.024291
BHRF1	C11orf35	0.02649
BHRF1	C16orf86	0.032802
BHRF1	FAM188B2	0.14659
BHRF1	FAM78A	0.023437
BHRF1	MAP1LC3C	0.012131
BHRF1	NUDCD3	0.060112
BHRF1	THBS1	0.016439
BHRF1	MAGEF1	0.021226
BHRF1	KIAA1407	0.046644
BHRF1	FAM184B	0.040568
BHRF1	PRMT1	0.013472
BHRF1	CCSER1	0.10057
BHRF1	CCDC144A	0.011034
BHRF1	PNMAL2	0.085848
BHRF1	AP1M1	0.015483
BHRF1	YPR022C	0.13824
BHRF1	TPPP	0.1084
BHRF1	CCDC146	0.079209
BHRF1	SYT11	0.18384
BHRF1	CCDC150	0.042642
BHRF1	C1orf105	0.086982
BHRF1	HSP90B1	0.085544
BHRF1	MACF1	0.098949
BHRF1	CCDC66	0.018583
BHRF1	C9orf170	0.0151
BHRF1	CCDC176	0.10987
BHRF1	GCH1	0.037098
BHRF1	C10orf67	0.12255
BHRF1	LINC00597	0.078694
BHRF1	FAM131C	0.02938
BHRF1	ZBTB8OS	0.015793
BHRF1	PRDM11	0.010567
BHRF1	NKD2	0.16073
BHRF1	MT1HL1	0.080337
BHRF1	C2orf88	0.027234
BHRF1	NCOR1P1	0.015499
BHRF1	ATP2B2	0.010006
BHRF1	C19orf71	0.066052
BHRF1	SNX29P2	0.03521
BHRF1	RHPN1-AS1	0.10574
BHRF1	C6orf118	0.052429
BHRF1	IL17REL	0.025651
BHRF1	C9orf172	0.010277
BHRF1	CCDC74B	0.071571

BHRF1	FAT1	0.07008
BHRF1	NXF3	0.058455
BHRF1	LINC00471	0.080167
BHRF1	S100A11	0.12557
BHRF1	CAB8	0.013118
BHRF1	MSANTD2	0.01993
BHRF1	CCDC137	0.13005
BHRF1	TIFAB	0.16042
BHRF1	C15orf54	0.048176
BHRF1	C16orf95	0.019814
BHRF1	NUTM2A	0.053994
BHRF1	NKD1	0.11348
BHRF1	PLAC8L1	0.035027
BHRF1	CROCCP3	0.026786
BHRF1	FAM151B	0.036204
BHRF1	GCSAML	0.086236
BHRF1	FNIP2	0.16102
BHRF1	FAM47B	0.064937
BHRF1	MBD3L5	0.014925
BHRF1	BEND4	0.020626
BHRF1	SUPT20HL2	0.087802
BHRF1	PPP2R3B	0.067833
BHRF1	ACTN4	0.063502
BHRF1	SPRYD7	0.019836
BHRF1	EHD1	0.043176
BHRF1	LINC00523	0.1565
BHRF1	RABGAP1L	0.017278
BHRF1	S100A13	0.13291
BHRF1	MELK	0.033476
BHRF1	C9orf152	0.031719
BHRF1	S100A1	0.082279
BHRF1	ARL14EPL	0.12785
BHRF1	UTRN	0.05185
BHRF1	GAGE12H	0.061401
BHRF1	INTS4L2	0.021783
BHRF1	SLC34A1	0.099576
BHRF1	FAM219B	0.0229
BHRF1	CXorf21	0.052024
BHRF1	ELN	0.14225
BHRF1	FAM127A	0.048102
BHRF1	C17orf66	0.024814
BHRF1	PIH1D2	0.10178
BHRF1	C16orf11	0.018397
BHRF1	POM121L12	0.077193
BHRF1	CALB1	0.11899
BHRF1	NDEL1	0.027776
BHRF1	MASP2	0.17273
BHRF1	PARK7	0.035627
BHRF1	LINC00472	0.081893
BHRF1	C20orf96	0.022361
BHRF1	GOLGA6L4	0.11965
BHRF1	GOLGA6L6	0.11277
BHRF1	KIAA1958	0.057942
BHRF1	SLAIN1	0.15335
BHRF1	C9orf169	0.032862
BHRF1	YPL025C	0.042358
BHRF1	ZNF326	0.072512
BHRF1	KIAA0226L	0.027014
BHRF1	GAS6	0.010965
BHRF1	ANXA2R	0.11193
BHRF1	CC2D2B	0.041283
BHRF1	YML009W-B	0.15461
BHRF1	C14orf178	0.052743
BHRF1	EFEMP1	0.15799
BHRF1	C1orf168	0.074869
BHRF1	YTHDF1	0.055679
BHRF1	SPARC	0.070944
BHRF1	MRFAP1L1	0.14985

BHRF1	NOTCH4	0.032605
BHRF1	CNPY1	0.013665
BHRF1	FAM160A1	0.15486
BHRF1	PCDH17	0.1354
BHRF1	PIR	0.073459
BHRF1	WHAMMP3	0.011756
BHRF1	YJR030C	0.08353
BHRF1	KIAA1841	0.081951
BHRF1	C1orf196	0.034052
BHRF1	RNF216P1	0.081379
BHRF1	REPS1	0.13211
BHRF1	DCUN1D2	0.013952
BHRF1	LINC00476	0.018185
BHRF1	STPG2	0.012976
BHRF1	YJEFN3	0.047259
BHRF1	C18orf21	0.051678
BHRF1	XAGE2	0.13356
BHRF1	YML009W-B	0.050608
BHRF1	LRCOL1	0.10778
BHRF1	GOLGA6L18	0.10513
BHRF1	C16orf71	0.01166
BHRF1	TXNIP	0.075004
BHRF1	BCORP1	0.074429
BHRF1	FAM160B2	0.044415
BHRF1	WIBG	0.097867
BHRF1	FAM221B	0.10654
BHRF1	NOTCH2	0.010566
BHRF1	MPTX1	0.023149
BHRF1	AMER2	0.11834
BHRF1	STIM1	0.061123
BHRF1	S100B	0.068119
BHRF1	CCDC172	0.017196
BHRF1	LDOC1L	0.036388
BHRF1	AC3.5	0.086326
BHRF1	LINS	0.038769
BHRF1	TNNC1	0.14393
BHRF1	C14orf80	0.14349
BHRF1	NBPF24	0.037765
BHRF1	C1orf94	0.18097
BHRF1	CETN2	0.12641
BHRF1	ANXA6	0.14738
BHRF1	C9orf153	0.035905
BHRF1	ACTN2	0.050501
BHRF1	PRSS2	0.15053
BHRF1	CT45A5	0.053521
BHRF1	C2orf78	0.075226
BHRF1	C21orf58	0.060453
BHRF1	PITPNM3	0.12437
BHRF1	VIL1	0.068494
BHRF1	C19orf35	0.085021
BHRF1	C4orf17	0.02108
BHRF1	GAGE6	0.033027
BHRF1	YIR021W-A	0.027927
BHRF1	COMMD9	0.056808
BHRF1	KCNJ4	0.02723
BHRF1	ANGEL2	0.02093
BHRF1	MIR7-3HG	0.023061
BHRF1	PP632	0.021937
BHRF1	C17orf104	0.066971
BHRF1	CETN1	0.15947
BHRF1	PRO1854	0.045283
BHRF1	GAGE4	0.077669
BHRF1	ATP5SL	0.018954
BHRF1	C2CD4D	0.025069
BHRF1	ZK1128.3	0.045054
BHRF1	YDL022C-A	0.021684
BHRF1	PP632	0.035045
BHRF1	PNMAL1	0.056466

BHRF1	C18orf42	0.03751
BHRF1	VLDLR	0.0576
BHRF1	CDH1	0.026734
BHRF1	GAGE13	0.033027
BHRF1	MAP1LC3B	0.025074
BHRF1	TTN	0.027501
BHRF1	ZYG11A	0.012403
BHRF1	CXorf30	0.076639
BHRF1	EGF	0.045803
BHRF1	CCDC132	0.031328
BHRF1	RPS19	0.050767
BHRF1	PLSCR4	0.11318
BHRF1	DNAJC9-AS1	0.083168
BHRF1	UBQLNL	0.012485
BHRF1	CCDC152	0.020648
BHRF1	KHDC1L	0.017247
BHRF1	FAM45A	0.14435
BHRF1	KIAA1751	0.035298
BHRF1	TCP10L2	0.08073
BHRF1	CCDC57	0.011807
BHRF1	SSUH2	0.012027
BHRF1	HBA1	0.071792
BHRF1	C1orf106	0.15503
BHRF1	GOLGA6L3	0.11965
BHRF1	WWC2-AS2	0.059604
BHRF1	SNX9	0.069624
BHRF1	FBXL18	0.028301
BHRF1	GUCD1	0.037223
BHRF1	FAM195B	0.04411
BHRF1	CCDC7	0.050513
BHRF1	YFL013W-A	0.081886
BHRF1	RYR2	0.025621
BHRF1	PRF1	0.051856
BHRF1	IL6ST	0.065326
BHRF1	MYO1E	0.034765
BHRF1	GAGE12F	0.061401
BHRF1	ANKRD26P1	0.011053
BHRF1	NELFA	0.13757
BHRF1	GPRIN3	0.054548
BHRF1	FAM92B	0.018162
BHRF1	SETD2	0.01288
BHRF1	C4orf22	0.027784
BHRF1	C6orf52	0.010416
BHRF1	CCDC74A	0.071571
BHRF1	CDRT4	0.022282
BHRF1	PRR19	0.07426
BHRF1	CCDC67	0.016019
BHRF1	S100A8	0.053789
BHRF1	NID2	0.16802
BHRF1	GAREML	0.1739
BHRF1	MIMI	0.11654
BHRF1	NELL1	0.082461
BHRF1	TICRR	0.12089
BHRF1	ytzL	0.1574
BHRF1	WDR96	0.054969
BHRF1	DNER	0.084962
BHRF1	COL17A1	0.12213
BHRF1	EIF6	0.030914
BHRF1	L1TD1	0.012005
BHRF1	C15orf65	0.027626
BHRF1	FAM160B1	0.08762
BHRF1	SAMD14	0.012414
BHRF1	PEF1	0.15656
BHRF1	CT45A4	0.030379
BHRF1	NUPR1L	0.068032
BHRF1	MEGF8	0.02054
BHRF1	C10orf82	0.045333
BHRF1	ERICH1-AS1	0.10145

BHRF1	CHCHD6	0.11737
BHRF1	C3orf65	0.080819
BHRF1	CDPF1	0.013352
BHRF1	CHP1	0.062693
BHRF1	CBY3	0.047851
BHRF1	C1orf228	0.033899
BHRF1	CFTR	0.010872
BHRF1	DYDC2	0.027405
BHRF1	EP400NL	0.097543
BHRF1	FAM203B	0.076286
BHRF1	FASN	0.056284
BHRF1	HEATR5A	0.04662
BHRF1	GPR98	0.052118
BHRF1	OTUD6A	0.033154
BHRF1	MICU1	0.11752
BHRF1	CALU	0.13063
BHRF1	PPP3CA	0.023935
BHRF1	VWA9	0.027581
BHRF1	MYOZ2	0.13898
BHRF1	C16orf72	0.13836
BHRF1	BEND2	0.033623
BHRF1	EIF4B	0.020588
BHRF1	LRP2	0.054279
BHRF1	SLC23A1	0.018203
BHRF1	UNC119	0.072877
BHRF1	POM121L2	0.076024
BHRF1	GOLGA6L10	0.10513
BHRF1	SAMD13	0.015418
BHRF1	FAM188B	0.12701
BHRF1	MISP	0.14454
BHRF1	FAM231B	0.076389
BHRF1	SELO	0.11855
BHRF1	ZNF728	0.019836
BHRF1	FAM27D1	0.10027
BHRF1	NUCB2	0.10042
BHRF1	UHRF1BP1L	0.10033
BHRF1	SHANK2-AS3	0.037932
BHRF1	FAM194B	0.013968
BHRF1	UNQ6126/PRO20091	0.042856
BHRF1	HIN1L	0.010307
BHRF1	ATXN7L2	0.043957
BHRF1	PSMD5	0.048066
BHRF1	PEX11A	0.10768
BHRF1	YGL014C-A	0.12492
BHRF1	S100A6	0.098474
BHRF1	LRP8	0.10507
BHRF1	CCDC151	0.018758
BHRF1	MBD3L2	0.012308
BHRF1	CT55	0.062814
BHRF1	S100P	0.098632
BHRF1	RWDD4	0.032765
BHRF1	PRDX6	0.014369
BHRF1	CHAF1A	0.18302
BHRF1	PTGES3L	0.032489
BHRF1	TP53INP1	0.097544
BHRF1	RUNDC3B	0.02517
BHRF1	MNCb-2875	0.03029
BHRF1	FAM127C	0.041808
BHRF1	RAB11FIP3	0.040692
BHRF1	EFCAB4B	0.11293
BHRF1	C8orf74	0.037024
BHRF1	CCDC104	0.033694
BHRF1	XAGE3	0.18173
BHRF1	TEX36	0.030751
BHRF1	C11orf65	0.025332
BHRF1	ASAH2B	0.010644
BHRF1	C1orf111	0.080849
BHRF1	TCL1B	0.01101

BHRF1	CCDC87	0.026888
BHRF1	CHGB	0.18457
BHRF1	DTNA	0.069701
BHRF1	REPS2	0.1734
BHRF1	PPEF2	0.069267
BHRF1	EFEMP2	0.15529
BHRF1	MAGED4	0.012222
BHRF1	ATP2A1	0.042194
BHRF1	ATXN7L3B	0.051103
BHRF1	WLS	0.043945
BHRF1	OCEL1	0.01415
BHRF1	CT45A3	0.030469
BHRF1	CAGE1	0.014594
BHRF1	LINC00205	0.061791
BHRF1	PKD2L1	0.075532
BHRF1	FAM46B	0.066222
BHRF1	PNAS-138	0.084167
BHRF1	NCOA6	0.11008
BHRF1	CCDC26	0.074279
BHRF1	EHD3	0.055515
BHRF1	FAM181A	0.046603
BHRF1	SUPT20HL1	0.087802
BHRF1	C7orf57	0.032775
BHRF1	C22orf43	0.060361
BHRF1	FAM166B	0.021175
BHRF1	VWA5B2	0.055114
BHRF1	THBS2	0.13447
BHRF1	PRAME	0.11577
BHRF1	CALM1	0.075639
BHRF1	EIF4E2	0.014198
BHRF1	MAP7D2	0.036316
BHRF1	S100A4	0.16399
BHRF1	S100A12	0.079241
BHRF1	FAM149B1	0.060008
BHRF1	RCN1	0.17139
BHRF1	DPP4	0.01595
BHRF1	EIF4E	0.018193
BHRF1	STAG3L4	0.037736
BHRF1	ACTN3	0.14863
BHRF1	ATP5F1	0.020977
BHRF1	FBN1	0.15897
BHRF1	C12orf63	0.020632
BHRF1	F10	0.073917
BHRF1	CAPN1	0.051868
BHRF1	UCKL1	0.039697
BHRF1	DPM2	0.09548
BHRF1	FAM46A	0.077346
BHRF1	CCNYL3	0.014596
BHRF1	CELSR3	0.079878
BHRF1	RILP	0.09377
BHRF1	SS18L2	0.033622
BHRF1	GAGE12J	0.033027
BHRF1	N6AMT1	0.014866
BHRF1	DEHA2C16280g	0.066556
BHRF1	KHNYN	0.042391
BHRF1	SIRT4	0.052992
BHRF1	TESC	0.094405
BHRF1	GAGE2A	0.061401
BHRF1	ATXN7L1	0.043957
BHRF1	LSM12	0.054225
BHRF1	KIAA0907	0.017524
BHRF1	C12orf57	0.039323
BHRF1	FAM228A	0.016785
BHRF1	YAE1D1	0.065318
BHRF1	CT45A1	0.038202
BHRF1	ERICH1	0.073984
BHRF1	RPH3A	0.064526
BHRF1	SRI	0.074235

BHRF1	LRP4	0.076639
BHRF1	BVES-AS1	0.053482
BHRF1	CCDC127	0.051863
BHRF1	CCDC120	0.092757
BHRF1	MAGEB16	0.012337
BHRF1	SLIT2	0.030797
BHRF1	C1orf145	0.097873
BHRF1	OTUD1	0.025203
BHRF1	MATN2	0.1365
BHRF1	CIB1	0.10404
BHRF1	PPP2R3A	0.098666
BHRF1	AKAP3	0.051663
BHRF1	DLL1	0.11846
BHRF1	BLOC1S1	0.11238
BHRF1	FAM195A	0.16088
BHRF1	RGAG1	0.033185
BHRF1	PNMA6A	0.023108
BHRF1	C9orf62	0.013414
BHRF1	EHD4	0.028633
BHRF1	PNPT1	0.035624
BHRF1	UPF3B	0.11533
BHRF1	C5orf58	0.073087
BHRF1	LINC00114	0.014147
BHRF1	PRSS3	0.17623
BHRF1	CCDC169	0.0443
BHRF1	C7orf62	0.074454
BHRF1	MYL2	0.17038
BHRF1	EDEM1	0.070276
BHRF1	LRP1	0.032612
BHRF1	MB21D2	0.049364
BHRF1	ARMCX5	0.033367
BHRF1	ATP5A1	0.022827
BHRF1	SNX19	0.093145
BHRF1	PDGFRB	0.017921
BHRF1	SPATA2L	0.067568
BHRF1	ITSN1	0.023444
BHRF1	C15orf37	0.01477
BHRF1	BTF3L4	0.040636
BHRF1	SPIDR	0.11764
BHRF1	DST	0.075412
BHRF1	MYL6	0.15526
BHRF1	COMP	0.10998
BHRF1	CCDC47	0.11712
BHRF1	XAGE5	0.093731
BHRF1	ANXA7	0.066797
BHRF1	CHP2	0.1164
BHRF1	LYRM9	0.12427
BHRF1	MUC1	0.10592
BHRF1	FAM127B	0.076021
BHRF1	PLN	0.1123
BHRF1	CDK5R1	0.048113
BHRF1	CXorf40B	0.025617
BHRF1	NECAB2	0.17358
BHRF1	ARMC5	0.010072
BHRF1	CANX	0.064788
BHRF1	SGD1	0.073914
BHRF1	MMP15	0.020862
BHRF1	FBXO34	0.020953
BHRF1	BSDC1	0.01612
BHRF1	ANXA11	0.073677
BHRF1	PPP3CB	0.03032
BHRF1	GOLGA6L19	0.10513
BHRF1	C8orf88	0.05724
BHRF1	C5orf34	0.0413
BHRF1	LURAP1L	0.14649
BHRF1	SHCBP1L	0.015986
BHRF1	C19orf47	0.040981
BHRF1	C4orf33	0.022061

BHRF1	C12orf50	0.12154
BHRF1	C17orf102	0.05946
BHRF1	IQGAP1	0.016351
BHRF1	RMND5B	0.013382
BHRF1	IQCG	0.039903
BHRF1	MATN3	0.17965
BHRF1	PNMA6C	0.036107
BHRF1	FAM47C	0.065873
BHRF1	SH3KBP1	0.01809
BHRF1	AMOTL1	0.054797
BHRF1	C1orf173	0.021662
BHRF1	RET	0.056448
BHRF1	PAGE3	0.064827
BHRF1	FAM43B	0.012403
BHRF1	MAGED2	0.010391
BHRF1	TLX1NB	0.028686
BHRF1	CDH23	0.042638
BHRF1	LCA10	0.098227
BHRF1	STATH	0.15098
BHRF1	RAB11FIP4	0.2164
BHRF1	CNGC19	0.14015
BHRF1	C9orf106	0.022526
BHRF1	DYDC1	0.019285
BHRF1	CCDC102B	0.010818
BHRF1	CNRIP1	0.023429
BHRF1	TCERG1L	0.061679
BHRF1	SUV39H2	0.012554
BHRF1	C6orf223	0.17392
BHRF1	C1orf64	0.077695
BHRF1	PACRGL	0.030721
BHRF1	TCTEX1D1	0.032101
BHRF1	C1orf140	0.11432
BHRF1	YGL006W-A	0.026472
BHRF1	GOLGA6L20	0.066907
BHRF1	LINC00588	0.094276
BHRF1	HYPK	0.026525
BHRF1	RUNDC1	0.024488
BHRF1	NIPSNAP3B	0.020267
BHRF1	SZRD1	0.045123
BHRF1	AC3.5	0.076297
BHRF1	DDX26B	0.14577
BHRF1	LCHN	0.032704
BHRF1	FAM45B	0.12166
BHRF1	FAM229B	0.085326
BHRF1	LINC00478	0.024201
BHRF1	C2orf81	0.087018
BHRF1	FAM84A	0.032688
BHRF1	SPANXA2-OT1	0.055595
BHRF1	CLIC4	0.051353
BHRF1	EIF4G1	0.011932
BHRF1	FAM49B	0.035134
BHRF1	BNIP2	0.062393
BHRF1	ZNF767	0.062835
BHRF1	FAM199X	0.036003
BHRF1	JAG2	0.087033
BHRF1	FAM90A2P	0.011508
BHRF1	HDGFL1	0.010013
BHRF1	SWT1	0.044259
BHRF1	GAGE12G	0.061401
BHRF1	VIMP	0.052256
BHRF1	FAM117A	0.11046
BHRF1	CADPS	0.17264
BHRF1	C17orf59	0.1932
BHRF1	DRC1	0.050446
BHRF1	FAM161B	0.1644
BHRF1	CCAR1	0.1423
BHRF1	C3orf55	0.010811
BHRF1	C16orf93	0.052927

BHRF1	KIAA1908	0.023075
BHRF1	C20orf62	0.024429
BHRF1	C6orf222	0.037291
BHRF1	LINC00299	0.010346
BHRF1	CCDC171	0.049328
BHRF1	PLAUR	0.071989
BHRF1	FAM178B	0.06611
BHRF1	MIMI	0.012491
BHRF1	C1orf195	0.17803
BHRF1	CRELD2	0.15923
BHRF1	NPIPA5	0.013348
BHRF1	PRR23A	0.072132
BHRF1	C15orf32	0.013434
BHRF1	C2orf72	0.11836
BHRF1	YBR056C-B	0.048196
BHRF1	SCIMP	0.13961
BHRF1	ARMC12	0.061787
BHRF1	GSN	0.061834
BHRF1	SLC11A2	0.020024
BHRF1	PLAC8	0.015265
BHRF1	C3orf22	0.085142
BHRF1	EXOC3L4	0.048436
BHRF1	CYCB3-1	0.12895
BHRF1	COA7	0.027758
BHRF1	CABP1	0.02127
BHRF1	C22orf26	0.092804
BHRF1	LETM1	0.13613
BHRF1	TNFAIP8L1	0.124
BHRF1	pkmyt1	0.074811
BHRF1	PGBD4	0.040762
BHRF1	SPHK1	0.010377
BHRF1	CRYGN	0.058699
BHRF1	FAM231A	0.27962
BHRF1	TFB1M	0.028772
BHRF1	C6orf229	0.074143
BHRF1	FAM124A	0.16327
BHRF1	AMER1	0.12181
BHRF1	FAM188A	0.1159
BHRF1	MAGEB4	0.016135
BHRF1	AP1B1	0.026588
BHRF1	RCN3	0.16772
BHRF1	PLSCR1	0.063154
BHRF1	GAGE5	0.033027
BHRF1	SPAC5H10.02c	0.010552
BHRF1	GAGE12B	0.061401
BHRF1	SLC6A4	0.013092
BHRF1	NKAPL	0.12937
BHRF1	NCKAP5L	0.12726
BHRF1	CCDC153	0.010618
BHRF1	PRR21	0.043722
BHRF1	TLDC2	0.080566
BHRF1	LTBP1	0.1658
BHRF1	MSANTD1	0.027877
BHRF1	F2	0.038895
BHRF1	SMEK3P	0.062666
BHRF1	SYT1	0.051799
BHRF1	C11orf52	0.035492
BHRF1	NSUN5P1	0.043949
BHRF1	PI4K2A	0.027805
BHRF1	NCS1	0.13785
BHRF1	TCAP	0.068261
BHRF1	C12orf71	0.019707
BHRF1	SRRM5	0.030306
BHRF1	CCDC81	0.033896
BHRF1	FBLN5	0.14854
BHRF1	DAG1	0.050565
BHRF1	AGTRAP	0.078733
BHRF1	C1orf192	0.053102

BHRF1	HRK	0.11685
BHRF1	C2orf62	0.035858
BHRF1	YAL042C-A	0.024414
BHRF1	C19orf67	0.033198
BHRF1	C5orf48	0.061521
BHRF1	SH3BP5L	0.079757
BHRF1	CCDC17	0.020846
BHRF1	NECAB3	0.098781
BHRF1	MLC1	0.022449
BHRF1	C22orf42	0.018462
BHRF1	C19orf82	0.088999
BHRF1	SUMO4	0.013815
BHRF1	SNX6	0.031352
BHRF1	SECISBP2L	0.10405
BHRF1	C11orf96	0.085303
BHRF1	CDKN2AIPNL	0.13624
BHRF1	INTS4L1	0.015317
BHRF1	FAM92A1	0.012609
BHRF1	NACA	0.036589
BHRF1	NPC1L1	0.039375
BHRF1	NBEAP1	0.010121
BHRF1	RSRC1	0.13954
BHRF1	MEGF6	0.16527
BHRF1	CALR	0.01714
BHRF1	RSRC2	0.12187
BHRF1	C16orf45	0.028963
BHRF1	GOLGA6L9	0.10513
BHRF1	ALS2CR11	0.042795
BHRF1	ZNRD1-AS1	0.024279
BHRF1	KCNIP2	0.052418
BHRF1	FBRSL1	0.075407
BHRF1	LRRC14	0.012206
BHRF1	ZK1128.3	0.13612
BHRF1	CUEDC1	0.062558
BHRF1	HEATR5B	0.081741
BHRF1	CTAGE15	0.021352
BHRF1	LINC00303	0.019195
BHRF1	CD93	0.10456
BHRF1	CCDC54	0.021167
BHRF1	GRIN1	0.047447
BHRF1	CLINT1	0.015779
BHRF1	C20orf195	0.038101
BHRF1	C19orf66	0.095977
BHRF1	DHH	0.10894
BHRF1	GAGE2E	0.033027
BHRF1	UNQ6125/PRO20090	0.11348
BHRF1	SPHK2	0.021189
BHRF1	VIPAS39	0.048279
BHRF1	CT45A2	0.030469
BHRF1	N4BP2L1	0.018008
BHRF1	FAM231D	0.14114
BHRF1	MAGEB17	0.018107
BHRF1	KIAA0087	0.083708
BHRF1	C11orf72	0.03321
BHRF1	C9orf171	0.11071
BHRF1	IQCF3	0.020702
BHRF1	LINC00615	0.07867
BHRF1	CCDC94	0.11832
BHRF1	MYL6B	0.12965
BHRF1	RBBP8NL	0.034606
BHRF1	RGAG4	0.06843
BHRF1	ATP6V0A1	0.01919
BHRF1	SCG5	0.14618
BHRF1	TTC23L	0.026737
BHRF1	MED4	0.061689
BHRF1	AP1G1	0.024571
BHRF1	CDHR2	0.12417
BHRF1	CDR2L	0.029957

BHRF1	SNX17	0.0131
BHRF1	CDH5	0.079498
BHRF1	GR6	0.029898
BHRF1	IL6R	0.022261
BHRF1	TP53INP2	0.07591
BHRF1	SYT2	0.12778
BHRF1	CAPNS1	0.031618
BHRF1	GTF2E2	0.1344
BHRF1	TPD52	0.16657
EBNA1	HPS4	0.16214
EBNA1	CBX7	0.14399
EBNA1	ACR	0.13658
EBNA1	ACVR1	0.020508
EBNA1	TRIB3	0.044514
EBNA1	ubl4ab	0.036835
EBNA1	FPR1	0.12236
EBNA1	BRAP	0.1106
EBNA1	SERPINB9	0.11979
EBNA1	SLC25A25	0.042153
EBNA1	MACROD1	0.18305
EBNA1	ITGB5	0.10198
EBNA1	HID1	0.14206
EBNA1	ECT2	0.024218
EBNA1	PEX10	0.14104
EBNA1	NLRP2	0.11811
EBNA1	RBL1	0.072278
EBNA1	BIN2	0.21561
EBNA1	BBS10	0.15805
EBNA1	USP2	0.068472
EBNA1	MED1	0.014146
EBNA1	CDC34	0.069779
EBNA1	CDK10	0.06775
EBNA1	EPHA3	0.079388
EBNA1	EFCAB1	0.044917
EBNA1	PIK3CD	0.090448
EBNA1	RAB7A	0.10057
EBNA1	TGFBR2	0.017184
EBNA1	N4BP1	0.16648
EBNA1	CTDNEP1	0.071759
EBNA1	FAM58A	0.093731
EBNA1	PSMD4	0.095488
EBNA1	FZD8	0.056858
EBNA1	PPP5C	0.066765
EBNA1	SLC2A4	0.1047
EBNA1	KEL	0.093818
EBNA1	IFT122	0.19667
EBNA1	NAE1	0.10344
EBNA1	PRPF6	0.16323
EBNA1	SPTA1	0.19673
EBNA1	SEC31A	0.11585
EBNA1	GZMA	0.1639
EBNA1	S100A7	0.25458
EBNA1	IL4	0.026646
EBNA1	NANOS1	0.1833
EBNA1	SLC26A8	0.14532
EBNA1	UBE2H	0.10793
EBNA1	HSPA5	0.13755
EBNA1	LDLR	0.10847
EBNA1	NEDD4	0.038411
EBNA1	GSPT2	0.11275
EBNA1	SERPINB5	0.15551
EBNA1	SSBP1	0.15297
EBNA1	BIRC6	0.095028
EBNA1	KLK13	0.18797
EBNA1	AGO2	0.075422
EBNA1	HEPACAM2	0.12131
EBNA1	CENPW	0.13633
EBNA1	UNC13D	0.13487

EBNA1	UPF3A	0.21128
EBNA1	ARHGAP33	0.14871
EBNA1	RABEP1	0.2297
EBNA1	SKA3	0.14335
EBNA1	VPS54	0.1844
EBNA1	EIF5A	0.075628
EBNA1	LIMK1	0.052426
EBNA1	SLC5A1	0.12662
EBNA1	LRP2BP	0.21561
EBNA1	MAPK8	0.048142
EBNA1	KCNS1	0.13553
EBNA1	PXN	0.07858
EBNA1	NUP85	0.087509
EBNA1	SOX13	0.051186
EBNA1	CTNND2	0.12283
EBNA1	UBE2D3	0.069249
EBNA1	C12orf61	0.037644
EBNA1	KRT5	0.12186
EBNA1	HSD17B8	0.12775
EBNA1	BTNL8	0.20909
EBNA1	PLSCR3	0.35346
EBNA1	KREMEN1	0.19147
EBNA1	TCF25	0.048659
EBNA1	NR0B1	0.031779
EBNA1	SNX20	0.17566
EBNA1	BIRC7	0.10804
EBNA1	MCM6	0.052796
EBNA1	FKBP10	0.049862
EBNA1	KRT17	0.098888
EBNA1	MAPKAPK2	0.034413
EBNA1	NOSIP	0.11367
EBNA1	SMURF2	0.06518
EBNA1	ANKIB1	0.17587
EBNA1	ATP6V0D2	0.1335
EBNA1	ORAI3	0.20909
EBNA1	PFKM	0.088004
EBNA1	ELAVL2	0.23412
EBNA1	RPUSD4	0.32528
EBNA1	EMR1	0.010521
EBNA1	CD248	0.048123
EBNA1	ASIC1	0.12478
EBNA1	CALN1	0.036955
EBNA1	F9	0.075093
EBNA1	CRB1	0.071126
EBNA1	TRAPPC2	0.087594
EBNA1	NIP7	0.24014
EBNA1	C16orf70	0.19053
EBNA1	USP12	0.15623
EBNA1	DDB2	0.11433
EBNA1	PIP5K1A	0.081487
EBNA1	PEAK1	0.11954
EBNA1	SUN1	0.10662
EBNA1	SFTPD	0.17441
EBNA1	PIP5K1B	0.19323
EBNA1	FAT3	0.037352
EBNA1	TAB3	0.075647
EBNA1	SBF2	0.10597
EBNA1	SLC7A11	0.087436
EBNA1	BNIP1	0.095003
EBNA1	FGFR1OP	0.08193
EBNA1	PCDH20	0.038413
EBNA1	PTPRH	0.023143
EBNA1	CALCOCO2	0.14723
EBNA1	USHBP1	0.19927
EBNA1	CD1B	0.16907
EBNA1	LALBA	0.027375
EBNA1	BRD4	0.11617
EBNA1	TFAP2B	0.021122

EBNA1	PRPF4B	0.085539
EBNA1	MYF5	0.079271
EBNA1	ARHGEF12	0.049357
EBNA1	RNF2	0.06778
EBNA1	LLGL2	0.13545
EBNA1	MYH7	0.14355
EBNA1	SORBS3	0.085877
EBNA1	POLD2	0.082034
EBNA1	SNAPIN	0.13501
EBNA1	STX3	0.10187
EBNA1	PCDH12	0.030656
EBNA1	PDE2A	0.042402
EBNA1	PLXNB1	0.083367
EBNA1	CARTPT	0.10217
EBNA1	HMGB1	0.036328
EBNA1	KCNJ5	0.17742
EBNA1	MLKL	0.076344
EBNA1	MKS1	0.13253
EBNA1	MAPK7	0.053609
EBNA1	USP11	0.13649
EBNA1	DTX1	0.076694
EBNA1	ADAMTS5	0.067743
EBNA1	RANBP9	0.1295
EBNA1	SPHKAP	0.20909
EBNA1	PXMP2	0.17418
EBNA1	SYTL1	0.15839
EBNA1	SOCS7	0.15193
EBNA1	TYRP1	0.13938
EBNA1	ZFPM1	0.023921
EBNA1	UBA1	0.094375
EBNA1	BTBD2	0.20364
EBNA1	EIF3J	0.22336
EBNA1	RTN2	0.1644
EBNA1	RP1	0.11049
EBNA1	F5	0.13356
EBNA1	IGSF1	0.16367
EBNA1	KCND2	0.08423
EBNA1	PTPN1	0.034242
EBNA1	FKBP1B	0.077178
EBNA1	APLP2	0.069105
EBNA1	C10orf62	0.22232
EBNA1	RNF167	0.11872
EBNA1	UNC119B	0.19375
EBNA1	RACGAP1	0.035101
EBNA1	RBL2	0.10787
EBNA1	CTBP2	0.10398
EBNA1	JAK3	0.058131
EBNA1	CCNB1IP1	0.12969
EBNA1	ACTA1	0.085037
EBNA1	PSMA1	0.062931
EBNA1	DHX30	0.19455
EBNA1	NLRX1	0.094987
EBNA1	VASP	0.12941
EBNA1	PCDHGA1	0.038413
EBNA1	CABP7	0.036955
EBNA1	CASC3	0.13485
EBNA1	WASF2	0.13483
EBNA1	CD58	0.16751
EBNA1	MAP2	0.11262
EBNA1	TBL1XR1	0.071886
EBNA1	ZFYVE27	0.12116
EBNA1	APBB1	0.029427
EBNA1	DNM3	0.017785
EBNA1	PCNT	0.15568
EBNA1	U2AF1	0.14793
EBNA1	ARHGDIA	0.1228
EBNA1	PDXP	0.080527
EBNA1	BOC	0.12767

EBNA1	P4HB	0.039279
EBNA1	SNRPA	0.17509
EBNA1	GAREM	0.22232
EBNA1	ALAS2	0.10582
EBNA1	IGFBP5	0.099693
EBNA1	PRKCD	0.019011
EBNA1	DVL2	0.078871
EBNA1	CHRD	0.10564
EBNA1	BICD2	0.22844
EBNA1	APOB	0.015948
EBNA1	KIF7	0.12795
EBNA1	ADAM9	0.030565
EBNA1	ATP5B	0.14322
EBNA1	NIN	0.33287
EBNA1	UBB	0.027046
EBNA1	TXNDC17	0.17809
EBNA1	ARHGEF1	0.06246
EBNA1	PYCARD	0.019909
EBNA1	MUL1	0.011209
EBNA1	ITCH	0.072151
EBNA1	SYTL2	0.10011
EBNA1	PGR	0.017136
EBNA1	CARD11	0.08975
EBNA1	LMO2	0.067245
EBNA1	KCNIP1	0.27614
EBNA1	CDC27	0.10787
EBNA1	VPS26A	0.13414
EBNA1	BATF	0.060057
EBNA1	SH3BP5	0.21457
EBNA1	CDH18	0.036654
EBNA1	AKTIP	0.12987
EBNA1	ANKEF1	0.044917
EBNA1	TNIP1	0.14006
EBNA1	TLE3	0.13961
EBNA1	STIM2	0.3315
EBNA1	FAM109A	0.15336
EBNA1	GP1BA	0.11341
EBNA1	XRN1	0.12719
EBNA1	WBP2	0.21059
EBNA1	CEACAM1	0.15488
EBNA1	NUP107	0.11158
EBNA1	ERBB3	0.052982
EBNA1	ARL11	0.18333
EBNA1	MEGF9	0.041587
EBNA1	SLAIN2	0.2136
EBNA1	SSSCA1	0.19513
EBNA1	CCDC130	0.21056
EBNA1	BNIPL	0.13544
EBNA1	KRT7	0.080268
EBNA1	ZHX2	0.026894
EBNA1	CTCFL	0.082738
EBNA1	RPLP1	0.17258
EBNA1	DERL3	0.16495
EBNA1	SERGEF	0.15011
EBNA1	CD247	0.13867
EBNA1	DSN1	0.13158
EBNA1	ELK1	0.034166
EBNA1	TRAF2	0.030667
EBNA1	TCEA2	0.084848
EBNA1	TMED10	0.11343
EBNA1	RALBP1	0.1606
EBNA1	NDUFAF4	0.22907
EBNA1	BUB1B	0.042236
EBNA1	CST5	0.24739
EBNA1	NFKBIB	0.092436
EBNA1	THOC5	0.15383
EBNA1	NAIP	0.14346
EBNA1	DPY30	0.12232

EBNA1	EFCAB14	0.044917
EBNA1	PVRL3	0.14811
EBNA1	LRIF1	0.17816
EBNA1	TNFAIP3	0.015028
EBNA1	TNKS	0.060386
EBNA1	TMEM30B	0.20278
EBNA1	PYDC1	0.14028
EBNA1	PITPNM2	0.022493
EBNA1	SOX4	0.013401
EBNA1	NCAPD2	0.13754
EBNA1	MIA3	0.15261
EBNA1	UHRF2	0.066605
EBNA1	PDIA3	0.040108
EBNA1	BIRC3	0.1005
EBNA1	PABPN1	0.11719
EBNA1	TAF3	0.089585
EBNA1	COMMD5	0.18246
EBNA1	ACAN	0.11398
EBNA1	CLASP2	0.059101
EBNA1	NACA	0.12674
EBNA1	JAK2	0.012903
EBNA1	WIPF1	0.22962
EBNA1	SPTAN1	0.24032
EBNA1	NDE1	0.062662
EBNA1	BECN1	0.10278
EBNA1	PTPN3	0.031321
EBNA1	CD81	0.11079
EBNA1	CDC37	0.10453
EBNA1	NOL8	0.18578
EBNA1	PPM1E	0.058914
EBNA1	SPNS1	0.18326
EBNA1	RIMS1	0.10519
EBNA1	ATRIP	0.18002
EBNA1	COPS2	0.14185
EBNA1	DDR1	0.068768
EBNA1	COL13A1	0.11964
EBNA1	RAD52	0.15244
EBNA1	TCF7	0.021002
EBNA1	LAMTOR1	0.12578
EBNA1	DHPS	0.21537
EBNA1	SMC3	0.07175
EBNA1	STAT5A	0.03026
EBNA1	THAP1	0.094028
EBNA1	TUBA3C	0.01393
EBNA1	CDK5RAP2	0.053674
EBNA1	PRX	0.1501
EBNA1	MCRS1	0.13032
EBNA1	POP4	0.18118
EBNA1	FBLN1	0.051538
EBNA1	EDIL3	0.038476
EBNA1	MASP1	0.36252
EBNA1	TBL1X	0.032182
EBNA1	AIF1	0.011278
EBNA1	ZNF205	0.10783
EBNA1	INPP5D	0.06366
EBNA1	GCC2	0.097809
EBNA1	SCUBE3	0.43005
EBNA1	KIF13B	0.070648
EBNA1	SLC9A7	0.11134
EBNA1	CNOT3	0.12024
EBNA1	PTPRM	0.030365
EBNA1	BCL9	0.15412
EBNA1	NGFR	0.042926
EBNA1	TADA3	0.022109
EBNA1	STRAP	0.10053
EBNA1	PROZ	0.01374
EBNA1	LSM3	0.15989
EBNA1	DDO	0.1284

EBNA1	PEA15	0.1723
EBNA1	RPS27L	0.12154
EBNA1	RBFOX1	0.1913
EBNA1	AMIGO2	0.15292
EBNA1	MTSS1	0.082499
EBNA1	PSMC3	0.04899
EBNA1	HUWE1	0.10403
EBNA1	UXT	0.08954
EBNA1	TRAPPC4	0.20458
EBNA1	TRIM17	0.1312
EBNA1	HECTD3	0.10498
EBNA1	TH	0.023407
EBNA1	TNFRSF10B	0.13436
EBNA1	SCAP	0.12058
EBNA1	C1QB	0.17538
EBNA1	TRIM13	0.076613
EBNA1	FBXO7	0.15357
EBNA1	HIPK1	0.046462
EBNA1	CAMK2A	0.056031
EBNA1	APOH	0.089458
EBNA1	PTPRS	0.036554
EBNA1	ATXN10	0.14244
EBNA1	SCN5A	0.05847
EBNA1	GEMIN5	0.19165
EBNA1	COMMD7	0.18486
EBNA1	FHIT	0.064773
EBNA1	ADAM17	0.025753
EBNA1	CSNK1A1	0.036227
EBNA1	ATG4B	0.16713
EBNA1	TERF1	0.056144
EBNA1	MAL2	0.18078
EBNA1	PCDHGB1	0.038413
EBNA1	AGTR1	0.058053
EBNA1	SEC24B	0.096949
EBNA1	ALYREF	0.14882
EBNA1	AATK	0.11302
EBNA1	NCF4	0.12662
EBNA1	MED8	0.11387
EBNA1	ATG16L1	0.17614
EBNA1	USP19	0.094899
EBNA1	RIN3	0.21185
EBNA1	LDOC1	0.18428
EBNA1	FSBP	0.16206
EBNA1	SH3D19	0.11658
EBNA1	TNS4	0.17999
EBNA1	MORF4L2	0.14843
EBNA1	TSR2	0.21559
EBNA1	SAV1	0.12558
EBNA1	EPB41	0.047778
EBNA1	CDH16	0.038413
EBNA1	PDE9A	0.083584
EBNA1	PEX19	0.11967
EBNA1	SYNCRIP	0.10419
EBNA1	NAGK	0.23979
EBNA1	SLC22A6	0.13942
EBNA1	BMP7	0.018642
EBNA1	PPAP2B	0.036309
EBNA1	TIA1	0.1802
EBNA1	SNRPG	0.12503
EBNA1	TP53RK	0.13608
EBNA1	PCDH18	0.037353
EBNA1	TECPR1	0.17472
EBNA1	RRM2	0.11626
EBNA1	CD300LD	0.20279
EBNA1	KCNJ10	0.073728
EBNA1	CUL4A	0.14904
EBNA1	CD244	0.17551
EBNA1	KCNH1	0.10178

14281	EBNA1	PAIP2B	0.20275
14282	EBNA1	PTPRN	0.097261
14283	EBNA1	KAT7	0.066154
14284	EBNA1	PML	0.019194
14285	EBNA1	RPGR	0.15967
14286	EBNA1	KBTBD6	0.20423
14287	EBNA1	WDR5	0.11341
14288	EBNA1	GOLGA4	0.13737
14289	EBNA1	MALL	0.15915
14290	EBNA1	CHMP4B	0.16383
14291	EBNA1	TLK2	0.11086
14292	EBNA1	NLRC4	0.067373
14293	EBNA1	PROC	0.3144
14294	EBNA1	PDCD6	0.34684
14295	EBNA1	DKC1	0.17498
14296	EBNA1	USP32	0.036456
14297	EBNA1	OTUD7B	0.102
14298	EBNA1	S100A9	0.11808
14299	EBNA1	ANXA2	0.20221
14300	EBNA1	TMPO	0.14821
14301	EBNA1	PSMD2	0.088407
14302	EBNA1	MMP20	0.25347
14303	EBNA1	RAET1G	0.1847
14304	EBNA1	DAZL	0.13411
14305	EBNA1	NR2C2AP	0.17517
14306	EBNA1	MCM4	0.064887
14307	EBNA1	RTN4	0.052116
14308	EBNA1	RUNX1	0.043262
14309	EBNA1	TOP3A	0.090619
14310	EBNA1	CDKN1A	0.044463
14311	EBNA1	KIF23	0.094087
14312	EBNA1	IL13RA1	0.19746
14313	EBNA1	EZH2	0.10261
14314	EBNA1	MATN4	0.45294
14315	EBNA1	SLX4IP	0.22232
14316	EBNA1	POLG	0.12985
14317	EBNA1	FMR1	0.22794
14318	EBNA1	MAP1A	0.046303
14319	EBNA1	NOTCH3	0.16376
14320	EBNA1	DNMT3L	0.11397
14321	EBNA1	VPS72	0.13476
14322	EBNA1	TIMM50	0.063938
14323	EBNA1	CXXC1	0.09203
14324	EBNA1	PVALB	0.049807
14325	EBNA1	RNH1	0.42583
14326	EBNA1	LRRC47	0.20242
14327	EBNA1	FOXRED2	0.24843
14328	EBNA1	PKM	0.078907
14329	EBNA1	ARHGEF6	0.065451
14330	EBNA1	PIK3C2B	0.1462
14331	EBNA1	MFF	0.079505
14332	EBNA1	MMRN2	0.20489
14333	EBNA1	FBXL12	0.20278
14334	EBNA1	EGFL7	0.02691
14335	EBNA1	POR	0.010712
14336	EBNA1	ASPN	0.056308
14337	EBNA1	MYL1	0.038417
14338	EBNA1	ARL4D	0.094318
14339	EBNA1	MED23	0.10855
14340	EBNA1	ADIPOQ	0.01716
14341	EBNA1	TBC1D24	0.15509
14342	EBNA1	HLA-DRB1	0.038657
14343	EBNA1	TGFB3	0.02832
14344	EBNA1	KIR2DL3	0.13242
14345	EBNA1	EGLN1	0.085491
14346	EBNA1	PLXNC1	0.11988
14347	EBNA1	SIAH1	0.054418
14348	EBNA1	C2CD5	0.042312

EBNA1	SNX27	0.13232
EBNA1	PTPRJ	0.015822
EBNA1	NDUFA9	0.1254
EBNA1	PVRL4	0.16946
EBNA1	PPP6R1	0.16607
EBNA1	SPSB2	0.18993
EBNA1	ROBO1	0.072418
EBNA1	PAIP1	0.17372
EBNA1	WRAP53	0.24278
EBNA1	MORF4L1	0.13347
EBNA1	CLIP1	0.17985
EBNA1	ATP1A1	0.06264
EBNA1	YTHDF3	0.22232
EBNA1	HCK	0.051689
EBNA1	USP6	0.11342
EBNA1	TEK	0.037552
EBNA1	TAP2	0.048586
EBNA1	UGP2	0.094796
EBNA1	EXOSC5	0.13988
EBNA1	UBXN7	0.15714
EBNA1	TSHR	0.13031
EBNA1	PLXDC2	0.20909
EBNA1	AMOT	0.12728
EBNA1	MAATS1	0.20916
EBNA1	TBC1D8	0.017147
EBNA1	ALOX5AP	0.079995
EBNA1	DLK2	0.063012
EBNA1	BHLHE40	0.028581
EBNA1	TTLL6	0.12678
EBNA1	SLC4A1	0.12579
EBNA1	PTK7	0.075192
EBNA1	ADI1	0.11565
EBNA1	PCDHB5	0.035257
EBNA1	TOB1	0.17758
EBNA1	WDR12	0.1281
EBNA1	HM13	0.10832
EBNA1	JUNB	0.015375
EBNA1	SLC25A17	0.10741
EBNA1	VPS28	0.15877
EBNA1	URM1	0.18581
EBNA1	NUF2	0.12747
EBNA1	CABS1	0.03791
EBNA1	KLF10	0.017185
EBNA1	MAGEA2	0.13476
EBNA1	TRAK2	0.077994
EBNA1	CASP8AP2	0.10894
EBNA1	FNTB	0.11378
EBNA1	RNF7	0.11416
EBNA1	HNRNPR	0.16105
EBNA1	CHFR	0.087701
EBNA1	CLN3	0.027274
EBNA1	CRTC1	0.11692
EBNA1	QKI	0.19606
EBNA1	RTN4R	0.15862
EBNA1	RBM15	0.18196
EBNA1	STXBP2	0.12182
EBNA1	POLR1A	0.10634
EBNA1	EPCAM	0.17664
EBNA1	UMODL1	0.034051
EBNA1	GNL3	0.099579
EBNA1	VPS33B	0.12454
EBNA1	UACA	0.10733
EBNA1	GLS	0.11438
EBNA1	PAK3	0.025765
EBNA1	C5orf30	0.16452
EBNA1	MYL4	0.039714
EBNA1	CD8B	0.14151
EBNA1	EIF4G2	0.22322

EBNA1	TOM1L1	0.14472
EBNA1	RABGGTB	0.20051
EBNA1	COLQ	0.092704
EBNA1	ORAI1	0.1341
EBNA1	RAD21	0.10896
EBNA1	CD80	0.12052
EBNA1	PCDH11Y	0.038413
EBNA1	METTL1	0.1842
EBNA1	MMS19	0.095152
EBNA1	ITGB3	0.03368
EBNA1	INSR	0.01606
EBNA1	TLN2	0.052849
EBNA1	ASB9	0.16541
EBNA1	TRPC3	0.10983
EBNA1	CIZ1	0.12601
EBNA1	CHD8	0.054113
EBNA1	RPL11	0.11211
EBNA1	NLRP1	0.08129
EBNA1	NCBP1	0.09151
EBNA1	EDC3	0.19799
EBNA1	MAD2L2	0.12281
EBNA1	MGRN1	0.077005
EBNA1	WDR77	0.11199
EBNA1	CRKL	0.14818
EBNA1	PPP2R2B	0.085382
EBNA1	TIMP3	0.14814
EBNA1	ALDH7A1	0.15411
EBNA1	GDI2	0.12714
EBNA1	PTK6	0.056864
EBNA1	ZRSR2	0.13128
EBNA1	CD200R1	0.17393
EBNA1	UHRF1	0.042286
EBNA1	SULT2A1	0.16084
EBNA1	NEBL	0.18922
EBNA1	KLKB1	0.17238
EBNA1	IL1R1	0.14964
EBNA1	LATS2	0.053325
EBNA1	CREB3L3	0.044082
EBNA1	NCOA7	0.13187
EBNA1	PCDHA5	0.036102
EBNA1	RABGAP1	0.13373
EBNA1	SMARCD1	0.13152
EBNA1	SHROOM2	0.092823
EBNA1	ZNF148	0.016853
EBNA1	PCDH11X	0.036172
EBNA1	KBTBD7	0.20423
EBNA1	ERCC4	0.048591
EBNA1	LTBR	0.13096
EBNA1	ARHGAP8	0.16084
EBNA1	MYOCD	0.028437
EBNA1	GH1	0.11474
EBNA1	CTH	0.045882
EBNA1	SHANK2	0.19839
EBNA1	CDH20	0.03731
EBNA1	SLA2	0.19117
EBNA1	CLTC	0.10418
EBNA1	FCGR1A	0.15181
EBNA1	SH3GL1	0.19997
EBNA1	ARVCF	0.16271
EBNA1	CBX2	0.11254
EBNA1	PLEKHA5	0.10473
EBNA1	EIF3F	0.19893
EBNA1	PPP3R2	0.044917
EBNA1	CIITA	0.088507
EBNA1	UPF2	0.2084
EBNA1	CACNB2	0.13065
EBNA1	SIN3B	0.11712
EBNA1	TNPO3	0.1269

EBNA1	CACNA1C	0.098484
EBNA1	SH3RF1	0.098755
EBNA1	TRPM6	0.13505
EBNA1	RAB5A	0.10349
EBNA1	ERBB2IP	0.1057
EBNA1	OPA1	0.061928
EBNA1	PPP2R5D	0.13014
EBNA1	BIN1	0.11321
EBNA1	BCL2L11	0.025544
EBNA1	SYNRG	0.03912
EBNA1	GNAI1	0.047267
EBNA1	HIST2H3A	0.10281
EBNA1	PSMB9	0.047824
EBNA1	RBMY1A1	0.22163
EBNA1	EEF1G	0.14527
EBNA1	COL7A1	0.15522
EBNA1	RAG1	0.050233
EBNA1	RRAD	0.15515
EBNA1	STX4	0.091431
EBNA1	LOXL4	0.15548
EBNA1	GFI1	0.065693
EBNA1	SIK3	0.075621
EBNA1	ROBO2	0.085505
EBNA1	NUP214	0.1318
EBNA1	NAA50	0.24807
EBNA1	STAU1	0.16434
EBNA1	CHAMP1	0.080716
EBNA1	ARHGEF15	0.14671
EBNA1	TPCN1	0.088723
EBNA1	MERTK	0.07794
EBNA1	UBL4A	0.18394
EBNA1	CREBZF	0.046398
EBNA1	STAT3	0.015775
EBNA1	ZNF488	0.098533
EBNA1	ACIN1	0.14464
EBNA1	BARD1	0.11601
EBNA1	PPFIA4	0.19672
EBNA1	CKB	0.15596
EBNA1	TNS3	0.19149
EBNA1	KMT2C	0.14645
EBNA1	KLHL20	0.11645
EBNA1	RAD23A	0.12284
EBNA1	HPCAL4	0.055068
EBNA1	VPS4A	0.089372
EBNA1	COCH	0.14817
EBNA1	PSPC1	0.18289
EBNA1	STK40	0.087205
EBNA1	SRPX2	0.11974
EBNA1	ORC4	0.084151
EBNA1	THBD	0.26062
EBNA1	GLYCTK	0.24013
EBNA1	ARHGEF4	0.056186
EBNA1	SMAD5	0.015564
EBNA1	DCHS1	0.037277
EBNA1	CREM	0.04172
EBNA1	ZFR	0.1831
EBNA1	RTP4	0.18606
EBNA1	FSHR	0.088895
EBNA1	FANCM	0.10591
EBNA1	EEF1D	0.13473
EBNA1	TRADD	0.051859
EBNA1	PYGO1	0.11735
EBNA1	DHX8	0.16168
EBNA1	MTERF	0.12929
EBNA1	BRAF	0.11389
EBNA1	NPBWR2	0.15515
EBNA1	ARPC2	0.1241
EBNA1	ARFGEF2	0.076367

EBNA1	TTC19	0.15746
EBNA1	BSG	0.095584
EBNA1	ORM1	0.19968
EBNA1	HSD17B10	0.084582
EBNA1	RBM5	0.13126
EBNA1	BOP1	0.10556
EBNA1	NOX1	0.055652
EBNA1	DSC2	0.037494
EBNA1	CAPS	0.017057
EBNA1	CLSPN	0.12911
EBNA1	MLST8	0.15465
EBNA1	WTIP	0.097066
EBNA1	SPTB	0.09666
EBNA1	IL1R2	0.21846
EBNA1	KLF13	0.080555
EBNA1	MSN	0.049828
EBNA1	SPDL1	0.097102
EBNA1	UBE2D4	0.098991
EBNA1	SMARCE1	0.092712
EBNA1	PIGA	0.1559
EBNA1	MEN1	0.029143
EBNA1	GNMT	0.16847
EBNA1	ZBTB14	0.10463
EBNA1	SCAMP1	0.16281
EBNA1	SEC23A	0.083893
EBNA1	RND1	0.078225
EBNA1	BCKDHB	0.17372
EBNA1	PDLIM7	0.12136
EBNA1	RNMT	0.24852
EBNA1	TNFRSF21	0.18269
EBNA1	TTF2	0.071086
EBNA1	TOPORS	0.048576
EBNA1	DDX47	0.16598
EBNA1	FREM2	0.017769
EBNA1	GNAI2	0.037882
EBNA1	DDX42	0.18128
EBNA1	GAPDHS	0.12786
EBNA1	CDKN1C	0.14071
EBNA1	GRIA1	0.076299
EBNA1	ANKLE2	0.13656
EBNA1	NUP153	0.089582
EBNA1	VPS41	0.10932
EBNA1	MAGOH	0.18609
EBNA1	SKI	0.021599
EBNA1	CEP41	0.1341
EBNA1	RAC1	0.022714
EBNA1	INTS9	0.17861
EBNA1	IPO5	0.1375
EBNA1	VDAC1	0.076408
EBNA1	PRMT5	0.11727
EBNA1	TNFAIP1	0.11036
EBNA1	CRNN	0.04006
EBNA1	ING4	0.097962
EBNA1	PECAM1	0.1346
EBNA1	EML1	0.42571
EBNA1	CEP170	0.17724
EBNA1	C19orf68	0.22232
EBNA1	ZWILCH	0.15746
EBNA1	VAV2	0.03293
EBNA1	KIF24	0.15112
EBNA1	MAGED1	0.11031
EBNA1	PTGER4	0.07028
EBNA1	RPRD1B	0.13112
EBNA1	CALCRL	0.051378
EBNA1	RBPMS	0.17058
EBNA1	DES	0.10141
EBNA1	WDR4	0.1639
EBNA1	PSMC1	0.070343

EBNA1	USP5	0.09251
EBNA1	CITED1	0.017097
EBNA1	HAT1	0.15731
EBNA1	SNCAIP	0.13976
EBNA1	FZD3	0.088404
EBNA1	LIN28A	0.098116
EBNA1	MPDZ	0.15404
EBNA1	ARID2	0.10865
EBNA1	TLDC1	0.22232
EBNA1	COMMD3	0.22232
EBNA1	SMAD7	0.019947
EBNA1	SERPINA1	0.14937
EBNA1	NCK1	0.057346
EBNA1	GABBR1	0.047813
EBNA1	CRTAC1	0.055656
EBNA1	SCP2	0.064385
EBNA1	MAD2L1	0.08553
EBNA1	PRRC2B	0.22232
EBNA1	KCNJ3	0.16694
EBNA1	BYSL	0.12565
EBNA1	ERN1	0.01717
EBNA1	SPRTN	0.13194
EBNA1	MYD88	0.03766
EBNA1	LSM5	0.1611
EBNA1	FBXW8	0.20282
EBNA1	SPPL2A	0.10843
EBNA1	PLS1	0.039716
EBNA1	SYVN1	0.069058
EBNA1	CACYBP	0.15213
EBNA1	INS	0.025271
EBNA1	IRAK4	0.074719
EBNA1	EPS15	0.22318
EBNA1	PLS3	0.042436
EBNA1	MYO5B	0.1832
EBNA1	WWC1	0.10806
EBNA1	HUS1	0.12426
EBNA1	PCSK2	0.22818
EBNA1	CRHR1	0.050724
EBNA1	APLF	0.10674
EBNA1	YY1	0.039897
EBNA1	NKRF	0.15235
EBNA1	ZNF335	0.10783
EBNA1	AURKB	0.026343
EBNA1	RRAGC	0.066391
EBNA1	LZTS1	0.028702
EBNA1	CD48	0.12822
EBNA1	VWCE	0.043666
EBNA1	CLEC3B	0.039608
EBNA1	PRMT8	0.18645
EBNA1	INPP5B	0.1312
EBNA1	ARHGEF16	0.049589
EBNA1	BST2	0.087187
EBNA1	NHLH2	0.11115
EBNA1	PEX11G	0.1698
EBNA1	OGDHL	0.19925
EBNA1	FKBP8	0.089369
EBNA1	DISC1	0.09659
EBNA1	PUM2	0.24571
EBNA1	ESPL1	0.090234
EBNA1	PCGF1	0.11213
EBNA1	CDH8	0.036654
EBNA1	AP3S1	0.24772
EBNA1	UHMK1	0.13396
EBNA1	BUB3	0.095615
EBNA1	RBMY1B	0.20974
EBNA1	SPRR3	0.066455
EBNA1	SDF4	0.31017
EBNA1	KIF14	0.11549

EBNA1	LEPR	0.17316
EBNA1	ANKHD1	0.24764
EBNA1	MGP	0.036538
EBNA1	BCKDHA	0.19008
EBNA1	TRIM33	0.047992
EBNA1	KIF5B	0.1054
EBNA1	HEG1	0.023294
EBNA1	PRKCQ	0.016022
EBNA1	PHIP	0.11384
EBNA1	TAF11	0.14148
EBNA1	TRPC7	0.11133
EBNA1	LAMTOR2	0.13311
EBNA1	MAZ	0.14389
EBNA1	HIST1H1E	0.11392
EBNA1	WSB1	0.20055
EBNA1	CDC45	0.11829
EBNA1	YAF2	0.098483
EBNA1	MYH9	0.050374
EBNA1	EID3	0.13865
EBNA1	SPACA3	0.1782
EBNA1	INSIG2	0.083669
EBNA1	CDCA8	0.10894
EBNA1	STAT1	0.052167
EBNA1	GJA1	0.026047
EBNA1	PSEN2	0.013796
EBNA1	FAT4	0.41219
EBNA1	DTNBP1	0.1141
EBNA1	CUL9	0.11978
EBNA1	PNMA2	0.18003
EBNA1	WWP1	0.080291
EBNA1	PLIN3	0.14121
EBNA1	KCNIP4	0.044549
EBNA1	USP34	0.1556
EBNA1	VPS18	0.11928
EBNA1	ARHGAP4	0.11276
EBNA1	PPEF1	0.32413
EBNA1	ZW10	0.061346
EBNA1	GRIN3A	0.069746
EBNA1	CXorf67	0.025675
EBNA1	NPW	0.1722
EBNA1	MAPK11	0.046968
EBNA1	A2M	0.1036
EBNA1	DCTN1	0.12318
EBNA1	FTSJ3	0.2337
EBNA1	ETV1	0.034777
EBNA1	PRKAA2	0.013468
EBNA1	NELL2	0.3203
EBNA1	CALB2	0.059635
EBNA1	DDX60	0.14071
EBNA1	CLTB	0.16248
EBNA1	PCDHA7	0.036102
EBNA1	CHD4	0.024188
EBNA1	ZC3H10	0.1706
EBNA1	ARRB2	0.050252
EBNA1	PRDX1	0.1421
EBNA1	RHOG	0.074259
EBNA1	TTC3	0.086033
EBNA1	SPON1	0.20248
EBNA1	IRAK3	0.04521
EBNA1	CYFIP1	0.10435
EBNA1	NR2F1	0.024485
EBNA1	ULK2	0.17243
EBNA1	SRSF3	0.18412
EBNA1	PDZK1	0.1896
EBNA1	MCC	0.14317
EBNA1	KAT2B	0.018119
EBNA1	CROCC	0.069642
EBNA1	LAMTOR3	0.15197

EBNA1	ARRB1	0.036915
EBNA1	ELOVL4	0.13507
EBNA1	UBE3A	0.070047
EBNA1	TCF4	0.03294
EBNA1	GTF3C1	0.12316
EBNA1	SMC1A	0.065698
EBNA1	DMAP1	0.11881
EBNA1	CHEK2	0.018197
EBNA1	STX1A	0.082901
EBNA1	DRD2	0.013511
EBNA1	APH1A	0.074649
EBNA1	BAZ1B	0.10246
EBNA1	AKIP1	0.1844
EBNA1	PCDHGC5	0.038413
EBNA1	KAT2A	0.10241
EBNA1	COL4A3BP	0.037732
EBNA1	TRPC4	0.10466
EBNA1	KIF5C	0.12178
EBNA1	FZD10	0.080304
EBNA1	PIN1	0.11733
EBNA1	TLX3	0.044183
EBNA1	SUV420H2	0.19732
EBNA1	PCDHGA7	0.038413
EBNA1	HECTD1	0.1283
EBNA1	NME1	0.087376
EBNA1	NOP58	0.15338
EBNA1	MAP1LC3C	0.25056
EBNA1	RFC3	0.084581
EBNA1	ATP6V1E1	0.13128
EBNA1	ATP11B	0.061006
EBNA1	RNF14	0.060188
EBNA1	TARBP2	0.1092
EBNA1	TAB1	0.06704
EBNA1	RHOA	0.014258
EBNA1	KCNH2	0.076593
EBNA1	KRT20	0.13596
EBNA1	ZNF646	0.10783
EBNA1	MAP4K4	0.099279
EBNA1	DCAF11	0.21589
EBNA1	THBS1	0.042062
EBNA1	RUNX3	0.016877
EBNA1	LMTK2	0.090096
EBNA1	DIS3	0.1285
EBNA1	ARR3	0.14696
EBNA1	ABCD3	0.12193
EBNA1	CMKLR1	0.11645
EBNA1	STK25	0.078483
EBNA1	ICAM3	0.13209
EBNA1	MRPL49	0.17288
EBNA1	EIF3I	0.22336
EBNA1	ARL2BP	0.16725
EBNA1	UBTF	0.11615
EBNA1	FGF2	0.018053
EBNA1	PFN1	0.092139
EBNA1	DLG3	0.083938
EBNA1	RBCK1	0.01885
EBNA1	DSG2	0.028872
EBNA1	MAP3K12	0.024456
EBNA1	CCNE1	0.033715
EBNA1	RHOC	0.054058
EBNA1	ADRA2B	0.079617
EBNA1	ATXN3	0.078383
EBNA1	EPB41L3	0.073634
EBNA1	CEBPG	0.018328
EBNA1	GIT2	0.09582
EBNA1	SNX33	0.20908
EBNA1	STK24	0.050421
EBNA1	PCDHB2	0.033158

EBNA1	MIF	0.073115
EBNA1	ISG20L2	0.12136
EBNA1	BICD1	0.13132
EBNA1	AP2M1	0.13587
EBNA1	ING2	0.061068
EBNA1	PIK3CG	0.056962
EBNA1	ADORA1	0.028053
EBNA1	CDC42EP2	0.12355
EBNA1	SF3A1	0.20417
EBNA1	DSG4	0.029625
EBNA1	TCTEX1D4	0.22232
EBNA1	TMCC2	0.20909
EBNA1	PLG	0.076033
EBNA1	TEAD3	0.031042
EBNA1	NOS2	0.054149
EBNA1	PRMT1	0.20609
EBNA1	USP10	0.10726
EBNA1	LASP1	0.1238
EBNA1	RASSF5	0.16109
EBNA1	CRB2	0.040121
EBNA1	DYNLT1	0.088723
EBNA1	ZMIZ2	0.10357
EBNA1	ALDOB	0.024978
EBNA1	S100Z	0.044917
EBNA1	MED26	0.10125
EBNA1	LRP5	0.064185
EBNA1	HSD17B12	0.086704
EBNA1	DGKQ	0.13432
EBNA1	CRP	0.13679
EBNA1	ARIH2	0.087234
EBNA1	DHX36	0.094825
EBNA1	DDOST	0.13107
EBNA1	AGR3	0.26315
EBNA1	AP1M1	0.17911
EBNA1	PUF60	0.12382
EBNA1	GMCL1P1	0.1954
EBNA1	SNX8	0.17105
EBNA1	MAPK6	0.044118
EBNA1	SPOP	0.17717
EBNA1	PRKCZ	0.021834
EBNA1	HDDC2	0.13934
EBNA1	SETDB1	0.15348
EBNA1	SEC22B	0.12018
EBNA1	ATP7A	0.01584
EBNA1	AGR2	0.24042
EBNA1	FHL2	0.026726
EBNA1	ADM	0.058795
EBNA1	TPPP	0.31299
EBNA1	ZNF746	0.077317
EBNA1	PRPF4	0.16232
EBNA1	DBNDD2	0.20909
EBNA1	BMP10	0.076676
EBNA1	TBXA2R	0.078668
EBNA1	SYT4	0.093808
EBNA1	CENPT	0.1119
EBNA1	RAD51D	0.13449
EBNA1	SYT11	0.39152
EBNA1	CD5	0.14548
EBNA1	SH2D4A	0.16444
EBNA1	ICAM1	0.04829
EBNA1	GZMB	0.14183
EBNA1	GGA2	0.13976
EBNA1	DCUN1D1	0.20913
EBNA1	LPAR1	0.043404
EBNA1	MOB3B	0.19362
EBNA1	MSH6	0.031502
EBNA1	SNRNP200	0.11028
EBNA1	PTCH1	0.028726

EBNA1	EIF2B1	0.10136
EBNA1	PDE3B	0.063737
EBNA1	DRD3	0.041382
EBNA1	CTNNA3	0.073522
EBNA1	DDX17	0.12281
EBNA1	RECQL	0.076254
EBNA1	BCS1L	0.12405
EBNA1	SH3PXD2A	0.17308
EBNA1	SFPQ	0.16325
EBNA1	PARPBP	0.1685
EBNA1	FBXL15	0.17321
EBNA1	FANCA	0.16194
EBNA1	EIF2B3	0.15091
EBNA1	KDM1A	0.010681
EBNA1	PCDHAC2	0.036102
EBNA1	RBMS1	0.17567
EBNA1	ATP6V0D1	0.11351
EBNA1	TPD52L1	0.1536
EBNA1	WDFY3	0.16693
EBNA1	PSMA4	0.046542
EBNA1	HSP90B1	0.22039
EBNA1	NR2C1	0.036646
EBNA1	PCDH7	0.037179
EBNA1	PIAS1	0.063477
EBNA1	HOXB7	0.036814
EBNA1	TNFSF13B	0.080016
EBNA1	MPP5	0.16679
EBNA1	TRIM2	0.13002
EBNA1	DDAH2	0.14068
EBNA1	ATPAF2	0.20285
EBNA1	MACF1	0.24754
EBNA1	AIFM1	0.077231
EBNA1	CKAP5	0.12024
EBNA1	POLR3F	0.099389
EBNA1	S100A3	0.02299
EBNA1	MEIS1	0.029426
EBNA1	WWOX	0.1314
EBNA1	ADORA2A	0.027329
EBNA1	SOS1	0.045495
EBNA1	APOD	0.087134
EBNA1	TXNL4A	0.17039
EBNA1	EVL	0.13339
EBNA1	BOK	0.12213
EBNA1	ALG2	0.12319
EBNA1	GLMN	0.18205
EBNA1	MAGEC2	0.17005
EBNA1	TPSAB1	0.21321
EBNA1	TXNRD2	0.21106
EBNA1	FGF21	0.17773
EBNA1	PCGF3	0.1229
EBNA1	MSR1	0.18929
EBNA1	DUOX2	0.092398
EBNA1	RTN1	0.16432
EBNA1	RBM14	0.088166
EBNA1	OSBPL6	0.14773
EBNA1	CCDC176	0.050439
EBNA1	STBD1	0.18474
EBNA1	CASQ2	0.042665
EBNA1	MCTP2	0.041209
EBNA1	PRKG1	0.061734
EBNA1	LPXN	0.091535
EBNA1	UBE2A	0.10722
EBNA1	EPHA7	0.086966
EBNA1	SNTB2	0.16692
EBNA1	CX3CL1	0.051781
EBNA1	PCDHGB4	0.037277
EBNA1	PPIE	0.18511
EBNA1	KLRK1	0.10969

EBNA1	TBC1D25	0.2192
EBNA1	DNAJC7	0.17595
EBNA1	GCH1	0.11938
EBNA1	PTGES3	0.088881
EBNA1	ANKRD1	0.063839
EBNA1	VPS11	0.18655
EBNA1	POLE3	0.13068
EBNA1	SLC9A3	0.13215
EBNA1	OGN	0.14483
EBNA1	HNRNPU	0.1187
EBNA1	OPRD1	0.099172
EBNA1	MDM2	0.032992
EBNA1	NKD2	0.31834
EBNA1	LSAMP	0.1883
EBNA1	PTPN23	0.053142
EBNA1	ADAMTSL2	0.1307
EBNA1	FLNC	0.17317
EBNA1	MAGI3	0.10641
EBNA1	SLAMF1	0.11479
EBNA1	IFT57	0.14728
EBNA1	ADAM23	0.096112
EBNA1	AP5B1	0.20073
EBNA1	TRIM29	0.044029
EBNA1	EIF2B5	0.1489
EBNA1	ZNF350	0.11534
EBNA1	MAPK8IP1	0.04955
EBNA1	PTPN4	0.037735
EBNA1	ATP2B2	0.063276
EBNA1	BTG2	0.10284
EBNA1	PEX3	0.12845
EBNA1	GHITM	0.17951
EBNA1	TBC1D9	0.018813
EBNA1	DOT1L	0.17161
EBNA1	JMJD1C	0.18749
EBNA1	GJD3	0.18124
EBNA1	FNBP1L	0.21134
EBNA1	ITGAV	0.05159
EBNA1	TXLNB	0.20909
EBNA1	SEC13	0.13369
EBNA1	CDH10	0.03561
EBNA1	CDKN2AIP	0.16583
EBNA1	KEAP1	0.13423
EBNA1	EPHA10	0.14334
EBNA1	CNOT6	0.13552
EBNA1	LCK	0.029456
EBNA1	HIPK2	0.024513
EBNA1	PSMC4	0.064836
EBNA1	RAD50	0.035387
EBNA1	FAT1	0.25156
EBNA1	REV1	0.083686
EBNA1	BAZ1A	0.089199
EBNA1	OCRL	0.08822
EBNA1	DYRK1B	0.066603
EBNA1	ABCF1	0.12685
EBNA1	HDAC3	0.020991
EBNA1	FBLN7	0.03759
EBNA1	NXF3	0.22716
EBNA1	H2AFY	0.098248
EBNA1	NGLY1	0.15806
EBNA1	MTCH1	0.16269
EBNA1	ERCC8	0.13754
EBNA1	CRELD1	0.043322
EBNA1	TGOLN2	0.15326
EBNA1	HABP4	0.12328
EBNA1	XAB2	0.14977
EBNA1	S100A11	0.34919
EBNA1	ITGA5	0.075638
EBNA1	GDF7	0.10935

EBNA1	SRSF5	0.15873
EBNA1	USH1G	0.13682
EBNA1	SORBS1	0.067624
EBNA1	CHMP1B	0.15724
EBNA1	HFE2	0.1439
EBNA1	IRF3	0.013384
EBNA1	EFCAB6	0.019747
EBNA1	RBBP6	0.09413
EBNA1	MAP3K3	0.074658
EBNA1	ESR2	0.027963
EBNA1	NCAPG	0.15645
EBNA1	ECSIT	0.1931
EBNA1	LRRFIP2	0.19886
EBNA1	AIRE	0.03587
EBNA1	XPO4	0.17239
EBNA1	NKD1	0.32221
EBNA1	NR1H2	0.015406
EBNA1	PPP1R9B	0.086894
EBNA1	PLEKHM2	0.10996
EBNA1	CYBB	0.081064
EBNA1	DERL2	0.13589
EBNA1	ACACA	0.097884
EBNA1	SPTBN4	0.038938
EBNA1	STAP1	0.035888
EBNA1	EDA2R	0.12321
EBNA1	PPP6C	0.11729
EBNA1	RBFOX2	0.12326
EBNA1	GCK	0.052647
EBNA1	RPS10	0.096245
EBNA1	CD2AP	0.15307
EBNA1	RAP2A	0.087945
EBNA1	CD1A	0.16854
EBNA1	CBX8	0.1338
EBNA1	RNF146	0.09388
EBNA1	IGHG1	0.13721
EBNA1	KIF18A	0.067859
EBNA1	GABPB1	0.018453
EBNA1	GREM1	0.027084
EBNA1	INTS12	0.13777
EBNA1	UBE2N	0.046577
EBNA1	MAGEB18	0.22232
EBNA1	MED31	0.1216
EBNA1	MAT2B	0.090073
EBNA1	HMCN1	0.079161
EBNA1	SLC9A3R2	0.086583
EBNA1	KNSTRN	0.11198
EBNA1	ODF2	0.04974
EBNA1	SERPINE1	0.033942
EBNA1	PCBP2	0.13031
EBNA1	SPC25	0.12747
EBNA1	RBM25	0.14051
EBNA1	PKP1	0.16007
EBNA1	DNAJB5	0.18123
EBNA1	EIF1AY	0.32197
EBNA1	FNIP2	0.16004
EBNA1	DEAF1	0.09004
EBNA1	ARID4B	0.12775
EBNA1	EMR2	0.016446
EBNA1	TREML1	0.17343
EBNA1	MOV10	0.15239
EBNA1	MAGI1	0.13069
EBNA1	PTGIS	0.024038
EBNA1	ARL4A	0.13686
EBNA1	BPTF	0.065434
EBNA1	STMN2	0.13273
EBNA1	CAPS2	0.044917
EBNA1	ARHGAP32	0.071083
EBNA1	PRRC2A	0.1844

EBNA1	MKKS	0.054915
EBNA1	GMNN	0.11284
EBNA1	PDZD11	0.19491
EBNA1	MYLPF	0.033919
EBNA1	PSMD10	0.037764
EBNA1	SDHAF2	0.19191
EBNA1	ST13	0.12524
EBNA1	APOA1	0.030109
EBNA1	PSMA5	0.049685
EBNA1	PPP2R3B	0.31517
EBNA1	PEG10	0.11466
EBNA1	INO80	0.084902
EBNA1	CTNNBIP1	0.11512
EBNA1	CENPH	0.084256
EBNA1	HDAC9	0.031902
EBNA1	HEY1	0.020274
EBNA1	ACTN4	0.19304
EBNA1	FBXO46	0.22232
EBNA1	KIAA0141	0.20285
EBNA1	DTX3L	0.12324
EBNA1	EHD1	0.20258
EBNA1	ATL1	0.057555
EBNA1	KIR2DL1	0.1744
EBNA1	RAB25	0.20342
EBNA1	TIMM44	0.12233
EBNA1	PARD6B	0.11572
EBNA1	GTF2E1	0.10994
EBNA1	RAN	0.066079
EBNA1	LILRA4	0.20909
EBNA1	EEA1	0.13284
EBNA1	SUN2	0.089097
EBNA1	SEPT8	0.1726
EBNA1	PINK1	0.013571
EBNA1	CTU2	0.18544
EBNA1	OXA1L	0.13089
EBNA1	INTS10	0.16914
EBNA1	LPL	0.034348
EBNA1	PKMYT1	0.032308
EBNA1	ERCC6	0.044222
EBNA1	PARVA	0.082245
EBNA1	VCAN	0.025183
EBNA1	CDR2	0.17885
EBNA1	KRIT1	0.13483
EBNA1	RABAC1	0.18914
EBNA1	RUNX2	0.013566
EBNA1	AMFR	0.044046
EBNA1	NEU4	0.095456
EBNA1	CNOT6L	0.12456
EBNA1	RPS19BP1	0.17461
EBNA1	COMMD1	0.11697
EBNA1	SEPT2	0.062698
EBNA1	RBM4	0.078222
EBNA1	ZNRF1	0.1559
EBNA1	GRB7	0.057465
EBNA1	ZSCAN21	0.030236
EBNA1	TESK1	0.10328
EBNA1	CABP4	0.039136
EBNA1	GEMIN6	0.14643
EBNA1	MAPK12	0.044343
EBNA1	MMP2	0.045668
EBNA1	INHA	0.068247
EBNA1	POLE4	0.14304
EBNA1	EIF2B2	0.14359
EBNA1	RPTN	0.06874
EBNA1	EXOSC1	0.17626
EBNA1	DSP	0.068299
EBNA1	RGS19	0.091287
EBNA1	ZFYVE20	0.095428

EBNA1	UBE2G2	0.099342
EBNA1	RMI1	0.17354
EBNA1	AIP	0.044084
EBNA1	S100A13	0.27319
EBNA1	STUB1	0.076328
EBNA1	MELK	0.20714
EBNA1	EXO1	0.060816
EBNA1	GEM	0.099452
EBNA1	NEK1	0.076812
EBNA1	HPGDS	0.050722
EBNA1	INHBB	0.12918
EBNA1	HSP90AB1	0.081582
EBNA1	CORO1B	0.18368
EBNA1	HTRA3	0.22536
EBNA1	PSMB1	0.049299
EBNA1	S100A1	0.30021
EBNA1	EIF3H	0.21661
EBNA1	TMED9	0.13697
EBNA1	PRMT6	0.12721
EBNA1	MYL7	0.060771
EBNA1	SNRPC	0.1776
EBNA1	SUMO1	0.063956
EBNA1	PPIA	0.11431
EBNA1	SUMO2	0.1737
EBNA1	USP1	0.13046
EBNA1	ARHGAP24	0.076591
EBNA1	UTRN	0.23879
EBNA1	TRAF3IP3	0.20909
EBNA1	STAM2	0.10074
EBNA1	EGR2	0.039873
EBNA1	UBE2D2	0.092353
EBNA1	EGFL8	0.053038
EBNA1	RAD23B	0.11331
EBNA1	HHIP	0.04592
EBNA1	GNS	0.028703
EBNA1	PCGF5	0.11958
EBNA1	S100A2	0.043979
EBNA1	PALM	0.093383
EBNA1	IDE	0.017808
EBNA1	AP2A2	0.12452
EBNA1	ASH2L	0.099462
EBNA1	IGF1	0.016898
EBNA1	SLC34A1	0.17842
EBNA1	SCUBE2	0.043666
EBNA1	TBC1D2B	0.075474
EBNA1	MCM7	0.070944
EBNA1	TOP2A	0.046546
EBNA1	PIK3CA	0.072537
EBNA1	MYL9	0.026839
EBNA1	NOP56	0.2013
EBNA1	TNFRSF1B	0.13593
EBNA1	BAHD1	0.12038
EBNA1	E4F1	0.012816
EBNA1	ENG	0.040845
EBNA1	MYO18A	0.12868
EBNA1	ACP1	0.021016
EBNA1	AQP1	0.045965
EBNA1	MAPKAPK3	0.038795
EBNA1	SWI5	0.19993
EBNA1	LTF	0.099196
EBNA1	PPM1B	0.089159
EBNA1	TBC1D10A	0.16409
EBNA1	EFHD1	0.041038
EBNA1	MAP2K3	0.026466
EBNA1	SMC2	0.10707
EBNA1	ZC3HAV1	0.1524
EBNA1	SAMM50	0.11201
EBNA1	FASLG	0.058718

EBNA1	VWF	0.11221
EBNA1	ZFPM2	0.049363
EBNA1	CIB4	0.044917
EBNA1	CRADD	0.10778
EBNA1	SPTBN1	0.058768
EBNA1	KANK1	0.10296
EBNA1	NUP37	0.11579
EBNA1	FLII	0.12818
EBNA1	EMD	0.10381
EBNA1	ECHS1	0.084837
EBNA1	NTRK1	0.044344
EBNA1	RBM17	0.21165
EBNA1	THBS3	0.022667
EBNA1	CUBN	0.02047
EBNA1	PSPH	0.012233
EBNA1	S100A14	0.022173
EBNA1	STAT6	0.13603
EBNA1	MPZL2	0.16926
EBNA1	INVS	0.13681
EBNA1	USP4	0.12225
EBNA1	RBPJ	0.012547
EBNA1	ATG3	0.12498
EBNA1	PRRG3	0.040146
EBNA1	ULK1	0.042068
EBNA1	SPOCK2	0.058895
EBNA1	HYAL2	0.039294
EBNA1	STAMBP	0.086236
EBNA1	CTLA4	0.16909
EBNA1	SLIT3	0.016426
EBNA1	ARPC5	0.13475
EBNA1	NELFE	0.15499
EBNA1	CLEC1B	0.17997
EBNA1	ATRAID	0.11525
EBNA1	FBXO4	0.14998
EBNA1	HNRNPL	0.14294
EBNA1	TLE2	0.12738
EBNA1	PPP2R2A	0.12701
EBNA1	CALB1	0.27919
EBNA1	CPSF2	0.18595
EBNA1	MAP4K1	0.091851
EBNA1	PCGF6	0.025419
EBNA1	PEX5	0.080489
EBNA1	RYK	0.11497
EBNA1	RASSF6	0.20252
EBNA1	NDEL1	0.07776
EBNA1	HHLA3	0.22232
EBNA1	NPHS2	0.17314
EBNA1	DIP2A	0.086846
EBNA1	GAN	0.18262
EBNA1	PDIA4	0.071749
EBNA1	MIER1	0.11508
EBNA1	MAP3K11	0.063519
EBNA1	BAZ2A	0.10318
EBNA1	CAPN11	0.045774
EBNA1	UFD1L	0.15868
EBNA1	LIN7A	0.12726
EBNA1	SETMAR	0.12243
EBNA1	VAV3	0.033358
EBNA1	MASP2	0.3645
EBNA1	BATF2	0.062355
EBNA1	NUDT21	0.17634
EBNA1	PCDHA9	0.038413
EBNA1	PARK7	0.13893
EBNA1	RP2	0.0888
EBNA1	FKBP1A	0.095605
EBNA1	TAF6L	0.0648
EBNA1	SLC25A23	0.042153
EBNA1	TRAF7	0.1189

EBNA1	BCL2L10	0.13278
EBNA1	RCN2	0.041908
EBNA1	PRCP	0.20843
EBNA1	TAOK3	0.10594
EBNA1	SSTR2	0.082703
EBNA1	PPP4C	0.083889
EBNA1	HSPA1A	0.047718
EBNA1	TAP1	0.062026
EBNA1	ATG9A	0.20733
EBNA1	CALML4	0.044917
EBNA1	MAP3K5	0.012714
EBNA1	PES1	0.1092
EBNA1	CCM2	0.1857
EBNA1	TRIM39	0.094298
EBNA1	ABI1	0.066864
EBNA1	PTBP1	0.18328
EBNA1	MAGEA3	0.22232
EBNA1	ZNF224	0.1011
EBNA1	RAMP2	0.062699
EBNA1	TAF13	0.026019
EBNA1	CAPSL	0.043657
EBNA1	RRAS2	0.094163
EBNA1	RAB4A	0.088107
EBNA1	FGF19	0.1717
EBNA1	RAB5B	0.1228
EBNA1	RNF32	0.15001
EBNA1	ARL14	0.24677
EBNA1	LRWD1	0.10841
EBNA1	TXNDC9	0.2038
EBNA1	ARL6	0.063061
EBNA1	EIF3C	0.22336
EBNA1	EIF3M	0.24028
EBNA1	ZNF326	0.15414
EBNA1	MYOZ3	0.21522
EBNA1	KLHL2	0.20592
EBNA1	DTX2	0.10145
EBNA1	COMMD10	0.22232
EBNA1	NOM1	0.20949
EBNA1	ACVR2A	0.021954
EBNA1	GRK5	0.02833
EBNA1	ITGB1BP2	0.012769
EBNA1	CENPA	0.053469
EBNA1	UBXN6	0.15579
EBNA1	SYNPO2	0.17424
EBNA1	IL5RA	0.19076
EBNA1	AIF1L	0.030427
EBNA1	CDH11	0.034352
EBNA1	RABL6	0.14648
EBNA1	APEX1	0.05895
EBNA1	ELK3	0.016869
EBNA1	CBLL1	0.11858
EBNA1	PCDHAC1	0.036102
EBNA1	EPPIN	0.18394
EBNA1	GAS6	0.03226
EBNA1	BET1	0.11446
EBNA1	AMPH	0.17213
EBNA1	ABLIM1	0.13145
EBNA1	RANGAP1	0.073976
EBNA1	CIRBP	0.14486
EBNA1	ERLEC1	0.19612
EBNA1	KIFAP3	0.13441
EBNA1	ABCB1	0.10926
EBNA1	PCDHB6	0.033158
EBNA1	EFEMP1	0.33789
EBNA1	EIF4A3	0.13582
EBNA1	LPAR2	0.16155
EBNA1	ZAP70	0.067659
EBNA1	IL1A	0.08115

EBNA1	COMMD2	0.22232
EBNA1	SDPR	0.12443
EBNA1	AGRN	0.061607
EBNA1	MKL1	0.11399
EBNA1	ZNF281	0.01187
EBNA1	TTC37	0.17347
EBNA1	PNMA1	0.15639
EBNA1	APC2	0.1335
EBNA1	CCDC53	0.21197
EBNA1	NIPSNAP3A	0.20955
EBNA1	SPATA13	0.042882
EBNA1	MYO9B	0.12603
EBNA1	C1D	0.14102
EBNA1	BAP1	0.080291
EBNA1	ULK3	0.078111
EBNA1	TBCB	0.16937
EBNA1	SPARC	0.17459
EBNA1	GTF2H1	0.043039
EBNA1	CREB5	0.039362
EBNA1	TCHHL1	0.044917
EBNA1	EWSR1	0.16735
EBNA1	F2RL1	0.025597
EBNA1	OC90	0.033792
EBNA1	EHMT2	0.13253
EBNA1	PALLD	0.14517
EBNA1	NOTCH4	0.12461
EBNA1	RAPGEF3	0.095012
EBNA1	PIK3C3	0.10241
EBNA1	TERT	0.10435
EBNA1	NUTF2	0.18803
EBNA1	ARPC4	0.1452
EBNA1	EXOSC10	0.08678
EBNA1	PCDHB1	0.038413
EBNA1	MEAF6	0.1418
EBNA1	KLHL12	0.23329
EBNA1	TSPYL5	0.12692
EBNA1	PIM1	0.021598
EBNA1	DLX4	0.051056
EBNA1	MBD2	0.090611
EBNA1	MST4	0.023952
EBNA1	TRAIP	0.12279
EBNA1	DFNB31	0.10487
EBNA1	CYTH2	0.098424
EBNA1	CTR9	0.099776
EBNA1	NAA15	0.097159
EBNA1	MATN1	0.21762
EBNA1	NOTCH2NL	0.039
EBNA1	CA4	0.073394
EBNA1	DEPDC1	0.12792
EBNA1	ID4	0.11023
EBNA1	PCDH17	0.41219
EBNA1	PIR	0.19417
EBNA1	IGSF8	0.17252
EBNA1	EPHA4	0.058535
EBNA1	SET	0.051215
EBNA1	MCL1	0.085309
EBNA1	PRICKLE1	0.09264
EBNA1	GPR37L1	0.15998
EBNA1	ARHGEF2	0.0188
EBNA1	SLC9A1	0.073095
EBNA1	KITLG	0.091783
EBNA1	DSC3	0.037818
EBNA1	SLC22A3	0.1228
EBNA1	B2M	0.099432
EBNA1	GLRB	0.09155
EBNA1	KCNN4	0.14057
EBNA1	PHB	0.06403
EBNA1	PRDX4	0.24322

EBNA1	APC	0.012497
EBNA1	PMS2	0.10051
EBNA1	XK	0.21694
EBNA1	SCAF11	0.127
EBNA1	EXOC3	0.20107
EBNA1	EIF3B	0.24214
EBNA1	CLGN	0.013967
EBNA1	CIB2	0.044917
EBNA1	LDB3	0.15778
EBNA1	PPM1D	0.060027
EBNA1	KHDRBS3	0.18548
EBNA1	ERBB4	0.023328
EBNA1	EXD3	0.22232
EBNA1	ADH1A	0.13517
EBNA1	SPEN	0.051287
EBNA1	CEP72	0.14966
EBNA1	ALB	0.056082
EBNA1	EAF2	0.11921
EBNA1	CASK	0.067436
EBNA1	ESRRG	0.041352
EBNA1	TARDBP	0.06096
EBNA1	RASSF4	0.20886
EBNA1	REPS1	0.41145
EBNA1	PLEK	0.054823
EBNA1	CIRH1A	0.14888
EBNA1	TYRO3	0.075866
EBNA1	RUFY1	0.15576
EBNA1	PEX2	0.10784
EBNA1	SLC18A2	0.011003
EBNA1	TNFRSF10A	0.12465
EBNA1	TRPS1	0.022798
EBNA1	LRRC26	0.16767
EBNA1	SIVA1	0.093534
EBNA1	NLGN3	0.082325
EBNA1	GCC1	0.1505
EBNA1	AURKAIP1	0.13739
EBNA1	CYTIP	0.2066
EBNA1	USP14	0.11438
EBNA1	PCDHGA9	0.038413
EBNA1	ELOVL3	0.13719
EBNA1	EVI5	0.1115
EBNA1	RASA1	0.032085
EBNA1	PALB2	0.14775
EBNA1	CAND2	0.13917
EBNA1	FABP3	0.18778
EBNA1	LRCOL1	0.016703
EBNA1	CDHR5	0.023641
EBNA1	AHI1	0.062119
EBNA1	MRPS27	0.1596
EBNA1	ZBTB16	0.044681
EBNA1	SAT1	0.18389
EBNA1	DCTN2	0.10619
EBNA1	TBC1D8B	0.018813
EBNA1	TUBGCP3	0.043212
EBNA1	AGO1	0.12461
EBNA1	CD53	0.18483
EBNA1	USP25	0.13214
EBNA1	SH3GL2	0.10756
EBNA1	TULP1	0.12295
EBNA1	NXF5	0.21625
EBNA1	SNX1	0.12912
EBNA1	CD151	0.16344
EBNA1	YTHDC1	0.17872
EBNA1	KRT16	0.13082
EBNA1	GUCA1C	0.055943
EBNA1	DOCK1	0.11713
EBNA1	CASP10	0.15416
EBNA1	GEMIN7	0.14629

EBNA1	TBC1D7	0.2018
EBNA1	CDH7	0.03561
EBNA1	SPI1	0.013375
EBNA1	PPP1R1A	0.19704
EBNA1	SEC24C	0.048972
EBNA1	PRKCSH	0.083277
EBNA1	ZCCHC11	0.11301
EBNA1	TGFBR3	0.036611
EBNA1	AMY1A	0.10281
EBNA1	GRAP2	0.13711
EBNA1	EXOC7	0.16234
EBNA1	UPF1	0.094198
EBNA1	STX16	0.099604
EBNA1	TXNIP	0.082752
EBNA1	ANKRD28	0.1844
EBNA1	DRD4	0.05006
EBNA1	FGF1	0.06855
EBNA1	ITPR3	0.024259
EBNA1	GAGE2D	0.21561
EBNA1	NEK11	0.063676
EBNA1	ARL13B	0.17247
EBNA1	ADAMTS10	0.084274
EBNA1	YLPM1	0.14793
EBNA1	BRAT1	0.1844
EBNA1	SIGIRR	0.15931
EBNA1	ADRA2C	0.093216
EBNA1	PCDHGA2	0.038413
EBNA1	RDX	0.13848
EBNA1	TYR	0.1337
EBNA1	POU3F2	0.027492
EBNA1	ITGB4	0.0917
EBNA1	KAL1	0.13529
EBNA1	ZXDC	0.019976
EBNA1	DDIT3	0.024784
EBNA1	GTF3C6	0.17796
EBNA1	WIBG	0.23686
EBNA1	ARHGAP22	0.045675
EBNA1	PCDH1	0.032426
EBNA1	SLC40A1	0.11051
EBNA1	FBXW5	0.062993
EBNA1	CFLAR	0.13504
EBNA1	EIF4EBP1	0.10174
EBNA1	TAX1BP1	0.14623
EBNA1	NOTCH2	0.089717
EBNA1	LOXL3	0.12073
EBNA1	ERLIN2	0.16
EBNA1	CDH6	0.015952
EBNA1	FZR1	0.11867
EBNA1	ROR1	0.14733
EBNA1	MPTX1	0.010549
EBNA1	AMER2	0.21447
EBNA1	MITD1	0.14514
EBNA1	SLC4A1AP	0.14201
EBNA1	SIAH2	0.070267
EBNA1	EXOSC3	0.13893
EBNA1	STIM1	0.22735
EBNA1	COG4	0.14103
EBNA1	S100B	0.22364
EBNA1	TRAF3IP1	0.20909
EBNA1	ZAK	0.037617
EBNA1	FZD6	0.064884
EBNA1	KAT6B	0.054942
EBNA1	HMG20B	0.04495
EBNA1	ESRRA	0.02221
EBNA1	SKP1	0.14338
EBNA1	VAMP1	0.19909
EBNA1	BIRC2	0.051938
EBNA1	ZNF212	0.088082

EBNA1	TNNC1	0.34001
EBNA1	DDB1	0.098576
EBNA1	RBMX	0.10769
EBNA1	ZNF24	0.045539
EBNA1	CHD3	0.047351
EBNA1	ADCYAP1R1	0.046868
EBNA1	CRYGC	0.16392
EBNA1	EFCAB4A	0.041252
EBNA1	ERBB2	0.020633
EBNA1	STEAP3	0.12407
EBNA1	TXLNA	0.18278
EBNA1	POLRMT	0.15767
EBNA1	BCAS2	0.1652
EBNA1	TMED2	0.15122
EBNA1	RAB3IL1	0.17901
EBNA1	CCNC	0.039085
EBNA1	C1orf94	0.22232
EBNA1	CASP6	0.061231
EBNA1	VCL	0.076113
EBNA1	PCDHB13	0.035257
EBNA1	CETN2	0.23997
EBNA1	ANXA6	0.38166
EBNA1	PCDHGC4	0.038413
EBNA1	LCP1	0.025246
EBNA1	DIXDC1	0.083702
EBNA1	SIRPG	0.14703
EBNA1	PA2G4	0.037709
EBNA1	FBXO44	0.21524
EBNA1	ANXA2P2	0.05915
EBNA1	CUL4B	0.15848
EBNA1	KPNB1	0.09357
EBNA1	FEZ1	0.13938
EBNA1	CBX4	0.083057
EBNA1	FKBP9	0.052128
EBNA1	NDC80	0.10965
EBNA1	ATP10D	0.10001
EBNA1	SMG5	0.13974
EBNA1	STK38L	0.022735
EBNA1	ACTN2	0.1324
EBNA1	COL3A1	0.089922
EBNA1	GGA1	0.15773
EBNA1	LRCH4	0.20593
EBNA1	EFNA1	0.096003
EBNA1	CNOT7	0.024096
EBNA1	IFIT3	0.16129
EBNA1	PRSS2	0.32047
EBNA1	STAT2	0.14372
EBNA1	PRDM5	0.076576
EBNA1	REEP1	0.165
EBNA1	PIFO	0.11028
EBNA1	METTL9	0.22232
EBNA1	CD59	0.15095
EBNA1	NCKAP1L	0.059966
EBNA1	LIMS1	0.13059
EBNA1	ZNF219	0.012777
EBNA1	ACTG1	0.049091
EBNA1	NCKAP1	0.13025
EBNA1	EIF4A2	0.17553
EBNA1	PDE4DIP	0.15569
EBNA1	RAB27A	0.059434
EBNA1	SESTD1	0.15562
EBNA1	FKBP4	0.065752
EBNA1	KANK2	0.14961
EBNA1	RPL5	0.11211
EBNA1	TECPR2	0.22232
EBNA1	CDCA5	0.13583
EBNA1	HAX1	0.17032
EBNA1	STAU2	0.1894

EBNA1	NOS3	0.019212
EBNA1	PITPNM3	0.36134
EBNA1	VIL1	0.16711
EBNA1	TNIP2	0.03565
EBNA1	ADCY6	0.011313
EBNA1	S100A7L2	0.02299
EBNA1	RNF126	0.17587
EBNA1	STX11	0.19375
EBNA1	FSTL3	0.077705
EBNA1	ELK4	0.053042
EBNA1	WEE1	0.044782
EBNA1	PSMA6	0.057661
EBNA1	PCDHA3	0.036102
EBNA1	MUC5B	0.17841
EBNA1	CIB3	0.044917
EBNA1	CD44	0.082341
EBNA1	PPID	0.21422
EBNA1	SMNDC1	0.2134
EBNA1	TFF2	0.15412
EBNA1	NEDD9	0.11693
EBNA1	PTPN9	0.049768
EBNA1	ATF7	0.012967
EBNA1	GUCY2C	0.021239
EBNA1	HPCA	0.042436
EBNA1	SMC5	0.12504
EBNA1	ZRANB1	0.08792
EBNA1	DNASE1L2	0.041082
EBNA1	PTPRO	0.027871
EBNA1	KCNJ4	0.22121
EBNA1	ANK3	0.076023
EBNA1	SYPL1	0.075803
EBNA1	DDX6	0.14967
EBNA1	APOBEC1	0.081185
EBNA1	ERLIN1	0.17533
EBNA1	NLRP3	0.091108
EBNA1	ZNF473	0.10407
EBNA1	RAD9B	0.18148
EBNA1	AHSA1	0.16817
EBNA1	GTF2A2	0.088254
EBNA1	VSIG4	0.18989
EBNA1	TOP1	0.068165
EBNA1	DNMT3B	0.0811
EBNA1	RAC3	0.059222
EBNA1	IGF2R	0.12579
EBNA1	SORCS1	0.18715
EBNA1	PYURF	0.18043
EBNA1	HRG	0.052296
EBNA1	ORC3	0.11304
EBNA1	SCYL3	0.17393
EBNA1	CCL18	0.1703
EBNA1	ARPC3	0.13369
EBNA1	NUAK1	0.086519
EBNA1	TXN	0.028217
EBNA1	PIK3R4	0.083544
EBNA1	XRCC1	0.13844
EBNA1	CETN1	0.32368
EBNA1	UBQLN4	0.14672
EBNA1	KCNJ9	0.2023
EBNA1	LPP	0.1187
EBNA1	ZBTB25	0.026195
EBNA1	BCOR	0.083917
EBNA1	TTBK2	0.10734
EBNA1	MPHOSPH10	0.10536
EBNA1	MLLT10	0.021003
EBNA1	PFKFB2	0.056784
EBNA1	CUL3	0.074847
EBNA1	NTNG1	0.17766
EBNA1	MC1R	0.087018

EBNA1	COG7	0.12895
EBNA1	PROCR	0.15936
EBNA1	ERC2	0.14739
EBNA1	ATP1B2	0.14087
EBNA1	MAP2K2	0.022026
EBNA1	HLA-A	0.086093
EBNA1	GNB2	0.10533
EBNA1	HSPB9	0.17904
EBNA1	PSMC6	0.066663
EBNA1	NDFIP1	0.07129
EBNA1	STK10	0.10672
EBNA1	ANXA13	0.041204
EBNA1	HTRA2	0.092383
EBNA1	NUP98	0.091321
EBNA1	ANK2	0.030089
EBNA1	NFKBIA	0.028893
EBNA1	SOD3	0.086936
EBNA1	SEPT6	0.098058
EBNA1	MYO5A	0.039987
EBNA1	CIC	0.14752
EBNA1	COMMD8	0.22232
EBNA1	IGFBP2	0.15184
EBNA1	DUSP2	0.016778
EBNA1	CCR5	0.069591
EBNA1	RPS13	0.11441
EBNA1	TAF8	0.10135
EBNA1	CRHR2	0.11249
EBNA1	IL1RAPL1	0.10205
EBNA1	HPN	0.097052
EBNA1	VLDLR	0.1328
EBNA1	INPPL1	0.059742
EBNA1	TRIP13	0.088233
EBNA1	CDH1	0.063631
EBNA1	HTT	0.029287
EBNA1	DNAJB4	0.16116
EBNA1	ADAMTS13	0.15919
EBNA1	BCAP31	0.049851
EBNA1	DIAPH1	0.052617
EBNA1	TEAD1	0.050191
EBNA1	BCL6	0.015462
EBNA1	MAP1LC3B	0.2147
EBNA1	USF2	0.039198
EBNA1	UBA5	0.060372
EBNA1	TTN	0.073913
EBNA1	ZDHHC17	0.17217
EBNA1	MICU3	0.042436
EBNA1	CAPNS2	0.04126
EBNA1	ABT1	0.14554
EBNA1	NDRG2	0.09106
EBNA1	TWIST2	0.020245
EBNA1	FSTL4	0.043666
EBNA1	ACTR3	0.032832
EBNA1	DCPS	0.076689
EBNA1	TUT1	0.13115
EBNA1	PSMC5	0.05028
EBNA1	NCALD	0.040705
EBNA1	MUS81	0.10684
EBNA1	PRDM16	0.064946
EBNA1	EGF	0.11534
EBNA1	RAPGEF2	0.026759
EBNA1	TFAM	0.04853
EBNA1	TJP1	0.12867
EBNA1	CBX1	0.085772
EBNA1	HOMER3	0.14696
EBNA1	AJUBA	0.036027
EBNA1	MIEF1	0.13322
EBNA1	TIRAP	0.056749
EBNA1	PDK3	0.14708

EBNA1	FYN	0.042052
EBNA1	PRKAB2	0.025819
EBNA1	USP26	0.15461
EBNA1	PPP1R16B	0.15977
EBNA1	HIVEP1	0.098533
EBNA1	ELOVL5	0.12669
EBNA1	LTBP4	0.29028
EBNA1	BLOC1S6	0.083407
EBNA1	MOAP1	0.20779
EBNA1	RANBP2	0.072941
EBNA1	PRMT2	0.13376
EBNA1	RPS19	0.12459
EBNA1	SMARCAD1	0.08395
EBNA1	CDKN1B	0.051414
EBNA1	GNB2L1	0.019703
EBNA1	NUP43	0.11579
EBNA1	PLSCR4	0.3504
EBNA1	LMNA	0.079581
EBNA1	CENPF	0.048319
EBNA1	MPP7	0.17486
EBNA1	VAMP2	0.13035
EBNA1	RBM8A	0.16246
EBNA1	ZFAND6	0.11529
EBNA1	SHANK1	0.092249
EBNA1	DCP2	0.13742
EBNA1	CCNK	0.09622
EBNA1	FBXL2	0.19635
EBNA1	SCOC	0.13242
EBNA1	LAMB3	0.074374
EBNA1	MBD4	0.086373
EBNA1	RXRB	0.035598
EBNA1	FBXO25	0.16828
EBNA1	KAT8	0.07966
EBNA1	NID1	0.043639
EBNA1	MEPE	0.20445
EBNA1	GOLGB1	0.15839
EBNA1	HBA1	0.15227
EBNA1	NXF1	0.1718
EBNA1	GMIP	0.13598
EBNA1	C1R	0.091496
EBNA1	TUBG1	0.085228
EBNA1	SLC6A2	0.2096
EBNA1	AKR1B10	0.1974
EBNA1	NOP2	0.2861
EBNA1	DNAJC1	0.091608
EBNA1	FCGBP	0.2091
EBNA1	ZC3H11A	0.1706
EBNA1	TSHZ3	0.010185
EBNA1	SERPINC1	0.13446
EBNA1	FN1	0.090943
EBNA1	SRSF2	0.17426
EBNA1	RHOT1	0.1913
EBNA1	RPS6KB1	0.010498
EBNA1	DDX39A	0.12448
EBNA1	SAP18	0.12719
EBNA1	SAFB	0.13298
EBNA1	ASB13	0.20676
EBNA1	TEAD4	0.030946
EBNA1	SNX9	0.20722
EBNA1	NLGN4X	0.079904
EBNA1	COL4A3	0.033828
EBNA1	CITED2	0.022517
EBNA1	CSRP3	0.069919
EBNA1	VPS53	0.1835
EBNA1	TTI1	0.22232
EBNA1	NCBP2	0.1011
EBNA1	ZC3H15	0.15359
EBNA1	TNPO1	0.1243

EBNA1	LSM1	0.14654
EBNA1	COPS4	0.16981
EBNA1	ABI3	0.1813
EBNA1	UBXN4	0.14584
EBNA1	NLRP12	0.11372
EBNA1	DNAJC10	0.065296
EBNA1	ZCCHC8	0.13413
EBNA1	TRAF3	0.023168
EBNA1	RYR2	0.09696
EBNA1	DLAT	0.17214
EBNA1	ACTR5	0.13423
EBNA1	AXL	0.053187
EBNA1	RAET1E	0.17903
EBNA1	SMC6	0.13703
EBNA1	PARP9	0.25533
EBNA1	OPRM1	0.061441
EBNA1	GTPBP4	0.066362
EBNA1	CRLF1	0.18711
EBNA1	PRF1	0.23714
EBNA1	CACNA1I	0.1561
EBNA1	TFF1	0.15743
EBNA1	PCDHB3	0.033158
EBNA1	RPS6KA4	0.019321
EBNA1	PPP1R13L	0.077221
EBNA1	HSD17B14	0.15209
EBNA1	EXOC4	0.060722
EBNA1	SEPT12	0.11813
EBNA1	CUL7	0.12974
EBNA1	CDC16	0.11151
EBNA1	IL6ST	0.11984
EBNA1	CDC20	0.080976
EBNA1	STRN	0.14624
EBNA1	MYO1E	0.15032
EBNA1	SPRY4	0.17195
EBNA1	ZNF366	0.10266
EBNA1	DDX20	0.072441
EBNA1	TEN1	0.14692
EBNA1	UBE2V1	0.057354
EBNA1	OCA2	0.11004
EBNA1	IGF1R	0.041976
EBNA1	DGKZ	0.1092
EBNA1	SNRPA1	0.15353
EBNA1	LAIR1	0.20279
EBNA1	MICU2	0.043725
EBNA1	F2RL2	0.12096
EBNA1	VEGFB	0.085484
EBNA1	NHLRC1	0.10002
EBNA1	SOCS2	0.11537
EBNA1	ZNF423	0.089138
EBNA1	MZT1	0.17242
EBNA1	IGF2BP1	0.067058
EBNA1	RNF144B	0.11109
EBNA1	PPP1R12C	0.21561
EBNA1	MCFD2	0.036078
EBNA1	MYL12B	0.011148
EBNA1	PI4KA	0.19756
EBNA1	SLC25A24	0.042153
EBNA1	MAP3K4	0.069657
EBNA1	GPR143	0.094558
EBNA1	CBS	0.020509
EBNA1	TIMM9	0.11003
EBNA1	PKD2L2	0.05071
EBNA1	VDAC2	0.079827
EBNA1	IL13	0.091585
EBNA1	USP22	0.070201
EBNA1	SHARPIN	0.087091
EBNA1	OLFM1	0.18702
EBNA1	SART3	0.17139

EBNA1	PRPF40A	0.14278
EBNA1	LSM4	0.14636
EBNA1	WNK3	0.098281
EBNA1	FBXO11	0.0829
EBNA1	SNRNP70	0.16668
EBNA1	LDLRAP1	0.076327
EBNA1	EPHB4	0.10918
EBNA1	PCSK9	0.054049
EBNA1	TRA2B	0.2153
EBNA1	DGKI	0.15834
EBNA1	HSPB7	0.16357
EBNA1	ELANE	0.1165
EBNA1	RING1	0.068031
EBNA1	ZNF496	0.027815
EBNA1	NELFA	0.13179
EBNA1	ACVR1B	0.026451
EBNA1	TCEB2	0.11758
EBNA1	MYPN	0.15469
EBNA1	BRD3	0.13547
EBNA1	SETD2	0.1251
EBNA1	ZRANB2	0.078793
EBNA1	TRIM32	0.083098
EBNA1	PLD2	0.019784
EBNA1	YWHAH	0.090963
EBNA1	PARP4	0.14607
EBNA1	HNRNPM	0.15755
EBNA1	KRT6A	0.13442
EBNA1	KLRF2	0.19652
EBNA1	PCDHA6	0.035121
EBNA1	SERPINB8	0.17992
EBNA1	MED28	0.12855
EBNA1	PLEC	0.13193
EBNA1	FADD	0.073245
EBNA1	TULP3	0.13259
EBNA1	ARRDC3	0.20312
EBNA1	RPS6KA5	0.017517
EBNA1	SERBP1	0.22042
EBNA1	TGFBRAP1	0.154
EBNA1	FHOD1	0.13549
EBNA1	XRCC4	0.076839
EBNA1	ZFC3H1	0.181
EBNA1	TP53BP2	0.12183
EBNA1	RNF187	0.09261
EBNA1	E2F3	0.034629
EBNA1	ZNF711	0.098723
EBNA1	PTPN2	0.043045
EBNA1	STIP1	0.14049
EBNA1	MALT1	0.056114
EBNA1	SUPT5H	0.07392
EBNA1	ELMO2	0.050507
EBNA1	ATP6V0A2	0.13356
EBNA1	SNCA	0.023149
EBNA1	FBXO27	0.20872
EBNA1	EMR3	0.013198
EBNA1	CARD10	0.17777
EBNA1	UBE3C	0.10879
EBNA1	RANBP6	0.17774
EBNA1	MCIDAS	0.10087
EBNA1	S100A8	0.15337
EBNA1	THAP11	0.11338
EBNA1	IL2RG	0.13969
EBNA1	NID2	0.40559
EBNA1	SYP	0.057857
EBNA1	F2R	0.023532
EBNA1	CCNB1	0.032786
EBNA1	CADM1	0.068286
EBNA1	DIS3L	0.18319
EBNA1	NELL1	0.27408

EBNA1	DIRAS3	0.12269
EBNA1	FAM84B	0.2091
EBNA1	SLX1A	0.13934
EBNA1	DEDD	0.11565
EBNA1	ZHX1	0.024733
EBNA1	ANXA1	0.047609
EBNA1	FAF2	0.18968
EBNA1	CDKN2B	0.051182
EBNA1	PFDN5	0.11983
EBNA1	ZMYND11	0.080477
EBNA1	CLDN7	0.066608
EBNA1	KRAS	0.05753
EBNA1	PTPRE	0.034757
EBNA1	C3	0.086654
EBNA1	PCDHB16	0.035257
EBNA1	PTPRK	0.018491
EBNA1	SNTN	0.042492
EBNA1	SIRT3	0.11592
EBNA1	ADA	0.03318
EBNA1	DNER	0.27613
EBNA1	FBXO17	0.20872
EBNA1	PRKAR1A	0.089059
EBNA1	EIF6	0.22897
EBNA1	CD72	0.12366
EBNA1	VSNL1	0.044917
EBNA1	RUVBL2	0.048799
EBNA1	FBXL14	0.20491
EBNA1	PCDHGA6	0.038413
EBNA1	CFL1	0.082868
EBNA1	MBD1	0.03898
EBNA1	MKL2	0.12613
EBNA1	SCNN1G	0.14379
EBNA1	POU2F1	0.039509
EBNA1	SLC25A10	0.095275
EBNA1	ATP8A1	0.13979
EBNA1	HMG20A	0.050926
EBNA1	TLX1	0.037187
EBNA1	IL17RA	0.1395
EBNA1	UBE2V2	0.10798
EBNA1	EFCAB2	0.044917
EBNA1	DSCAM	0.13632
EBNA1	NUCB1	0.044991
EBNA1	SAFB2	0.17708
EBNA1	CYTH1	0.10845
EBNA1	HEYL	0.026595
EBNA1	ELOVL6	0.14002
EBNA1	PPARGC1A	0.046077
EBNA1	NRXN1	0.12172
EBNA1	PEF1	0.42306
EBNA1	OFD1	0.1062
EBNA1	CD22	0.17865
EBNA1	RBM15B	0.14856
EBNA1	SHOX	0.046855
EBNA1	NOXA1	0.1571
EBNA1	DUX4L9	0.036367
EBNA1	ZWINT	0.11627
EBNA1	NAPG	0.16666
EBNA1	ZFYVE26	0.17329
EBNA1	RBBP8	0.093274
EBNA1	MAX	0.051676
EBNA1	ANXA3	0.031571
EBNA1	FBXL8	0.22232
EBNA1	SNRPE	0.12503
EBNA1	CGN	0.18838
EBNA1	XIAP	0.10862
EBNA1	GCHFR	0.11409
EBNA1	PLA2G16	0.081763
EBNA1	EZR	0.10488

EBNA1	COL16A1	0.16611
EBNA1	PSMA3	0.046542
EBNA1	RAD17	0.11107
EBNA1	FBL	0.22926
EBNA1	TTYH1	0.048339
EBNA1	CNNM3	0.19499
EBNA1	RASGRP2	0.029322
EBNA1	TSSK6	0.069659
EBNA1	MEGF8	0.31258
EBNA1	PDGFB	0.016832
EBNA1	PCDHGB5	0.038413
EBNA1	CHCHD6	0.2176
EBNA1	CDH13	0.016889
EBNA1	RNF168	0.067964
EBNA1	ATXN1	0.11173
EBNA1	FKBP9L	0.034342
EBNA1	GAP43	0.14394
EBNA1	TTYH2	0.17881
EBNA1	BAK1	0.028291
EBNA1	BEND7	0.22232
EBNA1	CHP1	0.13973
EBNA1	PRICKLE3	0.16687
EBNA1	PAF1	0.10588
EBNA1	REEP5	0.1967
EBNA1	ANTXR2	0.14356
EBNA1	MAP4K2	0.052933
EBNA1	NFATC1	0.027885
EBNA1	CYFIP2	0.16664
EBNA1	CSTF2	0.17403
EBNA1	TNK2	0.083812
EBNA1	CAMTA2	0.076904
EBNA1	MAPK3	0.021222
EBNA1	ERAP1	0.08614
EBNA1	UBE2E1	0.071239
EBNA1	APTX	0.046157
EBNA1	VPS52	0.18919
EBNA1	MYL12A	0.046094
EBNA1	CACNG2	0.11359
EBNA1	DAP3	0.07254
EBNA1	ZGPAT	0.026006
EBNA1	RASSF3	0.16283
EBNA1	CFTR	0.093345
EBNA1	RAB3IP	0.13555
EBNA1	SPG7	0.044636
EBNA1	GNAQ	0.024063
EBNA1	CTSH	0.062521
EBNA1	XPO5	0.1468
EBNA1	ABCD2	0.14212
EBNA1	CD86	0.08057
EBNA1	EGFL6	0.046655
EBNA1	KMT2E	0.10656
EBNA1	IMP4	0.12815
EBNA1	PRPF31	0.16525
EBNA1	SYMPK	0.14614
EBNA1	CUL5	0.086732
EBNA1	CNST	0.20993
EBNA1	COL6A2	0.13421
EBNA1	TRAPPC10	0.18735
EBNA1	LIPE	0.054295
EBNA1	HAP1	0.13961
EBNA1	ATP6V1C1	0.15259
EBNA1	PSMB7	0.049299
EBNA1	AHCYL1	0.15272
EBNA1	TGM2	0.14765
EBNA1	RPLP0	0.13586
EBNA1	NR3C1	0.020435
EBNA1	BAG1	0.097756
EBNA1	MAPK10	0.052049

EBNA1	FASN	0.13297
EBNA1	DLL4	0.020922
EBNA1	SEPT7	0.015057
EBNA1	MPZL1	0.082215
EBNA1	BRCA2	0.03514
EBNA1	LCMT1	0.26186
EBNA1	COL1A1	0.034333
EBNA1	ELOVL7	0.14151
EBNA1	RPS3A	0.13972
EBNA1	GPR98	0.19569
EBNA1	PDS5B	0.13211
EBNA1	CRY1	0.034361
EBNA1	NUMBL	0.21105
EBNA1	UBA3	0.038581
EBNA1	ACAA2	0.17605
EBNA1	DLG4	0.013785
EBNA1	LSM6	0.13777
EBNA1	MBIP	0.16825
EBNA1	MICU1	0.34655
EBNA1	CALU	0.34473
EBNA1	EIF2S1	0.16134
EBNA1	TBRG1	0.14737
EBNA1	ELF2	0.03536
EBNA1	ATP1B1	0.13051
EBNA1	CEP63	0.16991
EBNA1	ZIC3	0.013063
EBNA1	RIPK1	0.018815
EBNA1	AP2B1	0.093882
EBNA1	KIRREL3	0.18926
EBNA1	GPSM2	0.10262
EBNA1	PSMB5	0.04753
EBNA1	ATXN2	0.099239
EBNA1	LILRB4	0.13644
EBNA1	CD8A	0.066447
EBNA1	SSX2	0.15249
EBNA1	LENG8	0.22232
EBNA1	PRKRA	0.1591
EBNA1	KLK8	0.18509
EBNA1	PTPN13	0.024023
EBNA1	PPP3CA	0.12816
EBNA1	STRADB	0.021113
EBNA1	MTUS2	0.19562
EBNA1	ANXA8L2	0.023566
EBNA1	FBLN2	0.053468
EBNA1	BCL2A1	0.19667
EBNA1	HAND2	0.031358
EBNA1	PMEL	0.13904
EBNA1	TACR1	0.043699
EBNA1	MYOZ2	0.17814
EBNA1	VCP	0.053571
EBNA1	USP28	0.13243
EBNA1	PPP1CA	0.037458
EBNA1	ITK	0.039361
EBNA1	HOXC13	0.040616
EBNA1	GADD45G	0.14162
EBNA1	BTG1	0.10409
EBNA1	MCTP1	0.041209
EBNA1	PRKX	0.063249
EBNA1	MOCS3	0.092139
EBNA1	CCNG1	0.13196
EBNA1	VSTM2L	0.22232
EBNA1	RFX6	0.096495
EBNA1	ZFYVE28	0.13832
EBNA1	FCGR2C	0.19408
EBNA1	AKAP6	0.073428
EBNA1	BZRAP1	0.19449
EBNA1	EIF4B	0.21371
EBNA1	ANXA10	0.043662

EBNA1	PCDHGB3	0.038413
EBNA1	GLS2	0.12828
EBNA1	RNF40	0.092595
EBNA1	SOX5	0.022096
EBNA1	PVR	0.074115
EBNA1	NCOR2	0.092538
EBNA1	LRP2	0.1441
EBNA1	VMP1	0.1267
EBNA1	CAPN2	0.21173
EBNA1	PAFAH1B3	0.11421
EBNA1	KRT8	0.13243
EBNA1	SLC23A1	0.13953
EBNA1	OCLN	0.035146
EBNA1	SIRPB1	0.18459
EBNA1	CPSF7	0.19048
EBNA1	ABCG5	0.093247
EBNA1	UNC119	0.20566
EBNA1	ABL1	0.022966
EBNA1	DGCR8	0.16781
EBNA1	CLIC1	0.10773
EBNA1	MKRN3	0.096627
EBNA1	TTC1	0.19046
EBNA1	SUPT4H1	0.018089
EBNA1	SREBF2	0.022047
EBNA1	HNRNPC	0.1474
EBNA1	FAM188B	0.01989
EBNA1	TRIM37	0.083589
EBNA1	GRN	0.12982
EBNA1	PABPC4	0.14583
EBNA1	SLC22A11	0.17209
EBNA1	VPS33A	0.12839
EBNA1	TRIM5	0.073799
EBNA1	ANTXR1	0.12836
EBNA1	TNFRSF11A	0.072261
EBNA1	NPLOC4	0.11232
EBNA1	NAA10	0.18061
EBNA1	RGS2	0.10248
EBNA1	CTSL	0.11256
EBNA1	MON1B	0.22232
EBNA1	TIFA	0.19142
EBNA1	NUCB2	0.28019
EBNA1	TF	0.12302
EBNA1	CBR4	0.1977
EBNA1	CBX6	0.14507
EBNA1	MSX2	0.014033
EBNA1	PKDREJ	0.038361
EBNA1	PPP1R13B	0.15767
EBNA1	DCC	0.10334
EBNA1	LRSAM1	0.10359
EBNA1	SOX1	0.047164
EBNA1	HELZ2	0.087957
EBNA1	EED	0.11544
EBNA1	ASGR1	0.16333
EBNA1	NPC2	0.10809
EBNA1	HOXA9	0.045426
EBNA1	MAGEA11	0.1844
EBNA1	SH3RF2	0.13887
EBNA1	RAP1GAP	0.10082
EBNA1	USH1C	0.096441
EBNA1	PCDH19	0.038413
EBNA1	RNPS1	0.1641
EBNA1	SH3GLB1	0.074718
EBNA1	CDON	0.087214
EBNA1	BCL2L14	0.089226
EBNA1	PINX1	0.073933
EBNA1	PEX11A	0.15707
EBNA1	RIPK3	0.034201
EBNA1	S100A6	0.26568

EBNA1	RP1L1	0.19714
EBNA1	EPHB1	0.067748
EBNA1	AOC1	0.036456
EBNA1	STK16	0.052811
EBNA1	FAT2	0.02029
EBNA1	FBXW2	0.19598
EBNA1	PLCG2	0.02823
EBNA1	SMARCD3	0.034523
EBNA1	RNF20	0.061792
EBNA1	ZFP36	0.032627
EBNA1	PPP2R1A	0.037931
EBNA1	IPO13	0.1626
EBNA1	AFG3L2	0.042663
EBNA1	EPB42	0.11702
EBNA1	WDR6	0.17236
EBNA1	DDC	0.09305
EBNA1	SPRED2	0.12084
EBNA1	TLR4	0.011258
EBNA1	PKHD1	0.095782
EBNA1	RNF19B	0.13654
EBNA1	IKBKAP	0.047761
EBNA1	RABGEF1	0.12001
EBNA1	LRP8	0.24232
EBNA1	ZEB1	0.013561
EBNA1	NOX5	0.039067
EBNA1	SYNPO	0.13521
EBNA1	IGFLR1	0.20279
EBNA1	CASP2	0.082265
EBNA1	CT55	0.028489
EBNA1	RFC5	0.057338
EBNA1	PPP6R3	0.17007
EBNA1	KRT14	0.078713
EBNA1	CBFA2T2	0.021123
EBNA1	GABRG2	0.10492
EBNA1	NUAK2	0.069257
EBNA1	NXT1	0.14106
EBNA1	S100P	0.36335
EBNA1	SNAI1	0.040451
EBNA1	CARD8	0.1694
EBNA1	PLK3	0.042554
EBNA1	SEMA4D	0.045705
EBNA1	SMURF1	0.06754
EBNA1	SIX5	0.044478
EBNA1	TMEM67	0.139
EBNA1	WASL	0.046886
EBNA1	LRP1B	0.038041
EBNA1	DCAF7	0.17027
EBNA1	SGTA	0.19077
EBNA1	XPO6	0.15397
EBNA1	TRAK1	0.11982
EBNA1	PCDHGA5	0.038413
EBNA1	RNF103	0.11049
EBNA1	CLASP1	0.061837
EBNA1	DMD	0.076186
EBNA1	LCA5	0.17082
EBNA1	INTS4	0.17861
EBNA1	DYNLRB1	0.14094
EBNA1	GYPC	0.19057
EBNA1	SEPT1	0.16135
EBNA1	EGLN2	0.11922
EBNA1	PRDX6	0.20011
EBNA1	WT1	0.010978
EBNA1	YWHAZ	0.066304
EBNA1	CHAF1A	0.16292
EBNA1	BTF3	0.16123
EBNA1	KIAA0319L	0.20278
EBNA1	SLC22A1	0.12802
EBNA1	DAZ1	0.19758

EBNA1	SEPT9	0.10629
EBNA1	PPP2R5C	0.10784
EBNA1	TAF9	0.063063
EBNA1	PTCRA	0.20275
EBNA1	SETD1B	0.19756
EBNA1	RIMS2	0.14308
EBNA1	TP53INP1	0.14133
EBNA1	F7	0.020695
EBNA1	ELF4	0.038316
EBNA1	ECH1	0.1078
EBNA1	CPS1	0.036016
EBNA1	C4BPA	0.18764
EBNA1	TAPBP	0.045141
EBNA1	RAPGEF1	0.13418
EBNA1	FEM1B	0.13959
EBNA1	ATXN7L3	0.1149
EBNA1	FNBP1	0.21059
EBNA1	KMT2B	0.062753
EBNA1	MCM10	0.12309
EBNA1	KCNB1	0.13363
EBNA1	CASP9	0.12733
EBNA1	NR4A2	0.02114
EBNA1	VRK1	0.078285
EBNA1	LTN1	0.1381
EBNA1	MTOR	0.022542
EBNA1	PLAU	0.1238
EBNA1	DZIP1	0.11323
EBNA1	MADD	0.14829
EBNA1	NUP160	0.12787
EBNA1	NUP50	0.13723
EBNA1	TRIO	0.01674
EBNA1	RAB11FIP3	0.20197
EBNA1	MOB1B	0.1133
EBNA1	EFCAB4B	0.33972
EBNA1	APP	0.012752
EBNA1	CDHR1	0.018118
EBNA1	HPX	0.10508
EBNA1	UCHL1	0.057889
EBNA1	GAB2	0.046488
EBNA1	ALKBH8	0.20994
EBNA1	PARD6G	0.16821
EBNA1	CAMK1	0.030834
EBNA1	SYBU	0.20366
EBNA1	PCDHB8	0.038413
EBNA1	NOC4L	0.15947
EBNA1	YOD1	0.12259
EBNA1	GABPA	0.023563
EBNA1	ARL6IP1	0.23101
EBNA1	TMPRSS6	0.18661
EBNA1	BLOC1S4	0.16813
EBNA1	THAP3	0.17089
EBNA1	S100A7A	0.022404
EBNA1	ZNF256	0.022999
EBNA1	MUC17	0.13869
EBNA1	AP2A1	0.091141
EBNA1	TDGF1	0.04552
EBNA1	ZYX	0.10094
EBNA1	EFCAB3	0.044917
EBNA1	ANKZF1	0.17058
EBNA1	PFDN2	0.14377
EBNA1	PRRG2	0.039928
EBNA1	STK11	0.019358
EBNA1	DLGAP2	0.15723
EBNA1	SMOC2	0.025737
EBNA1	STXBP3	0.099911
EBNA1	TJP3	0.1762
EBNA1	SALL1	0.01778
EBNA1	SERPING1	0.13367

16389	EBNA1	HIP1R	0.12281
16390	EBNA1	THOC1	0.14608
16391	EBNA1	YTHDC2	0.15573
16392	EBNA1	RAD51B	0.12658
16393	EBNA1	HNRNPA2B1	0.15154
16394	EBNA1	CCT2	0.099687
16395	EBNA1	FOSL1	0.024827
16396	EBNA1	SETD6	0.21853
16397	EBNA1	MTPAP	0.12601
16398	EBNA1	CASR	0.12901
16399	EBNA1	YWHAE	0.11688
16400	EBNA1	TPO	0.079267
16401	EBNA1	NDUFA13	0.094946
16402	EBNA1	CHGB	0.20975
16403	EBNA1	DTNA	0.29371
16404	EBNA1	REPS2	0.44674
16405	EBNA1	IMP3	0.15192
16406	EBNA1	OPRK1	0.11164
16407	EBNA1	SH3BP4	0.20469
16408	EBNA1	SASS6	0.17366
16409	EBNA1	PPP3R1	0.066319
16410	EBNA1	RRAGB	0.10065
16411	EBNA1	CCDC50	0.21561
16412	EBNA1	CDH24	0.044226
16413	EBNA1	TRIM44	0.17058
16414	EBNA1	ARFGEF1	0.067714
16415	EBNA1	PPEF2	0.22857
16416	EBNA1	EFEMP2	0.41148
16417	EBNA1	DVL3	0.084398
16418	EBNA1	IFNA2	0.13089
16419	EBNA1	MAP4	0.067578
16420	EBNA1	MYLK	0.065788
16421	EBNA1	ZC3H13	0.1706
16422	EBNA1	ALX1	0.045179
16423	EBNA1	SART1	0.1411
16424	EBNA1	MT-CO1	0.069477
16425	EBNA1	TANK	0.14654
16426	EBNA1	HERC5	0.097219
16427	EBNA1	EIF3L	0.21994
16428	EBNA1	STARD13	0.15044
16429	EBNA1	ATCAY	0.13966
16430	EBNA1	MAP1B	0.016571
16431	EBNA1	HNRNPH1	0.14244
16432	EBNA1	PFN2	0.15377
16433	EBNA1	USP33	0.073455
16434	EBNA1	ATP2A1	0.13224
16435	EBNA1	CCS	0.08879
16436	EBNA1	POGZ	0.10778
16437	EBNA1	SIK2	0.048535
16438	EBNA1	TCEA1	0.067408
16439	EBNA1	TRRAP	0.13091
16440	EBNA1	FZD4	0.042887
16441	EBNA1	EXOSC4	0.10799
16442	EBNA1	CSE1L	0.16357
16443	EBNA1	RNF10	0.074132
16444	EBNA1	SCAMP5	0.13761
16445	EBNA1	HIST3H3	0.072933
16446	EBNA1	CAB39	0.11105
16447	EBNA1	WLS	0.23819
16448	EBNA1	NOC2L	0.10227
16449	EBNA1	ATP6V0A4	0.090516
16450	EBNA1	PPP2R1B	0.21564
16451	EBNA1	ITGA2	0.15267
16452	EBNA1	EIF3D	0.21661
16453	EBNA1	CXorf40A	0.22232
16454	EBNA1	HNRNPD	0.09269
16455	EBNA1	MYL3	0.036953
16456	EBNA1	AGXT	0.1231

EBNA1	BAG6	0.092349
EBNA1	CHD9	0.085226
EBNA1	CSF2RB	0.15185
EBNA1	CD47	0.12832
EBNA1	CHMP3	0.12359
EBNA1	PBK	0.12719
EBNA1	RFC4	0.059689
EBNA1	HOOK2	0.12258
EBNA1	CCP110	0.16367
EBNA1	SMG7	0.13399
EBNA1	CD200	0.16279
EBNA1	LSM2	0.059274
EBNA1	AP3M1	0.16782
EBNA1	DYNC1I1	0.097148
EBNA1	ZNF703	0.074464
EBNA1	NFYB	0.042143
EBNA1	SH3GL3	0.17398
EBNA1	DMBT1	0.087705
EBNA1	MINK1	0.061807
EBNA1	PKN2	0.035934
EBNA1	MECOM	0.017928
EBNA1	LONP1	0.097683
EBNA1	PDCL3	0.19074
EBNA1	JAM3	0.10652
EBNA1	ASB16	0.20676
EBNA1	MLH3	0.097947
EBNA1	ARHGAP17	0.15141
EBNA1	PKD2L1	0.22803
EBNA1	MOB1A	0.18257
EBNA1	ADRB3	0.059001
EBNA1	TCEB1	0.10738
EBNA1	VPS4B	0.086849
EBNA1	FEZ2	0.19123
EBNA1	RNF111	0.065894
EBNA1	DROSHA	0.11572
EBNA1	CD2BP2	0.17395
EBNA1	NINL	0.037212
EBNA1	CD2	0.13803
EBNA1	ACOT8	0.082733
EBNA1	TPPP3	0.045929
EBNA1	SMOC1	0.043706
EBNA1	SPPL2B	0.094988
EBNA1	ATR	0.075431
EBNA1	TRIM3	0.1317
EBNA1	EDC4	0.10101
EBNA1	PAX9	0.13416
EBNA1	ZNF408	0.088082
EBNA1	BTN3A1	0.17345
EBNA1	PLEKHA1	0.053456
EBNA1	DAB2	0.073817
EBNA1	HGS	0.085036
EBNA1	ARHGEF11	0.035141
EBNA1	SLC9A3R1	0.058844
EBNA1	TOPBP1	0.12311
EBNA1	BOLL	0.20298
EBNA1	MYLK2	0.024929
EBNA1	RYBP	0.089703
EBNA1	EHD3	0.24092
EBNA1	DYRK1A	0.033968
EBNA1	TGM1	0.20572
EBNA1	JAK1	0.092024
EBNA1	IGF2	0.079682
EBNA1	HSPA2	0.1418
EBNA1	FAM64A	0.21687
EBNA1	KIAA2018	0.015206
EBNA1	BCR	0.077068
EBNA1	TMEM8B	0.13592
EBNA1	SLC14A2	0.17297

EBNA1	ZZEF1	0.010597
EBNA1	AGER	0.092247
EBNA1	RICTOR	0.13606
EBNA1	MYL10	0.043725
EBNA1	PDGFRA	0.022022
EBNA1	UBE2M	0.097616
EBNA1	NF2	0.054902
EBNA1	MICALL1	0.16545
EBNA1	CSK	0.072619
EBNA1	SMAD2	0.013744
EBNA1	RPS2	0.13555
EBNA1	EXOSC8	0.16584
EBNA1	IL4R	0.12529
EBNA1	NR3C2	0.051358
EBNA1	KRT15	0.15171
EBNA1	NTN1	0.1451
EBNA1	ZXDA	0.020114
EBNA1	MAGEC1	0.22232
EBNA1	POU2F3	0.03054
EBNA1	P4HTM	0.03853
EBNA1	EFCAB11	0.044917
EBNA1	DLGAP5	0.11021
EBNA1	ATRX	0.087707
EBNA1	FKBP2	0.17613
EBNA1	RNF6	0.074313
EBNA1	TAF9B	0.08731
EBNA1	DTL	0.11626
EBNA1	UVRAG	0.15966
EBNA1	COPB1	0.071105
EBNA1	KIF6	0.15112
EBNA1	THBS2	0.38016
EBNA1	OIT3	0.017688
EBNA1	EPS15L1	0.010082
EBNA1	PELI2	0.13836
EBNA1	FAP	0.1318
EBNA1	NAF1	0.16485
EBNA1	MLLT3	0.13482
EBNA1	PRAME	0.11598
EBNA1	CALM1	0.15745
EBNA1	CRYAB	0.065902
EBNA1	SMARCA5	0.041193
EBNA1	ASAP2	0.017714
EBNA1	EIF4E2	0.26162
EBNA1	STAMBPL1	0.16997
EBNA1	ACVR2B	0.024301
EBNA1	DBNL	0.12212
EBNA1	FGF3	0.12856
EBNA1	E2F1	0.012487
EBNA1	STRN4	0.21219
EBNA1	AVPR1A	0.07124
EBNA1	RRP8	0.14205
EBNA1	HPRT1	0.058967
EBNA1	YWHAG	0.14382
EBNA1	NPM3	0.16831
EBNA1	DNASE1	0.13064
EBNA1	RNF216	0.089519
EBNA1	MAGI2	0.062964
EBNA1	EPOR	0.13371
EBNA1	EIF2A	0.11714
EBNA1	KCTD5	0.16244
EBNA1	CASP1	0.065023
EBNA1	ZRANB3	0.045573
EBNA1	GLUD1	0.104
EBNA1	FLT3	0.090042
EBNA1	EXOC2	0.18731
EBNA1	ATG12	0.17406
EBNA1	RARG	0.01386
EBNA1	KIF1B	0.16363

EBNA1	CAV3	0.040302
EBNA1	SMARCB1	0.074555
EBNA1	CDHR3	0.038413
EBNA1	YEATS2	0.14893
EBNA1	KPNA5	0.16908
EBNA1	ARHGAP10	0.055267
EBNA1	S100A4	0.39861
EBNA1	ERCC5	0.084859
EBNA1	ABCG2	0.14902
EBNA1	GUCA1B	0.056858
EBNA1	NAPA	0.1264
EBNA1	TNPO2	0.16258
EBNA1	COL4A1	0.14099
EBNA1	DNAJC21	0.16309
EBNA1	S100A12	0.22473
EBNA1	FYCO1	0.22897
EBNA1	RGS16	0.22505
EBNA1	WNT1	0.032065
EBNA1	RAB9B	0.16231
EBNA1	C19orf80	0.21561
EBNA1	RCN1	0.4344
EBNA1	PHC2	0.11828
EBNA1	MTMR2	0.024172
EBNA1	BMPR2	0.024
EBNA1	IKBKE	0.060227
EBNA1	DDX39B	0.11377
EBNA1	WNT3A	0.015715
EBNA1	BAZ2B	0.11451
EBNA1	CASP8	0.028068
EBNA1	NEFL	0.10349
EBNA1	KCNJ6	0.16593
EBNA1	MYH2	0.11628
EBNA1	FKBP7	0.049862
EBNA1	LRRFIP1	0.094334
EBNA1	PPP2R3C	0.017745
EBNA1	TNFRSF6B	0.19173
EBNA1	SMAD6	0.029828
EBNA1	TROAP	0.20853
EBNA1	MT1H	0.12595
EBNA1	RAPGEF4	0.066743
EBNA1	PSIP1	0.11828
EBNA1	SNRPD2	0.096138
EBNA1	SSX2IP	0.18708
EBNA1	DPP4	0.12338
EBNA1	RERE	0.04241
EBNA1	SPSB4	0.20676
EBNA1	EIF3E	0.1574
EBNA1	EIF4E	0.16278
EBNA1	DSCC1	0.11573
EBNA1	RAMP1	0.08557
EBNA1	SDC2	0.093234
EBNA1	AK6	0.23504
EBNA1	HRAS	0.018837
EBNA1	EMILIN2	0.19964
EBNA1	AIM2	0.064708
EBNA1	CLTCL1	0.15928
EBNA1	NPHP1	0.09253
EBNA1	CHD7	0.035622
EBNA1	SLC15A2	0.14689
EBNA1	DNMT3A	0.12771
EBNA1	ATP6V1F	0.12855
EBNA1	OAS3	0.11858
EBNA1	INHBA	0.03275
EBNA1	RQCD1	0.12781
EBNA1	PAG1	0.17009
EBNA1	NPB	0.17753
EBNA1	PIM3	0.095431
EBNA1	ACTN3	0.3658

EBNA1	ANXA4	0.03983
EBNA1	ATP5F1	0.20613
EBNA1	FBN1	0.36592
EBNA1	NR4A1	0.020338
EBNA1	RNF182	0.095336
EBNA1	MIF4GD	0.19263
EBNA1	MAP4K3	0.10145
EBNA1	TNNI2	0.14395
EBNA1	PDPK1	0.013193
EBNA1	RAD1	0.094696
EBNA1	ATG14	0.18568
EBNA1	F10	0.24072
EBNA1	PTK2B	0.011958
EBNA1	CAPN1	0.27383
EBNA1	PAX3	0.016065
EBNA1	UCKL1	0.22253
EBNA1	PLXDC1	0.1454
EBNA1	HSPB8	0.067456
EBNA1	MED15	0.10786
EBNA1	DSG1	0.021224
EBNA1	CEACAM6	0.18274
EBNA1	GSTZ1	0.19798
EBNA1	TLE1	0.068333
EBNA1	SKIL	0.070638
EBNA1	LILRB3	0.1699
EBNA1	ZNF346	0.1538
EBNA1	SRPK1	0.045161
EBNA1	IPO7	0.12317
EBNA1	CFL2	0.1914
EBNA1	ACAA1	0.11078
EBNA1	GHRHR	0.064564
EBNA1	CX3CR1	0.081159
EBNA1	PCDHA8	0.036102
EBNA1	NDOR1	0.11587
EBNA1	PPM1A	0.018497
EBNA1	LETMD1	0.13731
EBNA1	SYNM	0.13334
EBNA1	PPP2R5E	0.1214
EBNA1	SCGN	0.11647
EBNA1	SAE1	0.046138
EBNA1	TSN	0.2043
EBNA1	VPS51	0.16727
EBNA1	CELSR3	0.28499
EBNA1	CAND1	0.13781
EBNA1	FCHSD2	0.22232
EBNA1	CATSPER1	0.11114
EBNA1	RILP	0.18889
EBNA1	ING5	0.083293
EBNA1	SENP2	0.11145
EBNA1	RCOR3	0.14253
EBNA1	MAPKAPK5	0.049996
EBNA1	LMAN1	0.054241
EBNA1	UBE2B	0.056897
EBNA1	SDHA	0.18594
EBNA1	NR2C2	0.019767
EBNA1	WASF1	0.1117
EBNA1	CTIF	0.20825
EBNA1	RNF115	0.13035
EBNA1	SYNJ2	0.12128
EBNA1	KIAA1524	0.19667
EBNA1	ZNRF3	0.10017
EBNA1	TCEAL5	0.16206
EBNA1	CACNA1A	0.03218
EBNA1	AGO3	0.16395
EBNA1	RPGRIP1	0.16698
EBNA1	SPOCK1	0.070081
EBNA1	N6AMT1	0.28766
EBNA1	S100A5	0.011028

EBNA1	POLR2J	0.063635
EBNA1	CD4	0.046855
EBNA1	ARF1	0.047359
EBNA1	GEMIN8	0.16688
EBNA1	PEX13	0.15145
EBNA1	EMR4P	0.013602
EBNA1	MEOX2	0.037444
EBNA1	TFAP4	0.073682
EBNA1	SETD8	0.16486
EBNA1	MAGEA4	0.20423
EBNA1	TAGLN2	0.19597
EBNA1	SIRT4	0.29309
EBNA1	FYTTD1	0.25777
EBNA1	DNAJB6	0.14467
EBNA1	PCDHA4	0.036102
EBNA1	CDH15	0.027955
EBNA1	LDHD	0.19273
EBNA1	TESC	0.20467
EBNA1	CLIC3	0.1434
EBNA1	CHD1	0.07318
EBNA1	RBP4	0.088247
EBNA1	HOXB9	0.034345
EBNA1	SETD7	0.18215
EBNA1	PARD3	0.08338
EBNA1	FMNL1	0.13126
EBNA1	UBE2Q1	0.11657
EBNA1	IFNLR1	0.18563
EBNA1	EFHB	0.044917
EBNA1	LGI1	0.10371
EBNA1	NME2	0.044828
EBNA1	KIAA0319	0.15765
EBNA1	CRY2	0.056651
EBNA1	ARL2	0.071926
EBNA1	EYS	0.071713
EBNA1	MICAL1	0.20226
EBNA1	NDNL2	0.16139
EBNA1	ROPN1L	0.18913
EBNA1	OBSCN	0.053682
EBNA1	UCN	0.097716
EBNA1	PCDHGB7	0.038413
EBNA1	FGFR3	0.037067
EBNA1	RWDD1	0.22232
EBNA1	SMPDL3A	0.16225
EBNA1	RHO	0.088775
EBNA1	LIG3	0.051607
EBNA1	HSPB6	0.19195
EBNA1	ADAMTS4	0.086608
EBNA1	PJA1	0.13928
EBNA1	AHNAK	0.18434
EBNA1	CCNF	0.15034
EBNA1	HOOK3	0.11022
EBNA1	SETD1A	0.19126
EBNA1	GLB1	0.0782
EBNA1	SMARCC2	0.090732
EBNA1	FCN3	0.14426
EBNA1	PPFIA1	0.19597
EBNA1	PPP4R4	0.21737
EBNA1	DCLRE1B	0.10513
EBNA1	OSBPL1A	0.085223
EBNA1	GOPC	0.09033
EBNA1	CD82	0.19649
EBNA1	TNFRSF1A	0.067266
EBNA1	DZIP3	0.1794
EBNA1	RALA	0.090609
EBNA1	CD226	0.075525
EBNA1	MOB4	0.16004
EBNA1	BIN3	0.15722
EBNA1	POLR2A	0.061227

EBNA1	RPH3A	0.22765
EBNA1	DCP1A	0.099559
EBNA1	AMOTL2	0.22067
EBNA1	SMARCA1	0.063969
EBNA1	SRI	0.24619
EBNA1	SELL	0.13122
EBNA1	OCM	0.02299
EBNA1	RTP3	0.18606
EBNA1	LRP4	0.22478
EBNA1	KAT6A	0.047537
EBNA1	BDKRB2	0.076866
EBNA1	EVI5L	0.16509
EBNA1	NEK2	0.029047
EBNA1	RAD54L	0.098322
EBNA1	TSC2	0.045571
EBNA1	SLC7A8	0.034946
EBNA1	PSG5	0.20903
EBNA1	PIK3R2	0.10037
EBNA1	MAP3K14	0.028602
EBNA1	BMX	0.039372
EBNA1	INADL	0.16293
EBNA1	SEL1L	0.17821
EBNA1	GNL3L	0.10614
EBNA1	PTPN12	0.041221
EBNA1	FEN1	0.027124
EBNA1	PCDHB12	0.036102
EBNA1	NONO	0.14956
EBNA1	CUL2	0.13546
EBNA1	CCT3	0.11991
EBNA1	SRSF7	0.15255
EBNA1	CABP5	0.040569
EBNA1	SLIT2	0.070656
EBNA1	PKD1	0.017739
EBNA1	HERC2	0.071435
EBNA1	TBC1D5	0.14071
EBNA1	SNED1	0.042364
EBNA1	AKAP9	0.077106
EBNA1	MIB1	0.11449
EBNA1	PASK	0.083616
EBNA1	BLM	0.022212
EBNA1	ARNT	0.022257
EBNA1	SYNE3	0.12529
EBNA1	DOCK4	0.11203
EBNA1	NCLN	0.11691
EBNA1	HNRNPUL1	0.15094
EBNA1	GPRASP1	0.21561
EBNA1	MAGEE1	0.15226
EBNA1	CNPY2	0.19096
EBNA1	SFI1	0.16914
EBNA1	MATN2	0.34199
EBNA1	BCAR1	0.044198
EBNA1	PSMD9	0.079172
EBNA1	AXIN1	0.017253
EBNA1	SIRT6	0.12782
EBNA1	STK4	0.011186
EBNA1	GPD2	0.025755
EBNA1	PCDHA10	0.035121
EBNA1	CIB1	0.3061
EBNA1	IRAK2	0.026025
EBNA1	MAPK14	0.012
EBNA1	UBR5	0.10335
EBNA1	PRRG4	0.037952
EBNA1	ANKRD44	0.22232
EBNA1	KIF13A	0.070278
EBNA1	FNTA	0.1607
EBNA1	EIF5A2	0.12543
EBNA1	MAPRE3	0.15961
EBNA1	CLCN3	0.10244

EBNA1	DNAJB2	0.15504
EBNA1	DNMT1	0.10787
EBNA1	PCDHA11	0.036102
EBNA1	HDAC10	0.063278
EBNA1	PCDHB7	0.037277
EBNA1	TNK1	0.13079
EBNA1	PHF8	0.085354
EBNA1	PPP2R3A	0.32296
EBNA1	GSPT1	0.11237
EBNA1	DSC1	0.048269
EBNA1	AKAP3	0.23525
EBNA1	PRPF19	0.11325
EBNA1	EIF2B4	0.14347
EBNA1	NCF1	0.020207
EBNA1	PSMC2	0.06076
EBNA1	TSSK4	0.067242
EBNA1	USE1	0.13876
EBNA1	DLL1	0.22865
EBNA1	TAF2	0.08934
EBNA1	ACTN1	0.19379
EBNA1	CABP2	0.037748
EBNA1	VDR	0.026088
EBNA1	PEX11B	0.1716
EBNA1	PMP22	0.18302
EBNA1	RAB6A	0.11476
EBNA1	STK38	0.021826
EBNA1	TNKS2	0.0908
EBNA1	RCHY1	0.063996
EBNA1	CCND1	0.029518
EBNA1	CD164	0.12703
EBNA1	TBC1D15	0.15285
EBNA1	AOC3	0.081949
EBNA1	FUS	0.15304
EBNA1	NPHP3	0.093692
EBNA1	USP46	0.1665
EBNA1	UBD	0.12238
EBNA1	MYH10	0.066921
EBNA1	CDT1	0.11305
EBNA1	SYNE1	0.11241
EBNA1	FBXO3	0.17888
EBNA1	LRP6	0.012735
EBNA1	NCL	0.13075
EBNA1	ITGA1	0.12953
EBNA1	SVEP1	0.037841
EBNA1	NOS1	0.082
EBNA1	EHD4	0.21957
EBNA1	APBA3	0.14181
EBNA1	RYR3	0.018429
EBNA1	PCDHB14	0.035257
EBNA1	HNRNPK	0.089747
EBNA1	LCAT	0.14245
EBNA1	OBFC1	0.060341
EBNA1	TWIST1	0.0219
EBNA1	CSNK1E	0.041924
EBNA1	MEF2A	0.026681
EBNA1	PNPT1	0.077006
EBNA1	UPF3B	0.19722
EBNA1	AKAP13	0.038386
EBNA1	ATG2A	0.22232
EBNA1	ZC3H7B	0.12602
EBNA1	LZTFL1	0.18997
EBNA1	TLN1	0.016171
EBNA1	BTRC	0.071544
EBNA1	PCDHGA11	0.038413
EBNA1	RHBDL1	0.1283
EBNA1	HNF1B	0.018715
EBNA1	UBE2D1	0.064811
EBNA1	MYO6	0.066099

EBNA1	LCMT2	0.27834
EBNA1	USP8	0.080231
EBNA1	FIG4	0.077096
EBNA1	TNFSF14	0.092984
EBNA1	CDC6	0.050139
EBNA1	CDH3	0.061748
EBNA1	PLAT	0.13108
EBNA1	TAX1BP3	0.14459
EBNA1	SNX4	0.18254
EBNA1	NEGR1	0.17552
EBNA1	HTR6	0.13324
EBNA1	SEPT11	0.14927
EBNA1	DLGAP1	0.15733
EBNA1	MFI2	0.12147
EBNA1	FLNA	0.036183
EBNA1	HIST1H4A	0.058922
EBNA1	CTTNBP2NL	0.21445
EBNA1	SERPINF2	0.10972
EBNA1	FBXL5	0.15118
EBNA1	SGSM3	0.15017
EBNA1	DUOX1	0.10139
EBNA1	F3	0.041116
EBNA1	CECR2	0.15758
EBNA1	SPINK1	0.18512
EBNA1	ABCD1	0.10187
EBNA1	DDX3X	0.016628
EBNA1	PCDHA2	0.036102
EBNA1	SH3GLB2	0.15469
EBNA1	CDK16	0.057485
EBNA1	RASEF	0.016095
EBNA1	PRSS3	0.36715
EBNA1	ANXA8	0.022372
EBNA1	RPS6KC1	0.095205
EBNA1	MAP1S	0.064889
EBNA1	BTK	0.013044
EBNA1	CLEC4G	0.2027
EBNA1	PPIL3	0.19989
EBNA1	CALML5	0.033943
EBNA1	LRRC4C	0.20278
EBNA1	MYL2	0.32792
EBNA1	BAIAP2	0.078205
EBNA1	KAT5	0.080953
EBNA1	PIWIL1	0.16709
EBNA1	ALOX12	0.088685
EBNA1	EDEM1	0.27796
EBNA1	LRP1	0.12627
EBNA1	APBA2	0.1585
EBNA1	DHDDS	0.23463
EBNA1	MKI67	0.099463
EBNA1	JADE1	0.11166
EBNA1	MTF1	0.010021
EBNA1	FGFR1	0.014157
EBNA1	GEMIN2	0.14204
EBNA1	SELE	0.094271
EBNA1	TONSL	0.14158
EBNA1	CACNB4	0.071355
EBNA1	RBM12B	0.26974
EBNA1	CKS1B	0.15887
EBNA1	ATP10B	0.20755
EBNA1	TIMM23	0.1476
EBNA1	DICER1	0.026818
EBNA1	HADHB	0.062149
EBNA1	APPL1	0.047204
EBNA1	VPRBP	0.16167
EBNA1	SENP5	0.15513
EBNA1	SUV39H1	0.11321
EBNA1	COPS6	0.15497
EBNA1	SNX2	0.15113

EBNA1	MAP2K5	0.06812
EBNA1	RNF11	0.10051
EBNA1	ITGAM	0.097688
EBNA1	CDH17	0.053859
EBNA1	CDK11B	0.052192
EBNA1	EFCAB9	0.044917
EBNA1	PEX1	0.064438
EBNA1	NUDT18	0.17322
EBNA1	EXOSC2	0.1757
EBNA1	ERG	0.017429
EBNA1	ZNF622	0.10923
EBNA1	NFS1	0.1211
EBNA1	EEF1E1	0.13723
EBNA1	TNNC2	0.038469
EBNA1	GNA12	0.07483
EBNA1	ZC3H14	0.19122
EBNA1	LSM11	0.15336
EBNA1	BUB1	0.041045
EBNA1	RIT1	0.15511
EBNA1	KCNA4	0.14083
EBNA1	RIOK3	0.15459
EBNA1	DMC1	0.081812
EBNA1	ATP5A1	0.11313
EBNA1	RAB21	0.15208
EBNA1	SGPL1	0.027249
EBNA1	ADAR	0.14136
EBNA1	OIP5	0.12964
EBNA1	GAD1	0.084522
EBNA1	WDTC1	0.10499
EBNA1	SNX19	0.34801
EBNA1	MAP2K4	0.069325
EBNA1	ASPH	0.099083
EBNA1	LIN28B	0.13569
EBNA1	TUBGCP2	0.11003
EBNA1	HIP1	0.05697
EBNA1	MBD3	0.1123
EBNA1	ITGA4	0.12426
EBNA1	FSTL1	0.025921
EBNA1	SRC	0.021201
EBNA1	MRE11A	0.070238
EBNA1	USP47	0.1285
EBNA1	CLPP	0.23272
EBNA1	CHORDC1	0.14481
EBNA1	SMG6	0.063857
EBNA1	ID1	0.031865
EBNA1	TRPC1	0.098238
EBNA1	TOM1	0.16781
EBNA1	PITPNC1	0.21541
EBNA1	PDGFRB	0.047655
EBNA1	KIF2C	0.063011
EBNA1	CACNA1B	0.11071
EBNA1	SEPT5	0.02011
EBNA1	PAX8	0.010695
EBNA1	APPBP2	0.18501
EBNA1	C1QTNF3	0.12883
EBNA1	GAD2	0.093835
EBNA1	WNK1	0.10505
EBNA1	DACT1	0.084007
EBNA1	PCNA	0.019275
EBNA1	CD274	0.15779
EBNA1	ITSN1	0.16156
EBNA1	ATP7B	0.039809
EBNA1	KDM4A	0.102
EBNA1	TRAM2	0.19488
EBNA1	CEBPB	0.021547
EBNA1	BRCC3	0.096277
EBNA1	BBS1	0.16446
EBNA1	SLC22A4	0.13446

EBNA1	ETV7	0.048981
EBNA1	HNRNPF	0.14763
EBNA1	CCDC141	0.22232
EBNA1	PCDHGA12	0.038413
EBNA1	ELOVL1	0.13059
EBNA1	PRDX3	0.064956
EBNA1	DST	0.15826
EBNA1	HIF1AN	0.089158
EBNA1	COL18A1	0.056817
EBNA1	MYL6	0.37166
EBNA1	COMP	0.30496
EBNA1	RNF31	0.087359
EBNA1	EIF3G	0.22362
EBNA1	MLLT6	0.13388
EBNA1	CCDC47	0.36181
EBNA1	SNAP47	0.18989
EBNA1	EXOSC7	0.16035
EBNA1	LIMD1	0.062172
EBNA1	MX1	0.076634
EBNA1	SATB1	0.03363
EBNA1	RIN1	0.17075
EBNA1	RCOR1	0.089509
EBNA1	ANXA7	0.27449
EBNA1	SON	0.13782
EBNA1	MAP3K7CL	0.18485
EBNA1	RNF139	0.088634
EBNA1	PRC1	0.022231
EBNA1	CDH26	0.038413
EBNA1	FRK	0.11104
EBNA1	CHP2	0.33241
EBNA1	ADNP	0.01888
EBNA1	TOX3	0.12707
EBNA1	PSMA7	0.037888
EBNA1	ZFP36L1	0.01863
EBNA1	CD97	0.017957
EBNA1	ABCC2	0.16844
EBNA1	PTPRB	0.033851
EBNA1	MUC1	0.10295
EBNA1	RANBP3	0.1376
EBNA1	CTNNA1	0.049694
EBNA1	VBP1	0.11158
EBNA1	UBE2L6	0.14281
EBNA1	ZHX3	0.037479
EBNA1	RSPO1	0.11069
EBNA1	CDK5R1	0.048621
EBNA1	SUB1	0.11454
EBNA1	ANP32A	0.12032
EBNA1	LTBP2	0.033496
EBNA1	PPM1F	0.044917
EBNA1	AKT3	0.013897
EBNA1	TEAD2	0.05489
EBNA1	PLOD3	0.12746
EBNA1	TOLLIP	0.16456
EBNA1	EIF4EBP2	0.16199
EBNA1	RUVBL1	0.061312
EBNA1	LRP12	0.16081
EBNA1	NECAB2	0.46719
EBNA1	ELF3	0.030418
EBNA1	OGT	0.070405
EBNA1	PBRM1	0.13414
EBNA1	NCOR1	0.025689
EBNA1	APITD1	0.089669
EBNA1	HGF	0.077387
EBNA1	DNAJB9	0.1431
EBNA1	SLBP	0.13767
EBNA1	USH2A	0.11665
EBNA1	PHF2	0.1069
EBNA1	PODXL2	0.18421

EBNA1	APLP1	0.028103
EBNA1	RFC2	0.061458
EBNA1	TYROBP	0.088366
EBNA1	CANX	0.19558
EBNA1	HNF1A	0.016292
EBNA1	RHOT2	0.19724
EBNA1	DOCK9	0.080834
EBNA1	RTKN	0.095844
EBNA1	KLHL3	0.079023
EBNA1	EHMT1	0.1609
EBNA1	KDR	0.023595
EBNA1	SNAP23	0.1625
EBNA1	MMP15	0.2296
EBNA1	MKNK1	0.069574
EBNA1	TTC8	0.17217
EBNA1	PDE6D	0.24968
EBNA1	ASCC3	0.097791
EBNA1	PPP2R5B	0.12115
EBNA1	GADD45A	0.13593
EBNA1	PLCB3	0.02118
EBNA1	LCP2	0.17177
EBNA1	ORAI2	0.20909
EBNA1	SUSD1	0.042436
EBNA1	APBA1	0.11086
EBNA1	DKK1	0.075684
EBNA1	ANXA11	0.24178
EBNA1	SPINK9	0.19084
EBNA1	ITM2C	0.13964
EBNA1	USP54	0.20849
EBNA1	PPP3CB	0.1184
EBNA1	TAB2	0.061961
EBNA1	SNRPB	0.10015
EBNA1	KRT19	0.12971
EBNA1	PCBD2	0.09319
EBNA1	S100G	0.041818
EBNA1	PRKAA1	0.010528
EBNA1	ATG7	0.085251
EBNA1	MDM4	0.090292
EBNA1	SCYL2	0.067087
EBNA1	NCKIPSD	0.20544
EBNA1	SPINK7	0.1968
EBNA1	NQO1	0.16175
EBNA1	ACSS1	0.040196
EBNA1	ACACB	0.11379
EBNA1	RBM39	0.15739
EBNA1	IGF2BP3	0.14375
EBNA1	TRIM27	0.047059
EBNA1	CSPG5	0.062359
EBNA1	MC3R	0.12879
EBNA1	PAX2	0.016477
EBNA1	CNOT4	0.10316
EBNA1	DLG2	0.11094
EBNA1	ZNF136	0.10036
EBNA1	MAST1	0.065258
EBNA1	MAU2	0.16342
EBNA1	SH3PXD2B	0.10867
EBNA1	IQGAP1	0.1258
EBNA1	HIRA	0.051614
EBNA1	ATP6V0C	0.11836
EBNA1	STAP2	0.042498
EBNA1	CCNA2	0.062578
EBNA1	FZD1	0.036683
EBNA1	ELAVL1	0.098867
EBNA1	RAD51	0.041901
EBNA1	BMI1	0.051029
EBNA1	OPTN	0.14814
EBNA1	TMED1	0.11608
EBNA1	RAB9A	0.058461

EBNA1	TIMP1	0.10313
EBNA1	MATN3	0.44103
EBNA1	EFCAB8	0.044917
EBNA1	ATL2	0.070544
EBNA1	RAP1A	0.087112
EBNA1	MAST2	0.053783
EBNA1	SH3BP2	0.10308
EBNA1	YEATS4	0.063502
EBNA1	SFN	0.055288
EBNA1	FBN3	0.058344
EBNA1	MDC1	0.15216
EBNA1	PGRMC1	0.15432
EBNA1	NHP2L1	0.1737
EBNA1	SH3KBP1	0.20163
EBNA1	AMOTL1	0.20135
EBNA1	MYLIP	0.087545
EBNA1	TENC1	0.095049
EBNA1	STK3	0.036742
EBNA1	DLGAP4	0.1652
EBNA1	TOMM70A	0.12266
EBNA1	TCERG1	0.13913
EBNA1	PCDH8	0.028238
EBNA1	UBE4A	0.17618
EBNA1	LRRK2	0.018844
EBNA1	TSC1	0.05914
EBNA1	CPNE6	0.057072
EBNA1	CD276	0.10279
EBNA1	RNF41	0.069754
EBNA1	RASSF2	0.17325
EBNA1	TJAP1	0.18138
EBNA1	ETV6	0.048928
EBNA1	SCHIP1	0.17873
EBNA1	RET	0.13564
EBNA1	OS9	0.1691
EBNA1	ARHGAP26	0.058176
EBNA1	COL1A2	0.088112
EBNA1	LSM7	0.13778
EBNA1	SRY	0.040347
EBNA1	JAM2	0.16828
EBNA1	MAFB	0.036959
EBNA1	ALOX5	0.088717
EBNA1	SOD1	0.020754
EBNA1	WASH1	0.14634
EBNA1	SPRED1	0.037966
EBNA1	PARP1	0.051712
EBNA1	CHEK1	0.052963
EBNA1	FCRL4	0.20279
EBNA1	SF3B4	0.20329
EBNA1	EPS8	0.19236
EBNA1	PATL1	0.12859
EBNA1	TRAF5	0.094441
EBNA1	PARP10	0.19784
EBNA1	NSMCE4A	0.18854
EBNA1	SNTA1	0.10024
EBNA1	ATP11A	0.10025
EBNA1	CDH22	0.038413
EBNA1	THBS4	0.031599
EBNA1	FBXL3	0.15129
EBNA1	DDX1	0.068263
EBNA1	MC4R	0.11533
EBNA1	CDHR4	0.038413
EBNA1	CHD1L	0.13532
EBNA1	SLC3A2	0.09155
EBNA1	UBQLN1	0.12338
EBNA1	USP13	0.073665
EBNA1	SLC6A3	0.092913
EBNA1	CALCR	0.071435
EBNA1	PTGFRN	0.17533

EBNA1	EIF4EBP3	0.16663
EBNA1	PTPN11	0.025914
EBNA1	CPSF1	0.18158
EBNA1	MS4A3	0.19079
EBNA1	HOXA5	0.011298
EBNA1	HCFC1	0.030807
EBNA1	FLNB	0.12401
EBNA1	MSLN	0.16732
EBNA1	RAB11FIP5	0.071064
EBNA1	SUZ12	0.099604
EBNA1	CDH23	0.19208
EBNA1	KIT	0.024277
EBNA1	UBC	0.026336
EBNA1	RAB11FIP4	0.37102
EBNA1	RLF	0.084382
EBNA1	KRT18	0.089313
EBNA1	FGF13	0.079197
EBNA1	NTM	0.16748
EBNA1	PDCD6IP	0.14951
EBNA1	GAB1	0.069624
EBNA1	HSFX1	0.051131
EBNA1	RARRES2	0.14819
EBNA1	HSPB1	0.01868
EBNA1	BMPR1A	0.021129
EBNA1	MYCBPAP	0.14386
EBNA1	MEF2D	0.045637
EBNA1	PSMA2	0.049289
EBNA1	ASXL1	0.095461
EBNA1	APBB2	0.060073
EBNA1	TP53BP1	0.065572
EBNA1	MAP1LC3A	0.14558
EBNA1	UBXN1	0.12618
EBNA1	PRPF3	0.17474
EBNA1	ATP6V1H	0.13171
EBNA1	CDH12	0.03561
EBNA1	JMJD6	0.077697
EBNA1	RRAGA	0.094432
EBNA1	HSPB2	0.15859
EBNA1	EIF3A	0.096917
EBNA1	MGARP	0.10491
EBNA1	NFKB2	0.020282
EBNA1	UBE2I	0.022198
EBNA1	CASP7	0.077142
EBNA1	H2AFX	0.080354
EBNA1	RHOU	0.03867
EBNA1	SSRP1	0.12219
EBNA1	SUMO3	0.19667
EBNA1	TLR2	0.025309
EBNA1	ATF3	0.035905
EBNA1	SOS2	0.057023
EBNA1	SYNE2	0.080774
EBNA1	BCL2L2	0.16853
EBNA1	SUV39H2	0.15761
EBNA1	IL10RA	0.17772
EBNA1	CLIC5	0.090362
EBNA1	UBE2F	0.10976
EBNA1	AGTR2	0.064699
EBNA1	IFIH1	0.064915
EBNA1	C1orf64	0.010562
EBNA1	IER3	0.13065
EBNA1	CNKSR1	0.092449
EBNA1	LDHAL6B	0.18025
EBNA1	MAGEA1	0.14306
EBNA1	MBOAT7	0.18147
EBNA1	LOX	0.11281
EBNA1	ELP4	0.11295
EBNA1	ELTD1	0.02284
EBNA1	CAV2	0.065157

EBNA1	FXR2	0.27944
EBNA1	POLR2B	0.063508
EBNA1	VANGL1	0.20278
EBNA1	MPP1	0.15659
EBNA1	TRIP6	0.11509
EBNA1	PKP4	0.11819
EBNA1	COMMD4	0.21561
EBNA1	AGGF1	0.15669
EBNA1	ORC5	0.08819
EBNA1	CLU	0.040881
EBNA1	PPIB	0.17784
EBNA1	PRRG1	0.039928
EBNA1	NRG1	0.025779
EBNA1	A1CF	0.13495
EBNA1	NIFK	0.17855
EBNA1	ATXN7	0.13222
EBNA1	UBA6	0.086581
EBNA1	TDP2	0.10198
EBNA1	SUDS3	0.16573
EBNA1	CHCHD3	0.13613
EBNA1	PRKACB	0.020758
EBNA1	LPHN1	0.12091
EBNA1	WIPF2	0.20278
EBNA1	MCM2	0.036349
EBNA1	PLCE1	0.10532
EBNA1	CD163	0.17815
EBNA1	IL10RB	0.17703
EBNA1	DYNLL1	0.084298
EBNA1	PIDD	0.17814
EBNA1	ADAMTSL4	0.11698
EBNA1	MYOT	0.16261
EBNA1	PDCD1	0.16318
EBNA1	HNRNPLL	0.18408
EBNA1	IL12B	0.023062
EBNA1	WDR48	0.12177
EBNA1	PDCD11	0.16782
EBNA1	G3BP1	0.12926
EBNA1	RASSF1	0.070502
EBNA1	NELFCD	0.1406
EBNA1	KHDRBS1	0.13834
EBNA1	EID1	0.11369
EBNA1	PCDHB4	0.029863
EBNA1	IGHMBP2	0.022124
EBNA1	SNX3	0.18153
EBNA1	ATP6V1D	0.12917
EBNA1	ABL2	0.074246
EBNA1	DOCK3	0.19058
EBNA1	STX6	0.1126
EBNA1	MAK	0.02746
EBNA1	CYLD	0.028496
EBNA1	GP6	0.15582
EBNA1	SNW1	0.10906
EBNA1	PPL	0.13707
EBNA1	KLK3	0.24087
EBNA1	WAC	0.12716
EBNA1	TTR	0.18619
EBNA1	ATP2B4	0.11903
EBNA1	BBS7	0.15764
EBNA1	CRYAA	0.084594
EBNA1	NEDD4L	0.076642
EBNA1	PCDH9	0.034806
EBNA1	PPP6R2	0.17034
EBNA1	HPCAL1	0.044917
EBNA1	MIS12	0.13182
EBNA1	ARF4	0.10989
EBNA1	DAPK1	0.1038
EBNA1	SOX6	0.013815
EBNA1	ITGA3	0.15459

EBNA1	COG3	0.10739
EBNA1	TNIK	0.054216
EBNA1	BRD7	0.050137
EBNA1	RNASE1	0.18256
EBNA1	NSF	0.081774
EBNA1	RBBP7	0.086543
EBNA1	PTRF	0.10397
EBNA1	ARIH1	0.13473
EBNA1	ECM1	0.099136
EBNA1	KLC2	0.15622
EBNA1	BEGAIN	0.20909
EBNA1	ARF6	0.062089
EBNA1	PAAF1	0.19747
EBNA1	KISS1R	0.10253
EBNA1	DNASE1L3	0.021862
EBNA1	RIPK2	0.013106
EBNA1	SNRPD3	0.098006
EBNA1	CLEC2A	0.20772
EBNA1	CTSB	0.03986
EBNA1	ILKAP	0.087235
EBNA1	CLIC4	0.14861
EBNA1	RAB14	0.029502
EBNA1	STX12	0.12661
EBNA1	SAMHD1	0.13103
EBNA1	ACTB	0.02502
EBNA1	EIF4G1	0.16783
EBNA1	SLC22A5	0.13929
EBNA1	KIF3A	0.085838
EBNA1	SCNN1A	0.14957
EBNA1	CGREF1	0.03581
EBNA1	CHAF1B	0.1045
EBNA1	RUSC1	0.14771
EBNA1	NPNT	0.045466
EBNA1	PEX12	0.13306
EBNA1	FLOT1	0.13825
EBNA1	FAM49B	0.019313
EBNA1	BNIP2	0.26748
EBNA1	GABARAPL2	0.16937
EBNA1	PPIH	0.16427
EBNA1	PRKDC	0.025629
EBNA1	JAG2	0.23821
EBNA1	RGR	0.15543
EBNA1	MYRIP	0.15697
EBNA1	CTTN	0.18384
EBNA1	C1QA	0.18307
EBNA1	PPP1R16A	0.2091
EBNA1	EFCAB7	0.044917
EBNA1	TACC1	0.12082
EBNA1	CREB3	0.01068
EBNA1	LTBP3	0.034065
EBNA1	PLK4	0.076759
EBNA1	SKIV2L2	0.10198
EBNA1	ADRB2	0.029917
EBNA1	DDX18	0.1807
EBNA1	ATP2B1	0.13485
EBNA1	POLR1B	0.095545
EBNA1	KMT2D	0.12041
EBNA1	CEP97	0.20071
EBNA1	PTPRU	0.052326
EBNA1	TPCN2	0.026865
EBNA1	RUNX1T1	0.027908
EBNA1	CENPI	0.095451
EBNA1	SHC3	0.16254
EBNA1	LGALS3	0.13358
EBNA1	FCGR2B	0.17603
EBNA1	IST1	0.27783
EBNA1	MAP4K5	0.095502
EBNA1	FCHO2	0.14453

EBNA1	TIPRL	0.20923
EBNA1	SKP2	0.12032
EBNA1	PLAA	0.19657
EBNA1	DUSP15	0.015042
EBNA1	MIB2	0.10997
EBNA1	EHD2	0.20811
EBNA1	PVRL2	0.076009
EBNA1	COPS5	0.12317
EBNA1	RNF135	0.13954
EBNA1	CCNE2	0.056459
EBNA1	C5AR2	0.14729
EBNA1	CADPS	0.34812
EBNA1	JUND	0.028259
EBNA1	ABCA1	0.022531
EBNA1	MARK2	0.057482
EBNA1	CNTROB	0.16151
EBNA1	HEXIM2	0.13654
EBNA1	HRNR	0.028406
EBNA1	PKD2	0.026894
EBNA1	NCAPH	0.16182
EBNA1	PCDHGB2	0.038413
EBNA1	BBS4	0.036275
EBNA1	PDZD7	0.1789
EBNA1	PPP2CB	0.037674
EBNA1	DLC1	0.064392
EBNA1	STXBP4	0.014942
EBNA1	IPO9	0.15382
EBNA1	ANXA8L1	0.023566
EBNA1	PDIA6	0.057199
EBNA1	HDAC5	0.018762
EBNA1	TRPC6	0.10891
EBNA1	ZNF655	0.093521
EBNA1	ETF1	0.18901
EBNA1	EMILIN1	0.16215
EBNA1	ADRM1	0.13206
EBNA1	KLK6	0.14276
EBNA1	CCAR1	0.311
EBNA1	PFDN4	0.13595
EBNA1	C19orf57	0.2042
EBNA1	HIST1H3A	0.096821
EBNA1	CCNA1	0.067161
EBNA1	MYCN	0.031043
EBNA1	SPARCL1	0.052509
EBNA1	PDZD3	0.10573
EBNA1	CD40	0.043827
EBNA1	ENPP1	0.042435
EBNA1	PPP1R12A	0.071859
EBNA1	ERC1	0.079201
EBNA1	CTNND1	0.065404
EBNA1	PICALM	0.043629
EBNA1	PLEKHG6	0.051259
EBNA1	GUCA1A	0.061865
EBNA1	SEMA7A	0.13278
EBNA1	CRMP1	0.033889
EBNA1	TGFA	0.089352
EBNA1	IQCB1	0.23557
EBNA1	CCAR2	0.19533
EBNA1	CACTIN	0.12726
EBNA1	PLAUR	0.092212
EBNA1	UTP20	0.13708
EBNA1	DVL1	0.035711
EBNA1	GINS2	0.15357
EBNA1	WDR66	0.044917
EBNA1	LOXL2	0.042036
EBNA1	CASP3	0.048858
EBNA1	SHC1	0.0419
EBNA1	MITF	0.023406
EBNA1	NLGN4Y	0.14538

EBNA1	ELF1	0.04966
EBNA1	COPS3	0.16768
EBNA1	ZMYND8	0.13389
EBNA1	LONRF3	0.16176
EBNA1	GABARAP	0.13769
EBNA1	WWP2	0.044693
EBNA1	C1orf195	0.01723
EBNA1	TGFB1I1	0.078404
EBNA1	PIKFYVE	0.12719
EBNA1	MEX3B	0.034608
EBNA1	FBXW9	0.22232
EBNA1	CD37	0.19091
EBNA1	STAM	0.1463
EBNA1	GSK3A	0.029472
EBNA1	CBLB	0.13581
EBNA1	CRELD2	0.42634
EBNA1	PPP2R5A	0.081654
EBNA1	TMEM30A	0.19114
EBNA1	NCOA3	0.090045
EBNA1	MUM1	0.19477
EBNA1	TIMELESS	0.12859
EBNA1	NEK9	0.010213
EBNA1	OSBPL3	0.14773
EBNA1	MZF1	0.032811
EBNA1	BANF1	0.13713
EBNA1	KXD1	0.20793
EBNA1	CPSF6	0.15829
EBNA1	MAST3	0.096851
EBNA1	FKBP14	0.047025
EBNA1	TOX4	0.15957
EBNA1	ARHGAP44	0.17105
EBNA1	ORC1	0.080522
EBNA1	LEO1	0.12337
EBNA1	CSRP2BP	0.11689
EBNA1	RORA	0.023336
EBNA1	NSMCE1	0.12065
EBNA1	MED7	0.10513
EBNA1	TNFSF10	0.075653
EBNA1	GP1BB	0.15861
EBNA1	REL	0.038877
EBNA1	PCDHB10	0.035257
EBNA1	AIMP2	0.13933
EBNA1	APOA1BP	0.17317
EBNA1	LILRB2	0.10653
EBNA1	PFKFB1	0.08583
EBNA1	EME1	0.13855
EBNA1	APPL2	0.064533
EBNA1	RBM26	0.20926
EBNA1	PSMG1	0.20318
EBNA1	KIF2A	0.079683
EBNA1	PATL2	0.17302
EBNA1	SEPT14	0.16635
EBNA1	NBR1	0.20429
EBNA1	SENP6	0.134
EBNA1	EFCAB13	0.018427
EBNA1	HFE	0.15016
EBNA1	DPM1	0.11519
EBNA1	SF3A3	0.11543
EBNA1	IFT20	0.15192
EBNA1	GHR	0.019964
EBNA1	FBXW7	0.037956
EBNA1	MAGEA6	0.20423
EBNA1	RAB24	0.15665
EBNA1	RAB8A	0.11378
EBNA1	PFKL	0.10369
EBNA1	CXADR	0.038097
EBNA1	RAB11FIP2	0.096932
EBNA1	TRO	0.1529

EBNA1	ATP11C	0.10001
EBNA1	CD27	0.12637
EBNA1	LYST	0.097439
EBNA1	CASQ1	0.031429
EBNA1	ZNF652	0.10783
EBNA1	CEP57	0.10357
EBNA1	SCIMP	0.16948
EBNA1	TIPIN	0.13562
EBNA1	CHIC2	0.22539
EBNA1	AES	0.092308
EBNA1	STX18	0.13281
EBNA1	VAMP7	0.084911
EBNA1	N4BP2	0.15371
EBNA1	SDC1	0.062403
EBNA1	TRAM1	0.13947
EBNA1	DCAF8	0.21589
EBNA1	DNM1L	0.014971
EBNA1	DLGAP3	0.14194
EBNA1	USP9X	0.11638
EBNA1	PDIA2	0.10917
EBNA1	DERL1	0.11725
EBNA1	RSF1	0.069955
EBNA1	GSN	0.18207
EBNA1	RELB	0.04994
EBNA1	SLC11A2	0.042432
EBNA1	FAM96B	0.1375
EBNA1	TRMT112	0.26621
EBNA1	NUP133	0.10156
EBNA1	PCDHGA4	0.037832
EBNA1	PAIP2	0.18659
EBNA1	AMBRA1	0.23695
EBNA1	ADCY4	0.05426
EBNA1	MET	0.030971
EBNA1	TREML2	0.1858
EBNA1	PKP2	0.10923
EBNA1	VPS13A	0.17241
EBNA1	MME	0.026488
EBNA1	CEBPA	0.018426
EBNA1	CSNK1D	0.042875
EBNA1	HADHA	0.066576
EBNA1	PCDHGA3	0.038413
EBNA1	TBKBP1	0.22192
EBNA1	PPP1R27	0.19667
EBNA1	EIF4H	0.21285
EBNA1	HK1	0.12031
EBNA1	TELO2	0.15499
EBNA1	FRMPD2	0.17838
EBNA1	CCNB3	0.066492
EBNA1	PVRL1	0.11101
EBNA1	IGFBP3	0.091725
EBNA1	ASCL1	0.010998
EBNA1	IGFBP7	0.17432
EBNA1	S100A16	0.021529
EBNA1	BLNK	0.15844
EBNA1	RPGRIP1L	0.096726
EBNA1	ILF2	0.13228
EBNA1	POT1	0.079739
EBNA1	GDI1	0.1512
EBNA1	PPFIA3	0.19672
EBNA1	BMPR1B	0.031061
EBNA1	PAM	0.07916
EBNA1	CNTRL	0.16848
EBNA1	GOLGA2	0.19716
EBNA1	PHACTR2	0.20819
EBNA1	TRPC5	0.12714
EBNA1	SP100	0.062227
EBNA1	EIF4A1	0.16937
EBNA1	TDRD7	0.16821

EBNA1	CABP1	0.21009
EBNA1	DDX5	0.066879
EBNA1	RBBP5	0.13445
EBNA1	PILRA	0.17381
EBNA1	YBX1	0.033836
EBNA1	EAF1	0.16773
EBNA1	AKAP1	0.057992
EBNA1	SCEL	0.14884
EBNA1	SLC4A4	0.18348
EBNA1	PKN3	0.073543
EBNA1	ARF5	0.11102
EBNA1	LETM1	0.41282
EBNA1	CRH	0.079953
EBNA1	EIF2AK2	0.054459
EBNA1	FSTL5	0.043666
EBNA1	NDRG1	0.10246
EBNA1	TPR	0.088743
EBNA1	NEFM	0.13202
EBNA1	LEMD3	0.10032
EBNA1	BAG3	0.16264
EBNA1	SPHK1	0.08926
EBNA1	PACSIN3	0.15561
EBNA1	VAPA	0.042901
EBNA1	SCARF2	0.18392
EBNA1	MAPRE1	0.099596
EBNA1	CATSPER2	0.1108
EBNA1	GCA	0.059223
EBNA1	FNIP1	0.20887
EBNA1	TCTEX1D2	0.22232
EBNA1	VRK2	0.050394
EBNA1	SMG1	0.091803
EBNA1	ARAF	0.060676
EBNA1	SRSF10	0.148
EBNA1	GPC3	0.09664
EBNA1	LRRC3	0.20909
EBNA1	PPP4R1	0.16421
EBNA1	TSSK2	0.058773
EBNA1	ELL	0.13491
EBNA1	CTU1	0.22057
EBNA1	PIAS4	0.076533
EBNA1	BCAS3	0.17682
EBNA1	ANGPT2	0.044784
EBNA1	MAD2L1BP	0.15591
EBNA1	ACTR1B	0.14601
EBNA1	PIK3CB	0.093134
EBNA1	RHOB	0.036816
EBNA1	EFHD2	0.06297
EBNA1	CYP4F2	0.023035
EBNA1	FBF1	0.15316
EBNA1	PKN1	0.022862
EBNA1	IL1RL1	0.12349
EBNA1	NSMAF	0.17622
EBNA1	FAM231A	0.032949
EBNA1	TFB1M	0.20589
EBNA1	NCOA5	0.16206
EBNA1	ACHE	0.044769
EBNA1	DSG3	0.032545
EBNA1	YAP1	0.054502
EBNA1	MATR3	0.098576
EBNA1	PCDHB18	0.038413
EBNA1	PPP2CA	0.035785
EBNA1	CEP164	0.15457
EBNA1	MLLT1	0.13143
EBNA1	RCC1	0.1007
EBNA1	PABPC1	0.095961
EBNA1	PLEKHF2	0.085238
EBNA1	JMY	0.12744
EBNA1	SNTB1	0.093247

EBNA1	AMER1	0.179
EBNA1	TRIP11	0.11773
EBNA1	RTF1	0.085134
EBNA1	RWDD3	0.1844
EBNA1	LUC7L3	0.14343
EBNA1	MCM5	0.081734
EBNA1	CD28	0.093626
EBNA1	CTDSP1	0.078928
EBNA1	HLA-DRB1	0.035409
EBNA1	ITIH1	0.037954
EBNA1	PRTN3	0.19758
EBNA1	MAL	0.17938
EBNA1	FAM188A	0.39941
EBNA1	ZP3	0.038258
EBNA1	BCKDK	0.0912
EBNA1	MCF2	0.075039
EBNA1	UMOD	0.061239
EBNA1	C6	0.15106
EBNA1	HDHD3	0.18304
EBNA1	IPO8	0.15008
EBNA1	RNF43	0.073347
EBNA1	STX2	0.10376
EBNA1	RHBDL3	0.13671
EBNA1	AP1B1	0.19406
EBNA1	CCNT1	0.046322
EBNA1	GAPDH	0.11248
EBNA1	GRB10	0.08868
EBNA1	NBN	0.034956
EBNA1	RCN3	0.45238
EBNA1	MGST1	0.060088
EBNA1	RNF5	0.099131
EBNA1	PLSCR1	0.18944
EBNA1	CETN3	0.074947
EBNA1	CTNNBL1	0.16403
EBNA1	KDM2B	0.089149
EBNA1	FDXR	0.11334
EBNA1	FLT1	0.025677
EBNA1	BCLAF1	0.1143
EBNA1	EFTUD2	0.11212
EBNA1	SERPINE2	0.033954
EBNA1	NF1	0.02532
EBNA1	F8	0.15267
EBNA1	FBXO45	0.16858
EBNA1	KIF21A	0.15112
EBNA1	SCAMP2	0.10351
EBNA1	PCDH10	0.038413
EBNA1	GIT1	0.10831
EBNA1	GRIN2B	0.012931
EBNA1	SLC6A4	0.08533
EBNA1	CDH19	0.03731
EBNA1	VEGFC	0.086611
EBNA1	CLK2	0.086145
EBNA1	SLC12A3	0.16751
EBNA1	SNAP25	0.047368
EBNA1	DDX23	0.098547
EBNA1	LSM10	0.14397
EBNA1	SUPV3L1	0.058147
EBNA1	ATN1	0.12409
EBNA1	LRPPRC	0.12947
EBNA1	VAPB	0.049101
EBNA1	NHEJ1	0.13755
EBNA1	STAT5B	0.022981
EBNA1	COPRS	0.14897
EBNA1	RPA1	0.044911
EBNA1	HSPG2	0.073525
EBNA1	CELSR2	0.014262
EBNA1	SMARCA4	0.050305
EBNA1	ATG5	0.13621

EBNA1	ACTL6A	0.086023
EBNA1	PAXIP1	0.12318
EBNA1	EGFR	0.014162
EBNA1	ACTR8	0.1608
EBNA1	CAMLG	0.18651
EBNA1	SELP	0.063976
EBNA1	FBXO5	0.035453
EBNA1	PTGDR	0.17206
EBNA1	APAF1	0.0929
EBNA1	CTCF	0.015265
EBNA1	HSPD1	0.026213
EBNA1	PPFIA2	0.20276
EBNA1	PCDHGA8	0.038413
EBNA1	MST1R	0.096641
EBNA1	SCIN	0.028558
EBNA1	CCDC33	0.22232
EBNA1	NPC1	0.074283
EBNA1	SYTL5	0.16638
EBNA1	HINFP	0.018443
EBNA1	MORC4	0.15296
EBNA1	RAB11A	0.090751
EBNA1	LTBP1	0.41075
EBNA1	KIF5A	0.11577
EBNA1	PEX6	0.14867
EBNA1	SMARCC1	0.10087
EBNA1	TPD52L2	0.22099
EBNA1	ASPSCR1	0.14463
EBNA1	F2	0.11898
EBNA1	CELF1	0.14019
EBNA1	RHOXF2	0.054094
EBNA1	SYT7	0.094752
EBNA1	FAAP20	0.13641
EBNA1	DLG1	0.010269
EBNA1	PLEKHA2	0.082598
EBNA1	EREG	0.066864
EBNA1	IFIT2	0.16239
EBNA1	HEATR1	0.12439
EBNA1	SCNN1B	0.1439
EBNA1	SMYD2	0.12855
EBNA1	ITPR1	0.063124
EBNA1	VAMP3	0.13685
EBNA1	WIPI2	0.12944
EBNA1	CDH9	0.034636
EBNA1	SPAST	0.074577
EBNA1	PCDHB9	0.035257
EBNA1	RRAS	0.096468
EBNA1	FST	0.098644
EBNA1	CEP290	0.091662
EBNA1	KDM5B	0.023877
EBNA1	NOXO1	0.10799
EBNA1	SYT1	0.1534
EBNA1	NSMCE2	0.099768
EBNA1	RND2	0.17235
EBNA1	HMOX2	0.10263
EBNA1	WRNIP1	0.11369
EBNA1	CBL	0.13361
EBNA1	NEK3	0.09422
EBNA1	CORO1A	0.078006
EBNA1	PHF12	0.099684
EBNA1	SLX4	0.15002
EBNA1	ANKRD27	0.1795
EBNA1	EPHA2	0.044979
EBNA1	ARFRP1	0.10901
EBNA1	UCHL5	0.079714
EBNA1	PRPF8	0.17205
EBNA1	VIM	0.028962
EBNA1	NOA1	0.10794
EBNA1	SLC25A13	0.035815

EBNA1	KLRC2	0.17974
EBNA1	GDF2	0.0732
EBNA1	NAP1L4	0.13157
EBNA1	NSUN5P1	0.027218
EBNA1	MYCBP	0.13995
EBNA1	PI4K2A	0.14162
EBNA1	GRB2	0.012679
EBNA1	ADNP2	0.018591
EBNA1	NCS1	0.29425
EBNA1	TCAP	0.12754
EBNA1	CDK2	0.016321
EBNA1	MBL2	0.099087
EBNA1	NCK2	0.10364
EBNA1	IKZF3	0.022037
EBNA1	NMNAT1	0.15255
EBNA1	GPKOW	0.16941
EBNA1	FBLN5	0.3377
EBNA1	MPP6	0.20279
EBNA1	ARHGAP1	0.098416
EBNA1	CRBN	0.13687
EBNA1	HSCB	0.18213
EBNA1	BIRC5	0.033811
EBNA1	DAG1	0.097683
EBNA1	MCM3	0.05103
EBNA1	TOMM20	0.08977
EBNA1	OTOF	0.046948
EBNA1	AGTRAP	0.20647
EBNA1	ID2	0.042278
EBNA1	DOC2A	0.20088
EBNA1	MED30	0.14642
EBNA1	RHOBTB3	0.16324
EBNA1	XRCC3	0.12883
EBNA1	F11	0.17317
EBNA1	SRGAP2	0.091799
EBNA1	CALCOCO1	0.094417
EBNA1	FBXO6	0.17268
EBNA1	VPS35	0.13287
EBNA1	ADAM22	0.10275
EBNA1	PCDHA12	0.038413
EBNA1	TBC1D9B	0.017914
EBNA1	PTPRT	0.021719
EBNA1	SIGLEC8	0.185
EBNA1	SNRNP40	0.1451
EBNA1	ARID1A	0.077944
EBNA1	SCNN1D	0.18794
EBNA1	STON2	0.16408
EBNA1	CENPE	0.059252
EBNA1	HSPA8	0.017603
EBNA1	PLK2	0.060951
EBNA1	FBXW11	0.10674
EBNA1	SCAI	0.14006
EBNA1	NIPBL	0.081761
EBNA1	RPL7A	0.16733
EBNA1	KMT2A	0.039959
EBNA1	UBE2C	0.077206
EBNA1	SERPINA3	0.13191
EBNA1	ADRBK1	0.024664
EBNA1	IL7R	0.098598
EBNA1	MYL5	0.03726
EBNA1	DYX1C1	0.16097
EBNA1	BMF	0.10459
EBNA1	INTS2	0.15345
EBNA1	RAB22A	0.10516
EBNA1	MYO7A	0.074182
EBNA1	PIAS2	0.064126
EBNA1	MUC2	0.202
EBNA1	SPTLC1	0.09965
EBNA1	NLK	0.036564

EBNA1	ITGA6	0.13976
EBNA1	PILRB	0.19045
EBNA1	CTNNA2	0.070727
EBNA1	DEPTOR	0.18544
EBNA1	FTH1	0.12435
EBNA1	NOD1	0.036945
EBNA1	RCAN1	0.038642
EBNA1	DGKB	0.061667
EBNA1	ZFYVE9	0.2002
EBNA1	PDS5A	0.16796
EBNA1	TIGIT	0.13418
EBNA1	RNF8	0.062163
EBNA1	NPHS1	0.11796
EBNA1	NECAB3	0.33728
EBNA1	AKAP8L	0.13802
EBNA1	SNX12	0.22653
EBNA1	ATM	0.040382
EBNA1	TCHH	0.028419
EBNA1	RASSF9	0.1718
EBNA1	GORASP2	0.15485
EBNA1	KIR2DL4	0.17431
EBNA1	MLC1	0.14578
EBNA1	LBP	0.064138
EBNA1	TRIM16	0.088401
EBNA1	IFI35	0.18689
EBNA1	PXMP4	0.19084
EBNA1	ASGR2	0.15996
EBNA1	STAG1	0.13127
EBNA1	EFNA4	0.24124
EBNA1	DYNC1H1	0.10835
EBNA1	GRM7	0.016976
EBNA1	CNTNAP1	0.084212
EBNA1	RRAGD	0.13658
EBNA1	TRMT61B	0.2479
EBNA1	CCR1	0.085531
EBNA1	SDC4	0.12204
EBNA1	LONP2	0.10929
EBNA1	HSPA14	0.15818
EBNA1	PLXNB2	0.14134
EBNA1	APH1B	0.16277
EBNA1	SNX6	0.21294
EBNA1	APOA5	0.063403
EBNA1	KRT1	0.10589
EBNA1	ZNF143	0.084488
EBNA1	BRCA1	0.015254
EBNA1	RFX1	0.13875
EBNA1	CDK20	0.042168
EBNA1	KRT81	0.15179
EBNA1	RALB	0.098346
EBNA1	CDC73	0.093267
EBNA1	GSE1	0.22232
EBNA1	NCOA1	0.063416
EBNA1	FGA	0.13982
EBNA1	HLTF	0.049592
EBNA1	DUSP7	0.014411
EBNA1	PDE4A	0.071665
EBNA1	ZFYVE1	0.10439
EBNA1	NMI	0.17017
EBNA1	CDK5RAP3	0.087936
EBNA1	PCDHGB6	0.038413
EBNA1	WWTR1	0.078904
EBNA1	PCDHB11	0.033158
EBNA1	ASF1A	0.10342
EBNA1	ERRFI1	0.035142
EBNA1	IRF7	0.010439
EBNA1	MAPK9	0.049904
EBNA1	BBS2	0.10058
EBNA1	APOA2	0.027687

EBNA1	XPA	0.090056
EBNA1	CHMP5	0.19432
EBNA1	EIF2S2	0.22395
EBNA1	ZFYVE16	0.060949
EBNA1	NMRK2	0.21543
EBNA1	PHB2	0.11693
EBNA1	DCP1B	0.09621
EBNA1	MYB	0.034703
EBNA1	SNRPD1	0.13753
EBNA1	SOCS1	0.10747
EBNA1	C1S	0.073362
EBNA1	NPBWR1	0.14599
EBNA1	SNCB	0.018398
EBNA1	EGLN3	0.10811
EBNA1	NPC1L1	0.22553
EBNA1	PCGF2	0.028122
EBNA1	DNMBP	0.15125
EBNA1	PRAM1	0.24393
EBNA1	EFNB1	0.14396
EBNA1	NT5C3B	0.15707
EBNA1	CDYL2	0.1446
EBNA1	AASDHPPT	0.17576
EBNA1	FHL1	0.096593
EBNA1	GGA3	0.14405
EBNA1	UTP11L	0.14289
EBNA1	GLRA1	0.054822
EBNA1	AQP5	0.1651
EBNA1	ARL4C	0.087265
EBNA1	NR0B2	0.049352
EBNA1	ARHGAP21	0.14451
EBNA1	ARL3	0.031786
EBNA1	MEGF6	0.44062
EBNA1	RPS6KA1	0.019816
EBNA1	EIF2S3	0.15978
EBNA1	CALR	0.041195
EBNA1	JPH2	0.12042
EBNA1	BTBD1	0.18928
EBNA1	SOCS5	0.18115
EBNA1	EMX2	0.021636
EBNA1	MRPL11	0.17824
EBNA1	EGR1	0.019937
EBNA1	MC2R	0.11235
EBNA1	NUP205	0.14284
EBNA1	RAB33A	0.12559
EBNA1	DHX9	0.075885
EBNA1	HOXA10	0.036375
EBNA1	HSP90AA1	0.089481
EBNA1	TAF1	0.036121
EBNA1	TSPY1	0.11657
EBNA1	TNFRSF14	0.13191
EBNA1	LYL1	0.1277
EBNA1	NFKBIL1	0.16059
EBNA1	CBX5	0.075444
EBNA1	F12	0.13827
EBNA1	NRBP1	0.055271
EBNA1	SLC25A12	0.021398
EBNA1	ATF4	0.026467
EBNA1	PCDHA1	0.035121
EBNA1	ANK1	0.034119
EBNA1	PCBP1	0.17098
EBNA1	HSF1	0.040137
EBNA1	UNC5B	0.14989
EBNA1	SCRIB	0.095949
EBNA1	RPTOR	0.051404
EBNA1	SRRT	0.11979
EBNA1	SSTR3	0.10868
EBNA1	DGKG	0.058029
EBNA1	KHDRBS2	0.19278

EBNA1	MMP9	0.030687
EBNA1	PLXNB3	0.1599
EBNA1	KCNIP2	0.20661
EBNA1	TBCD	0.13787
EBNA1	THEG	0.16876
EBNA1	SLPI	0.16066
EBNA1	PDLIM4	0.17587
EBNA1	DAO	0.10003
EBNA1	PCDHA13	0.038413
EBNA1	KIF3B	0.095417
EBNA1	PRSS8	0.20037
EBNA1	FSCN1	0.15383
EBNA1	TRAF1	0.13232
EBNA1	CALML3	0.044917
EBNA1	RAMP3	0.11547
EBNA1	AGO4	0.16395
EBNA1	CRK	0.11658
EBNA1	RFWD2	0.094569
EBNA1	SPOCK3	0.042706
EBNA1	PIAS3	0.072266
EBNA1	CDH4	0.023572
EBNA1	TNS1	0.17453
EBNA1	ITGA2B	0.11286
EBNA1	PAFAH1B1	0.018715
EBNA1	MAPK13	0.092597
EBNA1	ADAM12	0.056009
EBNA1	UBA52	0.019435
EBNA1	KCNIP3	0.014667
EBNA1	SNRPN	0.18634
EBNA1	CDK14	0.037191
EBNA1	PARD6A	0.082785
EBNA1	SMARCA2	0.037534
EBNA1	PLCG1	0.076993
EBNA1	HEATR5B	0.010923
EBNA1	FAM109B	0.15336
EBNA1	ITGAX	0.15044
EBNA1	PDCD4	0.19613
EBNA1	PPP1R8	0.13466
EBNA1	NOTCH1	0.011348
EBNA1	PCLO	0.02944
EBNA1	IL8	0.095856
EBNA1	FBN2	0.048528
EBNA1	ZNHIT1	0.19362
EBNA1	TRAF4	0.03227
EBNA1	EYA2	0.020743
EBNA1	ERCC1	0.03314
EBNA1	PSME3	0.067005
EBNA1	ELOVL2	0.13167
EBNA1	EFCAB10	0.042183
EBNA1	CD93	0.30138
EBNA1	SRR	0.029909
EBNA1	EEF1B2	0.15438
EBNA1	TEX11	0.1638
EBNA1	LMX1B	0.031516
EBNA1	NLRC5	0.089519
EBNA1	DRD1	0.016153
EBNA1	RITA1	0.14132
EBNA1	GRIN1	0.13414
EBNA1	CST3	0.052965
EBNA1	FAN1	0.12704
EBNA1	CLINT1	0.24633
EBNA1	EIF2AK3	0.036524
EBNA1	ZBTB21	0.10154
EBNA1	MARK4	0.082679
EBNA1	DHH	0.28013
EBNA1	SLC25A4	0.080617
EBNA1	SPATA21	0.044917
EBNA1	NCOA2	0.031699

EBNA1	SIRT2	0.02708
EBNA1	IMMT	0.17465
EBNA1	UBE2L3	0.051772
EBNA1	IL5	0.1347
EBNA1	RRM1	0.12559
EBNA1	ILK	0.013952
EBNA1	FLT4	0.053854
EBNA1	MLF1	0.12854
EBNA1	STRN3	0.054746
EBNA1	RAP1B	0.078618
EBNA1	IKBKG	0.046194
EBNA1	SPHK2	0.18221
EBNA1	MARK3	0.10759
EBNA1	NR2F6	0.027724
EBNA1	VIPAS39	0.16811
EBNA1	ADAM15	0.10824
EBNA1	PRKCB	0.031688
EBNA1	PLD3	0.074995
EBNA1	PCDHGA10	0.038413
EBNA1	ADAM8	0.067665
EBNA1	TRIP12	0.15648
EBNA1	CSTF1	0.2018
EBNA1	U2AF2	0.16698
EBNA1	MAP3K8	0.059564
EBNA1	NOSTRIN	0.21574
EBNA1	ELMO1	0.081026
EBNA1	U2SURP	0.22291
EBNA1	AICDA	0.05624
EBNA1	RPS9	0.095047
EBNA1	DCHS2	0.038413
EBNA1	SNX18	0.17959
EBNA1	N4BP2L1	0.011504
EBNA1	CDC42EP1	0.14514
EBNA1	ADRB1	0.034692
EBNA1	SIRT7	0.091563
EBNA1	AP1G2	0.17734
EBNA1	PREX1	0.036591
EBNA1	ATP2C2	0.10467
EBNA1	PCDHGC3	0.037277
EBNA1	PDCD10	0.13249
EBNA1	USP44	0.12268
EBNA1	GFI1B	0.038108
EBNA1	CCBE1	0.030134
EBNA1	BLMH	0.12145
EBNA1	IRAK1	0.033918
EBNA1	CXCR4	0.060151
EBNA1	USP37	0.065853
EBNA1	ARFIP2	0.14827
EBNA1	SAC3D1	0.16969
EBNA1	MTMR9	0.18397
EBNA1	COL4A2	0.13977
EBNA1	PKD1L2	0.040498
EBNA1	CHMP1A	0.092537
EBNA1	PPM1G	0.063364
EBNA1	XPO1	0.10336
EBNA1	LBR	0.13741
EBNA1	ATP8B1	0.13404
EBNA1	TJP2	0.16023
EBNA1	NR1I2	0.032098
EBNA1	ITM2A	0.20909
EBNA1	RBBP4	0.074389
EBNA1	YWHAQ	0.18384
EBNA1	HOOK1	0.11443
EBNA1	INPP5K	0.026221
EBNA1	PPP1R3C	0.12325
EBNA1	ATP10A	0.083701
EBNA1	KLK5	0.21388
EBNA1	MARCH5	0.053681

EBNA1	STT3A	0.18743
EBNA1	ZBTB24	0.10783
EBNA1	TCF12	0.025968
EBNA1	SPC24	0.13517
EBNA1	NCF2	0.038553
EBNA1	CCL3	0.031115
EBNA1	MYL6B	0.35996
EBNA1	CNIH2	0.11344
EBNA1	GLI1	0.028425
EBNA1	GIPC1	0.068677
EBNA1	RAD51C	0.099973
EBNA1	NAA38	0.16937
EBNA1	IGF2BP2	0.14266
EBNA1	YWHAB	0.067898
EBNA1	SLC25A5	0.14129
EBNA1	GNA13	0.058261
EBNA1	BLOC1S5	0.16692
EBNA1	TRIM21	0.079218
EBNA1	TAF15	0.14696
EBNA1	RBX1	0.072511
EBNA1	TNNI3K	0.06536
EBNA1	WDR82	0.15625
EBNA1	RAB3A	0.050035
EBNA1	NSL1	0.12461
EBNA1	RHOH	0.14207
EBNA1	ATP6V0A1	0.16801
EBNA1	BRPF3	0.13683
EBNA1	GTF2I	0.050937
EBNA1	CPSF3	0.11984
EBNA1	KCNMA1	0.078841
EBNA1	ORC6	0.12412
EBNA1	CELSR1	0.020635
EBNA1	UBQLN2	0.17347
EBNA1	PLRG1	0.16085
EBNA1	CUL1	0.10279
EBNA1	CD9	0.11777
EBNA1	DHX58	0.081079
EBNA1	MYO9A	0.10403
EBNA1	SP3	0.045487
EBNA1	POLI	0.080372
EBNA1	SLC24A1	0.18271
EBNA1	DR1	0.084663
EBNA1	TBK1	0.04626
EBNA1	HSPA9	0.098328
EBNA1	PLSCR2	0.039001
EBNA1	GSTP1	0.075075
EBNA1	MMP14	0.064091
EBNA1	CDC5L	0.15879
EBNA1	MBNL1	0.12709
EBNA1	MED4	0.12682
EBNA1	RHBDD1	0.12316
EBNA1	TIE1	0.12193
EBNA1	FABP5	0.18432
EBNA1	AP1G1	0.15455
EBNA1	HMCN2	0.06882
EBNA1	CDHR2	0.37615
EBNA1	FAS	0.027538
EBNA1	TUBB2B	0.027922
EBNA1	WAS	0.12268
EBNA1	ALDOC	0.084979
EBNA1	TFG	0.16728
EBNA1	SNRPB2	0.14664
EBNA1	TNNI3	0.053705
EBNA1	ILF3	0.10279
EBNA1	WDR61	0.12178
EBNA1	SULT2B1	0.11882
EBNA1	PAWR	0.10338
EBNA1	ASIC2	0.16413

EBNA1	CBX3	0.0791
EBNA1	RINT1	0.16353
EBNA1	SHANK3	0.075934
EBNA1	IKBIP	0.17342
EBNA1	RCVRN	0.064872
EBNA1	AGFG1	0.14892
EBNA1	SNX17	0.16279
EBNA1	CDH5	0.26601
EBNA1	DDX58	0.044102
EBNA1	ARID1B	0.096544
EBNA1	IL6R	0.085671
EBNA1	HS3ST5	0.1456
EBNA1	TP53INP2	0.17365
EBNA1	ZNF165	0.032819
EBNA1	SEMA6A	0.12936
EBNA1	ZMYND10	0.17058
EBNA1	COTL1	0.17188
EBNA1	SMN1	0.18748
EBNA1	SNIP1	0.12535
EBNA1	SYT2	0.30973
EBNA1	CAPNS1	0.29546
EBNA1	GTF2E2	0.12988
EBNA1	KCNG3	0.14557
EBNA1	TRIM41	0.11134
EBNA1	MPZL3	0.19024
EBNA1	GABARAPL1	0.21336
EBNA1	GTF2B	0.086371
EBNA1	ANGPTL4	0.10563
EBNA1	WDR16	0.17034
EBNA1	IFIT1	0.17879
EBNA1	PCDHB15	0.036102
EBNA1	RAD18	0.067212
EBNA1	OCM2	0.044917
EBNA1	SIPA1	0.1046
EBNA1	TPD52	0.39603
EBNA1	MC5R	0.14493
EBNA1	RIPK4	0.098338
EBNA1	POLDIP3	0.19498
EBNA1	IRS2	0.02055
EBNA1	ARID5B	0.076657
EBNA1	NUFIP1	0.13153
EBNA1	KLC1	0.17228
EBNA1	FRMPD4	0.22597
EBNA1	LIG4	0.031195
EBNA1	KLHDC3	0.18647
EBNA1	XRCC5	0.030665
EBNA2	ubl4ab	0.06139
EBNA2	SPTA1	0.042956
EBNA2	S100A7	0.07621
EBNA2	HSPA5	0.041398
EBNA2	LDLR	0.032857
EBNA2	RABEP1	0.012527
EBNA2	C12orf61	0.059987
EBNA2	PLSCR3	0.10505
EBNA2	YMR013W-A	0.021405
EBNA2	ATP5B	0.058602
EBNA2	NIN	0.087158
EBNA2	KCNIP1	0.089982
EBNA2	STIM2	0.10605
EBNA2	WIPF1	0.023479
EBNA2	SPTAN1	0.06261
EBNA2	MASP1	0.15419
EBNA2	SCUBE3	0.131
EBNA2	PROC	0.1291
EBNA2	PDCD6	0.15047
EBNA2	S100A9	0.033268
EBNA2	ANXA2	0.065258
EBNA2	MMP20	0.02474

EBNA2	MATN4	0.134
EBNA2	NOTCH3	0.025671
EBNA2	RNH1	0.10928
EBNA2	CLIP1	0.051054
EBNA2	AMOT	0.015903
EBNA2	FAM83F	0.019793
EBNA2	THBD	0.078967
EBNA2	EML1	0.12609
EBNA2	EPS15	0.044083
EBNA2	PCSK2	0.023487
EBNA2	AP3S1	0.041963
EBNA2	SDF4	0.095196
EBNA2	SPACA3	0.037524
EBNA2	STAT1	0.010731
EBNA2	FAT4	0.12171
EBNA2	DTNBP1	0.011422
EBNA2	PPEF1	0.053957
EBNA2	CXorf67	0.064208
EBNA2	FAM188B2	0.060782
EBNA2	THBS1	0.013763
EBNA2	SYT11	0.17079
EBNA2	HSP90B1	0.068488
EBNA2	MACF1	0.085121
EBNA2	CCDC176	0.078105
EBNA2	GCH1	0.028825
EBNA2	NKD2	0.14086
EBNA2	FAT1	0.061992
EBNA2	S100A11	0.099965
EBNA2	CCDC137	0.033151
EBNA2	NKD1	0.096167
EBNA2	PPP2R3B	0.050319
EBNA2	ACTN4	0.053879
EBNA2	EHD1	0.034018
EBNA2	S100A13	0.10908
EBNA2	MELK	0.026428
EBNA2	S100A1	0.063417
EBNA2	UTRN	0.041318
EBNA2	SLC34A1	0.030236
EBNA2	CALB1	0.096842
EBNA2	NDEL1	0.015387
EBNA2	MASP2	0.13994
EBNA2	PARK7	0.0272
EBNA2	ANXA2R	0.044038
EBNA2	EFEMP1	0.12059
EBNA2	SPARC	0.057466
EBNA2	NOTCH4	0.023931
EBNA2	PCDH17	0.12171
EBNA2	KIAA1841	0.026684
EBNA2	REPS1	0.11069
EBNA2	LRCOL1	0.053504
EBNA2	MPTX1	0.019342
EBNA2	STIM1	0.053861
EBNA2	S100B	0.053821
EBNA2	TNNC1	0.11618
EBNA2	CETN2	0.10413
EBNA2	ANXA6	0.11786
EBNA2	ACTN2	0.043396
EBNA2	PRSS2	0.12192
EBNA2	PITPNM3	0.11266
EBNA2	VIL1	0.05906
EBNA2	KCNJ4	0.020689
EBNA2	CETN1	0.13034
EBNA2	C2CD4D	0.013706
EBNA2	VLDLR	0.05378
EBNA2	CDH1	0.024803
EBNA2	MAP1LC3B	0.016961
EBNA2	TTN	0.02238
EBNA2	EGF	0.03935

EBNA2	LTBP4	0.099981
EBNA2	PLSCR4	0.10158
EBNA2	SNX9	0.058744
EBNA2	RYR2	0.022661
EBNA2	PRF1	0.047335
EBNA2	IL6ST	0.062043
EBNA2	MYO1E	0.025847
EBNA2	S100A8	0.048819
EBNA2	NID2	0.13523
EBNA2	NELL1	0.064457
EBNA2	ytzL	0.068944
EBNA2	WDR96	0.018729
EBNA2	DNER	0.076235
EBNA2	PEF1	0.13326
EBNA2	C10orf82	0.019651
EBNA2	CHP1	0.05475
EBNA2	FASN	0.045018
EBNA2	GPR98	0.045352
EBNA2	MICU1	0.10627
EBNA2	CALU	0.10431
EBNA2	PPP3CA	0.018803
EBNA2	BEND2	0.01224
EBNA2	LRP2	0.049907
EBNA2	FAM188B	0.061566
EBNA2	NUCB2	0.084927
EBNA2	S100A6	0.083439
EBNA2	LRP8	0.097624
EBNA2	CT55	0.04386
EBNA2	S100P	0.07585
EBNA2	RAB11FIP3	0.032225
EBNA2	EFCAB4B	0.08939
EBNA2	DTNA	0.056072
EBNA2	REPS2	0.13862
EBNA2	PPEF2	0.054154
EBNA2	ATP2A1	0.037646
EBNA2	CAGE1	0.014297
EBNA2	PKD2L1	0.068164
EBNA2	EHD3	0.044478
EBNA2	THBS2	0.10653
EBNA2	CALM1	0.061662
EBNA2	S100A4	0.1323
EBNA2	S100A12	0.067486
EBNA2	RCN1	0.13723
EBNA2	DPP4	0.011935
EBNA2	ACTN3	0.12769
EBNA2	ATP5F1	0.0155
EBNA2	FBN1	0.12855
EBNA2	F10	0.057427
EBNA2	CAPN1	0.040077
EBNA2	CELSR3	0.06966
EBNA2	SIRT4	0.037422
EBNA2	TESC	0.077287
EBNA2	LSM12	0.015991
EBNA2	C12orf57	0.027224
EBNA2	RPH3A	0.053937
EBNA2	LRP4	0.069243
EBNA2	SLIT2	0.026725
EBNA2	MATN2	0.10958
EBNA2	CIB1	0.094105
EBNA2	PPP2R3A	0.076975
EBNA2	DLL1	0.11079
EBNA2	ACTN1	0.060664
EBNA2	EHD4	0.01982
EBNA2	PRSS3	0.14293
EBNA2	MYL2	0.13989
EBNA2	EDEM1	0.06118
EBNA2	LRP1	0.028843
EBNA2	SNX19	0.076104

EBNA2	PDGFRB	0.016178
EBNA2	ITSN1	0.018416
EBNA2	DST	0.070265
EBNA2	MYL6	0.12513
EBNA2	COMP	0.093442
EBNA2	CCDC47	0.10513
EBNA2	ANXA7	0.05063
EBNA2	CHP2	0.099028
EBNA2	CDK5R1	0.028035
EBNA2	NECAB2	0.13812
EBNA2	CANX	0.058632
EBNA2	MMP15	0.013507
EBNA2	ANXA11	0.057016
EBNA2	PPP3CB	0.021749
EBNA2	IQGAP1	0.01133
EBNA2	MATN3	0.14429
EBNA2	FAM47C	0.035695
EBNA2	SH3KBP1	0.011256
EBNA2	RET	0.04874
EBNA2	CDH23	0.036584
EBNA2	RAB11FIP4	0.17904
EBNA2	CNRIP1	0.011118
EBNA2	C1orf64	0.037365
EBNA2	AC3.5	0.022119
EBNA2	C2orf81	0.017973
EBNA2	FAM84A	0.010286
EBNA2	CLIC4	0.046444
EBNA2	FAM49B	0.027957
EBNA2	BNIP2	0.050112
EBNA2	JAG2	0.08451
EBNA2	CADPS	0.15131
EBNA2	CCAR1	0.10848
EBNA2	C1orf195	0.079937
EBNA2	CRELD2	0.12683
EBNA2	YBR056C-B	0.016554
EBNA2	GSN	0.052378
EBNA2	SLC11A2	0.018849
EBNA2	CABP1	0.014035
EBNA2	LETM1	0.12242
EBNA2	FAM231A	0.11867
EBNA2	FAM124A	0.016981
EBNA2	FAM188A	0.096208
EBNA2	AP1B1	0.018893
EBNA2	RCN3	0.13345
EBNA2	PLSCR1	0.055875
EBNA2	SLC6A4	0.010582
EBNA2	NKAPL	0.045179
EBNA2	TLDC2	0.025255
EBNA2	LTBP1	0.13304
EBNA2	F2	0.030659
EBNA2	SYT1	0.047392
EBNA2	NSUN5P1	0.038075
EBNA2	NCS1	0.11983
EBNA2	DAG1	0.028418
EBNA2	NECAB3	0.07679
EBNA2	SNX6	0.022844
EBNA2	FAM92A1	0.011411
EBNA2	NPC1L1	0.032358
EBNA2	MEGF6	0.13168
EBNA2	CALR	0.014192
EBNA2	KCNIP2	0.043226
EBNA2	HEATR5B	0.037935
EBNA2	CD93	0.088502
EBNA2	GRIN1	0.043254
EBNA2	DHH	0.093515
EBNA2	SPHK2	0.013842
EBNA2	N4BP2L1	0.016646
EBNA2	FAM231D	0.042942

EBNA2	C11orf72	0.016641
EBNA2	MYL6B	0.10297
EBNA2	ATP6V0A1	0.014003
EBNA2	AP1G1	0.018311
EBNA2	CDHR2	0.1117
EBNA2	SNX17	0.010507
EBNA2	CDH5	0.070753
EBNA2	IL6R	0.021414
EBNA2	SYT2	0.11754
EBNA2	CAPNS1	0.02037
EBNA2	TPD52	0.13427
EBNA3	HPS4	0.30022
EBNA3	CBX7	0.27987
EBNA3	ACR	0.2553
EBNA3	ACVR1	0.047342
EBNA3	TAF6	0.094729
EBNA3	GPAA1	0.21819
EBNA3	TRIB3	0.099692
EBNA3	HDAC6	0.018922
EBNA3	HERPUD1	0.11464
EBNA3	ubl4ab	0.11268
EBNA3	FPR1	0.25209
EBNA3	BRAP	0.2543
EBNA3	SERPINB9	0.24586
EBNA3	SLC25A25	0.18081
EBNA3	MACROD1	0.36948
EBNA3	ITGB5	0.21751
EBNA3	HID1	0.29723
EBNA3	ECT2	0.061518
EBNA3	SHQ1	0.32879
EBNA3	ATPIF1	0.098989
EBNA3	PEX10	0.29607
EBNA3	NLRP2	0.28542
EBNA3	RBL1	0.16427
EBNA3	BIN2	0.4246
EBNA3	BBS10	0.32078
EBNA3	USP2	0.15927
EBNA3	MED1	0.027327
EBNA3	CDC34	0.18539
EBNA3	RGCC	0.088536
EBNA3	EFHC2	0.21072
EBNA3	CDK10	0.19741
EBNA3	EPHA3	0.15607
EBNA3	EFCAB1	0.21072
EBNA3	PIK3CD	0.15281
EBNA3	RAB7A	0.20782
EBNA3	TGFBR2	0.038627
EBNA3	N4BP1	0.34403
EBNA3	CTDNEP1	0.1817
EBNA3	PANK2	0.1253
EBNA3	FAM58A	0.24915
EBNA3	PSMD4	0.17521
EBNA3	FZD8	0.13023
EBNA3	GAGE12I	0.061456
EBNA3	PAGE2B	0.17318
EBNA3	PPP5C	0.16688
EBNA3	SLC2A4	0.18494
EBNA3	KEL	0.23181
EBNA3	IFT122	0.35059
EBNA3	NAE1	0.24255
EBNA3	PRPF6	0.28273
EBNA3	SPTA1	0.31868
EBNA3	SEC31A	0.21375
EBNA3	GZMA	0.29369
EBNA3	S100A7	0.40392
EBNA3	NMRK1	0.019818
EBNA3	ROCK2	0.022917
EBNA3	IL4	0.059356

EBNA3	NANOS1	0.34584
EBNA3	TRMT2A	0.044091
EBNA3	SLC26A8	0.27868
EBNA3	UBE2H	0.2619
EBNA3	HSPA5	0.2205
EBNA3	LDLR	0.17194
EBNA3	NEDD4	0.076597
EBNA3	LAMTOR5	0.27977
EBNA3	GSPT2	0.25272
EBNA3	SERPINB5	0.30748
EBNA3	SSBP1	0.34043
EBNA3	BIRC6	0.18102
EBNA3	SERTAD3	0.31066
EBNA3	INO80B	0.26735
EBNA3	KLK13	0.34461
EBNA3	AGO2	0.13983
EBNA3	HEPACAM2	0.25128
EBNA3	RNMTL1	0.14445
EBNA3	CENPW	0.27525
EBNA3	UNC13D	0.26521
EBNA3	UPF3A	0.36633
EBNA3	ARHGAP33	0.29217
EBNA3	RB1CC1	0.13357
EBNA3	RABEP1	0.387
EBNA3	SKA3	0.27943
EBNA3	VPS54	0.35529
EBNA3	KIAA1377	0.43795
EBNA3	EIF5A	0.14696
EBNA3	PDSS2	0.019552
EBNA3	CYS1	0.32953
EBNA3	LIMK1	0.13609
EBNA3	FAF1	0.17664
EBNA3	IRF9	0.090417
EBNA3	SLC5A1	0.23881
EBNA3	LRP2BP	0.4246
EBNA3	MAPK8	0.095785
EBNA3	EDF1	0.148
EBNA3	KCNS1	0.26044
EBNA3	PXN	0.1582
EBNA3	NUP85	0.1603
EBNA3	SOX13	0.18859
EBNA3	CTNND2	0.24169
EBNA3	UBE2D3	0.1437
EBNA3	C12orf61	0.1062
EBNA3	KRT5	0.26805
EBNA3	FAM86B2	0.017188
EBNA3	PNMA6D	0.018621
EBNA3	HSD17B8	0.25694
EBNA3	BTNL8	0.41166
EBNA3	PLSCR3	0.56352
EBNA3	KREMEN1	0.37612
EBNA3	TCF25	0.17454
EBNA3	NR0B1	0.078513
EBNA3	SNX20	0.33335
EBNA3	PRSS16	0.14087
EBNA3	BIRC7	0.22259
EBNA3	MCM6	0.15307
EBNA3	FKBP10	0.17921
EBNA3	KRT17	0.22863
EBNA3	MAPKAPK2	0.082139
EBNA3	NOSIP	0.26078
EBNA3	SMURF2	0.14268
EBNA3	DYM	0.32121
EBNA3	ANKIB1	0.36919
EBNA3	ZC3HAV1L	0.10657
EBNA3	ATP6V0D2	0.25251
EBNA3	PTRH2	0.17177
EBNA3	ORAI3	0.41166

EBNA3	CSNK2A1	0.13078
EBNA3	PFKM	0.17366
EBNA3	BTBD16	0.022638
EBNA3	C9orf40	0.014113
EBNA3	SPRR2E	0.24184
EBNA3	ELAVL2	0.43092
EBNA3	RPUSD4	0.54675
EBNA3	CMPK2	0.13466
EBNA3	EMR1	0.1123
EBNA3	CD248	0.18454
EBNA3	CCDC159	0.0845
EBNA3	ASIC1	0.23161
EBNA3	ORC2	0.24756
EBNA3	C10orf68	0.082345
EBNA3	TAF12	0.055206
EBNA3	PEX16	0.24828
EBNA3	CALN1	0.1702
EBNA3	CCDC42	0.014674
EBNA3	HMGA2	0.015707
EBNA3	SLC4A11	0.011444
EBNA3	F9	0.18546
EBNA3	CRB1	0.19949
EBNA3	TRAPPC2	0.2017
EBNA3	NIP7	0.43748
EBNA3	C16orf70	0.3749
EBNA3	NAT16	0.14957
EBNA3	USP12	0.3084
EBNA3	DDB2	0.25157
EBNA3	TUBA1B	0.10215
EBNA3	PIP5K1A	0.15384
EBNA3	CCDC162P	0.014484
EBNA3	PEAK1	0.24634
EBNA3	SUN1	0.24478
EBNA3	SFTPD	0.30717
EBNA3	PIP5K1B	0.34702
EBNA3	FAT3	0.17465
EBNA3	TAB3	0.17097
EBNA3	GOLGA6L1	0.039423
EBNA3	SBF2	0.23955
EBNA3	SLC7A11	0.19541
EBNA3	BNIP1	0.20847
EBNA3	BRMS1	0.25927
EBNA3	FGFR1OP	0.18799
EBNA3	PCDH20	0.18006
EBNA3	DARS2	0.17253
EBNA3	PTPRH	0.12216
EBNA3	SPRR1A	0.23533
EBNA3	CALCOCO2	0.28331
EBNA3	USHBP1	0.40373
EBNA3	CD1B	0.3319
EBNA3	LALBA	0.1541
EBNA3	BRD4	0.24488
EBNA3	EFCAB5	0.21072
EBNA3	TFAP2B	0.054842
EBNA3	PRPF4B	0.20417
EBNA3	FXYD4	0.12137
EBNA3	MYF5	0.17089
EBNA3	ARHGEF12	0.14778
EBNA3	RNF2	0.14997
EBNA3	NT5C2	0.18306
EBNA3	LLGL2	0.29636
EBNA3	MYH7	0.24872
EBNA3	SORBS3	0.20239
EBNA3	POLD2	0.19975
EBNA3	SNAPIN	0.23037
EBNA3	INTS6	0.33666
EBNA3	ZDHHC8	0.09718
EBNA3	STX3	0.21166

EBNA3	PCDH12	0.1442
EBNA3	PDE2A	0.089929
EBNA3	KRI1	0.13247
EBNA3	SPATA1	0.024054
EBNA3	PLXNB1	0.16614
EBNA3	RAB13	0.01975
EBNA3	CARTPT	0.20274
EBNA3	CCDC69	0.04353
EBNA3	HMGB1	0.082634
EBNA3	KCNJ5	0.3228
EBNA3	MLKL	0.23252
EBNA3	MKS1	0.2714
EBNA3	HSPE1	0.28702
EBNA3	LYN	0.011069
EBNA3	MAPK7	0.10745
EBNA3	IGFL2	0.16873
EBNA3	USP11	0.28304
EBNA3	DTX1	0.17714
EBNA3	ADAMTS5	0.18521
EBNA3	ENDOU	0.045836
EBNA3	RANBP9	0.28126
EBNA3	SUFU	0.1118
EBNA3	SPHKAP	0.41166
EBNA3	PXMP2	0.34215
EBNA3	SYTL1	0.32188
EBNA3	SOCS7	0.30292
EBNA3	MAPK1	0.015437
EBNA3	TYRP1	0.25765
EBNA3	ZFPM1	0.065609
EBNA3	UBA1	0.24782
EBNA3	HDAC2	0.020851
EBNA3	BTBD2	0.40465
EBNA3	EIF3J	0.40527
EBNA3	RTN2	0.32963
EBNA3	RP1	0.21006
EBNA3	CENPJ	0.16766
EBNA3	BCCIP	0.34458
EBNA3	F5	0.27275
EBNA3	IGSF1	0.32492
EBNA3	KCND2	0.17108
EBNA3	EPN2	0.038201
EBNA3	PTPN1	0.095701
EBNA3	FKBP1B	0.15622
EBNA3	APLP2	0.16562
EBNA3	C10orf62	0.43795
EBNA3	TERF2	0.15364
EBNA3	RNF167	0.26031
EBNA3	UNC119B	0.36361
EBNA3	RACGAP1	0.08015
EBNA3	RBL2	0.23084
EBNA3	YMR013W-A	0.10082
EBNA3	CTBP2	0.21295
EBNA3	JAK3	0.11268
EBNA3	CCNB1IP1	0.27437
EBNA3	ACTA1	0.18846
EBNA3	PSMA1	0.11266
EBNA3	DHX30	0.36475
EBNA3	NLRX1	0.24152
EBNA3	VASP	0.2398
EBNA3	PCDHGA1	0.18006
EBNA3	TRAF6	0.017171
EBNA3	CABP7	0.1702
EBNA3	CASC3	0.24405
EBNA3	CHIC1	0.064681
EBNA3	CYP4F30P	0.014234
EBNA3	WASF2	0.25957
EBNA3	CD58	0.32992
EBNA3	DUSP1	0.064349

EBNA3	MAP2	0.22332
EBNA3	TBL1XR1	0.16705
EBNA3	ZFYVE27	0.24478
EBNA3	POP7	0.32014
EBNA3	APBB1	0.074567
EBNA3	DNM3	0.12484
EBNA3	PCNT	0.27558
EBNA3	MRPL13	0.34374
EBNA3	U2AF1	0.27386
EBNA3	ARHGDIA	0.24437
EBNA3	TFDP1	0.077338
EBNA3	PDXP	0.16867
EBNA3	BOC	0.26511
EBNA3	P4HB	0.12852
EBNA3	SNRPA	0.33617
EBNA3	GAREM	0.43795
EBNA3	ELP5	0.32235
EBNA3	ALAS2	0.21252
EBNA3	IGFBP5	0.18705
EBNA3	MAN1B1	0.091369
EBNA3	TRMT6	0.15478
EBNA3	ZNF675	0.15999
EBNA3	SLC16A2	0.022711
EBNA3	PRKCD	0.041448
EBNA3	DVL2	0.14534
EBNA3	MLH1	0.095348
EBNA3	CHRD	0.21426
EBNA3	BICD2	0.40458
EBNA3	APOB	0.039062
EBNA3	KIF7	0.29168
EBNA3	ADAM9	0.079544
EBNA3	ATP5B	0.22244
EBNA3	NIN	0.54129
EBNA3	UBB	0.048199
EBNA3	TXNDC17	0.35415
EBNA3	BGLAP	0.023571
EBNA3	ARHGEF1	0.17039
EBNA3	PYCARD	0.045392
EBNA3	MUL1	0.069828
EBNA3	ITCH	0.13234
EBNA3	CEP192	0.30533
EBNA3	SYTL2	0.21486
EBNA3	PGR	0.078609
EBNA3	CARD11	0.18682
EBNA3	LMO2	0.17028
EBNA3	KCNIP1	0.43706
EBNA3	CDC27	0.21742
EBNA3	MTO1	0.17142
EBNA3	VPS26A	0.27669
EBNA3	BATF	0.2015
EBNA3	SH3BP5	0.39784
EBNA3	ZC3HC1	0.18567
EBNA3	CDH18	0.166
EBNA3	PLCL2	0.022985
EBNA3	AKTIP	0.25316
EBNA3	MARCH8	0.037659
EBNA3	ANKEF1	0.21072
EBNA3	TNIP1	0.26902
EBNA3	TLE3	0.28371
EBNA3	STIM2	0.52538
EBNA3	KIN	0.071546
EBNA3	FUNDC1	0.27172
EBNA3	FAM109A	0.30214
EBNA3	GP1BA	0.23769
EBNA3	HUS1B	0.078944
EBNA3	XRN1	0.24432
EBNA3	WBP2	0.41358
EBNA3	CEACAM1	0.31013

EBNA3	NUP107	0.20061
EBNA3	ERBB3	0.097431
EBNA3	NEK6	0.049021
EBNA3	ARL11	0.37979
EBNA3	CABYR	0.10383
EBNA3	MEGF9	0.18833
EBNA3	CBLC	0.088565
EBNA3	SLAIN2	0.38908
EBNA3	EIF4E3	0.03066
EBNA3	SSSCA1	0.38498
EBNA3	CCDC130	0.41354
EBNA3	BNIPL	0.29624
EBNA3	KRT7	0.21413
EBNA3	ZHX2	0.11237
EBNA3	CTCFL	0.19139
EBNA3	RPLP1	0.28237
EBNA3	PSMG3	0.01001
EBNA3	DERL3	0.33031
EBNA3	KIAA1045	0.17787
EBNA3	SERGEF	0.3115
EBNA3	COG2	0.26159
EBNA3	CD247	0.24939
EBNA3	MAN1A2	0.070013
EBNA3	SLC23A2	0.054426
EBNA3	DSN1	0.26422
EBNA3	ELK1	0.12794
EBNA3	TRAF2	0.072608
EBNA3	TCEA2	0.20495
EBNA3	NKAP	0.23068
EBNA3	FIS1	0.11324
EBNA3	TMED10	0.20282
EBNA3	RALBP1	0.29249
EBNA3	NDUFAF4	0.43177
EBNA3	BUB1B	0.099862
EBNA3	CST5	0.4521
EBNA3	NFKBIB	0.19493
EBNA3	THOC5	0.28057
EBNA3	NAIP	0.28013
EBNA3	DPY30	0.24624
EBNA3	EFCAB14	0.21072
EBNA3	PVRL3	0.27926
EBNA3	LRIF1	0.3436
EBNA3	ANGEL1	0.039888
EBNA3	TNFAIP3	0.037348
EBNA3	PLCB4	0.092979
EBNA3	TNKS	0.12046
EBNA3	TMEM30B	0.39912
EBNA3	PYDC1	0.28793
EBNA3	PITPNM2	0.14421
EBNA3	SOX4	0.043615
EBNA3	C19orf44	0.047033
EBNA3	NCAPD2	0.27141
EBNA3	C15orf40	0.011532
EBNA3	MIA3	0.3004
EBNA3	NKAIN1	0.13994
EBNA3	FSIP1	0.1177
EBNA3	UHRF2	0.17979
EBNA3	CGA	0.2394
EBNA3	PDIA3	0.12497
EBNA3	BIRC3	0.20282
EBNA3	PABPN1	0.22662
EBNA3	TAF3	0.21322
EBNA3	COMMD5	0.37746
EBNA3	ACAN	0.23276
EBNA3	CLASP2	0.12652
EBNA3	NACA	0.27413
EBNA3	JAK2	0.021567
EBNA3	WIPF1	0.39604

EBNA3	SPTAN1	0.38538
EBNA3	AANAT	0.070892
EBNA3	NDE1	0.13108
EBNA3	TSGA10IP	0.098866
EBNA3	BECN1	0.20572
EBNA3	PTPN3	0.12977
EBNA3	FZD5	0.036584
EBNA3	CD81	0.216
EBNA3	CDC37	0.24077
EBNA3	TNFAIP8L3	0.031503
EBNA3	NOL8	0.37594
EBNA3	MT-ND4	0.076737
EBNA3	PPM1E	0.17323
EBNA3	SPNS1	0.35761
EBNA3	RIMS1	0.20578
EBNA3	ATRIP	0.3699
EBNA3	COPS2	0.29015
EBNA3	NDUFAB1	0.11868
EBNA3	PREP	0.065193
EBNA3	DDR1	0.13595
EBNA3	COL13A1	0.23739
EBNA3	RAD52	0.31734
EBNA3	TCF7	0.093043
EBNA3	LAMTOR1	0.22858
EBNA3	DHPS	0.38778
EBNA3	IL12A	0.13442
EBNA3	SMC3	0.15448
EBNA3	SNX24	0.12183
EBNA3	STAT5A	0.057682
EBNA3	THAP1	0.21752
EBNA3	CLP1	0.016547
EBNA3	TUBA3C	0.12669
EBNA3	CDK5RAP2	0.13305
EBNA3	PRX	0.30755
EBNA3	MCRS1	0.23975
EBNA3	POP4	0.33617
EBNA3	TARBP1	0.096116
EBNA3	FBLN1	0.16765
EBNA3	EDIL3	0.18015
EBNA3	C10orf35	0.15804
EBNA3	AIMP1	0.22889
EBNA3	MASP1	0.55866
EBNA3	TBL1X	0.10656
EBNA3	AIF1	0.051156
EBNA3	ZNF205	0.25167
EBNA3	INPP5D	0.12327
EBNA3	GCC2	0.2093
EBNA3	THRB	0.062353
EBNA3	SCUBE3	0.6847
EBNA3	CALML6	0.10976
EBNA3	KIF13B	0.19177
EBNA3	SLC9A7	0.22618
EBNA3	CNOT3	0.24939
EBNA3	PTPRM	0.1022
EBNA3	MFAP2	0.12635
EBNA3	BCL9	0.30915
EBNA3	NGFR	0.095469
EBNA3	TADA3	0.1102
EBNA3	STRAP	0.23033
EBNA3	ITPKC	0.088544
EBNA3	PROZ	0.12527
EBNA3	LSM3	0.30255
EBNA3	DDO	0.26704
EBNA3	PEA15	0.33607
EBNA3	RPS27L	0.25259
EBNA3	RBFOX1	0.36052
EBNA3	AMIGO2	0.31486
EBNA3	MTSS1	0.17299

EBNA3	PSMC3	0.10323
EBNA3	HUWE1	0.2294
EBNA3	PPP1R26	0.34028
EBNA3	UXT	0.18638
EBNA3	TRAPPC4	0.36737
EBNA3	TRIM17	0.28704
EBNA3	HECTD3	0.23917
EBNA3	TH	0.036178
EBNA3	TNFRSF10B	0.26166
EBNA3	SCAP	0.23055
EBNA3	C1QB	0.32965
EBNA3	TRIM13	0.17453
EBNA3	FBXO7	0.31197
EBNA3	HIPK1	0.11463
EBNA3	SPBC1539.02	0.059653
EBNA3	CAMK2A	0.13796
EBNA3	CEP152	0.18895
EBNA3	PTPN6	0.035599
EBNA3	APOH	0.17437
EBNA3	HEXIM1	0.27081
EBNA3	PTPRS	0.1365
EBNA3	ATXN10	0.29772
EBNA3	SCN5A	0.10494
EBNA3	GEMIN5	0.33453
EBNA3	COMMD7	0.36763
EBNA3	FHIT	0.19205
EBNA3	ADAM17	0.058705
EBNA3	CSNK1A1	0.11099
EBNA3	ATG4B	0.3258
EBNA3	TERF1	0.12079
EBNA3	MAL2	0.3544
EBNA3	EEF1A1	0.091049
EBNA3	PCDHGB1	0.18006
EBNA3	AGTR1	0.12028
EBNA3	SEC24B	0.19604
EBNA3	ALYREF	0.26259
EBNA3	KPNA1	0.22613
EBNA3	SERINC1	0.25758
EBNA3	AATK	0.23942
EBNA3	GLTSCR1L	0.16455
EBNA3	NCF4	0.25783
EBNA3	MED8	0.24332
EBNA3	ATG16L1	0.33752
EBNA3	USP19	0.20051
EBNA3	RIN3	0.37984
EBNA3	LDOC1	0.3798
EBNA3	FSBP	0.33039
EBNA3	SH3D19	0.25414
EBNA3	TNS4	0.35341
EBNA3	MORF4L2	0.28687
EBNA3	TSR2	0.42459
EBNA3	MAB21L3	0.017968
EBNA3	SAV1	0.25623
EBNA3	EPB41	0.13988
EBNA3	CDH16	0.18006
EBNA3	PDE9A	0.19327
EBNA3	PEX19	0.2274
EBNA3	SYNCRIP	0.21498
EBNA3	NAGK	0.43127
EBNA3	SLC22A6	0.26016
EBNA3	BMP7	0.037707
EBNA3	TMEM135	0.13037
EBNA3	PPAP2B	0.10183
EBNA3	RPS6	0.086684
EBNA3	TIA1	0.34267
EBNA3	SLC34A2	0.10639
EBNA3	SNRPG	0.23849
EBNA3	PRKCI	0.027539

EBNA3	TP53RK	0.27521
EBNA3	PCDH18	0.17465
EBNA3	MUC20	0.10229
EBNA3	TECPR1	0.33298
EBNA3	RRM2	0.23732
EBNA3	CD300LD	0.39913
EBNA3	KCNJ10	0.13987
EBNA3	PDP1	0.036169
EBNA3	CUL4A	0.27896
EBNA3	CD244	0.34382
EBNA3	GATA4	0.023765
EBNA3	KCNH1	0.19514
EBNA3	UFC1	0.41167
EBNA3	PAIP2B	0.39908
EBNA3	PTPRN	0.21403
EBNA3	KAT7	0.1704
EBNA3	PML	0.038658
EBNA3	RPGR	0.3018
EBNA3	KBTBD6	0.40098
EBNA3	WDR5	0.21706
EBNA3	GOLGA4	0.29121
EBNA3	MALL	0.32285
EBNA3	CHMP4B	0.32518
EBNA3	TLK2	0.21842
EBNA3	NLRC4	0.16279
EBNA3	PROC	0.48581
EBNA3	PDCD6	0.53606
EBNA3	DKC1	0.30748
EBNA3	USP32	0.162
EBNA3	OTUD7B	0.24041
EBNA3	COG1	0.30406
EBNA3	S100A9	0.18745
EBNA3	ANXA2	0.31965
EBNA3	TMPO	0.32481
EBNA3	PSMD2	0.16298
EBNA3	MMP20	0.42227
EBNA3	RAET1G	0.36338
EBNA3	DAZL	0.26725
EBNA3	NR2C2AP	0.35519
EBNA3	smf	0.028808
EBNA3	MCM4	0.17066
EBNA3	MAGEB1	0.013929
EBNA3	RTN4	0.10855
EBNA3	RUNX1	0.11095
EBNA3	TOP3A	0.22903
EBNA3	CDKN1A	0.083996
EBNA3	KIF23	0.18441
EBNA3	IL13RA1	0.37585
EBNA3	EZH2	0.19025
EBNA3	MATN4	0.72271
EBNA3	SLX4IP	0.43795
EBNA3	POLG	0.27201
EBNA3	MGST3	0.029113
EBNA3	NOLC1	0.056238
EBNA3	PSMF1	0.16212
EBNA3	FMR1	0.39656
EBNA3	MAP1A	0.19028
EBNA3	NOTCH3	0.26852
EBNA3	DNMT3L	0.24457
EBNA3	VPS72	0.28835
EBNA3	TIMM50	0.16
EBNA3	CXXC1	0.21193
EBNA3	PVALB	0.20324
EBNA3	C21orf67	0.15263
EBNA3	RNH1	0.67438
EBNA3	LRRC47	0.40246
EBNA3	GK5	0.042514
EBNA3	UBAP2	0.12822

EBNA3	FOXRED2	0.44521
EBNA3	PKM	0.15627
EBNA3	ARHGEF6	0.17398
EBNA3	PIK3C2B	0.25758
EBNA3	MFF	0.19412
EBNA3	ATF2	0.025013
EBNA3	MMRN2	0.3892
EBNA3	FBXL12	0.39911
EBNA3	THOC7	0.37471
EBNA3	EGFL7	0.1535
EBNA3	POR	0.056506
EBNA3	ASPN	0.19505
EBNA3	C17orf105	0.051707
EBNA3	MYL1	0.15678
EBNA3	FAM110D	0.11234
EBNA3	ARL4D	0.23606
EBNA3	NUMA1	0.1458
EBNA3	MED23	0.23649
EBNA3	ADIPOQ	0.032707
EBNA3	TBC1D24	0.31766
EBNA3	HLA-DRB1	0.090646
EBNA3	MED22	0.25511
EBNA3	TGFB3	0.055077
EBNA3	KIR2DL3	0.28848
EBNA3	EGLN1	0.17492
EBNA3	TRDMT1	0.012881
EBNA3	PLXNC1	0.26512
EBNA3	SIAH1	0.13283
EBNA3	FSCB	0.17987
EBNA3	C2CD5	0.18926
EBNA3	SNX27	0.27435
EBNA3	PTPRJ	0.061561
EBNA3	NDUFA9	0.25628
EBNA3	PRKCA	0.014944
EBNA3	PVRL4	0.31879
EBNA3	PPP6R1	0.3435
EBNA3	FRS2	0.1681
EBNA3	SLC13A4	0.026732
EBNA3	MEF2BNB	0.43347
EBNA3	SPSB2	0.37009
EBNA3	ROBO1	0.14495
EBNA3	PAIP1	0.33434
EBNA3	WRAP53	0.42922
EBNA3	MORF4L1	0.25613
EBNA3	SELPLG	0.25978
EBNA3	CLIP1	0.28781
EBNA3	ATP1A1	0.1296
EBNA3	BBS9	0.30985
EBNA3	YTHDF3	0.43795
EBNA3	HCK	0.087025
EBNA3	USP6	0.22112
EBNA3	TEK	0.065024
EBNA3	TAP2	0.10371
EBNA3	UGP2	0.2086
EBNA3	EXOSC5	0.26521
EBNA3	UBXN7	0.33629
EBNA3	TSHR	0.26872
EBNA3	NECAB1	0.10976
EBNA3	PLXDC2	0.41166
EBNA3	BLOC1S3	0.21525
EBNA3	AMOT	0.21066
EBNA3	DNAAF2	0.1645
EBNA3	CCDC154	0.032057
EBNA3	MAATS1	0.41174
EBNA3	TBC1D8	0.13728
EBNA3	THY1	0.056867
EBNA3	ALOX5AP	0.17869
EBNA3	DLK2	0.20759

EBNA3	BHLHE40	0.09811
EBNA3	TTLL6	0.26087
EBNA3	SLC4A1	0.2308
EBNA3	PTK7	0.1595
EBNA3	ADI1	0.23233
EBNA3	PCDHB5	0.16421
EBNA3	PAGE5	0.092288
EBNA3	SPAG5	0.1967
EBNA3	TOB1	0.3467
EBNA3	MYOD1	0.042174
EBNA3	BORA	0.26394
EBNA3	WDR12	0.26895
EBNA3	MAN1C1	0.075311
EBNA3	HM13	0.22549
EBNA3	JUNB	0.057086
EBNA3	SLC25A17	0.20081
EBNA3	CHUK	0.033895
EBNA3	VPS28	0.28468
EBNA3	URM1	0.36481
EBNA3	NUF2	0.25895
EBNA3	CABS1	0.17712
EBNA3	KLF10	0.10054
EBNA3	MAGEA2	0.27109
EBNA3	TRAK2	0.20146
EBNA3	GAGE10	0.10079
EBNA3	PRKACA	0.022289
EBNA3	SLC16A13	0.062263
EBNA3	CASP8AP2	0.23243
EBNA3	MZT2B	0.35484
EBNA3	FNTB	0.22431
EBNA3	RNF7	0.25117
EBNA3	HNRNPR	0.31133
EBNA3	CHFR	0.21156
EBNA3	CLN3	0.060558
EBNA3	CRTC1	0.23928
EBNA3	QKI	0.33832
EBNA3	RTN4R	0.31523
EBNA3	RBM15	0.33488
EBNA3	VEZT	0.16221
EBNA3	STXBP2	0.23022
EBNA3	KSR1	0.1027
EBNA3	POLR1A	0.22889
EBNA3	EPCAM	0.33544
EBNA3	UMODL1	0.16647
EBNA3	BOP	0.0127
EBNA3	GNL3	0.25032
EBNA3	VPS33B	0.2463
EBNA3	CLPX	0.23047
EBNA3	FAM117B	0.16293
EBNA3	UACA	0.2215
EBNA3	GLS	0.23327
EBNA3	PAK3	0.10846
EBNA3	C5orf30	0.32976
EBNA3	MYL4	0.15247
EBNA3	CD8B	0.25545
EBNA3	EIF4G2	0.40194
EBNA3	TOM1L1	0.28074
EBNA3	CAPN14	0.089042
EBNA3	RABGGTB	0.38015
EBNA3	COLQ	0.22989
EBNA3	ARHGEF7	0.067004
EBNA3	THRA	0.024797
EBNA3	NPAT	0.15027
EBNA3	ORAI1	0.26424
EBNA3	RAD21	0.21484
EBNA3	MAPT	0.074502
EBNA3	UCK1	0.047841
EBNA3	CD80	0.24551

EBNA3	PHYHIP	0.024369
EBNA3	PCDH11Y	0.18006
EBNA3	METTL1	0.33622
EBNA3	MRFAP1	0.36846
EBNA3	MMS19	0.19787
EBNA3	ITGB3	0.08095
EBNA3	INSR	0.02839
EBNA3	TLN2	0.16069
EBNA3	TRABD2B	0.20012
EBNA3	ASB9	0.3347
EBNA3	TRPC3	0.21964
EBNA3	GATA2	0.031079
EBNA3	CIZ1	0.28046
EBNA3	CHD8	0.13211
EBNA3	RPL11	0.19676
EBNA3	NLRP1	0.1863
EBNA3	NCBP1	0.16624
EBNA3	HDAC4	0.025809
EBNA3	MMP10	0.05405
EBNA3	EDC3	0.36452
EBNA3	MAD2L2	0.23357
EBNA3	MGRN1	0.18811
EBNA3	CEP76	0.33137
EBNA3	WDR77	0.2388
EBNA3	CRKL	0.2915
EBNA3	USP7	0.14397
EBNA3	GAGE3	0.031848
EBNA3	PPP2R2B	0.22104
EBNA3	BGLAP	0.17927
EBNA3	TIMP3	0.26291
EBNA3	RPL13	0.28237
EBNA3	ALDH7A1	0.28131
EBNA3	GDI2	0.26447
EBNA3	PTK6	0.14172
EBNA3	MESDC1	0.028775
EBNA3	ZRSR2	0.27327
EBNA3	CD200R1	0.34185
EBNA3	UHRF1	0.10858
EBNA3	SULT2A1	0.30151
EBNA3	NEBL	0.3511
EBNA3	KLKB1	0.302
EBNA3	TMF1	0.24827
EBNA3	C10orf91	0.14364
EBNA3	IL1R1	0.26589
EBNA3	LATS2	0.13507
EBNA3	MTA2	0.064144
EBNA3	CREB3L3	0.15546
EBNA3	NCOA7	0.27375
EBNA3	PCDHA5	0.16911
EBNA3	RABGAP1	0.26977
EBNA3	GK3P	0.085622
EBNA3	SMARCD1	0.27008
EBNA3	SHROOM2	0.16791
EBNA3	ZNF148	0.097646
EBNA3	CCDC28A	0.16676
EBNA3	PCDH11X	0.1692
EBNA3	TTC22	0.016787
EBNA3	KBTBD7	0.40098
EBNA3	ERCC4	0.11582
EBNA3	CYBA	0.089827
EBNA3	AKT1S1	0.22625
EBNA3	LTBR	0.26936
EBNA3	ARHGAP8	0.3178
EBNA3	MYOCD	0.063293
EBNA3	UHRF1BP1	0.38423
EBNA3	GH1	0.22119
EBNA3	CTH	0.11531
EBNA3	SHANK2	0.3461

EBNA3	CDH20	0.17459
EBNA3	LDB1	0.026843
EBNA3	SLA2	0.33466
EBNA3	CLTC	0.19722
EBNA3	XRCC6	0.050982
EBNA3	GATA1	0.023364
EBNA3	NADK2	0.04362
EBNA3	PPARA	0.036845
EBNA3	GPIHBP1	0.13093
EBNA3	FCGR1A	0.2872
EBNA3	SH3GL1	0.37528
EBNA3	ARVCF	0.33514
EBNA3	CBX2	0.2372
EBNA3	PLEKHA5	0.26993
EBNA3	FOXD3	0.05118
EBNA3	EIF3F	0.35979
EBNA3	PPP3R2	0.21072
EBNA3	FAM120C	0.026393
EBNA3	CIITA	0.19683
EBNA3	UPF2	0.38175
EBNA3	NECAP1	0.10706
EBNA3	CACNB2	0.24456
EBNA3	SIN3B	0.23193
EBNA3	TNPO3	0.28145
EBNA3	CACNA1C	0.18503
EBNA3	SIK1	0.057851
EBNA3	SH3RF1	0.21899
EBNA3	TRPM6	0.2514
EBNA3	RAB5A	0.2044
EBNA3	ERBB2IP	0.20944
EBNA3	MIR17HG	0.15684
EBNA3	CCDC65	0.016529
EBNA3	LPCAT2	0.095787
EBNA3	OPA1	0.15649
EBNA3	PPP2R5D	0.29349
EBNA3	BIN1	0.22647
EBNA3	BCL2L11	0.067022
EBNA3	SECTM1	0.13757
EBNA3	EIF4ENIF1	0.26812
EBNA3	SYNRG	0.16548
EBNA3	GNAI1	0.12785
EBNA3	HIST2H3A	0.25505
EBNA3	NRIP1	0.11785
EBNA3	PSMB9	0.099839
EBNA3	RBMY1A1	0.41125
EBNA3	EEF1G	0.30129
EBNA3	COL7A1	0.30045
EBNA3	STAT4	0.058429
EBNA3	RAG1	0.12416
EBNA3	RRAD	0.32431
EBNA3	GID4	0.049886
EBNA3	STX4	0.17504
EBNA3	PPP1R10	0.10658
EBNA3	LOXL4	0.31107
EBNA3	EVA1B	0.13988
EBNA3	GFI1	0.13542
EBNA3	SIK3	0.20679
EBNA3	FAM83F	0.10548
EBNA3	ROBO2	0.17945
EBNA3	CALCA	0.13799
EBNA3	NUP214	0.22694
EBNA3	NAA50	0.45042
EBNA3	STAU1	0.32131
EBNA3	YKT6	0.065222
EBNA3	CHAMP1	0.19044
EBNA3	ARHGEF15	0.29291
EBNA3	TPCN1	0.20289
EBNA3	MERTK	0.15599

EBNA3	MRPL20	0.28793
EBNA3	SRF	0.017937
EBNA3	UBL4A	0.35848
EBNA3	CREBZF	0.16801
EBNA3	PELP1	0.33747
EBNA3	NFKB1	0.031463
EBNA3	STAT3	0.045368
EBNA3	ZNF488	0.22951
EBNA3	RHBDL2	0.012771
EBNA3	FAM101A	0.07203
EBNA3	SLC5A2	0.036338
EBNA3	ACIN1	0.28452
EBNA3	BARD1	0.21644
EBNA3	PPFIA4	0.38703
EBNA3	CKB	0.29865
EBNA3	TNFSF12	0.18918
EBNA3	TNS3	0.37614
EBNA3	FAM160A2	0.36241
EBNA3	KMT2C	0.27851
EBNA3	KLHL20	0.23589
EBNA3	RAD23A	0.25646
EBNA3	SMG9	0.32001
EBNA3	PAK1	0.025412
EBNA3	HPCAL4	0.20147
EBNA3	VPS4A	0.1966
EBNA3	SNURFL	0.068186
EBNA3	COCH	0.32413
EBNA3	PSPC1	0.33292
EBNA3	STK40	0.22239
EBNA3	SRPX2	0.265
EBNA3	ORC4	0.22841
EBNA3	THBD	0.41453
EBNA3	KIAA0040	0.019625
EBNA3	GLYCTK	0.42792
EBNA3	ARHGEF4	0.15388
EBNA3	SMAD5	0.077908
EBNA3	KIAA0226	0.32013
EBNA3	DCHS1	0.17455
EBNA3	CREM	0.14112
EBNA3	ZFR	0.36039
EBNA3	RTP4	0.36917
EBNA3	FSHR	0.19031
EBNA3	FANCM	0.23559
EBNA3	EEF1D	0.28077
EBNA3	TRADD	0.15032
EBNA3	PYGO1	0.26919
EBNA3	DHX8	0.30581
EBNA3	MTERF	0.26846
EBNA3	ATG13	0.23587
EBNA3	BRAF	0.19542
EBNA3	NPBWR2	0.31408
EBNA3	ARPC2	0.26055
EBNA3	ARFGEF2	0.1438
EBNA3	TTC19	0.30662
EBNA3	BSG	0.19885
EBNA3	MT2A	0.26487
EBNA3	AIG1	0.41166
EBNA3	ORM1	0.38263
EBNA3	SLC18A3	0.01734
EBNA3	HSD17B10	0.17704
EBNA3	RBM5	0.26063
EBNA3	BOP1	0.24683
EBNA3	NOX1	0.10883
EBNA3	GYG1	0.3065
EBNA3	DSC2	0.15634
EBNA3	CAPS	0.15683
EBNA3	CLSPN	0.28069
EBNA3	FAM178A	0.10102

EBNA3	MLST8	0.29978
EBNA3	WTIP	0.20342
EBNA3	SPTB	0.19785
EBNA3	IL1R2	0.40281
EBNA3	KLF13	0.19909
EBNA3	MSN	0.12041
EBNA3	SPDL1	0.20352
EBNA3	UBE2D4	0.23929
EBNA3	PCSK1	0.021199
EBNA3	SMARCE1	0.21012
EBNA3	PIGA	0.29222
EBNA3	RPS26	0.20111
EBNA3	MEN1	0.057102
EBNA3	GNMT	0.30242
EBNA3	BRE	0.26988
EBNA3	ZBTB14	0.24405
EBNA3	SCAMP1	0.32388
EBNA3	SEC23A	0.18156
EBNA3	RND1	0.1986
EBNA3	BCKDHB	0.31184
EBNA3	PDLIM7	0.25095
EBNA3	SSH1	0.07931
EBNA3	RNMT	0.38885
EBNA3	C12orf36	0.036851
EBNA3	TNFRSF21	0.34584
EBNA3	AK5	0.058002
EBNA3	KIFC3	0.069687
EBNA3	TTF2	0.17627
EBNA3	TOPORS	0.11462
EBNA3	DDX47	0.32105
EBNA3	FREM2	0.13095
EBNA3	GOLM1	0.38672
EBNA3	GNAI2	0.097502
EBNA3	DDX42	0.34069
EBNA3	GAPDHS	0.25422
EBNA3	CDKN1C	0.27571
EBNA3	GRIA1	0.14955
EBNA3	ANKLE2	0.26752
EBNA3	NUP153	0.17282
EBNA3	ETS2	0.098358
EBNA3	TOMM22	0.24122
EBNA3	VPS41	0.22969
EBNA3	MAGOH	0.31208
EBNA3	SKI	0.05331
EBNA3	SRGN	0.21467
EBNA3	CEP41	0.25873
EBNA3	RAC1	0.045196
EBNA3	INTS9	0.36808
EBNA3	IPO5	0.27318
EBNA3	RPUSD3	0.028966
EBNA3	VDAC1	0.17207
EBNA3	PRMT5	0.20723
EBNA3	MUC16	0.31051
EBNA3	TNFAIP1	0.23965
EBNA3	GRIN2A	0.017562
EBNA3	CRNN	0.18637
EBNA3	ING4	0.20977
EBNA3	PECAM1	0.2649
EBNA3	EML1	0.67913
EBNA3	GTF2F2	0.15568
EBNA3	CEP170	0.35404
EBNA3	OTUD6B	0.017791
EBNA3	C19orf68	0.43795
EBNA3	ACRBP	0.21264
EBNA3	NOL3	0.30144
EBNA3	ZWILCH	0.30693
EBNA3	VAV2	0.11432
EBNA3	KIF24	0.3255

EBNA3	FAM83C	0.055511
EBNA3	MAGED1	0.23683
EBNA3	ETS1	0.046056
EBNA3	PTGER4	0.15778
EBNA3	IQCK	0.08945
EBNA3	RPRD1B	0.26954
EBNA3	ITFG2	0.017056
EBNA3	CWC15	0.43032
EBNA3	CALCRL	0.12318
EBNA3	RBPMS	0.31711
EBNA3	AK1	0.049973
EBNA3	DES	0.22253
EBNA3	WDR4	0.33275
EBNA3	PSMC1	0.13323
EBNA3	USP5	0.22007
EBNA3	CITED1	0.048896
EBNA3	IP6K1	0.047393
EBNA3	HAT1	0.29844
EBNA3	SNCAIP	0.29071
EBNA3	FZD3	0.16229
EBNA3	LIN28A	0.19848
EBNA3	FAM166A	0.10107
EBNA3	MPDZ	0.27888
EBNA3	NGFRAP1	0.26056
EBNA3	ARID2	0.24954
EBNA3	PGS1	0.11589
EBNA3	TLDC1	0.43795
EBNA3	COMMD3	0.43795
EBNA3	SMAD7	0.051219
EBNA3	SERPINA1	0.28682
EBNA3	CAV1	0.010765
EBNA3	NCK1	0.15219
EBNA3	GABBR1	0.10574
EBNA3	ADAD2	0.023361
EBNA3	CRTAC1	0.21517
EBNA3	SCP2	0.126
EBNA3	TMEM173	0.076844
EBNA3	MAD2L1	0.17103
EBNA3	PRRC2B	0.43795
EBNA3	CLN6	0.28791
EBNA3	KCNJ3	0.30353
EBNA3	XKRY	0.14761
EBNA3	BYSL	0.28369
EBNA3	ERN1	0.068957
EBNA3	EYA1	0.034757
EBNA3	SPRTN	0.2894
EBNA3	SFSWAP	0.07099
EBNA3	MYD88	0.081494
EBNA3	MUM1L1	0.09742
EBNA3	LSM5	0.31372
EBNA3	CTC1	0.25373
EBNA3	FBXW8	0.39916
EBNA3	SPPL2A	0.22562
EBNA3	PLS1	0.18592
EBNA3	SYVN1	0.17794
EBNA3	CACYBP	0.29339
EBNA3	FAM74A4	0.037221
EBNA3	INS	0.048021
EBNA3	IRAK4	0.16199
EBNA3	EPS15	0.36405
EBNA3	DIMT1	0.059966
EBNA3	PLS3	0.19819
EBNA3	MYO5B	0.32408
EBNA3	WWC1	0.21984
EBNA3	HUS1	0.25724
EBNA3	PCSK2	0.37892
EBNA3	CRHR1	0.10298
EBNA3	WHAMM	0.089897

EBNA3	APLF	0.24717
EBNA3	YY1	0.10823
EBNA3	NKRF	0.30514
EBNA3	ZNF335	0.25167
EBNA3	AURKB	0.079474
EBNA3	CDC42	0.013992
EBNA3	RRAGC	0.17395
EBNA3	LZTS1	0.10599
EBNA3	CD48	0.25099
EBNA3	VWCE	0.20437
EBNA3	CLEC3B	0.14932
EBNA3	PRMT8	0.3177
EBNA3	SLC17A7	0.018288
EBNA3	INPP5B	0.23995
EBNA3	ARHGEF16	0.15081
EBNA3	BST2	0.16228
EBNA3	NHLH2	0.238
EBNA3	PEX11G	0.33282
EBNA3	OGDHL	0.36723
EBNA3	FKBP8	0.17964
EBNA3	DISC1	0.1812
EBNA3	IL33	0.25786
EBNA3	PUM2	0.44062
EBNA3	ESPL1	0.19906
EBNA3	PCGF1	0.24531
EBNA3	CDH8	0.166
EBNA3	LTC4S	0.15471
EBNA3	AP3S1	0.40702
EBNA3	UHMK1	0.24933
EBNA3	LAT2	0.33285
EBNA3	BUB3	0.19452
EBNA3	ALDH3A1	0.015119
EBNA3	RBMY1B	0.39563
EBNA3	SPRR3	0.2167
EBNA3	SDF4	0.49197
EBNA3	KIF14	0.27207
EBNA3	AFF4	0.17797
EBNA3	LEPR	0.32679
EBNA3	ANKHD1	0.45124
EBNA3	MGP	0.1233
EBNA3	BCKDHA	0.3306
EBNA3	TRIM33	0.1324
EBNA3	HBB	0.23008
EBNA3	NACAP1	0.028517
EBNA3	KIF5B	0.22251
EBNA3	HEG1	0.097126
EBNA3	PRKCQ	0.045045
EBNA3	PHIP	0.24109
EBNA3	KRTAP2-2	0.05706
EBNA3	TAF11	0.25623
EBNA3	TRPC7	0.23503
EBNA3	LAMTOR2	0.26899
EBNA3	MAZ	0.28775
EBNA3	HIST1H1E	0.28164
EBNA3	WSB1	0.39195
EBNA3	CDC45	0.25648
EBNA3	YIPF5	0.090986
EBNA3	YAF2	0.22658
EBNA3	NSUN4	0.090755
EBNA3	MYH9	0.088641
EBNA3	EID3	0.27433
EBNA3	SPACA3	0.31519
EBNA3	HHAT	0.1143
EBNA3	PTPLB	0.26567
EBNA3	INSIG2	0.18082
EBNA3	CDCA8	0.22404
EBNA3	STAT1	0.1026
EBNA3	GPATCH1	0.11601

EBNA3	PRKAB1	0.13429
EBNA3	GJA1	0.05127
EBNA3	PSEN2	0.030851
EBNA3	FAT4	0.65766
EBNA3	DTNBP1	0.19248
EBNA3	SDE2	0.010378
EBNA3	CUL9	0.28133
EBNA3	PNMA2	0.3699
EBNA3	WWP1	0.18161
EBNA3	PLIN3	0.29619
EBNA3	KCNIP4	0.16203
EBNA3	USP34	0.30759
EBNA3	ZNHIT6	0.32125
EBNA3	VPS18	0.24522
EBNA3	ARHGAP4	0.23121
EBNA3	IFI16	0.10105
EBNA3	PPEF1	0.53303
EBNA3	AP1M2	0.03096
EBNA3	ZW10	0.13274
EBNA3	GRIN3A	0.1471
EBNA3	CANT1	0.087541
EBNA3	CXorf67	0.14467
EBNA3	NPW	0.35183
EBNA3	MAPK11	0.10151
EBNA3	TLL1	0.046703
EBNA3	C18orf25	0.05625
EBNA3	A2M	0.20926
EBNA3	DCTN1	0.23926
EBNA3	FTSJ3	0.42193
EBNA3	ETV1	0.15076
EBNA3	FCER1G	0.039951
EBNA3	PRKAA2	0.035526
EBNA3	NELL2	0.55342
EBNA3	CALB2	0.22028
EBNA3	PAICS	0.23117
EBNA3	DDX60	0.27883
EBNA3	CLTB	0.31368
EBNA3	PCDHA7	0.16911
EBNA3	CHD4	0.10269
EBNA3	ZC3H10	0.35798
EBNA3	ARRB2	0.099951
EBNA3	PRDX1	0.26018
EBNA3	RHOG	0.18769
EBNA3	SLC2A8	0.017799
EBNA3	C11orf35	0.019796
EBNA3	NAGS	0.063006
EBNA3	TTC3	0.21146
EBNA3	SPON1	0.39025
EBNA3	IRAK3	0.10617
EBNA3	CYFIP1	0.21776
EBNA3	SEC11A	0.090549
EBNA3	NR2F1	0.11114
EBNA3	C16orf86	0.029348
EBNA3	ULK2	0.30175
EBNA3	SRSF3	0.33765
EBNA3	PDZK1	0.34803
EBNA3	MCC	0.29505
EBNA3	KAT2B	0.055701
EBNA3	CROCC	0.19802
EBNA3	LAMTOR3	0.30644
EBNA3	ARRB1	0.068506
EBNA3	ELOVL4	0.26256
EBNA3	CAPN9	0.085562
EBNA3	FAM188B2	0.19342
EBNA3	UBE3A	0.15908
EBNA3	TCF4	0.11519
EBNA3	GTF3C1	0.26304
EBNA3	EIF1AX	0.099096

19789	EBNA3	SMC1A	0.13132
19790	EBNA3	DMAP1	0.23123
19791	EBNA3	RPS27	0.15299
19792	EBNA3	CHEK2	0.064629
19793	EBNA3	STX1A	0.14178
19794	EBNA3	DRD2	0.020493
19795	EBNA3	APH1A	0.17894
19796	EBNA3	GBAS	0.36858
19797	EBNA3	BAZ1B	0.17949
19798	EBNA3	AKIP1	0.37995
19799	EBNA3	PCDHGC5	0.18006
19800	EBNA3	KAT2A	0.17318
19801	EBNA3	COL4A3BP	0.096136
19802	EBNA3	SEMA4C	0.0221
19803	EBNA3	TRPC4	0.19734
19804	EBNA3	KIF5C	0.26396
19805	EBNA3	FZD10	0.16222
19806	EBNA3	PIN1	0.21565
19807	EBNA3	TLX3	0.16163
19808	EBNA3	SUV420H2	0.33975
19809	EBNA3	PCDHGA7	0.18006
19810	EBNA3	HECTD1	0.27249
19811	EBNA3	NME1	0.17733
19812	EBNA3	NOP58	0.3014
19813	EBNA3	MAP1LC3C	0.43298
19814	EBNA3	RFC3	0.20156
19815	EBNA3	ATP6V1E1	0.2471
19816	EBNA3	ATP11B	0.14526
19817	EBNA3	RNF14	0.16061
19818	EBNA3	TARBP2	0.20867
19819	EBNA3	SFR1	0.38815
19820	EBNA3	TAB1	0.14668
19821	EBNA3	RHOA	0.043191
19822	EBNA3	KCNH2	0.15033
19823	EBNA3	KRT20	0.30116
19824	EBNA3	NUDCD3	0.07085
19825	EBNA3	ZNF646	0.25167
19826	EBNA3	MAP4K4	0.23113
19827	EBNA3	DCAF11	0.42039
19828	EBNA3	THBS1	0.06533
19829	EBNA3	RUNX3	0.095522
19830	EBNA3	MAGEF1	0.019165
19831	EBNA3	LMTK2	0.18891
19832	EBNA3	GADD45GIP1	0.33588
19833	EBNA3	DIS3	0.23463
19834	EBNA3	ARR3	0.2986
19835	EBNA3	ABCD3	0.23324
19836	EBNA3	UBR2	0.19132
19837	EBNA3	CMKLR1	0.23593
19838	EBNA3	STK25	0.19509
19839	EBNA3	FANCF	0.3595
19840	EBNA3	SEMG1	0.25368
19841	EBNA3	PLA2G4A	0.028395
19842	EBNA3	ICAM3	0.28444
19843	EBNA3	FAM69C	0.21448
19844	EBNA3	MRPL49	0.34456
19845	EBNA3	ASS1	0.049328
19846	EBNA3	CAPN8	0.083886
19847	EBNA3	PANK4	0.01596
19848	EBNA3	EIF3I	0.40527
19849	EBNA3	ARL2BP	0.31308
19850	EBNA3	UBTF	0.24761
19851	EBNA3	SPACA4	0.044149
19852	EBNA3	FGF2	0.035078
19853	EBNA3	PFN1	0.19207
19854	EBNA3	DLG3	0.19416
19855	EBNA3	RBCK1	0.1005
19856	EBNA3	DSG2	0.13548

EBNA3	MAP3K12	0.10559
EBNA3	CCNE1	0.086847
EBNA3	RHOC	0.11707
EBNA3	PITX2	0.022742
EBNA3	ADRA2B	0.16726
EBNA3	ATXN3	0.15968
EBNA3	EPB41L3	0.20674
EBNA3	CEBPG	0.093099
EBNA3	ALDH16A1	0.010776
EBNA3	DEDD2	0.21354
EBNA3	MGMT	0.1023
EBNA3	GIT2	0.24172
EBNA3	SNX33	0.41164
EBNA3	STK24	0.14273
EBNA3	PCDHB2	0.15424
EBNA3	ATG101	0.32993
EBNA3	MIF	0.14596
EBNA3	ISG20L2	0.26715
EBNA3	BICD1	0.21114
EBNA3	AP2M1	0.22973
EBNA3	ING2	0.14539
EBNA3	PIK3CG	0.10285
EBNA3	ADORA1	0.051641
EBNA3	CDC42EP2	0.25069
EBNA3	SF3A1	0.36253
EBNA3	INTS3	0.34812
EBNA3	DSG4	0.13961
EBNA3	KIAA1407	0.054396
EBNA3	TCTEX1D4	0.43795
EBNA3	FAM184B	0.042479
EBNA3	TMCC2	0.41166
EBNA3	PLG	0.1428
EBNA3	TEAD3	0.13944
EBNA3	ROCK1	0.03696
EBNA3	NOS2	0.11082
EBNA3	LPCAT3	0.062989
EBNA3	PRMT1	0.3478
EBNA3	CCSER1	0.12362
EBNA3	USP10	0.21742
EBNA3	LASP1	0.25713
EBNA3	MT-ND1	0.30076
EBNA3	RASSF5	0.32919
EBNA3	CLTA	0.050141
EBNA3	CRB2	0.18644
EBNA3	DYNLT1	0.18333
EBNA3	ZMIZ2	0.23115
EBNA3	CCDC144A	0.011234
EBNA3	ALDOB	0.068381
EBNA3	S100Z	0.21072
EBNA3	MED26	0.23218
EBNA3	LRP5	0.13023
EBNA3	HSD17B12	0.19067
EBNA3	DGKQ	0.24317
EBNA3	CRP	0.26209
EBNA3	ARIH2	0.20411
EBNA3	DHX36	0.23772
EBNA3	C1QBP	0.25902
EBNA3	PNMAL2	0.083394
EBNA3	DDOST	0.24689
EBNA3	AGR3	0.48107
EBNA3	AP1M1	0.28381
EBNA3	PUF60	0.26398
EBNA3	GMCL1P1	0.37715
EBNA3	ECM29	0.024911
EBNA3	STRA13	0.27114
EBNA3	NOL9	0.019573
EBNA3	NECAP2	0.067283
EBNA3	SNX8	0.32743

EBNA3	MAPK6	0.14993
EBNA3	RPL23A	0.20776
EBNA3	GALK1	0.016936
EBNA3	SPOP	0.36193
EBNA3	PRKCZ	0.057887
EBNA3	HDDC2	0.30851
EBNA3	FANCI	0.34739
EBNA3	CYCS	0.12779
EBNA3	SETDB1	0.2881
EBNA3	CDC14A	0.077712
EBNA3	SEC22B	0.25551
EBNA3	ATP7A	0.030926
EBNA3	AGR2	0.43897
EBNA3	YPR022C	0.12046
EBNA3	UQCRC2	0.16336
EBNA3	FHL2	0.091707
EBNA3	ADM	0.10737
EBNA3	TPPP	0.50163
EBNA3	ZNF746	0.19224
EBNA3	GOLGA8CP	0.17213
EBNA3	PRPF4	0.30967
EBNA3	DBNDD2	0.41166
EBNA3	BMP10	0.15473
EBNA3	TBXA2R	0.16918
EBNA3	FYB	0.23614
EBNA3	SYT4	0.22209
EBNA3	IL17RE	0.38697
EBNA3	CENPT	0.24208
EBNA3	CCDC146	0.059189
EBNA3	RAD51D	0.30085
EBNA3	SYT11	0.60573
EBNA3	CD5	0.28798
EBNA3	CCDC150	0.051937
EBNA3	ATP5I	0.042511
EBNA3	SH2D4A	0.34127
EBNA3	ICAM1	0.085972
EBNA3	GZMB	0.27665
EBNA3	GGA2	0.2775
EBNA3	MAD1L1	0.17791
EBNA3	C1orf105	0.1241
EBNA3	DCUN1D1	0.4117
EBNA3	LPAR1	0.11144
EBNA3	MOB3B	0.39642
EBNA3	MSH6	0.091155
EBNA3	SNRNP200	0.23453
EBNA3	PTCH1	0.058603
EBNA3	EIF2B1	0.19478
EBNA3	PDE3B	0.13702
EBNA3	DRD3	0.075661
EBNA3	CTNNA3	0.21781
EBNA3	IFITM1	0.23506
EBNA3	FOXH1	0.022671
EBNA3	SWSAP1	0.36155
EBNA3	DDX17	0.23633
EBNA3	RECQL	0.20319
EBNA3	BCS1L	0.27154
EBNA3	SH3PXD2A	0.34074
EBNA3	SFPQ	0.30763
EBNA3	SYK	0.026793
EBNA3	PARPBP	0.35014
EBNA3	FBXL15	0.33719
EBNA3	FANCA	0.31724
EBNA3	FIP1L1	0.17902
EBNA3	EIF2B3	0.28021
EBNA3	TMEM11	0.33577
EBNA3	KDM1A	0.040571
EBNA3	PCDHAC2	0.16911
EBNA3	CLSTN2	0.10397

EBNA3	RBMS1	0.3773
EBNA3	ATP6V0D1	0.2128
EBNA3	TPD52L1	0.30167
EBNA3	ASPHD1	0.077482
EBNA3	WDFY3	0.32922
EBNA3	PSMA4	0.096971
EBNA3	HSP90B1	0.34904
EBNA3	NR2C1	0.12881
EBNA3	PCDH7	0.17442
EBNA3	PIAS1	0.1529
EBNA3	HOXB7	0.14786
EBNA3	TNFSF13B	0.18407
EBNA3	CDK6	0.032225
EBNA3	MPP5	0.31013
EBNA3	ZBP1	0.035773
EBNA3	TRIM2	0.28552
EBNA3	DDAH2	0.27814
EBNA3	ATPAF2	0.3992
EBNA3	MACF1	0.3904
EBNA3	AIFM1	0.1809
EBNA3	CCDC66	0.019867
EBNA3	CKAP5	0.23315
EBNA3	TNFRSF12A	0.25601
EBNA3	POLR3F	0.22474
EBNA3	S100A3	0.17787
EBNA3	MEIS1	0.12582
EBNA3	CCDC138	0.011307
EBNA3	WWOX	0.24322
EBNA3	ADORA2A	0.046533
EBNA3	SOS1	0.13164
EBNA3	APOD	0.16386
EBNA3	TXNL4A	0.32329
EBNA3	EVL	0.26337
EBNA3	BOK	0.24601
EBNA3	ALG2	0.23429
EBNA3	GLMN	0.34859
EBNA3	MAGEC2	0.34862
EBNA3	UVSSA	0.37579
EBNA3	PCM1	0.19354
EBNA3	PNLIPRP1	0.065986
EBNA3	TPSAB1	0.37766
EBNA3	TXNRD2	0.36903
EBNA3	FGF21	0.336
EBNA3	PCGF3	0.26919
EBNA3	MSR1	0.32945
EBNA3	DUOX2	0.18813
EBNA3	RTN1	0.32953
EBNA3	RBM14	0.19325
EBNA3	OSBPL6	0.32513
EBNA3	CCDC176	0.14344
EBNA3	STBD1	0.36343
EBNA3	CASQ2	0.13884
EBNA3	MCTP2	0.18784
EBNA3	PRKG1	0.14999
EBNA3	GSTO1	0.011374
EBNA3	TRPC4AP	0.38504
EBNA3	LPXN	0.18906
EBNA3	PUS7L	0.054505
EBNA3	UBE2A	0.20834
EBNA3	HMBS	0.064734
EBNA3	EPHA7	0.1593
EBNA3	SNTB2	0.31869
EBNA3	CX3CL1	0.11225
EBNA3	PCDHGB4	0.17455
EBNA3	AP5Z1	0.32306
EBNA3	PPIE	0.33268
EBNA3	KLRK1	0.21948
EBNA3	CAST	0.42332

EBNA3	TBC1D25	0.38928
EBNA3	DNAJC7	0.34439
EBNA3	GCH1	0.19435
EBNA3	PTGES3	0.20031
EBNA3	ANKRD1	0.14032
EBNA3	C10orf67	0.11917
EBNA3	VPS11	0.33854
EBNA3	POLE3	0.29395
EBNA3	LINC00597	0.078515
EBNA3	FAM131C	0.023068
EBNA3	SLC9A3	0.25028
EBNA3	OGN	0.28403
EBNA3	HNRNPU	0.25125
EBNA3	OPRD1	0.19463
EBNA3	ZBTB8OS	0.010264
EBNA3	MDM2	0.074565
EBNA3	PRDM11	0.010526
EBNA3	NKD2	0.49133
EBNA3	MT1HL1	0.066671
EBNA3	LSAMP	0.37204
EBNA3	TRAPPC1	0.04432
EBNA3	KRTAP13-4	0.053578
EBNA3	PTPN23	0.15501
EBNA3	ADAMTSL2	0.2865
EBNA3	FLNC	0.32681
EBNA3	CASD1	0.16974
EBNA3	MAGI3	0.23562
EBNA3	C2orf88	0.021063
EBNA3	SLAMF1	0.24855
EBNA3	IFT57	0.28741
EBNA3	ADAM23	0.22801
EBNA3	AP5B1	0.39218
EBNA3	TRIM29	0.18226
EBNA3	EIF2B5	0.25897
EBNA3	DENR	0.44087
EBNA3	ZNF350	0.24952
EBNA3	SV2A	0.032076
EBNA3	TMEM132D	0.37523
EBNA3	MAPK8IP1	0.11665
EBNA3	SCAF1	0.13144
EBNA3	NCOR1P1	0.011672
EBNA3	CHKA	0.059878
EBNA3	FOXN3	0.083468
EBNA3	PTPN4	0.14693
EBNA3	ATP2B2	0.10379
EBNA3	BTG2	0.19788
EBNA3	PEX3	0.25412
EBNA3	GHITM	0.35279
EBNA3	INCENP	0.20064
EBNA3	GPX6	0.024815
EBNA3	TBC1D9	0.15086
EBNA3	CDK1	0.019756
EBNA3	C19orf71	0.076303
EBNA3	DOT1L	0.33548
EBNA3	UCK2	0.043855
EBNA3	JMJD1C	0.33313
EBNA3	GJD3	0.34051
EBNA3	FNBP1L	0.36054
EBNA3	ITGAV	0.10286
EBNA3	TXLNB	0.41165
EBNA3	SEC13	0.23679
EBNA3	SNX29P2	0.0423
EBNA3	CDH10	0.16097
EBNA3	RHPN1-AS1	0.10926
EBNA3	CDKN2AIP	0.31979
EBNA3	KEAP1	0.25874
EBNA3	EPHA10	0.27512
EBNA3	CNOT6	0.24949

EBNA3	LCK	0.055641
EBNA3	C6orf118	0.067669
EBNA3	HIPK2	0.052979
EBNA3	IL17REL	0.023523
EBNA3	PSMC4	0.12219
EBNA3	RAD50	0.10639
EBNA3	CCDC74B	0.052333
EBNA3	FAT1	0.40554
EBNA3	REV1	0.21612
EBNA3	BAZ1A	0.20995
EBNA3	OCRL	0.1742
EBNA3	DYRK1B	0.18507
EBNA3	PRKD2	0.014396
EBNA3	ABCF1	0.25268
EBNA3	HDAC3	0.059567
EBNA3	FBLN7	0.18326
EBNA3	NXF3	0.40691
EBNA3	H2AFY	0.24183
EBNA3	FOXK1	0.052012
EBNA3	CCNH	0.047282
EBNA3	FCN1	0.04927
EBNA3	NGLY1	0.33326
EBNA3	IGFL3	0.14965
EBNA3	IRF1	0.023347
EBNA3	EDAR	0.25066
EBNA3	MTCH1	0.31667
EBNA3	ERCC8	0.25375
EBNA3	CRELD1	0.19055
EBNA3	ISOC2	0.29512
EBNA3	TGOLN2	0.3153
EBNA3	LINC00471	0.085613
EBNA3	HABP4	0.25036
EBNA3	XAB2	0.28277
EBNA3	S100A11	0.55805
EBNA3	ITGA5	0.15251
EBNA3	GDF7	0.21619
EBNA3	GPS2	0.33653
EBNA3	SRSF5	0.29025
EBNA3	USH1G	0.27646
EBNA3	SSR3	0.049131
EBNA3	SORBS1	0.13669
EBNA3	CHMP1B	0.31316
EBNA3	HFE2	0.29261
EBNA3	IRF3	0.051483
EBNA3	CAB8	0.012643
EBNA3	EFCAB6	0.14811
EBNA3	MSANTD2	0.010523
EBNA3	CCDC137	0.14561
EBNA3	GPR179	0.11235
EBNA3	RBBP6	0.22213
EBNA3	MAP3K3	0.19016
EBNA3	TIFAB	0.14662
EBNA3	ESR2	0.07699
EBNA3	TPP1	0.20289
EBNA3	NCAPG	0.30868
EBNA3	NUFIP2	0.44955
EBNA3	ECSIT	0.37024
EBNA3	LRRFIP2	0.38978
EBNA3	C15orf54	0.035047
EBNA3	AIRE	0.12094
EBNA3	XPO4	0.35162
EBNA3	CATSPERG	0.13722
EBNA3	INIP	0.37198
EBNA3	NUTM2A	0.036551
EBNA3	NKD1	0.51348
EBNA3	NR1H2	0.068574
EBNA3	PLAC8L1	0.034208
EBNA3	PPP1R9B	0.16911

EBNA3	PLEKHM2	0.27663
EBNA3	CBFA2T3	0.080595
EBNA3	DPM3	0.22775
EBNA3	CYBB	0.15928
EBNA3	CEP70	0.29338
EBNA3	DERL2	0.27567
EBNA3	ACACA	0.19041
EBNA3	SPTBN4	0.092984
EBNA3	STAP1	0.15136
EBNA3	EDA2R	0.26261
EBNA3	PPP6C	0.26547
EBNA3	TRIAP1	0.33244
EBNA3	FASTK	0.2974
EBNA3	RBFOX2	0.25349
EBNA3	GCK	0.094801
EBNA3	RPS10	0.19321
EBNA3	CD2AP	0.2886
EBNA3	RAP2A	0.20507
EBNA3	CD1A	0.33124
EBNA3	SPATA18	0.25299
EBNA3	CBX8	0.25545
EBNA3	BCDIN3D	0.13556
EBNA3	PAGR1	0.3759
EBNA3	RNF146	0.21865
EBNA3	CROCCP3	0.029672
EBNA3	IGHG1	0.29849
EBNA3	KIF18A	0.15127
EBNA3	GORAB	0.35864
EBNA3	GABPB1	0.13652
EBNA3	GREM1	0.063197
EBNA3	FAM151B	0.050726
EBNA3	TSKS	0.25258
EBNA3	INTS12	0.30733
EBNA3	UBE2N	0.1065
EBNA3	TAF7	0.065461
EBNA3	NSUN7	0.16527
EBNA3	MAGEB18	0.43795
EBNA3	GCSAML	0.082857
EBNA3	MED31	0.25325
EBNA3	MAT2B	0.21452
EBNA3	GEMIN4	0.26323
EBNA3	NOG	0.026045
EBNA3	HMCN1	0.22262
EBNA3	SLC9A3R2	0.2032
EBNA3	KNSTRN	0.22753
EBNA3	ODF2	0.15961
EBNA3	SERPINE1	0.068328
EBNA3	PCBP2	0.26089
EBNA3	METTL4	0.025214
EBNA3	SPC25	0.25895
EBNA3	RBM25	0.29656
EBNA3	DACH1	0.096856
EBNA3	PKP1	0.29724
EBNA3	DNAJB5	0.35494
EBNA3	EIF1AY	0.54659
EBNA3	PLA2G2E	0.074695
EBNA3	FNIP2	0.32561
EBNA3	DEAF1	0.1966
EBNA3	ARID4B	0.27551
EBNA3	EMR2	0.10813
EBNA3	TREML1	0.33752
EBNA3	MOV10	0.29077
EBNA3	MAGI1	0.27321
EBNA3	PTGIS	0.084145
EBNA3	ARL4A	0.29523
EBNA3	BPTF	0.16299
EBNA3	WRN	0.033209
EBNA3	USF1	0.047564

EBNA3	STMN2	0.26541
EBNA3	CAPS2	0.21072
EBNA3	FAM47B	0.090638
EBNA3	ARHGAP32	0.15833
EBNA3	MTDH	0.15956
EBNA3	RP9	0.29831
EBNA3	PRRC2A	0.37995
EBNA3	MBD3L5	0.012368
EBNA3	MKKS	0.11196
EBNA3	GMNN	0.24609
EBNA3	PDZD11	0.38479
EBNA3	METTL17	0.38635
EBNA3	MYLPF	0.1743
EBNA3	PSMD10	0.080593
EBNA3	SDHAF2	0.37668
EBNA3	ST13	0.25573
EBNA3	APOA1	0.052449
EBNA3	PSMA5	0.10345
EBNA3	HHEX	0.024232
EBNA3	SUPT20HL2	0.090443
EBNA3	PPP2R3B	0.51801
EBNA3	PEG10	0.2714
EBNA3	INO80	0.17224
EBNA3	CTNNBIP1	0.22893
EBNA3	CENPH	0.20053
EBNA3	HDAC9	0.080005
EBNA3	HEY1	0.065337
EBNA3	ACTN4	0.30794
EBNA3	SPRYD7	0.027475
EBNA3	FBXO46	0.43795
EBNA3	CDK9	0.04049
EBNA3	KIAA0141	0.3992
EBNA3	DTX3L	0.26732
EBNA3	EHD1	0.33755
EBNA3	ATL1	0.16605
EBNA3	KIR2DL1	0.34239
EBNA3	RAB25	0.36653
EBNA3	TIMM44	0.26248
EBNA3	GPRC5C	0.049641
EBNA3	PARD6B	0.2323
EBNA3	GTF2E1	0.21229
EBNA3	AP3S2	0.061175
EBNA3	RAN	0.13565
EBNA3	KIAA0408	0.43795
EBNA3	LILRA4	0.41166
EBNA3	EEA1	0.25373
EBNA3	SUN2	0.19566
EBNA3	SEPT8	0.35718
EBNA3	PINK1	0.073784
EBNA3	PREPL	0.013556
EBNA3	FANCG	0.26488
EBNA3	CTU2	0.36433
EBNA3	OXA1L	0.24917
EBNA3	INTS10	0.34745
EBNA3	LPL	0.097464
EBNA3	PKMYT1	0.11019
EBNA3	ERCC6	0.10398
EBNA3	PARVA	0.17484
EBNA3	VCAN	0.10635
EBNA3	CDR2	0.36838
EBNA3	SHFM1	0.39118
EBNA3	KRIT1	0.26231
EBNA3	LINC00523	0.14797
EBNA3	SLC12A1	0.016338
EBNA3	RABAC1	0.34733
EBNA3	LRAT	0.015852
EBNA3	RUNX2	0.058192
EBNA3	AMFR	0.12575

EBNA3	MAP3K13	0.060192
EBNA3	RABGAP1L	0.024566
EBNA3	NEU4	0.21481
EBNA3	INTS8	0.36808
EBNA3	CNOT6L	0.25203
EBNA3	RPS19BP1	0.35864
EBNA3	COMMD1	0.25143
EBNA3	SEPT2	0.13656
EBNA3	RBM4	0.15611
EBNA3	KPNA3	0.24365
EBNA3	ZNRF1	0.30143
EBNA3	GRB7	0.14894
EBNA3	PLCB1	0.04198
EBNA3	ZSCAN21	0.1392
EBNA3	TBP	0.046168
EBNA3	TESK1	0.23315
EBNA3	CDC42BPG	0.097325
EBNA3	PRMT10	0.049276
EBNA3	CDC25A	0.044742
EBNA3	CABP4	0.1594
EBNA3	GEMIN6	0.28306
EBNA3	MAPK12	0.10649
EBNA3	MMP2	0.11969
EBNA3	SLC1A5	0.022932
EBNA3	INHA	0.13951
EBNA3	CSN2	0.20787
EBNA3	POLE4	0.30303
EBNA3	BAD	0.039277
EBNA3	EIF2B2	0.25898
EBNA3	RPTN	0.22751
EBNA3	EXOSC1	0.32668
EBNA3	DSP	0.14689
EBNA3	RGS19	0.20931
EBNA3	ZFYVE20	0.22076
EBNA3	UBE2G2	0.23974
EBNA3	RMI1	0.38004
EBNA3	AIP	0.14035
EBNA3	S100A13	0.42308
EBNA3	PIK3R6	0.20731
EBNA3	STUB1	0.14743
EBNA3	MEA1	0.37545
EBNA3	MELK	0.34273
EBNA3	EXO1	0.15066
EBNA3	FAM69B	0.22317
EBNA3	GEM	0.23387
EBNA3	NEK1	0.1705
EBNA3	HPGDS	0.15396
EBNA3	MED18	0.26309
EBNA3	BNIP3	0.072498
EBNA3	INHBB	0.23496
EBNA3	HSP90AB1	0.16621
EBNA3	CORO1B	0.36207
EBNA3	HTRA3	0.39659
EBNA3	C9orf152	0.013802
EBNA3	PSMB1	0.10298
EBNA3	S100A1	0.48846
EBNA3	ARL14EPL	0.16094
EBNA3	ZDHHC13	0.014556
EBNA3	EIF3H	0.39292
EBNA3	TMED9	0.27088
EBNA3	GPATCH8	0.31803
EBNA3	PRMT6	0.22079
EBNA3	MYL7	0.20077
EBNA3	SNRPC	0.32851
EBNA3	SUMO1	0.14311
EBNA3	PPIA	0.20794
EBNA3	SUMO2	0.35747
EBNA3	USP1	0.26081

EBNA3	ARHGAP24	0.18541
EBNA3	UTRN	0.3918
EBNA3	TRAF3IP3	0.41166
EBNA3	STAM2	0.2244
EBNA3	EGR2	0.10471
EBNA3	UBE2D2	0.22062
EBNA3	EGFL8	0.20735
EBNA3	GAGE12H	0.061456
EBNA3	GPX4	0.022422
EBNA3	RAD23B	0.2353
EBNA3	HHIP	0.13238
EBNA3	FANCE	0.37075
EBNA3	GNS	0.12983
EBNA3	PCGF5	0.26146
EBNA3	S100A2	0.20017
EBNA3	INTS4L2	0.018631
EBNA3	POLD4	0.20675
EBNA3	RPL15	0.2912
EBNA3	PALM	0.18477
EBNA3	IDE	0.054059
EBNA3	AP2A2	0.21814
EBNA3	ASH2L	0.21561
EBNA3	DOHH	0.25177
EBNA3	IGF1	0.033208
EBNA3	SLC34A1	0.31023
EBNA3	SLN	0.14396
EBNA3	TRAF3IP2	0.3542
EBNA3	SCUBE2	0.20437
EBNA3	TBC1D2B	0.22014
EBNA3	MCM7	0.17339
EBNA3	TOP2A	0.095856
EBNA3	SGOL2	0.25052
EBNA3	PIK3CA	0.12942
EBNA3	MYL9	0.14613
EBNA3	NOP56	0.36535
EBNA3	KIAA0101	0.32628
EBNA3	TNFRSF1B	0.26658
EBNA3	BAHD1	0.2623
EBNA3	E4F1	0.07273
EBNA3	SPRY2	0.08021
EBNA3	FAM219B	0.014138
EBNA3	ENG	0.074926
EBNA3	MYO18A	0.28098
EBNA3	CXorf21	0.058504
EBNA3	RARA	0.012688
EBNA3	HAND1	0.03834
EBNA3	ACP1	0.12547
EBNA3	AQP1	0.074766
EBNA3	MAPKAPK3	0.10659
EBNA3	ELN	0.28593
EBNA3	SWI5	0.38815
EBNA3	LTF	0.1925
EBNA3	PPM1B	0.221
EBNA3	TBC1D10A	0.31863
EBNA3	EFHD1	0.19194
EBNA3	MAP2K3	0.091983
EBNA3	SMC2	0.23038
EBNA3	ZC3HAV1	0.27379
EBNA3	TMEM88	0.16293
EBNA3	SAMM50	0.25165
EBNA3	FASLG	0.10646
EBNA3	VWF	0.207
EBNA3	TMBIM4	0.30082
EBNA3	SAP30BP	0.28938
EBNA3	ZFPM2	0.12069
EBNA3	EEF2K	0.081289
EBNA3	CIB4	0.21072
EBNA3	TSG101	0.12112

EBNA3	CRADD	0.23953
EBNA3	EDN1	0.012324
EBNA3	SPTBN1	0.14462
EBNA3	EDEM2	0.11731
EBNA3	KANK1	0.20142
EBNA3	NUP37	0.20164
EBNA3	CHGA	0.1335
EBNA3	FLII	0.25958
EBNA3	FAM127A	0.043741
EBNA3	EMD	0.21202
EBNA3	C17orf66	0.036861
EBNA3	ECHS1	0.21613
EBNA3	NTRK1	0.089328
EBNA3	CNBD1	0.010136
EBNA3	RBM17	0.39809
EBNA3	THBS3	0.14811
EBNA3	CUBN	0.064092
EBNA3	PSPH	0.09901
EBNA3	S100A14	0.1333
EBNA3	STAT6	0.26756
EBNA3	MPZL2	0.33214
EBNA3	INVS	0.26716
EBNA3	DTNB	0.13937
EBNA3	REST	0.015982
EBNA3	USP4	0.24901
EBNA3	ATG3	0.25261
EBNA3	PRRG3	0.18648
EBNA3	ULK1	0.09741
EBNA3	LLGL1	0.088638
EBNA3	SPOCK2	0.1873
EBNA3	HYAL2	0.070038
EBNA3	PIH1D2	0.10183
EBNA3	STAMBP	0.19718
EBNA3	CTLA4	0.31339
EBNA3	SLIT3	0.097495
EBNA3	ARPC5	0.28428
EBNA3	NELFE	0.26646
EBNA3	CLEC1B	0.35339
EBNA3	FOXP3	0.011491
EBNA3	ATRAID	0.24299
EBNA3	FBXO4	0.30022
EBNA3	HNRNPL	0.28845
EBNA3	TLE2	0.2587
EBNA3	POM121L12	0.073877
EBNA3	PPP2R2A	0.27104
EBNA3	AKAP5	0.066787
EBNA3	CALB1	0.43841
EBNA3	DAZAP2	0.36838
EBNA3	CPSF2	0.33597
EBNA3	MAP4K1	0.21524
EBNA3	PCGF6	0.14085
EBNA3	PEX5	0.15063
EBNA3	RYK	0.20914
EBNA3	RASSF6	0.39878
EBNA3	NDEL1	0.13604
EBNA3	HHLA3	0.43795
EBNA3	NPHS2	0.33012
EBNA3	DIP2A	0.24114
EBNA3	GAN	0.3568
EBNA3	PDIA4	0.19927
EBNA3	MIER1	0.24905
EBNA3	MAP3K11	0.13316
EBNA3	BAZ2A	0.20686
EBNA3	CAPN11	0.17787
EBNA3	SFTPC	0.24604
EBNA3	UFD1L	0.32609
EBNA3	LIN7A	0.24991
EBNA3	SETMAR	0.22949

EBNA3	VAV3	0.096083
EBNA3	MASP2	0.56729
EBNA3	EPN3	0.015077
EBNA3	BATF2	0.21624
EBNA3	NUDT21	0.30707
EBNA3	PCDHA9	0.18006
EBNA3	PARK7	0.22243
EBNA3	RP2	0.19653
EBNA3	FKBP1A	0.17005
EBNA3	TAF6L	0.17487
EBNA3	SLC25A23	0.18081
EBNA3	TRAF7	0.23385
EBNA3	BCL2L10	0.27497
EBNA3	RCN2	0.19756
EBNA3	PRCP	0.37859
EBNA3	SUCLA2	0.17676
EBNA3	TAOK3	0.21257
EBNA3	SSTR2	0.17143
EBNA3	PPP4C	0.20579
EBNA3	HSPA1A	0.12676
EBNA3	TAP1	0.12097
EBNA3	ATG9A	0.36427
EBNA3	CALML4	0.21072
EBNA3	NDUFAF1	0.35895
EBNA3	COG5	0.24717
EBNA3	MAP3K5	0.068611
EBNA3	LINC00472	0.099554
EBNA3	FRA10AC1	0.39189
EBNA3	PES1	0.22941
EBNA3	CCM2	0.36467
EBNA3	BBIP1	0.37179
EBNA3	TRIM39	0.22564
EBNA3	ABI1	0.15733
EBNA3	PTBP1	0.33342
EBNA3	SOST	0.21592
EBNA3	MAGEA3	0.43795
EBNA3	C20orf96	0.020641
EBNA3	ZNF224	0.23607
EBNA3	GOLGA6L4	0.1116
EBNA3	RAMP2	0.12924
EBNA3	GOLGA6L6	0.10528
EBNA3	TAF13	0.117
EBNA3	KIAA1958	0.050283
EBNA3	SLAIN1	0.15727
EBNA3	CAPSL	0.20436
EBNA3	RRAS2	0.23229
EBNA3	RAB4A	0.19698
EBNA3	FGF19	0.32167
EBNA3	DDN	0.29848
EBNA3	RAB5B	0.25584
EBNA3	RNF32	0.32305
EBNA3	ARL14	0.45243
EBNA3	C9orf169	0.034294
EBNA3	LRWD1	0.23988
EBNA3	TXNDC9	0.40042
EBNA3	ARL6	0.15254
EBNA3	EIF3C	0.40527
EBNA3	EIF3M	0.4387
EBNA3	YPL025C	0.048831
EBNA3	ZNF326	0.3091
EBNA3	KIAA0226L	0.013944
EBNA3	STEAP1	0.050686
EBNA3	MYOZ3	0.41953
EBNA3	KLHL2	0.39066
EBNA3	DTX2	0.24521
EBNA3	COMMD10	0.43795
EBNA3	NOM1	0.41117
EBNA3	ACVR2A	0.058125

EBNA3	GRK5	0.10554
EBNA3	FANCC	0.27062
EBNA3	FRAT2	0.12956
EBNA3	RFX3	0.10969
EBNA3	ITGB1BP2	0.13923
EBNA3	CENPA	0.1422
EBNA3	MDFIC	0.1724
EBNA3	UBXN6	0.31856
EBNA3	SYNPO2	0.34811
EBNA3	BABAM1	0.31468
EBNA3	IL5RA	0.34614
EBNA3	AIF1L	0.1439
EBNA3	PON1	0.02903
EBNA3	LPCAT4	0.12287
EBNA3	CDH11	0.1523
EBNA3	RABL6	0.31943
EBNA3	APEX1	0.11287
EBNA3	ELK3	0.11317
EBNA3	CBLL1	0.26013
EBNA3	PCDHAC1	0.16911
EBNA3	EPPIN	0.36645
EBNA3	GAS6	0.050303
EBNA3	BET1	0.24818
EBNA3	ANXA2R	0.13381
EBNA3	AMPH	0.30771
EBNA3	ABLIM1	0.27765
EBNA3	RANGAP1	0.17134
EBNA3	CC2D2B	0.047026
EBNA3	CIRBP	0.28104
EBNA3	YML009W-B	0.15017
EBNA3	C14orf178	0.055925
EBNA3	ERLEC1	0.38631
EBNA3	MEF2C	0.01216
EBNA3	LATS1	0.069606
EBNA3	KIFAP3	0.25898
EBNA3	MED9	0.26309
EBNA3	ABCB1	0.21942
EBNA3	PCDHB6	0.15424
EBNA3	EFEMP1	0.53144
EBNA3	EIF4A3	0.23352
EBNA3	LPAR2	0.30421
EBNA3	ZAP70	0.13605
EBNA3	DAXX	0.11396
EBNA3	NIPA1	0.11729
EBNA3	IL1A	0.14443
EBNA3	BID	0.13086
EBNA3	COMMD2	0.43795
EBNA3	KANSL1	0.33278
EBNA3	SDPR	0.27544
EBNA3	AGRN	0.12334
EBNA3	MKL1	0.24463
EBNA3	ZNF281	0.096408
EBNA3	TTC37	0.35718
EBNA3	PNMA1	0.31934
EBNA3	APC2	0.26062
EBNA3	CCDC53	0.41536
EBNA3	C1orf168	0.068817
EBNA3	NIPSNAP3A	0.41224
EBNA3	SPATA13	0.13947
EBNA3	MYO9B	0.22461
EBNA3	SLC6A17	0.027831
EBNA3	C1D	0.27295
EBNA3	BAP1	0.17928
EBNA3	CIR1	0.15101
EBNA3	YTHDF1	0.069711
EBNA3	ULK3	0.19759
EBNA3	TBCB	0.33233
EBNA3	SPARC	0.2754

EBNA3	GTF2H1	0.10725
EBNA3	CREB5	0.14369
EBNA3	TCHHL1	0.21072
EBNA3	EWSR1	0.31217
EBNA3	F2RL1	0.052528
EBNA3	OC90	0.16614
EBNA3	MRFAP1L1	0.13721
EBNA3	EHMT2	0.2415
EBNA3	PALLD	0.28938
EBNA3	SRSF11	0.34948
EBNA3	PCSK7	0.012228
EBNA3	SMAGP	0.045842
EBNA3	NOTCH4	0.21503
EBNA3	RAPGEF3	0.20384
EBNA3	PIK3C3	0.19105
EBNA3	TERT	0.20464
EBNA3	NUTF2	0.35629
EBNA3	ARPC4	0.3012
EBNA3	EXOSC10	0.17403
EBNA3	FOXO1	0.02294
EBNA3	PCDHB1	0.18006
EBNA3	MEAF6	0.26577
EBNA3	ANG	0.052793
EBNA3	KLHL12	0.41081
EBNA3	TSPYL5	0.28528
EBNA3	TPH2	0.010573
EBNA3	PIM1	0.10335
EBNA3	CNPY1	0.01565
EBNA3	DLX4	0.19588
EBNA3	ADARB1	0.010289
EBNA3	MBD2	0.19287
EBNA3	MST4	0.11593
EBNA3	FAM160A1	0.14962
EBNA3	TRAIP	0.27267
EBNA3	DFNB31	0.2154
EBNA3	ATP5G3	0.013825
EBNA3	CYTH2	0.22837
EBNA3	CTR9	0.19515
EBNA3	NAA15	0.22165
EBNA3	MATN1	0.40171
EBNA3	NOTCH2NL	0.18082
EBNA3	CA4	0.15529
EBNA3	SLC13A1	0.027466
EBNA3	DEPDC1	0.26232
EBNA3	ID4	0.21308
EBNA3	KIAA1279	0.37133
EBNA3	PCDH17	0.65766
EBNA3	GLI2	0.017462
EBNA3	CSNK2A2	0.14507
EBNA3	PIR	0.35729
EBNA3	IGSF8	0.34005
EBNA3	EPHA4	0.10658
EBNA3	CSRNP2	0.16274
EBNA3	SET	0.1524
EBNA3	MCL1	0.18529
EBNA3	PRICKLE1	0.21414
EBNA3	FGFBP1	0.27868
EBNA3	GPR37L1	0.32396
EBNA3	ARHGEF2	0.066193
EBNA3	SLC9A1	0.14355
EBNA3	KITLG	0.17884
EBNA3	MARCH11	0.012737
EBNA3	DSC3	0.15013
EBNA3	SLC22A3	0.23142
EBNA3	WHAMMP3	0.017197
EBNA3	B2M	0.17997
EBNA3	GLRB	0.17541
EBNA3	KCNN4	0.25623

EBNA3	PRAMEF13	0.15917
EBNA3	PHB	0.15368
EBNA3	PRDX4	0.42023
EBNA3	CCDC85B	0.25074
EBNA3	NR5A1	0.052739
EBNA3	APC	0.02235
EBNA3	PMS2	0.21203
EBNA3	CLSTN1	0.061259
EBNA3	SRRM1	0.15746
EBNA3	XK	0.40885
EBNA3	SCAF11	0.28174
EBNA3	EXOC3	0.39261
EBNA3	EIF3B	0.41871
EBNA3	CLGN	0.12956
EBNA3	CIB2	0.21072
EBNA3	LDB3	0.314
EBNA3	PPM1D	0.17777
EBNA3	PLK1	0.026191
EBNA3	KHDRBS3	0.3354
EBNA3	ERBB4	0.045373
EBNA3	EXD3	0.43795
EBNA3	ADH1A	0.27917
EBNA3	SPEN	0.15464
EBNA3	CEP72	0.30341
EBNA3	ALB	0.11663
EBNA3	GGT1	0.24924
EBNA3	YJR030C	0.091133
EBNA3	NAP1L1	0.29823
EBNA3	SRSF1	0.2805
EBNA3	KIAA1841	0.08547
EBNA3	EAF2	0.25761
EBNA3	CASK	0.14101
EBNA3	ESRRG	0.14513
EBNA3	C1orf196	0.048855
EBNA3	RNF216P1	0.076168
EBNA3	TARDBP	0.17725
EBNA3	RASSF4	0.41136
EBNA3	REPS1	0.66083
EBNA3	DCUN1D2	0.01515
EBNA3	IL15RA	0.33406
EBNA3	PLEK	0.12276
EBNA3	CIRH1A	0.30243
EBNA3	TYRO3	0.14074
EBNA3	LINC00476	0.019171
EBNA3	RUFY1	0.30459
EBNA3	STPG2	0.017714
EBNA3	PEX2	0.21713
EBNA3	PRH1	0.41198
EBNA3	SLC18A2	0.045656
EBNA3	TNFRSF10A	0.25811
EBNA3	POP5	0.28752
EBNA3	NSD1	0.032039
EBNA3	GRIP1	0.014394
EBNA3	TRPS1	0.12177
EBNA3	LRRC26	0.32653
EBNA3	BBS5	0.27705
EBNA3	PRODH	0.078677
EBNA3	YJEFN3	0.052454
EBNA3	C18orf21	0.057143
EBNA3	SIVA1	0.20662
EBNA3	NLGN3	0.14997
EBNA3	GCC1	0.31946
EBNA3	AURKAIP1	0.30396
EBNA3	CYTIP	0.39554
EBNA3	XAGE2	0.15445
EBNA3	USP14	0.22542
EBNA3	PCDHGA9	0.18006
EBNA3	YML009W-B	0.051338

EBNA3	ELOVL3	0.26816
EBNA3	EVI5	0.2447
EBNA3	RASA1	0.10663
EBNA3	PALB2	0.30753
EBNA3	CAND2	0.30116
EBNA3	FABP3	0.33276
EBNA3	THUMPD3	0.045962
EBNA3	LRCOL1	0.12936
EBNA3	CDHR5	0.15311
EBNA3	AHI1	0.12573
EBNA3	MRPS27	0.34367
EBNA3	ZBTB16	0.10739
EBNA3	SAT1	0.33232
EBNA3	DTYMK	0.091425
EBNA3	DCTN2	0.21745
EBNA3	TBC1D8B	0.15086
EBNA3	TUBGCP3	0.14849
EBNA3	AGO1	0.2404
EBNA3	GOLGA6L18	0.093196
EBNA3	CD53	0.36354
EBNA3	USP25	0.27088
EBNA3	AFF1	0.21603
EBNA3	SH3GL2	0.21915
EBNA3	TULP1	0.23562
EBNA3	NXF5	0.39253
EBNA3	SNX1	0.24693
EBNA3	AQP2	0.037504
EBNA3	CD151	0.32112
EBNA3	MDFI	0.21107
EBNA3	YTHDC1	0.35571
EBNA3	MRPL28	0.34456
EBNA3	KRT16	0.29864
EBNA3	GUCA1C	0.21228
EBNA3	MAP2K7	0.040516
EBNA3	SMAD1	0.015377
EBNA3	DOCK1	0.23686
EBNA3	CASP10	0.30234
EBNA3	MRPS10	0.12984
EBNA3	MRPS9	0.29713
EBNA3	GEMIN7	0.28288
EBNA3	TBC1D7	0.36693
EBNA3	CDH7	0.16097
EBNA3	SPI1	0.066856
EBNA3	PPP1R1A	0.38333
EBNA3	SEC24C	0.12794
EBNA3	PRKCSH	0.21429
EBNA3	ZCCHC11	0.22894
EBNA3	LAMP5	0.072917
EBNA3	TGFBR3	0.070993
EBNA3	ASUN	0.34037
EBNA3	AMY1A	0.24411
EBNA3	GRAP2	0.28731
EBNA3	EXOC7	0.31626
EBNA3	UPF1	0.2036
EBNA3	STX16	0.22295
EBNA3	TXNIP	0.16163
EBNA3	ANKRD28	0.37995
EBNA3	DRD4	0.093413
EBNA3	FGF1	0.13678
EBNA3	ITPR3	0.063256
EBNA3	MMP1	0.050419
EBNA3	GAGE2D	0.4246
EBNA3	NEK11	0.17911
EBNA3	ARL13B	0.35702
EBNA3	ASAP1	0.099424
EBNA3	ADAMTS10	0.20644
EBNA3	YLPM1	0.30121
EBNA3	BCORP1	0.056087

EBNA3	BRAT1	0.37995
EBNA3	SIGIRR	0.30899
EBNA3	TAS2R16	0.28594
EBNA3	ADRA2C	0.1788
EBNA3	PCDHGA2	0.18006
EBNA3	FAM160B2	0.037031
EBNA3	RDX	0.26426
EBNA3	ATP5H	0.040603
EBNA3	TYR	0.24387
EBNA3	POU3F2	0.10423
EBNA3	ITGB4	0.19375
EBNA3	KAL1	0.2718
EBNA3	ZXDC	0.12652
EBNA3	SSR4	0.22051
EBNA3	RNASEH2A	0.11941
EBNA3	DDIT3	0.093501
EBNA3	GTF3C6	0.35777
EBNA3	WIBG	0.41442
EBNA3	ARHGAP22	0.143
EBNA3	PCDH1	0.1533
EBNA3	FAM221B	0.097409
EBNA3	PLCL1	0.022409
EBNA3	SLC40A1	0.22045
EBNA3	FBXW5	0.19378
EBNA3	PDE6H	0.10334
EBNA3	CFLAR	0.26541
EBNA3	EIF4EBP1	0.21436
EBNA3	MED29	0.29628
EBNA3	CDKN3	0.10826
EBNA3	TAX1BP1	0.3027
EBNA3	RPL18	0.053856
EBNA3	NOTCH2	0.14891
EBNA3	LOXL3	0.25322
EBNA3	ERLIN2	0.32027
EBNA3	EFCAB12	0.21072
EBNA3	CDH6	0.12868
EBNA3	FZR1	0.21821
EBNA3	ROR1	0.28503
EBNA3	MPTX1	0.039049
EBNA3	AMER2	0.3977
EBNA3	MITD1	0.31263
EBNA3	TAF1D	0.31984
EBNA3	SLC4A1AP	0.31678
EBNA3	SIAH2	0.17149
EBNA3	EXOSC3	0.25872
EBNA3	STIM1	0.36693
EBNA3	COG4	0.29591
EBNA3	S100B	0.35946
EBNA3	TRAF3IP1	0.41166
EBNA3	CCDC172	0.020543
EBNA3	ZAK	0.11243
EBNA3	FZD6	0.1337
EBNA3	LDOC1L	0.033447
EBNA3	EFHC1	0.11432
EBNA3	KAT6B	0.15192
EBNA3	HMG20B	0.1659
EBNA3	ESRRA	0.10158
EBNA3	AC3.5	0.066658
EBNA3	SKP1	0.25502
EBNA3	VAMP1	0.35036
EBNA3	BIRC2	0.11244
EBNA3	ZNF212	0.22143
EBNA3	LINS	0.035573
EBNA3	TNNC1	0.53633
EBNA3	DDB1	0.21365
EBNA3	RBMX	0.20348
EBNA3	SAP30	0.26561
EBNA3	ZNF24	0.15293

EBNA3	SHCBP1	0.2588
EBNA3	CHD3	0.13004
EBNA3	ADCYAP1R1	0.10174
EBNA3	CRYGC	0.33383
EBNA3	COX10	0.11429
EBNA3	EFCAB4A	0.19222
EBNA3	ERBB2	0.034783
EBNA3	HTATIP2	0.22192
EBNA3	STEAP3	0.24384
EBNA3	TXLNA	0.35705
EBNA3	POLRMT	0.31645
EBNA3	BCAS2	0.3384
EBNA3	TMED2	0.2589
EBNA3	C14orf80	0.12043
EBNA3	RAB3IL1	0.36864
EBNA3	NBPF24	0.02828
EBNA3	CCNC	0.13469
EBNA3	C1orf94	0.43795
EBNA3	CASP6	0.13727
EBNA3	VCL	0.14387
EBNA3	PCDHB13	0.16421
EBNA3	CETN2	0.37106
EBNA3	CAMSAP2	0.38694
EBNA3	ANXA6	0.60683
EBNA3	PCDHGC4	0.18006
EBNA3	LCP1	0.11643
EBNA3	DIXDC1	0.16467
EBNA3	SIRPG	0.29003
EBNA3	C9orf153	0.034381
EBNA3	PA2G4	0.13989
EBNA3	TINF2	0.22668
EBNA3	FBXO44	0.41955
EBNA3	ITM2B	0.14408
EBNA3	ANXA2P2	0.1986
EBNA3	CUL4B	0.30607
EBNA3	KPNB1	0.17279
EBNA3	FEZ1	0.25507
EBNA3	CBX4	0.17385
EBNA3	FKBP9	0.18577
EBNA3	NDC80	0.21959
EBNA3	ATP10D	0.22083
EBNA3	SMG5	0.29069
EBNA3	STK38L	0.10918
EBNA3	ACTN2	0.20839
EBNA3	COL3A1	0.17267
EBNA3	GGA1	0.30698
EBNA3	LRCH4	0.40761
EBNA3	EFNA1	0.18618
EBNA3	ATF5	0.15091
EBNA3	CNOT7	0.11366
EBNA3	HGD	0.096747
EBNA3	SEPN1	0.3514
EBNA3	IFIT3	0.30516
EBNA3	PRSS2	0.49894
EBNA3	STAT2	0.28336
EBNA3	PRDM5	0.18335
EBNA3	FSHB	0.24662
EBNA3	PSRC1	0.23441
EBNA3	REEP1	0.31966
EBNA3	PIFO	0.23552
EBNA3	METTL9	0.43795
EBNA3	CD59	0.31233
EBNA3	NCKAP1L	0.11999
EBNA3	LIMS1	0.27226
EBNA3	ZNF219	0.11304
EBNA3	ACTG1	0.14351
EBNA3	NCKAP1	0.23018
EBNA3	CT45A5	0.040327

EBNA3	EIF4A2	0.304
EBNA3	PNRC2	0.28666
EBNA3	C2orf78	0.05292
EBNA3	PDE4DIP	0.31419
EBNA3	RAB27A	0.13354
EBNA3	SESTD1	0.30772
EBNA3	FKBP4	0.14002
EBNA3	C21orf58	0.044135
EBNA3	KANK2	0.29017
EBNA3	RPL5	0.19676
EBNA3	TECPR2	0.43795
EBNA3	CDCA5	0.26081
EBNA3	HAX1	0.3168
EBNA3	STAU2	0.35703
EBNA3	SIRT1	0.014266
EBNA3	NOS3	0.03776
EBNA3	PITPNM3	0.57394
EBNA3	SEPSECS	0.25598
EBNA3	HEMK1	0.043578
EBNA3	VIL1	0.26254
EBNA3	TNIP2	0.11203
EBNA3	ADCY6	0.061675
EBNA3	S100A7L2	0.17787
EBNA3	RNF126	0.36919
EBNA3	EIF5B	0.31548
EBNA3	C19orf35	0.10026
EBNA3	STX11	0.36724
EBNA3	FSTL3	0.16075
EBNA3	ELK4	0.17147
EBNA3	WEE1	0.11964
EBNA3	PSMA6	0.10169
EBNA3	C4orf17	0.010973
EBNA3	PCDHA3	0.16911
EBNA3	MUC5B	0.3514
EBNA3	GAGE6	0.031848
EBNA3	CIB3	0.21072
EBNA3	CCDC59	0.33039
EBNA3	CD44	0.15301
EBNA3	PPID	0.38986
EBNA3	AGL	0.16073
EBNA3	SMNDC1	0.38093
EBNA3	AATF	0.13855
EBNA3	TFF2	0.30235
EBNA3	NEDD9	0.23153
EBNA3	CNOT1	0.27056
EBNA3	RPL14	0.26519
EBNA3	YIR021W-A	0.035314
EBNA3	PTPN9	0.17564
EBNA3	ATF7	0.096881
EBNA3	GUCY2C	0.11726
EBNA3	HPCA	0.19819
EBNA3	SMC5	0.2519
EBNA3	ZRANB1	0.19676
EBNA3	COMMD9	0.082427
EBNA3	DNASE1L2	0.16562
EBNA3	PTPRO	0.087977
EBNA3	KCNJ4	0.37027
EBNA3	ANK3	0.13297
EBNA3	SYPL1	0.11386
EBNA3	DDX6	0.28139
EBNA3	APOBEC1	0.16105
EBNA3	ERLIN1	0.35135
EBNA3	ANGEL2	0.026599
EBNA3	NLRP3	0.1928
EBNA3	ZNF473	0.23124
EBNA3	RAD9B	0.37416
EBNA3	AHSA1	0.33443
EBNA3	GTF2A2	0.17032

EBNA3	VSIG4	0.37004
EBNA3	TOP1	0.16611
EBNA3	DNMT3B	0.15921
EBNA3	RAC3	0.15473
EBNA3	MIR7-3HG	0.022213
EBNA3	IGF2R	0.23523
EBNA3	SORCS1	0.37517
EBNA3	PYURF	0.36193
EBNA3	HRG	0.11012
EBNA3	ORC3	0.27868
EBNA3	SCYL3	0.33829
EBNA3	FDFT1	0.033516
EBNA3	CCL18	0.3265
EBNA3	ARPC3	0.28298
EBNA3	SIT1	0.30023
EBNA3	NUAK1	0.20076
EBNA3	TXN	0.089574
EBNA3	PP632	0.024039
EBNA3	DIABLO	0.27502
EBNA3	C14orf166	0.20636
EBNA3	CDC25C	0.041093
EBNA3	PIK3R4	0.20155
EBNA3	C17orf104	0.026806
EBNA3	XRCC1	0.28975
EBNA3	CETN1	0.50359
EBNA3	PRO1854	0.030004
EBNA3	UBQLN4	0.29965
EBNA3	KCNJ9	0.36419
EBNA3	LPP	0.26383
EBNA3	AKAP12	0.31151
EBNA3	ZBTB25	0.13874
EBNA3	BCOR	0.17515
EBNA3	TTBK2	0.25179
EBNA3	GAGE4	0.074468
EBNA3	MPHOSPH10	0.24308
EBNA3	MLLT10	0.13442
EBNA3	PFKFB2	0.14378
EBNA3	SGOL1	0.19463
EBNA3	CUL3	0.14641
EBNA3	NTNG1	0.34662
EBNA3	MC1R	0.17464
EBNA3	COG7	0.27002
EBNA3	PROCR	0.31235
EBNA3	ERC2	0.31154
EBNA3	ATP5SL	0.017483
EBNA3	ATP1B2	0.27955
EBNA3	ZSWIM7	0.2993
EBNA3	C2CD4D	0.030504
EBNA3	MAP2K2	0.052858
EBNA3	ZK1128.3	0.037976
EBNA3	HLA-A	0.16984
EBNA3	GK	0.30161
EBNA3	YDL022C-A	0.020265
EBNA3	GNB2	0.22385
EBNA3	HSPB9	0.36861
EBNA3	PRMT7	0.028011
EBNA3	PP632	0.032223
EBNA3	PSMC6	0.12437
EBNA3	PHACTR4	0.042301
EBNA3	NDFIP1	0.1565
EBNA3	PGK2	0.033461
EBNA3	STK10	0.2313
EBNA3	ANXA13	0.18783
EBNA3	HTRA2	0.16864
EBNA3	NUP98	0.16818
EBNA3	ANK2	0.059417
EBNA3	NFKBIA	0.067466
EBNA3	LAT	0.17477

EBNA3	SOD3	0.1895
EBNA3	SEPT6	0.21059
EBNA3	MYO5A	0.093705
EBNA3	CIC	0.31984
EBNA3	SLC1A2	0.022375
EBNA3	FAR2	0.010042
EBNA3	COMMD8	0.43795
EBNA3	IGFBP2	0.26198
EBNA3	PNMAL1	0.052811
EBNA3	DUSP2	0.11398
EBNA3	CCR5	0.12469
EBNA3	RPS13	0.20852
EBNA3	TAF8	0.2246
EBNA3	C18orf42	0.013363
EBNA3	IMMP1L	0.011951
EBNA3	CRHR2	0.22538
EBNA3	IL1RAPL1	0.20476
EBNA3	PBX1	0.04856
EBNA3	HPN	0.1731
EBNA3	VLDLR	0.20425
EBNA3	NUP62	0.091643
EBNA3	INPPL1	0.12691
EBNA3	TRIP13	0.2005
EBNA3	CDH1	0.10432
EBNA3	HTT	0.051705
EBNA3	DNAJB4	0.31466
EBNA3	GAGE13	0.031848
EBNA3	ADAMTS13	0.27236
EBNA3	BCAP31	0.13104
EBNA3	DIAPH1	0.14337
EBNA3	TEAD1	0.18012
EBNA3	SMO	0.016122
EBNA3	BCL6	0.05342
EBNA3	MAP1LC3B	0.35939
EBNA3	USF2	0.13574
EBNA3	UBA5	0.18579
EBNA3	TTN	0.11695
EBNA3	ZDHHC17	0.29331
EBNA3	MICU3	0.19819
EBNA3	CAPNS2	0.19223
EBNA3	AK2	0.0157
EBNA3	NR1H3	0.024072
EBNA3	ABT1	0.29672
EBNA3	NDRG2	0.198
EBNA3	TWIST2	0.0878
EBNA3	FSTL4	0.20437
EBNA3	ZYG11A	0.014799
EBNA3	POLB	0.13576
EBNA3	ACTR3	0.10175
EBNA3	CXorf30	0.084418
EBNA3	BACE1	0.063766
EBNA3	DCPS	0.18172
EBNA3	TUT1	0.24981
EBNA3	PSMC5	0.099443
EBNA3	NCALD	0.17121
EBNA3	MUS81	0.24303
EBNA3	PRDM16	0.15486
EBNA3	EGF	0.20551
EBNA3	RAPGEF2	0.12394
EBNA3	TFAM	0.16049
EBNA3	TJP1	0.2384
EBNA3	PLA2G3	0.040323
EBNA3	CAMSAP3	0.10409
EBNA3	RPL8	0.058557
EBNA3	CBX1	0.2086
EBNA3	UIMC1	0.23883
EBNA3	HOMER3	0.29316
EBNA3	AJUBA	0.079524

EBNA3	MIEF1	0.30132
EBNA3	TIRAP	0.104
EBNA3	PDK3	0.29092
EBNA3	PALM3	0.31711
EBNA3	FYN	0.076325
EBNA3	PRKAB2	0.11078
EBNA3	AK7	0.053277
EBNA3	USP26	0.31698
EBNA3	PPP1R16B	0.33137
EBNA3	HIVEP1	0.22951
EBNA3	ELOVL5	0.24638
EBNA3	LTBP4	0.45788
EBNA3	BLOC1S6	0.16807
EBNA3	NABP1	0.077634
EBNA3	CCDC132	0.036217
EBNA3	MOAP1	0.39303
EBNA3	RANBP2	0.14614
EBNA3	PRMT2	0.23962
EBNA3	RPS19	0.20789
EBNA3	DCAF16	0.42039
EBNA3	SMARCAD1	0.20076
EBNA3	CDKN1B	0.099072
EBNA3	NPHP4	0.1805
EBNA3	GNB2L1	0.056365
EBNA3	NUP43	0.20164
EBNA3	AMELX	0.22146
EBNA3	BRIP1	0.013262
EBNA3	PLSCR4	0.55973
EBNA3	LMNA	0.17378
EBNA3	INSIG1	0.21128
EBNA3	DNAJC9-AS1	0.1051
EBNA3	HIRIP3	0.37995
EBNA3	CENPF	0.10491
EBNA3	LIN37	0.4116
EBNA3	UBQLNL	0.012212
EBNA3	MPP7	0.32258
EBNA3	VAMP2	0.23661
EBNA3	RBM8A	0.27591
EBNA3	ALKBH3	0.27692
EBNA3	CCDC152	0.015507
EBNA3	ZFAND6	0.25241
EBNA3	KHDC1L	0.012452
EBNA3	SHANK1	0.16102
EBNA3	DCP2	0.2648
EBNA3	CCNK	0.22682
EBNA3	FBXL2	0.36245
EBNA3	SCOC	0.28853
EBNA3	ALDH9A1	0.027557
EBNA3	LAMB3	0.2112
EBNA3	MBD4	0.20486
EBNA3	RXRB	0.11249
EBNA3	FAM45A	0.13637
EBNA3	KIAA1751	0.018255
EBNA3	FBXO25	0.34224
EBNA3	TCP10L2	0.06506
EBNA3	KAT8	0.16489
EBNA3	POLD3	0.14693
EBNA3	NID1	0.15441
EBNA3	MEPE	0.38872
EBNA3	CCDC57	0.011157
EBNA3	GOLGB1	0.32557
EBNA3	SETD3	0.043799
EBNA3	GALNT3	0.081943
EBNA3	HBA1	0.27922
EBNA3	ATP2C1	0.04312
EBNA3	NXF1	0.3046
EBNA3	GMIP	0.27911
EBNA3	C1R	0.22507

EBNA3	TUBG1	0.19592
EBNA3	SLC6A2	0.37034
EBNA3	C1orf106	0.15267
EBNA3	AKR1B10	0.34859
EBNA3	NOP2	0.48074
EBNA3	DNAJC1	0.20862
EBNA3	FCGBP	0.41167
EBNA3	ZC3H11A	0.35798
EBNA3	TSHZ3	0.073587
EBNA3	CASC5	0.1607
EBNA3	SERPINC1	0.27076
EBNA3	FN1	0.17039
EBNA3	GOLGA6L3	0.1116
EBNA3	ACE	0.06406
EBNA3	SRSF2	0.31899
EBNA3	RHOT1	0.33631
EBNA3	WWC2-AS2	0.069765
EBNA3	RPS6KB1	0.031672
EBNA3	DDX39A	0.27211
EBNA3	SAP18	0.26466
EBNA3	SAFB	0.26786
EBNA3	ASB13	0.40422
EBNA3	SYNGR3	0.13319
EBNA3	TEAD4	0.12281
EBNA3	MMP25	0.060929
EBNA3	SNX9	0.33083
EBNA3	NLGN4X	0.16962
EBNA3	COL4A3	0.12231
EBNA3	CITED2	0.044145
EBNA3	CSRP3	0.16572
EBNA3	VPS53	0.35792
EBNA3	TTI1	0.43795
EBNA3	NCBP2	0.16985
EBNA3	ZC3H15	0.31968
EBNA3	IFRD2	0.41354
EBNA3	TNPO1	0.2546
EBNA3	LSM1	0.28537
EBNA3	COPS4	0.3483
EBNA3	ABI3	0.35908
EBNA3	UBXN4	0.30573
EBNA3	NLRP12	0.23603
EBNA3	FBXL18	0.038376
EBNA3	GUCD1	0.0287
EBNA3	CSF2	0.23259
EBNA3	FAM195B	0.036977
EBNA3	PADI6	0.12099
EBNA3	DNAJC10	0.16195
EBNA3	CCDC7	0.057296
EBNA3	YFL013W-A	0.081212
EBNA3	PARN	0.20578
EBNA3	FAM175A	0.33412
EBNA3	ZCCHC8	0.28369
EBNA3	TRAF3	0.084225
EBNA3	RYR2	0.15586
EBNA3	DLAT	0.31723
EBNA3	STRADA	0.063253
EBNA3	ACTR5	0.26872
EBNA3	AXL	0.10077
EBNA3	RAET1E	0.35219
EBNA3	SMC6	0.2861
EBNA3	ESF1	0.15421
EBNA3	PARP9	0.45012
EBNA3	OPRM1	0.12044
EBNA3	GTPBP4	0.17737
EBNA3	CRLF1	0.33596
EBNA3	PRF1	0.38707
EBNA3	KIAA1109	0.34736
EBNA3	CACNA1I	0.29249

EBNA3	TFF1	0.3134
EBNA3	PCDHB3	0.15424
EBNA3	RPS6KA4	0.08115
EBNA3	PPP1R13L	0.18033
EBNA3	HSD17B14	0.3076
EBNA3	EXOC4	0.13894
EBNA3	SEPT12	0.2391
EBNA3	MMP19	0.040277
EBNA3	CUL7	0.25864
EBNA3	TPT1	0.097616
EBNA3	CDC16	0.21493
EBNA3	IL6ST	0.18533
EBNA3	CDC20	0.15638
EBNA3	AK4	0.014265
EBNA3	STRN	0.27109
EBNA3	NSUN5	0.052541
EBNA3	MYO1E	0.24836
EBNA3	SPRY4	0.33929
EBNA3	KRTAP16-1	0.11272
EBNA3	GAGE12F	0.061456
EBNA3	ZNF366	0.22931
EBNA3	DDX20	0.16856
EBNA3	TEN1	0.32674
EBNA3	NKX3-1	0.021697
EBNA3	UBE2V1	0.14301
EBNA3	METTL18	0.049276
EBNA3	TRMT10C	0.42201
EBNA3	OCA2	0.21989
EBNA3	IGF1R	0.07971
EBNA3	PLA2G2C	0.14321
EBNA3	DGKZ	0.21239
EBNA3	SNRPA1	0.30101
EBNA3	LAIR1	0.39913
EBNA3	VOPP1	0.055112
EBNA3	MICU2	0.20444
EBNA3	F2RL2	0.24451
EBNA3	VEGFB	0.16343
EBNA3	NHLRC1	0.23629
EBNA3	GFER	0.33147
EBNA3	SOCS2	0.22426
EBNA3	ZNF423	0.20526
EBNA3	MZT1	0.34405
EBNA3	IGF2BP1	0.15786
EBNA3	RNF144B	0.2439
EBNA3	PPP1R12C	0.4246
EBNA3	MCFD2	0.14796
EBNA3	CCDC28B	0.41144
EBNA3	MYL12B	0.12598
EBNA3	EIF3K	0.33521
EBNA3	PI4KA	0.34955
EBNA3	CBY1	0.2715
EBNA3	SLC25A24	0.18081
EBNA3	SGCE	0.14769
EBNA3	MAP3K4	0.178
EBNA3	GPR143	0.18903
EBNA3	CBS	0.067708
EBNA3	TIMM9	0.23026
EBNA3	LNX1	0.2574
EBNA3	PKD2L2	0.19178
EBNA3	VDAC2	0.19828
EBNA3	IL13	0.17678
EBNA3	USP22	0.15472
EBNA3	SHARPIN	0.1905
EBNA3	OLFM1	0.3664
EBNA3	SART3	0.34246
EBNA3	PRPF40A	0.28779
EBNA3	LSM4	0.28827
EBNA3	WNK3	0.2017

EBNA3	FBXO11	0.20731
EBNA3	SNRNP70	0.32192
EBNA3	LDLRAP1	0.14998
EBNA3	EPHB4	0.21698
EBNA3	PCSK9	0.09687
EBNA3	TRA2B	0.39168
EBNA3	DGKI	0.29192
EBNA3	HSPB7	0.32483
EBNA3	NOP10	0.28996
EBNA3	RB1	0.014458
EBNA3	ELANE	0.2159
EBNA3	RING1	0.15232
EBNA3	ZNF496	0.127
EBNA3	NELFA	0.24373
EBNA3	ACVR1B	0.066894
EBNA3	AP1S2	0.016395
EBNA3	GPRIN3	0.037001
EBNA3	TCEB2	0.21421
EBNA3	TRIM23	0.078889
EBNA3	FAM92B	0.023835
EBNA3	MYPN	0.30828
EBNA3	BRD3	0.27453
EBNA3	SETD2	0.2227
EBNA3	ZRANB2	0.22138
EBNA3	TRIM32	0.15991
EBNA3	PLD2	0.068402
EBNA3	YWHAH	0.21064
EBNA3	C4orf22	0.023154
EBNA3	HEMGN	0.34682
EBNA3	PARP4	0.28455
EBNA3	CNEP1R1	0.25145
EBNA3	EIF2D	0.016358
EBNA3	HNRNPM	0.29442
EBNA3	KRT6A	0.30324
EBNA3	KLRF2	0.37084
EBNA3	AKAP17A	0.33161
EBNA3	APOO	0.089971
EBNA3	PCDHA6	0.16404
EBNA3	SERPINB8	0.35332
EBNA3	MED28	0.26312
EBNA3	PLEC	0.25315
EBNA3	FADD	0.14596
EBNA3	C6orf52	0.013987
EBNA3	TULP3	0.27145
EBNA3	ARRDC3	0.3995
EBNA3	CCDC74A	0.052333
EBNA3	TSC22D4	0.17893
EBNA3	RPS6KA5	0.05847
EBNA3	SERBP1	0.40426
EBNA3	ANP32C	0.010404
EBNA3	TGFBRAP1	0.30218
EBNA3	FHOD1	0.28179
EBNA3	PRR19	0.068474
EBNA3	XRCC4	0.1503
EBNA3	ZFC3H1	0.37119
EBNA3	TP53BP2	0.25145
EBNA3	CCDC67	0.01313
EBNA3	RNF187	0.23077
EBNA3	E2F3	0.14174
EBNA3	ZNF711	0.24118
EBNA3	PTPN2	0.14772
EBNA3	STIP1	0.29523
EBNA3	MALT1	0.11776
EBNA3	SQSTM1	0.036355
EBNA3	SUPT5H	0.14196
EBNA3	ELMO2	0.16683
EBNA3	ATP6V0A2	0.24762
EBNA3	SNCA	0.035438

EBNA3	FBXO27	0.40674
EBNA3	EMR3	0.12214
EBNA3	CARD10	0.35057
EBNA3	UBE3C	0.24741
EBNA3	RANBP6	0.36266
EBNA3	MCIDAS	0.23256
EBNA3	S100A8	0.24178
EBNA3	THAP11	0.2559
EBNA3	XYLB	0.031153
EBNA3	MT-ND3	0.31301
EBNA3	CD70	0.24234
EBNA3	IL2RG	0.26308
EBNA3	DUSP26	0.10056
EBNA3	NID2	0.64123
EBNA3	GAREML	0.16685
EBNA3	PMS2P1	0.019847
EBNA3	SYP	0.13765
EBNA3	MIMI	0.10939
EBNA3	F2R	0.050881
EBNA3	CCNB1	0.069195
EBNA3	CADM1	0.13496
EBNA3	DIS3L	0.33247
EBNA3	NELL1	0.44265
EBNA3	DIRAS3	0.27999
EBNA3	TICRR	0.30747
EBNA3	SULT1C3	0.011607
EBNA3	FAM84B	0.41167
EBNA3	SLX1A	0.27989
EBNA3	ZNF365	0.36138
EBNA3	DEDD	0.24984
EBNA3	ZHX1	0.12757
EBNA3	ANXA1	0.087528
EBNA3	ytzL	0.1972
EBNA3	FAF2	0.36584
EBNA3	CDKN2B	0.12595
EBNA3	PFDN5	0.25206
EBNA3	WDR96	0.069133
EBNA3	ZMYND11	0.18838
EBNA3	CLDN7	0.20885
EBNA3	KRAS	0.12128
EBNA3	PTPRE	0.12874
EBNA3	C3	0.17221
EBNA3	PCDHB16	0.16421
EBNA3	PTPRK	0.070631
EBNA3	VAV1	0.051178
EBNA3	SNTN	0.19826
EBNA3	STT3B	0.054554
EBNA3	SIRT3	0.24398
EBNA3	DAD1	0.078602
EBNA3	ADA	0.058238
EBNA3	CRHBP	0.12877
EBNA3	DNER	0.44245
EBNA3	FBXO17	0.40674
EBNA3	COL17A1	0.27076
EBNA3	PRKAR1A	0.1887
EBNA3	EIF6	0.40111
EBNA3	CD72	0.27359
EBNA3	VSNL1	0.21072
EBNA3	RUVBL2	0.11199
EBNA3	FBXL14	0.40178
EBNA3	PCDHGA6	0.18006
EBNA3	CLN5	0.282
EBNA3	CFL1	0.1636
EBNA3	MBD1	0.14831
EBNA3	MKL2	0.25989
EBNA3	SCNN1G	0.26383
EBNA3	PHLDA1	0.34946
EBNA3	L1TD1	0.013726

EBNA3	C15orf65	0.036191
EBNA3	NDUFS3	0.25949
EBNA3	POU2F1	0.14881
EBNA3	FAM160B1	0.096401
EBNA3	SLC25A10	0.20089
EBNA3	ATP8A1	0.26091
EBNA3	HMG20A	0.18465
EBNA3	TLX1	0.15143
EBNA3	IL17RA	0.29382
EBNA3	CDK4	0.067527
EBNA3	UBE2V2	0.23673
EBNA3	PACSIN2	0.010853
EBNA3	EFCAB2	0.21072
EBNA3	DSCAM	0.26706
EBNA3	MAPKAP1	0.095969
EBNA3	NUCB1	0.18396
EBNA3	SAFB2	0.35304
EBNA3	CYTH1	0.24124
EBNA3	HEYL	0.080959
EBNA3	ELOVL6	0.2635
EBNA3	EPDR1	0.1981
EBNA3	PPARGC1A	0.10177
EBNA3	NRXN1	0.2257
EBNA3	SAMD14	0.013326
EBNA3	PEF1	0.67161
EBNA3	OFD1	0.21485
EBNA3	PARK2	0.010147
EBNA3	CD22	0.35169
EBNA3	RBM15B	0.27718
EBNA3	SHOX	0.17192
EBNA3	NOXA1	0.30977
EBNA3	DUX4L9	0.16003
EBNA3	SP1	0.025986
EBNA3	ZWINT	0.23325
EBNA3	NAPG	0.32526
EBNA3	CT45A4	0.034809
EBNA3	ZFYVE26	0.31888
EBNA3	RBBP8	0.19791
EBNA3	MAX	0.1351
EBNA3	ANXA3	0.11693
EBNA3	FBXL8	0.43795
EBNA3	SNRPE	0.23849
EBNA3	CREBBP	0.02888
EBNA3	CGN	0.36039
EBNA3	XIAP	0.21193
EBNA3	GCHFR	0.22516
EBNA3	PLA2G16	0.17873
EBNA3	TGFB2	0.011802
EBNA3	EZR	0.18501
EBNA3	MFAP1	0.15229
EBNA3	COL16A1	0.32112
EBNA3	PSMA3	0.096971
EBNA3	RAD17	0.24872
EBNA3	FBL	0.39431
EBNA3	TTYH1	0.17933
EBNA3	CNNM3	0.38062
EBNA3	NUPR1L	0.065575
EBNA3	RASGRP2	0.11412
EBNA3	TSSK6	0.18924
EBNA3	MEGF8	0.5391
EBNA3	MCU	0.28213
EBNA3	C10orf82	0.084243
EBNA3	ERICH1-AS1	0.11172
EBNA3	PDGFB	0.03133
EBNA3	PCDHGB5	0.18006
EBNA3	CHCHD6	0.41144
EBNA3	C3orf65	0.068957
EBNA3	CDH13	0.072317

EBNA3	RNF168	0.15097
EBNA3	ATXN1	0.22106
EBNA3	FKBP9L	0.19239
EBNA3	TRIM25	0.075988
EBNA3	GAP43	0.26512
EBNA3	SLC32A1	0.041639
EBNA3	TTYH2	0.3444
EBNA3	BAK1	0.056012
EBNA3	BEND7	0.43795
EBNA3	CHP1	0.21659
EBNA3	PRICKLE3	0.34888
EBNA3	CBY3	0.056311
EBNA3	PAF1	0.2074
EBNA3	REEP5	0.387
EBNA3	ANTXR2	0.29915
EBNA3	MAP4K2	0.14117
EBNA3	NFATC1	0.10942
EBNA3	CYFIP2	0.33248
EBNA3	CSTF2	0.31849
EBNA3	TNK2	0.15829
EBNA3	SYNGR1	0.12533
EBNA3	CAMTA2	0.2029
EBNA3	C1orf228	0.016967
EBNA3	MAPK3	0.037658
EBNA3	ERAP1	0.17571
EBNA3	UBE2E1	0.16593
EBNA3	CHCHD1	0.35872
EBNA3	APTX	0.12393
EBNA3	VPS52	0.36915
EBNA3	MYL12A	0.20749
EBNA3	CACNG2	0.21963
EBNA3	DAP3	0.20439
EBNA3	ZGPAT	0.13847
EBNA3	RASSF3	0.3353
EBNA3	CFTR	0.15781
EBNA3	RAB3IP	0.26642
EBNA3	DYDC2	0.032865
EBNA3	SPG7	0.14463
EBNA3	GNAQ	0.078173
EBNA3	CTSH	0.12569
EBNA3	XPO5	0.28546
EBNA3	ABCD2	0.27233
EBNA3	CD86	0.18353
EBNA3	EGFL6	0.1614
EBNA3	KMT2E	0.20449
EBNA3	IMP4	0.29482
EBNA3	PRPF31	0.29951
EBNA3	NOMO2	0.34542
EBNA3	SYMPK	0.2921
EBNA3	CUL5	0.177
EBNA3	CNST	0.3739
EBNA3	EP400NL	0.1018
EBNA3	PADI3	0.11864
EBNA3	COL6A2	0.2587
EBNA3	FAM203B	0.080765
EBNA3	FOXG1	0.052699
EBNA3	TRAPPC10	0.36291
EBNA3	LIPE	0.13932
EBNA3	HAP1	0.2773
EBNA3	ATP6V1C1	0.27437
EBNA3	PSMB7	0.10298
EBNA3	RXRA	0.023674
EBNA3	PIK3R1	0.016644
EBNA3	AHCYL1	0.30735
EBNA3	TGM2	0.26427
EBNA3	RPLP0	0.22722
EBNA3	NR3C1	0.046984
EBNA3	BAG1	0.20659

EBNA3	ATF7IP	0.26579
EBNA3	MAPK10	0.12005
EBNA3	EPM2A	0.06775
EBNA3	FASN	0.20968
EBNA3	DLL4	0.098559
EBNA3	QTRTD1	0.11174
EBNA3	LEP	0.012193
EBNA3	SEPT7	0.087691
EBNA3	SMUG1	0.26477
EBNA3	HEATR5A	0.058559
EBNA3	MPZL1	0.24115
EBNA3	TTYH3	0.16901
EBNA3	BRCA2	0.078215
EBNA3	LCMT1	0.45808
EBNA3	TRIM72	0.055475
EBNA3	COL1A1	0.074937
EBNA3	EID2	0.22784
EBNA3	ELOVL7	0.27665
EBNA3	RPS3A	0.23391
EBNA3	GPR98	0.31655
EBNA3	PDS5B	0.28482
EBNA3	CRY1	0.11628
EBNA3	NUMBL	0.37219
EBNA3	UBA3	0.14487
EBNA3	ACAA2	0.32476
EBNA3	DLG4	0.047681
EBNA3	OTUD6A	0.033529
EBNA3	LSM6	0.2711
EBNA3	MBIP	0.33455
EBNA3	AHSG	0.017565
EBNA3	E2F4	0.076664
EBNA3	MICU1	0.55121
EBNA3	CALU	0.54894
EBNA3	EIF2S1	0.2897
EBNA3	TBRG1	0.32341
EBNA3	ELF2	0.15489
EBNA3	ATP1B1	0.2492
EBNA3	CLPTM1	0.35241
EBNA3	CEP63	0.31449
EBNA3	ZIC3	0.092982
EBNA3	ISCU	0.092765
EBNA3	RIPK1	0.052152
EBNA3	TICAM1	0.090426
EBNA3	AP2B1	0.17503
EBNA3	KIRREL3	0.35784
EBNA3	GPSM2	0.23296
EBNA3	PSMB5	0.098162
EBNA3	COIL	0.31312
EBNA3	ATXN2	0.19825
EBNA3	LILRB4	0.29743
EBNA3	PADI2	0.15357
EBNA3	XPC	0.21072
EBNA3	CD8A	0.16057
EBNA3	SSX2	0.31063
EBNA3	DCK	0.052885
EBNA3	LENG8	0.43795
EBNA3	PRKRA	0.28727
EBNA3	KLK8	0.32359
EBNA3	PTPN13	0.12631
EBNA3	PPP3CA	0.21076
EBNA3	STRADB	0.12603
EBNA3	MTUS2	0.37347
EBNA3	ANXA8L2	0.17401
EBNA3	CRYL1	0.011784
EBNA3	VWA9	0.031454
EBNA3	FBLN2	0.19532
EBNA3	BCL2A1	0.38696
EBNA3	UNG	0.11555

EBNA3	MTTP	0.12206
EBNA3	PADI4	0.086935
EBNA3	HAND2	0.065625
EBNA3	PMEL	0.27973
EBNA3	TACR1	0.084342
EBNA3	SURF4	0.26155
EBNA3	MYOZ2	0.34724
EBNA3	C16orf72	0.12008
EBNA3	VCP	0.11255
EBNA3	APIP	0.1942
EBNA3	USP28	0.25492
EBNA3	PPP1CA	0.1035
EBNA3	ATF6	0.075111
EBNA3	ITK	0.12549
EBNA3	HOXC13	0.15959
EBNA3	GADD45G	0.2931
EBNA3	SIX1	0.031986
EBNA3	BTG1	0.2175
EBNA3	SEC11B	0.015914
EBNA3	SVOP	0.017885
EBNA3	MCTP1	0.18784
EBNA3	CBFB	0.16239
EBNA3	OTUB1	0.2613
EBNA3	BEND2	0.044498
EBNA3	PRKX	0.14988
EBNA3	MOCS3	0.20238
EBNA3	CCNG1	0.26444
EBNA3	VSTM2L	0.43795
EBNA3	RFX6	0.22379
EBNA3	ZFYVE28	0.29252
EBNA3	FCGR2C	0.3715
EBNA3	AKAP6	0.1781
EBNA3	BZRAP1	0.38434
EBNA3	EIF4B	0.37245
EBNA3	ANXA10	0.19977
EBNA3	PCDHGB3	0.18006
EBNA3	GLS2	0.26277
EBNA3	LAPTM4A	0.1256
EBNA3	RNF40	0.19751
EBNA3	SOX5	0.10805
EBNA3	POP1	0.27369
EBNA3	SELENBP1	0.29071
EBNA3	IL6	0.011723
EBNA3	PVR	0.16437
EBNA3	ARSA	0.055046
EBNA3	NCOR2	0.19548
EBNA3	LRP2	0.22729
EBNA3	VMP1	0.25742
EBNA3	CAPN2	0.35988
EBNA3	PAFAH1B3	0.25447
EBNA3	KRT8	0.28163
EBNA3	SLC23A1	0.23114
EBNA3	OCLN	0.13401
EBNA3	SIRPB1	0.36324
EBNA3	CPSF7	0.34907
EBNA3	ABCG5	0.19165
EBNA3	UNC119	0.3671
EBNA3	ABL1	0.053598
EBNA3	JMJD7	0.011345
EBNA3	POM121L2	0.074982
EBNA3	DGCR8	0.32237
EBNA3	CLIC1	0.22472
EBNA3	GOLGA6L10	0.093196
EBNA3	MKRN3	0.25042
EBNA3	SAMD13	0.01004
EBNA3	TTC1	0.38325
EBNA3	SUPT4H1	0.09305
EBNA3	SREBF2	0.072059

EBNA3	HNRNPC	0.29716
EBNA3	FAM188B	0.16057
EBNA3	TRIM37	0.19656
EBNA3	GRN	0.27458
EBNA3	MISP	0.13693
EBNA3	PABPC4	0.29173
EBNA3	SLC22A11	0.31593
EBNA3	VPS33A	0.25402
EBNA3	RHCG	0.056507
EBNA3	TRIM5	0.18403
EBNA3	MMP17	0.049865
EBNA3	ANTXR1	0.26266
EBNA3	FAM231B	0.086191
EBNA3	POMT2	0.028757
EBNA3	TNFRSF11A	0.15774
EBNA3	NPLOC4	0.24881
EBNA3	AK8	0.055129
EBNA3	NAA10	0.33767
EBNA3	RGS2	0.20029
EBNA3	SELO	0.10275
EBNA3	CTSL	0.23348
EBNA3	MON1B	0.43795
EBNA3	ZNF728	0.011365
EBNA3	TIFA	0.39355
EBNA3	FAM27D1	0.070413
EBNA3	NUCB2	0.47631
EBNA3	TF	0.22292
EBNA3	CBR4	0.35293
EBNA3	RAD9A	0.16719
EBNA3	CBX6	0.2907
EBNA3	UHRF1BP1L	0.099691
EBNA3	MRPL51	0.34374
EBNA3	MSX2	0.043781
EBNA3	CDKN2A	0.080151
EBNA3	SHANK2-AS3	0.03249
EBNA3	PKDREJ	0.16485
EBNA3	PPP1R13B	0.32476
EBNA3	DCC	0.19763
EBNA3	ITGB1	0.016298
EBNA3	LRSAM1	0.22207
EBNA3	SOX1	0.18274
EBNA3	HELZ2	0.1962
EBNA3	UNQ6126/PRO20091	0.051826
EBNA3	EED	0.23464
EBNA3	ASGR1	0.32101
EBNA3	NPC2	0.22529
EBNA3	TLL2	0.050885
EBNA3	HOXA9	0.17332
EBNA3	MAGEA11	0.37995
EBNA3	SH3RF2	0.29749
EBNA3	RAP1GAP	0.22683
EBNA3	HIN1L	0.01153
EBNA3	USH1C	0.20719
EBNA3	PCDH19	0.18006
EBNA3	RNPS1	0.28686
EBNA3	FAM162A	0.31623
EBNA3	SH3GLB1	0.16739
EBNA3	CDON	0.17486
EBNA3	ATXN7L2	0.035027
EBNA3	BCL2L14	0.23173
EBNA3	PINX1	0.17633
EBNA3	PSMD5	0.17667
EBNA3	PEX11A	0.29666
EBNA3	RIPK3	0.10644
EBNA3	YGL014C-A	0.10964
EBNA3	S100A6	0.42141
EBNA3	RP1L1	0.35297
EBNA3	EPHB1	0.12551

EBNA3	AOC1	0.10248
EBNA3	STK16	0.16616
EBNA3	VHL	0.14439
EBNA3	FAT2	0.1413
EBNA3	FBXW2	0.38185
EBNA3	PLCG2	0.0939
EBNA3	SMARCD3	0.098848
EBNA3	RNF20	0.15774
EBNA3	RHBG	0.064295
EBNA3	ZFP36	0.10294
EBNA3	PPP2R1A	0.093107
EBNA3	IPO13	0.33108
EBNA3	AFG3L2	0.11906
EBNA3	HENMT1	0.10145
EBNA3	EPB42	0.24235
EBNA3	WDR6	0.33982
EBNA3	DDC	0.17869
EBNA3	TERF2IP	0.14302
EBNA3	SPRED2	0.25208
EBNA3	ORMDL3	0.35135
EBNA3	TLR4	0.019095
EBNA3	PKHD1	0.20194
EBNA3	RNF19B	0.30168
EBNA3	IKBKAP	0.13648
EBNA3	MED11	0.26309
EBNA3	NUBP1	0.012798
EBNA3	RABGEF1	0.27168
EBNA3	LRP8	0.37515
EBNA3	ZEB1	0.088457
EBNA3	CCDC151	0.022512
EBNA3	NOX5	0.12182
EBNA3	SYNPO	0.26279
EBNA3	MAVS	0.058484
EBNA3	IGFLR1	0.39913
EBNA3	GGNBP2	0.16954
EBNA3	CASP2	0.17061
EBNA3	CT55	0.085561
EBNA3	RFC5	0.17013
EBNA3	PPP6R3	0.35281
EBNA3	KRT14	0.19639
EBNA3	CBFA2T2	0.13655
EBNA3	GABRG2	0.21083
EBNA3	NUAK2	0.1887
EBNA3	NXT1	0.29595
EBNA3	S100P	0.59017
EBNA3	SNAI1	0.11191
EBNA3	FLG	0.11232
EBNA3	BCL10	0.039158
EBNA3	CARD8	0.32076
EBNA3	PLK3	0.10359
EBNA3	SEMA4D	0.10899
EBNA3	SMURF1	0.14207
EBNA3	SIX5	0.1652
EBNA3	TMEM67	0.2568
EBNA3	WASL	0.11058
EBNA3	LRP1B	0.17553
EBNA3	DCAF7	0.34485
EBNA3	SGTA	0.37521
EBNA3	RABEP2	0.16148
EBNA3	SLC18A1	0.071768
EBNA3	XPO6	0.31253
EBNA3	TRAK1	0.2651
EBNA3	SERTAD1	0.33216
EBNA3	PCDHGA5	0.18006
EBNA3	RNF103	0.24973
EBNA3	FCN2	0.068526
EBNA3	CLASP1	0.12394
EBNA3	DMD	0.14189

EBNA3	E2F2	0.10133
EBNA3	RWDD4	0.031632
EBNA3	LCA5	0.33417
EBNA3	INTS4	0.36808
EBNA3	DYNLRB1	0.28576
EBNA3	GYPC	0.37495
EBNA3	SEPT1	0.33446
EBNA3	EGLN2	0.22697
EBNA3	RAF1	0.034094
EBNA3	PRDX6	0.33766
EBNA3	WT1	0.032885
EBNA3	YWHAZ	0.15893
EBNA3	CHAF1A	0.30454
EBNA3	BTF3	0.32186
EBNA3	KIAA0319L	0.39912
EBNA3	SLC22A1	0.22344
EBNA3	CAMKK2	0.062245
EBNA3	DAZ1	0.37227
EBNA3	SEPT9	0.24786
EBNA3	PPP2R5C	0.24988
EBNA3	TAF9	0.1287
EBNA3	PTCRA	0.39907
EBNA3	SETD1B	0.33477
EBNA3	PTGES3L	0.03991
EBNA3	RIMS2	0.29858
EBNA3	TP53INP1	0.26658
EBNA3	F7	0.076495
EBNA3	ELF4	0.15945
EBNA3	ECH1	0.25252
EBNA3	CPS1	0.073234
EBNA3	C4BPA	0.36715
EBNA3	TAPBP	0.13053
EBNA3	RAPGEF1	0.27041
EBNA3	FEM1B	0.27724
EBNA3	ATXN7L3	0.2548
EBNA3	FNBP1	0.37159
EBNA3	KMT2B	0.15988
EBNA3	MNCb-2875	0.03681
EBNA3	CATSPERB	0.1176
EBNA3	FAM127C	0.05011
EBNA3	MCM10	0.27936
EBNA3	KCNB1	0.25529
EBNA3	CASP9	0.24062
EBNA3	NR4A2	0.067054
EBNA3	VRK1	0.18642
EBNA3	ACO1	0.018457
EBNA3	LTN1	0.30363
EBNA3	TEFM	0.22992
EBNA3	MTOR	0.041506
EBNA3	EIF1B	0.062773
EBNA3	PTTG1	0.20829
EBNA3	PLAU	0.21958
EBNA3	SHMT2	0.026419
EBNA3	DZIP1	0.24354
EBNA3	CLUAP1	0.39189
EBNA3	MADD	0.29165
EBNA3	NUP160	0.22679
EBNA3	NUP50	0.25028
EBNA3	TRIO	0.1026
EBNA3	RAB11FIP3	0.33203
EBNA3	PROCA1	0.19153
EBNA3	MOB1B	0.26032
EBNA3	EFCAB4B	0.54585
EBNA3	APP	0.028257
EBNA3	CDHR1	0.13851
EBNA3	HPX	0.21866
EBNA3	CLNS1A	0.2699
EBNA3	UCHL1	0.15039

EBNA3	GAB2	0.15848
EBNA3	ALKBH8	0.35921
EBNA3	PARD6G	0.32379
EBNA3	CAMK1	0.10318
EBNA3	SYBU	0.37277
EBNA3	UTP14A	0.33956
EBNA3	IQGAP2	0.012651
EBNA3	PCDHB8	0.18006
EBNA3	SKAP1	0.036323
EBNA3	C8orf74	0.056281
EBNA3	NOC4L	0.33099
EBNA3	CCDC104	0.035976
EBNA3	YOD1	0.25557
EBNA3	GABPA	0.1231
EBNA3	ARL6IP1	0.41513
EBNA3	TMPRSS6	0.3341
EBNA3	BLOC1S4	0.33071
EBNA3	THAP3	0.37207
EBNA3	XAGE3	0.17318
EBNA3	FAM21C	0.33803
EBNA3	C11orf65	0.031404
EBNA3	GGN	0.15198
EBNA3	S100A7A	0.17252
EBNA3	ZNF256	0.13409
EBNA3	ASAH2B	0.010565
EBNA3	MUC17	0.27936
EBNA3	C1orf111	0.081513
EBNA3	AP2A1	0.16982
EBNA3	NME6	0.039663
EBNA3	TDGF1	0.091578
EBNA3	CSF1	0.038344
EBNA3	ZYX	0.21891
EBNA3	EFCAB3	0.21072
EBNA3	TCL1B	0.01021
EBNA3	ANKZF1	0.35796
EBNA3	PFDN2	0.3031
EBNA3	PRRG2	0.1862
EBNA3	STK11	0.051228
EBNA3	DLGAP2	0.30968
EBNA3	MMP24	0.051434
EBNA3	SMOC2	0.14468
EBNA3	STXBP3	0.20444
EBNA3	TJP3	0.35243
EBNA3	SALL1	0.056829
EBNA3	SERPING1	0.26083
EBNA3	HIP1R	0.25652
EBNA3	THOC1	0.26374
EBNA3	YTHDC2	0.33562
EBNA3	RAD51B	0.28286
EBNA3	CCDC87	0.016873
EBNA3	HNRNPA2B1	0.29272
EBNA3	CCT2	0.23673
EBNA3	FOSL1	0.09463
EBNA3	SETD6	0.39434
EBNA3	MTPAP	0.23514
EBNA3	CASR	0.26067
EBNA3	YWHAE	0.21939
EBNA3	EDEM3	0.11307
EBNA3	TPO	0.18588
EBNA3	GLA	0.15233
EBNA3	LAMP1	0.078193
EBNA3	NDUFA13	0.19114
EBNA3	CHGB	0.4078
EBNA3	ATP2A2	0.021081
EBNA3	PRKD3	0.090899
EBNA3	SLC16A11	0.07036
EBNA3	JMJD8	0.010026
EBNA3	DTNA	0.48022

EBNA3	CMTR2	0.050795
EBNA3	REPS2	0.71037
EBNA3	IMP3	0.3166
EBNA3	TMEM204	0.15007
EBNA3	OPRK1	0.22977
EBNA3	SH3BP4	0.37765
EBNA3	MVP	0.29713
EBNA3	SASS6	0.34137
EBNA3	PPP3R1	0.21486
EBNA3	RRAGB	0.24505
EBNA3	CCDC50	0.4246
EBNA3	CDH24	0.15838
EBNA3	TRIM44	0.35796
EBNA3	ARFGEF1	0.14792
EBNA3	PPEF2	0.37005
EBNA3	EFEMP2	0.65263
EBNA3	DVL3	0.18431
EBNA3	POU5F1	0.039989
EBNA3	EQTN	0.091053
EBNA3	IFNA2	0.2442
EBNA3	MAP4	0.19211
EBNA3	MYLK	0.14839
EBNA3	RLBP1	0.010369
EBNA3	ZC3H13	0.35798
EBNA3	ALX1	0.14042
EBNA3	SART1	0.27039
EBNA3	MT-CO1	0.16263
EBNA3	AUNIP	0.3529
EBNA3	TANK	0.31049
EBNA3	HERC5	0.21117
EBNA3	DLEU1	0.43795
EBNA3	MAGED4	0.012942
EBNA3	IBA57	0.081131
EBNA3	EIF3L	0.40083
EBNA3	STARD13	0.29764
EBNA3	ATCAY	0.26993
EBNA3	MAP1B	0.052726
EBNA3	HNRNPH1	0.27502
EBNA3	IFI6	0.31373
EBNA3	PFN2	0.30189
EBNA3	USP33	0.1682
EBNA3	DPH3	0.30354
EBNA3	ATP2A1	0.21075
EBNA3	ATXN7L3B	0.051461
EBNA3	CCS	0.1996
EBNA3	POGZ	0.2452
EBNA3	SIK2	0.15305
EBNA3	PCDH15	0.081801
EBNA3	TCEA1	0.15539
EBNA3	TRRAP	0.24184
EBNA3	FZD4	0.094702
EBNA3	RPA2	0.14323
EBNA3	EXOSC4	0.21171
EBNA3	CSE1L	0.32491
EBNA3	JUP	0.02331
EBNA3	RNF10	0.19973
EBNA3	SCAMP5	0.25783
EBNA3	PADI1	0.15357
EBNA3	HIST3H3	0.21778
EBNA3	GSK3B	0.014971
EBNA3	CAB39	0.24855
EBNA3	WLS	0.40363
EBNA3	NOC2L	0.20737
EBNA3	ATP6V0A4	0.1769
EBNA3	PPP2R1B	0.42465
EBNA3	ITGA2	0.28521
EBNA3	CEP250	0.16252
EBNA3	EIF3D	0.39292

EBNA3	PDGFRL	0.13217
EBNA3	IFT52	0.089096
EBNA3	CXorf40A	0.43795
EBNA3	HNRNPD	0.20343
EBNA3	CT45A3	0.038676
EBNA3	BCL2L1	0.030009
EBNA3	MYL3	0.13695
EBNA3	AGXT	0.22469
EBNA3	BAG6	0.18354
EBNA3	IL15	0.15487
EBNA3	CHD9	0.2062
EBNA3	NEUROD1	0.036962
EBNA3	CSF2RB	0.28992
EBNA3	RMDN3	0.18315
EBNA3	CD47	0.25398
EBNA3	CHMP3	0.24239
EBNA3	FOS	0.039511
EBNA3	PBK	0.27728
EBNA3	RFC4	0.17343
EBNA3	HOOK2	0.24656
EBNA3	FOXO3	0.017027
EBNA3	ZC3H12A	0.24212
EBNA3	CCP110	0.3213
EBNA3	SMG7	0.27991
EBNA3	CD200	0.33526
EBNA3	LSM2	0.16354
EBNA3	AP3M1	0.32673
EBNA3	DYNC1I1	0.20589
EBNA3	ZNF703	0.16715
EBNA3	CAGE1	0.024271
EBNA3	NFYB	0.1654
EBNA3	SH3GL3	0.33822
EBNA3	DMBT1	0.16463
EBNA3	MINK1	0.13258
EBNA3	PKN2	0.094567
EBNA3	MECOM	0.073926
EBNA3	LONP1	0.18829
EBNA3	PDCL3	0.37518
EBNA3	LINC00205	0.07381
EBNA3	JAM3	0.19462
EBNA3	ASB16	0.40422
EBNA3	MLH3	0.1973
EBNA3	ARHGAP17	0.2925
EBNA3	PKD2L1	0.3644
EBNA3	MOB1A	0.3732
EBNA3	FAM46B	0.071896
EBNA3	APOL1	0.2931
EBNA3	ADRB3	0.1245
EBNA3	TCEB1	0.20191
EBNA3	VPS4B	0.17487
EBNA3	FEZ2	0.38428
EBNA3	NFYA	0.16759
EBNA3	RNF111	0.15896
EBNA3	PNAS-138	0.088581
EBNA3	DROSHA	0.22768
EBNA3	CD2BP2	0.32781
EBNA3	NINL	0.17054
EBNA3	CD2	0.25332
EBNA3	ACOT8	0.17771
EBNA3	TPPP3	0.18972
EBNA3	NCOA6	0.14204
EBNA3	SMOC1	0.16086
EBNA3	SPPL2B	0.20569
EBNA3	ATR	0.16669
EBNA3	DMPK	0.060377
EBNA3	HTN1	0.37615
EBNA3	TRIM3	0.28053
EBNA3	CCDC26	0.081707

EBNA3	EDC4	0.23419
EBNA3	NSUN5P2	0.069279
EBNA3	PAX9	0.27223
EBNA3	ZNF408	0.22143
EBNA3	BTN3A1	0.36145
EBNA3	BIK	0.25033
EBNA3	PLEKHA1	0.13202
EBNA3	DAB2	0.13954
EBNA3	OR2L13	0.30154
EBNA3	HGS	0.18299
EBNA3	ARHGEF11	0.11531
EBNA3	SLC9A3R1	0.10565
EBNA3	TOPBP1	0.2643
EBNA3	BOLL	0.37553
EBNA3	MYLK2	0.075735
EBNA3	RYBP	0.20623
EBNA3	EHD3	0.40006
EBNA3	TUBB4A	0.091206
EBNA3	DYRK1A	0.11681
EBNA3	TGM1	0.36346
EBNA3	JAK1	0.18799
EBNA3	NQO2	0.032408
EBNA3	FAM181A	0.027696
EBNA3	IGF2	0.15672
EBNA3	HSPA2	0.3054
EBNA3	RPL23	0.064303
EBNA3	FAM64A	0.42622
EBNA3	KIAA2018	0.15123
EBNA3	OCIAD1	0.39767
EBNA3	BCR	0.15803
EBNA3	SUPT20HL1	0.090443
EBNA3	SRCIN1	0.17489
EBNA3	TMEM8B	0.27898
EBNA3	MMP11	0.055415
EBNA3	SLC14A2	0.3369
EBNA3	ZZEF1	0.15751
EBNA3	AGER	0.17558
EBNA3	RICTOR	0.26389
EBNA3	MYL10	0.20444
EBNA3	C7orf57	0.027514
EBNA3	PDGFRA	0.038031
EBNA3	LY96	0.21706
EBNA3	UBE2M	0.23752
EBNA3	NF2	0.11474
EBNA3	C22orf43	0.050636
EBNA3	MICALL1	0.34706
EBNA3	CSK	0.16094
EBNA3	SMAD2	0.035168
EBNA3	RPS2	0.23097
EBNA3	UTF1	0.2574
EBNA3	EXOSC8	0.30715
EBNA3	IL4R	0.237
EBNA3	NR3C2	0.15103
EBNA3	RNF4	0.037075
EBNA3	KRT15	0.33393
EBNA3	DUT	0.24489
EBNA3	NTN1	0.27827
EBNA3	FAM166B	0.016091
EBNA3	ZXDA	0.13312
EBNA3	MAGEC1	0.43795
EBNA3	POU2F3	0.13011
EBNA3	EIF4G3	0.011565
EBNA3	P4HTM	0.15446
EBNA3	EFCAB11	0.21072
EBNA3	MT1G	0.21531
EBNA3	DLGAP5	0.24629
EBNA3	ATRX	0.19746
EBNA3	FKBP2	0.34088

EBNA3	TMEM102	0.33135
EBNA3	CDC26	0.27332
EBNA3	VWA5B2	0.064597
EBNA3	RNF6	0.18366
EBNA3	TAF9B	0.20142
EBNA3	DTL	0.24074
EBNA3	UVRAG	0.31984
EBNA3	COPB1	0.16569
EBNA3	KIF6	0.3255
EBNA3	THBS2	0.60864
EBNA3	OIT3	0.16642
EBNA3	EPS15L1	0.1357
EBNA3	PELI2	0.27882
EBNA3	NELFB	0.24529
EBNA3	FAP	0.24263
EBNA3	SLIT1	0.07414
EBNA3	NAF1	0.31852
EBNA3	MLLT3	0.28093
EBNA3	PRAME	0.23532
EBNA3	CALM1	0.24459
EBNA3	CRYAB	0.12885
EBNA3	SMARCA5	0.11166
EBNA3	ASAP2	0.12158
EBNA3	EIF4E2	0.4394
EBNA3	PROM1	0.23457
EBNA3	STAMBPL1	0.34855
EBNA3	MT-RNR2	0.24898
EBNA3	ACVR2B	0.054227
EBNA3	DBNL	0.24055
EBNA3	FGF3	0.25148
EBNA3	E2F1	0.065605
EBNA3	STRN4	0.39381
EBNA3	JUN	0.012312
EBNA3	AVPR1A	0.12801
EBNA3	RRP8	0.25355
EBNA3	HPRT1	0.12153
EBNA3	YWHAG	0.28272
EBNA3	NPM3	0.34638
EBNA3	DNASE1	0.26731
EBNA3	RNF216	0.21005
EBNA3	MAGI2	0.13466
EBNA3	EPOR	0.26089
EBNA3	STRIP1	0.34612
EBNA3	ARAP1	0.047262
EBNA3	EIF2A	0.25403
EBNA3	KCTD5	0.33089
EBNA3	CASP1	0.12075
EBNA3	ZRANB3	0.12712
EBNA3	GLUD1	0.2181
EBNA3	TFAP2C	0.068766
EBNA3	FLT3	0.15369
EBNA3	EXOC2	0.36281
EBNA3	ATG12	0.34589
EBNA3	RARG	0.051648
EBNA3	KIF1B	0.2896
EBNA3	MAP7D2	0.019956
EBNA3	CAV3	0.073474
EBNA3	SMARCB1	0.14447
EBNA3	CDHR3	0.18006
EBNA3	YEATS2	0.30606
EBNA3	KPNA5	0.32837
EBNA3	ARHGAP10	0.15271
EBNA3	S100A4	0.62787
EBNA3	MMP21	0.050885
EBNA3	ERCC5	0.17616
EBNA3	ABCG2	0.27566
EBNA3	GUCA1B	0.18315
EBNA3	NAPA	0.24601

EBNA3	TNPO2	0.33106
EBNA3	FAM20B	0.040978
EBNA3	COL4A1	0.27597
EBNA3	DNAJC21	0.32436
EBNA3	S100A12	0.3564
EBNA3	FYCO1	0.40884
EBNA3	RGS16	0.4041
EBNA3	WNT1	0.059279
EBNA3	APOL6	0.071525
EBNA3	FAM149B1	0.07597
EBNA3	RAB9B	0.33175
EBNA3	C19orf80	0.42461
EBNA3	RCN1	0.69033
EBNA3	RASGRP3	0.075323
EBNA3	NUDT14	0.38233
EBNA3	PHC2	0.27636
EBNA3	MTMR2	0.083225
EBNA3	BMPR2	0.059719
EBNA3	IKBKE	0.14057
EBNA3	CDKN2A	0.027094
EBNA3	DDX39B	0.21322
EBNA3	WNT3A	0.030874
EBNA3	BAZ2B	0.26763
EBNA3	CASP8	0.065164
EBNA3	PRSS12	0.041249
EBNA3	NEFL	0.23439
EBNA3	FTSJ1	0.089398
EBNA3	KCNJ6	0.29161
EBNA3	PDSS1	0.021969
EBNA3	MYH2	0.22737
EBNA3	FKBP7	0.17921
EBNA3	MZB1	0.33256
EBNA3	LRRFIP1	0.22984
EBNA3	PPP2R3C	0.16201
EBNA3	TNFRSF6B	0.3764
EBNA3	SMAD6	0.093091
EBNA3	TROAP	0.41094
EBNA3	MT1H	0.2804
EBNA3	RAPGEF4	0.12899
EBNA3	PSIP1	0.21619
EBNA3	SNRPD2	0.21555
EBNA3	SSX2IP	0.36252
EBNA3	ACD	0.19443
EBNA3	DPP4	0.20417
EBNA3	RERE	0.14725
EBNA3	SPSB4	0.40422
EBNA3	EIF3E	0.29412
EBNA3	EIF4E	0.27331
EBNA3	STAG3L4	0.032693
EBNA3	DSCC1	0.28949
EBNA3	RAMP1	0.18092
EBNA3	BBC3	0.14884
EBNA3	HSPBP1	0.36458
EBNA3	SDC2	0.19833
EBNA3	AK6	0.42138
EBNA3	HRAS	0.052507
EBNA3	EMILIN2	0.38256
EBNA3	SDIM1	0.39615
EBNA3	AIM2	0.15104
EBNA3	CLTCL1	0.29449
EBNA3	NPHP1	0.19343
EBNA3	CHD7	0.088805
EBNA3	SLC15A2	0.26997
EBNA3	DNMT3A	0.23123
EBNA3	ATP6V1F	0.24617
EBNA3	OAS3	0.2335
EBNA3	INHBA	0.074462
EBNA3	CDH2	0.050945

EBNA3	RPS23	0.19802
EBNA3	RQCD1	0.25051
EBNA3	PAG1	0.31959
EBNA3	NPB	0.36287
EBNA3	PIM3	0.22297
EBNA3	ACTN3	0.57595
EBNA3	SREBF1	0.019663
EBNA3	ANXA4	0.18188
EBNA3	ATP5F1	0.34563
EBNA3	FBN1	0.57599
EBNA3	RPL6	0.063047
EBNA3	RNF182	0.23736
EBNA3	MIF4GD	0.38426
EBNA3	PUS7	0.054505
EBNA3	MAP4K3	0.23724
EBNA3	TNNI2	0.31493
EBNA3	RBKS	0.027307
EBNA3	SPAC22F8.05	0.012081
EBNA3	ITPKA	0.097454
EBNA3	PDPK1	0.044667
EBNA3	RAD1	0.21625
EBNA3	ATG14	0.36082
EBNA3	C12orf63	0.037162
EBNA3	F10	0.38673
EBNA3	PTK2B	0.020839
EBNA3	CAPN1	0.45147
EBNA3	PAX3	0.11839
EBNA3	UCKL1	0.3846
EBNA3	PLXDC1	0.2879
EBNA3	HSPB8	0.20509
EBNA3	MED15	0.23258
EBNA3	DSG1	0.11397
EBNA3	CEACAM6	0.36087
EBNA3	GSTZ1	0.34283
EBNA3	TLE1	0.15491
EBNA3	SKIL	0.15778
EBNA3	LILRB3	0.33292
EBNA3	ZNF3	0.10405
EBNA3	ZNF346	0.30794
EBNA3	RPSA	0.12302
EBNA3	SRPK1	0.12201
EBNA3	IPO7	0.24716
EBNA3	TRIM24	0.052211
EBNA3	CFL2	0.37199
EBNA3	ACAA1	0.22396
EBNA3	DPM2	0.24237
EBNA3	GHRHR	0.11819
EBNA3	CX3CR1	0.15961
EBNA3	NTMT1	0.032054
EBNA3	PCDHA8	0.16911
EBNA3	PSMG2	0.33615
EBNA3	NDOR1	0.25024
EBNA3	PPM1A	0.076321
EBNA3	LETMD1	0.31074
EBNA3	SEC11C	0.07098
EBNA3	SYNM	0.27933
EBNA3	FAM46A	0.073643
EBNA3	FOXK2	0.020725
EBNA3	PPP2R5E	0.28637
EBNA3	SCGN	0.27621
EBNA3	SAE1	0.15452
EBNA3	TSN	0.3844
EBNA3	VPS51	0.32603
EBNA3	CELSR3	0.45981
EBNA3	IARS	0.1533
EBNA3	CAND1	0.27815
EBNA3	FCHSD2	0.43795
EBNA3	CATSPER1	0.21417

EBNA3	RILP	0.33753
EBNA3	SS18L2	0.031345
EBNA3	ING5	0.20703
EBNA3	SENP2	0.21919
EBNA3	BAX	0.014
EBNA3	RCOR3	0.30935
EBNA3	MAPKAPK5	0.13969
EBNA3	LMAN1	0.13257
EBNA3	UBE2B	0.11159
EBNA3	MRPL48	0.34456
EBNA3	UCN3	0.12063
EBNA3	SDHA	0.33077
EBNA3	NR2C2	0.093063
EBNA3	WASF1	0.25475
EBNA3	CTIF	0.40183
EBNA3	RNF115	0.28595
EBNA3	SYNJ2	0.22958
EBNA3	KIAA1524	0.38697
EBNA3	ZNRF3	0.22074
EBNA3	PLA2G2D	0.074695
EBNA3	TCEAL5	0.33039
EBNA3	PEX14	0.17366
EBNA3	GAGE12J	0.031848
EBNA3	CACNA1A	0.058978
EBNA3	AGO3	0.31085
EBNA3	RPGRIP1	0.33892
EBNA3	SPOCK1	0.16885
EBNA3	N6AMT1	0.48745
EBNA3	POLR2E	0.13023
EBNA3	S100A5	0.14509
EBNA3	POLR2J	0.15157
EBNA3	CD4	0.086934
EBNA3	DEHA2C16280g	0.038073
EBNA3	ARF1	0.12092
EBNA3	GEMIN8	0.32915
EBNA3	CERS2	0.10106
EBNA3	IGFL1	0.41198
EBNA3	NFU1	0.056864
EBNA3	PEX13	0.29249
EBNA3	EMR4P	0.12951
EBNA3	IGBP1	0.2296
EBNA3	NOD2	0.013991
EBNA3	MEOX2	0.15183
EBNA3	TFAP4	0.16041
EBNA3	UBAP2L	0.41166
EBNA3	POM121	0.13145
EBNA3	KHNYN	0.057897
EBNA3	SETD8	0.28279
EBNA3	MAGEA4	0.40098
EBNA3	TAGLN2	0.39024
EBNA3	SIRT4	0.48424
EBNA3	FYTTD1	0.44884
EBNA3	DNAJB6	0.28375
EBNA3	PCDHA4	0.16911
EBNA3	CDH15	0.14409
EBNA3	FAM110A	0.36772
EBNA3	COBL	0.078971
EBNA3	LDHD	0.36161
EBNA3	GBE1	0.075896
EBNA3	PRNP	0.10497
EBNA3	TESC	0.33914
EBNA3	CLIC3	0.28216
EBNA3	TAF5	0.057692
EBNA3	CHD1	0.1897
EBNA3	GAGE2A	0.061456
EBNA3	RBP4	0.15684
EBNA3	HOXB9	0.13843
EBNA3	SETD7	0.31976

EBNA3	PORCN	0.067982
EBNA3	FAM161A	0.46632
EBNA3	ATPAF1	0.41174
EBNA3	PARD3	0.15294
EBNA3	ATXN7L1	0.035027
EBNA3	FMNL1	0.2424
EBNA3	ATP5G2	0.015708
EBNA3	UBE2Q1	0.28523
EBNA3	IFNLR1	0.35687
EBNA3	EFHB	0.21072
EBNA3	LGI1	0.23764
EBNA3	NME2	0.10624
EBNA3	KIAA0319	0.31368
EBNA3	CRY2	0.13864
EBNA3	ARL2	0.16271
EBNA3	EYS	0.23384
EBNA3	MICAL1	0.34542
EBNA3	CLVS2	0.040569
EBNA3	LSM12	0.094456
EBNA3	NDNL2	0.30981
EBNA3	ROPN1L	0.37311
EBNA3	HOXA1	0.014841
EBNA3	OBSCN	0.12896
EBNA3	UCN	0.18848
EBNA3	PCDHGB7	0.18006
EBNA3	RPA3	0.17856
EBNA3	FGFR3	0.061015
EBNA3	RWDD1	0.43795
EBNA3	TFPT	0.28243
EBNA3	SMPDL3A	0.32685
EBNA3	RHO	0.18015
EBNA3	LIG3	0.14991
EBNA3	KIAA0907	0.02062
EBNA3	C12orf57	0.074558
EBNA3	HSPB6	0.37672
EBNA3	ADAMTS4	0.21259
EBNA3	PJA1	0.30121
EBNA3	ALKBH2	0.07173
EBNA3	AHNAK	0.37987
EBNA3	CCNF	0.29415
EBNA3	HOOK3	0.20859
EBNA3	SETD1A	0.32414
EBNA3	GLB1	0.17628
EBNA3	IGFL4	0.16194
EBNA3	PAK2	0.029187
EBNA3	SMARCC2	0.20747
EBNA3	CSNK2B	0.059622
EBNA3	HIC1	0.08512
EBNA3	FCN3	0.30373
EBNA3	PPFIA1	0.3861
EBNA3	TAF4	0.091271
EBNA3	PPP4R4	0.40938
EBNA3	FAM228A	0.014867
EBNA3	YAE1D1	0.094471
EBNA3	DCLRE1B	0.21979
EBNA3	OSBPL1A	0.20821
EBNA3	GOPC	0.18307
EBNA3	CD82	0.38673
EBNA3	TNFRSF1A	0.11848
EBNA3	DZIP3	0.3406
EBNA3	RALA	0.18786
EBNA3	CD226	0.17973
EBNA3	CT45A1	0.040682
EBNA3	ERICH1	0.089325
EBNA3	MOB4	0.32772
EBNA3	GIF	0.093905
EBNA3	BIN3	0.31315
EBNA3	DRG1	0.23052

EBNA3	POLR2A	0.13275
EBNA3	RPH3A	0.36744
EBNA3	SPTSSA	0.28596
EBNA3	DCP1A	0.23229
EBNA3	ADAP1	0.10888
EBNA3	AMOTL2	0.39814
EBNA3	SMARCA1	0.16006
EBNA3	SRI	0.39597
EBNA3	SELL	0.26357
EBNA3	OCM	0.17787
EBNA3	PPP4R2	0.27972
EBNA3	RTP3	0.36917
EBNA3	LRP4	0.35683
EBNA3	DPPA4	0.32035
EBNA3	KAT6A	0.13048
EBNA3	PPP1CB	0.036186
EBNA3	CDK8	0.070424
EBNA3	PMF1	0.20055
EBNA3	BDKRB2	0.1541
EBNA3	KPNA2	0.25377
EBNA3	EVI5L	0.33822
EBNA3	NEK2	0.07988
EBNA3	RAD54L	0.22526
EBNA3	TSC2	0.08244
EBNA3	SLC7A8	0.11281
EBNA3	PSG5	0.41158
EBNA3	PIK3R2	0.20502
EBNA3	MAP3K14	0.11473
EBNA3	IMMP2L	0.022374
EBNA3	BMX	0.12792
EBNA3	CHST7	0.051869
EBNA3	INADL	0.31362
EBNA3	BVES-AS1	0.05209
EBNA3	SEL1L	0.35505
EBNA3	GNL3L	0.2667
EBNA3	PTPN12	0.14835
EBNA3	MARCKS	0.13305
EBNA3	PLA2G12B	0.15606
EBNA3	PLA2G4B	0.039817
EBNA3	FEN1	0.086331
EBNA3	TYMP	0.047674
EBNA3	TMSB4X	0.25527
EBNA3	METTL3	0.017756
EBNA3	PCDHB12	0.16911
EBNA3	VEGFA	0.012842
EBNA3	NONO	0.28892
EBNA3	PLK1S1	0.25313
EBNA3	CCDC127	0.028257
EBNA3	FLG2	0.05374
EBNA3	CUL2	0.26312
EBNA3	CCDC120	0.088797
EBNA3	CCT3	0.26618
EBNA3	SRSF7	0.29106
EBNA3	MAP3K7	0.031821
EBNA3	SPATA31E1	0.27845
EBNA3	CABP5	0.18284
EBNA3	MAGEB16	0.012314
EBNA3	SLIT2	0.1097
EBNA3	PKD1	0.050722
EBNA3	C1orf145	0.097846
EBNA3	HERC2	0.16891
EBNA3	TBC1D5	0.303
EBNA3	SNED1	0.1981
EBNA3	AKAP9	0.19708
EBNA3	MIB1	0.22319
EBNA3	PASK	0.18786
EBNA3	BLM	0.065479
EBNA3	ARNT	0.077718

EBNA3	SYNE3	0.27216
EBNA3	DOCK4	0.23603
EBNA3	NCLN	0.26142
EBNA3	AMY2A	0.027954
EBNA3	PANK3	0.13127
EBNA3	OTUD1	0.033168
EBNA3	HNRNPUL1	0.28233
EBNA3	GPRASP1	0.4246
EBNA3	MAGEE1	0.31033
EBNA3	RAD51AP1	0.41246
EBNA3	CNPY2	0.37546
EBNA3	NSRP1	0.34349
EBNA3	SFI1	0.33545
EBNA3	RPP38	0.32014
EBNA3	MATN2	0.54198
EBNA3	BCAR1	0.10982
EBNA3	PSMD9	0.1495
EBNA3	AXIN1	0.032449
EBNA3	SIRT6	0.2623
EBNA3	C2orf71	0.11305
EBNA3	INTS7	0.36477
EBNA3	STK4	0.060914
EBNA3	GPD2	0.12851
EBNA3	DHFR	0.028838
EBNA3	PCDHA10	0.16404
EBNA3	CIB1	0.48791
EBNA3	IRAK2	0.085839
EBNA3	MAPK14	0.026928
EBNA3	UBR5	0.20232
EBNA3	PRRG4	0.17542
EBNA3	ANKRD44	0.43795
EBNA3	KIF13A	0.16007
EBNA3	FNTA	0.29004
EBNA3	EIF5A2	0.23183
EBNA3	MAPRE3	0.32228
EBNA3	CLCN3	0.19304
EBNA3	DNAJB2	0.29108
EBNA3	DNMT1	0.19854
EBNA3	PCDHA11	0.16911
EBNA3	HDAC10	0.13851
EBNA3	RYR1	0.04688
EBNA3	PCDHB7	0.17455
EBNA3	TNK1	0.27006
EBNA3	PHF8	0.1699
EBNA3	GYS1	0.17483
EBNA3	PPP2R3A	0.52107
EBNA3	GSPT1	0.26673
EBNA3	DSC1	0.18092
EBNA3	AKAP3	0.4169
EBNA3	PRPF19	0.23188
EBNA3	CCDC155	0.081854
EBNA3	EIF2B4	0.25646
EBNA3	NCF1	0.074179
EBNA3	PSMC2	0.11456
EBNA3	TSSK4	0.18303
EBNA3	USE1	0.27316
EBNA3	LAPTM4B	0.39226
EBNA3	DLL1	0.34819
EBNA3	TAF2	0.20721
EBNA3	ACTN1	0.30703
EBNA3	CABP2	0.17515
EBNA3	VDR	0.094525
EBNA3	PEX11B	0.32152
EBNA3	PMP22	0.34063
EBNA3	RAB6A	0.23663
EBNA3	C11orf54	0.32378
EBNA3	STK38	0.11059
EBNA3	TNKS2	0.18589

EBNA3	RCHY1	0.17139
EBNA3	CCND1	0.063586
EBNA3	CD164	0.24942
EBNA3	EPRS	0.17538
EBNA3	TBC1D15	0.31479
EBNA3	AOC3	0.18675
EBNA3	FUS	0.31027
EBNA3	BLOC1S1	0.26224
EBNA3	FAM195A	0.15334
EBNA3	DCTN3	0.14016
EBNA3	NPHP3	0.21554
EBNA3	USP46	0.32502
EBNA3	UBD	0.24917
EBNA3	MYH10	0.12137
EBNA3	CDT1	0.26189
EBNA3	RGAG1	0.036924
EBNA3	MT-ATP8	0.039147
EBNA3	SYNE1	0.22061
EBNA3	TRMT11	0.029435
EBNA3	FBXO3	0.36406
EBNA3	LRP6	0.020773
EBNA3	EXOC8	0.17668
EBNA3	KNTC1	0.20433
EBNA3	NCL	0.28626
EBNA3	ITGA1	0.23552
EBNA3	SVEP1	0.17933
EBNA3	GTSF1	0.1747
EBNA3	NOS1	0.15375
EBNA3	EHD4	0.37265
EBNA3	APBA3	0.28346
EBNA3	RYR3	0.094826
EBNA3	SCUBE1	0.10726
EBNA3	PCDHB14	0.16421
EBNA3	HNRNPK	0.17555
EBNA3	LCAT	0.25082
EBNA3	OBFC1	0.17717
EBNA3	HJURP	0.20511
EBNA3	TWIST1	0.046729
EBNA3	E2F5	0.10547
EBNA3	CSNK1E	0.13691
EBNA3	FAM124B	0.43795
EBNA3	MEF2A	0.068089
EBNA3	PNPT1	0.13573
EBNA3	NPRL2	0.29141
EBNA3	UPF3B	0.33462
EBNA3	AKAP13	0.13642
EBNA3	RPL30	0.044923
EBNA3	ATG2A	0.43795
EBNA3	ZC3H7B	0.28048
EBNA3	LZTFL1	0.39169
EBNA3	TLN1	0.078465
EBNA3	BTRC	0.13652
EBNA3	PCDHGA11	0.18006
EBNA3	RHBDL1	0.28
EBNA3	HNF1B	0.061207
EBNA3	IRF4	0.036542
EBNA3	TFAP2A	0.02079
EBNA3	UBE2D1	0.14475
EBNA3	GCKR	0.22865
EBNA3	MYO6	0.11125
EBNA3	C5orf58	0.11509
EBNA3	LCMT2	0.49132
EBNA3	GATAD2A	0.12268
EBNA3	TGFBR1	0.016696
EBNA3	USP8	0.16799
EBNA3	DSPP	0.18899
EBNA3	WFS1	0.033613
EBNA3	FIG4	0.17273

EBNA3	TNFSF14	0.20051
EBNA3	CDC6	0.13197
EBNA3	CDH3	0.18601
EBNA3	PLAT	0.23088
EBNA3	TAX1BP3	0.2869
EBNA3	PLA2G12A	0.054483
EBNA3	SNX4	0.33884
EBNA3	NEGR1	0.3516
EBNA3	HTR6	0.27894
EBNA3	CUEDC2	0.3699
EBNA3	SEPT11	0.297
EBNA3	PNLIPRP2	0.039328
EBNA3	DLGAP1	0.31327
EBNA3	CCND3	0.10352
EBNA3	MFI2	0.25718
EBNA3	FLNA	0.08429
EBNA3	HIST1H4A	0.17517
EBNA3	CTTNBP2NL	0.42312
EBNA3	RAI2	0.40102
EBNA3	SERPINF2	0.19454
EBNA3	FBXL5	0.30536
EBNA3	SGSM3	0.29728
EBNA3	DUOX1	0.21161
EBNA3	F3	0.10351
EBNA3	CECR2	0.32085
EBNA3	PMAIP1	0.10854
EBNA3	SPINK1	0.36392
EBNA3	ABCD1	0.18666
EBNA3	CHAC1	0.29476
EBNA3	DDX3X	0.04205
EBNA3	PCDHA2	0.16911
EBNA3	SH3GLB2	0.32488
EBNA3	CDK16	0.16234
EBNA3	RASEF	0.15664
EBNA3	RPL10A	0.026044
EBNA3	DPP7	0.13479
EBNA3	PRSS3	0.57068
EBNA3	ANXA8	0.1637
EBNA3	CCDC169	0.056864
EBNA3	RPS6KC1	0.22268
EBNA3	MAP1S	0.15562
EBNA3	BTK	0.050549
EBNA3	CLEC4G	0.399
EBNA3	PPIL3	0.37146
EBNA3	CALML5	0.18729
EBNA3	C7orf62	0.074289
EBNA3	LRRC4C	0.39912
EBNA3	MYL2	0.5047
EBNA3	BAIAP2	0.1677
EBNA3	KAT5	0.16142
EBNA3	PIWIL1	0.30051
EBNA3	ALOX12	0.17489
EBNA3	EDEM1	0.45054
EBNA3	LRP1	0.20439
EBNA3	MSH3	0.040977
EBNA3	UFL1	0.28083
EBNA3	APBA2	0.31481
EBNA3	DHDDS	0.42747
EBNA3	MKI67	0.22669
EBNA3	JADE1	0.22457
EBNA3	MTF1	0.090645
EBNA3	FGFR1	0.02515
EBNA3	GEMIN2	0.27448
EBNA3	COL26A1	0.15046
EBNA3	SELE	0.17841
EBNA3	TONSL	0.2811
EBNA3	CACNB4	0.14361
EBNA3	RBM12B	0.49416

EBNA3	CKS1B	0.31528
EBNA3	ATP10B	0.36163
EBNA3	TIMM23	0.29075
EBNA3	DICER1	0.05081
EBNA3	HADHB	0.14316
EBNA3	APPL1	0.145
EBNA3	CCDC92	0.39788
EBNA3	VPRBP	0.32989
EBNA3	SENP5	0.31765
EBNA3	SUV39H1	0.19736
EBNA3	COPS6	0.31749
EBNA3	MB21D2	0.04609
EBNA3	SNX2	0.29215
EBNA3	MAP2K5	0.13919
EBNA3	RNF11	0.24502
EBNA3	ITGAM	0.20404
EBNA3	CDH17	0.17743
EBNA3	CDK11B	0.15011
EBNA3	EFCAB9	0.21072
EBNA3	PEX1	0.16997
EBNA3	ARMCX5	0.046728
EBNA3	GPX8	0.024163
EBNA3	NUDT18	0.36564
EBNA3	EXOSC2	0.31679
EBNA3	ERG	0.1197
EBNA3	ZNF622	0.24156
EBNA3	MAP2K1	0.016736
EBNA3	PRCC	0.30258
EBNA3	NFS1	0.23612
EBNA3	EEF1E1	0.28745
EBNA3	FANCD2	0.33786
EBNA3	TNNC2	0.17608
EBNA3	ATP5J	0.057542
EBNA3	GNA12	0.16995
EBNA3	ZC3H14	0.36318
EBNA3	MEPCE	0.090668
EBNA3	LSM11	0.29414
EBNA3	BUB1	0.10965
EBNA3	RIT1	0.32426
EBNA3	KCNA4	0.26722
EBNA3	RIOK3	0.31544
EBNA3	KISS1	0.3994
EBNA3	DMC1	0.19601
EBNA3	ATP5A1	0.19918
EBNA3	RAB21	0.29013
EBNA3	SGPL1	0.08352
EBNA3	ADAR	0.25204
EBNA3	OIP5	0.2677
EBNA3	GAD1	0.19719
EBNA3	WDTC1	0.22966
EBNA3	SNX19	0.56501
EBNA3	STXBP5	0.060111
EBNA3	MAP2K4	0.15146
EBNA3	ASPH	0.18817
EBNA3	CREB1	0.032676
EBNA3	THUMPD2	0.045962
EBNA3	OASL	0.032481
EBNA3	LIN28B	0.27654
EBNA3	TUBGCP2	0.23627
EBNA3	HIP1	0.13267
EBNA3	MBD3	0.2369
EBNA3	ITGA4	0.24085
EBNA3	FSTL1	0.16828
EBNA3	SRC	0.045201
EBNA3	MRE11A	0.13592
EBNA3	USP47	0.24462
EBNA3	CALR3	0.12875
EBNA3	CLPP	0.42223

EBNA3	CHORDC1	0.3009
EBNA3	SMG6	0.1618
EBNA3	ID1	0.10833
EBNA3	SOX10	0.049987
EBNA3	TRPC1	0.19323
EBNA3	TOM1	0.32674
EBNA3	PITPNC1	0.3992
EBNA3	PDGFRB	0.074213
EBNA3	SULF2	0.041168
EBNA3	S100A10	0.1104
EBNA3	SLC51A	0.14511
EBNA3	NDUFB10	0.34597
EBNA3	SPATA2L	0.063568
EBNA3	ACVRL1	0.019065
EBNA3	KIF2C	0.14262
EBNA3	CACNA1B	0.23215
EBNA3	SEPT5	0.12794
EBNA3	PAX8	0.046736
EBNA3	APPBP2	0.34172
EBNA3	C1QTNF3	0.24652
EBNA3	GAD2	0.18505
EBNA3	WNK1	0.22053
EBNA3	LAPTM5	0.143
EBNA3	TCF3	0.03113
EBNA3	DACT1	0.16691
EBNA3	SV2C	0.03975
EBNA3	PCNA	0.071785
EBNA3	ATXN1L	0.078603
EBNA3	DDRGK1	0.42519
EBNA3	EPO	0.12612
EBNA3	IRF8	0.051398
EBNA3	RPS3	0.037484
EBNA3	CD274	0.31043
EBNA3	ITSN1	0.268
EBNA3	ATP7B	0.09704
EBNA3	PTPRD	0.095165
EBNA3	RPUSD1	0.032836
EBNA3	KDM4A	0.20035
EBNA3	TRAM2	0.3805
EBNA3	XKRX	0.14319
EBNA3	SPAG9	0.032385
EBNA3	CEBPB	0.078114
EBNA3	C15orf37	0.022994
EBNA3	BRCC3	0.19389
EBNA3	BBS1	0.31104
EBNA3	BTF3L4	0.037021
EBNA3	SLC22A4	0.24027
EBNA3	SPIDR	0.11913
EBNA3	ETV7	0.17837
EBNA3	HNRNPF	0.28464
EBNA3	CCDC141	0.43795
EBNA3	PCDHGA12	0.18006
EBNA3	ELOVL1	0.25409
EBNA3	PRDX3	0.14177
EBNA3	GK2	0.085622
EBNA3	DST	0.2426
EBNA3	HIF1AN	0.16341
EBNA3	COL18A1	0.15803
EBNA3	OGG1	0.11499
EBNA3	MYL6	0.58688
EBNA3	DARC	0.09239
EBNA3	COMP	0.48524
EBNA3	RNF31	0.20427
EBNA3	EIF3G	0.39493
EBNA3	MLLT6	0.29829
EBNA3	CCDC47	0.57763
EBNA3	SNAP47	0.37409
EBNA3	EXOSC7	0.30002

EBNA3	LIMD1	0.13533
EBNA3	MX1	0.18489
EBNA3	SATB1	0.10542
EBNA3	NSUN2	0.033551
EBNA3	RIN1	0.33778
EBNA3	RCOR1	0.20346
EBNA3	XAGE5	0.089619
EBNA3	ANXA7	0.44911
EBNA3	SON	0.27648
EBNA3	MAP3K7CL	0.38053
EBNA3	RNF139	0.20587
EBNA3	PRC1	0.11982
EBNA3	SLC30A3	0.010762
EBNA3	CDH26	0.18006
EBNA3	DGUOK	0.030452
EBNA3	FRK	0.24152
EBNA3	CHP2	0.52974
EBNA3	ADNP	0.071125
EBNA3	TOX3	0.27923
EBNA3	SPG11	0.39379
EBNA3	PSMA7	0.085252
EBNA3	ZFP36L1	0.10784
EBNA3	CD97	0.11007
EBNA3	LYRM9	0.11288
EBNA3	ABCC2	0.31195
EBNA3	PTPRB	0.1451
EBNA3	POM121C	0.30788
EBNA3	MUC1	0.20363
EBNA3	RANBP3	0.29519
EBNA3	CTNNA1	0.10851
EBNA3	FAM127B	0.078192
EBNA3	VBP1	0.26182
EBNA3	MRVI1	0.29408
EBNA3	UBE2L6	0.2845
EBNA3	ZHX3	0.14034
EBNA3	RSPO1	0.25084
EBNA3	GOLPH3	0.29249
EBNA3	IRF6	0.064252
EBNA3	PLN	0.26022
EBNA3	CDK5R1	0.088998
EBNA3	SUB1	0.25746
EBNA3	ANP32A	0.24653
EBNA3	LTBP2	0.15832
EBNA3	PPM1F	0.12468
EBNA3	SEPT4	0.081572
EBNA3	AKT3	0.10985
EBNA3	CXorf40B	0.032006
EBNA3	TEAD2	0.15771
EBNA3	CAPN13	0.089042
EBNA3	SYNGR2	0.11278
EBNA3	PLOD3	0.2287
EBNA3	IKBKB	0.037342
EBNA3	TOLLIP	0.30951
EBNA3	EIF4EBP2	0.3303
EBNA3	RUVBL1	0.12395
EBNA3	LRP12	0.32499
EBNA3	NDUFS7	0.12039
EBNA3	NECAB2	0.74552
EBNA3	ELF3	0.11307
EBNA3	PROS1	0.081848
EBNA3	OTX2	0.055498
EBNA3	OGT	0.12914
EBNA3	GTF3C4	0.31469
EBNA3	PBRM1	0.27223
EBNA3	NCOR1	0.06508
EBNA3	APITD1	0.19488
EBNA3	HGF	0.1581
EBNA3	FAM103A1	0.4087

EBNA3	DNAJB9	0.28816
EBNA3	AVEN	0.37518
EBNA3	SLBP	0.25743
EBNA3	USH2A	0.22935
EBNA3	MMP16	0.04462
EBNA3	PHF2	0.21119
EBNA3	PODXL2	0.36276
EBNA3	APLP1	0.099493
EBNA3	FBP1	0.11973
EBNA3	RFC2	0.17881
EBNA3	TYROBP	0.20847
EBNA3	CANX	0.31122
EBNA3	HNF1A	0.05247
EBNA3	RHOT2	0.34686
EBNA3	DOCK9	0.20905
EBNA3	RTKN	0.24151
EBNA3	EYA3	0.08569
EBNA3	KLHL3	0.22481
EBNA3	EHMT1	0.27999
EBNA3	KDR	0.044559
EBNA3	SGD1	0.065025
EBNA3	SNAP23	0.29506
EBNA3	MMP15	0.38568
EBNA3	MKNK1	0.18665
EBNA3	FHAD1	0.010463
EBNA3	TTC8	0.3075
EBNA3	PDE6D	0.45573
EBNA3	ASCC3	0.20846
EBNA3	CCNDBP1	0.36842
EBNA3	PPP2R5B	0.24207
EBNA3	GADD45A	0.26784
EBNA3	PLCB3	0.11601
EBNA3	SNX15	0.10676
EBNA3	LCP2	0.33542
EBNA3	BSDC1	0.013373
EBNA3	ORAI2	0.41166
EBNA3	SUSD1	0.19819
EBNA3	APBA1	0.23449
EBNA3	DKK1	0.14592
EBNA3	ANXA11	0.3906
EBNA3	SPINK9	0.37531
EBNA3	ITM2C	0.28383
EBNA3	HNF4A	0.01981
EBNA3	USP54	0.41089
EBNA3	PPP3CB	0.20499
EBNA3	GOLGA6L19	0.093196
EBNA3	TAB2	0.1387
EBNA3	SNRPB	0.20175
EBNA3	KRT19	0.26809
EBNA3	PCBD2	0.23817
EBNA3	S100G	0.19298
EBNA3	LURAP1	0.14715
EBNA3	PRKAA1	0.02748
EBNA3	ATG7	0.16863
EBNA3	MDM4	0.20538
EBNA3	SCYL2	0.17285
EBNA3	NCKIPSD	0.38164
EBNA3	SPINK7	0.38713
EBNA3	NQO1	0.29986
EBNA3	NFATC2	0.095116
EBNA3	C8orf88	0.053775
EBNA3	C5orf34	0.056278
EBNA3	ACSS1	0.14981
EBNA3	ACACB	0.21959
EBNA3	RBM39	0.29416
EBNA3	LURAP1L	0.13935
EBNA3	IGF2BP3	0.28906
EBNA3	TRIM27	0.11772

EBNA3	CSPG5	0.14711
EBNA3	MC3R	0.25744
EBNA3	ANXA5	0.054069
EBNA3	PAX2	0.03357
EBNA3	CNOT4	0.21111
EBNA3	CDK12	0.090846
EBNA3	DLG2	0.2289
EBNA3	ZNF136	0.23214
EBNA3	MAST1	0.17745
EBNA3	SV2B	0.03863
EBNA3	MAU2	0.32109
EBNA3	C19orf47	0.04159
EBNA3	C4orf33	0.019082
EBNA3	SH3PXD2B	0.2157
EBNA3	UBN1	0.16955
EBNA3	PUSL1	0.054505
EBNA3	ALDH1A3	0.026441
EBNA3	C12orf50	0.12419
EBNA3	PCSK4	0.011759
EBNA3	C17orf102	0.055723
EBNA3	IQGAP1	0.20918
EBNA3	RMND5B	0.014738
EBNA3	FAAP24	0.29794
EBNA3	IQCG	0.048803
EBNA3	HIRA	0.16836
EBNA3	ATP6V0C	0.22101
EBNA3	STAP2	0.18178
EBNA3	EPAS1	0.054328
EBNA3	CCNA2	0.17622
EBNA3	FZD1	0.077084
EBNA3	ELAVL1	0.22127
EBNA3	RAD51	0.11318
EBNA3	BMI1	0.10994
EBNA3	OPTN	0.29144
EBNA3	TMED1	0.25024
EBNA3	C19orf60	0.43795
EBNA3	RAB9A	0.17016
EBNA3	TIMP1	0.2016
EBNA3	MATN3	0.69877
EBNA3	SKA1	0.21331
EBNA3	EFCAB8	0.21072
EBNA3	ATL2	0.1985
EBNA3	NR2F2	0.059477
EBNA3	RAP1A	0.20698
EBNA3	MAST2	0.15413
EBNA3	SLC16A3	0.053777
EBNA3	SUPT20H	0.35329
EBNA3	SH3BP2	0.26781
EBNA3	YEATS4	0.18369
EBNA3	SFN	0.14958
EBNA3	MMP13	0.043642
EBNA3	PNMA6C	0.018621
EBNA3	FBN3	0.21414
EBNA3	G0S2	0.33868
EBNA3	RPL27A	0.2912
EBNA3	FAM47C	0.092365
EBNA3	MDC1	0.29798
EBNA3	C10orf111	0.15169
EBNA3	PGRMC1	0.30374
EBNA3	NHP2L1	0.32341
EBNA3	SH3KBP1	0.33977
EBNA3	QTRT1	0.1217
EBNA3	AMOTL1	0.35977
EBNA3	MYLIP	0.19388
EBNA3	TENC1	0.21412
EBNA3	STK3	0.10536
EBNA3	DLGAP4	0.33442
EBNA3	TOMM70A	0.276

EBNA3	TCERG1	0.2831
EBNA3	PCDH8	0.12864
EBNA3	UBE4A	0.34847
EBNA3	GTF3C5	0.34134
EBNA3	LRRK2	0.0456
EBNA3	TSC1	0.11277
EBNA3	CPNE6	0.19226
EBNA3	CD276	0.22411
EBNA3	RNF41	0.18197
EBNA3	BASP1	0.17098
EBNA3	RASSF2	0.35689
EBNA3	TJAP1	0.36317
EBNA3	ETV6	0.18174
EBNA3	DARS	0.2478
EBNA3	SCHIP1	0.36824
EBNA3	RET	0.2266
EBNA3	OS9	0.32842
EBNA3	ARHGAP26	0.15644
EBNA3	COL1A2	0.17807
EBNA3	PAGE3	0.066776
EBNA3	LSM7	0.27724
EBNA3	LYPD6B	0.13936
EBNA3	SRY	0.15098
EBNA3	JAM2	0.32387
EBNA3	MAFB	0.13474
EBNA3	DGKA	0.095173
EBNA3	ALOX5	0.17876
EBNA3	SOD1	0.037215
EBNA3	WASH1	0.28911
EBNA3	NDUFS2	0.042164
EBNA3	SPRED1	0.13407
EBNA3	PARP1	0.11827
EBNA3	TAF10	0.080311
EBNA3	CLCF1	0.24638
EBNA3	CHEK1	0.1143
EBNA3	FCRL4	0.39913
EBNA3	SF3B4	0.37239
EBNA3	EPS8	0.35857
EBNA3	PATL1	0.25081
EBNA3	TRAF5	0.21338
EBNA3	PARP10	0.3698
EBNA3	WBP11	0.29534
EBNA3	NSMCE4A	0.36221
EBNA3	SNTA1	0.22991
EBNA3	ATP11A	0.22114
EBNA3	IRF2	0.10011
EBNA3	CDH22	0.18006
EBNA3	THBS4	0.11695
EBNA3	FBXL3	0.30547
EBNA3	DDX1	0.14183
EBNA3	MC4R	0.2263
EBNA3	HEY2	0.018494
EBNA3	CDHR4	0.18006
EBNA3	CHD1L	0.28155
EBNA3	RPP25	0.37072
EBNA3	SLC3A2	0.19321
EBNA3	UBQLN1	0.26623
EBNA3	USP13	0.17083
EBNA3	SLC6A3	0.15955
EBNA3	CALCR	0.16544
EBNA3	PTGFRN	0.35135
EBNA3	EIF4EBP3	0.3402
EBNA3	PTPN11	0.061645
EBNA3	CPSF1	0.3244
EBNA3	MAGED2	0.015567
EBNA3	TLX1NB	0.023791
EBNA3	MNS1	0.16267
EBNA3	FLVCR1	0.25031

EBNA3	MS4A3	0.37525
EBNA3	HOXA5	0.055907
EBNA3	HCFC1	0.093713
EBNA3	FLNB	0.24026
EBNA3	TMEM66	0.31518
EBNA3	SMAD4	0.025292
EBNA3	PICK1	0.14088
EBNA3	MSLN	0.32967
EBNA3	RAB11FIP5	0.19248
EBNA3	SUZ12	0.20411
EBNA3	CDH23	0.31377
EBNA3	LCA10	0.10567
EBNA3	KIT	0.040936
EBNA3	STATH	0.33493
EBNA3	UBC	0.046837
EBNA3	RAB11FIP4	0.56712
EBNA3	RLF	0.2114
EBNA3	KRT18	0.20412
EBNA3	TRAPPC5	0.10218
EBNA3	FGF13	0.15552
EBNA3	FARSA	0.098728
EBNA3	NTM	0.31311
EBNA3	PDCD6IP	0.29003
EBNA3	GAB1	0.17114
EBNA3	HSFX1	0.18851
EBNA3	RARRES2	0.30881
EBNA3	CNGC19	0.16422
EBNA3	C9orf106	0.019124
EBNA3	HSPB1	0.077151
EBNA3	BMPR1A	0.046225
EBNA3	MYCBPAP	0.30654
EBNA3	MEF2D	0.13741
EBNA3	PSMA2	0.10297
EBNA3	ASXL1	0.19645
EBNA3	APBB2	0.17534
EBNA3	TP53BP1	0.15609
EBNA3	MAP1LC3A	0.28263
EBNA3	DYDC1	0.018365
EBNA3	CCDC102B	0.014116
EBNA3	UBXN1	0.25703
EBNA3	CNRIP1	0.026985
EBNA3	PRPF3	0.33568
EBNA3	ATP6V1H	0.2224
EBNA3	CDH12	0.16097
EBNA3	PHYH	0.16626
EBNA3	JMJD6	0.13718
EBNA3	RRAGA	0.2112
EBNA3	HSPB2	0.32589
EBNA3	PIGT	0.25528
EBNA3	EIF3A	0.23661
EBNA3	MGARP	0.23594
EBNA3	NFKB2	0.08792
EBNA3	UBE2I	0.079361
EBNA3	CASP7	0.17514
EBNA3	H2AFX	0.17283
EBNA3	RHOU	0.13348
EBNA3	SSRP1	0.24461
EBNA3	SUMO3	0.38696
EBNA3	TLR2	0.048607
EBNA3	ATF3	0.13806
EBNA3	TCERG1L	0.042621
EBNA3	SOS2	0.17646
EBNA3	SYNE2	0.16044
EBNA3	DPYSL2	0.055821
EBNA3	BCL2L2	0.33126
EBNA3	SUV39H2	0.26534
EBNA3	IL10RA	0.35047
EBNA3	CLIC5	0.18715

EBNA3	CTBP1	0.059812
EBNA3	UBE2F	0.27655
EBNA3	C6orf223	0.15891
EBNA3	AGTR2	0.13532
EBNA3	IFIH1	0.14884
EBNA3	C1orf64	0.10012
EBNA3	IER3	0.26894
EBNA3	CNKSR1	0.23073
EBNA3	LDHAL6B	0.35042
EBNA3	MAGEA1	0.28814
EBNA3	MBOAT7	0.33878
EBNA3	FOCAD	0.38774
EBNA3	LOX	0.22608
EBNA3	ELP4	0.23731
EBNA3	ELTD1	0.13789
EBNA3	CAV2	0.13241
EBNA3	FXR2	0.49635
EBNA3	POLR2B	0.1431
EBNA3	VANGL1	0.39912
EBNA3	MPP1	0.31589
EBNA3	TK2	0.024796
EBNA3	TRIP6	0.22662
EBNA3	PKP4	0.24407
EBNA3	COMMD4	0.4246
EBNA3	PACRGL	0.032117
EBNA3	ENO1	0.052793
EBNA3	AGGF1	0.3024
EBNA3	ORC5	0.22674
EBNA3	PPP1R2P3	0.14409
EBNA3	TCTEX1D1	0.032127
EBNA3	CLU	0.080635
EBNA3	PPIB	0.33952
EBNA3	PRRG1	0.1862
EBNA3	NRG1	0.052777
EBNA3	A1CF	0.26275
EBNA3	C1orf140	0.10481
EBNA3	NIFK	0.3371
EBNA3	ATXN7	0.24423
EBNA3	UBA6	0.23007
EBNA3	TDP2	0.21812
EBNA3	YGL006W-A	0.013678
EBNA3	SUDS3	0.31712
EBNA3	CHCHD3	0.28248
EBNA3	PRKACB	0.070271
EBNA3	GOLGA6L20	0.051129
EBNA3	MLANA	0.3325
EBNA3	LINC00083	0.011516
EBNA3	LPHN1	0.25058
EBNA3	LINC00588	0.098173
EBNA3	HYPK	0.02645
EBNA3	BRINP1	0.36156
EBNA3	RUNDC1	0.022407
EBNA3	ETNK2	0.05804
EBNA3	WIPF2	0.39911
EBNA3	SNX5	0.094576
EBNA3	MCM2	0.13206
EBNA3	PLCE1	0.17625
EBNA3	NIPSNAP3B	0.020668
EBNA3	CD163	0.34725
EBNA3	IL10RB	0.34208
EBNA3	DYNLL1	0.16188
EBNA3	PIDD	0.34885
EBNA3	ADAMTSL4	0.25802
EBNA3	MYOT	0.32005
EBNA3	PDCD1	0.32081
EBNA3	HNRNPLL	0.36303
EBNA3	SDCCAG8	0.31133
EBNA3	IL12B	0.046318

EBNA3	WDR48	0.27489
EBNA3	PDCD11	0.32938
EBNA3	G3BP1	0.26152
EBNA3	RASSF1	0.16802
EBNA3	NELFCD	0.25987
EBNA3	KHDRBS1	0.26669
EBNA3	EID1	0.23815
EBNA3	PCDHB4	0.14323
EBNA3	IGHMBP2	0.077021
EBNA3	SNX3	0.33759
EBNA3	OLA1	0.28034
EBNA3	ATP6V1D	0.26704
EBNA3	TDP1	0.2869
EBNA3	ABL2	0.15087
EBNA3	DOCK3	0.37504
EBNA3	STX6	0.22569
EBNA3	DDX11	0.038395
EBNA3	MAK	0.088654
EBNA3	CYLD	0.086412
EBNA3	SZRD1	0.040943
EBNA3	GP6	0.30449
EBNA3	SNW1	0.21862
EBNA3	AC3.5	0.098027
EBNA3	DDX26B	0.13353
EBNA3	PPL	0.28725
EBNA3	KLK3	0.42715
EBNA3	SGIP1	0.18188
EBNA3	WAC	0.25848
EBNA3	TTR	0.36506
EBNA3	LCHN	0.035604
EBNA3	ATP2B4	0.22842
EBNA3	BBS7	0.2932
EBNA3	CRYAA	0.17066
EBNA3	NEDD4L	0.14869
EBNA3	PCDH9	0.17138
EBNA3	PPP6R2	0.36195
EBNA3	ITPKB	0.055443
EBNA3	HPCAL1	0.21072
EBNA3	SLC36A1	0.013473
EBNA3	MIS12	0.26454
EBNA3	ARF4	0.24567
EBNA3	ZNF274	0.13047
EBNA3	FAM45B	0.096749
EBNA3	DAPK1	0.21557
EBNA3	RNASET2	0.014199
EBNA3	SOX6	0.075964
EBNA3	FAM229B	0.081044
EBNA3	LINC00478	0.027819
EBNA3	ITGA3	0.29975
EBNA3	COG3	0.22152
EBNA3	TNIK	0.14278
EBNA3	BRD7	0.14177
EBNA3	RNASE1	0.35477
EBNA3	C2orf81	0.11269
EBNA3	NSF	0.17471
EBNA3	RBBP7	0.1991
EBNA3	PTRF	0.2045
EBNA3	ARIH1	0.27751
EBNA3	ECM1	0.19662
EBNA3	KLC2	0.29561
EBNA3	BEGAIN	0.41166
EBNA3	ARF6	0.13002
EBNA3	FAM84A	0.049209
EBNA3	PAAF1	0.38799
EBNA3	TREX2	0.051626
EBNA3	SNX21	0.12183
EBNA3	FLCN	0.35691
EBNA3	KISS1R	0.21307

EBNA3	DNASE1L3	0.13263
EBNA3	RIPK2	0.034532
EBNA3	SNRPD3	0.19909
EBNA3	SPANXA2-OT1	0.057182
EBNA3	CLEC2A	0.39293
EBNA3	CTSB	0.09384
EBNA3	ILKAP	0.22625
EBNA3	CLIC4	0.23639
EBNA3	ASIP	0.16462
EBNA3	PUS3	0.13074
EBNA3	RAB14	0.08662
EBNA3	STX12	0.24898
EBNA3	SAMHD1	0.26802
EBNA3	ACTB	0.10123
EBNA3	EIF4G1	0.29987
EBNA3	SLC22A5	0.24239
EBNA3	KIF3A	0.17234
EBNA3	SCNN1A	0.27643
EBNA3	CGREF1	0.17266
EBNA3	CHAF1B	0.21707
EBNA3	RUSC1	0.27338
EBNA3	NPNT	0.15146
EBNA3	PEX12	0.27879
EBNA3	FLOT1	0.27251
EBNA3	FAM49B	0.045002
EBNA3	BNIP2	0.43713
EBNA3	ZNF767	0.059415
EBNA3	GABARAPL2	0.30664
EBNA3	FAM199X	0.040174
EBNA3	PPIH	0.30294
EBNA3	RARS	0.14534
EBNA3	PRKDC	0.075273
EBNA3	SULF1	0.041427
EBNA3	JAG2	0.37408
EBNA3	RGR	0.30595
EBNA3	MYRIP	0.29085
EBNA3	CTTN	0.36235
EBNA3	C1QA	0.35738
EBNA3	PPP1R16A	0.41167
EBNA3	EFCAB7	0.21072
EBNA3	TACC1	0.25021
EBNA3	CCDC114	0.010537
EBNA3	CREB3	0.049704
EBNA3	LTBP3	0.15541
EBNA3	HDGFL1	0.010436
EBNA3	SWT1	0.043627
EBNA3	PAGE4	0.15604
EBNA3	PLK4	0.17647
EBNA3	SKIV2L2	0.22391
EBNA3	ADRB2	0.054513
EBNA3	OLAH	0.070961
EBNA3	DDX18	0.35066
EBNA3	GAGE12G	0.061456
EBNA3	ATP2B1	0.25362
EBNA3	POLR1B	0.20407
EBNA3	VIMP	0.15047
EBNA3	KMT2D	0.2338
EBNA3	CEP97	0.38292
EBNA3	PTPRU	0.13896
EBNA3	TPCN2	0.11198
EBNA3	RUNX1T1	0.14449
EBNA3	CENPI	0.22405
EBNA3	FAM117A	0.094915
EBNA3	SHC3	0.31319
EBNA3	LGALS3	0.2636
EBNA3	FCGR2B	0.33729
EBNA3	DYSF	0.07504
EBNA3	IST1	0.487

23597	EBNA3	MAP4K5	0.22305
23598	EBNA3	NR5A2	0.045305
23599	EBNA3	FCHO2	0.29003
23600	EBNA3	TIPRL	0.40743
23601	EBNA3	SKP2	0.24366
23602	EBNA3	PLAA	0.38684
23603	EBNA3	NR1H4	0.045874
23604	EBNA3	DUSP15	0.12758
23605	EBNA3	MIB2	0.2491
23606	EBNA3	EHD2	0.36086
23607	EBNA3	PVRL2	0.14634
23608	EBNA3	HS3ST3A1	0.25013
23609	EBNA3	CCDC13	0.010083
23610	EBNA3	COPS5	0.2392
23611	EBNA3	FGGY	0.071722
23612	EBNA3	RNF135	0.28368
23613	EBNA3	CCNE2	0.1727
23614	EBNA3	C5AR2	0.29695
23615	EBNA3	CADPS	0.53813
23616	EBNA3	JUND	0.13281
23617	EBNA3	MARCKSL1	0.11175
23618	EBNA3	ABCA1	0.054124
23619	EBNA3	MARK2	0.13581
23620	EBNA3	CNTROB	0.31852
23621	EBNA3	CDC42SE2	0.11477
23622	EBNA3	HEXIM2	0.27337
23623	EBNA3	HRNR	0.15926
23624	EBNA3	PKD2	0.04221
23625	EBNA3	NCAPH	0.31905
23626	EBNA3	CREBRF	0.15205
23627	EBNA3	TRMT10B	0.059675
23628	EBNA3	AURKA	0.060239
23629	EBNA3	ATG9B	0.10079
23630	EBNA3	PCDHGB2	0.18006
23631	EBNA3	BBS4	0.068855
23632	EBNA3	PDZD7	0.36845
23633	EBNA3	C17orf59	0.18409
23634	EBNA3	PPP2CB	0.12453
23635	EBNA3	DLC1	0.1299
23636	EBNA3	STXBP4	0.142
23637	EBNA3	FOXC1	0.019705
23638	EBNA3	IPO9	0.31576
23639	EBNA3	ANXA8L1	0.17401
23640	EBNA3	PDIA6	0.16493
23641	EBNA3	DRC1	0.029286
23642	EBNA3	HDAC5	0.050671
23643	EBNA3	TRPC6	0.21843
23644	EBNA3	ZNF655	0.21682
23645	EBNA3	ETF1	0.33397
23646	EBNA3	DCSTAMP	0.20204
23647	EBNA3	EMILIN1	0.32303
23648	EBNA3	CRYGD	0.35089
23649	EBNA3	ADRM1	0.27077
23650	EBNA3	KLK6	0.25751
23651	EBNA3	FAM161B	0.17016
23652	EBNA3	CCAR1	0.49005
23653	EBNA3	PFDN4	0.28582
23654	EBNA3	C19orf57	0.40094
23655	EBNA3	LPCAT1	0.080803
23656	EBNA3	HIST1H3A	0.23984
23657	EBNA3	GSTO2	0.011368
23658	EBNA3	CCNA1	0.17727
23659	EBNA3	MYCN	0.13067
23660	EBNA3	NKX2-1	0.0372
23661	EBNA3	SPARCL1	0.20236
23662	EBNA3	C16orf93	0.059931
23663	EBNA3	PDZD3	0.22486
23664	EBNA3	CD40	0.10521

EBNA3	ENPP1	0.092048
EBNA3	MED24	0.060259
EBNA3	PPP1R12A	0.17243
EBNA3	ERC1	0.18816
EBNA3	CTNND1	0.14304
EBNA3	PICALM	0.11514
EBNA3	PLEKHG6	0.17476
EBNA3	GUCA1A	0.20052
EBNA3	SEMA7A	0.25963
EBNA3	CRMP1	0.13883
EBNA3	C20orf62	0.025292
EBNA3	LITAF	0.25007
EBNA3	TGFA	0.17194
EBNA3	C6orf222	0.042181
EBNA3	IQCB1	0.41642
EBNA3	CCDC171	0.048471
EBNA3	CCAR2	0.35091
EBNA3	CACTIN	0.24722
EBNA3	PLAUR	0.17943
EBNA3	FAM178B	0.065733
EBNA3	UTP20	0.28046
EBNA3	AP1S3	0.01679
EBNA3	DVL1	0.070689
EBNA3	GINS2	0.33579
EBNA3	WDR66	0.21072
EBNA3	MAP3K1	0.034219
EBNA3	LOXL2	0.099525
EBNA3	CASP3	0.09959
EBNA3	SHC1	0.080303
EBNA3	MITF	0.09611
EBNA3	ZNF217	0.10823
EBNA3	PUS1	0.093073
EBNA3	MPHOSPH6	0.37693
EBNA3	GTF2A1	0.19597
EBNA3	NLGN4Y	0.28468
EBNA3	SLC2A2	0.066417
EBNA3	ELF1	0.17332
EBNA3	COPS3	0.33761
EBNA3	ZMYND8	0.29831
EBNA3	COMMD6	0.34762
EBNA3	LONRF3	0.34232
EBNA3	GABARAP	0.24822
EBNA3	WWP2	0.12231
EBNA3	C1orf195	0.21058
EBNA3	TGFB1I1	0.16788
EBNA3	PIKFYVE	0.22109
EBNA3	MEX3B	0.15889
EBNA3	FBXW9	0.43795
EBNA3	CD37	0.3754
EBNA3	STAM	0.28591
EBNA3	GSK3A	0.069661
EBNA3	CBLB	0.23573
EBNA3	CRELD2	0.67994
EBNA3	PPP2R5A	0.20618
EBNA3	BLOC1S2	0.21228
EBNA3	MYOZ1	0.34501
EBNA3	TMEM30A	0.37569
EBNA3	PRUNE	0.31164
EBNA3	NCOA3	0.16239
EBNA3	MUM1	0.38214
EBNA3	TIMELESS	0.23095
EBNA3	NEK9	0.10643
EBNA3	OSBPL3	0.32513
EBNA3	NPIPA5	0.018436
EBNA3	PTK2	0.021387
EBNA3	NIF3L1	0.323
EBNA3	MZF1	0.13779
EBNA3	SLC34A3	0.075532

EBNA3	BANF1	0.27502
EBNA3	KXD1	0.41016
EBNA3	CPSF6	0.3149
EBNA3	MAST3	0.24116
EBNA3	FKBP14	0.16853
EBNA3	TOX4	0.34808
EBNA3	ARHGAP44	0.33816
EBNA3	ORC1	0.2038
EBNA3	LEO1	0.24211
EBNA3	CSRP2BP	0.2616
EBNA3	PRR23A	0.047737
EBNA3	RORA	0.10707
EBNA3	ARL14EP	0.4246
EBNA3	NSMCE1	0.25436
EBNA3	MED7	0.22908
EBNA3	CLIC6	0.018043
EBNA3	WAPAL	0.19517
EBNA3	TNFSF10	0.17573
EBNA3	GP1BB	0.31148
EBNA3	REL	0.15719
EBNA3	PCDHB10	0.16421
EBNA3	BLCAP	0.387
EBNA3	AIMP2	0.28336
EBNA3	C2orf72	0.10345
EBNA3	APOA1BP	0.33809
EBNA3	RPS7	0.20429
EBNA3	LILRB2	0.19243
EBNA3	YBR056C-B	0.067479
EBNA3	PFKFB1	0.16916
EBNA3	URI1	0.17967
EBNA3	MNAT1	0.026299
EBNA3	EME1	0.2933
EBNA3	APPL2	0.19149
EBNA3	RBM26	0.3992
EBNA3	PSMG1	0.39963
EBNA3	SEC61B	0.21714
EBNA3	KIF2A	0.17483
EBNA3	PATL2	0.34341
EBNA3	IL7	0.20671
EBNA3	SEPT14	0.34495
EBNA3	NBR1	0.35159
EBNA3	SENP6	0.27322
EBNA3	EFCAB13	0.16291
EBNA3	HFE	0.25985
EBNA3	DPM1	0.22666
EBNA3	SF3A3	0.24042
EBNA3	MMP28	0.062247
EBNA3	IFT20	0.29941
EBNA3	GHR	0.045076
EBNA3	FBXW7	0.10762
EBNA3	MAGEA6	0.40098
EBNA3	RAB24	0.32065
EBNA3	RAB8A	0.23609
EBNA3	PFKL	0.20215
EBNA3	CXADR	0.079041
EBNA3	RAB11FIP2	0.21813
EBNA3	TRO	0.31483
EBNA3	ATP11C	0.22083
EBNA3	CD27	0.24871
EBNA3	LYST	0.19432
EBNA3	CASQ1	0.11396
EBNA3	SLC5A6	0.011552
EBNA3	ZNF652	0.25167
EBNA3	C1orf87	0.21072
EBNA3	CEP57	0.2091
EBNA3	SCIMP	0.33612
EBNA3	TIPIN	0.26178
EBNA3	CHIC2	0.40426

EBNA3	BNIP3L	0.14016
EBNA3	ARMC12	0.051953
EBNA3	AES	0.19237
EBNA3	STX18	0.27493
EBNA3	VAMP7	0.15939
EBNA3	N4BP2	0.33095
EBNA3	SDC1	0.12771
EBNA3	TRAM1	0.27406
EBNA3	DCAF8	0.42039
EBNA3	DNM1L	0.078313
EBNA3	NSUN3	0.033187
EBNA3	DLGAP3	0.29009
EBNA3	USP9X	0.21861
EBNA3	PDIA2	0.23866
EBNA3	DERL1	0.22906
EBNA3	RSF1	0.16884
EBNA3	SMG8	0.34793
EBNA3	GSN	0.29022
EBNA3	RELB	0.16126
EBNA3	SLC11A2	0.064099
EBNA3	FAM96B	0.2954
EBNA3	TRMT112	0.48058
EBNA3	FXN	0.013288
EBNA3	NUP133	0.18257
EBNA3	PCDHGA4	0.17529
EBNA3	PAIP2	0.37405
EBNA3	PRKD1	0.014661
EBNA3	NABP2	0.31804
EBNA3	GCSAM	0.35276
EBNA3	AMBRA1	0.41203
EBNA3	ADCY4	0.13317
EBNA3	MET	0.051341
EBNA3	TREML2	0.36473
EBNA3	PKP2	0.18874
EBNA3	SLC12A2	0.024592
EBNA3	VPS13A	0.33621
EBNA3	BMP2	0.017777
EBNA3	MME	0.094716
EBNA3	CEBPA	0.052413
EBNA3	PTPN22	0.082389
EBNA3	CSNK1D	0.11021
EBNA3	C3orf22	0.088431
EBNA3	EXOC3L1	0.04281
EBNA3	HADHA	0.14447
EBNA3	PCDHGA3	0.18006
EBNA3	EXOC3L4	0.061267
EBNA3	TFB2M	0.11916
EBNA3	TBKBP1	0.4327
EBNA3	PPP1R27	0.3912
EBNA3	EIF4H	0.3683
EBNA3	AK3	0.013266
EBNA3	HK1	0.23333
EBNA3	BPY2	0.37772
EBNA3	TELO2	0.32132
EBNA3	FRMPD2	0.34394
EBNA3	CCNB3	0.20255
EBNA3	PVRL1	0.19126
EBNA3	IGFBP3	0.18072
EBNA3	CYCB3-1	0.12254
EBNA3	ASCL1	0.033124
EBNA3	UBA2	0.11219
EBNA3	IGFBP7	0.33867
EBNA3	S100A16	0.14662
EBNA3	BLNK	0.30465
EBNA3	RPGRIP1L	0.17786
EBNA3	ILF2	0.28025
EBNA3	POT1	0.18717
EBNA3	GDI1	0.29894

EBNA3	PPFIA3	0.38703
EBNA3	BMPR1B	0.08384
EBNA3	PAM	0.16429
EBNA3	CNTRL	0.33115
EBNA3	CDK13	0.078934
EBNA3	GOLGA2	0.39177
EBNA3	PHACTR2	0.41057
EBNA3	TMEM97	0.2842
EBNA3	COA7	0.025662
EBNA3	TRPC5	0.2553
EBNA3	SP100	0.14031
EBNA3	EIF4A1	0.28919
EBNA3	TRUB1	0.051547
EBNA3	TDRD7	0.31049
EBNA3	MRPS12	0.28618
EBNA3	CABP1	0.35229
EBNA3	RHNO1	0.3432
EBNA3	DDX5	0.13101
EBNA3	PALD1	0.41224
EBNA3	RBBP5	0.26339
EBNA3	PILRA	0.34169
EBNA3	YBX1	0.10528
EBNA3	EAF1	0.33019
EBNA3	AKAP1	0.14015
EBNA3	BEND3	0.011816
EBNA3	SCEL	0.3061
EBNA3	SLC4A4	0.3277
EBNA3	PKN3	0.18876
EBNA3	ARF5	0.25394
EBNA3	C22orf26	0.05036
EBNA3	LETM1	0.65846
EBNA3	TNFAIP8L1	0.12611
EBNA3	CRH	0.16746
EBNA3	EIF2AK2	0.13634
EBNA3	FSTL5	0.20437
EBNA3	NDRG1	0.20529
EBNA3	pkmyt1	0.069132
EBNA3	CINP	0.36946
EBNA3	PLA2G10	0.034336
EBNA3	TPR	0.17138
EBNA3	NEFM	0.27427
EBNA3	LEMD3	0.24553
EBNA3	BAG3	0.32366
EBNA3	PGBD4	0.032688
EBNA3	SPHK1	0.16174
EBNA3	CRYGN	0.051846
EBNA3	PACSIN3	0.32211
EBNA3	MRPS7	0.06538
EBNA3	VAPA	0.13973
EBNA3	SCARF2	0.36643
EBNA3	MAPRE1	0.19531
EBNA3	CATSPER2	0.21373
EBNA3	GCA	0.20273
EBNA3	FNIP1	0.41137
EBNA3	TCTEX1D2	0.43795
EBNA3	VRK2	0.14048
EBNA3	SMG1	0.1944
EBNA3	ARAF	0.16644
EBNA3	SRSF10	0.27375
EBNA3	GPC3	0.20251
EBNA3	LRRC3	0.41166
EBNA3	RFC1	0.13091
EBNA3	PPP4R1	0.34111
EBNA3	TSSK2	0.16057
EBNA3	CUTA	0.37565
EBNA3	ELL	0.23975
EBNA3	CTU1	0.39328
EBNA3	AKT2	0.030962

EBNA3	PIAS4	0.17357
EBNA3	BCAS3	0.36585
EBNA3	ANGPT2	0.1147
EBNA3	MAD2L1BP	0.32679
EBNA3	ACTR1B	0.31087
EBNA3	PIK3CB	0.1589
EBNA3	RHOB	0.10272
EBNA3	EFHD2	0.22455
EBNA3	CYP4F2	0.070985
EBNA3	FBF1	0.31139
EBNA3	L3MBTL1	0.080056
EBNA3	MRPL12	0.39598
EBNA3	NFYC	0.13656
EBNA3	GTF2F1	0.11724
EBNA3	PKN1	0.071851
EBNA3	IL1RL1	0.22347
EBNA3	NSMAF	0.3607
EBNA3	NCAN	0.10784
EBNA3	FAM231A	0.33319
EBNA3	TFB1M	0.36718
EBNA3	NCOA5	0.33039
EBNA3	ALG1	0.13445
EBNA3	C6orf229	0.060313
EBNA3	ACHE	0.08394
EBNA3	DSG3	0.15345
EBNA3	FAM124A	0.21976
EBNA3	SLC1A3	0.02296
EBNA3	YAP1	0.11098
EBNA3	MATR3	0.25019
EBNA3	PCDHB18	0.18006
EBNA3	PPP2CA	0.081754
EBNA3	CEP164	0.2917
EBNA3	MLLT1	0.2838
EBNA3	RCC1	0.1991
EBNA3	PABPC1	0.18824
EBNA3	PLEKHF2	0.24926
EBNA3	JMY	0.25691
EBNA3	SNTB1	0.22093
EBNA3	MMP27	0.060454
EBNA3	CHST6	0.032992
EBNA3	AMER1	0.33432
EBNA3	TRIP11	0.26605
EBNA3	RTF1	0.19216
EBNA3	RWDD3	0.37995
EBNA3	PRKAG1	0.053422
EBNA3	MT3	0.060299
EBNA3	LUC7L3	0.30388
EBNA3	RGN	0.079353
EBNA3	MCM5	0.21425
EBNA3	CD28	0.16736
EBNA3	CTDSP1	0.2131
EBNA3	HLA-DRB1	0.082704
EBNA3	PNRC1	0.33039
EBNA3	IRS1	0.031538
EBNA3	ITIH1	0.17542
EBNA3	PRTN3	0.35135
EBNA3	TBX5	0.031396
EBNA3	MAL	0.32049
EBNA3	OOEP	0.12181
EBNA3	FAM188A	0.64536
EBNA3	ZP3	0.083089
EBNA3	BCKDK	0.21754
EBNA3	MCF2	0.19517
EBNA3	UMOD	0.14611
EBNA3	C6	0.2984
EBNA3	HDHD3	0.36947
EBNA3	IPO8	0.31122
EBNA3	RNF43	0.17796

EBNA3	MAGEB4	0.017325
EBNA3	STX2	0.20246
EBNA3	RHBDL3	0.2983
EBNA3	AP1B1	0.30403
EBNA3	CCNT1	0.11836
EBNA3	GAPDH	0.20997
EBNA3	NRD1	0.097896
EBNA3	DNM2	0.056755
EBNA3	PANK1	0.089776
EBNA3	GRB10	0.20328
EBNA3	NBN	0.088058
EBNA3	RCN3	0.72203
EBNA3	MGST1	0.14215
EBNA3	RNF5	0.22234
EBNA3	PLSCR1	0.3015
EBNA3	FRRS1	0.088285
EBNA3	CETN3	0.1946
EBNA3	METTL25	0.056183
EBNA3	SNX7	0.12183
EBNA3	CTNNBL1	0.32205
EBNA3	KDM2B	0.17012
EBNA3	GAGE5	0.031848
EBNA3	FDXR	0.2613
EBNA3	FLT1	0.057938
EBNA3	GNPNAT1	0.097087
EBNA3	BCLAF1	0.24508
EBNA3	CALY	0.096251
EBNA3	EFTUD2	0.24533
EBNA3	ITSN2	0.054869
EBNA3	SERPINE2	0.075691
EBNA3	YY1AP1	0.30228
EBNA3	NF1	0.047609
EBNA3	F8	0.29121
EBNA3	FBXO45	0.31904
EBNA3	NSG2	0.085477
EBNA3	KIF21A	0.3255
EBNA3	SCAMP2	0.2374
EBNA3	PCDH10	0.18006
EBNA3	GIT1	0.24703
EBNA3	CCL5	0.013499
EBNA3	GRIN2B	0.022988
EBNA3	GAGE12B	0.061456
EBNA3	SLC6A4	0.13977
EBNA3	CDH19	0.17459
EBNA3	VEGFC	0.16839
EBNA3	CLK2	0.19637
EBNA3	SLC12A3	0.29881
EBNA3	SNAP25	0.096215
EBNA3	DDX23	0.21105
EBNA3	LSM10	0.28208
EBNA3	SUPV3L1	0.1395
EBNA3	PSENEN	0.23488
EBNA3	ATN1	0.23083
EBNA3	LRPPRC	0.24103
EBNA3	VAPB	0.14256
EBNA3	CARM1	0.01532
EBNA3	NHEJ1	0.29055
EBNA3	ESR1	0.023638
EBNA3	STAT5B	0.045727
EBNA3	COPRS	0.3023
EBNA3	RPA1	0.10551
EBNA3	HSPG2	0.15729
EBNA3	CELSR2	0.092581
EBNA3	SMARCA4	0.11425
EBNA3	SGCA	0.16281
EBNA3	ATG5	0.26137
EBNA3	ACTL6A	0.17427
EBNA3	PAXIP1	0.22832

EBNA3	EGFR	0.027962
EBNA3	ACTR8	0.30278
EBNA3	IREB2	0.050777
EBNA3	CAMLG	0.36178
EBNA3	NKAPL	0.1497
EBNA3	SELP	0.12371
EBNA3	FBXO5	0.10407
EBNA3	NCKAP5L	0.12454
EBNA3	PTGDR	0.31771
EBNA3	HCST	0.17209
EBNA3	APAF1	0.19137
EBNA3	SCGB3A1	0.16307
EBNA3	ENTPD7	0.06194
EBNA3	SIKE1	0.33951
EBNA3	CTCF	0.073247
EBNA3	CCDC153	0.015073
EBNA3	HSPD1	0.063951
EBNA3	PPFIA2	0.39909
EBNA3	PCDHGA8	0.18006
EBNA3	MMP8	0.065219
EBNA3	PRR21	0.048324
EBNA3	MED12	0.037242
EBNA3	SAYSD1	0.082049
EBNA3	MST1R	0.18762
EBNA3	SCIN	0.12618
EBNA3	CCDC33	0.43795
EBNA3	NPC1	0.14409
EBNA3	SYTL5	0.33989
EBNA3	HINFP	0.084959
EBNA3	MORC4	0.33541
EBNA3	MPLKIP	0.336
EBNA3	PLP2	0.32858
EBNA3	TLDC2	0.098475
EBNA3	RAB11A	0.18365
EBNA3	LTBP1	0.65182
EBNA3	KIF5A	0.23865
EBNA3	PEX6	0.28658
EBNA3	SMARCC1	0.21103
EBNA3	TPD52L2	0.41819
EBNA3	C6orf165	0.013526
EBNA3	ASPSCR1	0.28696
EBNA3	ARL6IP4	0.37995
EBNA3	MSANTD1	0.02438
EBNA3	F2	0.18976
EBNA3	CELF1	0.2695
EBNA3	RHOXF2	0.20826
EBNA3	WDYHV1	0.30885
EBNA3	SYT7	0.22012
EBNA3	FAAP20	0.28111
EBNA3	DLG1	0.026419
EBNA3	THOC2	0.39736
EBNA3	HES1	0.019143
EBNA3	PLEKHA2	0.21138
EBNA3	DPP6	0.011569
EBNA3	EREG	0.12352
EBNA3	IFIT2	0.30654
EBNA3	HEATR1	0.28594
EBNA3	SCNN1B	0.26397
EBNA3	SMYD2	0.23178
EBNA3	ITPR1	0.13472
EBNA3	VAMP3	0.2428
EBNA3	WIPI2	0.26441
EBNA3	CDH9	0.15614
EBNA3	SPAST	0.15184
EBNA3	PCDHB9	0.16421
EBNA3	RRAS	0.23179
EBNA3	SMEK3P	0.060005
EBNA3	MIEF2	0.3013

EBNA3	FST	0.21039
EBNA3	CEP290	0.18992
EBNA3	KDM5B	0.1058
EBNA3	NOXO1	0.24081
EBNA3	SYT1	0.24347
EBNA3	NSMCE2	0.20554
EBNA3	MRPS16	0.31604
EBNA3	RND2	0.33626
EBNA3	HMOX2	0.224
EBNA3	WRNIP1	0.26168
EBNA3	TCOF1	0.35854
EBNA3	CBL	0.25044
EBNA3	NEK3	0.2153
EBNA3	CORO1A	0.13555
EBNA3	N4BP3	0.082048
EBNA3	METTL20	0.049276
EBNA3	PHF12	0.21719
EBNA3	SLX4	0.28305
EBNA3	CHST3	0.030574
EBNA3	ANKRD27	0.35283
EBNA3	EPHA2	0.086205
EBNA3	MYC	0.039552
EBNA3	ARFRP1	0.24789
EBNA3	UCHL5	0.15673
EBNA3	PRPF8	0.32128
EBNA3	VIM	0.099644
EBNA3	NOA1	0.24999
EBNA3	CTAG1A	0.4246
EBNA3	PEX26	0.3031
EBNA3	WARS	0.21221
EBNA3	SRXN1	0.020613
EBNA3	C11orf52	0.033782
EBNA3	TIMM10	0.22222
EBNA3	SLC25A13	0.1274
EBNA3	KLRC2	0.35309
EBNA3	NPM1	0.059513
EBNA3	GDF2	0.15691
EBNA3	NAP1L4	0.29916
EBNA3	NSUN5P1	0.070407
EBNA3	MYCBP	0.28416
EBNA3	PI4K2A	0.23394
EBNA3	GRB2	0.049158
EBNA3	ADNP2	0.11286
EBNA3	SLC38A1	0.033004
EBNA3	SHPK	0.070202
EBNA3	ZNF639	0.092936
EBNA3	TRMT10A	0.047257
EBNA3	NCS1	0.4577
EBNA3	TCAP	0.22278
EBNA3	HPSE	0.15933
EBNA3	CDK2	0.050677
EBNA3	MBL2	0.20833
EBNA3	C12orf71	0.021331
EBNA3	SRRM5	0.029457
EBNA3	NCK2	0.21418
EBNA3	CCDC81	0.028838
EBNA3	IKZF3	0.10539
EBNA3	NMNAT1	0.29788
EBNA3	GPKOW	0.34779
EBNA3	FBLN5	0.53027
EBNA3	MPP6	0.39913
EBNA3	ARHGAP1	0.21014
EBNA3	CRBN	0.2802
EBNA3	HSCB	0.3524
EBNA3	BIRC5	0.065294
EBNA3	DAG1	0.15506
EBNA3	AP5S1	0.29682
EBNA3	MCM3	0.15052

```
EBNA3	TOMM20	0.21329
EBNA3	GLTSCR1	0.43795
EBNA3	OTOF	0.15528
EBNA3	AGTRAP	0.3693
EBNA3	C1orf192	0.062255
EBNA3	AP1S1	0.015283
EBNA3	ID2	0.084798
EBNA3	ZNF133	0.14249
EBNA3	PACS1	0.29546
EBNA3	DOC2A	0.34712
EBNA3	MED30	0.28026
EBNA3	HRASLS5	0.010685
EBNA3	RHOBTB3	0.32524
EBNA3	XRCC3	0.28948
EBNA3	F11	0.3111
EBNA3	SRGAP2	0.16668
EBNA3	CALCOCO1	0.20766
EBNA3	FBXO6	0.31799
EBNA3	VPS35	0.28548
EBNA3	HRK	0.30531
EBNA3	ADAM22	0.23657
EBNA3	PI4K2B	0.032789
EBNA3	PCDHA12	0.18006
EBNA3	TBC1D9B	0.14196
EBNA3	C2orf62	0.025925
EBNA3	PTPRT	0.1045
EBNA3	SIGLEC8	0.36376
EBNA3	SNRNP40	0.28442
EBNA3	ARID1A	0.1756
EBNA3	SCNN1D	0.34662
EBNA3	STON2	0.32194
EBNA3	SNX22	0.12183
EBNA3	CENPE	0.13605
EBNA3	HSPA8	0.058284
EBNA3	TRUB2	0.051547
EBNA3	PLK2	0.14064
EBNA3	FBXW11	0.22165
EBNA3	SCAI	0.27806
EBNA3	PYGB	0.033305
EBNA3	KCNMB4	0.27353
EBNA3	AHR	0.030762
EBNA3	NIPBL	0.14825
EBNA3	RPL7A	0.27373
EBNA3	KMT2A	0.11267
EBNA3	UBE2C	0.16471
EBNA3	SERPINA3	0.2812
EBNA3	ADRBK1	0.065904
EBNA3	DOK1	0.18995
EBNA3	YAL042C-A	0.025004
EBNA3	IL7R	0.19581
EBNA3	MYL5	0.18277
EBNA3	TPM4	0.12085
EBNA3	NDUFAF3	0.33232
EBNA3	DYX1C1	0.329
EBNA3	BMF	0.23875
EBNA3	C19orf67	0.035924
EBNA3	INTS2	0.31933
EBNA3	RAB22A	0.2396
EBNA3	MYO7A	0.13698
EBNA3	PIAS2	0.17132
EBNA3	C5orf48	0.1215
EBNA3	MUC2	0.38171
EBNA3	ETNK1	0.06174
EBNA3	SPTLC1	0.20737
EBNA3	SH3BP5L	0.076775
EBNA3	CCDC17	0.024379
EBNA3	NLK	0.10705
EBNA3	ITGA6	0.27441
```

EBNA3	PILRB	0.37481
EBNA3	CTNNA2	0.15876
EBNA3	DEPTOR	0.36432
EBNA3	FTH1	0.24351
EBNA3	NOD1	0.080906
EBNA3	RCAN1	0.15052
EBNA3	DGKB	0.18833
EBNA3	ZFYVE9	0.35833
EBNA3	PDS5A	0.33418
EBNA3	TIGIT	0.29082
EBNA3	CARS	0.17517
EBNA3	RNF8	0.12622
EBNA3	DPEP1	0.14756
EBNA3	RFX5	0.18927
EBNA3	C8orf48	0.43795
EBNA3	NPHS1	0.22513
EBNA3	TAL1	0.037614
EBNA3	NECAB3	0.54624
EBNA3	AKAP8L	0.30334
EBNA3	SNX12	0.41709
EBNA3	ATM	0.076098
EBNA3	SYNGR4	0.21021
EBNA3	AQP6	0.049493
EBNA3	TCHH	0.15928
EBNA3	RASSF9	0.33914
EBNA3	GORASP2	0.32506
EBNA3	KIR2DL4	0.34233
EBNA3	MLC1	0.24622
EBNA3	LBP	0.12807
EBNA3	ADAP2	0.077879
EBNA3	TRIM16	0.21727
EBNA3	IFI35	0.3662
EBNA3	EDA	0.11687
EBNA3	PXMP4	0.37531
EBNA3	ASGR2	0.31665
EBNA3	STAG1	0.26685
EBNA3	EFNA4	0.43365
EBNA3	SIN3A	0.061355
EBNA3	ATP5O	0.053282
EBNA3	DYNC1H1	0.21338
EBNA3	GRM7	0.065102
EBNA3	GUK1	0.066606
EBNA3	C19orf82	0.074651
EBNA3	GALK2	0.041709
EBNA3	CNTNAP1	0.18919
EBNA3	PBXIP1	0.26165
EBNA3	RRAGD	0.29868
EBNA3	TRMT61B	0.44015
EBNA3	CCR1	0.15841
EBNA3	SDC4	0.22428
EBNA3	LONP2	0.21724
EBNA3	HSPA14	0.33875
EBNA3	PLXNB2	0.26003
EBNA3	APH1B	0.31345
EBNA3	SNX6	0.35431
EBNA3	APOA5	0.13061
EBNA3	KRT1	0.24067
EBNA3	ZNF143	0.2017
EBNA3	BRCA1	0.038955
EBNA3	RFX1	0.30061
EBNA3	CDK20	0.14843
EBNA3	KRT81	0.34309
EBNA3	ATIC	0.091568
EBNA3	ERGIC1	0.26883
EBNA3	RALB	0.22444
EBNA3	CDC73	0.18071
EBNA3	GSE1	0.43795
EBNA3	NCOA1	0.13248

EBNA3	FGA	0.2659
EBNA3	RASGRP1	0.089561
EBNA3	SECISBP2L	0.10684
EBNA3	HLTF	0.14136
EBNA3	DUSP7	0.085831
EBNA3	PDE4A	0.18086
EBNA3	ZFYVE1	0.24538
EBNA3	IFNAR2	0.18207
EBNA3	NMI	0.34578
EBNA3	CLSTN3	0.10397
EBNA3	CDK5RAP3	0.23767
EBNA3	TRIM28	0.049456
EBNA3	PCDHGB6	0.18006
EBNA3	CHMP4A	0.10122
EBNA3	WWTR1	0.15503
EBNA3	C11orf96	0.078348
EBNA3	PCDHB11	0.15424
EBNA3	ASF1A	0.2276
EBNA3	CYGB	0.068211
EBNA3	ERRFI1	0.12786
EBNA3	C19orf25	0.43795
EBNA3	IRF7	0.046564
EBNA3	MAPK9	0.11734
EBNA3	BBS2	0.1876
EBNA3	CDKN2AIPNL	0.12381
EBNA3	APOA2	0.05473
EBNA3	XPA	0.22228
EBNA3	CHMP5	0.35071
EBNA3	EIF2S2	0.39731
EBNA3	ZFYVE16	0.17039
EBNA3	PUS10	0.054505
EBNA3	NMRK2	0.39249
EBNA3	PPARG	0.02066
EBNA3	PHB2	0.23654
EBNA3	INTS4L1	0.011506
EBNA3	DCP1B	0.23138
EBNA3	FAM92A1	0.028692
EBNA3	MYB	0.11971
EBNA3	CLVS1	0.040569
EBNA3	SNRPD1	0.26229
EBNA3	SOCS1	0.18938
EBNA3	AKAP7	0.084529
EBNA3	C1S	0.19523
EBNA3	NACA	0.034461
EBNA3	NPBWR1	0.29529
EBNA3	SNCB	0.12846
EBNA3	EGLN3	0.21268
EBNA3	NPC1L1	0.37297
EBNA3	PCGF2	0.11171
EBNA3	DNMBP	0.30195
EBNA3	PRAM1	0.45195
EBNA3	EFNB1	0.27403
EBNA3	NT5C3B	0.32815
EBNA3	CDYL2	0.32365
EBNA3	AASDHPPT	0.33279
EBNA3	FHL1	0.21047
EBNA3	NBEAP1	0.012061
EBNA3	GGA3	0.28617
EBNA3	UTP11L	0.29831
EBNA3	AMER3	0.1121
EBNA3	RSRC1	0.31707
EBNA3	MBOAT4	0.08886
EBNA3	GLRA1	0.11244
EBNA3	AQP5	0.29568
EBNA3	ARL4C	0.21662
EBNA3	NR0B2	0.13037
EBNA3	ARHGAP21	0.27518
EBNA3	ARL3	0.096576

EBNA3	MEGF6	0.70252
EBNA3	MED20	0.21099
EBNA3	RPS6KA1	0.066483
EBNA3	STAG2	0.25142
EBNA3	EIF2S3	0.32376
EBNA3	NFRKB	0.28638
EBNA3	LAX1	0.19899
EBNA3	CALR	0.067781
EBNA3	JPH2	0.2438
EBNA3	BTBD1	0.37331
EBNA3	RSRC2	0.11886
EBNA3	SOCS5	0.33069
EBNA3	EMX2	0.11449
EBNA3	MRPL11	0.35537
EBNA3	EGR1	0.072856
EBNA3	MC2R	0.24874
EBNA3	NUP205	0.25442
EBNA3	RAB33A	0.29167
EBNA3	DHX9	0.17492
EBNA3	HOXA10	0.14725
EBNA3	HSP90AA1	0.18025
EBNA3	TAF1	0.081104
EBNA3	GAS1	0.10821
EBNA3	C16orf45	0.043621
EBNA3	TSPY1	0.25753
EBNA3	GOLGA6L9	0.093196
EBNA3	TNFRSF14	0.26142
EBNA3	LYL1	0.28635
EBNA3	NUB1	0.34052
EBNA3	PIK3C2A	0.037431
EBNA3	NFKBIL1	0.32861
EBNA3	CBX5	0.16187
EBNA3	ZFAND2B	0.35851
EBNA3	F12	0.24421
EBNA3	PCBD1	0.15765
EBNA3	NSUN6	0.045962
EBNA3	NRBP1	0.16024
EBNA3	SLC25A12	0.11986
EBNA3	ALS2CR11	0.032402
EBNA3	ATF4	0.10486
EBNA3	PCNP	0.36197
EBNA3	ATP6AP2	0.30805
EBNA3	PCDHA1	0.16404
EBNA3	ANK1	0.11599
EBNA3	PCBP1	0.34027
EBNA3	ZNRD1-AS1	0.026137
EBNA3	HSF1	0.13146
EBNA3	UNC5B	0.30026
EBNA3	SCRIB	0.17197
EBNA3	RPUSD2	0.052937
EBNA3	RPTOR	0.1344
EBNA3	SRRT	0.2585
EBNA3	SSTR3	0.21574
EBNA3	DGKG	0.17379
EBNA3	KHDRBS2	0.34787
EBNA3	MMP9	0.068592
EBNA3	PLXNB3	0.32009
EBNA3	KCNIP2	0.33571
EBNA3	TBCD	0.25814
EBNA3	THEG	0.34698
EBNA3	SLPI	0.31769
EBNA3	PDLIM4	0.36919
EBNA3	DAO	0.20739
EBNA3	PCDHA13	0.18006
EBNA3	FBRSL1	0.068596
EBNA3	KIF3B	0.20495
EBNA3	PRSS8	0.34883
EBNA3	FSCN1	0.28389

EBNA3	TRAF1	0.28128
EBNA3	CALML3	0.21072
EBNA3	PROX1	0.025766
EBNA3	POLG2	0.24462
EBNA3	ITGB1BP1	0.34108
EBNA3	MED25	0.18997
EBNA3	RAMP3	0.22931
EBNA3	LRRC14	0.019091
EBNA3	AGO4	0.31085
EBNA3	HPS1	0.30642
EBNA3	CRK	0.22576
EBNA3	RFWD2	0.21971
EBNA3	SPOCK3	0.16305
EBNA3	PIAS3	0.16441
EBNA3	CDH4	0.1351
EBNA3	TNS1	0.34261
EBNA3	ITGA2B	0.23412
EBNA3	PAFAH1B1	0.036051
EBNA3	MAPK13	0.18757
EBNA3	ADAM12	0.15848
EBNA3	UBA52	0.033235
EBNA3	INO80E	0.31322
EBNA3	FOXL2	0.036723
EBNA3	KCNIP3	0.073658
EBNA3	SNRPN	0.36447
EBNA3	ZK1128.3	0.12171
EBNA3	CDK14	0.12824
EBNA3	PARD6A	0.16783
EBNA3	CUEDC1	0.052242
EBNA3	SMARCA2	0.10337
EBNA3	PLCG1	0.12973
EBNA3	HEATR5B	0.1006
EBNA3	FAM109B	0.30214
EBNA3	ITGAX	0.29757
EBNA3	PDCD4	0.36213
EBNA3	CTAGE15	0.022666
EBNA3	PPP1R32	0.41727
EBNA3	PPP1R8	0.2513
EBNA3	NOTCH1	0.019004
EBNA3	PCLO	0.13321
EBNA3	IL8	0.17508
EBNA3	FBN2	0.17049
EBNA3	ZNHIT1	0.39642
EBNA3	TRAF4	0.11013
EBNA3	EYA2	0.098599
EBNA3	LINC00303	0.023599
EBNA3	ERCC1	0.076741
EBNA3	PSME3	0.12897
EBNA3	ELOVL2	0.25017
EBNA3	EFCAB10	0.19787
EBNA3	CD93	0.48056
EBNA3	MUC7	0.37466
EBNA3	SRR	0.097491
EBNA3	EEF1B2	0.32046
EBNA3	CCDC54	0.022053
EBNA3	PPP1R2	0.36066
EBNA3	MAN1A1	0.083615
EBNA3	ADAT1	0.030754
EBNA3	CAPN12	0.089042
EBNA3	TEX11	0.32161
EBNA3	LMX1B	0.14334
EBNA3	PLA2G2F	0.063794
EBNA3	NLRC5	0.21382
EBNA3	DRD1	0.030859
EBNA3	STK36	0.06956
EBNA3	RITA1	0.28589
EBNA3	SYT8	0.014604
EBNA3	GRIN1	0.21164

EBNA3	CST3	0.094652
EBNA3	FAN1	0.25893
EBNA3	CLINT1	0.42074
EBNA3	IRF5	0.1039
EBNA3	EIF2AK3	0.078729
EBNA3	C20orf195	0.047088
EBNA3	C19orf66	0.10083
EBNA3	ZBTB21	0.23667
EBNA3	MARK4	0.18923
EBNA3	BBS12	0.35351
EBNA3	DHH	0.44068
EBNA3	SLC25A4	0.15913
EBNA3	EIF4E1B	0.019803
EBNA3	SPATA21	0.21072
EBNA3	NDFIP2	0.21362
EBNA3	IK	0.35731
EBNA3	NCOA2	0.08959
EBNA3	CRIPAK	0.32385
EBNA3	PIGS	0.29754
EBNA3	SIRT2	0.085266
EBNA3	IMMT	0.34656
EBNA3	UBE2L3	0.13862
EBNA3	IL5	0.2732
EBNA3	TOMM7	0.24864
EBNA3	RRM1	0.261
EBNA3	ILK	0.038046
EBNA3	GAGE2E	0.031848
EBNA3	FLT4	0.10973
EBNA3	MLF1	0.27984
EBNA3	ANXA9	0.087484
EBNA3	STRN3	0.16278
EBNA3	RAP1B	0.18479
EBNA3	UNQ6125/PRO20090	0.1156
EBNA3	IKBKG	0.096413
EBNA3	SPHK2	0.30623
EBNA3	MARK3	0.25211
EBNA3	RPS20	0.061927
EBNA3	MECP2	0.01926
EBNA3	HDAC1	0.032
EBNA3	NR2F6	0.11348
EBNA3	VIPAS39	0.29698
EBNA3	ADAM15	0.21286
EBNA3	PRKCB	0.07152
EBNA3	PLD3	0.19078
EBNA3	PCDHGA10	0.18006
EBNA3	ADAM8	0.12293
EBNA3	TRIP12	0.29144
EBNA3	CSTF1	0.35944
EBNA3	SKA2	0.22652
EBNA3	U2AF2	0.30961
EBNA3	MAP3K8	0.1644
EBNA3	NOSTRIN	0.38482
EBNA3	TCL1A	0.27865
EBNA3	ELMO1	0.1859
EBNA3	SS18	0.23476
EBNA3	CT45A2	0.038676
EBNA3	U2SURP	0.4207
EBNA3	AICDA	0.16105
EBNA3	RPS9	0.17616
EBNA3	DCHS2	0.18006
EBNA3	SNX18	0.32865
EBNA3	TNFRSF13B	0.34419
EBNA3	SBDS	0.17072
EBNA3	PLCZ1	0.072965
EBNA3	N4BP2L1	0.026225
EBNA3	CDC42EP1	0.29762
EBNA3	ADRB1	0.077729
EBNA3	RPL28	0.28237

EBNA3	SIRT7	0.19869
EBNA3	AP1G2	0.30607
EBNA3	PREX1	0.1263
EBNA3	ATP2C2	0.22374
EBNA3	PCDHGC3	0.17455
EBNA3	PDCD10	0.27133
EBNA3	MRM1	0.14445
EBNA3	FAM231D	0.1539
EBNA3	USP44	0.24134
EBNA3	ZBTB17	0.1118
EBNA3	PURA	0.066332
EBNA3	GFI1B	0.12401
EBNA3	CCBE1	0.16145
EBNA3	PLA2G5	0.037359
EBNA3	BLMH	0.25374
EBNA3	IRAK1	0.074909
EBNA3	ERCC3	0.028985
EBNA3	CXCR4	0.10286
EBNA3	USP37	0.17554
EBNA3	ARFIP2	0.29161
EBNA3	SAC3D1	0.32599
EBNA3	MMS22L	0.34104
EBNA3	MTMR9	0.36705
EBNA3	SLC1A4	0.043565
EBNA3	COL4A2	0.27145
EBNA3	PKD1L2	0.17512
EBNA3	CHMP1A	0.17985
EBNA3	PPM1G	0.18861
EBNA3	GPRC5A	0.010628
EBNA3	GML	0.12344
EBNA3	MAGEB17	0.016295
EBNA3	XPO1	0.16797
EBNA3	LBR	0.27967
EBNA3	ATP8B1	0.24594
EBNA3	TJP2	0.30693
EBNA3	SLC17A6	0.026325
EBNA3	NR1I2	0.11167
EBNA3	ITM2A	0.41166
EBNA3	RBBP4	0.17034
EBNA3	KIAA0087	0.095589
EBNA3	YWHAQ	0.36228
EBNA3	CD68	0.097399
EBNA3	HOOK1	0.23066
EBNA3	GTF2H2	0.032579
EBNA3	INPP5K	0.05651
EBNA3	C11orf72	0.048437
EBNA3	PPP1R3C	0.26967
EBNA3	C9orf171	0.1038
EBNA3	ATP10A	0.19701
EBNA3	TSACC	0.1399
EBNA3	IQCF3	0.023659
EBNA3	LINC00615	0.080052
EBNA3	KLK5	0.38186
EBNA3	MARCH5	0.15538
EBNA3	PPP1R15A	0.34116
EBNA3	STT3A	0.34188
EBNA3	ZBTB24	0.25167
EBNA3	TCF12	0.11799
EBNA3	SPC24	0.27505
EBNA3	NCF2	0.10472
EBNA3	ESD	0.014504
EBNA3	CCDC94	0.10401
EBNA3	FGFR2	0.014339
EBNA3	CCL3	0.058272
EBNA3	MYL6B	0.57538
EBNA3	PCK2	0.24214
EBNA3	CNIH2	0.22938
EBNA3	GLI1	0.082453

EBNA3	GIPC1	0.15527
EBNA3	RAD51C	0.2219
EBNA3	NAA38	0.33872
EBNA3	IGF2BP2	0.28767
EBNA3	YWHAB	0.13932
EBNA3	SLC25A5	0.24754
EBNA3	GNA13	0.13783
EBNA3	BLOC1S5	0.32916
EBNA3	TRIM21	0.18112
EBNA3	GNAS	0.029726
EBNA3	SPTSSB	0.28596
EBNA3	RBBP8NL	0.030401
EBNA3	TAF15	0.32821
EBNA3	RGAG4	0.071197
EBNA3	RBX1	0.13932
EBNA3	TNNI3K	0.18764
EBNA3	WDR82	0.32294
EBNA3	RAB3A	0.11459
EBNA3	NSL1	0.24944
EBNA3	BCL3	0.033056
EBNA3	HMGB2	0.051133
EBNA3	RHOH	0.26819
EBNA3	PI4KAP2	0.051146
EBNA3	KCNQ1	0.036271
EBNA3	TCHP	0.31696
EBNA3	GPX7	0.024815
EBNA3	ATP6V0A1	0.28053
EBNA3	BRPF3	0.28712
EBNA3	APOL3	0.054558
EBNA3	GTF2I	0.16259
EBNA3	CPSF3	0.22805
EBNA3	ATP5C1	0.034383
EBNA3	KCNMA1	0.15882
EBNA3	ORC6	0.2879
EBNA3	TGM3	0.076834
EBNA3	CELSR1	0.07883
EBNA3	UBQLN2	0.35718
EBNA3	PLRG1	0.31803
EBNA3	CUL1	0.19904
EBNA3	SCG5	0.30327
EBNA3	CD9	0.22978
EBNA3	DHX58	0.18001
EBNA3	MYO9A	0.22725
EBNA3	SP3	0.10986
EBNA3	POLI	0.21824
EBNA3	SLC24A1	0.33004
EBNA3	DR1	0.20477
EBNA3	TTC23L	0.026138
EBNA3	TBK1	0.10783
EBNA3	HSPA9	0.23164
EBNA3	NCSTN	0.15501
EBNA3	SLC2A1	0.026538
EBNA3	PLSCR2	0.18082
EBNA3	GSTP1	0.13704
EBNA3	MMP14	0.12691
EBNA3	AFF2	0.0511
EBNA3	ELP3	0.06536
EBNA3	PIGK	0.1871
EBNA3	CDC5L	0.29222
EBNA3	MBNL1	0.24896
EBNA3	MED4	0.24618
EBNA3	RHBDD1	0.22071
EBNA3	SPEF2	0.045619
EBNA3	TIE1	0.23839
EBNA3	FABP5	0.34796
EBNA3	HRC	0.17608
EBNA3	AP1G1	0.24073
EBNA3	HMCN2	0.22883

EBNA3	CDHR2	0.59975
EBNA3	FAS	0.05376
EBNA3	APOE	0.020187
EBNA3	TUBB2B	0.12875
EBNA3	WAS	0.24123
EBNA3	ALDOC	0.18015
EBNA3	TFG	0.34509
EBNA3	SNRPB2	0.29309
EBNA3	TNNI3	0.13958
EBNA3	ILF3	0.23455
EBNA3	WDR61	0.24005
EBNA3	SULT2B1	0.24761
EBNA3	PAWR	0.20213
EBNA3	CDR2L	0.031383
EBNA3	ASIC2	0.3057
EBNA3	CBX3	0.18047
EBNA3	RINT1	0.3248
EBNA3	SHANK3	0.14071
EBNA3	IKBIP	0.3489
EBNA3	RCVRN	0.21226
EBNA3	AGFG1	0.28908
EBNA3	INTS5	0.34602
EBNA3	SCG3	0.034458
EBNA3	C17orf62	0.41166
EBNA3	SNX17	0.28029
EBNA3	CDH5	0.42676
EBNA3	DDX58	0.09653
EBNA3	ARID1B	0.22374
EBNA3	GR6	0.030906
EBNA3	IL6R	0.14462
EBNA3	HS3ST5	0.279
EBNA3	TP53INP2	0.32908
EBNA3	ZNF165	0.1435
EBNA3	SEMA6A	0.24715
EBNA3	ZMYND10	0.35795
EBNA3	COTL1	0.33936
EBNA3	SMN1	0.33496
EBNA3	SNIP1	0.27579
EBNA3	SYT2	0.48567
EBNA3	FSIP2	0.18917
EBNA3	CCNY	0.17135
EBNA3	CAPNS1	0.4964
EBNA3	GTF2E2	0.25395
EBNA3	MMP12	0.016262
EBNA3	KCNG3	0.27608
EBNA3	TRIM41	0.2654
EBNA3	RPL26	0.037287
EBNA3	MPZL3	0.37454
EBNA3	GABARAPL1	0.372
EBNA3	GTF2B	0.18102
EBNA3	ANGPTL4	0.22481
EBNA3	WDR16	0.36195
EBNA3	SLC14A1	0.11817
EBNA3	IFIT1	0.30995
EBNA3	PCDHB15	0.16911
EBNA3	RAD18	0.17592
EBNA3	DNM1	0.11511
EBNA3	OCM2	0.21072
EBNA3	SIPA1	0.21544
EBNA3	TPD52	0.62531
EBNA3	MC5R	0.29057
EBNA3	RIPK4	0.23669
EBNA3	POLDIP3	0.36528
EBNA3	IRS2	0.079973
EBNA3	ARID5B	0.16183
EBNA3	NUFIP1	0.2625
EBNA3	KLC1	0.32903
EBNA3	MRPS31	0.32493

EBNA3	FRMPD4	0.41637
EBNA3	SLC16A8	0.077125
EBNA3	LIG4	0.072443
EBNA3	KLHDC3	0.35795
EBNA3	XRCC5	0.08183
EBNA3	CDC42BPA	0.06024
EBNA4	ubl4ab	0.24991
EBNA4	MED1	0.0203
EBNA4	PIK3CD	0.019147
EBNA4	GAGE12I	0.1692
EBNA4	PAGE2B	0.36527
EBNA4	PRPF6	0.011001
EBNA4	SPTA1	0.083713
EBNA4	S100A7	0.12936
EBNA4	NANOS1	0.12256
EBNA4	HSPA5	0.070018
EBNA4	LDLR	0.055267
EBNA4	SERTAD3	0.28306
EBNA4	INO80B	0.24368
EBNA4	CENPW	0.11095
EBNA4	UPF3A	0.20071
EBNA4	RABEP1	0.27094
EBNA4	SKA3	0.099125
EBNA4	SLC5A1	0.084159
EBNA4	C12orf61	0.23546
EBNA4	FAM86B2	0.040562
EBNA4	PNMA6D	0.067743
EBNA4	PLSCR3	0.17983
EBNA4	DYM	0.23025
EBNA4	ZC3HAV1L	0.22877
EBNA4	ATP6V0D2	0.06759
EBNA4	BTBD16	0.054368
EBNA4	C9orf40	0.042715
EBNA4	RPUSD4	0.1588
EBNA4	CCDC159	0.19348
EBNA4	C10orf68	0.18676
EBNA4	CCDC42	0.03077
EBNA4	NIP7	0.07814
EBNA4	CCDC162P	0.050498
EBNA4	GOLGA6L1	0.097606
EBNA4	MYH7	0.021052
EBNA4	SNAPIN	0.023688
EBNA4	KRI1	0.28812
EBNA4	SPATA1	0.059877
EBNA4	CCDC69	0.13298
EBNA4	MKS1	0.15553
EBNA4	PXMP2	0.12972
EBNA4	EIF3J	0.13544
EBNA4	ANP32D	0.012772
EBNA4	C10orf62	0.23081
EBNA4	UNC119B	0.15775
EBNA4	YMR013W-A	0.21004
EBNA4	LEKR1	0.016908
EBNA4	CASC3	0.2783
EBNA4	C17orf64	0.017352
EBNA4	CYP4F30P	0.034247
EBNA4	ATP5B	0.088312
EBNA4	NIN	0.29423
EBNA4	KCNIP1	0.14844
EBNA4	C22orf39	0.013044
EBNA4	STIM2	0.17622
EBNA4	HUS1B	0.16928
EBNA4	WBP2	0.24521
EBNA4	C1orf222	0.029336
EBNA4	SLAIN2	0.11119
EBNA4	SSSCA1	0.17726
EBNA4	RPLP1	0.13281
EBNA4	PSMG3	0.027767

EBNA4	TMED10	0.011798
EBNA4	THOC5	0.067244
EBNA4	ANGEL1	0.085796
EBNA4	C19orf44	0.094404
EBNA4	C15orf40	0.022478
EBNA4	FSIP1	0.27675
EBNA4	COMMD5	0.24091
EBNA4	WIPF1	0.11705
EBNA4	SPTAN1	0.11372
EBNA4	TSGA10IP	0.20977
EBNA4	CCDC68	0.011725
EBNA4	TNFAIP8L3	0.064542
EBNA4	ATRIP	0.16134
EBNA4	LAMTOR1	0.091606
EBNA4	DHPS	0.13691
EBNA4	IL12A	0.11222
EBNA4	POP4	0.24533
EBNA4	MASP1	0.23196
EBNA4	SCUBE3	0.22227
EBNA4	BCL9	0.13567
EBNA4	UXT	0.10413
EBNA4	TRAPPC4	0.073089
EBNA4	TH	0.011117
EBNA4	WBSCR27	0.014779
EBNA4	FBXO7	0.15517
EBNA4	SPBC1539.02	0.13836
EBNA4	HEXIM1	0.25451
EBNA4	ATXN10	0.17034
EBNA4	C2orf43	0.052909
EBNA4	COMMD7	0.22271
EBNA4	ATG4B	0.17374
EBNA4	MAL2	0.14585
EBNA4	GLTSCR1L	0.34885
EBNA4	TSR2	0.21615
EBNA4	MAB21L3	0.050262
EBNA4	PEX19	0.12789
EBNA4	NAGK	0.030395
EBNA4	PRR15L	0.031069
EBNA4	TECPR1	0.22529
EBNA4	SYT14L	0.016709
EBNA4	MALL	0.12605
EBNA4	CHMP4B	0.18182
EBNA4	PROC	0.19619
EBNA4	PDCD6	0.2254
EBNA4	DKC1	0.11218
EBNA4	S100A9	0.057379
EBNA4	ANXA2	0.10763
EBNA4	TMPO	0.13553
EBNA4	PSMD2	0.049918
EBNA4	MMP20	0.076625
EBNA4	smf	0.067553
EBNA4	MAGEB1	0.030868
EBNA4	CDKN1A	0.016214
EBNA4	MATN4	0.23007
EBNA4	POLG	0.12533
EBNA4	PSMF1	0.13333
EBNA4	NOTCH3	0.059106
EBNA4	RNH1	0.19114
EBNA4	UBAP2	0.29103
EBNA4	C17orf105	0.13641
EBNA4	RRP7B	0.030318
EBNA4	FAM110D	0.26537
EBNA4	PAIP1	0.048129
EBNA4	SELPLG	0.32988
EBNA4	CLIP1	0.08828
EBNA4	YTHDF3	0.20935
EBNA4	PLXDC2	0.11809
EBNA4	BLOC1S3	0.22184

EBNA4	AMOT	0.064244
EBNA4	CCDC154	0.067531
EBNA4	MAATS1	0.13443
EBNA4	SLC4A1	0.053871
EBNA4	PAGE5	0.19286
EBNA4	C20orf196	0.032442
EBNA4	BORA	0.19221
EBNA4	URM1	0.16928
EBNA4	CABS1	0.40924
EBNA4	GAGE10	0.247
EBNA4	CASP8AP2	0.17797
EBNA4	CRTC1	0.12685
EBNA4	BOP	0.028999
EBNA4	CLPX	0.18648
EBNA4	FAM117B	0.36527
EBNA4	EIF4G2	0.06607
EBNA4	ORAI1	0.10952
EBNA4	PHYHIP	0.048998
EBNA4	METTL1	0.24048
EBNA4	MRFAP1	0.32709
EBNA4	MMS19	0.086767
EBNA4	SETD4	0.014089
EBNA4	RPL11	0.10248
EBNA4	NCBP1	0.032966
EBNA4	EDC3	0.10842
EBNA4	GAGE3	0.087217
EBNA4	MESDC1	0.065495
EBNA4	CCDC30	0.011991
EBNA4	C10orf91	0.33042
EBNA4	CCDC28A	0.35232
EBNA4	TTC22	0.037792
EBNA4	CYBA	0.067664
EBNA4	SHANK2	0.011813
EBNA4	SLA2	0.013976
EBNA4	FAM120C	0.072572
EBNA4	UPF2	0.034988
EBNA4	CCDC65	0.049041
EBNA4	GID4	0.11594
EBNA4	FAM83F	0.22188
EBNA4	FAM101A	0.15792
EBNA4	CKB	0.12206
EBNA4	MAGEB10	0.016831
EBNA4	SNURFL	0.15937
EBNA4	THBD	0.1339
EBNA4	KIAA0040	0.096892
EBNA4	RTP4	0.13334
EBNA4	MTERF	0.10603
EBNA4	ATG13	0.31473
EBNA4	BRAF	0.016524
EBNA4	ENTHD1	0.026574
EBNA4	MT2A	0.12498
EBNA4	CLSPN	0.16577
EBNA4	FAM178A	0.2218
EBNA4	MEN1	0.060757
EBNA4	BCKDHB	0.083343
EBNA4	RNMT	0.070404
EBNA4	C12orf36	0.094033
EBNA4	CCDC27	0.011488
EBNA4	MAGOH	0.035108
EBNA4	SRGN	0.22157
EBNA4	EML1	0.21633
EBNA4	OTUD6B	0.045198
EBNA4	NOL3	0.23384
EBNA4	FAM83C	0.15719
EBNA4	IQCK	0.23741
EBNA4	ITFG2	0.041427
EBNA4	HAT1	0.16952
EBNA4	FAM166A	0.26097

EBNA4	NGFRAP1	0.16817
EBNA4	CNTD1	0.016148
EBNA4	PRRC2B	0.31224
EBNA4	MUM1L1	0.21859
EBNA4	SPPL2A	0.098027
EBNA4	FAM74A4	0.085839
EBNA4	EPS15	0.090605
EBNA4	MYO5B	0.01456
EBNA4	HUS1	0.064275
EBNA4	PCSK2	0.069112
EBNA4	PRMT8	0.033105
EBNA4	PEX11G	0.25747
EBNA4	DISC1	0.041423
EBNA4	AP3S1	0.092507
EBNA4	TTC34	0.014673
EBNA4	SDF4	0.16015
EBNA4	BCKDHA	0.025565
EBNA4	NACAP1	0.059959
EBNA4	TAF11	0.15848
EBNA4	LAMTOR2	0.10117
EBNA4	CDC45	0.10382
EBNA4	EID3	0.21157
EBNA4	SPACA3	0.12491
EBNA4	STAT1	0.023647
EBNA4	FAT4	0.20906
EBNA4	DTNBP1	0.0631
EBNA4	SDE2	0.035764
EBNA4	PPEF1	0.12168
EBNA4	CXorf67	0.31187
EBNA4	C18orf25	0.13726
EBNA4	NELL2	0.074133
EBNA4	THEGL	0.029596
EBNA4	CLTB	0.047969
EBNA4	C11orf35	0.05988
EBNA4	C16orf86	0.078549
EBNA4	ULK2	0.01213
EBNA4	ELOVL4	0.1679
EBNA4	FAM188B2	0.37574
EBNA4	GTF3C1	0.14332
EBNA4	DMAP1	0.1296
EBNA4	STX1A	0.01227
EBNA4	FAM78A	0.037043
EBNA4	AKIP1	0.17803
EBNA4	KAT2A	0.019912
EBNA4	SUV420H2	0.022728
EBNA4	MAP1LC3C	0.034929
EBNA4	ATP6V1E1	0.082928
EBNA4	NUDCD3	0.15253
EBNA4	THBS1	0.021837
EBNA4	MAGEF1	0.044385
EBNA4	ARL2BP	0.10619
EBNA4	DEDD2	0.19622
EBNA4	BICD1	0.022455
EBNA4	AP2M1	0.012506
EBNA4	KIAA1407	0.14672
EBNA4	FAM184B	0.10102
EBNA4	TMCC2	0.12337
EBNA4	PRMT1	0.045853
EBNA4	CCSER1	0.26233
EBNA4	UNQ6126/PRO20091	0.010847
EBNA4	CCDC144A	0.021798
EBNA4	PNMAL2	0.20356
EBNA4	DDOST	0.13677
EBNA4	AGR3	0.10689
EBNA4	AP1M1	0.041674
EBNA4	AGR2	0.064848
EBNA4	YPR022C	0.27617
EBNA4	ADM	0.040345

EBNA4	TPPP	0.15202
EBNA4	EVPLL	0.01464
EBNA4	DBNDD2	0.15528
EBNA4	FYB	0.19871
EBNA4	IL17RE	0.29386
EBNA4	FBXO36	0.014157
EBNA4	CCDC146	0.15505
EBNA4	SYT11	0.25586
EBNA4	CCDC150	0.11889
EBNA4	MAD1L1	0.17066
EBNA4	C1orf105	0.25134
EBNA4	EIF2B1	0.062067
EBNA4	PARPBP	0.16243
EBNA4	FANCA	0.092432
EBNA4	ATP6V0D1	0.091629
EBNA4	TPD52L1	0.2321
EBNA4	HSP90B1	0.11245
EBNA4	MACF1	0.13779
EBNA4	CCDC66	0.055215
EBNA4	TNFRSF12A	0.11316
EBNA4	CCDC138	0.030224
EBNA4	C9orf170	0.021703
EBNA4	GLMN	0.1698
EBNA4	MSR1	0.018935
EBNA4	CCDC176	0.28363
EBNA4	HEATR2	0.015906
EBNA4	TBC1D25	0.019396
EBNA4	GCH1	0.11391
EBNA4	C10orf67	0.28493
EBNA4	LINC00597	0.20627
EBNA4	FAM131C	0.058475
EBNA4	ZBTB8OS	0.044916
EBNA4	PRDM11	0.019044
EBNA4	NKD2	0.2098
EBNA4	MT1HL1	0.17367
EBNA4	C2orf88	0.057648
EBNA4	IFT57	0.067563
EBNA4	AP5B1	0.16412
EBNA4	EIF2B5	0.011454
EBNA4	TMEM132D	0.24117
EBNA4	NCOR1P1	0.028194
EBNA4	ATP2B2	0.020444
EBNA4	INCENP	0.1115
EBNA4	C19orf71	0.1762
EBNA4	JMJD1C	0.044117
EBNA4	FNBP1L	0.039323
EBNA4	SNX29P2	0.092094
EBNA4	RHPN1-AS1	0.2397
EBNA4	CDKN2AIP	0.13249
EBNA4	C6orf118	0.1471
EBNA4	IL17REL	0.069222
EBNA4	C9orf172	0.017155
EBNA4	CCDC74B	0.15466
EBNA4	FAT1	0.11467
EBNA4	NXF3	0.13294
EBNA4	EDAR	0.10216
EBNA4	TGOLN2	0.23291
EBNA4	LINC00471	0.19895
EBNA4	HABP4	0.16524
EBNA4	S100A11	0.1737
EBNA4	GPS2	0.26262
EBNA4	HFE2	0.16526
EBNA4	FAM83E	0.012906
EBNA4	CAB8	0.030203
EBNA4	MSANTD2	0.035045
EBNA4	CCDC137	0.28343
EBNA4	TIFAB	0.31814
EBNA4	NCAPG	0.14742

EBNA4	CLHC1	0.012553
EBNA4	C15orf54	0.13264
EBNA4	C16orf95	0.043732
EBNA4	NUTM2A	0.099669
EBNA4	NKD1	0.16429
EBNA4	PLAC8L1	0.094765
EBNA4	DERL2	0.10571
EBNA4	GCK	0.011176
EBNA4	CROCCP3	0.067879
EBNA4	GORAB	0.31683
EBNA4	FAM151B	0.11677
EBNA4	GCSAML	0.20615
EBNA4	EIF1AY	0.041158
EBNA4	FNIP2	0.40281
EBNA4	EPSTI1	0.019273
EBNA4	FAM47B	0.20804
EBNA4	MBD3L5	0.035301
EBNA4	BEND4	0.036988
EBNA4	SDHAF2	0.12789
EBNA4	SUPT20HL2	0.19624
EBNA4	PPP2R3B	0.11553
EBNA4	CTNNBIP1	0.12061
EBNA4	SPRYD7	0.067208
EBNA4	FBXO46	0.28651
EBNA4	EHD1	0.075769
EBNA4	GTF2E1	0.1946
EBNA4	OXA1L	0.13452
EBNA4	INTS10	0.20805
EBNA4	CDR2	0.1875
EBNA4	LINC00523	0.3346
EBNA4	RABAC1	0.056415
EBNA4	RABGAP1L	0.053888
EBNA4	FAM83G	0.020832
EBNA4	GEMIN6	0.085108
EBNA4	CSN2	0.33722
EBNA4	EIF2B2	0.037439
EBNA4	EXOSC1	0.11578
EBNA4	S100A13	0.16668
EBNA4	KIAA2026	0.01525
EBNA4	MELK	0.066484
EBNA4	C9orf152	0.076381
EBNA4	S100A1	0.12635
EBNA4	ARL14EPL	0.34942
EBNA4	SNRPC	0.083615
EBNA4	UTRN	0.090947
EBNA4	GAGE12H	0.1692
EBNA4	INTS4L2	0.053972
EBNA4	PALM	0.11482
EBNA4	SLC34A1	0.23643
EBNA4	TOP2A	0.012179
EBNA4	NOP56	0.074864
EBNA4	FAM219B	0.048412
EBNA4	CXorf21	0.13976
EBNA4	AQP1	0.014061
EBNA4	ELN	0.28557
EBNA4	LTF	0.037051
EBNA4	FAM127A	0.12992
EBNA4	EMD	0.11988
EBNA4	C17orf66	0.076697
EBNA4	CNBD1	0.022892
EBNA4	STAT6	0.030484
EBNA4	HYAL2	0.018748
EBNA4	PIH1D2	0.23483
EBNA4	C16orf11	0.034629
EBNA4	POM121L12	0.1669
EBNA4	AKAP5	0.078689
EBNA4	CALB1	0.15546
EBNA4	DAZAP2	0.19318

EBNA4	CPSF2	0.053838
EBNA4	FAM86B1	0.014215
EBNA4	NDEL1	0.063009
EBNA4	MASP2	0.21778
EBNA4	NUDT21	0.06337
EBNA4	PARK7	0.053536
EBNA4	ATG9A	0.027876
EBNA4	LINC00472	0.22525
EBNA4	C20orf96	0.064044
EBNA4	GOLGA6L4	0.27013
EBNA4	RAMP2	0.071075
EBNA4	GOLGA6L6	0.25437
EBNA4	KIAA1958	0.12766
EBNA4	SLAIN1	0.33455
EBNA4	C9orf169	0.085441
EBNA4	EIF3C	0.070291
EBNA4	YPL025C	0.11417
EBNA4	ZNF326	0.16644
EBNA4	KIAA0226L	0.05946
EBNA4	MYOZ3	0.17778
EBNA4	NOM1	0.077073
EBNA4	FAM13C	0.011228
EBNA4	APEX1	0.020247
EBNA4	GAS6	0.015151
EBNA4	ANXA2R	0.29148
EBNA4	CC2D2B	0.11912
EBNA4	YML009W-B	0.33296
EBNA4	C14orf178	0.13386
EBNA4	MED9	0.22542
EBNA4	EFEMP1	0.19314
EBNA4	EIF4A3	0.01755
EBNA4	DAXX	0.11614
EBNA4	BID	0.18491
EBNA4	COMMD2	0.23223
EBNA4	C1orf168	0.17849
EBNA4	NIPSNAP3A	0.16164
EBNA4	YTHDF1	0.13804
EBNA4	SPARC	0.093929
EBNA4	FAM179A	0.018725
EBNA4	MRFAP1L1	0.31715
EBNA4	EHMT2	0.018292
EBNA4	NOTCH4	0.052088
EBNA4	TERT	0.13721
EBNA4	MEAF6	0.10825
EBNA4	KLHL12	0.011361
EBNA4	CNPY1	0.041673
EBNA4	FAM160A1	0.31181
EBNA4	PCDH17	0.20906
EBNA4	PIR	0.19303
EBNA4	WHAMMP3	0.036762
EBNA4	PRDX4	0.029445
EBNA4	CCDC85B	0.114
EBNA4	EIF3B	0.026094
EBNA4	ALB	0.078029
EBNA4	YJR030C	0.19445
EBNA4	ODF3L2	0.013076
EBNA4	KIAA1841	0.17511
EBNA4	EAF2	0.12599
EBNA4	LCA5L	0.016661
EBNA4	C1orf196	0.11553
EBNA4	RNF216P1	0.19148
EBNA4	RASSF4	0.14072
EBNA4	REPS1	0.19777
EBNA4	DCUN1D2	0.0358
EBNA4	LINC00476	0.048319
EBNA4	STPG2	0.0382
EBNA4	YJEFN3	0.11946
EBNA4	C18orf21	0.13679

EBNA4	XAGE2	0.32465
EBNA4	YML009W-B	0.1337
EBNA4	ELOVL3	0.1197
EBNA4	LRCOL1	0.26163
EBNA4	SAT1	0.071639
EBNA4	GOLGA6L18	0.23368
EBNA4	YTHDC1	0.20935
EBNA4	C16orf71	0.01347
EBNA4	GEMIN7	0.16873
EBNA4	TBC1D7	0.049337
EBNA4	TXNIP	0.1681
EBNA4	BCORP1	0.13849
EBNA4	BRAT1	0.17191
EBNA4	FAM160B2	0.096076
EBNA4	FAM211A	0.016488
EBNA4	GTF3C6	0.17962
EBNA4	WIBG	0.26092
EBNA4	FAM221B	0.22415
EBNA4	NOTCH2	0.024796
EBNA4	MPTX1	0.079464
EBNA4	AMER2	0.27133
EBNA4	TAF1D	0.12432
EBNA4	STIM1	0.10123
EBNA4	S100B	0.099953
EBNA4	TRAF3IP1	0.29174
EBNA4	CCDC172	0.043533
EBNA4	LDOC1L	0.096025
EBNA4	AC3.5	0.20167
EBNA4	SKP1	0.056393
EBNA4	VAMP1	0.011082
EBNA4	LINS	0.072393
EBNA4	TNNC1	0.18843
EBNA4	SAP30	0.2113
EBNA4	TXLNA	0.095438
EBNA4	POLRMT	0.15401
EBNA4	TMED2	0.026173
EBNA4	C14orf80	0.26053
EBNA4	NBPF24	0.099755
EBNA4	C1orf94	0.4389
EBNA4	CETN2	0.15635
EBNA4	ANXA6	0.19876
EBNA4	C9orf153	0.084437
EBNA4	TINF2	0.12793
EBNA4	FEZ1	0.068493
EBNA4	ACTN2	0.070761
EBNA4	PRSS2	0.1902
EBNA4	STAT2	0.031331
EBNA4	PSRC1	0.16597
EBNA4	NCKAP1	0.012015
EBNA4	CT45A5	0.11011
EBNA4	EIF4A2	0.015724
EBNA4	PNRC2	0.26732
EBNA4	C2orf78	0.14576
EBNA4	C21orf58	0.12609
EBNA4	CDCA5	0.13929
EBNA4	HAX1	0.14716
EBNA4	PITPNM3	0.18902
EBNA4	VIL1	0.094261
EBNA4	C19orf35	0.2188
EBNA4	C4orf17	0.048482
EBNA4	C9orf117	0.017451
EBNA4	GAGE6	0.087217
EBNA4	SMNDC1	0.038095
EBNA4	CNOT1	0.088389
EBNA4	YIR021W-A	0.073703
EBNA4	COMMD9	0.15644
EBNA4	KCNJ4	0.065532
EBNA4	ERLIN1	0.28825

EBNA4	ANGEL2	0.056986
EBNA4	AHSA1	0.19332
EBNA4	MIR7-3HG	0.057795
EBNA4	NBP35	0.010892
EBNA4	PP632	0.063351
EBNA4	C17orf104	0.14453
EBNA4	CETN1	0.19958
EBNA4	PRO1854	0.1124
EBNA4	KCNJ9	0.025629
EBNA4	GAGE4	0.1731
EBNA4	ATP5SL	0.042354
EBNA4	ZSWIM7	0.23418
EBNA4	C2CD4D	0.074564
EBNA4	ZK1128.3	0.10797
EBNA4	AXDND1	0.013376
EBNA4	YDL022C-A	0.050614
EBNA4	PP632	0.087795
EBNA4	NUP98	0.062115
EBNA4	IGFBP2	0.020221
EBNA4	PNMAL1	0.14129
EBNA4	C18orf42	0.069221
EBNA4	VLDLR	0.081027
EBNA4	NUP62	0.045988
EBNA4	CDH1	0.040029
EBNA4	HTT	0.017402
EBNA4	GAGE13	0.087217
EBNA4	ADAMTS13	0.025606
EBNA4	MAP1LC3B	0.05996
EBNA4	TTN	0.03684
EBNA4	ZDHHC17	0.027003
EBNA4	ZYG11A	0.031905
EBNA4	CXorf30	0.20611
EBNA4	EGF	0.068634
EBNA4	KIAA1755	0.010821
EBNA4	LTBP4	0.16179
EBNA4	CCDC132	0.084647
EBNA4	MOAP1	0.087411
EBNA4	PRMT2	0.028706
EBNA4	RPS19	0.13528
EBNA4	NPHP4	0.30339
EBNA4	PLSCR4	0.17581
EBNA4	INSIG1	0.092026
EBNA4	DNAJC9-AS1	0.25575
EBNA4	CENPF	0.044002
EBNA4	UBQLNL	0.029139
EBNA4	RBM8A	0.032295
EBNA4	CCDC152	0.035308
EBNA4	KHDC1L	0.039952
EBNA4	PRAMEF26	0.019315
EBNA4	FAM45A	0.28647
EBNA4	KIAA1751	0.074249
EBNA4	FBXO25	0.26408
EBNA4	TCP10L2	0.15577
EBNA4	CCDC57	0.026338
EBNA4	GOLGB1	0.14793
EBNA4	SSUH2	0.025115
EBNA4	HBA1	0.15392
EBNA4	NXF1	0.016208
EBNA4	C1orf106	0.3348
EBNA4	GOLGA6L3	0.27013
EBNA4	RHOT1	0.028627
EBNA4	WWC2-AS2	0.16742
EBNA4	SAP18	0.12045
EBNA4	GRAMD3	0.016776
EBNA4	SNX9	0.10129
EBNA4	CITED2	0.015438
EBNA4	VPS53	0.095466
EBNA4	TTI1	0.19005

EBNA4	UBXN4	0.17938
EBNA4	FBXL18	0.083684
EBNA4	GUCD1	0.093971
EBNA4	FAM195B	0.098413
EBNA4	CCDC7	0.13057
EBNA4	YFL013W-A	0.17686
EBNA4	RYR2	0.042783
EBNA4	PRF1	0.096995
EBNA4	IL6ST	0.089726
EBNA4	MYO1E	0.056681
EBNA4	GAGE12F	0.1692
EBNA4	ANKRD26P1	0.023277
EBNA4	MZT1	0.1686
EBNA4	PCSK9	0.020375
EBNA4	NELFA	0.27285
EBNA4	BLACE	0.011718
EBNA4	GPRIN3	0.11589
EBNA4	FAM92B	0.052167
EBNA4	SETD2	0.032539
EBNA4	C4orf22	0.063515
EBNA4	C6orf52	0.028737
EBNA4	ARRDC3	0.11498
EBNA4	CCDC74A	0.15466
EBNA4	SERBP1	0.17402
EBNA4	ANP32C	0.019584
EBNA4	CDRT4	0.059418
EBNA4	PRR19	0.17204
EBNA4	CCDC67	0.036798
EBNA4	SNCA	0.011457
EBNA4	S100A8	0.080554
EBNA4	THAP11	0.15389
EBNA4	NID2	0.22143
EBNA4	GAREML	0.34945
EBNA4	MIMI	0.24474
EBNA4	NELL1	0.12165
EBNA4	TICRR	0.24295
EBNA4	SLX1A	0.12759
EBNA4	ytzL	0.40601
EBNA4	WDR96	0.15747
EBNA4	CCDC158	0.010535
EBNA4	ADA	0.022778
EBNA4	DNER	0.13448
EBNA4	COL17A1	0.27758
EBNA4	EIF6	0.083862
EBNA4	L1TD1	0.040559
EBNA4	C15orf65	0.084707
EBNA4	FAM160B1	0.22154
EBNA4	ELOVL6	0.1197
EBNA4	CCDC121	0.01658
EBNA4	SAMD14	0.02951
EBNA4	PEF1	0.22299
EBNA4	OFD1	0.14346
EBNA4	ZWINT	0.088813
EBNA4	CT45A4	0.087607
EBNA4	RBBP8	0.11102
EBNA4	FBXL8	0.44209
EBNA4	GCHFR	0.081955
EBNA4	FBL	0.026159
EBNA4	TTYH1	0.35401
EBNA4	NUPR1L	0.14524
EBNA4	MEGF8	0.074716
EBNA4	C10orf82	0.16114
EBNA4	ERICH1-AS1	0.24625
EBNA4	CHCHD6	0.26752
EBNA4	C3orf65	0.18444
EBNA4	ATXN1	0.1169
EBNA4	GAP43	0.042847
EBNA4	CDPF1	0.013307

EBNA4	CHP1	0.083864
EBNA4	CBY3	0.13992
EBNA4	C1orf228	0.084731
EBNA4	CFTR	0.026108
EBNA4	DYDC2	0.070053
EBNA4	CNST	0.021858
EBNA4	EP400NL	0.22887
EBNA4	FAM203B	0.18521
EBNA4	TGM2	0.068006
EBNA4	RPLP0	0.052893
EBNA4	FASN	0.072544
EBNA4	HEATR5A	0.12707
EBNA4	LCMT1	0.067255
EBNA4	EID2	0.17408
EBNA4	ELOVL7	0.1197
EBNA4	GPR98	0.087514
EBNA4	OTUD6A	0.082132
EBNA4	MICU1	0.17965
EBNA4	CALU	0.17729
EBNA4	EIF2S1	0.037687
EBNA4	CEP63	0.034598
EBNA4	PPP3CA	0.045597
EBNA4	MTUS2	0.11707
EBNA4	VWA9	0.066895
EBNA4	MYOZ2	0.29756
EBNA4	C16orf72	0.27538
EBNA4	GADD45G	0.15492
EBNA4	BEND2	0.094243
EBNA4	EIF4B	0.054777
EBNA4	LRP2	0.080507
EBNA4	VMP1	0.15095
EBNA4	CAPN2	0.045384
EBNA4	SLC23A1	0.040992
EBNA4	UNC119	0.16245
EBNA4	JMJD7	0.020682
EBNA4	POM121L2	0.16388
EBNA4	GOLGA6L10	0.23368
EBNA4	SAMD13	0.029108
EBNA4	FAM188B	0.32663
EBNA4	MISP	0.28963
EBNA4	FAM231B	0.20956
EBNA4	SELO	0.25015
EBNA4	MON1B	0.17234
EBNA4	ZNF728	0.030849
EBNA4	FAM27D1	0.21502
EBNA4	NUCB2	0.14776
EBNA4	TF	0.026389
EBNA4	UHRF1BP1L	0.21572
EBNA4	SHANK2-AS3	0.086981
EBNA4	FAM194B	0.013709
EBNA4	UNQ6126/PRO20091	0.1139
EBNA4	NPC2	0.16651
EBNA4	HIN1L	0.022569
EBNA4	ATXN7L2	0.085504
EBNA4	FBXW12	0.010778
EBNA4	PSMD5	0.1075
EBNA4	PEX11A	0.24315
EBNA4	YGL014C-A	0.26434
EBNA4	S100A6	0.14015
EBNA4	VHL	0.10293
EBNA4	TERF2IP	0.14701
EBNA4	LRP8	0.14865
EBNA4	CCDC151	0.045157
EBNA4	SYNPO	0.19827
EBNA4	MBD3L2	0.020113
EBNA4	IGFLR1	0.2315
EBNA4	CT55	0.16685
EBNA4	PPP6R3	0.15759

EBNA4	S100P	0.1518
EBNA4	C11orf80	0.010959
EBNA4	SERTAD1	0.25811
EBNA4	RWDD4	0.089687
EBNA4	DYNLRB1	0.15016
EBNA4	EGLN2	0.074359
EBNA4	PRDX6	0.046542
EBNA4	CHAF1A	0.33805
EBNA4	PTCRA	0.12558
EBNA4	SETD1B	0.03637
EBNA4	PTGES3L	0.10048
EBNA4	TP53INP1	0.19995
EBNA4	RUNDC3B	0.050926
EBNA4	ATXN7L3	0.13718
EBNA4	MNCb-2875	0.09007
EBNA4	FAM127C	0.13391
EBNA4	MCM10	0.13493
EBNA4	PTTG1	0.063241
EBNA4	CLUAP1	0.31649
EBNA4	NUP160	0.08907
EBNA4	NUP50	0.072915
EBNA4	RAB11FIP3	0.073984
EBNA4	EFCAB4B	0.16095
EBNA4	ALKBH8	0.032121
EBNA4	SYBU	0.088697
EBNA4	C8orf74	0.14092
EBNA4	CCDC104	0.086918
EBNA4	ARL6IP1	0.061302
EBNA4	XAGE3	0.36527
EBNA4	TEX36	0.045596
EBNA4	C11orf65	0.069847
EBNA4	ASAH2B	0.02575
EBNA4	CCDC85A	0.018267
EBNA4	C1orf111	0.18622
EBNA4	TCL1B	0.022884
EBNA4	THOC1	0.018239
EBNA4	CCDC87	0.07302
EBNA4	CHGB	0.4272
EBNA4	JMJD8	0.019211
EBNA4	DTNA	0.11749
EBNA4	REPS2	0.23352
EBNA4	PPEF2	0.10301
EBNA4	EFEMP2	0.2199
EBNA4	SART1	0.17102
EBNA4	MAGED4	0.027817
EBNA4	EIF3L	0.060503
EBNA4	ATP2A1	0.064519
EBNA4	ATXN7L3B	0.12074
EBNA4	SCAMP5	0.098151
EBNA4	WLS	0.10118
EBNA4	OCEL1	0.02671
EBNA4	EIF3D	0.041867
EBNA4	CT45A3	0.097421
EBNA4	MAGEB3	0.016169
EBNA4	CD47	0.079554
EBNA4	CCP110	0.15231
EBNA4	CAGE1	0.047247
EBNA4	LINC00205	0.18078
EBNA4	PKD2L1	0.11623
EBNA4	FAM46B	0.16156
EBNA4	FEZ2	0.1383
EBNA4	PNAS-138	0.2117
EBNA4	CD2BP2	0.11251
EBNA4	ACOT8	0.13322
EBNA4	NCOA6	0.19897
EBNA4	HTN1	0.25222
EBNA4	CCDC26	0.19156
EBNA4	EHD3	0.094399

EBNA4	TGM1	0.055483
EBNA4	FAM181A	0.088806
EBNA4	SUPT20HL1	0.19624
EBNA4	SLC14A2	0.18122
EBNA4	C7orf57	0.080476
EBNA4	LY96	0.14308
EBNA4	C22orf43	0.1382
EBNA4	RPS2	0.052063
EBNA4	FAM166B	0.05565
EBNA4	VWA5B2	0.14544
EBNA4	THBS2	0.18681
EBNA4	NAF1	0.24169
EBNA4	PRAME	0.29073
EBNA4	CALM1	0.095142
EBNA4	EIF4E2	0.046673
EBNA4	SOGA2	0.010757
EBNA4	FLT3	0.014543
EBNA4	KIF1B	0.014471
EBNA4	MAP7D2	0.071395
EBNA4	S100A4	0.21619
EBNA4	NAPA	0.063699
EBNA4	S100A12	0.11434
EBNA4	FAM149B1	0.16763
EBNA4	RCN1	0.22977
EBNA4	KCNJ6	0.024347
EBNA4	ACD	0.13158
EBNA4	DPP4	0.035792
EBNA4	EIF3E	0.05551
EBNA4	EIF4E	0.052278
EBNA4	STAG3L4	0.085509
EBNA4	BBC3	0.21183
EBNA4	DNMT3A	0.022112
EBNA4	OAS3	0.095556
EBNA4	RQCD1	0.17998
EBNA4	NPB	0.24736
EBNA4	ACTN3	0.20548
EBNA4	ATP5F1	0.062121
EBNA4	FBN1	0.20642
EBNA4	MIF4GD	0.17701
EBNA4	SPAC22F8.05	0.02698
EBNA4	RAD1	0.11443
EBNA4	C12orf63	0.079175
EBNA4	F10	0.10787
EBNA4	CAPN1	0.096637
EBNA4	UCKL1	0.099241
EBNA4	GSTZ1	0.013895
EBNA4	IPO7	0.097839
EBNA4	DPM2	0.22673
EBNA4	FAM46A	0.17048
EBNA4	CCNYL3	0.012165
EBNA4	TSN	0.16005
EBNA4	CELSR3	0.13035
EBNA4	RILP	0.21167
EBNA4	LCNL1	0.013799
EBNA4	SS18L2	0.075757
EBNA4	SDHA	0.037402
EBNA4	CTIF	0.070195
EBNA4	PEX14	0.14753
EBNA4	GAGE12J	0.087217
EBNA4	N6AMT1	0.060506
EBNA4	DEHA2C16280g	0.15495
EBNA4	IGFL1	0.30003
EBNA4	IGBP1	0.1553
EBNA4	KHNYN	0.14327
EBNA4	SETD8	0.022732
EBNA4	SIRT4	0.096187
EBNA4	PRNP	0.092278
EBNA4	TESC	0.1279

EBNA4	GAGE2A	0.1692
EBNA4	ATXN7L1	0.085504
EBNA4	IFNLR1	0.08344
EBNA4	MICAL1	0.03728
EBNA4	LSM12	0.18144
EBNA4	UCN	0.083226
EBNA4	FGFR3	0.010903
EBNA4	KIAA0907	0.042228
EBNA4	C12orf57	0.15606
EBNA4	SETD1A	0.034895
EBNA4	FAM228A	0.038382
EBNA4	YAE1D1	0.19011
EBNA4	CT45A1	0.10486
EBNA4	ERICH1	0.19937
EBNA4	C21orf37	0.01445
EBNA4	RPH3A	0.10131
EBNA4	AMOTL2	0.032633
EBNA4	SRI	0.11338
EBNA4	PPP4R2	0.25122
EBNA4	LRP4	0.11646
EBNA4	BVES-AS1	0.12668
EBNA4	TMSB4X	0.24554
EBNA4	CCDC127	0.11821
EBNA4	CCDC120	0.20994
EBNA4	MAGEB16	0.028574
EBNA4	SLIT2	0.041832
EBNA4	C1orf145	0.20429
EBNA4	OTUD1	0.071594
EBNA4	GPRASP1	0.18217
EBNA4	SFI1	0.22398
EBNA4	MATN2	0.18194
EBNA4	SAMD9L	0.0161
EBNA4	CIB1	0.16026
EBNA4	FNTA	0.018604
EBNA4	PPP2R3A	0.14431
EBNA4	AKAP3	0.13762
EBNA4	EIF2B4	0.03849
EBNA4	DLL1	0.15994
EBNA4	PEX11B	0.069847
EBNA4	PMP22	0.056682
EBNA4	CD164	0.21028
EBNA4	BLOC1S1	0.23727
EBNA4	FAM195A	0.34344
EBNA4	RGAG1	0.092052
EBNA4	PNMA6A	0.04132
EBNA4	C9orf62	0.012194
EBNA4	EHD4	0.064656
EBNA4	PNPT1	0.10226
EBNA4	UPF3B	0.27226
EBNA4	MYO6	0.018184
EBNA4	C5orf58	0.23241
EBNA4	LINC00114	0.033533
EBNA4	PMAIP1	0.12339
EBNA4	PRSS3	0.22139
EBNA4	CCDC169	0.10918
EBNA4	C7orf62	0.174
EBNA4	MYL2	0.20879
EBNA4	EDEM1	0.11912
EBNA4	LRP1	0.055097
EBNA4	ATP10B	0.023387
EBNA4	TIMM23	0.10593
EBNA4	MB21D2	0.11293
EBNA4	ARMCX5	0.10958
EBNA4	LSM11	0.21313
EBNA4	ATP5A1	0.059118
EBNA4	ADAR	0.025122
EBNA4	SNX19	0.14756
EBNA4	YMR001C-A	0.017854

EBNA4	MRE11A	0.034864
EBNA4	CLPP	0.16714
EBNA4	PDGFRB	0.025397
EBNA4	SPATA2L	0.15082
EBNA4	DACT1	0.064992
EBNA4	ITSN1	0.051674
EBNA4	C15orf37	0.046281
EBNA4	BBS1	0.126
EBNA4	BTF3L4	0.086289
EBNA4	SPIDR	0.28335
EBNA4	ELOVL1	0.1197
EBNA4	DST	0.1037
EBNA4	HIF1AN	0.019998
EBNA4	MYL6	0.20406
EBNA4	COMP	0.15803
EBNA4	CCDC47	0.18164
EBNA4	XAGE5	0.22969
EBNA4	ANXA7	0.10786
EBNA4	CHP2	0.1693
EBNA4	LYRM9	0.24963
EBNA4	MUC1	0.28996
EBNA4	FAM127B	0.20055
EBNA4	PLN	0.25246
EBNA4	CDK5R1	0.12456
EBNA4	SUB1	0.16749
EBNA4	CXorf40B	0.074476
EBNA4	C16orf96	0.027044
EBNA4	EIF4EBP2	0.12206
EBNA4	NECAB2	0.23724
EBNA4	ARMC5	0.019844
EBNA4	PODXL2	0.14244
EBNA4	LINC00574	0.011851
EBNA4	CANX	0.099695
EBNA4	RHOT2	0.02941
EBNA4	EHMT1	0.010077
EBNA4	SGD1	0.16317
EBNA4	MMP15	0.060192
EBNA4	FHAD1	0.019353
EBNA4	PDE6D	0.069924
EBNA4	FBXO34	0.047947
EBNA4	GADD45A	0.15645
EBNA4	BSDC1	0.037565
EBNA4	ANXA11	0.10828
EBNA4	PPP3CB	0.049555
EBNA4	GOLGA6L19	0.23368
EBNA4	C8orf88	0.12539
EBNA4	C5orf34	0.12286
EBNA4	LURAP1L	0.31207
EBNA4	SHCBP1L	0.037412
EBNA4	C19orf47	0.10717
EBNA4	C4orf33	0.044821
EBNA4	C12orf50	0.28977
EBNA4	C17orf102	0.13555
EBNA4	IQGAP1	0.03867
EBNA4	RMND5B	0.036198
EBNA4	IQCG	0.10944
EBNA4	TIMP1	0.071505
EBNA4	MATN3	0.23829
EBNA4	MAGEC3	0.019442
EBNA4	PNMA6C	0.067743
EBNA4	FAM47C	0.19897
EBNA4	SH3KBP1	0.051646
EBNA4	AMOTL1	0.12483
EBNA4	C1orf173	0.023039
EBNA4	TJAP1	0.28077
EBNA4	DARS	0.1401
EBNA4	SCHIP1	0.28641
EBNA4	RET	0.081365

EBNA4	PAGE3	0.15003
EBNA4	PATL1	0.12305
EBNA4	FAM43B	0.022703
EBNA4	PARP10	0.11521
EBNA4	MAGED2	0.031393
EBNA4	TLX1NB	0.063341
EBNA4	MS4A3	0.10522
EBNA4	CDH23	0.077296
EBNA4	LCA10	0.21916
EBNA4	STATH	0.32002
EBNA4	RAB11FIP4	0.25986
EBNA4	CNGC19	0.33865
EBNA4	C9orf106	0.049171
EBNA4	DYDC1	0.040536
EBNA4	CCDC102B	0.027687
EBNA4	CNRIP1	0.057544
EBNA4	PRPF3	0.13573
EBNA4	ATP6V1H	0.049529
EBNA4	PHYH	0.18926
EBNA4	PLSCR5	0.018472
EBNA4	C5orf24	0.015527
EBNA4	TCERG1L	0.10212
EBNA4	SUV39H2	0.036354
EBNA4	C6orf223	0.34663
EBNA4	C1orf64	0.20239
EBNA4	LDHAL6B	0.077734
EBNA4	MBOAT7	0.14192
EBNA4	ELP4	0.14791
EBNA4	COMMD4	0.17127
EBNA4	PACRGL	0.081455
EBNA4	TCTEX1D1	0.076593
EBNA4	CLU	0.05929
EBNA4	NPIPB11	0.015125
EBNA4	C1orf140	0.24082
EBNA4	ATXN7	0.038276
EBNA4	YGL006W-A	0.051525
EBNA4	GOLGA6L20	0.14208
EBNA4	LINC00083	0.020179
EBNA4	LINC00588	0.25104
EBNA4	HYPK	0.057301
EBNA4	RUNDC1	0.059063
EBNA4	WIPF2	0.1613
EBNA4	PLCE1	0.026797
EBNA4	NIPSNAP3B	0.052368
EBNA4	EID1	0.14359
EBNA4	STX6	0.1479
EBNA4	SZRD1	0.12086
EBNA4	SNW1	0.092822
EBNA4	AC3.5	0.22843
EBNA4	DDX26B	0.28983
EBNA4	LCHN	0.10115
EBNA4	FAM45B	0.21358
EBNA4	FAM229B	0.18989
EBNA4	LINC00478	0.082437
EBNA4	C2orf81	0.24781
EBNA4	FAM84A	0.10349
EBNA4	SPANXA2-OT1	0.1457
EBNA4	ARMC2	0.012509
EBNA4	ATG2B	0.019637
EBNA4	CLIC4	0.077361
EBNA4	EIF4G1	0.031345
EBNA4	SLC22A5	0.019423
EBNA4	FAM49B	0.09668
EBNA4	BNIP2	0.10577
EBNA4	ZNF767	0.13372
EBNA4	GABARAPL2	0.024263
EBNA4	FAM199X	0.097262
EBNA4	OSER1	0.019073

EBNA4	JAG2	0.13488
EBNA4	FAM90A2P	0.028552
EBNA4	CCDC114	0.033065
EBNA4	HDGFL1	0.017103
EBNA4	SWT1	0.10854
EBNA4	GAGE12G	0.1692
EBNA4	VIMP	0.11946
EBNA4	FAM117A	0.24685
EBNA4	TIPRL	0.21331
EBNA4	EHD2	0.042327
EBNA4	CCDC13	0.02047
EBNA4	CADPS	0.22694
EBNA4	PKD2	0.010938
EBNA4	NCAPH	0.18578
EBNA4	C17orf59	0.38857
EBNA4	DRC1	0.11149
EBNA4	CRYGD	0.14365
EBNA4	ADRM1	0.12218
EBNA4	FAM161B	0.33782
EBNA4	CCAR1	0.20503
EBNA4	C3orf55	0.023758
EBNA4	C19orf57	0.19787
EBNA4	C16orf93	0.11987
EBNA4	KIAA1908	0.0396
EBNA4	C20orf62	0.05956
EBNA4	C6orf222	0.099797
EBNA4	LINC00299	0.019208
EBNA4	IQCB1	0.011351
EBNA4	CCDC171	0.11338
EBNA4	CCAR2	0.1581
EBNA4	PLAUR	0.16154
EBNA4	FAM178B	0.17724
EBNA4	GTF2A1	0.13119
EBNA4	COMMD6	0.2743
EBNA4	MIMI	0.015941
EBNA4	GABARAP	0.032667
EBNA4	C1orf195	0.45945
EBNA4	PIKFYVE	0.016598
EBNA4	CBLB	0.014289
EBNA4	CRELD2	0.21723
EBNA4	MYOZ1	0.19617
EBNA4	NCOA3	0.011106
EBNA4	TIMELESS	0.034427
EBNA4	NPIPA5	0.038189
EBNA4	PRR23A	0.16304
EBNA4	C15orf32	0.023454
EBNA4	C2orf72	0.2406
EBNA4	YBR056C-B	0.14629
EBNA4	PFKFB1	0.053608
EBNA4	EME1	0.1663
EBNA4	NBR1	0.035147
EBNA4	HFE	0.017336
EBNA4	CEP57	0.10055
EBNA4	SCIMP	0.34829
EBNA4	ARMC12	0.14854
EBNA4	DERL1	0.12346
EBNA4	GSN	0.090345
EBNA4	SLC11A2	0.027004
EBNA4	TRMT112	0.10113
EBNA4	PAIP2	0.17045
EBNA4	AMBRA1	0.025701
EBNA4	PLAC8	0.029097
EBNA4	VPS13A	0.10686
EBNA4	C3orf22	0.21522
EBNA4	EXOC3L4	0.14422
EBNA4	TBKBP1	0.13038
EBNA4	EIF4H	0.021734
EBNA4	PVRL1	0.010764

EBNA4	CYCB3-1	0.29179
EBNA4	COA7	0.067984
EBNA4	EIF4A1	0.027489
EBNA4	CABP1	0.056827
EBNA4	EAF1	0.23919
EBNA4	BEND3	0.022942
EBNA4	SLC4A4	0.064223
EBNA4	C22orf26	0.1921
EBNA4	LETM1	0.20991
EBNA4	TNFAIP8L1	0.2803
EBNA4	pkmyt1	0.15685
EBNA4	PGBD4	0.10261
EBNA4	SPHK1	0.025945
EBNA4	CRYGN	0.12698
EBNA4	CTU1	0.1668
EBNA4	PIK3CB	0.017084
EBNA4	GTF2F1	0.12317
EBNA4	FAM231A	0.60246
EBNA4	TFB1M	0.052083
EBNA4	C6orf229	0.14151
EBNA4	FAM124A	0.40683
EBNA4	AMER1	0.27946
EBNA4	PNRC1	0.26939
EBNA4	MAL	0.098089
EBNA4	FAM188A	0.18029
EBNA4	MAGEB4	0.038527
EBNA4	AP1B1	0.054694
EBNA4	RCN3	0.22941
EBNA4	PLSCR1	0.16875
EBNA4	GAGE5	0.087217
EBNA4	BCLAF1	0.10633
EBNA4	GAGE12B	0.1692
EBNA4	SLC6A4	0.026779
EBNA4	SLC12A3	0.055761
EBNA4	PSENEN	0.14233
EBNA4	ATN1	0.10696
EBNA4	ATG5	0.098812
EBNA4	CAMLG	0.17343
EBNA4	NKAPL	0.2893
EBNA4	NCKAP5L	0.29041
EBNA4	CCDC153	0.033785
EBNA4	PRR21	0.11947
EBNA4	TLDC2	0.20049
EBNA4	LTBP1	0.22078
EBNA4	TPD52L2	0.13165
EBNA4	C6orf165	0.025661
EBNA4	ASPSCR1	0.1754
EBNA4	ARL6IP4	0.16004
EBNA4	MSANTD1	0.066256
EBNA4	F2	0.055026
EBNA4	IFIT2	0.13878
EBNA4	SCNN1B	0.03862
EBNA4	SMEK3P	0.14143
EBNA4	MIEF2	0.32748
EBNA4	SYT1	0.080385
EBNA4	WRNIP1	0.098723
EBNA4	CBL	0.018942
EBNA4	C11orf52	0.11495
EBNA4	NSUN5P1	0.13708
EBNA4	PI4K2A	0.049416
EBNA4	NCS1	0.18311
EBNA4	TCAP	0.17598
EBNA4	C12orf71	0.054113
EBNA4	SRRM5	0.07413
EBNA4	CCDC81	0.095832
EBNA4	FBLN5	0.19195
EBNA4	DAG1	0.11791
EBNA4	AGTRAP	0.21715

EBNA4	C1orf192	0.14492
EBNA4	DOC2A	0.026861
EBNA4	MED30	0.038224
EBNA4	HRASLS5	0.023024
EBNA4	FBXO6	0.058257
EBNA4	HRK	0.29771
EBNA4	C2orf62	0.093585
EBNA4	SCAI	0.20736
EBNA4	NIPBL	0.029479
EBNA4	RPL7A	0.012212
EBNA4	MAGEB2	0.018512
EBNA4	YAL042C-A	0.063481
EBNA4	C19orf67	0.088893
EBNA4	C5orf48	0.27766
EBNA4	SH3BP5L	0.18874
EBNA4	CCDC17	0.050755
EBNA4	NECAB3	0.14746
EBNA4	AKAP8L	0.16659
EBNA4	MLC1	0.073982
EBNA4	PXMP4	0.17449
EBNA4	STAG1	0.12307
EBNA4	EFNA4	0.02663
EBNA4	C22orf42	0.046455
EBNA4	C19orf82	0.18543
EBNA4	SUMO4	0.023584
EBNA4	TRMT61B	0.15481
EBNA4	APH1B	0.10407
EBNA4	SNX6	0.065623
EBNA4	CDC73	0.064196
EBNA4	SECISBP2L	0.22564
EBNA4	C11orf96	0.21207
EBNA4	BBS2	0.044256
EBNA4	CDKN2AIPNL	0.2752
EBNA4	CHMP5	0.11202
EBNA4	INTS4L1	0.037336
EBNA4	FAM92A1	0.058281
EBNA4	NACA	0.076709
EBNA4	NPC1L1	0.078334
EBNA4	AASDHPPT	0.25177
EBNA4	NBEAP1	0.02498
EBNA4	RSRC1	0.29795
EBNA4	MEGF6	0.22514
EBNA4	CALR	0.022682
EBNA4	RSRC2	0.27336
EBNA4	MRPL11	0.20396
EBNA4	NUP205	0.13405
EBNA4	GAS1	0.09861
EBNA4	C16orf45	0.08899
EBNA4	GOLGA6L9	0.23368
EBNA4	C2CD4C	0.010443
EBNA4	ALS2CR11	0.098893
EBNA4	ZNRD1-AS1	0.056025
EBNA4	KCNIP2	0.086149
EBNA4	TBCD	0.071095
EBNA4	SLPI	0.13587
EBNA4	FBRSL1	0.17395
EBNA4	FSCN1	0.093997
EBNA4	LRRC14	0.039333
EBNA4	ZK1128.3	0.28593
EBNA4	CUEDC1	0.13031
EBNA4	PLCG1	0.016552
EBNA4	HEATR5B	0.19034
EBNA4	PDCD4	0.11792
EBNA4	CTAGE15	0.050462
EBNA4	LINC00303	0.062425
EBNA4	CD93	0.15207
EBNA4	CCDC54	0.050433
EBNA4	RITA1	0.16294

EBNA4	GRIN1	0.0716
EBNA4	FAN1	0.15846
EBNA4	CLINT1	0.045197
EBNA4	C20orf195	0.11807
EBNA4	C19orf66	0.2194
EBNA4	DHH	0.15219
EBNA4	NDFIP2	0.1213
EBNA4	GAGE2E	0.087217
EBNA4	UNQ6125/PRO20090	0.2612
EBNA4	SPHK2	0.048975
EBNA4	TTC29	0.022162
EBNA4	VIPAS39	0.11531
EBNA4	CT45A2	0.097421
EBNA4	N4BP2L1	0.05374
EBNA4	CDC42EP1	0.12643
EBNA4	TEX26	0.018205
EBNA4	AP1G2	0.022532
EBNA4	FAM231D	0.33938
EBNA4	SAC3D1	0.16063
EBNA4	MAGEB17	0.036972
EBNA4	XPO1	0.030315
EBNA4	KIAA0087	0.21412
EBNA4	C11orf72	0.094262
EBNA4	C9orf171	0.235
EBNA4	IQCF3	0.051105
EBNA4	LINC00615	0.20612
EBNA4	STT3A	0.067956
EBNA4	CCDC94	0.23173
EBNA4	MYL6B	0.17903
EBNA4	WDR63	0.010729
EBNA4	CCDC122	0.013106
EBNA4	SPTSSB	0.14901
EBNA4	RBBP8NL	0.082383
EBNA4	RGAG4	0.18048
EBNA4	ATP6V0A1	0.047321
EBNA4	SCG5	0.29392
EBNA4	TTC23L	0.070779
EBNA4	MED4	0.12854
EBNA4	RHBDD1	0.01722
EBNA4	AP1G1	0.046555
EBNA4	CDHR2	0.19133
EBNA4	CDR2L	0.067032
EBNA4	RINT1	0.12105
EBNA4	SNX17	0.034388
EBNA4	CDH5	0.12644
EBNA4	GR6	0.10334
EBNA4	IL6R	0.042478
EBNA4	TP53INP2	0.16578
EBNA4	SMN1	0.075387
EBNA4	SYT2	0.1843
EBNA4	CAPNS1	0.081172
EBNA4	GTF2E2	0.26069
EBNA4	C3orf14	0.016949
EBNA4	GABARAPL1	0.022799
EBNA4	TPD52	0.21856
EBNA-LP	HPS4	0.2955
EBNA-LP	CBX7	0.27592
EBNA-LP	ACR	0.25112
EBNA-LP	ACVR1	0.047085
EBNA-LP	TRIB3	0.099203
EBNA-LP	HDAC6	0.018947
EBNA-LP	ubl4ab	0.11042
EBNA-LP	FPR1	0.24899
EBNA-LP	BRAP	0.25175
EBNA-LP	SERPINB9	0.24281
EBNA-LP	SLC25A25	0.17996
EBNA-LP	MACROD1	0.36462
EBNA-LP	ITGB5	0.21516

```
EBNA-LP HID1	0.29367
EBNA-LP ECT2	0.060987
EBNA-LP PEX10	0.29255
EBNA-LP NLRP2	0.28264
EBNA-LP RBL1	0.16275
EBNA-LP BIN2	0.41868
EBNA-LP BBS10	0.31433
EBNA-LP USP2	0.15798
EBNA-LP MED1	0.025657
EBNA-LP CDC34	0.18421
EBNA-LP CDK10	0.19807
EBNA-LP EPHA3	0.1539
EBNA-LP EFCAB1	0.2099
EBNA-LP PIK3CD	0.14992
EBNA-LP RAB7A	0.205
EBNA-LP TGFBR2	0.038607
EBNA-LP N4BP1	0.33976
EBNA-LP CTDNEP1 0.18049
EBNA-LP FAM58A	0.2493
EBNA-LP PSMD4	0.18315
EBNA-LP FZD8	0.12915
EBNA-LP PPP5C	0.16574
EBNA-LP SLC2A4	0.18168
EBNA-LP KEL 0.23009
EBNA-LP IFT122	0.3446
EBNA-LP NAE1	0.24013
EBNA-LP PRPF6	0.26184
EBNA-LP SPTA1	0.31098
EBNA-LP SEC31A	0.21041
EBNA-LP GZMA	0.28907
EBNA-LP S100A7	0.39394
EBNA-LP NMRK1	0.02529
EBNA-LP PXDN	0.010764
EBNA-LP ROCK2	0.024862
EBNA-LP IL4 0.058798
EBNA-LP NANOS1	0.3418
EBNA-LP SLC26A8 0.27462
EBNA-LP UBE2H	0.25962
EBNA-LP LDLR	0.16777
EBNA-LP NEDD4	0.075731
EBNA-LP LAMTOR5 0.27596
EBNA-LP GSPT2	0.2498
EBNA-LP SERPINB5	0.30328
EBNA-LP BIRC6	0.17847
EBNA-LP SERTAD3 0.30669
EBNA-LP INO80B	0.26243
EBNA-LP KLK13	0.33948
EBNA-LP AGO2	0.13909
EBNA-LP HEPACAM2	0.2482
EBNA-LP RNMTL1	0.14844
EBNA-LP CENPW	0.27116
EBNA-LP UNC13D	0.26157
EBNA-LP UPF3A	0.36044
EBNA-LP FABP2	0.014584
EBNA-LP ARHGAP33	0.28812
EBNA-LP RB1CC1	0.13391
EBNA-LP RABEP1	0.3796
EBNA-LP SKA3	0.27549
EBNA-LP VPS54	0.35012
EBNA-LP EIF5A	0.14558
EBNA-LP LIMK1	0.13617
EBNA-LP FAF1	0.17571
EBNA-LP IRF9	0.091631
EBNA-LP SLC5A1	0.23506
EBNA-LP LRP2BP	0.41868
EBNA-LP MAPK8	0.095041
EBNA-LP EDF1	0.13689
EBNA-LP KCNS1	0.25667
```

EBNA-LP PXN 0.1562
EBNA-LP NUP85 0.16752
EBNA-LP SOX13 0.18804
EBNA-LP CTNND2 0.23838
EBNA-LP UBE2D3 0.14119
EBNA-LP C12orf61 0.10826
EBNA-LP KRT5 0.26506
EBNA-LP HSD17B8 0.25333
EBNA-LP BTNL8 0.40592
EBNA-LP PLSCR3 0.54958
EBNA-LP KREMEN1 0.37087
EBNA-LP TCF25 0.17397
EBNA-LP NR0B1 0.078033
EBNA-LP SNX20 0.32835
EBNA-LP PRSS16 0.089492
EBNA-LP BIRC7 0.21984
EBNA-LP MCM6 0.15183
EBNA-LP FKBP10 0.178
EBNA-LP KRT17 0.22626
EBNA-LP MAPKAPK2 0.081473
EBNA-LP NOSIP 0.25835
EBNA-LP SMURF2 0.14131
EBNA-LP DYM 0.3171
EBNA-LP ANKIB1 0.36476
EBNA-LP ZC3HAV1L 0.012018
EBNA-LP ATP6V0D2 0.24873
EBNA-LP ORAI3 0.40592
EBNA-LP CSNK2A1 0.13015
EBNA-LP PFKM 0.17153
EBNA-LP ELAVL2 0.42497
EBNA-LP RPUSD4 0.53764
EBNA-LP EMR1 0.11255
EBNA-LP CD248 0.18344
EBNA-LP ASIC1 0.22803
EBNA-LP ORC2 0.24434
EBNA-LP TAF12 0.056105
EBNA-LP CALN1 0.16954
EBNA-LP F9 0.18345
EBNA-LP CRB1 0.19741
EBNA-LP TRAPPC2 0.19987
EBNA-LP NIP7 0.43173
EBNA-LP C16orf70 0.36969
EBNA-LP USP12 0.30416
EBNA-LP DDB2 0.24732
EBNA-LP PIP5K1A 0.15146
EBNA-LP PEAK1 0.24315
EBNA-LP SUN1 0.2425
EBNA-LP SFTPD 0.30184
EBNA-LP PIP5K1B 0.34091
EBNA-LP FAT3 0.17399
EBNA-LP TAB3 0.16936
EBNA-LP SBF2 0.23545
EBNA-LP SLC7A11 0.19409
EBNA-LP BNIP1 0.2063
EBNA-LP FGFR1OP 0.1873
EBNA-LP PCDH20 0.17939
EBNA-LP PTPRH 0.12291
EBNA-LP SPRR1A 0.23391
EBNA-LP CALCOCO2 0.29653
EBNA-LP USHBP1 0.39845
EBNA-LP CD1B 0.32727
EBNA-LP LALBA 0.15379
EBNA-LP BRD4 0.2416
EBNA-LP EFCAB5 0.2099
EBNA-LP TFAP2B 0.051271
EBNA-LP PRPF4B 0.20367
EBNA-LP MYF5 0.16883
EBNA-LP ARHGEF12 0.14673

EBNA-LP	RNF2	0.14856
EBNA-LP	LLGL2	0.29319
EBNA-LP	MYH7	0.24269
EBNA-LP	SORBS3	0.20068
EBNA-LP	POLD2	0.19749
EBNA-LP	SNAPIN	0.22627
EBNA-LP	ZDHHC8	0.099125
EBNA-LP	STX3	0.20936
EBNA-LP	PCDH12	0.14369
EBNA-LP	PDE2A	0.089039
EBNA-LP	PLXNB1	0.1639
EBNA-LP	RAB13	0.024394
EBNA-LP	CARTPT	0.20018
EBNA-LP	HMGB1	0.081693
EBNA-LP	KCNJ5	0.31752
EBNA-LP	MLKL	0.23202
EBNA-LP	MKS1	0.26799
EBNA-LP	LYN	0.010971
EBNA-LP	MAPK7	0.10663
EBNA-LP	USP11	0.27959
EBNA-LP	DTX1	0.16525
EBNA-LP	ADAMTS5	0.18431
EBNA-LP	ENDOU	0.051393
EBNA-LP	RANBP9	0.27804
EBNA-LP	SPHKAP	0.40592
EBNA-LP	PXMP2	0.33739
EBNA-LP	SYTL1	0.31774
EBNA-LP	SOCS7	0.29886
EBNA-LP	MAPK1	0.015365
EBNA-LP	TYRP1	0.2536
EBNA-LP	ZFPM1	0.065328
EBNA-LP	UBA1	0.24657
EBNA-LP	HDAC2	0.021242
EBNA-LP	BTBD2	0.39913
EBNA-LP	EIF3J	0.39866
EBNA-LP	RTN2	0.32525
EBNA-LP	RP1	0.20574
EBNA-LP	CENPJ	0.16783
EBNA-LP	BCCIP	0.33806
EBNA-LP	F5	0.2693
EBNA-LP	IGSF1	0.32052
EBNA-LP	KCND2	0.16895
EBNA-LP	PTPN1	0.095918
EBNA-LP	FKBP1B	0.15435
EBNA-LP	APLP2	0.16407
EBNA-LP	C10orf62	0.43184
EBNA-LP	TERF2	0.15179
EBNA-LP	RNF167	0.25767
EBNA-LP	UNC119B	0.35801
EBNA-LP	RACGAP1	0.079944
EBNA-LP	RBL2	0.22786
EBNA-LP	YMR013W-A	0.043946
EBNA-LP	CTBP2	0.20998
EBNA-LP	JAK3	0.11112
EBNA-LP	CCNB1IP1	0.2713
EBNA-LP	ACTA1	0.18636
EBNA-LP	PSMA1	0.11805
EBNA-LP	DHX30	0.36
EBNA-LP	NLRX1	0.23957
EBNA-LP	VASP	0.23621
EBNA-LP	PCDHGA1	0.17939
EBNA-LP	TRAF6	0.017428
EBNA-LP	CABP7	0.16954
EBNA-LP	CASC3	0.24075
EBNA-LP	WASF2	0.25583
EBNA-LP	CD58	0.32535
EBNA-LP	DUSP1	0.065239
EBNA-LP	MAP2	0.21831

EBNA-LP	TBL1XR1	0.16509
EBNA-LP	ZFYVE27	0.24162
EBNA-LP	APBB1	0.073631
EBNA-LP	DNM3	0.12486
EBNA-LP	PCNT	0.26905
EBNA-LP	U2AF1	0.2702
EBNA-LP	ARHGDIA	0.2411
EBNA-LP	TFDP1	0.073983
EBNA-LP	PDXP	0.16671
EBNA-LP	BOC	0.2619
EBNA-LP	P4HB	0.12844
EBNA-LP	GAREM	0.43184
EBNA-LP	ALAS2	0.20887
EBNA-LP	IGFBP5	0.18425
EBNA-LP	MAN1B1	0.091939
EBNA-LP	ZNF675	0.15851
EBNA-LP	PRKCD	0.041315
EBNA-LP	DVL2	0.14271
EBNA-LP	MLH1	0.094231
EBNA-LP	CHRD	0.21155
EBNA-LP	BICD2	0.39755
EBNA-LP	APOB	0.038731
EBNA-LP	KIF7	0.28853
EBNA-LP	ADAM9	0.079168
EBNA-LP	ATP5B	0.21683
EBNA-LP	NIN	0.52809
EBNA-LP	UBB	0.050245
EBNA-LP	TXNDC17	0.34938
EBNA-LP	BGLAP	0.02463
EBNA-LP	ARHGEF1	0.16886
EBNA-LP	PYCARD	0.044708
EBNA-LP	MUL1	0.070523
EBNA-LP	ITCH	0.13086
EBNA-LP	SYTL2	0.21249
EBNA-LP	PGR	0.07872
EBNA-LP	CARD11	0.18461
EBNA-LP	LMO2	0.16892
EBNA-LP	KCNIP1	0.42621
EBNA-LP	CDC27	0.2145
EBNA-LP	VPS26A	0.27328
EBNA-LP	BATF	0.20055
EBNA-LP	SH3BP5	0.39154
EBNA-LP	CDH18	0.16534
EBNA-LP	PLCL2	0.025725
EBNA-LP	AKTIP	0.24962
EBNA-LP	MARCH8	0.041833
EBNA-LP	ANKEF1	0.2099
EBNA-LP	TNIP1	0.26512
EBNA-LP	TLE3	0.28008
EBNA-LP	STIM2	0.51232
EBNA-LP	KIN	0.0418
EBNA-LP	FAM109A	0.29716
EBNA-LP	GP1BA	0.23489
EBNA-LP	XRN1	0.24118
EBNA-LP	WBP2	0.40778
EBNA-LP	CEACAM1	0.30601
EBNA-LP	NUP107	0.20957
EBNA-LP	ERBB3	0.095941
EBNA-LP	NEK6	0.05133
EBNA-LP	SYNPR	0.011429
EBNA-LP	ARL11	0.37468
EBNA-LP	MEGF9	0.18755
EBNA-LP	CBLC	0.089541
EBNA-LP	SLAIN2	0.3827
EBNA-LP	EIF4E3	0.037699
EBNA-LP	SSSCA1	0.37965
EBNA-LP	CCDC130	0.40774
EBNA-LP	BNIPL	0.29306

EBNA-LP	KRT7	0.21249
EBNA-LP	ZHX2	0.11225
EBNA-LP	CTCFL	0.18918
EBNA-LP	RPLP1	0.29518
EBNA-LP	DERL3	0.32591
EBNA-LP	SERGEF	0.30768
EBNA-LP	COG2	0.25858
EBNA-LP	CD247	0.26057
EBNA-LP	MAN1A2	0.07124
EBNA-LP	SLC23A2	0.034041
EBNA-LP	DSN1	0.26075
EBNA-LP	ELK1	0.12755
EBNA-LP	TRAF2	0.072138
EBNA-LP	TCEA2	0.20303
EBNA-LP	TMED10	0.19945
EBNA-LP	RALBP1	0.28774
EBNA-LP	NDUFAF4	0.4218
EBNA-LP	BUB1B	0.099601
EBNA-LP	CST5	0.44471
EBNA-LP	NFKBIB	0.18116
EBNA-LP	THOC5	0.27696
EBNA-LP	NAIP	0.27594
EBNA-LP	DPY30	0.24304
EBNA-LP	EFCAB14	0.2099
EBNA-LP	PVRL3	0.27505
EBNA-LP	LRIF1	0.33861
EBNA-LP	ANGEL1	0.016382
EBNA-LP	TNFAIP3	0.037268
EBNA-LP	PLCB4	0.093499
EBNA-LP	TNKS	0.11895
EBNA-LP	TMEM30B	0.39355
EBNA-LP	PYDC1	0.28432
EBNA-LP	PITPNM2	0.14408
EBNA-LP	SOX4	0.043505
EBNA-LP	NCAPD2	0.26788
EBNA-LP	MIA3	0.29626
EBNA-LP	UHRF2	0.1789
EBNA-LP	PDIA3	0.12478
EBNA-LP	BIRC3	0.20031
EBNA-LP	PABPN1	0.22399
EBNA-LP	TAF3	0.21146
EBNA-LP	COMMD5	0.37277
EBNA-LP	ACAN	0.22985
EBNA-LP	CLASP2	0.12518
EBNA-LP	NACA	0.27064
EBNA-LP	JAK2	0.021392
EBNA-LP	WIPF1	0.3888
EBNA-LP	SPTAN1	0.37413
EBNA-LP	NDE1	0.12959
EBNA-LP	BECN1	0.20304
EBNA-LP	PTPN3	0.13016
EBNA-LP	FZD5	0.037857
EBNA-LP	CD81	0.213
EBNA-LP	CDC37	0.23987
EBNA-LP	NOL8	0.37125
EBNA-LP	PPM1E	0.17305
EBNA-LP	SPNS1	0.35255
EBNA-LP	RIMS1	0.20166
EBNA-LP	ATRIP	0.36257
EBNA-LP	COPS2	0.28633
EBNA-LP	NDUFAB1	0.11831
EBNA-LP	DDR1	0.1341
EBNA-LP	COL13A1	0.23421
EBNA-LP	RAD52	0.31131
EBNA-LP	TCF7	0.093136
EBNA-LP	LAMTOR1	0.22489
EBNA-LP	DHPS	0.38127
EBNA-LP	IL12A	0.13315

EBNA-LP SMC3 0.15169
EBNA-LP SNX24 0.12516
EBNA-LP STAT5A 0.056459
EBNA-LP THAP1 0.21521
EBNA-LP TUBA3C 0.12741
EBNA-LP CDK5RAP2 0.13118
EBNA-LP PRX 0.30368
EBNA-LP MCRS1 0.2349
EBNA-LP POP4 0.33204
EBNA-LP FBLN1 0.1664
EBNA-LP EDIL3 0.17947
EBNA-LP AIMP1 0.2256
EBNA-LP MASP1 0.54472
EBNA-LP TBL1X 0.10646
EBNA-LP AIF1 0.051063
EBNA-LP ZNF205 0.24903
EBNA-LP INPP5D 0.12161
EBNA-LP GCC2 0.20698
EBNA-LP THRB 0.063223
EBNA-LP SCUBE3 0.66771
EBNA-LP CALML6 0.11139
EBNA-LP KIF13B 0.19167
EBNA-LP SLC9A7 0.22334
EBNA-LP CNOT3 0.24637
EBNA-LP PTPRM 0.1022
EBNA-LP BCL9 0.30506
EBNA-LP NGFR 0.094602
EBNA-LP TADA3 0.104
EBNA-LP STRAP 0.22829
EBNA-LP ITPKC 0.043878
EBNA-LP PROZ 0.12584
EBNA-LP LSM3 0.29909
EBNA-LP DDO 0.26363
EBNA-LP PEA15 0.33132
EBNA-LP RPS27L 0.24813
EBNA-LP RBFOX1 0.35589
EBNA-LP AMIGO2 0.31094
EBNA-LP MTSS1 0.17107
EBNA-LP PSMC3 0.10175
EBNA-LP HUWE1 0.22546
EBNA-LP UXT 0.18418
EBNA-LP TRAPPC4 0.36126
EBNA-LP TRIM17 0.28409
EBNA-LP HECTD3 0.23702
EBNA-LP TH 0.035481
EBNA-LP TNFRSF10B 0.25799
EBNA-LP SCAP 0.22722
EBNA-LP C1QB 0.32461
EBNA-LP TRIM13 0.173
EBNA-LP FBXO7 0.30796
EBNA-LP HIPK1 0.11455
EBNA-LP CAMK2A 0.13633
EBNA-LP CEP152 0.18964
EBNA-LP PTPN6 0.037937
EBNA-LP APOH 0.17152
EBNA-LP HEXIM1 0.26713
EBNA-LP PTPRS 0.13668
EBNA-LP ATXN10 0.29415
EBNA-LP SCN5A 0.10254
EBNA-LP GEMIN5 0.32957
EBNA-LP COMMD7 0.36263
EBNA-LP FHIT 0.1916
EBNA-LP ADAM17 0.058467
EBNA-LP CSNK1A1 0.11158
EBNA-LP ATG4B 0.3212
EBNA-LP TERF1 0.11934
EBNA-LP MAL2 0.34943
EBNA-LP EEF1A1 0.093306

EBNA-LP	PCDHGB1	0.17939
EBNA-LP	AGTR1	0.11891
EBNA-LP	SEC24B	0.19355
EBNA-LP	ALYREF	0.2586
EBNA-LP	KPNA1	0.23626
EBNA-LP	AATK	0.23783
EBNA-LP	NCF4	0.25455
EBNA-LP	MED8	0.24177
EBNA-LP	ATG16L1	0.33246
EBNA-LP	USP19	0.19822
EBNA-LP	RIN3	0.37341
EBNA-LP	LDOC1	0.37504
EBNA-LP	FSBP	0.32616
EBNA-LP	SH3D19	0.25142
EBNA-LP	TNS4	0.34848
EBNA-LP	SUV420H1	0.011607
EBNA-LP	MORF4L2	0.28135
EBNA-LP	TSR2	0.41866
EBNA-LP	SAV1	0.253
EBNA-LP	EPB41	0.13744
EBNA-LP	CDH16	0.17939
EBNA-LP	PDE9A	0.19188
EBNA-LP	PEX19	0.22405
EBNA-LP	NAGK	0.42365
EBNA-LP	SLC22A6	0.25615
EBNA-LP	BMP7	0.037097
EBNA-LP	PPAP2B	0.10168
EBNA-LP	TIA1	0.3384
EBNA-LP	SLC34A2	0.045539
EBNA-LP	SNRPG	0.23587
EBNA-LP	PRKCI	0.028823
EBNA-LP	TP53RK	0.27289
EBNA-LP	PCDH18	0.17399
EBNA-LP	TECPR1	0.32719
EBNA-LP	RRM2	0.23384
EBNA-LP	CD300LD	0.39356
EBNA-LP	KCNJ10	0.13736
EBNA-LP	PDP1	0.038981
EBNA-LP	CUL4A	0.2734
EBNA-LP	CD244	0.339
EBNA-LP	GATA4	0.023757
EBNA-LP	KCNH1	0.19076
EBNA-LP	UFC1	0.40593
EBNA-LP	PAIP2B	0.39352
EBNA-LP	PTPRN	0.21182
EBNA-LP	KAT7	0.16755
EBNA-LP	PML	0.036166
EBNA-LP	RPGR	0.29549
EBNA-LP	KBTBD6	0.39536
EBNA-LP	WDR5	0.21392
EBNA-LP	GOLGA4	0.28784
EBNA-LP	MALL	0.31868
EBNA-LP	CHMP4B	0.32077
EBNA-LP	TLK2	0.21543
EBNA-LP	NLRC4	0.16136
EBNA-LP	PROC	0.47375
EBNA-LP	PDCD6	0.52241
EBNA-LP	DKC1	0.30304
EBNA-LP	USP32	0.16132
EBNA-LP	OTUD7B	0.23796
EBNA-LP	S100A9	0.1829
EBNA-LP	ANXA2	0.3118
EBNA-LP	TMPO	0.32076
EBNA-LP	MMP20	0.4123
EBNA-LP	RAET1G	0.35833
EBNA-LP	DAZL	0.26441
EBNA-LP	NR2C2AP	0.35058
EBNA-LP	MCM4	0.16888

EBNA-LP	RTN4	0.10734
EBNA-LP	RUNX1	0.10898
EBNA-LP	TOP3A	0.22683
EBNA-LP	CDKN1A	0.082409
EBNA-LP	KIF23	0.18173
EBNA-LP	IL13RA1	0.37022
EBNA-LP	EZH2	0.18708
EBNA-LP	MATN4	0.70481
EBNA-LP	SLX4IP	0.43184
EBNA-LP	POLG	0.26764
EBNA-LP	PSMF1	0.16931
EBNA-LP	FMR1	0.39057
EBNA-LP	MAP1A	0.19043
EBNA-LP	NOTCH3	0.26206
EBNA-LP	DNMT3L	0.24171
EBNA-LP	VPS72	0.28457
EBNA-LP	CXXC1	0.20929
EBNA-LP	PVALB	0.20216
EBNA-LP	RNH1	0.66193
EBNA-LP	LRRC47	0.39853
EBNA-LP	FOXRED2	0.43764
EBNA-LP	PKM	0.1543
EBNA-LP	ARHGEF6	0.17234
EBNA-LP	PIK3C2B	0.25292
EBNA-LP	MFF	0.19267
EBNA-LP	ATF2	0.025075
EBNA-LP	MMRN2	0.38347
EBNA-LP	FBXL12	0.39355
EBNA-LP	EGFL7	0.15322
EBNA-LP	POR	0.056905
EBNA-LP	ASPN	0.19363
EBNA-LP	MYL1	0.1562
EBNA-LP	ARL4D	0.23395
EBNA-LP	NUMA1	0.14501
EBNA-LP	MED23	0.22115
EBNA-LP	ADIPOQ	0.032441
EBNA-LP	TBC1D24	0.31365
EBNA-LP	HLA-DRB1	0.09048
EBNA-LP	MED22	0.25377
EBNA-LP	TGFB3	0.054343
EBNA-LP	KIR2DL3	0.28537
EBNA-LP	EGLN1	0.17277
EBNA-LP	TRDMT1	0.020177
EBNA-LP	PLXNC1	0.2625
EBNA-LP	SIAH1	0.13195
EBNA-LP	C2CD5	0.18845
EBNA-LP	SNX27	0.271
EBNA-LP	PTPRJ	0.062288
EBNA-LP	NDUFA9	0.25314
EBNA-LP	PRKCA	0.014993
EBNA-LP	PVRL4	0.31393
EBNA-LP	PPP6R1	0.33925
EBNA-LP	FRS2	0.16607
EBNA-LP	SPSB2	0.36483
EBNA-LP	ROBO1	0.14312
EBNA-LP	PAIP1	0.3299
EBNA-LP	WRAP53	0.42304
EBNA-LP	MORF4L1	0.25126
EBNA-LP	SELPLG	0.257
EBNA-LP	CLIP1	0.282
EBNA-LP	ATP1A1	0.12813
EBNA-LP	YTHDF3	0.43184
EBNA-LP	HCK	0.090675
EBNA-LP	USP6	0.2162
EBNA-LP	TEK	0.06396
EBNA-LP	TAP2	0.10302
EBNA-LP	UGP2	0.20619
EBNA-LP	EXOSC5	0.26222

EBNA-LP	UBXN7	0.33246
EBNA-LP	TSHR	0.2654
EBNA-LP	NECAB1	0.11139
EBNA-LP	PLXDC2	0.40592
EBNA-LP	BLOC1S3	0.21239
EBNA-LP	AMOT	0.20658
EBNA-LP	MAATS1	0.406
EBNA-LP	TBC1D8	0.13737
EBNA-LP	THY1	0.05892
EBNA-LP	ALOX5AP	0.1757
EBNA-LP	DLK2	0.20592
EBNA-LP	BHLHE40	0.097794
EBNA-LP	TTLL6	0.25775
EBNA-LP	SLC4A1	0.22713
EBNA-LP	PTK7	0.1578
EBNA-LP	ADI1	0.2293
EBNA-LP	PCDHB5	0.1636
EBNA-LP	TOB1	0.34164
EBNA-LP	MYOD1	0.040386
EBNA-LP	BORA	0.26318
EBNA-LP	WDR12	0.26578
EBNA-LP	MAN1C1	0.076695
EBNA-LP	HM13	0.22281
EBNA-LP	JUNB	0.053806
EBNA-LP	SLC25A17	0.19778
EBNA-LP	CHUK	0.034942
EBNA-LP	VPS28	0.29743
EBNA-LP	URM1	0.35971
EBNA-LP	NUF2	0.25563
EBNA-LP	CABS1	0.17471
EBNA-LP	KLF10	0.10099
EBNA-LP	MAGEA2	0.26754
EBNA-LP	TRAK2	0.20018
EBNA-LP	PRKACA	0.023471
EBNA-LP	CASP8AP2	0.22928
EBNA-LP	FNTB	0.22156
EBNA-LP	RNF7	0.24854
EBNA-LP	CHFR	0.20971
EBNA-LP	CLN3	0.060098
EBNA-LP	CRTC1	0.23629
EBNA-LP	QKI	0.3324
EBNA-LP	RTN4R	0.31095
EBNA-LP	RBM15	0.3303
EBNA-LP	STXBP2	0.22679
EBNA-LP	KSR1	0.10601
EBNA-LP	POLR1A	0.22585
EBNA-LP	EPCAM	0.32957
EBNA-LP	UMODL1	0.16593
EBNA-LP	GNL3	0.2481
EBNA-LP	VPS33B	0.24297
EBNA-LP	UACA	0.21881
EBNA-LP	GLS	0.23034
EBNA-LP	PAK3	0.11005
EBNA-LP	C5orf30	0.32537
EBNA-LP	MYL4	0.15168
EBNA-LP	CD8B	0.26693
EBNA-LP	EIF4G2	0.39551
EBNA-LP	TOM1L1	0.27684
EBNA-LP	CAPN14	0.09102
EBNA-LP	RABGGTB	0.37381
EBNA-LP	COLQ	0.22823
EBNA-LP	ARHGEF7	0.068242
EBNA-LP	THRA	0.025537
EBNA-LP	NPAT	0.14094
EBNA-LP	ORAI1	0.26062
EBNA-LP	RAD21	0.21084
EBNA-LP	MAPT	0.074932
EBNA-LP	UCK1	0.051751

EBNA-LP	CD80	0.24195
EBNA-LP	PCDH11Y	0.17939
EBNA-LP	METTL1	0.33245
EBNA-LP	MRFAP1	0.36383
EBNA-LP	MMS19	0.18264
EBNA-LP	ITGB3	0.080452
EBNA-LP	INSR	0.028136
EBNA-LP	TLN2	0.16004
EBNA-LP	ASB9	0.33034
EBNA-LP	TRPC3	0.21676
EBNA-LP	GATA2	0.031626
EBNA-LP	CIZ1	0.2776
EBNA-LP	CHD8	0.13072
EBNA-LP	RPL11	0.20618
EBNA-LP	NLRP1	0.18427
EBNA-LP	NCBP1	0.17404
EBNA-LP	HDAC4	0.026017
EBNA-LP	MMP10	0.055871
EBNA-LP	EDC3	0.35991
EBNA-LP	MAD2L2	0.22869
EBNA-LP	MGRN1	0.18679
EBNA-LP	CRKL	0.28747
EBNA-LP	USP7	0.14165
EBNA-LP	PPP2R2B	0.21966
EBNA-LP	BGLAP	0.17892
EBNA-LP	TIMP3	0.25717
EBNA-LP	ALDH7A1	0.27678
EBNA-LP	GDI2	0.26128
EBNA-LP	PTK6	0.14162
EBNA-LP	ZRSR2	0.27076
EBNA-LP	CD200R1	0.33709
EBNA-LP	UHRF1	0.107
EBNA-LP	SULT2A1	0.29689
EBNA-LP	NEBL	0.34556
EBNA-LP	KLKB1	0.29699
EBNA-LP	TMF1	0.24543
EBNA-LP	IL1R1	0.26136
EBNA-LP	LATS2	0.13505
EBNA-LP	MTA2	0.065711
EBNA-LP	CREB3L3	0.15492
EBNA-LP	NCOA7	0.27043
EBNA-LP	PCDHA5	0.16848
EBNA-LP	RABGAP1	0.26627
EBNA-LP	SMARCD1	0.25079
EBNA-LP	SHROOM2	0.16522
EBNA-LP	ZNF148	0.098077
EBNA-LP	PCDH11X	0.16857
EBNA-LP	KBTBD7	0.39536
EBNA-LP	ERCC4	0.11496
EBNA-LP	CYBA	0.088801
EBNA-LP	LTBR	0.26601
EBNA-LP	ARHGAP8	0.31345
EBNA-LP	MYOCD	0.059128
EBNA-LP	GH1	0.21817
EBNA-LP	CTH	0.11342
EBNA-LP	SHANK2	0.34013
EBNA-LP	CDH20	0.17394
EBNA-LP	SLA2	0.32874
EBNA-LP	XRCC6	0.052871
EBNA-LP	GATA1	0.02381
EBNA-LP	PPARA	0.037172
EBNA-LP	GPIHBP1	0.1305
EBNA-LP	FCGR1A	0.28288
EBNA-LP	SH3GL1	0.36949
EBNA-LP	ARVCF	0.33095
EBNA-LP	CBX2	0.23403
EBNA-LP	PLEKHA5	0.26714
EBNA-LP	FOXD3	0.052152

```
EBNA-LP EIF3F   0.35391
EBNA-LP PPP3R2  0.2099
EBNA-LP CIITA   0.18171
EBNA-LP UPF2    0.37585
EBNA-LP CACNB2  0.24014
EBNA-LP SIN3B   0.22868
EBNA-LP TNPO3   0.27855
EBNA-LP CACNA1C 0.18083
EBNA-LP SIK1    0.060127
EBNA-LP SH3RF1  0.21686
EBNA-LP TRPM6   0.24839
EBNA-LP RAB5A   0.20138
EBNA-LP ERBB2IP 0.20671
EBNA-LP LPCAT2  0.09343
EBNA-LP OPA1    0.15392
EBNA-LP PPP2R5D 0.29059
EBNA-LP BIN1    0.2235
EBNA-LP BCL2L11 0.066749
EBNA-LP EIF4ENIF1   0.26546
EBNA-LP SYNRG   0.16469
EBNA-LP GNAI1   0.127
EBNA-LP HIST2H3A    0.2528
EBNA-LP PSMB9   0.10457
EBNA-LP RBMY1A1 0.40569
EBNA-LP EEF1G   0.29771
EBNA-LP COL7A1  0.29624
EBNA-LP STAT4   0.059718
EBNA-LP RAG1    0.12448
EBNA-LP RRAD    0.31715
EBNA-LP STX4    0.17211
EBNA-LP LOXL4   0.30692
EBNA-LP GFI1    0.14171
EBNA-LP SIK3    0.20716
EBNA-LP FAM83F  0.041183
EBNA-LP ROBO2   0.17684
EBNA-LP CALCA   0.13698
EBNA-LP NUP214  0.23683
EBNA-LP NAA50   0.44093
EBNA-LP STAU1   0.31804
EBNA-LP YKT6    0.067689
EBNA-LP CHAMP1  0.18883
EBNA-LP ARHGEF15    0.289
EBNA-LP TPCN1   0.20126
EBNA-LP MERTK   0.1539
EBNA-LP SRF 0.017928
EBNA-LP UBL4A   0.35339
EBNA-LP CREBZF  0.16749
EBNA-LP NFKB1   0.031568
EBNA-LP STAT3   0.044985
EBNA-LP ZNF488  0.2271
EBNA-LP RHBDL2  0.019192
EBNA-LP SLC5A2  0.04072
EBNA-LP ACIN1   0.2802
EBNA-LP BARD1   0.21288
EBNA-LP PPFIA4  0.38163
EBNA-LP CKB 0.29435
EBNA-LP TNFSF12 0.18732
EBNA-LP TNS3    0.37088
EBNA-LP KMT2C   0.27403
EBNA-LP KLHL20  0.23288
EBNA-LP RAD23A  0.25216
EBNA-LP SMG9    0.31611
EBNA-LP PAK1    0.02703
EBNA-LP HPCAL4  0.20013
EBNA-LP VPS4A   0.19435
EBNA-LP COCH    0.32067
EBNA-LP PSPC1   0.32823
EBNA-LP STK40   0.22229
```

EBNA-LP	SRPX2	0.26239
EBNA-LP	ORC4	0.22581
EBNA-LP	THBD	0.4043
EBNA-LP	GLYCTK	0.42023
EBNA-LP	ARHGEF4	0.15253
EBNA-LP	SMAD5	0.07774
EBNA-LP	KIAA0226	0.31587
EBNA-LP	DCHS1	0.1739
EBNA-LP	CREM	0.14054
EBNA-LP	ZFR	0.35591
EBNA-LP	RTP4	0.36413
EBNA-LP	FSHR	0.18822
EBNA-LP	FANCM	0.23132
EBNA-LP	EEF1D	0.27748
EBNA-LP	TRADD	0.14981
EBNA-LP	PYGO1	0.26667
EBNA-LP	DHX8	0.30195
EBNA-LP	MTERF	0.26471
EBNA-LP	ATG13	0.23597
EBNA-LP	BRAF	0.19167
EBNA-LP	NPBWR2	0.31
EBNA-LP	ARPC2	0.25748
EBNA-LP	ARFGEF2	0.1417
EBNA-LP	TTC19	0.30229
EBNA-LP	BSG	0.19649
EBNA-LP	MT2A	0.26138
EBNA-LP	ORM1	0.37698
EBNA-LP	SLC18A3	0.021674
EBNA-LP	HSD17B10	0.17477
EBNA-LP	RBM5	0.25724
EBNA-LP	BOP1	0.24466
EBNA-LP	NOX1	0.10779
EBNA-LP	GYG1	0.30233
EBNA-LP	DSC2	0.15558
EBNA-LP	CAPS	0.15702
EBNA-LP	CLSPN	0.27514
EBNA-LP	MLST8	0.2955
EBNA-LP	WTIP	0.20094
EBNA-LP	SPTB	0.19542
EBNA-LP	IL1R2	0.39636
EBNA-LP	KLF13	0.19734
EBNA-LP	MSN	0.12042
EBNA-LP	SPDL1	0.20114
EBNA-LP	UBE2D4	0.23719
EBNA-LP	PCSK1	0.022527
EBNA-LP	SMARCE1	0.19543
EBNA-LP	PIGA	0.28775
EBNA-LP	RPS26	0.21036
EBNA-LP	MEN1	0.056017
EBNA-LP	GNMT	0.29759
EBNA-LP	ZBTB14	0.24149
EBNA-LP	SCAMP1	0.31951
EBNA-LP	SEC23A	0.17962
EBNA-LP	RND1	0.19702
EBNA-LP	BCKDHB	0.30693
EBNA-LP	PDLIM7	0.24788
EBNA-LP	SSH1	0.080521
EBNA-LP	RNMT	0.40614
EBNA-LP	TNFRSF21	0.34062
EBNA-LP	AK5	0.060839
EBNA-LP	KIFC3	0.071711
EBNA-LP	TTF2	0.17454
EBNA-LP	TOPORS	0.11292
EBNA-LP	DDX47	0.31725
EBNA-LP	FREM2	0.13107
EBNA-LP	GOLM1	0.38133
EBNA-LP	GNAI2	0.096788
EBNA-LP	DDX42	0.33629

EBNA-LP	GAPDHS	0.25056
EBNA-LP	CDKN1C	0.27135
EBNA-LP	GRIA1	0.14767
EBNA-LP	ANKLE2	0.26381
EBNA-LP	NUP153	0.18059
EBNA-LP	ETS2	0.10043
EBNA-LP	TOMM22	0.23874
EBNA-LP	VPS41	0.22701
EBNA-LP	MAGOH	0.30692
EBNA-LP	SKI	0.053238
EBNA-LP	SRGN	0.21207
EBNA-LP	CEP41	0.2551
EBNA-LP	RAC1	0.047175
EBNA-LP	INTS9	0.36347
EBNA-LP	IPO5	0.26785
EBNA-LP	RPUSD3	0.036358
EBNA-LP	VDAC1	0.17043
EBNA-LP	PRMT5	0.20387
EBNA-LP	TNFAIP1	0.23665
EBNA-LP	GRIN2A	0.017701
EBNA-LP	CRNN	0.18565
EBNA-LP	ING4	0.19477
EBNA-LP	PECAM1	0.26126
EBNA-LP	EML1	0.66231
EBNA-LP	GTF2F2	0.16334
EBNA-LP	CEP170	0.34938
EBNA-LP	C19orf68	0.43184
EBNA-LP	NOL3	0.29783
EBNA-LP	ZWILCH	0.30259
EBNA-LP	VAV2	0.11381
EBNA-LP	KIF24	0.32149
EBNA-LP	ETS1	0.04677
EBNA-LP	PTGER4	0.15568
EBNA-LP	RPRD1B	0.26619
EBNA-LP	CWC15	0.42433
EBNA-LP	CALCRL	0.12226
EBNA-LP	RBPMS	0.29419
EBNA-LP	AK1	0.053818
EBNA-LP	DES	0.22006
EBNA-LP	WDR4	0.32846
EBNA-LP	PSMC1	0.13926
EBNA-LP	USP5	0.21824
EBNA-LP	CITED1	0.04595
EBNA-LP	HAT1	0.29249
EBNA-LP	SNCAIP	0.28719
EBNA-LP	FZD3	0.15977
EBNA-LP	LIN28A	0.19631
EBNA-LP	MPDZ	0.27429
EBNA-LP	NGFRAP1	0.25764
EBNA-LP	ARID2	0.24681
EBNA-LP	TLDC1	0.43184
EBNA-LP	COMMD3	0.43184
EBNA-LP	SMAD7	0.0507
EBNA-LP	SERPINA1	0.28265
EBNA-LP	CAV1	0.010744
EBNA-LP	NCK1	0.15146
EBNA-LP	GABBR1	0.10475
EBNA-LP	ADAD2	0.027299
EBNA-LP	CRTAC1	0.21388
EBNA-LP	SCP2	0.12386
EBNA-LP	TMEM173	0.078724
EBNA-LP	MAD2L1	0.16873
EBNA-LP	PRRC2B	0.43184
EBNA-LP	KCNJ3	0.29857
EBNA-LP	BYSL	0.2809
EBNA-LP	ERN1	0.070823
EBNA-LP	SPRTN	0.28423
EBNA-LP	MYD88	0.080759

EBNA-LP	LSM5	0.31048
EBNA-LP	CTC1	0.25151
EBNA-LP	FBXW8	0.3936
EBNA-LP	SPPL2A	0.22293
EBNA-LP	PLS1	0.18522
EBNA-LP	SYVN1	0.17694
EBNA-LP	CACYBP	0.28916
EBNA-LP	INS	0.04747
EBNA-LP	IRAK4	0.16113
EBNA-LP	EPS15	0.3553
EBNA-LP	PLS3	0.19742
EBNA-LP	MYO5B	0.31639
EBNA-LP	WWC1	0.20417
EBNA-LP	HUS1	0.25223
EBNA-LP	PCSK2	0.37151
EBNA-LP	CRHR1	0.10176
EBNA-LP	WHAMM	0.094428
EBNA-LP	APLF	0.24313
EBNA-LP	YY1	0.10086
EBNA-LP	NKRF	0.3016
EBNA-LP	ZNF335	0.24903
EBNA-LP	AURKB	0.079907
EBNA-LP	CDC42	0.014301
EBNA-LP	RRAGC	0.17264
EBNA-LP	LZTS1	0.10578
EBNA-LP	CD48	0.24752
EBNA-LP	VWCE	0.20358
EBNA-LP	CLEC3B	0.14843
EBNA-LP	PRMT8	0.31201
EBNA-LP	SLC17A7	0.021009
EBNA-LP	INPP5B	0.23611
EBNA-LP	ARHGEF16	0.14977
EBNA-LP	BST2	0.15984
EBNA-LP	NHLH2	0.23492
EBNA-LP	PEX11G	0.32817
EBNA-LP	OGDHL	0.36135
EBNA-LP	FKBP8	0.17735
EBNA-LP	DISC1	0.17849
EBNA-LP	PUM2	0.43454
EBNA-LP	ESPL1	0.19735
EBNA-LP	PCGF1	0.24271
EBNA-LP	CDH8	0.16534
EBNA-LP	LTC4S	0.15338
EBNA-LP	AP3S1	0.39907
EBNA-LP	UHMK1	0.24726
EBNA-LP	BUB3	0.19198
EBNA-LP	ALDH3A1	0.019375
EBNA-LP	RBMY1B	0.39055
EBNA-LP	SPRR3	0.21604
EBNA-LP	SDF4	0.47976
EBNA-LP	KIF14	0.26935
EBNA-LP	AFF4	0.17759
EBNA-LP	LEPR	0.32184
EBNA-LP	ANKHD1	0.44531
EBNA-LP	MGP	0.12242
EBNA-LP	BCKDHA	0.32467
EBNA-LP	TRIM33	0.13173
EBNA-LP	KIF5B	0.21971
EBNA-LP	HEG1	0.09672
EBNA-LP	PRKCQ	0.045304
EBNA-LP	PHIP	0.23792
EBNA-LP	TAF11	0.25137
EBNA-LP	TRPC7	0.23231
EBNA-LP	LAMTOR2	0.26551
EBNA-LP	MAZ	0.28439
EBNA-LP	HIST1H1E	0.2791
EBNA-LP	WSB1	0.3864
EBNA-LP	CDC45	0.25311

EBNA-LP	YIPF5	0.049846
EBNA-LP	YAF2	0.21067
EBNA-LP	NSUN4	0.047982
EBNA-LP	MYH9	0.086584
EBNA-LP	EID3	0.2692
EBNA-LP	SPACA3	0.30974
EBNA-LP	INSIG2	0.17891
EBNA-LP	CDCA8	0.22127
EBNA-LP	STAT1	0.1002
EBNA-LP	PRKAB1	0.13591
EBNA-LP	GJA1	0.050726
EBNA-LP	PSEN2	0.030697
EBNA-LP	FAT4	0.64138
EBNA-LP	DTNBP1	0.1886
EBNA-LP	CUL9	0.2785
EBNA-LP	PNMA2	0.36524
EBNA-LP	WWP1	0.17998
EBNA-LP	PLIN3	0.29266
EBNA-LP	KCNIP4	0.16097
EBNA-LP	USP34	0.30338
EBNA-LP	ZNHIT6	0.31769
EBNA-LP	VPS18	0.24219
EBNA-LP	ARHGAP4	0.22834
EBNA-LP	IFI16	0.10014
EBNA-LP	PPEF1	0.52025
EBNA-LP	AP1M2	0.03586
EBNA-LP	ZW10	0.13136
EBNA-LP	GRIN3A	0.14545
EBNA-LP	CXorf67	0.11779
EBNA-LP	NPW	0.34733
EBNA-LP	MAPK11	0.10102
EBNA-LP	TLL1	0.04908
EBNA-LP	A2M	0.20648
EBNA-LP	DCTN1	0.2359
EBNA-LP	FTSJ3	0.4152
EBNA-LP	ETV1	0.15072
EBNA-LP	PRKAA2	0.035704
EBNA-LP	NELL2	0.54054
EBNA-LP	CALB2	0.21883
EBNA-LP	DDX60	0.2758
EBNA-LP	CLTB	0.30909
EBNA-LP	PCDHA7	0.16848
EBNA-LP	CHD4	0.1028
EBNA-LP	ZC3H10	0.35368
EBNA-LP	ARRB2	0.098724
EBNA-LP	PRDX1	0.25697
EBNA-LP	RHOG	0.18583
EBNA-LP	SLC2A8	0.02182
EBNA-LP	TTC3	0.21027
EBNA-LP	SPON1	0.38456
EBNA-LP	IRAK3	0.10591
EBNA-LP	CYFIP1	0.21518
EBNA-LP	NR2F1	0.10476
EBNA-LP	ULK2	0.29751
EBNA-LP	SRSF3	0.33299
EBNA-LP	PDZK1	0.34242
EBNA-LP	MCC	0.29139
EBNA-LP	KAT2B	0.052395
EBNA-LP	CROCC	0.19703
EBNA-LP	LAMTOR3	0.30244
EBNA-LP	ARRB1	0.06761
EBNA-LP	ELOVL4	0.25886
EBNA-LP	CAPN9	0.087369
EBNA-LP	FAM188B2	0.10997
EBNA-LP	UBE3A	0.14838
EBNA-LP	TCF4	0.11461
EBNA-LP	GTF3C1	0.2596
EBNA-LP	EIF1AX	0.026496

EBNA-LP	SMC1A	0.12882
EBNA-LP	DMAP1	0.22789
EBNA-LP	CHEK2	0.065037
EBNA-LP	STX1A	0.13926
EBNA-LP	DRD2	0.020188
EBNA-LP	APH1A	0.17763
EBNA-LP	BAZ1B	0.17571
EBNA-LP	AKIP1	0.37519
EBNA-LP	PCDHGC5	0.17939
EBNA-LP	KAT2A	0.15987
EBNA-LP	COL4A3BP	0.095267
EBNA-LP	SEMA4C	0.026288
EBNA-LP	TRPC4	0.19441
EBNA-LP	KIF5C	0.26202
EBNA-LP	FZD10	0.16018
EBNA-LP	PIN1	0.21216
EBNA-LP	TLX3	0.16117
EBNA-LP	SUV420H2	0.33377
EBNA-LP	PCDHGA7	0.17939
EBNA-LP	HECTD1	0.26948
EBNA-LP	NME1	0.17496
EBNA-LP	NOP58	0.29723
EBNA-LP	MAP1LC3C	0.42508
EBNA-LP	ATP6V1E1	0.24337
EBNA-LP	ATP11B	0.14419
EBNA-LP	RNF14	0.1502
EBNA-LP	TARBP2	0.20656
EBNA-LP	TAB1	0.14543
EBNA-LP	RHOA	0.043107
EBNA-LP	KCNH2	0.14833
EBNA-LP	KRT20	0.29783
EBNA-LP	ZNF646	0.24903
EBNA-LP	MAP4K4	0.23035
EBNA-LP	DCAF11	0.41438
EBNA-LP	THBS1	0.063809
EBNA-LP	RUNX3	0.095916
EBNA-LP	LMTK2	0.18763
EBNA-LP	DIS3	0.23282
EBNA-LP	ARR3	0.29277
EBNA-LP	ABCD3	0.22965
EBNA-LP	UBR2	0.18965
EBNA-LP	CMKLR1	0.23292
EBNA-LP	STK25	0.19486
EBNA-LP	PLA2G4A	0.028418
EBNA-LP	ICAM3	0.28139
EBNA-LP	MRPL49	0.3399
EBNA-LP	CAPN8	0.085744
EBNA-LP	PANK4	0.014336
EBNA-LP	EIF3I	0.39866
EBNA-LP	ARL2BP	0.28981
EBNA-LP	UBTF	0.24437
EBNA-LP	FGF2	0.034679
EBNA-LP	PFN1	0.18989
EBNA-LP	DLG3	0.19243
EBNA-LP	RBCK1	0.10091
EBNA-LP	DSG2	0.13502
EBNA-LP	MAP3K12	0.10784
EBNA-LP	CCNE1	0.081778
EBNA-LP	RHOC	0.11575
EBNA-LP	PITX2	0.022858
EBNA-LP	ADRA2B	0.1653
EBNA-LP	ATXN3	0.15773
EBNA-LP	EPB41L3	0.20553
EBNA-LP	CEBPG	0.093374
EBNA-LP	ALDH16A1	0.018149
EBNA-LP	DEDD2	0.21097
EBNA-LP	GIT2	0.24004
EBNA-LP	SNX33	0.4059

EBNA-LP STK24 0.14315
EBNA-LP PCDHB2 0.15367
EBNA-LP ATG101 0.3259
EBNA-LP MIF 0.14414
EBNA-LP ISG20L2 0.26435
EBNA-LP BICD1 0.2199
EBNA-LP AP2M1 0.23962
EBNA-LP ING2 0.14384
EBNA-LP PIK3CG 0.10165
EBNA-LP ADORA1 0.050976
EBNA-LP CDC42EP2 0.24749
EBNA-LP SF3A1 0.35741
EBNA-LP INTS3 0.3415
EBNA-LP DSG4 0.13913
EBNA-LP TCTEX1D4 0.43184
EBNA-LP TMCC2 0.40592
EBNA-LP PLG 0.1409
EBNA-LP TEAD3 0.13953
EBNA-LP ROCK1 0.037866
EBNA-LP NOS2 0.10854
EBNA-LP LPCAT3 0.023115
EBNA-LP PRMT1 0.34167
EBNA-LP USP10 0.21349
EBNA-LP LASP1 0.25401
EBNA-LP RASSF5 0.325
EBNA-LP CLTA 0.058222
EBNA-LP CRB2 0.18572
EBNA-LP DYNLT1 0.18113
EBNA-LP ZMIZ2 0.22883
EBNA-LP ALDOB 0.068303
EBNA-LP S100Z 0.2099
EBNA-LP MED26 0.21705
EBNA-LP LRP5 0.12842
EBNA-LP HSD17B12 0.18849
EBNA-LP DGKQ 0.23815
EBNA-LP CRP 0.25828
EBNA-LP ARIH2 0.20244
EBNA-LP DHX36 0.23572
EBNA-LP C1QBP 0.25571
EBNA-LP DDOST 0.24318
EBNA-LP AGR3 0.4732
EBNA-LP AP1M1 0.29567
EBNA-LP PUF60 0.26124
EBNA-LP GMCL1P1 0.37167
EBNA-LP ECM29 0.015554
EBNA-LP STRA13 0.26592
EBNA-LP SNX8 0.32262
EBNA-LP MAPK6 0.15213
EBNA-LP SPOP 0.3573
EBNA-LP PRKCZ 0.058129
EBNA-LP HDDC2 0.30579
EBNA-LP CYCS 0.12786
EBNA-LP SETDB1 0.28332
EBNA-LP CDC14A 0.079569
EBNA-LP SEC22B 0.2526
EBNA-LP ATP7A 0.030794
EBNA-LP AGR2 0.43178
EBNA-LP UQCRC2 0.16332
EBNA-LP FHL2 0.086358
EBNA-LP ADM 0.10585
EBNA-LP TPPP 0.48931
EBNA-LP ZNF746 0.19058
EBNA-LP PRPF4 0.30509
EBNA-LP DBNDD2 0.40592
EBNA-LP BMP10 0.15261
EBNA-LP TBXA2R 0.16847
EBNA-LP FYB 0.23408
EBNA-LP SYT4 0.21907

EBNA-LP IL17RE 0.38158
EBNA-LP CENPT 0.23901
EBNA-LP RAD51D 0.29539
EBNA-LP SYT11 0.59029
EBNA-LP CD5 0.28406
EBNA-LP SH2D4A 0.33709
EBNA-LP ICAM1 0.084631
EBNA-LP GZMB 0.27313
EBNA-LP GGA2 0.27376
EBNA-LP MAD1L1 0.17568
EBNA-LP DCUN1D1 0.40596
EBNA-LP LPAR1 0.10982
EBNA-LP MOB3B 0.39136
EBNA-LP MSH6 0.090677
EBNA-LP PTCH1 0.058022
EBNA-LP EIF2B1 0.19208
EBNA-LP PDE3B 0.13571
EBNA-LP DRD3 0.074566
EBNA-LP CTNNA3 0.21676
EBNA-LP IFITM1 0.23171
EBNA-LP FOXH1 0.023661
EBNA-LP RECQL 0.20123
EBNA-LP BCS1L 0.26834
EBNA-LP SH3PXD2A 0.33602
EBNA-LP SYK 0.027009
EBNA-LP PARPBP 0.34346
EBNA-LP FBXL15 0.3324
EBNA-LP FANCA 0.31121
EBNA-LP EIF2B3 0.27579
EBNA-LP KDM1A 0.040592
EBNA-LP PCDHAC2 0.16848
EBNA-LP CLSTN2 0.10494
EBNA-LP RBMS1 0.37257
EBNA-LP ATP6V0D1 0.20958
EBNA-LP TPD52L1 0.29749
EBNA-LP WDFY3 0.32468
EBNA-LP PSMA4 0.10156
EBNA-LP HSP90B1 0.34098
EBNA-LP NR2C1 0.12838
EBNA-LP PCDH7 0.17377
EBNA-LP PIAS1 0.14222
EBNA-LP HOXB7 0.14769
EBNA-LP TNFSF13B 0.18249
EBNA-LP CDK6 0.033373
EBNA-LP MPP5 0.30431
EBNA-LP TRIM2 0.28262
EBNA-LP DDAH2 0.27472
EBNA-LP ATPAF2 0.39363
EBNA-LP MACF1 0.3807
EBNA-LP AIFM1 0.1791
EBNA-LP CKAP5 0.22986
EBNA-LP TNFRSF12A 0.25265
EBNA-LP POLR3F 0.22221
EBNA-LP S100A3 0.17789
EBNA-LP MEIS1 0.12583
EBNA-LP WWOX 0.23911
EBNA-LP ADORA2A 0.045811
EBNA-LP SOS1 0.13051
EBNA-LP APOD 0.16144
EBNA-LP TXNL4A 0.31844
EBNA-LP EVL 0.25979
EBNA-LP BOK 0.24282
EBNA-LP ALG2 0.23085
EBNA-LP GLMN 0.34345
EBNA-LP MAGEC2 0.34421
EBNA-LP PNLIPRP1 0.068524
EBNA-LP TPSAB1 0.37151
EBNA-LP TXNRD2 0.36248

EBNA-LP	FGF21	0.33092
EBNA-LP	PCGF3	0.26633
EBNA-LP	MSR1	0.32356
EBNA-LP	DUOX2	0.185
EBNA-LP	RTN1	0.32515
EBNA-LP	RBM14	0.19233
EBNA-LP	OSBPL6	0.32063
EBNA-LP	CCDC176	0.13931
EBNA-LP	STBD1	0.35837
EBNA-LP	CASQ2	0.13775
EBNA-LP	MCTP2	0.18708
EBNA-LP	PRKG1	0.15002
EBNA-LP	GSTO1	0.016524
EBNA-LP	LPXN	0.18679
EBNA-LP	PUS7L	0.061464
EBNA-LP	UBE2A	0.20435
EBNA-LP	EPHA7	0.15673
EBNA-LP	SNTB2	0.31065
EBNA-LP	CX3CL1	0.11118
EBNA-LP	PCDHGB4	0.1739
EBNA-LP	PPIE	0.32783
EBNA-LP	KLRK1	0.21661
EBNA-LP	TBC1D25	0.38256
EBNA-LP	DNAJC7	0.33956
EBNA-LP	GCH1	0.18981
EBNA-LP	PTGES3	0.1984
EBNA-LP	ANKRD1	0.13868
EBNA-LP	VPS11	0.33269
EBNA-LP	POLE3	0.28986
EBNA-LP	SLC9A3	0.24658
EBNA-LP	OGN	0.28009
EBNA-LP	OPRD1	0.19197
EBNA-LP	MDM2	0.073901
EBNA-LP	NKD2	0.47881
EBNA-LP	LSAMP	0.36691
EBNA-LP	TRAPPC1	0.045346
EBNA-LP	PTPN23	0.15449
EBNA-LP	ADAMTSL2	0.28345
EBNA-LP	MAGI3	0.23294
EBNA-LP	SLAMF1	0.24584
EBNA-LP	IFT57	0.28282
EBNA-LP	ADAM23	0.2261
EBNA-LP	AP5B1	0.38662
EBNA-LP	TRIM29	0.18224
EBNA-LP	EIF2B5	0.25382
EBNA-LP	ZNF350	0.24636
EBNA-LP	SV2A	0.035956
EBNA-LP	TMEM132D	0.37001
EBNA-LP	MAPK8IP1	0.1164
EBNA-LP	CHKA	0.022157
EBNA-LP	FOXN3	0.084441
EBNA-LP	PTPN4	0.1472
EBNA-LP	ATP2B2	0.10095
EBNA-LP	BTG2	0.1941
EBNA-LP	PEX3	0.25069
EBNA-LP	GHITM	0.34788
EBNA-LP	INCENP	0.19812
EBNA-LP	GPX6	0.031297
EBNA-LP	TBC1D9	0.15094
EBNA-LP	DOT1L	0.33037
EBNA-LP	UCK2	0.047269
EBNA-LP	JMJD1C	0.32739
EBNA-LP	GJD3	0.33529
EBNA-LP	FNBP1L	0.35382
EBNA-LP	ITGAV	0.10159
EBNA-LP	TXLNB	0.40591
EBNA-LP	CDH10	0.16032
EBNA-LP	CDKN2AIP	0.31636

EBNA-LP	KEAP1	0.25503
EBNA-LP	EPHA10	0.26999
EBNA-LP	CNOT6	0.24639
EBNA-LP	LCK	0.05836
EBNA-LP	HIPK2	0.052391
EBNA-LP	PSMC4	0.12769
EBNA-LP	FAT1	0.39566
EBNA-LP	REV1	0.21282
EBNA-LP	BAZ1A	0.20807
EBNA-LP	OCRL	0.17194
EBNA-LP	DYRK1B	0.17417
EBNA-LP	PRKD2	0.016084
EBNA-LP	ABCF1	0.24907
EBNA-LP	HDAC3	0.059285
EBNA-LP	FBLN7	0.18264
EBNA-LP	NXF3	0.40085
EBNA-LP	H2AFY	0.23964
EBNA-LP	FOXK1	0.053608
EBNA-LP	CCNH	0.050776
EBNA-LP	FCN1	0.052047
EBNA-LP	NGLY1	0.32932
EBNA-LP	IRF1	0.024135
EBNA-LP	EDAR	0.24746
EBNA-LP	MTCH1	0.31219
EBNA-LP	ERCC8	0.24863
EBNA-LP	CRELD1	0.18971
EBNA-LP	TGOLN2	0.31136
EBNA-LP	HABP4	0.24717
EBNA-LP	XAB2	0.27718
EBNA-LP	S100A11	0.54428
EBNA-LP	ITGA5	0.1506
EBNA-LP	GDF7	0.21288
EBNA-LP	GPS2	0.3297
EBNA-LP	SRSF5	0.28623
EBNA-LP	USH1G	0.2729
EBNA-LP	SORBS1	0.13567
EBNA-LP	CHMP1B	0.30895
EBNA-LP	HFE2	0.28886
EBNA-LP	IRF3	0.051012
EBNA-LP	EFCAB6	0.14813
EBNA-LP	CCDC137	0.06539
EBNA-LP	RBBP6	0.22034
EBNA-LP	MAP3K3	0.19009
EBNA-LP	ESR2	0.071975
EBNA-LP	TPP1	0.2003
EBNA-LP	NCAPG	0.30444
EBNA-LP	NUFIP2	0.44367
EBNA-LP	ECSIT	0.36479
EBNA-LP	LRRFIP2	0.3843
EBNA-LP	AIRE	0.12078
EBNA-LP	XPO4	0.34712
EBNA-LP	NKD1	0.50079
EBNA-LP	NR1H2	0.06866
EBNA-LP	PPP1R9B	0.1674
EBNA-LP	PLEKHM2	0.27362
EBNA-LP	CBFA2T3	0.081992
EBNA-LP	CYBB	0.15717
EBNA-LP	DERL2	0.27213
EBNA-LP	ACACA	0.18792
EBNA-LP	SPTBN4	0.091993
EBNA-LP	STAP1	0.15102
EBNA-LP	EDA2R	0.25963
EBNA-LP	PPP6C	0.26289
EBNA-LP	RBFOX2	0.25094
EBNA-LP	GCK	0.093372
EBNA-LP	RPS10	0.2024
EBNA-LP	CD2AP	0.28424
EBNA-LP	RAP2A	0.20293

EBNA-LP	CD1A	0.32663
EBNA-LP	CBX8	0.25171
EBNA-LP	BCDIN3D	0.082323
EBNA-LP	RNF146	0.21682
EBNA-LP	IGHG1	0.29525
EBNA-LP	KIF18A	0.14916
EBNA-LP	GORAB	0.35412
EBNA-LP	GABPB1	0.13741
EBNA-LP	GREM1	0.062673
EBNA-LP	INTS12	0.30419
EBNA-LP	UBE2N	0.10488
EBNA-LP	NSUN7	0.050303
EBNA-LP	MAGEB18	0.43184
EBNA-LP	MED31	0.25139
EBNA-LP	MAT2B	0.21248
EBNA-LP	HMCN1	0.21824
EBNA-LP	SLC9A3R2	0.20156
EBNA-LP	KNSTRN	0.22465
EBNA-LP	ODF2	0.15921
EBNA-LP	SERPINE1	0.067594
EBNA-LP	SPC25	0.25564
EBNA-LP	RBM25	0.29275
EBNA-LP	DACH1	0.097245
EBNA-LP	PKP1	0.29269
EBNA-LP	EIF1AY	0.53784
EBNA-LP	PLA2G2E	0.075881
EBNA-LP	FNIP2	0.31968
EBNA-LP	DEAF1	0.19421
EBNA-LP	ARID4B	0.27198
EBNA-LP	EMR2	0.10808
EBNA-LP	TREML1	0.33273
EBNA-LP	MOV10	0.2872
EBNA-LP	MAGI1	0.26966
EBNA-LP	PTGIS	0.08426
EBNA-LP	ARL4A	0.29162
EBNA-LP	BPTF	0.16162
EBNA-LP	WRN	0.033272
EBNA-LP	USF1	0.049167
EBNA-LP	STMN2	0.2619
EBNA-LP	TAAR1	0.012412
EBNA-LP	CAPS2	0.2099
EBNA-LP	FAM47B	0.014277
EBNA-LP	ARHGAP32	0.15679
EBNA-LP	MTDH	0.14865
EBNA-LP	DNA2	0.010828
EBNA-LP	RP9	0.29526
EBNA-LP	PRRC2A	0.37519
EBNA-LP	MKKS	0.10982
EBNA-LP	GMNN	0.24291
EBNA-LP	PDZD11	0.37946
EBNA-LP	METTL17	0.38053
EBNA-LP	MYLPF	0.1738
EBNA-LP	PSMD10	0.084429
EBNA-LP	SDHAF2	0.37141
EBNA-LP	ST13	0.25251
EBNA-LP	APOA1	0.051523
EBNA-LP	PSMA5	0.10835
EBNA-LP	HHEX	0.024446
EBNA-LP	PPP2R3B	0.50558
EBNA-LP	PEG10	0.26864
EBNA-LP	INO80	0.16885
EBNA-LP	CTNNBIP1	0.22589
EBNA-LP	CENPH	0.19887
EBNA-LP	HDAC9	0.0795
EBNA-LP	HEY1	0.065114
EBNA-LP	ACTN4	0.3004
EBNA-LP	FBXO46	0.43184
EBNA-LP	CDK9	0.043215

EBNA-LP	KIAA0141	0.39363
EBNA-LP	DTX3L	0.26315
EBNA-LP	EHD1	0.32944
EBNA-LP	ATL1	0.1651
EBNA-LP	KIR2DL1	0.33762
EBNA-LP	RAB25	0.3601
EBNA-LP	TIMM44	0.25926
EBNA-LP	PARD6B	0.22927
EBNA-LP	GTF2E1	0.22218
EBNA-LP	AP3S2	0.065362
EBNA-LP	RAN	0.13344
EBNA-LP	KIAA0408	0.43184
EBNA-LP	LILRA4	0.40592
EBNA-LP	EEA1	0.24798
EBNA-LP	SUN2	0.19364
EBNA-LP	SEPT8	0.35239
EBNA-LP	PINK1	0.075067
EBNA-LP	FANCG	0.26029
EBNA-LP	CTU2	0.35924
EBNA-LP	OXA1L	0.2455
EBNA-LP	INTS10	0.34308
EBNA-LP	LPL	0.097416
EBNA-LP	PKMYT1	0.11106
EBNA-LP	ERCC6	0.1023
EBNA-LP	PARVA	0.1729
EBNA-LP	VCAN	0.1059
EBNA-LP	CDR2	0.36376
EBNA-LP	KRIT1	0.25862
EBNA-LP	RABAC1	0.34174
EBNA-LP	RUNX2	0.057934
EBNA-LP	AMFR	0.12539
EBNA-LP	MAP3K13	0.064186
EBNA-LP	NEU4	0.21271
EBNA-LP	CNOT6L	0.24879
EBNA-LP	RPS19BP1	0.35412
EBNA-LP	COMMD1	0.24864
EBNA-LP	SEPT2	0.13501
EBNA-LP	RBM4	0.15452
EBNA-LP	KPNA3	0.24027
EBNA-LP	ZNRF1	0.29721
EBNA-LP	GRB7	0.14925
EBNA-LP	PLCB1	0.041135
EBNA-LP	ZSCAN21	0.14721
EBNA-LP	TBP	0.04956
EBNA-LP	TESK1	0.23212
EBNA-LP	CDC42BPG	0.099272
EBNA-LP	PRMT10	0.055343
EBNA-LP	CDC25A	0.047514
EBNA-LP	CABP4	0.15762
EBNA-LP	GEMIN6	0.27901
EBNA-LP	MAPK12	0.10641
EBNA-LP	MMP2	0.11905
EBNA-LP	SLC1A5	0.018442
EBNA-LP	INHA	0.138
EBNA-LP	CSN2	0.20619
EBNA-LP	POLE4	0.29882
EBNA-LP	EIF2B2	0.2541
EBNA-LP	RPTN	0.22568
EBNA-LP	EXOSC1	0.32266
EBNA-LP	DSP	0.14524
EBNA-LP	RGS19	0.20738
EBNA-LP	ZFYVE20	0.21876
EBNA-LP	UBE2G2	0.23763
EBNA-LP	RMI1	0.37527
EBNA-LP	AIP	0.1319
EBNA-LP	S100A13	0.41237
EBNA-LP	PIK3R6	0.20558
EBNA-LP	STUB1	0.14465

EBNA-LP	MELK	0.33561
EBNA-LP	EXO1	0.15059
EBNA-LP	FAM69B	0.13447
EBNA-LP	GEM	0.2291
EBNA-LP	NEK1	0.16869
EBNA-LP	HPGDS	0.1526
EBNA-LP	MED18	0.2617
EBNA-LP	INHBB	0.2313
EBNA-LP	HSP90AB1	0.164
EBNA-LP	CORO1B	0.35706
EBNA-LP	HTRA3	0.39002
EBNA-LP	PSMB1	0.10787
EBNA-LP	S100A1	0.47663
EBNA-LP	ZDHHC13	0.017752
EBNA-LP	EIF3H	0.38652
EBNA-LP	TMED9	0.2672
EBNA-LP	GPATCH8	0.31419
EBNA-LP	PRMT6	0.21647
EBNA-LP	MYL7	0.19917
EBNA-LP	SNRPC	0.32443
EBNA-LP	SUMO1	0.14175
EBNA-LP	PPIA	0.21731
EBNA-LP	SUMO2	0.35299
EBNA-LP	USP1	0.25599
EBNA-LP	ARHGAP24	0.1833
EBNA-LP	UTRN	0.3825
EBNA-LP	TRAF3IP3	0.40592
EBNA-LP	STAM2	0.22215
EBNA-LP	EGR2	0.10409
EBNA-LP	UBE2D2	0.21864
EBNA-LP	EGFL8	0.20614
EBNA-LP	GPX4	0.028141
EBNA-LP	RAD23B	0.23134
EBNA-LP	HHIP	0.13229
EBNA-LP	GNS	0.12991
EBNA-LP	PCGF5	0.25867
EBNA-LP	S100A2	0.19934
EBNA-LP	RPL15	0.30443
EBNA-LP	PALM	0.18244
EBNA-LP	IDE	0.054099
EBNA-LP	AP2A2	0.22775
EBNA-LP	ASH2L	0.21174
EBNA-LP	IGF1	0.03283
EBNA-LP	SLC34A1	0.30469
EBNA-LP	SCUBE2	0.20358
EBNA-LP	TBC1D2B	0.21839
EBNA-LP	MCM7	0.1703
EBNA-LP	TOP2A	0.093942
EBNA-LP	PIK3CA	0.12773
EBNA-LP	MYL9	0.14581
EBNA-LP	NOP56	0.36052
EBNA-LP	TNFRSF1B	0.26289
EBNA-LP	BAHD1	0.25902
EBNA-LP	E4F1	0.068838
EBNA-LP	SPRY2	0.08061
EBNA-LP	ENG	0.073853
EBNA-LP	MYO18A	0.27718
EBNA-LP	RARA	0.012416
EBNA-LP	HAND1	0.036737
EBNA-LP	ACP1	0.12641
EBNA-LP	AQP1	0.07321
EBNA-LP	MAPKAPK3	0.10681
EBNA-LP	ELN	0.28179
EBNA-LP	SWI5	0.38068
EBNA-LP	LTF	0.18944
EBNA-LP	PPM1B	0.21984
EBNA-LP	TBC1D10A	0.31408
EBNA-LP	EFHD1	0.1912

EBNA-LP	MAP2K3	0.093333
EBNA-LP	SMC2	0.22759
EBNA-LP	ZC3HAV1	0.26998
EBNA-LP	SAMM50	0.24916
EBNA-LP	FASLG	0.10486
EBNA-LP	VWF	0.20377
EBNA-LP	TMBIM4	0.29743
EBNA-LP	ZFPM2	0.11869
EBNA-LP	EEF2K	0.08143
EBNA-LP	CIB4	0.2099
EBNA-LP	TSG101	0.12648
EBNA-LP	CRADD	0.23711
EBNA-LP	SPTBN1	0.14139
EBNA-LP	EDEM2	0.11776
EBNA-LP	KANK1	0.19866
EBNA-LP	NUP37	0.21047
EBNA-LP	CHGA	0.094812
EBNA-LP	FLII	0.25624
EBNA-LP	EMD	0.20944
EBNA-LP	ECHS1	0.21511
EBNA-LP	NTRK1	0.088223
EBNA-LP	RBM17	0.39293
EBNA-LP	THBS3	0.14799
EBNA-LP	CUBN	0.063642
EBNA-LP	PSPH	0.099442
EBNA-LP	S100A14	0.13313
EBNA-LP	STAT6	0.26143
EBNA-LP	MPZL2	0.32751
EBNA-LP	INVS	0.26118
EBNA-LP	DTNB	0.1401
EBNA-LP	REST	0.017178
EBNA-LP	USP4	0.24587
EBNA-LP	RBPJ	0.055416
EBNA-LP	ATG3	0.24932
EBNA-LP	PRRG3	0.18575
EBNA-LP	ULK1	0.097742
EBNA-LP	LLGL1	0.091209
EBNA-LP	SPOCK2	0.18571
EBNA-LP	HYAL2	0.06903
EBNA-LP	STAMBP	0.19536
EBNA-LP	CTLA4	0.30706
EBNA-LP	SLIT3	0.097405
EBNA-LP	ARPC5	0.28095
EBNA-LP	NELFE	0.27882
EBNA-LP	CLEC1B	0.34846
EBNA-LP	FOXP3	0.011601
EBNA-LP	ATRAID	0.24017
EBNA-LP	FBXO4	0.29624
EBNA-LP	HNRNPL	0.28547
EBNA-LP	TLE2	0.25526
EBNA-LP	PPP2R2A	0.26796
EBNA-LP	AKAP5	0.066077
EBNA-LP	CALB1	0.42744
EBNA-LP	DAZAP2	0.36377
EBNA-LP	CPSF2	0.33148
EBNA-LP	MAP4K1	0.21458
EBNA-LP	PCGF6	0.14131
EBNA-LP	PEX5	0.14833
EBNA-LP	RYK	0.20561
EBNA-LP	RASSF6	0.39323
EBNA-LP	NDEL1	0.13308
EBNA-LP	HHLA3	0.43184
EBNA-LP	NPHS2	0.32522
EBNA-LP	DIP2A	0.24049
EBNA-LP	GAN	0.35176
EBNA-LP	PDIA4	0.19839
EBNA-LP	MIER1	0.24591
EBNA-LP	MAP3K11	0.13231

EBNA-LP	BAZ2A	0.20455
EBNA-LP	CAPN11	0.17683
EBNA-LP	SFTPC	0.24223
EBNA-LP	UFD1L	0.322
EBNA-LP	LIN7A	0.24647
EBNA-LP	SETMAR	0.22604
EBNA-LP	VAV3	0.094615
EBNA-LP	MASP2	0.55328
EBNA-LP	BATF2	0.21534
EBNA-LP	NUDT21	0.30249
EBNA-LP	PCDHA9	0.17939
EBNA-LP	PARK7	0.21833
EBNA-LP	RP2	0.19363
EBNA-LP	FKBP1A	0.16722
EBNA-LP	TAF6L	0.16345
EBNA-LP	SLC25A23	0.17996
EBNA-LP	TRAF7	0.23074
EBNA-LP	BCL2L10	0.27161
EBNA-LP	PRCP	0.37266
EBNA-LP	SUCLA2	0.17663
EBNA-LP	TAOK3	0.20968
EBNA-LP	SSTR2	0.16939
EBNA-LP	PPP4C	0.20426
EBNA-LP	TAP1	0.11974
EBNA-LP	ATG9A	0.35786
EBNA-LP	CALML4	0.2099
EBNA-LP	NDUFAF1	0.35403
EBNA-LP	COG5	0.24466
EBNA-LP	MAP3K5	0.070434
EBNA-LP	PES1	0.22673
EBNA-LP	CCM2	0.35958
EBNA-LP	TRIM39	0.2239
EBNA-LP	ABI1	0.15603
EBNA-LP	SOST	0.21393
EBNA-LP	MAGEA3	0.43184
EBNA-LP	ZNF224	0.2336
EBNA-LP	RAMP2	0.12774
EBNA-LP	TAF13	0.12377
EBNA-LP	CAPSL	0.20356
EBNA-LP	RRAS2	0.23014
EBNA-LP	RAB4A	0.19472
EBNA-LP	FGF19	0.31672
EBNA-LP	RAB5B	0.25239
EBNA-LP	RNF32	0.31943
EBNA-LP	ARL14	0.4447
EBNA-LP	LRWD1	0.23702
EBNA-LP	TXNDC9	0.39482
EBNA-LP	ARL6	0.14941
EBNA-LP	EIF3C	0.39866
EBNA-LP	EIF3M	0.43163
EBNA-LP	ZNF326	0.30484
EBNA-LP	MYOZ3	0.41355
EBNA-LP	KLHL2	0.38477
EBNA-LP	DTX2	0.24337
EBNA-LP	COMMD10	0.43184
EBNA-LP	NOM1	0.40599
EBNA-LP	ACVR2A	0.058295
EBNA-LP	GRK5	0.10605
EBNA-LP	RFX3	0.10955
EBNA-LP	ITGB1BP2	0.13963
EBNA-LP	CENPA	0.14126
EBNA-LP	MDFIC	0.17086
EBNA-LP	UBXN6	0.31452
EBNA-LP	SYNPO2	0.34334
EBNA-LP	IL5RA	0.34044
EBNA-LP	AIF1L	0.14341
EBNA-LP	LPCAT4	0.12102
EBNA-LP	CDH11	0.15167

```
EBNA-LP RABL6   0.3156
EBNA-LP APEX1   0.10503
EBNA-LP ELK3    0.1137
EBNA-LP CBLL1   0.25749
EBNA-LP PCDHAC1 0.16848
EBNA-LP EPPIN   0.36149
EBNA-LP GAS6    0.049175
EBNA-LP BET1    0.24549
EBNA-LP ANXA2R  0.084037
EBNA-LP AMPH    0.30249
EBNA-LP ABLIM1  0.27344
EBNA-LP RANGAP1 0.16983
EBNA-LP CIRBP   0.27778
EBNA-LP ERLEC1  0.38093
EBNA-LP MEF2C   0.012095
EBNA-LP LATS1   0.071774
EBNA-LP KIFAP3  0.25526
EBNA-LP MED9    0.2617
EBNA-LP ABCB1   0.21636
EBNA-LP PCDHB6  0.15367
EBNA-LP EFEMP1  0.51719
EBNA-LP EIF4A3  0.22975
EBNA-LP LPAR2   0.2995
EBNA-LP ZAP70   0.13427
EBNA-LP DAXX    0.11272
EBNA-LP IL1A    0.14206
EBNA-LP BID 0.12979
EBNA-LP COMMD2  0.43184
EBNA-LP KANSL1  0.32796
EBNA-LP SDPR    0.2717
EBNA-LP AGRN    0.12185
EBNA-LP MKL1    0.22709
EBNA-LP ZNF281  0.09699
EBNA-LP TTC37   0.3527
EBNA-LP PNMA1   0.31527
EBNA-LP APC2    0.25698
EBNA-LP CCDC53  0.4095
EBNA-LP NIPSNAP3A   0.40649
EBNA-LP SPATA13 0.13867
EBNA-LP MYO9B   0.21923
EBNA-LP SLC6A17 0.032149
EBNA-LP C1D 0.26931
EBNA-LP BAP1    0.17752
EBNA-LP CIR1    0.15049
EBNA-LP ULK3    0.19747
EBNA-LP TBCB    0.3277
EBNA-LP SPARC   0.26865
EBNA-LP GTF2H1  0.11198
EBNA-LP CREB5   0.14315
EBNA-LP TCHHL1  0.2099
EBNA-LP EWSR1   0.30582
EBNA-LP F2RL1   0.052035
EBNA-LP OC90    0.16561
EBNA-LP EHMT2   0.23727
EBNA-LP PALLD   0.28542
EBNA-LP SRSF11  0.34463
EBNA-LP PCSK7   0.01826
EBNA-LP SMAGP   0.051507
EBNA-LP NOTCH4  0.2099
EBNA-LP RAPGEF3 0.20137
EBNA-LP PIK3C3  0.18805
EBNA-LP TERT    0.20207
EBNA-LP NUTF2   0.35092
EBNA-LP ARPC4   0.29753
EBNA-LP EXOSC10 0.17252
EBNA-LP FOXO1   0.023623
EBNA-LP PCDHB1  0.17939
EBNA-LP MEAF6   0.26172
```

```
EBNA-LP ANG 0.053642
EBNA-LP KLHL12  0.40359
EBNA-LP TSPYL5  0.28244
EBNA-LP TPH2    0.014429
EBNA-LP PIM1    0.10484
EBNA-LP DLX4    0.19544
EBNA-LP ADARB1  0.014486
EBNA-LP MBD2    0.19051
EBNA-LP MST4    0.11764
EBNA-LP TRAIP   0.26987
EBNA-LP DFNB31  0.21277
EBNA-LP CYTH2   0.22553
EBNA-LP CTR9    0.19248
EBNA-LP NAA15   0.21921
EBNA-LP MATN1   0.3953
EBNA-LP NOTCH2NL    0.18012
EBNA-LP CA4 0.15356
EBNA-LP DEPDC1  0.25905
EBNA-LP ID4 0.20997
EBNA-LP PCDH17  0.64138
EBNA-LP GLI2    0.017708
EBNA-LP CSNK2A2 0.14514
EBNA-LP PIR 0.35155
EBNA-LP IGSF8   0.33535
EBNA-LP EPHA4   0.1049
EBNA-LP MCL1    0.18336
EBNA-LP PRICKLE1    0.2122
EBNA-LP FGFBP1  0.27505
EBNA-LP GPR37L1 0.31975
EBNA-LP ARHGEF2 0.065973
EBNA-LP SLC9A1  0.14042
EBNA-LP KITLG   0.17638
EBNA-LP MARCH11 0.019548
EBNA-LP DSC3    0.14933
EBNA-LP SLC22A3 0.22795
EBNA-LP B2M 0.18803
EBNA-LP GLRB    0.17239
EBNA-LP KCNN4   0.25023
EBNA-LP PHB 0.152
EBNA-LP PRDX4   0.41256
EBNA-LP CCDC85B 0.24754
EBNA-LP NR5A1   0.04932
EBNA-LP APC 0.022115
EBNA-LP PMS2    0.21016
EBNA-LP CLSTN1  0.062965
EBNA-LP SRRM1   0.014794
EBNA-LP XK  0.40259
EBNA-LP SCAF11  0.27883
EBNA-LP EXOC3   0.38704
EBNA-LP CLGN    0.12975
EBNA-LP CIB2    0.2099
EBNA-LP LDB3    0.30976
EBNA-LP PPM1D   0.1776
EBNA-LP PLK1    0.027047
EBNA-LP KHDRBS3 0.33092
EBNA-LP ERBB4   0.044774
EBNA-LP EXD3    0.43184
EBNA-LP ADH1A   0.27543
EBNA-LP SPEN    0.15387
EBNA-LP CEP72   0.2995
EBNA-LP ALB 0.11509
EBNA-LP GGT1    0.24452
EBNA-LP YJR030C 0.017571
EBNA-LP NAP1L1  0.29518
EBNA-LP KIAA1841    0.049373
EBNA-LP EAF2    0.25434
EBNA-LP CASK    0.13885
EBNA-LP TARDBP  0.17656
```

EBNA-LP	RASSF4	0.40563
EBNA-LP	REPS1	0.64458
EBNA-LP	PLEK	0.12121
EBNA-LP	CIRH1A	0.29855
EBNA-LP	TYRO3	0.13851
EBNA-LP	RUFY1	0.30032
EBNA-LP	PEX2	0.21433
EBNA-LP	SLC18A2	0.046197
EBNA-LP	TNFRSF10A	0.25497
EBNA-LP	NSD1	0.034211
EBNA-LP	GRIP1	0.017649
EBNA-LP	TRPS1	0.12214
EBNA-LP	LRRC26	0.3219
EBNA-LP	PRODH	0.056382
EBNA-LP	SIVA1	0.20451
EBNA-LP	NLGN3	0.14762
EBNA-LP	GCC1	0.31576
EBNA-LP	AURKAIP1	0.29979
EBNA-LP	CYTIP	0.38969
EBNA-LP	USP14	0.22237
EBNA-LP	PCDHGA9	0.17939
EBNA-LP	ELOVL3	0.26443
EBNA-LP	EVI5	0.24212
EBNA-LP	RASA1	0.10616
EBNA-LP	PALB2	0.30169
EBNA-LP	CAND2	0.29731
EBNA-LP	FABP3	0.32703
EBNA-LP	LRCOL1	0.093215
EBNA-LP	CDHR5	0.15297
EBNA-LP	AHI1	0.12423
EBNA-LP	MRPS27	0.33981
EBNA-LP	ZBTB16	0.10628
EBNA-LP	SAT1	0.32533
EBNA-LP	DCTN2	0.21476
EBNA-LP	TBC1D8B	0.15094
EBNA-LP	TUBGCP3	0.1483
EBNA-LP	AGO1	0.23787
EBNA-LP	CD53	0.35848
EBNA-LP	USP25	0.26749
EBNA-LP	AFF1	0.21569
EBNA-LP	SH3GL2	0.2164
EBNA-LP	TULP1	0.23079
EBNA-LP	NXF5	0.38692
EBNA-LP	SNX1	0.24333
EBNA-LP	AQP2	0.038296
EBNA-LP	CD151	0.31666
EBNA-LP	MDFI	0.20923
EBNA-LP	YTHDC1	0.35089
EBNA-LP	KRT16	0.29557
EBNA-LP	GUCA1C	0.20973
EBNA-LP	MAP2K7	0.042863
EBNA-LP	SMAD1	0.016622
EBNA-LP	DOCK1	0.23382
EBNA-LP	CASP10	0.29814
EBNA-LP	GEMIN7	0.27883
EBNA-LP	TBC1D7	0.36089
EBNA-LP	CDH7	0.16032
EBNA-LP	SPI1	0.06754
EBNA-LP	PPP1R1A	0.37786
EBNA-LP	SEC24C	0.12724
EBNA-LP	PRKCSH	0.2129
EBNA-LP	ZCCHC11	0.22591
EBNA-LP	LAMP5	0.053428
EBNA-LP	TGFBR3	0.070113
EBNA-LP	AMY1A	0.24475
EBNA-LP	GRAP2	0.28389
EBNA-LP	EXOC7	0.31178
EBNA-LP	UPF1	0.20119

EBNA-LP	STX16	0.22074
EBNA-LP	TXNIP	0.15944
EBNA-LP	ANKRD28	0.37519
EBNA-LP	DRD4	0.092087
EBNA-LP	FGF1	0.13504
EBNA-LP	ALDH8A1	0.013414
EBNA-LP	ITPR3	0.062998
EBNA-LP	MMP1	0.05205
EBNA-LP	GAGE2D	0.41868
EBNA-LP	NEK11	0.17955
EBNA-LP	ARL13B	0.35222
EBNA-LP	ASAP1	0.10031
EBNA-LP	ADAMTS10	0.20488
EBNA-LP	YLPM1	0.29736
EBNA-LP	BRAT1	0.37519
EBNA-LP	SIGIRR	0.30458
EBNA-LP	ADRA2C	0.17621
EBNA-LP	PCDHGA2	0.17939
EBNA-LP	RDX	0.26038
EBNA-LP	TYR	0.2393
EBNA-LP	POU3F2	0.10407
EBNA-LP	ITGB4	0.1917
EBNA-LP	KAL1	0.26824
EBNA-LP	ZXDC	0.12714
EBNA-LP	DDIT3	0.092433
EBNA-LP	GTF3C6	0.35236
EBNA-LP	WIBG	0.40828
EBNA-LP	ARHGAP22	0.14209
EBNA-LP	PCDH1	0.15276
EBNA-LP	PLCL1	0.025064
EBNA-LP	SLC40A1	0.21755
EBNA-LP	FBXW5	0.19514
EBNA-LP	CFLAR	0.26176
EBNA-LP	EIF4EBP1	0.21189
EBNA-LP	MED29	0.2943
EBNA-LP	CDKN3	0.10958
EBNA-LP	TAX1BP1	0.29897
EBNA-LP	NOTCH2	0.14548
EBNA-LP	LOXL3	0.25023
EBNA-LP	ERLIN2	0.316
EBNA-LP	CDH6	0.12877
EBNA-LP	FZR1	0.21381
EBNA-LP	ROR1	0.28079
EBNA-LP	MPTX1	0.03301
EBNA-LP	AMER2	0.39141
EBNA-LP	MITD1	0.30914
EBNA-LP	TAF1D	0.31576
EBNA-LP	SLC4A1AP	0.31355
EBNA-LP	SIAH2	0.17019
EBNA-LP	EXOSC3	0.25563
EBNA-LP	STIM1	0.35802
EBNA-LP	COG4	0.29239
EBNA-LP	S100B	0.35078
EBNA-LP	TRAF3IP1	0.40592
EBNA-LP	ZAK	0.11288
EBNA-LP	FZD6	0.13214
EBNA-LP	EFHC1	0.11566
EBNA-LP	KAT6B	0.14972
EBNA-LP	HMG20B	0.16544
EBNA-LP	ESRRA	0.10106
EBNA-LP	SKP1	0.26638
EBNA-LP	VAMP1	0.34444
EBNA-LP	BIRC2	0.10485
EBNA-LP	ZNF212	0.21956
EBNA-LP	TNNC1	0.52293
EBNA-LP	DDB1	0.20987
EBNA-LP	RBMX	0.20096
EBNA-LP	SAP30	0.26239

EBNA-LP	ZNF24	0.16034
EBNA-LP	SHCBP1	0.21541
EBNA-LP	CHD3	0.12912
EBNA-LP	ADCYAP1R1	0.10074
EBNA-LP	CRYGC	0.32846
EBNA-LP	EFCAB4A	0.19147
EBNA-LP	ERBB2	0.034195
EBNA-LP	HTATIP2	0.2061
EBNA-LP	STEAP3	0.24026
EBNA-LP	TXLNA	0.35201
EBNA-LP	POLRMT	0.31152
EBNA-LP	BCAS2	0.33412
EBNA-LP	TMED2	0.25438
EBNA-LP	RAB3IL1	0.36402
EBNA-LP	CCNC	0.13687
EBNA-LP	C1orf94	0.43184
EBNA-LP	CASP6	0.136
EBNA-LP	VCL	0.14166
EBNA-LP	PCDHB13	0.1636
EBNA-LP	CETN2	0.36087
EBNA-LP	CAMSAP2	0.38155
EBNA-LP	ANXA6	0.59178
EBNA-LP	PCDHGC4	0.17939
EBNA-LP	LCP1	0.11603
EBNA-LP	DIXDC1	0.16248
EBNA-LP	SIRPG	0.28605
EBNA-LP	PA2G4	0.14045
EBNA-LP	TINF2	0.22383
EBNA-LP	FBXO44	0.41357
EBNA-LP	ANXA2P2	0.19717
EBNA-LP	CUL4B	0.30017
EBNA-LP	KPNB1	0.18063
EBNA-LP	FEZ1	0.25099
EBNA-LP	CBX4	0.17171
EBNA-LP	FKBP9	0.1845
EBNA-LP	NDC80	0.21671
EBNA-LP	ATP10D	0.21833
EBNA-LP	SMG5	0.28717
EBNA-LP	STK38L	0.11078
EBNA-LP	ACTN2	0.20327
EBNA-LP	COL3A1	0.17022
EBNA-LP	GGA1	0.30263
EBNA-LP	LRCH4	0.40199
EBNA-LP	EFNA1	0.18365
EBNA-LP	ATF5	0.15039
EBNA-LP	CNOT7	0.11393
EBNA-LP	SEPN1	0.34671
EBNA-LP	BMP1	0.012221
EBNA-LP	IFIT3	0.30057
EBNA-LP	PRSS2	0.48665
EBNA-LP	STAT2	0.27688
EBNA-LP	PRDM5	0.18159
EBNA-LP	PSRC1	0.23132
EBNA-LP	REEP1	0.31509
EBNA-LP	PIFO	0.23403
EBNA-LP	METTL9	0.43184
EBNA-LP	CD59	0.30848
EBNA-LP	NCKAP1L	0.11849
EBNA-LP	LIMS1	0.26899
EBNA-LP	ZNF219	0.11394
EBNA-LP	ACTG1	0.14385
EBNA-LP	NCKAP1	0.22624
EBNA-LP	EIF4A2	0.29921
EBNA-LP	PNRC2	0.2831
EBNA-LP	PDE4DIP	0.31006
EBNA-LP	RAB27A	0.13208
EBNA-LP	SESTD1	0.30351
EBNA-LP	FKBP4	0.1385

EBNA-LP	KANK2	0.28604
EBNA-LP	RPL5	0.20618
EBNA-LP	TECPR2	0.43184
EBNA-LP	CDCA5	0.25704
EBNA-LP	STAU2	0.35286
EBNA-LP	SIRT1	0.014044
EBNA-LP	NOS3	0.037045
EBNA-LP	PITPNM3	0.55968
EBNA-LP	SEPSECS	0.25423
EBNA-LP	HEMK1	0.048944
EBNA-LP	VIL1	0.25613
EBNA-LP	TNIP2	0.1127
EBNA-LP	ADCY6	0.062632
EBNA-LP	S100A7L2	0.17789
EBNA-LP	RNF126	0.36476
EBNA-LP	STX11	0.3617
EBNA-LP	FSTL3	0.15885
EBNA-LP	ELK4	0.17057
EBNA-LP	WEE1	0.11975
EBNA-LP	PSMA6	0.10655
EBNA-LP	PCDHA3	0.16848
EBNA-LP	MUC5B	0.34653
EBNA-LP	CIB3	0.2099
EBNA-LP	CD44	0.1507
EBNA-LP	PPID	0.38346
EBNA-LP	SMNDC1	0.37562
EBNA-LP	AATF	0.13667
EBNA-LP	TFF2	0.29815
EBNA-LP	NEDD9	0.2284
EBNA-LP	CNOT1	0.26689
EBNA-LP	PTPN9	0.17566
EBNA-LP	ATF7	0.097549
EBNA-LP	GUCY2C	0.1179
EBNA-LP	HPCA	0.19742
EBNA-LP	SMC5	0.24713
EBNA-LP	ZRANB1	0.19483
EBNA-LP	DNASE1L2	0.16649
EBNA-LP	PTPRO	0.087895
EBNA-LP	KCNJ4	0.36331
EBNA-LP	ANK3	0.13072
EBNA-LP	SYPL1	0.15152
EBNA-LP	DDX6	0.2778
EBNA-LP	APOBEC1	0.15958
EBNA-LP	ERLIN1	0.34666
EBNA-LP	NLRP3	0.18915
EBNA-LP	ZNF473	0.22851
EBNA-LP	RAD9B	0.3686
EBNA-LP	AHSA1	0.3299
EBNA-LP	GTF2A2	0.16768
EBNA-LP	VSIG4	0.36478
EBNA-LP	TOP1	0.16417
EBNA-LP	DNMT3B	0.15685
EBNA-LP	RAC3	0.15354
EBNA-LP	IGF2R	0.23165
EBNA-LP	SORCS1	0.37014
EBNA-LP	PYURF	0.35712
EBNA-LP	HRG	0.10893
EBNA-LP	ORC3	0.27506
EBNA-LP	SCYL3	0.33347
EBNA-LP	CCL18	0.32171
EBNA-LP	ARPC3	0.27969
EBNA-LP	NUAK1	0.19883
EBNA-LP	TXN	0.089522
EBNA-LP	C14orf166	0.20413
EBNA-LP	CDC25C	0.043617
EBNA-LP	PIK3R4	0.20113
EBNA-LP	XRCC1	0.28488
EBNA-LP	CETN1	0.49102

EBNA-LP	UBQLN4	0.29585
EBNA-LP	KCNJ9	0.35809
EBNA-LP	LPP	0.26113
EBNA-LP	ZBTB25	0.13913
EBNA-LP	BCOR	0.17301
EBNA-LP	TTBK2	0.25099
EBNA-LP	MPHOSPH10	0.24086
EBNA-LP	MLLT10	0.13507
EBNA-LP	PFKFB2	0.14375
EBNA-LP	CUL3	0.14445
EBNA-LP	NTNG1	0.34172
EBNA-LP	MC1R	0.17241
EBNA-LP	COG7	0.26681
EBNA-LP	PROCR	0.308
EBNA-LP	ERC2	0.3079
EBNA-LP	ATP1B2	0.27575
EBNA-LP	ZSWIM7	0.29405
EBNA-LP	C2CD4D	0.023671
EBNA-LP	MAP2K2	0.052877
EBNA-LP	HLA-A	0.1779
EBNA-LP	GNB2	0.22121
EBNA-LP	HSPB9	0.36399
EBNA-LP	PRMT7	0.030636
EBNA-LP	PSMC6	0.12994
EBNA-LP	NDFIP1	0.15492
EBNA-LP	STK10	0.22996
EBNA-LP	ANXA13	0.18707
EBNA-LP	HTRA2	0.16618
EBNA-LP	NUP98	0.17551
EBNA-LP	ANK2	0.058978
EBNA-LP	NFKBIA	0.066813
EBNA-LP	SOD3	0.18846
EBNA-LP	SEPT6	0.20803
EBNA-LP	MYO5A	0.09069
EBNA-LP	CIC	0.31576
EBNA-LP	SLC1A2	0.02227
EBNA-LP	COMMD8	0.43184
EBNA-LP	IGFBP2	0.25726
EBNA-LP	DUSP2	0.11499
EBNA-LP	CCR5	0.13023
EBNA-LP	TAF8	0.22194
EBNA-LP	CRHR2	0.22259
EBNA-LP	IL1RAPL1	0.20219
EBNA-LP	PBX1	0.049116
EBNA-LP	HPN	0.17042
EBNA-LP	VLDLR	0.19912
EBNA-LP	NUP62	0.095711
EBNA-LP	INPPL1	0.12555
EBNA-LP	TRIP13	0.19837
EBNA-LP	CDH1	0.10159
EBNA-LP	HTT	0.050985
EBNA-LP	DNAJB4	0.31023
EBNA-LP	ADAMTS13	0.26616
EBNA-LP	BCAP31	0.13042
EBNA-LP	DIAPH1	0.1428
EBNA-LP	TEAD1	0.17952
EBNA-LP	SMO	0.016047
EBNA-LP	BCL6	0.0529
EBNA-LP	MAP1LC3B	0.35245
EBNA-LP	USF2	0.1351
EBNA-LP	UBA5	0.18543
EBNA-LP	TTN	0.11396
EBNA-LP	ZDHHC17	0.2869
EBNA-LP	MICU3	0.19742
EBNA-LP	CAPNS2	0.19148
EBNA-LP	AK2	0.020437
EBNA-LP	NR1H3	0.023283
EBNA-LP	ABT1	0.27562

EBNA-LP	NDRG2	0.1959
EBNA-LP	FAM19A3	0.15046
EBNA-LP	TWIST2	0.087883
EBNA-LP	FSTL4	0.20358
EBNA-LP	POLB	0.13429
EBNA-LP	ACTR3	0.10144
EBNA-LP	BACE1	0.066884
EBNA-LP	DCPS	0.18054
EBNA-LP	TUT1	0.24651
EBNA-LP	PSMC5	0.10393
EBNA-LP	NCALD	0.17038
EBNA-LP	MUS81	0.2408
EBNA-LP	PRDM16	0.1441
EBNA-LP	EGF	0.20048
EBNA-LP	RAPGEF2	0.12351
EBNA-LP	TFAM	0.15955
EBNA-LP	TJP1	0.23289
EBNA-LP	CBX1	0.20685
EBNA-LP	HOMER3	0.28924
EBNA-LP	AJUBA	0.078769
EBNA-LP	MIEF1	0.29837
EBNA-LP	TIRAP	0.10244
EBNA-LP	PDK3	0.28678
EBNA-LP	FYN	0.079699
EBNA-LP	PRKAB2	0.11216
EBNA-LP	AK7	0.05772
EBNA-LP	USP26	0.31299
EBNA-LP	PPP1R16B	0.3273
EBNA-LP	HIVEP1	0.2271
EBNA-LP	ELOVL5	0.24292
EBNA-LP	LTBP4	0.44647
EBNA-LP	BLOC1S6	0.16595
EBNA-LP	MOAP1	0.38706
EBNA-LP	RANBP2	0.15312
EBNA-LP	PRMT2	0.22125
EBNA-LP	RPS19	0.21747
EBNA-LP	DCAF16	0.41438
EBNA-LP	SMARCAD1	0.19828
EBNA-LP	CDKN1B	0.097715
EBNA-LP	NPHP4	0.17911
EBNA-LP	GNB2L1	0.056303
EBNA-LP	NUP43	0.21047
EBNA-LP	BRIP1	0.014772
EBNA-LP	PLSCR4	0.54577
EBNA-LP	LMNA	0.17194
EBNA-LP	INSIG1	0.20843
EBNA-LP	CENPF	0.10373
EBNA-LP	MPP7	0.31745
EBNA-LP	VAMP2	0.23277
EBNA-LP	RBM8A	0.27128
EBNA-LP	ALKBH3	0.27142
EBNA-LP	ZFAND6	0.24929
EBNA-LP	SHANK1	0.15834
EBNA-LP	DCP2	0.26199
EBNA-LP	CCNK	0.22615
EBNA-LP	FBXL2	0.35398
EBNA-LP	SCOC	0.28542
EBNA-LP	ALDH9A1	0.030765
EBNA-LP	LAMB3	0.21012
EBNA-LP	MBD4	0.20188
EBNA-LP	RXRB	0.10536
EBNA-LP	FBXO25	0.33784
EBNA-LP	KAT8	0.16169
EBNA-LP	NID1	0.15345
EBNA-LP	MEPE	0.38288
EBNA-LP	GOLGB1	0.3216
EBNA-LP	SETD3	0.010205
EBNA-LP	GALNT3	0.082584

EBNA-LP	HBA1	0.27475
EBNA-LP	ATP2C1	0.044046
EBNA-LP	NXF1	0.29999
EBNA-LP	GMIP	0.27562
EBNA-LP	C1R	0.22257
EBNA-LP	TUBG1	0.19381
EBNA-LP	SLC6A2	0.36389
EBNA-LP	AKR1B10	0.34252
EBNA-LP	NOP2	0.47306
EBNA-LP	DNAJC1	0.20633
EBNA-LP	FCGBP	0.40593
EBNA-LP	ZC3H11A	0.35368
EBNA-LP	TSHZ3	0.073689
EBNA-LP	SERPINC1	0.26722
EBNA-LP	FN1	0.1679
EBNA-LP	ACE	0.064851
EBNA-LP	SRSF2	0.31442
EBNA-LP	RHOT1	0.3286
EBNA-LP	RPS6KB1	0.031969
EBNA-LP	DDX39A	0.26889
EBNA-LP	SAP18	0.26132
EBNA-LP	SAFB	0.26453
EBNA-LP	ASB13	0.3985
EBNA-LP	TEAD4	0.12274
EBNA-LP	MMP25	0.062435
EBNA-LP	SNX9	0.32403
EBNA-LP	NLGN4X	0.16773
EBNA-LP	COL4A3	0.12231
EBNA-LP	CITED2	0.040914
EBNA-LP	CSRP3	0.16465
EBNA-LP	VPS53	0.35285
EBNA-LP	TTI1	0.43184
EBNA-LP	NCBP2	0.17756
EBNA-LP	ZC3H15	0.31579
EBNA-LP	IFRD2	0.40774
EBNA-LP	TNPO1	0.25142
EBNA-LP	LSM1	0.28243
EBNA-LP	COPS4	0.3439
EBNA-LP	ABI3	0.35416
EBNA-LP	UBXN4	0.30209
EBNA-LP	NLRP12	0.23293
EBNA-LP	DNAJC10	0.16085
EBNA-LP	PARN	0.20462
EBNA-LP	FAM175A	0.32759
EBNA-LP	ZCCHC8	0.28039
EBNA-LP	TRAF3	0.085126
EBNA-LP	RYR2	0.15139
EBNA-LP	DLAT	0.3107
EBNA-LP	STRADA	0.066743
EBNA-LP	ACTR5	0.26376
EBNA-LP	AXL	0.099315
EBNA-LP	RAET1E	0.3473
EBNA-LP	SMC6	0.28086
EBNA-LP	PARP9	0.44222
EBNA-LP	OPRM1	0.11887
EBNA-LP	GTPBP4	0.17608
EBNA-LP	CRLF1	0.32956
EBNA-LP	PRF1	0.37776
EBNA-LP	KIAA1109	0.34299
EBNA-LP	CACNA1I	0.288
EBNA-LP	TFF1	0.30918
EBNA-LP	PCDHB3	0.15367
EBNA-LP	RPS6KA4	0.082206
EBNA-LP	PPP1R13L	0.17866
EBNA-LP	HSD17B14	0.30329
EBNA-LP	EXOC4	0.13772
EBNA-LP	SEPT12	0.23579
EBNA-LP	MMP19	0.042126

```
EBNA-LP CUL7	0.2552
EBNA-LP TPT1	0.098427
EBNA-LP CDC16	0.21177
EBNA-LP IL6ST	0.18031
EBNA-LP CDC20	0.15404
EBNA-LP AK4 0.018447
EBNA-LP STRN	0.2648
EBNA-LP NSUN5	0.048481
EBNA-LP MYO1E	0.24208
EBNA-LP SPRY4	0.33461
EBNA-LP ZNF366	0.22664
EBNA-LP DDX20	0.16665
EBNA-LP TEN1	0.32281
EBNA-LP NKX3-1	0.021914
EBNA-LP UBE2V1	0.14184
EBNA-LP METTL18 0.055343
EBNA-LP TRMT10C 0.41528
EBNA-LP OCA2	0.21701
EBNA-LP IGF1R	0.078602
EBNA-LP PLA2G2C 0.14352
EBNA-LP DGKZ	0.20922
EBNA-LP SNRPA1	0.29796
EBNA-LP LAIR1	0.39356
EBNA-LP MICU2	0.20365
EBNA-LP F2RL2	0.24137
EBNA-LP VEGFB	0.1611
EBNA-LP NHLRC1	0.23439
EBNA-LP SOCS2	0.22112
EBNA-LP ZNF423	0.20271
EBNA-LP MZT1	0.3394
EBNA-LP IGF2BP1 0.15709
EBNA-LP RNF144B 0.24145
EBNA-LP PPP1R12C	0.41868
EBNA-LP MCFD2	0.14722
EBNA-LP CCDC28B 0.40571
EBNA-LP MYL12B	0.12628
EBNA-LP EIF3K	0.33008
EBNA-LP PI4KA	0.34322
EBNA-LP SLC25A24	0.17996
EBNA-LP SGCE	0.14729
EBNA-LP MAP3K4	0.17796
EBNA-LP GPR143	0.18597
EBNA-LP CBS 0.067847
EBNA-LP TIMM9	0.22755
EBNA-LP LNX1	0.25502
EBNA-LP PKD2L2	0.19059
EBNA-LP VDAC2	0.19661
EBNA-LP IL13	0.17435
EBNA-LP USP22	0.1439
EBNA-LP SHARPIN 0.18852
EBNA-LP OLFM1	0.36126
EBNA-LP SART3	0.3388
EBNA-LP PRPF40A 0.28403
EBNA-LP LSM4	0.28543
EBNA-LP WNK3	0.20016
EBNA-LP FBXO11	0.20588
EBNA-LP SNRNP70 0.3181
EBNA-LP LDLRAP1 0.14799
EBNA-LP EPHB4	0.21396
EBNA-LP PCSK9	0.095351
EBNA-LP TRA2B	0.3861
EBNA-LP DGKI	0.28562
EBNA-LP HSPB7	0.32042
EBNA-LP RB1 0.014096
EBNA-LP ELANE	0.21283
EBNA-LP RING1	0.15094
EBNA-LP ZNF496	0.13429
EBNA-LP NELFA	0.25484
```

EBNA-LP	ACVR1B	0.066975
EBNA-LP	AP1S2	0.020709
EBNA-LP	TCEB2	0.22387
EBNA-LP	TRIM23	0.079803
EBNA-LP	MYPN	0.30405
EBNA-LP	BRD3	0.27103
EBNA-LP	SETD2	0.21861
EBNA-LP	ZRANB2	0.22097
EBNA-LP	TRIM32	0.14897
EBNA-LP	PLD2	0.06832
EBNA-LP	YWHAH	0.20829
EBNA-LP	HEMGN	0.34247
EBNA-LP	PARP4	0.27861
EBNA-LP	KRT6A	0.30003
EBNA-LP	KLRF2	0.36519
EBNA-LP	PCDHA6	0.16343
EBNA-LP	SERPINB8	0.34839
EBNA-LP	MED28	0.25982
EBNA-LP	PLEC	0.24948
EBNA-LP	FADD	0.1441
EBNA-LP	TULP3	0.26804
EBNA-LP	ARRDC3	0.39393
EBNA-LP	TSC22D4	0.17868
EBNA-LP	RPS6KA5	0.059008
EBNA-LP	TGFBRAP1	0.29799
EBNA-LP	FHOD1	0.27838
EBNA-LP	XRCC4	0.15631
EBNA-LP	ZFC3H1	0.36649
EBNA-LP	TP53BP2	0.24837
EBNA-LP	RNF187	0.22887
EBNA-LP	E2F3	0.1415
EBNA-LP	ZNF711	0.23855
EBNA-LP	PTPN2	0.14769
EBNA-LP	STIP1	0.29173
EBNA-LP	MALT1	0.11647
EBNA-LP	SQSTM1	0.039579
EBNA-LP	SUPT5H	0.14844
EBNA-LP	ELMO2	0.16588
EBNA-LP	ATP6V0A2	0.24377
EBNA-LP	SNCA	0.034691
EBNA-LP	FBXO27	0.40094
EBNA-LP	EMR3	0.12231
EBNA-LP	CARD10	0.34572
EBNA-LP	UBE3C	0.24517
EBNA-LP	RANBP6	0.35801
EBNA-LP	MCIDAS	0.23006
EBNA-LP	S100A8	0.23583
EBNA-LP	THAP11	0.25298
EBNA-LP	MT-ND3	0.30775
EBNA-LP	CD70	0.23978
EBNA-LP	IL2RG	0.25911
EBNA-LP	DUSP26	0.10257
EBNA-LP	NID2	0.62533
EBNA-LP	SYP	0.13585
EBNA-LP	F2R	0.050408
EBNA-LP	CCNB1	0.068815
EBNA-LP	CADM1	0.13326
EBNA-LP	DIS3L	0.32796
EBNA-LP	NELL1	0.43187
EBNA-LP	DIRAS3	0.27685
EBNA-LP	TICRR	0.30164
EBNA-LP	SULT1C3	0.018496
EBNA-LP	FAM84B	0.40593
EBNA-LP	SLX1A	0.27633
EBNA-LP	ZNF365	0.35658
EBNA-LP	DEDD	0.24666
EBNA-LP	ZHX1	0.12777
EBNA-LP	ANXA1	0.085563

EBNA-LP	ytzL	0.12453
EBNA-LP	FAF2	0.36052
EBNA-LP	CDKN2B	0.12584
EBNA-LP	PFDN5	0.24897
EBNA-LP	WDR96	0.037054
EBNA-LP	ZMYND11	0.18647
EBNA-LP	CLDN7	0.20807
EBNA-LP	KRAS	0.11977
EBNA-LP	PTPRE	0.1289
EBNA-LP	C3	0.17003
EBNA-LP	PCDHB16	0.1636
EBNA-LP	PTPRK	0.071412
EBNA-LP	VAV1	0.052348
EBNA-LP	SNTN	0.19749
EBNA-LP	SIRT3	0.24113
EBNA-LP	ADA	0.057174
EBNA-LP	DNER	0.43159
EBNA-LP	FBXO17	0.40094
EBNA-LP	COL17A1	0.26689
EBNA-LP	PRKAR1A	0.18637
EBNA-LP	EIF6	0.39423
EBNA-LP	CD72	0.27088
EBNA-LP	VSNL1	0.2099
EBNA-LP	FBXL14	0.39613
EBNA-LP	PCDHGA6	0.17939
EBNA-LP	CFL1	0.16143
EBNA-LP	MBD1	0.14787
EBNA-LP	MKL2	0.24146
EBNA-LP	SCNN1G	0.25892
EBNA-LP	POU2F1	0.14847
EBNA-LP	SLC25A10	0.19859
EBNA-LP	ATP8A1	0.25669
EBNA-LP	HMG20A	0.18406
EBNA-LP	TLX1	0.15129
EBNA-LP	IL17RA	0.29047
EBNA-LP	CDK4	0.069002
EBNA-LP	UBE2V2	0.23424
EBNA-LP	PACSIN2	0.017128
EBNA-LP	EFCAB2	0.2099
EBNA-LP	DSCAM	0.26336
EBNA-LP	NUCB1	0.18288
EBNA-LP	SAFB2	0.34845
EBNA-LP	CYTH1	0.238
EBNA-LP	HEYL	0.080481
EBNA-LP	ELOVL6	0.25952
EBNA-LP	PPARGC1A	0.094892
EBNA-LP	NRXN1	0.2222
EBNA-LP	PEF1	0.6549
EBNA-LP	OFD1	0.21219
EBNA-LP	PARK2	0.012581
EBNA-LP	CD22	0.34682
EBNA-LP	RBM15B	0.27357
EBNA-LP	SHOX	0.17143
EBNA-LP	NOXA1	0.30535
EBNA-LP	DUX4L9	0.16005
EBNA-LP	SP1	0.026606
EBNA-LP	ZWINT	0.23019
EBNA-LP	NAPG	0.32067
EBNA-LP	ZFYVE26	0.31238
EBNA-LP	RBBP8	0.19548
EBNA-LP	MAX	0.12595
EBNA-LP	ANXA3	0.11624
EBNA-LP	FBXL8	0.43184
EBNA-LP	SNRPE	0.23587
EBNA-LP	CREBBP	0.027599
EBNA-LP	CGN	0.35506
EBNA-LP	XIAP	0.20801
EBNA-LP	GCHFR	0.222

EBNA-LP	PLA2G16	0.17689
EBNA-LP	TGFB2	0.011699
EBNA-LP	EZR	0.18187
EBNA-LP	COL16A1	0.3165
EBNA-LP	RAD17	0.2441
EBNA-LP	FBL	0.38847
EBNA-LP	TTYH1	0.15471
EBNA-LP	CNNM3	0.37523
EBNA-LP	RASGRP2	0.11352
EBNA-LP	TSSK6	0.18956
EBNA-LP	MEGF8	0.52654
EBNA-LP	C10orf82	0.038589
EBNA-LP	PDGFB	0.03089
EBNA-LP	PCDHGB5	0.17939
EBNA-LP	CHCHD6	0.40325
EBNA-LP	CDH13	0.072071
EBNA-LP	RNF168	0.14876
EBNA-LP	ATXN1	0.21844
EBNA-LP	FKBP9L	0.19197
EBNA-LP	TRIM25	0.077608
EBNA-LP	GAP43	0.25911
EBNA-LP	SLC32A1	0.044442
EBNA-LP	TTYH2	0.33939
EBNA-LP	BAK1	0.055391
EBNA-LP	BEND7	0.43184
EBNA-LP	CHP1	0.21118
EBNA-LP	PRICKLE3	0.34466
EBNA-LP	PAF1	0.20456
EBNA-LP	REEP5	0.3816
EBNA-LP	ANTXR2	0.29553
EBNA-LP	MAP4K2	0.14136
EBNA-LP	NFATC1	0.10919
EBNA-LP	CYFIP2	0.32801
EBNA-LP	CSTF2	0.31408
EBNA-LP	TNK2	0.15586
EBNA-LP	CAMTA2	0.20145
EBNA-LP	MAPK3	0.037146
EBNA-LP	ERAP1	0.17401
EBNA-LP	UBE2E1	0.16428
EBNA-LP	APTX	0.12257
EBNA-LP	VPS52	0.36392
EBNA-LP	MYL12A	0.2066
EBNA-LP	CACNG2	0.21653
EBNA-LP	DAP3	0.20352
EBNA-LP	ZGPAT	0.13886
EBNA-LP	RASSF3	0.33111
EBNA-LP	CFTR	0.15483
EBNA-LP	RAB3IP	0.26274
EBNA-LP	SPG7	0.14422
EBNA-LP	GNAQ	0.077987
EBNA-LP	CTSH	0.12415
EBNA-LP	XPO5	0.28278
EBNA-LP	ABCD2	0.26812
EBNA-LP	CD86	0.18154
EBNA-LP	EGFL6	0.16034
EBNA-LP	KMT2E	0.18928
EBNA-LP	IMP4	0.29206
EBNA-LP	PRPF31	0.29672
EBNA-LP	SYMPK	0.28821
EBNA-LP	CUL5	0.18548
EBNA-LP	CNST	0.36749
EBNA-LP	COL6A2	0.25499
EBNA-LP	FOXG1	0.053549
EBNA-LP	TRAPPC10	0.35769
EBNA-LP	LIPE	0.13812
EBNA-LP	HAP1	0.27357
EBNA-LP	ATP6V1C1	0.2698
EBNA-LP	PSMB7	0.10787

EBNA-LP	RXRA	0.022674
EBNA-LP	PIK3R1	0.016546
EBNA-LP	TGM2	0.25868
EBNA-LP	RPLP0	0.23771
EBNA-LP	NR3C1	0.046498
EBNA-LP	BAG1	0.20423
EBNA-LP	MAPK10	0.1197
EBNA-LP	EPM2A	0.069428
EBNA-LP	FASN	0.20525
EBNA-LP	DLL4	0.098265
EBNA-LP	SEPT7	0.088016
EBNA-LP	MPZL1	0.23992
EBNA-LP	TTYH3	0.12189
EBNA-LP	BRCA2	0.076872
EBNA-LP	LCMT1	0.45024
EBNA-LP	TRIM72	0.059571
EBNA-LP	COL1A1	0.073887
EBNA-LP	EID2	0.22503
EBNA-LP	ELOVL7	0.27279
EBNA-LP	GPR98	0.3076
EBNA-LP	PDS5B	0.28116
EBNA-LP	CRY1	0.11617
EBNA-LP	NUMBL	0.36568
EBNA-LP	UBA3	0.14526
EBNA-LP	ACAA2	0.31843
EBNA-LP	DLG4	0.048258
EBNA-LP	LSM6	0.26842
EBNA-LP	MBIP	0.33002
EBNA-LP	E2F4	0.077445
EBNA-LP	MICU1	0.53755
EBNA-LP	CALU	0.53535
EBNA-LP	EIF2S1	0.28589
EBNA-LP	TBRG1	0.3194
EBNA-LP	ELF2	0.15478
EBNA-LP	ATP1B1	0.24553
EBNA-LP	CEP63	0.30812
EBNA-LP	ZIC3	0.093557
EBNA-LP	ISCU	0.094251
EBNA-LP	RIPK1	0.052352
EBNA-LP	TICAM1	0.091104
EBNA-LP	AP2B1	0.18302
EBNA-LP	KIRREL3	0.35242
EBNA-LP	GPSM2	0.23074
EBNA-LP	PSMB5	0.10279
EBNA-LP	ATXN2	0.19646
EBNA-LP	LILRB4	0.29422
EBNA-LP	XPC	0.2073
EBNA-LP	CD8A	0.16028
EBNA-LP	SSX2	0.30666
EBNA-LP	DCK	0.05535
EBNA-LP	LENG8	0.43184
EBNA-LP	PRKRA	0.28345
EBNA-LP	KLK8	0.31819
EBNA-LP	PTPN13	0.12707
EBNA-LP	PPP3CA	0.20463
EBNA-LP	STRADB	0.12681
EBNA-LP	MTUS2	0.36792
EBNA-LP	ANXA8L2	0.17398
EBNA-LP	FBLN2	0.19403
EBNA-LP	BCL2A1	0.38157
EBNA-LP	MTTP	0.012178
EBNA-LP	HAND2	0.060828
EBNA-LP	PMEL	0.27607
EBNA-LP	TACR1	0.083251
EBNA-LP	MYOZ2	0.34232
EBNA-LP	VCP	0.11064
EBNA-LP	APIP	0.1926
EBNA-LP	USP28	0.25001

EBNA-LP	PPP1CA	0.1031
EBNA-LP	ATF6	0.071578
EBNA-LP	ITK	0.12465
EBNA-LP	HOXC13	0.15933
EBNA-LP	GADD45G	0.2895
EBNA-LP	SIX1	0.032184
EBNA-LP	BTG1	0.21482
EBNA-LP	SVOP	0.023362
EBNA-LP	CCDC88A	0.010382
EBNA-LP	MCTP1	0.18708
EBNA-LP	BEND2	0.022978
EBNA-LP	PRKX	0.14953
EBNA-LP	MOCS3	0.20024
EBNA-LP	CCNG1	0.26096
EBNA-LP	VSTM2L	0.43184
EBNA-LP	RFX6	0.22103
EBNA-LP	ZFYVE28	0.28911
EBNA-LP	FCGR2C	0.36601
EBNA-LP	AKAP6	0.17671
EBNA-LP	BZRAP1	0.37902
EBNA-LP	EIF4B	0.36663
EBNA-LP	ANXA10	0.19895
EBNA-LP	PCDHGB3	0.17939
EBNA-LP	GLS2	0.25948
EBNA-LP	RNF40	0.19539
EBNA-LP	SOX5	0.10827
EBNA-LP	POP1	0.27053
EBNA-LP	PVR	0.16275
EBNA-LP	ARSA	0.056937
EBNA-LP	NCOR2	0.1928
EBNA-LP	LRP2	0.22171
EBNA-LP	VMP1	0.25441
EBNA-LP	CAPN2	0.35157
EBNA-LP	PAFAH1B3	0.2519
EBNA-LP	KRT8	0.27822
EBNA-LP	SLC23A1	0.22665
EBNA-LP	OCLN	0.13503
EBNA-LP	SIRPB1	0.35819
EBNA-LP	CPSF7	0.34423
EBNA-LP	ABCG5	0.18913
EBNA-LP	UNC119	0.35897
EBNA-LP	ABL1	0.053072
EBNA-LP	DGCR8	0.31886
EBNA-LP	CLIC1	0.22205
EBNA-LP	MKRN3	0.24898
EBNA-LP	TTC1	0.37818
EBNA-LP	SUPT4H1	0.098597
EBNA-LP	SREBF2	0.071737
EBNA-LP	HNRNPC	0.2941
EBNA-LP	FAM188B	0.11523
EBNA-LP	TRIM37	0.19498
EBNA-LP	GRN	0.27139
EBNA-LP	PABPC4	0.28865
EBNA-LP	SLC22A11	0.31087
EBNA-LP	VPS33A	0.25058
EBNA-LP	TRIM5	0.18284
EBNA-LP	MMP17	0.051712
EBNA-LP	ANTXR1	0.25956
EBNA-LP	TNFRSF11A	0.15612
EBNA-LP	NPLOC4	0.24625
EBNA-LP	AK8	0.059704
EBNA-LP	NAA10	0.33081
EBNA-LP	RGS2	0.19585
EBNA-LP	CTSL	0.23084
EBNA-LP	MON1B	0.43184
EBNA-LP	TIFA	0.38858
EBNA-LP	NUCB2	0.46458
EBNA-LP	TF	0.2193

```
	EBNA-LP CBR4	0.34664
	EBNA-LP RAD9A	0.1653
	EBNA-LP CBX6	0.28686
	EBNA-LP MSX2	0.043665
	EBNA-LP PKDREJ	0.16409
	EBNA-LP PPP1R13B	0.32071
	EBNA-LP DCC 0.18322
	EBNA-LP ITGB1	0.016395
	EBNA-LP LRSAM1	0.21988
	EBNA-LP SOX1	0.18237
	EBNA-LP HELZ2	0.18297
	EBNA-LP UNQ6126/PRO20091	0.011222
	EBNA-LP EED 0.23167
	EBNA-LP ASGR1	0.31655
	EBNA-LP NPC2	0.22249
	EBNA-LP TLL2	0.053497
	EBNA-LP HOXA9	0.17279
	EBNA-LP MAGEA11 0.37519
	EBNA-LP SH3RF2	0.2955
	EBNA-LP RAP1GAP 0.2245
	EBNA-LP USH1C	0.20493
	EBNA-LP PCDH19	0.17939
	EBNA-LP RNPS1	0.28238
	EBNA-LP SH3GLB1 0.16578
	EBNA-LP CDON	0.17263
	EBNA-LP BCL2L14 0.23173
	EBNA-LP GLRX3	0.012817
	EBNA-LP PINX1	0.17491
	EBNA-LP PSMD5	0.18444
	EBNA-LP PEX11A	0.29219
	EBNA-LP RIPK3	0.10072
	EBNA-LP S100A6	0.41097
	EBNA-LP RP1L1	0.34518
	EBNA-LP EPHB1	0.12315
	EBNA-LP AOC1	0.10153
	EBNA-LP STK16	0.16682
	EBNA-LP VHL 0.14309
	EBNA-LP FAT2	0.14126
	EBNA-LP FBXW2	0.37642
	EBNA-LP PLCG2	0.09345
	EBNA-LP SMARCD3 0.092759
	EBNA-LP RNF20	0.1475
	EBNA-LP RHBG	0.067689
	EBNA-LP ZFP36	0.10342
	EBNA-LP PPP2R1A 0.092375
	EBNA-LP IPO13	0.32684
	EBNA-LP AFG3L2	0.11788
	EBNA-LP HENMT1	0.10579
	EBNA-LP EPB42	0.23941
	EBNA-LP WDR6	0.33513
	EBNA-LP DDC 0.17577
	EBNA-LP TERF2IP 0.14185
	EBNA-LP SPRED2	0.25026
	EBNA-LP TLR4	0.018902
	EBNA-LP PKHD1	0.1996
	EBNA-LP RNF19B	0.29865
	EBNA-LP IKBKAP	0.1359
	EBNA-LP MED11	0.2617
	EBNA-LP NUBP1	0.016556
	EBNA-LP RABGEF1 0.26858
	EBNA-LP LRP8	0.36566
	EBNA-LP ZEB1	0.083736
	EBNA-LP NOX5	0.12082
	EBNA-LP SYNPO	0.25909
	EBNA-LP IGFLR1	0.39356
	EBNA-LP CASP2	0.16851
	EBNA-LP CT55	0.077606
	EBNA-LP RFC5	0.16869
```

EBNA-LP	PPP6R3	0.34847
EBNA-LP	KRT14	0.19486
EBNA-LP	CBFA2T2	0.13715
EBNA-LP	GABRG2	0.2081
EBNA-LP	NUAK2	0.18904
EBNA-LP	NXT1	0.29243
EBNA-LP	S100P	0.57585
EBNA-LP	CMTR1	0.014509
EBNA-LP	SNAI1	0.11117
EBNA-LP	FLG	0.11362
EBNA-LP	BCL10	0.037444
EBNA-LP	CARD8	0.3141
EBNA-LP	PLK3	0.10285
EBNA-LP	SEMA4D	0.10823
EBNA-LP	SMURF1	0.14054
EBNA-LP	SIX5	0.16477
EBNA-LP	TMEM67	0.25295
EBNA-LP	WASL	0.10972
EBNA-LP	LRP1B	0.17485
EBNA-LP	DCAF7	0.34037
EBNA-LP	SGTA	0.36999
EBNA-LP	RABEP2	0.15882
EBNA-LP	SLC18A1	0.074339
EBNA-LP	XPO6	0.3085
EBNA-LP	TRAK1	0.26236
EBNA-LP	SERTAD1	0.32753
EBNA-LP	PCDHGA5	0.17939
EBNA-LP	RNF103	0.24742
EBNA-LP	FCN2	0.070582
EBNA-LP	CLASP1	0.12239
EBNA-LP	DMD	0.13987
EBNA-LP	E2F2	0.10233
EBNA-LP	LCA5	0.32948
EBNA-LP	INTS4	0.36347
EBNA-LP	DYNLRB1	0.28207
EBNA-LP	GYPC	0.36973
EBNA-LP	SEPT1	0.32999
EBNA-LP	EGLN2	0.22364
EBNA-LP	RAF1	0.034947
EBNA-LP	PRDX6	0.33155
EBNA-LP	WT1	0.0326
EBNA-LP	YWHAZ	0.15769
EBNA-LP	CHAF1A	0.29802
EBNA-LP	BTF3	0.31754
EBNA-LP	KIAA0319L	0.39355
EBNA-LP	SLC22A1	0.21953
EBNA-LP	CAMKK2	0.062999
EBNA-LP	DAZ1	0.36749
EBNA-LP	SEPT9	0.24523
EBNA-LP	PPP2R5C	0.24761
EBNA-LP	TAF9	0.12537
EBNA-LP	PTCRA	0.39351
EBNA-LP	SETD1B	0.32929
EBNA-LP	RIMS2	0.29498
EBNA-LP	TP53INP1	0.26203
EBNA-LP	F7	0.076352
EBNA-LP	ELF4	0.1592
EBNA-LP	ECH1	0.25086
EBNA-LP	CPS1	0.07182
EBNA-LP	C4BPA	0.36198
EBNA-LP	TAPBP	0.13102
EBNA-LP	RAPGEF1	0.26689
EBNA-LP	FEM1B	0.27351
EBNA-LP	ATXN7L3	0.23675
EBNA-LP	FNBP1	0.36511
EBNA-LP	KMT2B	0.15836
EBNA-LP	MCM10	0.27603
EBNA-LP	KCNB1	0.25155

```
	EBNA-LP CASP9	0.2359
	EBNA-LP NR4A2	0.066728
	EBNA-LP VRK1	0.18604
	EBNA-LP LTN1	0.30054
	EBNA-LP MTOR	0.04115
	EBNA-LP PTTG1	0.20426
	EBNA-LP PLAU	0.21609
	EBNA-LP DZIP1	0.24093
	EBNA-LP CLUAP1	0.38697
	EBNA-LP MADD	0.28762
	EBNA-LP NUP160	0.23686
	EBNA-LP NUP50	0.2616
	EBNA-LP TRIO	0.10382
	EBNA-LP RAB11FIP3	0.32412
	EBNA-LP MOB1B	0.2579
	EBNA-LP EFCAB4B	0.53245
	EBNA-LP APP	0.028098
	EBNA-LP CDHR1	0.13856
	EBNA-LP HPX	0.21606
	EBNA-LP UCHL1	0.14961
	EBNA-LP GAB2	0.15767
	EBNA-LP ALKBH8	0.35257
	EBNA-LP PARD6G	0.31908
	EBNA-LP CAMK1	0.10277
	EBNA-LP SYBU	0.36672
	EBNA-LP PCDHB8	0.17939
	EBNA-LP SKAP1	0.040732
	EBNA-LP NOC4L	0.32693
	EBNA-LP YOD1	0.25251
	EBNA-LP GABPA	0.1162
	EBNA-LP ARL6IP1	0.40812
	EBNA-LP TMPRSS6	0.32881
	EBNA-LP BLOC1S4	0.32612
	EBNA-LP THAP3	0.36734
	EBNA-LP S100A7A	0.17254
	EBNA-LP ZNF256	0.13462
	EBNA-LP MUC17	0.27571
	EBNA-LP AP2A1	0.17756
	EBNA-LP TDGF1	0.090533
	EBNA-LP ZYX	0.21656
	EBNA-LP EFCAB3	0.2099
	EBNA-LP ANKZF1	0.35366
	EBNA-LP PFDN2	0.29953
	EBNA-LP PRRG2	0.18548
	EBNA-LP STK11	0.051032
	EBNA-LP DLGAP2	0.30541
	EBNA-LP MMP24	0.053232
	EBNA-LP SMOC2	0.14451
	EBNA-LP STXBP3	0.20191
	EBNA-LP TJP3	0.34771
	EBNA-LP SALL1	0.056366
	EBNA-LP SERPING1	0.25719
	EBNA-LP HIP1R	0.25269
	EBNA-LP THOC1	0.25956
	EBNA-LP YTHDC2	0.33149
	EBNA-LP RAD51B	0.27773
	EBNA-LP CCT2	0.23444
	EBNA-LP FOSL1	0.094503
	EBNA-LP SETD6	0.3881
	EBNA-LP MTPAP	0.23202
	EBNA-LP CASR	0.25731
	EBNA-LP YWHAE	0.21598
	EBNA-LP EDEM3	0.11341
	EBNA-LP TPO	0.18335
	EBNA-LP LAMP1	0.028389
	EBNA-LP NDUFA13	0.1887
	EBNA-LP CHGB	0.40223
	EBNA-LP ATP2A2	0.022435
```

EBNA-LP	PRKD3	0.093043
EBNA-LP	DTNA	0.46864
EBNA-LP	REPS2	0.69272
EBNA-LP	IMP3	0.31411
EBNA-LP	OPRK1	0.22694
EBNA-LP	SH3BP4	0.37162
EBNA-LP	MVP	0.29375
EBNA-LP	SASS6	0.33673
EBNA-LP	PPP3R1	0.20996
EBNA-LP	RRAGB	0.24283
EBNA-LP	CCDC50	0.41868
EBNA-LP	CDH24	0.15733
EBNA-LP	TRIM44	0.35366
EBNA-LP	ARFGEF1	0.14642
EBNA-LP	PPEF2	0.35956
EBNA-LP	EFEMP2	0.6365
EBNA-LP	DVL3	0.18215
EBNA-LP	POU5F1	0.041088
EBNA-LP	IFNA2	0.24045
EBNA-LP	MAP4	0.19105
EBNA-LP	MYLK	0.14629
EBNA-LP	RLBP1	0.014573
EBNA-LP	ZC3H13	0.35368
EBNA-LP	ALX1	0.13934
EBNA-LP	SART1	0.26645
EBNA-LP	MT-CO1	0.16128
EBNA-LP	AUNIP	0.34817
EBNA-LP	TANK	0.30688
EBNA-LP	HERC5	0.20901
EBNA-LP	DLEU1	0.43184
EBNA-LP	EIF3L	0.39437
EBNA-LP	STARD13	0.29359
EBNA-LP	ATCAY	0.26754
EBNA-LP	MAP1B	0.052668
EBNA-LP	HNRNPH1	0.27178
EBNA-LP	PFN2	0.2977
EBNA-LP	USP33	0.16667
EBNA-LP	ATP2A1	0.20572
EBNA-LP	CCS	0.19859
EBNA-LP	POGZ	0.24246
EBNA-LP	SIK2	0.15394
EBNA-LP	PCDH15	0.082985
EBNA-LP	TCEA1	0.16283
EBNA-LP	TRRAP	0.22387
EBNA-LP	FZD4	0.093685
EBNA-LP	RPA2	0.14074
EBNA-LP	EXOSC4	0.20964
EBNA-LP	JUP	0.022789
EBNA-LP	RNF10	0.1981
EBNA-LP	SCAMP5	0.2539
EBNA-LP	HIST3H3	0.21684
EBNA-LP	GSK3B	0.015235
EBNA-LP	CAB39	0.24766
EBNA-LP	WLS	0.39622
EBNA-LP	NOC2L	0.20479
EBNA-LP	ATP6V0A4	0.17451
EBNA-LP	PPP2R1B	0.41872
EBNA-LP	ITGA2	0.28082
EBNA-LP	CEP250	0.16268
EBNA-LP	EIF3D	0.38652
EBNA-LP	PDGFRL	0.13706
EBNA-LP	IFT52	0.057936
EBNA-LP	CXorf40A	0.43184
EBNA-LP	BCL2L1	0.030873
EBNA-LP	MYL3	0.13611
EBNA-LP	AGXT	0.22142
EBNA-LP	BAG6	0.18112
EBNA-LP	IL15	0.15257

EBNA-LP	CHD9	0.20403
EBNA-LP	NEUROD1	0.035154
EBNA-LP	CSF2RB	0.28565
EBNA-LP	CD47	0.25054
EBNA-LP	CHMP3	0.23906
EBNA-LP	FOS	0.039675
EBNA-LP	PBK	0.27569
EBNA-LP	RFC4	0.17202
EBNA-LP	HOOK2	0.24335
EBNA-LP	FOXO3	0.018356
EBNA-LP	ZC3H12A	0.24116
EBNA-LP	CCP110	0.31693
EBNA-LP	SMG7	0.27656
EBNA-LP	CD200	0.33107
EBNA-LP	LSM2	0.16488
EBNA-LP	AP3M1	0.32209
EBNA-LP	DYNC1I1	0.20355
EBNA-LP	ZNF703	0.16555
EBNA-LP	CAGE1	0.023073
EBNA-LP	NFYB	0.16496
EBNA-LP	SH3GL3	0.3334
EBNA-LP	DMBT1	0.16215
EBNA-LP	MINK1	0.13185
EBNA-LP	PKN2	0.094714
EBNA-LP	MECOM	0.073913
EBNA-LP	LONP1	0.18507
EBNA-LP	PDCL3	0.36995
EBNA-LP	JAM3	0.19101
EBNA-LP	ASB16	0.3985
EBNA-LP	MLH3	0.19452
EBNA-LP	ARHGAP17	0.2883
EBNA-LP	PKD2L1	0.35542
EBNA-LP	MOB1A	0.36844
EBNA-LP	ADRB3	0.12314
EBNA-LP	TCEB1	0.21118
EBNA-LP	VPS4B	0.17248
EBNA-LP	FEZ2	0.37918
EBNA-LP	NFYA	0.16738
EBNA-LP	RNF111	0.15758
EBNA-LP	DROSHA	0.22678
EBNA-LP	CD2BP2	0.32282
EBNA-LP	NINL	0.16987
EBNA-LP	CD2	0.24929
EBNA-LP	ACOT8	0.17586
EBNA-LP	TPPP3	0.18874
EBNA-LP	NCOA6	0.13104
EBNA-LP	SMOC1	0.15994
EBNA-LP	SPPL2B	0.20348
EBNA-LP	ATR	0.16426
EBNA-LP	DMPK	0.061765
EBNA-LP	HTN1	0.37089
EBNA-LP	TRIM3	0.27732
EBNA-LP	EDC4	0.23209
EBNA-LP	NSUN5P2	0.087967
EBNA-LP	PAX9	0.26823
EBNA-LP	ZNF408	0.21956
EBNA-LP	BTN3A1	0.35705
EBNA-LP	PLEKHA1	0.13062
EBNA-LP	DAB2	0.13726
EBNA-LP	HGS	0.18101
EBNA-LP	ARHGEF11	0.11459
EBNA-LP	SLC9A3R1	0.10403
EBNA-LP	TOPBP1	0.25948
EBNA-LP	BOLL	0.37045
EBNA-LP	MYLK2	0.07519
EBNA-LP	RYBP	0.20389
EBNA-LP	EHD3	0.39039
EBNA-LP	TUBB4A	0.091912

EBNA-LP	DYRK1A	0.11774
EBNA-LP	TGM1	0.35561
EBNA-LP	JAK1	0.18555
EBNA-LP	IGF2	0.15412
EBNA-LP	HSPA2	0.30165
EBNA-LP	FAM64A	0.42025
EBNA-LP	KIAA2018	0.15144
EBNA-LP	OCIAD1	0.39236
EBNA-LP	BCR	0.15634
EBNA-LP	SRCIN1	0.17431
EBNA-LP	TMEM8B	0.27549
EBNA-LP	MMP11	0.0573
EBNA-LP	SLC14A2	0.33212
EBNA-LP	ZZEF1	0.158
EBNA-LP	AGER	0.17305
EBNA-LP	RICTOR	0.26015
EBNA-LP	MYL10	0.20365
EBNA-LP	PDGFRA	0.037477
EBNA-LP	LY96	0.21404
EBNA-LP	UBE2M	0.23548
EBNA-LP	NF2	0.11355
EBNA-LP	MICALL1	0.3429
EBNA-LP	CSK	0.15923
EBNA-LP	SMAD2	0.034613
EBNA-LP	UTF1	0.23939
EBNA-LP	EXOSC8	0.30337
EBNA-LP	IL4R	0.23347
EBNA-LP	NR3C2	0.14993
EBNA-LP	RNF4	0.036439
EBNA-LP	KRT15	0.33017
EBNA-LP	DUT	0.24216
EBNA-LP	NTN1	0.27431
EBNA-LP	ZXDA	0.13382
EBNA-LP	MAGEC1	0.43184
EBNA-LP	POU2F3	0.13011
EBNA-LP	EIF4G3	0.014502
EBNA-LP	P4HTM	0.15364
EBNA-LP	EFCAB11	0.2099
EBNA-LP	MT1G	0.21378
EBNA-LP	DLGAP5	0.24381
EBNA-LP	ATRX	0.19395
EBNA-LP	FKBP2	0.33598
EBNA-LP	RNF6	0.18185
EBNA-LP	TAF9B	0.19948
EBNA-LP	DTL	0.23618
EBNA-LP	UVRAG	0.31559
EBNA-LP	COPB1	0.16407
EBNA-LP	KIF6	0.32149
EBNA-LP	THBS2	0.59374
EBNA-LP	OIT3	0.16662
EBNA-LP	EPS15L1	0.1361
EBNA-LP	PELI2	0.27529
EBNA-LP	FAP	0.23882
EBNA-LP	SLIT1	0.075176
EBNA-LP	NAF1	0.31513
EBNA-LP	MLLT3	0.27754
EBNA-LP	PRAME	0.23233
EBNA-LP	CALM1	0.23847
EBNA-LP	CRYAB	0.12719
EBNA-LP	SMARCA5	0.11098
EBNA-LP	ASAP2	0.12199
EBNA-LP	EIF4E2	0.43219
EBNA-LP	STAMBPL1	0.34414
EBNA-LP	ACVR2B	0.05411
EBNA-LP	DBNL	0.23727
EBNA-LP	FGF3	0.24799
EBNA-LP	E2F1	0.065786
EBNA-LP	STRN4	0.38463

```
EBNA-LP JUN 0.011973
EBNA-LP AVPR1A  0.12597
EBNA-LP RRP8    0.24948
EBNA-LP HPRT1   0.12001
EBNA-LP YWHAG   0.27881
EBNA-LP NPM3    0.34203
EBNA-LP DNASE1  0.26557
EBNA-LP RNF216  0.20834
EBNA-LP MAGI2   0.13323
EBNA-LP EPOR    0.25725
EBNA-LP ARAP1   0.048877
EBNA-LP EIF2A   0.25168
EBNA-LP KCTD5   0.32665
EBNA-LP CASP1   0.11829
EBNA-LP ZRANB3  0.12658
EBNA-LP TFAP2C  0.069273
EBNA-LP FLT3    0.15086
EBNA-LP EXOC2   0.35759
EBNA-LP ATG12   0.34109
EBNA-LP RARG    0.051651
EBNA-LP KIF1B   0.28398
EBNA-LP CAV3    0.072401
EBNA-LP SMARCB1 0.1341
EBNA-LP CDHR3   0.17939
EBNA-LP YEATS2  0.30223
EBNA-LP KPNA5   0.32369
EBNA-LP ARHGAP10    0.1514
EBNA-LP S100A4  0.61217
EBNA-LP MMP21   0.053497
EBNA-LP ERCC5   0.17407
EBNA-LP ABCG2   0.2711
EBNA-LP GUCA1B  0.17966
EBNA-LP NAPA    0.24256
EBNA-LP TNPO2   0.32682
EBNA-LP COL4A1  0.27221
EBNA-LP DNAJC21 0.31996
EBNA-LP S100A12 0.34761
EBNA-LP FYCO1   0.40185
EBNA-LP RGS16   0.3933
EBNA-LP WNT1    0.058242
EBNA-LP RAB9B   0.32719
EBNA-LP C19orf80    0.41869
EBNA-LP RASGRP3 0.076003
EBNA-LP PHC2    0.27345
EBNA-LP MTMR2   0.083289
EBNA-LP BMPR2   0.059784
EBNA-LP IKBKE   0.13929
EBNA-LP CDKN2A  0.028604
EBNA-LP DDX39B  0.21051
EBNA-LP WNT3A   0.028953
EBNA-LP BAZ2B   0.26482
EBNA-LP CASP8   0.064707
EBNA-LP PRSS12  0.045824
EBNA-LP NEFL    0.23197
EBNA-LP FTSJ1   0.015087
EBNA-LP KCNJ6   0.28652
EBNA-LP MYH2    0.22231
EBNA-LP FKBP7   0.178
EBNA-LP LRRFIP1 0.22772
EBNA-LP PPP2R3C 0.16219
EBNA-LP TNFRSF6B    0.37113
EBNA-LP SMAD6   0.092328
EBNA-LP TROAP   0.40522
EBNA-LP MT1H    0.27753
EBNA-LP RAPGEF4 0.12711
EBNA-LP PSIP1   0.22468
EBNA-LP SSX2IP  0.35731
EBNA-LP ACD 0.19234
```

EBNA-LP	DPP4	0.20027
EBNA-LP	RERE	0.14679
EBNA-LP	SPSB4	0.3985
EBNA-LP	EIF3E	0.28966
EBNA-LP	EIF4E	0.2689
EBNA-LP	DSCC1	0.286
EBNA-LP	RAMP1	0.1789
EBNA-LP	BBC3	0.14791
EBNA-LP	HSPBP1	0.35968
EBNA-LP	SDC2	0.1961
EBNA-LP	AK6	0.4139
EBNA-LP	HRAS	0.052285
EBNA-LP	EMILIN2	0.37691
EBNA-LP	AIM2	0.14953
EBNA-LP	CLTCL1	0.28977
EBNA-LP	NPHP1	0.19095
EBNA-LP	CHD7	0.088046
EBNA-LP	SLC15A2	0.26566
EBNA-LP	DNMT3A	0.22714
EBNA-LP	ATP6V1F	0.24259
EBNA-LP	OAS3	0.23083
EBNA-LP	INHBA	0.073857
EBNA-LP	CDH2	0.051694
EBNA-LP	RQCD1	0.24705
EBNA-LP	PAG1	0.31471
EBNA-LP	NPB	0.35822
EBNA-LP	PIM3	0.22226
EBNA-LP	ACTN3	0.56153
EBNA-LP	SREBF1	0.020744
EBNA-LP	ANXA4	0.18115
EBNA-LP	ATP5F1	0.33899
EBNA-LP	FBN1	0.56166
EBNA-LP	NR4A1	0.077571
EBNA-LP	RNF182	0.23576
EBNA-LP	MIF4GD	0.37968
EBNA-LP	MAP4K3	0.23647
EBNA-LP	TNNI2	0.311
EBNA-LP	RBKS	0.032933
EBNA-LP	PDPK1	0.0454
EBNA-LP	RAD1	0.21266
EBNA-LP	ATG14	0.35556
EBNA-LP	C12orf63	0.010302
EBNA-LP	F10	0.37697
EBNA-LP	PTK2B	0.020619
EBNA-LP	CAPN1	0.44068
EBNA-LP	PAX3	0.11913
EBNA-LP	UCKL1	0.37732
EBNA-LP	PLXDC1	0.28399
EBNA-LP	HSPB8	0.20466
EBNA-LP	MED15	0.23119
EBNA-LP	DSG1	0.11374
EBNA-LP	CEACAM6	0.35589
EBNA-LP	GSTZ1	0.33697
EBNA-LP	TLE1	0.15339
EBNA-LP	SKIL	0.15575
EBNA-LP	LILRB3	0.32826
EBNA-LP	ZNF3	0.10509
EBNA-LP	ZNF346	0.30507
EBNA-LP	RPSA	0.12905
EBNA-LP	SRPK1	0.12227
EBNA-LP	IPO7	0.2441
EBNA-LP	TRIM24	0.049913
EBNA-LP	CFL2	0.36667
EBNA-LP	ACAA1	0.22013
EBNA-LP	DPM2	0.23916
EBNA-LP	GHRHR	0.11639
EBNA-LP	CX3CR1	0.15749
EBNA-LP	NTMT1	0.037185

EBNA-LP	PCDHA8	0.16848
EBNA-LP	PSMG2	0.33193
EBNA-LP	NDOR1	0.24748
EBNA-LP	PPM1A	0.076781
EBNA-LP	LETMD1	0.30769
EBNA-LP	SYNM	0.27583
EBNA-LP	FOXK2	0.023628
EBNA-LP	PPP2R5E	0.28388
EBNA-LP	SCGN	0.27243
EBNA-LP	SAE1	0.15461
EBNA-LP	TSN	0.38062
EBNA-LP	VPS51	0.32142
EBNA-LP	CELSR3	0.4486
EBNA-LP	CAND1	0.27454
EBNA-LP	FCHSD2	0.43184
EBNA-LP	CATSPER1	0.21112
EBNA-LP	RILP	0.3318
EBNA-LP	ING5	0.20491
EBNA-LP	SENP2	0.2162
EBNA-LP	BAX	0.013822
EBNA-LP	RCOR3	0.30542
EBNA-LP	MAPKAPK5	0.14006
EBNA-LP	LMAN1	0.13163
EBNA-LP	UBE2B	0.10955
EBNA-LP	MRPL48	0.3399
EBNA-LP	SDHA	0.32511
EBNA-LP	NR2C2	0.087836
EBNA-LP	WASF1	0.25233
EBNA-LP	CTIF	0.3965
EBNA-LP	RNF115	0.28304
EBNA-LP	SYNJ2	0.22673
EBNA-LP	KIAA1524	0.38158
EBNA-LP	ZNRF3	0.21856
EBNA-LP	PLA2G2D	0.075881
EBNA-LP	TCEAL5	0.32616
EBNA-LP	PEX14	0.17173
EBNA-LP	CACNA1A	0.057716
EBNA-LP	AGO3	0.30732
EBNA-LP	ADAM10	0.010875
EBNA-LP	RPGRIP1	0.33228
EBNA-LP	SPOCK1	0.16665
EBNA-LP	N6AMT1	0.47852
EBNA-LP	POLR2E	0.13679
EBNA-LP	S100A5	0.14549
EBNA-LP	POLR2J	0.15924
EBNA-LP	CD4	0.091135
EBNA-LP	ARF1	0.12707
EBNA-LP	GEMIN8	0.32461
EBNA-LP	CERS2	0.10207
EBNA-LP	IGFL1	0.40623
EBNA-LP	NFU1	0.059587
EBNA-LP	PEX13	0.28829
EBNA-LP	EMR4P	0.1297
EBNA-LP	IGBP1	0.22705
EBNA-LP	NOD2	0.014167
EBNA-LP	MEOX2	0.15167
EBNA-LP	TFAP4	0.14884
EBNA-LP	SETD8	0.27772
EBNA-LP	MAGEA4	0.39536
EBNA-LP	TAGLN2	0.38507
EBNA-LP	SIRT4	0.47451
EBNA-LP	FYTTD1	0.44206
EBNA-LP	PCDHA4	0.16848
EBNA-LP	CDH15	0.14371
EBNA-LP	COBL	0.037225
EBNA-LP	LDHD	0.35648
EBNA-LP	PRNP	0.10421
EBNA-LP	TESC	0.33064

EBNA-LP	CLIC3	0.27828
EBNA-LP	TAF5	0.061798
EBNA-LP	CHD1	0.18803
EBNA-LP	RBP4	0.15345
EBNA-LP	HOXB9	0.13829
EBNA-LP	SETD7	0.31439
EBNA-LP	PARD3	0.15057
EBNA-LP	FMNL1	0.23849
EBNA-LP	UBE2Q1	0.28278
EBNA-LP	IFNLR1	0.35165
EBNA-LP	EFHB	0.2099
EBNA-LP	LGI1	0.23553
EBNA-LP	NME2	0.10492
EBNA-LP	KIAA0319	0.30945
EBNA-LP	CRY2	0.1369
EBNA-LP	ARL2	0.15982
EBNA-LP	EYS	0.22937
EBNA-LP	MICAL1	0.33912
EBNA-LP	CLVS2	0.045167
EBNA-LP	LSM12	0.032332
EBNA-LP	NDNL2	0.30379
EBNA-LP	ROPN1L	0.36794
EBNA-LP	HOXA1	0.017086
EBNA-LP	OBSCN	0.12695
EBNA-LP	UCN	0.18594
EBNA-LP	PCDHGB7	0.17939
EBNA-LP	FGFR3	0.059813
EBNA-LP	RWDD1	0.43184
EBNA-LP	TFPT	0.27705
EBNA-LP	SMPDL3A	0.32256
EBNA-LP	RHO	0.17669
EBNA-LP	LIG3	0.14787
EBNA-LP	C12orf57	0.048624
EBNA-LP	HSPB6	0.37145
EBNA-LP	ADAMTS4	0.211
EBNA-LP	PJA1	0.29801
EBNA-LP	AHNAK	0.37511
EBNA-LP	CCNF	0.29014
EBNA-LP	HOOK3	0.20551
EBNA-LP	SETD1A	0.31885
EBNA-LP	GLB1	0.17489
EBNA-LP	PAK2	0.032994
EBNA-LP	SMARCC2	0.19268
EBNA-LP	CSNK2B	0.062271
EBNA-LP	HIC1	0.086042
EBNA-LP	FCN3	0.30027
EBNA-LP	PPFIA1	0.38073
EBNA-LP	TAF4	0.090911
EBNA-LP	PPP4R4	0.4031
EBNA-LP	DCLRE1B	0.21592
EBNA-LP	OSBPL1A	0.20587
EBNA-LP	GOPC	0.18077
EBNA-LP	CD82	0.38135
EBNA-LP	TNFRSF1A	0.11652
EBNA-LP	DZIP3	0.33684
EBNA-LP	RALA	0.18535
EBNA-LP	CD226	0.17929
EBNA-LP	MOB4	0.32369
EBNA-LP	BIN3	0.30894
EBNA-LP	POLR2A	0.13907
EBNA-LP	RPH3A	0.35851
EBNA-LP	SPTSSA	0.28275
EBNA-LP	DCP1A	0.23024
EBNA-LP	ADAP1	0.10972
EBNA-LP	AMOTL2	0.39148
EBNA-LP	SMARCA1	0.15831
EBNA-LP	SRI	0.38646
EBNA-LP	SELL	0.26011

EBNA-LP OCM 0.17789
EBNA-LP PPP4R2 0.27606
EBNA-LP RTP3 0.36413
EBNA-LP LRP4 0.34803
EBNA-LP KAT6A 0.12104
EBNA-LP PPP1CB 0.038947
EBNA-LP CDK8 0.073433
EBNA-LP PMF1 0.18617
EBNA-LP BDKRB2 0.15215
EBNA-LP KPNA2 0.25008
EBNA-LP EVI5L 0.33394
EBNA-LP NEK2 0.080134
EBNA-LP RAD54L 0.22131
EBNA-LP TSC2 0.081208
EBNA-LP SLC7A8 0.11405
EBNA-LP PSG5 0.40584
EBNA-LP PIK3R2 0.20247
EBNA-LP MAP3K14 0.11601
EBNA-LP BMX 0.1271
EBNA-LP INADL 0.30907
EBNA-LP SEL1L 0.35025
EBNA-LP GNL3L 0.26431
EBNA-LP PTPN12 0.14844
EBNA-LP PLA2G4B 0.040571
EBNA-LP FEN1 0.086516
EBNA-LP TMSB4X 0.25207
EBNA-LP METTL3 0.024902
EBNA-LP PCDHB12 0.16848
EBNA-LP VEGFA 0.012704
EBNA-LP FLG2 0.05766
EBNA-LP CUL2 0.2594
EBNA-LP CCT3 0.26315
EBNA-LP SRSF7 0.28749
EBNA-LP MAP3K7 0.03235
EBNA-LP SPATA31E1 0.18933
EBNA-LP CABP5 0.18207
EBNA-LP SLIT2 0.10679
EBNA-LP PKD1 0.050977
EBNA-LP HERC2 0.16654
EBNA-LP TBC1D5 0.29962
EBNA-LP SNED1 0.19733
EBNA-LP AKAP9 0.19592
EBNA-LP MIB1 0.22017
EBNA-LP PASK 0.18704
EBNA-LP BLM 0.064711
EBNA-LP ARNT 0.073006
EBNA-LP SYNE3 0.2692
EBNA-LP DOCK4 0.23328
EBNA-LP NCLN 0.25879
EBNA-LP AMY2A 0.021451
EBNA-LP PANK3 0.039248
EBNA-LP HNRNPUL1 0.27886
EBNA-LP GPRASP1 0.41868
EBNA-LP MAGEE1 0.30637
EBNA-LP CNPY2 0.37023
EBNA-LP NSRP1 0.33953
EBNA-LP SFI1 0.331
EBNA-LP MATN2 0.5286
EBNA-LP BCAR1 0.10978
EBNA-LP PSMD9 0.14682
EBNA-LP AXIN1 0.032171
EBNA-LP SIRT6 0.25902
EBNA-LP STK4 0.062006
EBNA-LP GPD2 0.12815
EBNA-LP DHFR 0.016696
EBNA-LP PCDHA10 0.16343
EBNA-LP CIB1 0.47452
EBNA-LP IRAK2 0.085751

EBNA-LP	MAPK14	0.026824
EBNA-LP	PRRG4	0.17474
EBNA-LP	ANKRD44	0.43184
EBNA-LP	KIF13A	0.15842
EBNA-LP	FNTA	0.28524
EBNA-LP	EIF5A2	0.22908
EBNA-LP	MAPRE3	0.31746
EBNA-LP	CLCN3	0.19
EBNA-LP	DNAJB2	0.28663
EBNA-LP	DNMT1	0.19576
EBNA-LP	PCDHA11	0.16848
EBNA-LP	HDAC10	0.13704
EBNA-LP	RYR1	0.046107
EBNA-LP	PCDHB7	0.1739
EBNA-LP	TNK1	0.26656
EBNA-LP	PHF8	0.16752
EBNA-LP	GYS1	0.17536
EBNA-LP	PPP2R3A	0.50834
EBNA-LP	GSPT1	0.264
EBNA-LP	DSC1	0.17979
EBNA-LP	AKAP3	0.40966
EBNA-LP	PRPF19	0.22742
EBNA-LP	CCDC155	0.083912
EBNA-LP	EIF2B4	0.25157
EBNA-LP	NCF1	0.074296
EBNA-LP	TSSK4	0.18336
EBNA-LP	USE1	0.26941
EBNA-LP	DLL1	0.33925
EBNA-LP	TAF2	0.20535
EBNA-LP	ACTN1	0.29946
EBNA-LP	CABP2	0.17448
EBNA-LP	VDR	0.094319
EBNA-LP	PEX11B	0.31657
EBNA-LP	PMP22	0.33436
EBNA-LP	RAB6A	0.23339
EBNA-LP	STK38	0.1123
EBNA-LP	TNKS2	0.18351
EBNA-LP	RCHY1	0.17068
EBNA-LP	CCND1	0.062933
EBNA-LP	CD164	0.24607
EBNA-LP	TBC1D15	0.31086
EBNA-LP	AOC3	0.18409
EBNA-LP	FUS	0.30645
EBNA-LP	BLOC1S1	0.25868
EBNA-LP	DCTN3	0.13943
EBNA-LP	NPHP3	0.21356
EBNA-LP	USP46	0.32044
EBNA-LP	UBD	0.24601
EBNA-LP	MYH10	0.11856
EBNA-LP	CDT1	0.25863
EBNA-LP	SYNE1	0.21759
EBNA-LP	FBXO3	0.35937
EBNA-LP	LRP6	0.02044
EBNA-LP	EXOC8	0.17583
EBNA-LP	ITGA1	0.23171
EBNA-LP	SVEP1	0.17868
EBNA-LP	NOS1	0.15028
EBNA-LP	EHD4	0.36382
EBNA-LP	APBA3	0.27955
EBNA-LP	RYR3	0.092905
EBNA-LP	SCUBE1	0.10755
EBNA-LP	PCDHB14	0.1636
EBNA-LP	LCAT	0.24543
EBNA-LP	OBFC1	0.17637
EBNA-LP	TWIST1	0.046195
EBNA-LP	E2F5	0.10647
EBNA-LP	CSNK1E	0.13781
EBNA-LP	MEF2A	0.067605

EBNA-LP	PNPT1	0.13431
EBNA-LP	NPRL2	0.28909
EBNA-LP	UPF3B	0.32895
EBNA-LP	AKAP13	0.13582
EBNA-LP	ATG2A	0.43184
EBNA-LP	ZC3H7B	0.27761
EBNA-LP	LZTFL1	0.38678
EBNA-LP	TLN1	0.078784
EBNA-LP	BTRC	0.14265
EBNA-LP	PCDHGA11	0.17939
EBNA-LP	RHBDL1	0.2757
EBNA-LP	HNF1B	0.060963
EBNA-LP	IRF4	0.03824
EBNA-LP	TFAP2A	0.019761
EBNA-LP	UBE2D1	0.14318
EBNA-LP	GCKR	0.22573
EBNA-LP	MYO6	0.10856
EBNA-LP	LCMT2	0.48307
EBNA-LP	TGFBR1	0.016714
EBNA-LP	USP8	0.16606
EBNA-LP	FIG4	0.17107
EBNA-LP	TNFSF14	0.19842
EBNA-LP	CDC6	0.13081
EBNA-LP	CDH3	0.18238
EBNA-LP	PLAT	0.22714
EBNA-LP	TAX1BP3	0.28302
EBNA-LP	SNX4	0.33351
EBNA-LP	NEGR1	0.34691
EBNA-LP	HTR6	0.27561
EBNA-LP	SEPT11	0.29271
EBNA-LP	PNLIPRP2	0.041813
EBNA-LP	DLGAP1	0.30905
EBNA-LP	CCND3	0.10627
EBNA-LP	MFI2	0.25421
EBNA-LP	FLNA	0.083596
EBNA-LP	HIST1H4A	0.17443
EBNA-LP	CTTNBP2NL	0.41724
EBNA-LP	SERPINF2	0.19126
EBNA-LP	FBXL5	0.30139
EBNA-LP	SGSM3	0.29323
EBNA-LP	DUOX1	0.20819
EBNA-LP	F3	0.10249
EBNA-LP	CECR2	0.31674
EBNA-LP	PMAIP1	0.10714
EBNA-LP	SPINK1	0.35885
EBNA-LP	ABCD1	0.18349
EBNA-LP	DDX3X	0.042016
EBNA-LP	PCDHA2	0.16848
EBNA-LP	SH3GLB2	0.32101
EBNA-LP	CDK16	0.16278
EBNA-LP	RASEF	0.15661
EBNA-LP	DPP7	0.088575
EBNA-LP	PRSS3	0.55657
EBNA-LP	ANXA8	0.16367
EBNA-LP	CCDC169	0.018644
EBNA-LP	RPS6KC1	0.22198
EBNA-LP	MAP1S	0.15417
EBNA-LP	BTK	0.050444
EBNA-LP	CLEC4G	0.39344
EBNA-LP	PPIL3	0.36562
EBNA-LP	CALML5	0.18685
EBNA-LP	LRRC4C	0.39355
EBNA-LP	MYL2	0.49289
EBNA-LP	BAIAP2	0.16588
EBNA-LP	KAT5	0.14889
EBNA-LP	PIWIL1	0.29644
EBNA-LP	ALOX12	0.17256
EBNA-LP	EDEM1	0.43961

EBNA-LP	LRP1	0.19951
EBNA-LP	UFL1	0.2779
EBNA-LP	APBA2	0.31054
EBNA-LP	DHDDS	0.42044
EBNA-LP	MKI67	0.22428
EBNA-LP	JADE1	0.22165
EBNA-LP	MTF1	0.086098
EBNA-LP	FGFR1	0.024739
EBNA-LP	GEMIN2	0.27055
EBNA-LP	SELE	0.17579
EBNA-LP	TONSL	0.27591
EBNA-LP	CACNB4	0.14181
EBNA-LP	RBM12B	0.48723
EBNA-LP	CKS1B	0.311
EBNA-LP	ATP10B	0.35512
EBNA-LP	TIMM23	0.28675
EBNA-LP	DICER1	0.050595
EBNA-LP	HADHB	0.14117
EBNA-LP	APPL1	0.14403
EBNA-LP	VPRBP	0.32568
EBNA-LP	SENP5	0.31364
EBNA-LP	SUV39H1	0.19404
EBNA-LP	COPS6	0.31348
EBNA-LP	SNX2	0.28796
EBNA-LP	MAP2K5	0.13815
EBNA-LP	RNF11	0.24274
EBNA-LP	ITGAM	0.20164
EBNA-LP	CDH17	0.17603
EBNA-LP	CDK11B	0.1506
EBNA-LP	EFCAB9	0.2099
EBNA-LP	PEX1	0.16878
EBNA-LP	GPX8	0.030447
EBNA-LP	NUDT18	0.36131
EBNA-LP	EXOSC2	0.31255
EBNA-LP	ERG	0.1204
EBNA-LP	ZNF622	0.23908
EBNA-LP	MAP2K1	0.017049
EBNA-LP	NFS1	0.23314
EBNA-LP	EEF1E1	0.28402
EBNA-LP	FANCD2	0.33143
EBNA-LP	TNNC2	0.17538
EBNA-LP	ATP5J	0.060637
EBNA-LP	GNA12	0.1681
EBNA-LP	ZC3H14	0.35903
EBNA-LP	MEPCE	0.09501
EBNA-LP	LSM11	0.29093
EBNA-LP	BUB1	0.10983
EBNA-LP	RIT1	0.3171
EBNA-LP	KCNA4	0.26325
EBNA-LP	RIOK3	0.31317
EBNA-LP	DMC1	0.1939
EBNA-LP	ATP5A1	0.19562
EBNA-LP	RAB21	0.28568
EBNA-LP	SGPL1	0.083595
EBNA-LP	ADAR	0.2473
EBNA-LP	OIP5	0.26441
EBNA-LP	GAD1	0.19581
EBNA-LP	WDTC1	0.22742
EBNA-LP	SNX19	0.55364
EBNA-LP	STXBP5	0.063633
EBNA-LP	MAP2K4	0.15069
EBNA-LP	ASPH	0.18403
EBNA-LP	CREB1	0.032579
EBNA-LP	THUMPD2	0.054091
EBNA-LP	OASL	0.037101
EBNA-LP	LIN28B	0.27353
EBNA-LP	TUBGCP2	0.23366
EBNA-LP	HIP1	0.1311

EBNA-LP	MBD3	0.23373
EBNA-LP	ITGA4	0.23744
EBNA-LP	FSTL1	0.16812
EBNA-LP	SRC	0.044819
EBNA-LP	MRE11A	0.1338
EBNA-LP	USP47	0.23986
EBNA-LP	CALR3	0.12923
EBNA-LP	CLPP	0.41489
EBNA-LP	CHORDC1	0.29723
EBNA-LP	SMG6	0.16174
EBNA-LP	ID1	0.10804
EBNA-LP	SOX10	0.047877
EBNA-LP	TRPC1	0.19063
EBNA-LP	TOM1	0.3221
EBNA-LP	PITPNC1	0.39286
EBNA-LP	PDGFRB	0.072749
EBNA-LP	SULF2	0.042159
EBNA-LP	S100A10	0.11201
EBNA-LP	ACVRL1	0.019838
EBNA-LP	KIF2C	0.14113
EBNA-LP	CACNA1B	0.22917
EBNA-LP	SEPT5	0.12834
EBNA-LP	PAX8	0.046846
EBNA-LP	APPBP2	0.33653
EBNA-LP	C1QTNF3	0.24293
EBNA-LP	GAD2	0.18282
EBNA-LP	WNK1	0.21901
EBNA-LP	TCF3	0.029997
EBNA-LP	DACT1	0.16473
EBNA-LP	SV2C	0.044555
EBNA-LP	PCNA	0.071618
EBNA-LP	DDRGK1	0.41925
EBNA-LP	IRF8	0.053051
EBNA-LP	RPS3	0.036795
EBNA-LP	CD274	0.30613
EBNA-LP	ITSN1	0.26124
EBNA-LP	ATP7B	0.096429
EBNA-LP	PTPRD	0.096445
EBNA-LP	KDM4A	0.19762
EBNA-LP	TRAM2	0.37511
EBNA-LP	SPAG9	0.035831
EBNA-LP	CEBPB	0.077906
EBNA-LP	C15orf37	0.012035
EBNA-LP	BRCC3	0.19032
EBNA-LP	BBS1	0.30454
EBNA-LP	SLC22A4	0.23612
EBNA-LP	ETV7	0.17782
EBNA-LP	HNRNPF	0.28127
EBNA-LP	CCDC141	0.43184
EBNA-LP	PCDHGA12	0.17939
EBNA-LP	ELOVL1	0.25052
EBNA-LP	PRDX3	0.14105
EBNA-LP	DST	0.23654
EBNA-LP	HIF1AN	0.16086
EBNA-LP	COL18A1	0.1565
EBNA-LP	MYL6	0.57222
EBNA-LP	COMP	0.47324
EBNA-LP	RNF31	0.20259
EBNA-LP	EIF3G	0.38896
EBNA-LP	MLLT6	0.29524
EBNA-LP	CCDC47	0.56336
EBNA-LP	SNAP47	0.3689
EBNA-LP	EXOSC7	0.29646
EBNA-LP	LIMD1	0.13388
EBNA-LP	MX1	0.18312
EBNA-LP	SATB1	0.10488
EBNA-LP	RIN1	0.33315
EBNA-LP	RCOR1	0.2012

EBNA-LP	ANXA7	0.43831
EBNA-LP	SON	0.27331
EBNA-LP	MAP3K7CL	0.37575
EBNA-LP	RNF139	0.20414
EBNA-LP	PRC1	0.12172
EBNA-LP	CDH26	0.17939
EBNA-LP	DGUOK	0.033864
EBNA-LP	FRK	0.23875
EBNA-LP	CHP2	0.51665
EBNA-LP	ADNP	0.071068
EBNA-LP	TOX3	0.27529
EBNA-LP	PSMA7	0.089421
EBNA-LP	ZFP36L1	0.10829
EBNA-LP	CD97	0.10997
EBNA-LP	ABCC2	0.30678
EBNA-LP	PTPRB	0.14558
EBNA-LP	POM121C	0.30383
EBNA-LP	MUC1	0.2009
EBNA-LP	RANBP3	0.29187
EBNA-LP	CTNNA1	0.10737
EBNA-LP	VBP1	0.25953
EBNA-LP	UBE2L6	0.28079
EBNA-LP	ZHX3	0.14002
EBNA-LP	RSPO1	0.24758
EBNA-LP	GOLPH3	0.28823
EBNA-LP	IRF6	0.065815
EBNA-LP	PLN	0.25673
EBNA-LP	CDK5R1	0.087555
EBNA-LP	SUB1	0.23931
EBNA-LP	ANP32A	0.24346
EBNA-LP	LTBP2	0.15775
EBNA-LP	PPM1F	0.12441
EBNA-LP	SEPT4	0.082682
EBNA-LP	AKT3	0.11141
EBNA-LP	TEAD2	0.15651
EBNA-LP	CAPN13	0.09102
EBNA-LP	PLOD3	0.2249
EBNA-LP	IKBKB	0.038881
EBNA-LP	TOLLIP	0.30465
EBNA-LP	EIF4EBP2	0.32608
EBNA-LP	LRP12	0.32075
EBNA-LP	NECAB2	0.72705
EBNA-LP	ELF3	0.10614
EBNA-LP	PROS1	0.082513
EBNA-LP	OTX2	0.055986
EBNA-LP	OGT	0.12718
EBNA-LP	GTF3C4	0.30796
EBNA-LP	PBRM1	0.26823
EBNA-LP	NCOR1	0.064484
EBNA-LP	APITD1	0.19141
EBNA-LP	HGF	0.1564
EBNA-LP	DNAJB9	0.2844
EBNA-LP	SLBP	0.25437
EBNA-LP	USH2A	0.22487
EBNA-LP	MMP16	0.046156
EBNA-LP	PHF2	0.19596
EBNA-LP	PODXL2	0.35772
EBNA-LP	APLP1	0.10141
EBNA-LP	RFC2	0.17735
EBNA-LP	TYROBP	0.20671
EBNA-LP	CANX	0.30358
EBNA-LP	HNF1A	0.052276
EBNA-LP	RHOT2	0.3389
EBNA-LP	DOCK9	0.20694
EBNA-LP	RTKN	0.23678
EBNA-LP	EYA3	0.084747
EBNA-LP	KLHL3	0.22354
EBNA-LP	EHMT1	0.27522

EBNA-LP KDR 0.044006
EBNA-LP SNAP23 0.29023
EBNA-LP MMP15 0.37666
EBNA-LP MKNK1 0.18686
EBNA-LP TTC8 0.30228
EBNA-LP PDE6D 0.44329
EBNA-LP ASCC3 0.20477
EBNA-LP PPP2R5B 0.23887
EBNA-LP GADD45A 0.26281
EBNA-LP PLCB3 0.11365
EBNA-LP SNX15 0.10955
EBNA-LP LCP2 0.33069
EBNA-LP ORAI2 0.40592
EBNA-LP SUSD1 0.19742
EBNA-LP APBA1 0.23179
EBNA-LP DKK1 0.14391
EBNA-LP ANXA11 0.38111
EBNA-LP SPINK9 0.37008
EBNA-LP ITM2C 0.28019
EBNA-LP HNF4A 0.019961
EBNA-LP USP54 0.40517
EBNA-LP PPP3CB 0.19903
EBNA-LP TAB2 0.13739
EBNA-LP SNRPB 0.19998
EBNA-LP KRT19 0.26464
EBNA-LP PCBD2 0.23626
EBNA-LP S100G 0.19221
EBNA-LP PRKAA1 0.027651
EBNA-LP ATG7 0.16646
EBNA-LP MDM4 0.20331
EBNA-LP SCYL2 0.17196
EBNA-LP NCKIPSD 0.37577
EBNA-LP SPINK7 0.38174
EBNA-LP NQO1 0.29515
EBNA-LP NFATC2 0.094569
EBNA-LP ACSS1 0.15038
EBNA-LP ACACB 0.21664
EBNA-LP RBM39 0.29033
EBNA-LP IGF2BP3 0.28607
EBNA-LP TRIM27 0.11686
EBNA-LP CSPG5 0.14592
EBNA-LP MC3R 0.25403
EBNA-LP ANXA5 0.054812
EBNA-LP PAX2 0.033002
EBNA-LP CNOT4 0.20918
EBNA-LP CDK12 0.093011
EBNA-LP DLG2 0.22611
EBNA-LP ZNF136 0.22953
EBNA-LP MAST1 0.17777
EBNA-LP SV2B 0.043288
EBNA-LP MAU2 0.31663
EBNA-LP SH3PXD2B 0.21283
EBNA-LP ALDH1A3 0.029809
EBNA-LP PCSK4 0.017425
EBNA-LP IQGAP1 0.20293
EBNA-LP ACRV1 0.01224
EBNA-LP HIRA 0.16756
EBNA-LP ATP6V0C 0.21783
EBNA-LP STAP2 0.18137
EBNA-LP EPAS1 0.054279
EBNA-LP CCNA2 0.17638
EBNA-LP FZD1 0.076203
EBNA-LP ELAVL1 0.22088
EBNA-LP RAD51 0.11171
EBNA-LP BMI1 0.10882
EBNA-LP OPTN 0.28742
EBNA-LP TMED1 0.24759
EBNA-LP RAB9A 0.16922

EBNA-LP	TIMP1	0.1989
EBNA-LP	MATN3	0.68135
EBNA-LP	SKA1	0.21039
EBNA-LP	EFCAB8	0.2099
EBNA-LP	ATL2	0.19724
EBNA-LP	NR2F2	0.059914
EBNA-LP	RAP1A	0.20491
EBNA-LP	MAST2	0.15466
EBNA-LP	SH3BP2	0.26508
EBNA-LP	YEATS4	0.18204
EBNA-LP	SFN	0.14886
EBNA-LP	MMP13	0.04446
EBNA-LP	FBN3	0.21272
EBNA-LP	RPL27A	0.30443
EBNA-LP	FAM47C	0.065732
EBNA-LP	MDC1	0.29233
EBNA-LP	PGRMC1	0.2995
EBNA-LP	NHP2L1	0.31949
EBNA-LP	SH3KBP1	0.33349
EBNA-LP	AMOTL1	0.35364
EBNA-LP	MYLIP	0.192
EBNA-LP	TENC1	0.21205
EBNA-LP	STK3	0.10578
EBNA-LP	DLGAP4	0.33008
EBNA-LP	TOMM70A	0.27327
EBNA-LP	TCERG1	0.26279
EBNA-LP	PCDH8	0.12817
EBNA-LP	UBE4A	0.34369
EBNA-LP	LRRK2	0.045601
EBNA-LP	TSC1	0.11083
EBNA-LP	CPNE6	0.19078
EBNA-LP	CD276	0.22183
EBNA-LP	RNF41	0.18098
EBNA-LP	RASSF2	0.35242
EBNA-LP	TJAP1	0.35831
EBNA-LP	ETV6	0.18125
EBNA-LP	SCHIP1	0.36362
EBNA-LP	RET	0.22069
EBNA-LP	OS9	0.32374
EBNA-LP	ARHGAP26	0.15502
EBNA-LP	COL1A2	0.17583
EBNA-LP	LSM7	0.27474
EBNA-LP	SRY	0.14716
EBNA-LP	JAM2	0.31917
EBNA-LP	MAFB	0.13371
EBNA-LP	DGKA	0.095258
EBNA-LP	ALOX5	0.17551
EBNA-LP	SOD1	0.036887
EBNA-LP	WASH1	0.28516
EBNA-LP	SPRED1	0.1352
EBNA-LP	PARP1	0.11635
EBNA-LP	CLCF1	0.24276
EBNA-LP	CHEK1	0.11249
EBNA-LP	FCRL4	0.39356
EBNA-LP	SF3B4	0.3672
EBNA-LP	EPS8	0.35296
EBNA-LP	PATL1	0.24826
EBNA-LP	TRAF5	0.21133
EBNA-LP	PARP10	0.36373
EBNA-LP	NSMCE4A	0.35515
EBNA-LP	SNTA1	0.22456
EBNA-LP	ATP11A	0.21863
EBNA-LP	IRF2	0.10103
EBNA-LP	CDH22	0.17939
EBNA-LP	THBS4	0.11633
EBNA-LP	FBXL3	0.30149
EBNA-LP	DDX1	0.14032
EBNA-LP	MC4R	0.22322

EBNA-LP	HEY2	0.018732
EBNA-LP	CDHR4	0.17939
EBNA-LP	CHD1L	0.27605
EBNA-LP	SLC3A2	0.1913
EBNA-LP	UBQLN1	0.26329
EBNA-LP	USP13	0.16934
EBNA-LP	SLC6A3	0.15676
EBNA-LP	CALCR	0.164
EBNA-LP	PTGFRN	0.34666
EBNA-LP	EIF4EBP3	0.33586
EBNA-LP	PTPN11	0.061253
EBNA-LP	CPSF1	0.31992
EBNA-LP	MAGED2	0.010748
EBNA-LP	MS4A3	0.37002
EBNA-LP	HOXA5	0.056139
EBNA-LP	HCFC1	0.087888
EBNA-LP	FLNB	0.23693
EBNA-LP	TMEM66	0.3109
EBNA-LP	SMAD4	0.025288
EBNA-LP	PICK1	0.1394
EBNA-LP	MSLN	0.32512
EBNA-LP	RAB11FIP5	0.1915
EBNA-LP	SUZ12	0.2016
EBNA-LP	CDH23	0.30492
EBNA-LP	KIT	0.040292
EBNA-LP	STATH	0.33038
EBNA-LP	RAB11FIP4	0.55251
EBNA-LP	RLF	0.20927
EBNA-LP	KRT18	0.20208
EBNA-LP	FGF13	0.15343
EBNA-LP	NTM	0.30828
EBNA-LP	PDCD6IP	0.28591
EBNA-LP	GAB1	0.16926
EBNA-LP	HSFX1	0.18797
EBNA-LP	RARRES2	0.30519
EBNA-LP	BMPR1A	0.046039
EBNA-LP	MYCBPAP	0.30305
EBNA-LP	MEF2D	0.13559
EBNA-LP	PSMA2	0.10786
EBNA-LP	ASXL1	0.18245
EBNA-LP	APBB2	0.17474
EBNA-LP	TP53BP1	0.15358
EBNA-LP	MAP1LC3A	0.27788
EBNA-LP	UBXN1	0.25377
EBNA-LP	CNRIP1	0.02017
EBNA-LP	PRPF3	0.33077
EBNA-LP	ATP6V1H	0.23197
EBNA-LP	CDH12	0.16032
EBNA-LP	PHYH	0.1657
EBNA-LP	JMJD6	0.13532
EBNA-LP	RRAGA	0.20887
EBNA-LP	HSPB2	0.32181
EBNA-LP	EIF3A	0.23458
EBNA-LP	MGARP	0.23363
EBNA-LP	NFKB2	0.082837
EBNA-LP	UBE2I	0.079329
EBNA-LP	CASP7	0.17351
EBNA-LP	H2AFX	0.16991
EBNA-LP	RHOU	0.13318
EBNA-LP	SSRP1	0.2542
EBNA-LP	SUMO3	0.38157
EBNA-LP	TLR2	0.048032
EBNA-LP	ATF3	0.1377
EBNA-LP	SOS2	0.1751
EBNA-LP	SYNE2	0.15853
EBNA-LP	DPYSL2	0.057641
EBNA-LP	BCL2L2	0.32665
EBNA-LP	SUV39H2	0.26048

EBNA-LP	IL10RA	0.34563
EBNA-LP	CLIC5	0.18494
EBNA-LP	CTBP1	0.060257
EBNA-LP	UBE2F	0.27437
EBNA-LP	AGTR2	0.13355
EBNA-LP	IFIH1	0.14845
EBNA-LP	C1orf64	0.069205
EBNA-LP	IER3	0.26561
EBNA-LP	CNKSR1	0.22822
EBNA-LP	LDHAL6B	0.34552
EBNA-LP	MBOAT7	0.33189
EBNA-LP	FOCAD	0.38232
EBNA-LP	LOX	0.22312
EBNA-LP	ELP4	0.23452
EBNA-LP	ELTD1	0.1377
EBNA-LP	CAV2	0.13081
EBNA-LP	FXR2	0.48927
EBNA-LP	POLR2B	0.15005
EBNA-LP	VANGL1	0.39355
EBNA-LP	MPP1	0.31176
EBNA-LP	TK2	0.028944
EBNA-LP	TRIP6	0.22352
EBNA-LP	PKP4	0.24108
EBNA-LP	COMMD4	0.41868
EBNA-LP	ENO1	0.05408
EBNA-LP	AGGF1	0.29804
EBNA-LP	ORC5	0.22419
EBNA-LP	CLU	0.079658
EBNA-LP	PPIB	0.3345
EBNA-LP	PRRG1	0.18548
EBNA-LP	NRG1	0.052295
EBNA-LP	A1CF	0.25973
EBNA-LP	NIFK	0.33281
EBNA-LP	ATXN7	0.23973
EBNA-LP	UBA6	0.22899
EBNA-LP	TDP2	0.21431
EBNA-LP	SUDS3	0.31256
EBNA-LP	CHCHD3	0.27904
EBNA-LP	PRKACB	0.070903
EBNA-LP	LPHN1	0.24753
EBNA-LP	ETNK2	0.026937
EBNA-LP	WIPF2	0.39355
EBNA-LP	SNX5	0.096567
EBNA-LP	MCM2	0.13161
EBNA-LP	PLCE1	0.17214
EBNA-LP	CD163	0.34233
EBNA-LP	IL10RB	0.33715
EBNA-LP	DYNLL1	0.1596
EBNA-LP	PIDD	0.34221
EBNA-LP	ADAMTSL4	0.25534
EBNA-LP	MYOT	0.31563
EBNA-LP	PDCD1	0.31636
EBNA-LP	HNRNPLL	0.35895
EBNA-LP	IL12B	0.045853
EBNA-LP	WDR48	0.27219
EBNA-LP	PDCD11	0.32606
EBNA-LP	G3BP1	0.25963
EBNA-LP	RASSF1	0.16553
EBNA-LP	NELFCD	0.27171
EBNA-LP	EID1	0.2352
EBNA-LP	PCDHB4	0.14275
EBNA-LP	IGHMBP2	0.077451
EBNA-LP	SNX3	0.3323
EBNA-LP	OLA1	0.27754
EBNA-LP	ATP6V1D	0.26377
EBNA-LP	ABL2	0.14894
EBNA-LP	DOCK3	0.36982
EBNA-LP	STX6	0.22275

EBNA-LP	DDX11	0.039844
EBNA-LP	MAK	0.084103
EBNA-LP	CYLD	0.086891
EBNA-LP	GP6	0.30022
EBNA-LP	SNW1	0.21577
EBNA-LP	AC3.5	0.045862
EBNA-LP	PPL	0.28384
EBNA-LP	KLK3	0.42019
EBNA-LP	SGIP1	0.18023
EBNA-LP	WAC	0.25335
EBNA-LP	TTR	0.36019
EBNA-LP	ATP2B4	0.22347
EBNA-LP	BBS7	0.28698
EBNA-LP	CRYAA	0.16738
EBNA-LP	NEDD4L	0.14672
EBNA-LP	PCDH9	0.17082
EBNA-LP	PPP6R2	0.35774
EBNA-LP	ITPKB	0.029545
EBNA-LP	HPCAL1	0.2099
EBNA-LP	SLC36A1	0.016911
EBNA-LP	MIS12	0.26105
EBNA-LP	ARF4	0.24282
EBNA-LP	ZNF274	0.13764
EBNA-LP	DAPK1	0.21207
EBNA-LP	SOX6	0.076297
EBNA-LP	ITGA3	0.29547
EBNA-LP	COG3	0.2189
EBNA-LP	TNIK	0.14291
EBNA-LP	BRD7	0.13279
EBNA-LP	RNASE1	0.35166
EBNA-LP	C2orf81	0.040809
EBNA-LP	NSF	0.17267
EBNA-LP	RBBP7	0.19697
EBNA-LP	PTRF	0.20252
EBNA-LP	ARIH1	0.27418
EBNA-LP	ECM1	0.19391
EBNA-LP	KLC2	0.29117
EBNA-LP	BEGAIN	0.40592
EBNA-LP	ARF6	0.12846
EBNA-LP	FAM84A	0.018289
EBNA-LP	PAAF1	0.38256
EBNA-LP	SNX21	0.12516
EBNA-LP	KISS1R	0.21051
EBNA-LP	DNASE1L3	0.13416
EBNA-LP	RIPK2	0.034705
EBNA-LP	SNRPD3	0.19731
EBNA-LP	CLEC2A	0.38697
EBNA-LP	CTSB	0.09315
EBNA-LP	ILKAP	0.22528
EBNA-LP	CLIC4	0.23155
EBNA-LP	PUS3	0.030198
EBNA-LP	RAB14	0.086283
EBNA-LP	STX12	0.24557
EBNA-LP	SAMHD1	0.26617
EBNA-LP	ACTB	0.10214
EBNA-LP	EIF4G1	0.29502
EBNA-LP	SLC22A5	0.23793
EBNA-LP	KIF3A	0.1695
EBNA-LP	SCNN1A	0.27133
EBNA-LP	CGREF1	0.17207
EBNA-LP	CHAF1B	0.21313
EBNA-LP	RUSC1	0.26909
EBNA-LP	NPNT	0.14959
EBNA-LP	EZH1	0.010379
EBNA-LP	PEX12	0.27547
EBNA-LP	FLOT1	0.26878
EBNA-LP	FAM49B	0.047777
EBNA-LP	BNIP2	0.42661

EBNA-LP	GABARAPL2	0.30159
EBNA-LP	FAM199X	0.015276
EBNA-LP	PPIH	0.29813
EBNA-LP	PRKDC	0.074907
EBNA-LP	SULF1	0.041886
EBNA-LP	JAG2	0.36475
EBNA-LP	RGR	0.29979
EBNA-LP	MYRIP	0.28628
EBNA-LP	CTTN	0.35732
EBNA-LP	C1QA	0.35232
EBNA-LP	PPP1R16A	0.40593
EBNA-LP	EFCAB7	0.2099
EBNA-LP	TACC1	0.24717
EBNA-LP	CREB3	0.049862
EBNA-LP	LTBP3	0.1548
EBNA-LP	PLK4	0.17592
EBNA-LP	SKIV2L2	0.22132
EBNA-LP	ADRB2	0.053771
EBNA-LP	OLAH	0.07509
EBNA-LP	DDX18	0.34654
EBNA-LP	ATP2B1	0.24801
EBNA-LP	POLR1B	0.20134
EBNA-LP	VIMP	0.14832
EBNA-LP	KMT2D	0.23022
EBNA-LP	CEP97	0.37417
EBNA-LP	PTPRU	0.13822
EBNA-LP	TPCN2	0.11353
EBNA-LP	RUNX1T1	0.14484
EBNA-LP	CENPI	0.2221
EBNA-LP	SHC3	0.30865
EBNA-LP	LGALS3	0.26001
EBNA-LP	FCGR2B	0.33234
EBNA-LP	DYSF	0.079386
EBNA-LP	IST1	0.47833
EBNA-LP	MAP4K5	0.22233
EBNA-LP	NR5A2	0.046277
EBNA-LP	FCHO2	0.28621
EBNA-LP	TIPRL	0.40161
EBNA-LP	SKP2	0.24054
EBNA-LP	PLAA	0.38145
EBNA-LP	NR1H4	0.043552
EBNA-LP	DUSP15	0.12891
EBNA-LP	MIB2	0.2468
EBNA-LP	EHD2	0.35247
EBNA-LP	PVRL2	0.14435
EBNA-LP	COPS5	0.22186
EBNA-LP	FGGY	0.07612
EBNA-LP	RNF135	0.28016
EBNA-LP	CCNE2	0.17337
EBNA-LP	C5AR2	0.29307
EBNA-LP	CADPS	0.52394
EBNA-LP	JUND	0.13297
EBNA-LP	ABCA1	0.053558
EBNA-LP	MARK2	0.13552
EBNA-LP	CNTROB	0.31424
EBNA-LP	CDC42SE2	0.11624
EBNA-LP	HEXIM2	0.26976
EBNA-LP	HRNR	0.15894
EBNA-LP	PKD2	0.041318
EBNA-LP	NCAPH	0.31466
EBNA-LP	AURKA	0.061979
EBNA-LP	ATG9B	0.034485
EBNA-LP	PCDHGB2	0.17939
EBNA-LP	BBS4	0.06756
EBNA-LP	PDZD7	0.36383
EBNA-LP	PPP2CB	0.12446
EBNA-LP	DLC1	0.12831
EBNA-LP	STXBP4	0.14221

EBNA-LP	FOXC1	0.020128
EBNA-LP	IPO9	0.31202
EBNA-LP	ANXA8L1	0.17398
EBNA-LP	PDIA6	0.16437
EBNA-LP	HDAC5	0.050495
EBNA-LP	TRPC6	0.21559
EBNA-LP	ZNF655	0.21453
EBNA-LP	ETF1	0.3292
EBNA-LP	EMILIN1	0.31868
EBNA-LP	CRYGD	0.34377
EBNA-LP	ADRM1	0.26739
EBNA-LP	KLK6	0.25288
EBNA-LP	CCAR1	0.47781
EBNA-LP	PFDN4	0.28244
EBNA-LP	C19orf57	0.39532
EBNA-LP	LPCAT1	0.078777
EBNA-LP	HIST1H3A	0.23772
EBNA-LP	GSTO2	0.016518
EBNA-LP	CCNA1	0.1774
EBNA-LP	MYCN	0.13053
EBNA-LP	NKX2-1	0.037253
EBNA-LP	SPARCL1	0.20114
EBNA-LP	PDZD3	0.22232
EBNA-LP	CD40	0.10438
EBNA-LP	ENPP1	0.091304
EBNA-LP	PPP1R12A	0.17207
EBNA-LP	ERC1	0.18662
EBNA-LP	CTNND1	0.14166
EBNA-LP	PICALM	0.11396
EBNA-LP	PLEKHG6	0.17383
EBNA-LP	GUCA1A	0.19669
EBNA-LP	SEMA7A	0.25602
EBNA-LP	CRMP1	0.13921
EBNA-LP	TGFA	0.16952
EBNA-LP	IQCB1	0.40637
EBNA-LP	CCDC171	0.014189
EBNA-LP	CCAR2	0.3461
EBNA-LP	CACTIN	0.24367
EBNA-LP	PLAUR	0.17687
EBNA-LP	UTP20	0.27692
EBNA-LP	AP1S3	0.021241
EBNA-LP	DVL1	0.069985
EBNA-LP	GINS2	0.33159
EBNA-LP	WDR66	0.2099
EBNA-LP	MAP3K1	0.035394
EBNA-LP	LOXL2	0.098771
EBNA-LP	CASP3	0.097896
EBNA-LP	SHC1	0.079049
EBNA-LP	MITF	0.096022
EBNA-LP	ZNF217	0.10927
EBNA-LP	GTF2A1	0.19315
EBNA-LP	NLGN4Y	0.28072
EBNA-LP	SLC2A2	0.067582
EBNA-LP	ELF1	0.1725
EBNA-LP	COPS3	0.33316
EBNA-LP	ZMYND8	0.29526
EBNA-LP	COMMD6	0.34344
EBNA-LP	LONRF3	0.3383
EBNA-LP	GABARAP	0.24412
EBNA-LP	WWP2	0.12185
EBNA-LP	C1orf195	0.15232
EBNA-LP	TGFB1I1	0.15596
EBNA-LP	PIKFYVE	0.21702
EBNA-LP	MEX3B	0.15953
EBNA-LP	FBXW9	0.43184
EBNA-LP	CD37	0.37017
EBNA-LP	STAM	0.28191
EBNA-LP	GSK3A	0.069595

EBNA-LP	CBLB	0.23124
EBNA-LP	CRELD2	0.6631
EBNA-LP	PPP2R5A	0.20478
EBNA-LP	BLOC1S2	0.20975
EBNA-LP	MYOZ1	0.34052
EBNA-LP	TMEM30A	0.37045
EBNA-LP	NCOA3	0.14943
EBNA-LP	MUM1	0.37485
EBNA-LP	TIMELESS	0.22616
EBNA-LP	NEK9	0.10997
EBNA-LP	OSBPL3	0.32063
EBNA-LP	PTK2	0.021739
EBNA-LP	MZF1	0.14564
EBNA-LP	SLC34A3	0.052059
EBNA-LP	BANF1	0.28771
EBNA-LP	KXD1	0.40447
EBNA-LP	CPSF6	0.31148
EBNA-LP	MAST3	0.2409
EBNA-LP	FKBP14	0.16739
EBNA-LP	TOX4	0.34369
EBNA-LP	ARHGAP44	0.33352
EBNA-LP	ORC1	0.20145
EBNA-LP	LEO1	0.23878
EBNA-LP	CSRP2BP	0.25897
EBNA-LP	RORA	0.10721
EBNA-LP	ARL14EP	0.41868
EBNA-LP	NSMCE1	0.25005
EBNA-LP	MED7	0.21423
EBNA-LP	CLIC6	0.022506
EBNA-LP	TNFSF10	0.17428
EBNA-LP	GP1BB	0.30715
EBNA-LP	REL	0.15687
EBNA-LP	PCDHB10	0.1636
EBNA-LP	AIMP2	0.27974
EBNA-LP	APOA1BP	0.333
EBNA-LP	RPS7	0.21375
EBNA-LP	LILRB2	0.1899
EBNA-LP	YBR056C-B	0.032761
EBNA-LP	PFKFB1	0.16697
EBNA-LP	MNAT1	0.028212
EBNA-LP	EME1	0.28948
EBNA-LP	APPL2	0.19004
EBNA-LP	RBM26	0.39423
EBNA-LP	PSMG1	0.39405
EBNA-LP	KIF2A	0.17282
EBNA-LP	PATL2	0.34009
EBNA-LP	SEPT14	0.34034
EBNA-LP	NBR1	0.34516
EBNA-LP	SENP6	0.26976
EBNA-LP	EFCAB13	0.16306
EBNA-LP	HFE	0.25519
EBNA-LP	DPM1	0.22357
EBNA-LP	SF3A3	0.23754
EBNA-LP	MMP28	0.064384
EBNA-LP	IFT20	0.29539
EBNA-LP	GHR	0.045029
EBNA-LP	FBXW7	0.10619
EBNA-LP	MAGEA6	0.39536
EBNA-LP	RAB24	0.31626
EBNA-LP	RAB8A	0.23299
EBNA-LP	PFKL	0.19944
EBNA-LP	CXADR	0.078234
EBNA-LP	RAB11FIP2	0.21738
EBNA-LP	TRO	0.3109
EBNA-LP	ATP11C	0.21833
EBNA-LP	CD27	0.24531
EBNA-LP	LYST	0.19178
EBNA-LP	CASQ1	0.11334

EBNA-LP	ZNF652	0.24903
EBNA-LP	CEP57	0.20643
EBNA-LP	SCIMP	0.33154
EBNA-LP	TIPIN	0.25675
EBNA-LP	CHIC2	0.39742
EBNA-LP	BNIP3L	0.13961
EBNA-LP	AES	0.19
EBNA-LP	STX18	0.27157
EBNA-LP	VAMP7	0.15703
EBNA-LP	N4BP2	0.32889
EBNA-LP	SDC1	0.12582
EBNA-LP	TRAM1	0.27028
EBNA-LP	DCAF8	0.41438
EBNA-LP	DNM1L	0.078634
EBNA-LP	NSUN3	0.0171
EBNA-LP	DLGAP3	0.28642
EBNA-LP	USP9X	0.21532
EBNA-LP	PDIA2	0.23611
EBNA-LP	DERL1	0.22589
EBNA-LP	RSF1	0.16744
EBNA-LP	GSN	0.28311
EBNA-LP	RELB	0.1603
EBNA-LP	SLC11A2	0.062796
EBNA-LP	FAM96B	0.29207
EBNA-LP	TRMT112	0.47251
EBNA-LP	FXN	0.014458
EBNA-LP	NUP133	0.19071
EBNA-LP	PCDHGA4	0.17462
EBNA-LP	PAIP2	0.36906
EBNA-LP	PRKD1	0.01567
EBNA-LP	AMBRA1	0.40461
EBNA-LP	ADCY4	0.13206
EBNA-LP	MET	0.050472
EBNA-LP	TREML2	0.35963
EBNA-LP	PKP2	0.18545
EBNA-LP	VPS13A	0.33145
EBNA-LP	BMP2	0.017648
EBNA-LP	MME	0.094849
EBNA-LP	CEBPA	0.052068
EBNA-LP	PTPN22	0.083733
EBNA-LP	CSNK1D	0.11026
EBNA-LP	HADHA	0.14304
EBNA-LP	PCDHGA3	0.17939
EBNA-LP	TFB2M	0.060231
EBNA-LP	TBKBP1	0.42653
EBNA-LP	PPP1R27	0.38588
EBNA-LP	EIF4H	0.36244
EBNA-LP	HK1	0.2301
EBNA-LP	TELO2	0.31737
EBNA-LP	FRMPD2	0.33895
EBNA-LP	CCNB3	0.20346
EBNA-LP	PVRL1	0.18785
EBNA-LP	IGFBP3	0.1783
EBNA-LP	ASCL1	0.033064
EBNA-LP	UBA2	0.11405
EBNA-LP	IGFBP7	0.33384
EBNA-LP	S100A16	0.14655
EBNA-LP	BLNK	0.30023
EBNA-LP	RPGRIP1L	0.17509
EBNA-LP	ILF2	0.27696
EBNA-LP	POT1	0.18529
EBNA-LP	GDI1	0.29485
EBNA-LP	PPFIA3	0.38163
EBNA-LP	BMPR1B	0.084054
EBNA-LP	PAM	0.16259
EBNA-LP	CNTRL	0.32655
EBNA-LP	CDK13	0.080766
EBNA-LP	GOLGA2	0.38655

EBNA-LP PHACTR2 0.40487
EBNA-LP TRPC5 0.25196
EBNA-LP EIF4A1 0.28446
EBNA-LP TDRD7 0.30661
EBNA-LP CABP1 0.34404
EBNA-LP PALD1 0.40649
EBNA-LP RBBP5 0.2584
EBNA-LP PILRA 0.33694
EBNA-LP YBX1 0.10532
EBNA-LP EAF1 0.32562
EBNA-LP AKAP1 0.14068
EBNA-LP SCEL 0.30227
EBNA-LP SLC4A4 0.32213
EBNA-LP PKN3 0.18873
EBNA-LP ARF5 0.25112
EBNA-LP LETM1 0.64216
EBNA-LP CRH 0.16574
EBNA-LP EIF2AK2 0.13667
EBNA-LP FSTL5 0.20358
EBNA-LP NDRG1 0.20263
EBNA-LP PLA2G10 0.035712
EBNA-LP TPR 0.1792
EBNA-LP NEFM 0.27078
EBNA-LP LEMD3 0.24298
EBNA-LP BAG3 0.31929
EBNA-LP SPHK1 0.15779
EBNA-LP PACSIN3 0.31814
EBNA-LP VAPA 0.13935
EBNA-LP SCARF2 0.36147
EBNA-LP MAPRE1 0.19263
EBNA-LP CATSPER2 0.2107
EBNA-LP GCA 0.20121
EBNA-LP FNIP1 0.40564
EBNA-LP TCTEX1D2 0.43184
EBNA-LP VRK2 0.14082
EBNA-LP SMG1 0.19207
EBNA-LP ARAF 0.16676
EBNA-LP SRSF10 0.27009
EBNA-LP GPC3 0.2003
EBNA-LP LRRC3 0.40592
EBNA-LP RFC1 0.12996
EBNA-LP PPP4R1 0.33693
EBNA-LP TSSK2 0.16098
EBNA-LP ELL 0.25042
EBNA-LP CTU1 0.38808
EBNA-LP AKT2 0.031207
EBNA-LP PIAS4 0.17174
EBNA-LP BCAS3 0.36131
EBNA-LP ANGPT2 0.1141
EBNA-LP MAD2L1BP 0.32286
EBNA-LP ACTR1B 0.30695
EBNA-LP PIK3CB 0.15592
EBNA-LP RHOB 0.10214
EBNA-LP EFHD2 0.22297
EBNA-LP CYP4F2 0.070934
EBNA-LP FBF1 0.3075
EBNA-LP L3MBTL1 0.080948
EBNA-LP MRPL12 0.3904
EBNA-LP NFYC 0.12869
EBNA-LP GTF2F1 0.11638
EBNA-LP PKN1 0.072443
EBNA-LP IL1RL1 0.21983
EBNA-LP NSMAF 0.35612
EBNA-LP NCAN 0.10811
EBNA-LP FAM231A 0.21066
EBNA-LP TFB1M 0.36036
EBNA-LP NCOA5 0.32616
EBNA-LP ACHE 0.082647

EBNA-LP	DSG3	0.15291
EBNA-LP	FAM124A	0.047773
EBNA-LP	SLC1A3	0.0185
EBNA-LP	YAP1	0.10264
EBNA-LP	MATR3	0.24902
EBNA-LP	PCDHB18	0.17939
EBNA-LP	PPP2CA	0.080976
EBNA-LP	CEP164	0.28602
EBNA-LP	MLLT1	0.28017
EBNA-LP	RCC1	0.20834
EBNA-LP	PABPC1	0.18619
EBNA-LP	PLEKHF2	0.24727
EBNA-LP	JMY	0.23717
EBNA-LP	SNTB1	0.21585
EBNA-LP	MMP27	0.062525
EBNA-LP	CHST6	0.030678
EBNA-LP	AMER1	0.32914
EBNA-LP	TRIP11	0.24773
EBNA-LP	RTF1	0.19001
EBNA-LP	RWDD3	0.37519
EBNA-LP	PRKAG1	0.056167
EBNA-LP	LUC7L3	0.29979
EBNA-LP	MCM5	0.21196
EBNA-LP	CD28	0.1748
EBNA-LP	CTDSP1	0.21197
EBNA-LP	HLA-DRB1	0.082559
EBNA-LP	PNRC1	0.32616
EBNA-LP	IRS1	0.032287
EBNA-LP	ITIH1	0.17474
EBNA-LP	PRTN3	0.34554
EBNA-LP	TBX5	0.032325
EBNA-LP	MAL	0.31424
EBNA-LP	OOEP	0.034904
EBNA-LP	FAM188A	0.62958
EBNA-LP	ZP3	0.082173
EBNA-LP	BCKDK	0.21699
EBNA-LP	MCF2	0.19323
EBNA-LP	UMOD	0.1442
EBNA-LP	C6	0.29431
EBNA-LP	HDHD3	0.36461
EBNA-LP	IPO8	0.30741
EBNA-LP	RNF43	0.17669
EBNA-LP	STX2	0.19967
EBNA-LP	RHBDL3	0.2937
EBNA-LP	AP1B1	0.31663
EBNA-LP	CCNT1	0.12521
EBNA-LP	GAPDH	0.20659
EBNA-LP	NRD1	0.099722
EBNA-LP	DNM2	0.057187
EBNA-LP	GRB10	0.20074
EBNA-LP	NBN	0.08701
EBNA-LP	RCN3	0.70415
EBNA-LP	MGST1	0.14101
EBNA-LP	RNF5	0.22026
EBNA-LP	PLSCR1	0.29402
EBNA-LP	CETN3	0.1924
EBNA-LP	SNX7	0.12516
EBNA-LP	CTNNBL1	0.31741
EBNA-LP	KDM2B	0.16789
EBNA-LP	FDXR	0.25841
EBNA-LP	FLT1	0.057463
EBNA-LP	BCLAF1	0.24192
EBNA-LP	EFTUD2	0.24234
EBNA-LP	ITSN2	0.056843
EBNA-LP	SERPINE2	0.075084
EBNA-LP	NF1	0.047029
EBNA-LP	F8	0.2869
EBNA-LP	FBXO45	0.31274

EBNA-LP NSG2 0.089611
EBNA-LP KIF21A 0.32149
EBNA-LP SCAMP2 0.23519
EBNA-LP PCDH10 0.17939
EBNA-LP GIT1 0.24469
EBNA-LP CCL5 0.013415
EBNA-LP GRIN2B 0.02276
EBNA-LP SLC6A4 0.13706
EBNA-LP CDH19 0.17394
EBNA-LP VEGFC 0.16608
EBNA-LP CLK2 0.1956
EBNA-LP SLC12A3 0.29374
EBNA-LP SNAP25 0.095097
EBNA-LP DDX23 0.20848
EBNA-LP LSM10 0.27925
EBNA-LP SUPV3L1 0.1394
EBNA-LP PSENEN 0.23211
EBNA-LP ATN1 0.22717
EBNA-LP VAPB 0.14176
EBNA-LP CARM1 0.01625
EBNA-LP NHEJ1 0.28512
EBNA-LP ESR1 0.023628
EBNA-LP STAT5B 0.044833
EBNA-LP COPRS 0.29862
EBNA-LP RPA1 0.1037
EBNA-LP HSPG2 0.15565
EBNA-LP CELSR2 0.092556
EBNA-LP SMARCA4 0.10612
EBNA-LP ATG5 0.2575
EBNA-LP ACTL6A 0.16102
EBNA-LP PAXIP1 0.22375
EBNA-LP EGFR 0.027703
EBNA-LP ACTR8 0.29677
EBNA-LP IREB2 0.053547
EBNA-LP CAMLG 0.35659
EBNA-LP NKAPL 0.08598
EBNA-LP SELP 0.12204
EBNA-LP FBXO5 0.10451
EBNA-LP PTGDR 0.31386
EBNA-LP APAF1 0.18885
EBNA-LP ENTPD7 0.067794
EBNA-LP SIKE1 0.33538
EBNA-LP CTCF 0.073328
EBNA-LP PPFIA2 0.39353
EBNA-LP PCDHGA8 0.17939
EBNA-LP MMP8 0.066593
EBNA-LP MED12 0.032594
EBNA-LP MST1R 0.18481
EBNA-LP SCIN 0.12568
EBNA-LP CCDC33 0.43184
EBNA-LP NPC1 0.14213
EBNA-LP SYTL5 0.33556
EBNA-LP HINFP 0.080159
EBNA-LP MORC4 0.3318
EBNA-LP PLP2 0.32372
EBNA-LP TLDC2 0.049479
EBNA-LP RAB11A 0.18121
EBNA-LP LTBP1 0.6356
EBNA-LP KIF5A 0.23547
EBNA-LP PEX6 0.28219
EBNA-LP SMARCC1 0.19543
EBNA-LP TPD52L2 0.41184
EBNA-LP ASPSCR1 0.28308
EBNA-LP ARL6IP4 0.37518
EBNA-LP F2 0.18535
EBNA-LP CELF1 0.26627
EBNA-LP RHOXF2 0.20779
EBNA-LP SYT7 0.21705

EBNA-LP	FAAP20	0.27608
EBNA-LP	DLG1	0.026321
EBNA-LP	THOC2	0.39154
EBNA-LP	HES1	0.019004
EBNA-LP	PLEKHA2	0.20919
EBNA-LP	EREG	0.12167
EBNA-LP	IFIT2	0.30191
EBNA-LP	HEATR1	0.28327
EBNA-LP	SCNN1B	0.25905
EBNA-LP	SMYD2	0.22816
EBNA-LP	ITPR1	0.13328
EBNA-LP	VAMP3	0.23867
EBNA-LP	WIPI2	0.26099
EBNA-LP	CDH9	0.15551
EBNA-LP	SPAST	0.14982
EBNA-LP	PCDHB9	0.1636
EBNA-LP	RRAS	0.22951
EBNA-LP	MIEF2	0.29835
EBNA-LP	FST	0.20804
EBNA-LP	CEP290	0.18731
EBNA-LP	KDM5B	0.10578
EBNA-LP	NOXO1	0.23745
EBNA-LP	SYT1	0.23605
EBNA-LP	NSMCE2	0.20198
EBNA-LP	RND2	0.33107
EBNA-LP	HMOX2	0.22163
EBNA-LP	WRNIP1	0.25679
EBNA-LP	CBL	0.24485
EBNA-LP	NEK3	0.21445
EBNA-LP	CORO1A	0.13322
EBNA-LP	N4BP3	0.087526
EBNA-LP	METTL20	0.055343
EBNA-LP	PHF12	0.21496
EBNA-LP	SLX4	0.27746
EBNA-LP	ANKRD27	0.34792
EBNA-LP	EPHA2	0.085018
EBNA-LP	MYC	0.039341
EBNA-LP	ARFRP1	0.24511
EBNA-LP	UCHL5	0.15385
EBNA-LP	VIM	0.10031
EBNA-LP	NOA1	0.24729
EBNA-LP	PEX26	0.29888
EBNA-LP	WARS	0.21064
EBNA-LP	TIMM10	0.21963
EBNA-LP	SLC25A13	0.12657
EBNA-LP	KLRC2	0.34817
EBNA-LP	NPM1	0.057215
EBNA-LP	GDF2	0.15495
EBNA-LP	NAP1L4	0.29627
EBNA-LP	NSUN5P1	0.066347
EBNA-LP	MYCBP	0.26376
EBNA-LP	PI4K2A	0.22924
EBNA-LP	GRB2	0.049776
EBNA-LP	ADNP2	0.11339
EBNA-LP	SHPK	0.033153
EBNA-LP	ZNF639	0.094072
EBNA-LP	NCS1	0.44621
EBNA-LP	TCAP	0.21888
EBNA-LP	HPSE	0.15741
EBNA-LP	CDK2	0.050498
EBNA-LP	MBL2	0.206
EBNA-LP	NCK2	0.21158
EBNA-LP	IKZF3	0.10558
EBNA-LP	NMNAT1	0.29328
EBNA-LP	GPKOW	0.3434
EBNA-LP	FBLN5	0.51697
EBNA-LP	MPP6	0.39356
EBNA-LP	ARHGAP1	0.2078

EBNA-LP	CRBN	0.27668
EBNA-LP	HSCB	0.34732
EBNA-LP	BIRC5	0.064473
EBNA-LP	DAG1	0.15134
EBNA-LP	AP5S1	0.291
EBNA-LP	MCM3	0.14936
EBNA-LP	TOMM20	0.21152
EBNA-LP	OTOF	0.15409
EBNA-LP	AGTRAP	0.36326
EBNA-LP	AP1S1	0.019211
EBNA-LP	ID2	0.083651
EBNA-LP	ZNF133	0.14304
EBNA-LP	DOC2A	0.34082
EBNA-LP	MED30	0.27658
EBNA-LP	RHOBTB3	0.32055
EBNA-LP	XRCC3	0.28425
EBNA-LP	F11	0.30622
EBNA-LP	SRGAP2	0.16402
EBNA-LP	CALCOCO1	0.19296
EBNA-LP	FBXO6	0.31151
EBNA-LP	VPS35	0.28228
EBNA-LP	HRK	0.30118
EBNA-LP	ADAM22	0.23439
EBNA-LP	PI4K2B	0.037679
EBNA-LP	PCDHA12	0.17939
EBNA-LP	TBC1D9B	0.14203
EBNA-LP	PTPRT	0.10495
EBNA-LP	SIGLEC8	0.35869
EBNA-LP	SNRNP40	0.28046
EBNA-LP	ARID1A	0.16313
EBNA-LP	SCNN1D	0.34016
EBNA-LP	STON2	0.31746
EBNA-LP	SNX22	0.12516
EBNA-LP	CENPE	0.13468
EBNA-LP	PLK2	0.14026
EBNA-LP	FBXW11	0.21859
EBNA-LP	SCAI	0.27417
EBNA-LP	KCNMB4	0.2695
EBNA-LP	AHR	0.031711
EBNA-LP	NIPBL	0.14527
EBNA-LP	KMT2A	0.11185
EBNA-LP	UBE2C	0.1627
EBNA-LP	SERPINA3	0.2775
EBNA-LP	ADRBK1	0.065828
EBNA-LP	DOK1	0.18842
EBNA-LP	IL7R	0.19322
EBNA-LP	MYL5	0.18216
EBNA-LP	TPM4	0.12073
EBNA-LP	DYX1C1	0.32482
EBNA-LP	BMF	0.2365
EBNA-LP	INTS2	0.31545
EBNA-LP	RAB22A	0.23693
EBNA-LP	MYO7A	0.13343
EBNA-LP	PIAS2	0.15995
EBNA-LP	C5orf48	0.013461
EBNA-LP	MUC2	0.37591
EBNA-LP	ETNK1	0.016515
EBNA-LP	SPTLC1	0.20396
EBNA-LP	NLK	0.10753
EBNA-LP	ITGA6	0.27062
EBNA-LP	PILRB	0.3696
EBNA-LP	CTNNA2	0.15705
EBNA-LP	DEPTOR	0.35924
EBNA-LP	FTH1	0.24014
EBNA-LP	NOD1	0.080073
EBNA-LP	RCAN1	0.15027
EBNA-LP	DGKB	0.1864
EBNA-LP	ZFYVE9	0.35225

EBNA-LP PDS5A 0.32966
EBNA-LP TIGIT 0.28775
EBNA-LP RNF8 0.12415
EBNA-LP RFX5 0.18887
EBNA-LP NPHS1 0.22192
EBNA-LP TAL1 0.037656
EBNA-LP NECAB3 0.53294
EBNA-LP SNX12 0.41039
EBNA-LP ATM 0.074857
EBNA-LP SYNGR4 0.16891
EBNA-LP AQP6 0.053508
EBNA-LP TCHH 0.15896
EBNA-LP RASSF9 0.33446
EBNA-LP GORASP2 0.32118
EBNA-LP KIR2DL4 0.33756
EBNA-LP MLC1 0.2416
EBNA-LP LBP 0.12651
EBNA-LP ADAP2 0.079193
EBNA-LP TRIM16 0.21497
EBNA-LP IFI35 0.36106
EBNA-LP EDA 0.11638
EBNA-LP PXMP4 0.37008
EBNA-LP ASGR2 0.31233
EBNA-LP STAG1 0.26344
EBNA-LP EFNA4 0.42491
EBNA-LP SIN3A 0.061774
EBNA-LP DYNC1H1 0.21029
EBNA-LP GRM7 0.064436
EBNA-LP GALK2 0.033054
EBNA-LP CNTNAP1 0.18745
EBNA-LP PBXIP1 0.25887
EBNA-LP RRAGD 0.29514
EBNA-LP TRMT61B 0.43287
EBNA-LP CCR1 0.156
EBNA-LP SDC4 0.22021
EBNA-LP LONP2 0.21411
EBNA-LP HSPA14 0.33452
EBNA-LP PLXNB2 0.25591
EBNA-LP APH1B 0.3089
EBNA-LP SNX6 0.34758
EBNA-LP APOA5 0.12863
EBNA-LP KRT1 0.23821
EBNA-LP ZNF143 0.19974
EBNA-LP BRCA1 0.036554
EBNA-LP RFX1 0.29679
EBNA-LP CDK20 0.14964
EBNA-LP KRT81 0.33945
EBNA-LP ERGIC1 0.26581
EBNA-LP RALB 0.22196
EBNA-LP CDC73 0.1782
EBNA-LP GSE1 0.43184
EBNA-LP NCOA1 0.12218
EBNA-LP FGA 0.26205
EBNA-LP RASGRP1 0.089895
EBNA-LP HLTF 0.14081
EBNA-LP DUSP7 0.086526
EBNA-LP PDE4A 0.17986
EBNA-LP ZFYVE1 0.24335
EBNA-LP IFNAR2 0.18172
EBNA-LP NMI 0.34097
EBNA-LP CLSTN3 0.10494
EBNA-LP CDK5RAP3 0.23794
EBNA-LP TRIM28 0.047427
EBNA-LP PCDHGB6 0.17939
EBNA-LP CHMP4A 0.10456
EBNA-LP WWTR1 0.14386
EBNA-LP PCDHB11 0.15367
EBNA-LP ASF1A 0.22542

EBNA-LP ERRFI1 0.12902
EBNA-LP C19orf25 0.43184
EBNA-LP IRF7 0.046255
EBNA-LP MAPK9 0.11708
EBNA-LP BBS2 0.18368
EBNA-LP APOA2 0.053969
EBNA-LP XPA 0.21888
EBNA-LP CHMP5 0.34486
EBNA-LP EIF2S2 0.3918
EBNA-LP ZFYVE16 0.16966
EBNA-LP PUS10 0.061464
EBNA-LP NMRK2 0.38573
EBNA-LP PPARG 0.020466
EBNA-LP PHB2 0.23351
EBNA-LP DCP1B 0.22952
EBNA-LP FAM92A1 0.019069
EBNA-LP MYB 0.11916
EBNA-LP CLVS1 0.045167
EBNA-LP SNRPD1 0.2594
EBNA-LP SOCS1 0.18678
EBNA-LP AKAP7 0.087038
EBNA-LP C1S 0.19339
EBNA-LP NPBWR1 0.29146
EBNA-LP SNCB 0.12844
EBNA-LP EGLN3 0.20979
EBNA-LP NPC1L1 0.3658
EBNA-LP PCGF2 0.11161
EBNA-LP DNMBP 0.29801
EBNA-LP PRAM1 0.44476
EBNA-LP EFNB1 0.26998
EBNA-LP NT5C3B 0.32388
EBNA-LP CDYL2 0.32089
EBNA-LP AASDHPPT 0.32781
EBNA-LP FHL1 0.20832
EBNA-LP GGA3 0.28231
EBNA-LP UTP11L 0.29472
EBNA-LP RSRC1 0.31273
EBNA-LP GLRA1 0.11075
EBNA-LP AQP5 0.29071
EBNA-LP ARL4C 0.21466
EBNA-LP NR0B2 0.12907
EBNA-LP ARHGAP21 0.27062
EBNA-LP ARL3 0.096178
EBNA-LP MEGF6 0.68511
EBNA-LP MED20 0.21026
EBNA-LP RPS6KA1 0.06707
EBNA-LP STAG2 0.2482
EBNA-LP EIF2S3 0.31921
EBNA-LP CALR 0.066197
EBNA-LP JPH2 0.24067
EBNA-LP BTBD1 0.36814
EBNA-LP SOCS5 0.32531
EBNA-LP EMX2 0.11484
EBNA-LP MRPL11 0.35056
EBNA-LP EGR1 0.072683
EBNA-LP MC2R 0.24619
EBNA-LP NUP205 0.26575
EBNA-LP RAB33A 0.28852
EBNA-LP HOXA10 0.14709
EBNA-LP HSP90AA1 0.17778
EBNA-LP TAF1 0.080027
EBNA-LP GAS1 0.10721
EBNA-LP C16orf45 0.016235
EBNA-LP TSPY1 0.25486
EBNA-LP TNFRSF14 0.25789
EBNA-LP LYL1 0.28249
EBNA-LP PIK3C2A 0.039399
EBNA-LP NFKBIL1 0.32434

EBNA-LP CBX5 0.16004
EBNA-LP ZFAND2B 0.35419
EBNA-LP F12 0.24027
EBNA-LP PCBD1 0.14742
EBNA-LP NRBP1 0.15978
EBNA-LP SLC25A12 0.11965
EBNA-LP ATF4 0.10468
EBNA-LP ATP6AP2 0.30451
EBNA-LP PCDHA1 0.16343
EBNA-LP ANK1 0.11569
EBNA-LP PCBP1 0.33621
EBNA-LP HSF1 0.13085
EBNA-LP UNC5B 0.29628
EBNA-LP SCRIB 0.16915
EBNA-LP RPUSD2 0.035092
EBNA-LP RPTOR 0.13451
EBNA-LP SRRT 0.2552
EBNA-LP SSTR3 0.21286
EBNA-LP DGKG 0.17197
EBNA-LP KHDRBS2 0.34319
EBNA-LP MMP9 0.06803
EBNA-LP PLXNB3 0.31583
EBNA-LP KCNIP2 0.32762
EBNA-LP TBCD 0.2542
EBNA-LP THEG 0.34262
EBNA-LP SLPI 0.31319
EBNA-LP PDLIM4 0.36476
EBNA-LP DAO 0.20478
EBNA-LP PCDHA13 0.17939
EBNA-LP KIF3B 0.20187
EBNA-LP PRSS8 0.34294
EBNA-LP FSCN1 0.27941
EBNA-LP TRAF1 0.27804
EBNA-LP CALML3 0.2099
EBNA-LP PROX1 0.025749
EBNA-LP POLG2 0.24139
EBNA-LP RAMP3 0.22625
EBNA-LP AGO4 0.30732
EBNA-LP CRK 0.22258
EBNA-LP RFWD2 0.21771
EBNA-LP SPOCK3 0.16209
EBNA-LP PIAS3 0.16287
EBNA-LP CDH4 0.13487
EBNA-LP TNS1 0.33783
EBNA-LP ITGA2B 0.23129
EBNA-LP PAFAH1B1 0.035666
EBNA-LP MAPK13 0.18606
EBNA-LP ADAM12 0.15785
EBNA-LP UBA52 0.034579
EBNA-LP FOXL2 0.03814
EBNA-LP KCNIP3 0.073416
EBNA-LP SNRPN 0.36073
EBNA-LP CDK14 0.12925
EBNA-LP PARD6A 0.17584
EBNA-LP SMARCA2 0.096571
EBNA-LP PLCG1 0.12657
EBNA-LP HEATR5B 0.06835
EBNA-LP FAM109B 0.29716
EBNA-LP ITGAX 0.29351
EBNA-LP PDCD4 0.35759
EBNA-LP PPP1R8 0.24881
EBNA-LP NOTCH1 0.018655
EBNA-LP PCLO 0.1327
EBNA-LP IL8 0.17231
EBNA-LP FBN2 0.1693
EBNA-LP ZNHIT1 0.39136
EBNA-LP EYA2 0.098091
EBNA-LP ERCC1 0.076168

```
EBNA-LP PSME3   0.13485
EBNA-LP ELOVL2  0.24647
EBNA-LP EFCAB10 0.19711
EBNA-LP CD93    0.4687
EBNA-LP SRR 0.094841
EBNA-LP EEF1B2  0.31665
EBNA-LP MAN1A1  0.084633
EBNA-LP CAPN12  0.09102
EBNA-LP TEX11   0.31713
EBNA-LP LMX1B   0.14344
EBNA-LP PLA2G2F 0.065212
EBNA-LP NLRC5   0.2118
EBNA-LP DRD1    0.030556
EBNA-LP STK36   0.072534
EBNA-LP RITA1   0.2822
EBNA-LP SYT8    0.020182
EBNA-LP GRIN1   0.2055
EBNA-LP CST3    0.092953
EBNA-LP FAN1    0.25538
EBNA-LP CLINT1  0.41289
EBNA-LP IRF5    0.10508
EBNA-LP EIF2AK3 0.078384
EBNA-LP ZBTB21  0.23418
EBNA-LP MARK4   0.18852
EBNA-LP BBS12   0.34895
EBNA-LP DHH 0.42888
EBNA-LP SLC25A4 0.16651
EBNA-LP EIF4E1B 0.010236
EBNA-LP SPATA21 0.2099
EBNA-LP NDFIP2  0.21117
EBNA-LP NCOA2   0.084184
EBNA-LP SIRT2   0.085146
EBNA-LP IMMT    0.34184
EBNA-LP UBE2L3  0.1296
EBNA-LP IL5 0.26918
EBNA-LP RRM1    0.25736
EBNA-LP ILK 0.038445
EBNA-LP FLT4    0.10837
EBNA-LP MLF1    0.27633
EBNA-LP GAS2L1  0.029042
EBNA-LP ANXA9   0.088345
EBNA-LP STRN3   0.15982
EBNA-LP RAP1B   0.1829
EBNA-LP IKBKG   0.094765
EBNA-LP SPHK2   0.30018
EBNA-LP MARK3   0.2513
EBNA-LP MECP2   0.019347
EBNA-LP HDAC1   0.031853
EBNA-LP NR2F6   0.11342
EBNA-LP VIPAS39 0.29184
EBNA-LP ADAM15  0.20996
EBNA-LP PRKCB   0.071403
EBNA-LP PLD3    0.1899
EBNA-LP PCDHGA10    0.17939
EBNA-LP ADAM8   0.12037
EBNA-LP TRIP12  0.28568
EBNA-LP CSTF1   0.35441
EBNA-LP U2AF2   0.30534
EBNA-LP MAP3K8  0.16477
EBNA-LP NOSTRIN 0.37823
EBNA-LP ELMO1   0.19455
EBNA-LP SS18    0.23205
EBNA-LP U2SURP  0.4153
EBNA-LP AICDA   0.16079
EBNA-LP DCHS2   0.17939
EBNA-LP SNX18   0.32334
EBNA-LP PLCZ1   0.074204
EBNA-LP N4BP2L1 0.027148
```

EBNA-LP	CDC42EP1	0.29388
EBNA-LP	ADRB1	0.077076
EBNA-LP	SIRT7	0.19657
EBNA-LP	AP1G2	0.3007
EBNA-LP	PREX1	0.1256
EBNA-LP	ATP2C2	0.22133
EBNA-LP	PCDHGC3	0.1739
EBNA-LP	PDCD10	0.26792
EBNA-LP	MRM1	0.14844
EBNA-LP	FAM231D	0.085259
EBNA-LP	USP44	0.23803
EBNA-LP	ZBTB17	0.11224
EBNA-LP	GFI1B	0.12363
EBNA-LP	CCBE1	0.16105
EBNA-LP	BLMH	0.25099
EBNA-LP	IRAK1	0.074507
EBNA-LP	ERCC3	0.030358
EBNA-LP	CXCR4	0.10766
EBNA-LP	USP37	0.17572
EBNA-LP	ARFIP2	0.28759
EBNA-LP	SAC3D1	0.32122
EBNA-LP	MMS22L	0.33463
EBNA-LP	MTMR9	0.36207
EBNA-LP	SLC1A4	0.04496
EBNA-LP	COL4A2	0.26769
EBNA-LP	PKD1L2	0.17431
EBNA-LP	CHMP1A	0.17736
EBNA-LP	PPM1G	0.18844
EBNA-LP	XPO1	0.17551
EBNA-LP	LBR	0.27558
EBNA-LP	PRMT3	0.0132
EBNA-LP	ATP8B1	0.24185
EBNA-LP	TJP2	0.30244
EBNA-LP	SLC17A6	0.030533
EBNA-LP	NR1I2	0.1047
EBNA-LP	ITM2A	0.40592
EBNA-LP	RBBP4	0.16873
EBNA-LP	YWHAQ	0.35726
EBNA-LP	HOOK1	0.22769
EBNA-LP	GTF2H2	0.034651
EBNA-LP	INPP5K	0.055936
EBNA-LP	C11orf72	0.029667
EBNA-LP	PPP1R3C	0.26664
EBNA-LP	ATP10A	0.19512
EBNA-LP	IQCF3	0.0112
EBNA-LP	KLK5	0.37574
EBNA-LP	MARCH5	0.15493
EBNA-LP	STT3A	0.3363
EBNA-LP	ZBTB24	0.24903
EBNA-LP	TCF12	0.11807
EBNA-LP	SPC24	0.27154
EBNA-LP	NCF2	0.10429
EBNA-LP	ESD	0.011511
EBNA-LP	FGFR2	0.014113
EBNA-LP	CCL3	0.057542
EBNA-LP	MYL6B	0.56118
EBNA-LP	CNIH2	0.22644
EBNA-LP	GLI1	0.082049
EBNA-LP	GIPC1	0.1539
EBNA-LP	RAD51C	0.21788
EBNA-LP	NAA38	0.33555
EBNA-LP	IGF2BP2	0.28472
EBNA-LP	YWHAB	0.13751
EBNA-LP	SLC25A5	0.25845
EBNA-LP	GNA13	0.13649
EBNA-LP	BLOC1S5	0.32462
EBNA-LP	TRIM21	0.18006
EBNA-LP	SPTSSB	0.28275

EBNA-LP	TAF15	0.32458
EBNA-LP	RBX1	0.14498
EBNA-LP	TNNI3K	0.1882
EBNA-LP	WDR82	0.31895
EBNA-LP	RAB3A	0.11341
EBNA-LP	NSL1	0.24615
EBNA-LP	BCL3	0.033332
EBNA-LP	HMGB2	0.051583
EBNA-LP	RHOH	0.26255
EBNA-LP	PI4KAP2	0.056596
EBNA-LP	KCNQ1	0.036866
EBNA-LP	GPX7	0.031297
EBNA-LP	ATP6V0A1	0.27527
EBNA-LP	BRPF3	0.28371
EBNA-LP	GTF2I	0.16171
EBNA-LP	CPSF3	0.22684
EBNA-LP	KCNMA1	0.15667
EBNA-LP	ORC6	0.28415
EBNA-LP	TGM3	0.076687
EBNA-LP	CELSR1	0.078442
EBNA-LP	UBQLN2	0.3527
EBNA-LP	PLRG1	0.31351
EBNA-LP	CUL1	0.19625
EBNA-LP	SCG5	0.29904
EBNA-LP	CD9	0.22659
EBNA-LP	DHX58	0.17835
EBNA-LP	MYO9A	0.22382
EBNA-LP	SP3	0.10875
EBNA-LP	POLI	0.21509
EBNA-LP	SLC24A1	0.3241
EBNA-LP	DR1	0.20273
EBNA-LP	TBK1	0.1075
EBNA-LP	HSPA9	0.22936
EBNA-LP	NCSTN	0.15379
EBNA-LP	SLC2A1	0.028439
EBNA-LP	PLSCR2	0.18012
EBNA-LP	GSTP1	0.13448
EBNA-LP	MMP14	0.12426
EBNA-LP	ELP3	0.065259
EBNA-LP	PIGK	0.18631
EBNA-LP	CDC5L	0.28781
EBNA-LP	MBNL1	0.24648
EBNA-LP	MED4	0.24312
EBNA-LP	RHBDD1	0.21706
EBNA-LP	TIE1	0.23494
EBNA-LP	FABP5	0.34268
EBNA-LP	AP1G1	0.25063
EBNA-LP	HMCN2	0.22578
EBNA-LP	CDHR2	0.58491
EBNA-LP	FAS	0.053148
EBNA-LP	APOE	0.020021
EBNA-LP	TUBB2B	0.12876
EBNA-LP	WAS	0.23793
EBNA-LP	ALDOC	0.1784
EBNA-LP	TFG	0.34078
EBNA-LP	TNNI3	0.13966
EBNA-LP	WDR61	0.23678
EBNA-LP	SULT2B1	0.24464
EBNA-LP	PAWR	0.19915
EBNA-LP	ASIC2	0.30095
EBNA-LP	CBX3	0.17869
EBNA-LP	RINT1	0.32039
EBNA-LP	SHANK3	0.13858
EBNA-LP	IKBIP	0.34429
EBNA-LP	RCVRN	0.20822
EBNA-LP	AGFG1	0.28536
EBNA-LP	C17orf62	0.40592
EBNA-LP	SNX17	0.27544

EBNA-LP	CDH5	0.41639
EBNA-LP	DDX58	0.096391
EBNA-LP	ARID1B	0.2082
EBNA-LP	IL6R	0.14021
EBNA-LP	HS3ST5	0.27493
EBNA-LP	TP53INP2	0.32347
EBNA-LP	ZNF165	0.15176
EBNA-LP	SEMA6A	0.24355
EBNA-LP	ZMYND10	0.35366
EBNA-LP	COTL1	0.33452
EBNA-LP	SMN1	0.33032
EBNA-LP	SNIP1	0.27289
EBNA-LP	SYT2	0.47349
EBNA-LP	CCNY	0.17219
EBNA-LP	CAPNS1	0.48474
EBNA-LP	GTF2E2	0.26549
EBNA-LP	MMP12	0.019462
EBNA-LP	KCNG3	0.27197
EBNA-LP	TRIM41	0.2633
EBNA-LP	MPZL3	0.36933
EBNA-LP	GABARAPL1	0.36536
EBNA-LP	GTF2B	0.18954
EBNA-LP	ANGPTL4	0.22236
EBNA-LP	WDR16	0.35774
EBNA-LP	IFIT1	0.30528
EBNA-LP	PCDHB15	0.16848
EBNA-LP	RAD18	0.1737
EBNA-LP	DNM1	0.11567
EBNA-LP	OCM2	0.2099
EBNA-LP	SIPA1	0.21281
EBNA-LP	TPD52	0.60968
EBNA-LP	MC5R	0.28673
EBNA-LP	RIPK4	0.23614
EBNA-LP	POLDIP3	0.3605
EBNA-LP	IRS2	0.080438
EBNA-LP	ARID5B	0.15028
EBNA-LP	NUFIP1	0.25944
EBNA-LP	KLC1	0.32417
EBNA-LP	FRMPD4	0.40968
EBNA-LP	LIG4	0.075388
EBNA-LP	KLHDC3	0.3527
EBNA-LP	CDC42BPA	0.062262
EBNA6	ubl4ab	0.14518
EBNA6	MED1	0.016936
EBNA6	PIK3CD	0.011815
EBNA6	GAGE12I	0.12718
EBNA6	PAGE2B	0.2897
EBNA6	SPTA1	0.065429
EBNA6	S100A7	0.1051
EBNA6	NANOS1	0.098223
EBNA6	HSPA5	0.057119
EBNA6	LDLR	0.045071
EBNA6	SERTAD3	0.2274
EBNA6	INO80B	0.19478
EBNA6	CENPW	0.092387
EBNA6	UPF3A	0.14576
EBNA6	RABEP1	0.19779
EBNA6	SLC5A1	0.056091
EBNA6	C12orf61	0.1358
EBNA6	FAM86B2	0.027772
EBNA6	PNMA6D	0.061082
EBNA6	PLSCR3	0.14572
EBNA6	DYM	0.17596
EBNA6	ZC3HAV1L	0.16516
EBNA6	BTBD16	0.036414
EBNA6	C9orf40	0.036462
EBNA6	RPUSD4	0.095293
EBNA6	CCDC159	0.12621

EBNA6	C10orf68	0.12756
EBNA6	CCDC42	0.022566
EBNA6	CCDC162P	0.036833
EBNA6	GOLGA6L1	0.075634
EBNA6	SNAPIN	0.012086
EBNA6	KRI1	0.23179
EBNA6	SPATA1	0.038522
EBNA6	CCDC69	0.10208
EBNA6	EIF3J	0.12376
EBNA6	C10orf62	0.16833
EBNA6	UNC119B	0.10881
EBNA6	YMR013W-A	0.13038
EBNA6	LEKR1	0.012942
EBNA6	CASC3	0.20417
EBNA6	C17orf64	0.016191
EBNA6	CYP4F30P	0.024885
EBNA6	ATP5B	0.074601
EBNA6	NIN	0.21947
EBNA6	C5orf52	0.027007
EBNA6	KCNIP1	0.12164
EBNA6	C22orf39	0.01824
EBNA6	STIM2	0.14407
EBNA6	HUS1B	0.13282
EBNA6	WBP2	0.17398
EBNA6	C1orf222	0.04709
EBNA6	SLAIN2	0.069407
EBNA6	SSSCA1	0.13029
EBNA6	RPLP1	0.089411
EBNA6	PSMG3	0.022303
EBNA6	THOC5	0.0564
EBNA6	ANGEL1	0.058007
EBNA6	C19orf44	0.067222
EBNA6	C15orf40	0.014699
EBNA6	FSIP1	0.1939
EBNA6	COMMD5	0.18743
EBNA6	WIPF1	0.081818
EBNA6	SPTAN1	0.091264
EBNA6	TSGA10IP	0.15218
EBNA6	TNFAIP8L3	0.051008
EBNA6	ATRIP	0.10009
EBNA6	LAMTOR1	0.060316
EBNA6	DHPS	0.10441
EBNA6	IL12A	0.093154
EBNA6	POP4	0.1923
EBNA6	MASP1	0.19583
EBNA6	CCDC174	0.02368
EBNA6	SCUBE3	0.18056
EBNA6	TRAPPC4	0.049966
EBNA6	WBSCR27	0.011401
EBNA6	FBXO7	0.1234
EBNA6	SPBC1539.02	0.091922
EBNA6	C19orf45	0.012645
EBNA6	HEXIM1	0.20318
EBNA6	C2orf43	0.046125
EBNA6	COMMD7	0.14821
EBNA6	ATG4B	0.12954
EBNA6	GLTSCR1L	0.27738
EBNA6	TSR2	0.17675
EBNA6	MAB21L3	0.03374
EBNA6	PEX19	0.076616
EBNA6	NAGK	0.040402
EBNA6	PRR15L	0.051843
EBNA6	TECPR1	0.13676
EBNA6	SYT14L	0.011221
EBNA6	CHMP4B	0.11365
EBNA6	PROC	0.16511
EBNA6	PDCD6	0.19035
EBNA6	DKC1	0.086919

EBNA6	S100A9	0.046442
EBNA6	ANXA2	0.088202
EBNA6	MMP20	0.053904
EBNA6	smf	0.047489
EBNA6	MAGEB1	0.021388
EBNA6	MATN4	0.18624
EBNA6	POLG	0.084561
EBNA6	PSMF1	0.1079
EBNA6	NOTCH3	0.044334
EBNA6	RNH1	0.15222
EBNA6	UBAP2	0.21313
EBNA6	C17orf105	0.11876
EBNA6	RRP7B	0.032096
EBNA6	FAM110D	0.21499
EBNA6	SELPLG	0.23174
EBNA6	CLIP1	0.070897
EBNA6	YTHDF3	0.16443
EBNA6	SRRM3	0.010851
EBNA6	BLOC1S3	0.17723
EBNA6	AMOT	0.043855
EBNA6	CCDC154	0.055913
EBNA6	SLC4A1	0.035085
EBNA6	PAGE5	0.125
EBNA6	C20orf196	0.037615
EBNA6	URM1	0.12972
EBNA6	GAGE10	0.17579
EBNA6	CASP8AP2	0.1444
EBNA6	BOP	0.021556
EBNA6	CLPX	0.15425
EBNA6	FAM117B	0.2897
EBNA6	EIF4G2	0.054778
EBNA6	PHYHIP	0.037305
EBNA6	METTL1	0.17695
EBNA6	MRFAP1	0.26289
EBNA6	MMS19	0.073502
EBNA6	SETD4	0.012575
EBNA6	RPL11	0.07985
EBNA6	EDC3	0.08449
EBNA6	GAGE3	0.061919
EBNA6	MESDC1	0.041189
EBNA6	CCDC30	0.012772
EBNA6	C10orf91	0.26509
EBNA6	CCDC28A	0.27966
EBNA6	TTC22	0.023197
EBNA6	CYBA	0.045145
EBNA6	FAM120C	0.060283
EBNA6	UPF2	0.027019
EBNA6	CCDC65	0.046299
EBNA6	GID4	0.075861
EBNA6	FAM83F	0.14695
EBNA6	FAM101A	0.12834
EBNA6	CKB	0.081341
EBNA6	MAGEB10	0.013664
EBNA6	SNURFL	0.13734
EBNA6	THBD	0.10882
EBNA6	KIAA0040	0.075461
EBNA6	ATG13	0.24973
EBNA6	ENTHD1	0.020544
EBNA6	FAM46D	0.024661
EBNA6	MT2A	0.10556
EBNA6	CLSPN	0.12977
EBNA6	FAM178A	0.15112
EBNA6	MEN1	0.047754
EBNA6	BCKDHB	0.055082
EBNA6	RNMT	0.043791
EBNA6	C12orf36	0.063832
EBNA6	CCDC27	0.013279
EBNA6	MAGOH	0.019676

EBNA6	SRGN	0.17912
EBNA6	EML1	0.17516
EBNA6	OTUD6B	0.034765
EBNA6	NOL3	0.19117
EBNA6	FAM83C	0.11449
EBNA6	IQCK	0.15725
EBNA6	ITFG2	0.028541
EBNA6	HAT1	0.11729
EBNA6	FAM166A	0.19657
EBNA6	NGFRAP1	0.13829
EBNA6	CNTD1	0.014846
EBNA6	PRRC2B	0.25028
EBNA6	MUM1L1	0.1743
EBNA6	FAM74A4	0.049
EBNA6	EPS15	0.069819
EBNA6	PCSK2	0.048514
EBNA6	PRMT8	0.017044
EBNA6	PEX11G	0.15869
EBNA6	AP3S1	0.069348
EBNA6	TTC34	0.010156
EBNA6	SDF4	0.13045
EBNA6	BCKDHA	0.015163
EBNA6	NACAP1	0.0456
EBNA6	TAF11	0.12783
EBNA6	EID3	0.16128
EBNA6	SPACA3	0.071595
EBNA6	STAT1	0.018149
EBNA6	FAT4	0.16922
EBNA6	DTNBP1	0.038524
EBNA6	SDE2	0.035042
EBNA6	PPEF1	0.091586
EBNA6	CXorf67	0.21544
EBNA6	C18orf25	0.11453
EBNA6	NELL2	0.045661
EBNA6	THEGL	0.055289
EBNA6	C11orf35	0.052816
EBNA6	C16orf86	0.066139
EBNA6	ELOVL4	0.10986
EBNA6	FAM188B2	0.23091
EBNA6	DMAP1	0.085916
EBNA6	FAM78A	0.058669
EBNA6	KAT2A	0.01128
EBNA6	MAP1LC3C	0.016983
EBNA6	ATP6V1E1	0.055973
EBNA6	NUDCD3	0.11414
EBNA6	THBS1	0.018175
EBNA6	MAGEF1	0.033509
EBNA6	ARL2BP	0.080859
EBNA6	DEDD2	0.15859
EBNA6	BICD1	0.011843
EBNA6	KIAA1407	0.098792
EBNA6	FAM184B	0.073337
EBNA6	PRMT1	0.027791
EBNA6	CCSER1	0.21085
EBNA6	UNQ6126/PRO20091	0.012015
EBNA6	CCDC144A	0.015778
EBNA6	PNMAL2	0.16445
EBNA6	DDOST	0.086756
EBNA6	AGR3	0.075153
EBNA6	AP1M1	0.026798
EBNA6	YPR022C	0.20864
EBNA6	ADM	0.032527
EBNA6	TPPP	0.12203
EBNA6	EVPLL	0.01096
EBNA6	IL17RE	0.2101
EBNA6	FBXO36	0.012494
EBNA6	CCDC146	0.13776
EBNA6	SYT11	0.21605

EBNA6	CCDC150	0.078974
EBNA6	MAD1L1	0.12069
EBNA6	C1orf105	0.1866
EBNA6	PARPBP	0.11729
EBNA6	ATP6V0D1	0.06018
EBNA6	TPD52L1	0.14391
EBNA6	HSP90B1	0.092246
EBNA6	MACF1	0.11357
EBNA6	CCDC66	0.044874
EBNA6	TNFRSF12A	0.093368
EBNA6	CCDC138	0.019418
EBNA6	C9orf170	0.029463
EBNA6	GLMN	0.13168
EBNA6	CCDC176	0.17148
EBNA6	HEATR2	0.013733
EBNA6	GCH1	0.071247
EBNA6	C10orf67	0.23907
EBNA6	LINC00597	0.15246
EBNA6	FAM131C	0.047249
EBNA6	ZBTB8OS	0.038809
EBNA6	PRDM11	0.015152
EBNA6	NKD2	0.17751
EBNA6	MT1HL1	0.14658
EBNA6	C2orf88	0.050786
EBNA6	IFT57	0.056978
EBNA6	AP5B1	0.11963
EBNA6	TMEM132D	0.17458
EBNA6	NCOR1P1	0.02212
EBNA6	ATP2B2	0.015341
EBNA6	INCENP	0.097468
EBNA6	C19orf71	0.11932
EBNA6	JMJD1C	0.035242
EBNA6	FNBP1L	0.020923
EBNA6	SNX29P2	0.063455
EBNA6	RHPN1-AS1	0.16713
EBNA6	CDKN2AIP	0.11174
EBNA6	C6orf118	0.11454
EBNA6	IL17REL	0.047996
EBNA6	C9orf172	0.017803
EBNA6	CCDC74B	0.13547
EBNA6	FAT1	0.090894
EBNA6	NXF3	0.093677
EBNA6	TGOLN2	0.16325
EBNA6	LINC00471	0.12649
EBNA6	HABP4	0.12695
EBNA6	S100A11	0.14013
EBNA6	GPS2	0.18857
EBNA6	HFE2	0.12793
EBNA6	FAM83E	0.019649
EBNA6	CAB8	0.022241
EBNA6	MSANTD2	0.033297
EBNA6	CCDC137	0.20035
EBNA6	TIFAB	0.25556
EBNA6	C15orf54	0.10697
EBNA6	C16orf95	0.051613
EBNA6	NUTM2A	0.088446
EBNA6	NKD1	0.13321
EBNA6	PLAC8L1	0.068682
EBNA6	CROCCP3	0.045492
EBNA6	GORAB	0.25469
EBNA6	FAM151B	0.072694
EBNA6	GCSAML	0.13638
EBNA6	EIF1AY	0.014932
EBNA6	FNIP2	0.2828
EBNA6	EPSTI1	0.017298
EBNA6	FAM47B	0.13171
EBNA6	MBD3L5	0.026783
EBNA6	BEND4	0.037569

EBNA6	SUPT20HL2	0.15604
EBNA6	PPP2R3B	0.086584
EBNA6	CTNNBIP1	0.09813
EBNA6	ACTN4	0.075758
EBNA6	SPRYD7	0.043171
EBNA6	FBXO46	0.22197
EBNA6	EHD1	0.057457
EBNA6	GTF2E1	0.14888
EBNA6	OXA1L	0.08967
EBNA6	INTS10	0.14942
EBNA6	LINC00523	0.2505
EBNA6	RABGAP1L	0.03322
EBNA6	FAM83G	0.019046
EBNA6	EXOSC1	0.076705
EBNA6	S100A13	0.13988
EBNA6	KIAA2026	0.012467
EBNA6	MELK	0.048618
EBNA6	C9orf152	0.06906
EBNA6	S100A1	0.098131
EBNA6	ARL14EPL	0.22676
EBNA6	SNRPC	0.07159
EBNA6	UTRN	0.06885
EBNA6	GAGE12H	0.12718
EBNA6	INTS4L2	0.045556
EBNA6	PALM	0.083348
EBNA6	SLC34A1	0.14724
EBNA6	FAM219B	0.04445
EBNA6	CXorf21	0.10284
EBNA6	AQP1	0.010338
EBNA6	ELN	0.22573
EBNA6	FAM127A	0.097661
EBNA6	EMD	0.089545
EBNA6	C17orf66	0.04578
EBNA6	CNBD1	0.015157
EBNA6	STAT6	0.016957
EBNA6	LOH12CR1	0.012146
EBNA6	HYAL2	0.012916
EBNA6	PIH1D2	0.16437
EBNA6	C16orf11	0.041635
EBNA6	POM121L12	0.10946
EBNA6	AKAP5	0.061463
EBNA6	CALB1	0.12846
EBNA6	DAZAP2	0.16014
EBNA6	FAM86B1	0.011687
EBNA6	NDEL1	0.044768
EBNA6	MASP2	0.1819
EBNA6	NUDT21	0.05634
EBNA6	PARK7	0.041434
EBNA6	ATG9A	0.029685
EBNA6	LINC00472	0.17381
EBNA6	C20orf96	0.044664
EBNA6	GOLGA6L4	0.20339
EBNA6	GOLGA6L6	0.19157
EBNA6	KIAA1958	0.087913
EBNA6	SLAIN1	0.25929
EBNA6	C9orf169	0.069395
EBNA6	EIF3C	0.057274
EBNA6	YPL025C	0.093342
EBNA6	ZNF326	0.13043
EBNA6	KIAA0226L	0.052377
EBNA6	MYOZ3	0.12811
EBNA6	FAM13C	0.015509
EBNA6	GAS6	0.012493
EBNA6	ANXA2R	0.20328
EBNA6	CC2D2B	0.086505
EBNA6	YML009W-B	0.24689
EBNA6	C14orf178	0.095025
EBNA6	MED9	0.18514

EBNA6	EFEMP1	0.15999
EBNA6	DAXX	0.095795
EBNA6	BID	0.13041
EBNA6	COMMD2	0.15636
EBNA6	C1orf168	0.12135
EBNA6	YTHDF1	0.10991
EBNA6	SPARC	0.077197
EBNA6	FAM179A	0.013701
EBNA6	MRFAP1L1	0.25493
EBNA6	NOTCH4	0.04012
EBNA6	TERT	0.099795
EBNA6	MEAF6	0.085942
EBNA6	CNPY1	0.03034
EBNA6	FAM160A1	0.23204
EBNA6	PCDH17	0.16922
EBNA6	PIR	0.13413
EBNA6	WHAMMP3	0.024147
EBNA6	CCDC85B	0.099121
EBNA6	ALB	0.051024
EBNA6	YJR030C	0.12347
EBNA6	ODF3L2	0.012559
EBNA6	KIAA1841	0.12985
EBNA6	EAF2	0.103
EBNA6	LCA5L	0.021748
EBNA6	C1orf196	0.075054
EBNA6	RNF216P1	0.15995
EBNA6	REPS1	0.15825
EBNA6	DCUN1D2	0.025203
EBNA6	LINC00476	0.039263
EBNA6	STPG2	0.027445
EBNA6	YJEFN3	0.093644
EBNA6	C18orf21	0.099427
EBNA6	LINC00469	0.012465
EBNA6	XAGE2	0.25627
EBNA6	YML009W-B	0.087829
EBNA6	ELOVL3	0.082659
EBNA6	LRCOL1	0.17057
EBNA6	SAT1	0.053695
EBNA6	GOLGA6L18	0.17856
EBNA6	YTHDC1	0.16443
EBNA6	C16orf71	0.033505
EBNA6	GEMIN7	0.11806
EBNA6	TXNIP	0.11039
EBNA6	BCORP1	0.11243
EBNA6	FAM160B2	0.068959
EBNA6	FAM211A	0.012046
EBNA6	GTF3C6	0.13953
EBNA6	WIBG	0.20405
EBNA6	FAM221B	0.1806
EBNA6	NOTCH2	0.017149
EBNA6	MPTX1	0.044754
EBNA6	AMER2	0.1895
EBNA6	STIM1	0.079885
EBNA6	S100B	0.07912
EBNA6	TRAF3IP1	0.22023
EBNA6	CCDC172	0.030833
EBNA6	LDOC1L	0.067359
EBNA6	AC3.5	0.17076
EBNA6	LINS	0.056901
EBNA6	TNNC1	0.15519
EBNA6	SAP30	0.1715
EBNA6	POLRMT	0.10913
EBNA6	TMED2	0.013039
EBNA6	C14orf80	0.20404
EBNA6	NBPF24	0.071001
EBNA6	C1orf94	0.28655
EBNA6	CETN2	0.13224
EBNA6	ANXA6	0.16177

EBNA6	C9orf153	0.062143
EBNA6	TINF2	0.10543
EBNA6	FEZ1	0.047171
EBNA6	ACTN2	0.05823
EBNA6	PRSS2	0.15876
EBNA6	STAT2	0.01701
EBNA6	PSRC1	0.13067
EBNA6	CT45A5	0.091245
EBNA6	PNRC2	0.21315
EBNA6	C2orf78	0.12574
EBNA6	C21orf58	0.10041
EBNA6	CDCA5	0.11266
EBNA6	HAX1	0.10739
EBNA6	PITPNM3	0.15408
EBNA6	VIL1	0.078025
EBNA6	C19orf35	0.17049
EBNA6	C4orf17	0.049741
EBNA6	C9orf117	0.020226
EBNA6	GAGE6	0.061919
EBNA6	YIR021W-A	0.04573
EBNA6	COMMD9	0.10075
EBNA6	KCNJ4	0.045254
EBNA6	NBAS	0.015836
EBNA6	ERLIN1	0.19379
EBNA6	ANGEL2	0.03938
EBNA6	AHSA1	0.13166
EBNA6	MIR7-3HG	0.043603
EBNA6	PP632	0.042723
EBNA6	C17orf104	0.13206
EBNA6	CETN1	0.1673
EBNA6	PRO1854	0.080594
EBNA6	FAM78B	0.023306
EBNA6	GAGE4	0.11453
EBNA6	ATP5SL	0.028063
EBNA6	ZSWIM7	0.18881
EBNA6	C2CD4D	0.047871
EBNA6	ZK1128.3	0.075471
EBNA6	AXDND1	0.01177
EBNA6	YDL022C-A	0.033603
EBNA6	PP632	0.072543
EBNA6	NUP98	0.038803
EBNA6	PNMAL1	0.10712
EBNA6	C18orf42	0.064092
EBNA6	VLDLR	0.068348
EBNA6	NUP62	0.035759
EBNA6	CDH1	0.033706
EBNA6	HTT	0.013625
EBNA6	GAGE13	0.061919
EBNA6	ADAMTS13	0.012168
EBNA6	MAP1LC3B	0.040635
EBNA6	TTN	0.030379
EBNA6	ZDHHC17	0.012692
EBNA6	ZYG11A	0.0204
EBNA6	CXorf30	0.14714
EBNA6	EGF	0.056294
EBNA6	KIAA1755	0.017023
EBNA6	LTBP4	0.13335
EBNA6	CCDC132	0.050241
EBNA6	PRMT2	0.016649
EBNA6	RPS19	0.10025
EBNA6	NPHP4	0.23957
EBNA6	PLSCR4	0.14205
EBNA6	INSIG1	0.070325
EBNA6	DNAJC9-AS1	0.17925
EBNA6	UBQLNL	0.021215
EBNA6	RBM8A	0.01893
EBNA6	CCDC152	0.028758
EBNA6	KHDC1L	0.030325

EBNA6	PRAMEF26	0.017096
EBNA6	FAM45A	0.2283
EBNA6	KIAA1751	0.068107
EBNA6	FBXO25	0.21042
EBNA6	TCP10L2	0.12805
EBNA6	CCDC57	0.019305
EBNA6	SSUH2	0.034221
EBNA6	HBA1	0.11382
EBNA6	C1orf106	0.26115
EBNA6	NOP2	0.021661
EBNA6	GOLGA6L3	0.20339
EBNA6	RHOT1	0.012524
EBNA6	WWC2-AS2	0.1102
EBNA6	GRAMD3	0.011669
EBNA6	SNX9	0.08143
EBNA6	CITED2	0.012924
EBNA6	TTI1	0.13917
EBNA6	FBXL18	0.055906
EBNA6	GUCD1	0.085035
EBNA6	FAM195B	0.089305
EBNA6	CCDC7	0.094569
EBNA6	YFL013W-A	0.11658
EBNA6	RYR2	0.03404
EBNA6	PRF1	0.074796
EBNA6	IL6ST	0.076935
EBNA6	MYO1E	0.043268
EBNA6	GAGE12F	0.12718
EBNA6	ANKRD26P1	0.01651
EBNA6	MZT1	0.13979
EBNA6	PCSK9	0.015061
EBNA6	NELFA	0.20941
EBNA6	BLACE	0.024358
EBNA6	GPRIN3	0.099856
EBNA6	FAM92B	0.03713
EBNA6	SETD2	0.021025
EBNA6	C4orf22	0.048546
EBNA6	C6orf52	0.019339
EBNA6	C9orf141	0.013484
EBNA6	CCDC74A	0.13547
EBNA6	SERBP1	0.13518
EBNA6	ANP32C	0.013714
EBNA6	CDRT4	0.065562
EBNA6	PRR19	0.12517
EBNA6	CCDC67	0.031238
EBNA6	S100A8	0.066039
EBNA6	NID2	0.18178
EBNA6	GAREML	0.27675
EBNA6	MIMI	0.19823
EBNA6	NELL1	0.095881
EBNA6	TICRR	0.19413
EBNA6	SLX1A	0.10062
EBNA6	ytzL	0.26474
EBNA6	WDR96	0.094549
EBNA6	CCDC158	0.010405
EBNA6	ADA	0.016114
EBNA6	DNER	0.10803
EBNA6	COL17A1	0.19308
EBNA6	EIF6	0.059095
EBNA6	L1TD1	0.030862
EBNA6	C15orf65	0.05825
EBNA6	FAM160B1	0.14041
EBNA6	ELOVL6	0.082659
EBNA6	CCDC121	0.017905
EBNA6	SAMD14	0.022688
EBNA6	PEF1	0.1819
EBNA6	OFD1	0.10985
EBNA6	CT45A4	0.059101
EBNA6	RBBP8	0.081658

EBNA6	FBXL8	0.26994
EBNA6	FBL	0.020194
EBNA6	TTYH1	0.20483
EBNA6	NUPR1L	0.10632
EBNA6	MEGF8	0.046875
EBNA6	C10orf82	0.10104
EBNA6	ERICH1-AS1	0.17365
EBNA6	YJR005C-A	0.014218
EBNA6	CHCHD6	0.20002
EBNA6	C3orf65	0.14883
EBNA6	ATXN1	0.079197
EBNA6	CDPF1	0.040943
EBNA6	CHP1	0.070363
EBNA6	CBY3	0.11974
EBNA6	C1orf228	0.067302
EBNA6	CFTR	0.017964
EBNA6	DYDC2	0.046752
EBNA6	EP400NL	0.19178
EBNA6	FAM203B	0.12902
EBNA6	TGM2	0.04118
EBNA6	RPLP0	0.043347
EBNA6	FASN	0.060045
EBNA6	HEATR5A	0.091221
EBNA6	ODF3L1	0.01451
EBNA6	LCMT1	0.051075
EBNA6	EID2	0.14155
EBNA6	GPR98	0.069233
EBNA6	OTUD6A	0.064054
EBNA6	MICU1	0.14611
EBNA6	CALU	0.14396
EBNA6	PPP3CA	0.034206
EBNA6	MTUS2	0.095664
EBNA6	VWA9	0.039957
EBNA6	MYOZ2	0.23599
EBNA6	C16orf72	0.22112
EBNA6	BEND2	0.060798
EBNA6	EIF4B	0.039
EBNA6	LRP2	0.066456
EBNA6	VMP1	0.10874
EBNA6	CAPN2	0.02691
EBNA6	SLC23A1	0.028717
EBNA6	UNC119	0.11203
EBNA6	JMJD7	0.014573
EBNA6	POM121L2	0.10837
EBNA6	GOLGA6L10	0.17856
EBNA6	SAMD13	0.023683
EBNA6	FAM188B	0.22769
EBNA6	MISP	0.23026
EBNA6	FAM231B	0.14862
EBNA6	SELO	0.18902
EBNA6	ZNF728	0.028023
EBNA6	FAM27D1	0.18134
EBNA6	NUCB2	0.11917
EBNA6	UHRF1BP1L	0.15993
EBNA6	SHANK2-AS3	0.071066
EBNA6	FAM194B	0.026543
EBNA6	UNQ6126/PRO20091	0.07811
EBNA6	TSGA13	0.010057
EBNA6	NPC2	0.13253
EBNA6	HIN1L	0.017218
EBNA6	ATXN7L2	0.072672
EBNA6	PSMD5	0.08687
EBNA6	PEX11A	0.15129
EBNA6	YGL014C-A	0.199
EBNA6	S100A6	0.11452
EBNA6	TERF2IP	0.12343
EBNA6	LRP8	0.1249
EBNA6	CCDC151	0.03646

EBNA6	SYNPO	0.14609
EBNA6	MBD3L2	0.01955
EBNA6	IGFLR1	0.15112
EBNA6	CT55	0.12247
EBNA6	S100P	0.11774
EBNA6	ZNF815P	0.011503
EBNA6	SERTAD1	0.20743
EBNA6	RWDD4	0.077443
EBNA6	EGLN2	0.060057
EBNA6	PRDX6	0.028851
EBNA6	CHAF1A	0.25997
EBNA6	SETD1B	0.020415
EBNA6	PTGES3L	0.072341
EBNA6	TP53INP1	0.15514
EBNA6	RUNDC3B	0.040337
EBNA6	MNCb-2875	0.063092
EBNA6	FAM127C	0.099015
EBNA6	PTTG1	0.055424
EBNA6	CLUAP1	0.25445
EBNA6	NUP160	0.066615
EBNA6	RAB11FIP3	0.055475
EBNA6	EFCAB4B	0.12852
EBNA6	ALKBH8	0.015255
EBNA6	SYBU	0.063838
EBNA6	C8orf74	0.088432
EBNA6	CCDC104	0.061592
EBNA6	ARL6IP1	0.042224
EBNA6	XAGE3	0.2897
EBNA6	TEX36	0.052971
EBNA6	C11orf65	0.051229
EBNA6	ASAH2B	0.016654
EBNA6	CCDC85A	0.01239
EBNA6	C1orf111	0.13898
EBNA6	TCL1B	0.016999
EBNA6	CCDC87	0.066028
EBNA6	CHGB	0.29153
EBNA6	JMJD8	0.013864
EBNA6	DTNA	0.090065
EBNA6	REPS2	0.19011
EBNA6	PPEF2	0.081518
EBNA6	EFEMP2	0.17975
EBNA6	SART1	0.10223
EBNA6	MAGED4	0.019073
EBNA6	ATP2A1	0.052535
EBNA6	ATXN7L3B	0.098649
EBNA6	SCAMP5	0.067321
EBNA6	WLS	0.065831
EBNA6	OCEL1	0.024665
EBNA6	CT45A3	0.065517
EBNA6	MAGEB3	0.013165
EBNA6	CAGE1	0.030292
EBNA6	LINC00205	0.12386
EBNA6	PKD2L1	0.094317
EBNA6	FAM46B	0.11639
EBNA6	PNAS-138	0.14488
EBNA6	CD2BP2	0.085572
EBNA6	ACOT8	0.099793
EBNA6	NCOA6	0.15732
EBNA6	HTN1	0.20418
EBNA6	FAM183B	0.012855
EBNA6	CCDC26	0.12981
EBNA6	EHD3	0.07241
EBNA6	TGM1	0.035847
EBNA6	FAM181A	0.072241
EBNA6	SUPT20HL1	0.15604
EBNA6	SLC14A2	0.10575
EBNA6	C7orf57	0.057995
EBNA6	LY96	0.11219

EBNA6	C22orf43	0.12302
EBNA6	RPS2	0.042392
EBNA6	FAM166B	0.04982
EBNA6	VWA5B2	0.093225
EBNA6	THBS2	0.15026
EBNA6	NAF1	0.19192
EBNA6	PRAME	0.22592
EBNA6	CALM1	0.079786
EBNA6	EIF4E2	0.025018
EBNA6	MAP7D2	0.064144
EBNA6	S100A4	0.17763
EBNA6	S100A12	0.092962
EBNA6	FAM149B1	0.13179
EBNA6	RCN1	0.1874
EBNA6	KCNJ6	0.016598
EBNA6	DPP4	0.025039
EBNA6	EIF4E	0.034536
EBNA6	STAG3L4	0.067909
EBNA6	BBC3	0.13967
EBNA6	DNMT3A	0.010651
EBNA6	RQCD1	0.1414
EBNA6	NPB	0.19718
EBNA6	ACTN3	0.16964
EBNA6	ATP5F1	0.039873
EBNA6	FBN1	0.17049
EBNA6	MIF4GD	0.13987
EBNA6	SPAC22F8.05	0.020797
EBNA6	C12orf63	0.043435
EBNA6	F10	0.085531
EBNA6	CAPN1	0.07161
EBNA6	UCKL1	0.069969
EBNA6	DPM2	0.16067
EBNA6	FAM46A	0.13824
EBNA6	CCNYL3	0.023915
EBNA6	TSN	0.10996
EBNA6	CELSR3	0.10332
EBNA6	RILP	0.14901
EBNA6	SS18L2	0.053153
EBNA6	SDHA	0.031767
EBNA6	GAGE12J	0.061919
EBNA6	N6AMT1	0.035261
EBNA6	DEHA2C16280g	0.11253
EBNA6	IGFL1	0.24263
EBNA6	IGBP1	0.12835
EBNA6	KHNYN	0.089848
EBNA6	SIRT4	0.069556
EBNA6	TESC	0.10666
EBNA6	GAGE2A	0.12718
EBNA6	ATXN7L1	0.072672
EBNA6	MICAL1	0.019531
EBNA6	LSM12	0.12283
EBNA6	UCN	0.065891
EBNA6	KIAA0907	0.02971
EBNA6	C12orf57	0.072528
EBNA6	SETD1A	0.019475
EBNA6	FAM228A	0.027111
EBNA6	YAE1D1	0.13242
EBNA6	CT45A1	0.070825
EBNA6	ERICH1	0.14482
EBNA6	C21orf37	0.010461
EBNA6	RPH3A	0.079971
EBNA6	SRI	0.090138
EBNA6	PPP4R2	0.19736
EBNA6	LRP4	0.094892
EBNA6	BVES-AS1	0.095374
EBNA6	TMSB4X	0.19691
EBNA6	CCDC127	0.10005
EBNA6	CCDC120	0.16564

EBNA6	MAGEB16	0.023334
EBNA6	SLIT2	0.034937
EBNA6	C1orf145	0.15893
EBNA6	OTUD1	0.050482
EBNA6	SFI1	0.14602
EBNA6	MATN2	0.14875
EBNA6	SAMD9L	0.016786
EBNA6	CIB1	0.13056
EBNA6	PPP2R3A	0.11394
EBNA6	AKAP3	0.1052
EBNA6	EIF2B4	0.030046
EBNA6	DLL1	0.13674
EBNA6	ACTN1	0.082688
EBNA6	CD164	0.14343
EBNA6	BLOC1S1	0.1903
EBNA6	FAM195A	0.27243
EBNA6	RGAG1	0.060609
EBNA6	PNMA6A	0.037748
EBNA6	C9orf62	0.032859
EBNA6	EHD4	0.045329
EBNA6	PNPT1	0.064132
EBNA6	UPF3B	0.19878
EBNA6	MYO6	0.012674
EBNA6	C5orf58	0.17949
EBNA6	LINC00114	0.029986
EBNA6	PMAIP1	0.1008
EBNA6	PRSS3	0.18519
EBNA6	CCDC169	0.079326
EBNA6	C7orf62	0.14472
EBNA6	MYL2	0.17614
EBNA6	EDEM1	0.093087
EBNA6	LRP1	0.043324
EBNA6	MB21D2	0.071664
EBNA6	ARMCX5	0.065487
EBNA6	LSM11	0.1623
EBNA6	ATP5A1	0.041071
EBNA6	ADAR	0.013073
EBNA6	C19orf48	0.034578
EBNA6	SNX19	0.11452
EBNA6	YMR001C-A	0.016254
EBNA6	C7orf71	0.014319
EBNA6	MRE11A	0.028469
EBNA6	CLPP	0.12467
EBNA6	AAR2	0.010883
EBNA6	PDGFRB	0.021138
EBNA6	SPATA2L	0.11387
EBNA6	ITSN1	0.037255
EBNA6	C1orf189	0.020908
EBNA6	C15orf37	0.030709
EBNA6	BBS1	0.096034
EBNA6	BTF3L4	0.06945
EBNA6	SPIDR	0.21189
EBNA6	ELOVL1	0.082659
EBNA6	DST	0.088015
EBNA6	MYL6	0.16778
EBNA6	COMP	0.12852
EBNA6	CCDC47	0.14678
EBNA6	XAGE5	0.18267
EBNA6	ANXA7	0.082339
EBNA6	CHP2	0.13724
EBNA6	LYRM9	0.19779
EBNA6	MUC1	0.18999
EBNA6	FAM127B	0.14561
EBNA6	PLN	0.17808
EBNA6	CDK5R1	0.073017
EBNA6	CXorf40B	0.051135
EBNA6	C16orf96	0.02201
EBNA6	C6orf211	0.010427

EBNA6	Gm3414	0.011248
EBNA6	NECAB2	0.19202
EBNA6	ARMC5	0.017368
EBNA6	LINC00574	0.011513
EBNA6	CANX	0.080978
EBNA6	RHOT2	0.012796
EBNA6	SGD1	0.11345
EBNA6	MMP15	0.039924
EBNA6	FHAD1	0.01297
EBNA6	FBXO34	0.044033
EBNA6	GADD45A	0.096934
EBNA6	BSDC1	0.0291
EBNA6	ANXA11	0.085495
EBNA6	PPP3CB	0.038274
EBNA6	GOLGA6L19	0.17856
EBNA6	C8orf88	0.1049
EBNA6	C5orf34	0.079614
EBNA6	LURAP1L	0.24777
EBNA6	SHCBP1L	0.039842
EBNA6	C19orf47	0.080938
EBNA6	C4orf33	0.033264
EBNA6	C12orf50	0.20656
EBNA6	C17orf102	0.098548
EBNA6	IQGAP1	0.027145
EBNA6	RMND5B	0.028134
EBNA6	IQCG	0.090604
EBNA6	MATN3	0.19515
EBNA6	MAGEC3	0.014942
EBNA6	PNMA6C	0.061082
EBNA6	FAM47C	0.11648
EBNA6	SH3KBP1	0.033506
EBNA6	AMOTL1	0.089951
EBNA6	C1orf173	0.041869
EBNA6	TJAP1	0.2097
EBNA6	SCHIP1	0.2257
EBNA6	RET	0.067874
EBNA6	PAGE3	0.12444
EBNA6	PATL1	0.10157
EBNA6	FAM43B	0.017592
EBNA6	PARP10	0.085582
EBNA6	MAGED2	0.019814
EBNA6	TLX1NB	0.045372
EBNA6	CDH23	0.059586
EBNA6	LCA10	0.14011
EBNA6	STATH	0.25652
EBNA6	RAB11FIP4	0.22172
EBNA6	CNGC19	0.21675
EBNA6	C9orf106	0.037429
EBNA6	DYDC1	0.033285
EBNA6	CCDC102B	0.019027
EBNA6	CNRIP1	0.035299
EBNA6	PRPF3	0.10888
EBNA6	ATP6V1H	0.034128
EBNA6	PLSCR5	0.017955
EBNA6	C5orf24	0.014067
EBNA6	TCERG1L	0.086951
EBNA6	SUV39H2	0.022651
EBNA6	C6orf223	0.27871
EBNA6	C1orf64	0.13308
EBNA6	MBOAT7	0.090656
EBNA6	PACRGL	0.047923
EBNA6	TCTEX1D1	0.056178
EBNA6	CLU	0.040008
EBNA6	NPIPB11	0.010249
EBNA6	C1orf140	0.19376
EBNA6	YGL006W-A	0.04348
EBNA6	GOLGA6L20	0.11347
EBNA6	LINC00083	0.013394

EBNA6	LINC00588	0.16318
EBNA6	HYPK	0.043854
EBNA6	RUNDC1	0.043576
EBNA6	WIPF2	0.12547
EBNA6	PLCE1	0.017778
EBNA6	NIPSNAP3B	0.034983
EBNA6	STX6	0.099336
EBNA6	SZRD1	0.09214
EBNA6	SNW1	0.078814
EBNA6	AC3.5	0.15309
EBNA6	DDX26B	0.23298
EBNA6	LCHN	0.080918
EBNA6	FAM45B	0.17017
EBNA6	FAM229B	0.15347
EBNA6	LINC00478	0.055719
EBNA6	C2orf81	0.16879
EBNA6	FAM84A	0.062578
EBNA6	SPANXA2-OT1	0.11536
EBNA6	ATG2B	0.01846
EBNA6	CLIC4	0.062888
EBNA6	EIF4G1	0.020846
EBNA6	FAM49B	0.063768
EBNA6	BNIP2	0.080935
EBNA6	ZNF767	0.093633
EBNA6	FAM199X	0.07039
EBNA6	OSER1	0.01762
EBNA6	JAG2	0.11166
EBNA6	FAM90A2P	0.0217
EBNA6	CCDC114	0.028233
EBNA6	HDGFL1	0.013199
EBNA6	SWT1	0.094579
EBNA6	GAGE12G	0.12718
EBNA6	VIMP	0.0893
EBNA6	FAM117A	0.17598
EBNA6	TIPRL	0.13275
EBNA6	CYorf17	0.020478
EBNA6	EHD2	0.024556
EBNA6	CCDC13	0.01508
EBNA6	CADPS	0.19175
EBNA6	NCAPH	0.15006
EBNA6	C17orf59	0.30813
EBNA6	DRC1	0.097366
EBNA6	CRYGD	0.12781
EBNA6	FAM161B	0.26879
EBNA6	CCAR1	0.15071
EBNA6	C3orf55	0.021095
EBNA6	C19orf57	0.16192
EBNA6	C16orf93	0.082286
EBNA6	KIAA1908	0.061073
EBNA6	C20orf62	0.041675
EBNA6	C6orf222	0.069246
EBNA6	LINC00299	0.021905
EBNA6	CCDC171	0.077679
EBNA6	CCAR2	0.11069
EBNA6	PLAUR	0.11312
EBNA6	FAM178B	0.12777
EBNA6	GTF2A1	0.11265
EBNA6	COMMD6	0.2214
EBNA6	MIMI	0.020036
EBNA6	GABARAP	0.023915
EBNA6	C1orf195	0.28105
EBNA6	CRELD2	0.17598
EBNA6	MYOZ1	0.16726
EBNA6	TIMELESS	0.02606
EBNA6	NPIPA5	0.022926
EBNA6	PRR23A	0.13927
EBNA6	C15orf32	0.020023
EBNA6	C2orf72	0.16226

EBNA6	YBR056C-B	0.092403
EBNA6	EME1	0.12541
EBNA6	C11orf86	0.030014
EBNA6	NBR1	0.01712
EBNA6	SCIMP	0.23866
EBNA6	ARMC12	0.106
EBNA6	DERL1	0.083711
EBNA6	GSN	0.07308
EBNA6	SLC11A2	0.023121
EBNA6	TRMT112	0.065846
EBNA6	PAIP2	0.14507
EBNA6	PLAC8	0.028627
EBNA6	C3orf22	0.13517
EBNA6	EXOC3L4	0.11612
EBNA6	CYCB3-1	0.20508
EBNA6	COA7	0.046999
EBNA6	EIF4A1	0.013856
EBNA6	CABP1	0.038223
EBNA6	EAF1	0.15729
EBNA6	BEND3	0.015613
EBNA6	SLC4A4	0.042084
EBNA6	C22orf26	0.16835
EBNA6	LETM1	0.16999
EBNA6	TNFAIP8L1	0.2225
EBNA6	pkmyt1	0.12668
EBNA6	PGBD4	0.08084
EBNA6	SPHK1	0.019127
EBNA6	CRYGN	0.10384
EBNA6	CTU1	0.11988
EBNA6	FAM231A	0.44187
EBNA6	TFB1M	0.049218
EBNA6	C6orf229	0.11578
EBNA6	FAM124A	0.29514
EBNA6	AMER1	0.19517
EBNA6	PNRC1	0.21714
EBNA6	MAL	0.065486
EBNA6	FAM188A	0.14235
EBNA6	MAGEB4	0.027951
EBNA6	AP1B1	0.038289
EBNA6	RCN3	0.18563
EBNA6	PLSCR1	0.11106
EBNA6	GAGE5	0.061919
EBNA6	SPAC5H10.02c	0.017544
EBNA6	GAGE12B	0.12718
EBNA6	SLC6A4	0.019472
EBNA6	SLC12A3	0.03773
EBNA6	PSENEN	0.10752
EBNA6	ATN1	0.089172
EBNA6	ATG5	0.071099
EBNA6	CAMLG	0.10785
EBNA6	NKAPL	0.19041
EBNA6	NCKAP5L	0.20243
EBNA6	CCDC153	0.019646
EBNA6	PRR21	0.080885
EBNA6	TLDC2	0.14339
EBNA6	LTBP1	0.18056
EBNA6	C6orf183	0.011914
EBNA6	C6orf165	0.016161
EBNA6	ASPSCR1	0.12382
EBNA6	MSANTD1	0.050284
EBNA6	F2	0.044101
EBNA6	IFIT2	0.10677
EBNA6	SMEK3P	0.10643
EBNA6	MIEF2	0.22505
EBNA6	SYT1	0.065881
EBNA6	C11orf52	0.095429
EBNA6	NSUN5P1	0.083319
EBNA6	PI4K2A	0.036925

EBNA6	NCS1	0.15364
EBNA6	TCAP	0.11283
EBNA6	C12orf71	0.044255
EBNA6	SRRM5	0.05546
EBNA6	CCDC81	0.070642
EBNA6	DAG1	0.077928
EBNA6	AGTRAP	0.14771
EBNA6	C1orf192	0.098238
EBNA6	MED30	0.031709
EBNA6	HRASLS5	0.014673
EBNA6	HRK	0.2097
EBNA6	C2orf62	0.074541
EBNA6	SCAI	0.13462
EBNA6	NIPBL	0.019086
EBNA6	MAGEB2	0.012991
EBNA6	YAL042C-A	0.044556
EBNA6	C19orf67	0.068969
EBNA6	C5orf48	0.17602
EBNA6	SH3BP5L	0.15296
EBNA6	CCDC17	0.035964
EBNA6	NECAB3	0.11566
EBNA6	MLC1	0.040959
EBNA6	EFNA4	0.019651
EBNA6	C22orf42	0.035422
EBNA6	C19orf82	0.14321
EBNA6	SUMO4	0.019843
EBNA6	TRMT61B	0.10108
EBNA6	SNX6	0.046233
EBNA6	SECISBP2L	0.17551
EBNA6	C11orf96	0.17767
EBNA6	CDKN2AIPNL	0.21801
EBNA6	CHMP5	0.071899
EBNA6	INTS4L1	0.031891
EBNA6	FAM92A1	0.032714
EBNA6	NACA	0.051436
EBNA6	NPC1L1	0.057366
EBNA6	AASDHPPT	0.1662
EBNA6	NBEAP1	0.016366
EBNA6	RSRC1	0.23721
EBNA6	MEGF6	0.18248
EBNA6	CALR	0.018878
EBNA6	RSRC2	0.21966
EBNA6	MRPL11	0.16329
EBNA6	NUP205	0.088538
EBNA6	GAS1	0.076063
EBNA6	C16orf45	0.053615
EBNA6	GOLGA6L9	0.17856
EBNA6	ALS2CR11	0.075832
EBNA6	ZNRD1-AS1	0.044622
EBNA6	OAF	0.01888
EBNA6	KCNIP2	0.066928
EBNA6	FBRSL1	0.12683
EBNA6	FSCN1	0.06958
EBNA6	LRRC14	0.026244
EBNA6	ZK1128.3	0.23155
EBNA6	CUEDC1	0.10664
EBNA6	PLCG1	0.01016
EBNA6	HEATR5B	0.1259
EBNA6	PDCD4	0.091827
EBNA6	CTAGE15	0.031639
EBNA6	LINC00303	0.049441
EBNA6	CD93	0.1231
EBNA6	CCDC54	0.038047
EBNA6	GRIN1	0.058991
EBNA6	FAN1	0.13189
EBNA6	CLINT1	0.023694
EBNA6	C20orf195	0.084234
EBNA6	C19orf66	0.14929

EBNA6	DHH	0.12548
EBNA6	GAGE2E	0.061919
EBNA6	UNQ6125/PRO20090	0.20862
EBNA6	SPHK2	0.033434
EBNA6	TTC29	0.018143
EBNA6	VIPAS39	0.070784
EBNA6	CT45A2	0.065517
EBNA6	N4BP2L1	0.034185
EBNA6	TEX26	0.013307
EBNA6	FAM231D	0.20821
EBNA6	SAC3D1	0.12775
EBNA6	MAGEB17	0.028148
EBNA6	XPO1	0.021031
EBNA6	KIAA0087	0.16299
EBNA6	C11orf72	0.067281
EBNA6	C9orf171	0.1878
EBNA6	IQCF3	0.035517
EBNA6	LINC00615	0.13977
EBNA6	STT3A	0.046591
EBNA6	CCDC94	0.18874
EBNA6	MYL6B	0.14441
EBNA6	CCDC122	0.011577
EBNA6	RBBP8NL	0.067169
EBNA6	RGAG4	0.12318
EBNA6	ATP6V0A1	0.032294
EBNA6	SCG5	0.21955
EBNA6	TTC23L	0.045174
EBNA6	MED4	0.10472
EBNA6	AP1G1	0.033503
EBNA6	CDHR2	0.155
EBNA6	CDR2L	0.045765
EBNA6	SNX17	0.019468
EBNA6	CDH5	0.10117
EBNA6	GR6	0.082509
EBNA6	IL6R	0.033583
EBNA6	TP53INP2	0.11355
EBNA6	SMN1	0.060136
EBNA6	SYT2	0.15339
EBNA6	CAPNS1	0.054699
EBNA6	GTF2E2	0.20229
EBNA6	C3orf14	0.011394
EBNA6	TPD52	0.17979
gB	ubl4ab	0.034648
gB	SPTA1	0.010505
gB	S100A7	0.039551
gB	HSPA5	0.02235
gB	LDLR	0.01596
gB	C12orf61	0.030655
gB	PLSCR3	0.047373
gB	YMR013W-A	0.016103
gB	ATP5B	0.03899
gB	NIN	0.038362
gB	KCNIP1	0.047457
gB	STIM2	0.052164
gB	SPTAN1	0.025468
gB	MASP1	0.11078
gB	SCUBE3	0.068116
gB	PROC	0.090876
gB	PDCD6	0.10124
gB	S100A9	0.015221
gB	ANXA2	0.036922
gB	MATN4	0.067367
gB	RNH1	0.047397
gB	CLIP1	0.021274
gB	FAM83F	0.012389
gB	THBD	0.036666
gB	EML1	0.06349
gB	SDF4	0.050378

gB	FAT4	0.054338
gB	CXorf67	0.0353
gB	FAM188B2	0.031679
gB	SYT11	0.10795
gB	HSP90B1	0.040145
gB	MACF1	0.04752
gB	CCDC176	0.031539
gB	GCH1	0.014596
gB	NKD2	0.09518
gB	FAT1	0.020596
gB	S100A11	0.048071
gB	CCDC137	0.027798
gB	NKD1	0.045877
gB	ACTN4	0.025086
gB	S100A13	0.074476
gB	S100A1	0.014666
gB	CALB1	0.058517
gB	MASP2	0.093724
gB	ANXA2R	0.028101
gB	EFEMP1	0.079727
gB	SPARC	0.033177
gB	PCDH17	0.054338
gB	KIAA1841	0.017121
gB	REPS1	0.045423
gB	LRCOL1	0.029627
gB	MPTX1	0.010517
gB	STIM1	0.016372
gB	S100B	0.019062
gB	TNNC1	0.068878
gB	CETN2	0.074568
gB	ANXA6	0.062638
gB	ACTN2	0.024874
gB	PRSS2	0.081144
gB	PITPNM3	0.053879
gB	VIL1	0.036029
gB	CETN1	0.088637
gB	VLDLR	0.033357
gB	CDH1	0.015798
gB	TTN	0.012816
gB	EGF	0.020861
gB	PLSCR4	0.04435
gB	SNX9	0.025824
gB	IL6ST	0.041779
gB	S100A8	0.025852
gB	NID2	0.078783
gB	NELL1	0.021435
gB	ytzL	0.035895
gB	WDR96	0.012272
gB	DNER	0.030615
gB	PEF1	0.06838
gB	C10orf82	0.014335
gB	CHP1	0.03475
gB	FASN	0.028371
gB	GPR98	0.014131
gB	MICU1	0.049654
gB	CALU	0.054134
gB	BEND2	0.010153
gB	LRP2	0.026395
gB	FAM188B	0.037782
gB	NUCB2	0.038323
gB	S100A6	0.043675
gB	LRP8	0.059496
gB	CT55	0.028483
gB	S100P	0.016951
gB	EFCAB4B	0.037253
gB	REPS2	0.074038
gB	PPEF2	0.018465
gB	EFEMP2	0.069518

gB	ATP2A1	0.018089
gB	PKD2L1	0.030994
gB	THBS2	0.049661
gB	CALM1	0.041713
gB	S100A4	0.077321
gB	S100A12	0.033218
gB	RCN1	0.074717
gB	ACTN3	0.077391
gB	FBN1	0.078739
gB	F10	0.021473
gB	CELSR3	0.023516
gB	TESC	0.050203
gB	LSM12	0.014846
gB	C12orf57	0.018714
gB	RPH3A	0.016898
gB	SRI	0.022752
gB	LRP4	0.032737
gB	SLIT2	0.016783
gB	MATN2	0.061286
gB	CIB1	0.046962
gB	PPP2R3A	0.02648
gB	DLL1	0.075198
gB	ACTN1	0.031135
gB	PRSS3	0.096719
gB	MYL2	0.099786
gB	EDEM1	0.015289
gB	SNX19	0.018131
gB	DST	0.045702
gB	MYL6	0.073085
gB	COMP	0.049395
gB	CCDC47	0.045992
gB	CHP2	0.047257
gB	CANX	0.02677
gB	ANXA11	0.019742
gB	MATN3	0.082165
gB	FAM47C	0.01769
gB	RET	0.030269
gB	RAB11FIP4	0.13579
gB	C1orf64	0.019639
gB	AC3.5	0.017474
gB	C2orf81	0.017675
gB	CLIC4	0.021732
gB	FAM49B	0.01584
gB	JAG2	0.046462
gB	CADPS	0.10142
gB	CCAR1	0.073375
gB	C1orf195	0.043126
gB	CRELD2	0.064233
gB	YBR056C-B	0.013359
gB	GSN	0.025376
gB	SLC11A2	0.012362
gB	LETM1	0.05496
gB	FAM231A	0.07041
gB	FAM124A	0.027148
gB	FAM188A	0.033611
gB	RCN3	0.066969
gB	PLSCR1	0.02527
gB	NKAPL	0.032212
gB	TLDC2	0.01996
gB	LTBP1	0.074685
gB	F2	0.013169
gB	SYT1	0.022709
gB	NSUN5P1	0.014776
gB	NCS1	0.07723
gB	DAG1	0.013487
gB	C5orf48	0.011955
gB	NECAB3	0.023248
gB	MEGF6	0.067095

gB	HEATR5B	0.022553
gB	CD93	0.041699
gB	GRIN1	0.022153
gB	DHH	0.051341
gB	N4BP2L1	0.011224
gB	FAM231D	0.027384
gB	C11orf72	0.011727
gB	MYL6B	0.049265
gB	CDHR2	0.050305
gB	CDH5	0.027253
gB	SYT2	0.067308
gB	TPD52	0.07903
gL	ubl4ab	0.14594
gL	MED1	0.013496
gL	GAGE12I	0.070023
gL	PAGE2B	0.20296
gL	S100A7	0.040035
gL	HSPA5	0.022494
gL	LDLR	0.016675
gL	UPF3A	0.15042
gL	RABEP1	0.13771
gL	C12orf61	0.12907
gL	FAM86B2	0.020089
gL	PNMA6D	0.030257
gL	PLSCR3	0.095618
gL	ZC3HAV1L	0.12468
gL	BTBD16	0.028192
gL	C9orf40	0.017916
gL	RPUSD4	0.081987
gL	CCDC159	0.1048
gL	C10orf68	0.096724
gL	CCDC42	0.018249
gL	CCDC162P	0.020408
gL	GOLGA6L1	0.048252
gL	KRI1	0.15578
gL	SPATA1	0.031246
gL	CCDC69	0.058454
gL	YMR013W-A	0.13167
gL	LEKR1	0.010847
gL	CASC3	0.16282
gL	CYP4F30P	0.017987
gL	ATP5B	0.041103
gL	NIN	0.18768
gL	KCNIP1	0.048685
gL	STIM2	0.05662
gL	HUS1B	0.08636
gL	PSMG3	0.012806
gL	ANGEL1	0.054457
gL	C19orf44	0.050715
gL	C15orf40	0.014454
gL	FSIP1	0.14662
gL	SPTAN1	0.024908
gL	TSGA10IP	0.1126
gL	TNFAIP8L3	0.040285
gL	MASP1	0.11775
gL	SCUBE3	0.069208
gL	SPBC1539.02	0.078383
gL	GLTSCR1L	0.19256
gL	MAB21L3	0.022988
gL	SYT14L	0.011006
gL	PROC	0.096289
gL	PDCD6	0.10738
gL	S100A9	0.014994
gL	ANXA2	0.037393
gL	smf	0.030036
gL	MAGEB1	0.015654
gL	MATN4	0.068049
gL	RNH1	0.046195

gL	UBAP2	0.16971
gL	C17orf105	0.062343
gL	RRP7B	0.011553
gL	FAM110D	0.14032
gL	CLIP1	0.020798
gL	AMOT	0.036596
gL	CCDC154	0.038158
gL	PAGE5	0.10211
gL	GAGE10	0.12611
gL	BOP	0.015632
gL	FAM117B	0.20296
gL	PHYHIP	0.029324
gL	GAGE3	0.037989
gL	MESDC1	0.037044
gL	C10orf91	0.17948
gL	CCDC28A	0.1955
gL	TTC22	0.017261
gL	FAM120C	0.034977
gL	CCDC65	0.021307
gL	GID4	0.064932
gL	FAM83F	0.12039
gL	FAM101A	0.096
gL	SNURFL	0.081378
gL	THBD	0.039219
gL	KIAA0040	0.034537
gL	ENTHD1	0.010461
gL	FAM178A	0.11824
gL	MEN1	0.036407
gL	C12orf36	0.042054
gL	EML1	0.064128
gL	OTUD6B	0.019962
gL	FAM83C	0.071593
gL	IQCK	0.11216
gL	ITFG2	0.02055
gL	FAM166A	0.13423
gL	MUM1L1	0.10763
gL	FAM74A4	0.042871
gL	AP3S1	0.026496
gL	SDF4	0.051243
gL	NACAP1	0.032977
gL	FAT4	0.058111
gL	DTNBP1	0.037458
gL	SDE2	0.012639
gL	CXorf67	0.17574
gL	C18orf25	0.07007
gL	C11orf35	0.020044
gL	C16orf86	0.035233
gL	FAM188B2	0.22232
gL	NUDCD3	0.087799
gL	MAGEF1	0.022936
gL	KIAA1407	0.06498
gL	FAM184B	0.054804
gL	CCSER1	0.15281
gL	CCDC144A	0.015156
gL	PNMAL2	0.1032
gL	YPR022C	0.15976
gL	TPPP	0.038219
gL	CCDC146	0.074497
gL	SYT11	0.12173
gL	CCDC150	0.056211
gL	C1orf105	0.14567
gL	HSP90B1	0.041334
gL	MACF1	0.049095
gL	CCDC66	0.020271
gL	CCDC138	0.013255
gL	CCDC176	0.15433
gL	GCH1	0.059384
gL	C10orf67	0.14982

gL	LINC00597	0.097749
gL	FAM131C	0.026882
gL	ZBTB8OS	0.013646
gL	PRDM11	0.012224
gL	NKD2	0.10108
gL	MT1HL1	0.078892
gL	C2orf88	0.022489
gL	NCOR1P1	0.01543
gL	C19orf71	0.098708
gL	SNX29P2	0.057863
gL	RHPN1-AS1	0.1276
gL	C6orf118	0.07084
gL	IL17REL	0.028763
gL	CCDC74B	0.067492
gL	FAT1	0.020353
gL	LINC00471	0.084951
gL	S100A11	0.048096
gL	CAB8	0.015793
gL	MSANTD2	0.014347
gL	CCDC137	0.19206
gL	TIFAB	0.17126
gL	C15orf54	0.049179
gL	NUTM2A	0.051785
gL	NKD1	0.046347
gL	PLAC8L1	0.041419
gL	CROCCP3	0.033807
gL	FAM151B	0.04952
gL	GCSAML	0.10904
gL	FNIP2	0.22822
gL	FAM47B	0.11108
gL	MBD3L5	0.018068
gL	BEND4	0.010415
gL	SUPT20HL2	0.11166
gL	PPP2R3B	0.014053
gL	ACTN4	0.02479
gL	SPRYD7	0.03517
gL	LINC00523	0.17362
gL	RABGAP1L	0.026309
gL	S100A13	0.078592
gL	C9orf152	0.023228
gL	S100A1	0.01167
gL	ARL14EPL	0.17656
gL	GAGE12H	0.070023
gL	INTS4L2	0.023401
gL	SLC34A1	0.13847
gL	FAM219B	0.018494
gL	CXorf21	0.065689
gL	ELN	0.16067
gL	FAM127A	0.051687
gL	C17orf66	0.043037
gL	CNBD1	0.010944
gL	PIH1D2	0.12585
gL	POM121L12	0.096892
gL	CALB1	0.060717
gL	NDEL1	0.041403
gL	MASP2	0.098772
gL	PARK7	0.016336
gL	LINC00472	0.1238
gL	C20orf96	0.026947
gL	GOLGA6L4	0.13843
gL	GOLGA6L6	0.13053
gL	KIAA1958	0.06969
gL	SLAIN1	0.19495
gL	C9orf169	0.042578
gL	YPL025C	0.048298
gL	ZNF326	0.10096
gL	KIAA0226L	0.01851
gL	ANXA2R	0.17323

gL	CC2D2B	0.055753
gL	YML009W-B	0.18643
gL	C14orf178	0.069442
gL	EFEMP1	0.082709
gL	C1orf168	0.090416
gL	YTHDF1	0.081849
gL	SPARC	0.033762
gL	MRFAP1L1	0.1715
gL	CNPY1	0.019289
gL	FAM160A1	0.17504
gL	PCDH17	0.058111
gL	WHAMMP3	0.019354
gL	YJR030C	0.11851
gL	KIAA1841	0.1171
gL	C1orf196	0.066861
gL	RNF216P1	0.095488
gL	REPS1	0.044601
gL	DCUN1D2	0.016589
gL	LINC00476	0.020004
gL	STPG2	0.023103
gL	YJEFN3	0.061699
gL	C18orf21	0.064089
gL	XAGE2	0.18086
gL	YML009W-B	0.075338
gL	LRCOL1	0.15712
gL	GOLGA6L18	0.11631
gL	TXNIP	0.11365
gL	BCORP1	0.08419
gL	FAM160B2	0.034395
gL	FAM211A	0.010593
gL	WIBG	0.13919
gL	FAM221B	0.11346
gL	MPTX1	0.03965
gL	STIM1	0.015688
gL	S100B	0.017735
gL	CCDC172	0.026869
gL	LDOC1L	0.038783
gL	AC3.5	0.088688
gL	LINS	0.048205
gL	TNNC1	0.071335
gL	C14orf80	0.14065
gL	NBPF24	0.034672
gL	CETN2	0.0791
gL	ANXA6	0.063775
gL	C9orf153	0.035173
gL	ACTN2	0.025408
gL	PRSS2	0.085398
gL	CT45A5	0.047797
gL	C2orf78	0.067863
gL	C21orf58	0.058475
gL	PITPNM3	0.058261
gL	VIL1	0.037269
gL	C19orf35	0.11827
gL	C4orf17	0.013204
gL	GAGE6	0.037989
gL	YIR021W-A	0.043183
gL	COMMD9	0.071434
gL	ANGEL2	0.031989
gL	MIR7-3HG	0.030716
gL	PP632	0.032886
gL	C17orf104	0.044904
gL	CETN1	0.093565
gL	PRO1854	0.039007
gL	GAGE4	0.092213
gL	ATP5SL	0.02078
gL	C2CD4D	0.041153
gL	ZK1128.3	0.059584
gL	YDL022C-A	0.023887

gL	PP632	0.034502
gL	PNMAL1	0.068914
gL	C18orf42	0.019379
gL	VLDLR	0.03705
gL	CDH1	0.017097
gL	GAGE13	0.037989
gL	TTN	0.012911
gL	ZYG11A	0.015968
gL	CXorf30	0.11051
gL	EGF	0.022289
gL	LTBP4	0.061666
gL	CCDC132	0.039972
gL	PLSCR4	0.092134
gL	DNAJC9-AS1	0.1399
gL	UBQLNL	0.013987
gL	CCDC152	0.01987
gL	KHDC1L	0.016747
gL	FAM45A	0.15954
gL	KIAA1751	0.025615
gL	TCP10L2	0.086654
gL	CCDC57	0.013804
gL	C1orf106	0.18979
gL	GOLGA6L3	0.13843
gL	WWC2-AS2	0.095946
gL	SNX9	0.025514
gL	FBXL18	0.049689
gL	GUCD1	0.037793
gL	FAM195B	0.034881
gL	CCDC7	0.067267
gL	YFL013W-A	0.10071
gL	IL6ST	0.046992
gL	GAGE12F	0.070023
gL	ANKRD26P1	0.010385
gL	NELFA	0.18458
gL	GPRIN3	0.042846
gL	FAM92B	0.023975
gL	C4orf22	0.02543
gL	C6orf52	0.015187
gL	CCDC74A	0.067492
gL	ANP32C	0.012133
gL	PRR19	0.079599
gL	CCDC67	0.016352
gL	S100A8	0.026343
gL	NID2	0.080796
gL	GAREML	0.19535
gL	MIMI	0.12923
gL	NELL1	0.019568
gL	ytzL	0.24079
gL	WDR96	0.094646
gL	DNER	0.03186
gL	COL17A1	0.14854
gL	L1TD1	0.016408
gL	C15orf65	0.049566
gL	FAM160B1	0.119
gL	SAMD14	0.016497
gL	PEF1	0.070024
gL	CT45A4	0.041897
gL	FBXL8	0.26438
gL	NUPR1L	0.069917
gL	C10orf82	0.085914
gL	ERICH1-AS1	0.13147
gL	C3orf65	0.092286
gL	CHP1	0.036382
gL	CBY3	0.073282
gL	C1orf228	0.027471
gL	DYDC2	0.044923
gL	EP400NL	0.11328
gL	FAM203B	0.10041

gL	FASN	0.02932
gL	HEATR5A	0.067656
gL	GPR98	0.01341
gL	OTUD6A	0.037417
gL	MICU1	0.053447
gL	CALU	0.054877
gL	VWA9	0.037938
gL	C16orf72	0.15927
gL	BEND2	0.057804
gL	LRP2	0.028597
gL	JMJD7	0.013796
gL	POM121L2	0.093013
gL	GOLGA6L10	0.11631
gL	SAMD13	0.011477
gL	FAM188B	0.20972
gL	MISP	0.16035
gL	FAM231B	0.094266
gL	SELO	0.12809
gL	ZNF728	0.01494
gL	FAM27D1	0.093262
gL	NUCB2	0.038228
gL	UHRF1BP1L	0.12428
gL	SHANK2-AS3	0.040535
gL	UNQ6126/PRO20091	0.071208
gL	HIN1L	0.013675
gL	ATXN7L2	0.048433
gL	YGL014C-A	0.13586
gL	S100A6	0.044651
gL	LRP8	0.0665
gL	CCDC151	0.025717
gL	CT55	0.1242
gL	S100P	0.013392
gL	RWDD4	0.038739
gL	CHAF1A	0.20205
gL	PTGES3L	0.045005
gL	TP53INP1	0.13213
gL	RUNDC3B	0.014283
gL	MNCb-2875	0.045278
gL	FAM127C	0.055905
gL	EFCAB4B	0.036204
gL	C8orf74	0.065723
gL	CCDC104	0.04393
gL	XAGE3	0.20296
gL	C11orf65	0.036589
gL	ASAH2B	0.0126
gL	CCDC85A	0.013116
gL	C1orf111	0.10139
gL	TCL1B	0.011876
gL	CCDC87	0.01849
gL	CHGB	0.26062
gL	JMJD8	0.011696
gL	REPS2	0.075547
gL	PPEF2	0.01692
gL	EFEMP2	0.071243
gL	MAGED4	0.015284
gL	ATP2A1	0.019076
gL	ATXN7L3B	0.064152
gL	WLS	0.056892
gL	OCEL1	0.010835
gL	CT45A3	0.047071
gL	CAGE1	0.030564
gL	LINC00205	0.10237
gL	PKD2L1	0.032855
gL	FAM46B	0.090659
gL	PNAS-138	0.098442
gL	NCOA6	0.12088
gL	CCDC26	0.075784
gL	FAM181A	0.035864

gL SUPT20HL1 0.11166
gL C7orf57 0.033351
gL C22orf43 0.064709
gL FAM166B 0.019613
gL VWA5B2 0.059433
gL THBS2 0.049285
gL PRAME 0.17682
gL CALM1 0.04374
gL MAP7D2 0.027173
gL S100A4 0.07995
gL S100A12 0.033601
gL FAM149B1 0.07431
gL RCN1 0.076524
gL DPP4 0.01851
gL STAG3L4 0.040382
gL ACTN3 0.080431
gL ATP5F1 0.041223
gL FBN1 0.081375
gL SPAC22F8.05 0.015627
gL C12orf63 0.045549
gL F10 0.020307
gL FAM46A 0.091735
gL CELSR3 0.023361
gL RILP 0.1116
gL SS18L2 0.041082
gL GAGE12J 0.037989
gL DEHA2C16280g 0.056604
gL KHNYN 0.067356
gL TESC 0.052573
gL GAGE2A 0.070023
gL ATXN7L1 0.048433
gL LSM12 0.086109
gL KIAA0907 0.025732
gL C12orf57 0.086082
gL FAM228A 0.019766
gL YAE1D1 0.11049
gL CT45A1 0.049091
gL ERICH1 0.10553
gL RPH3A 0.015423
gL SRI 0.021626
gL LRP4 0.03507
gL BVES-AS1 0.063269
gL CCDC127 0.042549
gL CCDC120 0.10976
gL MAGEB16 0.014569
gL SLIT2 0.017324
gL C1orf145 0.1275
gL OTUD1 0.032371
gL MATN2 0.062369
gL CIB1 0.047694
gL PPP2R3A 0.024557
gL DLL1 0.085216
gL ACTN1 0.031632
gL FAM195A 0.19095
gL RGAG1 0.042009
gL PNMA6A 0.014492
gL PNPT1 0.054914
gL UPF3B 0.16748
gL C5orf58 0.14169
gL PRSS3 0.10206
gL CCDC169 0.068734
gL C7orf62 0.078185
gL MYL2 0.10546
gL EDEM1 0.015254
gL MB21D2 0.064644
gL ARMCX5 0.06186
gL SNX19 0.015036
gL SPATA2L 0.079093

gL	C15orf37	0.02466
gL	BTF3L4	0.048224
gL	SPIDR	0.13869
gL	DST	0.051166
gL	MYL6	0.075558
gL	COMP	0.049795
gL	CCDC47	0.04891
gL	XAGE5	0.11291
gL	CHP2	0.04771
gL	LYRM9	0.13943
gL	MUC1	0.16114
gL	FAM127B	0.086381
gL	CDK5R1	0.068043
gL	CXorf40B	0.043097
gL	C16orf96	0.011155
gL	NECAB2	0.070072
gL	ARMC5	0.010656
gL	CANX	0.028391
gL	SGD1	0.074533
gL	FHAD1	0.011925
gL	FBXO34	0.012235
gL	BSDC1	0.019579
gL	ANXA11	0.018148
gL	GOLGA6L19	0.11631
gL	C8orf88	0.059697
gL	C5orf34	0.06845
gL	LURAP1L	0.17349
gL	C19orf47	0.05202
gL	C4orf33	0.022879
gL	C12orf50	0.13676
gL	C17orf102	0.076832
gL	RMND5B	0.018152
gL	IQCG	0.060334
gL	MATN3	0.084118
gL	MAGEC3	0.0106
gL	PNMA6C	0.030257
gL	FAM47C	0.10638
gL	RET	0.033207
gL	PAGE3	0.08327
gL	FAM43B	0.010754
gL	MAGED2	0.016646
gL	TLX1NB	0.030606
gL	LCA10	0.13815
gL	RAB11FIP4	0.14517
gL	CNGC19	0.16989
gL	C9orf106	0.017763
gL	DYDC1	0.023774
gL	CCDC102B	0.016378
gL	CNRIP1	0.034193
gL	TCERG1L	0.057563
gL	C6orf223	0.18702
gL	C1orf64	0.11416
gL	PACRGL	0.035441
gL	TCTEX1D1	0.036768
gL	C1orf140	0.13078
gL	YGL006W-A	0.018901
gL	GOLGA6L20	0.063692
gL	LINC00083	0.012612
gL	LINC00588	0.12045
gL	HYPK	0.034614
gL	RUNDC1	0.030229
gL	NIPSNAP3B	0.026945
gL	SZRD1	0.055656
gL	AC3.5	0.13473
gL	DDX26B	0.15705
gL	LCHN	0.04477
gL	FAM45B	0.13537
gL	FAM229B	0.10134

gL	LINC00478	0.035201
gL	C2orf81	0.13918
gL	FAM84A	0.052867
gL	SPANXA2-OT1	0.060491
gL	CLIC4	0.022992
gL	FAM49B	0.060511
gL	ZNF767	0.061467
gL	FAM199X	0.054048
gL	JAG2	0.051034
gL	CCDC114	0.013057
gL	HDGFL1	0.013005
gL	SWT1	0.054717
gL	GAGE12G	0.070023
gL	FAM117A	0.11722
gL	CCDC13	0.012651
gL	CADPS	0.1077
gL	C17orf59	0.21579
gL	DRC1	0.043499
gL	FAM161B	0.19939
gL	CCAR1	0.14387
gL	C16orf93	0.070099
gL	C20orf62	0.032228
gL	C6orf222	0.045858
gL	CCDC171	0.065781
gL	FAM178B	0.086572
gL	C1orf195	0.27373
gL	CRELD2	0.064944
gL	NPIPA5	0.020938
gL	PRR23A	0.068028
gL	C15orf32	0.011044
gL	C2orf72	0.12399
gL	YBR056C-B	0.092977
gL	ARMC12	0.067065
gL	GSN	0.025238
gL	SLC11A2	0.013394
gL	C3orf22	0.09763
gL	EXOC3L4	0.077782
gL	CYCB3-1	0.15244
gL	COA7	0.035165
gL	BEND3	0.011976
gL	C22orf26	0.071708
gL	LETM1	0.10867
gL	TNFAIP8L1	0.15685
gL	pkmyt1	0.090571
gL	PGBD4	0.047106
gL	CRYGN	0.058972
gL	FAM231A	0.3827
gL	C6orf229	0.075699
gL	FAM124A	0.24071
gL	AMER1	0.15078
gL	FAM188A	0.031312
gL	MAGEB4	0.020545
gL	RCN3	0.067613
gL	PLSCR1	0.10069
gL	GAGE5	0.037989
gL	GAGE12B	0.070023
gL	NKAPL	0.1728
gL	NCKAP5L	0.15442
gL	CCDC153	0.01943
gL	PRR21	0.060066
gL	TLDC2	0.13654
gL	LTBP1	0.076251
gL	C6orf165	0.013818
gL	MSANTD1	0.035922
gL	F2	0.01264
gL	SMEK3P	0.066102
gL	SYT1	0.024349
gL	C11orf52	0.042685

gL NSUN5P1 0.08069
gL NCS1 0.081365
gL TCAP 0.096786
gL C12orf71 0.026943
gL SRRM5 0.041013
gL CCDC81 0.037237
gL FBLN5 0.07768
gL DAG1 0.075037
gL C1orf192 0.068895
gL HRASLS5 0.011491
gL C2orf62 0.036609
gL MAGEB2 0.010737
gL YAL042C-A 0.032079
gL C19orf67 0.047243
gL C5orf48 0.15294
gL SH3BP5L 0.089547
gL CCDC17 0.032695
gL NECAB3 0.020685
gL MLC1 0.041973
gL C19orf82 0.099936
gL SUMO4 0.011587
gL SECISBP2L 0.13261
gL C11orf96 0.098477
gL CDKN2AIPNL 0.15502
gL INTS4L1 0.014388
gL FAM92A1 0.028025
gL NACA 0.047416
gL NBEAP1 0.014511
gL MEGF6 0.067922
gL RSRC2 0.1484
gL C16orf45 0.040328
gL GOLGA6L9 0.11631
gL ALS2CR11 0.039854
gL ZNRD1-AS1 0.032259
gL FBRSL1 0.07979
gL LRRC14 0.022775
gL ZK1128.3 0.15237
gL CUEDC1 0.069123
gL HEATR5B 0.12408
gL CTAGE15 0.02885
gL LINC00303 0.027956
gL CD93 0.044365
gL CCDC54 0.026865
gL GRIN1 0.023894
gL C20orf195 0.057869
gL C19orf66 0.1252
gL DHH 0.053091
gL GAGE2E 0.037989
gL UNQ6125/PRO20090 0.12055
gL CT45A2 0.047071
gL N4BP2L1 0.031528
gL FAM231D 0.19935
gL MAGEB17 0.019389
gL KIAA0087 0.11264
gL C11orf72 0.054975
gL C9orf171 0.12932
gL IQCF3 0.031358
gL LINC00615 0.099861
gL CCDC94 0.12265
gL MYL6B 0.049259
gL RBBP8NL 0.03826
gL RGAG4 0.078541
gL TTC23L 0.032739
gL CDHR2 0.053794
gL CDR2L 0.037156
gL CDH5 0.028026
gL GR6 0.041338
gL SYT2 0.074653

gL	C3orf14	0.010414
gL	TPD52	0.081821
gM	ubl4ab	0.044588
gM	SPTA1	0.030712
gM	S100A7	0.063324
gM	HSPA5	0.034866
gM	LDLR	0.025838
gM	C12orf61	0.046782
gM	PLSCR3	0.077207
gM	YMR013W-A	0.011175
gM	ATP5B	0.048908
gM	NIN	0.071151
gM	KCNIP1	0.071497
gM	STIM2	0.079061
gM	SPTAN1	0.047596
gM	MASP1	0.13747
gM	SCUBE3	0.10874
gM	PROC	0.11456
gM	PDCD6	0.12575
gM	S100A9	0.02591
gM	ANXA2	0.055442
gM	MMP20	0.010984
gM	MATN4	0.11077
gM	NOTCH3	0.016852
gM	RNH1	0.08847
gM	CLIP1	0.039251
gM	THBD	0.058465
gM	EML1	0.10426
gM	EPS15	0.030441
gM	PCSK2	0.010616
gM	AP3S1	0.026848
gM	SDF4	0.079163
gM	FAT4	0.089228
gM	PPEF1	0.036476
gM	CXorf67	0.048941
gM	FAM188B2	0.035465
gM	THBS1	0.011772
gM	SYT11	0.13403
gM	HSP90B1	0.058456
gM	MACF1	0.068245
gM	CCDC176	0.060357
gM	GCH1	0.023331
gM	NKD2	0.11742
gM	FAT1	0.043613
gM	S100A11	0.082033
gM	CCDC137	0.025016
gM	NKD1	0.074797
gM	PPP2R3B	0.033439
gM	ACTN4	0.043942
gM	EHD1	0.022812
gM	S100A13	0.095666
gM	MELK	0.015724
gM	S100A1	0.047517
gM	UTRN	0.026981
gM	CALB1	0.083024
gM	MASP2	0.12287
gM	PARK7	0.020612
gM	ANXA2R	0.030877
gM	EFEMP1	0.11058
gM	SPARC	0.048966
gM	NOTCH4	0.018208
gM	PCDH17	0.089228
gM	KIAA1841	0.022388
gM	REPS1	0.084169
gM	LRCOL1	0.036072
gM	MPTX1	0.013871
gM	STIM1	0.037412
gM	S100B	0.04227

gM	TNNC1	0.099085
gM	CETN2	0.092477
gM	ANXA6	0.098431
gM	ACTN2	0.036825
gM	PRSS2	0.10693
gM	PITPNM3	0.083512
gM	VIL1	0.050716
gM	CETN1	0.11435
gM	C2CD4D	0.010345
gM	VLDLR	0.042352
gM	CDH1	0.020694
gM	TTN	0.019108
gM	EGF	0.031456
gM	LTBP4	0.085658
gM	PLSCR4	0.074276
gM	SNX9	0.045258
gM	RYR2	0.016047
gM	PRF1	0.030927
gM	IL6ST	0.049555
gM	MYO1E	0.018653
gM	S100A8	0.038911
gM	NID2	0.11528
gM	NELL1	0.05018
gM	ytzL	0.043511
gM	WDR96	0.012692
gM	DNER	0.055174
gM	PEF1	0.10519
gM	C10orf82	0.013764
gM	CHP1	0.045271
gM	FASN	0.039718
gM	GPR98	0.032141
gM	MICU1	0.078458
gM	CALU	0.08671
gM	PPP3CA	0.0115
gM	LRP2	0.038086
gM	FAM188B	0.058673
gM	NUCB2	0.065647
gM	S100A6	0.066648
gM	LRP8	0.076317
gM	CT55	0.045496
gM	S100P	0.056612
gM	RAB11FIP3	0.02025
gM	EFCAB4B	0.071549
gM	DTNA	0.038148
gM	REPS2	0.11584
gM	PPEF2	0.042315
gM	EFEMP2	0.1051
gM	ATP2A1	0.028421
gM	CAGE1	0.012812
gM	PKD2L1	0.050287
gM	EHD3	0.030897
gM	THBS2	0.087052
gM	CALM1	0.054385
gM	S100A4	0.11239
gM	S100A12	0.052953
gM	RCN1	0.115
gM	ACTN3	0.10936
gM	ATP5F1	0.012344
gM	FBN1	0.11068
gM	F10	0.045698
gM	CAPN1	0.02394
gM	CELSR3	0.049575
gM	SIRT4	0.021685
gM	TESC	0.067104
gM	C12orf57	0.017473
gM	RPH3A	0.039611
gM	SRI	0.046449
gM	LRP4	0.051356

gM	SLIT2	0.022337
gM	MATN2	0.092707
gM	CIB1	0.074019
gM	PPP2R3A	0.060019
gM	DLL1	0.088679
gM	ACTN1	0.048059
gM	PRSS3	0.12577
gM	MYL2	0.12438
gM	EDEM1	0.0416
gM	LRP1	0.020071
gM	SNX19	0.054102
gM	PDGFRB	0.013226
gM	DST	0.055905
gM	MYL6	0.10628
gM	COMP	0.078207
gM	ANXA7	0.035959
gM	CHP2	0.077095
gM	NECAB2	0.11415
gM	CANX	0.043419
gM	ANXA11	0.045099
gM	PPP3CB	0.016708
gM	MATN3	0.12236
gM	FAM47C	0.025073
gM	RET	0.040386
gM	CDH23	0.02381
gM	RAB11FIP4	0.16104
gM	C1orf64	0.0282
gM	AC3.5	0.012292
gM	CLIC4	0.03458
gM	FAM49B	0.022251
gM	BNIP2	0.035986
gM	JAG2	0.064659
gM	CADPS	0.12593
gM	CCAR1	0.10442
gM	C1orf195	0.052329
gM	CRELD2	0.10498
gM	GSN	0.043039
gM	SLC11A2	0.015303
gM	LETM1	0.089831
gM	FAM231A	0.093932
gM	FAM188A	0.075303
gM	RCN3	0.11023
gM	PLSCR1	0.041314
gM	NKAPL	0.03989
gM	TLDC2	0.020868
gM	LTBP1	0.11253
gM	F2	0.024954
gM	SYT1	0.03529
gM	NSUN5P1	0.035347
gM	NCS1	0.099253
gM	FBLN5	0.10389
gM	DAG1	0.02241
gM	NECAB3	0.059096
gM	SNX6	0.010557
gM	NPC1L1	0.01801
gM	MEGF6	0.10911
gM	CALR	0.012828
gM	KCNIP2	0.030485
gM	HEATR5B	0.033861
gM	CD93	0.068917
gM	GRIN1	0.0327
gM	DHH	0.075148
gM	N4BP2L1	0.015742
gM	FAM231D	0.025083
gM	C11orf72	0.014216
gM	MYL6B	0.084311
gM	CDHR2	0.082062
gM	CDH5	0.050812

gM	IL6R	0.014218
gM	SYT2	0.090407
gM	TPD52	0.11432
gN	ubl4ab	0.037184
gN	SPTA1	0.033367
gN	S100A7	0.070137
gN	HSPA5	0.038365
gN	LDLR	0.028298
gN	C12orf61	0.04006
gN	PLSCR3	0.085553
gN	ATP5B	0.054397
gN	NIN	0.078588
gN	KCNIP1	0.07936
gN	STIM2	0.08778
gN	SPTAN1	0.052329
gN	MASP1	0.15399
gN	SCUBE3	0.12077
gN	PROC	0.12818
gN	PDCD6	0.14066
gN	S100A9	0.028352
gN	ANXA2	0.061257
gN	MMP20	0.01061
gN	MATN4	0.12294
gN	NOTCH3	0.017751
gN	RNH1	0.097508
gN	CLIP1	0.043118
gN	THBD	0.064702
gN	EML1	0.11569
gN	EPS15	0.032813
gN	PCSK2	0.010299
gN	AP3S1	0.028569
gN	SDF4	0.087814
gN	FAT4	0.098937
gN	PPEF1	0.039109
gN	CXorf67	0.036737
gN	FAM188B2	0.030177
gN	THBS1	0.012641
gN	SYT11	0.15004
gN	HSP90B1	0.064775
gN	MACF1	0.075807
gN	CCDC176	0.050406
gN	GCH1	0.025407
gN	NKD2	0.1314
gN	FAT1	0.047839
gN	S100A11	0.090836
gN	CCDC137	0.015797
gN	NKD1	0.082913
gN	PPP2R3B	0.035721
gN	ACTN4	0.048385
gN	EHD1	0.024367
gN	S100A13	0.10685
gN	MELK	0.01624
gN	S100A1	0.05182
gN	UTRN	0.028921
gN	CALB1	0.092385
gN	MASP2	0.13736
gN	PARK7	0.022135
gN	ANXA2R	0.020301
gN	EFEMP1	0.12328
gN	SPARC	0.05414
gN	NOTCH4	0.019468
gN	PCDH17	0.098937
gN	KIAA1841	0.015868
gN	REPS1	0.093044
gN	LRCOL1	0.031052
gN	MPTX1	0.012726
gN	STIM1	0.040907
gN	S100B	0.046346

gN	TNNC1	0.1103
gN	CETN2	0.10337
gN	ANXA6	0.10928
gN	ACTN2	0.040652
gN	PRSS2	0.11945
gN	PITPNM3	0.092698
gN	VIL1	0.056235
gN	CETN1	0.1278
gN	VLDLR	0.047021
gN	CDH1	0.02266
gN	TTN	0.020832
gN	EGF	0.034616
gN	LTBP4	0.095295
gN	PLSCR4	0.082232
gN	SNX9	0.049801
gN	RYR2	0.017262
gN	PRF1	0.033456
gN	IL6ST	0.055188
gN	MYO1E	0.019857
gN	S100A8	0.042967
gN	NID2	0.1282
gN	NELL1	0.055014
gN	ytzL	0.034846
gN	DNER	0.060863
gN	PEF1	0.11698
gN	CHP1	0.050255
gN	FASN	0.04398
gN	GPR98	0.034993
gN	MICU1	0.087015
gN	CALU	0.096184
gN	PPP3CA	0.011928
gN	LRP2	0.042069
gN	FAM188B	0.044482
gN	NUCB2	0.07258
gN	S100A6	0.073934
gN	LRP8	0.085125
gN	CT55	0.034171
gN	S100P	0.061878
gN	RAB11FIP3	0.021335
gN	EFCAB4B	0.078956
gN	DTNA	0.041342
gN	REPS2	0.12876
gN	PPEF2	0.046327
gN	EFEMP2	0.11673
gN	ATP2A1	0.03116
gN	PKD2L1	0.055468
gN	EHD3	0.033367
gN	THBS2	0.096212
gN	CALM1	0.060478
gN	S100A4	0.12513
gN	S100A12	0.058578
gN	RCN1	0.12793
gN	ACTN3	0.12185
gN	ATP5F1	0.010212
gN	FBN1	0.12321
gN	F10	0.050265
gN	CAPN1	0.025343
gN	CELSR3	0.054446
gN	SIRT4	0.022374
gN	TESC	0.074713
gN	C12orf57	0.016968
gN	RPH3A	0.043364
gN	SRI	0.051011
gN	LRP4	0.056787
gN	SLIT2	0.024516
gN	MATN2	0.1029
gN	CIB1	0.082046
gN	PPP2R3A	0.065992

gN DLL1 0.099263
gN ACTN1 0.053129
gN PRSS3 0.14065
gN MYL2 0.13915
gN EDEM1 0.045409
gN LRP1 0.021669
gN SNX19 0.059051
gN PDGFRB 0.014267
gN DST 0.062191
gN MYL6 0.11831
gN COMP 0.08673
gN CCDC47 0.085111
gN ANXA7 0.038813
gN CHP2 0.085427
gN NECAB2 0.12671
gN CANX 0.047903
gN ANXA11 0.049427
gN PPP3CB 0.017829
gN MATN3 0.1362
gN FAM47C 0.020266
gN RET 0.044735
gN CDH23 0.025628
gN RAB11FIP4 0.18063
gN C1orf64 0.023144
gN CLIC4 0.038022
gN FAM49B 0.016454
gN BNIP2 0.038768
gN JAG2 0.071791
gN CADPS 0.14096
gN CCAR1 0.11635
gN C1orf195 0.043678
gN CRELD2 0.11651
gN GSN 0.047416
gN SLC11A2 0.016667
gN LETM1 0.09962
gN FAM231A 0.075141
gN FAM188A 0.082881
gN RCN3 0.12234
gN PLSCR1 0.04554
gN NKAPL 0.029816
gN TLDC2 0.013928
gN LTBP1 0.12518
gN F2 0.027241
gN SYT1 0.038926
gN NSUN5P1 0.028899
gN NCS1 0.11075
gN FBLN5 0.11579
gN DAG1 0.024352
gN NECAB3 0.064794
gN SNX6 0.010149
gN NPC1L1 0.01867
gN MEGF6 0.12113
gN CALR 0.013823
gN KCNIP2 0.033051
gN HEATR5B 0.027731
gN CD93 0.076286
gN GRIN1 0.03605
gN DHH 0.083595
gN N4BP2L1 0.013569
gN FAM231D 0.019983
gN C11orf72 0.010811
gN MYL6B 0.093366
gN CDHR2 0.090939
gN CDH5 0.055955
gN IL6R 0.01508
gN SYT2 0.10069
gN TPD52 0.12731
BLLF1 ubl4ab 0.034653

BLLF1	GAGE12I	0.014025
BLLF1	PAGE2B	0.058996
BLLF1	SPTA1	0.0317
BLLF1	S100A7	0.068075
BLLF1	HSPA5	0.037238
BLLF1	LDLR	0.026036
BLLF1	UPF3A	0.044714
BLLF1	RABEP1	0.042702
BLLF1	C12orf61	0.03677
BLLF1	PLSCR3	0.082972
BLLF1	ZC3HAV1L	0.041382
BLLF1	CCDC159	0.027736
BLLF1	C10orf68	0.021225
BLLF1	GOLGA6L1	0.013414
BLLF1	KRI1	0.041263
BLLF1	CCDC69	0.011526
BLLF1	YMR013W-A	0.038079
BLLF1	ATP5B	0.053498
BLLF1	NIN	0.075569
BLLF1	KCNIP1	0.077286
BLLF1	STIM2	0.08543
BLLF1	HUS1B	0.020355
BLLF1	ANGEL1	0.016307
BLLF1	C19orf44	0.016925
BLLF1	FSIP1	0.036268
BLLF1	SPTAN1	0.050597
BLLF1	TSGA10IP	0.034752
BLLF1	TNFAIP8L3	0.011737
BLLF1	MASP1	0.15153
BLLF1	SCUBE3	0.11725
BLLF1	SPBC1539.02	0.018858
BLLF1	GLTSCR1L	0.055679
BLLF1	PROC	0.12601
BLLF1	PDCD6	0.13838
BLLF1	S100A9	0.027437
BLLF1	ANXA2	0.059413
BLLF1	MATN4	0.11919
BLLF1	NOTCH3	0.016158
BLLF1	RNH1	0.094001
BLLF1	UBAP2	0.050342
BLLF1	C17orf105	0.012123
BLLF1	FAM110D	0.034294
BLLF1	CLIP1	0.04166
BLLF1	AMOT	0.013808
BLLF1	CCDC154	0.01122
BLLF1	PAGE5	0.035861
BLLF1	GAGE10	0.034207
BLLF1	FAM117B	0.055662
BLLF1	PHYHIP	0.010129
BLLF1	MESDC1	0.014417
BLLF1	C10orf91	0.053274
BLLF1	CCDC28A	0.060154
BLLF1	GID4	0.018562
BLLF1	FAM83F	0.03143
BLLF1	FAM101A	0.025889
BLLF1	SNURFL	0.013979
BLLF1	THBD	0.062799
BLLF1	FAM178A	0.028778
BLLF1	C12orf36	0.010806
BLLF1	EML1	0.11217
BLLF1	FAM83C	0.013471
BLLF1	IQCK	0.030662
BLLF1	FAM166A	0.036771
BLLF1	MUM1L1	0.028632
BLLF1	FAM74A4	0.016949
BLLF1	EPS15	0.030878
BLLF1	AP3S1	0.026312
BLLF1	SDF4	0.085311

33865	BLLF1	NACAP1	0.010821
33866	BLLF1	FAT4	0.09592
33867	BLLF1	DTNBP1	0.011568
33868	BLLF1	PPEF1	0.035995
33869	BLLF1	CXorf67	0.06555
33870	BLLF1	C18orf25	0.016778
33871	BLLF1	FAM188B2	0.06742
33872	BLLF1	NUDCD3	0.019825
33873	BLLF1	THBS1	0.012316
33874	BLLF1	KIAA1407	0.018092
33875	BLLF1	FAM184B	0.013821
33876	BLLF1	CCSER1	0.034531
33877	BLLF1	PNMAL2	0.028418
33878	BLLF1	YPR022C	0.045033
33879	BLLF1	TPPP	0.074482
33880	BLLF1	CCDC146	0.016439
33881	BLLF1	SYT11	0.14762
33882	BLLF1	CCDC150	0.013388
33883	BLLF1	C1orf105	0.034495
33884	BLLF1	HSP90B1	0.063219
33885	BLLF1	MACF1	0.073975
33886	BLLF1	CCDC176	0.03948
33887	BLLF1	GCH1	0.024316
33888	BLLF1	C10orf67	0.035754
33889	BLLF1	LINC00597	0.026831
33890	BLLF1	NKD2	0.12932
33891	BLLF1	MT1HL1	0.019769
33892	BLLF1	C19orf71	0.024273
33893	BLLF1	SNX29P2	0.016881
33894	BLLF1	RHPN1-AS1	0.041155
33895	BLLF1	C6orf118	0.020603
33896	BLLF1	CCDC74B	0.010062
33897	BLLF1	FAT1	0.045891
33898	BLLF1	LINC00471	0.029629
33899	BLLF1	S100A11	0.08791
33900	BLLF1	CCDC137	0.060958
33901	BLLF1	TIFAB	0.048197
33902	BLLF1	NUTM2A	0.010598
33903	BLLF1	NKD1	0.080411
33904	BLLF1	PLAC8L1	0.010043
33905	BLLF1	CROCCP3	0.01048
33906	BLLF1	FAM151B	0.016669
33907	BLLF1	GCSAML	0.033174
33908	BLLF1	FAM47B	0.03144
33909	BLLF1	SUPT20HL2	0.034789
33910	BLLF1	PPP2R3B	0.032704
33911	BLLF1	ACTN4	0.04677
33912	BLLF1	SPRYD7	0.010935
33913	BLLF1	EHD1	0.022444
33914	BLLF1	LINC00523	0.056153
33915	BLLF1	S100A13	0.10491
33916	BLLF1	MELK	0.014412
33917	BLLF1	S100A1	0.049072
33918	BLLF1	ARL14EPL	0.049784
33919	BLLF1	UTRN	0.026781
33920	BLLF1	GAGE12H	0.014025
33921	BLLF1	SLC34A1	0.050662
33922	BLLF1	CXorf21	0.01713
33923	BLLF1	FAM127A	0.011724
33924	BLLF1	C17orf66	0.010492
33925	BLLF1	PIH1D2	0.035655
33926	BLLF1	POM121L12	0.027665
33927	BLLF1	CALB1	0.090246
33928	BLLF1	MASP2	0.13475
33929	BLLF1	PARK7	0.021076
33930	BLLF1	LINC00472	0.034443
33931	BLLF1	GOLGA6L4	0.040075
33932	BLLF1	GOLGA6L6	0.037974

BLLF1	KIAA1958	0.022071
BLLF1	SLAIN1	0.057086
BLLF1	C9orf169	0.010065
BLLF1	YPL025C	0.011626
BLLF1	ANXA2R	0.063102
BLLF1	CC2D2B	0.014238
BLLF1	YML009W-B	0.04939
BLLF1	C14orf178	0.01899
BLLF1	EFEMP1	0.12013
BLLF1	C1orf168	0.022208
BLLF1	YTHDF1	0.022636
BLLF1	SPARC	0.052573
BLLF1	MRFAP1L1	0.045195
BLLF1	NOTCH4	0.018239
BLLF1	FAM160A1	0.051905
BLLF1	PCDH17	0.09592
BLLF1	YJR030C	0.033659
BLLF1	KIAA1841	0.032403
BLLF1	C1orf196	0.016382
BLLF1	RNF216P1	0.020939
BLLF1	REPS1	0.08973
BLLF1	YJEFN3	0.015839
BLLF1	C18orf21	0.010757
BLLF1	XAGE2	0.041953
BLLF1	YML009W-B	0.018626
BLLF1	LRCOL1	0.051838
BLLF1	GOLGA6L18	0.032573
BLLF1	BCORP1	0.021982
BLLF1	FAM160B2	0.010451
BLLF1	FAM221B	0.034742
BLLF1	MPTX1	0.015199
BLLF1	STIM1	0.039133
BLLF1	S100B	0.044421
BLLF1	AC3.5	0.018661
BLLF1	LINS	0.015366
BLLF1	TNNC1	0.10764
BLLF1	C14orf80	0.032736
BLLF1	CETN2	0.10174
BLLF1	ANXA6	0.10617
BLLF1	C9orf153	0.011053
BLLF1	ACTN2	0.039609
BLLF1	PRSS2	0.11715
BLLF1	CT45A5	0.010012
BLLF1	C2orf78	0.012874
BLLF1	C21orf58	0.015077
BLLF1	PITPNM3	0.090117
BLLF1	VIL1	0.054937
BLLF1	C19orf35	0.029607
BLLF1	YIR021W-A	0.01283
BLLF1	COMMD9	0.024735
BLLF1	ANGEL2	0.011075
BLLF1	PP632	0.011376
BLLF1	CETN1	0.1255
BLLF1	GAGE4	0.027414
BLLF1	C2CD4D	0.014001
BLLF1	ZK1128.3	0.017353
BLLF1	PNMAL1	0.020188
BLLF1	VLDLR	0.046208
BLLF1	CDH1	0.022233
BLLF1	TTN	0.020305
BLLF1	CXorf30	0.029887
BLLF1	EGF	0.033712
BLLF1	LTBP4	0.092682
BLLF1	CCDC132	0.012542
BLLF1	PLSCR4	0.079606
BLLF1	DNAJC9-AS1	0.032564
BLLF1	FAM45A	0.044533
BLLF1	TCP10L2	0.022153

BLLF1	C1orf106	0.053448
BLLF1	GOLGA6L3	0.040075
BLLF1	WWC2-AS2	0.030117
BLLF1	SNX9	0.048163
BLLF1	FBXL18	0.012159
BLLF1	CCDC7	0.021545
BLLF1	YFL013W-A	0.027723
BLLF1	RYR2	0.016584
BLLF1	PRF1	0.031508
BLLF1	IL6ST	0.054445
BLLF1	MYO1E	0.018505
BLLF1	GAGE12F	0.014025
BLLF1	CCDC74A	0.010062
BLLF1	PRR19	0.025671
BLLF1	S100A8	0.041828
BLLF1	NID2	0.12453
BLLF1	GAREML	0.057505
BLLF1	MIMI	0.036921
BLLF1	NELL1	0.052587
BLLF1	ytzL	0.086845
BLLF1	WDR96	0.023165
BLLF1	DNER	0.058789
BLLF1	C15orf65	0.015699
BLLF1	FAM160B1	0.028872
BLLF1	PEF1	0.11376
BLLF1	CT45A4	0.01186
BLLF1	NUPR1L	0.022387
BLLF1	C10orf82	0.023204
BLLF1	ERICH1-AS1	0.038495
BLLF1	C3orf65	0.021497
BLLF1	CHP1	0.049305
BLLF1	CBY3	0.01493
BLLF1	DYDC2	0.012591
BLLF1	EP400NL	0.027099
BLLF1	FAM203B	0.028173
BLLF1	FASN	0.042943
BLLF1	HEATR5A	0.017322
BLLF1	GPR98	0.033493
BLLF1	OTUD6A	0.011238
BLLF1	MICU1	0.084513
BLLF1	CALU	0.093349
BLLF1	PPP3CA	0.010857
BLLF1	VWA9	0.014104
BLLF1	C16orf72	0.038017
BLLF1	BEND2	0.017261
BLLF1	LRP2	0.041066
BLLF1	POM121L2	0.025611
BLLF1	GOLGA6L10	0.032573
BLLF1	FAM188B	0.071758
BLLF1	MISP	0.046619
BLLF1	FAM231B	0.025654
BLLF1	SELO	0.034701
BLLF1	FAM27D1	0.013737
BLLF1	NUCB2	0.07023
BLLF1	UHRF1BP1L	0.036726
BLLF1	SHANK2-AS3	0.010281
BLLF1	UNQ6126/PRO20091	0.022215
BLLF1	ATXN7L2	0.012816
BLLF1	YGL014C-A	0.041949
BLLF1	S100A6	0.071924
BLLF1	LRP8	0.083607
BLLF1	CT55	0.03566
BLLF1	S100P	0.058564
BLLF1	CHAF1A	0.06508
BLLF1	PTGES3L	0.011795
BLLF1	MNCb-2875	0.013995
BLLF1	FAM127C	0.013089
BLLF1	RAB11FIP3	0.019505

BLLF1	EFCAB4B	0.076031
BLLF1	C8orf74	0.018308
BLLF1	CCDC104	0.013182
BLLF1	XAGE3	0.058996
BLLF1	C1orf111	0.027448
BLLF1	DTNA	0.03876
BLLF1	REPS2	0.12512
BLLF1	PPEF2	0.044317
BLLF1	EFEMP2	0.11363
BLLF1	ATP2A1	0.03029
BLLF1	ATXN7L3B	0.012104
BLLF1	WLS	0.020531
BLLF1	CT45A3	0.013604
BLLF1	CAGE1	0.010865
BLLF1	LINC00205	0.03026
BLLF1	PKD2L1	0.053789
BLLF1	FAM46B	0.02465
BLLF1	PNAS-138	0.026506
BLLF1	CCDC26	0.0225
BLLF1	EHD3	0.031118
BLLF1	SUPT20HL1	0.034789
BLLF1	C22orf43	0.012469
BLLF1	VWA5B2	0.022272
BLLF1	THBS2	0.093018
BLLF1	CALM1	0.059333
BLLF1	S100A4	0.12204
BLLF1	S100A12	0.056868
BLLF1	FAM149B1	0.01756
BLLF1	RCN1	0.1244
BLLF1	ACTN3	0.11902
BLLF1	ATP5F1	0.01407
BLLF1	FBN1	0.11997
BLLF1	C12orf63	0.014211
BLLF1	F10	0.048229
BLLF1	CAPN1	0.022842
BLLF1	FAM46A	0.023769
BLLF1	CELSR3	0.052238
BLLF1	SS18L2	0.011466
BLLF1	KHNYN	0.018399
BLLF1	SIRT4	0.019467
BLLF1	TESC	0.073207
BLLF1	GAGE2A	0.014025
BLLF1	ATXN7L1	0.012816
BLLF1	LSM12	0.027734
BLLF1	C12orf57	0.023084
BLLF1	YAE1D1	0.028769
BLLF1	CT45A1	0.013839
BLLF1	ERICH1	0.024313
BLLF1	RPH3A	0.041475
BLLF1	SRI	0.048998
BLLF1	LRP4	0.055172
BLLF1	BVES-AS1	0.022907
BLLF1	CCDC120	0.0331
BLLF1	SLIT2	0.024027
BLLF1	C1orf145	0.042457
BLLF1	OTUD1	0.010989
BLLF1	MATN2	0.099776
BLLF1	CIB1	0.079696
BLLF1	PPP2R3A	0.063157
BLLF1	DLL1	0.097966
BLLF1	ACTN1	0.051658
BLLF1	FAM195A	0.052548
BLLF1	RGAG1	0.010596
BLLF1	UPF3B	0.048875
BLLF1	C5orf58	0.027411
BLLF1	PRSS3	0.13803
BLLF1	CCDC169	0.017235
BLLF1	C7orf62	0.016182

BLLF1	MYL2	0.13652
BLLF1	EDEM1	0.043202
BLLF1	LRP1	0.020658
BLLF1	MB21D2	0.019837
BLLF1	ARMCX5	0.013323
BLLF1	SNX19	0.056062
BLLF1	PDGFRB	0.013912
BLLF1	SPATA2L	0.023183
BLLF1	BTF3L4	0.01268
BLLF1	SPIDR	0.041295
BLLF1	DST	0.061246
BLLF1	MYL6	0.11538
BLLF1	COMP	0.08386
BLLF1	CCDC47	0.082448
BLLF1	XAGE5	0.025455
BLLF1	ANXA7	0.036252
BLLF1	CHP2	0.082846
BLLF1	LYRM9	0.04433
BLLF1	FAM127B	0.022457
BLLF1	CXorf40B	0.012949
BLLF1	NECAB2	0.12283
BLLF1	CANX	0.046477
BLLF1	SGD1	0.020277
BLLF1	ANXA11	0.047287
BLLF1	PPP3CB	0.016758
BLLF1	GOLGA6L19	0.032573
BLLF1	C8orf88	0.014343
BLLF1	C5orf34	0.018882
BLLF1	LURAP1L	0.047952
BLLF1	C19orf47	0.012108
BLLF1	C12orf50	0.038403
BLLF1	C17orf102	0.027511
BLLF1	IQCG	0.012374
BLLF1	MATN3	0.13216
BLLF1	FAM47C	0.031806
BLLF1	RET	0.04375
BLLF1	PAGE3	0.020033
BLLF1	CDH23	0.02408
BLLF1	LCA10	0.048209
BLLF1	RAB11FIP4	0.16765
BLLF1	CNGC19	0.065682
BLLF1	CNRIP1	0.01176
BLLF1	TCERG1L	0.012497
BLLF1	C6orf223	0.051787
BLLF1	C1orf64	0.043447
BLLF1	PACRGL	0.011176
BLLF1	C1orf140	0.037229
BLLF1	GOLGA6L20	0.015308
BLLF1	LINC00588	0.035251
BLLF1	SZRD1	0.01131
BLLF1	AC3.5	0.040983
BLLF1	DDX26B	0.04174
BLLF1	FAM45B	0.038417
BLLF1	FAM229B	0.025573
BLLF1	C2orf81	0.038223
BLLF1	FAM84A	0.014096
BLLF1	SPANXA2-OT1	0.012436
BLLF1	CLIC4	0.036928
BLLF1	FAM49B	0.018278
BLLF1	BNIP2	0.036267
BLLF1	ZNF767	0.022975
BLLF1	FAM199X	0.017148
BLLF1	JAG2	0.070176
BLLF1	SWT1	0.011501
BLLF1	GAGE12G	0.014025
BLLF1	FAM117A	0.033944
BLLF1	CADPS	0.1387
BLLF1	C17orf59	0.066324

BLLF1	FAM161B	0.059516
BLLF1	CCAR1	0.1136
BLLF1	C16orf93	0.025529
BLLF1	C20orf62	0.012926
BLLF1	C6orf222	0.012723
BLLF1	CCDC171	0.01972
BLLF1	FAM178B	0.020287
BLLF1	C1orf195	0.087999
BLLF1	CRELD2	0.11298
BLLF1	C2orf72	0.042686
BLLF1	YBR056C-B	0.024927
BLLF1	ARMC12	0.014016
BLLF1	GSN	0.045899
BLLF1	SLC11A2	0.016429
BLLF1	C3orf22	0.030821
BLLF1	EXOC3L4	0.016219
BLLF1	CYCB3-1	0.047392
BLLF1	LETM1	0.096603
BLLF1	TNFAIP8L1	0.044015
BLLF1	pkmyt1	0.025455
BLLF1	CRYGN	0.014873
BLLF1	FAM231A	0.14702
BLLF1	C6orf229	0.025732
BLLF1	FAM124A	0.073048
BLLF1	FAM188A	0.079353
BLLF1	RCN3	0.11858
BLLF1	PLSCR1	0.044135
BLLF1	GAGE12B	0.014025
BLLF1	NKAPL	0.070904
BLLF1	NCKAP5L	0.043879
BLLF1	PRR21	0.017132
BLLF1	TLDC2	0.039093
BLLF1	LTBP1	0.12138
BLLF1	MSANTD1	0.010725
BLLF1	F2	0.026248
BLLF1	SMEK3P	0.018422
BLLF1	SYT1	0.037947
BLLF1	NSUN5P1	0.024699
BLLF1	PI4K2A	0.013473
BLLF1	NCS1	0.10874
BLLF1	TCAP	0.03513
BLLF1	SRRM5	0.012808
BLLF1	FBLN5	0.11283
BLLF1	DAG1	0.023409
BLLF1	C1orf192	0.019567
BLLF1	C19orf67	0.012567
BLLF1	C5orf48	0.042307
BLLF1	SH3BP5L	0.023112
BLLF1	CCDC17	0.011177
BLLF1	NECAB3	0.061777
BLLF1	MLC1	0.012164
BLLF1	C19orf82	0.029396
BLLF1	SECISBP2L	0.044649
BLLF1	C11orf96	0.022476
BLLF1	CDKN2AIPNL	0.04371
BLLF1	NACA	0.014073
BLLF1	NPC1L1	0.016776
BLLF1	MEGF6	0.11749
BLLF1	CALR	0.013409
BLLF1	RSRC2	0.037491
BLLF1	C16orf45	0.015538
BLLF1	GOLGA6L9	0.032573
BLLF1	KCNIP2	0.031276
BLLF1	FBRSL1	0.022927
BLLF1	ZK1128.3	0.039479
BLLF1	CUEDC1	0.018757
BLLF1	HEATR5B	0.03877
BLLF1	CTAGE15	0.010761

BLLF1	CD93	0.069679
BLLF1	GRIN1	0.035199
BLLF1	C20orf195	0.015421
BLLF1	C19orf66	0.039429
BLLF1	DHH	0.081585
BLLF1	UNQ6125/PRO20090	0.036584
BLLF1	CT45A2	0.013604
BLLF1	N4BP2L1	0.011232
BLLF1	FAM231D	0.072714
BLLF1	KIAA0087	0.031663
BLLF1	C11orf72	0.020098
BLLF1	C9orf171	0.030334
BLLF1	LINC00615	0.025266
BLLF1	CCDC94	0.03189
BLLF1	MYL6B	0.090349
BLLF1	RGAG4	0.020903
BLLF1	TTC23L	0.010904
BLLF1	CDHR2	0.088191
BLLF1	CDR2L	0.010429
BLLF1	CDH5	0.053942
BLLF1	IL6R	0.014169
BLLF1	SYT2	0.098615
BLLF1	TPD52	0.1242
BZLF2	ubl4ab	0.049115
BZLF2	GAGE12I	0.042935
BZLF2	PAGE2B	0.10168
BZLF2	SPTA1	0.05219
BZLF2	S100A7	0.095153
BZLF2	HSPA5	0.051662
BZLF2	LDLR	0.036167
BZLF2	UPF3A	0.059304
BZLF2	RABEP1	0.063583
BZLF2	C12orf61	0.039033
BZLF2	FAM86B2	0.012022
BZLF2	PNMA6D	0.023454
BZLF2	PLSCR3	0.11662
BZLF2	ZC3HAV1L	0.045974
BZLF2	BTBD16	0.01376
BZLF2	C9orf40	0.01581
BZLF2	RPUSD4	0.034334
BZLF2	CCDC159	0.04514
BZLF2	C10orf68	0.040501
BZLF2	CCDC162P	0.018362
BZLF2	GOLGA6L1	0.032194
BZLF2	KRI1	0.087506
BZLF2	SPATA1	0.015848
BZLF2	CCDC69	0.034755
BZLF2	YMR013W-A	0.048332
BZLF2	CASC3	0.075634
BZLF2	CYP4F30P	0.011017
BZLF2	ATP5B	0.066928
BZLF2	NIN	0.11155
BZLF2	C5orf52	0.017947
BZLF2	KCNIP1	0.10496
BZLF2	STIM2	0.11668
BZLF2	HUS1B	0.047269
BZLF2	FAM229A	0.015094
BZLF2	C1orf222	0.021648
BZLF2	RPLP1	0.033149
BZLF2	PSMG3	0.010063
BZLF2	ANGEL1	0.021923
BZLF2	C19orf44	0.020974
BZLF2	FSIP1	0.072621
BZLF2	WIPF1	0.022519
BZLF2	SPTAN1	0.074494
BZLF2	TSGA10IP	0.066905
BZLF2	TNFAIP8L3	0.015972
BZLF2	MASP1	0.18872

BZLF2	CCDC174	0.022281
BZLF2	SCUBE3	0.16358
BZLF2	SPBC1539.02	0.033881
BZLF2	C19orf45	0.016083
BZLF2	C2orf43	0.023132
BZLF2	GLTSCR1L	0.097571
BZLF2	MAB21L3	0.015577
BZLF2	PRR15L	0.02786
BZLF2	PROC	0.1583
BZLF2	PDCD6	0.17225
BZLF2	S100A9	0.039132
BZLF2	ANXA2	0.080858
BZLF2	MMP20	0.036619
BZLF2	smf	0.017507
BZLF2	MATN4	0.16816
BZLF2	NOTCH3	0.034265
BZLF2	RNH1	0.13884
BZLF2	UBAP2	0.079109
BZLF2	C17orf105	0.036458
BZLF2	RRP7B	0.013711
BZLF2	FAM110D	0.076756
BZLF2	CLIP1	0.06046
BZLF2	SRRM3	0.015484
BZLF2	AMOT	0.021141
BZLF2	CCDC154	0.015216
BZLF2	PAGE5	0.039802
BZLF2	C20orf196	0.016702
BZLF2	GAGE10	0.050047
BZLF2	FAM117B	0.10168
BZLF2	EIF4G2	0.019072
BZLF2	TRAPPC13	0.015396
BZLF2	PHYHIP	0.012016
BZLF2	GAGE3	0.021152
BZLF2	MESDC1	0.015311
BZLF2	C10orf91	0.093711
BZLF2	CCDC28A	0.10438
BZLF2	TTC22	0.010112
BZLF2	FAM120C	0.020938
BZLF2	CCDC65	0.015543
BZLF2	GID4	0.032885
BZLF2	FAM83F	0.04846
BZLF2	FAM101A	0.052859
BZLF2	SNURFL	0.052279
BZLF2	THBD	0.082419
BZLF2	KIAA0040	0.030918
BZLF2	FAM46D	0.022593
BZLF2	FAM178A	0.066087
BZLF2	MEN1	0.019877
BZLF2	RNMT	0.017437
BZLF2	C12orf36	0.022266
BZLF2	EML1	0.15817
BZLF2	OTUD6B	0.013103
BZLF2	FAM83C	0.045008
BZLF2	IQCK	0.060964
BZLF2	ITFG2	0.011648
BZLF2	FAM166A	0.066206
BZLF2	MUM1L1	0.062127
BZLF2	FAM74A4	0.019904
BZLF2	EPS15	0.054167
BZLF2	PCSK2	0.032784
BZLF2	AP3S1	0.053102
BZLF2	SDF4	0.11823
BZLF2	NACAP1	0.017997
BZLF2	STAT1	0.015627
BZLF2	FAT4	0.13523
BZLF2	DTNBP1	0.015092
BZLF2	SDE2	0.014329
BZLF2	PPEF1	0.072503

BZLF2	CXorf67	0.064616
BZLF2	C18orf25	0.040023
BZLF2	NELL2	0.020456
BZLF2	THEGL	0.02331
BZLF2	C11orf35	0.019727
BZLF2	C16orf86	0.02376
BZLF2	FAM188B2	0.088483
BZLF2	FAM78A	0.030946
BZLF2	NUDCD3	0.036197
BZLF2	THBS1	0.016485
BZLF2	MAGEF1	0.013409
BZLF2	YIR021W-A	0.010999
BZLF2	KIAA1407	0.036402
BZLF2	FAM184B	0.022834
BZLF2	PRMT1	0.013555
BZLF2	CCSER1	0.071082
BZLF2	PNMAL2	0.049902
BZLF2	AP1M1	0.014698
BZLF2	YPR022C	0.083631
BZLF2	TPPP	0.10849
BZLF2	CCDC146	0.048483
BZLF2	SYT11	0.1837
BZLF2	CCDC150	0.028536
BZLF2	C1orf105	0.063613
BZLF2	HSP90B1	0.085474
BZLF2	MACF1	0.098848
BZLF2	CCDC66	0.017139
BZLF2	CCDC138	0.010134
BZLF2	C9orf170	0.01791
BZLF2	CCDC176	0.061425
BZLF2	GCH1	0.037163
BZLF2	C10orf67	0.083974
BZLF2	LINC00597	0.045452
BZLF2	FAM131C	0.01909
BZLF2	ZBTB8OS	0.015428
BZLF2	NKD2	0.16062
BZLF2	MT1HL1	0.051409
BZLF2	C2orf88	0.017081
BZLF2	NCOR1P1	0.010431
BZLF2	ATP2B2	0.010141
BZLF2	C19orf71	0.040376
BZLF2	SNX29P2	0.019973
BZLF2	RHPN1-AS1	0.059664
BZLF2	C6orf118	0.03912
BZLF2	IL17REL	0.015911
BZLF2	CCDC74B	0.048564
BZLF2	FAT1	0.070148
BZLF2	LINC00471	0.046588
BZLF2	S100A11	0.12568
BZLF2	FAM83E	0.010867
BZLF2	MSANTD2	0.014962
BZLF2	CCDC137	0.077538
BZLF2	TIFAB	0.095871
BZLF2	C15orf54	0.041054
BZLF2	C16orf95	0.023122
BZLF2	NUTM2A	0.034853
BZLF2	NKD1	0.11334
BZLF2	PLAC8L1	0.020833
BZLF2	CROCCP3	0.01729
BZLF2	FAM151B	0.025818
BZLF2	GCSAML	0.049156
BZLF2	FAM47B	0.031381
BZLF2	MBD3L5	0.012307
BZLF2	BEND4	0.015553
BZLF2	SUPT20HL2	0.052105
BZLF2	PPP2R3B	0.067939
BZLF2	ACTN4	0.067399
BZLF2	SPRYD7	0.014751

34477	BZLF2	EHD1	0.043263
34478	BZLF2	LINC00523	0.093954
34479	BZLF2	RABGAP1L	0.013102
34480	BZLF2	S100A13	0.13298
34481	BZLF2	MELK	0.035345
34482	BZLF2	C9orf152	0.024809
34483	BZLF2	S100A1	0.082376
34484	BZLF2	ARL14EPL	0.073149
34485	BZLF2	FAM53C	0.012522
34486	BZLF2	UTRN	0.051924
34487	BZLF2	GAGE12H	0.042935
34488	BZLF2	INTS4L2	0.016824
34489	BZLF2	SLC34A1	0.051162
34490	BZLF2	FAM219B	0.01739
34491	BZLF2	CXorf21	0.041351
34492	BZLF2	FAM127A	0.031768
34493	BZLF2	C17orf66	0.017605
34494	BZLF2	PIH1D2	0.058222
34495	BZLF2	C16orf11	0.019415
34496	BZLF2	FAM154B	0.010859
34497	BZLF2	POM121L12	0.041771
34498	BZLF2	CALB1	0.11948
34499	BZLF2	NDEL1	0.014188
34500	BZLF2	MASP2	0.17264
34501	BZLF2	PARK7	0.03567
34502	BZLF2	LINC00472	0.060987
34503	BZLF2	C20orf96	0.015034
34504	BZLF2	GOLGA6L4	0.072534
34505	BZLF2	GOLGA6L6	0.068445
34506	BZLF2	KIAA1958	0.028535
34507	BZLF2	SLAIN1	0.090075
34508	BZLF2	C9orf169	0.027462
34509	BZLF2	YPL025C	0.032441
34510	BZLF2	ZNF326	0.045807
34511	BZLF2	KIAA0226L	0.018525
34512	BZLF2	GAS6	0.010952
34513	BZLF2	ANXA2R	0.064667
34514	BZLF2	CC2D2B	0.029642
34515	BZLF2	YML009W-B	0.091747
34516	BZLF2	C14orf178	0.03948
34517	BZLF2	EFEMP1	0.15801
34518	BZLF2	C1orf168	0.041548
34519	BZLF2	YTHDF1	0.043687
34520	BZLF2	SPARC	0.070992
34521	BZLF2	MRFAP1L1	0.090428
34522	BZLF2	NOTCH4	0.032648
34523	BZLF2	CNPY1	0.010947
34524	BZLF2	FAM160A1	0.092073
34525	BZLF2	PCDH17	0.13523
34526	BZLF2	YJR030C	0.046039
34527	BZLF2	KIAA1841	0.048133
34528	BZLF2	LCA5L	0.010565
34529	BZLF2	C1orf196	0.024279
34530	BZLF2	RNF216P1	0.055229
34531	BZLF2	REPS1	0.13192
34532	BZLF2	LINC00476	0.015265
34533	BZLF2	YJEFN3	0.028595
34534	BZLF2	C18orf21	0.038914
34535	BZLF2	XAGE2	0.090623
34536	BZLF2	YML009W-B	0.03096
34537	BZLF2	LRCOL1	0.048987
34538	BZLF2	GOLGA6L18	0.064501
34539	BZLF2	C16orf71	0.020432
34540	BZLF2	BCORP1	0.043789
34541	BZLF2	FAM160B2	0.028433
34542	BZLF2	WIBG	0.069698
34543	BZLF2	FAM221B	0.054283
34544	BZLF2	NOTCH2	0.010598

BZLF2	CCDC60	0.010398
BZLF2	MPTX1	0.011473
BZLF2	STIM1	0.061026
BZLF2	S100B	0.068585
BZLF2	LDOC1L	0.026746
BZLF2	AC3.5	0.056048
BZLF2	LINS	0.01968
BZLF2	TNNC1	0.14379
BZLF2	C14orf80	0.085444
BZLF2	NBPF24	0.026003
BZLF2	CETN2	0.12648
BZLF2	ANXA6	0.14721
BZLF2	C9orf153	0.025234
BZLF2	ACTN2	0.053445
BZLF2	PRSS2	0.15044
BZLF2	CT45A5	0.028819
BZLF2	C2orf78	0.044626
BZLF2	C21orf58	0.03597
BZLF2	PITPNM3	0.12423
BZLF2	VIL1	0.072582
BZLF2	C19orf35	0.059935
BZLF2	C4orf17	0.018687
BZLF2	GAGE6	0.021152
BZLF2	YIR021W-A	0.019247
BZLF2	COMMD9	0.036521
BZLF2	KCNJ4	0.027961
BZLF2	NBAS	0.013436
BZLF2	ANGEL2	0.014401
BZLF2	MIR7-3HG	0.017539
BZLF2	PP632	0.011336
BZLF2	C17orf104	0.056063
BZLF2	CETN1	0.15933
BZLF2	PRO1854	0.031849
BZLF2	FAM78B	0.01931
BZLF2	GAGE4	0.039468
BZLF2	ATP5SL	0.011173
BZLF2	C2CD4D	0.016478
BZLF2	ZK1128.3	0.026431
BZLF2	YDL022C-A	0.011672
BZLF2	PP632	0.025293
BZLF2	PNMAL1	0.031682
BZLF2	C18orf42	0.024224
BZLF2	VLDLR	0.057915
BZLF2	CDH1	0.028221
BZLF2	GAGE13	0.021152
BZLF2	MAP1LC3B	0.025373
BZLF2	TTN	0.027541
BZLF2	CXorf30	0.051733
BZLF2	EGF	0.045811
BZLF2	LTBP4	0.1236
BZLF2	CCDC132	0.021216
BZLF2	PLSCR4	0.11304
BZLF2	FAM91A1	0.013267
BZLF2	DNAJC9-AS1	0.065557
BZLF2	UBQLNL	0.010089
BZLF2	CCDC152	0.011046
BZLF2	KHDC1L	0.012806
BZLF2	FAM45A	0.085221
BZLF2	KIAA1751	0.023816
BZLF2	TCP10L2	0.046541
BZLF2	SSUH2	0.017113
BZLF2	C1orf106	0.09206
BZLF2	GOLGA6L3	0.072534
BZLF2	WWC2-AS2	0.03629
BZLF2	SNX9	0.069559
BZLF2	FBXL18	0.018053
BZLF2	GUCD1	0.030122
BZLF2	FAM195B	0.033756

BZLF2	CCDC7	0.030799
BZLF2	YFL013W-A	0.047291
BZLF2	RYR2	0.025579
BZLF2	PRF1	0.054833
BZLF2	IL6ST	0.065907
BZLF2	MYO1E	0.034733
BZLF2	GAGE12F	0.042935
BZLF2	BLACE	0.013644
BZLF2	GPRIN3	0.038044
BZLF2	FAM92B	0.014079
BZLF2	SETD2	0.013045
BZLF2	C4orf22	0.01849
BZLF2	C9orf141	0.010821
BZLF2	CCDC74A	0.048564
BZLF2	CDRT4	0.030609
BZLF2	PRR19	0.043323
BZLF2	CCDC67	0.010356
BZLF2	S100A8	0.056941
BZLF2	NID2	0.16784
BZLF2	GAREML	0.10306
BZLF2	MIMI	0.067622
BZLF2	NELL1	0.082541
BZLF2	ytzL	0.084263
BZLF2	WDR96	0.035243
BZLF2	DNER	0.085268
BZLF2	L1TD1	0.011418
BZLF2	C15orf65	0.020547
BZLF2	CCDC96	0.010201
BZLF2	FAM160B1	0.058594
BZLF2	PEF1	0.15638
BZLF2	CT45A4	0.01869
BZLF2	C2orf91	0.01148
BZLF2	NUPR1L	0.044854
BZLF2	MEGF8	0.020415
BZLF2	C10orf82	0.032004
BZLF2	ERICH1-AS1	0.060757
BZLF2	YJR005C-A	0.016435
BZLF2	C3orf65	0.048289
BZLF2	CDPF1	0.027559
BZLF2	CHP1	0.062734
BZLF2	CBY3	0.042929
BZLF2	C1orf228	0.01591
BZLF2	DDA1	0.011784
BZLF2	CFTR	0.010844
BZLF2	DYDC2	0.017083
BZLF2	EP400NL	0.072281
BZLF2	FAM203B	0.039014
BZLF2	FAM106B	0.011878
BZLF2	FASN	0.05664
BZLF2	HEATR5A	0.030988
BZLF2	ODF3L1	0.013157
BZLF2	GPR98	0.052181
BZLF2	OTUD6A	0.021501
BZLF2	MICU1	0.11738
BZLF2	CALU	0.13047
BZLF2	PPP3CA	0.023973
BZLF2	VWA9	0.016096
BZLF2	C16orf72	0.078686
BZLF2	BEND2	0.02022
BZLF2	LRP2	0.054641
BZLF2	SLC23A1	0.018141
BZLF2	POM121L2	0.044188
BZLF2	GOLGA6L10	0.064501
BZLF2	SAMD13	0.010877
BZLF2	FAM188B	0.082048
BZLF2	MISP	0.086458
BZLF2	FAM231B	0.051947
BZLF2	SELO	0.067717

BZLF2	ZNF728	0.013262
BZLF2	FAM27D1	0.067747
BZLF2	NUCB2	0.1005
BZLF2	UHRF1BP1L	0.060044
BZLF2	SHANK2-AS3	0.024291
BZLF2	FAM194B	0.013419
BZLF2	UNQ6126/PRO20091	0.026565
BZLF2	TSGA13	0.011766
BZLF2	ATXN7L2	0.028805
BZLF2	YGL014C-A	0.071043
BZLF2	S100A6	0.098545
BZLF2	LRP8	0.10553
BZLF2	CCDC151	0.012069
BZLF2	CT55	0.044228
BZLF2	S100P	0.098749
BZLF2	RWDD4	0.030577
BZLF2	PRDX6	0.015043
BZLF2	CHAF1A	0.10663
BZLF2	PTGES3L	0.026064
BZLF2	TP53INP1	0.061428
BZLF2	RUNDC3B	0.015199
BZLF2	MNCb-2875	0.01887
BZLF2	FAM127C	0.033838
BZLF2	RAB11FIP3	0.040753
BZLF2	EFCAB4B	0.11303
BZLF2	C8orf74	0.03072
BZLF2	CCDC104	0.019265
BZLF2	XAGE3	0.10168
BZLF2	TEX36	0.030194
BZLF2	C11orf65	0.019087
BZLF2	C1orf111	0.053408
BZLF2	SPATA33	0.011662
BZLF2	CCDC87	0.026122
BZLF2	DTNA	0.069558
BZLF2	REPS2	0.1732
BZLF2	PPEF2	0.069157
BZLF2	EFEMP2	0.15515
BZLF2	C9orf129	0.016501
BZLF2	TXNRD3NB	0.011294
BZLF2	ATP2A1	0.04217
BZLF2	ATXN7L3B	0.038353
BZLF2	WLS	0.022182
BZLF2	OCEL1	0.01112
BZLF2	CT45A3	0.020639
BZLF2	C13orf35	0.010659
BZLF2	CAGE1	0.01139
BZLF2	LINC00205	0.032945
BZLF2	PKD2L1	0.075441
BZLF2	FAM46B	0.046877
BZLF2	PNAS-138	0.052182
BZLF2	NCOA6	0.063192
BZLF2	FAM183B	0.010277
BZLF2	CCDC26	0.049737
BZLF2	EHD3	0.055815
BZLF2	FAM181A	0.031119
BZLF2	SUPT20HL1	0.052105
BZLF2	C7orf57	0.018616
BZLF2	C22orf43	0.042994
BZLF2	FAM166B	0.016686
BZLF2	VWA5B2	0.030218
BZLF2	THBS2	0.1343
BZLF2	CALM1	0.075574
BZLF2	DPH6	0.010854
BZLF2	MAP7D2	0.023962
BZLF2	S100A4	0.1641
BZLF2	S100A12	0.079304
BZLF2	FAM149B1	0.044153
BZLF2	RCN1	0.1712

34749	BZLF2	DPP4	0.015916
34750	BZLF2	EIF4E	0.012254
34751	BZLF2	STAG3L4	0.024832
34752	BZLF2	ACTN3	0.1578
34753	BZLF2	ATP5F1	0.016438
34754	BZLF2	FBN1	0.15881
34755	BZLF2	C12orf63	0.017745
34756	BZLF2	F10	0.074027
34757	BZLF2	CAPN1	0.051735
34758	BZLF2	UCKL1	0.023046
34759	BZLF2	FAM46A	0.047604
34760	BZLF2	CCNYL3	0.012754
34761	BZLF2	CELSR3	0.07977
34762	BZLF2	RILP	0.053437
34763	BZLF2	SS18L2	0.022759
34764	BZLF2	GAGE12J	0.021152
34765	BZLF2	N6AMT1	0.014692
34766	BZLF2	DEHA2C16280g	0.049346
34767	BZLF2	KHNYN	0.03158
34768	BZLF2	SIRT4	0.052832
34769	BZLF2	TESC	0.094458
34770	BZLF2	GAGE2A	0.042935
34771	BZLF2	ATXN7L1	0.028805
34772	BZLF2	LSM12	0.040069
34773	BZLF2	KIAA0907	0.010974
34774	BZLF2	C12orf57	0.028967
34775	BZLF2	FAM228A	0.013599
34776	BZLF2	YAE1D1	0.046253
34777	BZLF2	CT45A1	0.021934
34778	BZLF2	ERICH1	0.050908
34779	BZLF2	RPH3A	0.064785
34780	BZLF2	SRI	0.074141
34781	BZLF2	LRP4	0.07655
34782	BZLF2	BVES-AS1	0.029779
34783	BZLF2	CCDC127	0.034652
34784	BZLF2	CCDC120	0.047244
34785	BZLF2	MAGEB16	0.010133
34786	BZLF2	SLIT2	0.030771
34787	BZLF2	C1orf145	0.062213
34788	BZLF2	OTUD1	0.016223
34789	BZLF2	MATN2	0.13635
34790	BZLF2	CIB1	0.11058
34791	BZLF2	PPP2R3A	0.09851
34792	BZLF2	DLL1	0.11839
34793	BZLF2	ACTN1	0.071004
34794	BZLF2	FAM195A	0.095774
34795	BZLF2	RGAG1	0.02169
34796	BZLF2	PNMA6A	0.016422
34797	BZLF2	C9orf62	0.022131
34798	BZLF2	EHD4	0.028735
34799	BZLF2	UPF3B	0.067421
34800	BZLF2	C5orf58	0.062236
34801	BZLF2	LINC00114	0.012597
34802	BZLF2	PRSS3	0.17614
34803	BZLF2	CCDC169	0.026644
34804	BZLF2	C7orf62	0.051829
34805	BZLF2	MYL2	0.17026
34806	BZLF2	C16orf52	0.010617
34807	BZLF2	EDEM1	0.070155
34808	BZLF2	LRP1	0.032917
34809	BZLF2	MB21D2	0.027172
34810	BZLF2	ARMCX5	0.024558
34811	BZLF2	ATP5A1	0.017097
34812	BZLF2	C19orf48	0.026981
34813	BZLF2	SNX19	0.093003
34814	BZLF2	C7orf71	0.014235
34815	BZLF2	PDGFRB	0.017985
34816	BZLF2	SPATA2L	0.039217

BZLF2	ITSN1	0.024753
BZLF2	C1orf189	0.017734
BZLF2	C15orf37	0.011385
BZLF2	BTF3L4	0.024852
BZLF2	SPIDR	0.070562
BZLF2	DST	0.075447
BZLF2	MYL6	0.15511
BZLF2	COMP	0.11669
BZLF2	CCDC47	0.11697
BZLF2	XAGE5	0.06143
BZLF2	ANXA7	0.066888
BZLF2	CHP2	0.11649
BZLF2	LYRM9	0.074196
BZLF2	C2orf27A	0.011473
BZLF2	FAM127B	0.047545
BZLF2	CDK5R1	0.025072
BZLF2	CXorf40B	0.019562
BZLF2	Gm3414	0.0116
BZLF2	NECAB2	0.17336
BZLF2	CANX	0.061284
BZLF2	SGD1	0.045613
BZLF2	MMP15	0.020745
BZLF2	FBXO34	0.015777
BZLF2	BSDC1	0.01
BZLF2	ANXA11	0.073748
BZLF2	PPP3CB	0.030252
BZLF2	GOLGA6L19	0.064501
BZLF2	C8orf88	0.039225
BZLF2	C5orf34	0.021056
BZLF2	LURAP1L	0.087373
BZLF2	SHCBP1L	0.016234
BZLF2	C19orf47	0.027165
BZLF2	C4orf33	0.014231
BZLF2	C12orf50	0.073949
BZLF2	C17orf102	0.033711
BZLF2	IQGAP1	0.016386
BZLF2	IQCG	0.031606
BZLF2	MATN3	0.17945
BZLF2	PNMA6C	0.023454
BZLF2	FAM47C	0.026369
BZLF2	SH3KBP1	0.018903
BZLF2	C1orf173	0.019214
BZLF2	RET	0.056502
BZLF2	PAGE3	0.039206
BZLF2	TLX1NB	0.01816
BZLF2	CDH23	0.042696
BZLF2	LCA10	0.054988
BZLF2	RAB11FIP4	0.20341
BZLF2	CNGC19	0.075411
BZLF2	C9orf106	0.011716
BZLF2	C3orf84	0.010353
BZLF2	DYDC1	0.013368
BZLF2	CNRIP1	0.011304
BZLF2	TCERG1L	0.03953
BZLF2	SUV39H2	0.012628
BZLF2	C6orf223	0.10477
BZLF2	C10orf85	0.012114
BZLF2	C1orf64	0.043247
BZLF2	PACRGL	0.018813
BZLF2	TCTEX1D1	0.020356
BZLF2	C1orf140	0.065371
BZLF2	YGL006W-A	0.016806
BZLF2	GOLGA6L20	0.042856
BZLF2	LINC00588	0.060594
BZLF2	HYPK	0.01926
BZLF2	RUNDC1	0.016888
BZLF2	NIPSNAP3B	0.0123
BZLF2	SZRD1	0.02909

BZLF2	AC3.5	0.039633
BZLF2	DDX26B	0.082858
BZLF2	LCHN	0.028742
BZLF2	FAM45B	0.067926
BZLF2	FAM229B	0.049897
BZLF2	LINC00478	0.020609
BZLF2	C2orf81	0.048617
BZLF2	FAM84A	0.023001
BZLF2	SPANXA2-OT1	0.038123
BZLF2	CLIC4	0.05141
BZLF2	FAM49B	0.018769
BZLF2	BNIP2	0.06602
BZLF2	ZNF767	0.035904
BZLF2	FAM199X	0.021924
BZLF2	JAG2	0.092315
BZLF2	FAM90A2P	0.010248
BZLF2	CCDC114	0.011201
BZLF2	SWT1	0.037613
BZLF2	GAGE12G	0.042935
BZLF2	FAM117A	0.063207
BZLF2	CYorf17	0.022037
BZLF2	CADPS	0.17274
BZLF2	C17orf59	0.11469
BZLF2	DRC1	0.0308
BZLF2	FAM161B	0.097965
BZLF2	CCAR1	0.15107
BZLF2	C3orf55	0.010973
BZLF2	C16orf93	0.034029
BZLF2	KIAA1908	0.030494
BZLF2	C20orf62	0.011603
BZLF2	C6orf222	0.023125
BZLF2	LINC00299	0.011058
BZLF2	CCDC171	0.025135
BZLF2	FAM178B	0.04076
BZLF2	C1orf195	0.094654
BZLF2	CRELD2	0.15904
BZLF2	NPIPA5	0.011005
BZLF2	PRR23A	0.056229
BZLF2	C2orf72	0.070749
BZLF2	YBR056C-B	0.03442
BZLF2	C11orf86	0.021331
BZLF2	ARMC12	0.042484
BZLF2	C1orf141	0.012227
BZLF2	GSN	0.065483
BZLF2	SLC11A2	0.020101
BZLF2	PLAC8	0.010001
BZLF2	FAM217B	0.013122
BZLF2	C3orf22	0.053038
BZLF2	EXOC3L4	0.041692
BZLF2	CYCB3-1	0.064698
BZLF2	COA7	0.013651
BZLF2	CABP1	0.021746
BZLF2	C22orf26	0.073051
BZLF2	LETM1	0.13596
BZLF2	TNFAIP8L1	0.069757
BZLF2	pkmyt1	0.04624
BZLF2	PGBD4	0.035672
BZLF2	CRYGN	0.032475
BZLF2	FAM231A	0.11568
BZLF2	TFB1M	0.026085
BZLF2	C6orf229	0.047906
BZLF2	FAM124A	0.10559
BZLF2	FAM188A	0.12324
BZLF2	MAGEB4	0.011931
BZLF2	AP1B1	0.026499
BZLF2	RCN3	0.16752
BZLF2	PLSCR1	0.062011
BZLF2	GAGE5	0.021152

BZLF2	SPAC5H10.02c	0.014164
BZLF2	GAGE12B	0.042935
BZLF2	SLC6A4	0.013127
BZLF2	NKAPL	0.078426
BZLF2	NCKAP5L	0.075845
BZLF2	PRR21	0.032345
BZLF2	TLDC2	0.055488
BZLF2	LTBP1	0.16562
BZLF2	C6orf183	0.014585
BZLF2	MSANTD1	0.019445
BZLF2	F2	0.038867
BZLF2	SMEK3P	0.035008
BZLF2	SYT1	0.052042
BZLF2	C11orf52	0.025644
BZLF2	NSUN5P1	0.025152
BZLF2	PI4K2A	0.028007
BZLF2	NCS1	0.13774
BZLF2	TCAP	0.034054
BZLF2	C12orf71	0.017029
BZLF2	SRRM5	0.017584
BZLF2	CCDC81	0.023903
BZLF2	FBLN5	0.1484
BZLF2	DAG1	0.031523
BZLF2	AGTRAP	0.046254
BZLF2	C1orf192	0.039505
BZLF2	C2orf62	0.028654
BZLF2	ymzD	0.01367
BZLF2	YAL042C-A	0.017371
BZLF2	C19orf67	0.02358
BZLF2	C5orf48	0.062663
BZLF2	SH3BP5L	0.047942
BZLF2	CCDC17	0.014946
BZLF2	NPCDR1	0.010184
BZLF2	NECAB3	0.098887
BZLF2	MLC1	0.016944
BZLF2	C22orf42	0.017815
BZLF2	C19orf82	0.055094
BZLF2	SNX6	0.031441
BZLF2	SECISBP2L	0.06565
BZLF2	FAM106A	0.011878
BZLF2	C11orf96	0.06114
BZLF2	CDKN2AIPNL	0.08172
BZLF2	INTS4L1	0.013177
BZLF2	FAM92A1	0.010301
BZLF2	NACA	0.021073
BZLF2	NPC1L1	0.039973
BZLF2	MEGF6	0.16507
BZLF2	CALR	0.017152
BZLF2	RSRC2	0.078262
BZLF2	C16orf45	0.013883
BZLF2	GOLGA6L9	0.064501
BZLF2	ALS2CR11	0.025265
BZLF2	ZNRD1-AS1	0.017801
BZLF2	OAF	0.012307
BZLF2	KCNIP2	0.052662
BZLF2	FBRSL1	0.04659
BZLF2	ZK1128.3	0.082696
BZLF2	CUEDC1	0.033694
BZLF2	HEATR5B	0.049233
BZLF2	CTAGE15	0.012155
BZLF2	LINC00303	0.018028
BZLF2	CD93	0.092618
BZLF2	CCDC54	0.015184
BZLF2	GRIN1	0.047393
BZLF2	CLINT1	0.010472
BZLF2	C20orf195	0.025587
BZLF2	C19orf66	0.04443
BZLF2	DHH	0.10883

BZLF2	GAGE2E	0.021152
BZLF2	UNQ6125/PRO20090	0.073468
BZLF2	SPHK2	0.021232
BZLF2	CT45A2	0.020639
BZLF2	N4BP2L1	0.013661
BZLF2	FAM231D	0.053115
BZLF2	MAGEB17	0.011695
BZLF2	KIAA0087	0.056873
BZLF2	C11orf72	0.0218
BZLF2	C9orf171	0.06708
BZLF2	IQCF3	0.013405
BZLF2	LINC00615	0.050971
BZLF2	CCDC94	0.068043
BZLF2	MYL6B	0.12949
BZLF2	RBBP8NL	0.024801
BZLF2	RGAG4	0.042907
BZLF2	ATP6V0A1	0.01941
BZLF2	TTC23L	0.017824
BZLF2	AP1G1	0.024514
BZLF2	CDHR2	0.12402
BZLF2	CDR2L	0.019465
BZLF2	CDH5	0.079404
BZLF2	GR6	0.029774
BZLF2	IL6R	0.023618
BZLF2	TP53INP2	0.045338
BZLF2	SYT2	0.12766
BZLF2	CAPNS1	0.031463
BZLF2	TPD52	0.1664
BMLF1	HPS4	0.31589
BMLF1	HNRNPA1	0.014157
BMLF1	CBX7	0.29964
BMLF1	ACR	0.26225
BMLF1	ACVR1	0.053325
BMLF1	TAF6	0.13343
BMLF1	TRIB3	0.11449
BMLF1	CST9	0.034289
BMLF1	HDAC6	0.023801
BMLF1	RRP7A	0.039667
BMLF1	ubl4ab	0.088531
BMLF1	FPR1	0.27761
BMLF1	BRAP	0.28796
BMLF1	SERPINB9	0.26923
BMLF1	SLC25A25	0.21019
BMLF1	MACROD1	0.40189
BMLF1	ITGB5	0.2429
BMLF1	HID1	0.32933
BMLF1	GPT2	0.013903
BMLF1	ECT2	0.068553
BMLF1	SHQ1	0.35506
BMLF1	ATPIF1	0.041966
BMLF1	PEX10	0.32767
BMLF1	NLRP2	0.32563
BMLF1	RBL1	0.18698
BMLF1	BIN2	0.45783
BMLF1	BBS10	0.34191
BMLF1	USP2	0.18261
BMLF1	MED1	0.028622
BMLF1	CDC34	0.21895
BMLF1	PMS2P11	0.048165
BMLF1	EFHC2	0.26298
BMLF1	CDK10	0.24021
BMLF1	EPHA3	0.16666
BMLF1	EFCAB1	0.26298
BMLF1	PIK3CD	0.15238
BMLF1	AKAP14	0.093072
BMLF1	RAB7A	0.21235
BMLF1	TGFBR2	0.044682
BMLF1	N4BP1	0.3775

BMLF1	CTDNEP1	0.21345
BMLF1	PANK2	0.059989
BMLF1	FAM58A	0.30247
BMLF1	AR	0.014198
BMLF1	PSMD4	0.19919
BMLF1	FZD8	0.14981
BMLF1	PPP5C	0.19587
BMLF1	SLC2A4	0.18004
BMLF1	KEL	0.27094
BMLF1	HMGCS1	0.011741
BMLF1	MCTS1	0.064499
BMLF1	SERPINA6	0.044197
BMLF1	IFT122	0.36142
BMLF1	NAE1	0.26999
BMLF1	PTAR1	0.042745
BMLF1	PRPF6	0.27851
BMLF1	SLC16A12	0.031311
BMLF1	SPTA1	0.31007
BMLF1	NME2P1	0.023305
BMLF1	SEC31A	0.21177
BMLF1	GZMA	0.30355
BMLF1	S100A7	0.38834
BMLF1	NMRK1	0.080833
BMLF1	PXDN	0.059379
BMLF1	ROCK2	0.050572
BMLF1	IL4	0.066794
BMLF1	NANOS1	0.35581
BMLF1	TRMT2A	0.12434
BMLF1	SEC14L3	0.043358
BMLF1	SLC26A8	0.27982
BMLF1	UBE2H	0.30195
BMLF1	MICALCL	0.026273
BMLF1	HSPA5	0.21204
BMLF1	LDLR	0.15643
BMLF1	NEDD4	0.083962
BMLF1	LAMTOR5	0.30251
BMLF1	GSPT2	0.28231
BMLF1	SERPINB5	0.33222
BMLF1	SSBP1	0.36735
BMLF1	BIRC6	0.19288
BMLF1	GFPT2	0.040209
BMLF1	SERTAD3	0.33915
BMLF1	INO80B	0.28811
BMLF1	KLK13	0.35882
BMLF1	AGO2	0.14548
BMLF1	HEPACAM2	0.27344
BMLF1	RNMTL1	0.19088
BMLF1	CENPW	0.29166
BMLF1	UNC13D	0.28646
BMLF1	UPF3A	0.31728
BMLF1	FABP2	0.063283
BMLF1	ARHGAP33	0.29658
BMLF1	RB1CC1	0.16486
BMLF1	RABEP1	0.36598
BMLF1	SKA3	0.29946
BMLF1	VPS54	0.35724
BMLF1	KIAA1377	0.47224
BMLF1	EIF5A	0.13752
BMLF1	PDSS2	0.084535
BMLF1	CYS1	0.35577
BMLF1	LIMK1	0.15969
BMLF1	FAF1	0.20059
BMLF1	IRF9	0.13239
BMLF1	SLC5A1	0.23953
BMLF1	LRP2BP	0.45783
BMLF1	MAPK8	0.1036
BMLF1	EDF1	0.16837
BMLF1	KCNS1	0.26206

BMLF1	PXN	0.17263
BMLF1	NUP85	0.1596
BMLF1	SOX13	0.23288
BMLF1	LRGUK	0.062971
BMLF1	CTNND2	0.25921
BMLF1	TIPARP	0.018896
BMLF1	UBE2D3	0.15674
BMLF1	C12orf61	0.086917
BMLF1	KRT5	0.29833
BMLF1	IP6K2	0.022653
BMLF1	HSD17B8	0.27669
BMLF1	BTNL8	0.44386
BMLF1	PLSCR3	0.54331
BMLF1	KREMEN1	0.40552
BMLF1	DDX10	0.01449
BMLF1	TCF25	0.21426
BMLF1	NR0B1	0.086211
BMLF1	N6AMT2	0.05805
BMLF1	SNX20	0.33196
BMLF1	PRSS16	0.1438
BMLF1	RBP2	0.024337
BMLF1	BIRC7	0.24369
BMLF1	MCM6	0.179
BMLF1	FKBP10	0.21661
BMLF1	SLC5A4	0.049538
BMLF1	KRT17	0.25707
BMLF1	MAPKAPK2	0.094158
BMLF1	NOSIP	0.29732
BMLF1	SMURF2	0.16102
BMLF1	DYM	0.35272
BMLF1	ANKIB1	0.40834
BMLF1	ZC3HAV1L	0.042691
BMLF1	STOML3	0.01263
BMLF1	ATP6V0D2	0.25267
BMLF1	ORAI3	0.44386
BMLF1	CSNK2A1	0.14552
BMLF1	PFKM	0.1854
BMLF1	SEC31B	0.056937
BMLF1	ELAVL2	0.43363
BMLF1	RPUSD4	0.52663
BMLF1	CMPK2	0.030509
BMLF1	EMR1	0.14937
BMLF1	CD248	0.2241
BMLF1	CCDC159	0.015438
BMLF1	ASIC1	0.23001
BMLF1	SYT5	0.023462
BMLF1	ORC2	0.27067
BMLF1	CEP85L	0.03058
BMLF1	TAF12	0.094792
BMLF1	RDH8	0.027738
BMLF1	PEX16	0.27011
BMLF1	CALN1	0.21218
BMLF1	HMGA2	0.020116
BMLF1	SLC4A11	0.03395
BMLF1	F9	0.20617
BMLF1	CRB1	0.22822
BMLF1	TRAPPC2	0.21663
BMLF1	NIP7	0.44066
BMLF1	C16orf70	0.40397
BMLF1	NAT16	0.061709
BMLF1	USP12	0.33248
BMLF1	DDB2	0.27484
BMLF1	ANAPC5	0.030486
BMLF1	TUBA1B	0.14243
BMLF1	PIP5K1A	0.16096
BMLF1	PEAK1	0.26701
BMLF1	SUN1	0.2794
BMLF1	SFTPD	0.31674

BMLF1	PIP5K1B	0.35538
BMLF1	FAT3	0.2181
BMLF1	TAB3	0.19513
BMLF1	SBF2	0.26631
BMLF1	SLC7A11	0.21071
BMLF1	BNIP1	0.22157
BMLF1	BRMS1	0.28484
BMLF1	FGFR1OP	0.21652
BMLF1	PCDH20	0.22489
BMLF1	PTPRH	0.16566
BMLF1	SPRR1A	0.27934
BMLF1	CALCOCO2	0.3269
BMLF1	USHBP1	0.44049
BMLF1	HMGCR	0.016241
BMLF1	CD1B	0.35782
BMLF1	LALBA	0.19668
BMLF1	BRD4	0.2645
BMLF1	EFCAB5	0.26298
BMLF1	TFAP2B	0.062182
BMLF1	PRPF4B	0.23391
BMLF1	MYF5	0.18721
BMLF1	ARHGEF12	0.18154
BMLF1	RNF2	0.16963
BMLF1	NT5C2	0.21832
BMLF1	LLGL2	0.33377
BMLF1	MYH7	0.24674
BMLF1	SORBS3	0.23234
BMLF1	POLD2	0.2242
BMLF1	SNAPIN	0.23429
BMLF1	INTS6	0.36842
BMLF1	ZDHHC8	0.14365
BMLF1	STX3	0.22107
BMLF1	PCDH12	0.18034
BMLF1	PDE2A	0.09902
BMLF1	PLXNB1	0.18028
BMLF1	RAB13	0.069208
BMLF1	CARTPT	0.21937
BMLF1	HMGB1	0.088952
BMLF1	KCNJ5	0.31622
BMLF1	MLKL	0.28798
BMLF1	MKS1	0.29745
BMLF1	HSPE1	0.31747
BMLF1	LYN	0.012647
BMLF1	MAPK7	0.11644
BMLF1	IGFL2	0.056701
BMLF1	USP11	0.31033
BMLF1	DTX1	0.20291
BMLF1	BLVRA	0.058227
BMLF1	ADAMTS5	0.22195
BMLF1	ENDOU	0.095457
BMLF1	RANBP9	0.31377
BMLF1	TMPRSS4	0.039928
BMLF1	SUFU	0.14005
BMLF1	SPHKAP	0.44386
BMLF1	PXMP2	0.36883
BMLF1	SYTL1	0.35178
BMLF1	SOCS7	0.32761
BMLF1	MAPK1	0.017396
BMLF1	TYRP1	0.26946
BMLF1	ZFPM1	0.078665
BMLF1	RPL21	0.04266
BMLF1	UBA1	0.29021
BMLF1	HDAC2	0.031041
BMLF1	BTBD2	0.43806
BMLF1	VWA3A	0.034866
BMLF1	EIF3J	0.42145
BMLF1	RTN2	0.35846
BMLF1	SLC31A2	0.011785

BMLF1	ZDHHC11	0.012269
BMLF1	STARD6	0.021189
BMLF1	RP1	0.22057
BMLF1	CENPJ	0.20307
BMLF1	DPP10	0.08038
BMLF1	BCCIP	0.36793
BMLF1	F5	0.29904
BMLF1	IGSF1	0.35202
BMLF1	KCND2	0.17657
BMLF1	EPN2	0.10011
BMLF1	PTPN1	0.11774
BMLF1	FKBP1B	0.17199
BMLF1	APLP2	0.18837
BMLF1	APOA4	0.011807
BMLF1	C10orf62	0.47224
BMLF1	TERF2	0.16827
BMLF1	TAAR2	0.043938
BMLF1	RNF167	0.29318
BMLF1	HKDC1	0.044606
BMLF1	UNC119B	0.361
BMLF1	RACGAP1	0.087656
BMLF1	RBL2	0.25127
BMLF1	YMR013W-A	0.055828
BMLF1	CTBP2	0.2309
BMLF1	JAK3	0.12001
BMLF1	CCNB1IP1	0.30372
BMLF1	ACTA1	0.21114
BMLF1	PSMA1	0.12232
BMLF1	DHX30	0.36923
BMLF1	NLRX1	0.28228
BMLF1	VASP	0.2522
BMLF1	PCDHGA1	0.22489
BMLF1	TRAF6	0.022837
BMLF1	CABP7	0.21218
BMLF1	CASC3	0.21894
BMLF1	CHIC1	0.067116
BMLF1	RAVER2	0.011483
BMLF1	WASF2	0.27764
BMLF1	CD58	0.35609
BMLF1	DUSP1	0.09375
BMLF1	MAP2	0.23259
BMLF1	SRSF9	0.026072
BMLF1	TBL1XR1	0.18368
BMLF1	ZFYVE27	0.26776
BMLF1	POP7	0.35397
BMLF1	APBB1	0.086138
BMLF1	DNM3	0.16954
BMLF1	PCNT	0.27924
BMLF1	MRPL13	0.37696
BMLF1	U2AF1	0.27687
BMLF1	ARHGDIA	0.26449
BMLF1	TFDP1	0.11463
BMLF1	PDXP	0.18663
BMLF1	BOC	0.29276
BMLF1	WHSC1	0.01476
BMLF1	P4HB	0.1644
BMLF1	SNRPA	0.3449
BMLF1	GAREM	0.47224
BMLF1	ELP5	0.34635
BMLF1	TCIRG1	0.026705
BMLF1	ALAS2	0.22452
BMLF1	IGFBP5	0.19827
BMLF1	MAN1B1	0.12753
BMLF1	TRMT6	0.025244
BMLF1	ZNF675	0.1818
BMLF1	VWA7	0.058111
BMLF1	SLC16A2	0.043768
BMLF1	PRKCD	0.046858

BMLF1	COLGALT2	0.012878
BMLF1	TGS1	0.021108
BMLF1	MLH1	0.10564
BMLF1	CHRD	0.23451
BMLF1	BICD2	0.39074
BMLF1	APOB	0.043999
BMLF1	CNN3	0.031215
BMLF1	KIF7	0.32904
BMLF1	ADAM9	0.095365
BMLF1	ATP5B	0.19969
BMLF1	NIN	0.52474
BMLF1	NUP93	0.010796
BMLF1	UBB	0.054779
BMLF1	TXNDC17	0.38574
BMLF1	BGLAP	0.045808
BMLF1	ARHGEF1	0.20377
BMLF1	PYCARD	0.051791
BMLF1	MUL1	0.097757
BMLF1	SLC6A12	0.022568
BMLF1	ITCH	0.13974
BMLF1	CEP192	0.33069
BMLF1	SYTL2	0.23952
BMLF1	RAE1	0.023066
BMLF1	PGR	0.10153
BMLF1	CARD11	0.20666
BMLF1	LMO2	0.19787
BMLF1	KCNIP1	0.39471
BMLF1	CDC27	0.23576
BMLF1	MTO1	0.045982
BMLF1	VPS26A	0.28651
BMLF1	BATF	0.24419
BMLF1	SH3BP5	0.41764
BMLF1	TNRC6C	0.024204
BMLF1	ZC3HC1	0.23719
BMLF1	CDH18	0.20658
BMLF1	ILDR2	0.064562
BMLF1	PLCL2	0.084268
BMLF1	AKTIP	0.25633
BMLF1	MARCH8	0.088338
BMLF1	ANKEF1	0.26298
BMLF1	PGM3	0.031874
BMLF1	TNIP1	0.28623
BMLF1	TLE3	0.30976
BMLF1	B9D1	0.030509
BMLF1	STIM2	0.47478
BMLF1	KIN	0.043666
BMLF1	FUNDC1	0.31023
BMLF1	PCTP	0.019236
BMLF1	FAM109A	0.32426
BMLF1	GP1BA	0.26326
BMLF1	HUS1B	0.012321
BMLF1	XRN1	0.24751
BMLF1	WBP2	0.44546
BMLF1	CEACAM1	0.33715
BMLF1	NUP107	0.19842
BMLF1	VCAM1	0.023081
BMLF1	ERBB3	0.10137
BMLF1	CST6	0.038117
BMLF1	NEK6	0.077292
BMLF1	SYNPR	0.06924
BMLF1	ARL11	0.41402
BMLF1	CABYR	0.049239
BMLF1	MEGF9	0.23412
BMLF1	CBLC	0.12948
BMLF1	TRMT1L	0.023667
BMLF1	SLAIN2	0.40515
BMLF1	EIF4E3	0.10138
BMLF1	SSSCA1	0.4154

BMLF1	CCDC130	0.44542
BMLF1	BNIPL	0.332
BMLF1	KRT7	0.24808
BMLF1	ZHX2	0.14254
BMLF1	NUP54	0.010965
BMLF1	CTCFL	0.21002
BMLF1	RPLP1	0.29723
BMLF1	CER1	0.011751
BMLF1	DERL3	0.35902
BMLF1	KIAA1045	0.23305
BMLF1	SERGEF	0.34204
BMLF1	LY6G6F	0.040492
BMLF1	COG2	0.27288
BMLF1	CD247	0.28082
BMLF1	MAN1A2	0.10965
BMLF1	SLC23A2	0.037728
BMLF1	DSN1	0.28374
BMLF1	ELK1	0.15726
BMLF1	TRAF2	0.084845
BMLF1	GRIK1	0.012564
BMLF1	TCEA2	0.23349
BMLF1	NKAP	0.24937
BMLF1	FIS1	0.14275
BMLF1	TMED10	0.19763
BMLF1	RALBP1	0.28638
BMLF1	NDUFAF4	0.44799
BMLF1	BUB1B	0.11335
BMLF1	MSI2	0.038568
BMLF1	CST5	0.47077
BMLF1	NFKBIB	0.21604
BMLF1	THOC5	0.25163
BMLF1	NAIP	0.29802
BMLF1	DPY30	0.26754
BMLF1	EFCAB14	0.26298
BMLF1	TMOD3	0.014375
BMLF1	PVRL3	0.29576
BMLF1	LRIF1	0.36649
BMLF1	CORO7	0.01893
BMLF1	ANGEL1	0.023185
BMLF1	TNFAIP3	0.043866
BMLF1	PLCB4	0.12729
BMLF1	TNKS	0.11648
BMLF1	TMEM30B	0.43033
BMLF1	DDX55	0.014902
BMLF1	PYDC1	0.31509
BMLF1	PITPNM2	0.17555
BMLF1	SOX4	0.053159
BMLF1	NCAPD2	0.29272
BMLF1	MIA3	0.30531
BMLF1	UHRF2	0.2118
BMLF1	CGA	0.26086
BMLF1	PDIA3	0.15803
BMLF1	BIRC3	0.22081
BMLF1	PABPN1	0.23385
BMLF1	METTL21B	0.054759
BMLF1	SYPL2	0.02487
BMLF1	TAF3	0.24649
BMLF1	COMMD5	0.41402
BMLF1	DAZ3	0.011338
BMLF1	ACAN	0.25531
BMLF1	CLASP2	0.13939
BMLF1	NACA	0.28146
BMLF1	JAK2	0.022729
BMLF1	WIPF1	0.40157
BMLF1	SPTAN1	0.36794
BMLF1	AANAT	0.016251
BMLF1	NDE1	0.14371
BMLF1	BECN1	0.22389

BMLF1	PTPN3	0.1708
BMLF1	FZD5	0.059065
BMLF1	CD81	0.23262
BMLF1	ST8SIA3	0.011956
BMLF1	CHERP	0.035505
BMLF1	CDC37	0.27889
BMLF1	TNFAIP8L3	0.015318
BMLF1	ABCG4	0.016144
BMLF1	NOL8	0.38334
BMLF1	MT-ND4	0.026542
BMLF1	PPM1E	0.21124
BMLF1	SPNS1	0.36151
BMLF1	RIMS1	0.20532
BMLF1	ATRIP	0.39135
BMLF1	COPS2	0.3169
BMLF1	NDUFAB1	0.14888
BMLF1	PREP	0.021706
BMLF1	DDR1	0.14579
BMLF1	COL13A1	0.25763
BMLF1	RAD52	0.33803
BMLF1	TCF7	0.1198
BMLF1	LAMTOR1	0.23829
BMLF1	DHPS	0.40142
BMLF1	IL12A	0.15307
BMLF1	SMC3	0.16453
BMLF1	ARMCX4	0.039802
BMLF1	SNX24	0.16649
BMLF1	STAT5A	0.058249
BMLF1	THAP1	0.24413
BMLF1	CLP1	0.039054
BMLF1	TUBA3C	0.17278
BMLF1	CDK5RAP2	0.15163
BMLF1	PRX	0.33633
BMLF1	MCRS1	0.24897
BMLF1	POP4	0.34166
BMLF1	TARBP1	0.14961
BMLF1	FBLN1	0.19882
BMLF1	EDIL3	0.22496
BMLF1	AIMP1	0.22491
BMLF1	MASP1	0.52872
BMLF1	CARD17	0.024942
BMLF1	TBL1X	0.13289
BMLF1	AIF1	0.064119
BMLF1	ZNF205	0.28277
BMLF1	INPP5D	0.13266
BMLF1	GCC2	0.21938
BMLF1	THRB	0.094145
BMLF1	SCUBE3	0.65964
BMLF1	CALML6	0.16765
BMLF1	RABGGTA	0.018637
BMLF1	KIF13B	0.23183
BMLF1	SLC9A7	0.23315
BMLF1	CNOT3	0.27469
BMLF1	TM2D3	0.085995
BMLF1	PTPRM	0.12984
BMLF1	MFAP2	0.066065
BMLF1	BCL9	0.33544
BMLF1	NGFR	0.10807
BMLF1	TADA3	0.14382
BMLF1	STRAP	0.26282
BMLF1	TGM5	0.015082
BMLF1	ITPKC	0.063539
BMLF1	PROZ	0.16496
BMLF1	LSM3	0.31136
BMLF1	DDO	0.29229
BMLF1	PEA15	0.33976
BMLF1	RPS27L	0.27887
BMLF1	RBFOX1	0.36615

BMLF1	KLK9	0.049094
BMLF1	AMIGO2	0.34496
BMLF1	MTSS1	0.19309
BMLF1	PSMC3	0.12198
BMLF1	HUWE1	0.25059
BMLF1	UXT	0.20555
BMLF1	TRAPPC4	0.35641
BMLF1	TRIM17	0.32288
BMLF1	HECTD3	0.27243
BMLF1	TH	0.035619
BMLF1	TNFRSF10B	0.28144
BMLF1	SCAP	0.2464
BMLF1	C1QB	0.34843
BMLF1	TRIM13	0.1998
BMLF1	FBXO7	0.3401
BMLF1	HIPK1	0.13346
BMLF1	WDR93	0.047728
BMLF1	CAMK2A	0.15635
BMLF1	LYZL2	0.066586
BMLF1	CEP152	0.23525
BMLF1	PTPN6	0.069876
BMLF1	APOH	0.18695
BMLF1	XPO7	0.018779
BMLF1	HEXIM1	0.29232
BMLF1	PTPRS	0.17647
BMLF1	ATXN10	0.32904
BMLF1	SCN5A	0.10143
BMLF1	GEMIN5	0.33041
BMLF1	COMMD7	0.39824
BMLF1	FHIT	0.23114
BMLF1	ADAM17	0.066028
BMLF1	CSNK1A1	0.13654
BMLF1	ATG4B	0.32839
BMLF1	TERF1	0.1319
BMLF1	MAL2	0.38168
BMLF1	EEF1A1	0.13796
BMLF1	PCDHGB1	0.22489
BMLF1	AGTR1	0.13349
BMLF1	MEI4	0.054669
BMLF1	SEC24B	0.20257
BMLF1	ALYREF	0.22949
BMLF1	KPNA1	0.23915
BMLF1	SERINC1	0.28843
BMLF1	AATK	0.26376
BMLF1	NCF4	0.28264
BMLF1	SLC22A8	0.035702
BMLF1	MED8	0.27247
BMLF1	ATG16L1	0.33846
BMLF1	ESAM	0.014824
BMLF1	FABP12	0.045899
BMLF1	PARP15	0.034508
BMLF1	USP19	0.22242
BMLF1	RBM12	0.012783
BMLF1	RIN3	0.39268
BMLF1	LDOC1	0.41596
BMLF1	FSBP	0.36073
BMLF1	SH3D19	0.28489
BMLF1	TNS4	0.38072
BMLF1	SUV420H1	0.056088
BMLF1	MORF4L2	0.30426
BMLF1	TSR2	0.45782
BMLF1	SAV1	0.28005
BMLF1	EPB41	0.16316
BMLF1	CDH16	0.22489
BMLF1	PDE9A	0.22076
BMLF1	PEX19	0.24117
BMLF1	SYNCRIP	0.22824
BMLF1	NAGK	0.44148

BMLF1	SLC22A6	0.2761
BMLF1	BMP7	0.041094
BMLF1	PPAP2B	0.12331
BMLF1	RPS6	0.12116
BMLF1	TIA1	0.34975
BMLF1	SLC34A2	0.061179
BMLF1	SNRPG	0.24652
BMLF1	PRKCI	0.048889
BMLF1	TP53RK	0.29547
BMLF1	PCDH18	0.2181
BMLF1	MUC20	0.021709
BMLF1	TECPR1	0.35225
BMLF1	RRM2	0.25287
BMLF1	CD300LD	0.43034
BMLF1	KCNJ10	0.13775
BMLF1	GTPBP1	0.077827
BMLF1	PDP1	0.083192
BMLF1	CUL4A	0.29252
BMLF1	CD244	0.37029
BMLF1	GATA4	0.032065
BMLF1	KCNH1	0.19195
BMLF1	UFC1	0.44387
BMLF1	PAIP2B	0.43029
BMLF1	PTPRN	0.24126
BMLF1	KAT7	0.18598
BMLF1	PML	0.042377
BMLF1	RPGR	0.31547
BMLF1	ATP5G1	0.057256
BMLF1	KBTBD6	0.43187
BMLF1	WDR5	0.23105
BMLF1	GOLGA4	0.324
BMLF1	MALL	0.35284
BMLF1	CHMP4B	0.33128
BMLF1	TLK2	0.23309
BMLF1	NLRC4	0.18784
BMLF1	PROC	0.46081
BMLF1	PDCD6	0.50735
BMLF1	DKC1	0.30556
BMLF1	USP32	0.20026
BMLF1	SLC12A7	0.02628
BMLF1	RPS4Y1	0.014928
BMLF1	OTUD7B	0.27017
BMLF1	COG1	0.31727
BMLF1	S100A9	0.18081
BMLF1	EXOC1	0.019437
BMLF1	ANXA2	0.30807
BMLF1	TMPO	0.35583
BMLF1	PSMD2	0.18563
BMLF1	MMP20	0.41607
BMLF1	RAET1G	0.39187
BMLF1	DAZL	0.27905
BMLF1	NR2C2AP	0.38689
BMLF1	TRA2A	0.027445
BMLF1	MCM4	0.19376
BMLF1	PLCD3	0.028368
BMLF1	RTN4	0.12035
BMLF1	PPIL4	0.030788
BMLF1	RUNX1	0.12121
BMLF1	TOP3A	0.26237
BMLF1	CDKN1A	0.088906
BMLF1	KIF23	0.19649
BMLF1	IL13RA1	0.39921
BMLF1	EZH2	0.19285
BMLF1	MATN4	0.69673
BMLF1	SLX4IP	0.47224
BMLF1	POLG	0.28854
BMLF1	MGST3	0.037347
BMLF1	PSMF1	0.1836

35701	BMLF1	FMR1	0.34555
35702	BMLF1	MAP1A	0.24488
35703	BMLF1	NOTCH3	0.26335
35704	BMLF1	DNMT3L	0.27154
35705	BMLF1	VPS72	0.31332
35706	BMLF1	TIMM50	0.17532
35707	BMLF1	CXXC1	0.23177
35708	BMLF1	PVALB	0.25001
35709	BMLF1	RNH1	0.65508
35710	BMLF1	LRRC47	0.42246
35711	BMLF1	UBAP2	0.025065
35712	BMLF1	FOXRED2	0.46067
35713	BMLF1	PKM	0.1668
35714	BMLF1	GFRA2	0.044198
35715	BMLF1	ARHGEF6	0.20753
35716	BMLF1	TNNT1	0.013246
35717	BMLF1	PIK3C2B	0.26162
35718	BMLF1	MFF	0.22751
35719	BMLF1	ATF2	0.046373
35720	BMLF1	MMRN2	0.41424
35721	BMLF1	FBXL12	0.43033
35722	BMLF1	THOC7	0.32819
35723	BMLF1	EGFL7	0.19619
35724	BMLF1	POR	0.078928
35725	BMLF1	ASPN	0.23277
35726	BMLF1	ANO8	0.02331
35727	BMLF1	SATL1	0.018989
35728	BMLF1	MYL1	0.19331
35729	BMLF1	ARL4D	0.27195
35730	BMLF1	NUMA1	0.17255
35731	BMLF1	MED23	0.26681
35732	BMLF1	ADIPOQ	0.036328
35733	BMLF1	PTAFR	0.035731
35734	BMLF1	PRAMEF1	0.049776
35735	BMLF1	VWA8	0.011919
35736	BMLF1	TBC1D24	0.34871
35737	BMLF1	CFB	0.044702
35738	BMLF1	HLA-DRB1	0.10678
35739	BMLF1	MED22	0.28886
35740	BMLF1	TDRKH	0.050618
35741	BMLF1	TGFB3	0.058689
35742	BMLF1	KIR2DL3	0.32441
35743	BMLF1	EGLN1	0.19113
35744	BMLF1	TRDMT1	0.078211
35745	BMLF1	PLXNC1	0.29935
35746	BMLF1	SIAH1	0.15483
35747	BMLF1	FSCB	0.2247
35748	BMLF1	C2CD5	0.23504
35749	BMLF1	SNX27	0.28382
35750	BMLF1	PTPRJ	0.081851
35751	BMLF1	NDUFA9	0.27724
35752	BMLF1	PRKCA	0.017521
35753	BMLF1	PVRL4	0.33687
35754	BMLF1	PPP6R1	0.3769
35755	BMLF1	FRS2	0.1849
35756	BMLF1	SLC13A4	0.044689
35757	BMLF1	MEF2BNB	0.46518
35758	BMLF1	SPSB2	0.39744
35759	BMLF1	ROBO1	0.15838
35760	BMLF1	PAIP1	0.33719
35761	BMLF1	WRAP53	0.42682
35762	BMLF1	MORF4L1	0.27106
35763	BMLF1	SELPLG	0.29061
35764	BMLF1	CLIP1	0.26394
35765	BMLF1	ATP1A1	0.13369
35766	BMLF1	BBS9	0.32124
35767	BMLF1	YTHDF3	0.47224
35768	BMLF1	HCK	0.094442

BMLF1	USP6	0.23289
BMLF1	TEK	0.066516
BMLF1	TAP2	0.10877
BMLF1	UGP2	0.23534
BMLF1	EXOSC5	0.27275
BMLF1	UBXN7	0.37417
BMLF1	TSHR	0.29564
BMLF1	NECAB1	0.16765
BMLF1	PLXDC2	0.44386
BMLF1	BLOC1S3	0.23367
BMLF1	AMOT	0.21028
BMLF1	DNAAF2	0.042542
BMLF1	MAATS1	0.44394
BMLF1	TBC1D8	0.18076
BMLF1	CHRNA1	0.010607
BMLF1	SLC17A9	0.010316
BMLF1	THY1	0.086403
BMLF1	ALOX5AP	0.20009
BMLF1	DLK2	0.24538
BMLF1	BHLHE40	0.12024
BMLF1	TTLL6	0.28661
BMLF1	SLC4A1	0.22751
BMLF1	PTK7	0.17748
BMLF1	ADI1	0.25153
BMLF1	PCDHB5	0.20505
BMLF1	PAGE5	0.021888
BMLF1	SPAG5	0.21535
BMLF1	TOB1	0.3725
BMLF1	MYOD1	0.060883
BMLF1	BORA	0.30896
BMLF1	WDR12	0.29714
BMLF1	MAN1C1	0.119
BMLF1	HM13	0.24888
BMLF1	JUNB	0.072102
BMLF1	SLC25A17	0.20003
BMLF1	RAB35	0.016385
BMLF1	CHUK	0.051865
BMLF1	SLC6A11	0.022762
BMLF1	VPS28	0.30121
BMLF1	CHRNB4	0.015934
BMLF1	URM1	0.39308
BMLF1	NUF2	0.27937
BMLF1	CABS1	0.21868
BMLF1	KLF10	0.1342
BMLF1	NAPB	0.022217
BMLF1	MAGEA2	0.29464
BMLF1	TRAK2	0.22467
BMLF1	PRKACA	0.036227
BMLF1	SLC16A13	0.031311
BMLF1	CASP8AP2	0.25255
BMLF1	MZT2B	0.3839
BMLF1	FNTB	0.24147
BMLF1	RNF7	0.2822
BMLF1	HNRNPR	0.32065
BMLF1	CHFR	0.24264
BMLF1	CLN3	0.065519
BMLF1	CRTC1	0.26213
BMLF1	QKI	0.29076
BMLF1	RTN4R	0.34054
BMLF1	RBM15	0.33646
BMLF1	VEZT	0.058246
BMLF1	STXBP2	0.23014
BMLF1	KSR1	0.15778
BMLF1	GSTA5	0.041163
BMLF1	POLR1A	0.25071
BMLF1	EPCAM	0.35501
BMLF1	UMODL1	0.20889
BMLF1	GNL3	0.2886

35837	BMLF1	DENND1C	0.061394
35838	BMLF1	VPS33B	0.25099
35839	BMLF1	CLPX	0.25328
35840	BMLF1	UACA	0.24329
35841	BMLF1	GLS	0.25513
35842	BMLF1	PAK3	0.14065
35843	BMLF1	HAUS6	0.030987
35844	BMLF1	C5orf30	0.33708
35845	BMLF1	MMP3	0.035317
35846	BMLF1	PRSS53	0.049094
35847	BMLF1	MYL4	0.18526
35848	BMLF1	CD8B	0.28821
35849	BMLF1	EIF4G2	0.38953
35850	BMLF1	CNN2	0.023181
35851	BMLF1	AKIRIN2	0.060209
35852	BMLF1	TOM1L1	0.28365
35853	BMLF1	CAPN14	0.14594
35854	BMLF1	RABGGTB	0.39903
35855	BMLF1	KCNK1	0.024444
35856	BMLF1	COLQ	0.2705
35857	BMLF1	ARHGEF7	0.10035
35858	BMLF1	THRA	0.041536
35859	BMLF1	IL1RL2	0.023289
35860	BMLF1	NPAT	0.18667
35861	BMLF1	ORAI1	0.26871
35862	BMLF1	RAD21	0.22771
35863	BMLF1	LVP1a	0.080977
35864	BMLF1	MAPT	0.095104
35865	BMLF1	UCK1	0.096265
35866	BMLF1	CD80	0.26229
35867	BMLF1	PCDH11Y	0.22489
35868	BMLF1	METTL1	0.33742
35869	BMLF1	MRFAP1	0.40347
35870	BMLF1	MMS19	0.21105
35871	BMLF1	ITGB3	0.09473
35872	BMLF1	INSR	0.030271
35873	BMLF1	TLN2	0.19509
35874	BMLF1	TRABD2B	0.23155
35875	BMLF1	ASB9	0.36411
35876	BMLF1	TRPC3	0.22461
35877	BMLF1	GATA2	0.04644
35878	BMLF1	CIZ1	0.31617
35879	BMLF1	OVCH1	0.02953
35880	BMLF1	CHD8	0.1471
35881	BMLF1	RPL11	0.21479
35882	BMLF1	NLRP1	0.21028
35883	BMLF1	NCBP1	0.15787
35884	BMLF1	HDAC4	0.03549
35885	BMLF1	SLC2A13	0.035787
35886	BMLF1	MMP10	0.097389
35887	BMLF1	EDC3	0.37066
35888	BMLF1	MAD2L2	0.2393
35889	BMLF1	MGRN1	0.21928
35890	BMLF1	CEP76	0.35726
35891	BMLF1	WDR77	0.26581
35892	BMLF1	CRKL	0.31461
35893	BMLF1	USP7	0.16007
35894	BMLF1	PPP2R2B	0.26317
35895	BMLF1	BGLAP	0.22903
35896	BMLF1	TIMP3	0.26808
35897	BMLF1	RPL13	0.29723
35898	BMLF1	ALDH7A1	0.29175
35899	BMLF1	GDI2	0.29222
35900	BMLF1	PTK6	0.16476
35901	BMLF1	SLC36A4	0.02916
35902	BMLF1	MESDC1	0.01537
35903	BMLF1	TPSG1	0.046166
35904	BMLF1	ZRSR2	0.29044

BMLF1	CD200R1	0.36857
BMLF1	UHRF1	0.1224
BMLF1	SULT2A1	0.31816
BMLF1	IFIT5	0.043748
BMLF1	NEBL	0.36777
BMLF1	KLKB1	0.3095
BMLF1	TMF1	0.27581
BMLF1	IL1R1	0.27413
BMLF1	LATS2	0.15786
BMLF1	MTA2	0.10406
BMLF1	CREB3L3	0.19107
BMLF1	METTL6	0.059751
BMLF1	METTL13	0.058034
BMLF1	NCOA7	0.30108
BMLF1	PCDHA5	0.21127
BMLF1	RABGAP1	0.29459
BMLF1	PARP12	0.029105
BMLF1	GK3P	0.044086
BMLF1	SMARCD1	0.29612
BMLF1	SHROOM2	0.17527
BMLF1	ZNF148	0.13038
BMLF1	SCN2A	0.015375
BMLF1	PCDH11X	0.21134
BMLF1	KBTBD7	0.43187
BMLF1	SLC6A15	0.024586
BMLF1	ERCC4	0.13329
BMLF1	CYBA	0.099158
BMLF1	PABPC3	0.041775
BMLF1	LTBR	0.29611
BMLF1	ARHGAP8	0.34369
BMLF1	MYOCD	0.070701
BMLF1	PRDM7	0.045845
BMLF1	RBM11	0.010387
BMLF1	UHRF1BP1	0.42094
BMLF1	GH1	0.23642
BMLF1	CTH	0.13129
BMLF1	MARCH1	0.045135
BMLF1	SHANK2	0.35344
BMLF1	CDH20	0.21805
BMLF1	SLA2	0.34196
BMLF1	CLTC	0.20671
BMLF1	XRCC6	0.069372
BMLF1	GATA1	0.035229
BMLF1	NADK2	0.084922
BMLF1	PPARA	0.050349
BMLF1	PNKP	0.03281
BMLF1	ASH1L	0.025627
BMLF1	GPIHBP1	0.14506
BMLF1	SF3B5	0.038072
BMLF1	RCAN3	0.037518
BMLF1	FCGR1A	0.30501
BMLF1	SH3GL1	0.39628
BMLF1	ARVCF	0.36713
BMLF1	CBX2	0.25652
BMLF1	PLEKHA5	0.31838
BMLF1	FOXD3	0.078408
BMLF1	EIF3F	0.37304
BMLF1	PPP3R2	0.26298
BMLF1	CIITA	0.20907
BMLF1	UPF2	0.37429
BMLF1	NECAP1	0.045462
BMLF1	CACNB2	0.24116
BMLF1	SIN3B	0.25004
BMLF1	TNPO3	0.29865
BMLF1	CACNA1C	0.18063
BMLF1	SIK1	0.086992
BMLF1	SH3RF1	0.24768
BMLF1	TRPM6	0.24816

BMLF1	RAB5A	0.20641
BMLF1	ERBB2IP	0.22696
BMLF1	LPCAT2	0.11952
BMLF1	GPRC5B	0.054212
BMLF1	OPA1	0.17867
BMLF1	PPP2R5D	0.33247
BMLF1	BIN1	0.24559
BMLF1	BCL2L11	0.081188
BMLF1	SECTM1	0.082456
BMLF1	EIF4ENIF1	0.28581
BMLF1	NME7	0.022492
BMLF1	SYNRG	0.19214
BMLF1	GNAI1	0.15104
BMLF1	HIST2H3A	0.29272
BMLF1	NRIP1	0.14507
BMLF1	ADCK2	0.01553
BMLF1	PSMB9	0.11787
BMLF1	RBMY1A1	0.41418
BMLF1	EEF1G	0.33402
BMLF1	CPEB4	0.03147
BMLF1	SLC6A19	0.024798
BMLF1	COL7A1	0.32277
BMLF1	STAT4	0.095224
BMLF1	RAG1	0.1448
BMLF1	RRAD	0.34562
BMLF1	GID4	0.017481
BMLF1	STX4	0.17604
BMLF1	PPP1R10	0.022144
BMLF1	LOXL4	0.3375
BMLF1	GFI1	0.15711
BMLF1	SIK3	0.24737
BMLF1	FAM83F	0.059816
BMLF1	ROBO2	0.19805
BMLF1	CALCA	0.15894
BMLF1	NUP214	0.22243
BMLF1	NAA50	0.45831
BMLF1	STAU1	0.33699
BMLF1	YKT6	0.096427
BMLF1	CHAMP1	0.21863
BMLF1	ARHGEF15	0.31786
BMLF1	THOC3	0.043627
BMLF1	TPCN1	0.2187
BMLF1	MERTK	0.16764
BMLF1	MRPL20	0.32765
BMLF1	SRF	0.021606
BMLF1	DNAJC8	0.024169
BMLF1	PLCH1	0.015631
BMLF1	UBL4A	0.36218
BMLF1	CTRL	0.047945
BMLF1	CREBZF	0.20744
BMLF1	PELP1	0.36934
BMLF1	NFKB1	0.041175
BMLF1	STAT3	0.053428
BMLF1	ZNF488	0.25782
BMLF1	NCR3	0.031606
BMLF1	RHBDL2	0.085753
BMLF1	SLC5A2	0.08574
BMLF1	SLC24A5	0.036879
BMLF1	ADAD1	0.065097
BMLF1	ACIN1	0.3025
BMLF1	BARD1	0.21917
BMLF1	PPFIA4	0.41727
BMLF1	CKB	0.31381
BMLF1	TNFSF12	0.21385
BMLF1	TNS3	0.40517
BMLF1	FAM160A2	0.36527
BMLF1	KMT2C	0.28456
BMLF1	KLHL20	0.25738

BMLF1	RAD23A	0.28098
BMLF1	SMG9	0.35321
BMLF1	PAK1	0.042221
BMLF1	HPCAL4	0.24248
BMLF1	VPS4A	0.20673
BMLF1	COCH	0.36512
BMLF1	PSPC1	0.33278
BMLF1	STK40	0.25988
BMLF1	SRPX2	0.29979
BMLF1	PXDNL	0.058601
BMLF1	ORC4	0.25651
BMLF1	THBD	0.39966
BMLF1	GLYCTK	0.43661
BMLF1	SPATC1L	0.039658
BMLF1	ARHGEF4	0.18466
BMLF1	SMAD5	0.1014
BMLF1	LMOD3	0.014706
BMLF1	NOVA2	0.023545
BMLF1	KIAA0226	0.34803
BMLF1	DCHS1	0.21802
BMLF1	CREM	0.17187
BMLF1	LPO	0.062021
BMLF1	ZFR	0.36605
BMLF1	RTP4	0.39952
BMLF1	FSHR	0.21298
BMLF1	FANCM	0.25469
BMLF1	EEF1D	0.31186
BMLF1	TRADD	0.18328
BMLF1	PYGO1	0.30702
BMLF1	DHX8	0.31015
BMLF1	NEFH	0.06325
BMLF1	MTERF	0.28932
BMLF1	ATG13	0.28542
BMLF1	CHST5	0.043467
BMLF1	BRAF	0.19519
BMLF1	NPBWR2	0.34294
BMLF1	ARPC2	0.28892
BMLF1	ARFGEF2	0.14398
BMLF1	TTC19	0.32947
BMLF1	BSG	0.2199
BMLF1	MT2A	0.28688
BMLF1	AIG1	0.44386
BMLF1	ORM1	0.38362
BMLF1	SLC18A3	0.06327
BMLF1	HSD17B10	0.19288
BMLF1	RBM5	0.26391
BMLF1	PARP6	0.064778
BMLF1	BOP1	0.28356
BMLF1	NOX1	0.11858
BMLF1	GYG1	0.33094
BMLF1	DSC2	0.19233
BMLF1	CAPS	0.20661
BMLF1	CLSPN	0.29683
BMLF1	MLST8	0.32147
BMLF1	UPK3BL	0.02786
BMLF1	WTIP	0.2241
BMLF1	SPTB	0.21692
BMLF1	IL1R2	0.42195
BMLF1	KLF13	0.22855
BMLF1	MSN	0.14222
BMLF1	FETUB	0.037114
BMLF1	SPDL1	0.22232
BMLF1	UBE2D4	0.27572
BMLF1	PCSK1	0.038061
BMLF1	SMARCE1	0.23424
BMLF1	PIGA	0.30836
BMLF1	RPS26	0.23408
BMLF1	MEN1	0.059184

BMLF1	RAVER1	0.011483
BMLF1	GNMT	0.30969
BMLF1	PRSS45	0.050518
BMLF1	BRE	0.28591
BMLF1	ZBTB14	0.27419
BMLF1	BEX2	0.075645
BMLF1	SCAMP1	0.33024
BMLF1	SEC23A	0.19252
BMLF1	RND1	0.23007
BMLF1	BCKDHB	0.32077
BMLF1	PDLIM7	0.27648
BMLF1	SSH1	0.11786
BMLF1	RNMT	0.39148
BMLF1	TNFRSF21	0.36739
BMLF1	TTLL3	0.044569
BMLF1	FN3K	0.056235
BMLF1	AK5	0.097348
BMLF1	KIFC3	0.1065
BMLF1	TTF2	0.19942
BMLF1	TOPORS	0.12638
BMLF1	DDX47	0.32911
BMLF1	FREM2	0.17173
BMLF1	GOLM1	0.41701
BMLF1	GNAI2	0.11401
BMLF1	DDX42	0.34541
BMLF1	GAPDHS	0.27191
BMLF1	CDKN1C	0.28829
BMLF1	GRIA1	0.1523
BMLF1	ANKLE2	0.28811
BMLF1	NUP153	0.17361
BMLF1	ETS2	0.15639
BMLF1	TOMM22	0.2569
BMLF1	VPS41	0.23972
BMLF1	MAGOH	0.26737
BMLF1	SKI	0.063316
BMLF1	SRGN	0.23676
BMLF1	CEP41	0.26019
BMLF1	RAC1	0.052693
BMLF1	INTS9	0.40314
BMLF1	IPO5	0.27546
BMLF1	RPUSD3	0.11174
BMLF1	VDAC1	0.18412
BMLF1	PRMT5	0.21219
BMLF1	MUC16	0.33504
BMLF1	TNFAIP1	0.26252
BMLF1	GRIN2A	0.020853
BMLF1	CRNN	0.2325
BMLF1	GSTM2	0.04854
BMLF1	ING4	0.22802
BMLF1	PECAM1	0.28594
BMLF1	EML1	0.65472
BMLF1	GTF2F2	0.18243
BMLF1	CEP170	0.38318
BMLF1	AKAP4	0.027062
BMLF1	GJA8	0.010077
BMLF1	BRK1	0.029187
BMLF1	C19orf68	0.47224
BMLF1	ACRBP	0.069112
BMLF1	NOL3	0.30804
BMLF1	ZWILCH	0.32653
BMLF1	VAV2	0.14521
BMLF1	KIF24	0.35982
BMLF1	MAGED1	0.25882
BMLF1	ETS1	0.068204
BMLF1	PTGER4	0.17727
BMLF1	RPRD1B	0.29555
BMLF1	CWC15	0.42896
BMLF1	CALCRL	0.14398

BMLF1	AK1	0.098958
BMLF1	DES	0.24771
BMLF1	WDR4	0.3624
BMLF1	PSMC1	0.15113
BMLF1	USP5	0.25439
BMLF1	CITED1	0.058024
BMLF1	IP6K1	0.028994
BMLF1	HAT1	0.30851
BMLF1	SNCAIP	0.32005
BMLF1	FZD3	0.17077
BMLF1	LIN28A	0.205
BMLF1	MPDZ	0.29002
BMLF1	NGFRAP1	0.29022
BMLF1	ARID2	0.27866
BMLF1	PGS1	0.14637
BMLF1	TLDC1	0.47224
BMLF1	COMMD3	0.47224
BMLF1	SMAD7	0.05936
BMLF1	SERPINA1	0.30633
BMLF1	CAV1	0.01235
BMLF1	NCK1	0.18177
BMLF1	GABBR1	0.12043
BMLF1	ADAD2	0.10268
BMLF1	CRTAC1	0.26139
BMLF1	SCP2	0.12458
BMLF1	TMEM173	0.11469
BMLF1	CDK5R2	0.020061
BMLF1	FAR1	0.051306
BMLF1	MAD2L1	0.18352
BMLF1	PRRC2B	0.47224
BMLF1	CLN6	0.30169
BMLF1	KCNJ3	0.29734
BMLF1	BYSL	0.32183
BMLF1	ERN1	0.091785
BMLF1	EYA1	0.049005
BMLF1	SPRTN	0.31483
BMLF1	SFSWAP	0.019924
BMLF1	MYD88	0.092488
BMLF1	LSM5	0.32746
BMLF1	CTC1	0.2911
BMLF1	FBXW8	0.43037
BMLF1	SPPL2A	0.249
BMLF1	PLS1	0.2321
BMLF1	SYVN1	0.21083
BMLF1	CACYBP	0.31289
BMLF1	DPP8	0.043975
BMLF1	INS	0.052225
BMLF1	IRAK4	0.18064
BMLF1	EPS15	0.33482
BMLF1	DIMT1	0.11047
BMLF1	ALG1L	0.015725
BMLF1	PLS3	0.24729
BMLF1	MYO5B	0.30749
BMLF1	WWC1	0.24023
BMLF1	HUS1	0.27353
BMLF1	PCSK2	0.37623
BMLF1	CRHR1	0.11296
BMLF1	WHAMM	0.15271
BMLF1	APLF	0.27682
BMLF1	YY1	0.12065
BMLF1	NKRF	0.3135
BMLF1	ZNF335	0.28277
BMLF1	AURKB	0.096134
BMLF1	CDC42	0.019812
BMLF1	RRAGC	0.20338
BMLF1	LZTS1	0.13177
BMLF1	CD48	0.27094
BMLF1	VWCE	0.25502

BMLF1	CLEC3B	0.18104
BMLF1	PRMT8	0.31684
BMLF1	SLC17A7	0.048135
BMLF1	INPP5B	0.25045
BMLF1	ARHGEF16	0.1863
BMLF1	BST2	0.17152
BMLF1	NHLH2	0.25956
BMLF1	PEX11G	0.35851
BMLF1	SYT12	0.054739
BMLF1	OGDHL	0.38366
BMLF1	FKBP8	0.19694
BMLF1	DISC1	0.19214
BMLF1	IL33	0.27627
BMLF1	PUM2	0.44132
BMLF1	ESPL1	0.22284
BMLF1	PCGF1	0.27527
BMLF1	CDH8	0.20658
BMLF1	LTC4S	0.17732
BMLF1	AP3S1	0.3803
BMLF1	UHMK1	0.25407
BMLF1	ASCC1	0.043406
BMLF1	LAT2	0.35361
BMLF1	BUB3	0.21022
BMLF1	ALDH3A1	0.062188
BMLF1	RBMY1B	0.40188
BMLF1	LYZL6	0.068224
BMLF1	SPRR3	0.26633
BMLF1	SDF4	0.47313
BMLF1	KIF14	0.30802
BMLF1	AFF4	0.22145
BMLF1	DNASE1L1	0.011243
BMLF1	LEPR	0.34618
BMLF1	ANKHD1	0.45452
BMLF1	MGP	0.14705
BMLF1	BCKDHA	0.33577
BMLF1	TRIM33	0.1581
BMLF1	PRDM6	0.037237
BMLF1	KIF5B	0.24444
BMLF1	HEG1	0.11996
BMLF1	PRKCQ	0.055159
BMLF1	PHIP	0.26168
BMLF1	KRTAP2-2	0.045197
BMLF1	ROBO3	0.017459
BMLF1	TAF11	0.28107
BMLF1	TRPC7	0.24458
BMLF1	LAMTOR2	0.29368
BMLF1	MAZ	0.29516
BMLF1	HIST1H1E	0.32278
BMLF1	WSB1	0.42135
BMLF1	CDC45	0.28003
BMLF1	YIPF5	0.054209
BMLF1	YAF2	0.25331
BMLF1	NSUN4	0.055779
BMLF1	MYH9	0.088904
BMLF1	EID3	0.29377
BMLF1	SPACA3	0.32363
BMLF1	HHAT	0.039762
BMLF1	PTPLB	0.30474
BMLF1	INSIG2	0.20344
BMLF1	CDCA8	0.2424
BMLF1	LGI3	0.055487
BMLF1	GLTP	0.013846
BMLF1	STAT1	0.10094
BMLF1	GPATCH1	0.026152
BMLF1	PRKAB1	0.1807
BMLF1	GJA1	0.053817
BMLF1	PSEN2	0.036013
BMLF1	FAT4	0.63413

BMLF1	DTNBP1	0.19343
BMLF1	CUL9	0.32031
BMLF1	PNMA2	0.40475
BMLF1	WWP1	0.20669
BMLF1	BPIFB3	0.022337
BMLF1	PLIN3	0.30876
BMLF1	KCNIP4	0.18364
BMLF1	USP34	0.332
BMLF1	ZNHIT6	0.35963
BMLF1	VPS18	0.25391
BMLF1	ARHGAP4	0.25345
BMLF1	IFI16	0.11472
BMLF1	PPEF1	0.52231
BMLF1	AP1M2	0.072319
BMLF1	ZW10	0.13815
BMLF1	GRIN3A	0.15355
BMLF1	CANT1	0.12719
BMLF1	CXorf67	0.10862
BMLF1	NPW	0.38386
BMLF1	MAPK11	0.11334
BMLF1	TLL1	0.096358
BMLF1	ATP13A2	0.011639
BMLF1	JAG1	0.016477
BMLF1	A2M	0.22859
BMLF1	DCTN1	0.24178
BMLF1	FTSJ3	0.43282
BMLF1	ETV1	0.19003
BMLF1	KLK4	0.028743
BMLF1	PRKAA2	0.043268
BMLF1	NELL2	0.54984
BMLF1	CALB2	0.2656
BMLF1	PAICS	0.25384
BMLF1	MYH6	0.023024
BMLF1	DDX60	0.28993
BMLF1	CLTB	0.33216
BMLF1	PCDHA7	0.21127
BMLF1	CHD4	0.13184
BMLF1	ZC3H10	0.39591
BMLF1	ARRB2	0.1027
BMLF1	PRDX1	0.27193
BMLF1	RHOG	0.21367
BMLF1	SLC2A8	0.063611
BMLF1	TTC3	0.2473
BMLF1	SPON1	0.41705
BMLF1	IRAK3	0.12156
BMLF1	CYFIP1	0.24103
BMLF1	SEC11A	0.038462
BMLF1	NR2F1	0.14338
BMLF1	ULK2	0.30814
BMLF1	SRSF3	0.33867
BMLF1	PDZK1	0.34259
BMLF1	MCC	0.32376
BMLF1	KAT2B	0.067411
BMLF1	CROCC	0.2364
BMLF1	LAMTOR3	0.33399
BMLF1	ARRB1	0.069085
BMLF1	ELOVL4	0.28122
BMLF1	CAPN9	0.13872
BMLF1	FAM188B2	0.11541
BMLF1	UBE3A	0.18158
BMLF1	SRSF8	0.011243
BMLF1	TCF4	0.13885
BMLF1	GTF3C1	0.28608
BMLF1	EIF1AX	0.094784
BMLF1	SMC1A	0.13901
BMLF1	DMAP1	0.24776
BMLF1	ACAD9	0.015568
BMLF1	CHEK2	0.082043

BMLF1	STX1A	0.13583
BMLF1	MYEF2	0.014128
BMLF1	DRD2	0.020691
BMLF1	APH1A	0.20646
BMLF1	GBAS	0.39645
BMLF1	PABPN1L	0.038568
BMLF1	BAZ1B	0.18087
BMLF1	AKIP1	0.41608
BMLF1	PCDHGC5	0.22489
BMLF1	KAT2A	0.1738
BMLF1	COL4A3BP	0.10602
BMLF1	SEMA4C	0.070237
BMLF1	MSH2	0.01894
BMLF1	TRPC4	0.19693
BMLF1	KIF5C	0.29444
BMLF1	FZD10	0.17758
BMLF1	PIN1	0.22593
BMLF1	TLX3	0.19939
BMLF1	SUV420H2	0.34152
BMLF1	SCGB1D1	0.03677
BMLF1	PCDHGA7	0.22489
BMLF1	HECTD1	0.30238
BMLF1	TNNI1	0.016037
BMLF1	NME1	0.19104
BMLF1	CARD16	0.033346
BMLF1	NOP58	0.32508
BMLF1	MAP1LC3C	0.439
BMLF1	RFC3	0.2205
BMLF1	PSMD6	0.013519
BMLF1	ATP6V1E1	0.24557
BMLF1	ATP11B	0.16656
BMLF1	RNF14	0.18929
BMLF1	TARBP2	0.21597
BMLF1	SFR1	0.41185
BMLF1	TAB1	0.16516
BMLF1	RHOA	0.052852
BMLF1	KCNH2	0.15322
BMLF1	KRT20	0.3357
BMLF1	NUDCD3	0.019892
BMLF1	ZNF646	0.28277
BMLF1	MAP4K4	0.26357
BMLF1	DCAF11	0.4511
BMLF1	THBS1	0.063274
BMLF1	RUNX3	0.12643
BMLF1	LMTK2	0.2076
BMLF1	GADD45GIP1	0.36776
BMLF1	DIS3	0.24104
BMLF1	ARR3	0.32131
BMLF1	ABCD3	0.23194
BMLF1	UBR2	0.2182
BMLF1	CMKLR1	0.25806
BMLF1	STK25	0.22731
BMLF1	FANCF	0.38287
BMLF1	SEMG1	0.30027
BMLF1	PLA2G4A	0.036091
BMLF1	SLC38A7	0.033127
BMLF1	ICAM3	0.31842
BMLF1	FAM69C	0.13754
BMLF1	MRPL49	0.37763
BMLF1	CAPN8	0.13733
BMLF1	PANK4	0.080943
BMLF1	SLC22A23	0.039472
BMLF1	EIF3I	0.42145
BMLF1	ARL2BP	0.32857
BMLF1	UBTF	0.26927
BMLF1	SPACA4	0.060386
BMLF1	FGF2	0.038024
BMLF1	PFN1	0.21199

BMLF1	DLG3	0.22262
BMLF1	RBCK1	0.13344
BMLF1	DSG2	0.16949
BMLF1	MAP3K12	0.14132
BMLF1	SLC6A1	0.029293
BMLF1	POMT1	0.033926
BMLF1	CCNE1	0.10306
BMLF1	COMTD1	0.053261
BMLF1	RHOC	0.13023
BMLF1	PITX2	0.029839
BMLF1	ADRA2B	0.18592
BMLF1	ATXN3	0.17469
BMLF1	EPB41L3	0.24611
BMLF1	CEBPG	0.12212
BMLF1	ALDH16A1	0.090188
BMLF1	DEDD2	0.23503
BMLF1	MGMT	0.11841
BMLF1	GIT2	0.28549
BMLF1	SNX33	0.44385
BMLF1	RBM38	0.015281
BMLF1	CST9L	0.034289
BMLF1	STK24	0.17204
BMLF1	PCDHB2	0.19264
BMLF1	ATG101	0.36367
BMLF1	MIF	0.15959
BMLF1	ISG20L2	0.30157
BMLF1	BICD1	0.21221
BMLF1	AP2M1	0.23683
BMLF1	ING2	0.16185
BMLF1	PIK3CG	0.10664
BMLF1	ADORA1	0.05537
BMLF1	CDC42EP2	0.2743
BMLF1	SF3A1	0.36053
BMLF1	PRSS47	0.029242
BMLF1	INTS3	0.37079
BMLF1	DSG4	0.1747
BMLF1	ACOX1	0.012384
BMLF1	METTL2B	0.059751
BMLF1	TCTEX1D4	0.47224
BMLF1	TMCC2	0.44386
BMLF1	PLG	0.15119
BMLF1	TEAD3	0.17917
BMLF1	ROCK1	0.055238
BMLF1	NOS2	0.1195
BMLF1	LPCAT3	0.053203
BMLF1	PRMT1	0.34553
BMLF1	CCSER1	0.012201
BMLF1	USP10	0.23467
BMLF1	LASP1	0.26697
BMLF1	ALG1L2	0.015725
BMLF1	MT-ND1	0.31101
BMLF1	RASSF5	0.36075
BMLF1	CLTA	0.051858
BMLF1	CRB2	0.23256
BMLF1	DYNLT1	0.18987
BMLF1	KIF16B	0.028607
BMLF1	PLA2G2A	0.022487
BMLF1	CTSG	0.022434
BMLF1	ZMIZ2	0.26125
BMLF1	ALDOB	0.082698
BMLF1	S100Z	0.26298
BMLF1	PNO1	0.024242
BMLF1	CSTF2T	0.011252
BMLF1	MED26	0.26108
BMLF1	LRP5	0.13961
BMLF1	HSD17B12	0.21184
BMLF1	DGKQ	0.24798
BMLF1	CRP	0.27977

BMLF1	HK3	0.021797
BMLF1	ARIH2	0.23461
BMLF1	RNPC3	0.011854
BMLF1	ZNHIT3	0.027648
BMLF1	DHX36	0.27434
BMLF1	C1QBP	0.28233
BMLF1	DDOST	0.26176
BMLF1	AGR3	0.50141
BMLF1	AP1M1	0.28473
BMLF1	PUF60	0.27679
BMLF1	GMCL1P1	0.402
BMLF1	ECM29	0.062025
BMLF1	STRA13	0.28684
BMLF1	EPB41L5	0.017872
BMLF1	NOL9	0.088451
BMLF1	NECAP2	0.044185
BMLF1	SNX8	0.32838
BMLF1	MAPK6	0.19057
BMLF1	RPL23A	0.22879
BMLF1	GALK1	0.058315
BMLF1	SPOP	0.39506
BMLF1	PRKCZ	0.069299
BMLF1	HDDC2	0.34725
BMLF1	FANCI	0.37025
BMLF1	CYCS	0.16467
BMLF1	RDH12	0.024758
BMLF1	SETDB1	0.29264
BMLF1	CDC14A	0.124
BMLF1	SEC22B	0.26809
BMLF1	ATP7A	0.032591
BMLF1	AGR2	0.45742
BMLF1	UQCRC2	0.20182
BMLF1	FHL2	0.11536
BMLF1	ADM	0.11321
BMLF1	LARP4	0.037432
BMLF1	TPPP	0.48426
BMLF1	ZNF746	0.22091
BMLF1	GOLGA8CP	0.12419
BMLF1	ARC	0.031041
BMLF1	CAMKV	0.043188
BMLF1	PRPF4	0.32975
BMLF1	DBNDD2	0.44386
BMLF1	BMP10	0.16534
BMLF1	TBXA2R	0.19135
BMLF1	FYB	0.2699
BMLF1	TTC14	0.053548
BMLF1	SYT4	0.22922
BMLF1	TCF7L2	0.014884
BMLF1	IL17RE	0.41722
BMLF1	CENPT	0.26143
BMLF1	RAD51D	0.32674
BMLF1	KCNK12	0.020607
BMLF1	SYT11	0.53983
BMLF1	CD5	0.31175
BMLF1	CCDC150	0.015575
BMLF1	SH2D4A	0.37508
BMLF1	ICAM1	0.08973
BMLF1	GZMB	0.29736
BMLF1	PSME2	0.017209
BMLF1	GGA2	0.28301
BMLF1	MAD1L1	0.19298
BMLF1	C1orf105	0.03315
BMLF1	PRADC1	0.042199
BMLF1	DCUN1D1	0.4439
BMLF1	LPAR1	0.13014
BMLF1	SDCBP	0.012217
BMLF1	MOB3B	0.43446
BMLF1	SLC15A4	0.024963

BMLF1	MSH6	0.10807
BMLF1	SNRNP200	0.25702
BMLF1	PTCH1	0.065284
BMLF1	EIF2B1	0.20871
BMLF1	PDE3B	0.15283
BMLF1	DRD3	0.080241
BMLF1	CTNNA3	0.26248
BMLF1	TMPRSS11F	0.044876
BMLF1	IFITM1	0.25199
BMLF1	FOXH1	0.042287
BMLF1	TMEM184C	0.028205
BMLF1	CLIP3	0.025523
BMLF1	GZMH	0.032093
BMLF1	SWSAP1	0.37391
BMLF1	DDX17	0.24208
BMLF1	RECQL	0.23103
BMLF1	BCS1L	0.30244
BMLF1	SH3PXD2A	0.36757
BMLF1	SFPQ	0.30157
BMLF1	SYK	0.033578
BMLF1	PARPBP	0.37264
BMLF1	FBXL15	0.36221
BMLF1	FANCA	0.33793
BMLF1	FIP1L1	0.037937
BMLF1	EIF2B3	0.29607
BMLF1	ANGPT1	0.017261
BMLF1	TMEM11	0.36353
BMLF1	KDM1A	0.051573
BMLF1	HABP2	0.044653
BMLF1	PCDHAC2	0.21127
BMLF1	CLSTN2	0.15104
BMLF1	RBMS1	0.38451
BMLF1	ATP6V0D1	0.21206
BMLF1	TPD52L1	0.32512
BMLF1	ASPHD1	0.027277
BMLF1	WDFY3	0.35558
BMLF1	PSMA4	0.11444
BMLF1	PRSS56	0.050518
BMLF1	HSP90B1	0.32046
BMLF1	NR2C1	0.15811
BMLF1	PCDH7	0.21791
BMLF1	PIAS1	0.17054
BMLF1	HOXB7	0.1862
BMLF1	FRG1	0.018642
BMLF1	TNFSF13B	0.21114
BMLF1	CDK6	0.053521
BMLF1	MPP5	0.3251
BMLF1	ZBP1	0.055478
BMLF1	TRIM2	0.32162
BMLF1	NMT2	0.012272
BMLF1	DDAH2	0.29902
BMLF1	ATPAF2	0.4304
BMLF1	DNAJC3	0.019264
BMLF1	MACF1	0.37414
BMLF1	AIFM1	0.20588
BMLF1	CKAP5	0.24799
BMLF1	TNFRSF12A	0.27871
BMLF1	POLR3F	0.25015
BMLF1	PRSS22	0.049094
BMLF1	S100A3	0.23305
BMLF1	MEIS1	0.16036
BMLF1	WWOX	0.25334
BMLF1	ADORA2A	0.048412
BMLF1	SOS1	0.15799
BMLF1	APOD	0.16399
BMLF1	TXNL4A	0.34237
BMLF1	EVL	0.28463
BMLF1	BOK	0.26787

BMLF1	ALG2	0.24822
BMLF1	GLMN	0.37148
BMLF1	MAGEC2	0.38112
BMLF1	UVSSA	0.39496
BMLF1	SLC22A16	0.012539
BMLF1	PCM1	0.21736
BMLF1	PNLIPRP1	0.11772
BMLF1	TPSAB1	0.38884
BMLF1	TXNRD2	0.37732
BMLF1	FGF21	0.35618
BMLF1	PCGF3	0.30207
BMLF1	MSR1	0.33556
BMLF1	DUOX2	0.19631
BMLF1	RTN1	0.35838
BMLF1	RBM14	0.20662
BMLF1	OSBPL6	0.3426
BMLF1	CCDC176	0.10876
BMLF1	STBD1	0.3919
BMLF1	CASQ2	0.16411
BMLF1	MCTP2	0.2338
BMLF1	PRKG1	0.17213
BMLF1	GSTO1	0.067007
BMLF1	TRPC4AP	0.41559
BMLF1	LPXN	0.20787
BMLF1	PUS7L	0.12827
BMLF1	UBE2A	0.22021
BMLF1	HMBS	0.02167
BMLF1	EPHA7	0.16502
BMLF1	SNTB2	0.33088
BMLF1	CX3CL1	0.12678
BMLF1	PCDHGB4	0.21802
BMLF1	CST7	0.030652
BMLF1	AP5Z1	0.32252
BMLF1	PPIE	0.33106
BMLF1	KLRK1	0.23877
BMLF1	CAST	0.44113
BMLF1	TBC1D25	0.40051
BMLF1	DNAJC7	0.36912
BMLF1	PLOD2	0.010513
BMLF1	GCH1	0.18807
BMLF1	PTGES3	0.22828
BMLF1	ANKRD1	0.15537
BMLF1	VPS11	0.32982
BMLF1	POLE3	0.31927
BMLF1	SLC9A3	0.25006
BMLF1	OGN	0.30602
BMLF1	HNRNPU	0.26242
BMLF1	OPRD1	0.20945
BMLF1	MDM2	0.085445
BMLF1	NKD2	0.43755
BMLF1	RDH5	0.023908
BMLF1	ACBD4	0.020988
BMLF1	LSAMP	0.4019
BMLF1	TRAPPC1	0.051605
BMLF1	KRTAP13-4	0.048423
BMLF1	PTPN23	0.18896
BMLF1	ADAMTSL2	0.32184
BMLF1	FLNC	0.34622
BMLF1	CASD1	0.07232
BMLF1	MAGI3	0.26238
BMLF1	SLAMF1	0.27909
BMLF1	IFT57	0.30094
BMLF1	ADAM23	0.26354
BMLF1	AP5B1	0.39651
BMLF1	TRIM29	0.23293
BMLF1	EIF2B5	0.26045
BMLF1	DENR	0.45899
BMLF1	ZNF350	0.27244

36721	BMLF1	SV2A	0.07584
36722	BMLF1	TMEM132D	0.40455
36723	BMLF1	NUBP2	0.081687
36724	BMLF1	MAPK8IP1	0.13639
36725	BMLF1	SCAF1	0.019954
36726	BMLF1	FNDC3A	0.017662
36727	BMLF1	SLC4A5	0.013374
36728	BMLF1	CHKA	0.05735
36729	BMLF1	FOXN3	0.11962
36730	BMLF1	PTPN4	0.19143
36731	BMLF1	ATP2B2	0.094755
36732	BMLF1	BTG2	0.21057
36733	BMLF1	PEX3	0.27465
36734	BMLF1	GHITM	0.38033
36735	BMLF1	INCENP	0.21629
36736	BMLF1	BPIFB1	0.017645
36737	BMLF1	GPX6	0.097277
36738	BMLF1	TBC1D9	0.19859
36739	BMLF1	CDK1	0.023481
36740	BMLF1	C19orf71	0.029047
36741	BMLF1	DOT1L	0.34607
36742	BMLF1	UCK2	0.086389
36743	BMLF1	JMJD1C	0.34106
36744	BMLF1	GJD3	0.35982
36745	BMLF1	FNBP1L	0.36335
36746	BMLF1	ITGAV	0.11232
36747	BMLF1	TXLNB	0.44386
36748	BMLF1	SEC13	0.2145
36749	BMLF1	SNX29P2	0.020635
36750	BMLF1	CDH10	0.2003
36751	BMLF1	CDKN2AIP	0.33203
36752	BMLF1	KEAP1	0.27642
36753	BMLF1	EPHA10	0.28784
36754	BMLF1	CNOT6	0.25452
36755	BMLF1	LCK	0.064819
36756	BMLF1	HIPK2	0.058196
36757	BMLF1	PSMC4	0.13845
36758	BMLF1	RAD50	0.12701
36759	BMLF1	FAT1	0.39294
36760	BMLF1	REV1	0.24601
36761	BMLF1	BAZ1A	0.24138
36762	BMLF1	OCRL	0.18844
36763	BMLF1	DYRK1B	0.21778
36764	BMLF1	PRKD2	0.037979
36765	BMLF1	ABCF1	0.27002
36766	BMLF1	HDAC3	0.071863
36767	BMLF1	FBLN7	0.22993
36768	BMLF1	NXF3	0.35707
36769	BMLF1	H2AFY	0.27634
36770	BMLF1	FOXK1	0.087459
36771	BMLF1	CCNH	0.070686
36772	BMLF1	FCN1	0.10562
36773	BMLF1	NGLY1	0.36894
36774	BMLF1	IRF1	0.040917
36775	BMLF1	EDAR	0.27431
36776	BMLF1	LMOD1	0.01387
36777	BMLF1	MTCH1	0.31998
36778	BMLF1	ERCC8	0.26376
36779	BMLF1	CRELD1	0.23596
36780	BMLF1	ISOC2	0.33203
36781	BMLF1	TGOLN2	0.34535
36782	BMLF1	SLC6A6	0.023473
36783	BMLF1	HABP4	0.27326
36784	BMLF1	XAB2	0.29683
36785	BMLF1	S100A11	0.5377
36786	BMLF1	ITGA5	0.16676
36787	BMLF1	GDF7	0.22762
36788	BMLF1	GPS2	0.35862

BMLF1	SRSF5	0.29098
BMLF1	USH1G	0.30198
BMLF1	SSR3	0.035295
BMLF1	SLC12A4	0.043649
BMLF1	ABRA	0.032592
BMLF1	NYX	0.013743
BMLF1	SORBS1	0.14179
BMLF1	CHMP1B	0.31949
BMLF1	ADORA3	0.030402
BMLF1	HFE2	0.31961
BMLF1	IRF3	0.06437
BMLF1	AKR1B15	0.019723
BMLF1	EFCAB6	0.19286
BMLF1	CCDC137	0.076865
BMLF1	DUS2	0.034331
BMLF1	GPR179	0.086481
BMLF1	RBBP6	0.25589
BMLF1	MAP3K3	0.22325
BMLF1	ESR2	0.08899
BMLF1	TPP1	0.2214
BMLF1	NCAPG	0.33234
BMLF1	NANOS2	0.012612
BMLF1	NUFIP2	0.45313
BMLF1	ECSIT	0.3935
BMLF1	LRRFIP2	0.4198
BMLF1	AIRE	0.14978
BMLF1	XPO4	0.36087
BMLF1	INIP	0.39591
BMLF1	NKD1	0.4952
BMLF1	BCL7C	0.080543
BMLF1	NR1H2	0.088204
BMLF1	PPP1R9B	0.18177
BMLF1	PLEKHM2	0.32395
BMLF1	CBFA2T3	0.12265
BMLF1	DPM3	0.24942
BMLF1	CYBB	0.1735
BMLF1	DENND1B	0.061394
BMLF1	CEP70	0.3222
BMLF1	SLC16A6	0.028609
BMLF1	DERL2	0.30114
BMLF1	ACACA	0.20195
BMLF1	SPTBN4	0.10812
BMLF1	STAP1	0.19832
BMLF1	EDA2R	0.29269
BMLF1	PPP6C	0.30203
BMLF1	TRIAP1	0.35808
BMLF1	FASTK	0.32409
BMLF1	RBFOX2	0.26759
BMLF1	GCK	0.097322
BMLF1	RPS10	0.22703
BMLF1	CD2AP	0.30593
BMLF1	RAP2A	0.2322
BMLF1	CD1A	0.35723
BMLF1	MPO	0.010765
BMLF1	CBX8	0.27166
BMLF1	BCDIN3D	0.1338
BMLF1	ZNF830	0.04134
BMLF1	PAGR1	0.41254
BMLF1	RNF146	0.25101
BMLF1	CROCCP3	0.013573
BMLF1	NXT2	0.016179
BMLF1	IGHG1	0.33525
BMLF1	KIF18A	0.15713
BMLF1	GORAB	0.39241
BMLF1	GABPB1	0.18699
BMLF1	GREM1	0.072203
BMLF1	FAM151B	0.011471
BMLF1	INTS12	0.34659

BMLF1	UBE2N	0.11977
BMLF1	TAF7	0.093709
BMLF1	NSUN7	0.077134
BMLF1	MAGEB18	0.47224
BMLF1	SNX11	0.019801
BMLF1	C2orf40	0.060064
BMLF1	MED31	0.2807
BMLF1	MAT2B	0.24501
BMLF1	CISD2	0.03038
BMLF1	GEMIN4	0.28665
BMLF1	HMCN1	0.25023
BMLF1	SLC9A3R2	0.23401
BMLF1	KNSTRN	0.24834
BMLF1	PRSS21	0.043385
BMLF1	ODF2	0.19605
BMLF1	SERPINE1	0.075714
BMLF1	PCBP2	0.26803
BMLF1	METTL4	0.056386
BMLF1	SPC25	0.27938
BMLF1	RBM25	0.32464
BMLF1	DACH1	0.12971
BMLF1	PKP1	0.3132
BMLF1	DNAJB5	0.38042
BMLF1	EIF1AY	0.53012
BMLF1	PLA2G2E	0.11501
BMLF1	FNIP2	0.35226
BMLF1	DEAF1	0.21564
BMLF1	ARID4B	0.3005
BMLF1	EMR2	0.13929
BMLF1	TREML1	0.3623
BMLF1	MOV10	0.29756
BMLF1	MAGI1	0.29886
BMLF1	CST2	0.034289
BMLF1	PTGIS	0.10938
BMLF1	ARL4A	0.32598
BMLF1	ADK	0.045401
BMLF1	BPTF	0.18739
BMLF1	WRN	0.045699
BMLF1	USF1	0.076983
BMLF1	STMN2	0.28922
BMLF1	TAAR1	0.075294
BMLF1	NPIPA1	0.032041
BMLF1	CAPS2	0.26298
BMLF1	FAM47B	0.040384
BMLF1	ARHGAP32	0.18017
BMLF1	MTDH	0.18037
BMLF1	DNA2	0.035277
BMLF1	RP9	0.33631
BMLF1	PRRC2A	0.41608
BMLF1	MKKS	0.12016
BMLF1	GMNN	0.26946
BMLF1	PDZD11	0.41534
BMLF1	METTL17	0.40194
BMLF1	MYLPF	0.22011
BMLF1	PSMD10	0.097051
BMLF1	RDM1	0.027719
BMLF1	SDHAF2	0.40583
BMLF1	ST13	0.2805
BMLF1	APOA1	0.051606
BMLF1	PSMA5	0.12202
BMLF1	HHEX	0.032948
BMLF1	PPP2R3B	0.5067
BMLF1	PEG10	0.3061
BMLF1	INO80	0.17993
BMLF1	CTNNBIP1	0.24806
BMLF1	CENPH	0.22894
BMLF1	HDAC9	0.094254
BMLF1	HEY1	0.079389

BMLF1	ACTN4	0.2794
BMLF1	ARHGAP30	0.054603
BMLF1	FBXO46	0.47224
BMLF1	CDK9	0.062532
BMLF1	KIAA0141	0.4304
BMLF1	GJA3	0.012769
BMLF1	DTX3L	0.29634
BMLF1	EHD1	0.33207
BMLF1	ATL1	0.20013
BMLF1	PRSS57	0.049094
BMLF1	MYH11	0.014975
BMLF1	KIR2DL1	0.3691
BMLF1	RAB25	0.35436
BMLF1	CXXC4	0.018033
BMLF1	TIMM44	0.27305
BMLF1	GPRC5C	0.11016
BMLF1	PARD6B	0.25216
BMLF1	GTF2E1	0.24587
BMLF1	AP3S2	0.10903
BMLF1	RAN	0.14626
BMLF1	KIAA0408	0.47224
BMLF1	LILRA4	0.44386
BMLF1	EEA1	0.26509
BMLF1	SUN2	0.22003
BMLF1	SEPT8	0.38931
BMLF1	PINK1	0.10073
BMLF1	PREPL	0.088768
BMLF1	FANCG	0.28987
BMLF1	CTU2	0.39268
BMLF1	PRSS42	0.049094
BMLF1	OXA1L	0.2652
BMLF1	INTS10	0.38021
BMLF1	AP2S1	0.010825
BMLF1	LPL	0.11865
BMLF1	PKMYT1	0.13886
BMLF1	ERCC6	0.11563
BMLF1	PARVA	0.19446
BMLF1	VCAN	0.13137
BMLF1	CDR2	0.4034
BMLF1	KRIT1	0.28188
BMLF1	SLC12A1	0.03921
BMLF1	RABAC1	0.36287
BMLF1	LRAT	0.038413
BMLF1	RUNX2	0.07298
BMLF1	RHD	0.032086
BMLF1	PLLP	0.015589
BMLF1	AMFR	0.15369
BMLF1	GRSF1	0.040784
BMLF1	MAP3K13	0.107
BMLF1	NEU4	0.24366
BMLF1	RBM19	0.010038
BMLF1	CNOT6L	0.27653
BMLF1	RGL4	0.064395
BMLF1	RPS19BP1	0.39241
BMLF1	COMMD1	0.28013
BMLF1	SEPT2	0.15143
BMLF1	RBM4	0.16352
BMLF1	KPNA3	0.24624
BMLF1	ZNRF1	0.32265
BMLF1	GRB7	0.1761
BMLF1	PLCB1	0.050074
BMLF1	ZSCAN21	0.18823
BMLF1	TBP	0.076788
BMLF1	TESK1	0.26269
BMLF1	CDC42BPG	0.14251
BMLF1	PRMT10	0.11408
BMLF1	CDC25A	0.087223
BMLF1	DNAJC13	0.016778

BMLF1	CABP4	0.19317
BMLF1	GEMIN6	0.30328
BMLF1	MAPK12	0.12298
BMLF1	MMP2	0.14229
BMLF1	SLC1A5	0.062328
BMLF1	INHA	0.15372
BMLF1	ABCA4	0.030559
BMLF1	CSN2	0.23182
BMLF1	POLE4	0.32917
BMLF1	BAD	0.056855
BMLF1	EIF2B2	0.26576
BMLF1	RPTN	0.26909
BMLF1	EXOSC1	0.33243
BMLF1	POLA1	0.024052
BMLF1	TPSD1	0.049094
BMLF1	DSP	0.1627
BMLF1	RGS19	0.2399
BMLF1	ZFYVE20	0.23864
BMLF1	UBE2G2	0.27607
BMLF1	RMI1	0.41616
BMLF1	AIP	0.17514
BMLF1	S100A13	0.37718
BMLF1	PIK3R6	0.2402
BMLF1	STUB1	0.15708
BMLF1	MEA1	0.4047
BMLF1	MELK	0.33601
BMLF1	EXO1	0.17521
BMLF1	FAM69B	0.12906
BMLF1	GEM	0.25815
BMLF1	NEK1	0.1891
BMLF1	HPGDS	0.17924
BMLF1	MED18	0.29791
BMLF1	BNIP3	0.086849
BMLF1	INHBB	0.24517
BMLF1	HSP90AB1	0.1802
BMLF1	CORO1B	0.39069
BMLF1	HTRA3	0.40694
BMLF1	PSMB1	0.12158
BMLF1	S100A1	0.47526
BMLF1	ARL14EPL	0.058656
BMLF1	ZDHHC13	0.055262
BMLF1	EIF3H	0.4086
BMLF1	TMED9	0.2759
BMLF1	GPATCH8	0.35123
BMLF1	PRMT6	0.22198
BMLF1	MYL7	0.23733
BMLF1	SNRPC	0.33449
BMLF1	SUMO1	0.16193
BMLF1	PPIA	0.2354
BMLF1	PRSS41	0.043369
BMLF1	SUMO2	0.39136
BMLF1	USP1	0.27957
BMLF1	ARHGAP24	0.21484
BMLF1	PRSS46	0.050518
BMLF1	UTRN	0.38318
BMLF1	TRAF3IP3	0.44386
BMLF1	STAM2	0.23874
BMLF1	EGR2	0.1146
BMLF1	UBE2D2	0.25347
BMLF1	EGFL8	0.25234
BMLF1	GPX4	0.086363
BMLF1	RAD23B	0.25743
BMLF1	SLC5A8	0.047973
BMLF1	HHIP	0.16183
BMLF1	GNS	0.16643
BMLF1	PCGF5	0.29328
BMLF1	S100A2	0.24893
BMLF1	CUZD1	0.061724

BMLF1	ATP6V1E2	0.039393
BMLF1	KCNH5	0.013911
BMLF1	RPL15	0.30654
BMLF1	PALM	0.20145
BMLF1	KLK15	0.049094
BMLF1	IDE	0.066033
BMLF1	AP2A2	0.22834
BMLF1	ASH2L	0.23373
BMLF1	DOHH	0.2869
BMLF1	IGF1	0.035633
BMLF1	SLC34A1	0.29852
BMLF1	METTL21A	0.056371
BMLF1	SCUBE2	0.25502
BMLF1	TBC1D2B	0.26892
BMLF1	MCM7	0.19046
BMLF1	TOP2A	0.09981
BMLF1	SGOL2	0.27244
BMLF1	PIK3CA	0.13324
BMLF1	MYL9	0.18526
BMLF1	CD1C	0.049493
BMLF1	NOP56	0.36759
BMLF1	KIAA0101	0.34773
BMLF1	TNFRSF1B	0.28718
BMLF1	BAHD1	0.28723
BMLF1	E4F1	0.096095
BMLF1	SPRY2	0.10111
BMLF1	ENG	0.079179
BMLF1	MYO18A	0.3058
BMLF1	RARA	0.020802
BMLF1	RAB37	0.065335
BMLF1	HAND1	0.056077
BMLF1	ACP1	0.17261
BMLF1	AQP1	0.069789
BMLF1	MAPKAPK3	0.12782
BMLF1	ELN	0.30559
BMLF1	SWI5	0.41185
BMLF1	LTF	0.19023
BMLF1	PPM1B	0.25804
BMLF1	TBC1D10A	0.34185
BMLF1	EFHD1	0.23958
BMLF1	ASMT	0.055338
BMLF1	MAP2K3	0.11721
BMLF1	RPL18A	0.043889
BMLF1	OGDH	0.010687
BMLF1	SMC2	0.25415
BMLF1	ZC3HAV1	0.27376
BMLF1	SAMM50	0.28623
BMLF1	FASLG	0.11146
BMLF1	VWF	0.21765
BMLF1	TMBIM4	0.33472
BMLF1	FAXDC2	0.017538
BMLF1	SAP30BP	0.31667
BMLF1	ZFPM2	0.13461
BMLF1	EEF2K	0.11462
BMLF1	CIB4	0.26298
BMLF1	DGKE	0.021449
BMLF1	TSG101	0.12939
BMLF1	CRADD	0.27068
BMLF1	SPTBN1	0.16343
BMLF1	EDEM2	0.15995
BMLF1	KANK1	0.2167
BMLF1	NUP37	0.19672
BMLF1	CHGA	0.093778
BMLF1	FLII	0.28305
BMLF1	EMD	0.23125
BMLF1	C17orf66	0.011473
BMLF1	ECHS1	0.25462
BMLF1	NTRK1	0.096943

BMLF1	RBM17	0.40394
BMLF1	THBS3	0.19168
BMLF1	CUBN	0.072366
BMLF1	PSPH	0.12839
BMLF1	S100A14	0.17131
BMLF1	STAT6	0.26748
BMLF1	MPZL2	0.35798
BMLF1	INVS	0.28024
BMLF1	DTNB	0.19325
BMLF1	REST	0.036579
BMLF1	USP4	0.27108
BMLF1	RBPJ	0.071608
BMLF1	ATG3	0.25972
BMLF1	PRRG3	0.23259
BMLF1	ULK1	0.1138
BMLF1	LLGL1	0.13802
BMLF1	SPOCK2	0.22013
BMLF1	HYAL2	0.073378
BMLF1	PIH1D2	0.013295
BMLF1	TLR1	0.037803
BMLF1	PRSS29P	0.049094
BMLF1	IWS1	0.05378
BMLF1	STAMBP	0.22453
BMLF1	CTLA4	0.32703
BMLF1	TMPRSS11B	0.044876
BMLF1	SLIT3	0.12513
BMLF1	ALDH3B2	0.051586
BMLF1	ARPC5	0.3157
BMLF1	NELFE	0.28267
BMLF1	CLEC1B	0.38085
BMLF1	FOXP3	0.01518
BMLF1	ATRAID	0.26919
BMLF1	FBXO4	0.32617
BMLF1	NXF2	0.031023
BMLF1	CORIN	0.028055
BMLF1	HNRNPL	0.30263
BMLF1	TLE2	0.28232
BMLF1	POM121L12	0.013016
BMLF1	PPP2R2A	0.30238
BMLF1	AKAP5	0.078434
BMLF1	CALB1	0.41909
BMLF1	PVRIG	0.034023
BMLF1	DAZAP2	0.4034
BMLF1	IL18R1	0.032129
BMLF1	CPSF2	0.33681
BMLF1	ATP13A1	0.011979
BMLF1	MAP4K1	0.24604
BMLF1	SLC38A9	0.018197
BMLF1	SLC13A2	0.046422
BMLF1	PCGF6	0.18607
BMLF1	PEX5	0.15041
BMLF1	RYK	0.21505
BMLF1	RASSF6	0.43005
BMLF1	NDEL1	0.12942
BMLF1	HHLA3	0.47224
BMLF1	NPHS2	0.35125
BMLF1	DIP2A	0.28884
BMLF1	GAN	0.38364
BMLF1	PDIA4	0.24433
BMLF1	MIER1	0.2722
BMLF1	MAP3K11	0.14651
BMLF1	BAZ2A	0.21312
BMLF1	AK9	0.02816
BMLF1	CAPN11	0.21524
BMLF1	SFTPC	0.25952
BMLF1	UFD1L	0.35691
BMLF1	RBM7	0.038448
BMLF1	SPINK5	0.017536

BMLF1	LIN7A	0.25381
BMLF1	SETMAR	0.23335
BMLF1	VAV3	0.11633
BMLF1	MASP2	0.54007
BMLF1	EPN3	0.076446
BMLF1	BATF2	0.26367
BMLF1	NUDT21	0.30181
BMLF1	SLC2A7	0.034805
BMLF1	PCDHA9	0.22489
BMLF1	PARK7	0.20851
BMLF1	RP2	0.20487
BMLF1	FKBP1A	0.17654
BMLF1	TAF6L	0.2065
BMLF1	SLC25A23	0.21019
BMLF1	UNC93B1	0.016421
BMLF1	TRAF7	0.25262
BMLF1	BCL2L10	0.30211
BMLF1	RCN2	0.24685
BMLF1	PRCP	0.39646
BMLF1	FXR1	0.033466
BMLF1	SUCLA2	0.21736
BMLF1	TAOK3	0.22797
BMLF1	SSTR2	0.18877
BMLF1	PPP4C	0.23981
BMLF1	HSPA1A	0.15
BMLF1	AP3B2	0.039896
BMLF1	TAP1	0.12257
BMLF1	ATG9A	0.35039
BMLF1	CALML4	0.26298
BMLF1	NDUFAF1	0.38778
BMLF1	COG5	0.26264
BMLF1	MAP3K5	0.095166
BMLF1	FICD	0.019155
BMLF1	FRA10AC1	0.42917
BMLF1	PES1	0.25373
BMLF1	CCM2	0.39299
BMLF1	BBIP1	0.40156
BMLF1	SHROOM3	0.014514
BMLF1	TRIM39	0.26185
BMLF1	SPATA17	0.011871
BMLF1	ABI1	0.1817
BMLF1	PTBP1	0.33328
BMLF1	SOST	0.24693
BMLF1	MAGEA3	0.47224
BMLF1	ZNF224	0.26539
BMLF1	RAMP2	0.1347
BMLF1	PKIG	0.017181
BMLF1	TAF13	0.15966
BMLF1	KIAA1958	0.013568
BMLF1	SLAIN1	0.038371
BMLF1	SAA2	0.029317
BMLF1	CAPSL	0.25501
BMLF1	RRAS2	0.26751
BMLF1	RAB4A	0.207
BMLF1	FGF19	0.33963
BMLF1	DDN	0.32679
BMLF1	RAB5B	0.26347
BMLF1	RNF32	0.36096
BMLF1	ARL14	0.46827
BMLF1	LRWD1	0.26414
BMLF1	TXNDC9	0.43141
BMLF1	ARL6	0.17202
BMLF1	EFNB3	0.037149
BMLF1	EIF3C	0.42145
BMLF1	EIF3M	0.45718
BMLF1	ACOXL	0.043025
BMLF1	ZNF326	0.31867
BMLF1	STEAP1	0.017627

BMLF1	OXTR	0.015702
BMLF1	MYOZ3	0.45039
BMLF1	YBL100W-C	0.021197
BMLF1	TXNDC12	0.015382
BMLF1	KLHL2	0.41504
BMLF1	DTX2	0.28437
BMLF1	COMMD10	0.47224
BMLF1	NOM1	0.417
BMLF1	CSTL1	0.034289
BMLF1	ACVR2A	0.069916
BMLF1	GRK5	0.13415
BMLF1	FANCC	0.29363
BMLF1	RFX3	0.13714
BMLF1	ITGB1BP2	0.18623
BMLF1	CENPA	0.1653
BMLF1	MDFIC	0.19688
BMLF1	UBXN6	0.34809
BMLF1	SYNPO2	0.37527
BMLF1	BABAM1	0.33345
BMLF1	IL5RA	0.35995
BMLF1	AIF1L	0.18
BMLF1	PON1	0.068395
BMLF1	LPCAT4	0.14472
BMLF1	CELA2B	0.049094
BMLF1	CDH11	0.18915
BMLF1	RABL6	0.35391
BMLF1	APEX1	0.11543
BMLF1	ART3	0.058135
BMLF1	ELK3	0.15355
BMLF1	CBLL1	0.29303
BMLF1	PCDHAC1	0.21127
BMLF1	EPPIN	0.39719
BMLF1	GAS6	0.049222
BMLF1	ATP13A3	0.011457
BMLF1	BET1	0.2623
BMLF1	ANXA2R	0.09211
BMLF1	SYT6	0.03531
BMLF1	AMPH	0.31682
BMLF1	ABLIM1	0.30511
BMLF1	RANGAP1	0.19413
BMLF1	CIRBP	0.28982
BMLF1	FGF23	0.012299
BMLF1	LINC00116	0.045912
BMLF1	MYBPC3	0.028652
BMLF1	ERLEC1	0.41675
BMLF1	MEF2C	0.014264
BMLF1	LATS1	0.099621
BMLF1	KIFAP3	0.27718
BMLF1	MED9	0.29791
BMLF1	ABCB1	0.22218
BMLF1	PCDHB6	0.19264
BMLF1	EFEMP1	0.50641
BMLF1	EIF4A3	0.20127
BMLF1	LPAR2	0.32251
BMLF1	ZAP70	0.14677
BMLF1	DAXX	0.1275
BMLF1	EIF3CL	0.032002
BMLF1	PPRC1	0.014918
BMLF1	IL1A	0.14951
BMLF1	BID	0.15173
BMLF1	IFT74	0.028386
BMLF1	LY6E	0.016701
BMLF1	COMMD2	0.47224
BMLF1	KANSL1	0.35457
BMLF1	SDPR	0.30938
BMLF1	AGRN	0.1349
BMLF1	MKL1	0.26657
BMLF1	ZNF281	0.1325

BMLF1	TTC37	0.39112
BMLF1	PNMA1	0.34873
BMLF1	APC2	0.28045
BMLF1	CCDC53	0.44693
BMLF1	RBP7	0.041125
BMLF1	NIPSNAP3A	0.44435
BMLF1	SPATA13	0.17372
BMLF1	MYO9B	0.22603
BMLF1	MAGOHB	0.047526
BMLF1	GLT8D1	0.014742
BMLF1	SLC6A17	0.075466
BMLF1	C1D	0.27554
BMLF1	BAP1	0.20229
BMLF1	CIR1	0.18653
BMLF1	ULK3	0.23146
BMLF1	TBCB	0.35814
BMLF1	SPARC	0.2645
BMLF1	GTF2H1	0.1335
BMLF1	NRGN	0.011282
BMLF1	CREB5	0.17556
BMLF1	APLNR	0.018889
BMLF1	TCHHL1	0.26298
BMLF1	RBM28	0.012762
BMLF1	EWSR1	0.30984
BMLF1	F2RL1	0.05894
BMLF1	OC90	0.2088
BMLF1	EHMT2	0.24192
BMLF1	PALLD	0.31185
BMLF1	SRSF11	0.35032
BMLF1	PCSK7	0.080789
BMLF1	SMAGP	0.11239
BMLF1	NOTCH4	0.21018
BMLF1	RAPGEF3	0.22549
BMLF1	PIK3C3	0.19856
BMLF1	TERT	0.20931
BMLF1	NUTF2	0.35602
BMLF1	C2	0.043666
BMLF1	ARPC4	0.33254
BMLF1	EXOSC10	0.18276
BMLF1	ZMAT3	0.017571
BMLF1	FOXO1	0.039297
BMLF1	PCDHB1	0.22489
BMLF1	MEAF6	0.27979
BMLF1	ANG	0.071798
BMLF1	KLHL12	0.39577
BMLF1	TSPYL5	0.32307
BMLF1	TPH2	0.05204
BMLF1	PIM1	0.13705
BMLF1	DLX4	0.24357
BMLF1	ADARB1	0.058885
BMLF1	MBD2	0.20981
BMLF1	MST4	0.15533
BMLF1	MICAL2	0.029328
BMLF1	TRAIP	0.30834
BMLF1	DFNB31	0.23742
BMLF1	ATP5G3	0.063315
BMLF1	CYTH2	0.26083
BMLF1	CTR9	0.21015
BMLF1	PRSS58	0.049094
BMLF1	NAA15	0.24735
BMLF1	MATN1	0.42093
BMLF1	NOTCH2NL	0.22552
BMLF1	CA4	0.1726
BMLF1	SLC13A1	0.044838
BMLF1	DEPDC1	0.28754
BMLF1	ID4	0.22828
BMLF1	KIAA1279	0.39845
BMLF1	PCDH17	0.63413

BMLF1	GLI2	0.024934
BMLF1	CSNK2A2	0.17063
BMLF1	PIR	0.37196
BMLF1	IGSF8	0.36705
BMLF1	EPHA4	0.1104
BMLF1	VTI1A	0.018924
BMLF1	CSRNP2	0.20659
BMLF1	SET	0.1826
BMLF1	MCL1	0.20842
BMLF1	PRICKLE1	0.24403
BMLF1	FGFBP1	0.30398
BMLF1	GPR37L1	0.35373
BMLF1	ARHGEF2	0.084458
BMLF1	SLC9A1	0.14299
BMLF1	KITLG	0.19271
BMLF1	MARCH11	0.085925
BMLF1	DSC3	0.18338
BMLF1	SLC22A3	0.23114
BMLF1	B2M	0.20374
BMLF1	GLRB	0.17473
BMLF1	KCNN4	0.24635
BMLF1	PRAMEF13	0.096914
BMLF1	PHB	0.17122
BMLF1	HNRNPDL	0.011605
BMLF1	PRDX4	0.42526
BMLF1	CCDC85B	0.2736
BMLF1	NR5A1	0.072238
BMLF1	APC	0.024347
BMLF1	PMS2	0.23032
BMLF1	CLSTN1	0.10527
BMLF1	SRRM1	0.065856
BMLF1	XK	0.4073
BMLF1	SCAF11	0.31729
BMLF1	EXOC3	0.39685
BMLF1	EIF3B	0.4078
BMLF1	CLGN	0.17183
BMLF1	CIB2	0.26298
BMLF1	LDB3	0.34024
BMLF1	PPM1D	0.21711
BMLF1	PLK1	0.036779
BMLF1	PRSS44	0.049094
BMLF1	KHDRBS3	0.33706
BMLF1	SLC17A8	0.049567
BMLF1	ERBB4	0.048045
BMLF1	EXD3	0.47224
BMLF1	ADH1A	0.30289
BMLF1	SPEN	0.17411
BMLF1	CEP72	0.33178
BMLF1	ALB	0.119
BMLF1	GGT1	0.26252
BMLF1	YJR030C	0.041867
BMLF1	NAP1L1	0.33654
BMLF1	SRSF1	0.27949
BMLF1	KIAA1841	0.050068
BMLF1	EAF2	0.28139
BMLF1	CASK	0.15178
BMLF1	SOX9	0.010738
BMLF1	ESRRG	0.17424
BMLF1	C1orf196	0.022142
BMLF1	BPIFB6	0.017788
BMLF1	TARDBP	0.20054
BMLF1	RASSF4	0.44362
BMLF1	REPS1	0.63958
BMLF1	SLC15A1	0.037225
BMLF1	IL15RA	0.34058
BMLF1	PLEK	0.13914
BMLF1	CIRH1A	0.32991
BMLF1	TYRO3	0.1463

BMLF1	RUFY1	0.30809
BMLF1	PEX2	0.23709
BMLF1	SLC18A2	0.055784
BMLF1	TNFRSF10A	0.28389
BMLF1	POP5	0.3215
BMLF1	NSD1	0.057437
BMLF1	GRIP1	0.060676
BMLF1	TRPS1	0.16046
BMLF1	LRRC26	0.32997
BMLF1	BBS5	0.29101
BMLF1	PRODH	0.059482
BMLF1	CHRNG	0.015722
BMLF1	SIVA1	0.23272
BMLF1	MFSD6	0.085714
BMLF1	NLGN3	0.1577
BMLF1	GCC1	0.35551
BMLF1	AURKAIP1	0.34013
BMLF1	CYTIP	0.42139
BMLF1	PARP8	0.062907
BMLF1	USP14	0.24329
BMLF1	PCDHGA9	0.22489
BMLF1	ELOVL3	0.28786
BMLF1	EVI5	0.27399
BMLF1	RASA1	0.1349
BMLF1	SEC23IP	0.02633
BMLF1	PALB2	0.32761
BMLF1	CAND2	0.32878
BMLF1	FABP3	0.32241
BMLF1	THUMPD3	0.12189
BMLF1	LRCOL1	0.094603
BMLF1	CDHR5	0.19803
BMLF1	AHI1	0.13702
BMLF1	MRPS27	0.38436
BMLF1	ZBTB16	0.1202
BMLF1	SAT1	0.33756
BMLF1	DTYMK	0.031061
BMLF1	STARD10	0.018586
BMLF1	DCTN2	0.23871
BMLF1	TBC1D8B	0.19859
BMLF1	TUBGCP3	0.18425
BMLF1	AGO1	0.25105
BMLF1	CD53	0.392
BMLF1	USP25	0.2957
BMLF1	AFF1	0.27067
BMLF1	SH3GL2	0.23995
BMLF1	TULP1	0.24711
BMLF1	NXF5	0.34711
BMLF1	SNX1	0.24795
BMLF1	AQP2	0.049263
BMLF1	CD151	0.34627
BMLF1	MDFI	0.24183
BMLF1	YTHDC1	0.38408
BMLF1	MRPL28	0.37763
BMLF1	KRT16	0.3366
BMLF1	GUCA1C	0.25416
BMLF1	MAP2K7	0.06768
BMLF1	METTL10	0.059751
BMLF1	SMAD1	0.035133
BMLF1	DOCK1	0.25833
BMLF1	CASP10	0.32507
BMLF1	MRPS9	0.33289
BMLF1	GEMIN7	0.30383
BMLF1	TBC1D7	0.38177
BMLF1	CDH7	0.2003
BMLF1	SPI1	0.090531
BMLF1	PPP1R1A	0.41115
BMLF1	SEC24C	0.14425
BMLF1	PRKCSH	0.23597

BMLF1	ZCCHC11	0.25011
BMLF1	LAMP5	0.099734
BMLF1	CFI	0.038022
BMLF1	TGFBR3	0.07721
BMLF1	TAAR6	0.043938
BMLF1	TGFBI	0.025654
BMLF1	ASUN	0.36609
BMLF1	AMY1A	0.28136
BMLF1	GRAP2	0.31714
BMLF1	EXOC7	0.31965
BMLF1	UPF1	0.20941
BMLF1	STX16	0.23918
BMLF1	TXNIP	0.17405
BMLF1	ANKRD28	0.41608
BMLF1	SAT2	0.054871
BMLF1	DRD4	0.099666
BMLF1	FGF1	0.14878
BMLF1	ALDH8A1	0.073375
BMLF1	ITPR3	0.071851
BMLF1	MMP1	0.089731
BMLF1	GAGE2D	0.45783
BMLF1	NEK11	0.2152
BMLF1	ARL13B	0.38917
BMLF1	ASAP1	0.14925
BMLF1	NOVA1	0.048327
BMLF1	ADAMTS10	0.24044
BMLF1	YLPM1	0.32883
BMLF1	BRAT1	0.41608
BMLF1	SIGIRR	0.33135
BMLF1	TAS2R16	0.30832
BMLF1	ADRA2C	0.19159
BMLF1	PCDHGA2	0.22489
BMLF1	RDX	0.28131
BMLF1	ATP5H	0.084946
BMLF1	TYR	0.25229
BMLF1	POU3F2	0.13015
BMLF1	ITGB4	0.21629
BMLF1	KAL1	0.29549
BMLF1	ZXDC	0.17001
BMLF1	SSR4	0.24788
BMLF1	DDIT3	0.1142
BMLF1	GTF3C6	0.37821
BMLF1	WIBG	0.40963
BMLF1	ARHGAP22	0.17745
BMLF1	PCDH1	0.19176
BMLF1	IFT140	0.027105
BMLF1	PLCL1	0.081806
BMLF1	SLCO1A2	0.024937
BMLF1	ATP13A5	0.015227
BMLF1	SLC40A1	0.22556
BMLF1	FBXW5	0.24362
BMLF1	PDE6H	0.04393
BMLF1	CFLAR	0.2864
BMLF1	EIF4EBP1	0.23766
BMLF1	MED29	0.33053
BMLF1	CDKN3	0.15675
BMLF1	TAX1BP1	0.33233
BMLF1	RPL18	0.04147
BMLF1	NOTCH2	0.14804
BMLF1	LOXL3	0.27924
BMLF1	ERLIN2	0.3481
BMLF1	EFCAB12	0.26298
BMLF1	CDH6	0.16845
BMLF1	FZR1	0.22668
BMLF1	ROR1	0.30121
BMLF1	MPTX1	0.026706
BMLF1	AMER2	0.41777
BMLF1	MITD1	0.32909

BMLF1	TAF1D	0.34933
BMLF1	SLC4A1AP	0.35764
BMLF1	SIAH2	0.1993
BMLF1	EXOSC3	0.2638
BMLF1	STIM1	0.33539
BMLF1	COG4	0.30853
BMLF1	S100B	0.34826
BMLF1	TRAF3IP1	0.44386
BMLF1	CCDC172	0.012591
BMLF1	YTHDF2	0.044825
BMLF1	ZAK	0.13738
BMLF1	FZD6	0.14785
BMLF1	EFHC1	0.17038
BMLF1	KAT6B	0.17134
BMLF1	HMG20B	0.20484
BMLF1	ESRRA	0.12706
BMLF1	SKP1	0.28609
BMLF1	VAMP1	0.36114
BMLF1	BIRC2	0.1266
BMLF1	ZNF212	0.25531
BMLF1	TNNC1	0.51391
BMLF1	NME4	0.0187
BMLF1	DDB1	0.23221
BMLF1	PDHA1	0.023188
BMLF1	PRPS1L1	0.010114
BMLF1	SAP30	0.29446
BMLF1	ZNF24	0.19342
BMLF1	SHCBP1	0.18249
BMLF1	CHD3	0.15251
BMLF1	ADCYAP1R1	0.11522
BMLF1	CRYGC	0.36128
BMLF1	COX10	0.031423
BMLF1	EFCAB4A	0.2398
BMLF1	ERBB2	0.035295
BMLF1	HTATIP2	0.24285
BMLF1	STEAP3	0.24493
BMLF1	TXLNA	0.38399
BMLF1	POLRMT	0.337
BMLF1	BCAS2	0.36995
BMLF1	TMED2	0.24781
BMLF1	C14orf80	0.018147
BMLF1	RAB3IL1	0.40397
BMLF1	CCNC	0.17587
BMLF1	C1orf94	0.47224
BMLF1	CASP6	0.15446
BMLF1	SLC5A7	0.025679
BMLF1	VCL	0.15049
BMLF1	PCDHB13	0.20505
BMLF1	CETN2	0.34984
BMLF1	CAMSAP2	0.4172
BMLF1	ANXA6	0.5842
BMLF1	PCDHGC4	0.22489
BMLF1	LCP1	0.14525
BMLF1	SLC16A14	0.031311
BMLF1	DIXDC1	0.17835
BMLF1	SIRPG	0.3134
BMLF1	GJE1	0.02044
BMLF1	PA2G4	0.17164
BMLF1	TINF2	0.24735
BMLF1	FBXO44	0.45041
BMLF1	ITM2B	0.17716
BMLF1	ANXA2P2	0.23671
BMLF1	CUL4B	0.32424
BMLF1	KPNB1	0.1848
BMLF1	FEZ1	0.25101
BMLF1	CBX4	0.19215
BMLF1	FKBP9	0.22415
BMLF1	NDC80	0.23601

BMLF1	ATP10D	0.24724
BMLF1	SMG5	0.32006
BMLF1	STK38L	0.14544
BMLF1	ACTN2	0.20053
BMLF1	COL3A1	0.18472
BMLF1	GGA1	0.31028
BMLF1	CWC22	0.025151
BMLF1	LRCH4	0.44061
BMLF1	EFNA1	0.20058
BMLF1	ATF5	0.18718
BMLF1	CNOT7	0.14865
BMLF1	HGD	0.065516
BMLF1	SEPN1	0.38196
BMLF1	BMP1	0.051274
BMLF1	IFIT3	0.32367
BMLF1	PRG2	0.038518
BMLF1	PRSS2	0.47495
BMLF1	STAT2	0.28349
BMLF1	PRDM5	0.20822
BMLF1	FSHB	0.2649
BMLF1	PSRC1	0.25413
BMLF1	REEP1	0.34269
BMLF1	PIFO	0.26455
BMLF1	ANKS1B	0.01002
BMLF1	METTL9	0.47224
BMLF1	CD59	0.3441
BMLF1	PABPC1L	0.039667
BMLF1	NCKAP1L	0.13127
BMLF1	LIMS1	0.30062
BMLF1	DDX43	0.012942
BMLF1	ZNF219	0.15685
BMLF1	ACTG1	0.17777
BMLF1	NCKAP1	0.23676
BMLF1	EIF4A2	0.29575
BMLF1	PNRC2	0.31436
BMLF1	PDE4DIP	0.34231
BMLF1	RAB27A	0.14984
BMLF1	SESTD1	0.33275
BMLF1	FKBP4	0.15676
BMLF1	KANK2	0.31147
BMLF1	RPL5	0.21479
BMLF1	TECPR2	0.47224
BMLF1	CDCA5	0.27767
BMLF1	HAX1	0.3323
BMLF1	STAU2	0.34465
BMLF1	THUMPD1	0.053548
BMLF1	KHSRP	0.031842
BMLF1	SIRT1	0.014439
BMLF1	NOS3	0.040988
BMLF1	PITPNM3	0.51877
BMLF1	SEPSECS	0.28913
BMLF1	HEMK1	0.10083
BMLF1	VIL1	0.25177
BMLF1	TNIP2	0.14244
BMLF1	CPSF4L	0.017834
BMLF1	PLCH2	0.044039
BMLF1	CELF4	0.030514
BMLF1	TAOK2	0.020485
BMLF1	FAM210A	0.086478
BMLF1	ADCY6	0.085271
BMLF1	S100A7L2	0.23305
BMLF1	RNF126	0.40834
BMLF1	EIF5B	0.33909
BMLF1	STX11	0.3672
BMLF1	FSTL3	0.1773
BMLF1	ELK4	0.20571
BMLF1	WEE1	0.14181
BMLF1	PSMA6	0.11013

BMLF1	MYH3	0.010294
BMLF1	TMOD4	0.014055
BMLF1	PCDHA3	0.21127
BMLF1	MUC5B	0.37918
BMLF1	GSTM3	0.059754
BMLF1	MED16	0.012675
BMLF1	CIB3	0.26298
BMLF1	CCDC59	0.36073
BMLF1	CD44	0.16164
BMLF1	PPID	0.40697
BMLF1	AGL	0.17662
BMLF1	SMNDC1	0.38064
BMLF1	AATF	0.1619
BMLF1	TFF2	0.32591
BMLF1	KLK11	0.047704
BMLF1	NEDD9	0.249
BMLF1	CNOT1	0.29324
BMLF1	RPL14	0.27919
BMLF1	YIR021W-A	0.012446
BMLF1	PTPN9	0.22451
BMLF1	HDLBP	0.041315
BMLF1	ATF7	0.13243
BMLF1	GUCY2C	0.15819
BMLF1	HPCA	0.24729
BMLF1	SMC5	0.26884
BMLF1	ZRANB1	0.22204
BMLF1	COMMD9	0.028077
BMLF1	DNASE1L2	0.20553
BMLF1	TST	0.06786
BMLF1	CHRNA2	0.016819
BMLF1	PTPRO	0.11033
BMLF1	SLC6A9	0.024487
BMLF1	KCNJ4	0.34919
BMLF1	ANK3	0.13686
BMLF1	SYPL1	0.14904
BMLF1	DDX6	0.2859
BMLF1	APOBEC1	0.17025
BMLF1	ERLIN1	0.38192
BMLF1	UBXN11	0.033386
BMLF1	ANGEL2	0.01296
BMLF1	NLRP3	0.21033
BMLF1	ZNF473	0.25607
BMLF1	RAD9B	0.40063
BMLF1	AHSA1	0.36244
BMLF1	GTF2A2	0.19667
BMLF1	VSIG4	0.39725
BMLF1	TOP1	0.18484
BMLF1	DNMT3B	0.16561
BMLF1	RAC3	0.18162
BMLF1	IGF2R	0.23486
BMLF1	CRB3	0.035371
BMLF1	SORCS1	0.40607
BMLF1	SETDB2	0.045193
BMLF1	PYURF	0.39353
BMLF1	HRG	0.12291
BMLF1	ORC3	0.30505
BMLF1	SCYL3	0.36262
BMLF1	HBP1	0.021559
BMLF1	FDFT1	0.035967
BMLF1	CCL18	0.34823
BMLF1	ARPC3	0.31462
BMLF1	SIT1	0.32825
BMLF1	NUAK1	0.22612
BMLF1	TXN	0.1137
BMLF1	DIABLO	0.29872
BMLF1	C14orf166	0.23091
BMLF1	CDC25C	0.079772
BMLF1	PIK3R4	0.23239

BMLF1	XRCC1	0.31758
BMLF1	CETN1	0.47863
BMLF1	PRSS48	0.049094
BMLF1	UBQLN4	0.32756
BMLF1	HCN1	0.014033
BMLF1	CHRNA7	0.025146
BMLF1	KCNJ9	0.35474
BMLF1	CRABP1	0.038664
BMLF1	LPP	0.29725
BMLF1	ZBTB25	0.18244
BMLF1	BCOR	0.1928
BMLF1	TTBK2	0.28771
BMLF1	MPHOSPH10	0.27805
BMLF1	MLLT10	0.18077
BMLF1	PFKFB2	0.16842
BMLF1	LCN1	0.047487
BMLF1	CUL3	0.14865
BMLF1	DPH5	0.054554
BMLF1	NTNG1	0.37289
BMLF1	MC1R	0.19085
BMLF1	NAT8	0.02031
BMLF1	COG7	0.28152
BMLF1	PROCR	0.33666
BMLF1	ERC2	0.34591
BMLF1	ATP1B2	0.28439
BMLF1	PIWIL2	0.018508
BMLF1	ZSWIM7	0.32764
BMLF1	C2CD4D	0.022468
BMLF1	MAP2K2	0.061479
BMLF1	HLA-A	0.19914
BMLF1	RRP1B	0.038503
BMLF1	GK	0.30723
BMLF1	GNB2	0.24777
BMLF1	HSPB9	0.4036
BMLF1	PRMT7	0.054458
BMLF1	PSMC6	0.1404
BMLF1	PHACTR4	0.029988
BMLF1	NDFIP1	0.17712
BMLF1	PGK2	0.047861
BMLF1	STK10	0.25731
BMLF1	ANXA13	0.23379
BMLF1	HTRA2	0.17639
BMLF1	NUP98	0.16428
BMLF1	ANK2	0.065812
BMLF1	NFKBIA	0.076663
BMLF1	SOD3	0.21317
BMLF1	SLC1A1	0.027576
BMLF1	SEPT6	0.23257
BMLF1	MYO5A	0.09207
BMLF1	CIC	0.34933
BMLF1	SLC1A2	0.046623
BMLF1	FAR2	0.045652
BMLF1	RALYL	0.039667
BMLF1	COMMD8	0.47224
BMLF1	IGFBP2	0.26607
BMLF1	HTR3B	0.019841
BMLF1	DUSP2	0.15812
BMLF1	CCR5	0.14099
BMLF1	RPS13	0.23812
BMLF1	TAF8	0.24799
BMLF1	IMMP1L	0.069289
BMLF1	CRHR2	0.24582
BMLF1	METTL22	0.059751
BMLF1	IL1RAPL1	0.22378
BMLF1	PBX1	0.073466
BMLF1	HPN	0.17949
BMLF1	ATP5S	0.011824
BMLF1	VLDLR	0.18116

BMLF1	CEP55	0.018203
BMLF1	NUP62	0.092582
BMLF1	INPPL1	0.14163
BMLF1	TRIP13	0.22566
BMLF1	CDH1	0.099508
BMLF1	HTT	0.054334
BMLF1	DNAJB4	0.33704
BMLF1	ADAMTS13	0.27108
BMLF1	BCAP31	0.14837
BMLF1	DIAPH1	0.17403
BMLF1	TEAD1	0.22126
BMLF1	SMO	0.018475
BMLF1	BCL6	0.065425
BMLF1	MAP1LC3B	0.35906
BMLF1	USF2	0.16395
BMLF1	UBA5	0.22708
BMLF1	TTN	0.11098
BMLF1	ZDHHC17	0.27456
BMLF1	UGT1A8	0.010237
BMLF1	MICU3	0.24729
BMLF1	CAPNS2	0.23981
BMLF1	AK2	0.067757
BMLF1	NR1H3	0.036468
BMLF1	ABT1	0.30456
BMLF1	NDRG2	0.22215
BMLF1	FAM19A3	0.12243
BMLF1	TWIST2	0.1124
BMLF1	TRMT12	0.058026
BMLF1	FSTL4	0.25502
BMLF1	RBMXL3	0.039667
BMLF1	POLB	0.16186
BMLF1	MICAL3	0.03219
BMLF1	ACTR3	0.12599
BMLF1	BACE1	0.10775
BMLF1	RELA	0.014522
BMLF1	DCPS	0.20864
BMLF1	TUT1	0.25604
BMLF1	PSMC5	0.11215
BMLF1	NCALD	0.21086
BMLF1	MUS81	0.27135
BMLF1	PRDM16	0.17239
BMLF1	EGF	0.19815
BMLF1	RAPGEF2	0.15488
BMLF1	TFAM	0.19181
BMLF1	TJP1	0.24502
BMLF1	CAMSAP3	0.046028
BMLF1	RPL8	0.015074
BMLF1	CBX1	0.24086
BMLF1	UIMC1	0.2548
BMLF1	HOMER3	0.31806
BMLF1	AJUBA	0.090261
BMLF1	MIEF1	0.34327
BMLF1	TIRAP	0.11009
BMLF1	PDK3	0.3103
BMLF1	FYN	0.085504
BMLF1	PRKAB2	0.14999
BMLF1	AK7	0.10953
BMLF1	USP26	0.34583
BMLF1	PPP1R16B	0.36396
BMLF1	HIVEP1	0.25782
BMLF1	ELOVL5	0.264
BMLF1	LTBP4	0.43916
BMLF1	BLOC1S6	0.18379
BMLF1	NABP1	0.040477
BMLF1	CCDC132	0.012901
BMLF1	MOAP1	0.4164
BMLF1	RANBP2	0.15147
BMLF1	PRMT2	0.23809

BMLF1	RPS19	0.22106
BMLF1	DCAF16	0.4511
BMLF1	SMARCAD1	0.22008
BMLF1	CDKN1B	0.10661
BMLF1	SDSL	0.041529
BMLF1	NPHP4	0.20892
BMLF1	GNB2L1	0.069864
BMLF1	NUP43	0.19672
BMLF1	AMELX	0.2387
BMLF1	BRIP1	0.061821
BMLF1	ATG4C	0.028992
BMLF1	FAM21B	0.034991
BMLF1	PLSCR4	0.54006
BMLF1	LMNA	0.19278
BMLF1	INSIG1	0.22938
BMLF1	CST8	0.032511
BMLF1	DNAJC9-AS1	0.021739
BMLF1	CENPF	0.11359
BMLF1	LIN37	0.44381
BMLF1	MPP7	0.33761
BMLF1	VAMP2	0.24617
BMLF1	RBM8A	0.23644
BMLF1	ALKBH3	0.2892
BMLF1	METTL12	0.058106
BMLF1	ZFAND6	0.26123
BMLF1	SDR42E2	0.021214
BMLF1	SHANK1	0.16499
BMLF1	DCP2	0.27474
BMLF1	RBM23	0.011483
BMLF1	CCNK	0.26546
BMLF1	FBXL2	0.37306
BMLF1	SRSF6	0.026072
BMLF1	RBP1	0.035315
BMLF1	SCOC	0.32438
BMLF1	ALDH9A1	0.065128
BMLF1	LAMB3	0.25293
BMLF1	MBD4	0.22833
BMLF1	RXRB	0.13445
BMLF1	FBXO25	0.37314
BMLF1	KAT8	0.17251
BMLF1	IQGAP3	0.031711
BMLF1	NID1	0.18599
BMLF1	MEPE	0.41288
BMLF1	GOLGB1	0.35805
BMLF1	SETD3	0.042729
BMLF1	GALNT3	0.11415
BMLF1	HBA1	0.27451
BMLF1	ATP2C1	0.063508
BMLF1	NXF1	0.26669
BMLF1	NNMT	0.045821
BMLF1	GMIP	0.3068
BMLF1	C1R	0.2496
BMLF1	TUBG1	0.22093
BMLF1	SLC6A2	0.36018
BMLF1	C1orf106	0.017751
BMLF1	AKR1B10	0.35689
BMLF1	NOP2	0.45934
BMLF1	DNAJC1	0.23247
BMLF1	FCGBP	0.44387
BMLF1	CPEB3	0.020892
BMLF1	ZC3H11A	0.39591
BMLF1	TSHZ3	0.10037
BMLF1	SERPINC1	0.29512
BMLF1	FN1	0.18113
BMLF1	ACE	0.088613
BMLF1	KRTAP12-4	0.045197
BMLF1	SRSF2	0.31964
BMLF1	WIPF3	0.09008

BMLF1	TMED3	0.045347
BMLF1	RHOT1	0.33756
BMLF1	WWC2-AS2	0.020392
BMLF1	RPS6KB1	0.039925
BMLF1	DDX39A	0.30042
BMLF1	SAP18	0.29147
BMLF1	SAFB	0.27332
BMLF1	ASB13	0.43457
BMLF1	SYNGR3	0.046121
BMLF1	TEAD4	0.15464
BMLF1	MMP25	0.10154
BMLF1	SNX9	0.30396
BMLF1	NLGN4X	0.18899
BMLF1	COL4A3	0.15467
BMLF1	CITED2	0.045731
BMLF1	CSRP3	0.19176
BMLF1	VPS53	0.36182
BMLF1	TTI1	0.47224
BMLF1	NCBP2	0.15839
BMLF1	ZC3H15	0.35143
BMLF1	IFRD2	0.44542
BMLF1	TNPO1	0.26237
BMLF1	LSM1	0.29801
BMLF1	COPS4	0.38094
BMLF1	ABI3	0.38831
BMLF1	UBXN4	0.33748
BMLF1	NLRP12	0.25788
BMLF1	FBXL18	0.014361
BMLF1	CSF2	0.24981
BMLF1	KCNK13	0.012189
BMLF1	PADI6	0.17147
BMLF1	DNAJC10	0.19203
BMLF1	PARN	0.21986
BMLF1	FAM175A	0.3542
BMLF1	ZCCHC8	0.31384
BMLF1	TRAF3	0.1113
BMLF1	RYR2	0.14119
BMLF1	DLAT	0.32535
BMLF1	STRADA	0.11281
BMLF1	ACTR5	0.28904
BMLF1	NDUFAF7	0.033504
BMLF1	SLC22A10	0.018323
BMLF1	AXL	0.10627
BMLF1	RAET1E	0.37983
BMLF1	SMC6	0.30966
BMLF1	ESF1	0.045694
BMLF1	PARP9	0.45951
BMLF1	OPRM1	0.13074
BMLF1	GTPBP4	0.20844
BMLF1	CRLF1	0.34645
BMLF1	PRF1	0.37865
BMLF1	KIAA1109	0.38013
BMLF1	CACNA1I	0.29019
BMLF1	TFF1	0.3399
BMLF1	PCDHB3	0.19264
BMLF1	RGS9BP	0.010999
BMLF1	RPS6KA4	0.10613
BMLF1	PPP1R13L	0.20675
BMLF1	HSD17B14	0.33153
BMLF1	EXOC4	0.15027
BMLF1	TRIM71	0.019518
BMLF1	SEPT12	0.25794
BMLF1	MMP19	0.079856
BMLF1	CUL7	0.27993
BMLF1	TPT1	0.1392
BMLF1	CDC16	0.22878
BMLF1	IL6ST	0.17286
BMLF1	CDC20	0.16565

BMLF1	AK4	0.060418
BMLF1	STRN	0.27896
BMLF1	NSUN5	0.059751
BMLF1	MYO1E	0.24109
BMLF1	SPRY4	0.36631
BMLF1	KRTAP16-1	0.045197
BMLF1	ZNF366	0.25361
BMLF1	DDX20	0.18765
BMLF1	TEN1	0.35996
BMLF1	NKX3-1	0.029803
BMLF1	UBE2V1	0.16495
BMLF1	METTL18	0.11408
BMLF1	TRMT10C	0.43289
BMLF1	OCA2	0.22587
BMLF1	IGF1R	0.084582
BMLF1	PLA2G2C	0.19202
BMLF1	DGKZ	0.22509
BMLF1	SNRPA1	0.31416
BMLF1	LAIR1	0.43034
BMLF1	MICU2	0.25509
BMLF1	F2RL2	0.26718
BMLF1	VEGFB	0.17482
BMLF1	NHLRC1	0.27217
BMLF1	GFER	0.34696
BMLF1	SOCS2	0.24109
BMLF1	ZNF423	0.22468
BMLF1	MZT1	0.37221
BMLF1	IGF2BP1	0.17664
BMLF1	RNF144B	0.27494
BMLF1	PPP1R12C	0.45783
BMLF1	MCFD2	0.17167
BMLF1	TAAR3	0.043938
BMLF1	CCDC28B	0.44364
BMLF1	MYL12B	0.16852
BMLF1	EIF3K	0.35228
BMLF1	PI4KA	0.35435
BMLF1	GMFB	0.01449
BMLF1	CBY1	0.29255
BMLF1	SLC25A24	0.21019
BMLF1	SGCE	0.18524
BMLF1	RBMY1D	0.011243
BMLF1	MAP3K4	0.20915
BMLF1	GPR143	0.2048
BMLF1	CBS	0.08527
BMLF1	TIMM9	0.24043
BMLF1	PARP14	0.032664
BMLF1	LNX1	0.29342
BMLF1	PKD2L2	0.21875
BMLF1	VDAC2	0.21698
BMLF1	TMPRSS9	0.044875
BMLF1	IL13	0.19022
BMLF1	USP22	0.17031
BMLF1	SHARPIN	0.21431
BMLF1	OLFM1	0.3946
BMLF1	SART3	0.35757
BMLF1	PACS2	0.046964
BMLF1	AFM	0.030802
BMLF1	PRPF40A	0.31282
BMLF1	LSM4	0.30232
BMLF1	WNK3	0.21999
BMLF1	FBXO11	0.24364
BMLF1	SNRNP70	0.33128
BMLF1	LDLRAP1	0.15326
BMLF1	EPHB4	0.23216
BMLF1	PCSK9	0.10134
BMLF1	DGKI	0.29985
BMLF1	HSPB7	0.35164
BMLF1	PGK1	0.029837

BMLF1	NOP10	0.32552
BMLF1	RB1	0.021925
BMLF1	SHMT1	0.010744
BMLF1	RBM27	0.020474
BMLF1	ELANE	0.22632
BMLF1	CNPY4	0.095066
BMLF1	RING1	0.17291
BMLF1	ZNF496	0.17165
BMLF1	NELFA	0.2773
BMLF1	VCPKMT	0.05621
BMLF1	ACVR1B	0.079252
BMLF1	AP1S2	0.058855
BMLF1	TCEB2	0.24244
BMLF1	TRIM23	0.11309
BMLF1	TMPRSS13	0.039928
BMLF1	MYPN	0.33221
BMLF1	BRD3	0.29953
BMLF1	SETD2	0.22036
BMLF1	ZRANB2	0.25468
BMLF1	TRIM32	0.16636
BMLF1	OBP2B	0.011448
BMLF1	PLD2	0.086401
BMLF1	UBXN2A	0.034106
BMLF1	YWHAH	0.23776
BMLF1	HEMGN	0.37966
BMLF1	PARP4	0.27589
BMLF1	CNEP1R1	0.28731
BMLF1	EIF2D	0.095251
BMLF1	HNRNPM	0.29847
BMLF1	DHFRL1	0.018961
BMLF1	KRT6A	0.34024
BMLF1	KLRF2	0.3926
BMLF1	AKR1CL1	0.019238
BMLF1	AKAP17A	0.33619
BMLF1	APOO	0.038264
BMLF1	PCDHA6	0.2049
BMLF1	SERPINB8	0.38069
BMLF1	MED28	0.28809
BMLF1	PLEC	0.27042
BMLF1	KLK1	0.024559
BMLF1	FADD	0.15907
BMLF1	PRSS55	0.047697
BMLF1	TULP3	0.29692
BMLF1	FAM19A4	0.032535
BMLF1	ARRDC3	0.43074
BMLF1	IPMK	0.03125
BMLF1	TSC22D4	0.22434
BMLF1	RPS6KA5	0.073774
BMLF1	SERBP1	0.40872
BMLF1	PTBP2	0.023498
BMLF1	TGFBRAP1	0.32577
BMLF1	FHOD1	0.31003
BMLF1	XRCC4	0.16929
BMLF1	ZFC3H1	0.4071
BMLF1	TP53BP2	0.27603
BMLF1	RNF187	0.26532
BMLF1	E2F3	0.17698
BMLF1	ZNF711	0.26986
BMLF1	PTPN2	0.18701
BMLF1	NKX2-5	0.014963
BMLF1	STIP1	0.32541
BMLF1	MALT1	0.13069
BMLF1	SQSTM1	0.07271
BMLF1	SUPT5H	0.1643
BMLF1	ELMO2	0.20925
BMLF1	ATP6V0A2	0.24497
BMLF1	SNCA	0.034526
BMLF1	FBXO27	0.43665

BMLF1	EMR3	0.16114
BMLF1	CARD10	0.37838
BMLF1	UBE3C	0.28164
BMLF1	SLC2A10	0.030391
BMLF1	RANBP6	0.3722
BMLF1	MCIDAS	0.26045
BMLF1	CSDE1	0.043078
BMLF1	S100A8	0.23235
BMLF1	THAP11	0.28431
BMLF1	RBP5	0.041125
BMLF1	XYLB	0.092454
BMLF1	MT-ND3	0.3231
BMLF1	CD70	0.2716
BMLF1	IL2RG	0.27873
BMLF1	DUSP26	0.15579
BMLF1	NID2	0.61605
BMLF1	MBOAT1	0.028482
BMLF1	PMS2P1	0.048165
BMLF1	SYP	0.1422
BMLF1	F2R	0.057107
BMLF1	SMYD3	0.024468
BMLF1	CCNB1	0.077417
BMLF1	CADM1	0.14695
BMLF1	DIS3L	0.33572
BMLF1	TNRC6A	0.017836
BMLF1	NELL1	0.42945
BMLF1	DIRAS3	0.31434
BMLF1	SLC16A7	0.027478
BMLF1	SLC4A3	0.013281
BMLF1	TICRR	0.32753
BMLF1	SULT1C3	0.086999
BMLF1	FAM84B	0.44387
BMLF1	SLX1A	0.30543
BMLF1	ZNF365	0.39277
BMLF1	DEDD	0.27288
BMLF1	SRBD1	0.076036
BMLF1	ZHX1	0.16704
BMLF1	CPEB2	0.027163
BMLF1	GSTA3	0.041163
BMLF1	ANXA1	0.089947
BMLF1	ytzL	0.13583
BMLF1	FAF2	0.39131
BMLF1	CDKN2B	0.14971
BMLF1	EMID1	0.021576
BMLF1	PFDN5	0.27831
BMLF1	PCSK6	0.027155
BMLF1	WDR96	0.040617
BMLF1	ZMYND11	0.21233
BMLF1	CLDN7	0.2548
BMLF1	KRAS	0.1326
BMLF1	PTPRE	0.16534
BMLF1	C3	0.1874
BMLF1	PCDHB16	0.20505
BMLF1	FOXO4	0.032887
BMLF1	PTPRK	0.093785
BMLF1	RPN1	0.023945
BMLF1	VAV1	0.088441
BMLF1	SNTN	0.24735
BMLF1	STT3B	0.022056
BMLF1	SIRT3	0.26991
BMLF1	DAD1	0.02143
BMLF1	ADA	0.060107
BMLF1	CRHBP	0.13549
BMLF1	DNER	0.42804
BMLF1	FBXO17	0.43665
BMLF1	TAAR8	0.043938
BMLF1	COL17A1	0.28954
BMLF1	PRKAR1A	0.20792

BMLF1	EIF6	0.4105
BMLF1	CD72	0.30882
BMLF1	VSNL1	0.26298
BMLF1	RUVBL2	0.12573
BMLF1	FBXL14	0.43266
BMLF1	PCDHGA6	0.22489
BMLF1	CLN5	0.29469
BMLF1	CFL1	0.17662
BMLF1	CST4	0.034289
BMLF1	MBD1	0.18411
BMLF1	CARD18	0.044047
BMLF1	MKL2	0.28536
BMLF1	SCNN1G	0.25867
BMLF1	PHLDA1	0.36728
BMLF1	TNRC6B	0.019043
BMLF1	C15orf65	0.016825
BMLF1	NDUFS3	0.27588
BMLF1	POU2F1	0.18521
BMLF1	SLC25A10	0.20945
BMLF1	ATP8A1	0.27302
BMLF1	HMG20A	0.22699
BMLF1	TLX1	0.19083
BMLF1	IL17RA	0.32621
BMLF1	CDK4	0.09538
BMLF1	UBE2V2	0.26579
BMLF1	PACSIN2	0.080214
BMLF1	EFCAB2	0.26298
BMLF1	DSCAM	0.28861
BMLF1	MAPKAP1	0.037111
BMLF1	NUCB1	0.22368
BMLF1	SVOPL	0.018323
BMLF1	ABCC6	0.037823
BMLF1	SAFB2	0.35803
BMLF1	CHRNA6	0.01603
BMLF1	NDUFA5	0.028852
BMLF1	TMPRSS12	0.047697
BMLF1	CYTH1	0.27146
BMLF1	HEYL	0.096221
BMLF1	ELOVL6	0.27846
BMLF1	EPDR1	0.24721
BMLF1	PPARGC1A	0.10652
BMLF1	NRXN1	0.23655
BMLF1	PEF1	0.6463
BMLF1	OFD1	0.23422
BMLF1	TMPRSS5	0.030365
BMLF1	PARK2	0.040045
BMLF1	CD22	0.37941
BMLF1	RBM15B	0.28081
BMLF1	SHOX	0.21227
BMLF1	NOXA1	0.33439
BMLF1	DUX4L9	0.20458
BMLF1	SP1	0.040903
BMLF1	ZWINT	0.25078
BMLF1	NAPG	0.32894
BMLF1	ZFYVE26	0.33161
BMLF1	RBBP8	0.2166
BMLF1	MAX	0.15359
BMLF1	ANXA3	0.14206
BMLF1	FBXL8	0.47224
BMLF1	SLC22A17	0.021873
BMLF1	SNRPE	0.24652
BMLF1	CREBBP	0.039142
BMLF1	CGN	0.38379
BMLF1	XIAP	0.22567
BMLF1	GCHFR	0.24238
BMLF1	PLA2G16	0.20116
BMLF1	TGFB2	0.012452
BMLF1	EZR	0.19007

BMLF1	MFAP1	0.023253
BMLF1	COL16A1	0.34368
BMLF1	PSMA3	0.11444
BMLF1	RAD17	0.27019
BMLF1	FBL	0.383
BMLF1	TTYH1	0.14623
BMLF1	CNNM3	0.38475
BMLF1	RASGRP2	0.13917
BMLF1	SHBG	0.063184
BMLF1	TSSK6	0.22609
BMLF1	MEGF8	0.53519
BMLF1	MCU	0.28874
BMLF1	RBM41	0.039667
BMLF1	C10orf82	0.040339
BMLF1	ERICH1-AS1	0.028141
BMLF1	PDGFB	0.032785
BMLF1	PCDHGB5	0.22489
BMLF1	CHCHD6	0.43272
BMLF1	CDH13	0.090009
BMLF1	RNF168	0.16934
BMLF1	ATXN1	0.22623
BMLF1	FKBP9L	0.24509
BMLF1	TRIM25	0.11855
BMLF1	GAP43	0.27307
BMLF1	SLC32A1	0.073497
BMLF1	TTYH2	0.34608
BMLF1	BAK1	0.061734
BMLF1	CHP1	0.19371
BMLF1	PRICKLE3	0.38535
BMLF1	PAF1	0.22338
BMLF1	REEP5	0.41725
BMLF1	ANTXR2	0.33067
BMLF1	MAP4K2	0.16813
BMLF1	NFATC1	0.13584
BMLF1	CYFIP2	0.36069
BMLF1	CSTF2	0.31927
BMLF1	TNK2	0.1655
BMLF1	SYNGR1	0.038961
BMLF1	CAMTA2	0.2372
BMLF1	MAPK3	0.039395
BMLF1	ERAP1	0.18811
BMLF1	UBE2E1	0.18855
BMLF1	SUGP1	0.039088
BMLF1	CHCHD1	0.39248
BMLF1	APTX	0.14408
BMLF1	CST9LP1	0.034289
BMLF1	VPS52	0.37317
BMLF1	MYL12A	0.2576
BMLF1	CACNG2	0.22178
BMLF1	DAP3	0.24848
BMLF1	GOLPH3L	0.0103
BMLF1	ORMDL2	0.060541
BMLF1	SOX2	0.015982
BMLF1	ZGPAT	0.18219
BMLF1	ANKRD17	0.021243
BMLF1	RASSF3	0.36842
BMLF1	CFTR	0.1492
BMLF1	RAB3IP	0.26907
BMLF1	DYDC2	0.015638
BMLF1	SPG7	0.17879
BMLF1	GNAQ	0.097025
BMLF1	CTSH	0.13641
BMLF1	XPO5	0.28083
BMLF1	ABCD2	0.27075
BMLF1	CD86	0.20577
BMLF1	EGFL6	0.19362
BMLF1	KMT2E	0.21017
BMLF1	IMP4	0.33659

BMLF1	PRPF31	0.30409
BMLF1	NOMO2	0.37729
BMLF1	PPP1CC	0.03325
BMLF1	SYMPK	0.31623
BMLF1	CUL5	0.20904
BMLF1	CNST	0.38516
BMLF1	PADI3	0.16743
BMLF1	COL6A2	0.27671
BMLF1	FAM203B	0.016941
BMLF1	FOXG1	0.078561
BMLF1	TRAPPC10	0.36561
BMLF1	BCL9L	0.025295
BMLF1	LIPE	0.16546
BMLF1	HAP1	0.30034
BMLF1	ATP6V1C1	0.26892
BMLF1	PSMB7	0.12158
BMLF1	RXRA	0.032436
BMLF1	TET1	0.016033
BMLF1	PIK3R1	0.01989
BMLF1	AHCYL1	0.33419
BMLF1	TGM2	0.26796
BMLF1	RPLP0	0.24177
BMLF1	NR3C1	0.05198
BMLF1	BAG1	0.22993
BMLF1	ATF7IP	0.27955
BMLF1	MAPK10	0.13663
BMLF1	EPM2A	0.10935
BMLF1	FASN	0.20084
BMLF1	TAAR9	0.043938
BMLF1	DLL4	0.12378
BMLF1	SEPT7	0.11556
BMLF1	SLC17A2	0.02815
BMLF1	SMUG1	0.28537
BMLF1	HEATR5A	0.011327
BMLF1	BEX1	0.083798
BMLF1	MPZL1	0.28983
BMLF1	TTYH3	0.11655
BMLF1	TECTB	0.013054
BMLF1	BRCA2	0.084854
BMLF1	LCMT1	0.46405
BMLF1	TRIM72	0.10174
BMLF1	COL1A1	0.08291
BMLF1	EID2	0.24882
BMLF1	TMPRSS11A	0.042436
BMLF1	ELOVL7	0.29696
BMLF1	RPS3A	0.24903
BMLF1	GPR98	0.30458
BMLF1	PRPH2	0.019751
BMLF1	PDS5B	0.31019
BMLF1	PCYT1A	0.012738
BMLF1	CRY1	0.14439
BMLF1	NUMBL	0.38171
BMLF1	UBA3	0.18323
BMLF1	ACAA2	0.33348
BMLF1	PABPC5	0.038568
BMLF1	DLG4	0.060744
BMLF1	LSM6	0.28428
BMLF1	MBIP	0.36116
BMLF1	AHSG	0.053146
BMLF1	E2F4	0.10816
BMLF1	MICU1	0.49911
BMLF1	CALU	0.52897
BMLF1	EIF2S1	0.29027
BMLF1	PLCD4	0.026745
BMLF1	TBRG1	0.35469
BMLF1	ELF2	0.19608
BMLF1	ATP1B1	0.24991
BMLF1	CLPTM1	0.38

BMLF1	CEP63	0.32781
BMLF1	ZIC3	0.12658
BMLF1	ISCU	0.15086
BMLF1	RIPK1	0.06365
BMLF1	TICAM1	0.11736
BMLF1	AP2B1	0.18839
BMLF1	EXTL1	0.018808
BMLF1	KIRREL3	0.38016
BMLF1	GPSM2	0.26491
BMLF1	PSMB5	0.11549
BMLF1	ATXN2	0.20986
BMLF1	LILRB4	0.33451
BMLF1	PADI2	0.20209
BMLF1	XPC	0.23572
BMLF1	CD8A	0.18976
BMLF1	SSX2	0.33913
BMLF1	DCK	0.086258
BMLF1	LENG8	0.47224
BMLF1	PRKRA	0.28882
BMLF1	KLK2	0.046378
BMLF1	KLK8	0.33118
BMLF1	PTPN13	0.17107
BMLF1	PPP3CA	0.20433
BMLF1	STRADB	0.17075
BMLF1	MTUS2	0.39704
BMLF1	ANXA8L2	0.22732
BMLF1	CRYL1	0.064205
BMLF1	VWA9	0.01534
BMLF1	FBLN2	0.23528
BMLF1	ACADS	0.024509
BMLF1	BCL2A1	0.41721
BMLF1	MTTP	0.045949
BMLF1	PADI4	0.12779
BMLF1	HAND2	0.069756
BMLF1	PMEL	0.30508
BMLF1	TACR1	0.091345
BMLF1	SURF4	0.2922
BMLF1	MYOZ2	0.37297
BMLF1	PRODH2	0.015631
BMLF1	CTRC	0.050518
BMLF1	VCP	0.11537
BMLF1	APIP	0.22459
BMLF1	USP28	0.26927
BMLF1	PPP1CA	0.12608
BMLF1	DDX46	0.010537
BMLF1	ATF6	0.10793
BMLF1	GJB1	0.014893
BMLF1	ITK	0.15376
BMLF1	HOXC13	0.19992
BMLF1	GADD45G	0.32187
BMLF1	SIX1	0.04567
BMLF1	BTG1	0.23938
BMLF1	SEC11B	0.074508
BMLF1	GSTM4	0.030606
BMLF1	SVOP	0.076565
BMLF1	CCDC88A	0.045073
BMLF1	MCTP1	0.2338
BMLF1	CBFB	0.20596
BMLF1	OTUB1	0.28124
BMLF1	BEND2	0.025622
BMLF1	PRKX	0.17174
BMLF1	MOCS3	0.22579
BMLF1	CCNG1	0.28659
BMLF1	VSTM2L	0.47224
BMLF1	RFX6	0.2454
BMLF1	ZFYVE28	0.32508
BMLF1	FCGR2C	0.39674
BMLF1	AKAP6	0.20722

BMLF1	BZRAP1	0.4149
BMLF1	EIF4B	0.36446
BMLF1	ANXA10	0.24868
BMLF1	PCDHGB3	0.22489
BMLF1	GLS2	0.28771
BMLF1	RNF40	0.21966
BMLF1	SOX5	0.14036
BMLF1	POP1	0.30272
BMLF1	SELENBP1	0.30579
BMLF1	PVR	0.18595
BMLF1	ARSA	0.093781
BMLF1	NCOR2	0.21177
BMLF1	LRP2	0.21929
BMLF1	VMP1	0.28213
BMLF1	CAPN2	0.358
BMLF1	PAFAH1B3	0.28759
BMLF1	KRT8	0.30852
BMLF1	SLC23A1	0.21716
BMLF1	OCLN	0.17513
BMLF1	SIRPB1	0.39174
BMLF1	CPSF7	0.35003
BMLF1	ABCG5	0.19612
BMLF1	GSTT1	0.050499
BMLF1	UNC119	0.35167
BMLF1	ABL1	0.059971
BMLF1	DGCR8	0.33435
BMLF1	CLIC1	0.23293
BMLF1	MKRN3	0.29784
BMLF1	TTC1	0.41674
BMLF1	SUPT4H1	0.13019
BMLF1	SREBF2	0.086651
BMLF1	HNRNPC	0.31188
BMLF1	FAM188B	0.10335
BMLF1	TRIM37	0.22717
BMLF1	GRN	0.30517
BMLF1	PABPC4	0.30504
BMLF1	RBM45	0.010743
BMLF1	SLC22A11	0.31269
BMLF1	VPS33A	0.25885
BMLF1	RHCG	0.04299
BMLF1	TRIM5	0.21572
BMLF1	MMP17	0.092253
BMLF1	SLC22A15	0.018129
BMLF1	ANTXR1	0.28866
BMLF1	POMT2	0.047406
BMLF1	KCNJ12	0.014205
BMLF1	TNFRSF11A	0.17791
BMLF1	NPLOC4	0.2802
BMLF1	AK8	0.11312
BMLF1	NAA10	0.34706
BMLF1	RGS2	0.21112
BMLF1	CTSL	0.25646
BMLF1	MON1B	0.47224
BMLF1	TIFA	0.43209
BMLF1	CELA3A	0.047985
BMLF1	NUCB2	0.45939
BMLF1	TF	0.21968
BMLF1	CBR4	0.35981
BMLF1	RAD9A	0.19236
BMLF1	CBX6	0.31538
BMLF1	UHRF1BP1L	0.010157
BMLF1	MRPL51	0.37696
BMLF1	CNN1	0.011674
BMLF1	MSX2	0.053431
BMLF1	CDKN2A	0.094763
BMLF1	PKDREJ	0.19055
BMLF1	PPP1R13B	0.35588
BMLF1	DCC	0.21112

BMLF1	ITGB1	0.019842
BMLF1	LRSAM1	0.23388
BMLF1	SOX1	0.22773
BMLF1	HELZ2	0.2093
BMLF1	UNQ6126/PRO20091	0.024952
BMLF1	NODAL	0.011741
BMLF1	EED	0.25611
BMLF1	ASGR1	0.34613
BMLF1	NPC2	0.24921
BMLF1	TLL2	0.10542
BMLF1	HOXA9	0.21551
BMLF1	MAGEA11	0.41608
BMLF1	SH3RF2	0.3331
BMLF1	RAP1GAP	0.25667
BMLF1	USH1C	0.23205
BMLF1	PCDH19	0.22489
BMLF1	RNPS1	0.28176
BMLF1	FAM162A	0.34221
BMLF1	EPX	0.066021
BMLF1	SH3GLB1	0.19043
BMLF1	CDON	0.1909
BMLF1	BCL2L14	0.2795
BMLF1	GLRX3	0.081344
BMLF1	CELF6	0.010992
BMLF1	PINX1	0.20352
BMLF1	PSMD5	0.19739
BMLF1	PEX11A	0.31427
BMLF1	SYT9	0.025341
BMLF1	RIPK3	0.13207
BMLF1	S100A6	0.40364
BMLF1	RP1L1	0.36001
BMLF1	EPHB1	0.13012
BMLF1	AOC1	0.11755
BMLF1	STK16	0.20336
BMLF1	VHL	0.16568
BMLF1	FAT2	0.18265
BMLF1	FBXW2	0.41015
BMLF1	PLCG2	0.11869
BMLF1	CDCP1	0.027736
BMLF1	SMARCD3	0.11913
BMLF1	RNF20	0.18343
BMLF1	RHBG	0.10462
BMLF1	ZFP36	0.12911
BMLF1	PPP2R1A	0.10756
BMLF1	IPO13	0.33968
BMLF1	AFG3L2	0.14001
BMLF1	HENMT1	0.14795
BMLF1	EPB42	0.26709
BMLF1	WDR6	0.36689
BMLF1	DDC	0.18954
BMLF1	TERF2IP	0.16503
BMLF1	SPRED2	0.28156
BMLF1	ORMDL3	0.38192
BMLF1	NME3	0.020119
BMLF1	TLR4	0.020589
BMLF1	PKHD1	0.22349
BMLF1	RNF19B	0.34033
BMLF1	IKBKAP	0.16384
BMLF1	MED11	0.29791
BMLF1	NUBP1	0.082964
BMLF1	RABGEF1	0.28472
BMLF1	SLC17A4	0.02815
BMLF1	LRP8	0.35535
BMLF1	ZEB1	0.11986
BMLF1	NOX5	0.14356
BMLF1	SYNPO	0.28215
BMLF1	SULT4A1	0.044958
BMLF1	MAVS	0.085298

BMLF1	IGFLR1	0.43034
BMLF1	GGNBP2	0.045973
BMLF1	CASP2	0.18752
BMLF1	CT55	0.067561
BMLF1	RFC5	0.20005
BMLF1	PPP6R3	0.38744
BMLF1	KRT14	0.22712
BMLF1	EPG5	0.037125
BMLF1	B3GNTL1	0.021703
BMLF1	CBFA2T2	0.18435
BMLF1	GABRG2	0.21629
BMLF1	NUAK2	0.22564
BMLF1	SLC17A3	0.028768
BMLF1	NXT1	0.30739
BMLF1	S100P	0.57356
BMLF1	CMTR1	0.06873
BMLF1	SNAI1	0.13115
BMLF1	FLG	0.16525
BMLF1	NDUFS1	0.049241
BMLF1	BCL10	0.05184
BMLF1	CARD8	0.33611
BMLF1	PLK3	0.11897
BMLF1	SEMA4D	0.12697
BMLF1	SMURF1	0.15778
BMLF1	SIX5	0.2045
BMLF1	PAQR5	0.042637
BMLF1	KCNJ8	0.012695
BMLF1	TMEM67	0.2712
BMLF1	WASL	0.12805
BMLF1	SLC23A3	0.017366
BMLF1	LRP1B	0.21883
BMLF1	FAM131A	0.033611
BMLF1	DAZAP1	0.012258
BMLF1	DCAF7	0.37526
BMLF1	SGTA	0.40453
BMLF1	RABEP2	0.13836
BMLF1	SLC18A1	0.10622
BMLF1	XPO6	0.32039
BMLF1	TRAK1	0.2985
BMLF1	TMED7	0.060302
BMLF1	SERTAD1	0.35817
BMLF1	PCDHGA5	0.22489
BMLF1	RNF103	0.28444
BMLF1	FCN2	0.11961
BMLF1	CLASP1	0.13338
BMLF1	DMD	0.14999
BMLF1	E2F2	0.14274
BMLF1	LCA5	0.33829
BMLF1	INTS4	0.40314
BMLF1	DYNLRB1	0.29263
BMLF1	GYPC	0.40432
BMLF1	SEPT1	0.36487
BMLF1	EGLN2	0.24048
BMLF1	RAF1	0.048836
BMLF1	PRDX6	0.33639
BMLF1	WT1	0.038897
BMLF1	YWHAZ	0.18397
BMLF1	DNAJC15	0.015703
BMLF1	CHAF1A	0.30901
BMLF1	BTF3	0.34843
BMLF1	IRGM	0.011535
BMLF1	KIAA0319L	0.43033
BMLF1	SLC22A1	0.21698
BMLF1	CAMKK2	0.096681
BMLF1	DAZ1	0.37809
BMLF1	SEPT9	0.28033
BMLF1	PPP2R5C	0.28592
BMLF1	TAF9	0.14656

BMLF1	PTCRA	0.43029
BMLF1	SETD1B	0.31923
BMLF1	SAA1	0.015183
BMLF1	RIMS2	0.33012
BMLF1	PRSS3P2	0.028743
BMLF1	TP53INP1	0.274
BMLF1	RPS16	0.015162
BMLF1	F7	0.092312
BMLF1	ELF4	0.19988
BMLF1	CADPS2	0.049855
BMLF1	ECH1	0.29203
BMLF1	CPS1	0.078048
BMLF1	C4BPA	0.3949
BMLF1	TAPBP	0.16275
BMLF1	RAPGEF1	0.29484
BMLF1	FEM1B	0.29965
BMLF1	SRSF4	0.025151
BMLF1	ATXN7L3	0.28108
BMLF1	FNBP1	0.38092
BMLF1	KMT2B	0.17686
BMLF1	MCM10	0.31041
BMLF1	KCNB1	0.25608
BMLF1	CASP9	0.25223
BMLF1	NR4A2	0.080166
BMLF1	VRK1	0.21303
BMLF1	MVK	0.05041
BMLF1	ACO1	0.066908
BMLF1	LTN1	0.34207
BMLF1	TEFM	0.25728
BMLF1	MTOR	0.044266
BMLF1	EIF1B	0.033537
BMLF1	PTTG1	0.21955
BMLF1	PLAU	0.22687
BMLF1	SHMT2	0.036008
BMLF1	DZIP1	0.27128
BMLF1	CLUAP1	0.42917
BMLF1	MADD	0.3147
BMLF1	NUP160	0.22291
BMLF1	NUP50	0.25104
BMLF1	TRIO	0.13954
BMLF1	RAB11FIP3	0.30616
BMLF1	PROCA1	0.24175
BMLF1	MOB1B	0.29636
BMLF1	EFCAB4B	0.49612
BMLF1	APP	0.032698
BMLF1	CDHR1	0.18164
BMLF1	HPX	0.2277
BMLF1	CLNS1A	0.27833
BMLF1	UCHL1	0.17864
BMLF1	GAB2	0.20079
BMLF1	ALKBH8	0.34079
BMLF1	PARD6G	0.34589
BMLF1	CAMK1	0.12446
BMLF1	SYBU	0.38913
BMLF1	UTP14A	0.37372
BMLF1	IQGAP2	0.064973
BMLF1	GPT	0.016642
BMLF1	PCDHB8	0.22489
BMLF1	SKAP1	0.10489
BMLF1	NOC4L	0.36376
BMLF1	YOD1	0.28164
BMLF1	GABPA	0.16175
BMLF1	ARL6IP1	0.42931
BMLF1	TMPRSS6	0.34599
BMLF1	BLOC1S4	0.35673
BMLF1	THAP3	0.40791
BMLF1	FAM21C	0.34374
BMLF1	GGN	0.048096

BMLF1	S100A7A	0.226
BMLF1	ZNF256	0.17833
BMLF1	MUC17	0.30454
BMLF1	PPIL1	0.015481
BMLF1	NTHL1	0.042306
BMLF1	AP2A1	0.18276
BMLF1	NME6	0.025496
BMLF1	TDGF1	0.10045
BMLF1	ZYX	0.24472
BMLF1	BICC1	0.047617
BMLF1	EFCAB3	0.26298
BMLF1	ANKZF1	0.3959
BMLF1	PFDN2	0.3349
BMLF1	PRRG2	0.23236
BMLF1	STK11	0.060918
BMLF1	DLGAP2	0.334
BMLF1	MMP24	0.093553
BMLF1	SMOC2	0.18581
BMLF1	CHST2	0.048567
BMLF1	STXBP3	0.21064
BMLF1	TJP3	0.38134
BMLF1	SALL1	0.067933
BMLF1	METTL23	0.056371
BMLF1	SERPING1	0.28056
BMLF1	HIP1R	0.28204
BMLF1	THOC1	0.22743
BMLF1	YTHDC2	0.3698
BMLF1	RAD51B	0.30718
BMLF1	CHRNE	0.020607
BMLF1	SLC4A9	0.013281
BMLF1	HNRNPA2B1	0.30147
BMLF1	CCT2	0.26983
BMLF1	FOSL1	0.11824
BMLF1	SETD6	0.405
BMLF1	MTPAP	0.23915
BMLF1	CASR	0.28465
BMLF1	YWHAE	0.23203
BMLF1	EDEM3	0.15282
BMLF1	TMEM120B	0.025567
BMLF1	ALDH1A2	0.020609
BMLF1	TPO	0.20237
BMLF1	GLA	0.17183
BMLF1	LAMP1	0.044905
BMLF1	NDUFA13	0.20784
BMLF1	CHGB	0.43778
BMLF1	ATP2A2	0.042918
BMLF1	PRKD3	0.13683
BMLF1	SLC16A11	0.031311
BMLF1	DTNA	0.46937
BMLF1	CMTR2	0.056371
BMLF1	ALDH1L1	0.043474
BMLF1	REPS2	0.68351
BMLF1	IMP3	0.34029
BMLF1	TMEM204	0.017127
BMLF1	TRNAU1AP	0.010742
BMLF1	OPRK1	0.25298
BMLF1	SH3BP4	0.39445
BMLF1	MVP	0.29358
BMLF1	SASS6	0.36816
BMLF1	PPP3R1	0.24556
BMLF1	RRAGB	0.28187
BMLF1	CCDC50	0.45783
BMLF1	CDH24	0.19044
BMLF1	LCN9	0.014749
BMLF1	TRIM44	0.3959
BMLF1	ARFGEF1	0.15674
BMLF1	PPEF2	0.35617
BMLF1	EFEMP2	0.62818

BMLF1	DVL3	0.20321
BMLF1	POU5F1	0.06605
BMLF1	EQTN	0.058139
BMLF1	IFNA2	0.2576
BMLF1	SLC1A7	0.028857
BMLF1	MAP4	0.22946
BMLF1	TGM7	0.024946
BMLF1	MYLK	0.16399
BMLF1	RLBP1	0.068114
BMLF1	ZC3H13	0.39591
BMLF1	ALX1	0.16444
BMLF1	NT5C3A	0.06263
BMLF1	SART1	0.28858
BMLF1	MT-CO1	0.18849
BMLF1	AUNIP	0.38331
BMLF1	TANK	0.34516
BMLF1	HERC5	0.23662
BMLF1	SUGP2	0.020066
BMLF1	DLEU1	0.47224
BMLF1	IBA57	0.045217
BMLF1	EIF3L	0.41754
BMLF1	STARD13	0.32217
BMLF1	ATCAY	0.2747
BMLF1	MAP1B	0.065425
BMLF1	HNRNPH1	0.28318
BMLF1	IFI6	0.33765
BMLF1	PFN2	0.32546
BMLF1	USP33	0.19195
BMLF1	DPH3	0.33828
BMLF1	ATP2A1	0.19128
BMLF1	DDX25	0.032366
BMLF1	UGGT2	0.017675
BMLF1	CCS	0.22857
BMLF1	POGZ	0.27264
BMLF1	SIK2	0.18906
BMLF1	PCDH15	0.12479
BMLF1	SLC6A16	0.028986
BMLF1	TCEA1	0.18412
BMLF1	TRRAP	0.25266
BMLF1	FZD4	0.10568
BMLF1	RPA2	0.15763
BMLF1	EXOSC4	0.22135
BMLF1	CSE1L	0.33082
BMLF1	JUP	0.035096
BMLF1	RNF10	0.23104
BMLF1	SCAMP5	0.25661
BMLF1	PADI1	0.20209
BMLF1	HIST3H3	0.26394
BMLF1	GSK3B	0.01899
BMLF1	CAB39	0.28371
BMLF1	WLS	0.40774
BMLF1	NOC2L	0.22651
BMLF1	ATP6V0A4	0.18011
BMLF1	PPP2R1B	0.45787
BMLF1	ITGA2	0.30059
BMLF1	CEP250	0.19685
BMLF1	EIF3D	0.4086
BMLF1	PDGFRL	0.20551
BMLF1	IFT52	0.057158
BMLF1	CXorf40A	0.47224
BMLF1	HNRNPD	0.21082
BMLF1	BCL2L1	0.045908
BMLF1	MYL3	0.16557
BMLF1	SCN8A	0.026073
BMLF1	AGXT	0.23369
BMLF1	BAG6	0.18702
BMLF1	IL15	0.16364
BMLF1	CHD9	0.23197

BMLF1	NEUROD1	0.049179
BMLF1	CSF2RB	0.30903
BMLF1	CD47	0.27486
BMLF1	CHMP3	0.24613
BMLF1	FOS	0.052065
BMLF1	PBK	0.30883
BMLF1	RFC4	0.20289
BMLF1	HOOK2	0.25292
BMLF1	FOXO3	0.041633
BMLF1	ZC3H12A	0.27562
BMLF1	CCP110	0.34639
BMLF1	SMG7	0.30871
BMLF1	CD200	0.36839
BMLF1	LSM2	0.19803
BMLF1	AP3M1	0.33016
BMLF1	DYNC1I1	0.21551
BMLF1	ZNF703	0.18973
BMLF1	CAGE1	0.01882
BMLF1	NFYB	0.20466
BMLF1	SH3GL3	0.36281
BMLF1	DMBT1	0.16439
BMLF1	MINK1	0.1479
BMLF1	PKN2	0.11227
BMLF1	MECOM	0.093344
BMLF1	LONP1	0.19469
BMLF1	PDCL3	0.4045
BMLF1	LINC00205	0.025703
BMLF1	JAM3	0.20105
BMLF1	ASB16	0.43457
BMLF1	MLH3	0.21274
BMLF1	ARHGAP17	0.31325
BMLF1	PKD2L1	0.33063
BMLF1	MOB1A	0.40884
BMLF1	FAM46B	0.011
BMLF1	APOL1	0.28904
BMLF1	ADRB3	0.13863
BMLF1	NAT8B	0.02083
BMLF1	TCEB1	0.23159
BMLF1	VPS4B	0.17721
BMLF1	FEZ2	0.41726
BMLF1	NFYA	0.21062
BMLF1	RNF111	0.18163
BMLF1	CMPK1	0.038807
BMLF1	DROSHA	0.24249
BMLF1	CD2BP2	0.3474
BMLF1	NINL	0.21246
BMLF1	CD2	0.26554
BMLF1	ACOT8	0.19863
BMLF1	TPPP3	0.23293
BMLF1	SWAP70	0.057653
BMLF1	NCOA6	0.14927
BMLF1	SMOC1	0.19508
BMLF1	SPPL2B	0.23064
BMLF1	ATR	0.17626
BMLF1	CYB5RL	0.019466
BMLF1	DMPK	0.085992
BMLF1	HTN1	0.40527
BMLF1	TRIM3	0.29384
BMLF1	EDC4	0.26855
BMLF1	NSUN5P2	0.065329
BMLF1	PAX9	0.2894
BMLF1	ZNF408	0.25531
BMLF1	BTN3A1	0.39911
BMLF1	BIK	0.27601
BMLF1	PLEKHA1	0.15377
BMLF1	MIP	0.025705
BMLF1	PRSS36	0.058072
BMLF1	DAB2	0.13592

BMLF1	OR2L13	0.32351
BMLF1	GSTT2	0.025079
BMLF1	HGS	0.19319
BMLF1	ARHGEF11	0.1432
BMLF1	SLC9A3R1	0.11008
BMLF1	GFOD2	0.017181
BMLF1	TOPBP1	0.28535
BMLF1	BOLL	0.37908
BMLF1	MYLK2	0.090029
BMLF1	RYBP	0.23062
BMLF1	EHD3	0.39174
BMLF1	TUBB4A	0.12692
BMLF1	DYRK1A	0.14707
BMLF1	TGM1	0.3653
BMLF1	JAK1	0.20285
BMLF1	NQO2	0.098428
BMLF1	ZCCHC4	0.024129
BMLF1	CAPN3	0.015052
BMLF1	IGF2	0.16863
BMLF1	FES	0.032162
BMLF1	HSPA2	0.33738
BMLF1	FAM64A	0.45926
BMLF1	KIAA2018	0.20011
BMLF1	OCIAD1	0.43227
BMLF1	BCR	0.1717
BMLF1	PARP11	0.064778
BMLF1	SRCIN1	0.20399
BMLF1	TMEM8B	0.30546
BMLF1	MMP11	0.10015
BMLF1	INSRR	0.025569
BMLF1	SLC14A2	0.34043
BMLF1	ZZEF1	0.21325
BMLF1	AGER	0.18791
BMLF1	RICTOR	0.28312
BMLF1	MYL10	0.25509
BMLF1	PDGFRA	0.03932
BMLF1	LY96	0.2335
BMLF1	UBE2M	0.27412
BMLF1	DPP9	0.071044
BMLF1	NF2	0.12722
BMLF1	MICALL1	0.38383
BMLF1	CSK	0.17957
BMLF1	SMAD2	0.039487
BMLF1	RPS2	0.24911
BMLF1	UTF1	0.28774
BMLF1	EXOSC8	0.31255
BMLF1	IL4R	0.25177
BMLF1	NR3C2	0.17624
BMLF1	RNF4	0.06669
BMLF1	KRT15	0.37143
BMLF1	DUT	0.27187
BMLF1	NTN1	0.29734
BMLF1	ZXDA	0.17964
BMLF1	COQ3	0.058688
BMLF1	MAGEC1	0.47224
BMLF1	POU2F3	0.1663
BMLF1	EIF4G3	0.062129
BMLF1	P4HTM	0.18809
BMLF1	EFCAB11	0.26298
BMLF1	MT1G	0.25241
BMLF1	DLGAP5	0.27763
BMLF1	ATRX	0.21395
BMLF1	FKBP2	0.3664
BMLF1	TMEM102	0.35726
BMLF1	VWA5B2	0.026199
BMLF1	RNF6	0.20735
BMLF1	TAF9B	0.23021
BMLF1	DTL	0.25639

BMLF1	UVRAG	0.34778
BMLF1	COPB1	0.17643
BMLF1	IP6K3	0.017343
BMLF1	KIF6	0.35982
BMLF1	THBS2	0.58898
BMLF1	OIT3	0.21945
BMLF1	EPS15L1	0.18232
BMLF1	PELI2	0.30394
BMLF1	NELFB	0.27876
BMLF1	FAP	0.25431
BMLF1	NKAIN2	0.069175
BMLF1	SLIT1	0.10128
BMLF1	NAF1	0.33098
BMLF1	MLLT3	0.30959
BMLF1	PRAME	0.25685
BMLF1	CALM1	0.23247
BMLF1	CRYAB	0.139
BMLF1	SMARCA5	0.13066
BMLF1	ASAP2	0.16986
BMLF1	EIF4E2	0.42369
BMLF1	SLCO5A1	0.012309
BMLF1	PROM1	0.24615
BMLF1	STAMBPL1	0.38157
BMLF1	ACVR2B	0.062068
BMLF1	TAS2R10	0.030553
BMLF1	DBNL	0.25981
BMLF1	FGF3	0.27097
BMLF1	E2F1	0.086213
BMLF1	STRN4	0.40596
BMLF1	JUN	0.017169
BMLF1	AVPR1A	0.1333
BMLF1	RRP8	0.25924
BMLF1	HPRT1	0.13294
BMLF1	YWHAG	0.30506
BMLF1	NPM3	0.37927
BMLF1	DNASE1	0.29624
BMLF1	POLD1	0.033414
BMLF1	RNF216	0.24155
BMLF1	MAGI2	0.14983
BMLF1	EPOR	0.28065
BMLF1	STRIP1	0.37904
BMLF1	ARAP1	0.08982
BMLF1	EIF2A	0.28603
BMLF1	KCTD5	0.36114
BMLF1	CASP1	0.12609
BMLF1	ZRANB3	0.14991
BMLF1	GLUD1	0.23846
BMLF1	TFAP2C	0.094806
BMLF1	FLT3	0.15406
BMLF1	EXOC2	0.36582
BMLF1	ATG12	0.37416
BMLF1	RARG	0.065141
BMLF1	KIF1B	0.29507
BMLF1	SLC10A1	0.04133
BMLF1	CAV3	0.07769
BMLF1	SMARCB1	0.1555
BMLF1	CDHR3	0.22489
BMLF1	YEATS2	0.3351
BMLF1	KPNA5	0.33034
BMLF1	ARHGAP10	0.18392
BMLF1	S100A4	0.60064
BMLF1	MMP21	0.10542
BMLF1	IFNGR1	0.035367
BMLF1	ERCC5	0.1896
BMLF1	RPS4Y2	0.012026
BMLF1	ABCG2	0.26966
BMLF1	GUCA1B	0.21098
BMLF1	NAPA	0.24889

BMLF1	TNPO2	0.33966
BMLF1	GRID2	0.026438
BMLF1	FAM20B	0.068055
BMLF1	COL4A1	0.29758
BMLF1	DNAJC21	0.35082
BMLF1	S100A12	0.34275
BMLF1	FYCO1	0.39651
BMLF1	RGS16	0.40797
BMLF1	PGRMC2	0.034148
BMLF1	WNT1	0.061434
BMLF1	APOL6	0.018495
BMLF1	RAB9B	0.33861
BMLF1	C19orf80	0.45784
BMLF1	RCN1	0.66403
BMLF1	RASGRP3	0.10795
BMLF1	NUDT14	0.41039
BMLF1	PHC2	0.31032
BMLF1	MTMR2	0.10585
BMLF1	BMPR2	0.070592
BMLF1	IKBKE	0.15904
BMLF1	CDKN2A	0.050652
BMLF1	DDX39B	0.19164
BMLF1	WNT3A	0.033746
BMLF1	BAZ2B	0.30074
BMLF1	CASP8	0.075459
BMLF1	PRSS12	0.092978
BMLF1	NEFL	0.26394
BMLF1	FTSJ1	0.056358
BMLF1	KCNJ6	0.28148
BMLF1	PDSS1	0.069463
BMLF1	MYH2	0.23793
BMLF1	FKBP7	0.21661
BMLF1	MZB1	0.35833
BMLF1	LRRFIP1	0.26265
BMLF1	PPP2R3C	0.21335
BMLF1	KHK	0.030946
BMLF1	TNFRSF6B	0.40563
BMLF1	SMAD6	0.11194
BMLF1	TROAP	0.44326
BMLF1	MT1H	0.31612
BMLF1	ATG10	0.043871
BMLF1	RAPGEF4	0.13785
BMLF1	PSIP1	0.23696
BMLF1	SNRPD2	0.24406
BMLF1	KLK7	0.039031
BMLF1	SSX2IP	0.38656
BMLF1	ACD	0.21847
BMLF1	DPP4	0.2048
BMLF1	RERE	0.18012
BMLF1	SPSB4	0.43457
BMLF1	EIF3E	0.3097
BMLF1	EIF4E	0.26438
BMLF1	ST14	0.02866
BMLF1	DSCC1	0.31892
BMLF1	RAMP1	0.18919
BMLF1	BBC3	0.17693
BMLF1	HSPBP1	0.39571
BMLF1	SDC2	0.22131
BMLF1	AK6	0.43107
BMLF1	HRAS	0.062937
BMLF1	EMILIN2	0.40779
BMLF1	AIM2	0.17032
BMLF1	CLTCL1	0.30581
BMLF1	CELA2A	0.049094
BMLF1	NPHP1	0.20935
BMLF1	CHD7	0.10189
BMLF1	SLC15A2	0.26674
BMLF1	DNMT3A	0.23228

BMLF1	ATP6V1F	0.24773
BMLF1	OAS3	0.24359
BMLF1	INHBA	0.084435
BMLF1	CDH2	0.070263
BMLF1	RPS23	0.23306
BMLF1	RQCD1	0.26964
BMLF1	PAG1	0.33795
BMLF1	NPB	0.39591
BMLF1	PIM3	0.25462
BMLF1	ACTN3	0.55138
BMLF1	SREBF1	0.036314
BMLF1	ANXA4	0.22648
BMLF1	ATP5F1	0.32582
BMLF1	FBN1	0.55207
BMLF1	RPL6	0.037868
BMLF1	NR4A1	0.096679
BMLF1	RNF182	0.2791
BMLF1	MIF4GD	0.39296
BMLF1	PUS7	0.12827
BMLF1	MAP4K3	0.2709
BMLF1	TNNI2	0.34495
BMLF1	C19orf12	0.042181
BMLF1	RBKS	0.091293
BMLF1	ITPKA	0.044524
BMLF1	PDPK1	0.057772
BMLF1	RAD1	0.24051
BMLF1	ATG14	0.38666
BMLF1	C12orf63	0.016911
BMLF1	F10	0.37412
BMLF1	PTK2B	0.022266
BMLF1	CD46	0.014687
BMLF1	CAPN1	0.44275
BMLF1	PAX3	0.16168
BMLF1	UCKL1	0.3847
BMLF1	PTPRC	0.020223
BMLF1	PLXDC1	0.31134
BMLF1	HSPB8	0.25292
BMLF1	MED15	0.26141
BMLF1	DSG1	0.14497
BMLF1	CEACAM6	0.38978
BMLF1	GSTZ1	0.34805
BMLF1	TLE1	0.17617
BMLF1	SKIL	0.17345
BMLF1	LILRB3	0.35868
BMLF1	ZNF3	0.14742
BMLF1	RGS1	0.015899
BMLF1	ZNF346	0.32521
BMLF1	RPSA	0.15069
BMLF1	SRPK1	0.14541
BMLF1	IPO7	0.25305
BMLF1	TRIM24	0.07342
BMLF1	CFL2	0.39773
BMLF1	ACAA1	0.2382
BMLF1	DPM2	0.26344
BMLF1	GHRHR	0.12436
BMLF1	CX3CR1	0.17293
BMLF1	NTMT1	0.084859
BMLF1	PCDHA8	0.21127
BMLF1	PSMG2	0.36797
BMLF1	NDOR1	0.27962
BMLF1	PPM1A	0.10004
BMLF1	LETMD1	0.35403
BMLF1	SEC11C	0.025687
BMLF1	SYNM	0.30512
BMLF1	FOXK2	0.067335
BMLF1	PPP2R5E	0.33074
BMLF1	MTHFSD	0.018054
BMLF1	SCGN	0.30232

BMLF1	SAE1	0.19141
BMLF1	TSN	0.38594
BMLF1	VPS51	0.32957
BMLF1	CELSR3	0.44531
BMLF1	CAND1	0.30269
BMLF1	FCHSD2	0.47224
BMLF1	CATSPER1	0.2159
BMLF1	RILP	0.32777
BMLF1	ING5	0.23268
BMLF1	SENP2	0.20959
BMLF1	FOXM1	0.027435
BMLF1	BAX	0.015015
BMLF1	RCOR3	0.33802
BMLF1	SDS	0.019017
BMLF1	SLC22A18	0.042974
BMLF1	PPIP5K1	0.029278
BMLF1	MAPKAPK5	0.16766
BMLF1	LMAN1	0.14682
BMLF1	UBE2B	0.11869
BMLF1	MRPL48	0.37763
BMLF1	RAB5C	0.033773
BMLF1	SDHA	0.34201
BMLF1	NR2C2	0.12111
BMLF1	WASF1	0.29119
BMLF1	CTIF	0.4051
BMLF1	RNF115	0.32198
BMLF1	SYNJ2	0.2338
BMLF1	KIAA1524	0.41722
BMLF1	ZNRF3	0.24945
BMLF1	PLA2G2D	0.11501
BMLF1	SLC6A8	0.01557
BMLF1	TCEAL5	0.36073
BMLF1	PEX14	0.18229
BMLF1	CACNA1A	0.057941
BMLF1	AGO3	0.32109
BMLF1	ADAM10	0.037231
BMLF1	RPGRIP1	0.36477
BMLF1	SPOCK1	0.18612
BMLF1	N6AMT1	0.48504
BMLF1	POLR2E	0.15866
BMLF1	S100A5	0.19422
BMLF1	POLR2J	0.18481
BMLF1	CD4	0.10065
BMLF1	ARF1	0.14228
BMLF1	GEMIN8	0.35633
BMLF1	CERS2	0.14285
BMLF1	IGFL1	0.44416
BMLF1	NFU1	0.12343
BMLF1	PEX13	0.29474
BMLF1	EMR4P	0.17104
BMLF1	IGBP1	0.25659
BMLF1	NOD2	0.018517
BMLF1	GLI3	0.010731
BMLF1	MEOX2	0.19122
BMLF1	TFAP4	0.17225
BMLF1	UBAP2L	0.44386
BMLF1	POM121	0.022394
BMLF1	SETD8	0.28347
BMLF1	MAGEA4	0.43187
BMLF1	TAGLN2	0.42297
BMLF1	SIRT4	0.4811
BMLF1	FYTTD1	0.39098
BMLF1	DNAJB6	0.3042
BMLF1	PCDHA4	0.21127
BMLF1	CDH15	0.18224
BMLF1	FAM110A	0.40099
BMLF1	COBL	0.045807
BMLF1	LDHD	0.38134

BMLF1	GBE1	0.037613
BMLF1	PRNP	0.12227
BMLF1	TESC	0.30331
BMLF1	CLIC3	0.28547
BMLF1	TAF5	0.093783
BMLF1	CHD1	0.21838
BMLF1	RBP4	0.15109
BMLF1	HOXB9	0.17458
BMLF1	SETD7	0.32417
BMLF1	PORCN	0.023759
BMLF1	FAM161A	0.47289
BMLF1	ATPAF1	0.44394
BMLF1	DEFB129	0.080219
BMLF1	PARD3	0.16005
BMLF1	FMNL1	0.25443
BMLF1	ATP5G2	0.068194
BMLF1	UBE2Q1	0.32945
BMLF1	IFNLR1	0.38101
BMLF1	EFHB	0.26298
BMLF1	LGI1	0.27143
BMLF1	NME2	0.11576
BMLF1	KIAA0319	0.3401
BMLF1	CRY2	0.15637
BMLF1	ARL2	0.18048
BMLF1	EYS	0.26961
BMLF1	MICAL1	0.34867
BMLF1	CLVS2	0.091118
BMLF1	LSM12	0.044251
BMLF1	NDNL2	0.32734
BMLF1	ROPN1L	0.40278
BMLF1	HOXA1	0.043529
BMLF1	OBSCN	0.14466
BMLF1	UCN	0.20192
BMLF1	PCDHGB7	0.22489
BMLF1	RPA3	0.19619
BMLF1	FGFR3	0.060507
BMLF1	RWDD1	0.47224
BMLF1	TFPT	0.30056
BMLF1	CHRNA4	0.012097
BMLF1	ENPP2	0.021326
BMLF1	GC	0.013607
BMLF1	SMPDL3A	0.35543
BMLF1	RHO	0.19427
BMLF1	AGK	0.032411
BMLF1	LIG3	0.17398
BMLF1	PRDX2	0.053784
BMLF1	C12orf57	0.044236
BMLF1	HSPB6	0.40438
BMLF1	ADAMTS4	0.24783
BMLF1	SLC18B1	0.022068
BMLF1	PJA1	0.3373
BMLF1	ALKBH2	0.026639
BMLF1	TMEM8A	0.069874
BMLF1	AHNAK	0.41601
BMLF1	METTL7B	0.058034
BMLF1	PODXL	0.046145
BMLF1	CCNF	0.3157
BMLF1	RBM46	0.039667
BMLF1	HOOK3	0.20869
BMLF1	SETD1A	0.30918
BMLF1	ATP6V0B	0.012117
BMLF1	GLB1	0.19926
BMLF1	IGFL4	0.030621
BMLF1	PAK2	0.055523
BMLF1	SMARCC2	0.22686
BMLF1	CSNK2B	0.09841
BMLF1	HIC1	0.12186
BMLF1	FCN3	0.33695

BMLF1	PPFIA1	0.4165
BMLF1	TAF4	0.12462
BMLF1	PPP4R4	0.43286
BMLF1	YAE1D1	0.036547
BMLF1	DCLRE1B	0.24121
BMLF1	OSBPL1A	0.22692
BMLF1	GOPC	0.18878
BMLF1	CD82	0.41702
BMLF1	TNFRSF1A	0.12227
BMLF1	DZIP3	0.35173
BMLF1	SLC8A1	0.010037
BMLF1	RALA	0.20574
BMLF1	CD226	0.21117
BMLF1	MOB4	0.33795
BMLF1	CTF1	0.021868
BMLF1	BIN3	0.33962
BMLF1	DRG1	0.26904
BMLF1	POLR2A	0.15773
BMLF1	RPH3A	0.33605
BMLF1	SPTSSA	0.31925
BMLF1	NAA30	0.015943
BMLF1	DCP1A	0.26612
BMLF1	ADAP1	0.16075
BMLF1	AMOTL2	0.41268
BMLF1	SMARCA1	0.17909
BMLF1	SRI	0.36114
BMLF1	SELL	0.2871
BMLF1	OCM	0.23305
BMLF1	PPP4R2	0.3041
BMLF1	RTP3	0.39952
BMLF1	LRP4	0.34385
BMLF1	DPPA4	0.34975
BMLF1	RBMS2	0.010182
BMLF1	KAT6A	0.14709
BMLF1	PPP1CB	0.075939
BMLF1	CDK8	0.10777
BMLF1	PMF1	0.21541
BMLF1	BDKRB2	0.16828
BMLF1	KPNA2	0.25428
BMLF1	EVI5L	0.37122
BMLF1	NEK2	0.096086
BMLF1	RAD54L	0.24665
BMLF1	ENKUR	0.010352
BMLF1	TSC2	0.086453
BMLF1	SLC7A8	0.13655
BMLF1	PSG5	0.4438
BMLF1	PIK3R2	0.225
BMLF1	MAP3K14	0.14882
BMLF1	IMMP2L	0.055263
BMLF1	TRIM9	0.021642
BMLF1	BMX	0.15727
BMLF1	CHST7	0.099892
BMLF1	INADL	0.33511
BMLF1	RBM42	0.011483
BMLF1	MTR	0.012229
BMLF1	SEL1L	0.38499
BMLF1	GNL3L	0.30739
BMLF1	PTPN12	0.1904
BMLF1	MARCKS	0.029626
BMLF1	PLA2G12B	0.20736
BMLF1	PLA2G4B	0.062643
BMLF1	FEN1	0.10713
BMLF1	TMSB4X	0.27924
BMLF1	METTL3	0.082315
BMLF1	PCDHB12	0.21127
BMLF1	AMELY	0.015897
BMLF1	VEGFA	0.013304
BMLF1	NONO	0.28686

BMLF1	PLK1S1	0.30323
BMLF1	FLG2	0.1291
BMLF1	CUL2	0.2827
BMLF1	CCT3	0.2976
BMLF1	SRSF7	0.2977
BMLF1	MAP3K7	0.042872
BMLF1	SPATA31E1	0.19237
BMLF1	CABP5	0.22719
BMLF1	SLIT2	0.10481
BMLF1	PKD1	0.063414
BMLF1	C1orf145	0.022899
BMLF1	HERC2	0.19314
BMLF1	TBC1D5	0.33924
BMLF1	SNED1	0.24721
BMLF1	AKAP9	0.22043
BMLF1	MIB1	0.24012
BMLF1	PASK	0.21114
BMLF1	BLM	0.075058
BMLF1	ARNT	0.094838
BMLF1	SYNE3	0.30441
BMLF1	44M2.3	0.041459
BMLF1	DOCK4	0.26191
BMLF1	CELA1	0.036024
BMLF1	DIS3L2	0.062387
BMLF1	NCLN	0.29565
BMLF1	AMY2A	0.051535
BMLF1	PANK3	0.080943
BMLF1	RPL3L	0.04266
BMLF1	DMXL2	0.048365
BMLF1	KLK10	0.047742
BMLF1	SLC24A2	0.028706
BMLF1	HNRNPUL1	0.28898
BMLF1	CPA3	0.027189
BMLF1	GPRASP1	0.45783
BMLF1	MAGEE1	0.33845
BMLF1	RAD51AP1	0.40774
BMLF1	SLC22A20	0.018129
BMLF1	CNPY2	0.40477
BMLF1	NSRP1	0.35322
BMLF1	SFI1	0.36319
BMLF1	MATN2	0.52109
BMLF1	BCAR1	0.13138
BMLF1	PSMD9	0.16787
BMLF1	AXIN1	0.035166
BMLF1	SIRT6	0.28632
BMLF1	C2orf71	0.011469
BMLF1	INTS7	0.38723
BMLF1	SLC5A12	0.04937
BMLF1	STK4	0.082673
BMLF1	GPD2	0.16108
BMLF1	DHFR	0.056193
BMLF1	FBLL1	0.043224
BMLF1	SLC30A8	0.041134
BMLF1	PCDHA10	0.2049
BMLF1	CIB1	0.46749
BMLF1	IRAK2	0.10756
BMLF1	MAPK14	0.031216
BMLF1	UBR5	0.21036
BMLF1	PRRG4	0.21874
BMLF1	ANKRD44	0.47224
BMLF1	KIF13A	0.17067
BMLF1	FNTA	0.30098
BMLF1	EIF5A2	0.21048
BMLF1	MAPRE3	0.34206
BMLF1	CLCN3	0.19088
BMLF1	DNAJB2	0.30752
BMLF1	DNMT1	0.19338
BMLF1	RBM34	0.012783

BMLF1	PCDHA11	0.21127
BMLF1	PRDX5	0.019185
BMLF1	HDAC10	0.15573
BMLF1	RYR1	0.059142
BMLF1	PCDHB7	0.21802
BMLF1	TNK1	0.29293
BMLF1	PHF8	0.1786
BMLF1	GYS1	0.21107
BMLF1	PPP2R3A	0.50652
BMLF1	GSPT1	0.30326
BMLF1	DSC1	0.21881
BMLF1	PRSS27	0.049094
BMLF1	AKAP3	0.42815
BMLF1	GOLGA8I	0.034933
BMLF1	PRPF19	0.24586
BMLF1	CCDC155	0.13846
BMLF1	EIF2B4	0.26223
BMLF1	NCF1	0.096627
BMLF1	PSMC2	0.12986
BMLF1	TSSK4	0.21881
BMLF1	RBM22	0.013715
BMLF1	USE1	0.27763
BMLF1	LAPTM4B	0.39657
BMLF1	DLL1	0.32728
BMLF1	TAF2	0.2373
BMLF1	ACTN1	0.29503
BMLF1	CABP2	0.21852
BMLF1	VDR	0.11703
BMLF1	PEX11B	0.33971
BMLF1	DAZ4	0.011338
BMLF1	PMP22	0.35406
BMLF1	RAB6A	0.24132
BMLF1	C11orf54	0.36557
BMLF1	STK38	0.14821
BMLF1	TNKS2	0.2033
BMLF1	RCHY1	0.20549
BMLF1	CCND1	0.071805
BMLF1	CD164	0.26934
BMLF1	RPH3AL	0.028633
BMLF1	EPRS	0.20406
BMLF1	TBC1D15	0.34616
BMLF1	AOC3	0.20052
BMLF1	FUS	0.31719
BMLF1	IFNA16	0.010526
BMLF1	BLOC1S1	0.28388
BMLF1	NAALAD2	0.025792
BMLF1	DCTN3	0.1671
BMLF1	ARPP21	0.010482
BMLF1	PRDM9	0.019672
BMLF1	NPHP3	0.24692
BMLF1	USP46	0.34881
BMLF1	UBD	0.2721
BMLF1	MYH10	0.12368
BMLF1	CDT1	0.28678
BMLF1	SYNE1	0.23726
BMLF1	TRMT11	0.056379
BMLF1	FBXO3	0.39695
BMLF1	LRP6	0.021143
BMLF1	EXOC8	0.21082
BMLF1	KNTC1	0.22407
BMLF1	SLC5A5	0.015728
BMLF1	NCL	0.3033
BMLF1	CGGBP1	0.042769
BMLF1	ITGA1	0.24531
BMLF1	SLIRP	0.013901
BMLF1	SVEP1	0.22424
BMLF1	GTSF1	0.10978
BMLF1	NOS1	0.1602

BMLF1	EHD4	0.36737
BMLF1	STARD5	0.014631
BMLF1	APBA3	0.28944
BMLF1	RYR3	0.10953
BMLF1	SCUBE1	0.14441
BMLF1	PCDHB14	0.20505
BMLF1	HNRNPK	0.17856
BMLF1	LCAT	0.25219
BMLF1	OBFC1	0.214
BMLF1	TWIST1	0.050272
BMLF1	E2F5	0.14829
BMLF1	CSNK1E	0.17066
BMLF1	FAM124B	0.47224
BMLF1	MEF2A	0.077716
BMLF1	HTRA4	0.051231
BMLF1	PNPT1	0.12986
BMLF1	NPRL2	0.337
BMLF1	PRSS1	0.026761
BMLF1	UPF3B	0.28616
BMLF1	AKAP13	0.1734
BMLF1	RPL30	0.04147
BMLF1	ATG2A	0.47224
BMLF1	ZC3H7B	0.31618
BMLF1	LZTFL1	0.43035
BMLF1	TLN1	0.10372
BMLF1	BTRC	0.15426
BMLF1	PCDHGA11	0.22489
BMLF1	RHBDL1	0.30109
BMLF1	HNF1B	0.073846
BMLF1	IRF4	0.070544
BMLF1	TFAP2A	0.025441
BMLF1	UBE2D1	0.16333
BMLF1	GCKR	0.24907
BMLF1	MYO6	0.10369
BMLF1	C5orf58	0.044051
BMLF1	LCMT2	0.49977
BMLF1	TMPRSS2	0.035552
BMLF1	GATAD2A	0.15455
BMLF1	TGFBR1	0.019195
BMLF1	USP8	0.18546
BMLF1	DSPP	0.22355
BMLF1	GSTA2	0.041163
BMLF1	WFS1	0.013224
BMLF1	FIG4	0.19602
BMLF1	TNFSF14	0.22488
BMLF1	CDC6	0.15201
BMLF1	CDH3	0.21159
BMLF1	PLAT	0.23764
BMLF1	TAX1BP3	0.30998
BMLF1	SNX4	0.33502
BMLF1	NEGR1	0.38189
BMLF1	HTR6	0.30886
BMLF1	CUEDC2	0.40475
BMLF1	DHTKD1	0.024946
BMLF1	SEPT11	0.31966
BMLF1	PNLIPRP2	0.08255
BMLF1	METTL21EP	0.059751
BMLF1	DLGAP1	0.33959
BMLF1	CCND3	0.15716
BMLF1	MFI2	0.26927
BMLF1	FLNA	0.097241
BMLF1	FUBP3	0.035535
BMLF1	HIST1H4A	0.21235
BMLF1	CTTNBP2NL	0.45652
BMLF1	RAI2	0.43191
BMLF1	SERPINF2	0.20063
BMLF1	FBXL5	0.3333
BMLF1	SGSM3	0.32168

BMLF1	DUOX1	0.22249
BMLF1	F3	0.1221
BMLF1	CECR2	0.34976
BMLF1	PMAIP1	0.12808
BMLF1	CHST9	0.020218
BMLF1	SPINK1	0.39224
BMLF1	ABCD1	0.18243
BMLF1	CHAC1	0.31736
BMLF1	DDX3X	0.048151
BMLF1	PCDHA2	0.21127
BMLF1	SH3GLB2	0.35809
BMLF1	CHRNA3	0.013717
BMLF1	CDK16	0.19638
BMLF1	RASEF	0.20536
BMLF1	RPL10A	0.039281
BMLF1	DPP7	0.13891
BMLF1	PRSS3	0.54288
BMLF1	ANXA8	0.21378
BMLF1	CCDC169	0.027187
BMLF1	RPS6KC1	0.25438
BMLF1	PIK3C2G	0.047059
BMLF1	MAP1S	0.1764
BMLF1	BTK	0.064653
BMLF1	CLEC4G	0.43024
BMLF1	PPIL3	0.39167
BMLF1	CALML5	0.23825
BMLF1	SLC16A5	0.027392
BMLF1	LRRC4C	0.43033
BMLF1	MYL2	0.47974
BMLF1	BAIAP2	0.18609
BMLF1	KAT5	0.16715
BMLF1	PIWIL1	0.30147
BMLF1	ALOX12	0.18874
BMLF1	EDEM1	0.43862
BMLF1	SLC6A18	0.021934
BMLF1	LRP1	0.19922
BMLF1	MSH3	0.076912
BMLF1	UFL1	0.31418
BMLF1	APBA2	0.32063
BMLF1	DHDDS	0.44495
BMLF1	MKI67	0.25527
BMLF1	JADE1	0.24399
BMLF1	MTF1	0.126
BMLF1	DGAT2L7P	0.011639
BMLF1	FGFR1	0.026362
BMLF1	GEMIN2	0.29407
BMLF1	COL26A1	0.092184
BMLF1	SELE	0.18969
BMLF1	TONSL	0.30083
BMLF1	CACNB4	0.14773
BMLF1	RBM12B	0.49583
BMLF1	CTRB2	0.044034
BMLF1	CKS1B	0.34139
BMLF1	ATP10B	0.3681
BMLF1	ALDH3B1	0.04594
BMLF1	TIMM23	0.29543
BMLF1	DICER1	0.053859
BMLF1	SLC25A18	0.030525
BMLF1	HADHB	0.1599
BMLF1	APPL1	0.17882
BMLF1	CCDC92	0.42925
BMLF1	VPRBP	0.36006
BMLF1	SENP5	0.34649
BMLF1	SUV39H1	0.19951
BMLF1	COPS6	0.3472
BMLF1	SNX2	0.29447
BMLF1	MAP2K5	0.15057
BMLF1	RNF11	0.27936

BMLF1	ITGAM	0.22545
BMLF1	CDH17	0.19813
BMLF1	CDK11B	0.18158
BMLF1	EFCAB9	0.26298
BMLF1	PEX1	0.18866
BMLF1	METTL21C	0.059751
BMLF1	COPZ2	0.024432
BMLF1	SLC17A1	0.058692
BMLF1	PMVK	0.030459
BMLF1	CHKB	0.044356
BMLF1	GPX8	0.094397
BMLF1	NUDT18	0.40483
BMLF1	EXOSC2	0.31678
BMLF1	ERG	0.16278
BMLF1	ZNF622	0.27198
BMLF1	MAP2K1	0.021343
BMLF1	NFS1	0.25155
BMLF1	EEF1E1	0.31751
BMLF1	FANCD2	0.35983
BMLF1	TNNC2	0.21923
BMLF1	ATP5J	0.095348
BMLF1	GNA12	0.19099
BMLF1	ZC3H14	0.37367
BMLF1	MEPCE	0.1467
BMLF1	LSM11	0.30492
BMLF1	BUB1	0.12871
BMLF1	RIT1	0.34559
BMLF1	KCNA4	0.26737
BMLF1	RIOK3	0.3419
BMLF1	CNGB3	0.017673
BMLF1	KISS1	0.43056
BMLF1	DMC1	0.22031
BMLF1	ATP5A1	0.19289
BMLF1	RAB21	0.28859
BMLF1	TLR6	0.032809
BMLF1	SGPL1	0.10347
BMLF1	ADAR	0.24206
BMLF1	IFNGR2	0.036182
BMLF1	OIP5	0.29475
BMLF1	GAD1	0.22544
BMLF1	SLC24A4	0.027524
BMLF1	CYB561A3	0.02749
BMLF1	WDTC1	0.25802
BMLF1	SNX19	0.52308
BMLF1	STXBP5	0.10139
BMLF1	MAP2K4	0.1686
BMLF1	SNX10	0.029451
BMLF1	ASPH	0.19112
BMLF1	CREB1	0.039207
BMLF1	THUMPD2	0.12189
BMLF1	OASL	0.079359
BMLF1	LIN28B	0.286
BMLF1	TUBGCP2	0.26301
BMLF1	HIP1	0.15182
BMLF1	MBD3	0.25634
BMLF1	ITGA4	0.2584
BMLF1	FSTL1	0.21751
BMLF1	SRC	0.050522
BMLF1	MRE11A	0.14352
BMLF1	USP47	0.25768
BMLF1	CALR3	0.17531
BMLF1	CLPP	0.43705
BMLF1	CHORDC1	0.33176
BMLF1	SMG6	0.19093
BMLF1	ID1	0.13339
BMLF1	SLTM	0.01458
BMLF1	SOX10	0.072939
BMLF1	YBX2	0.018017

BMLF1	TRPC1	0.19633
BMLF1	TOM1	0.33014
BMLF1	PITPNC1	0.39367
BMLF1	PDGFRB	0.073166
BMLF1	SULF2	0.067518
BMLF1	S100A10	0.16715
BMLF1	SLC51A	0.027023
BMLF1	NDUFB10	0.36665
BMLF1	ACVRL1	0.028758
BMLF1	KIF2C	0.15844
BMLF1	CACNA1B	0.23956
BMLF1	SEPT5	0.16978
BMLF1	METTL2A	0.059751
BMLF1	PAX8	0.060381
BMLF1	APPBP2	0.3384
BMLF1	MYO1D	0.01601
BMLF1	C1QTNF3	0.26311
BMLF1	GAD2	0.1995
BMLF1	WNK1	0.24257
BMLF1	LAPTM5	0.047688
BMLF1	TCF3	0.04656
BMLF1	DACT1	0.18108
BMLF1	SV2C	0.094004
BMLF1	PCNA	0.089822
BMLF1	SLC4A10	0.032254
BMLF1	ATXN1L	0.033738
BMLF1	DDRGK1	0.45835
BMLF1	EPO	0.13465
BMLF1	IRF8	0.088712
BMLF1	RPS3	0.066562
BMLF1	CD274	0.3349
BMLF1	SLC15A5	0.047519
BMLF1	ITSN1	0.26471
BMLF1	ATP7B	0.10579
BMLF1	PTPRD	0.13845
BMLF1	RPUSD1	0.10341
BMLF1	SYT3	0.042147
BMLF1	KDM4A	0.21525
BMLF1	TRAM2	0.38464
BMLF1	SPAG9	0.074231
BMLF1	CEBPB	0.09627
BMLF1	C15orf37	0.012153
BMLF1	BRCC3	0.20884
BMLF1	BBS1	0.32515
BMLF1	SLC22A4	0.2343
BMLF1	ETV7	0.21986
BMLF1	HNRNPF	0.29289
BMLF1	CCDC141	0.47224
BMLF1	PCDHGA12	0.22489
BMLF1	ELOVL1	0.27226
BMLF1	PRDX3	0.1608
BMLF1	GK2	0.044086
BMLF1	DST	0.23025
BMLF1	SLC16A9	0.03047
BMLF1	HIF1AN	0.1711
BMLF1	COL18A1	0.18689
BMLF1	OGG1	0.13037
BMLF1	MYL6	0.56208
BMLF1	COMP	0.46755
BMLF1	RNF31	0.23572
BMLF1	CSTF3	0.033501
BMLF1	EIF3G	0.38821
BMLF1	MLLT6	0.3363
BMLF1	L1RE1	0.050463
BMLF1	CCDC47	0.55742
BMLF1	SNAP47	0.4036
BMLF1	EXOSC7	0.30629
BMLF1	LIMD1	0.15175

BMLF1	MX1	0.21092
BMLF1	SATB1	0.12574
BMLF1	NSUN2	0.041241
BMLF1	RIN1	0.36539
BMLF1	RCOR1	0.22661
BMLF1	ANXA7	0.43843
BMLF1	SON	0.28429
BMLF1	MAP3K7CL	0.41657
BMLF1	RNF139	0.237
BMLF1	PRC1	0.16564
BMLF1	SLC30A3	0.038387
BMLF1	CDH26	0.22489
BMLF1	DGUOK	0.070965
BMLF1	FRK	0.26617
BMLF1	TPH1	0.053038
BMLF1	CHP2	0.47926
BMLF1	ADNP	0.089525
BMLF1	TOX3	0.2992
BMLF1	SPG11	0.4079
BMLF1	PSMA7	0.10317
BMLF1	ZFP36L1	0.1436
BMLF1	CD97	0.14101
BMLF1	ABCC2	0.30539
BMLF1	PTPRB	0.19194
BMLF1	POM121C	0.29692
BMLF1	MUC1	0.21935
BMLF1	RANBP3	0.30928
BMLF1	CTNNA1	0.12032
BMLF1	LCN2	0.028302
BMLF1	VBP1	0.30068
BMLF1	UBE2L6	0.30875
BMLF1	ZHX3	0.1742
BMLF1	RSPO1	0.28298
BMLF1	GOLPH3	0.31077
BMLF1	IRF6	0.10403
BMLF1	PLN	0.28194
BMLF1	CDK5R1	0.093471
BMLF1	SUB1	0.2856
BMLF1	ANP32A	0.27028
BMLF1	LTBP2	0.19811
BMLF1	PPM1F	0.15079
BMLF1	SEPT4	0.11971
BMLF1	AKT3	0.15255
BMLF1	CXorf40B	0.01357
BMLF1	TEAD2	0.18356
BMLF1	CAPN13	0.14594
BMLF1	SYNGR2	0.064682
BMLF1	TRAPPC6A	0.053702
BMLF1	PLOD3	0.23679
BMLF1	CHRND	0.014842
BMLF1	SNX13	0.019167
BMLF1	IKBKB	0.057248
BMLF1	TOLLIP	0.32629
BMLF1	EIF4EBP2	0.36165
BMLF1	RUVBL1	0.13275
BMLF1	S1PR3	0.01083
BMLF1	LRP12	0.35461
BMLF1	NECAB2	0.71871
BMLF1	ELF3	0.13908
BMLF1	PROS1	0.11691
BMLF1	OTX2	0.076882
BMLF1	CACNA2D3	0.04484
BMLF1	FAM168B	0.010535
BMLF1	OGT	0.13557
BMLF1	GTF3C4	0.31692
BMLF1	PBRM1	0.28932
BMLF1	NCOR1	0.073703
BMLF1	APITD1	0.20938

BMLF1	HGF	0.17304
BMLF1	FAM103A1	0.41217
BMLF1	DNAJB9	0.31278
BMLF1	AVEN	0.4045
BMLF1	SLBP	0.24875
BMLF1	USH2A	0.24499
BMLF1	MMP16	0.08064
BMLF1	PHF2	0.22726
BMLF1	PODXL2	0.39135
BMLF1	ALDH5A1	0.03637
BMLF1	APLP1	0.13289
BMLF1	FBP1	0.14763
BMLF1	RFC2	0.20921
BMLF1	TYROBP	0.24018
BMLF1	CANX	0.30068
BMLF1	HNF1A	0.063448
BMLF1	RHOT2	0.34815
BMLF1	DOCK9	0.24711
BMLF1	RTKN	0.2771
BMLF1	EYA3	0.11479
BMLF1	KLHL3	0.2683
BMLF1	EHMT1	0.28239
BMLF1	NUP88	0.019781
BMLF1	PMP2	0.042149
BMLF1	IL1RAP	0.052678
BMLF1	KDR	0.04763
BMLF1	SNAP23	0.28827
BMLF1	MMP15	0.38169
BMLF1	MKNK1	0.2214
BMLF1	TTC8	0.31735
BMLF1	PDE6D	0.46478
BMLF1	ASCC3	0.22698
BMLF1	CCNDBP1	0.40344
BMLF1	PPP2R5B	0.26309
BMLF1	GADD45A	0.2863
BMLF1	PLCB3	0.1422
BMLF1	SNX15	0.1446
BMLF1	LCP2	0.36067
BMLF1	ORAI2	0.44386
BMLF1	SUSD1	0.24729
BMLF1	LYZL1	0.064665
BMLF1	APBA1	0.24441
BMLF1	SLC2A11	0.034805
BMLF1	DKK1	0.15741
BMLF1	ANXA11	0.37828
BMLF1	SPINK9	0.40453
BMLF1	ITM2C	0.31022
BMLF1	SLC12A8	0.030702
BMLF1	HNF4A	0.029194
BMLF1	BFSP1	0.030932
BMLF1	USP54	0.44318
BMLF1	SCN11A	0.013026
BMLF1	PPP3CB	0.19785
BMLF1	CTRB1	0.044034
BMLF1	TAB2	0.15804
BMLF1	SNRPB	0.21415
BMLF1	KRT19	0.2917
BMLF1	PCBD2	0.27593
BMLF1	S100G	0.24047
BMLF1	PRKAA1	0.033735
BMLF1	ATG7	0.17004
BMLF1	MDM4	0.23336
BMLF1	SCYL2	0.20435
BMLF1	NCKIPSD	0.40197
BMLF1	SPINK7	0.41728
BMLF1	CMTM6	0.0524
BMLF1	NQO1	0.31415
BMLF1	NFATC2	0.12589

BMLF1	TMEM248	0.048718
BMLF1	RPL12	0.043889
BMLF1	C5orf34	0.02195
BMLF1	ACSS1	0.1905
BMLF1	ACACB	0.23241
BMLF1	RBM39	0.29814
BMLF1	METTL14	0.028683
BMLF1	CDC23	0.014343
BMLF1	GMFG	0.016158
BMLF1	IGF2BP3	0.30301
BMLF1	TRIM27	0.13644
BMLF1	CSPG5	0.17063
BMLF1	MC3R	0.28014
BMLF1	ANXA5	0.090102
BMLF1	PAX2	0.03585
BMLF1	PAX6	0.010258
BMLF1	CNOT4	0.22356
BMLF1	CDK12	0.13044
BMLF1	DLG2	0.25199
BMLF1	ZNF136	0.25992
BMLF1	MAST1	0.21216
BMLF1	SCARB1	0.010474
BMLF1	SV2B	0.091218
BMLF1	MAU2	0.34512
BMLF1	GNE	0.050591
BMLF1	SH3PXD2B	0.23404
BMLF1	UBN1	0.21189
BMLF1	ALDH1A3	0.0657
BMLF1	PCSK4	0.076158
BMLF1	C17orf102	0.022729
BMLF1	IQGAP1	0.20412
BMLF1	ACRV1	0.068714
BMLF1	FAAP24	0.32677
BMLF1	HIRA	0.20462
BMLF1	ATP6V0C	0.21974
BMLF1	STAP2	0.23839
BMLF1	EPAS1	0.071954
BMLF1	OBP2A	0.011448
BMLF1	CCNA2	0.21332
BMLF1	FZD1	0.084663
BMLF1	ELAVL1	0.24527
BMLF1	RAD51	0.1318
BMLF1	BMI1	0.12346
BMLF1	OPTN	0.31447
BMLF1	TMED1	0.26398
BMLF1	C19orf60	0.47224
BMLF1	RAB9A	0.19331
BMLF1	IVD	0.028539
BMLF1	TIMP1	0.21797
BMLF1	MATN3	0.67106
BMLF1	SKA1	0.22762
BMLF1	EFCAB8	0.26298
BMLF1	ATL2	0.23664
BMLF1	ARCN1	0.022833
BMLF1	NR2F2	0.082464
BMLF1	RAP1A	0.23555
BMLF1	MAST2	0.18706
BMLF1	SLC16A3	0.01633
BMLF1	SUPT20H	0.385
BMLF1	METTL11B	0.059751
BMLF1	FABP9	0.018257
BMLF1	SH3BP2	0.31664
BMLF1	YEATS4	0.20993
BMLF1	SFN	0.17973
BMLF1	MMP13	0.068407
BMLF1	FBN3	0.25803
BMLF1	G0S2	0.36565
BMLF1	RPL27A	0.30654

BMLF1	FAM47C	0.062073
BMLF1	MDC1	0.31749
BMLF1	C10orf111	0.084808
BMLF1	PGRMC1	0.32572
BMLF1	NHP2L1	0.33012
BMLF1	SH3KBP1	0.34223
BMLF1	AMOTL1	0.37116
BMLF1	MYLIP	0.21927
BMLF1	TENC1	0.24341
BMLF1	STK3	0.12802
BMLF1	ATP13A4	0.015227
BMLF1	DLGAP4	0.36482
BMLF1	TOMM70A	0.31404
BMLF1	TCERG1	0.30905
BMLF1	PCDH8	0.16042
BMLF1	ALDH1A1	0.05337
BMLF1	UBE4A	0.37675
BMLF1	GTF3C5	0.35995
BMLF1	SLC24A3	0.010162
BMLF1	LRRK2	0.053473
BMLF1	TSC1	0.11931
BMLF1	ZAR1L	0.028393
BMLF1	CPNE6	0.22958
BMLF1	CD276	0.25242
BMLF1	RNF41	0.21636
BMLF1	RASSF2	0.39088
BMLF1	TJAP1	0.39309
BMLF1	ETV6	0.22481
BMLF1	DARS	0.27947
BMLF1	SCHIP1	0.40456
BMLF1	AQR	0.03729
BMLF1	RET	0.21548
BMLF1	OS9	0.35238
BMLF1	ARHGAP26	0.18706
BMLF1	COL1A2	0.19459
BMLF1	LSM7	0.29398
BMLF1	SNRNP35	0.011854
BMLF1	LYPD6B	0.10664
BMLF1	SRY	0.17766
BMLF1	JAM2	0.34589
BMLF1	MAFB	0.16477
BMLF1	DGKA	0.13576
BMLF1	ALOX5	0.19244
BMLF1	SOD1	0.039733
BMLF1	WASH1	0.29412
BMLF1	NDUFS2	0.088495
BMLF1	SPRED1	0.1744
BMLF1	ELAVL4	0.029707
BMLF1	PARP1	0.13106
BMLF1	TAF10	0.1135
BMLF1	CLCF1	0.26093
BMLF1	CHEK1	0.12278
BMLF1	FCRL4	0.43034
BMLF1	SLC16A1	0.020372
BMLF1	SF3B4	0.37312
BMLF1	EPS8	0.37716
BMLF1	PATL1	0.26233
BMLF1	TRAF5	0.24327
BMLF1	GPX5	0.066161
BMLF1	PARP10	0.38469
BMLF1	NSMCE4A	0.38259
BMLF1	SNTA1	0.25522
BMLF1	ATP11A	0.2475
BMLF1	IRF2	0.14046
BMLF1	CDH22	0.22489
BMLF1	THBS4	0.14249
BMLF1	FBXL3	0.3322
BMLF1	SLC4A2	0.016739

BMLF1	DDX1	0.1481
BMLF1	PNMT	0.052629
BMLF1	MC4R	0.24477
BMLF1	HEY2	0.025791
BMLF1	CDHR4	0.22489
BMLF1	CHD1L	0.29596
BMLF1	RPP25	0.37633
BMLF1	SLC3A2	0.20169
BMLF1	UBQLN1	0.29641
BMLF1	USP13	0.19617
BMLF1	SLC6A3	0.15442
BMLF1	CALCR	0.1906
BMLF1	PAQR7	0.041454
BMLF1	PTGFRN	0.38192
BMLF1	PYGM	0.017351
BMLF1	EIF4EBP3	0.37261
BMLF1	PTPN11	0.071996
BMLF1	CPSF1	0.32386
BMLF1	EFNA3	0.056729
BMLF1	MAGED2	0.010235
BMLF1	MNS1	0.057292
BMLF1	FLVCR1	0.2509
BMLF1	MS4A3	0.40456
BMLF1	HOXA5	0.07387
BMLF1	HCFC1	0.11272
BMLF1	FLNB	0.2578
BMLF1	TMEM66	0.32116
BMLF1	SMAD4	0.031278
BMLF1	PICK1	0.15793
BMLF1	HGFAC	0.04526
BMLF1	MSLN	0.35589
BMLF1	RAB11FIP5	0.2189
BMLF1	SUZ12	0.22374
BMLF1	CDH23	0.30401
BMLF1	LCA10	0.029523
BMLF1	KIT	0.041711
BMLF1	STATH	0.36258
BMLF1	UBC	0.053215
BMLF1	RAB11FIP4	0.50302
BMLF1	KCNN3	0.033445
BMLF1	RLF	0.23876
BMLF1	KRT18	0.22968
BMLF1	OPN4	0.024031
BMLF1	NOL6	0.020605
BMLF1	TRAPPC5	0.058047
BMLF1	FGF13	0.16762
BMLF1	FARSA	0.1556
BMLF1	KLC3	0.025153
BMLF1	NTM	0.33158
BMLF1	PDCD6IP	0.29275
BMLF1	GAB1	0.19952
BMLF1	HSFX1	0.23281
BMLF1	BRICD5	0.026302
BMLF1	KIAA0020	0.024139
BMLF1	RARRES2	0.34119
BMLF1	P4HA3	0.014083
BMLF1	CNGC19	0.06112
BMLF1	HSPB1	0.10356
BMLF1	BMPR1A	0.051724
BMLF1	MYCBPAP	0.34194
BMLF1	PLA2G4D	0.052548
BMLF1	ENY2	0.019048
BMLF1	LETM2	0.034376
BMLF1	MEF2D	0.15886
BMLF1	RPL4	0.025118
BMLF1	PSMA2	0.12157
BMLF1	ASXL1	0.21535
BMLF1	APBB2	0.21384

40257	BMLF1	TP53BP1	0.17245
40258	BMLF1	MAP1LC3A	0.30141
40259	BMLF1	UBXN1	0.28061
40260	BMLF1	GSTM1	0.042344
40261	BMLF1	CNRIP1	0.018539
40262	BMLF1	PRPF3	0.35821
40263	BMLF1	ATP6V1H	0.23
40264	BMLF1	UAP1L1	0.018345
40265	BMLF1	SLCO1B7	0.011458
40266	BMLF1	CDH12	0.2003
40267	BMLF1	PHYH	0.19986
40268	BMLF1	KLK14	0.041512
40269	BMLF1	JMJD6	0.13678
40270	BMLF1	RRAGA	0.23645
40271	BMLF1	HSPB2	0.35667
40272	BMLF1	EIF3A	0.27008
40273	BMLF1	MGARP	0.26852
40274	BMLF1	NFKB2	0.11271
40275	BMLF1	CASP7	0.19903
40276	BMLF1	H2AFX	0.18741
40277	BMLF1	OVCH2	0.02953
40278	BMLF1	RHOU	0.16733
40279	BMLF1	SSRP1	0.27141
40280	BMLF1	SUMO3	0.41721
40281	BMLF1	TLR2	0.053123
40282	BMLF1	ATF3	0.17272
40283	BMLF1	SOS2	0.21491
40284	BMLF1	SYNE2	0.1746
40285	BMLF1	DPYSL2	0.089265
40286	BMLF1	BCL2L2	0.35719
40287	BMLF1	SUV39H2	0.26261
40288	BMLF1	IL10RA	0.37848
40289	BMLF1	CLIC5	0.19455
40290	BMLF1	CTBP1	0.082267
40291	BMLF1	UBE2F	0.32212
40292	BMLF1	AGTR2	0.14691
40293	BMLF1	IFIH1	0.163
40294	BMLF1	PYROXD2	0.019723
40295	BMLF1	C1orf64	0.061915
40296	BMLF1	DAZ2	0.011111
40297	BMLF1	IER3	0.29588
40298	BMLF1	CNKSR1	0.27005
40299	BMLF1	LDHAL6B	0.37
40300	BMLF1	MAGEA1	0.31333
40301	BMLF1	MBOAT7	0.34933
40302	BMLF1	FOCAD	0.41774
40303	BMLF1	LOX	0.24493
40304	BMLF1	ELP4	0.26219
40305	BMLF1	ELTD1	0.17662
40306	BMLF1	CAV2	0.14527
40307	BMLF1	FXR2	0.49427
40308	BMLF1	POLR2B	0.17223
40309	BMLF1	VANGL1	0.43033
40310	BMLF1	MPP1	0.34414
40311	BMLF1	TK2	0.072338
40312	BMLF1	TRIP6	0.24323
40313	BMLF1	PKP4	0.26788
40314	BMLF1	NAA35	0.053054
40315	BMLF1	COMMD4	0.45783
40316	BMLF1	PACRGL	0.010348
40317	BMLF1	ENO1	0.086435
40318	BMLF1	AGGF1	0.32371
40319	BMLF1	ORC5	0.25501
40320	BMLF1	CLU	0.087592
40321	BMLF1	PPIB	0.3624
40322	BMLF1	PRRG1	0.23236
40323	BMLF1	NRG1	0.058819
40324	BMLF1	A1CF	0.27118

BMLF1	SLC5A3	0.045451
BMLF1	NIFK	0.34288
BMLF1	FAM69A	0.05421
BMLF1	ATXN7	0.25403
BMLF1	PABPC4L	0.039667
BMLF1	UBA6	0.27025
BMLF1	TDP2	0.24034
BMLF1	SUDS3	0.33823
BMLF1	CHCHD3	0.31064
BMLF1	PRKACB	0.08881
BMLF1	LPHN1	0.27535
BMLF1	BRINP1	0.39035
BMLF1	ETNK2	0.040508
BMLF1	WIPF2	0.43033
BMLF1	SNX5	0.12317
BMLF1	MCM2	0.16149
BMLF1	PLCE1	0.17517
BMLF1	CD163	0.37305
BMLF1	IL10RB	0.36625
BMLF1	DYNLL1	0.16297
BMLF1	PIDD	0.37149
BMLF1	ADAMTSL4	0.29122
BMLF1	MYOT	0.34532
BMLF1	PDCD1	0.34601
BMLF1	HNRNPLL	0.37663
BMLF1	SDCCAG8	0.33562
BMLF1	IL12B	0.051567
BMLF1	WDR48	0.31186
BMLF1	PDCD11	0.34374
BMLF1	G3BP1	0.25494
BMLF1	RASSF1	0.19165
BMLF1	NELFCD	0.29556
BMLF1	KHDRBS1	0.26876
BMLF1	EID1	0.26302
BMLF1	CHRNB1	0.011404
BMLF1	PCDHB4	0.17963
BMLF1	IGHMBP2	0.095519
BMLF1	SNX3	0.33406
BMLF1	OLA1	0.31809
BMLF1	ATP6V1D	0.27766
BMLF1	TDP1	0.3049
BMLF1	ABL2	0.16291
BMLF1	DOCK3	0.40431
BMLF1	STX6	0.23203
BMLF1	YIF1B	0.036573
BMLF1	BAIAP2L2	0.05526
BMLF1	DDX11	0.067905
BMLF1	MAK	0.11036
BMLF1	CYLD	0.10899
BMLF1	GP6	0.32778
BMLF1	SNW1	0.23761
BMLF1	AC3.5	0.064825
BMLF1	PPL	0.31651
BMLF1	KLK3	0.43984
BMLF1	SGIP1	0.2181
BMLF1	WAC	0.27282
BMLF1	TTR	0.37013
BMLF1	ATP2B4	0.22365
BMLF1	GLT8D2	0.015012
BMLF1	BBS7	0.30446
BMLF1	CRYAA	0.18288
BMLF1	TGM6	0.0243
BMLF1	NEDD4L	0.16012
BMLF1	PCDH9	0.21539
BMLF1	PPP6R2	0.40266
BMLF1	ITPKB	0.077715
BMLF1	HPCAL1	0.26298
BMLF1	SLC36A1	0.055977

BMLF1	MIS12	0.284
BMLF1	ARF4	0.2579
BMLF1	ZNF274	0.1731
BMLF1	DAPK1	0.2311
BMLF1	TAT	0.017083
BMLF1	RNASET2	0.080492
BMLF1	SOX6	0.10027
BMLF1	ITGA3	0.32167
BMLF1	COG3	0.22887
BMLF1	TNIK	0.16901
BMLF1	BRD7	0.16986
BMLF1	RNASE1	0.38556
BMLF1	C2orf81	0.062539
BMLF1	SF1	0.012136
BMLF1	NSF	0.18143
BMLF1	RBBP7	0.22302
BMLF1	PTRF	0.21504
BMLF1	ARIH1	0.30511
BMLF1	KCNJ16	0.012695
BMLF1	ECM1	0.21324
BMLF1	KLC2	0.31344
BMLF1	BEGAIN	0.44386
BMLF1	ARF6	0.13425
BMLF1	FAM84A	0.02373
BMLF1	PAAF1	0.41812
BMLF1	TREX2	0.053538
BMLF1	SNX21	0.16649
BMLF1	FLCN	0.3909
BMLF1	MRPL23	0.032261
BMLF1	KISS1R	0.23453
BMLF1	DNASE1L3	0.173
BMLF1	RIPK2	0.042042
BMLF1	SNRPD3	0.21201
BMLF1	INMT	0.056088
BMLF1	CLEC2A	0.41633
BMLF1	CTSB	0.10813
BMLF1	ILKAP	0.26768
BMLF1	CLIC4	0.21657
BMLF1	ASIP	0.056389
BMLF1	DBT	0.012542
BMLF1	PUS3	0.097128
BMLF1	RAB14	0.099498
BMLF1	STX12	0.25371
BMLF1	SAMHD1	0.28446
BMLF1	ACTB	0.13396
BMLF1	EIF4G1	0.28993
BMLF1	SLC22A5	0.23304
BMLF1	KIF3A	0.18505
BMLF1	SCNN1A	0.27183
BMLF1	CGREF1	0.21645
BMLF1	CACNA2D4	0.051319
BMLF1	CHAF1B	0.23166
BMLF1	RUSC1	0.28627
BMLF1	NPNT	0.17906
BMLF1	AKAP8	0.02137
BMLF1	ALPK1	0.018992
BMLF1	EZH1	0.052962
BMLF1	PEX12	0.30842
BMLF1	FLOT1	0.29446
BMLF1	FAM49B	0.042779
BMLF1	BNIP2	0.42773
BMLF1	GABARAPL2	0.29959
BMLF1	FAM199X	0.025734
BMLF1	PPIH	0.31875
BMLF1	RARS	0.18661
BMLF1	PRKDC	0.088905
BMLF1	SULF1	0.05971
BMLF1	JAG2	0.35812

BMLF1 PIP5K1C 0.016486
BMLF1 RGR 0.32502
BMLF1 MYRIP 0.30534
BMLF1 CTTN 0.39101
BMLF1 C1QA 0.38405
BMLF1 PPP1R16A 0.44387
BMLF1 EFCAB7 0.26298
BMLF1 TACC1 0.27493
BMLF1 CREB3 0.065243
BMLF1 LTBP3 0.19347
BMLF1 PAGE4 0.031857
BMLF1 PLK4 0.19975
BMLF1 SKIV2L2 0.24818
BMLF1 ADRB2 0.057957
BMLF1 OLAH 0.12882
BMLF1 DDX18 0.35986
BMLF1 ATP2B1 0.24619
BMLF1 POLR1B 0.22323
BMLF1 VIMP 0.16175
BMLF1 KMT2D 0.24124
BMLF1 CEP97 0.39833
BMLF1 PTPRU 0.16618
BMLF1 TPCN2 0.14222
BMLF1 RUNX1T1 0.18897
BMLF1 CENPI 0.2546
BMLF1 SHC3 0.33498
BMLF1 LGALS3 0.28403
BMLF1 FCGR2B 0.36047
BMLF1 DYSF 0.13381
BMLF1 IST1 0.49729
BMLF1 MAP4K5 0.25468
BMLF1 NR5A2 0.07685
BMLF1 FCHO2 0.31569
BMLF1 TIPRL 0.43722
BMLF1 SKP2 0.2664
BMLF1 PLAA 0.41711
BMLF1 NR1H4 0.061782
BMLF1 DUSP15 0.18159
BMLF1 IL1RAPL2 0.028232
BMLF1 MIB2 0.28389
BMLF1 EHD2 0.35869
BMLF1 PVRL2 0.15718
BMLF1 EIF1AD 0.076306
BMLF1 BPIFB2 0.017788
BMLF1 RBMY1C 0.011243
BMLF1 COPS5 0.25572
BMLF1 FGGY 0.12856
BMLF1 RNF135 0.31145
BMLF1 CCNE2 0.21676
BMLF1 SLC1A6 0.024584
BMLF1 C5AR2 0.32379
BMLF1 CADPS 0.47988
BMLF1 AKR1C1 0.0262
BMLF1 JUND 0.17238
BMLF1 MARCKSL1 0.021636
BMLF1 ABCA1 0.059401
BMLF1 RELN 0.014271
BMLF1 MARK2 0.15565
BMLF1 CNTROB 0.34403
BMLF1 RSL1D1 0.061123
BMLF1 CDC42SE2 0.16637
BMLF1 HEXIM2 0.29658
BMLF1 PRAMEF14 0.073446
BMLF1 HRNR 0.20276
BMLF1 PKD2 0.039439
BMLF1 AKR1E2 0.018668
BMLF1 CSTB 0.014383
BMLF1 NCAPH 0.34337

BMLF1	SOCS3	0.03136
BMLF1	CREBRF	0.17325
BMLF1	TRMT10B	0.059751
BMLF1	AURKA	0.084406
BMLF1	PABPC1L2A	0.039667
BMLF1	ATG9B	0.057303
BMLF1	PCDHGB2	0.22489
BMLF1	BBS4	0.072607
BMLF1	PDZD7	0.40347
BMLF1	PPP2CB	0.15705
BMLF1	DLC1	0.14121
BMLF1	STXBP4	0.18759
BMLF1	HS3ST1	0.020489
BMLF1	FOXC1	0.032322
BMLF1	IPO9	0.32533
BMLF1	ANXA8L1	0.22732
BMLF1	PDIA6	0.20456
BMLF1	HDAC5	0.061444
BMLF1	TRPC6	0.22365
BMLF1	ZNF655	0.24331
BMLF1	ETF1	0.33215
BMLF1	DCSTAMP	0.22111
BMLF1	EMILIN1	0.35025
BMLF1	CRYGD	0.37081
BMLF1	ADRM1	0.29646
BMLF1	KLK6	0.26422
BMLF1	FAM161B	0.028274
BMLF1	CCAR1	0.46985
BMLF1	PFDN4	0.31555
BMLF1	SLC22A2	0.0245
BMLF1	C19orf57	0.43184
BMLF1	LPCAT1	0.10099
BMLF1	HIST1H3A	0.27522
BMLF1	GSTO2	0.067002
BMLF1	CCNA1	0.2142
BMLF1	MYCN	0.16462
BMLF1	NKX2-1	0.047248
BMLF1	SPARCL1	0.24581
BMLF1	C16orf93	0.02413
BMLF1	PDZD3	0.25057
BMLF1	CD40	0.12296
BMLF1	ENPP1	0.10347
BMLF1	PPP1R12A	0.20331
BMLF1	ERC1	0.20574
BMLF1	CTNND1	0.16136
BMLF1	FABP5P3	0.045899
BMLF1	PRSS23	0.023934
BMLF1	PICALM	0.13459
BMLF1	PLEKHG6	0.22011
BMLF1	GUCA1A	0.23112
BMLF1	SEMA7A	0.27941
BMLF1	CRMP1	0.18241
BMLF1	C20orf62	0.010555
BMLF1	LITAF	0.27198
BMLF1	TGFA	0.18382
BMLF1	IQCB1	0.41995
BMLF1	CCDC171	0.026591
BMLF1	CCAR2	0.36364
BMLF1	CACTIN	0.26456
BMLF1	PLAUR	0.19327
BMLF1	UTP20	0.30674
BMLF1	AP1S3	0.060597
BMLF1	DVL1	0.076381
BMLF1	GINS2	0.36767
BMLF1	WDR66	0.26298
BMLF1	MAP3K1	0.049676
BMLF1	LOXL2	0.11653
BMLF1	CASP3	0.10863

BMLF1	SHC1	0.086282
BMLF1	MITF	0.12123
BMLF1	ZNF217	0.15282
BMLF1	PUS1	0.10116
BMLF1	MPHOSPH6	0.38164
BMLF1	COLGALT1	0.017687
BMLF1	GTF2A1	0.23053
BMLF1	NLGN4Y	0.3063
BMLF1	SLC2A2	0.084699
BMLF1	ELF1	0.20929
BMLF1	COPS3	0.36645
BMLF1	ZMYND8	0.33632
BMLF1	COMMD6	0.38319
BMLF1	LONRF3	0.37965
BMLF1	GABARAP	0.24236
BMLF1	WWP2	0.14762
BMLF1	C1orf195	0.14962
BMLF1	CYBRD1	0.020328
BMLF1	TGFB1I1	0.18312
BMLF1	PIKFYVE	0.22318
BMLF1	MEX3B	0.19588
BMLF1	FBXW9	0.47224
BMLF1	CD37	0.40469
BMLF1	STAM	0.28955
BMLF1	GSK3A	0.081117
BMLF1	CBLB	0.2395
BMLF1	SNX25	0.031811
BMLF1	GSTA1	0.041163
BMLF1	WFDC5	0.028202
BMLF1	CRELD2	0.65545
BMLF1	HEPH	0.014214
BMLF1	PPP2R5A	0.24193
BMLF1	BLOC1S2	0.23386
BMLF1	MYOZ1	0.37539
BMLF1	TMEM30A	0.40496
BMLF1	PRUNE	0.33892
BMLF1	NCOA3	0.16071
BMLF1	MUM1	0.40706
BMLF1	TIMELESS	0.23687
BMLF1	NEK9	0.15458
BMLF1	OSBPL3	0.3426
BMLF1	ACTC1	0.010987
BMLF1	SLC5A11	0.046784
BMLF1	TRMT5	0.017817
BMLF1	KCNN2	0.048772
BMLF1	NPIPA5	0.010499
BMLF1	SCAF8	0.030655
BMLF1	PTK2	0.028832
BMLF1	NIF3L1	0.363
BMLF1	SDHB	0.016512
BMLF1	MZF1	0.18493
BMLF1	SLC34A3	0.088294
BMLF1	BANF1	0.31491
BMLF1	SLC2A12	0.032096
BMLF1	KXD1	0.44262
BMLF1	CPSF6	0.32838
BMLF1	MAST3	0.28072
BMLF1	FKBP14	0.20367
BMLF1	TOX4	0.38081
BMLF1	ARHGAP44	0.36571
BMLF1	ORC1	0.22835
BMLF1	CD14	0.017882
BMLF1	LEO1	0.26074
BMLF1	CSRP2BP	0.2955
BMLF1	RORA	0.13846
BMLF1	ARL14EP	0.45783
BMLF1	CCDC22	0.044265
BMLF1	NSMCE1	0.27902

BMLF1	SLC38A3	0.054703
BMLF1	MED7	0.2585
BMLF1	CLIC6	0.064251
BMLF1	WAPAL	0.21802
BMLF1	MPST	0.038566
BMLF1	TNFSF10	0.20263
BMLF1	GP1BB	0.33586
BMLF1	REL	0.19565
BMLF1	KCNN1	0.026143
BMLF1	PCDHB10	0.20505
BMLF1	BLCAP	0.41725
BMLF1	AIMP2	0.3093
BMLF1	PUM1	0.037374
BMLF1	APOA1BP	0.36128
BMLF1	RPS7	0.21809
BMLF1	LILRB2	0.20091
BMLF1	YBR056C-B	0.040313
BMLF1	PFKFB1	0.17981
BMLF1	MNAT1	0.043955
BMLF1	EME1	0.31914
BMLF1	APPL2	0.23382
BMLF1	CCPG1	0.046662
BMLF1	RBM26	0.40863
BMLF1	PSMG1	0.43078
BMLF1	SEC61B	0.22816
BMLF1	RBMY1E	0.011243
BMLF1	KCNA1	0.0194
BMLF1	KIF2A	0.19228
BMLF1	PATL2	0.36132
BMLF1	IL7	0.22814
BMLF1	SEPT14	0.37632
BMLF1	NBR1	0.35565
BMLF1	SENP6	0.29773
BMLF1	EFCAB13	0.21413
BMLF1	HFE	0.24858
BMLF1	DPM1	0.2434
BMLF1	SF3A3	0.26488
BMLF1	MMP28	0.11294
BMLF1	IFT20	0.32344
BMLF1	GHR	0.052908
BMLF1	FBXW7	0.12598
BMLF1	SCAF4	0.010768
BMLF1	MAGEA6	0.43187
BMLF1	RAB24	0.32753
BMLF1	RAB8A	0.24276
BMLF1	PFKL	0.21444
BMLF1	CXADR	0.087737
BMLF1	C6orf10	0.04466
BMLF1	RAB11FIP2	0.23759
BMLF1	TRO	0.34493
BMLF1	ATP11C	0.24724
BMLF1	CD27	0.26855
BMLF1	LYST	0.19901
BMLF1	CASQ1	0.13783
BMLF1	SLC5A6	0.057722
BMLF1	ZNF652	0.28277
BMLF1	C1orf87	0.26298
BMLF1	CEP57	0.22781
BMLF1	SCIMP	0.36381
BMLF1	TNNT2	0.04962
BMLF1	TIPIN	0.27722
BMLF1	CHIC2	0.41772
BMLF1	BNIP3L	0.17045
BMLF1	AES	0.21248
BMLF1	STX18	0.28594
BMLF1	VAMP7	0.15948
BMLF1	N4BP2	0.37091
BMLF1	METTL7A	0.053669

BMLF1	SDC1	0.14027
BMLF1	HEPHL1	0.012446
BMLF1	TRAM1	0.27857
BMLF1	DCAF8	0.4511
BMLF1	DNM1L	0.10505
BMLF1	NSUN3	0.059751
BMLF1	PARP2	0.032468
BMLF1	DLGAP3	0.31774
BMLF1	USP9X	0.23136
BMLF1	PDIA2	0.26973
BMLF1	DERL1	0.23234
BMLF1	RSF1	0.19204
BMLF1	SMG8	0.3765
BMLF1	GSN	0.28056
BMLF1	RELB	0.19318
BMLF1	SLC11A2	0.058348
BMLF1	FAM96B	0.32796
BMLF1	TRMT112	0.4976
BMLF1	FXN	0.03448
BMLF1	NUP133	0.18067
BMLF1	OXNAD1	0.019312
BMLF1	PCDHGA4	0.21863
BMLF1	LUZP2	0.050056
BMLF1	KIAA1456	0.058034
BMLF1	PAIP2	0.40649
BMLF1	PRKD1	0.029572
BMLF1	NABP2	0.33863
BMLF1	CRNKL1	0.032363
BMLF1	GFPT1	0.047317
BMLF1	GCSAM	0.3803
BMLF1	IL23R	0.010275
BMLF1	AMBRA1	0.41919
BMLF1	ADCY4	0.15406
BMLF1	TMPRSS7	0.046359
BMLF1	SLC22A31	0.018129
BMLF1	MET	0.051622
BMLF1	TREML2	0.39307
BMLF1	PKP2	0.19237
BMLF1	SLC12A2	0.033841
BMLF1	DHRS7C	0.021124
BMLF1	VPS13A	0.33987
BMLF1	SULT1C4	0.03219
BMLF1	BMP2	0.019731
BMLF1	MME	0.12088
BMLF1	CEBPA	0.060939
BMLF1	OSBPL2	0.021189
BMLF1	PTPN22	0.12285
BMLF1	CSNK1D	0.12928
BMLF1	EXOC3L1	0.073587
BMLF1	HADHA	0.16003
BMLF1	PCDHGA3	0.22489
BMLF1	TFB2M	0.06546
BMLF1	TBKBP1	0.46443
BMLF1	PPP1R27	0.42374
BMLF1	EIF4H	0.35896
BMLF1	AK3	0.044266
BMLF1	HK1	0.24515
BMLF1	TPSB2	0.049094
BMLF1	TELO2	0.35295
BMLF1	FRMPD2	0.36754
BMLF1	CCNB3	0.25415
BMLF1	PVRL1	0.19439
BMLF1	TDRD10	0.039667
BMLF1	IGFBP3	0.19494
BMLF1	ASCL1	0.040599
BMLF1	UBA2	0.16137
BMLF1	IGFBP7	0.3634
BMLF1	SLC12A5	0.01693

BMLF1	S100A16	0.18925
BMLF1	BLNK	0.32551
BMLF1	RPGRIP1L	0.18677
BMLF1	ILF2	0.28718
BMLF1	POT1	0.21085
BMLF1	GDI1	0.32236
BMLF1	PPFIA3	0.41727
BMLF1	SF3B14	0.030605
BMLF1	BMPR1B	0.10081
BMLF1	PAM	0.17991
BMLF1	CNTRL	0.35713
BMLF1	CDK13	0.1127
BMLF1	GOLGA2	0.42389
BMLF1	PHACTR2	0.44296
BMLF1	SNG1	0.032578
BMLF1	STARD4	0.021189
BMLF1	GZMK	0.049094
BMLF1	SPACA5	0.048305
BMLF1	TMEM97	0.31219
BMLF1	TRPC5	0.26126
BMLF1	EIF4A1	0.27903
BMLF1	TDRD7	0.31618
BMLF1	MRPS12	0.32649
BMLF1	CABP1	0.34867
BMLF1	PRAP1	0.033277
BMLF1	RHNO1	0.36426
BMLF1	DDX5	0.13009
BMLF1	PALD1	0.44435
BMLF1	RBBP5	0.28057
BMLF1	CST1	0.034289
BMLF1	GYG2	0.026325
BMLF1	PILRA	0.36843
BMLF1	YBX1	0.12403
BMLF1	EAF1	0.3564
BMLF1	AKAP1	0.16345
BMLF1	SCEL	0.33607
BMLF1	SLC4A4	0.32001
BMLF1	PKN3	0.22112
BMLF1	ARF5	0.26858
BMLF1	LETM1	0.63479
BMLF1	CRH	0.18637
BMLF1	EIF2AK2	0.14402
BMLF1	WBSCR22	0.03009
BMLF1	FSTL5	0.25502
BMLF1	NDRG1	0.22301
BMLF1	CINP	0.3938
BMLF1	PLA2G10	0.064823
BMLF1	TPR	0.17488
BMLF1	KCNJ18	0.011171
BMLF1	NEFM	0.29987
BMLF1	LEMD3	0.2773
BMLF1	BAG3	0.35089
BMLF1	SPHK1	0.1594
BMLF1	TMEM98	0.046732
BMLF1	PACSIN3	0.35488
BMLF1	VAPA	0.17164
BMLF1	SCARF2	0.39724
BMLF1	CATSPER2	0.21553
BMLF1	GCA	0.24124
BMLF1	FNIP1	0.44362
BMLF1	DND1	0.010206
BMLF1	TCTEX1D2	0.47224
BMLF1	VRK2	0.16785
BMLF1	SMG1	0.21154
BMLF1	ARAF	0.19964
BMLF1	SRSF10	0.27563
BMLF1	GPC3	0.22467
BMLF1	LRRC3	0.44386

BMLF1	RFC1	0.15346
BMLF1	PPP4R1	0.37636
BMLF1	TSSK2	0.19231
BMLF1	CUTA	0.40503
BMLF1	ELL	0.26902
BMLF1	CTU1	0.39339
BMLF1	AKT2	0.037669
BMLF1	PIP5KL1	0.048532
BMLF1	PIAS4	0.19369
BMLF1	BCAS3	0.4013
BMLF1	ANGPT2	0.13641
BMLF1	MAD2L1BP	0.35929
BMLF1	ACTR1B	0.34242
BMLF1	PIK3CB	0.15865
BMLF1	RHOB	0.11529
BMLF1	EFHD2	0.26914
BMLF1	CYP4F2	0.089425
BMLF1	GRM4	0.028592
BMLF1	PFKFB3	0.011351
BMLF1	FBF1	0.34028
BMLF1	L3MBTL1	0.11267
BMLF1	MRPL12	0.39887
BMLF1	NFYC	0.1767
BMLF1	GTF2F1	0.14705
BMLF1	PKN1	0.089607
BMLF1	IL1RL1	0.23281
BMLF1	NSMAF	0.39545
BMLF1	NCAN	0.14408
BMLF1	FAM231A	0.2152
BMLF1	TFB1M	0.3642
BMLF1	NCOA5	0.36073
BMLF1	ALG1	0.062896
BMLF1	ACHE	0.087129
BMLF1	DSG3	0.19191
BMLF1	FAM124A	0.093418
BMLF1	TMEM181	0.032658
BMLF1	SLC1A3	0.017637
BMLF1	YAP1	0.12057
BMLF1	MATR3	0.27953
BMLF1	RBM18	0.039667
BMLF1	PCDHB18	0.22489
BMLF1	PPP2CA	0.092303
BMLF1	CEP164	0.30601
BMLF1	MLLT1	0.3097
BMLF1	RCC1	0.22695
BMLF1	PABPC1	0.19573
BMLF1	PLEKHF2	0.30424
BMLF1	JMY	0.27719
BMLF1	SNTB1	0.24778
BMLF1	MMP27	0.10959
BMLF1	CHST6	0.044692
BMLF1	AMER1	0.35155
BMLF1	TRIP11	0.30193
BMLF1	RTF1	0.21409
BMLF1	SLC22A13	0.012696
BMLF1	ABCC3	0.016713
BMLF1	RWDD3	0.41608
BMLF1	PRKAG1	0.092401
BMLF1	MT3	0.065889
BMLF1	LUC7L3	0.32857
BMLF1	RGN	0.12831
BMLF1	MCM5	0.24208
BMLF1	CD28	0.18829
BMLF1	CTDSP1	0.25463
BMLF1	HLA-DRB1	0.097423
BMLF1	PNRC1	0.36073
BMLF1	IRS1	0.048738
BMLF1	ITIH1	0.21867

BMLF1	NPTX1	0.043355
BMLF1	PRTN3	0.36234
BMLF1	TBX5	0.052472
BMLF1	MAL	0.32712
BMLF1	OOEP	0.063456
BMLF1	FAM188A	0.62545
BMLF1	ZP3	0.092423
BMLF1	BCKDK	0.25012
BMLF1	MCF2	0.23108
BMLF1	UMOD	0.16024
BMLF1	TRMT1	0.079225
BMLF1	C6	0.32258
BMLF1	HDHD3	0.40189
BMLF1	IPO8	0.32172
BMLF1	RNF43	0.20729
BMLF1	STX2	0.21864
BMLF1	RHBDL3	0.32067
BMLF1	AP1B1	0.30336
BMLF1	ADARB2	0.035094
BMLF1	SLC35D1	0.048367
BMLF1	CCNT1	0.14993
BMLF1	GAPDH	0.21844
BMLF1	NRD1	0.14475
BMLF1	DNM2	0.085088
BMLF1	PANK1	0.060658
BMLF1	GRB10	0.23182
BMLF1	NBN	0.10329
BMLF1	RCN3	0.69624
BMLF1	MGST1	0.16421
BMLF1	RNF5	0.2528
BMLF1	CSF1R	0.022916
BMLF1	PLSCR1	0.2904
BMLF1	CRYZ	0.023102
BMLF1	FRRS1	0.0279
BMLF1	CETN3	0.21856
BMLF1	METTL25	0.059751
BMLF1	SNX7	0.16649
BMLF1	CTNNBL1	0.34725
BMLF1	KDM2B	0.17057
BMLF1	FDXR	0.293
BMLF1	FLT1	0.065854
BMLF1	GNPNAT1	0.057467
BMLF1	BCLAF1	0.26694
BMLF1	PTGS2	0.015
BMLF1	EFTUD2	0.27184
BMLF1	ITSN2	0.11476
BMLF1	PCSK5	0.023679
BMLF1	SERPINE2	0.08684
BMLF1	NF1	0.051405
BMLF1	F8	0.30928
BMLF1	FBXO45	0.336
BMLF1	ABCB10	0.044526
BMLF1	NSG2	0.14174
BMLF1	AKR7L	0.019723
BMLF1	KIF21A	0.35982
BMLF1	IFFO1	0.048423
BMLF1	SCAMP2	0.25505
BMLF1	PCDH10	0.22489
BMLF1	GIT1	0.2823
BMLF1	CCL5	0.014872
BMLF1	GRIN2B	0.023507
BMLF1	SLC6A4	0.13145
BMLF1	CDH19	0.21805
BMLF1	VEGFC	0.18167
BMLF1	CLK2	0.22143
BMLF1	SLC12A3	0.29161
BMLF1	SNAP25	0.10639
BMLF1	SLC22A14	0.012696

BMLF1	SULT1C2	0.026998
BMLF1	DDX23	0.23192
BMLF1	LSM10	0.29625
BMLF1	SUPV3L1	0.15492
BMLF1	PSENEN	0.25898
BMLF1	ATN1	0.2432
BMLF1	LRPPRC	0.21173
BMLF1	VAPB	0.17062
BMLF1	CARM1	0.033082
BMLF1	NHEJ1	0.31093
BMLF1	ESR1	0.029161
BMLF1	DDX49	0.014902
BMLF1	STAT5B	0.047568
BMLF1	COPRS	0.32971
BMLF1	RPA1	0.1161
BMLF1	HSPG2	0.17667
BMLF1	CELSR2	0.11965
BMLF1	SMARCA4	0.12412
BMLF1	SGCA	0.19587
BMLF1	ATG5	0.27886
BMLF1	ACTL6A	0.18876
BMLF1	PAXIP1	0.23799
BMLF1	CDK5	0.011871
BMLF1	EGFR	0.031042
BMLF1	ACTR8	0.31743
BMLF1	MARCH3	0.068359
BMLF1	IREB2	0.10337
BMLF1	G3BP2	0.048782
BMLF1	CAMLG	0.3878
BMLF1	NKAPL	0.091624
BMLF1	SELP	0.13318
BMLF1	FBXO5	0.1296
BMLF1	NCKAP5L	0.010322
BMLF1	PTGDR	0.33776
BMLF1	APAF1	0.208
BMLF1	SLURP1	0.032274
BMLF1	ENTPD7	0.13361
BMLF1	SIKE1	0.37484
BMLF1	CTCF	0.0949
BMLF1	HSPD1	0.07339
BMLF1	PPFIA2	0.43031
BMLF1	PCDHGA8	0.22489
BMLF1	MMP8	0.10509
BMLF1	PRR21	0.012153
BMLF1	MED12	0.028505
BMLF1	MST1R	0.19893
BMLF1	SCIN	0.1567
BMLF1	CCDC33	0.47224
BMLF1	NPC1	0.15528
BMLF1	HTRA1	0.043481
BMLF1	SYTL5	0.37268
BMLF1	HINFP	0.10947
BMLF1	MORC4	0.37699
BMLF1	MPLKIP	0.35995
BMLF1	PLP2	0.35016
BMLF1	TLDC2	0.058803
BMLF1	RAB11A	0.18714
BMLF1	LTBP1	0.6266
BMLF1	KIF5A	0.25983
BMLF1	SHROOM1	0.01114
BMLF1	PEX6	0.30308
BMLF1	SMARCC1	0.22197
BMLF1	TPD52L2	0.44306
BMLF1	ASPSCR1	0.31085
BMLF1	RAB11FIP1	0.017781
BMLF1	ARL6IP4	0.41608
BMLF1	F2	0.18407
BMLF1	CELF1	0.27668

BMLF1	RHOXF2	0.25902
BMLF1	WDYHV1	0.3418
BMLF1	SYT7	0.22623
BMLF1	FAAP20	0.30574
BMLF1	DLG1	0.032503
BMLF1	THOC2	0.34818
BMLF1	HES1	0.021176
BMLF1	PLEKHA2	0.24795
BMLF1	DPP6	0.07137
BMLF1	EREG	0.13064
BMLF1	IFIT2	0.32486
BMLF1	HEATR1	0.32643
BMLF1	SCNN1B	0.25878
BMLF1	SMYD2	0.23817
BMLF1	ITPR1	0.14217
BMLF1	VAMP3	0.23546
BMLF1	DNAJC19	0.018009
BMLF1	CELF5	0.011251
BMLF1	WIPI2	0.28868
BMLF1	GSTM5	0.029786
BMLF1	CDH9	0.19426
BMLF1	SPAST	0.16379
BMLF1	PCDHB9	0.20505
BMLF1	RRAS	0.26424
BMLF1	MIEF2	0.34326
BMLF1	FST	0.23377
BMLF1	CEP290	0.19191
BMLF1	KDM5B	0.1349
BMLF1	NOXO1	0.27121
BMLF1	PTBP3	0.026313
BMLF1	SYT1	0.21777
BMLF1	NSMCE2	0.22344
BMLF1	MRPS16	0.3541
BMLF1	RND2	0.35603
BMLF1	HMOX2	0.25132
BMLF1	WRNIP1	0.27974
BMLF1	TCOF1	0.36165
BMLF1	CBL	0.25171
BMLF1	NEK3	0.24385
BMLF1	CORO1A	0.13824
BMLF1	DCT	0.016391
BMLF1	N4BP3	0.15372
BMLF1	METTL20	0.11408
BMLF1	PHF12	0.24371
BMLF1	SLX4	0.29654
BMLF1	CHST3	0.080521
BMLF1	ANKRD27	0.38052
BMLF1	EPHA2	0.091705
BMLF1	MYC	0.051431
BMLF1	ARFRP1	0.2781
BMLF1	DENND1A	0.03558
BMLF1	TP63	0.015931
BMLF1	UCHL5	0.16771
BMLF1	PRPF8	0.3284
BMLF1	VIM	0.12755
BMLF1	NOA1	0.28215
BMLF1	CTAG1A	0.45783
BMLF1	ACADM	0.026849
BMLF1	PEX26	0.30691
BMLF1	WARS	0.24849
BMLF1	SRXN1	0.051912
BMLF1	TIMM10	0.23232
BMLF1	SLC25A13	0.14468
BMLF1	KLRC2	0.3806
BMLF1	NPM1	0.078053
BMLF1	GDF2	0.17215
BMLF1	ABHD10	0.073421
BMLF1	NAP1L4	0.3397

BMLF1	NSUN5P1	0.050284
BMLF1	MYCBP	0.30998
BMLF1	PI4K2A	0.22957
BMLF1	GRB2	0.064881
BMLF1	ADNP2	0.15093
BMLF1	SLC38A1	0.080108
BMLF1	SHPK	0.082632
BMLF1	ZNF639	0.13424
BMLF1	TRMT10A	0.059751
BMLF1	FAM24A	0.036182
BMLF1	NCS1	0.43558
BMLF1	TCAP	0.22799
BMLF1	HPSE	0.1708
BMLF1	PIWIL3	0.02771
BMLF1	CDK2	0.061376
BMLF1	MBL2	0.23147
BMLF1	NCK2	0.23576
BMLF1	IKZF3	0.13663
BMLF1	NMNAT1	0.31771
BMLF1	GPKOW	0.38051
BMLF1	FBLN5	0.50678
BMLF1	TTLL8	0.047735
BMLF1	MPP6	0.43034
BMLF1	ARHGAP1	0.23486
BMLF1	CRBN	0.3063
BMLF1	HSCB	0.37734
BMLF1	BIRC5	0.069814
BMLF1	DAG1	0.15038
BMLF1	AP5S1	0.2948
BMLF1	MCM3	0.17684
BMLF1	TOMM20	0.23276
BMLF1	GLTSCR1	0.47224
BMLF1	OTOF	0.18415
BMLF1	AGTRAP	0.38375
BMLF1	C1orf192	0.011177
BMLF1	AP1S1	0.053943
BMLF1	SLC12A6	0.034543
BMLF1	ID2	0.090843
BMLF1	ZNF133	0.18958
BMLF1	PACS1	0.32969
BMLF1	DOC2A	0.33186
BMLF1	MED30	0.29118
BMLF1	RHOBTB3	0.32709
BMLF1	HAUS5	0.014444
BMLF1	XRCC3	0.31483
BMLF1	F11	0.32278
BMLF1	SUMF2	0.059338
BMLF1	SRGAP2	0.17307
BMLF1	CALCOCO1	0.22861
BMLF1	FBXO6	0.33092
BMLF1	VPS35	0.30036
BMLF1	RBM47	0.011722
BMLF1	HRK	0.33072
BMLF1	ADAM22	0.27063
BMLF1	THOC6	0.064594
BMLF1	PI4K2B	0.08942
BMLF1	PCDHA12	0.22489
BMLF1	TBC1D9B	0.1868
BMLF1	PTPRT	0.14071
BMLF1	SIGLEC8	0.39221
BMLF1	SNRNP40	0.30573
BMLF1	ARID1A	0.19109
BMLF1	SCNN1D	0.34008
BMLF1	STON2	0.34696
BMLF1	SNX22	0.16649
BMLF1	CENPE	0.15171
BMLF1	TMPRSS15	0.016911
BMLF1	HSPA8	0.073994

BMLF1	COPZ1	0.017468
BMLF1	PLK2	0.16036
BMLF1	FBXW11	0.23882
BMLF1	SCAI	0.30033
BMLF1	PYGB	0.081497
BMLF1	KCNMB4	0.274
BMLF1	TP53I3	0.015468
BMLF1	AHR	0.052195
BMLF1	NIPBL	0.15337
BMLF1	RPL7A	0.28815
BMLF1	KMT2A	0.12695
BMLF1	UBE2C	0.18239
BMLF1	SLC6A7	0.025145
BMLF1	DDX60L	0.014902
BMLF1	SERPINA3	0.30495
BMLF1	ADRBK1	0.079036
BMLF1	DOK1	0.23132
BMLF1	IL7R	0.21243
BMLF1	TAS2R31	0.027632
BMLF1	MYL5	0.22952
BMLF1	FAM21A	0.033858
BMLF1	TPM4	0.15598
BMLF1	NDUFAF3	0.36762
BMLF1	DYX1C1	0.35931
BMLF1	BMF	0.27311
BMLF1	INTS2	0.35129
BMLF1	RAB22A	0.25319
BMLF1	MYO7A	0.13978
BMLF1	PIAS2	0.19824
BMLF1	C5orf48	0.056993
BMLF1	MUC2	0.4043
BMLF1	ETNK1	0.040978
BMLF1	SPTLC1	0.22139
BMLF1	CCDC17	0.011412
BMLF1	NLK	0.13012
BMLF1	ESRP2	0.024692
BMLF1	ITGA6	0.29627
BMLF1	PILRB	0.4042
BMLF1	CHMP4C	0.023491
BMLF1	CTNNA2	0.17751
BMLF1	DEPTOR	0.39265
BMLF1	FTH1	0.26193
BMLF1	NOD1	0.091051
BMLF1	RCAN1	0.18791
BMLF1	DGKB	0.21622
BMLF1	STMN3	0.020455
BMLF1	B4GALNT4	0.039844
BMLF1	ZFYVE9	0.37273
BMLF1	PDS5A	0.3611
BMLF1	TIGIT	0.32627
BMLF1	RNF8	0.13702
BMLF1	DPEP1	0.16405
BMLF1	RFX5	0.23558
BMLF1	RBMX2	0.039667
BMLF1	C8orf48	0.47224
BMLF1	NPHS1	0.24014
BMLF1	TAL1	0.048242
BMLF1	SULT6B1	0.041937
BMLF1	NECAB3	0.53028
BMLF1	AKAP8L	0.33258
BMLF1	SNX12	0.41045
BMLF1	ATM	0.077896
BMLF1	SYNGR4	0.15743
BMLF1	AQP6	0.09476
BMLF1	TCHH	0.20268
BMLF1	NOP9	0.050553
BMLF1	PROM2	0.021281
BMLF1	RASSF9	0.36688

41277	BMLF1	GORASP2	0.36046
41278	BMLF1	KIR2DL4	0.369
41279	BMLF1	MLC1	0.23303
41280	BMLF1	LBP	0.13151
41281	BMLF1	ADAP2	0.12796
41282	BMLF1	TRIM16	0.24445
41283	BMLF1	IFI35	0.39293
41284	BMLF1	EDA	0.14154
41285	BMLF1	PXMP4	0.40462
41286	BMLF1	ASGR2	0.34256
41287	BMLF1	STAG1	0.28796
41288	BMLF1	EFNA4	0.44577
41289	BMLF1	GLT1D1	0.014682
41290	BMLF1	SIN3A	0.083567
41291	BMLF1	ATP5O	0.086257
41292	BMLF1	DYNC1H1	0.21458
41293	BMLF1	GRM7	0.079372
41294	BMLF1	SMIM9	0.051338
41295	BMLF1	GUK1	0.045194
41296	BMLF1	GALK2	0.085831
41297	BMLF1	CNTNAP1	0.21523
41298	BMLF1	ELAVL3	0.035396
41299	BMLF1	TMPRSS11E	0.043624
41300	BMLF1	PBXIP1	0.2957
41301	BMLF1	RRAGD	0.33127
41302	BMLF1	MAP1LC3B2	0.059227
41303	BMLF1	TRMT61B	0.44896
41304	BMLF1	CCR1	0.16744
41305	BMLF1	PLCB2	0.036593
41306	BMLF1	SDC4	0.23418
41307	BMLF1	LONP2	0.23416
41308	BMLF1	HSPA14	0.3738
41309	BMLF1	PLXNB2	0.27269
41310	BMLF1	APH1B	0.33506
41311	BMLF1	KLK12	0.047938
41312	BMLF1	SNX6	0.33248
41313	BMLF1	APOA5	0.135
41314	BMLF1	KRT1	0.27112
41315	BMLF1	ZNF143	0.22901
41316	BMLF1	BRCA1	0.044783
41317	BMLF1	RFX1	0.32834
41318	BMLF1	CHRNA5	0.016783
41319	BMLF1	CDK20	0.18768
41320	BMLF1	KRT81	0.38502
41321	BMLF1	ATIC	0.018644
41322	BMLF1	ERGIC1	0.28285
41323	BMLF1	RALB	0.25225
41324	BMLF1	CDC73	0.19387
41325	BMLF1	GSE1	0.47224
41326	BMLF1	NCOA1	0.13657
41327	BMLF1	FGA	0.28326
41328	BMLF1	RASGRP1	0.12183
41329	BMLF1	SECISBP2L	0.025416
41330	BMLF1	HLTF	0.16585
41331	BMLF1	DUSP7	0.11884
41332	BMLF1	PDE4A	0.21128
41333	BMLF1	ZFYVE1	0.28338
41334	BMLF1	IFNAR2	0.2153
41335	BMLF1	NMI	0.37315
41336	BMLF1	CLSTN3	0.15104
41337	BMLF1	CDK5RAP3	0.29005
41338	BMLF1	TRIM28	0.072604
41339	BMLF1	PCDHGB6	0.22489
41340	BMLF1	GSTT2B	0.025079
41341	BMLF1	CHMP4A	0.14411
41342	BMLF1	WWTR1	0.16753
41343	BMLF1	PCDHB11	0.19264
41344	BMLF1	ASF1A	0.2564

BMLF1	CYGB	0.019544
BMLF1	ERRFI1	0.16667
BMLF1	C19orf25	0.47224
BMLF1	IRF7	0.059704
BMLF1	MAPK9	0.13389
BMLF1	BBS2	0.19539
BMLF1	RBM44	0.087842
BMLF1	APOA2	0.05659
BMLF1	XPA	0.25025
BMLF1	CHMP5	0.34116
BMLF1	EIF2S2	0.39595
BMLF1	ZFYVE16	0.20769
BMLF1	PUS10	0.12827
BMLF1	NMRK2	0.4047
BMLF1	PPARG	0.024239
BMLF1	PHB2	0.25792
BMLF1	DCP1B	0.26783
BMLF1	FAM92A1	0.01963
BMLF1	MYB	0.14435
BMLF1	CLVS1	0.091118
BMLF1	SNRPD1	0.27096
BMLF1	SOCS1	0.19561
BMLF1	AKAP7	0.12607
BMLF1	C1S	0.22108
BMLF1	NPBWR1	0.32241
BMLF1	SNCB	0.16647
BMLF1	PYROXD1	0.019723
BMLF1	EGLN3	0.22861
BMLF1	EFNA2	0.012856
BMLF1	NPC1L1	0.37215
BMLF1	PCGF2	0.14037
BMLF1	SLC6A5	0.025983
BMLF1	DNMBP	0.32784
BMLF1	PRAM1	0.47413
BMLF1	EFNB1	0.29035
BMLF1	NT5C3B	0.35622
BMLF1	CDYL2	0.3642
BMLF1	AASDHPPT	0.35258
BMLF1	FHL1	0.23607
BMLF1	GGA3	0.29188
BMLF1	UTP11L	0.32878
BMLF1	AMER3	0.019327
BMLF1	RSRC1	0.34177
BMLF1	CNNM4	0.013909
BMLF1	MBOAT4	0.034177
BMLF1	DPPA5	0.050543
BMLF1	GLRA1	0.11565
BMLF1	CHST8	0.02672
BMLF1	AQP5	0.28789
BMLF1	LY6G6C	0.032602
BMLF1	ARL4C	0.23433
BMLF1	NR0B2	0.14751
BMLF1	ARHGAP21	0.29353
BMLF1	ARL3	0.11002
BMLF1	WDR19	0.01641
BMLF1	FCGR1B	0.046166
BMLF1	MEGF6	0.677
BMLF1	MED20	0.24347
BMLF1	RPS6KA1	0.083201
BMLF1	STAG2	0.27125
BMLF1	EIF2S3	0.34831
BMLF1	UGT2A1	0.011893
BMLF1	NFRKB	0.30186
BMLF1	CALR	0.065409
BMLF1	JPH2	0.26658
BMLF1	BTBD1	0.40295
BMLF1	SOCS5	0.34492
BMLF1	EMX2	0.15092

BMLF1	MRPL11	0.3895
BMLF1	ERMAP	0.064822
BMLF1	EGR1	0.089393
BMLF1	MC2R	0.28169
BMLF1	NUP205	0.25256
BMLF1	RAB33A	0.32926
BMLF1	DHX9	0.1895
BMLF1	HOXA10	0.18563
BMLF1	HSP90AA1	0.1938
BMLF1	TAF1	0.0962
BMLF1	GAS1	0.12332
BMLF1	C16orf45	0.022515
BMLF1	TSPY1	0.28996
BMLF1	TNFRSF14	0.2829
BMLF1	TGM4	0.025124
BMLF1	LYL1	0.31006
BMLF1	NUB1	0.3716
BMLF1	PIK3C2A	0.063105
BMLF1	NOX3	0.015333
BMLF1	NFKBIL1	0.35908
BMLF1	CBX5	0.18042
BMLF1	ZFAND2B	0.39639
BMLF1	F12	0.25155
BMLF1	ASCC2	0.0431
BMLF1	PCBD1	0.18646
BMLF1	NSUN6	0.12189
BMLF1	SYT10	0.047852
BMLF1	IFNK	0.015591
BMLF1	NRBP1	0.19658
BMLF1	SLC25A12	0.14412
BMLF1	ATF4	0.13117
BMLF1	ATP6AP2	0.34227
BMLF1	PCDHA1	0.2049
BMLF1	ANK1	0.14424
BMLF1	PCBP1	0.34815
BMLF1	HSF1	0.15867
BMLF1	UNC5B	0.32593
BMLF1	SCRIB	0.17836
BMLF1	WHSC1L1	0.028491
BMLF1	RPUSD2	0.10341
BMLF1	RPTOR	0.16304
BMLF1	CLCN7	0.030415
BMLF1	ABHD17B	0.045183
BMLF1	SRRT	0.28185
BMLF1	SSTR3	0.23408
BMLF1	DGKG	0.19885
BMLF1	MMP9	0.07879
BMLF1	PLXNB3	0.34736
BMLF1	CELA3B	0.047979
BMLF1	KCNIP2	0.30789
BMLF1	TBCD	0.27257
BMLF1	LARP1B	0.053548
BMLF1	THEG	0.37977
BMLF1	SLPI	0.34324
BMLF1	PDLIM4	0.40834
BMLF1	DAO	0.22754
BMLF1	PCDHA13	0.22489
BMLF1	C1RL	0.047596
BMLF1	ESRP1	0.025557
BMLF1	KIF3B	0.22606
BMLF1	PRSS8	0.35613
BMLF1	ALDH1L2	0.04226
BMLF1	FSCN1	0.29777
BMLF1	TRAF1	0.31298
BMLF1	CALML3	0.26298
BMLF1	PRSS33	0.047938
BMLF1	PROX1	0.031978
BMLF1	POLG2	0.27039

BMLF1	MED25	0.21683
BMLF1	RAMP3	0.23427
BMLF1	LRRC14	0.011921
BMLF1	RPL3	0.025119
BMLF1	AGO4	0.32109
BMLF1	CRK	0.24194
BMLF1	RFWD2	0.25151
BMLF1	SPOCK3	0.19801
BMLF1	PIAS3	0.18718
BMLF1	CDH4	0.17231
BMLF1	TNS1	0.36908
BMLF1	ITGA2B	0.25816
BMLF1	PAFAH1B1	0.037331
BMLF1	MAPK13	0.20364
BMLF1	SLC15A3	0.043034
BMLF1	ADAM12	0.19182
BMLF1	UBA52	0.037774
BMLF1	NDUFA10	0.028684
BMLF1	INO80E	0.3326
BMLF1	FOXL2	0.065456
BMLF1	KCNIP3	0.087202
BMLF1	SNRPN	0.3803
BMLF1	TMPRSS11D	0.040123
BMLF1	CDK14	0.16182
BMLF1	PARD6A	0.19718
BMLF1	SMARCA2	0.12003
BMLF1	PLCG1	0.12908
BMLF1	HEATR5B	0.060195
BMLF1	ACBD7	0.020988
BMLF1	FAM109B	0.32426
BMLF1	ITGAX	0.32223
BMLF1	PDCD4	0.36688
BMLF1	CTAGE15	0.012288
BMLF1	PPP1R32	0.45172
BMLF1	PPP1R8	0.25177
BMLF1	NOTCH1	0.01915
BMLF1	PCLO	0.15667
BMLF1	IL8	0.18326
BMLF1	FBN2	0.20429
BMLF1	ZNHIT1	0.43446
BMLF1	TRAF4	0.14061
BMLF1	EYA2	0.13091
BMLF1	ERCC1	0.088013
BMLF1	CARHSP1	0.025897
BMLF1	SDR42E1	0.020169
BMLF1	ELOVL2	0.26558
BMLF1	EFCAB10	0.24702
BMLF1	CD93	0.46355
BMLF1	SRR	0.1107
BMLF1	EEF1B2	0.35531
BMLF1	PPP1R2	0.38673
BMLF1	MAN1A1	0.1242
BMLF1	ADAT1	0.059171
BMLF1	CAPN12	0.14594
BMLF1	TEX11	0.34664
BMLF1	LMX1B	0.18417
BMLF1	PLA2G2F	0.10437
BMLF1	NLRC5	0.24446
BMLF1	DRD1	0.03378
BMLF1	STK36	0.11347
BMLF1	RITA1	0.31131
BMLF1	SYT8	0.072846
BMLF1	GRIN1	0.18916
BMLF1	CST3	0.098235
BMLF1	FAN1	0.27843
BMLF1	CLINT1	0.42401
BMLF1	IRF5	0.14908
BMLF1	EIF2AK3	0.088337

BMLF1	C19orf66	0.037477
BMLF1	ZBTB21	0.26588
BMLF1	MARK4	0.21496
BMLF1	BBS12	0.38634
BMLF1	DHH	0.4177
BMLF1	SLC25A4	0.17568
BMLF1	APEH	0.073186
BMLF1	EIF4E1B	0.083797
BMLF1	SPATA21	0.26298
BMLF1	NDFIP2	0.23778
BMLF1	NCOA2	0.10965
BMLF1	PIGS	0.32382
BMLF1	SIRT2	0.10712
BMLF1	IMMT	0.37516
BMLF1	UBE2L3	0.16432
BMLF1	IL5	0.29072
BMLF1	M6PR	0.032791
BMLF1	RRM1	0.27615
BMLF1	ILK	0.04682
BMLF1	FLT4	0.11883
BMLF1	MLF1	0.3063
BMLF1	GAS2L1	0.031604
BMLF1	ANXA9	0.13981
BMLF1	STRN3	0.18848
BMLF1	RAP1B	0.20962
BMLF1	IKBKG	0.10631
BMLF1	SPHK2	0.30373
BMLF1	F13A1	0.019932
BMLF1	MARK3	0.28797
BMLF1	RPS20	0.015525
BMLF1	MECP2	0.024541
BMLF1	HDAC1	0.04113
BMLF1	NR2F6	0.14375
BMLF1	VIPAS39	0.28711
BMLF1	ADAM15	0.22867
BMLF1	PRKCB	0.081361
BMLF1	PLD3	0.22449
BMLF1	PCDHGA10	0.22489
BMLF1	ADAM8	0.12727
BMLF1	TRIP12	0.30385
BMLF1	CSTF1	0.35801
BMLF1	SKA2	0.24179
BMLF1	U2AF2	0.31223
BMLF1	MAP3K8	0.19744
BMLF1	NOSTRIN	0.39636
BMLF1	ELMO1	0.2274
BMLF1	SS18	0.25975
BMLF1	U2SURP	0.42743
BMLF1	AICDA	0.19464
BMLF1	GCDH	0.024061
BMLF1	RPS9	0.19845
BMLF1	DCHS2	0.22489
BMLF1	RPLP2	0.04266
BMLF1	SNX18	0.323
BMLF1	TNFRSF13B	0.37059
BMLF1	SBDS	0.19847
BMLF1	PLCZ1	0.11278
BMLF1	N4BP2L1	0.021355
BMLF1	CDC42EP1	0.32672
BMLF1	ADRB1	0.088905
BMLF1	RPL28	0.29723
BMLF1	SIRT7	0.22218
BMLF1	AP1G2	0.29406
BMLF1	PREX1	0.16043
BMLF1	ATP2C2	0.23321
BMLF1	PCDHGC3	0.21802
BMLF1	SLC6A13	0.025267
BMLF1	PDCD10	0.29789

BMLF1	MRM1	0.19088
BMLF1	FAM231D	0.096862
BMLF1	USP44	0.25901
BMLF1	ZBTB17	0.14848
BMLF1	PURA	0.10045
BMLF1	GFI1B	0.15957
BMLF1	CCBE1	0.20485
BMLF1	PLA2G5	0.069989
BMLF1	BLMH	0.2795
BMLF1	IRAK1	0.082921
BMLF1	ERCC3	0.040532
BMLF1	CXCR4	0.11443
BMLF1	USP37	0.2121
BMLF1	ARFIP2	0.31465
BMLF1	ANAPC10	0.022024
BMLF1	SAC3D1	0.3471
BMLF1	MMS22L	0.36442
BMLF1	MTMR9	0.39741
BMLF1	SLC1A4	0.0663
BMLF1	COL4A2	0.29193
BMLF1	PKD1L2	0.2025
BMLF1	CHMP1A	0.18164
BMLF1	PPM1G	0.23063
BMLF1	GPRC5A	0.075709
BMLF1	GML	0.061575
BMLF1	XPO1	0.15452
BMLF1	ABCA3	0.021652
BMLF1	GGPS1	0.011557
BMLF1	LBR	0.29765
BMLF1	PRMT3	0.061604
BMLF1	ATP8B1	0.255
BMLF1	TJP2	0.32591
BMLF1	SLC17A6	0.072493
BMLF1	PLCD1	0.037698
BMLF1	NR1I2	0.13512
BMLF1	ITM2A	0.44386
BMLF1	RBBP4	0.19103
BMLF1	YWHAQ	0.39088
BMLF1	CD68	0.042136
BMLF1	HOOK1	0.23681
BMLF1	GTF2H2	0.059672
BMLF1	INPP5K	0.062442
BMLF1	C11orf72	0.027007
BMLF1	PPP1R3C	0.30353
BMLF1	ATP10A	0.22555
BMLF1	TSACC	0.031972
BMLF1	IQCF3	0.014605
BMLF1	ZFYVE21	0.045728
BMLF1	KLK5	0.39469
BMLF1	PRSS38	0.049094
BMLF1	MARCH5	0.19115
BMLF1	PPP1R15A	0.36132
BMLF1	CSDC2	0.018846
BMLF1	STT3A	0.35635
BMLF1	ZBTB24	0.28277
BMLF1	TCF12	0.15082
BMLF1	SLC16A4	0.028609
BMLF1	SPC24	0.29685
BMLF1	NCF2	0.12788
BMLF1	ESD	0.05379
BMLF1	FGFR2	0.014492
BMLF1	CCL3	0.062637
BMLF1	MYL6B	0.55554
BMLF1	PCK2	0.26826
BMLF1	CNIH2	0.25055
BMLF1	GLI1	0.098696
BMLF1	GIPC1	0.1786
BMLF1	RAD51C	0.24072

41685	BMLF1	NAA38	0.35757
41686	BMLF1	IGF2BP2	0.30186
41687	BMLF1	YWHAB	0.15265
41688	BMLF1	SLC25A5	0.25953
41689	BMLF1	GNA13	0.15721
41690	BMLF1	BLOC1S5	0.35544
41691	BMLF1	AP3B1	0.036147
41692	BMLF1	TRIM21	0.19823
41693	BMLF1	GNAS	0.062442
41694	BMLF1	SPTSSB	0.31925
41695	BMLF1	TAF15	0.34089
41696	BMLF1	RBX1	0.15979
41697	BMLF1	TNNI3K	0.22641
41698	BMLF1	WDR82	0.35419
41699	BMLF1	RAB3A	0.12214
41700	BMLF1	NSL1	0.26766
41701	BMLF1	BCL3	0.051839
41702	BMLF1	HMGB2	0.070052
41703	BMLF1	RHOH	0.27982
41704	BMLF1	PI4KAP2	0.11592
41705	BMLF1	KCNQ1	0.054679
41706	BMLF1	TCHP	0.34298
41707	BMLF1	GPX7	0.097277
41708	BMLF1	FFAR4	0.033261
41709	BMLF1	ATP6V0A1	0.26397
41710	BMLF1	BRPF3	0.31716
41711	BMLF1	APOL3	0.025834
41712	BMLF1	SLCO3A1	0.010833
41713	BMLF1	GTF2I	0.19407
41714	BMLF1	CPSF3	0.23906
41715	BMLF1	ATP5C1	0.038912
41716	BMLF1	KCNMA1	0.16205
41717	BMLF1	C6orf106	0.05326
41718	BMLF1	ORC6	0.31502
41719	BMLF1	TGM3	0.10481
41720	BMLF1	CELSR1	0.096094
41721	BMLF1	UBQLN2	0.39112
41722	BMLF1	PLRG1	0.34239
41723	BMLF1	CUL1	0.21387
41724	BMLF1	SCG5	0.30604
41725	BMLF1	CD9	0.24751
41726	BMLF1	DHX58	0.19255
41727	BMLF1	MYO9A	0.25123
41728	BMLF1	DNAJC17	0.060204
41729	BMLF1	SP3	0.12328
41730	BMLF1	POLI	0.2526
41731	BMLF1	SLC24A1	0.32034
41732	BMLF1	DR1	0.23274
41733	BMLF1	TBK1	0.12363
41734	BMLF1	HSPA9	0.26329
41735	BMLF1	NCSTN	0.17861
41736	BMLF1	SLC2A1	0.049295
41737	BMLF1	MFSD7	0.030646
41738	BMLF1	PLSCR2	0.22552
41739	BMLF1	GSTP1	0.14255
41740	BMLF1	MMP14	0.1312
41741	BMLF1	AFF2	0.037538
41742	BMLF1	ELP3	0.094635
41743	BMLF1	PIGK	0.21384
41744	BMLF1	CDC5L	0.28772
41745	BMLF1	MBNL1	0.26052
41746	BMLF1	MED4	0.25776
41747	BMLF1	PLA2G1B	0.027519
41748	BMLF1	RHBDD1	0.22869
41749	BMLF1	SPEF2	0.05817
41750	BMLF1	TIE1	0.25324
41751	BMLF1	FABP5	0.34715
41752	BMLF1	AP1G1	0.23965

BMLF1	HMCN2	0.26791
BMLF1	TAAR5	0.041346
BMLF1	CDHR2	0.57815
BMLF1	FAS	0.058713
BMLF1	APOE	0.021996
BMLF1	TUBB2B	0.16479
BMLF1	CMA1	0.042552
BMLF1	COQ6	0.036848
BMLF1	WAS	0.26073
BMLF1	ALDOC	0.20017
BMLF1	TFG	0.37939
BMLF1	SNRPB2	0.30583
BMLF1	TNNI3	0.16888
BMLF1	ILF3	0.24972
BMLF1	WDR61	0.25903
BMLF1	SULT2B1	0.27254
BMLF1	PAWR	0.21716
BMLF1	IL2RA	0.026436
BMLF1	SEC14L4	0.043358
BMLF1	SULT1E1	0.042507
BMLF1	ASIC2	0.30352
BMLF1	CBX3	0.20442
BMLF1	RINT1	0.33101
BMLF1	SHANK3	0.14795
BMLF1	ZDHHC11B	0.012269
BMLF1	IKBIP	0.37989
BMLF1	RCVRN	0.245
BMLF1	AGFG1	0.25909
BMLF1	CELF3	0.03488
BMLF1	SCG3	0.066627
BMLF1	C17orf62	0.44386
BMLF1	SNX17	0.269
BMLF1	CDH5	0.41322
BMLF1	EMG1	0.049507
BMLF1	GSTA4	0.062769
BMLF1	DDX58	0.10632
BMLF1	ARID1B	0.25117
BMLF1	IL6R	0.13993
BMLF1	HS3ST5	0.29753
BMLF1	CD302	0.018918
BMLF1	TP53INP2	0.33838
BMLF1	ZNF165	0.19408
BMLF1	SEMA6A	0.26361
BMLF1	ZMYND10	0.39589
BMLF1	COTL1	0.36637
BMLF1	SMN1	0.33528
BMLF1	SNIP1	0.30969
BMLF1	SYT2	0.43661
BMLF1	FSIP2	0.061184
BMLF1	PIP4K2A	0.020071
BMLF1	CCNY	0.21602
BMLF1	CAPNS1	0.49219
BMLF1	GTF2E2	0.28832
BMLF1	CHRNB3	0.015938
BMLF1	MMP12	0.066889
BMLF1	NDUFAF5	0.038567
BMLF1	KCNG3	0.27614
BMLF1	TRIM41	0.30622
BMLF1	RPL26	0.03887
BMLF1	TRMT61A	0.021345
BMLF1	KLHL9	0.011801
BMLF1	MPZL3	0.40397
BMLF1	GABARAPL1	0.37898
BMLF1	GTF2B	0.21065
BMLF1	LMOD2	0.014706
BMLF1	ANGPTL4	0.2509
BMLF1	WDR16	0.40266
BMLF1	SLC8B1	0.023168

BMLF1	IFIT1	0.30505
BMLF1	STS	0.011281
BMLF1	PCDHB15	0.21127
BMLF1	RAD18	0.20405
BMLF1	DNM1	0.16594
BMLF1	OCM2	0.26298
BMLF1	SIPA1	0.23662
BMLF1	TPD52	0.59931
BMLF1	MC5R	0.31637
BMLF1	RIPK4	0.27236
BMLF1	POLDIP3	0.37139
BMLF1	IRS2	0.10584
BMLF1	ARID5B	0.17549
BMLF1	NUFIP1	0.26915
BMLF1	KLC1	0.35026
BMLF1	MRPS31	0.36432
BMLF1	FRMPD4	0.43579
BMLF1	SLC16A8	0.024299
BMLF1	LIG4	0.084946
BMLF1	KLHDC3	0.38166
BMLF1	XRCC5	0.10002
BMLF1	CDC42BPA	0.098137
BGLF4	HPS4	0.1729
BGLF4	CBX7	0.1769
BGLF4	ACR	0.14865
BGLF4	ACVR1	0.015954
BGLF4	TAF6	0.042972
BGLF4	GPAA1	0.11472
BGLF4	TRIB3	0.048645
BGLF4	HDAC6	0.011349
BGLF4	FPR1	0.13807
BGLF4	BRAP	0.12069
BGLF4	SERPINB9	0.13537
BGLF4	SLC25A25	0.060535
BGLF4	MACROD1	0.20195
BGLF4	ITGB5	0.11465
BGLF4	HID1	0.16336
BGLF4	ECT2	0.032206
BGLF4	SHQ1	0.18459
BGLF4	PEX10	0.16059
BGLF4	NLRP2	0.13208
BGLF4	RBL1	0.096747
BGLF4	BIN2	0.23693
BGLF4	BBS10	0.15566
BGLF4	USP2	0.08291
BGLF4	MED1	0.019145
BGLF4	CDC34	0.078596
BGLF4	RGCC	0.04474
BGLF4	EFHC2	0.068073
BGLF4	CDK10	0.049388
BGLF4	EPHA3	0.051298
BGLF4	EFCAB1	0.068073
BGLF4	PIK3CD	0.06126
BGLF4	RAB7A	0.11082
BGLF4	TGFBR2	0.012712
BGLF4	N4BP1	0.18838
BGLF4	CTDNEP1	0.091779
BGLF4	FAM58A	0.13449
BGLF4	PSMD4	0.10394
BGLF4	FZD8	0.067627
BGLF4	PPP5C	0.090179
BGLF4	SLC2A4	0.10797
BGLF4	KEL	0.11739
BGLF4	IFT122	0.20719
BGLF4	NAE1	0.12508
BGLF4	PRPF6	0.16757
BGLF4	SPTA1	0.19227
BGLF4	SEC31A	0.1238

BGLF4	GZMA	0.16997
BGLF4	S100A7	0.24533
BGLF4	IL4	0.033084
BGLF4	NANOS1	0.19547
BGLF4	SLC26A8	0.15699
BGLF4	UBE2H	0.11452
BGLF4	HSPA5	0.11861
BGLF4	LDLR	0.10532
BGLF4	NEDD4	0.041618
BGLF4	LAMTOR5	0.15595
BGLF4	GSPT2	0.13093
BGLF4	SERPINB5	0.17175
BGLF4	SSBP1	0.1899
BGLF4	BIRC6	0.091036
BGLF4	SERTAD3	0.19343
BGLF4	INO80B	0.16636
BGLF4	KLK13	0.19437
BGLF4	AGO2	0.090045
BGLF4	HEPACAM2	0.13238
BGLF4	RNMTL1	0.043747
BGLF4	CENPW	0.15582
BGLF4	UNC13D	0.14772
BGLF4	UPF3A	0.20577
BGLF4	ARHGAP33	0.1626
BGLF4	RB1CC1	0.075655
BGLF4	RABEP1	0.23248
BGLF4	SKA3	0.15638
BGLF4	VPS54	0.20076
BGLF4	KIAA1377	0.24432
BGLF4	EIF5A	0.08279
BGLF4	CYS1	0.184
BGLF4	LIMK1	0.036074
BGLF4	FAF1	0.096322
BGLF4	IRF9	0.035335
BGLF4	SLC5A1	0.13679
BGLF4	LRP2BP	0.23693
BGLF4	MAPK8	0.030289
BGLF4	EDF1	0.079055
BGLF4	KCNS1	0.14829
BGLF4	PXN	0.089071
BGLF4	NUP85	0.091169
BGLF4	SOX13	0.098594
BGLF4	CTNND2	0.1518
BGLF4	UBE2D3	0.072085
BGLF4	KRT5	0.14073
BGLF4	HSD17B8	0.13475
BGLF4	BTNL8	0.22976
BGLF4	PLSCR3	0.34169
BGLF4	KREMEN1	0.20997
BGLF4	TCF25	0.092345
BGLF4	NR0B1	0.047323
BGLF4	SNX20	0.18947
BGLF4	BIRC7	0.11324
BGLF4	MCM6	0.06861
BGLF4	FKBP10	0.06649
BGLF4	KRT17	0.11927
BGLF4	MAPKAPK2	0.02343
BGLF4	NOSIP	0.13856
BGLF4	SMURF2	0.077515
BGLF4	DYM	0.17539
BGLF4	ANKIB1	0.20031
BGLF4	ATP6V0D2	0.14542
BGLF4	PTRH2	0.075496
BGLF4	ORAI3	0.22976
BGLF4	CSNK2A1	0.043799
BGLF4	PFKM	0.079049
BGLF4	SPRR2E	0.1142
BGLF4	ELAVL2	0.24953

BGLF4	RPUSD4	0.3253
BGLF4	EMR1	0.026874
BGLF4	CD248	0.065736
BGLF4	ASIC1	0.13315
BGLF4	ORC2	0.13612
BGLF4	TAF12	0.011146
BGLF4	PEX16	0.13774
BGLF4	CALN1	0.055551
BGLF4	F9	0.080543
BGLF4	CRB1	0.08217
BGLF4	TRAPPC2	0.11794
BGLF4	NIP7	0.25251
BGLF4	C16orf70	0.20864
BGLF4	USP12	0.17033
BGLF4	DDB2	0.13622
BGLF4	TUBA1B	0.035366
BGLF4	PIP5K1A	0.068854
BGLF4	PEAK1	0.076229
BGLF4	SUN1	0.13026
BGLF4	SFTPD	0.18281
BGLF4	PIP5K1B	0.15596
BGLF4	FAT3	0.056504
BGLF4	TAB3	0.090982
BGLF4	SBF2	0.12644
BGLF4	SLC7A11	0.10475
BGLF4	BNIP1	0.11256
BGLF4	BRMS1	0.16068
BGLF4	FGFR1OP	0.096179
BGLF4	PCDH20	0.058173
BGLF4	DARS2	0.070666
BGLF4	PTPRH	0.049915
BGLF4	SPRR1A	0.11055
BGLF4	CALCOCO2	0.16637
BGLF4	USHBP1	0.22271
BGLF4	CD1B	0.18659
BGLF4	LALBA	0.046075
BGLF4	BRD4	0.12849
BGLF4	EFCAB5	0.068073
BGLF4	TFAP2B	0.034511
BGLF4	PRPF4B	0.055885
BGLF4	MYF5	0.10038
BGLF4	ARHGEF12	0.071972
BGLF4	RNF2	0.085342
BGLF4	NT5C2	0.084583
BGLF4	LLGL2	0.15969
BGLF4	MYH7	0.1307
BGLF4	SORBS3	0.10464
BGLF4	POLD2	0.10586
BGLF4	SNAPIN	0.13769
BGLF4	INTS6	0.18506
BGLF4	ZDHHC8	0.022104
BGLF4	STX3	0.11533
BGLF4	PCDH12	0.046478
BGLF4	PDE2A	0.047446
BGLF4	PLXNB1	0.092069
BGLF4	CARTPT	0.11235
BGLF4	HMGB1	0.044855
BGLF4	KCNJ5	0.18732
BGLF4	MLKL	0.074365
BGLF4	MKS1	0.14936
BGLF4	HSPE1	0.14653
BGLF4	MAPK7	0.033712
BGLF4	USP11	0.15325
BGLF4	DTX1	0.092636
BGLF4	ADAMTS5	0.090528
BGLF4	RANBP9	0.15074
BGLF4	SUFU	0.060452
BGLF4	SPHKAP	0.22976

BGLF4	PXMP2	0.19129
BGLF4	SYTL1	0.17625
BGLF4	SOCS7	0.16963
BGLF4	TYRP1	0.14732
BGLF4	ZFPM1	0.036937
BGLF4	UBA1	0.10471
BGLF4	BTBD2	0.22509
BGLF4	EIF3J	0.2373
BGLF4	RTN2	0.18282
BGLF4	RP1	0.11804
BGLF4	CENPJ	0.087142
BGLF4	BCCIP	0.19103
BGLF4	F5	0.15034
BGLF4	IGSF1	0.19258
BGLF4	KCND2	0.095598
BGLF4	PTPN1	0.046721
BGLF4	FKBP1B	0.08538
BGLF4	APLP2	0.088852
BGLF4	C10orf62	0.24432
BGLF4	TERF2	0.08532
BGLF4	RNF167	0.13115
BGLF4	UNC119B	0.20689
BGLF4	RACGAP1	0.043479
BGLF4	RBL2	0.14364
BGLF4	CTBP2	0.1253
BGLF4	JAK3	0.03718
BGLF4	CCNB1IP1	0.14066
BGLF4	ACTA1	0.0898
BGLF4	PSMA1	0.066588
BGLF4	DHX30	0.18285
BGLF4	NLRX1	0.11163
BGLF4	VASP	0.13834
BGLF4	PCDHGA1	0.058173
BGLF4	CABP7	0.055551
BGLF4	CASC3	0.14232
BGLF4	WASF2	0.14588
BGLF4	CD58	0.18441
BGLF4	DUSP1	0.023474
BGLF4	MAP2	0.12365
BGLF4	TBL1XR1	0.1044
BGLF4	ZFYVE27	0.13521
BGLF4	POP7	0.1717
BGLF4	APBB1	0.042607
BGLF4	DNM3	0.050605
BGLF4	PCNT	0.1596
BGLF4	MRPL13	0.1898
BGLF4	U2AF1	0.14955
BGLF4	ARHGDIA	0.13528
BGLF4	TFDP1	0.0274
BGLF4	PDXP	0.091429
BGLF4	BOC	0.14356
BGLF4	P4HB	0.070013
BGLF4	SNRPA	0.18132
BGLF4	GAREM	0.24432
BGLF4	ELP5	0.20349
BGLF4	ALAS2	0.11836
BGLF4	IGFBP5	0.10688
BGLF4	MAN1B1	0.016852
BGLF4	ZNF675	0.093789
BGLF4	PRKCD	0.013721
BGLF4	DVL2	0.087084
BGLF4	MLH1	0.04863
BGLF4	CHRD	0.11894
BGLF4	BICD2	0.23597
BGLF4	APOB	0.021597
BGLF4	KIF7	0.13869
BGLF4	ADAM9	0.039916
BGLF4	ATP5B	0.12314

BGLF4	NIN	0.30802
BGLF4	UBB	0.031409
BGLF4	TXNDC17	0.20754
BGLF4	ARHGEF1	0.085325
BGLF4	PYCARD	0.024345
BGLF4	MUL1	0.025198
BGLF4	ITCH	0.074436
BGLF4	CEP192	0.17034
BGLF4	SYTL2	0.11554
BGLF4	MAML1	0.013576
BGLF4	PGR	0.038145
BGLF4	CARD11	0.10269
BGLF4	LMO2	0.088512
BGLF4	KCNIP1	0.26705
BGLF4	CDC27	0.12033
BGLF4	VPS26A	0.15222
BGLF4	BATF	0.10836
BGLF4	SH3BP5	0.22804
BGLF4	ZC3HC1	0.088139
BGLF4	CDH18	0.054855
BGLF4	AKTIP	0.14278
BGLF4	ANKEF1	0.068073
BGLF4	TNIP1	0.15158
BGLF4	TLE3	0.17662
BGLF4	STIM2	0.31989
BGLF4	FUNDC1	0.14433
BGLF4	FAM109A	0.17206
BGLF4	GP1BA	0.12953
BGLF4	XRN1	0.14138
BGLF4	WBP2	0.23113
BGLF4	CEACAM1	0.17177
BGLF4	NUP107	0.11517
BGLF4	ERBB3	0.03431
BGLF4	ARL11	0.19777
BGLF4	MEGF9	0.061899
BGLF4	CBLC	0.012014
BGLF4	SLAIN2	0.22382
BGLF4	SSSCA1	0.21467
BGLF4	CCDC130	0.23111
BGLF4	BNIPL	0.15917
BGLF4	KRT7	0.10669
BGLF4	ZHX2	0.057062
BGLF4	CTCFL	0.11726
BGLF4	RPLP1	0.17554
BGLF4	DERL3	0.18334
BGLF4	KIAA1045	0.048404
BGLF4	SERGEF	0.17004
BGLF4	COG2	0.14324
BGLF4	CD247	0.14987
BGLF4	DSN1	0.14071
BGLF4	ELK1	0.068089
BGLF4	TRAF2	0.033929
BGLF4	TCEA2	0.12221
BGLF4	NKAP	0.14543
BGLF4	FIS1	0.052254
BGLF4	TMED10	0.1179
BGLF4	RALBP1	0.16816
BGLF4	NDUFAF4	0.24269
BGLF4	BUB1B	0.026984
BGLF4	CST5	0.25643
BGLF4	NFKBIB	0.11316
BGLF4	THOC5	0.16151
BGLF4	NAIP	0.13831
BGLF4	DPY30	0.15499
BGLF4	EFCAB14	0.068073
BGLF4	PVRL3	0.15969
BGLF4	LRIF1	0.21819
BGLF4	TNFAIP3	0.018579

BGLF4	PLCB4	0.017983
BGLF4	TNKS	0.064318
BGLF4	TMEM30B	0.22281
BGLF4	PYDC1	0.15866
BGLF4	PITPNM2	0.042193
BGLF4	SOX4	0.02419
BGLF4	NCAPD2	0.15136
BGLF4	MIA3	0.16753
BGLF4	UHRF2	0.08421
BGLF4	CGA	0.13277
BGLF4	PDIA3	0.067816
BGLF4	BIRC3	0.10636
BGLF4	PABPN1	0.12193
BGLF4	TAF3	0.12173
BGLF4	COMMD5	0.2066
BGLF4	ACAN	0.12857
BGLF4	CLASP2	0.065087
BGLF4	NACA	0.16006
BGLF4	WIPF1	0.23421
BGLF4	SPTAN1	0.2323
BGLF4	NDE1	0.068957
BGLF4	BECN1	0.11445
BGLF4	PTPN3	0.054994
BGLF4	FZD5	0.013274
BGLF4	CD81	0.12153
BGLF4	CDC37	0.12802
BGLF4	NOL8	0.20241
BGLF4	PPM1E	0.08472
BGLF4	SPNS1	0.2002
BGLF4	RIMS1	0.11272
BGLF4	ATRIP	0.20736
BGLF4	COPS2	0.16003
BGLF4	NDUFAB1	0.038213
BGLF4	DDR1	0.044948
BGLF4	COL13A1	0.13309
BGLF4	RAD52	0.17636
BGLF4	TCF7	0.046484
BGLF4	LAMTOR1	0.13325
BGLF4	DHPS	0.21566
BGLF4	IL12A	0.070772
BGLF4	SMC3	0.072713
BGLF4	SNX24	0.026854
BGLF4	STAT5A	0.038691
BGLF4	THAP1	0.13158
BGLF4	TUBA3C	0.046595
BGLF4	CDK5RAP2	0.069633
BGLF4	PRX	0.16935
BGLF4	MCRS1	0.1543
BGLF4	POP4	0.19082
BGLF4	TARBP1	0.014841
BGLF4	FBLN1	0.064417
BGLF4	EDIL3	0.058233
BGLF4	AIMP1	0.13431
BGLF4	MASP1	0.34365
BGLF4	TBL1X	0.060756
BGLF4	AIF1	0.017293
BGLF4	ZNF205	0.15166
BGLF4	INPP5D	0.069391
BGLF4	GCC2	0.11352
BGLF4	THRB	0.026957
BGLF4	SCUBE3	0.415
BGLF4	CALML6	0.010117
BGLF4	KIF13B	0.087443
BGLF4	SLC9A7	0.12594
BGLF4	CNOT3	0.15476
BGLF4	PTPRM	0.047028
BGLF4	BCL9	0.17394
BGLF4	NGFR	0.051863

BGLF4	TADA3	0.052056
BGLF4	STRAP	0.11797
BGLF4	PROZ	0.033711
BGLF4	LSM3	0.17255
BGLF4	DDO	0.13393
BGLF4	PEA15	0.18822
BGLF4	RPS27L	0.13643
BGLF4	RBFOX1	0.19458
BGLF4	AMIGO2	0.17285
BGLF4	MTSS1	0.092598
BGLF4	PSMC3	0.052093
BGLF4	HUWE1	0.11875
BGLF4	PPP1R26	0.18607
BGLF4	UXT	0.11483
BGLF4	TRAPPC4	0.2138
BGLF4	TRIM17	0.14475
BGLF4	HECTD3	0.11917
BGLF4	TH	0.023479
BGLF4	TNFRSF10B	0.14704
BGLF4	SCAP	0.13174
BGLF4	C1QB	0.18871
BGLF4	TRIM13	0.086617
BGLF4	FBXO7	0.17261
BGLF4	HIPK1	0.035199
BGLF4	CAMK2A	0.034063
BGLF4	CEP152	0.093528
BGLF4	APOH	0.099209
BGLF4	HEXIM1	0.17168
BGLF4	PTPRS	0.060314
BGLF4	ATXN10	0.16244
BGLF4	SCN5A	0.060897
BGLF4	GEMIN5	0.19793
BGLF4	COMMD7	0.20457
BGLF4	FHIT	0.095085
BGLF4	ADAM17	0.027338
BGLF4	CSNK1A1	0.027938
BGLF4	ATG4B	0.18026
BGLF4	TERF1	0.0669
BGLF4	MAL2	0.19842
BGLF4	EEF1A1	0.039511
BGLF4	PCDHGB1	0.058173
BGLF4	TDG	0.075453
BGLF4	AGTR1	0.065984
BGLF4	SEC24B	0.10977
BGLF4	ALYREF	0.14586
BGLF4	KPNA1	0.13492
BGLF4	SERINC1	0.13909
BGLF4	AATK	0.066024
BGLF4	NCF4	0.14245
BGLF4	MED8	0.15016
BGLF4	ATG16L1	0.1897
BGLF4	USP19	0.10758
BGLF4	RIN3	0.22047
BGLF4	LDOC1	0.20837
BGLF4	FSBP	0.20561
BGLF4	SH3D19	0.13689
BGLF4	TNS4	0.19755
BGLF4	MORF4L2	0.18108
BGLF4	TSR2	0.23692
BGLF4	SAV1	0.14152
BGLF4	EPB41	0.06568
BGLF4	CDH16	0.058173
BGLF4	PDE9A	0.10143
BGLF4	PEX19	0.12899
BGLF4	SYNCRIP	0.11324
BGLF4	NAGK	0.19263
BGLF4	SLC22A6	0.14996
BGLF4	BMP7	0.021796

BGLF4	PPAP2B	0.049919
BGLF4	RPS6	0.042982
BGLF4	TIA1	0.18418
BGLF4	SNRPG	0.13584
BGLF4	TP53RK	0.075877
BGLF4	PCDH18	0.056505
BGLF4	TECPR1	0.19043
BGLF4	RRM2	0.1327
BGLF4	CD300LD	0.22282
BGLF4	KCNJ10	0.071349
BGLF4	CUL4A	0.15806
BGLF4	CD244	0.19251
BGLF4	GATA4	0.011047
BGLF4	KCNH1	0.11735
BGLF4	UFC1	0.22977
BGLF4	PAIP2B	0.22278
BGLF4	PTPRN	0.11408
BGLF4	KAT7	0.10113
BGLF4	PML	0.025164
BGLF4	RPGR	0.16837
BGLF4	KBTBD6	0.22414
BGLF4	WDR5	0.13823
BGLF4	GOLGA4	0.15879
BGLF4	MALL	0.17904
BGLF4	CHMP4B	0.18105
BGLF4	TLK2	0.065904
BGLF4	NLRC4	0.076453
BGLF4	PROC	0.2977
BGLF4	PDCD6	0.33149
BGLF4	DKC1	0.18183
BGLF4	USP32	0.05154
BGLF4	OTUD7B	0.12607
BGLF4	COG1	0.16591
BGLF4	S100A9	0.11413
BGLF4	ANXA2	0.19456
BGLF4	TMPO	0.17853
BGLF4	PSMD2	0.096484
BGLF4	MMP20	0.24916
BGLF4	RAET1G	0.20294
BGLF4	DAZL	0.14167
BGLF4	NR2C2AP	0.19658
BGLF4	MCM4	0.079017
BGLF4	RTN4	0.059639
BGLF4	RUNX1	0.062758
BGLF4	TOP3A	0.10833
BGLF4	CDKN1A	0.048111
BGLF4	KIF23	0.092366
BGLF4	IL13RA1	0.21267
BGLF4	EZH2	0.12123
BGLF4	MATN4	0.43782
BGLF4	SLX4IP	0.24432
BGLF4	POLG	0.15152
BGLF4	NOLC1	0.012594
BGLF4	PSMF1	0.096814
BGLF4	FMR1	0.23223
BGLF4	MAP1A	0.086897
BGLF4	NOTCH3	0.18253
BGLF4	DNMT3L	0.13223
BGLF4	VPS72	0.18012
BGLF4	TIMM50	0.083332
BGLF4	CXXC1	0.1312
BGLF4	PVALB	0.069762
BGLF4	RNH1	0.41053
BGLF4	LRRC47	0.22286
BGLF4	FOXRED2	0.25993
BGLF4	PKM	0.069699
BGLF4	ARHGEF6	0.088249
BGLF4	PIK3C2B	0.10748

BGLF4	MFF	0.10086
BGLF4	MMRN2	0.22073
BGLF4	FBXL12	0.22281
BGLF4	THOC7	0.21856
BGLF4	EGFL7	0.045793
BGLF4	POR	0.029104
BGLF4	ASPN	0.072738
BGLF4	MYL1	0.053773
BGLF4	ARL4D	0.11769
BGLF4	NUMA1	0.068851
BGLF4	MED23	0.14389
BGLF4	ADIPOQ	0.018949
BGLF4	TBC1D24	0.17297
BGLF4	HLA-DRB1	0.049262
BGLF4	MED22	0.15491
BGLF4	TGFB3	0.032537
BGLF4	KIR2DL3	0.15553
BGLF4	EGLN1	0.095392
BGLF4	PLXNC1	0.13714
BGLF4	SIAH1	0.063915
BGLF4	FSCB	0.058136
BGLF4	C2CD5	0.062546
BGLF4	SNX27	0.15044
BGLF4	PTPRJ	0.028965
BGLF4	NDUFA9	0.13662
BGLF4	PVRL4	0.18138
BGLF4	PPP6R1	0.18758
BGLF4	FRS2	0.094721
BGLF4	MEF2BNB	0.24365
BGLF4	SPSB2	0.20775
BGLF4	ROBO1	0.080729
BGLF4	PAIP1	0.19362
BGLF4	WRAP53	0.25193
BGLF4	MORF4L1	0.16202
BGLF4	SELPLG	0.13639
BGLF4	CLIP1	0.17344
BGLF4	ATP1A1	0.066038
BGLF4	BBS9	0.17606
BGLF4	YTHDF3	0.24432
BGLF4	HCK	0.031826
BGLF4	USP6	0.12108
BGLF4	TEK	0.023885
BGLF4	TAP2	0.051927
BGLF4	UGP2	0.10141
BGLF4	EXOSC5	0.15161
BGLF4	UBXN7	0.18007
BGLF4	TSHR	0.14777
BGLF4	NECAB1	0.010117
BGLF4	PLXDC2	0.22976
BGLF4	BLOC1S3	0.12008
BGLF4	AMOT	0.12757
BGLF4	MAATS1	0.22982
BGLF4	TBC1D8	0.03526
BGLF4	THY1	0.019767
BGLF4	ALOX5AP	0.099859
BGLF4	DLK2	0.080107
BGLF4	BHLHE40	0.052889
BGLF4	TTLL6	0.13033
BGLF4	SLC4A1	0.13448
BGLF4	PTK7	0.059688
BGLF4	ADI1	0.12795
BGLF4	PCDHB5	0.053256
BGLF4	SPAG5	0.10808
BGLF4	TOB1	0.19465
BGLF4	MYOD1	0.014853
BGLF4	BORA	0.13884
BGLF4	WDR12	0.147
BGLF4	HM13	0.12325

BGLF4	JUNB	0.030377
BGLF4	SLC25A17	0.11519
BGLF4	VPS28	0.17065
BGLF4	URM1	0.20402
BGLF4	NUF2	0.1372
BGLF4	CABS1	0.057223
BGLF4	KLF10	0.045978
BGLF4	MAGEA2	0.17005
BGLF4	TRAK2	0.098207
BGLF4	CASP8AP2	0.14584
BGLF4	MZT2B	0.19678
BGLF4	FNTB	0.12233
BGLF4	RNF7	0.1344
BGLF4	HNRNPR	0.16734
BGLF4	CHFR	0.097321
BGLF4	CLN3	0.032993
BGLF4	CRTC1	0.14898
BGLF4	QKI	0.19685
BGLF4	RTN4R	0.17507
BGLF4	RBM15	0.18332
BGLF4	STXBP2	0.12979
BGLF4	KSR1	0.010756
BGLF4	POLR1A	0.13165
BGLF4	EPCAM	0.18978
BGLF4	UMODL1	0.052855
BGLF4	GNL3	0.12455
BGLF4	VPS33B	0.13717
BGLF4	CLPX	0.11187
BGLF4	UACA	0.12189
BGLF4	GLS	0.12691
BGLF4	PAK3	0.024064
BGLF4	C5orf30	0.18315
BGLF4	MYL4	0.054216
BGLF4	CD8B	0.15211
BGLF4	EIF4G2	0.24103
BGLF4	TOM1L1	0.15876
BGLF4	RABGGTB	0.20742
BGLF4	COLQ	0.12034
BGLF4	ARHGEF7	0.027129
BGLF4	NPAT	0.078468
BGLF4	ORAI1	0.149
BGLF4	RAD21	0.11497
BGLF4	MAPT	0.037208
BGLF4	CD80	0.14002
BGLF4	PCDH11Y	0.058173
BGLF4	METTL1	0.18532
BGLF4	MRFAP1	0.20224
BGLF4	MMS19	0.1222
BGLF4	ITGB3	0.041062
BGLF4	INSR	0.012414
BGLF4	TLN2	0.074478
BGLF4	TRABD2B	0.1032
BGLF4	ASB9	0.18527
BGLF4	TRPC3	0.12361
BGLF4	GATA2	0.011706
BGLF4	CIZ1	0.1491
BGLF4	CHD8	0.072504
BGLF4	RPL11	0.11942
BGLF4	NLRP1	0.088552
BGLF4	NCBP1	0.10162
BGLF4	HDAC4	0.011799
BGLF4	EDC3	0.21015
BGLF4	MAD2L2	0.15182
BGLF4	MGRN1	0.090453
BGLF4	CEP76	0.18566
BGLF4	WDR77	0.13047
BGLF4	CRKL	0.16315
BGLF4	USP7	0.075099

BGLF4	PPP2R2B	0.11115
BGLF4	BGLAP	0.053666
BGLF4	TIMP3	0.15299
BGLF4	RPL13	0.17554
BGLF4	ALDH7A1	0.16429
BGLF4	GDI2	0.14325
BGLF4	PTK6	0.03555
BGLF4	ZRSR2	0.14199
BGLF4	CD200R1	0.19107
BGLF4	UHRF1	0.059495
BGLF4	SULT2A1	0.16578
BGLF4	NEBL	0.19978
BGLF4	KLKB1	0.17523
BGLF4	TMF1	0.15546
BGLF4	IL1R1	0.15519
BGLF4	LATS2	0.036432
BGLF4	MTA2	0.015671
BGLF4	CREB3L3	0.082476
BGLF4	NCOA7	0.16895
BGLF4	PCDHA5	0.05466
BGLF4	RABGAP1	0.1492
BGLF4	SMARCD1	0.14857
BGLF4	SHROOM2	0.098846
BGLF4	ZNF148	0.045042
BGLF4	PCDH11X	0.054729
BGLF4	KBTBD7	0.22414
BGLF4	ERCC4	0.058866
BGLF4	CYBA	0.053209
BGLF4	AKT1S1	0.12043
BGLF4	LTBR	0.1481
BGLF4	ARHGAP8	0.17669
BGLF4	MYOCD	0.03923
BGLF4	UHRF1BP1	0.21166
BGLF4	GH1	0.12643
BGLF4	CTH	0.058797
BGLF4	SHANK2	0.20255
BGLF4	CDH20	0.056464
BGLF4	SLA2	0.19704
BGLF4	CLTC	0.11091
BGLF4	XRCC6	0.021024
BGLF4	PPARA	0.016504
BGLF4	GPIHBP1	0.058804
BGLF4	FCGR1A	0.16326
BGLF4	SH3GL1	0.21334
BGLF4	ARVCF	0.18391
BGLF4	CBX2	0.14882
BGLF4	PLEKHA5	0.13905
BGLF4	FOXD3	0.01648
BGLF4	EIF3F	0.20909
BGLF4	PPP3R2	0.068073
BGLF4	CIITA	0.11044
BGLF4	UPF2	0.2271
BGLF4	CACNB2	0.14038
BGLF4	SIN3B	0.14723
BGLF4	TNPO3	0.15133
BGLF4	CACNA1C	0.10537
BGLF4	SIK1	0.0114
BGLF4	SH3RF1	0.10947
BGLF4	TRPM6	0.087983
BGLF4	RAB5A	0.11075
BGLF4	ERBB2IP	0.11711
BGLF4	LPCAT2	0.02933
BGLF4	OPA1	0.076747
BGLF4	PPP2R5D	0.15432
BGLF4	BIN1	0.12608
BGLF4	BCL2L11	0.034965
BGLF4	EIF4ENIF1	0.14314
BGLF4	SYNRG	0.055832

BGLF4	GNAI1	0.062639
BGLF4	HIST2H3A	0.13386
BGLF4	NRIP1	0.065219
BGLF4	PSMB9	0.055111
BGLF4	RBMY1A1	0.22263
BGLF4	EEF1G	0.16668
BGLF4	COL7A1	0.16911
BGLF4	RAG1	0.059596
BGLF4	RRAD	0.16848
BGLF4	STX4	0.098858
BGLF4	LOXL4	0.1709
BGLF4	GFI1	0.087212
BGLF4	SIK3	0.052735
BGLF4	ROBO2	0.097611
BGLF4	CALCA	0.066758
BGLF4	NUP214	0.13907
BGLF4	NAA50	0.25605
BGLF4	STAU1	0.18089
BGLF4	YKT6	0.013141
BGLF4	CHAMP1	0.099267
BGLF4	ARHGEF15	0.1614
BGLF4	TPCN1	0.10928
BGLF4	MERTK	0.050932
BGLF4	MRPL20	0.15412
BGLF4	SRF	0.01082
BGLF4	UBL4A	0.2009
BGLF4	CREBZF	0.088334
BGLF4	PELP1	0.19708
BGLF4	NFKB1	0.01603
BGLF4	STAT3	0.025839
BGLF4	ZNF488	0.13844
BGLF4	ACIN1	0.15127
BGLF4	BARD1	0.11798
BGLF4	PPFIA4	0.21613
BGLF4	CKB	0.12228
BGLF4	TNFSF12	0.098531
BGLF4	TNS3	0.2102
BGLF4	FAM160A2	0.20361
BGLF4	KMT2C	0.17514
BGLF4	KLHL20	0.13062
BGLF4	RAD23A	0.13834
BGLF4	SMG9	0.17576
BGLF4	HPCAL4	0.073284
BGLF4	VPS4A	0.094743
BGLF4	COCH	0.18134
BGLF4	PSPC1	0.20586
BGLF4	STK40	0.058854
BGLF4	SRPX2	0.13708
BGLF4	ORC4	0.10901
BGLF4	THBD	0.25203
BGLF4	GLYCTK	0.18164
BGLF4	ARHGEF4	0.077734
BGLF4	SMAD5	0.037657
BGLF4	KIAA0226	0.17737
BGLF4	DCHS1	0.056436
BGLF4	CREM	0.074998
BGLF4	ZFR	0.20602
BGLF4	RTP4	0.20555
BGLF4	FSHR	0.103
BGLF4	FANCM	0.11245
BGLF4	EEF1D	0.15498
BGLF4	TRADD	0.07246
BGLF4	PYGO1	0.14123
BGLF4	DHX8	0.1528
BGLF4	MTERF	0.16956
BGLF4	ATG13	0.12
BGLF4	BRAF	0.063972
BGLF4	NPBWR2	0.17298

BGLF4	ARPC2	0.14237
BGLF4	ARFGEF2	0.082624
BGLF4	TTC19	0.17228
BGLF4	BSG	0.10947
BGLF4	MT2A	0.14666
BGLF4	AIG1	0.22976
BGLF4	ORM1	0.21523
BGLF4	HSD17B10	0.09365
BGLF4	RBM5	0.14075
BGLF4	BOP1	0.12999
BGLF4	NOX1	0.060435
BGLF4	GYG1	0.16318
BGLF4	DSC2	0.053342
BGLF4	CAPS	0.040702
BGLF4	CLSPN	0.15844
BGLF4	MLST8	0.16888
BGLF4	WTIP	0.12467
BGLF4	SPTB	0.10872
BGLF4	IL1R2	0.23022
BGLF4	KLF13	0.11767
BGLF4	MSN	0.060621
BGLF4	SPDL1	0.10606
BGLF4	UBE2D4	0.10491
BGLF4	SMARCE1	0.11391
BGLF4	PIGA	0.15929
BGLF4	RPS26	0.11742
BGLF4	MEN1	0.03772
BGLF4	GNMT	0.17011
BGLF4	BRE	0.15099
BGLF4	ZBTB14	0.14712
BGLF4	SCAMP1	0.18
BGLF4	SEC23A	0.097785
BGLF4	RND1	0.094995
BGLF4	BCKDHB	0.17941
BGLF4	PDLIM7	0.13734
BGLF4	SSH1	0.02758
BGLF4	RNMT	0.23677
BGLF4	TNFRSF21	0.19611
BGLF4	TTF2	0.091362
BGLF4	TOPORS	0.064668
BGLF4	DDX47	0.15928
BGLF4	FREM2	0.035882
BGLF4	GOLM1	0.21589
BGLF4	GNAI2	0.048646
BGLF4	DDX42	0.17157
BGLF4	GAPDHS	0.13171
BGLF4	CDKN1C	0.15851
BGLF4	GRIA1	0.082854
BGLF4	ANKLE2	0.14833
BGLF4	NUP153	0.10334
BGLF4	ETS2	0.030689
BGLF4	TOMM22	0.12947
BGLF4	VPS41	0.12494
BGLF4	MAGOH	0.18717
BGLF4	SKI	0.028521
BGLF4	SRGN	0.11853
BGLF4	CEP41	0.14712
BGLF4	RAC1	0.025654
BGLF4	INTS9	0.20196
BGLF4	IPO5	0.15173
BGLF4	VDAC1	0.092424
BGLF4	PRMT5	0.13297
BGLF4	MUC16	0.17344
BGLF4	TNFAIP1	0.13245
BGLF4	GRIN2A	0.010184
BGLF4	CRNN	0.060496
BGLF4	ING4	0.11546
BGLF4	PECAM1	0.14796

BGLF4	EML1	0.41151
BGLF4	GTF2F2	0.087885
BGLF4	CEP170	0.19656
BGLF4	C19orf68	0.24432
BGLF4	NOL3	0.17291
BGLF4	ZWILCH	0.16565
BGLF4	VAV2	0.053953
BGLF4	KIF24	0.15748
BGLF4	MAGED1	0.13928
BGLF4	ETS1	0.016927
BGLF4	PTGER4	0.084808
BGLF4	RPRD1B	0.16737
BGLF4	CWC15	0.24985
BGLF4	CALCRL	0.064085
BGLF4	RBPMS	0.19486
BGLF4	DES	0.11716
BGLF4	WDR4	0.18363
BGLF4	PSMC1	0.069699
BGLF4	USP5	0.11267
BGLF4	CITED1	0.027717
BGLF4	HAT1	0.16766
BGLF4	SNCAIP	0.15937
BGLF4	FZD3	0.094567
BGLF4	LIN28A	0.11875
BGLF4	MPDZ	0.1615
BGLF4	NGFRAP1	0.14125
BGLF4	ARID2	0.15108
BGLF4	PGS1	0.03713
BGLF4	TLDC1	0.24432
BGLF4	COMMD3	0.24432
BGLF4	SMAD7	0.029948
BGLF4	SERPINA1	0.16243
BGLF4	NCK1	0.073382
BGLF4	GABBR1	0.056314
BGLF4	MPG	0.053049
BGLF4	CRTAC1	0.076244
BGLF4	SCP2	0.069217
BGLF4	TMEM173	0.030798
BGLF4	MAD2L1	0.091976
BGLF4	PRRC2B	0.24432
BGLF4	CLN6	0.16396
BGLF4	KCNJ3	0.17624
BGLF4	BYSL	0.15162
BGLF4	ERN1	0.016683
BGLF4	EYA1	0.016401
BGLF4	SPRTN	0.15754
BGLF4	MYD88	0.043586
BGLF4	LSM5	0.17718
BGLF4	CTC1	0.13241
BGLF4	FBXW8	0.22285
BGLF4	SPPL2A	0.12341
BGLF4	PLS1	0.060156
BGLF4	SYVN1	0.083757
BGLF4	CACYBP	0.16695
BGLF4	INS	0.029484
BGLF4	IRAK4	0.04745
BGLF4	EPS15	0.21954
BGLF4	PLS3	0.064189
BGLF4	MYO5B	0.16773
BGLF4	WWC1	0.13702
BGLF4	HUS1	0.13524
BGLF4	PCSK2	0.22348
BGLF4	CRHR1	0.056014
BGLF4	APLF	0.12277
BGLF4	YY1	0.060589
BGLF4	NKRF	0.19669
BGLF4	ZNF335	0.15166
BGLF4	AURKB	0.018856

BGLF4	RRAGC	0.090668
BGLF4	LZTS1	0.055642
BGLF4	CD48	0.14112
BGLF4	VWCE	0.066109
BGLF4	CLEC3B	0.055771
BGLF4	PRMT8	0.18158
BGLF4	INPP5B	0.14065
BGLF4	ARHGEF16	0.072979
BGLF4	BST2	0.093798
BGLF4	NHLH2	0.14807
BGLF4	PEX11G	0.18637
BGLF4	OGDHL	0.20759
BGLF4	FKBP8	0.10049
BGLF4	DISC1	0.10365
BGLF4	IL33	0.1453
BGLF4	PUM2	0.25537
BGLF4	ESPL1	0.10629
BGLF4	PCGF1	0.14822
BGLF4	CDH8	0.054855
BGLF4	LTC4S	0.081563
BGLF4	AP3S1	0.24752
BGLF4	UHMK1	0.077683
BGLF4	LAT2	0.18889
BGLF4	BUB3	0.10319
BGLF4	RBMY1B	0.21373
BGLF4	SPRR3	0.098148
BGLF4	SDF4	0.30046
BGLF4	KIF14	0.12762
BGLF4	AFF4	0.091442
BGLF4	LEPR	0.18596
BGLF4	ANKHD1	0.2604
BGLF4	MGP	0.047945
BGLF4	BCKDHA	0.19174
BGLF4	TRIM33	0.070548
BGLF4	HBB	0.13176
BGLF4	KIF5B	0.10827
BGLF4	HEG1	0.033632
BGLF4	PRKCQ	0.013686
BGLF4	PHIP	0.13517
BGLF4	TAF11	0.17397
BGLF4	TRPC7	0.12906
BGLF4	LAMTOR2	0.14867
BGLF4	MAZ	0.18463
BGLF4	HIST1H1E	0.14794
BGLF4	WSB1	0.21963
BGLF4	CDC45	0.14167
BGLF4	YAF2	0.13765
BGLF4	MYH9	0.046913
BGLF4	EID3	0.17167
BGLF4	SPACA3	0.18409
BGLF4	PTPLB	0.13933
BGLF4	INSIG2	0.096863
BGLF4	CDCA8	0.11802
BGLF4	STAT1	0.069375
BGLF4	PRKAB1	0.060681
BGLF4	GJA1	0.029571
BGLF4	PSEN2	0.016858
BGLF4	FAT4	0.39838
BGLF4	DTNBP1	0.11523
BGLF4	CUL9	0.13181
BGLF4	PNMA2	0.20333
BGLF4	WWP1	0.091011
BGLF4	PLIN3	0.16316
BGLF4	KCNIP4	0.060061
BGLF4	USP34	0.16953
BGLF4	ZNHIT6	0.17358
BGLF4	VPS18	0.13501
BGLF4	ARHGAP4	0.12713

BGLF4	IFI16	0.060449
BGLF4	PPEF1	0.31748
BGLF4	ZW10	0.067883
BGLF4	GRIN3A	0.081987
BGLF4	CANT1	0.011197
BGLF4	NPW	0.19226
BGLF4	MAPK11	0.033922
BGLF4	A2M	0.11584
BGLF4	DCTN1	0.13379
BGLF4	FTSJ3	0.23544
BGLF4	ETV1	0.075952
BGLF4	PRKAA2	0.011592
BGLF4	NELL2	0.31904
BGLF4	CALB2	0.078865
BGLF4	PAICS	0.11009
BGLF4	DDX60	0.13724
BGLF4	CLTB	0.17458
BGLF4	PCDHA7	0.05466
BGLF4	CHD4	0.043157
BGLF4	ZC3H10	0.19428
BGLF4	ARRB2	0.055342
BGLF4	PRDX1	0.14867
BGLF4	RHOG	0.094834
BGLF4	TTC3	0.10165
BGLF4	SPON1	0.22019
BGLF4	IRAK3	0.030679
BGLF4	CYFIP1	0.11916
BGLF4	NR2F1	0.054892
BGLF4	ULK2	0.098543
BGLF4	SRSF3	0.1846
BGLF4	PDZK1	0.20105
BGLF4	MCC	0.16191
BGLF4	KAT2B	0.033007
BGLF4	CROCC	0.094577
BGLF4	LAMTOR3	0.16962
BGLF4	ARRB1	0.044607
BGLF4	ELOVL4	0.14256
BGLF4	UBE3A	0.079979
BGLF4	TCF4	0.060668
BGLF4	GTF3C1	0.15533
BGLF4	SMC1A	0.063059
BGLF4	DMAP1	0.1464
BGLF4	RPS27	0.082704
BGLF4	CHEK2	0.01862
BGLF4	STX1A	0.083967
BGLF4	DRD2	0.01351
BGLF4	APH1A	0.091168
BGLF4	GBAS	0.20622
BGLF4	BAZ1B	0.085233
BGLF4	AKIP1	0.20848
BGLF4	PCDHGC5	0.058173
BGLF4	KAT2A	0.11257
BGLF4	COL4A3BP	0.038843
BGLF4	TRPC4	0.11466
BGLF4	KIF5C	0.12801
BGLF4	FZD10	0.09057
BGLF4	PIN1	0.12594
BGLF4	TLX3	0.080111
BGLF4	SUV420H2	0.21804
BGLF4	PCDHGA7	0.058173
BGLF4	HECTD1	0.14207
BGLF4	NME1	0.07872
BGLF4	NOP58	0.16955
BGLF4	MAP1LC3C	0.25543
BGLF4	RFC3	0.10419
BGLF4	ATP6V1E1	0.13844
BGLF4	ATP11B	0.069664
BGLF4	RNF14	0.085438

BGLF4	TARBP2	0.12627
BGLF4	SFR1	0.21635
BGLF4	TAB1	0.080297
BGLF4	RHOA	0.020989
BGLF4	KCNH2	0.090719
BGLF4	KRT20	0.1582
BGLF4	ZNF646	0.15166
BGLF4	MAP4K4	0.063931
BGLF4	DCAF11	0.23579
BGLF4	THBS1	0.040851
BGLF4	RUNX3	0.039326
BGLF4	LMTK2	0.052874
BGLF4	GADD45GIP1	0.18453
BGLF4	DIS3	0.13403
BGLF4	ARR3	0.16015
BGLF4	ABCD3	0.11944
BGLF4	UBR2	0.095033
BGLF4	CMKLR1	0.1299
BGLF4	STK25	0.052583
BGLF4	FANCF	0.19965
BGLF4	SEMG1	0.12143
BGLF4	ICAM3	0.14916
BGLF4	MRPL49	0.19035
BGLF4	ASS1	0.0198
BGLF4	EIF3I	0.2373
BGLF4	ARL2BP	0.18137
BGLF4	UBTF	0.15577
BGLF4	FGF2	0.022457
BGLF4	PFN1	0.10419
BGLF4	DLG3	0.10101
BGLF4	RBCK1	0.038877
BGLF4	DSG2	0.043805
BGLF4	MAP3K12	0.024003
BGLF4	CCNE1	0.042608
BGLF4	RHOC	0.060701
BGLF4	PITX2	0.011556
BGLF4	ADRA2B	0.090745
BGLF4	ATXN3	0.098491
BGLF4	EPB41L3	0.098046
BGLF4	CEBPG	0.044265
BGLF4	DEDD2	0.1322
BGLF4	MGMT	0.038727
BGLF4	GIT2	0.12198
BGLF4	SNX33	0.22975
BGLF4	STK24	0.03666
BGLF4	PCDHB2	0.050061
BGLF4	ATG101	0.18044
BGLF4	MIF	0.080599
BGLF4	ISG20L2	0.1435
BGLF4	BICD1	0.13117
BGLF4	AP2M1	0.14164
BGLF4	ING2	0.08882
BGLF4	PIK3CG	0.03323
BGLF4	ADORA1	0.029766
BGLF4	CDC42EP2	0.13898
BGLF4	SF3A1	0.21164
BGLF4	INTS3	0.19331
BGLF4	DSG4	0.045034
BGLF4	TCTEX1D4	0.24432
BGLF4	TMCC2	0.22976
BGLF4	PLG	0.079586
BGLF4	TEAD3	0.068437
BGLF4	NOS2	0.059987
BGLF4	PRMT1	0.1982
BGLF4	USP10	0.1179
BGLF4	LASP1	0.14036
BGLF4	MT-ND1	0.17274
BGLF4	RASSF5	0.18139

BGLF4	CRB2	0.060554
BGLF4	DYNLT1	0.10035
BGLF4	ZMIZ2	0.13856
BGLF4	ALDOB	0.03503
BGLF4	S100Z	0.068073
BGLF4	MED26	0.1426
BGLF4	LRP5	0.074198
BGLF4	HSD17B12	0.097129
BGLF4	DGKQ	0.10899
BGLF4	CRP	0.1489
BGLF4	ARIH2	0.10023
BGLF4	DHX36	0.10748
BGLF4	C1QBP	0.14275
BGLF4	DDOST	0.13655
BGLF4	AGR3	0.27761
BGLF4	AP1M1	0.18005
BGLF4	PUF60	0.15775
BGLF4	GMCL1P1	0.21221
BGLF4	STRA13	0.15141
BGLF4	SNX8	0.18473
BGLF4	MAPK6	0.036194
BGLF4	RPL23A	0.11787
BGLF4	SPOP	0.19946
BGLF4	PRKCZ	0.016208
BGLF4	HDDC2	0.16661
BGLF4	FANCI	0.19278
BGLF4	CYCS	0.067687
BGLF4	SETDB1	0.18211
BGLF4	CDC14A	0.021419
BGLF4	SEC22B	0.13913
BGLF4	ATP7A	0.01721
BGLF4	AGR2	0.25349
BGLF4	UQCRC2	0.080303
BGLF4	FHL2	0.048105
BGLF4	ADM	0.06213
BGLF4	TPPP	0.30521
BGLF4	ZNF746	0.1123
BGLF4	PRPF4	0.176
BGLF4	DBNDD2	0.22976
BGLF4	BMP10	0.088402
BGLF4	TBXA2R	0.091025
BGLF4	FYB	0.1135
BGLF4	SYT4	0.10265
BGLF4	IL17RE	0.21609
BGLF4	CENPT	0.12817
BGLF4	RAD51D	0.14558
BGLF4	SYT11	0.37446
BGLF4	CD5	0.16027
BGLF4	SH2D4A	0.18677
BGLF4	ICAM1	0.051558
BGLF4	GZMB	0.15454
BGLF4	GGA2	0.15525
BGLF4	MAD1L1	0.093515
BGLF4	DCUN1D1	0.22979
BGLF4	LPAR1	0.056681
BGLF4	MOB3B	0.21811
BGLF4	MSH6	0.041073
BGLF4	SNRNP200	0.11255
BGLF4	PTCH1	0.031119
BGLF4	EIF2B1	0.11113
BGLF4	PDE3B	0.073825
BGLF4	DRD3	0.04418
BGLF4	CTNNA3	0.10163
BGLF4	IFITM1	0.13343
BGLF4	SWSAP1	0.20695
BGLF4	DDX17	0.13226
BGLF4	RECQL	0.092546
BGLF4	BCS1L	0.1311

BGLF4	SH3PXD2A	0.19018
BGLF4	SFPQ	0.19119
BGLF4	PARPBP	0.19487
BGLF4	FBXL15	0.1895
BGLF4	FANCA	0.17631
BGLF4	EIF2B3	0.16386
BGLF4	TMEM11	0.18701
BGLF4	KDM1A	0.020246
BGLF4	PCDHAC2	0.05466
BGLF4	CLSTN2	0.016916
BGLF4	RBMS1	0.20264
BGLF4	ATP6V0D1	0.12296
BGLF4	TPD52L1	0.16977
BGLF4	WDFY3	0.18387
BGLF4	PSMA4	0.053606
BGLF4	HSP90B1	0.1891
BGLF4	NR2C1	0.068997
BGLF4	PCDH7	0.056325
BGLF4	PIAS1	0.088073
BGLF4	HOXB7	0.07476
BGLF4	TNFSF13B	0.094483
BGLF4	MPP5	0.17654
BGLF4	TRIM2	0.14367
BGLF4	DDAH2	0.15591
BGLF4	ATPAF2	0.22287
BGLF4	MACF1	0.23827
BGLF4	AIFM1	0.10538
BGLF4	CKAP5	0.12637
BGLF4	TNFRSF12A	0.14169
BGLF4	POLR3F	0.13119
BGLF4	S100A3	0.048404
BGLF4	MEIS1	0.06186
BGLF4	WWOX	0.13104
BGLF4	ADORA2A	0.029898
BGLF4	SOS1	0.066393
BGLF4	APOD	0.093938
BGLF4	TXNL4A	0.18333
BGLF4	EVL	0.14734
BGLF4	BOK	0.13627
BGLF4	ALG2	0.12994
BGLF4	GLMN	0.19668
BGLF4	MAGEC2	0.19203
BGLF4	UVSSA	0.2119
BGLF4	PCM1	0.10485
BGLF4	TPSAB1	0.2183
BGLF4	TXNRD2	0.21888
BGLF4	FGF21	0.19394
BGLF4	PCGF3	0.16255
BGLF4	MSR1	0.19363
BGLF4	DUOX2	0.092127
BGLF4	RTN1	0.18275
BGLF4	LARS	0.041067
BGLF4	RBM14	0.1129
BGLF4	OSBPL6	0.17557
BGLF4	STBD1	0.20298
BGLF4	CASQ2	0.053544
BGLF4	MCTP2	0.061609
BGLF4	PRKG1	0.041917
BGLF4	TRPC4AP	0.21482
BGLF4	LPXN	0.11869
BGLF4	UBE2A	0.10062
BGLF4	EPHA7	0.053582
BGLF4	SNTB2	0.18046
BGLF4	CX3CL1	0.059407
BGLF4	PCDHGB4	0.056436
BGLF4	AP5Z1	0.18017
BGLF4	PPIE	0.19559
BGLF4	KLRK1	0.12188

BGLF4	CAST	0.24004
BGLF4	TBC1D25	0.22556
BGLF4	DNAJC7	0.19219
BGLF4	GCH1	0.11134
BGLF4	PTGES3	0.10846
BGLF4	ANKRD1	0.076958
BGLF4	VPS11	0.18655
BGLF4	POLE3	0.15972
BGLF4	SLC9A3	0.1415
BGLF4	OGN	0.15931
BGLF4	HNRNPU	0.12577
BGLF4	OPRD1	0.10896
BGLF4	MDM2	0.037572
BGLF4	NKD2	0.30273
BGLF4	LSAMP	0.2075
BGLF4	PTPN23	0.074576
BGLF4	ADAMTSL2	0.15332
BGLF4	FLNC	0.18586
BGLF4	MAGI3	0.11285
BGLF4	SLAMF1	0.13458
BGLF4	IFT57	0.18785
BGLF4	ADAM23	0.11836
BGLF4	AP5B1	0.21937
BGLF4	TRIM29	0.084604
BGLF4	EIF2B5	0.14611
BGLF4	DENR	0.25717
BGLF4	ZNF350	0.15468
BGLF4	TMEM132D	0.20959
BGLF4	MAPK8IP1	0.065096
BGLF4	FOXN3	0.032118
BGLF4	PTPN4	0.063425
BGLF4	ATP2B2	0.057373
BGLF4	BTG2	0.1247
BGLF4	PEX3	0.14241
BGLF4	GHITM	0.19717
BGLF4	INCENP	0.10615
BGLF4	TBC1D9	0.038744
BGLF4	DOT1L	0.18404
BGLF4	JMJD1C	0.21649
BGLF4	GJD3	0.19664
BGLF4	FNBP1L	0.21394
BGLF4	ITGAV	0.05725
BGLF4	TXLNB	0.22975
BGLF4	SEC13	0.1342
BGLF4	CDH10	0.053252
BGLF4	CDKN2AIP	0.18059
BGLF4	KEAP1	0.16568
BGLF4	EPHA10	0.086542
BGLF4	CNOT6	0.16205
BGLF4	LCK	0.020572
BGLF4	HIPK2	0.019013
BGLF4	PSMC4	0.064164
BGLF4	RAD50	0.044603
BGLF4	FAT1	0.24461
BGLF4	REV1	0.1105
BGLF4	BAZ1A	0.12274
BGLF4	OCRL	0.09983
BGLF4	DYRK1B	0.046599
BGLF4	ABCF1	0.12589
BGLF4	HDAC3	0.032145
BGLF4	FBLN7	0.059325
BGLF4	NXF3	0.22299
BGLF4	H2AFY	0.12666
BGLF4	FOXK1	0.011587
BGLF4	CCNH	0.01773
BGLF4	NGLY1	0.17918
BGLF4	EDAR	0.13852
BGLF4	MTCH1	0.1776

BGLF4	ERCC8	0.14478
BGLF4	CRELD1	0.063655
BGLF4	ISOC2	0.15881
BGLF4	TGOLN2	0.17317
BGLF4	HABP4	0.15618
BGLF4	XAB2	0.16915
BGLF4	S100A11	0.33887
BGLF4	ITGA5	0.084147
BGLF4	GDF7	0.12414
BGLF4	GPS2	0.18732
BGLF4	SRSF5	0.15909
BGLF4	USH1G	0.15882
BGLF4	GTF2H4	0.014149
BGLF4	SORBS1	0.07677
BGLF4	CHMP1B	0.17402
BGLF4	HFE2	0.16138
BGLF4	IRF3	0.027658
BGLF4	EFCAB6	0.045351
BGLF4	RBBP6	0.1084
BGLF4	MAP3K3	0.050703
BGLF4	ESR2	0.045135
BGLF4	TPP1	0.10933
BGLF4	NCAPG	0.17212
BGLF4	NUFIP2	0.25913
BGLF4	ECSIT	0.20907
BGLF4	LRRFIP2	0.21783
BGLF4	AIRE	0.063882
BGLF4	XPO4	0.19354
BGLF4	INIP	0.20677
BGLF4	NKD1	0.31124
BGLF4	NR1H2	0.034531
BGLF4	PPP1R9B	0.093511
BGLF4	PLEKHM2	0.14418
BGLF4	CBFA2T3	0.028363
BGLF4	DPM3	0.12567
BGLF4	CYBB	0.096028
BGLF4	CEP70	0.16118
BGLF4	DERL2	0.15196
BGLF4	ACACA	0.09527
BGLF4	SPTBN4	0.051246
BGLF4	STAP1	0.069804
BGLF4	EDA2R	0.14159
BGLF4	PPP6C	0.13979
BGLF4	TRIAP1	0.18597
BGLF4	FASTK	0.097573
BGLF4	RBFOX2	0.13181
BGLF4	GCK	0.043039
BGLF4	TPX2	0.060787
BGLF4	RPS10	0.11132
BGLF4	CD2AP	0.16363
BGLF4	RAP2A	0.10457
BGLF4	CD1A	0.18607
BGLF4	SPATA18	0.13319
BGLF4	CBX8	0.16316
BGLF4	PAGR1	0.20562
BGLF4	RNF146	0.10744
BGLF4	KIF18A	0.06945
BGLF4	GORAB	0.1972
BGLF4	GABPB1	0.058805
BGLF4	GREM1	0.034098
BGLF4	TSKS	0.12923
BGLF4	INTS12	0.16313
BGLF4	UBE2N	0.048152
BGLF4	TAF7	0.022597
BGLF4	MAGEB18	0.24432
BGLF4	MED31	0.15748
BGLF4	MAT2B	0.10479
BGLF4	GEMIN4	0.14728

BGLF4	HMCN1	0.088281
BGLF4	SLC9A3R2	0.10464
BGLF4	KNSTRN	0.12568
BGLF4	ODF2	0.072866
BGLF4	SERPINE1	0.039667
BGLF4	PCBP2	0.14917
BGLF4	SPC25	0.1372
BGLF4	RBM25	0.15309
BGLF4	DACH1	0.041494
BGLF4	PKP1	0.17077
BGLF4	DNAJB5	0.19799
BGLF4	EIF1AY	0.32778
BGLF4	FNIP2	0.16789
BGLF4	DEAF1	0.12217
BGLF4	ARID4B	0.17187
BGLF4	EMR2	0.029551
BGLF4	TREML1	0.1896
BGLF4	MOV10	0.16454
BGLF4	MAGI1	0.13334
BGLF4	PTGIS	0.044883
BGLF4	ARL4A	0.15192
BGLF4	BPTF	0.094425
BGLF4	WRN	0.012683
BGLF4	USF1	0.015973
BGLF4	STMN2	0.14743
BGLF4	CAPS2	0.068073
BGLF4	ARHGAP32	0.08528
BGLF4	MTDH	0.08567
BGLF4	RP9	0.15847
BGLF4	PRRC2A	0.20848
BGLF4	MKKS	0.05563
BGLF4	GMNN	0.13559
BGLF4	PDZD11	0.2145
BGLF4	METTL17	0.21404
BGLF4	MYLPF	0.054336
BGLF4	PSMD10	0.044651
BGLF4	SDHAF2	0.21069
BGLF4	ST13	0.13888
BGLF4	APOA1	0.031639
BGLF4	PSMA5	0.057192
BGLF4	HHEX	0.011517
BGLF4	PPP2R3B	0.30831
BGLF4	PEG10	0.14422
BGLF4	INO80	0.086386
BGLF4	CTNNBIP1	0.12931
BGLF4	CENPH	0.099326
BGLF4	HDAC9	0.044364
BGLF4	HEY1	0.035336
BGLF4	ACTN4	0.18833
BGLF4	FBXO46	0.24432
BGLF4	KIAA0141	0.22287
BGLF4	DTX3L	0.1334
BGLF4	EHD1	0.18161
BGLF4	ATL1	0.078689
BGLF4	KIR2DL1	0.19146
BGLF4	RAB25	0.20209
BGLF4	TIMM44	0.12684
BGLF4	PARD6B	0.12883
BGLF4	GTF2E1	0.13901
BGLF4	RAN	0.076365
BGLF4	KIAA0408	0.24432
BGLF4	LILRA4	0.22976
BGLF4	EEA1	0.14229
BGLF4	SUN2	0.10628
BGLF4	SEPT8	0.18614
BGLF4	PINK1	0.015698
BGLF4	FANCG	0.14278
BGLF4	CTU2	0.20364

BGLF4	OXA1L	0.14202
BGLF4	INTS10	0.19104
BGLF4	LPL	0.046758
BGLF4	PKMYT1	0.026794
BGLF4	ERCC6	0.055527
BGLF4	PARVA	0.095061
BGLF4	VCAN	0.036598
BGLF4	CDR2	0.20219
BGLF4	SHFM1	0.22014
BGLF4	KRIT1	0.14752
BGLF4	RABAC1	0.19997
BGLF4	RUNX2	0.028622
BGLF4	AMFR	0.056501
BGLF4	NEU4	0.11376
BGLF4	INTS8	0.20196
BGLF4	CNOT6L	0.1394
BGLF4	RPS19BP1	0.1972
BGLF4	COMMD1	0.13723
BGLF4	SEPT2	0.070385
BGLF4	RBM4	0.081871
BGLF4	KPNA3	0.13674
BGLF4	ZNRF1	0.16239
BGLF4	GRB7	0.077933
BGLF4	PLCB1	0.014337
BGLF4	ZSCAN21	0.070116
BGLF4	TBP	0.010348
BGLF4	TESK1	0.062133
BGLF4	CDC42BPG	0.016465
BGLF4	CABP4	0.053473
BGLF4	GEMIN6	0.16041
BGLF4	MAPK12	0.033161
BGLF4	MMP2	0.059439
BGLF4	INHA	0.074782
BGLF4	CSN2	0.080664
BGLF4	POLE4	0.16459
BGLF4	BAD	0.017762
BGLF4	EIF2B2	0.14157
BGLF4	RPTN	0.0874
BGLF4	EXOSC1	0.18763
BGLF4	DSP	0.077572
BGLF4	RGS19	0.11062
BGLF4	ZFYVE20	0.117
BGLF4	UBE2G2	0.10519
BGLF4	RMI1	0.20853
BGLF4	AIP	0.067558
BGLF4	S100A13	0.26091
BGLF4	PIK3R6	0.10865
BGLF4	STUB1	0.077762
BGLF4	MEA1	0.20982
BGLF4	MELK	0.11144
BGLF4	EXO1	0.0765
BGLF4	GEM	0.11572
BGLF4	NEK1	0.046135
BGLF4	HPGDS	0.063844
BGLF4	MED18	0.1597
BGLF4	BNIP3	0.038364
BGLF4	INHBB	0.13771
BGLF4	HSP90AB1	0.082154
BGLF4	CORO1B	0.20182
BGLF4	HTRA3	0.23045
BGLF4	PSMB1	0.05682
BGLF4	S100A1	0.29477
BGLF4	EIF3H	0.23012
BGLF4	TMED9	0.15109
BGLF4	GPATCH8	0.1731
BGLF4	PRMT6	0.13906
BGLF4	MYL7	0.075976
BGLF4	SNRPC	0.18995

43385	BGLF4	SUMO1	0.080417
43386	BGLF4	PPIA	0.12453
43387	BGLF4	SUMO2	0.19633
43388	BGLF4	USP1	0.1419
43389	BGLF4	ARHGAP24	0.096773
43390	BGLF4	UTRN	0.23419
43391	BGLF4	TRAF3IP3	0.22976
43392	BGLF4	STAM2	0.121
43393	BGLF4	EGR2	0.063109
43394	BGLF4	UBE2D2	0.09744
43395	BGLF4	EGFL8	0.072824
43396	BGLF4	RAD23B	0.12742
43397	BGLF4	HHIP	0.058184
43398	BGLF4	FANCE	0.20584
43399	BGLF4	GNS	0.046007
43400	BGLF4	PCGF5	0.15804
43401	BGLF4	S100A2	0.065606
43402	BGLF4	POLD4	0.10977
43403	BGLF4	RPL15	0.18098
43404	BGLF4	PALM	0.10425
43405	BGLF4	IDE	0.02343
43406	BGLF4	AP2A2	0.13164
43407	BGLF4	ASH2L	0.13215
43408	BGLF4	DOHH	0.1308
43409	BGLF4	IGF1	0.020118
43410	BGLF4	SLC34A1	0.18204
43411	BGLF4	TRAF3IP2	0.19817
43412	BGLF4	SCUBE2	0.066109
43413	BGLF4	TBC1D2B	0.10753
43414	BGLF4	MCM7	0.081787
43415	BGLF4	TOP2A	0.051091
43416	BGLF4	SGOL2	0.13167
43417	BGLF4	PIK3CA	0.046229
43418	BGLF4	MYL9	0.044354
43419	BGLF4	NOP56	0.21142
43420	BGLF4	KIAA0101	0.1809
43421	BGLF4	TNFRSF1B	0.14823
43422	BGLF4	BAHD1	0.16166
43423	BGLF4	E4F1	0.029394
43424	BGLF4	SPRY2	0.04235
43425	BGLF4	ENG	0.046642
43426	BGLF4	MYO18A	0.13875
43427	BGLF4	HAND1	0.014923
43428	BGLF4	ACP1	0.049028
43429	BGLF4	AQP1	0.045305
43430	BGLF4	MAPKAPK3	0.028171
43431	BGLF4	ELN	0.16167
43432	BGLF4	SWI5	0.21635
43433	BGLF4	LTF	0.12348
43434	BGLF4	PPM1B	0.11357
43435	BGLF4	TBC1D10A	0.17686
43436	BGLF4	EFHD1	0.062141
43437	BGLF4	MAP2K3	0.021987
43438	BGLF4	SMC2	0.11196
43439	BGLF4	ZC3HAV1	0.15867
43440	BGLF4	SAMM50	0.13447
43441	BGLF4	FASLG	0.068632
43442	BGLF4	VWF	0.11976
43443	BGLF4	TMBIM4	0.16418
43444	BGLF4	SAP30BP	0.17939
43445	BGLF4	ZFPM2	0.072974
43446	BGLF4	CIB4	0.068073
43447	BGLF4	TSG101	0.072414
43448	BGLF4	CRADD	0.12527
43449	BGLF4	SPTBN1	0.074572
43450	BGLF4	EDEM2	0.026309
43451	BGLF4	KANK1	0.12938
43452	BGLF4	NUP37	0.11759

BGLF4	FLII	0.1622
BGLF4	EMD	0.11716
BGLF4	ECHS1	0.11017
BGLF4	NTRK1	0.030472
BGLF4	RBM17	0.21536
BGLF4	THBS3	0.042815
BGLF4	CUBN	0.027306
BGLF4	PSPH	0.025965
BGLF4	S100A14	0.038679
BGLF4	STAT6	0.17573
BGLF4	MPZL2	0.18591
BGLF4	INVS	0.14773
BGLF4	DTNB	0.02812
BGLF4	USP4	0.13764
BGLF4	RBPJ	0.02744
BGLF4	ATG3	0.13225
BGLF4	PRRG3	0.060576
BGLF4	ULK1	0.02896
BGLF4	LLGL1	0.026396
BGLF4	SPOCK2	0.075498
BGLF4	HYAL2	0.041381
BGLF4	STAMBP	0.10332
BGLF4	CTLA4	0.17849
BGLF4	SLIT3	0.028932
BGLF4	ARPC5	0.15485
BGLF4	NELFE	0.17441
BGLF4	CLEC1B	0.19769
BGLF4	ATRAID	0.13242
BGLF4	FBXO4	0.16757
BGLF4	HNRNPL	0.15159
BGLF4	TLE2	0.16135
BGLF4	PPP2R2A	0.14604
BGLF4	AKAP5	0.034155
BGLF4	CALB1	0.26944
BGLF4	DAZAP2	0.20219
BGLF4	CPSF2	0.193
BGLF4	MAP4K1	0.059333
BGLF4	PCGF6	0.065579
BGLF4	PEX5	0.085903
BGLF4	RYK	0.077998
BGLF4	RASSF6	0.22254
BGLF4	NDEL1	0.07804
BGLF4	HHLA3	0.24432
BGLF4	NPHS2	0.18675
BGLF4	DIP2A	0.11916
BGLF4	GAN	0.20002
BGLF4	PDIA4	0.11054
BGLF4	MIER1	0.15502
BGLF4	MAP3K11	0.03807
BGLF4	BAZ2A	0.13191
BGLF4	CAPN11	0.060875
BGLF4	SFTPC	0.14139
BGLF4	UFD1L	0.17943
BGLF4	LIN7A	0.1393
BGLF4	SETMAR	0.12692
BGLF4	VAV3	0.047301
BGLF4	MASP2	0.34626
BGLF4	BATF2	0.10856
BGLF4	NUDT21	0.17958
BGLF4	PCDHA9	0.058173
BGLF4	PARK7	0.13505
BGLF4	RP2	0.093929
BGLF4	FKBP1A	0.099564
BGLF4	TAF6L	0.099323
BGLF4	SLC25A23	0.060535
BGLF4	TRAF7	0.14168
BGLF4	BCL2L10	0.15102
BGLF4	RCN2	0.063812

BGLF4	PRCP	0.21576
BGLF4	SUCLA2	0.070282
BGLF4	TAOK3	0.063337
BGLF4	SSTR2	0.094279
BGLF4	PPP4C	0.10546
BGLF4	HSPA1A	0.05846
BGLF4	TAP1	0.062691
BGLF4	ATG9A	0.21296
BGLF4	CALML4	0.068073
BGLF4	NDUFAF1	0.20007
BGLF4	COG5	0.13252
BGLF4	MAP3K5	0.015202
BGLF4	FRA10AC1	0.21497
BGLF4	PES1	0.12554
BGLF4	CCM2	0.20401
BGLF4	BBIP1	0.20708
BGLF4	TRIM39	0.10916
BGLF4	ABI1	0.083004
BGLF4	PTBP1	0.18262
BGLF4	SOST	0.1131
BGLF4	MAGEA3	0.24432
BGLF4	ZNF224	0.14226
BGLF4	RAMP2	0.070866
BGLF4	TAF13	0.056334
BGLF4	CAPSL	0.0661
BGLF4	RRAS2	0.11623
BGLF4	RAB4A	0.10154
BGLF4	FGF19	0.18678
BGLF4	DDN	0.16374
BGLF4	RAB5B	0.13585
BGLF4	RNF32	0.17374
BGLF4	ARL14	0.24726
BGLF4	LRWD1	0.13092
BGLF4	TXNDC9	0.22377
BGLF4	ARL6	0.076454
BGLF4	EIF3C	0.2373
BGLF4	EIF3M	0.2556
BGLF4	ZNF326	0.20239
BGLF4	MYOZ3	0.235
BGLF4	KLHL2	0.22307
BGLF4	DTX2	0.11806
BGLF4	COMMD10	0.24432
BGLF4	NOM1	0.23589
BGLF4	ACVR2A	0.016698
BGLF4	GRK5	0.025167
BGLF4	FANCC	0.14929
BGLF4	RFX3	0.054525
BGLF4	ITGB1BP2	0.033789
BGLF4	CENPA	0.068827
BGLF4	MDFIC	0.10161
BGLF4	UBXN6	0.17562
BGLF4	SYNPO2	0.19338
BGLF4	BABAM1	0.17574
BGLF4	IL5RA	0.20226
BGLF4	AIF1L	0.046341
BGLF4	LPCAT4	0.047333
BGLF4	CDH11	0.050946
BGLF4	RABL6	0.16361
BGLF4	APEX1	0.071827
BGLF4	ELK3	0.051857
BGLF4	CBLL1	0.13102
BGLF4	PCDHAC1	0.05466
BGLF4	EPPIN	0.20345
BGLF4	GAS6	0.02955
BGLF4	BET1	0.13377
BGLF4	AMPH	0.17867
BGLF4	ABLIM1	0.14839
BGLF4	RANGAP1	0.085118

BGLF4	CIRBP	0.15006
BGLF4	ERLEC1	0.21569
BGLF4	LATS1	0.013807
BGLF4	KIFAP3	0.14602
BGLF4	MED9	0.1597
BGLF4	ABCB1	0.10975
BGLF4	PCDHB6	0.050061
BGLF4	EFEMP1	0.34444
BGLF4	EIF4A3	0.1224
BGLF4	LPAR2	0.17306
BGLF4	ZAP70	0.043611
BGLF4	DAXX	0.068768
BGLF4	IL1A	0.084479
BGLF4	BID	0.070073
BGLF4	COMMD2	0.24432
BGLF4	KANSL1	0.18696
BGLF4	SDPR	0.149
BGLF4	AGRN	0.06869
BGLF4	MKL1	0.15302
BGLF4	ZNF281	0.041478
BGLF4	TTC37	0.1961
BGLF4	PNMA1	0.17632
BGLF4	APC2	0.14777
BGLF4	CCDC53	0.23222
BGLF4	NIPSNAP3A	0.23023
BGLF4	SPATA13	0.067169
BGLF4	MYO9B	0.11624
BGLF4	C1D	0.17811
BGLF4	BAP1	0.095049
BGLF4	CIR1	0.078877
BGLF4	ULK3	0.052738
BGLF4	TBCB	0.18592
BGLF4	SPARC	0.16899
BGLF4	GTF2H1	0.051791
BGLF4	CREB5	0.078034
BGLF4	TCHHL1	0.068073
BGLF4	EWSR1	0.18891
BGLF4	F2RL1	0.028767
BGLF4	OC90	0.052072
BGLF4	EHMT2	0.13654
BGLF4	PALLD	0.16116
BGLF4	SRSF11	0.19121
BGLF4	NOTCH4	0.14711
BGLF4	RAPGEF3	0.10488
BGLF4	PIK3C3	0.066345
BGLF4	TERT	0.11472
BGLF4	NUTF2	0.20372
BGLF4	ARPC4	0.16309
BGLF4	EXOSC10	0.092553
BGLF4	PCDHB1	0.058173
BGLF4	MEAF6	0.17028
BGLF4	ANG	0.022241
BGLF4	KLHL12	0.23986
BGLF4	TSPYL5	0.15203
BGLF4	PIM1	0.021536
BGLF4	DLX4	0.095503
BGLF4	MBD2	0.11974
BGLF4	MST4	0.024689
BGLF4	TRAIP	0.14498
BGLF4	DFNB31	0.12307
BGLF4	CYTH2	0.12205
BGLF4	CTR9	0.12416
BGLF4	NAA15	0.13407
BGLF4	MATN1	0.23082
BGLF4	NOTCH2NL	0.058806
BGLF4	CA4	0.083945
BGLF4	DEPDC1	0.16235
BGLF4	ID4	0.12204

BGLF4	KIAA1279	0.20896
BGLF4	PCDH17	0.39838
BGLF4	CSNK2A2	0.043168
BGLF4	PIR	0.206
BGLF4	IGSF8	0.1898
BGLF4	EPHA4	0.035512
BGLF4	CSRNP2	0.081299
BGLF4	SET	0.076107
BGLF4	MCL1	0.10073
BGLF4	PRICKLE1	0.11271
BGLF4	FGFBP1	0.15469
BGLF4	GPR37L1	0.17836
BGLF4	ARHGEF2	0.031197
BGLF4	SLC9A1	0.078821
BGLF4	KITLG	0.10067
BGLF4	DSC3	0.052312
BGLF4	SLC22A3	0.13289
BGLF4	B2M	0.10808
BGLF4	GLRB	0.098133
BGLF4	KCNN4	0.14616
BGLF4	PHB	0.088186
BGLF4	PRDX4	0.24533
BGLF4	CCDC85B	0.15649
BGLF4	NR5A1	0.02345
BGLF4	APC	0.01402
BGLF4	PMS2	0.10753
BGLF4	XK	0.23399
BGLF4	SCAF11	0.15006
BGLF4	EXOC3	0.21895
BGLF4	EIF3B	0.23679
BGLF4	CLGN	0.033086
BGLF4	CIB2	0.068073
BGLF4	LDB3	0.17445
BGLF4	PPM1D	0.086685
BGLF4	KHDRBS3	0.21961
BGLF4	ERBB4	0.018626
BGLF4	EXD3	0.24432
BGLF4	ADH1A	0.14396
BGLF4	SPEN	0.08455
BGLF4	CEP72	0.16842
BGLF4	ALB	0.064878
BGLF4	GGT1	0.13325
BGLF4	NAP1L1	0.15944
BGLF4	SRSF1	0.15463
BGLF4	EAF2	0.16051
BGLF4	CASK	0.041498
BGLF4	ESRRG	0.080485
BGLF4	TARDBP	0.095581
BGLF4	RASSF4	0.22951
BGLF4	REPS1	0.39931
BGLF4	IL15RA	0.19855
BGLF4	PLEK	0.067115
BGLF4	CIRH1A	0.18863
BGLF4	TYRO3	0.048115
BGLF4	RUFY1	0.16975
BGLF4	PEX2	0.12019
BGLF4	PRH1	0.23002
BGLF4	SLC18A2	0.017245
BGLF4	TNFRSF10A	0.14086
BGLF4	POP5	0.15232
BGLF4	TRPS1	0.057172
BGLF4	LRRC26	0.18387
BGLF4	BBS5	0.15382
BGLF4	SIVA1	0.1103
BGLF4	NLGN3	0.087351
BGLF4	GCC1	0.17414
BGLF4	AURKAIP1	0.1633
BGLF4	CYTIP	0.22394

BGLF4	USP14	0.12489
BGLF4	PCDHGA9	0.058173
BGLF4	ELOVL3	0.14532
BGLF4	EVI5	0.13013
BGLF4	RASA1	0.048311
BGLF4	PALB2	0.17095
BGLF4	CAND2	0.18755
BGLF4	FABP3	0.19517
BGLF4	CDHR5	0.044047
BGLF4	AHI1	0.06954
BGLF4	MRPS27	0.18583
BGLF4	ZBTB16	0.066099
BGLF4	SAT1	0.18916
BGLF4	DCTN2	0.11904
BGLF4	TBC1D8B	0.038744
BGLF4	TUBGCP3	0.066061
BGLF4	AGO1	0.15371
BGLF4	CD53	0.20305
BGLF4	USP25	0.14736
BGLF4	AFF1	0.10346
BGLF4	SH3GL2	0.12149
BGLF4	TULP1	0.13029
BGLF4	NXF5	0.21462
BGLF4	SNX1	0.14072
BGLF4	AQP2	0.012277
BGLF4	CD151	0.1795
BGLF4	MDFI	0.10968
BGLF4	YTHDC1	0.19747
BGLF4	MRPL28	0.19035
BGLF4	KRT16	0.15459
BGLF4	GUCA1C	0.073287
BGLF4	DOCK1	0.13015
BGLF4	CASP10	0.16738
BGLF4	MRPS9	0.16003
BGLF4	GEMIN7	0.16063
BGLF4	TBC1D7	0.21054
BGLF4	CDH7	0.053252
BGLF4	SPI1	0.034889
BGLF4	PPP1R1A	0.21386
BGLF4	SEC24C	0.065602
BGLF4	PRKCSH	0.092457
BGLF4	ZCCHC11	0.12305
BGLF4	TGFBR3	0.040822
BGLF4	ASUN	0.1899
BGLF4	AMY1A	0.12861
BGLF4	GRAP2	0.1571
BGLF4	EXOC7	0.17694
BGLF4	UPF1	0.10294
BGLF4	STX16	0.12029
BGLF4	TXNIP	0.10281
BGLF4	ANKRD28	0.20848
BGLF4	DRD4	0.053771
BGLF4	FGF1	0.07834
BGLF4	ITPR3	0.033134
BGLF4	GAGE2D	0.23693
BGLF4	NEK11	0.045696
BGLF4	ARL13B	0.18603
BGLF4	ASAP1	0.035445
BGLF4	ADAMTS10	0.10477
BGLF4	YLPM1	0.18759
BGLF4	HMGA1	0.020718
BGLF4	BRAT1	0.20848
BGLF4	SIGIRR	0.174
BGLF4	TAS2R16	0.15677
BGLF4	ADRA2C	0.10137
BGLF4	PCDHGA2	0.058173
BGLF4	RDX	0.14922
BGLF4	TYR	0.13924

BGLF4	POU3F2	0.056805
BGLF4	ITGB4	0.10222
BGLF4	KAL1	0.15115
BGLF4	ZXDC	0.057078
BGLF4	SSR4	0.0946
BGLF4	DDIT3	0.047895
BGLF4	GTF3C6	0.21532
BGLF4	WIBG	0.24416
BGLF4	ARHGAP22	0.077823
BGLF4	PCDH1	0.049259
BGLF4	SLC40A1	0.12285
BGLF4	FBXW5	0.09592
BGLF4	CFLAR	0.14874
BGLF4	EIF4EBP1	0.11692
BGLF4	MED29	0.18221
BGLF4	CDKN3	0.038977
BGLF4	TAX1BP1	0.16452
BGLF4	NOTCH2	0.10123
BGLF4	LOXL3	0.13668
BGLF4	ERLIN2	0.17782
BGLF4	EFCAB12	0.068073
BGLF4	CDH6	0.035075
BGLF4	FZR1	0.12495
BGLF4	ROR1	0.092779
BGLF4	AMER2	0.22694
BGLF4	MITD1	0.16963
BGLF4	TAF1D	0.19898
BGLF4	SLC4A1AP	0.1692
BGLF4	SIAH2	0.0824
BGLF4	EXOSC3	0.14913
BGLF4	STIM1	0.2214
BGLF4	COG4	0.16187
BGLF4	S100B	0.21562
BGLF4	TRAF3IP1	0.22976
BGLF4	ZAK	0.028713
BGLF4	FZD6	0.075123
BGLF4	EFHC1	0.014406
BGLF4	KAT6B	0.088653
BGLF4	HMG20B	0.086159
BGLF4	ESRRA	0.054355
BGLF4	SKP1	0.15322
BGLF4	VAMP1	0.20632
BGLF4	BIRC2	0.06511
BGLF4	ZNF212	0.13003
BGLF4	TNNC1	0.32784
BGLF4	DDB1	0.11653
BGLF4	RBMX	0.11675
BGLF4	SAP30	0.1633
BGLF4	ZNF24	0.081096
BGLF4	CHD3	0.064336
BGLF4	ADCYAP1R1	0.05543
BGLF4	CRYGC	0.18222
BGLF4	EFCAB4A	0.062336
BGLF4	ERBB2	0.01513
BGLF4	HTATIP2	0.11361
BGLF4	STEAP3	0.12912
BGLF4	TXLNA	0.1997
BGLF4	POLRMT	0.18579
BGLF4	BCAS2	0.18638
BGLF4	TMED2	0.15445
BGLF4	RAB3IL1	0.20103
BGLF4	CCNC	0.072887
BGLF4	C1orf94	0.24432
BGLF4	CASP6	0.071404
BGLF4	VCL	0.083001
BGLF4	PCDHB13	0.053256
BGLF4	CETN2	0.21305
BGLF4	CAMSAP2	0.21607

BGLF4	ANXA6	0.36866
BGLF4	PCDHGC4	0.058173
BGLF4	LCP1	0.038478
BGLF4	DIXDC1	0.092297
BGLF4	SIRPG	0.16261
BGLF4	PA2G4	0.073046
BGLF4	TINF2	0.12464
BGLF4	FBXO44	0.23502
BGLF4	ITM2B	0.068152
BGLF4	ANXA2P2	0.075001
BGLF4	CUL4B	0.17131
BGLF4	KPNB1	0.10183
BGLF4	FEZ1	0.14624
BGLF4	CBX4	0.10125
BGLF4	FKBP9	0.069072
BGLF4	NDC80	0.11763
BGLF4	ATP10D	0.10673
BGLF4	SMG5	0.1595
BGLF4	STK38L	0.023388
BGLF4	ACTN2	0.1279
BGLF4	COL3A1	0.098087
BGLF4	GGA1	0.17303
BGLF4	LRCH4	0.2266
BGLF4	EFNA1	0.1021
BGLF4	ATF5	0.078355
BGLF4	CNOT7	0.05469
BGLF4	SEPN1	0.19494
BGLF4	IFIT3	0.17382
BGLF4	PRSS2	0.30468
BGLF4	STAT2	0.18569
BGLF4	PRDM5	0.10877
BGLF4	FSHB	0.14103
BGLF4	PSRC1	0.13
BGLF4	REEP1	0.17968
BGLF4	PIFO	0.12951
BGLF4	METTL9	0.24432
BGLF4	CD59	0.17104
BGLF4	NCKAP1L	0.067338
BGLF4	LIMS1	0.14837
BGLF4	ZNF219	0.048066
BGLF4	ACTG1	0.064484
BGLF4	NCKAP1	0.13582
BGLF4	EIF4A2	0.16009
BGLF4	PNRC2	0.17786
BGLF4	PDE4DIP	0.17343
BGLF4	RAB27A	0.068597
BGLF4	SESTD1	0.17186
BGLF4	FKBP4	0.071748
BGLF4	KANK2	0.18411
BGLF4	RPL5	0.11942
BGLF4	TECPR2	0.24432
BGLF4	CDCA5	0.14699
BGLF4	HAX1	0.18163
BGLF4	STAU2	0.20344
BGLF4	SIRT1	0.010519
BGLF4	NOS3	0.021904
BGLF4	PITPNM3	0.34682
BGLF4	SEPSECS	0.13019
BGLF4	VIL1	0.16101
BGLF4	TNIP2	0.060631
BGLF4	ADCY6	0.023357
BGLF4	S100A7L2	0.048404
BGLF4	RNF126	0.20031
BGLF4	EIF5B	0.17091
BGLF4	STX11	0.20826
BGLF4	FSTL3	0.10032
BGLF4	ELK4	0.095706
BGLF4	WEE1	0.029099

BGLF4	PSMA6	0.060508
BGLF4	PCDHA3	0.05466
BGLF4	MUC5B	0.19598
BGLF4	CIB3	0.068073
BGLF4	CCDC59	0.20561
BGLF4	CD44	0.087682
BGLF4	PPID	0.22517
BGLF4	AGL	0.083902
BGLF4	SMNDC1	0.22232
BGLF4	AATF	0.071894
BGLF4	TFF2	0.16924
BGLF4	NEDD9	0.12893
BGLF4	CNOT1	0.17086
BGLF4	RPL14	0.16495
BGLF4	PTPN9	0.07893
BGLF4	ATF7	0.041057
BGLF4	GUCY2C	0.029613
BGLF4	HPCA	0.064188
BGLF4	SMC5	0.12384
BGLF4	ZRANB1	0.10291
BGLF4	DNASE1L2	0.053915
BGLF4	PTPRO	0.041859
BGLF4	KCNJ4	0.22307
BGLF4	ANK3	0.077605
BGLF4	SYPL1	0.023562
BGLF4	DDX6	0.14167
BGLF4	APOBEC1	0.089151
BGLF4	ERLIN1	0.1949
BGLF4	NLRP3	0.093765
BGLF4	ZNF473	0.13487
BGLF4	RAD9B	0.21094
BGLF4	AHSA1	0.18561
BGLF4	GTF2A2	0.11031
BGLF4	VSIG4	0.20771
BGLF4	TOP1	0.07709
BGLF4	DNMT3B	0.088974
BGLF4	RAC3	0.076282
BGLF4	IGF2R	0.13631
BGLF4	SORCS1	0.20687
BGLF4	PYURF	0.20071
BGLF4	HRG	0.058865
BGLF4	ORC3	0.15317
BGLF4	SCYL3	0.1487
BGLF4	CCL18	0.18397
BGLF4	ARPC3	0.15389
BGLF4	SIT1	0.16484
BGLF4	NUAK1	0.05537
BGLF4	TXN	0.054056
BGLF4	DIABLO	0.15186
BGLF4	C14orf166	0.12609
BGLF4	PIK3R4	0.054939
BGLF4	XRCC1	0.15613
BGLF4	CETN1	0.28826
BGLF4	UBQLN4	0.16498
BGLF4	KCNJ9	0.21212
BGLF4	LPP	0.14024
BGLF4	AKAP12	0.17425
BGLF4	ZBTB25	0.065515
BGLF4	BCOR	0.1069
BGLF4	TTBK2	0.069111
BGLF4	MPHOSPH10	0.12851
BGLF4	MLLT10	0.060564
BGLF4	PFKFB2	0.060505
BGLF4	SGOL1	0.10287
BGLF4	CUL3	0.082572
BGLF4	NTNG1	0.19441
BGLF4	MC1R	0.096892
BGLF4	COG7	0.14798

BGLF4	PROCR	0.17486
BGLF4	ERC2	0.16918
BGLF4	ATP1B2	0.15536
BGLF4	ZSWIM7	0.1617
BGLF4	MAP2K2	0.015091
BGLF4	HLA-A	0.095719
BGLF4	GK	0.13539
BGLF4	GNB2	0.1236
BGLF4	HSPB9	0.20235
BGLF4	PSMC6	0.064746
BGLF4	NDFIP1	0.085263
BGLF4	STK10	0.066797
BGLF4	ANXA13	0.061603
BGLF4	HTRA2	0.096891
BGLF4	NUP98	0.09915
BGLF4	ANK2	0.034005
BGLF4	NFKBIA	0.036551
BGLF4	LAT	0.092381
BGLF4	SOD3	0.10112
BGLF4	SEPT6	0.1088
BGLF4	MYO5A	0.037267
BGLF4	CIC	0.19898
BGLF4	COMMD8	0.24432
BGLF4	IGFBP2	0.15554
BGLF4	DUSP2	0.043817
BGLF4	CCR5	0.074572
BGLF4	RPS13	0.12332
BGLF4	TAF8	0.13642
BGLF4	CRHR2	0.12467
BGLF4	IL1RAPL1	0.11092
BGLF4	PBX1	0.016472
BGLF4	HPN	0.10021
BGLF4	VLDLR	0.12374
BGLF4	NUP62	0.057452
BGLF4	INPPL1	0.068344
BGLF4	TRIP13	0.097727
BGLF4	CDH1	0.065917
BGLF4	HTT	0.030589
BGLF4	DNAJB4	0.17588
BGLF4	ADAMTS13	0.15775
BGLF4	BCAP31	0.06429
BGLF4	DIAPH1	0.072847
BGLF4	TEAD1	0.095062
BGLF4	SMO	0.010063
BGLF4	BCL6	0.028106
BGLF4	MAP1LC3B	0.21603
BGLF4	USF2	0.074384
BGLF4	UBA5	0.083662
BGLF4	TTN	0.039208
BGLF4	ZDHHC17	0.171
BGLF4	MICU3	0.064189
BGLF4	CAPNS2	0.062342
BGLF4	ABT1	0.17931
BGLF4	NDRG2	0.10685
BGLF4	TWIST2	0.042895
BGLF4	FSTL4	0.066109
BGLF4	POLB	0.06682
BGLF4	ACTR3	0.044961
BGLF4	DCPS	0.094848
BGLF4	TUT1	0.12231
BGLF4	PSMC5	0.051868
BGLF4	NCALD	0.059525
BGLF4	MUS81	0.12777
BGLF4	PRDM16	0.094987
BGLF4	EGF	0.12609
BGLF4	RAPGEF2	0.040418
BGLF4	TFAM	0.08809
BGLF4	TJP1	0.13542

BGLF4	CBX1	0.10729
BGLF4	UIMC1	0.14935
BGLF4	HOMER3	0.16278
BGLF4	AJUBA	0.047863
BGLF4	MIEF1	0.16033
BGLF4	TIRAP	0.060171
BGLF4	PDK3	0.1074
BGLF4	PALM3	0.15377
BGLF4	FYN	0.026863
BGLF4	PRKAB2	0.050316
BGLF4	USP26	0.17221
BGLF4	PPP1R16B	0.18065
BGLF4	HIVEP1	0.13844
BGLF4	ELOVL5	0.13396
BGLF4	LTBP4	0.28119
BGLF4	BLOC1S6	0.094175
BGLF4	MOAP1	0.22418
BGLF4	RANBP2	0.075231
BGLF4	PRMT2	0.13696
BGLF4	RPS19	0.12843
BGLF4	DCAF16	0.23579
BGLF4	SMARCAD1	0.095914
BGLF4	CDKN1B	0.058052
BGLF4	NPHP4	0.088127
BGLF4	GNB2L1	0.026912
BGLF4	NUP43	0.11759
BGLF4	AMELX	0.12352
BGLF4	PLSCR4	0.33864
BGLF4	LMNA	0.091682
BGLF4	INSIG1	0.1183
BGLF4	HIRIP3	0.20848
BGLF4	CENPF	0.055334
BGLF4	LIN37	0.22971
BGLF4	MPP7	0.18487
BGLF4	VAMP2	0.13705
BGLF4	RBM8A	0.15554
BGLF4	ALKBH3	0.1559
BGLF4	ZFAND6	0.13809
BGLF4	SHANK1	0.094203
BGLF4	DCP2	0.1486
BGLF4	CCNK	0.13485
BGLF4	FBXL2	0.20589
BGLF4	SCOC	0.1559
BGLF4	LAMB3	0.09893
BGLF4	MBD4	0.10821
BGLF4	RXRB	0.060547
BGLF4	FBXO25	0.18918
BGLF4	KAT8	0.10308
BGLF4	POLD3	0.073988
BGLF4	NID1	0.056806
BGLF4	MEPE	0.22042
BGLF4	GOLGB1	0.18007
BGLF4	GALNT3	0.017466
BGLF4	HBA1	0.15992
BGLF4	NXF1	0.16875
BGLF4	GMIP	0.15312
BGLF4	C1R	0.097833
BGLF4	TUBG1	0.10035
BGLF4	SLC6A2	0.2165
BGLF4	AKR1B10	0.20278
BGLF4	NOP2	0.27652
BGLF4	DNAJC1	0.1132
BGLF4	FCGBP	0.22977
BGLF4	ZC3H11A	0.19428
BGLF4	TSHZ3	0.032174
BGLF4	CASC5	0.079094
BGLF4	SERPINC1	0.1505
BGLF4	FN1	0.097918

BGLF4	ACE	0.024561
BGLF4	SRSF2	0.17459
BGLF4	DMP1	0.02421
BGLF4	RHOT1	0.18574
BGLF4	DDX39A	0.12837
BGLF4	SAP18	0.16394
BGLF4	SAFB	0.16299
BGLF4	ASB13	0.22645
BGLF4	TEAD4	0.062566
BGLF4	SNX9	0.20305
BGLF4	NLGN4X	0.09379
BGLF4	COL4A3	0.057385
BGLF4	CITED2	0.029834
BGLF4	CSRP3	0.086466
BGLF4	VPS53	0.20143
BGLF4	TTI1	0.24432
BGLF4	NCBP2	0.099316
BGLF4	ZC3H15	0.17452
BGLF4	IFRD2	0.2311
BGLF4	TNPO1	0.14055
BGLF4	LSM1	0.16126
BGLF4	COPS4	0.19166
BGLF4	ABI3	0.20001
BGLF4	UBXN4	0.16699
BGLF4	NLRP12	0.11676
BGLF4	CSF2	0.1309
BGLF4	PADI6	0.018373
BGLF4	DNAJC10	0.090184
BGLF4	PARN	0.10892
BGLF4	FAM175A	0.18635
BGLF4	ZCCHC8	0.15355
BGLF4	TRAF3	0.035823
BGLF4	RYR2	0.09451
BGLF4	DLAT	0.17915
BGLF4	ACTR5	0.15738
BGLF4	AXL	0.034383
BGLF4	RAET1E	0.19672
BGLF4	SMC6	0.13881
BGLF4	PARP9	0.2516
BGLF4	OPRM1	0.067973
BGLF4	GTPBP4	0.086867
BGLF4	CRLF1	0.1973
BGLF4	PRF1	0.23258
BGLF4	KIAA1109	0.19097
BGLF4	CACNA1I	0.16819
BGLF4	TFF1	0.17412
BGLF4	PCDHB3	0.050061
BGLF4	RPS6KA4	0.019227
BGLF4	PPP1R13L	0.10558
BGLF4	HSD17B14	0.16044
BGLF4	EXOC4	0.073537
BGLF4	SEPT12	0.12675
BGLF4	CUL7	0.14429
BGLF4	TPT1	0.015028
BGLF4	CDC16	0.12134
BGLF4	IL6ST	0.11428
BGLF4	CDC20	0.084703
BGLF4	STRN	0.15296
BGLF4	MYO1E	0.13226
BGLF4	SPRY4	0.18921
BGLF4	ZNF366	0.14053
BGLF4	DDX20	0.080712
BGLF4	TEN1	0.17821
BGLF4	NKX3-1	0.010153
BGLF4	UBE2V1	0.079633
BGLF4	TRMT10C	0.23549
BGLF4	OCA2	0.12415
BGLF4	IGF1R	0.028132

BGLF4	PLA2G2C	0.034765
BGLF4	DGKZ	0.093612
BGLF4	SNRPA1	0.16877
BGLF4	LAIR1	0.22282
BGLF4	MICU2	0.066162
BGLF4	F2RL2	0.13501
BGLF4	VEGFB	0.093995
BGLF4	NHLRC1	0.11498
BGLF4	GFER	0.18838
BGLF4	SOCS2	0.12699
BGLF4	ZNF423	0.12842
BGLF4	MZT1	0.19085
BGLF4	IGF2BP1	0.078405
BGLF4	RNF144B	0.12285
BGLF4	PPP1R12C	0.23693
BGLF4	MCFD2	0.0511
BGLF4	CCDC28B	0.22953
BGLF4	MYL12B	0.030873
BGLF4	EIF3K	0.19445
BGLF4	PI4KA	0.14396
BGLF4	CBY1	0.15578
BGLF4	SLC25A24	0.060535
BGLF4	SGCE	0.047318
BGLF4	MAP3K4	0.047441
BGLF4	GPR143	0.10333
BGLF4	CBS	0.033432
BGLF4	TIMM9	0.132
BGLF4	LNX1	0.12748
BGLF4	PKD2L2	0.068841
BGLF4	VDAC2	0.097657
BGLF4	IL13	0.10125
BGLF4	USP22	0.0945
BGLF4	SHARPIN	0.10185
BGLF4	OLFM1	0.20525
BGLF4	SART3	0.18184
BGLF4	PRPF40A	0.15939
BGLF4	LSM4	0.16198
BGLF4	WNK3	0.059946
BGLF4	FBXO11	0.11127
BGLF4	SNRNP70	0.17291
BGLF4	LDLRAP1	0.083803
BGLF4	EPHB4	0.069959
BGLF4	PCSK9	0.055907
BGLF4	TRA2B	0.21339
BGLF4	DGKI	0.1299
BGLF4	HSPB7	0.18152
BGLF4	NOP10	0.15738
BGLF4	ELANE	0.12141
BGLF4	RING1	0.085725
BGLF4	ZNF496	0.064084
BGLF4	NELFA	0.16252
BGLF4	ACVR1B	0.020409
BGLF4	TCEB2	0.14414
BGLF4	TRIM23	0.025968
BGLF4	MYPN	0.17176
BGLF4	BRD3	0.17159
BGLF4	SETD2	0.14375
BGLF4	ZRANB2	0.11624
BGLF4	TRIM32	0.087601
BGLF4	PLD2	0.032848
BGLF4	YWHAH	0.11379
BGLF4	HEMGN	0.19054
BGLF4	PARP4	0.15425
BGLF4	CNEP1R1	0.13304
BGLF4	HNRNPM	0.16012
BGLF4	KRT6A	0.15747
BGLF4	KLRF2	0.21212
BGLF4	AKAP17A	0.19097

BGLF4	PCDHA6	0.053108
BGLF4	SERPINB8	0.19767
BGLF4	MED28	0.16373
BGLF4	PLEC	0.14304
BGLF4	FADD	0.082019
BGLF4	TULP3	0.15901
BGLF4	ARRDC3	0.22309
BGLF4	TSC22D4	0.090935
BGLF4	RPS6KA5	0.015842
BGLF4	SERBP1	0.23258
BGLF4	TGFBRAP1	0.17973
BGLF4	FHOD1	0.15428
BGLF4	XRCC4	0.084552
BGLF4	ZFC3H1	0.20393
BGLF4	TP53BP2	0.13855
BGLF4	RNF187	0.11299
BGLF4	E2F3	0.071914
BGLF4	ZNF711	0.14648
BGLF4	PTPN2	0.06729
BGLF4	STIP1	0.16043
BGLF4	MALT1	0.06421
BGLF4	SUPT5H	0.093257
BGLF4	ELMO2	0.078528
BGLF4	ATP6V0A2	0.14256
BGLF4	SNCA	0.022615
BGLF4	FBXO27	0.22789
BGLF4	EMR3	0.03049
BGLF4	CARD10	0.19557
BGLF4	UBE3C	0.12342
BGLF4	RANBP6	0.19956
BGLF4	MCIDAS	0.14165
BGLF4	S100A8	0.14738
BGLF4	THAP11	0.15707
BGLF4	MT-ND3	0.18015
BGLF4	CD70	0.127
BGLF4	IL2RG	0.15006
BGLF4	DUSP26	0.030509
BGLF4	NID2	0.39033
BGLF4	SYP	0.064825
BGLF4	F2R	0.027941
BGLF4	CCNB1	0.034168
BGLF4	CADM1	0.074206
BGLF4	DIS3L	0.19178
BGLF4	NELL1	0.26673
BGLF4	DIRAS3	0.1431
BGLF4	TICRR	0.17081
BGLF4	FAM84B	0.22977
BGLF4	SLX1A	0.15036
BGLF4	ZNF365	0.19933
BGLF4	DEDD	0.15575
BGLF4	ZHX1	0.060648
BGLF4	ANXA1	0.048906
BGLF4	FAF2	0.20604
BGLF4	CDKN2B	0.070057
BGLF4	PFDN5	0.14597
BGLF4	ZMYND11	0.11329
BGLF4	CLDN7	0.09569
BGLF4	KRAS	0.064097
BGLF4	PTPRE	0.054561
BGLF4	C3	0.093867
BGLF4	PCDHB16	0.053256
BGLF4	PTPRK	0.033561
BGLF4	VAV1	0.011941
BGLF4	SNTN	0.064239
BGLF4	SIRT3	0.1302
BGLF4	ADA	0.034297
BGLF4	CRHBP	0.0741
BGLF4	DNER	0.26664

BGLF4	FBXO17	0.22789
BGLF4	COL17A1	0.15322
BGLF4	PRKAR1A	0.098183
BGLF4	EIF6	0.23819
BGLF4	CD72	0.14159
BGLF4	VSNL1	0.068073
BGLF4	RUVBL2	0.057839
BGLF4	FBXL14	0.22492
BGLF4	PCDHGA6	0.058173
BGLF4	CLN5	0.16077
BGLF4	CFL1	0.086157
BGLF4	MBD1	0.075918
BGLF4	MKL2	0.16116
BGLF4	SCNN1G	0.15122
BGLF4	PHLDA1	0.20268
BGLF4	NDUFS3	0.15449
BGLF4	POU2F1	0.077004
BGLF4	SLC25A10	0.10934
BGLF4	ATP8A1	0.13412
BGLF4	HMG20A	0.096494
BGLF4	TLX1	0.072694
BGLF4	IL17RA	0.15986
BGLF4	CDK4	0.012956
BGLF4	UBE2V2	0.12726
BGLF4	EFCAB2	0.068073
BGLF4	DSCAM	0.14945
BGLF4	NUCB1	0.065213
BGLF4	SAFB2	0.21587
BGLF4	CYTH1	0.13049
BGLF4	HEYL	0.045079
BGLF4	ELOVL6	0.14563
BGLF4	EPDR1	0.064124
BGLF4	PPARGC1A	0.061496
BGLF4	NRXN1	0.12891
BGLF4	PEF1	0.40779
BGLF4	OFD1	0.11919
BGLF4	CD22	0.19628
BGLF4	RBM15B	0.17009
BGLF4	SHOX	0.08999
BGLF4	NOXA1	0.17418
BGLF4	DUX4L9	0.079192
BGLF4	ZWINT	0.12478
BGLF4	NAPG	0.18196
BGLF4	ZFYVE26	0.18183
BGLF4	RBBP8	0.1065
BGLF4	MAX	0.081253
BGLF4	ANXA3	0.04118
BGLF4	FBXL8	0.24432
BGLF4	SNRPE	0.13584
BGLF4	CREBBP	0.013704
BGLF4	CGN	0.20331
BGLF4	XIAP	0.11044
BGLF4	GCHFR	0.12624
BGLF4	PLA2G16	0.094875
BGLF4	EZR	0.10865
BGLF4	COL16A1	0.18086
BGLF4	PSMA3	0.053606
BGLF4	RAD17	0.1211
BGLF4	FBL	0.22496
BGLF4	TTYH1	0.047895
BGLF4	CNNM3	0.21303
BGLF4	RASGRP2	0.040061
BGLF4	TSSK6	0.048649
BGLF4	MEGF8	0.31129
BGLF4	MCU	0.15743
BGLF4	PDGFB	0.018758
BGLF4	PCDHGB5	0.058173
BGLF4	CHCHD6	0.23175

BGLF4	CDH13	0.025192
BGLF4	RNF168	0.074768
BGLF4	ATXN1	0.14378
BGLF4	FKBP9L	0.057372
BGLF4	TRIM25	0.017341
BGLF4	GAP43	0.15019
BGLF4	TTYH2	0.19426
BGLF4	BAK1	0.032513
BGLF4	BEND7	0.24432
BGLF4	CHP1	0.1344
BGLF4	PRICKLE3	0.18971
BGLF4	PAF1	0.13177
BGLF4	REEP5	0.21611
BGLF4	ANTXR2	0.16377
BGLF4	MAP4K2	0.037415
BGLF4	NFATC1	0.056712
BGLF4	CYFIP2	0.18454
BGLF4	CSTF2	0.17441
BGLF4	TNK2	0.045843
BGLF4	CAMTA2	0.11614
BGLF4	MAPK3	0.014578
BGLF4	ERAP1	0.086966
BGLF4	UBE2E1	0.074559
BGLF4	CHCHD1	0.19726
BGLF4	APTX	0.063559
BGLF4	VPS52	0.20769
BGLF4	MYL12A	0.068128
BGLF4	CACNG2	0.12548
BGLF4	DAP3	0.1015
BGLF4	ZGPAT	0.065129
BGLF4	RASSF3	0.18422
BGLF4	CFTR	0.083806
BGLF4	RAB3IP	0.14872
BGLF4	SPG7	0.060642
BGLF4	GNAQ	0.036515
BGLF4	CTSH	0.06839
BGLF4	XPO5	0.15824
BGLF4	ABCD2	0.13919
BGLF4	CD86	0.10035
BGLF4	EGFL6	0.060076
BGLF4	KMT2E	0.1281
BGLF4	IMP4	0.15593
BGLF4	PRPF31	0.17518
BGLF4	NOMO2	0.19116
BGLF4	SYMPK	0.16199
BGLF4	CUL5	0.10142
BGLF4	CNST	0.21779
BGLF4	PADI3	0.01888
BGLF4	COL6A2	0.14426
BGLF4	FOXG1	0.018669
BGLF4	TRAPPC10	0.20357
BGLF4	LIPE	0.066405
BGLF4	HAP1	0.15464
BGLF4	ATP6V1C1	0.16393
BGLF4	PSMB7	0.05682
BGLF4	RXRA	0.010622
BGLF4	AHCYL1	0.16831
BGLF4	TGM2	0.15137
BGLF4	RPLP0	0.14009
BGLF4	NR3C1	0.029415
BGLF4	BAG1	0.11316
BGLF4	ATF7IP	0.17073
BGLF4	MAPK10	0.034417
BGLF4	EPM2A	0.018438
BGLF4	FASN	0.11703
BGLF4	DLL4	0.031906
BGLF4	SEPT7	0.033862
BGLF4	SMUG1	0.14408

BGLF4	MPZL1	0.11304
BGLF4	BRCA2	0.04375
BGLF4	LCMT1	0.25963
BGLF4	COL1A1	0.043108
BGLF4	EID2	0.14222
BGLF4	ELOVL7	0.14988
BGLF4	RPS3A	0.14409
BGLF4	GPR98	0.19137
BGLF4	PDS5B	0.14818
BGLF4	CRY1	0.060123
BGLF4	NUMBL	0.21884
BGLF4	UBA3	0.054336
BGLF4	ACAA2	0.18517
BGLF4	DLG4	0.018108
BGLF4	LSM6	0.15245
BGLF4	MBIP	0.18706
BGLF4	E2F4	0.03046
BGLF4	MICU1	0.33446
BGLF4	CALU	0.33309
BGLF4	EIF2S1	0.1695
BGLF4	TBRG1	0.17726
BGLF4	ELF2	0.078505
BGLF4	ATP1B1	0.14175
BGLF4	CLPTM1	0.19688
BGLF4	CEP63	0.17897
BGLF4	ZIC3	0.041231
BGLF4	ISCU	0.020117
BGLF4	RIPK1	0.014878
BGLF4	TICAM1	0.043377
BGLF4	AP2B1	0.10314
BGLF4	KIRREL3	0.20217
BGLF4	GPSM2	0.12393
BGLF4	PSMB5	0.054507
BGLF4	COIL	0.17298
BGLF4	ATXN2	0.11047
BGLF4	LILRB4	0.16029
BGLF4	PADI2	0.038208
BGLF4	XPC	0.11061
BGLF4	CD8A	0.085189
BGLF4	SSX2	0.19341
BGLF4	LENG8	0.24432
BGLF4	PRKRA	0.15727
BGLF4	KLK8	0.18955
BGLF4	PTPN13	0.051575
BGLF4	PPP3CA	0.12467
BGLF4	STRADB	0.0313
BGLF4	MTUS2	0.21127
BGLF4	ANXA8L2	0.048161
BGLF4	FBLN2	0.070964
BGLF4	BCL2A1	0.21608
BGLF4	PADI4	0.010123
BGLF4	HAND2	0.042098
BGLF4	PMEL	0.15498
BGLF4	TACR1	0.047621
BGLF4	SURF4	0.14275
BGLF4	MYOZ2	0.19445
BGLF4	VCP	0.053119
BGLF4	APIP	0.10133
BGLF4	USP28	0.14191
BGLF4	PPP1CA	0.051793
BGLF4	ATF6	0.031248
BGLF4	ITK	0.034666
BGLF4	HOXC13	0.081423
BGLF4	GADD45G	0.16078
BGLF4	SIX1	0.014721
BGLF4	BTG1	0.12745
BGLF4	MCTP1	0.061609
BGLF4	CBFB	0.072365

BGLF4	OTUB1	0.14302
BGLF4	PRKX	0.041817
BGLF4	MOCS3	0.0966
BGLF4	CCNG1	0.14648
BGLF4	VSTM2L	0.24432
BGLF4	RFX6	0.13793
BGLF4	ZFYVE28	0.15924
BGLF4	FCGR2C	0.20948
BGLF4	AKAP6	0.088363
BGLF4	BZRAP1	0.21409
BGLF4	EIF4B	0.21004
BGLF4	ANXA10	0.065373
BGLF4	PCDHGB3	0.058173
BGLF4	GLS2	0.14252
BGLF4	RNF40	0.10179
BGLF4	SOX5	0.051543
BGLF4	POP1	0.14681
BGLF4	SELENBP1	0.1569
BGLF4	PVR	0.088952
BGLF4	NCOR2	0.12357
BGLF4	LRP2	0.13956
BGLF4	VMP1	0.14256
BGLF4	CAPN2	0.21078
BGLF4	PAFAH1B3	0.13402
BGLF4	KRT8	0.14951
BGLF4	SLC23A1	0.13931
BGLF4	OCLN	0.06141
BGLF4	SIRPB1	0.20281
BGLF4	CPSF7	0.1906
BGLF4	ABCG5	0.095228
BGLF4	UNC119	0.20989
BGLF4	ABL1	0.01848
BGLF4	DGCR8	0.18324
BGLF4	CLIC1	0.12373
BGLF4	MKRN3	0.1169
BGLF4	TTC1	0.21161
BGLF4	SUPT4H1	0.044824
BGLF4	SREBF2	0.04056
BGLF4	HNRNPC	0.15744
BGLF4	TRIM37	0.096711
BGLF4	GRN	0.15018
BGLF4	PABPC4	0.15448
BGLF4	SLC22A11	0.18303
BGLF4	VPS33A	0.14141
BGLF4	TRIM5	0.088643
BGLF4	ANTXR1	0.1447
BGLF4	TNFRSF11A	0.0847
BGLF4	NPLOC4	0.13263
BGLF4	NAA10	0.19058
BGLF4	RGS2	0.11093
BGLF4	CTSL	0.12615
BGLF4	MON1B	0.24432
BGLF4	TIFA	0.21491
BGLF4	NUCB2	0.28911
BGLF4	TF	0.13018
BGLF4	CBR4	0.19612
BGLF4	RAD9A	0.089658
BGLF4	CBX6	0.18159
BGLF4	MRPL51	0.1898
BGLF4	MSX2	0.024406
BGLF4	CDKN2A	0.045833
BGLF4	PKDREJ	0.055134
BGLF4	PPP1R13B	0.17829
BGLF4	DCC	0.11187
BGLF4	LRSAM1	0.11273
BGLF4	SOX1	0.093568
BGLF4	HELZ2	0.10689
BGLF4	EED	0.14621

BGLF4	ASGR1	0.18072
BGLF4	NPC2	0.1232
BGLF4	HOXA9	0.089195
BGLF4	MAGEA11	0.20848
BGLF4	SH3RF2	0.14805
BGLF4	RAP1GAP	0.12342
BGLF4	USH1C	0.11635
BGLF4	PCDH19	0.058173
BGLF4	RNPS1	0.17074
BGLF4	FAM162A	0.17638
BGLF4	SH3GLB1	0.090863
BGLF4	CDON	0.095972
BGLF4	BCL2L14	0.11869
BGLF4	PINX1	0.092712
BGLF4	PSMD5	0.10629
BGLF4	PEX11A	0.16961
BGLF4	RIPK3	0.026526
BGLF4	S100A6	0.25609
BGLF4	RP1L1	0.20324
BGLF4	EPHB1	0.04112
BGLF4	AOC1	0.04104
BGLF4	STK16	0.039692
BGLF4	VHL	0.079872
BGLF4	FAT2	0.039817
BGLF4	FBXW2	0.21444
BGLF4	PLCG2	0.04483
BGLF4	SMARCD3	0.052438
BGLF4	RNF20	0.080287
BGLF4	ZFP36	0.050708
BGLF4	PPP2R1A	0.053515
BGLF4	IPO13	0.18243
BGLF4	AFG3L2	0.051011
BGLF4	HENMT1	0.022364
BGLF4	EPB42	0.12574
BGLF4	WDR6	0.18954
BGLF4	DDC	0.10281
BGLF4	TERF2IP	0.08422
BGLF4	SPRED2	0.13909
BGLF4	ORMDL3	0.1949
BGLF4	TLR4	0.012246
BGLF4	PKHD1	0.10977
BGLF4	RNF19B	0.15111
BGLF4	IKBKAP	0.059063
BGLF4	MED11	0.1597
BGLF4	RABGEF1	0.14661
BGLF4	LRP8	0.2283
BGLF4	ZEB1	0.040392
BGLF4	NOX5	0.04823
BGLF4	SYNPO	0.1473
BGLF4	MAVS	0.024677
BGLF4	IGFLR1	0.22282
BGLF4	CASP2	0.09235
BGLF4	RFC5	0.07718
BGLF4	PPP6R3	0.1923
BGLF4	KRT14	0.098575
BGLF4	CBFA2T2	0.057688
BGLF4	GABRG2	0.11852
BGLF4	NUAK2	0.048395
BGLF4	NXT1	0.16138
BGLF4	S100P	0.35416
BGLF4	SNAI1	0.057662
BGLF4	BCL10	0.018793
BGLF4	CARD8	0.18218
BGLF4	PLK3	0.028708
BGLF4	SEMA4D	0.055188
BGLF4	SMURF1	0.075079
BGLF4	SIX5	0.08588
BGLF4	TMEM67	0.14761

BGLF4	WASL	0.065714
BGLF4	LRP1B	0.057243
BGLF4	DCAF7	0.19081
BGLF4	SGTA	0.20958
BGLF4	SLC18A1	0.011374
BGLF4	XPO6	0.17257
BGLF4	TRAK1	0.1422
BGLF4	SERTAD1	0.20979
BGLF4	PCDHGA5	0.058173
BGLF4	RNF103	0.12405
BGLF4	CLASP1	0.065823
BGLF4	DMD	0.086652
BGLF4	E2F2	0.039663
BGLF4	LCA5	0.18691
BGLF4	INTS4	0.20196
BGLF4	DYNLRB1	0.15688
BGLF4	GYPC	0.20938
BGLF4	SEPT1	0.17421
BGLF4	EGLN2	0.12872
BGLF4	PRDX6	0.19902
BGLF4	WT1	0.018243
BGLF4	YWHAZ	0.082142
BGLF4	CHAF1A	0.20216
BGLF4	BTF3	0.20227
BGLF4	KIAA0319L	0.22281
BGLF4	SLC22A1	0.1337
BGLF4	DAZ1	0.20127
BGLF4	SEPT9	0.1258
BGLF4	PPP2R5C	0.13025
BGLF4	TAF9	0.080551
BGLF4	PTCRA	0.22278
BGLF4	SETD1B	0.20391
BGLF4	RIMS2	0.16297
BGLF4	TP53INP1	0.17561
BGLF4	F7	0.02393
BGLF4	ELF4	0.081541
BGLF4	ECH1	0.13141
BGLF4	CPS1	0.035429
BGLF4	C4BPA	0.20578
BGLF4	TAPBP	0.064862
BGLF4	RAPGEF1	0.14987
BGLF4	FEM1B	0.15463
BGLF4	ATXN7L3	0.15733
BGLF4	FNBP1	0.21766
BGLF4	KMT2B	0.091767
BGLF4	MCM10	0.15181
BGLF4	KCNB1	0.14582
BGLF4	CASP9	0.13462
BGLF4	NR4A2	0.037732
BGLF4	VRK1	0.05123
BGLF4	LTN1	0.15264
BGLF4	TEFM	0.14028
BGLF4	MTOR	0.016068
BGLF4	PTTG1	0.11972
BGLF4	PLAU	0.12714
BGLF4	DZIP1	0.13294
BGLF4	CLUAP1	0.21497
BGLF4	MADD	0.16299
BGLF4	NUP160	0.13105
BGLF4	NUP50	0.14816
BGLF4	TRIO	0.020888
BGLF4	RAB11FIP3	0.2002
BGLF4	PROCA1	0.058753
BGLF4	MOB1B	0.13677
BGLF4	EFCAB4B	0.3298
BGLF4	APP	0.015172
BGLF4	CDHR1	0.037876
BGLF4	HPX	0.12013

BGLF4	CLNS1A	0.14884
BGLF4	UCHL1	0.069629
BGLF4	GAB2	0.076047
BGLF4	ALKBH8	0.19232
BGLF4	PARD6G	0.18215
BGLF4	CAMK1	0.021295
BGLF4	SYBU	0.2163
BGLF4	UTP14A	0.18583
BGLF4	PCDHB8	0.058173
BGLF4	NOC4L	0.18138
BGLF4	YOD1	0.1375
BGLF4	GABPA	0.058216
BGLF4	ARL6IP1	0.23987
BGLF4	TMPRSS6	0.19198
BGLF4	BLOC1S4	0.18479
BGLF4	THAP3	0.20471
BGLF4	FAM21C	0.18835
BGLF4	S100A7A	0.047037
BGLF4	ZNF256	0.06147
BGLF4	MUC17	0.15484
BGLF4	AP2A1	0.1002
BGLF4	TDGF1	0.050507
BGLF4	ZYX	0.11779
BGLF4	EFCAB3	0.068073
BGLF4	ANKZF1	0.19427
BGLF4	PFDN2	0.16462
BGLF4	PRRG2	0.060357
BGLF4	STK11	0.01465
BGLF4	DLGAP2	0.17358
BGLF4	SMOC2	0.04442
BGLF4	STXBP3	0.11232
BGLF4	TJP3	0.19537
BGLF4	SALL1	0.032449
BGLF4	SERPING1	0.14661
BGLF4	HIP1R	0.14235
BGLF4	THOC1	0.176
BGLF4	YTHDC2	0.15924
BGLF4	RAD51B	0.137
BGLF4	HNRNPA2B1	0.15745
BGLF4	CCT2	0.11141
BGLF4	FOSL1	0.046254
BGLF4	SETD6	0.22069
BGLF4	MTPAP	0.12437
BGLF4	CASR	0.14377
BGLF4	YWHAE	0.12562
BGLF4	EDEM3	0.026815
BGLF4	TPO	0.083048
BGLF4	GLA	0.079673
BGLF4	NDUFA13	0.1008
BGLF4	CHGB	0.22818
BGLF4	PRKD3	0.014145
BGLF4	DTNA	0.28681
BGLF4	REPS2	0.4314
BGLF4	IMP3	0.17464
BGLF4	OPRK1	0.12603
BGLF4	SH3BP4	0.21737
BGLF4	MVP	0.16094
BGLF4	SASS6	0.1911
BGLF4	PPP3R1	0.080491
BGLF4	RRAGB	0.12203
BGLF4	CCDC50	0.23693
BGLF4	CDH24	0.060423
BGLF4	TRIM44	0.19427
BGLF4	ARFGEF1	0.079214
BGLF4	PPEF2	0.21977
BGLF4	EFEMP2	0.39626
BGLF4	DVL3	0.10032
BGLF4	IFNA2	0.13953

BGLF4	MAP4	0.09236
BGLF4	MYLK	0.042129
BGLF4	ZC3H13	0.19428
BGLF4	ALX1	0.079509
BGLF4	SART1	0.15349
BGLF4	MT-CO1	0.090971
BGLF4	AUNIP	0.19592
BGLF4	TANK	0.16816
BGLF4	HERC5	0.10811
BGLF4	DLEU1	0.24432
BGLF4	EIF3L	0.23386
BGLF4	STARD13	0.16542
BGLF4	ATCAY	0.15118
BGLF4	MAP1B	0.024324
BGLF4	HNRNPH1	0.14766
BGLF4	IFI6	0.17588
BGLF4	PFN2	0.16876
BGLF4	USP33	0.087173
BGLF4	DPH3	0.1643
BGLF4	ATP2A1	0.11593
BGLF4	CCS	0.10672
BGLF4	POGZ	0.13222
BGLF4	SIK2	0.037116
BGLF4	PCDH15	0.012325
BGLF4	TCEA1	0.099437
BGLF4	TRRAP	0.14291
BGLF4	FZD4	0.054807
BGLF4	RPA2	0.076921
BGLF4	EXOSC4	0.1197
BGLF4	CSE1L	0.18139
BGLF4	RNF10	0.1158
BGLF4	SCAMP5	0.14796
BGLF4	PADI1	0.038208
BGLF4	HIST3H3	0.10705
BGLF4	CAB39	0.11457
BGLF4	WLS	0.24168
BGLF4	NOC2L	0.12922
BGLF4	ATP6V0A4	0.1037
BGLF4	PPP2R1B	0.23696
BGLF4	ITGA2	0.16214
BGLF4	CEP250	0.08346
BGLF4	EIF3D	0.23012
BGLF4	PDGFRL	0.039633
BGLF4	CXorf40A	0.24432
BGLF4	HNRNPD	0.12313
BGLF4	BCL2L1	0.013372
BGLF4	MYL3	0.049825
BGLF4	AGXT	0.12354
BGLF4	BAG6	0.10244
BGLF4	IL15	0.089986
BGLF4	CHD9	0.10936
BGLF4	NEUROD1	0.017523
BGLF4	CSF2RB	0.1644
BGLF4	RMDN3	0.090483
BGLF4	CD47	0.14238
BGLF4	CHMP3	0.13562
BGLF4	FOS	0.018306
BGLF4	PBK	0.078846
BGLF4	RFC4	0.079523
BGLF4	HOOK2	0.13695
BGLF4	ZC3H12A	0.12634
BGLF4	CCP110	0.18007
BGLF4	SMG7	0.15301
BGLF4	CD200	0.18419
BGLF4	LSM2	0.083371
BGLF4	AP3M1	0.18319
BGLF4	DYNC1I1	0.11084
BGLF4	ZNF703	0.10013

BGLF4	NFYB	0.086301
BGLF4	SH3GL3	0.18981
BGLF4	DMBT1	0.094169
BGLF4	MINK1	0.038988
BGLF4	PKN2	0.028715
BGLF4	MECOM	0.037849
BGLF4	LONP1	0.095888
BGLF4	PDCL3	0.20955
BGLF4	JAM3	0.11103
BGLF4	ASB16	0.22645
BGLF4	MLH3	0.10159
BGLF4	ARHGAP17	0.16384
BGLF4	PKD2L1	0.22211
BGLF4	MOB1A	0.20559
BGLF4	APOL1	0.16775
BGLF4	ADRB3	0.068304
BGLF4	TCEB1	0.13413
BGLF4	VPS4B	0.087427
BGLF4	FEZ2	0.21226
BGLF4	NFYA	0.085091
BGLF4	RNF111	0.084178
BGLF4	DROSHA	0.12513
BGLF4	CD2BP2	0.18741
BGLF4	NINL	0.055831
BGLF4	CD2	0.14569
BGLF4	ACOT8	0.092361
BGLF4	TPPP3	0.066538
BGLF4	NCOA6	0.090467
BGLF4	SMOC1	0.058646
BGLF4	SPPL2B	0.11194
BGLF4	ATR	0.050111
BGLF4	DMPK	0.011965
BGLF4	HTN1	0.20982
BGLF4	TRIM3	0.15132
BGLF4	EDC4	0.12472
BGLF4	PAX9	0.17451
BGLF4	ZNF408	0.13003
BGLF4	BTN3A1	0.19772
BGLF4	BIK	0.1374
BGLF4	PLEKHA1	0.069065
BGLF4	DAB2	0.082127
BGLF4	OR2L13	0.16625
BGLF4	HGS	0.099874
BGLF4	ARHGEF11	0.05553
BGLF4	SLC9A3R1	0.061252
BGLF4	TOPBP1	0.1441
BGLF4	BOLL	0.20402
BGLF4	MYLK2	0.016664
BGLF4	RYBP	0.12535
BGLF4	EHD3	0.21514
BGLF4	TUBB4A	0.032796
BGLF4	DYRK1A	0.025256
BGLF4	TGM1	0.20962
BGLF4	JAK1	0.059196
BGLF4	IGF2	0.093516
BGLF4	HSPA2	0.14804
BGLF4	FAM64A	0.23815
BGLF4	KIAA2018	0.038448
BGLF4	OCIAD1	0.22044
BGLF4	BCR	0.055752
BGLF4	SRCIN1	0.09261
BGLF4	TMEM8B	0.15342
BGLF4	SLC14A2	0.18894
BGLF4	ZZEF1	0.035943
BGLF4	AGER	0.099794
BGLF4	RICTOR	0.14861
BGLF4	MYL10	0.066162
BGLF4	PDGFRA	0.015014

44949	BGLF4	LY96	0.12207
44950	BGLF4	UBE2M	0.10364
44951	BGLF4	NF2	0.062696
44952	BGLF4	MICALL1	0.18841
44953	BGLF4	CSK	0.049171
44954	BGLF4	SMAD2	0.020211
44955	BGLF4	RPS2	0.14164
44956	BGLF4	UTF1	0.15701
44957	BGLF4	EXOSC8	0.17652
44958	BGLF4	IL4R	0.13515
44959	BGLF4	NR3C2	0.086968
44960	BGLF4	KRT15	0.17506
44961	BGLF4	DUT	0.13099
44962	BGLF4	NTN1	0.15715
44963	BGLF4	ZXDA	0.059449
44964	BGLF4	MAGEC1	0.24432
44965	BGLF4	POU2F3	0.06476
44966	BGLF4	P4HTM	0.051878
44967	BGLF4	EFCAB11	0.068073
44968	BGLF4	MT1G	0.10888
44969	BGLF4	DLGAP5	0.13172
44970	BGLF4	ATRX	0.10141
44971	BGLF4	FKBP2	0.19295
44972	BGLF4	TMEM102	0.18522
44973	BGLF4	CDC26	0.15498
44974	BGLF4	RNF6	0.098214
44975	BGLF4	TAF9B	0.1167
44976	BGLF4	DTL	0.13446
44977	BGLF4	UVRAG	0.17716
44978	BGLF4	COPB1	0.086957
44979	BGLF4	KIF6	0.15748
44980	BGLF4	THBS2	0.36828
44981	BGLF4	OIT3	0.042856
44982	BGLF4	EPS15L1	0.032823
44983	BGLF4	PELI2	0.147
44984	BGLF4	NELFB	0.16402
44985	BGLF4	FAP	0.13669
44986	BGLF4	SLIT1	0.018254
44987	BGLF4	NAF1	0.17991
44988	BGLF4	MLLT3	0.17291
44989	BGLF4	PRAME	0.14685
44990	BGLF4	CALM1	0.15045
44991	BGLF4	CRYAB	0.072923
44992	BGLF4	SMARCA5	0.050269
44993	BGLF4	ASAP2	0.048999
44994	BGLF4	EIF4E2	0.26459
44995	BGLF4	PROM1	0.13415
44996	BGLF4	STAMBPL1	0.18923
44997	BGLF4	MT-RNR2	0.12823
44998	BGLF4	ACVR2B	0.01768
44999	BGLF4	DBNL	0.13422
45000	BGLF4	FGF3	0.1411
45001	BGLF4	E2F1	0.030849
45002	BGLF4	STRN4	0.22284
45003	BGLF4	AVPR1A	0.074931
45004	BGLF4	RRP8	0.1608
45005	BGLF4	HPRT1	0.062131
45006	BGLF4	YWHAG	0.15683
45007	BGLF4	NPM3	0.19023
45008	BGLF4	DNASE1	0.14327
45009	BGLF4	RNF216	0.10287
45010	BGLF4	MAGI2	0.072811
45011	BGLF4	EPOR	0.14719
45012	BGLF4	STRIP1	0.19004
45013	BGLF4	EIF2A	0.12709
45014	BGLF4	KCTD5	0.18232
45015	BGLF4	CASP1	0.069766
45016	BGLF4	ZRANB3	0.05563

BGLF4	GLUD1	0.10585
BGLF4	TFAP2C	0.030826
BGLF4	FLT3	0.054376
BGLF4	EXOC2	0.20346
BGLF4	ATG12	0.19233
BGLF4	RARG	0.027127
BGLF4	KIF1B	0.15432
BGLF4	CAV3	0.043212
BGLF4	SMARCB1	0.092533
BGLF4	CDHR3	0.058173
BGLF4	YEATS2	0.17888
BGLF4	KPNA5	0.18458
BGLF4	ARHGAP10	0.076756
BGLF4	S100A4	0.38326
BGLF4	ERCC5	0.09689
BGLF4	ABCG2	0.14334
BGLF4	GUCA1B	0.067297
BGLF4	NAPA	0.13805
BGLF4	TNPO2	0.18241
BGLF4	COL4A1	0.15413
BGLF4	DNAJC21	0.17914
BGLF4	S100A12	0.21664
BGLF4	FYCO1	0.23762
BGLF4	RGS16	0.23077
BGLF4	WNT1	0.035246
BGLF4	RAB9B	0.17362
BGLF4	C19orf80	0.23694
BGLF4	RCN1	0.41933
BGLF4	RASGRP3	0.012039
BGLF4	NUDT14	0.21259
BGLF4	PHC2	0.14803
BGLF4	MTMR2	0.037925
BGLF4	BMPR2	0.017579
BGLF4	IKBKE	0.039583
BGLF4	DDX39B	0.10709
BGLF4	WNT3A	0.018209
BGLF4	BAZ2B	0.16117
BGLF4	CASP8	0.034739
BGLF4	NEFL	0.12171
BGLF4	KCNJ6	0.17134
BGLF4	MYH2	0.11363
BGLF4	FKBP7	0.06649
BGLF4	MZB1	0.18618
BGLF4	LRRFIP1	0.13733
BGLF4	PPP2R3C	0.042118
BGLF4	TNFRSF6B	0.21047
BGLF4	SMAD6	0.048366
BGLF4	TROAP	0.22914
BGLF4	MT1H	0.14905
BGLF4	RAPGEF4	0.069345
BGLF4	PSIP1	0.14771
BGLF4	SNRPD2	0.11541
BGLF4	SSX2IP	0.20356
BGLF4	ACD	0.10395
BGLF4	DPP4	0.12085
BGLF4	RERE	0.078353
BGLF4	SPSB4	0.22645
BGLF4	EIF3E	0.17049
BGLF4	EIF4E	0.16525
BGLF4	DSCC1	0.15844
BGLF4	RAMP1	0.098313
BGLF4	BBC3	0.076671
BGLF4	HSPBP1	0.20184
BGLF4	SDC2	0.10781
BGLF4	AK6	0.18962
BGLF4	HRAS	0.026124
BGLF4	EMILIN2	0.2164
BGLF4	SDIM1	0.22642

BGLF4	AIM2	0.081191
BGLF4	CLTCL1	0.16692
BGLF4	NPHP1	0.10356
BGLF4	CHD7	0.049103
BGLF4	SLC15A2	0.15573
BGLF4	DNMT3A	0.13268
BGLF4	ATP6V1F	0.13958
BGLF4	OAS3	0.1152
BGLF4	INHBA	0.039743
BGLF4	CDH2	0.013604
BGLF4	RPS23	0.11492
BGLF4	RQCD1	0.15902
BGLF4	PAG1	0.18238
BGLF4	NPB	0.19823
BGLF4	PIM3	0.061591
BGLF4	ACTN3	0.35297
BGLF4	ANXA4	0.059596
BGLF4	ATP5F1	0.20699
BGLF4	FBN1	0.35201
BGLF4	NR4A1	0.041524
BGLF4	RNF182	0.11367
BGLF4	MIF4GD	0.21841
BGLF4	MAP4K3	0.065362
BGLF4	TNNI2	0.173
BGLF4	PDPK1	0.013422
BGLF4	RAD1	0.11788
BGLF4	ATG14	0.20199
BGLF4	F10	0.23369
BGLF4	CAPN1	0.26788
BGLF4	PAX3	0.05812
BGLF4	UCKL1	0.17267
BGLF4	PLXDC1	0.15957
BGLF4	HSPB8	0.084253
BGLF4	MED15	0.14295
BGLF4	DSG1	0.034315
BGLF4	CEACAM6	0.2014
BGLF4	GSTZ1	0.19737
BGLF4	TLE1	0.091102
BGLF4	SKIL	0.08738
BGLF4	LILRB3	0.18648
BGLF4	ZNF3	0.042518
BGLF4	ZNF346	0.1711
BGLF4	RPSA	0.068674
BGLF4	SRPK1	0.032399
BGLF4	IPO7	0.13818
BGLF4	TRIM24	0.013069
BGLF4	CFL2	0.20848
BGLF4	ACAA1	0.12632
BGLF4	DPM2	0.13493
BGLF4	GHRHR	0.068059
BGLF4	CX3CR1	0.088766
BGLF4	PCDHA8	0.05466
BGLF4	PSMG2	0.18468
BGLF4	NDOR1	0.13614
BGLF4	PPM1A	0.0372
BGLF4	LETMD1	0.16527
BGLF4	SYNM	0.14914
BGLF4	PPP2R5E	0.14904
BGLF4	SCGN	0.12582
BGLF4	SAE1	0.06478
BGLF4	TSN	0.21994
BGLF4	VPS51	0.18269
BGLF4	CELSR3	0.27638
BGLF4	IARS	0.056786
BGLF4	CAND1	0.174
BGLF4	FCHSD2	0.24432
BGLF4	CATSPER1	0.12266
BGLF4	RILP	0.19602

BGLF4	ING5	0.12377
BGLF4	SENP2	0.1208
BGLF4	RCOR3	0.19222
BGLF4	MAPKAPK5	0.035398
BGLF4	LMAN1	0.069072
BGLF4	UBE2B	0.054248
BGLF4	MRPL48	0.19035
BGLF4	SDHA	0.19429
BGLF4	NR2C2	0.045672
BGLF4	WASF1	0.13498
BGLF4	CTIF	0.2316
BGLF4	RNF115	0.14402
BGLF4	SYNJ2	0.12407
BGLF4	KIAA1524	0.21609
BGLF4	ZNRF3	0.11087
BGLF4	TCEAL5	0.20561
BGLF4	PEX14	0.091513
BGLF4	CACNA1A	0.03488
BGLF4	AGO3	0.17669
BGLF4	RPGRIP1	0.1838
BGLF4	SPOCK1	0.074754
BGLF4	N6AMT1	0.27691
BGLF4	POLR2E	0.063138
BGLF4	S100A5	0.035309
BGLF4	POLR2J	0.073132
BGLF4	CD4	0.052539
BGLF4	ARF1	0.062504
BGLF4	GEMIN8	0.18546
BGLF4	CERS2	0.039914
BGLF4	IGFL1	0.23002
BGLF4	PEX13	0.16493
BGLF4	EMR4P	0.032001
BGLF4	IGBP1	0.123
BGLF4	MEOX2	0.072894
BGLF4	TFAP4	0.1028
BGLF4	UBAP2L	0.22976
BGLF4	SETD8	0.18121
BGLF4	MAGEA4	0.22414
BGLF4	TAGLN2	0.21681
BGLF4	SIRT4	0.27518
BGLF4	FYTTD1	0.26256
BGLF4	DNAJB6	0.15811
BGLF4	PCDHA4	0.05466
BGLF4	CDH15	0.044287
BGLF4	FAM110A	0.20246
BGLF4	LDHD	0.20779
BGLF4	PRNP	0.056315
BGLF4	TESC	0.20861
BGLF4	CLIC3	0.15874
BGLF4	TAF5	0.016784
BGLF4	CHD1	0.096672
BGLF4	RBP4	0.091355
BGLF4	HOXB9	0.07029
BGLF4	SETD7	0.2031
BGLF4	FAM161A	0.27124
BGLF4	ATPAF1	0.22982
BGLF4	PARD3	0.088129
BGLF4	FMNL1	0.13991
BGLF4	UBE2Q1	0.12386
BGLF4	IFNLR1	0.20142
BGLF4	EFHB	0.068073
BGLF4	LGI1	0.12094
BGLF4	NME2	0.052682
BGLF4	KIAA0319	0.17475
BGLF4	CRY2	0.077932
BGLF4	ARL2	0.083692
BGLF4	EYS	0.085139
BGLF4	MICAL1	0.20257

BGLF4	NDNL2	0.18462
BGLF4	ROPN1L	0.20789
BGLF4	OBSCN	0.034437
BGLF4	UCN	0.10532
BGLF4	PCDHGB7	0.058173
BGLF4	RPA3	0.09614
BGLF4	FGFR3	0.023109
BGLF4	RWDD1	0.24432
BGLF4	TFPT	0.17747
BGLF4	SMPDL3A	0.17894
BGLF4	RHO	0.091988
BGLF4	LIG3	0.062816
BGLF4	HSPB6	0.21194
BGLF4	ADAMTS4	0.10773
BGLF4	PJA1	0.1526
BGLF4	AHNAK	0.20842
BGLF4	CCNF	0.16501
BGLF4	HOOK3	0.11866
BGLF4	SETD1A	0.19744
BGLF4	GLB1	0.093279
BGLF4	SMARCC2	0.12945
BGLF4	HIC1	0.03441
BGLF4	FCN3	0.16111
BGLF4	PPFIA1	0.21543
BGLF4	TAF4	0.044261
BGLF4	PPP4R4	0.23255
BGLF4	DCLRE1B	0.11841
BGLF4	OSBPL1A	0.10864
BGLF4	GOPC	0.10136
BGLF4	CD82	0.2159
BGLF4	TNFRSF1A	0.069444
BGLF4	DZIP3	0.18431
BGLF4	RALA	0.10034
BGLF4	CD226	0.09481
BGLF4	MOB4	0.17965
BGLF4	BIN3	0.17417
BGLF4	DRG1	0.11667
BGLF4	POLR2A	0.068382
BGLF4	RPH3A	0.22239
BGLF4	SPTSSA	0.15482
BGLF4	DCP1A	0.12086
BGLF4	ADAP1	0.039939
BGLF4	AMOTL2	0.23098
BGLF4	SMARCA1	0.085746
BGLF4	SRI	0.23657
BGLF4	SELL	0.14724
BGLF4	OCM	0.048404
BGLF4	PPP4R2	0.15344
BGLF4	RTP3	0.20555
BGLF4	LRP4	0.21669
BGLF4	DPPA4	0.19941
BGLF4	KAT6A	0.076368
BGLF4	CDK8	0.012478
BGLF4	PMF1	0.12019
BGLF4	BDKRB2	0.085399
BGLF4	KPNA2	0.14344
BGLF4	EVI5L	0.1841
BGLF4	NEK2	0.02146
BGLF4	RAD54L	0.10718
BGLF4	TSC2	0.048711
BGLF4	SLC7A8	0.05442
BGLF4	PSG5	0.22971
BGLF4	PIK3R2	0.112
BGLF4	MAP3K14	0.025963
BGLF4	BMX	0.034995
BGLF4	INADL	0.17662
BGLF4	SEL1L	0.19754
BGLF4	GNL3L	0.13266

BGLF4	PTPN12	0.066248
BGLF4	PLA2G12B	0.039329
BGLF4	FEN1	0.040504
BGLF4	TMSB4X	0.14088
BGLF4	PCDHB12	0.05466
BGLF4	NONO	0.17787
BGLF4	PLK1S1	0.13117
BGLF4	CUL2	0.14809
BGLF4	CCT3	0.12781
BGLF4	SRSF7	0.15648
BGLF4	CABP5	0.060268
BGLF4	SLIT2	0.068227
BGLF4	PKD1	0.025846
BGLF4	HERC2	0.081699
BGLF4	TBC1D5	0.16194
BGLF4	SNED1	0.064124
BGLF4	AKAP9	0.097523
BGLF4	MIB1	0.12108
BGLF4	PASK	0.053218
BGLF4	BLM	0.029448
BGLF4	ARNT	0.041293
BGLF4	SYNE3	0.14661
BGLF4	DOCK4	0.12716
BGLF4	NCLN	0.14018
BGLF4	HNRNPUL1	0.18203
BGLF4	GPRASP1	0.23693
BGLF4	MAGEE1	0.1707
BGLF4	RAD51AP1	0.23831
BGLF4	CNPY2	0.20968
BGLF4	NSRP1	0.19479
BGLF4	SFI1	0.18702
BGLF4	RPP38	0.1717
BGLF4	MATN2	0.32951
BGLF4	BCAR1	0.057258
BGLF4	PSMD9	0.089693
BGLF4	AXIN1	0.020408
BGLF4	SIRT6	0.14186
BGLF4	INTS7	0.20381
BGLF4	STK4	0.013176
BGLF4	GPD2	0.038879
BGLF4	PCDHA10	0.053108
BGLF4	CIB1	0.29484
BGLF4	IRAK2	0.027105
BGLF4	UBR5	0.10739
BGLF4	PRRG4	0.057144
BGLF4	ANKRD44	0.24432
BGLF4	KIF13A	0.077496
BGLF4	FNTA	0.16137
BGLF4	EIF5A2	0.13384
BGLF4	MAPRE3	0.18171
BGLF4	CLCN3	0.1002
BGLF4	DNAJB2	0.16555
BGLF4	DNMT1	0.12554
BGLF4	PCDHA11	0.05466
BGLF4	HDAC10	0.082036
BGLF4	RYR1	0.010427
BGLF4	PCDHB7	0.056436
BGLF4	TNK1	0.083396
BGLF4	PHF8	0.10767
BGLF4	GYS1	0.087823
BGLF4	PPP2R3A	0.31284
BGLF4	GSPT1	0.13445
BGLF4	DSC1	0.06419
BGLF4	AKAP3	0.24359
BGLF4	PRPF19	0.13018
BGLF4	EIF2B4	0.14925
BGLF4	NCF1	0.039408
BGLF4	PSMC2	0.060205

BGLF4	TSSK4	0.047081
BGLF4	USE1	0.15218
BGLF4	LAPTM4B	0.2194
BGLF4	DLL1	0.21742
BGLF4	TAF2	0.11885
BGLF4	ACTN1	0.18777
BGLF4	CABP2	0.056932
BGLF4	VDR	0.05026
BGLF4	PEX11B	0.18413
BGLF4	PMP22	0.19214
BGLF4	RAB6A	0.126
BGLF4	C11orf54	0.16986
BGLF4	STK38	0.023199
BGLF4	TNKS2	0.098492
BGLF4	RCHY1	0.076213
BGLF4	CCND1	0.029655
BGLF4	CD164	0.14026
BGLF4	EPRS	0.075664
BGLF4	TBC1D15	0.17093
BGLF4	AOC3	0.08586
BGLF4	FUS	0.16673
BGLF4	BLOC1S1	0.14591
BGLF4	DCTN3	0.066638
BGLF4	NPHP3	0.11377
BGLF4	USP46	0.18034
BGLF4	UBD	0.13795
BGLF4	MYH10	0.06274
BGLF4	CDT1	0.14411
BGLF4	SYNE1	0.12461
BGLF4	FBXO3	0.20113
BGLF4	LRP6	0.01376
BGLF4	EXOC8	0.083233
BGLF4	KNTC1	0.10525
BGLF4	NCL	0.14981
BGLF4	ITGA1	0.13562
BGLF4	SVEP1	0.057532
BGLF4	NOS1	0.087784
BGLF4	EHD4	0.19787
BGLF4	APBA3	0.15683
BGLF4	RYR3	0.030258
BGLF4	SCUBE1	0.025306
BGLF4	PCDHB14	0.053256
BGLF4	HNRNPK	0.11407
BGLF4	LCAT	0.1436
BGLF4	OBFC1	0.088117
BGLF4	HJURP	0.10782
BGLF4	TWIST1	0.030533
BGLF4	E2F5	0.042156
BGLF4	CSNK1E	0.033251
BGLF4	FAM124B	0.24432
BGLF4	MEF2A	0.039938
BGLF4	PNPT1	0.076931
BGLF4	NPRL2	0.12458
BGLF4	UPF3B	0.18877
BGLF4	AKAP13	0.06458
BGLF4	ATG2A	0.24432
BGLF4	ZC3H7B	0.14911
BGLF4	LZTFL1	0.21499
BGLF4	TLN1	0.032465
BGLF4	BTRC	0.079225
BGLF4	PCDHGA11	0.058173
BGLF4	RHBDL1	0.1298
BGLF4	HNF1B	0.03327
BGLF4	TFAP2A	0.013408
BGLF4	UBE2D1	0.070435
BGLF4	GCKR	0.1263
BGLF4	MYO6	0.060878
BGLF4	LCMT2	0.27592

BGLF4	GATAD2A	0.061096
BGLF4	USP8	0.091213
BGLF4	DSPP	0.072287
BGLF4	FIG4	0.091601
BGLF4	TNFSF14	0.10508
BGLF4	CDC6	0.060719
BGLF4	CDH3	0.071495
BGLF4	PLAT	0.13418
BGLF4	TAX1BP3	0.16201
BGLF4	SNX4	0.19342
BGLF4	NEGR1	0.19468
BGLF4	HTR6	0.15182
BGLF4	CUEDC2	0.20333
BGLF4	SEPT11	0.15697
BGLF4	DLGAP1	0.17413
BGLF4	CCND3	0.038965
BGLF4	MFI2	0.14009
BGLF4	FLNA	0.045651
BGLF4	HIST1H4A	0.086191
BGLF4	CTTNBP2NL	0.23566
BGLF4	RAI2	0.22417
BGLF4	SERPINF2	0.11421
BGLF4	FBXL5	0.16934
BGLF4	SGSM3	0.16418
BGLF4	DUOX1	0.10154
BGLF4	F3	0.054299
BGLF4	CECR2	0.1767
BGLF4	PMAIP1	0.055379
BGLF4	SPINK1	0.20342
BGLF4	ABCD1	0.097668
BGLF4	CHAC1	0.16518
BGLF4	DDX3X	0.02163
BGLF4	PCDHA2	0.05466
BGLF4	SH3GLB2	0.17695
BGLF4	CDK16	0.041453
BGLF4	RASEF	0.040468
BGLF4	PRSS3	0.34865
BGLF4	ANXA8	0.045475
BGLF4	RPS6KC1	0.06146
BGLF4	MAP1S	0.083579
BGLF4	BTK	0.016164
BGLF4	CLEC4G	0.22273
BGLF4	PPIL3	0.21318
BGLF4	CALML5	0.056876
BGLF4	LRRC4C	0.22281
BGLF4	MYL2	0.31132
BGLF4	BAIAP2	0.090944
BGLF4	KAT5	0.10292
BGLF4	PIWIL1	0.17356
BGLF4	ALOX12	0.096907
BGLF4	EDEM1	0.27016
BGLF4	LRP1	0.12358
BGLF4	UFL1	0.14244
BGLF4	APBA2	0.17497
BGLF4	DHDDS	0.23669
BGLF4	MKI67	0.10787
BGLF4	JADE1	0.13987
BGLF4	MTF1	0.038806
BGLF4	FGFR1	0.011357
BGLF4	GEMIN2	0.1556
BGLF4	SELE	0.10151
BGLF4	TONSL	0.15401
BGLF4	CACNB4	0.080435
BGLF4	RBM12B	0.2695
BGLF4	CKS1B	0.1756
BGLF4	ATP10B	0.19087
BGLF4	TIMM23	0.16233
BGLF4	DICER1	0.026404

BGLF4	HADHB	0.079345
BGLF4	APPL1	0.07209
BGLF4	CCDC92	0.22233
BGLF4	VPRBP	0.18197
BGLF4	SENP5	0.17273
BGLF4	SUV39H1	0.12528
BGLF4	COPS6	0.17486
BGLF4	SNX2	0.16569
BGLF4	MAP2K5	0.040433
BGLF4	RNF11	0.129
BGLF4	ITGAM	0.1119
BGLF4	CDH17	0.068813
BGLF4	CDK11B	0.040475
BGLF4	EFCAB9	0.068073
BGLF4	PEX1	0.077846
BGLF4	NUDT18	0.19641
BGLF4	EXOSC2	0.18565
BGLF4	ERG	0.051065
BGLF4	ZNF622	0.12888
BGLF4	PRCC	0.16184
BGLF4	NFS1	0.1289
BGLF4	EEF1E1	0.15703
BGLF4	FANCD2	0.18772
BGLF4	TNNC2	0.057678
BGLF4	GNA12	0.087235
BGLF4	ZC3H14	0.2056
BGLF4	MEPCE	0.011002
BGLF4	LSM11	0.16722
BGLF4	BUB1	0.027058
BGLF4	RIT1	0.16845
BGLF4	KCNA4	0.15294
BGLF4	RIOK3	0.10561
BGLF4	KISS1	0.22302
BGLF4	DMC1	0.093731
BGLF4	ATP5A1	0.10617
BGLF4	RAB21	0.15923
BGLF4	SGPL1	0.040699
BGLF4	ADAR	0.15024
BGLF4	OIP5	0.14775
BGLF4	GAD1	0.10346
BGLF4	WDTC1	0.1242
BGLF4	SNX19	0.34251
BGLF4	MAP2K4	0.042056
BGLF4	ASPH	0.11101
BGLF4	CREB1	0.017969
BGLF4	LIN28B	0.16576
BGLF4	TUBGCP2	0.12684
BGLF4	HIP1	0.08064
BGLF4	MBD3	0.14886
BGLF4	ITGA4	0.13578
BGLF4	FSTL1	0.048814
BGLF4	SRC	0.014906
BGLF4	MRE11A	0.076627
BGLF4	USP47	0.13614
BGLF4	CALR3	0.029174
BGLF4	CLPP	0.21375
BGLF4	CHORDC1	0.16428
BGLF4	SMG6	0.082048
BGLF4	ID1	0.0535
BGLF4	SOX10	0.018875
BGLF4	TRPC1	0.10907
BGLF4	TOM1	0.18324
BGLF4	PITPNC1	0.22769
BGLF4	PDGFRB	0.028521
BGLF4	NDUFB10	0.19632
BGLF4	KIF2C	0.064353
BGLF4	CACNA1B	0.11545
BGLF4	SEPT5	0.049882

BGLF4	PAX8	0.023287
BGLF4	APPBP2	0.19644
BGLF4	C1QTNF3	0.14149
BGLF4	GAD2	0.10442
BGLF4	WNK1	0.064749
BGLF4	TCF3	0.011078
BGLF4	DACT1	0.094444
BGLF4	PCNA	0.032879
BGLF4	DDRGK1	0.23728
BGLF4	EPO	0.072015
BGLF4	IRF8	0.010961
BGLF4	CD274	0.17349
BGLF4	ITSN1	0.16142
BGLF4	ATP7B	0.045466
BGLF4	PTPRD	0.031406
BGLF4	KDM4A	0.12505
BGLF4	TRAM2	0.21292
BGLF4	CEBPB	0.042614
BGLF4	BRCC3	0.10566
BGLF4	BBS1	0.17352
BGLF4	SLC22A4	0.12675
BGLF4	ETV7	0.093672
BGLF4	HNRNPF	0.15304
BGLF4	CCDC141	0.24432
BGLF4	PCDHGA12	0.058173
BGLF4	ELOVL1	0.13809
BGLF4	PRDX3	0.075349
BGLF4	DST	0.15045
BGLF4	HIF1AN	0.10562
BGLF4	COL18A1	0.074614
BGLF4	OGG1	0.06358
BGLF4	MYL6	0.35714
BGLF4	COMP	0.29555
BGLF4	RNF31	0.098907
BGLF4	EIF3G	0.22179
BGLF4	MLLT6	0.16842
BGLF4	CCDC47	0.34985
BGLF4	SNAP47	0.20873
BGLF4	EXOSC7	0.1722
BGLF4	LIMD1	0.077343
BGLF4	MX1	0.093638
BGLF4	SATB1	0.057355
BGLF4	RIN1	0.18763
BGLF4	RCOR1	0.1233
BGLF4	ANXA7	0.26885
BGLF4	SON	0.15779
BGLF4	MAP3K7CL	0.20895
BGLF4	RNF139	0.10069
BGLF4	PRC1	0.051237
BGLF4	CDH26	0.058173
BGLF4	FRK	0.073173
BGLF4	CHP2	0.32112
BGLF4	ADNP	0.036589
BGLF4	TOX3	0.17796
BGLF4	SPG11	0.22825
BGLF4	PSMA7	0.045792
BGLF4	ZFP36L1	0.046364
BGLF4	CD97	0.030973
BGLF4	ABCC2	0.16115
BGLF4	PTPRB	0.06158
BGLF4	POM121C	0.17042
BGLF4	MUC1	0.11506
BGLF4	RANBP3	0.15982
BGLF4	CTNNA1	0.057898
BGLF4	VBP1	0.13772
BGLF4	MRVI1	0.15924
BGLF4	UBE2L6	0.15132
BGLF4	ZHX3	0.073714

BGLF4	RSPO1	0.1279
BGLF4	GOLPH3	0.16517
BGLF4	IRF6	0.019432
BGLF4	PLN	0.14496
BGLF4	CDK5R1	0.042647
BGLF4	SUB1	0.15726
BGLF4	ANP32A	0.1536
BGLF4	LTBP2	0.052103
BGLF4	PPM1F	0.062442
BGLF4	SEPT4	0.026121
BGLF4	AKT3	0.021333
BGLF4	TEAD2	0.090255
BGLF4	PLOD3	0.13212
BGLF4	TOLLIP	0.16463
BGLF4	EIF4EBP2	0.1812
BGLF4	RUVBL1	0.06902
BGLF4	LRP12	0.17979
BGLF4	NDUFS7	0.049752
BGLF4	NECAB2	0.45159
BGLF4	ELF3	0.059917
BGLF4	PROS1	0.015646
BGLF4	OTX2	0.022935
BGLF4	OGT	0.071076
BGLF4	GTF3C4	0.19085
BGLF4	PBRM1	0.17405
BGLF4	NCOR1	0.038
BGLF4	APITD1	0.10138
BGLF4	HGF	0.088178
BGLF4	FAM103A1	0.23561
BGLF4	DNAJB9	0.15867
BGLF4	AVEN	0.20956
BGLF4	SLBP	0.14781
BGLF4	USH2A	0.12983
BGLF4	PHF2	0.13128
BGLF4	PODXL2	0.2024
BGLF4	APLP1	0.046841
BGLF4	FBP1	0.057972
BGLF4	RFC2	0.081921
BGLF4	TYROBP	0.1085
BGLF4	CANX	0.18905
BGLF4	HNF1A	0.028971
BGLF4	RHOT2	0.19152
BGLF4	DOCK9	0.10758
BGLF4	RTKN	0.11925
BGLF4	EYA3	0.035923
BGLF4	KLHL3	0.10681
BGLF4	EHMT1	0.15757
BGLF4	KDR	0.016464
BGLF4	SNAP23	0.16991
BGLF4	MMP15	0.22629
BGLF4	MKNK1	0.048312
BGLF4	TTC8	0.18163
BGLF4	PDE6D	0.25736
BGLF4	ASCC3	0.11158
BGLF4	CCNDBP1	0.20222
BGLF4	PPP2R5B	0.13465
BGLF4	GADD45A	0.14866
BGLF4	PLCB3	0.033204
BGLF4	SNX15	0.025042
BGLF4	LCP2	0.18829
BGLF4	ORAI2	0.22976
BGLF4	SUSD1	0.064189
BGLF4	APBA1	0.12733
BGLF4	DKK1	0.082682
BGLF4	ANXA11	0.23474
BGLF4	SPINK9	0.20973
BGLF4	ITM2C	0.14771
BGLF4	USP54	0.22921

BGLF4	PPP3CB	0.1151
BGLF4	TAB2	0.074427
BGLF4	SNRPB	0.11235
BGLF4	KRT19	0.14459
BGLF4	PCBD2	0.12356
BGLF4	S100G	0.06299
BGLF4	ATG7	0.091707
BGLF4	MDM4	0.10804
BGLF4	SCYL2	0.058225
BGLF4	NCKIPSD	0.21887
BGLF4	SPINK7	0.21629
BGLF4	NQO1	0.1697
BGLF4	NFATC2	0.042592
BGLF4	ACSS1	0.058481
BGLF4	ACACB	0.10989
BGLF4	RBM39	0.18021
BGLF4	IGF2BP3	0.15129
BGLF4	TRIM27	0.064689
BGLF4	CSPG5	0.077514
BGLF4	MC3R	0.14268
BGLF4	PAX2	0.021884
BGLF4	CNOT4	0.11891
BGLF4	CDK12	0.016434
BGLF4	DLG2	0.1249
BGLF4	ZNF136	0.1406
BGLF4	MAST1	0.04568
BGLF4	MAU2	0.17951
BGLF4	SH3PXD2B	0.12078
BGLF4	UBN1	0.076225
BGLF4	IQGAP1	0.1249
BGLF4	FAAP24	0.16164
BGLF4	HIRA	0.089468
BGLF4	ATP6V0C	0.12701
BGLF4	STAP2	0.083434
BGLF4	EPAS1	0.024022
BGLF4	CCNA2	0.089666
BGLF4	FZD1	0.043291
BGLF4	ELAVL1	0.11366
BGLF4	RAD51	0.050892
BGLF4	BMI1	0.06746
BGLF4	OPTN	0.16277
BGLF4	TMED1	0.13162
BGLF4	C19orf60	0.24432
BGLF4	RAB9A	0.081637
BGLF4	TIMP1	0.11344
BGLF4	MATN3	0.42517
BGLF4	SKA1	0.11464
BGLF4	EFCAB8	0.068073
BGLF4	ATL2	0.093595
BGLF4	NR2F2	0.026895
BGLF4	RAP1A	0.10486
BGLF4	MAST2	0.038922
BGLF4	SUPT20H	0.19551
BGLF4	SH3BP2	0.13749
BGLF4	YEATS4	0.10523
BGLF4	SFN	0.075228
BGLF4	FBN3	0.077559
BGLF4	G0S2	0.18911
BGLF4	RPL27A	0.18098
BGLF4	MDC1	0.1658
BGLF4	PGRMC1	0.17304
BGLF4	NHP2L1	0.18571
BGLF4	SH3KBP1	0.20384
BGLF4	AMOTL1	0.20935
BGLF4	MYLIP	0.09703
BGLF4	TENC1	0.11305
BGLF4	STK3	0.027298
BGLF4	DLGAP4	0.18434

BGLF4	TOMM70A	0.14729
BGLF4	TCERG1	0.17637
BGLF4	PCDH8	0.041925
BGLF4	UBE4A	0.19408
BGLF4	GTF3C5	0.2066
BGLF4	LRRK2	0.014526
BGLF4	TSC1	0.063699
BGLF4	CPNE6	0.073953
BGLF4	CD276	0.1174
BGLF4	RNF41	0.084653
BGLF4	BASP1	0.088455
BGLF4	RASSF2	0.19585
BGLF4	TJAP1	0.20117
BGLF4	ETV6	0.09468
BGLF4	DARS	0.11283
BGLF4	SCHIP1	0.20223
BGLF4	RET	0.080828
BGLF4	OS9	0.18486
BGLF4	ARHGAP26	0.079365
BGLF4	COL1A2	0.097604
BGLF4	LSM7	0.15467
BGLF4	SRY	0.077169
BGLF4	JAM2	0.18223
BGLF4	MAFB	0.071455
BGLF4	DGKA	0.019704
BGLF4	ALOX5	0.10068
BGLF4	SOD1	0.022929
BGLF4	WASH1	0.16128
BGLF4	SPRED1	0.062771
BGLF4	PARP1	0.067432
BGLF4	TAF10	0.035289
BGLF4	CLCF1	0.13969
BGLF4	CHEK1	0.035553
BGLF4	FCRL4	0.22282
BGLF4	SF3B4	0.20362
BGLF4	EPS8	0.20671
BGLF4	PATL1	0.14195
BGLF4	TRAF5	0.11215
BGLF4	PARP10	0.19079
BGLF4	WBP11	0.16204
BGLF4	NSMCE4A	0.20285
BGLF4	SNTA1	0.12095
BGLF4	ATP11A	0.10692
BGLF4	IRF2	0.042851
BGLF4	CDH22	0.058173
BGLF4	THBS4	0.042737
BGLF4	FBXL3	0.17001
BGLF4	DDX1	0.081502
BGLF4	MC4R	0.1265
BGLF4	CDHR4	0.058173
BGLF4	CHD1L	0.13686
BGLF4	RPP25	0.21045
BGLF4	SLC3A2	0.10627
BGLF4	UBQLN1	0.14292
BGLF4	USP13	0.09437
BGLF4	SLC6A3	0.093301
BGLF4	CALCR	0.086414
BGLF4	PTGFRN	0.1949
BGLF4	EIF4EBP3	0.18651
BGLF4	PTPN11	0.031994
BGLF4	CPSF1	0.18896
BGLF4	FLVCR1	0.14345
BGLF4	MS4A3	0.2096
BGLF4	HOXA5	0.026824
BGLF4	HCFC1	0.048138
BGLF4	FLNB	0.13542
BGLF4	TMEM66	0.17544
BGLF4	SMAD4	0.014292

BGLF4	PICK1	0.069675
BGLF4	MSLN	0.18421
BGLF4	RAB11FIP5	0.097373
BGLF4	SUZ12	0.12666
BGLF4	CDH23	0.18947
BGLF4	KIT	0.016217
BGLF4	STATH	0.1858
BGLF4	UBC	0.030564
BGLF4	RAB11FIP4	0.35336
BGLF4	RLF	0.12698
BGLF4	KRT18	0.10578
BGLF4	FGF13	0.086801
BGLF4	FARSA	0.018734
BGLF4	NTM	0.17901
BGLF4	PDCD6IP	0.16292
BGLF4	GAB1	0.090532
BGLF4	HSFX1	0.098524
BGLF4	RARRES2	0.16449
BGLF4	HSPB1	0.03488
BGLF4	BMPR1A	0.015379
BGLF4	MYCBPAP	0.17229
BGLF4	MEF2D	0.077517
BGLF4	PSMA2	0.056809
BGLF4	ASXL1	0.12199
BGLF4	APBB2	0.089288
BGLF4	TP53BP1	0.094225
BGLF4	MAP1LC3A	0.16034
BGLF4	UBXN1	0.14168
BGLF4	PRPF3	0.18988
BGLF4	ATP6V1H	0.1391
BGLF4	CDH12	0.053252
BGLF4	PHYH	0.091299
BGLF4	JMJD6	0.090586
BGLF4	RRAGA	0.10905
BGLF4	HSPB2	0.17916
BGLF4	PIGT	0.14147
BGLF4	EIF3A	0.12216
BGLF4	MGARP	0.12594
BGLF4	NFKB2	0.044058
BGLF4	UBE2I	0.029515
BGLF4	CASP7	0.092179
BGLF4	H2AFX	0.0948
BGLF4	RHOU	0.062195
BGLF4	SSRP1	0.165
BGLF4	SUMO3	0.21608
BGLF4	TLR2	0.028247
BGLF4	ATF3	0.071659
BGLF4	SOS2	0.086673
BGLF4	SYNE2	0.089653
BGLF4	DPYSL2	0.018613
BGLF4	BCL2L2	0.18526
BGLF4	SUV39H2	0.17011
BGLF4	IL10RA	0.19561
BGLF4	CLIC5	0.10675
BGLF4	CTBP1	0.022328
BGLF4	UBE2F	0.11833
BGLF4	AGTR2	0.075435
BGLF4	IFIH1	0.072662
BGLF4	IER3	0.14814
BGLF4	CNKSR1	0.11808
BGLF4	LDHAL6B	0.18686
BGLF4	MAGEA1	0.18035
BGLF4	MBOAT7	0.19258
BGLF4	FOCAD	0.21656
BGLF4	LOX	0.12504
BGLF4	ELP4	0.1452
BGLF4	ELTD1	0.039333
BGLF4	CAV2	0.073727

BGLF4	FXR2	0.28916
BGLF4	POLR2B	0.070155
BGLF4	VANGL1	0.22281
BGLF4	MPP1	0.17425
BGLF4	TRIP6	0.14081
BGLF4	PKP4	0.13252
BGLF4	COMMD4	0.23693
BGLF4	ENO1	0.013282
BGLF4	AGGF1	0.17001
BGLF4	ORC5	0.10798
BGLF4	CLU	0.046389
BGLF4	PPIB	0.19326
BGLF4	PRRG1	0.060357
BGLF4	NRG1	0.02999
BGLF4	A1CF	0.14067
BGLF4	NIFK	0.18213
BGLF4	ATXN7	0.15616
BGLF4	UBA6	0.096506
BGLF4	TDP2	0.11565
BGLF4	SUDS3	0.20361
BGLF4	CHCHD3	0.15467
BGLF4	PRKACB	0.017674
BGLF4	MLANA	0.18465
BGLF4	LPHN1	0.13623
BGLF4	BRINP1	0.20157
BGLF4	WIPF2	0.22281
BGLF4	SNX5	0.02675
BGLF4	MCM2	0.056916
BGLF4	PLCE1	0.10508
BGLF4	CD163	0.19363
BGLF4	IL10RB	0.19206
BGLF4	DYNLL1	0.10305
BGLF4	PIDD	0.19381
BGLF4	ADAMTSL4	0.13873
BGLF4	MYOT	0.17865
BGLF4	PDCD1	0.17931
BGLF4	HNRNPLL	0.19239
BGLF4	SDCCAG8	0.1744
BGLF4	IL12B	0.026848
BGLF4	WDR48	0.14701
BGLF4	PDCD11	0.18288
BGLF4	G3BP1	0.12874
BGLF4	RASSF1	0.085896
BGLF4	NELFCD	0.17345
BGLF4	KHDRBS1	0.17416
BGLF4	EID1	0.1471
BGLF4	PCDHB4	0.045607
BGLF4	IGHMBP2	0.034795
BGLF4	SNX3	0.1926
BGLF4	OLA1	0.1299
BGLF4	ATP6V1D	0.14795
BGLF4	TDP1	0.15977
BGLF4	ABL2	0.048068
BGLF4	DOCK3	0.20935
BGLF4	STX6	0.12604
BGLF4	MAK	0.024809
BGLF4	CYLD	0.041218
BGLF4	GP6	0.17086
BGLF4	SNW1	0.1223
BGLF4	PPL	0.15661
BGLF4	KLK3	0.24662
BGLF4	SGIP1	0.093718
BGLF4	WAC	0.16376
BGLF4	TTR	0.20358
BGLF4	ATP2B4	0.11604
BGLF4	BBS7	0.16642
BGLF4	CRYAA	0.092867
BGLF4	NEDD4L	0.085304

BGLF4	PCDH9	0.054326
BGLF4	PPP6R2	0.19737
BGLF4	HPCAL1	0.068073
BGLF4	MIS12	0.14097
BGLF4	ARF4	0.12665
BGLF4	ZNF274	0.068707
BGLF4	DAPK1	0.062883
BGLF4	SOX6	0.035647
BGLF4	ITGA3	0.16844
BGLF4	COG3	0.12091
BGLF4	TNIK	0.037905
BGLF4	BRD7	0.078074
BGLF4	RNASE1	0.19538
BGLF4	NSF	0.083879
BGLF4	RBBP7	0.12123
BGLF4	PTRF	0.13227
BGLF4	ARIH1	0.14433
BGLF4	ECM1	0.11052
BGLF4	KLC2	0.16812
BGLF4	BEGAIN	0.22976
BGLF4	ARF6	0.070032
BGLF4	PAAF1	0.21686
BGLF4	SNX21	0.026854
BGLF4	FLCN	0.19591
BGLF4	KISS1R	0.11562
BGLF4	DNASE1L3	0.035317
BGLF4	RIPK2	0.010463
BGLF4	SNRPD3	0.11022
BGLF4	CLEC2A	0.22411
BGLF4	CTSB	0.048487
BGLF4	ILKAP	0.11465
BGLF4	CLIC4	0.14547
BGLF4	RAB14	0.04197
BGLF4	STX12	0.14073
BGLF4	SAMHD1	0.14747
BGLF4	ACTB	0.042776
BGLF4	EIF4G1	0.18103
BGLF4	SLC22A5	0.12887
BGLF4	KIF3A	0.086578
BGLF4	SCNN1A	0.15787
BGLF4	CGREF1	0.054985
BGLF4	CHAF1B	0.13606
BGLF4	RUSC1	0.15728
BGLF4	NPNT	0.057887
BGLF4	PEX12	0.1514
BGLF4	FLOT1	0.15191
BGLF4	BNIP2	0.26118
BGLF4	GABARAPL2	0.17801
BGLF4	PPIH	0.17536
BGLF4	RARS	0.052421
BGLF4	PRKDC	0.019967
BGLF4	JAG2	0.22919
BGLF4	RGR	0.16734
BGLF4	MYRIP	0.16625
BGLF4	CTTN	0.20219
BGLF4	C1QA	0.20033
BGLF4	PPP1R16A	0.22977
BGLF4	EFCAB7	0.068073
BGLF4	TACC1	0.13688
BGLF4	CREB3	0.025364
BGLF4	LTBP3	0.050833
BGLF4	PLK4	0.047713
BGLF4	SKIV2L2	0.10592
BGLF4	ADRB2	0.032111
BGLF4	DDX18	0.17363
BGLF4	ATP2B1	0.12973
BGLF4	POLR1B	0.11831
BGLF4	VIMP	0.085072

BGLF4	KMT2D	0.14536
BGLF4	CEP97	0.21441
BGLF4	PTPRU	0.071335
BGLF4	TPCN2	0.052417
BGLF4	RUNX1T1	0.067923
BGLF4	CENPI	0.11162
BGLF4	SHC3	0.17699
BGLF4	LGALS3	0.148
BGLF4	FCGR2B	0.19002
BGLF4	IST1	0.28609
BGLF4	MAP4K5	0.061621
BGLF4	NR5A2	0.011403
BGLF4	FCHO2	0.16126
BGLF4	TIPRL	0.2291
BGLF4	SKP2	0.1353
BGLF4	PLAA	0.21548
BGLF4	NR1H4	0.022098
BGLF4	DUSP15	0.047426
BGLF4	MIB2	0.12362
BGLF4	EHD2	0.1888
BGLF4	PVRL2	0.083093
BGLF4	HS3ST3A1	0.0317
BGLF4	COPS5	0.13538
BGLF4	RNF135	0.14946
BGLF4	CCNE2	0.084621
BGLF4	C5AR2	0.16393
BGLF4	CADPS	0.33316
BGLF4	JUND	0.064405
BGLF4	ABCA1	0.027136
BGLF4	MARK2	0.038
BGLF4	CNTROB	0.17801
BGLF4	CDC42SE2	0.038753
BGLF4	HEXIM2	0.17192
BGLF4	HRNR	0.048542
BGLF4	PKD2	0.026384
BGLF4	NCAPH	0.17809
BGLF4	CREBRF	0.081977
BGLF4	AURKA	0.012873
BGLF4	PCDHGB2	0.058173
BGLF4	BBS4	0.03941
BGLF4	PDZD7	0.20224
BGLF4	PPP2CB	0.057319
BGLF4	DLC1	0.073055
BGLF4	STXBP4	0.036281
BGLF4	IPO9	0.17288
BGLF4	ANXA8L1	0.048161
BGLF4	PDIA6	0.090951
BGLF4	HDAC5	0.027469
BGLF4	TRPC6	0.12223
BGLF4	ZNF655	0.13113
BGLF4	ETF1	0.19567
BGLF4	DCSTAMP	0.11265
BGLF4	EMILIN1	0.18029
BGLF4	CRYGD	0.19284
BGLF4	ADRM1	0.1493
BGLF4	KLK6	0.14855
BGLF4	CCAR1	0.3381
BGLF4	PFDN4	0.15552
BGLF4	C19orf57	0.22411
BGLF4	LPCAT1	0.024524
BGLF4	HIST1H3A	0.12599
BGLF4	CCNA1	0.090495
BGLF4	MYCN	0.063064
BGLF4	NKX2-1	0.018905
BGLF4	SPARCL1	0.071853
BGLF4	PDZD3	0.12173
BGLF4	CD40	0.055111
BGLF4	ENPP1	0.047881

BGLF4	PPP1R12A	0.091478
BGLF4	ERC1	0.10458
BGLF4	CTNND1	0.084343
BGLF4	PICALM	0.061345
BGLF4	PLEKHG6	0.081881
BGLF4	GUCA1A	0.073322
BGLF4	SEMA7A	0.14521
BGLF4	CRMP1	0.060532
BGLF4	LITAF	0.15706
BGLF4	TGFA	0.097362
BGLF4	IQCB1	0.24224
BGLF4	CCAR2	0.20225
BGLF4	CACTIN	0.13935
BGLF4	PLAUR	0.10089
BGLF4	UTP20	0.15465
BGLF4	DVL1	0.041189
BGLF4	GINS2	0.18445
BGLF4	WDR66	0.068073
BGLF4	LOXL2	0.062819
BGLF4	CASP3	0.057351
BGLF4	SHC1	0.043924
BGLF4	MITF	0.04891
BGLF4	ZNF217	0.043121
BGLF4	MPHOSPH6	0.21648
BGLF4	GTF2A1	0.12304
BGLF4	NLGN4Y	0.15919
BGLF4	SLC2A2	0.01925
BGLF4	ELF1	0.09599
BGLF4	COPS3	0.18728
BGLF4	ZMYND8	0.15847
BGLF4	COMMD6	0.18968
BGLF4	LONRF3	0.18483
BGLF4	GABARAP	0.14456
BGLF4	WWP2	0.055657
BGLF4	TGFB1I1	0.092757
BGLF4	PIKFYVE	0.10117
BGLF4	MEX3B	0.048724
BGLF4	FBXW9	0.24432
BGLF4	CD37	0.20974
BGLF4	STAM	0.16051
BGLF4	GSK3A	0.020905
BGLF4	CBLB	0.13386
BGLF4	CRELD2	0.41204
BGLF4	PPP2R5A	0.10356
BGLF4	BLOC1S2	0.11643
BGLF4	MYOZ1	0.1909
BGLF4	TMEM30A	0.20986
BGLF4	PRUNE	0.16998
BGLF4	NCOA3	0.10599
BGLF4	MUM1	0.21204
BGLF4	TIMELESS	0.15125
BGLF4	NEK9	0.019185
BGLF4	OSBPL3	0.17557
BGLF4	NIF3L1	0.17103
BGLF4	MZF1	0.070166
BGLF4	BANF1	0.16084
BGLF4	KXD1	0.22862
BGLF4	CPSF6	0.16637
BGLF4	MAST3	0.063983
BGLF4	FKBP14	0.062643
BGLF4	TOX4	0.19156
BGLF4	ARHGAP44	0.18795
BGLF4	ORC1	0.097875
BGLF4	LEO1	0.15365
BGLF4	CSRP2BP	0.13869
BGLF4	RORA	0.052811
BGLF4	ARL14EP	0.23693
BGLF4	NSMCE1	0.12963

46173	BGLF4	MED7	0.13938
46174	BGLF4	WAPAL	0.10619
46175	BGLF4	TNFSF10	0.089658
46176	BGLF4	GP1BB	0.17421
46177	BGLF4	REL	0.081037
46178	BGLF4	PCDHB10	0.053256
46179	BGLF4	BLCAP	0.21611
46180	BGLF4	AIMP2	0.15695
46181	BGLF4	APOA1BP	0.18306
46182	BGLF4	RPS7	0.12588
46183	BGLF4	LILRB2	0.1103
46184	BGLF4	PFKFB1	0.074995
46185	BGLF4	URI1	0.11187
46186	BGLF4	EME1	0.15794
46187	BGLF4	APPL2	0.09573
46188	BGLF4	RBM26	0.21384
46189	BGLF4	PSMG1	0.22311
46190	BGLF4	SEC61B	0.11676
46191	BGLF4	KIF2A	0.080323
46192	BGLF4	PATL2	0.19163
46193	BGLF4	IL7	0.11391
46194	BGLF4	SEPT14	0.17963
46195	BGLF4	NBR1	0.20838
46196	BGLF4	SENP6	0.14899
46197	BGLF4	EFCAB13	0.042785
46198	BGLF4	HFE	0.15352
46199	BGLF4	DPM1	0.12146
46200	BGLF4	SF3A3	0.13147
46201	BGLF4	IFT20	0.16748
46202	BGLF4	GHR	0.025627
46203	BGLF4	FBXW7	0.056185
46204	BGLF4	MAGEA6	0.22414
46205	BGLF4	RAB24	0.16776
46206	BGLF4	RAB8A	0.12282
46207	BGLF4	PFKL	0.090858
46208	BGLF4	CXADR	0.04424
46209	BGLF4	RAB11FIP2	0.11957
46210	BGLF4	TRO	0.17281
46211	BGLF4	ATP11C	0.10673
46212	BGLF4	CD27	0.13879
46213	BGLF4	LYST	0.1082
46214	BGLF4	CASQ1	0.039936
46215	BGLF4	ZNF652	0.15166
46216	BGLF4	C1orf87	0.068073
46217	BGLF4	CEP57	0.11674
46218	BGLF4	SCIMP	0.18727
46219	BGLF4	TIPIN	0.14725
46220	BGLF4	CHIC2	0.23584
46221	BGLF4	BNIP3L	0.070955
46222	BGLF4	AES	0.11903
46223	BGLF4	STX18	0.151
46224	BGLF4	VAMP7	0.091701
46225	BGLF4	N4BP2	0.1556
46226	BGLF4	SDC1	0.070665
46227	BGLF4	TRAM1	0.15352
46228	BGLF4	DCAF8	0.23579
46229	BGLF4	DNM1L	0.033391
46230	BGLF4	DLGAP3	0.15965
46231	BGLF4	USP9X	0.13117
46232	BGLF4	PDIA2	0.14115
46233	BGLF4	DERL1	0.12817
46234	BGLF4	RSF1	0.099859
46235	BGLF4	SMG8	0.1944
46236	BGLF4	GSN	0.17684
46237	BGLF4	RELB	0.087729
46238	BGLF4	SLC11A2	0.0415
46239	BGLF4	FAM96B	0.15901
46240	BGLF4	TRMT112	0.27714

46241	BGLF4	NUP133	0.10489
46242	BGLF4	PCDHGA4	0.057027
46243	BGLF4	PAIP2	0.20693
46244	BGLF4	NABP2	0.17689
46245	BGLF4	GCSAM	0.19715
46246	BGLF4	AMBRA1	0.24263
46247	BGLF4	ADCY4	0.061992
46248	BGLF4	MET	0.019934
46249	BGLF4	TREML2	0.20394
46250	BGLF4	PKP2	0.11197
46251	BGLF4	VPS13A	0.18833
46252	BGLF4	BMP2	0.01171
46253	BGLF4	MME	0.041065
46254	BGLF4	CEBPA	0.030428
46255	BGLF4	PTPN22	0.026618
46256	BGLF4	CSNK1D	0.030136
46257	BGLF4	HADHA	0.075208
46258	BGLF4	PCDHGA3	0.058173
46259	BGLF4	TBKBP1	0.24267
46260	BGLF4	PPP1R27	0.21731
46261	BGLF4	EIF4H	0.20831
46262	BGLF4	HK1	0.10186
46263	BGLF4	BPY2	0.20988
46264	BGLF4	TELO2	0.17607
46265	BGLF4	FRMPD2	0.19327
46266	BGLF4	CCNB3	0.099253
46267	BGLF4	PVRL1	0.11366
46268	BGLF4	IGFBP3	0.096068
46269	BGLF4	ASCL1	0.018969
46270	BGLF4	UBA2	0.030416
46271	BGLF4	IGFBP7	0.19073
46272	BGLF4	S100A16	0.043027
46273	BGLF4	BLNK	0.17157
46274	BGLF4	RPGRIP1L	0.10265
46275	BGLF4	ILF2	0.1643
46276	BGLF4	POT1	0.099778
46277	BGLF4	GDI1	0.16589
46278	BGLF4	PPFIA3	0.21613
46279	BGLF4	BMPR1B	0.022748
46280	BGLF4	PAM	0.090036
46281	BGLF4	CNTRL	0.18517
46282	BGLF4	CDK13	0.014885
46283	BGLF4	GOLGA2	0.21724
46284	BGLF4	PHACTR2	0.22899
46285	BGLF4	TMEM97	0.15605
46286	BGLF4	TRPC5	0.14267
46287	BGLF4	SP100	0.079182
46288	BGLF4	EIF4A1	0.15272
46289	BGLF4	TDRD7	0.17741
46290	BGLF4	MRPS12	0.15295
46291	BGLF4	CD24	0.058694
46292	BGLF4	CABP1	0.20928
46293	BGLF4	RHNO1	0.19191
46294	BGLF4	DDX5	0.075103
46295	BGLF4	PALD1	0.23023
46296	BGLF4	RBBP5	0.16405
46297	BGLF4	PILRA	0.19094
46298	BGLF4	YBX1	0.058887
46299	BGLF4	EAF1	0.20912
46300	BGLF4	AKAP1	0.073051
46301	BGLF4	SCEL	0.16823
46302	BGLF4	SLC4A4	0.19193
46303	BGLF4	PKN3	0.05005
46304	BGLF4	ARF5	0.12969
46305	BGLF4	LETM1	0.39904
46306	BGLF4	CRH	0.088884
46307	BGLF4	EIF2AK2	0.032699
46308	BGLF4	FSTL5	0.066109

BGLF4	NDRG1	0.11453
BGLF4	CINP	0.20491
BGLF4	TPR	0.089065
BGLF4	NEFM	0.14745
BGLF4	LEMD3	0.12332
BGLF4	BAG3	0.17965
BGLF4	SPHK1	0.074529
BGLF4	PACSIN3	0.17646
BGLF4	VAPA	0.062865
BGLF4	SCARF2	0.20289
BGLF4	MAPRE1	0.10449
BGLF4	CATSPER2	0.12236
BGLF4	GCA	0.076748
BGLF4	FNIP1	0.22957
BGLF4	TCTEX1D2	0.24432
BGLF4	VRK2	0.036101
BGLF4	SMG1	0.058744
BGLF4	ARAF	0.043162
BGLF4	SRSF10	0.15836
BGLF4	GPC3	0.11074
BGLF4	LRRC3	0.22976
BGLF4	RFC1	0.067323
BGLF4	PPP4R1	0.17628
BGLF4	TSSK2	0.041525
BGLF4	CUTA	0.20902
BGLF4	ELL	0.16335
BGLF4	CTU1	0.20406
BGLF4	PIAS4	0.099354
BGLF4	BCAS3	0.20039
BGLF4	ANGPT2	0.055896
BGLF4	MAD2L1BP	0.17791
BGLF4	ACTR1B	0.15122
BGLF4	PIK3CB	0.067118
BGLF4	RHOB	0.050293
BGLF4	EFHD2	0.081997
BGLF4	CYP4F2	0.037933
BGLF4	FBF1	0.1713
BGLF4	L3MBTL1	0.032927
BGLF4	MRPL12	0.22951
BGLF4	NFYC	0.066421
BGLF4	GTF2F1	0.069084
BGLF4	PKN1	0.02055
BGLF4	IL1RL1	0.12964
BGLF4	NSMAF	0.19852
BGLF4	NCAN	0.025992
BGLF4	TFB1M	0.23731
BGLF4	NCOA5	0.20561
BGLF4	ACHE	0.050642
BGLF4	DSG3	0.049477
BGLF4	YAP1	0.068119
BGLF4	MATR3	0.11776
BGLF4	PCDHB18	0.058173
BGLF4	PPP2CA	0.047924
BGLF4	CEP164	0.16481
BGLF4	MLLT1	0.17697
BGLF4	RCC1	0.11527
BGLF4	PABPC1	0.10092
BGLF4	PLEKHF2	0.12372
BGLF4	JMY	0.14115
BGLF4	SNTB1	0.11497
BGLF4	AMER1	0.19283
BGLF4	TRIP11	0.1422
BGLF4	RTF1	0.1174
BGLF4	RWDD3	0.20848
BGLF4	PRKAG1	0.015606
BGLF4	MT3	0.034532
BGLF4	LUC7L3	0.16855
BGLF4	MCM5	0.10023

BGLF4	CD28	0.1012
BGLF4	CTDSP1	0.10518
BGLF4	HLA-DRB1	0.045061
BGLF4	PNRC1	0.20561
BGLF4	IRS1	0.013614
BGLF4	ITIH1	0.057215
BGLF4	PRTN3	0.20237
BGLF4	MAL	0.18379
BGLF4	FAM188A	0.389
BGLF4	ZP3	0.045772
BGLF4	BCKDK	0.059135
BGLF4	MCF2	0.10021
BGLF4	UMOD	0.066526
BGLF4	C6	0.16691
BGLF4	HDHD3	0.20194
BGLF4	IPO8	0.17036
BGLF4	RNF43	0.085685
BGLF4	STX2	0.11434
BGLF4	RHBDL3	0.13832
BGLF4	AP1B1	0.1951
BGLF4	CCNT1	0.068415
BGLF4	GAPDH	0.10758
BGLF4	NRD1	0.03326
BGLF4	DNM2	0.021576
BGLF4	GRB10	0.10859
BGLF4	NBN	0.043673
BGLF4	RCN3	0.43741
BGLF4	MGST1	0.074736
BGLF4	RNF5	0.11135
BGLF4	PLSCR1	0.18325
BGLF4	CETN3	0.084875
BGLF4	SNX7	0.026854
BGLF4	CTNNBL1	0.18086
BGLF4	KDM2B	0.1111
BGLF4	FDXR	0.13008
BGLF4	FLT1	0.019354
BGLF4	BCLAF1	0.15319
BGLF4	CALY	0.01645
BGLF4	EFTUD2	0.12813
BGLF4	SERPINE2	0.040368
BGLF4	YY1AP1	0.18851
BGLF4	NF1	0.027704
BGLF4	F8	0.16332
BGLF4	FBXO45	0.17996
BGLF4	KIF21A	0.15748
BGLF4	SCAMP2	0.12633
BGLF4	PCDH10	0.058173
BGLF4	GIT1	0.1305
BGLF4	GRIN2B	0.014221
BGLF4	SLC6A4	0.085964
BGLF4	CDH19	0.056464
BGLF4	VEGFC	0.095165
BGLF4	CLK2	0.052324
BGLF4	SLC12A3	0.17448
BGLF4	SNAP25	0.053696
BGLF4	DDX23	0.10109
BGLF4	LSM10	0.15929
BGLF4	SUPV3L1	0.067224
BGLF4	PSENEN	0.12872
BGLF4	ATN1	0.14843
BGLF4	LRPPRC	0.15936
BGLF4	VAPB	0.067695
BGLF4	NHEJ1	0.1604
BGLF4	ESR1	0.013695
BGLF4	STAT5B	0.030235
BGLF4	COPRS	0.18763
BGLF4	RPA1	0.057648
BGLF4	HSPG2	0.084839

BGLF4	CELSR2	0.027487
BGLF4	SMARCA4	0.063834
BGLF4	SGCA	0.059214
BGLF4	ATG5	0.14751
BGLF4	ACTL6A	0.10165
BGLF4	PAXIP1	0.14661
BGLF4	EGFR	0.011056
BGLF4	ACTR8	0.19298
BGLF4	CAMLG	0.20347
BGLF4	SELP	0.070349
BGLF4	FBXO5	0.051703
BGLF4	PTGDR	0.17962
BGLF4	APAF1	0.095836
BGLF4	SIKE1	0.18701
BGLF4	CTCF	0.035254
BGLF4	HSPD1	0.030959
BGLF4	PPFIA2	0.22279
BGLF4	PCDHGA8	0.058173
BGLF4	MST1R	0.061445
BGLF4	SCIN	0.042206
BGLF4	CCDC33	0.24432
BGLF4	NPC1	0.081178
BGLF4	SYTL5	0.18674
BGLF4	HINFP	0.041203
BGLF4	MORC4	0.16858
BGLF4	MPLKIP	0.18845
BGLF4	PLP2	0.18607
BGLF4	RAB11A	0.098117
BGLF4	LTBP1	0.39648
BGLF4	KIF5A	0.11771
BGLF4	PEX6	0.14443
BGLF4	SMARCC1	0.13717
BGLF4	TPD52L2	0.23843
BGLF4	ASPSCR1	0.15909
BGLF4	ARL6IP4	0.20848
BGLF4	F2	0.11367
BGLF4	CELF1	0.14562
BGLF4	RHOXF2	0.10141
BGLF4	WDYHV1	0.16549
BGLF4	SYT7	0.10264
BGLF4	FAAP20	0.15337
BGLF4	DLG1	0.014147
BGLF4	THOC2	0.23145
BGLF4	HES1	0.01287
BGLF4	PLEKHA2	0.10898
BGLF4	EREG	0.071454
BGLF4	IFIT2	0.17489
BGLF4	HEATR1	0.15131
BGLF4	SCNN1B	0.15133
BGLF4	SMYD2	0.14568
BGLF4	ITPR1	0.074541
BGLF4	VAMP3	0.14191
BGLF4	WIPI2	0.14545
BGLF4	CDH9	0.051735
BGLF4	SPAST	0.074373
BGLF4	PCDHB9	0.053256
BGLF4	RRAS	0.11648
BGLF4	MIEF2	0.16031
BGLF4	FST	0.11403
BGLF4	CEP290	0.1074
BGLF4	KDM5B	0.051218
BGLF4	NOXO1	0.13096
BGLF4	SYT1	0.14906
BGLF4	NSMCE2	0.10548
BGLF4	MRPS16	0.17009
BGLF4	RND2	0.18162
BGLF4	HMOX2	0.12027
BGLF4	WRNIP1	0.12731

BGLF4	TCOF1	0.20286
BGLF4	CBL	0.14761
BGLF4	NEK3	0.060165
BGLF4	CORO1A	0.081788
BGLF4	PHF12	0.1315
BGLF4	SLX4	0.15948
BGLF4	ANKRD27	0.19659
BGLF4	EPHA2	0.029605
BGLF4	MYC	0.018984
BGLF4	ARFRP1	0.12741
BGLF4	UCHL5	0.097735
BGLF4	PRPF8	0.1842
BGLF4	VIM	0.045543
BGLF4	NOA1	0.12738
BGLF4	CTAG1A	0.23693
BGLF4	PEX26	0.16916
BGLF4	WARS	0.090626
BGLF4	TIMM10	0.1273
BGLF4	SLC25A13	0.047435
BGLF4	KLRC2	0.19745
BGLF4	NPM1	0.029225
BGLF4	GDF2	0.087822
BGLF4	NAP1L4	0.15882
BGLF4	MYCBP	0.17733
BGLF4	PI4K2A	0.10725
BGLF4	GRB2	0.022198
BGLF4	ADNP2	0.051532
BGLF4	ZNF639	0.034988
BGLF4	NCS1	0.2812
BGLF4	TCAP	0.13113
BGLF4	HPSE	0.088912
BGLF4	CDK2	0.014199
BGLF4	MBL2	0.10874
BGLF4	NCK2	0.11746
BGLF4	IKZF3	0.051776
BGLF4	NMNAT1	0.14747
BGLF4	GPKOW	0.1913
BGLF4	FBLN5	0.32578
BGLF4	MPP6	0.22282
BGLF4	ARHGAP1	0.11422
BGLF4	CRBN	0.15429
BGLF4	HSCB	0.19752
BGLF4	BIRC5	0.037612
BGLF4	DAG1	0.095504
BGLF4	AP5S1	0.16668
BGLF4	MCM3	0.068625
BGLF4	TOMM20	0.1119
BGLF4	GLTSCR1	0.24432
BGLF4	OTOF	0.064007
BGLF4	AGTRAP	0.21718
BGLF4	ID2	0.048787
BGLF4	ZNF133	0.065177
BGLF4	PACS1	0.17847
BGLF4	DOC2A	0.20605
BGLF4	MED30	0.18572
BGLF4	RHOBTB3	0.15981
BGLF4	XRCC3	0.13982
BGLF4	F11	0.17918
BGLF4	SRGAP2	0.096877
BGLF4	CALCOCO1	0.12463
BGLF4	FBXO6	0.18204
BGLF4	VPS35	0.15518
BGLF4	HRK	0.17006
BGLF4	ADAM22	0.12481
BGLF4	PCDHA12	0.058173
BGLF4	TBC1D9B	0.036637
BGLF4	PTPRT	0.045289
BGLF4	SIGLEC8	0.20323

BGLF4	SNRNP40	0.15952
BGLF4	ARID1A	0.10985
BGLF4	SCNN1D	0.19774
BGLF4	STON2	0.18008
BGLF4	SNX22	0.026854
BGLF4	CENPE	0.061719
BGLF4	HSPA8	0.027713
BGLF4	PLK2	0.03982
BGLF4	FBXW11	0.12503
BGLF4	SCAI	0.17608
BGLF4	KCNMB4	0.15545
BGLF4	NIPBL	0.089706
BGLF4	RPL7A	0.17021
BGLF4	KMT2A	0.064256
BGLF4	UBE2C	0.076867
BGLF4	SERPINA3	0.15577
BGLF4	ADRBK1	0.019071
BGLF4	DOK1	0.09503
BGLF4	IL7R	0.10973
BGLF4	MYL5	0.058125
BGLF4	TPM4	0.036049
BGLF4	NDUFAF3	0.18087
BGLF4	DYX1C1	0.18133
BGLF4	BMF	0.12665
BGLF4	INTS2	0.17472
BGLF4	RAB22A	0.12322
BGLF4	MYO7A	0.071738
BGLF4	PIAS2	0.093363
BGLF4	MUC2	0.21602
BGLF4	SPTLC1	0.11442
BGLF4	NLK	0.030147
BGLF4	ITGA6	0.15348
BGLF4	PILRB	0.20927
BGLF4	CTNNA2	0.081615
BGLF4	DEPTOR	0.2037
BGLF4	FTH1	0.13544
BGLF4	NOD1	0.040064
BGLF4	RCAN1	0.071235
BGLF4	DGKB	0.063673
BGLF4	ZFYVE9	0.20751
BGLF4	PDS5A	0.18559
BGLF4	TIGIT	0.15214
BGLF4	CARS	0.067438
BGLF4	RNF8	0.066097
BGLF4	DPEP1	0.078719
BGLF4	RFX5	0.097266
BGLF4	C8orf48	0.24432
BGLF4	NPHS1	0.12739
BGLF4	TAL1	0.018995
BGLF4	NECAB3	0.32893
BGLF4	AKAP8L	0.16516
BGLF4	SNX12	0.23861
BGLF4	ATM	0.025592
BGLF4	TCHH	0.047722
BGLF4	RASSF9	0.19131
BGLF4	GORASP2	0.17832
BGLF4	KIR2DL4	0.19148
BGLF4	MLC1	0.14856
BGLF4	LBP	0.06946
BGLF4	ADAP2	0.02226
BGLF4	TRIM16	0.11633
BGLF4	IFI35	0.20521
BGLF4	EDA	0.056826
BGLF4	PXMP4	0.20964
BGLF4	ASGR2	0.1766
BGLF4	STAG1	0.14127
BGLF4	EFNA4	0.24325
BGLF4	SIN3A	0.026764

BGLF4	DYNC1H1	0.10681
BGLF4	GRM7	0.024875
BGLF4	CNTNAP1	0.097641
BGLF4	PBXIP1	0.14065
BGLF4	RRAGD	0.1529
BGLF4	TRMT61B	0.24685
BGLF4	CCR1	0.090478
BGLF4	SDC4	0.12888
BGLF4	LONP2	0.10567
BGLF4	HSPA14	0.16437
BGLF4	PLXNB2	0.1502
BGLF4	APH1B	0.17658
BGLF4	SNX6	0.21442
BGLF4	APOA5	0.07293
BGLF4	KRT1	0.12498
BGLF4	ZNF143	0.12113
BGLF4	BRCA1	0.019741
BGLF4	RFX1	0.18709
BGLF4	CDK20	0.034751
BGLF4	KRT81	0.17791
BGLF4	ERGIC1	0.14589
BGLF4	RALB	0.1151
BGLF4	CDC73	0.11535
BGLF4	GSE1	0.24432
BGLF4	NCOA1	0.084176
BGLF4	FGA	0.14574
BGLF4	RASGRP1	0.021712
BGLF4	HLTF	0.06602
BGLF4	DUSP7	0.034462
BGLF4	PDE4A	0.092239
BGLF4	ZFYVE1	0.12823
BGLF4	IFNAR2	0.095442
BGLF4	NMI	0.18392
BGLF4	CLSTN3	0.016916
BGLF4	CDK5RAP3	0.12035
BGLF4	TRIM28	0.011405
BGLF4	PCDHGB6	0.058173
BGLF4	CHMP4A	0.018169
BGLF4	WWTR1	0.099001
BGLF4	PCDHB11	0.050061
BGLF4	ASF1A	0.13711
BGLF4	ERRFI1	0.059167
BGLF4	C19orf25	0.24432
BGLF4	IRF7	0.02226
BGLF4	MAPK9	0.033129
BGLF4	BBS2	0.10665
BGLF4	APOA2	0.031506
BGLF4	XPA	0.1109
BGLF4	CHMP5	0.20431
BGLF4	EIF2S2	0.23374
BGLF4	ZFYVE16	0.085617
BGLF4	NMRK2	0.17567
BGLF4	PPARG	0.01243
BGLF4	PHB2	0.1479
BGLF4	DCP1B	0.11971
BGLF4	MYB	0.064552
BGLF4	SNRPD1	0.14932
BGLF4	SOCS1	0.11405
BGLF4	AKAP7	0.033053
BGLF4	C1S	0.08139
BGLF4	NPBWR1	0.16273
BGLF4	SNCB	0.036443
BGLF4	EGLN3	0.11813
BGLF4	NPC1L1	0.22418
BGLF4	PCGF2	0.056539
BGLF4	DNMBP	0.16748
BGLF4	PRAM1	0.25804
BGLF4	EFNB1	0.15454

BGLF4	NT5C3B	0.16171
BGLF4	CDYL2	0.17396
BGLF4	AASDHPPT	0.1817
BGLF4	FHL1	0.1129
BGLF4	GGA3	0.16008
BGLF4	UTP11L	0.1631
BGLF4	RSRC1	0.16622
BGLF4	GLRA1	0.061707
BGLF4	AQP5	0.17253
BGLF4	ARL4C	0.10801
BGLF4	LOR	0.081223
BGLF4	NR0B2	0.075676
BGLF4	ARHGAP21	0.15821
BGLF4	ARL3	0.046688
BGLF4	MEGF6	0.42584
BGLF4	MED20	0.12727
BGLF4	RPS6KA1	0.01678
BGLF4	STAG2	0.13341
BGLF4	EIF2S3	0.17514
BGLF4	NFRKB	0.18149
BGLF4	LAX1	0.10536
BGLF4	CALR	0.0449
BGLF4	JPH2	0.13504
BGLF4	BTBD1	0.20849
BGLF4	SOCS5	0.19216
BGLF4	EMX2	0.050911
BGLF4	MRPL11	0.19627
BGLF4	EGR1	0.039
BGLF4	MC2R	0.13279
BGLF4	NUP205	0.15203
BGLF4	RAB33A	0.14831
BGLF4	DHX9	0.082826
BGLF4	HOXA10	0.073726
BGLF4	HSP90AA1	0.090236
BGLF4	TAF1	0.035093
BGLF4	GAS1	0.057636
BGLF4	TSPY1	0.13833
BGLF4	TNFRSF14	0.14512
BGLF4	LYL1	0.18001
BGLF4	NUB1	0.18784
BGLF4	NFKBIL1	0.1811
BGLF4	CBX5	0.085941
BGLF4	ZFAND2B	0.1946
BGLF4	F12	0.14148
BGLF4	PCBD1	0.090643
BGLF4	NRBP1	0.053656
BGLF4	SLC25A12	0.035877
BGLF4	ATF4	0.05416
BGLF4	PCNP	0.19949
BGLF4	ATP6AP2	0.16658
BGLF4	PCDHA1	0.053108
BGLF4	ANK1	0.051256
BGLF4	PCBP1	0.19474
BGLF4	HSF1	0.067654
BGLF4	UNC5B	0.16626
BGLF4	SCRIB	0.099447
BGLF4	RPTOR	0.069068
BGLF4	SRRT	0.16092
BGLF4	SSTR3	0.12105
BGLF4	DGKG	0.05933
BGLF4	KHDRBS2	0.22793
BGLF4	MMP9	0.036715
BGLF4	PLXNB3	0.17725
BGLF4	KCNIP2	0.20313
BGLF4	TBCD	0.14618
BGLF4	THEG	0.19073
BGLF4	SLPI	0.17682
BGLF4	PDLIM4	0.20031

BGLF4	DAO	0.10477
BGLF4	PCDHA13	0.058173
BGLF4	KIF3B	0.099376
BGLF4	PRSS8	0.20317
BGLF4	FSCN1	0.16123
BGLF4	TRAF1	0.15184
BGLF4	CALML3	0.068073
BGLF4	PROX1	0.014612
BGLF4	POLG2	0.11702
BGLF4	ITGB1BP1	0.19053
BGLF4	MED25	0.11235
BGLF4	RAMP3	0.12784
BGLF4	AGO4	0.17669
BGLF4	HPS1	0.17277
BGLF4	CRK	0.12574
BGLF4	RFWD2	0.10814
BGLF4	SPOCK3	0.058267
BGLF4	PIAS3	0.091024
BGLF4	CDH4	0.040444
BGLF4	TNS1	0.19155
BGLF4	ITGA2B	0.12817
BGLF4	PAFAH1B1	0.020369
BGLF4	MAPK13	0.063429
BGLF4	ADAM12	0.07642
BGLF4	UBA52	0.022147
BGLF4	INO80E	0.19744
BGLF4	KCNIP3	0.027695
BGLF4	SNRPN	0.20467
BGLF4	CDK14	0.030506
BGLF4	PARD6A	0.094393
BGLF4	SMARCA2	0.053635
BGLF4	PLCG1	0.0771
BGLF4	FAM109B	0.17206
BGLF4	ITGAX	0.16592
BGLF4	PDCD4	0.20663
BGLF4	PPP1R32	0.23184
BGLF4	PPP1R8	0.1632
BGLF4	NOTCH1	0.013758
BGLF4	PCLO	0.045127
BGLF4	IL8	0.10105
BGLF4	FBN2	0.06344
BGLF4	ZNHIT1	0.21811
BGLF4	TRAF4	0.052868
BGLF4	EYA2	0.045062
BGLF4	ERCC1	0.040182
BGLF4	PSME3	0.075327
BGLF4	ELOVL2	0.13771
BGLF4	EFCAB10	0.063928
BGLF4	CD93	0.29126
BGLF4	MUC7	0.20916
BGLF4	SRR	0.038136
BGLF4	EEF1B2	0.17717
BGLF4	PPP1R2	0.20139
BGLF4	TEX11	0.17985
BGLF4	LMX1B	0.070363
BGLF4	NLRC5	0.10071
BGLF4	DRD1	0.017895
BGLF4	RITA1	0.15945
BGLF4	GRIN1	0.12957
BGLF4	CST3	0.054364
BGLF4	FAN1	0.14239
BGLF4	CLINT1	0.24936
BGLF4	IRF5	0.042439
BGLF4	EIF2AK3	0.024559
BGLF4	ZBTB21	0.14271
BGLF4	MARK4	0.053347
BGLF4	BBS12	0.18346
BGLF4	DHH	0.26462

BGLF4	SLC25A4	0.092675
BGLF4	SPATA21	0.068073
BGLF4	NDFIP2	0.11756
BGLF4	IK	0.19622
BGLF4	NCOA2	0.04695
BGLF4	CRIPAK	0.18018
BGLF4	PIGS	0.16453
BGLF4	SIRT2	0.040798
BGLF4	IMMT	0.19302
BGLF4	UBE2L3	0.065429
BGLF4	IL5	0.15472
BGLF4	TOMM7	0.13339
BGLF4	RRM1	0.13052
BGLF4	ILK	0.01186
BGLF4	FLT4	0.035844
BGLF4	MLF1	0.16331
BGLF4	STRN3	0.077637
BGLF4	RAP1B	0.093899
BGLF4	IKBKG	0.058972
BGLF4	SPHK2	0.14171
BGLF4	MARK3	0.069254
BGLF4	HDAC1	0.015113
BGLF4	NR2F6	0.058454
BGLF4	VIPAS39	0.1961
BGLF4	ADAM15	0.11736
BGLF4	PRKCB	0.023615
BGLF4	PLD3	0.097339
BGLF4	PCDHGA10	0.058173
BGLF4	ADAM8	0.069822
BGLF4	TRIP12	0.15831
BGLF4	CSTF1	0.20941
BGLF4	SKA2	0.12156
BGLF4	U2AF2	0.16833
BGLF4	MAP3K8	0.042205
BGLF4	NOSTRIN	0.22439
BGLF4	TCL1A	0.14953
BGLF4	ELMO1	0.10226
BGLF4	SS18	0.14347
BGLF4	U2SURP	0.22696
BGLF4	AICDA	0.07793
BGLF4	RPS9	0.10443
BGLF4	DCHS2	0.058173
BGLF4	SNX18	0.18958
BGLF4	TNFRSF13B	0.19308
BGLF4	SBDS	0.088174
BGLF4	CDC42EP1	0.16371
BGLF4	ADRB1	0.041559
BGLF4	RPL28	0.17554
BGLF4	SIRT7	0.11903
BGLF4	AP1G2	0.18319
BGLF4	PREX1	0.060408
BGLF4	ATP2C2	0.11148
BGLF4	PCDHGC3	0.056436
BGLF4	PDCD10	0.15073
BGLF4	MRM1	0.043747
BGLF4	USP44	0.13286
BGLF4	ZBTB17	0.05159
BGLF4	PURA	0.021464
BGLF4	GFI1B	0.069641
BGLF4	CCBE1	0.049259
BGLF4	BLMH	0.137
BGLF4	IRAK1	0.0238
BGLF4	CXCR4	0.063249
BGLF4	USP37	0.087573
BGLF4	ARFIP2	0.16298
BGLF4	SAC3D1	0.18338
BGLF4	MMS22L	0.1873
BGLF4	MTMR9	0.20327

BGLF4	COL4A2	0.16148
BGLF4	PKD1L2	0.05836
BGLF4	CHMP1A	0.11385
BGLF4	PPM1G	0.091768
BGLF4	XPO1	0.10346
BGLF4	LBR	0.15989
BGLF4	ATP8B1	0.12681
BGLF4	TJP2	0.17289
BGLF4	NR1I2	0.060154
BGLF4	ITM2A	0.22976
BGLF4	RBBP4	0.10362
BGLF4	YWHAQ	0.20212
BGLF4	HOOK1	0.12795
BGLF4	INPP5K	0.031237
BGLF4	PPP1R3C	0.14403
BGLF4	ATP10A	0.093487
BGLF4	KLK5	0.21997
BGLF4	MARCH5	0.069882
BGLF4	PPP1R15A	0.19066
BGLF4	STT3A	0.18947
BGLF4	ZBTB24	0.15166
BGLF4	TCF12	0.057412
BGLF4	SPC24	0.14553
BGLF4	NCF2	0.057859
BGLF4	CCL3	0.026665
BGLF4	MYL6B	0.3487
BGLF4	PCK2	0.12323
BGLF4	CNIH2	0.12671
BGLF4	GLI1	0.046561
BGLF4	GIPC1	0.081429
BGLF4	RAD51C	0.1081
BGLF4	NAA38	0.18871
BGLF4	IGF2BP2	0.15024
BGLF4	YWHAB	0.077599
BGLF4	SLC25A5	0.15043
BGLF4	GNA13	0.069584
BGLF4	BLOC1S5	0.1836
BGLF4	TRIM21	0.092718
BGLF4	SPTSSB	0.15482
BGLF4	TAF15	0.17376
BGLF4	RBX1	0.076274
BGLF4	TNNI3K	0.047193
BGLF4	WDR82	0.17713
BGLF4	RAB3A	0.058751
BGLF4	NSL1	0.13317
BGLF4	HMGB2	0.020862
BGLF4	RHOH	0.15337
BGLF4	TCHP	0.17646
BGLF4	ATP6V0A1	0.16983
BGLF4	BRPF3	0.15517
BGLF4	GTF2I	0.088474
BGLF4	CPSF3	0.12705
BGLF4	KCNMA1	0.085122
BGLF4	ORC6	0.15831
BGLF4	TGM3	0.019549
BGLF4	CELSR1	0.02832
BGLF4	UBQLN2	0.1961
BGLF4	PLRG1	0.17733
BGLF4	CUL1	0.11229
BGLF4	SCG5	0.16863
BGLF4	CD9	0.12921
BGLF4	DHX58	0.087752
BGLF4	MYO9A	0.10853
BGLF4	SP3	0.06678
BGLF4	POLI	0.1111
BGLF4	SLC24A1	0.18812
BGLF4	DR1	0.12138
BGLF4	TBK1	0.030974

BGLF4	HSPA9	0.10889
BGLF4	NCSTN	0.07939
BGLF4	PLSCR2	0.058805
BGLF4	GSTP1	0.076554
BGLF4	MMP14	0.075051
BGLF4	ELP3	0.02541
BGLF4	PIGK	0.095272
BGLF4	CDC5L	0.19517
BGLF4	MBNL1	0.13973
BGLF4	MED4	0.16246
BGLF4	RHBDD1	0.12584
BGLF4	TIE1	0.077333
BGLF4	FABP5	0.19858
BGLF4	HRC	0.057633
BGLF4	AP1G1	0.15525
BGLF4	HMCN2	0.085066
BGLF4	CDHR2	0.36346
BGLF4	FAS	0.030749
BGLF4	APOE	0.012175
BGLF4	TUBB2B	0.052927
BGLF4	WAS	0.13543
BGLF4	ALDOC	0.098074
BGLF4	TFG	0.18938
BGLF4	SNRPB2	0.15557
BGLF4	TNNI3	0.07174
BGLF4	ILF3	0.14742
BGLF4	WDR61	0.15213
BGLF4	SULT2B1	0.13237
BGLF4	PAWR	0.1286
BGLF4	ASIC2	0.17565
BGLF4	CBX3	0.10702
BGLF4	RINT1	0.18065
BGLF4	SHANK3	0.080443
BGLF4	IKBIP	0.19307
BGLF4	RCVRN	0.077134
BGLF4	AGFG1	0.16713
BGLF4	INTS5	0.18996
BGLF4	C17orf62	0.22976
BGLF4	SNX17	0.16377
BGLF4	CDH5	0.25628
BGLF4	DDX58	0.046478
BGLF4	ARID1B	0.1365
BGLF4	IL6R	0.083645
BGLF4	HS3ST5	0.15183
BGLF4	TP53INP2	0.21638
BGLF4	ZNF165	0.072217
BGLF4	SEMA6A	0.13972
BGLF4	ZMYND10	0.19427
BGLF4	COTL1	0.18892
BGLF4	SMN1	0.19632
BGLF4	SNIP1	0.15615
BGLF4	SYT2	0.29937
BGLF4	CCNY	0.083434
BGLF4	CAPNS1	0.29389
BGLF4	GTF2E2	0.16975
BGLF4	KCNG3	0.158
BGLF4	TRIM41	0.12838
BGLF4	MPZL3	0.20901
BGLF4	GABARAPL1	0.21895
BGLF4	GTF2B	0.11472
BGLF4	ANGPTL4	0.11881
BGLF4	WDR16	0.19737
BGLF4	IFIT1	0.18321
BGLF4	PCDHB15	0.05466
BGLF4	RAD18	0.083338
BGLF4	DNM1	0.042966
BGLF4	OCM2	0.068073
BGLF4	SIPA1	0.11819

BGLF4	TPD52	0.38188
BGLF4	MC5R	0.16065
BGLF4	RIPK4	0.064219
BGLF4	POLDIP3	0.19984
BGLF4	IRS2	0.039197
BGLF4	ARID5B	0.10252
BGLF4	NUFIP1	0.14764
BGLF4	KLC1	0.18612
BGLF4	MRPS31	0.17462
BGLF4	FRMPD4	0.23834
BGLF4	LIG4	0.03519
BGLF4	KLHDC3	0.20153
BGLF4	XRCC5	0.041575
LMP1	ubl4ab	0.053101
LMP1	SPTA1	0.020268
LMP1	S100A7	0.049285
LMP1	HSPA5	0.027169
LMP1	LDLR	0.019115
LMP1	C12orf61	0.052596
LMP1	PLSCR3	0.059517
LMP1	ATP5B	0.041277
LMP1	NIN	0.051763
LMP1	KCNIP1	0.056443
LMP1	STIM2	0.062444
LMP1	SPTAN1	0.035362
LMP1	MASP1	0.11601
LMP1	SCUBE3	0.084282
LMP1	PROC	0.096226
LMP1	PDCD6	0.10662
LMP1	S100A9	0.019918
LMP1	ANXA2	0.043681
LMP1	MATN4	0.085013
LMP1	RNH1	0.065198
LMP1	CLIP1	0.028911
LMP1	THBD	0.045448
LMP1	EML1	0.080071
LMP1	EPS15	0.01873
LMP1	AP3S1	0.013836
LMP1	SDF4	0.061759
LMP1	FAT4	0.068568
LMP1	PPEF1	0.018487
LMP1	CXorf67	0.051995
LMP1	FAM188B2	0.03485
LMP1	SYT11	0.11371
LMP1	HSP90B1	0.046711
LMP1	MACF1	0.055014
LMP1	CCDC176	0.069195
LMP1	GCH1	0.02292
LMP1	NKD2	0.1001
LMP1	FAT1	0.031213
LMP1	S100A11	0.062286
LMP1	CCDC137	0.017946
LMP1	NKD1	0.05795
LMP1	PPP2R3B	0.016058
LMP1	ACTN4	0.033226
LMP1	EHD1	0.012247
LMP1	S100A13	0.080304
LMP1	S100A1	0.03077
LMP1	UTRN	0.01463
LMP1	CALB1	0.066925
LMP1	MASP2	0.10144
LMP1	ANXA2R	0.028942
LMP1	EFEMP1	0.089673
LMP1	SPARC	0.038934
LMP1	NOTCH4	0.011099
LMP1	PCDH17	0.068568
LMP1	KIAA1841	0.018829
LMP1	REPS1	0.062293

LMP1	LRCOL1	0.037431
LMP1	MPTX1	0.014618
LMP1	STIM1	0.026283
LMP1	S100B	0.029823
LMP1	TNNC1	0.079609
LMP1	CETN2	0.078578
LMP1	ANXA6	0.0767
LMP1	ACTN2	0.029494
LMP1	PRSS2	0.088209
LMP1	PITPNM3	0.06541
LMP1	VIL1	0.041221
LMP1	CETN1	0.095644
LMP1	C2CD4D	0.010147
LMP1	VLDLR	0.035748
LMP1	CDH1	0.017421
LMP1	TTN	0.01529
LMP1	EGF	0.025077
LMP1	LTBP4	0.068735
LMP1	PLSCR4	0.056729
LMP1	SNX9	0.034263
LMP1	RYR2	0.011583
LMP1	PRF1	0.019246
LMP1	IL6ST	0.042827
LMP1	MYO1E	0.010441
LMP1	S100A8	0.030993
LMP1	NID2	0.091531
LMP1	NELL1	0.03476
LMP1	ytzL	0.041977
LMP1	DNER	0.041096
LMP1	PEF1	0.082612
LMP1	C10orf82	0.010816
LMP1	CHP1	0.037886
LMP1	FASN	0.03141
LMP1	GPR98	0.022129
LMP1	MICU1	0.0611
LMP1	CALU	0.067248
LMP1	LRP2	0.030663
LMP1	FAM188B	0.057059
LMP1	NUCB2	0.049842
LMP1	S100A6	0.052025
LMP1	LRP8	0.063919
LMP1	CT55	0.048708
LMP1	S100P	0.03642
LMP1	EFCAB4B	0.052437
LMP1	DTNA	0.02286
LMP1	REPS2	0.09037
LMP1	PPEF2	0.029649
LMP1	EFEMP2	0.082858
LMP1	ATP2A1	0.021908
LMP1	CAGE1	0.014089
LMP1	PKD2L1	0.03885
LMP1	EHD3	0.018247
LMP1	THBS2	0.065403
LMP1	CALM1	0.045068
LMP1	S100A4	0.090105
LMP1	S100A12	0.041317
LMP1	RCN1	0.090347
LMP1	ACTN3	0.088423
LMP1	ATP5F1	0.014105
LMP1	FBN1	0.089206
LMP1	F10	0.032283
LMP1	CELSR3	0.034796
LMP1	TESC	0.05552
LMP1	C12orf57	0.019204
LMP1	RPH3A	0.027497
LMP1	SRI	0.033516
LMP1	LRP4	0.040335
LMP1	SLIT2	0.018697

LMP1	MATN2	0.072802
LMP1	CIB1	0.057692
LMP1	DLL1	0.076892
LMP1	ACTN1	0.037835
LMP1	PRSS3	0.10425
LMP1	MYL2	0.10504
LMP1	EDEM1	0.027985
LMP1	LRP1	0.013737
LMP1	SNX19	0.035689
LMP1	PDGFRB	0.01055
LMP1	DST	0.047744
LMP1	MYL6	0.085203
LMP1	COMP	0.060555
LMP1	ANXA7	0.020783
LMP1	CHP2	0.059411
LMP1	NECAB2	0.087577
LMP1	CANX	0.033568
LMP1	ANXA11	0.030914
LMP1	MATN3	0.096643
LMP1	FAM47C	0.02503
LMP1	RET	0.033236
LMP1	CDH23	0.014603
LMP1	RAB11FIP4	0.13086
LMP1	C1orf64	0.028137
LMP1	CLIC4	0.027066
LMP1	FAM49B	0.025
LMP1	BNIP2	0.021015
LMP1	JAG2	0.052621
LMP1	CADPS	0.10698
LMP1	CCAR1	0.084266
LMP1	C1orf195	0.055176
LMP1	CRELD2	0.080747
LMP1	GSN	0.032955
LMP1	SLC11A2	0.013205
LMP1	LETM1	0.069135
LMP1	FAM231A	0.091531
LMP1	FAM188A	0.052866
LMP1	RCN3	0.084651
LMP1	PLSCR1	0.03192
LMP1	NKAPL	0.035366
LMP1	TLDC2	0.015658
LMP1	LTBP1	0.088532
LMP1	F2	0.018336
LMP1	SYT1	0.027837
LMP1	NSUN5P1	0.038093
LMP1	NCS1	0.083076
LMP1	FBLN5	0.084266
LMP1	DAG1	0.016482
LMP1	NECAB3	0.040261
LMP1	MEGF6	0.084048
LMP1	CALR	0.010295
LMP1	KCNIP2	0.019503
LMP1	HEATR5B	0.035535
LMP1	CD93	0.049935
LMP1	GRIN1	0.026319
LMP1	DHH	0.059639
LMP1	N4BP2L1	0.017654
LMP1	FAM231D	0.021873
LMP1	C11orf72	0.013254
LMP1	MYL6B	0.064096
LMP1	CDHR2	0.063202
LMP1	CDH5	0.037613
LMP1	SYT2	0.074436
LMP1	TPD52	0.091645
LMP2	ubl4ab	0.063273
LMP2	SPTA1	0.030226
LMP2	S100A7	0.06421
LMP2	HSPA5	0.035394

LMP2	LDLR	0.02455
LMP2	C12orf61	0.06272
LMP2	PLSCR3	0.077982
LMP2	YMR013W-A	0.012269
LMP2	ATP5B	0.049903
LMP2	NIN	0.071767
LMP2	KCNIP1	0.072051
LMP2	STIM2	0.080034
LMP2	SPTAN1	0.048043
LMP2	MASP1	0.14052
LMP2	SCUBE3	0.10995
LMP2	PROC	0.11712
LMP2	PDCD6	0.12819
LMP2	S100A9	0.026167
LMP2	ANXA2	0.05572
LMP2	MATN4	0.1119
LMP2	NOTCH3	0.016329
LMP2	RNH1	0.088976
LMP2	CLIP1	0.038706
LMP2	THBD	0.05904
LMP2	EML1	0.10533
LMP2	EPS15	0.029949
LMP2	AP3S1	0.0264
LMP2	SDF4	0.080044
LMP2	SPACA3	0.044303
LMP2	FAT4	0.090123
LMP2	DTNBP1	0.011712
LMP2	PPEF1	0.035738
LMP2	CXorf67	0.064403
LMP2	FAM188B2	0.0474
LMP2	THBS1	0.011777
LMP2	SYT11	0.13671
LMP2	HSP90B1	0.060012
LMP2	MACF1	0.069305
LMP2	CCDC176	0.081332
LMP2	GCH1	0.027809
LMP2	NKD2	0.12
LMP2	FAT1	0.043709
LMP2	S100A11	0.082776
LMP2	CCDC137	0.029035
LMP2	NKD1	0.075861
LMP2	PPP2R3B	0.03256
LMP2	ACTN4	0.04424
LMP2	EHD1	0.022571
LMP2	S100A13	0.097643
LMP2	MELK	0.015873
LMP2	S100A1	0.047246
LMP2	UTRN	0.026458
LMP2	CALB1	0.083893
LMP2	NDEL1	0.016535
LMP2	MASP2	0.12538
LMP2	PARK7	0.020794
LMP2	ANXA2R	0.039108
LMP2	EFEMP1	0.11194
LMP2	SPARC	0.049328
LMP2	NOTCH4	0.017944
LMP2	PCDH17	0.090123
LMP2	KIAA1841	0.022164
LMP2	REPS1	0.084725
LMP2	LRCOL1	0.046847
LMP2	MPTX1	0.017326
LMP2	STIM1	0.037415
LMP2	S100B	0.042203
LMP2	TNNC1	0.10046
LMP2	CETN2	0.094482
LMP2	ANXA6	0.099533
LMP2	ACTN2	0.037233
LMP2	PRSS2	0.10908

LMP2	PITPNM3	0.084277
LMP2	VIL1	0.051423
LMP2	CETN1	0.11674
LMP2	C2CD4D	0.012761
LMP2	VLDLR	0.042966
LMP2	CDH1	0.0209
LMP2	TTN	0.019615
LMP2	EGF	0.031789
LMP2	LTBP4	0.086601
LMP2	PLSCR4	0.07492
LMP2	SNX9	0.045705
LMP2	RYR2	0.016089
LMP2	PRF1	0.030463
LMP2	IL6ST	0.050514
LMP2	MYO1E	0.018533
LMP2	S100A8	0.039473
LMP2	NID2	0.11632
LMP2	NELL1	0.050184
LMP2	ytzL	0.055841
LMP2	WDR96	0.015464
LMP2	DNER	0.055195
LMP2	PEF1	0.10653
LMP2	C10orf82	0.016002
LMP2	CHP1	0.046003
LMP2	FASN	0.040241
LMP2	GPR98	0.032063
LMP2	MICU1	0.079321
LMP2	CALU	0.087751
LMP2	PPP3CA	0.011237
LMP2	BEND2	0.010695
LMP2	LRP2	0.038354
LMP2	FAM188B	0.072846
LMP2	NUCB2	0.06619
LMP2	S100A6	0.067542
LMP2	LRP8	0.077459
LMP2	CT55	0.05754
LMP2	S100P	0.056227
LMP2	RAB11FIP3	0.019519
LMP2	EFCAB4B	0.071938
LMP2	DTNA	0.03772
LMP2	REPS2	0.1172
LMP2	PPEF2	0.04239
LMP2	EFEMP2	0.10629
LMP2	ATP2A1	0.028941
LMP2	CAGE1	0.016168
LMP2	PKD2L1	0.050675
LMP2	EHD3	0.03077
LMP2	THBS2	0.08762
LMP2	CALM1	0.055273
LMP2	S100A4	0.11429
LMP2	S100A12	0.053625
LMP2	RCN1	0.11661
LMP2	ACTN3	0.11101
LMP2	ATP5F1	0.01664
LMP2	FBN1	0.11186
LMP2	F10	0.046179
LMP2	CAPN1	0.023186
LMP2	CELSR3	0.049682
LMP2	SIRT4	0.020575
LMP2	TESC	0.068235
LMP2	C12orf57	0.025002
LMP2	RPH3A	0.03932
LMP2	SRI	0.046549
LMP2	LRP4	0.052069
LMP2	SLIT2	0.022627
LMP2	MATN2	0.093406
LMP2	CIB1	0.074814
LMP2	PPP2R3A	0.060295

LMP2	DLL1	0.090599
LMP2	ACTN1	0.048539
LMP2	PRSS3	0.12838
LMP2	MYL2	0.12645
LMP2	EDEM1	0.04147
LMP2	LRP1	0.019765
LMP2	SNX19	0.054165
LMP2	PDGFRB	0.013018
LMP2	DST	0.056846
LMP2	MYL6	0.10792
LMP2	COMP	0.078722
LMP2	ANXA7	0.03544
LMP2	CHP2	0.077866
LMP2	CDK5R1	0.027009
LMP2	NECAB2	0.11532
LMP2	CANX	0.04366
LMP2	ANXA11	0.045127
LMP2	PPP3CB	0.01661
LMP2	MATN3	0.12353
LMP2	FAM47C	0.03195
LMP2	RET	0.041209
LMP2	CDH23	0.023537
LMP2	RAB11FIP4	0.15475
LMP2	CNRIP1	0.012163
LMP2	C1orf64	0.035966
LMP2	AC3.5	0.013382
LMP2	CLIC4	0.034985
LMP2	FAM49B	0.029022
LMP2	BNIP2	0.035532
LMP2	JAG2	0.065541
LMP2	CADPS	0.12878
LMP2	CCAR1	0.106
LMP2	C1orf195	0.072065
LMP2	CRELD2	0.10607
LMP2	YBR056C-B	0.011114
LMP2	GSN	0.043449
LMP2	SLC11A2	0.015378
LMP2	LETM1	0.090745
LMP2	FAM231A	0.11632
LMP2	FAM188A	0.075448
LMP2	RCN3	0.1115
LMP2	PLSCR1	0.041593
LMP2	NKAPL	0.047868
LMP2	TLDC2	0.023998
LMP2	LTBP1	0.11354
LMP2	F2	0.025165
LMP2	SYT1	0.035647
LMP2	NSUN5P1	0.044887
LMP2	NCS1	0.10098
LMP2	FBLN5	0.1051
LMP2	DAG1	0.021328
LMP2	NECAB3	0.059033
LMP2	FAM92A1	0.010514
LMP2	NPC1L1	0.01712
LMP2	MEGF6	0.11026
LMP2	CALR	0.012827
LMP2	KCNIP2	0.029968
LMP2	HEATR5B	0.044299
LMP2	CD93	0.065576
LMP2	GRIN1	0.033191
LMP2	DHH	0.075093
LMP2	N4BP2L1	0.020293
LMP2	FAM231D	0.033749
LMP2	C11orf72	0.016915
LMP2	MYL6B	0.085211
LMP2	CDHR2	0.082866
LMP2	CDH5	0.051072
LMP2	IL6R	0.014423

47465	LMP2	SYT2	0.09181
47466	LMP2	TPD52	0.11595
47467	BGRF1/BDRF1	ubl4ab	0.029023
47468	BGRF1/BDRF1	PIK3CD	0.013998
47469	BGRF1/BDRF1	SPTA1	0.075792
47470	BGRF1/BDRF1	S100A7	0.12217
47471	BGRF1/BDRF1	HSPA5	0.066009
47472	BGRF1/BDRF1	LDLR	0.054896
47473	BGRF1/BDRF1	UPF3A	0.016724
47474	BGRF1/BDRF1	RABEP1	0.045647
47475	BGRF1/BDRF1	C12orf61	0.027326
47476	BGRF1/BDRF1	PLSCR3	0.16963
47477	BGRF1/BDRF1	ZC3HAV1L	0.012199
47478	BGRF1/BDRF1	RPUSD4	0.034166
47479	BGRF1/BDRF1	SNAPIN	0.015511
47480	BGRF1/BDRF1	YMR013W-A	0.017571
47481	BGRF1/BDRF1	ATP5B	0.0861
47482	BGRF1/BDRF1	NIN	0.14678
47483	BGRF1/BDRF1	KCNIP1	0.14126
47484	BGRF1/BDRF1	STIM2	0.16743
47485	BGRF1/BDRF1	WIPF1	0.017466
47486	BGRF1/BDRF1	SPTAN1	0.10393
47487	BGRF1/BDRF1	MASP1	0.22862
47488	BGRF1/BDRF1	SCUBE3	0.20999
47489	BGRF1/BDRF1	TH	0.010287
47490	BGRF1/BDRF1	PROC	0.19266
47491	BGRF1/BDRF1	PDCD6	0.22182
47492	BGRF1/BDRF1	S100A9	0.053608
47493	BGRF1/BDRF1	ANXA2	0.10248
47494	BGRF1/BDRF1	MMP20	0.062301
47495	BGRF1/BDRF1	MATN4	0.217
47496	BGRF1/BDRF1	NOTCH3	0.05712
47497	BGRF1/BDRF1	RNH1	0.18282
47498	BGRF1/BDRF1	CLIP1	0.083632
47499	BGRF1/BDRF1	AMOT	0.035344
47500	BGRF1/BDRF1	EIF4G2	0.0176
47501	BGRF1/BDRF1	FAM83F	0.01853
47502	BGRF1/BDRF1	THBD	0.12652
47503	BGRF1/BDRF1	RNMT	0.038764
47504	BGRF1/BDRF1	MAGOH	0.024825
47505	BGRF1/BDRF1	EML1	0.20405
47506	BGRF1/BDRF1	EPS15	0.080698
47507	BGRF1/BDRF1	PCSK2	0.058095
47508	BGRF1/BDRF1	PRMT8	0.021367
47509	BGRF1/BDRF1	AP3S1	0.082498
47510	BGRF1/BDRF1	SDF4	0.15125
47511	BGRF1/BDRF1	SPACA3	0.022259
47512	BGRF1/BDRF1	STAT1	0.02384
47513	BGRF1/BDRF1	FAT4	0.1971
47514	BGRF1/BDRF1	DTNBP1	0.019021
47515	BGRF1/BDRF1	PPEF1	0.10635
47516	BGRF1/BDRF1	CXorf67	0.040172
47517	BGRF1/BDRF1	NELL2	0.052756
47518	BGRF1/BDRF1	FAM188B2	0.035399
47519	BGRF1/BDRF1	KAT2A	0.015277
47520	BGRF1/BDRF1	MAP1LC3C	0.017385
47521	BGRF1/BDRF1	THBS1	0.020573
47522	BGRF1/BDRF1	BICD1	0.015135
47523	BGRF1/BDRF1	PRMT1	0.034328
47524	BGRF1/BDRF1	AP1M1	0.035359
47525	BGRF1/BDRF1	TPPP	0.14195
47526	BGRF1/BDRF1	SYT11	0.25158
47527	BGRF1/BDRF1	HSP90B1	0.10798
47528	BGRF1/BDRF1	MACF1	0.13186
47529	BGRF1/BDRF1	CCDC176	0.042326
47530	BGRF1/BDRF1	GCH1	0.04949
47531	BGRF1/BDRF1	NKD2	0.20633
47532	BGRF1/BDRF1	ATP2B2	0.016651

BGRF1/BDRF1	FNBP1L	0.025903
BGRF1/BDRF1	FAT1	0.10555
BGRF1/BDRF1	S100A11	0.16311
BGRF1/BDRF1	CCDC137	0.032234
BGRF1/BDRF1	NKD1	0.15502
BGRF1/BDRF1	PPP2R3B	0.1005
BGRF1/BDRF1	ACTN4	0.087697
BGRF1/BDRF1	EHD1	0.066332
BGRF1/BDRF1	S100A13	0.16262
BGRF1/BDRF1	MELK	0.057554
BGRF1/BDRF1	S100A1	0.11401
BGRF1/BDRF1	ARL14EPL	0.011654
BGRF1/BDRF1	UTRN	0.079869
BGRF1/BDRF1	SLC34A1	0.020728
BGRF1/BDRF1	AQP1	0.011593
BGRF1/BDRF1	STAT6	0.020932
BGRF1/BDRF1	CALB1	0.14947
BGRF1/BDRF1	MASP2	0.21236
BGRF1/BDRF1	PARK7	0.049399
BGRF1/BDRF1	GAS6	0.013933
BGRF1/BDRF1	ANXA2R	0.02844
BGRF1/BDRF1	EFEMP1	0.19708
BGRF1/BDRF1	SPARC	0.089609
BGRF1/BDRF1	NOTCH4	0.051691
BGRF1/BDRF1	PCDH17	0.1971
BGRF1/BDRF1	PRDX4	0.012149
BGRF1/BDRF1	EIF3B	0.011175
BGRF1/BDRF1	YJR030C	0.011533
BGRF1/BDRF1	KIAA1841	0.024899
BGRF1/BDRF1	REPS1	0.18428
BGRF1/BDRF1	LRCOL1	0.027418
BGRF1/BDRF1	NOTCH2	0.021301
BGRF1/BDRF1	MPTX1	0.010767
BGRF1/BDRF1	STIM1	0.092481
BGRF1/BDRF1	S100B	0.091881
BGRF1/BDRF1	TNNC1	0.18072
BGRF1/BDRF1	TMED2	0.016177
BGRF1/BDRF1	CETN2	0.15302
BGRF1/BDRF1	ANXA6	0.1884
BGRF1/BDRF1	ACTN2	0.067389
BGRF1/BDRF1	PRSS2	0.18526
BGRF1/BDRF1	STAT2	0.022084
BGRF1/BDRF1	PITPNM3	0.1791
BGRF1/BDRF1	VIL1	0.090629
BGRF1/BDRF1	KCNJ4	0.054267
BGRF1/BDRF1	CETN1	0.19507
BGRF1/BDRF1	VLDLR	0.079121
BGRF1/BDRF1	CDH1	0.038438
BGRF1/BDRF1	ADAMTS13	0.013521
BGRF1/BDRF1	MAP1LC3B	0.048821
BGRF1/BDRF1	TTN	0.034701
BGRF1/BDRF1	ZDHHC17	0.0146
BGRF1/BDRF1	EGF	0.065124
BGRF1/BDRF1	LTBP4	0.15519
BGRF1/BDRF1	PRMT2	0.020339
BGRF1/BDRF1	PLSCR4	0.16518
BGRF1/BDRF1	RBM8A	0.023648
BGRF1/BDRF1	RHOT1	0.014006
BGRF1/BDRF1	SNX9	0.096063
BGRF1/BDRF1	RYR2	0.038163
BGRF1/BDRF1	PRF1	0.0865
BGRF1/BDRF1	IL6ST	0.088579
BGRF1/BDRF1	MYO1E	0.049744
BGRF1/BDRF1	SETD2	0.027993
BGRF1/BDRF1	SNCA	0.010627
BGRF1/BDRF1	S100A8	0.076514
BGRF1/BDRF1	NID2	0.21192
BGRF1/BDRF1	NELL1	0.11138

BGRF1/BDRF1	ytzL	0.038637
BGRF1/BDRF1	WDR96	0.013656
BGRF1/BDRF1	DNER	0.12559
BGRF1/BDRF1	PEF1	0.21193
BGRF1/BDRF1	MEGF8	0.054174
BGRF1/BDRF1	C10orf82	0.016475
BGRF1/BDRF1	CHP1	0.081466
BGRF1/BDRF1	CFTR	0.021037
BGRF1/BDRF1	FASN	0.070561
BGRF1/BDRF1	GPR98	0.078931
BGRF1/BDRF1	MICU1	0.1698
BGRF1/BDRF1	CALU	0.16759
BGRF1/BDRF1	PPP3CA	0.037976
BGRF1/BDRF1	LRP2	0.077008
BGRF1/BDRF1	CAPN2	0.030747
BGRF1/BDRF1	SLC23A1	0.034007
BGRF1/BDRF1	FAM188B	0.046407
BGRF1/BDRF1	NUCB2	0.13861
BGRF1/BDRF1	S100A6	0.13317
BGRF1/BDRF1	LRP8	0.14532
BGRF1/BDRF1	CT55	0.024888
BGRF1/BDRF1	S100P	0.13692
BGRF1/BDRF1	PRDX6	0.035326
BGRF1/BDRF1	CHAF1A	0.016153
BGRF1/BDRF1	SETD1B	0.028999
BGRF1/BDRF1	RAB11FIP3	0.063943
BGRF1/BDRF1	EFCAB4B	0.14922
BGRF1/BDRF1	ALKBH8	0.019527
BGRF1/BDRF1	DTNA	0.10457
BGRF1/BDRF1	REPS2	0.22152
BGRF1/BDRF1	PPEF2	0.093067
BGRF1/BDRF1	EFEMP2	0.20954
BGRF1/BDRF1	ATP2A1	0.060573
BGRF1/BDRF1	WLS	0.038856
BGRF1/BDRF1	PKD2L1	0.10936
BGRF1/BDRF1	EHD3	0.083784
BGRF1/BDRF1	THBS2	0.17507
BGRF1/BDRF1	CALM1	0.092587
BGRF1/BDRF1	EIF4E2	0.032489
BGRF1/BDRF1	S100A4	0.20694
BGRF1/BDRF1	S100A12	0.10798
BGRF1/BDRF1	RCN1	0.21835
BGRF1/BDRF1	DPP4	0.029755
BGRF1/BDRF1	EIF4E	0.019003
BGRF1/BDRF1	DNMT3A	0.01332
BGRF1/BDRF1	ACTN3	0.1976
BGRF1/BDRF1	ATP5F1	0.037775
BGRF1/BDRF1	FBN1	0.19871
BGRF1/BDRF1	F10	0.098939
BGRF1/BDRF1	CAPN1	0.083001
BGRF1/BDRF1	UCKL1	0.01814
BGRF1/BDRF1	CELSR3	0.12007
BGRF1/BDRF1	N6AMT1	0.043711
BGRF1/BDRF1	SETD8	0.012203
BGRF1/BDRF1	SIRT4	0.083094
BGRF1/BDRF1	TESC	0.12383
BGRF1/BDRF1	MICAL1	0.024301
BGRF1/BDRF1	LSM12	0.01725
BGRF1/BDRF1	C12orf57	0.016862
BGRF1/BDRF1	SETD1A	0.027653
BGRF1/BDRF1	RPH3A	0.092574
BGRF1/BDRF1	SRI	0.10462
BGRF1/BDRF1	LRP4	0.11024
BGRF1/BDRF1	SLIT2	0.039869
BGRF1/BDRF1	MATN2	0.17329
BGRF1/BDRF1	CIB1	0.15039
BGRF1/BDRF1	PPP2R3A	0.13248
BGRF1/BDRF1	DLL1	0.15917

BGRF1/BDRF1	ACTN1	0.095972
BGRF1/BDRF1	EHD4	0.052124
BGRF1/BDRF1	UPF3B	0.030718
BGRF1/BDRF1	MYO6	0.013925
BGRF1/BDRF1	PRSS3	0.2162
BGRF1/BDRF1	MYL2	0.20644
BGRF1/BDRF1	EDEM1	0.10812
BGRF1/BDRF1	LRP1	0.04996
BGRF1/BDRF1	ADAR	0.016089
BGRF1/BDRF1	SNX19	0.136
BGRF1/BDRF1	PDGFRB	0.024441
BGRF1/BDRF1	ITSN1	0.042334
BGRF1/BDRF1	DST	0.1023
BGRF1/BDRF1	MYL6	0.19543
BGRF1/BDRF1	COMP	0.14954
BGRF1/BDRF1	CCDC47	0.17088
BGRF1/BDRF1	ANXA7	0.095545
BGRF1/BDRF1	CHP2	0.15944
BGRF1/BDRF1	NECAB2	0.22375
BGRF1/BDRF1	CANX	0.093973
BGRF1/BDRF1	RHOT2	0.014325
BGRF1/BDRF1	MMP15	0.04596
BGRF1/BDRF1	ANXA11	0.099243
BGRF1/BDRF1	PPP3CB	0.042814
BGRF1/BDRF1	IQGAP1	0.029449
BGRF1/BDRF1	MATN3	0.22742
BGRF1/BDRF1	FAM47C	0.019611
BGRF1/BDRF1	SH3KBP1	0.040629
BGRF1/BDRF1	RET	0.078408
BGRF1/BDRF1	CDH23	0.06761
BGRF1/BDRF1	RAB11FIP4	0.27465
BGRF1/BDRF1	CNGC19	0.011582
BGRF1/BDRF1	SUV39H2	0.030441
BGRF1/BDRF1	C1orf64	0.025418
BGRF1/BDRF1	PLCE1	0.020028
BGRF1/BDRF1	AC3.5	0.016223
BGRF1/BDRF1	C2orf81	0.016759
BGRF1/BDRF1	CLIC4	0.07394
BGRF1/BDRF1	EIF4G1	0.020523
BGRF1/BDRF1	FAM49B	0.014573
BGRF1/BDRF1	BNIP2	0.093906
BGRF1/BDRF1	JAG2	0.12984
BGRF1/BDRF1	EHD2	0.028074
BGRF1/BDRF1	CADPS	0.22237
BGRF1/BDRF1	CCAR1	0.18945
BGRF1/BDRF1	C1orf195	0.048839
BGRF1/BDRF1	CRELD2	0.205
BGRF1/BDRF1	YBR056C-B	0.012752
BGRF1/BDRF1	NBR1	0.021214
BGRF1/BDRF1	GSN	0.084741
BGRF1/BDRF1	SLC11A2	0.026567
BGRF1/BDRF1	EIF4A1	0.01903
BGRF1/BDRF1	CABP1	0.043976
BGRF1/BDRF1	LETM1	0.19801
BGRF1/BDRF1	PIK3CB	0.011407
BGRF1/BDRF1	FAM231A	0.073975
BGRF1/BDRF1	FAM124A	0.025119
BGRF1/BDRF1	FAM188A	0.16568
BGRF1/BDRF1	AP1B1	0.047791
BGRF1/BDRF1	RCN3	0.21629
BGRF1/BDRF1	PLSCR1	0.089822
BGRF1/BDRF1	SLC6A4	0.022652
BGRF1/BDRF1	NKAPL	0.039411
BGRF1/BDRF1	TLDC2	0.021005
BGRF1/BDRF1	LTBP1	0.21036
BGRF1/BDRF1	F2	0.051072
BGRF1/BDRF1	SYT1	0.074418
BGRF1/BDRF1	NSUN5P1	0.023154

BGRF1/BDRF1	PI4K2A	0.043706
BGRF1/BDRF1	NCS1	0.17899
BGRF1/BDRF1	TCAP	0.011826
BGRF1/BDRF1	FBLN5	0.18507
BGRF1/BDRF1	DAG1	0.047995
BGRF1/BDRF1	DOC2A	0.012136
BGRF1/BDRF1	C5orf48	0.013477
BGRF1/BDRF1	NECAB3	0.13449
BGRF1/BDRF1	MLC1	0.025354
BGRF1/BDRF1	SNX6	0.055287
BGRF1/BDRF1	NPC1L1	0.068592
BGRF1/BDRF1	MEGF6	0.2126
BGRF1/BDRF1	CALR	0.022521
BGRF1/BDRF1	KCNIP2	0.07734
BGRF1/BDRF1	PLCG1	0.011371
BGRF1/BDRF1	HEATR5B	0.028626
BGRF1/BDRF1	CD93	0.15218
BGRF1/BDRF1	GRIN1	0.067097
BGRF1/BDRF1	CLINT1	0.0295
BGRF1/BDRF1	DHH	0.1452
BGRF1/BDRF1	SPHK2	0.040069
BGRF1/BDRF1	N4BP2L1	0.010534
BGRF1/BDRF1	AP1G2	0.010237
BGRF1/BDRF1	FAM231D	0.029729
BGRF1/BDRF1	C11orf72	0.011796
BGRF1/BDRF1	MYL6B	0.1681
BGRF1/BDRF1	ATP6V0A1	0.038615
BGRF1/BDRF1	AP1G1	0.04159
BGRF1/BDRF1	CDHR2	0.18049
BGRF1/BDRF1	SNX17	0.011257
BGRF1/BDRF1	CDH5	0.11766
BGRF1/BDRF1	IL6R	0.036948
BGRF1/BDRF1	SYT2	0.17837
BGRF1/BDRF1	CAPNS1	0.063221
BGRF1/BDRF1	TPD52	0.20947
BBRF2	KCT2	0.019331
BBRF2	EPC2	0.014315
BBRF2	RRP7A	0.036115
BBRF2	HERPUD1	0.041567
BBRF2	ubl4ab	0.10086
BBRF2	PIBF1	0.051989
BBRF2	ATPIF1	0.058508
BBRF2	BIN2	0.011895
BBRF2	AKAP14	0.10178
BBRF2	PANK2	0.066558
BBRF2	PAGE2B	0.0619
BBRF2	GDNF	0.010074
BBRF2	SLC16A12	0.023426
BBRF2	LTV1	0.032875
BBRF2	NRIP2	0.01713
BBRF2	S100A7	0.010647
BBRF2	NANOS1	0.019104
BBRF2	TVP23A	0.013324
BBRF2	MICALCL	0.033026
BBRF2	PTTG2	0.042298
BBRF2	CHMP6	0.016427
BBRF2	SERTAD3	0.040156
BBRF2	PPP1R15B	0.036236
BBRF2	INO80B	0.040628
BBRF2	SHISA4	0.032098
BBRF2	DDI1	0.013012
BBRF2	UPF3A	0.083138
BBRF2	JSRP1	0.044763
BBRF2	NPAP1	0.033738
BBRF2	RABEP1	0.089126
BBRF2	SKA3	0.023922
BBRF2	CACNA2D1	0.013787
BBRF2	MON1A	0.017685

BBRF2	SPATS2L	0.034923
BBRF2	TMPRSS11BNL	0.028763
BBRF2	SLC5A1	0.019034
BBRF2	NUP85	0.011393
BBRF2	C12orf61	0.099032
BBRF2	IP6K2	0.030404
BBRF2	MAP7D3	0.015415
BBRF2	MT1A	0.011424
BBRF2	PLSCR3	0.059776
BBRF2	TCF25	0.027293
BBRF2	CC2D1B	0.013127
BBRF2	N6AMT2	0.012113
BBRF2	PRSS16	0.098066
BBRF2	OR52N4	0.032276
BBRF2	ASXL3	0.0174
BBRF2	FAM150A	0.033586
BBRF2	NRN1	0.017268
BBRF2	DYM	0.021045
BBRF2	ZC3HAV1L	0.068016
BBRF2	TCP10	0.011082
BBRF2	TIMD4	0.010808
BBRF2	STOML3	0.02009
BBRF2	ATP6V0D2	0.02163
BBRF2	CLC	0.014682
BBRF2	XKR7	0.06099
BBRF2	PRRT2	0.013948
BBRF2	OR2T2	0.033165
BBRF2	RPUSD4	0.032198
BBRF2	CMPK2	0.062791
BBRF2	CCDC159	0.038098
BBRF2	C10orf68	0.0278
BBRF2	CEP85L	0.041176
BBRF2	TNIP3	0.010431
BBRF2	NAT16	0.089253
BBRF2	ANAPC5	0.040268
BBRF2	TSPAN32	0.017195
BBRF2	WDPCP	0.028315
BBRF2	HMGCR	0.010741
BBRF2	MCM3AP	0.013124
BBRF2	CARD6	0.014615
BBRF2	FXYD4	0.041675
BBRF2	TMEM119	0.017716
BBRF2	TMEM132C	0.037743
BBRF2	TEX12	0.033524
BBRF2	KRI1	0.034319
BBRF2	SPATA1	0.010645
BBRF2	MKS1	0.016762
BBRF2	IGFL2	0.093099
BBRF2	BLVRA	0.072315
BBRF2	CCDC109B	0.031245
BBRF2	SPHKAP	0.020043
BBRF2	STMN4	0.010474
BBRF2	IRAK1BP1	0.081421
BBRF2	ZPBP2	0.046595
BBRF2	THRAP3	0.019424
BBRF2	VWA3A	0.038207
BBRF2	SLC31A2	0.01983
BBRF2	ZDHHC11	0.012988
BBRF2	ARSB	0.012391
BBRF2	TAS2R13	0.026913
BBRF2	ARV1	0.010846
BBRF2	WASH3P	0.011082
BBRF2	AKAP11	0.022202
BBRF2	UNC119B	0.040686
BBRF2	YMR013W-A	0.072526
BBRF2	SLC39A4	0.017597
BBRF2	GFRAL	0.010023
BBRF2	ATG4D	0.011543

BBRF2	BTBD10	0.013798
BBRF2	CASC3	0.065311
BBRF2	CHIC1	0.029575
BBRF2	NKAIN3	0.038317
BBRF2	IER3IP1	0.016853
BBRF2	ATP6V1B2	0.010799
BBRF2	HBG2	0.044846
BBRF2	CCM2L	0.018843
BBRF2	MAN1B1	0.043765
BBRF2	TRMT6	0.06442
BBRF2	VWA7	0.074468
BBRF2	SPATC1	0.0409
BBRF2	ZC3H12C	0.010236
BBRF2	ATP5B	0.020761
BBRF2	NIN	0.11778
BBRF2	NUP93	0.021355
BBRF2	FGFBP3	0.034112
BBRF2	MTO1	0.083615
BBRF2	TNRC6C	0.027734
BBRF2	NACA2	0.01824
BBRF2	ILDR2	0.069982
BBRF2	PGM3	0.058749
BBRF2	B9D1	0.033086
BBRF2	STIM2	0.01173
BBRF2	KIN	0.057363
BBRF2	HUS1B	0.0379
BBRF2	WBP2	0.031764
BBRF2	EID2B	0.03006
BBRF2	TMEM2	0.017265
BBRF2	PLBD2	0.035882
BBRF2	TIGD2	0.025308
BBRF2	CABYR	0.067515
BBRF2	SLAIN2	0.028918
BBRF2	TAS2R50	0.013747
BBRF2	SSSCA1	0.010743
BBRF2	CCDC130	0.026673
BBRF2	BNIPL	0.011898
BBRF2	NUP54	0.032268
BBRF2	MCMDC2	0.018815
BBRF2	RPLP1	0.021439
BBRF2	DERL3	0.014805
BBRF2	LY6G6F	0.045985
BBRF2	MAN1A2	0.026075
BBRF2	SLC23A2	0.046918
BBRF2	ZBTB47	0.01261
BBRF2	SSX6	0.019425
BBRF2	NDUFAF4	0.02157
BBRF2	OTOA	0.013932
BBRF2	DPY30	0.022054
BBRF2	OR2Z1	0.034512
BBRF2	ANGEL1	0.031901
BBRF2	TMEM30B	0.022972
BBRF2	PITPNM2	0.021861
BBRF2	C19orf44	0.022902
BBRF2	MIA3	0.010347
BBRF2	OR4K14	0.034222
BBRF2	NKAIN1	0.053525
BBRF2	SLAMF7	0.02861
BBRF2	FSIP1	0.046215
BBRF2	SYPL2	0.030076
BBRF2	COMMD5	0.050607
BBRF2	LGALS7	0.010568
BBRF2	WIPF1	0.048708
BBRF2	AANAT	0.033938
BBRF2	TSGA10IP	0.02765
BBRF2	CD81	0.01357
BBRF2	ST8SIA3	0.01623
BBRF2	TNFAIP8L3	0.026244

BBRF2	SLC30A5	0.011814
BBRF2	MT-ND4	0.038194
BBRF2	GBGT1	0.031803
BBRF2	NBPF15	0.039537
BBRF2	ATRIP	0.070196
BBRF2	NDUFAB1	0.025586
BBRF2	CCDC136	0.013657
BBRF2	PREP	0.040033
BBRF2	LAMTOR1	0.024174
BBRF2	DHPS	0.031871
BBRF2	ARMCX4	0.064168
BBRF2	THAP1	0.010072
BBRF2	CLP1	0.011355
BBRF2	POP4	0.043494
BBRF2	C10orf35	0.056715
BBRF2	MCAT	0.015462
BBRF2	APOL2	0.031165
BBRF2	TM2D3	0.093431
BBRF2	MFAP2	0.09115
BBRF2	BCL9	0.017616
BBRF2	TSNAX	0.026044
BBRF2	TADA3	0.016564
BBRF2	ITPKC	0.068117
BBRF2	LSM3	0.015935
BBRF2	PEA15	0.013299
BBRF2	UXT	0.018546
BBRF2	TRAPPC4	0.026861
BBRF2	TAS2R43	0.025657
BBRF2	POLR3D	0.014922
BBRF2	FBXO7	0.02198
BBRF2	WDR93	0.065413
BBRF2	SPBC1539.02	0.023956
BBRF2	LYZL2	0.072652
BBRF2	XPO7	0.034168
BBRF2	HEXIM1	0.044626
BBRF2	ATXN10	0.034683
BBRF2	TRAPPC6B	0.028924
BBRF2	COMMD7	0.035804
BBRF2	DUOXA1	0.028834
BBRF2	ATG4B	0.028104
BBRF2	MAL2	0.035954
BBRF2	ZFAND3	0.013359
BBRF2	ITIH5	0.011393
BBRF2	MEI4	0.060744
BBRF2	FAM187B	0.010449
BBRF2	ATP6V1B1	0.010562
BBRF2	GLTSCR1L	0.05584
BBRF2	MED8	0.012584
BBRF2	FAM21D	0.038061
BBRF2	TSR2	0.010927
BBRF2	PEX19	0.017488
BBRF2	TMEM135	0.043803
BBRF2	SLC34A2	0.08112
BBRF2	SNRPG	0.010889
BBRF2	MUC20	0.034942
BBRF2	TECPR1	0.07565
BBRF2	GTPBP1	0.1038
BBRF2	SERINC3	0.028867
BBRF2	MALL	0.031295
BBRF2	CHMP4B	0.074287
BBRF2	SELV	0.049446
BBRF2	DKC1	0.025249
BBRF2	CHMP4BP1	0.033219
BBRF2	RPS4Y1	0.021623
BBRF2	BTG4	0.010463
BBRF2	EXOC1	0.036809
BBRF2	TMPO	0.011589
BBRF2	PSMD2	0.015126

BBRF2	ATP6V1G2	0.028493
BBRF2	NRIP3	0.026212
BBRF2	TMEM132A	0.012659
BBRF2	PGAM1	0.048159
BBRF2	DMRTB1	0.012772
BBRF2	ZSCAN5C	0.01458
BBRF2	CMTM3	0.040103
BBRF2	C21orf67	0.05765
BBRF2	STOML2	0.010638
BBRF2	UBAP2	0.065801
BBRF2	GFRA2	0.051326
BBRF2	TNNT1	0.022131
BBRF2	XKR4	0.06099
BBRF2	ANO8	0.04224
BBRF2	SATL1	0.021666
BBRF2	FAM110D	0.021319
BBRF2	PRAMEF1	0.072886
BBRF2	VWA8	0.013711
BBRF2	TSPAN19	0.010038
BBRF2	MS4A4E	0.025963
BBRF2	LEPROT	0.013932
BBRF2	TEKT3	0.015574
BBRF2	LMF1	0.03399
BBRF2	PPP6R1	0.016876
BBRF2	SLC13A4	0.020227
BBRF2	TBC1D3H	0.01421
BBRF2	SELPLG	0.05136
BBRF2	YTHDF3	0.050648
BBRF2	LVP1a	0.013209
BBRF2	PLXDC2	0.014957
BBRF2	BLOC1S3	0.034013
BBRF2	AMOT	0.024722
BBRF2	DNAAF2	0.092024
BBRF2	CCDC154	0.014886
BBRF2	MAATS1	0.028492
BBRF2	SLC4A1	0.020227
BBRF2	CARNS1	0.011024
BBRF2	PAGE5	0.047665
BBRF2	TOB1	0.018771
BBRF2	CLPTM1L	0.044198
BBRF2	PLAC1	0.03097
BBRF2	MAN1C1	0.020274
BBRF2	UGCG	0.042096
BBRF2	CABS1	0.14389
BBRF2	NAPB	0.024135
BBRF2	HHIPL2	0.014659
BBRF2	TRAK2	0.013479
BBRF2	GAGE10	0.029042
BBRF2	SLC16A13	0.037699
BBRF2	CASP8AP2	0.035389
BBRF2	FNTB	0.017311
BBRF2	LINC00474	0.013669
BBRF2	MORC1	0.016598
BBRF2	CRTC1	0.029691
BBRF2	VEZT	0.098685
BBRF2	CLPX	0.017983
BBRF2	FAM117B	0.0619
BBRF2	GLS	0.01054
BBRF2	HAUS6	0.040071
BBRF2	OR2M3	0.030984
BBRF2	HELB	0.012179
BBRF2	AKIRIN2	0.080736
BBRF2	KCNK1	0.015124
BBRF2	RPRD1A	0.019507
BBRF2	LVP1a	0.10943
BBRF2	ZNF569	0.012444
BBRF2	PHYHIP	0.014026
BBRF2	METTL1	0.047175

BBRF2	MRFAP1	0.0392
BBRF2	CDC42EP4	0.012277
BBRF2	NCBP1	0.010009
BBRF2	EDC3	0.011279
BBRF2	WFDC3	0.01282
BBRF2	SAMD4B	0.012706
BBRF2	LYZL4	0.026746
BBRF2	GDI2	0.01003
BBRF2	GADD45B	0.044287
BBRF2	TMED6	0.013655
BBRF2	MESDC1	0.021413
BBRF2	ARSD	0.01188
BBRF2	C10orf91	0.042759
BBRF2	PENK	0.014884
BBRF2	KCNJ14	0.011506
BBRF2	DMRTA1	0.010666
BBRF2	IGHV1OR21-1	0.019262
BBRF2	LGALS4	0.013124
BBRF2	NCOA7	0.024525
BBRF2	GK3P	0.039527
BBRF2	CCDC28A	0.058752
BBRF2	MEMO1	0.010787
BBRF2	ZC3H18	0.058585
BBRF2	RPF2	0.039268
BBRF2	MRPL46	0.042754
BBRF2	CEP170P1	0.010386
BBRF2	P4HA1	0.029995
BBRF2	SSBP2	0.033011
BBRF2	GNPTG	0.014436
BBRF2	TIGAR	0.042765
BBRF2	LMAN2L	0.030138
BBRF2	XRCC6	0.014639
BBRF2	SPEF1	0.041455
BBRF2	SF3B5	0.049231
BBRF2	RCAN3	0.012084
BBRF2	ICA1L	0.024615
BBRF2	NECAP1	0.073227
BBRF2	OAZ2	0.034248
BBRF2	CNNM2	0.016239
BBRF2	MAGEA9	0.013015
BBRF2	MIR17HG	0.051551
BBRF2	SECTM1	0.10883
BBRF2	SERTAD2	0.032467
BBRF2	NME7	0.023585
BBRF2	HBE1	0.044846
BBRF2	NREP	0.011508
BBRF2	HORMAD1	0.034374
BBRF2	RTFDC1	0.015951
BBRF2	GID4	0.036033
BBRF2	CMTM5	0.050672
BBRF2	PPP1R10	0.054479
BBRF2	EVA1B	0.053461
BBRF2	FAM83F	0.072001
BBRF2	LUZP4	0.022117
BBRF2	NCR3	0.036831
BBRF2	FAM101A	0.017702
BBRF2	CKB	0.026062
BBRF2	NCCRP1	0.035588
BBRF2	OR5F1	0.040464
BBRF2	OR2T5	0.032661
BBRF2	SPATC1L	0.057823
BBRF2	ANO4	0.021152
BBRF2	RTP4	0.010351
BBRF2	ATG13	0.054404
BBRF2	ANO5	0.014408
BBRF2	CNP	0.010202
BBRF2	IFNA6	0.012114
BBRF2	CLSPN	0.034576

BBRF2	UPK3BL	0.048033
BBRF2	GOLGA8F	0.016357
BBRF2	RTP1	0.011807
BBRF2	ASTL	0.019816
BBRF2	OR51S1	0.033745
BBRF2	MEN1	0.017637
BBRF2	BEX2	0.091856
BBRF2	SEC23A	0.014496
BBRF2	BCKDHB	0.019747
BBRF2	CTAGE6	0.011431
BBRF2	TBATA	0.033518
BBRF2	TAS2R4	0.026206
BBRF2	FXYD1	0.038515
BBRF2	DDX47	0.012552
BBRF2	LMAN1L	0.010671
BBRF2	TMEM92	0.019993
BBRF2	OR2T35	0.038211
BBRF2	SRGN	0.024565
BBRF2	RPUSD3	0.011929
BBRF2	TNFAIP1	0.01162
BBRF2	NUDT15	0.010051
BBRF2	AKAP4	0.040615
BBRF2	BRK1	0.038088
BBRF2	C19orf68	0.027467
BBRF2	ACRBP	0.12223
BBRF2	NOL3	0.01458
BBRF2	MAGED1	0.010836
BBRF2	EPPK1	0.012802
BBRF2	IP6K1	0.04065
BBRF2	HAT1	0.02862
BBRF2	FAM166A	0.034695
BBRF2	ZNF649	0.010214
BBRF2	NGFRAP1	0.010325
BBRF2	TLDC1	0.037805
BBRF2	COMMD3	0.05018
BBRF2	ADAD2	0.01442
BBRF2	CDK5R2	0.028411
BBRF2	TAS2R46	0.023843
BBRF2	PRRC2B	0.040269
BBRF2	XKRY	0.069714
BBRF2	BYSL	0.018066
BBRF2	SFSWAP	0.0384
BBRF2	MUM1L1	0.034056
BBRF2	SPPL2A	0.034482
BBRF2	CACYBP	0.015306
BBRF2	FAM74A4	0.015194
BBRF2	COBLL1	0.026957
BBRF2	UBLCP1	0.026373
BBRF2	RPP14	0.028873
BBRF2	WHAMM	0.020354
BBRF2	LZTS1	0.034259
BBRF2	OR2B11	0.033572
BBRF2	TCTN2	0.022183
BBRF2	PEX11G	0.049902
BBRF2	IFNA5	0.015847
BBRF2	LTC4S	0.013421
BBRF2	AP3S1	0.016936
BBRF2	LYZL6	0.07588
BBRF2	AFF4	0.011384
BBRF2	DNASE1L1	0.041202
BBRF2	MGP	0.025786
BBRF2	FBXL22	0.03064
BBRF2	NACAP1	0.013535
BBRF2	TAF11	0.02224
BBRF2	LAMTOR2	0.018272
BBRF2	DDHD1	0.02488
BBRF2	YIPF5	0.068299
BBRF2	EBAG9	0.024399

48213	BBRF2	NSUN4	0.060298
48214	BBRF2	BEAN1	0.038271
48215	BBRF2	EID3	0.078171
48216	BBRF2	SPACA3	0.06209
48217	BBRF2	HHAT	0.06435
48218	BBRF2	GLTP	0.015538
48219	BBRF2	GPATCH1	0.057398
48220	BBRF2	FAM9A	0.026881
48221	BBRF2	RRN3	0.014636
48222	BBRF2	DTNBP1	0.025724
48223	BBRF2	TAS2R60	0.029197
48224	BBRF2	PLIN3	0.011073
48225	BBRF2	CXorf67	0.12854
48226	BBRF2	FCER1G	0.023985
48227	BBRF2	ISY1	0.037124
48228	BBRF2	CLTB	0.024315
48229	BBRF2	OR2T29	0.032661
48230	BBRF2	NAGS	0.024384
48231	BBRF2	SEC11A	0.058541
48232	BBRF2	ZNF777	0.010498
48233	BBRF2	ELOVL4	0.049947
48234	BBRF2	FAM188B2	0.14254
48235	BBRF2	OR2I1P	0.033572
48236	BBRF2	GALNT4	0.013592
48237	BBRF2	GTF3C1	0.033944
48238	BBRF2	WTAP	0.024773
48239	BBRF2	EIF1AX	0.057939
48240	BBRF2	ytzL	0.011875
48241	BBRF2	DMAP1	0.026662
48242	BBRF2	DPP3	0.038363
48243	BBRF2	GG1	0.03104
48244	BBRF2	FXYD6P3	0.021276
48245	BBRF2	CTAG2	0.045108
48246	BBRF2	ZSWIM3	0.028978
48247	BBRF2	AKIP1	0.024926
48248	BBRF2	POLR2D	0.022192
48249	BBRF2	SF3B2	0.028855
48250	BBRF2	MEI1	0.032932
48251	BBRF2	SCGB1D1	0.048066
48252	BBRF2	OR4N2	0.033572
48253	BBRF2	TNNI1	0.015989
48254	BBRF2	NOP58	0.016913
48255	BBRF2	ATP6V1E1	0.011788
48256	BBRF2	ZNF444	0.015408
48257	BBRF2	FLVCR2	0.010663
48258	BBRF2	NUDCD3	0.038886
48259	BBRF2	MAGEF1	0.013015
48260	BBRF2	AMY2B	0.024747
48261	BBRF2	GSTCD	0.014597
48262	BBRF2	CLUH	0.018689
48263	BBRF2	UNC93A	0.042457
48264	BBRF2	FAM69C	0.15973
48265	BBRF2	G4	0.047365
48266	BBRF2	TBC1D3F	0.01312
48267	BBRF2	XKRY2	0.062496
48268	BBRF2	MS4A2	0.011624
48269	BBRF2	POC5	0.011159
48270	BBRF2	ARL2BP	0.014511
48271	BBRF2	PFN1	0.011749
48272	BBRF2	POMT1	0.015301
48273	BBRF2	FITM1	0.035008
48274	BBRF2	DEDD2	0.024874
48275	BBRF2	CST9L	0.010693
48276	BBRF2	TAS2R38	0.026913
48277	BBRF2	OR2M5	0.030984
48278	BBRF2	METTL2B	0.011223
48279	BBRF2	FAM184B	0.014149
48280	BBRF2	SEC61A1	0.033845

BBRF2	SLC4A8	0.014165
BBRF2	LPCAT3	0.03921
BBRF2	POLDIP2	0.038228
BBRF2	CCSER1	0.050871
BBRF2	CDC42EP5	0.012277
BBRF2	CLTA	0.055479
BBRF2	CCDC144A	0.010458
BBRF2	TMEM183B	0.058755
BBRF2	MED26	0.015022
BBRF2	CRP	0.020466
BBRF2	PANX1	0.016742
BBRF2	CENPC	0.018579
BBRF2	PNMAL2	0.024843
BBRF2	DDOST	0.023744
BBRF2	AGR3	0.021526
BBRF2	ECM29	0.016767
BBRF2	NECAP2	0.0468
BBRF2	TXLNG	0.012343
BBRF2	AP4M1	0.010675
BBRF2	ZNF236	0.011068
BBRF2	AGR2	0.026988
BBRF2	TMSB15B	0.029111
BBRF2	HBQ1	0.042266
BBRF2	YPR022C	0.038026
BBRF2	IRF2BP1	0.0179
BBRF2	ZNF746	0.011376
BBRF2	GOLGA8CP	0.14406
BBRF2	DBNDD2	0.052244
BBRF2	FYB	0.034036
BBRF2	IL17RE	0.025765
BBRF2	KCNK12	0.025562
BBRF2	CMBL	0.01022
BBRF2	TMEM39B	0.016836
BBRF2	CCDC150	0.033141
BBRF2	PSME2	0.030062
BBRF2	MAD1L1	0.025043
BBRF2	C1orf105	0.054425
BBRF2	PRADC1	0.049087
BBRF2	VPREB1	0.013209
BBRF2	TMEM184C	0.037872
BBRF2	PMM1	0.01099
BBRF2	PARPBP	0.02516
BBRF2	FANCA	0.014668
BBRF2	FIP1L1	0.085158
BBRF2	ATP6V0D1	0.023053
BBRF2	TPD52L1	0.092914
BBRF2	ASPHD1	0.045885
BBRF2	ZNF17	0.011643
BBRF2	SPAG17	0.037234
BBRF2	PGBD1	0.015958
BBRF2	ZNHIT2	0.023033
BBRF2	FRG1	0.039307
BBRF2	ZBP1	0.027257
BBRF2	ZNF615	0.012384
BBRF2	TC2N	0.010668
BBRF2	ALKBH1	0.017489
BBRF2	NMT2	0.024559
BBRF2	TMEM252	0.010601
BBRF2	TBC1D28	0.020655
BBRF2	POLR3F	0.03307
BBRF2	S100A3	0.013366
BBRF2	GIMAP2	0.010507
BBRF2	DCST1	0.014911
BBRF2	GLMN	0.015202
BBRF2	OR4F4	0.030153
BBRF2	MAGEC2	0.013043
BBRF2	POU2AF1	0.014642
BBRF2	TMEM215	0.023625

BBRF2	CCDC176	0.1297
BBRF2	CASQ2	0.01164
BBRF2	GOLGA6C	0.028446
BBRF2	SELT	0.012774
BBRF2	PUS7L	0.019977
BBRF2	HMBS	0.027066
BBRF2	CRTAP	0.016014
BBRF2	CTAGE4	0.015032
BBRF2	PLOD2	0.01848
BBRF2	GCH1	0.046917
BBRF2	DAGLB	0.011409
BBRF2	LINC00597	0.015502
BBRF2	ZNF793	0.01038
BBRF2	NKD2	0.010431
BBRF2	UBL3	0.034381
BBRF2	TRAPPC1	0.045343
BBRF2	KRTAP13-4	0.037638
BBRF2	CASD1	0.10857
BBRF2	RGMA	0.052272
BBRF2	AP5B1	0.042768
BBRF2	ZNF350	0.012756
BBRF2	SPIRE2	0.022471
BBRF2	TMEM132D	0.014028
BBRF2	NBPF14	0.039537
BBRF2	HORMAD2	0.021668
BBRF2	SCAF1	0.04933
BBRF2	SLC4A5	0.017699
BBRF2	CHKA	0.035933
BBRF2	C19orf71	0.055228
BBRF2	RNASE9	0.022754
BBRF2	SNX29P2	0.034873
BBRF2	SLC31A1	0.018219
BBRF2	RHPN1-AS1	0.045291
BBRF2	C6orf118	0.019416
BBRF2	NDRG3	0.017579
BBRF2	NXF3	0.035764
BBRF2	IGFL3	0.054628
BBRF2	TGOLN2	0.052001
BBRF2	LINC00471	0.0182
BBRF2	HABP4	0.05235
BBRF2	LFNG	0.011304
BBRF2	DEFB132	0.031966
BBRF2	GPS2	0.034995
BBRF2	CHIT1	0.012852
BBRF2	KLKP1	0.031802
BBRF2	B4GALT3	0.012393
BBRF2	TMC3	0.022962
BBRF2	SSR3	0.021399
BBRF2	ATXN2L	0.012154
BBRF2	PLD4	0.026014
BBRF2	ABRA	0.045115
BBRF2	CHMP1B	0.015625
BBRF2	ADORA3	0.045184
BBRF2	AKAP2	0.036187
BBRF2	HFE2	0.026053
BBRF2	G4	0.012811
BBRF2	ICA1	0.016644
BBRF2	IDH3B	0.012351
BBRF2	PRIMPOL	0.014104
BBRF2	ABHD17A	0.017314
BBRF2	CCDC137	0.11046
BBRF2	GPR179	0.097943
BBRF2	TIFAB	0.038763
BBRF2	NCAPG	0.057262
BBRF2	NANOS2	0.024171
BBRF2	CATSPERG	0.050747
BBRF2	BCL7C	0.098953
BBRF2	TAS2R9	0.026913

BBRF2	SLC16A6	0.019122
BBRF2	DERL2	0.014805
BBRF2	ZNF415	0.012644
BBRF2	OR14L1P	0.030984
BBRF2	HILS1	0.0182
BBRF2	JOSD1	0.025792
BBRF2	BCDIN3D	0.088033
BBRF2	ZNF830	0.055374
BBRF2	CROCCP3	0.02242
BBRF2	NXT2	0.032554
BBRF2	GORAB	0.037885
BBRF2	FAM151B	0.025289
BBRF2	NSUN7	0.11133
BBRF2	MAGEB18	0.021521
BBRF2	SNX11	0.01007
BBRF2	GCSAML	0.033861
BBRF2	NOG	0.01437
BBRF2	METTL4	0.017388
BBRF2	PRPSAP1	0.010878
BBRF2	TBRG4	0.038362
BBRF2	OR2V1	0.03146
BBRF2	FNIP2	0.14339
BBRF2	OR4F15	0.030728
BBRF2	TREML1	0.017232
BBRF2	RPP21	0.055462
BBRF2	STMN2	0.027224
BBRF2	NPIPA1	0.045329
BBRF2	FAM47B	0.062093
BBRF2	CD33	0.010235
BBRF2	RDM1	0.04174
BBRF2	SDHAF2	0.013479
BBRF2	SUPT20HL2	0.0384
BBRF2	TCEB3C	0.015422
BBRF2	MSMB	0.016134
BBRF2	CTNNBIP1	0.01248
BBRF2	CENPH	0.027032
BBRF2	ENPP5	0.010967
BBRF2	SLC5A9	0.012058
BBRF2	GCLC	0.012169
BBRF2	FBXO46	0.068604
BBRF2	TWISTNB	0.012739
BBRF2	SRL	0.03795
BBRF2	TIMM44	0.033412
BBRF2	GTF2E1	0.066786
BBRF2	AP3S2	0.011907
BBRF2	GOLGA2P5	0.035662
BBRF2	OXA1L	0.021983
BBRF2	INTS10	0.036492
BBRF2	UTP3	0.029454
BBRF2	CDR2	0.028809
BBRF2	RSC1A1	0.015244
BBRF2	LINC00523	0.048623
BBRF2	SLC12A1	0.012307
BBRF2	OR2T8	0.030984
BBRF2	TSPAN16	0.01457
BBRF2	SMIM14	0.030558
BBRF2	RABAC1	0.016944
BBRF2	TBC1D3G	0.01421
BBRF2	LIN54	0.016756
BBRF2	LRAT	0.01048
BBRF2	GRINA	0.01499
BBRF2	RHD	0.053219
BBRF2	RABGAP1L	0.012249
BBRF2	COMMD1	0.010939
BBRF2	ZNF354A	0.01139
BBRF2	ALKBH5	0.013005
BBRF2	MAGEB6	0.014725
BBRF2	GEMIN6	0.030484

BBRF2	SLC1A5	0.019121
BBRF2	CSN2	0.071261
BBRF2	EXOSC1	0.027399
BBRF2	SEC61G	0.012417
BBRF2	VWA5B1	0.029515
BBRF2	RMI1	0.026874
BBRF2	FAM69B	0.159
BBRF2	CACNG4	0.017083
BBRF2	ZFP1	0.014006
BBRF2	ROM1	0.043111
BBRF2	SOAT1	0.010907
BBRF2	MFSD4	0.019789
BBRF2	LAS2	0.011145
BBRF2	ARL14EPL	0.080171
BBRF2	TMED9	0.012237
BBRF2	PLBD1	0.01535
BBRF2	SNRPC	0.010754
BBRF2	YKL023C-A	0.036558
BBRF2	STXBP6	0.017868
BBRF2	PITPNM1	0.019595
BBRF2	GNS	0.011461
BBRF2	PALM2	0.014312
BBRF2	OGFR	0.041133
BBRF2	TAS2R30	0.026913
BBRF2	OGFOD2	0.014532
BBRF2	ATP6V1E2	0.042312
BBRF2	PALM	0.024325
BBRF2	NRDE2	0.019017
BBRF2	SLC34A1	0.088685
BBRF2	SLN	0.05835
BBRF2	OR10P1	0.032661
BBRF2	NOP56	0.0171
BBRF2	PLEKHH3	0.01063
BBRF2	BAHD1	0.014035
BBRF2	MBD5	0.040355
BBRF2	UGT92A1	0.03492
BBRF2	ELN	0.051571
BBRF2	LTF	0.015051
BBRF2	TMEM233	0.018995
BBRF2	KIAA1217	0.012925
BBRF2	NPTXR	0.013585
BBRF2	ORM2	0.025139
BBRF2	TMEM88	0.05839
BBRF2	SAMM50	0.013777
BBRF2	VPS13C	0.027854
BBRF2	ZNF41	0.011701
BBRF2	C17orf99	0.01851
BBRF2	EDEM2	0.011942
BBRF2	XKR3	0.059235
BBRF2	CHGA	0.11073
BBRF2	TMEM136	0.024494
BBRF2	LCN6	0.010208
BBRF2	UQCRQ	0.013918
BBRF2	C17orf66	0.01996
BBRF2	TAS2R42	0.01389
BBRF2	TET3	0.027221
BBRF2	ZNF33A	0.011463
BBRF2	FAM174B	0.03286
BBRF2	SPON2	0.011196
BBRF2	OR10H5	0.030153
BBRF2	R3HCC1L	0.015385
BBRF2	PIH1D2	0.048092
BBRF2	IWS1	0.076725
BBRF2	BTBD17	0.010792
BBRF2	NT5DC3	0.016975
BBRF2	POM121L12	0.037499
BBRF2	SPINK13	0.014227
BBRF2	PVRIG	0.055857

BBRF2	CPSF2	0.013975
BBRF2	SLC38A9	0.020174
BBRF2	RASSF6	0.012909
BBRF2	NDEL1	0.029331
BBRF2	PTX3	0.01062
BBRF2	SPINK5	0.01879
BBRF2	ACOT7	0.018435
BBRF2	PRO0255	0.02712
BBRF2	UNC93B1	0.036974
BBRF2	LINC00472	0.036154
BBRF2	PAQR4	0.018047
BBRF2	PNMA3	0.012409
BBRF2	OR2AK2	0.029347
BBRF2	GOLGA6L4	0.035662
BBRF2	GOLGA6L6	0.034065
BBRF2	KIAA1958	0.031593
BBRF2	SLAIN1	0.085096
BBRF2	SAA2	0.051546
BBRF2	zag-1	0.01142
BBRF2	ARL6IP5	0.030321
BBRF2	G4	0.031415
BBRF2	DMRTC2	0.010933
BBRF2	ZNF326	0.054158
BBRF2	STEAP1	0.021316
BBRF2	MYOZ3	0.016103
BBRF2	OR4F21	0.030984
BBRF2	DTX2	0.011892
BBRF2	NOM1	0.01259
BBRF2	SMYD5	0.038808
BBRF2	GG1	0.016493
BBRF2	FRAT2	0.036766
BBRF2	ITGB1BP2	0.010091
BBRF2	ANXA2R	0.10781
BBRF2	YML009W-B	0.062731
BBRF2	C14orf178	0.021797
BBRF2	LINC00116	0.06081
BBRF2	NAGA	0.016914
BBRF2	MED9	0.011104
BBRF2	LGALS13	0.023069
BBRF2	TEX19	0.025245
BBRF2	NIPA1	0.042986
BBRF2	PANX3	0.014369
BBRF2	BID	0.045634
BBRF2	COMMD2	0.081409
BBRF2	SDPR	0.02438
BBRF2	C1orf168	0.017918
BBRF2	UAP1	0.010077
BBRF2	NIPSNAP3A	0.03022
BBRF2	YTHDF1	0.032922
BBRF2	NRGN	0.021887
BBRF2	PIGL	0.03153
BBRF2	MGST2	0.017264
BBRF2	CALCA	0.012401
BBRF2	MRFAP1L1	0.038113
BBRF2	MFAP5	0.019975
BBRF2	TERT	0.013494
BBRF2	MEAF6	0.02503
BBRF2	FAM160A1	0.057935
BBRF2	SLC13A1	0.020324
BBRF2	PIR	0.046053
BBRF2	SPIN1	0.041178
BBRF2	MAFK	0.013003
BBRF2	WHAMMP3	0.011409
BBRF2	ID2B	0.013994
BBRF2	PRAMEF13	0.13334
BBRF2	PMS2	0.010221
BBRF2	SRRM1	0.10056
BBRF2	ALB	0.025671

BBRF2	RBAK	0.012161
BBRF2	YJR030C	0.065608
BBRF2	KIAA1841	0.079311
BBRF2	SDF2L1	0.013702
BBRF2	STAG3L1	0.01842
BBRF2	C1orf196	0.03334
BBRF2	PTGES	0.015115
BBRF2	STPG2	0.012573
BBRF2	WBSCR23	0.022486
BBRF2	HTN3	0.039639
BBRF2	ARL6IP6	0.03201
BBRF2	PRODH	0.060299
BBRF2	YJEFN3	0.010637
BBRF2	MFSD6	0.10297
BBRF2	AURKAIP1	0.016169
BBRF2	ZC4H2	0.011073
BBRF2	XAGE2	0.055577
BBRF2	YML009W-B	0.022266
BBRF2	ELOVL3	0.013748
BBRF2	SEC23IP	0.01174
BBRF2	LRCOL1	0.106
BBRF2	PTTG3P	0.026007
BBRF2	DTYMK	0.038121
BBRF2	GOLGA6L18	0.019773
BBRF2	AFF1	0.011276
BBRF2	MRPL53	0.014485
BBRF2	YTHDC1	0.050648
BBRF2	CSHL1	0.01209
BBRF2	SLC52A3	0.022251
BBRF2	NBPF6	0.018081
BBRF2	TM6SF1	0.034385
BBRF2	MRPS10	0.057904
BBRF2	GEMIN7	0.04689
BBRF2	LAMP5	0.060114
BBRF2	VSTM5	0.012432
BBRF2	AMY1A	0.026981
BBRF2	G4	0.014018
BBRF2	TXNIP	0.074815
BBRF2	PITPNA	0.03781
BBRF2	TSPAN11	0.010037
BBRF2	TIMM17A	0.011949
BBRF2	APRG1	0.042484
BBRF2	BCORP1	0.016114
BBRF2	BRAT1	0.013001
BBRF2	P4HA2	0.033078
BBRF2	TBC1D3	0.011181
BBRF2	RNASEH2A	0.049833
BBRF2	GTF3C6	0.041796
BBRF2	WIBG	0.069309
BBRF2	IFT140	0.017343
BBRF2	FAM221B	0.027953
BBRF2	SLCO1A2	0.028553
BBRF2	FRYL	0.018022
BBRF2	PDE6H	0.050309
BBRF2	TRH	0.020163
BBRF2	RIF1	0.024798
BBRF2	DMRTA2	0.010933
BBRF2	RPL18	0.022099
BBRF2	ERLIN2	0.023231
BBRF2	MPTX1	0.030552
BBRF2	AMER2	0.05677
BBRF2	EXOSC3	0.017769
BBRF2	TRAF3IP1	0.03586
BBRF2	CCDC172	0.016175
BBRF2	ERI3	0.018299
BBRF2	YTHDF2	0.060378
BBRF2	SKP1	0.022722
BBRF2	ZNF212	0.013928

BBRF2	LINS	0.020963
BBRF2	KHDC3L	0.035992
BBRF2	UPK1B	0.014467
BBRF2	OR2J2	0.030153
BBRF2	SAP30	0.024739
BBRF2	SHCBP1	0.19517
BBRF2	COX10	0.05382
BBRF2	POLRMT	0.036172
BBRF2	BCAS2	0.010635
BBRF2	C14orf80	0.064535
BBRF2	C1orf94	0.17182
BBRF2	OR10G2	0.040757
BBRF2	SLC16A14	0.012159
BBRF2	WFDC12	0.032062
BBRF2	UBAC2	0.011623
BBRF2	NDUFB11	0.010402
BBRF2	MCUR1	0.016031
BBRF2	HGD	0.078765
BBRF2	OR5L1	0.030979
BBRF2	PSRC1	0.012703
BBRF2	REEP1	0.012453
BBRF2	BSN	0.079084
BBRF2	C17orf74	0.038362
BBRF2	C10orf54	0.021867
BBRF2	ANAPC1	0.020297
BBRF2	PNRC2	0.049997
BBRF2	CMTM4	0.011143
BBRF2	CDCA5	0.019173
BBRF2	FAM198B	0.017578
BBRF2	GOLGA6A	0.018934
BBRF2	HEMK1	0.027938
BBRF2	FAM210A	0.09568
BBRF2	PNLDC1	0.035752
BBRF2	C19orf35	0.027298
BBRF2	TSPAN10	0.030022
BBRF2	ANO9	0.034614
BBRF2	HBZ	0.041088
BBRF2	DDHD2	0.014149
BBRF2	YIR021W-A	0.024432
BBRF2	TACC3	0.014784
BBRF2	LAGE3	0.024826
BBRF2	ICOS	0.033846
BBRF2	COMMD9	0.045442
BBRF2	DNASE1L2	0.088344
BBRF2	UBXN2B	0.012226
BBRF2	SYPL1	0.11899
BBRF2	ERLIN1	0.08516
BBRF2	SBSPON	0.010588
BBRF2	UBXN11	0.031592
BBRF2	ANGEL2	0.019256
BBRF2	AHSA1	0.048103
BBRF2	CRB3	0.041528
BBRF2	FDFT1	0.015746
BBRF2	ITIH2	0.020794
BBRF2	PP632	0.01309
BBRF2	ZNF222	0.011444
BBRF2	OR6N2	0.036492
BBRF2	KCNJ9	0.011798
BBRF2	CTAGE3P	0.010068
BBRF2	GAGE4	0.031361
BBRF2	MPHOSPH10	0.022485
BBRF2	AOC2	0.02596
BBRF2	ZSWIM7	0.024962
BBRF2	C2CD4D	0.028459
BBRF2	ZK1128.3	0.024813
BBRF2	RRP1B	0.0593
BBRF2	AGTPBP1	0.017338
BBRF2	PHACTR4	0.020718

BBRF2	PGK2	0.021573
BBRF2	TBC1D3B	0.010188
BBRF2	NUP98	0.019133
BBRF2	SLC1A1	0.01869
BBRF2	MYADM	0.014577
BBRF2	SLC37A3	0.010277
BBRF2	SLC1A2	0.01869
BBRF2	COMMD8	0.022317
BBRF2	PNMAL1	0.01991
BBRF2	LY6K	0.043764
BBRF2	GLCCI1	0.032095
BBRF2	SLC30A7	0.011164
BBRF2	ATP5S	0.018807
BBRF2	NCOA4	0.019994
BBRF2	TSPAN15	0.011502
BBRF2	IFITM5	0.01178
BBRF2	TPT1P8	0.030793
BBRF2	GALNT15	0.013265
BBRF2	RGS7BP	0.052263
BBRF2	GRIFIN	0.011302
BBRF2	ABT1	0.011507
BBRF2	NDRG2	0.015842
BBRF2	FAM19A3	0.13552
BBRF2	PSME1	0.016014
BBRF2	CPED1	0.047952
BBRF2	CXorf30	0.035984
BBRF2	CD164L2	0.036233
BBRF2	MUS81	0.014519
BBRF2	MLF2	0.027381
BBRF2	CAMSAP3	0.054231
BBRF2	RPL8	0.024389
BBRF2	RPL35	0.017003
BBRF2	ZNF785	0.012132
BBRF2	USP53	0.020688
BBRF2	OR2L5	0.033291
BBRF2	NABP1	0.030933
BBRF2	CCDC132	0.020914
BBRF2	OR4D6	0.030153
BBRF2	MOAP1	0.02478
BBRF2	RPS19	0.02792
BBRF2	SDSL	0.044305
BBRF2	MSL1	0.022106
BBRF2	NPHP4	0.057092
BBRF2	CRLF2	0.012824
BBRF2	ATG4C	0.038813
BBRF2	FAM21B	0.068867
BBRF2	PLSCR4	0.057372
BBRF2	DNAJC9-AS1	0.058562
BBRF2	CENPF	0.014727
BBRF2	OR4K13	0.037163
BBRF2	TMEM30C	0.011771
BBRF2	STAG3L2	0.027947
BBRF2	SCAMP3	0.011256
BBRF2	SYCE3	0.031861
BBRF2	FAM45A	0.049897
BBRF2	FBXO25	0.041941
BBRF2	TCP10L2	0.011082
BBRF2	GOLGB1	0.027685
BBRF2	SETD3	0.032035
BBRF2	HBA1	0.034656
BBRF2	CYB561D1	0.054262
BBRF2	EVC	0.010731
BBRF2	C1orf106	0.070285
BBRF2	OR2T34	0.030153
BBRF2	GOLGA6L3	0.035662
BBRF2	KRTAP12-4	0.01492
BBRF2	WIPF3	0.098761
BBRF2	C5orf55	0.044878

BBRF2	WWC2-AS2	0.040779
BBRF2	EVA1C	0.021348
BBRF2	SAP18	0.02642
BBRF2	SYNGR3	0.074252
BBRF2	CHI3L1	0.010497
BBRF2	CAP1	0.021884
BBRF2	SLC9B2	0.023209
BBRF2	UBXN4	0.025013
BBRF2	FBXL18	0.023855
BBRF2	KCNK13	0.014689
BBRF2	RPP25L	0.052114
BBRF2	CCDC7	0.0212
BBRF2	YFL013W-A	0.033247
BBRF2	HHATL	0.020335
BBRF2	NDUFAF7	0.059592
BBRF2	NBPF20	0.039537
BBRF2	ESF1	0.083055
BBRF2	GAGE1	0.031361
BBRF2	RGS9BP	0.012476
BBRF2	OR2T10	0.036492
BBRF2	CUL7	0.010467
BBRF2	BEX4	0.021144
BBRF2	ANO2	0.044222
BBRF2	NSUN5	0.042633
BBRF2	KRTAP16-1	0.038557
BBRF2	TMEM45B	0.016963
BBRF2	ZNF366	0.011557
BBRF2	LAMP2	0.016267
BBRF2	TEN1	0.010245
BBRF2	AMPD2	0.011335
BBRF2	MZT1	0.018084
BBRF2	OR13D1	0.029794
BBRF2	SLC5A10	0.012058
BBRF2	PACS2	0.070891
BBRF2	AFM	0.039996
BBRF2	PGK1	0.022133
BBRF2	BOLA1	0.018465
BBRF2	SHMT1	0.018361
BBRF2	RBM27	0.030311
BBRF2	MTL5	0.028292
BBRF2	CNPY4	0.10692
BBRF2	NELFA	0.08515
BBRF2	PHACTR1	0.03773
BBRF2	FAM92B	0.012425
BBRF2	ZNF660	0.011239
BBRF2	UBXN2A	0.053179
BBRF2	ANO6	0.016605
BBRF2	DHFRL1	0.031431
BBRF2	APOO	0.050292
BBRF2	MED28	0.013678
BBRF2	FUNDC2	0.022215
BBRF2	FAM19A4	0.044084
BBRF2	ARRDC3	0.048332
BBRF2	IPMK	0.010795
BBRF2	SERBP1	0.045358
BBRF2	PRR19	0.012719
BBRF2	STAG3	0.015975
BBRF2	RRBP1	0.012664
BBRF2	RPRML	0.010515
BBRF2	FAM110C	0.020231
BBRF2	MT1X	0.011438
BBRF2	THAP11	0.017512
BBRF2	WBP2NL	0.036541
BBRF2	GAREML	0.063006
BBRF2	MBOAT1	0.039484
BBRF2	SYP	0.014341
BBRF2	MIMI	0.018125
BBRF2	OR2T3	0.030153

BBRF2	TNRC6A	0.020273
BBRF2	TMCO6	0.034082
BBRF2	SLC16A7	0.019868
BBRF2	SLC4A3	0.020151
BBRF2	SPG20	0.024549
BBRF2	TICRR	0.035177
BBRF2	SLX1A	0.030519
BBRF2	IL27	0.025571
BBRF2	ytzL	0.15663
BBRF2	EMID1	0.02927
BBRF2	ZNF691	0.011104
BBRF2	WDR96	0.055252
BBRF2	GRAMD1C	0.01526
BBRF2	STT3B	0.022198
BBRF2	DAD1	0.032924
BBRF2	COL17A1	0.055072
BBRF2	EIF6	0.029098
BBRF2	OR4K2	0.033572
BBRF2	TSEN2	0.063698
BBRF2	TNRC6B	0.032993
BBRF2	C15orf65	0.028283
BBRF2	FAM160B1	0.039496
BBRF2	MAPKAP1	0.057236
BBRF2	NUCB1	0.034863
BBRF2	PRIMA1	0.072656
BBRF2	ELOVL6	0.013748
BBRF2	ZNF577	0.011225
BBRF2	OFD1	0.043985
BBRF2	YIPF7	0.018798
BBRF2	SSX1	0.055064
BBRF2	MRPL32	0.018591
BBRF2	ZWINT	0.016488
BBRF2	NAPG	0.022022
BBRF2	RBBP8	0.011886
BBRF2	FCF1	0.030814
BBRF2	FBXL8	0.17743
BBRF2	STX17	0.012235
BBRF2	TAS2R45	0.023629
BBRF2	MFAP1	0.067123
BBRF2	BSCL2	0.010122
BBRF2	OR14J1	0.030153
BBRF2	TTYH1	0.14553
BBRF2	NUPR1L	0.012674
BBRF2	ZNF12	0.012272
BBRF2	C10orf82	0.052403
BBRF2	ERICH1-AS1	0.057245
BBRF2	CCRN4L	0.034371
BBRF2	CHCHD6	0.037104
BBRF2	DRG2	0.016946
BBRF2	ATXN1	0.042574
BBRF2	TTYH2	0.013269
BBRF2	C6orf89	0.01561
BBRF2	BEND7	0.014253
BBRF2	REEP5	0.011098
BBRF2	MYBBP1A	0.011217
BBRF2	SYNGR1	0.069958
BBRF2	DAP3	0.010329
BBRF2	OR2T1	0.033291
BBRF2	GOLPH3L	0.025349
BBRF2	ORMDL2	0.087326
BBRF2	XKR5	0.026947
BBRF2	RAB3IP	0.015346
BBRF2	DYDC2	0.022707
BBRF2	XPO5	0.011475
BBRF2	PRPF31	0.010268
BBRF2	RPL10	0.01553
BBRF2	LSM14B	0.012104
BBRF2	FAM203B	0.04241

BBRF2	TYW5	0.014137
BBRF2	BCL9L	0.053827
BBRF2	KCNE1	0.0184
BBRF2	PRUNE2	0.013953
BBRF2	TET1	0.024724
BBRF2	TGM2	0.02068
BBRF2	ST20	0.034369
BBRF2	BAG1	0.017542
BBRF2	CCDC91	0.019621
BBRF2	QTRTD1	0.031246
BBRF2	YIF1A	0.050191
BBRF2	HEATR5A	0.028806
BBRF2	BEX1	0.10064
BBRF2	TTYH3	0.13869
BBRF2	TECTB	0.015769
BBRF2	PLGLB1	0.01457
BBRF2	PSMD13	0.019379
BBRF2	EID2	0.019917
BBRF2	ELOVL7	0.013748
BBRF2	FAM184A	0.019142
BBRF2	PRPH2	0.028782
BBRF2	PCYT1A	0.020691
BBRF2	OR2AJ1	0.03146
BBRF2	OR2T12	0.030984
BBRF2	DDIT4L	0.017042
BBRF2	ARRDC4	0.027159
BBRF2	CNIH4	0.021187
BBRF2	CEP63	0.011393
BBRF2	CD8A	0.011788
BBRF2	C11orf44	0.021876
BBRF2	LENG8	0.020276
BBRF2	VWA9	0.02531
BBRF2	UNG	0.033559
BBRF2	MTTP	0.075395
BBRF2	TREM2	0.01714
BBRF2	MYOZ2	0.051623
BBRF2	C16orf72	0.037807
BBRF2	TM9SF2	0.020849
BBRF2	GADD45G	0.049933
BBRF2	HNRNPH2	0.011731
BBRF2	BEND2	0.038464
BBRF2	EIF4B	0.018298
BBRF2	LAPTM4A	0.049549
BBRF2	VMP1	0.016414
BBRF2	DKKL1	0.0381
BBRF2	EXOC3L2	0.034236
BBRF2	UNC119	0.041718
BBRF2	SLC52A2	0.022251
BBRF2	KIAA1429	0.022494
BBRF2	POM121L2	0.029641
BBRF2	DGCR8	0.013148
BBRF2	GOLGA6L10	0.019773
BBRF2	RIC8B	0.014144
BBRF2	FAM188B	0.14872
BBRF2	OR2V2	0.03146
BBRF2	GRN	0.022504
BBRF2	MISP	0.046922
BBRF2	CCDC180	0.029857
BBRF2	RHCG	0.038832
BBRF2	GXYLT1	0.018016
BBRF2	ANTXR1	0.035751
BBRF2	FAM231B	0.037149
BBRF2	POMT2	0.013402
BBRF2	KCNJ12	0.016918
BBRF2	YIPF6	0.023612
BBRF2	SELO	0.02891
BBRF2	NUCB2	0.011041
BBRF2	SMIM19	0.019167

BBRF2	MGAT1	0.030328
BBRF2	UHRF1BP1L	0.043462
BBRF2	UNQ6126/PRO20091	0.042701
BBRF2	OR1K1	0.037527
BBRF2	NPC2	0.03245
BBRF2	EME2	0.033319
BBRF2	ZNF786	0.012501
BBRF2	GOLGA6D	0.038285
BBRF2	PEX11A	0.040627
BBRF2	APOF	0.021557
BBRF2	YGL014C-A	0.036639
BBRF2	AOC1	0.015946
BBRF2	CDCP1	0.035258
BBRF2	OAS1	0.023818
BBRF2	AFAP1L1	0.019738
BBRF2	RHBG	0.023536
BBRF2	EPB42	0.020286
BBRF2	SLC39A5	0.011026
BBRF2	THRSP	0.031207
BBRF2	MED13L	0.015936
BBRF2	IKBKAP	0.011312
BBRF2	INSL4	0.047839
BBRF2	CCDC151	0.013813
BBRF2	SYNPO	0.042273
BBRF2	IGFLR1	0.062679
BBRF2	CHST13	0.018603
BBRF2	GGNBP2	0.095399
BBRF2	CT55	0.085274
BBRF2	JAKMIP2	0.01747
BBRF2	EPG5	0.053295
BBRF2	B3GNTL1	0.027669
BBRF2	SPATA4	0.021445
BBRF2	TTLL4	0.012565
BBRF2	NXT1	0.020319
BBRF2	UPRT	0.029509
BBRF2	DGCR14	0.011941
BBRF2	FLG	0.045338
BBRF2	CHMP7	0.043884
BBRF2	PIH1D1	0.010674
BBRF2	NO66	0.017158
BBRF2	NIPAL4	0.041785
BBRF2	CMTM2	0.035502
BBRF2	FAM126B	0.061937
BBRF2	KCNJ8	0.015016
BBRF2	SLC23A3	0.028883
BBRF2	DET1	0.014487
BBRF2	FAM131A	0.050729
BBRF2	RABEP2	0.15886
BBRF2	SLC18A1	0.01398
BBRF2	CYB561	0.053921
BBRF2	XPO6	0.021715
BBRF2	SERTAD1	0.036943
BBRF2	SYS1	0.024301
BBRF2	IFI27	0.041192
BBRF2	INTS4	0.015009
BBRF2	DYNLRB1	0.016819
BBRF2	GYPC	0.020109
BBRF2	EGLN2	0.011426
BBRF2	CHAF1A	0.1142
BBRF2	KIAA0319L	0.018138
BBRF2	ARSJ	0.011506
BBRF2	OR10R2	0.030153
BBRF2	UNQ5830/PRO19650/PRO19816	0.017057
BBRF2	PTCRA	0.020726
BBRF2	SAA1	0.037473
BBRF2	TP53INP1	0.060582
BBRF2	TAS2R39	0.025432
BBRF2	ATXN7L3	0.040017

BBRF2	MNCb-2875	0.016765
BBRF2	CATSPERB	0.029652
BBRF2	CBWD7	0.023066
BBRF2	EIF1B	0.032929
BBRF2	MUC12	0.026751
BBRF2	SHMT2	0.010972
BBRF2	TBC1D3C	0.01343
BBRF2	CLUAP1	0.037567
BBRF2	GPATCH3	0.027808
BBRF2	SLC52A1	0.022251
BBRF2	ADAT3	0.010252
BBRF2	PGLYRP2	0.010946
BBRF2	C8orf74	0.014946
BBRF2	CCDC104	0.015788
BBRF2	YOD1	0.02108
BBRF2	TMEM52	0.029159
BBRF2	FAIM3	0.030288
BBRF2	MAB21L2	0.025653
BBRF2	XAGE3	0.0619
BBRF2	GGN	0.089885
BBRF2	ZNF256	0.011822
BBRF2	CCDC85A	0.010016
BBRF2	C1orf111	0.036753
BBRF2	NME6	0.021185
BBRF2	BPGM	0.0187
BBRF2	COX18	0.010679
BBRF2	CSF1	0.019893
BBRF2	PFDN2	0.022571
BBRF2	AMPD1	0.010201
BBRF2	OR2AE1	0.032661
BBRF2	WFDC1	0.019071
BBRF2	MTERFD3	0.01973
BBRF2	SLC4A9	0.020151
BBRF2	MIS18BP1	0.037047
BBRF2	EXOSC9	0.013642
BBRF2	TMEM120B	0.034462
BBRF2	G4	0.01209
BBRF2	BEX5	0.01103
BBRF2	LAMP1	0.058265
BBRF2	CHGB	0.18973
BBRF2	SLC16A11	0.038964
BBRF2	CMTR2	0.022461
BBRF2	TMEM204	0.068631
BBRF2	FH	0.02118
BBRF2	EQTN	0.03248
BBRF2	SLC1A7	0.014146
BBRF2	HERPUD2	0.038161
BBRF2	TGM7	0.012287
BBRF2	VPS36	0.018348
BBRF2	CD3E	0.01531
BBRF2	LGALS9B	0.014359
BBRF2	NT5C3A	0.071571
BBRF2	SART1	0.035814
BBRF2	MT-CO1	0.021405
BBRF2	MAGED4	0.011013
BBRF2	IBA57	0.054798
BBRF2	EIF3L	0.020708
BBRF2	ATCAY	0.019711
BBRF2	PPA1	0.013263
BBRF2	G4	0.06308
BBRF2	FAM162B	0.039674
BBRF2	MPPED2	0.01812
BBRF2	TMED5	0.014632
BBRF2	TCTN3	0.021153
BBRF2	TUBGCP4	0.010034
BBRF2	OR2M7	0.030153
BBRF2	MT1L	0.011424
BBRF2	LINC00596	0.020124

BBRF2	SCAMP5	0.011256
BBRF2	PPP1R3E	0.01561
BBRF2	WLS	0.029021
BBRF2	TBC1D17	0.013045
BBRF2	IFT52	0.073415
BBRF2	RPS10P5	0.012589
BBRF2	CT45A3	0.011107
BBRF2	TMEM81	0.021137
BBRF2	CCP110	0.019436
BBRF2	CAGE1	0.021763
BBRF2	VWA5A	0.065499
BBRF2	LINC00205	0.040962
BBRF2	FAM46B	0.036664
BBRF2	FEZ2	0.030592
BBRF2	PNAS-138	0.015108
BBRF2	CD2BP2	0.020846
BBRF2	ACOT8	0.021909
BBRF2	TPPP3	0.062166
BBRF2	SAP130	0.032933
BBRF2	NCOA6	0.045871
BBRF2	HTN1	0.025694
BBRF2	CCDC26	0.016924
BBRF2	NSUN5P2	0.06602
BBRF2	GRAMD1A	0.01633
BBRF2	MT1F	0.011195
BBRF2	CCDC85C	0.034846
BBRF2	TXLNG2P	0.021923
BBRF2	TGM1	0.018576
BBRF2	RPL23	0.016088
BBRF2	SUPT20HL1	0.0384
BBRF2	TMEM8B	0.022681
BBRF2	TSPAN9	0.013209
BBRF2	SPATA31A6	0.012426
BBRF2	SLC14A2	0.042863
BBRF2	GBP3	0.013293
BBRF2	RPL10L	0.01553
BBRF2	ZNF778	0.010975
BBRF2	PROK2	0.010558
BBRF2	RPL26L1	0.013552
BBRF2	IL4R	0.01536
BBRF2	MAGEC1	0.013015
BBRF2	SAP25	0.043553
BBRF2	MT1G	0.027346
BBRF2	MID1IP1	0.024975
BBRF2	DLGAP5	0.018684
BBRF2	AHSP	0.02001
BBRF2	VWA5B2	0.041226
BBRF2	KCNJ11	0.013018
BBRF2	IP6K3	0.024681
BBRF2	PEPD	0.044062
BBRF2	NKAIN2	0.093389
BBRF2	NAF1	0.035694
BBRF2	PRAME	0.078998
BBRF2	EIF4E2	0.011971
BBRF2	SLCO5A1	0.016951
BBRF2	ENPP3	0.0108
BBRF2	TAS2R10	0.043074
BBRF2	TMSB10	0.029111
BBRF2	MB	0.028133
BBRF2	EXOC6	0.014255
BBRF2	EIF2A	0.010648
BBRF2	AGRP	0.047249
BBRF2	ATG12	0.01246
BBRF2	PBOV1	0.021356
BBRF2	RPS4Y2	0.012056
BBRF2	OR9K2	0.03146
BBRF2	NAPA	0.014278
BBRF2	DRAXIN	0.022962

49233	BBRF2	APOL6	0.026347
49234	BBRF2	BZW1	0.011665
49235	BBRF2	FAM149B1	0.015871
49236	BBRF2	FTSJ1	0.049715
49237	BBRF2	KCNJ6	0.011468
49238	BBRF2	LRRFIP1	0.014054
49239	BBRF2	PPP2R3C	0.04975
49240	BBRF2	MT1H	0.011424
49241	BBRF2	ATG10	0.048791
49242	BBRF2	ACD	0.012556
49243	BBRF2	TAS2R40	0.014064
49244	BBRF2	EIF3E	0.013626
49245	BBRF2	EIF4E	0.016313
49246	BBRF2	BBC3	0.054383
49247	BBRF2	OAS3	0.023777
49248	BBRF2	RQCD1	0.038238
49249	BBRF2	NPB	0.041268
49250	BBRF2	LMF2	0.03399
49251	BBRF2	ATP5F1	0.027688
49252	BBRF2	RPL6	0.025455
49253	BBRF2	MIF4GD	0.011727
49254	BBRF2	C19orf12	0.06681
49255	BBRF2	ISYNA1	0.031437
49256	BBRF2	ITPKA	0.043484
49257	BBRF2	C12orf63	0.028822
49258	BBRF2	SLC16A10	0.020164
49259	BBRF2	TAS2R1	0.022169
49260	BBRF2	UCKL1	0.026128
49261	BBRF2	DMRT2	0.011817
49262	BBRF2	IPO7	0.021268
49263	BBRF2	LEMD2	0.024817
49264	BBRF2	ZPBP	0.012675
49265	BBRF2	DPM2	0.03168
49266	BBRF2	SEC11C	0.043779
49267	BBRF2	FAM46A	0.018199
49268	BBRF2	PLGLA	0.01457
49269	BBRF2	TSN	0.042938
49270	BBRF2	LSM14A	0.038416
49271	BBRF2	FRAT1	0.015582
49272	BBRF2	RILP	0.035161
49273	BBRF2	SS18L2	0.012994
49274	BBRF2	SDS	0.019813
49275	BBRF2	PPIP5K1	0.012961
49276	BBRF2	LMAN1	0.015014
49277	BBRF2	UCN3	0.049948
49278	BBRF2	ZNF597	0.013162
49279	BBRF2	CTIF	0.018511
49280	BBRF2	OR4F3	0.030984
49281	BBRF2	TRAP1	0.011923
49282	BBRF2	TCEAL5	0.039329
49283	BBRF2	PEX14	0.02028
49284	BBRF2	JOSD2	0.010239
49285	BBRF2	NIPAL1	0.041785
49286	BBRF2	RPGRIP1	0.02064
49287	BBRF2	SPG21	0.015902
49288	BBRF2	OR10AD1	0.031196
49289	BBRF2	SPPL2C	0.0152
49290	BBRF2	KCNJ13	0.011403
49291	BBRF2	IGFL1	0.032047
49292	BBRF2	POM121	0.059009
49293	BBRF2	KHNYN	0.024639
49294	BBRF2	TNFSF9	0.024763
49295	BBRF2	G4	0.020647
49296	BBRF2	COBL	0.05735
49297	BBRF2	GBE1	0.043835
49298	BBRF2	PORCN	0.03627
49299	BBRF2	STC2	0.014311
49300	BBRF2	DEFB129	0.066872

BBRF2	FAM110B	0.021319
BBRF2	ZNF230	0.010533
BBRF2	OR2AG2	0.032661
BBRF2	LSM12	0.062505
BBRF2	ZNF544	0.011443
BBRF2	ZER1	0.013177
BBRF2	ROPN1L	0.010338
BBRF2	CRYGB	0.014855
BBRF2	UCN	0.019783
BBRF2	TRAPPC9	0.040725
BBRF2	NCDN	0.037625
BBRF2	GC	0.0214
BBRF2	MANF	0.010884
BBRF2	KIAA0907	0.014968
BBRF2	C12orf57	0.056478
BBRF2	SLC18B1	0.025591
BBRF2	ALKBH2	0.041019
BBRF2	TMEM8A	0.07336
BBRF2	IGFL4	0.083108
BBRF2	CRTC2	0.028742
BBRF2	YAE1D1	0.054295
BBRF2	CD82	0.010647
BBRF2	ERICH1	0.024249
BBRF2	GIF	0.038759
BBRF2	AMOTL2	0.010439
BBRF2	PPP4R2	0.047234
BBRF2	STAG3L3	0.027947
BBRF2	TMCO1	0.012474
BBRF2	LIN9	0.010449
BBRF2	LGALS12	0.020949
BBRF2	BVES-AS1	0.023423
BBRF2	NDUFS5	0.013711
BBRF2	TCEAL1	0.037766
BBRF2	MARCKS	0.07298
BBRF2	CACHD1	0.021313
BBRF2	TMSB4X	0.033597
BBRF2	AMELY	0.033519
BBRF2	LEPREL2	0.017619
BBRF2	FLG2	0.018197
BBRF2	CCDC120	0.03817
BBRF2	SPATA31E1	0.2155
BBRF2	POLR2L	0.03838
BBRF2	C1orf145	0.056881
BBRF2	AKAP9	0.026712
BBRF2	NUDC	0.020098
BBRF2	SYNE3	0.013896
BBRF2	NYAP2	0.010054
BBRF2	AMY2A	0.02423
BBRF2	PANK3	0.082983
BBRF2	OTUD1	0.015469
BBRF2	TMEM158	0.021844
BBRF2	MAB21L1	0.016319
BBRF2	GPRASP1	0.027461
BBRF2	VGLL3	0.021117
BBRF2	SFI1	0.09409
BBRF2	C1orf186	0.027526
BBRF2	C2orf71	0.049348
BBRF2	DHFR	0.018514
BBRF2	SMEK2	0.023884
BBRF2	OR2T4	0.032661
BBRF2	PRRG4	0.033523
BBRF2	TRDN	0.030405
BBRF2	ZNF710	0.010551
BBRF2	UBXN8	0.02674
BBRF2	APCS	0.038692
BBRF2	AKAP3	0.04179
BBRF2	GOLGA8I	0.048183
BBRF2	LYRM4	0.023509

BBRF2	RHAG	0.026303
BBRF2	OMP	0.026636
BBRF2	USE1	0.019158
BBRF2	LGALS9	0.014359
BBRF2	CD164	0.053794
BBRF2	AOC3	0.025084
BBRF2	IFNA16	0.023914
BBRF2	BLOC1S1	0.031392
BBRF2	FAM195A	0.058436
BBRF2	GAST	0.020249
BBRF2	TRMT11	0.016983
BBRF2	EXOC8	0.027911
BBRF2	CGGBP1	0.059676
BBRF2	TIGD1	0.045559
BBRF2	GTSF1	0.14184
BBRF2	FAM134B	0.014154
BBRF2	SCAPER	0.019617
BBRF2	Pom121l2	0.011815
BBRF2	ITFG1	0.036856
BBRF2	PNPT1	0.020952
BBRF2	UPF3B	0.086313
BBRF2	RPL30	0.022982
BBRF2	LZTFL1	0.015008
BBRF2	NUPR1	0.026825
BBRF2	ZNF668	0.010899
BBRF2	ZNF260	0.011821
BBRF2	C5orf58	0.071441
BBRF2	WFS1	0.012534
BBRF2	DHTKD1	0.011142
BBRF2	LHB	0.026359
BBRF2	MFI2	0.01494
BBRF2	CRTC3	0.028742
BBRF2	OR10Q1	0.036492
BBRF2	PMAIP1	0.011468
BBRF2	OR13C9	0.034093
BBRF2	SPINK1	0.011752
BBRF2	RPRD2	0.026742
BBRF2	PPP1R3A	0.012537
BBRF2	OR51J1	0.035661
BBRF2	DPP7	0.094876
BBRF2	CD3EAP	0.015548
BBRF2	CCDC169	0.039316
BBRF2	OVCA2	0.036977
BBRF2	SLC16A5	0.022648
BBRF2	RPP30	0.011792
BBRF2	FOPNL	0.017148
BBRF2	NBPF4	0.018081
BBRF2	MKI67	0.031517
BBRF2	LVP1a	0.013209
BBRF2	COL26A1	0.12029
BBRF2	OGFOD3	0.012597
BBRF2	CKLF	0.023886
BBRF2	TIMM23	0.014713
BBRF2	TNFAIP2	0.017179
BBRF2	ANO1	0.026888
BBRF2	MB21D2	0.020705
BBRF2	HBD	0.044846
BBRF2	ARMCX5	0.020535
BBRF2	VMAC	0.053999
BBRF2	SUPT7L	0.04866
BBRF2	C5orf64	0.010395
BBRF2	KYNU	0.01922
BBRF2	LSM11	0.064019
BBRF2	CYB561A3	0.048386
BBRF2	SNX19	0.01007
BBRF2	CLPP	0.0337
BBRF2	CHORDC1	0.0181
BBRF2	FUT1	0.012948

BBRF2	SLC51A	0.072602
BBRF2	SPATA2L	0.021969
BBRF2	ADGB	0.020989
BBRF2	LAPTM5	0.081731
BBRF2	CDCA2	0.021685
BBRF2	MGAT3	0.019997
BBRF2	ATXN1L	0.055813
BBRF2	RPS3	0.020203
BBRF2	UTP6	0.012454
BBRF2	ZNF782	0.011579
BBRF2	XKRX	0.067691
BBRF2	PHACTR3	0.034279
BBRF2	WIZ	0.01353
BBRF2	C15orf37	0.015947
BBRF2	BBS1	0.019529
BBRF2	SPIDR	0.045852
BBRF2	HMGN1	0.016457
BBRF2	ITPA	0.014575
BBRF2	ELOVL1	0.013748
BBRF2	GK2	0.039527
BBRF2	SLC16A9	0.016731
BBRF2	DARC	0.036514
BBRF2	OR5I1	0.041922
BBRF2	CSTF3	0.011718
BBRF2	OR5L2	0.03752
BBRF2	NSUN2	0.01529
BBRF2	ZNF500	0.010393
BBRF2	SLC39A7	0.011026
BBRF2	GOLGA8M	0.013529
BBRF2	LYRM9	0.051992
BBRF2	CMTM7	0.029208
BBRF2	MUC1	0.10862
BBRF2	PLN	0.035394
BBRF2	CDK5R1	0.050918
BBRF2	SUB1	0.058625
BBRF2	APOPT1	0.035855
BBRF2	HAS2	0.010234
BBRF2	OR2L3	0.035476
BBRF2	OR2M2	0.038586
BBRF2	TMEM176B	0.030937
BBRF2	CXorf40B	0.024128
BBRF2	PPP1R14B	0.015541
BBRF2	PAGE1	0.013335
BBRF2	ZNF775	0.011503
BBRF2	SYNGR2	0.064734
BBRF2	TRAPPC6A	0.074382
BBRF2	MPV17L2	0.015907
BBRF2	CDC42EP3	0.016663
BBRF2	EIF4EBP2	0.015936
BBRF2	CACNA2D3	0.054842
BBRF2	FAM168B	0.018059
BBRF2	TAS2R19	0.026913
BBRF2	ZNF329	0.010866
BBRF2	PODXL2	0.032107
BBRF2	RNASEK	0.01006
BBRF2	PDE6D	0.017623
BBRF2	GADD45A	0.061635
BBRF2	LYZL1	0.070543
BBRF2	IL32	0.041139
BBRF2	IL17RC	0.056746
BBRF2	USP54	0.024974
BBRF2	GOLGA6L19	0.019773
BBRF2	LURAP1	0.05495
BBRF2	SPINK7	0.011258
BBRF2	CMTM6	0.060268
BBRF2	TMEM248	0.075733
BBRF2	NMRAL1	0.010178
BBRF2	C5orf34	0.033314

BBRF2	GAS2L2	0.016376
BBRF2	LURAP1L	0.052916
BBRF2	XKR6	0.046097
BBRF2	RPL36AL	0.010732
BBRF2	C12orf50	0.030309
BBRF2	NPTX2	0.038136
BBRF2	C17orf102	0.039731
BBRF2	ACRV1	0.012285
BBRF2	IQCG	0.011745
BBRF2	CTAGE8	0.01372
BBRF2	KIAA1598	0.010221
BBRF2	SNX31	0.013616
BBRF2	OR2H1	0.039625
BBRF2	TMED1	0.030063
BBRF2	PDCD2L	0.032527
BBRF2	TIMP1	0.02903
BBRF2	SLC16A3	0.028961
BBRF2	GKN2	0.02585
BBRF2	B3GALTL	0.010071
BBRF2	SLC39A13	0.011053
BBRF2	MAGEL2	0.013015
BBRF2	UCMA	0.011621
BBRF2	FAM47C	0.074965
BBRF2	C10orf111	0.11112
BBRF2	NHP2L1	0.017754
BBRF2	QTRT1	0.038603
BBRF2	AMOTL1	0.043732
BBRF2	DLGAP4	0.011132
BBRF2	ZNF717	0.010726
BBRF2	GTPBP2	0.02592
BBRF2	OR2AG1	0.032661
BBRF2	CCHCR1	0.06838
BBRF2	ZAR1L	0.067935
BBRF2	TJAP1	0.046948
BBRF2	SCHIP1	0.059716
BBRF2	PAGE3	0.016722
BBRF2	LYPD6B	0.1133
BBRF2	FBXO10	0.024814
BBRF2	ZSCAN5A	0.011263
BBRF2	C17orf80	0.029769
BBRF2	GRAMD1B	0.015222
BBRF2	SLC4A7	0.013436
BBRF2	EMP3	0.020417
BBRF2	CHST11	0.011688
BBRF2	SLC16A1	0.010954
BBRF2	PARP10	0.036645
BBRF2	ALKBH4	0.027206
BBRF2	SLC4A2	0.021392
BBRF2	NYAP1	0.010054
BBRF2	PCNXL3	0.013253
BBRF2	LHPP	0.025531
BBRF2	TMEM183A	0.058755
BBRF2	HBG1	0.044846
BBRF2	MAGED2	0.014106
BBRF2	MNS1	0.092613
BBRF2	MACC1	0.033834
BBRF2	MAGEH1	0.014168
BBRF2	LCA10	0.068257
BBRF2	ATP6V1C2	0.014218
BBRF2	BRINP3	0.027756
BBRF2	STATH	0.042829
BBRF2	NOL6	0.025977
BBRF2	TRAPPC5	0.057779
BBRF2	BTG3	0.020246
BBRF2	YIPF1	0.014832
BBRF2	BRICD5	0.035851
BBRF2	PGLYRP4	0.010387
BBRF2	P4HA3	0.017124

BBRF2	CNGC19	0.093487
BBRF2	ENY2	0.033189
BBRF2	LETM2	0.046175
BBRF2	ANO3	0.034614
BBRF2	ATG4A	0.014755
BBRF2	CCDC102B	0.011239
BBRF2	CNRIP1	0.024667
BBRF2	PRPF3	0.030247
BBRF2	FGFBP2	0.022237
BBRF2	SLCO1B7	0.010739
BBRF2	ZNF33B	0.011463
BBRF2	PHYH	0.029456
BBRF2	EIF3A	0.016822
BBRF2	SAP30L	0.015702
BBRF2	PTN	0.015618
BBRF2	FAM203A	0.04241
BBRF2	C6orf223	0.040559
BBRF2	C1orf64	0.079763
BBRF2	ELMOD1	0.013537
BBRF2	MBOAT7	0.040612
BBRF2	ELP4	0.03055
BBRF2	NAA35	0.06203
BBRF2	PACRGL	0.019773
BBRF2	NIPA2	0.041785
BBRF2	ORC5	0.016065
BBRF2	OCIAD2	0.036139
BBRF2	PPP1R2P3	0.054724
BBRF2	CLU	0.016316
BBRF2	C1orf140	0.030189
BBRF2	FAM69A	0.070762
BBRF2	TAS2R20	0.025552
BBRF2	CHCHD3	0.013807
BBRF2	RIPPLY1	0.030565
BBRF2	ETNK2	0.038895
BBRF2	WIPF2	0.043208
BBRF2	OR2T27	0.030728
BBRF2	HNRNPLL	0.010036
BBRF2	ZSWIM8	0.017731
BBRF2	EID1	0.034411
BBRF2	STX6	0.022155
BBRF2	YIF1B	0.05372
BBRF2	AC3.5	0.080689
BBRF2	DDX26B	0.035554
BBRF2	TTR	0.021918
BBRF2	TGM6	0.011759
BBRF2	ITPKB	0.042284
BBRF2	APOBEC2	0.014342
BBRF2	MIS12	0.010716
BBRF2	FAM45B	0.044334
BBRF2	FAM229B	0.017706
BBRF2	C2orf81	0.080243
BBRF2	KCNJ16	0.015016
BBRF2	ECM1	0.020176
BBRF2	FAM84A	0.028348
BBRF2	TREX2	0.034556
BBRF2	DNASE1L3	0.034625
BBRF2	CABIN1	0.010444
BBRF2	ASIP	0.095963
BBRF2	PUS3	0.061291
BBRF2	CGREF1	0.022697
BBRF2	CACNA2D4	0.063012
BBRF2	AKAP8	0.041131
BBRF2	ALPK1	0.024959
BBRF2	ZNF763	0.013084
BBRF2	FAM49B	0.045135
BBRF2	ZNF767	0.032341
BBRF2	FAM199X	0.033366
BBRF2	SLC39A6	0.01372

BBRF2	NOL12	0.014948
BBRF2	TBCA	0.024406
BBRF2	VN1R4	0.010409
BBRF2	HDGFL1	0.010989
BBRF2	SMPX	0.022834
BBRF2	PAGE4	0.072818
BBRF2	FAM117A	0.029004
BBRF2	LGALS3	0.010981
BBRF2	EIF2S3L	0.012129
BBRF2	TIPRL	0.076581
BBRF2	FXYD6	0.017046
BBRF2	MILR1	0.024665
BBRF2	SPA17	0.01077
BBRF2	MT1E	0.011195
BBRF2	MRPL44	0.010312
BBRF2	BPIFB2	0.029457
BBRF2	TMEM50A	0.010488
BBRF2	FGGY	0.024995
BBRF2	SEC22C	0.031151
BBRF2	FAM63B	0.037365
BBRF2	SLC1A6	0.019194
BBRF2	COPG1	0.012056
BBRF2	OR12D3	0.030153
BBRF2	ISOC1	0.011518
BBRF2	NBPF16	0.039537
BBRF2	CADPS	0.046983
BBRF2	MARCKSL1	0.059219
BBRF2	PRAMEF14	0.082959
BBRF2	NCAPH	0.02187
BBRF2	NIPSNAP1	0.024397
BBRF2	ATG9B	0.069833
BBRF2	NKAIN4	0.01578
BBRF2	C17orf59	0.065637
BBRF2	G4	0.017675
BBRF2	DPPA2	0.030935
BBRF2	NCKAP5	0.028949
BBRF2	ADRM1	0.022284
BBRF2	FAM161B	0.079815
BBRF2	CCAR1	0.10367
BBRF2	PFDN4	0.030683
BBRF2	C19orf57	0.013604
BBRF2	C16orf93	0.039099
BBRF2	MED24	0.029384
BBRF2	MT1B	0.011424
BBRF2	C20orf62	0.017869
BBRF2	C6orf222	0.010806
BBRF2	CCDC171	0.036059
BBRF2	CCAR2	0.076161
BBRF2	PLAUR	0.031063
BBRF2	FAM178B	0.022902
BBRF2	OR2L2	0.032661
BBRF2	PUS1	0.031871
BBRF2	COLGALT1	0.027614
BBRF2	PRAF2	0.038036
BBRF2	COMMD6	0.029319
BBRF2	TAS2R14	0.012481
BBRF2	C1orf195	0.18135
BBRF2	VANGL2	0.010502
BBRF2	SNX25	0.043956
BBRF2	WFDC5	0.038139
BBRF2	HEPH	0.020733
BBRF2	CCDC11	0.045836
BBRF2	kup	0.010188
BBRF2	MUM1	0.028418
BBRF2	IL21	0.02688
BBRF2	SLC5A11	0.012624
BBRF2	NPIPA5	0.015353
BBRF2	RASIP1	0.010229

BBRF2	RBBP9	0.0854
BBRF2	CNTLN	0.026903
BBRF2	SLC34A3	0.056079
BBRF2	TRIP4	0.024187
BBRF2	LEO1	0.018584
BBRF2	CCDC22	0.071798
BBRF2	MED7	0.014881
BBRF2	OR10G3	0.040757
BBRF2	C2orf72	0.024799
BBRF2	OR5M3	0.033572
BBRF2	YBR056C-B	0.051889
BBRF2	EME1	0.019579
BBRF2	CCPG1	0.053226
BBRF2	TMSB4Y	0.02991
BBRF2	SF3A3	0.023488
BBRF2	C6orf10	0.079406
BBRF2	TRO	0.014637
BBRF2	TAS2R3	0.023592
BBRF2	ZNF652	0.013936
BBRF2	OR4F6	0.029347
BBRF2	CEP57	0.030275
BBRF2	SCIMP	0.077798
BBRF2	NDN	0.013925
BBRF2	CACNG5	0.012749
BBRF2	CEP44	0.022488
BBRF2	HEPHL1	0.018059
BBRF2	NSUN3	0.023881
BBRF2	DERL1	0.013995
BBRF2	FAM96B	0.012792
BBRF2	TRMT112	0.031055
BBRF2	PRKRIP1	0.053192
BBRF2	LUZP2	0.067474
BBRF2	PAIP2	0.010956
BBRF2	ZSWIM1	0.019912
BBRF2	MFNG	0.013224
BBRF2	YIPF2	0.033113
BBRF2	EEF1DP3	0.013373
BBRF2	SLC12A2	0.015161
BBRF2	C3orf22	0.014929
BBRF2	EXOC3L1	0.028717
BBRF2	OXR1	0.018268
BBRF2	TFB2M	0.086232
BBRF2	DKK4	0.016116
BBRF2	CYCB3-1	0.045996
BBRF2	RRP1	0.019703
BBRF2	RIC8A	0.018814
BBRF2	PPFIA3	0.010239
BBRF2	B3GNT1	0.03234
BBRF2	PHACTR2	0.01894
BBRF2	SNG1	0.052308
BBRF2	STARD4	0.0117
BBRF2	SPACA5	0.056201
BBRF2	ADSL	0.012971
BBRF2	ZNF432	0.012464
BBRF2	SYCP1	0.018709
BBRF2	PRAP1	0.040767
BBRF2	GYG2	0.033453
BBRF2	PILRA	0.032567
BBRF2	EAF1	0.10684
BBRF2	BEND3	0.010359
BBRF2	SCEL	0.017286
BBRF2	SLC4A4	0.020151
BBRF2	ZNF449	0.012594
BBRF2	LETM1	0.066534
BBRF2	TNFAIP8L1	0.050096
BBRF2	GGT6	0.017823
BBRF2	pkmyt1	0.023546
BBRF2	KCNJ18	0.013023

49777	BBRF2	ARTN	0.011448
49778	BBRF2	TMEM98	0.073004
49779	BBRF2	KCNE3	0.018657
49780	BBRF2	G4	0.014062
49781	BBRF2	ZNF282	0.014035
49782	BBRF2	PMS2CL	0.020694
49783	BBRF2	LVP1a	0.033086
49784	BBRF2	IFNA1	0.023741
49785	BBRF2	CMTM8	0.014458
49786	BBRF2	G4	0.013467
49787	BBRF2	CTU1	0.023857
49788	BBRF2	MTHFR	0.024184
49789	BBRF2	MAD2L1BP	0.027902
49790	BBRF2	PFKFB3	0.014155
49791	BBRF2	FAM231A	0.25695
49792	BBRF2	NCOA5	0.01954
49793	BBRF2	SPCS3	0.019712
49794	BBRF2	ALG1	0.059069
49795	BBRF2	C6orf229	0.012795
49796	BBRF2	FAM124A	0.13269
49797	BBRF2	TMEM181	0.049869
49798	BBRF2	SLC1A3	0.019158
49799	BBRF2	TCEB3CL2	0.015422
49800	BBRF2	CEP164	0.014701
49801	BBRF2	PATZ1	0.012624
49802	BBRF2	JMY	0.032193
49803	BBRF2	CHST6	0.029685
49804	BBRF2	AMER1	0.058203
49805	BBRF2	TRIP11	0.028457
49806	BBRF2	RTF1	0.01088
49807	BBRF2	RWDD3	0.031337
49808	BBRF2	PNRC1	0.034829
49809	BBRF2	ITIH1	0.012023
49810	BBRF2	NPTX1	0.016604
49811	BBRF2	MAL	0.013669
49812	BBRF2	OOEP	0.062866
49813	BBRF2	LGALS9C	0.014359
49814	BBRF2	FAM188A	0.01209
49815	BBRF2	ZP3	0.010965
49816	BBRF2	DUOXA2	0.039579
49817	BBRF2	MAGEB4	0.012304
49818	BBRF2	RASSF10	0.02047
49819	BBRF2	GG1	0.019368
49820	BBRF2	CCL28	0.033816
49821	BBRF2	ZNF749	0.010833
49822	BBRF2	PANK1	0.040027
49823	BBRF2	PLSCR1	0.061784
49824	BBRF2	FRRS1	0.048712
49825	BBRF2	METTL25	0.038524
49826	BBRF2	OR2M4	0.030984
49827	BBRF2	FAM63A	0.017024
49828	BBRF2	CHMP2B	0.015991
49829	BBRF2	GNPNAT1	0.069917
49830	BBRF2	SYNE4	0.019606
49831	BBRF2	BCLAF1	0.018314
49832	BBRF2	BLZF1	0.01549
49833	BBRF2	C9orf89	0.068726
49834	BBRF2	ZNF497	0.013045
49835	BBRF2	SCAMP2	0.011305
49836	BBRF2	SLC12A3	0.014484
49837	BBRF2	COPRS	0.045562
49838	BBRF2	ATG5	0.010739
49839	BBRF2	MRPL42	0.032864
49840	BBRF2	MED13	0.02066
49841	BBRF2	CAMLG	0.044599
49842	BBRF2	NKAPL	0.12647
49843	BBRF2	NCKAP5L	0.053711
49844	BBRF2	TPI1	0.011377

49845	BBRF2	SPINK6	0.012959
49846	BBRF2	HCST	0.060668
49847	BBRF2	SCGB3A1	0.058536
49848	BBRF2	SLURP1	0.047341
49849	BBRF2	CTCF	0.010453
49850	BBRF2	CCDC153	0.011397
49851	BBRF2	PRR21	0.033162
49852	BBRF2	MED12	0.035384
49853	BBRF2	NAP1L5	0.011967
49854	BBRF2	KCNMB2	0.01181
49855	BBRF2	TLDC2	0.078776
49856	BBRF2	G4	0.013628
49857	BBRF2	SHROOM1	0.020026
49858	BBRF2	TPD52L2	0.045227
49859	BBRF2	C6orf165	0.010085
49860	BBRF2	ASPSCR1	0.03201
49861	BBRF2	IFIT2	0.04223
49862	BBRF2	SCNN1B	0.016226
49863	BBRF2	GPR123	0.012641
49864	BBRF2	SMEK3P	0.021493
49865	BBRF2	MIEF2	0.066918
49866	BBRF2	CEP290	0.011374
49867	BBRF2	NIPAL3	0.041785
49868	BBRF2	RFNG	0.010498
49869	BBRF2	TMEM191B	0.019252
49870	BBRF2	PGAM4	0.018246
49871	BBRF2	VGLL1	0.010153
49872	BBRF2	RIMKLB	0.011796
49873	BBRF2	DCT	0.020219
49874	BBRF2	N4BP3	0.033902
49875	BBRF2	METTL20	0.029032
49876	BBRF2	PRPF8	0.012555
49877	BBRF2	OR4F17	0.030153
49878	BBRF2	TBC1D20	0.034421
49879	BBRF2	NSUN5P1	0.053278
49880	BBRF2	SEC16B	0.019118
49881	BBRF2	SHPK	0.03341
49882	BBRF2	TRMT10A	0.023819
49883	BBRF2	FAM24A	0.062811
49884	BBRF2	TCAP	0.054238
49885	BBRF2	AGBL1	0.023125
49886	BBRF2	APOBR	0.035244
49887	BBRF2	SRRM5	0.014871
49888	BBRF2	GPKOW	0.012567
49889	BBRF2	CRBN	0.014414
49890	BBRF2	DAG1	0.049798
49891	BBRF2	MMEL1	0.010777
49892	BBRF2	AGTRAP	0.061116
49893	BBRF2	C1orf192	0.031393
49894	BBRF2	MBOAT2	0.0151
49895	BBRF2	CSRNP3	0.050111
49896	BBRF2	HAUS5	0.025355
49897	BBRF2	POM121L1P	0.027276
49898	BBRF2	SUMF2	0.084062
49899	BBRF2	MIDN	0.013981
49900	BBRF2	FBXO6	0.013334
49901	BBRF2	RHCE	0.024266
49902	BBRF2	DMRT3	0.012228
49903	BBRF2	HRK	0.042638
49904	BBRF2	ASXL2	0.013769
49905	BBRF2	CHCHD4	0.022233
49906	BBRF2	NIPAL2	0.041785
49907	BBRF2	HAS1	0.011343
49908	BBRF2	ZSCAN20	0.010224
49909	BBRF2	TNMD	0.037172
49910	BBRF2	SCAI	0.071921
49911	BBRF2	OR2T7	0.033291
49912	BBRF2	SUCO	0.016393

BBRF2	CRYBB2	0.025717
BBRF2	NIPBL	0.016914
BBRF2	ARSH	0.010487
BBRF2	TAS2R31	0.04723
BBRF2	FAM21A	0.065637
BBRF2	BSND	0.01806
BBRF2	C19orf67	0.010833
BBRF2	C5orf48	0.07459
BBRF2	ETNK1	0.040698
BBRF2	SH3BP5L	0.030681
BBRF2	CCDC17	0.019263
BBRF2	ESRP2	0.014796
BBRF2	PILRB	0.026669
BBRF2	CHMP4C	0.030358
BBRF2	AKIRIN1	0.056925
BBRF2	RCAN1	0.020298
BBRF2	STMN3	0.018703
BBRF2	PDS5A	0.012203
BBRF2	AKAP8L	0.055272
BBRF2	SYNGR4	0.17095
BBRF2	SNF8	0.010103
BBRF2	NOP9	0.022836
BBRF2	MLC1	0.023057
BBRF2	IFI35	0.0195
BBRF2	POLR2H	0.017267
BBRF2	SSBP3	0.010509
BBRF2	RSBN1	0.041327
BBRF2	PXMP4	0.046764
BBRF2	STAG1	0.032215
BBRF2	CNOT2	0.018652
BBRF2	DMRT1	0.010338
BBRF2	SMIM9	0.070055
BBRF2	GAS2	0.011925
BBRF2	GUK1	0.043374
BBRF2	C19orf82	0.017191
BBRF2	GALK2	0.016588
BBRF2	ABHD17C	0.022958
BBRF2	OAS2	0.011652
BBRF2	TRMT61B	0.0406
BBRF2	GALNT2	0.021481
BBRF2	USB1	0.010983
BBRF2	OR2H2	0.03752
BBRF2	SNX6	0.010038
BBRF2	ATIC	0.042933
BBRF2	CDC73	0.012162
BBRF2	ATP6V1G3	0.028895
BBRF2	SECISBP2L	0.052249
BBRF2	NMI	0.01081
BBRF2	CDK5RAP3	0.02717
BBRF2	C15orf62	0.028482
BBRF2	CHMP4A	0.057869
BBRF2	CYGB	0.036239
BBRF2	TBCCD1	0.026976
BBRF2	HECA	0.041859
BBRF2	GON4L	0.031699
BBRF2	BBS2	0.01224
BBRF2	CDKN2AIPNL	0.0462
BBRF2	CHMP5	0.043518
BBRF2	GINS4	0.030307
BBRF2	PUS10	0.012596
BBRF2	PGAM2	0.018246
BBRF2	FAM92A1	0.023239
BBRF2	ATP6V1G1	0.022602
BBRF2	GOLGA6B	0.043782
BBRF2	NACA	0.017793
BBRF2	EGLN3	0.012115
BBRF2	ARMCX6	0.034533
BBRF2	AASDHPPT	0.091156

BBRF2	NBEAP1	0.011385
BBRF2	AMER3	0.05677
BBRF2	RSRC1	0.047923
BBRF2	CNNM4	0.015537
BBRF2	MBOAT4	0.049019
BBRF2	LY6G6C	0.053366
BBRF2	EIF2S3	0.011946
BBRF2	RINL	0.038374
BBRF2	RSRC2	0.03076
BBRF2	MRPL11	0.028159
BBRF2	OR10AC1P	0.04192
BBRF2	NUP205	0.034115
BBRF2	C16orf45	0.029449
BBRF2	GOLGA6L9	0.019773
BBRF2	TMSB15A	0.029111
BBRF2	ASCC2	0.066084
BBRF2	IFNK	0.029063
BBRF2	ATF4	0.022323
BBRF2	OR2T11	0.032661
BBRF2	RPUSD2	0.022181
BBRF2	ABHD17B	0.053869
BBRF2	TBCD	0.014134
BBRF2	SLPI	0.048162
BBRF2	OR2D3	0.032661
BBRF2	ESRP1	0.013921
BBRF2	FBRSL1	0.010654
BBRF2	CKMT2	0.011923
BBRF2	LYZ	0.058734
BBRF2	GPHB5	0.010374
BBRF2	FSCN1	0.040526
BBRF2	STC1	0.028914
BBRF2	MOBP	0.018951
BBRF2	C15orf60	0.028735
BBRF2	LRRC14	0.015387
BBRF2	RPL3	0.013214
BBRF2	NGB	0.045772
BBRF2	NDUFA10	0.016023
BBRF2	CHMP2A	0.013363
BBRF2	ZK1128.3	0.027542
BBRF2	TSPAN33	0.012614
BBRF2	OR2T6	0.033291
BBRF2	MGAT5	0.015599
BBRF2	LYPD3	0.024292
BBRF2	FITM2	0.018018
BBRF2	TMEM176A	0.028726
BBRF2	HEATR5B	0.084601
BBRF2	OR2J1	0.030153
BBRF2	PDCD4	0.03059
BBRF2	CTAGE15	0.01372
BBRF2	IFITM3	0.011294
BBRF2	CARHSP1	0.015301
BBRF2	CSPP1	0.0352
BBRF2	MAN1A1	0.011952
BBRF2	ADAT1	0.024309
BBRF2	POM121B	0.033738
BBRF2	SYN2	0.016759
BBRF2	RITA1	0.026937
BBRF2	FAN1	0.01484
BBRF2	OAZ3	0.037055
BBRF2	ZNF641	0.012348
BBRF2	C20orf195	0.013582
BBRF2	C19orf66	0.063763
BBRF2	NXPH1	0.034248
BBRF2	EIF4E1B	0.013566
BBRF2	HBM	0.042266
BBRF2	C4orf6	0.039545
BBRF2	M6PR	0.048865
BBRF2	MLF1	0.018483

BBRF2	GAS2L1	0.025232
BBRF2	CCDC89	0.015975
BBRF2	UNQ6125/PRO20090	0.038664
BBRF2	F13A1	0.012737
BBRF2	RPS20	0.024584
BBRF2	VIPAS39	0.037735
BBRF2	RSPH9	0.02056
BBRF2	SPATA2	0.044996
BBRF2	PCF11	0.015461
BBRF2	CT45A2	0.011107
BBRF2	PLB1	0.012423
BBRF2	PAPOLA	0.011854
BBRF2	N4BP2L1	0.024745
BBRF2	CDC42EP1	0.012277
BBRF2	SOGA1	0.031518
BBRF2	ROPN1B	0.016221
BBRF2	FAM231D	0.12411
BBRF2	ESM1	0.017317
BBRF2	ANAPC10	0.024948
BBRF2	SAC3D1	0.038437
BBRF2	SLC1A4	0.019157
BBRF2	CHMP1A	0.01041
BBRF2	GML	0.079503
BBRF2	MAGEB17	0.011104
BBRF2	TMEM99	0.028293
BBRF2	KIAA0087	0.030913
BBRF2	CD68	0.063315
BBRF2	OR4F6	0.035185
BBRF2	C11orf72	0.036859
BBRF2	PPP1R3C	0.018087
BBRF2	C9orf171	0.035306
BBRF2	TSACC	0.074378
BBRF2	IQCF3	0.018823
BBRF2	SLC16A4	0.011845
BBRF2	OR2D2	0.030153
BBRF2	ESD	0.011679
BBRF2	CCDC94	0.018855
BBRF2	BLOC1S5	0.027516
BBRF2	GNAS	0.014973
BBRF2	MYNN	0.012887
BBRF2	RBBP8NL	0.010019
BBRF2	RGAG4	0.011216
BBRF2	SEC61A2	0.023293
BBRF2	NSL1	0.021946
BBRF2	FAM74A1	0.01407
BBRF2	APOL3	0.012387
BBRF2	SLCO3A1	0.011958
BBRF2	ATP5C1	0.011706
BBRF2	C6orf106	0.073056
BBRF2	ORC6	0.015797
BBRF2	PPP1R11	0.029073
BBRF2	STON1	0.013929
BBRF2	TOB2	0.010056
BBRF2	SGSH	0.012391
BBRF2	SCG5	0.048674
BBRF2	PRAMEF10	0.017478
BBRF2	SLCO2B1	0.017318
BBRF2	GNRH1	0.032001
BBRF2	MFSD7	0.041671
BBRF2	PLSCR2	0.059776
BBRF2	AFF2	0.011508
BBRF2	TNFSF18	0.025583
BBRF2	MED4	0.025786
BBRF2	SPEF2	0.023559
BBRF2	GPI	0.016818
BBRF2	TNNI3	0.010116
BBRF2	ZNF625	0.010813
BBRF2	SPINK14	0.012441

BBRF2	IL2RA	0.029691
BBRF2	AIM1L	0.017942
BBRF2	CDR2L	0.011371
BBRF2	REEP2	0.010922
BBRF2	ZDHHC11B	0.012988
BBRF2	SNX17	0.01618
BBRF2	DFFB	0.013279
BBRF2	HPS6	0.02105
BBRF2	TP53INP2	0.052086
BBRF2	FSIP2	0.10428
BBRF2	ZNF467	0.013089
BBRF2	GTF2E2	0.080055
BBRF2	DAK	0.038978
BBRF2	ATXN3L	0.012013
BBRF2	RPL26	0.014345
BBRF2	TRMT61A	0.027895
BBRF2	FAM198A	0.015804
BBRF2	THAP4	0.020805
BBRF2	SLC14A1	0.046798
BBRF2	MINPP1	0.015708
BBRF2	SLC8B1	0.028715
BBRF2	STS	0.011738
BBRF2	SGMS2	0.011787
BBRF2	ZNF146	0.012279
BBRF2	TPD52	0.023755
BBRF2	POLDIP3	0.013227
BBRF2	INPP4B	0.012773
BBRF2	TMEM55B	0.013813
BBRF2	SLC16A8	0.042674
BBRF2	LCN12	0.010247
BBRF2	RIPPLY3	0.037877
BXRF1	ubl4ab	0.061891
BXRF1	SPTA1	0.060676
BXRF1	S100A7	0.10244
BXRF1	HSPA5	0.055248
BXRF1	LDLR	0.046048
BXRF1	RABEP1	0.027991
BXRF1	C12orf61	0.057702
BXRF1	PLSCR3	0.14185
BXRF1	RPUSD4	0.010574
BXRF1	YMR013W-A	0.016613
BXRF1	ATP5B	0.074893
BXRF1	NIN	0.12042
BXRF1	KCNIP1	0.11955
BXRF1	STIM2	0.14137
BXRF1	WIPF1	0.021731
BXRF1	SPTAN1	0.08549
BXRF1	MASP1	0.19997
BXRF1	SCUBE3	0.1761
BXRF1	PROC	0.16796
BXRF1	PDCD6	0.19391
BXRF1	S100A9	0.044665
BXRF1	ANXA2	0.086765
BXRF1	MMP20	0.042921
BXRF1	MATN4	0.18131
BXRF1	NOTCH3	0.043136
BXRF1	RNH1	0.15028
BXRF1	CLIP1	0.069485
BXRF1	AMOT	0.025578
BXRF1	FAM83F	0.016025
BXRF1	THBD	0.10609
BXRF1	MAGOH	0.011161
BXRF1	EML1	0.17052
BXRF1	EPS15	0.063465
BXRF1	PCSK2	0.040602
BXRF1	AP3S1	0.063299
BXRF1	SDF4	0.12714
BXRF1	SPACA3	0.040287

BXRF1	STAT1	0.01824
BXRF1	FAT4	0.16465
BXRF1	DTNBP1	0.011544
BXRF1	PPEF1	0.081253
BXRF1	CXorf67	0.059011
BXRF1	NELL2	0.027974
BXRF1	FAM188B2	0.052839
BXRF1	THBS1	0.017571
BXRF1	PRMT1	0.018719
BXRF1	AP1M1	0.021104
BXRF1	SYT11	0.2199
BXRF1	HSP90B1	0.091084
BXRF1	MACF1	0.11227
BXRF1	CCDC176	0.074069
BXRF1	GCH1	0.040353
BXRF1	NKD2	0.18068
BXRF1	ATP2B2	0.012311
BXRF1	FAT1	0.086003
BXRF1	S100A11	0.13575
BXRF1	CCDC137	0.02991
BXRF1	NKD1	0.12971
BXRF1	PPP2R3B	0.076341
BXRF1	ACTN4	0.072824
BXRF1	EHD1	0.050867
BXRF1	S100A13	0.1412
BXRF1	MELK	0.042099
BXRF1	S100A1	0.090666
BXRF1	UTRN	0.061447
BXRF1	SLC34A1	0.026972
BXRF1	CALB1	0.12776
BXRF1	NDEL1	0.014681
BXRF1	MASP2	0.18373
BXRF1	PARK7	0.03919
BXRF1	GAS6	0.011729
BXRF1	ANXA2R	0.04147
BXRF1	EFEMP1	0.1687
BXRF1	SPARC	0.076083
BXRF1	NOTCH4	0.040402
BXRF1	PCDH17	0.16465
BXRF1	KIAA1841	0.024102
BXRF1	REPS1	0.15195
BXRF1	LRCOL1	0.049472
BXRF1	NOTCH2	0.013912
BXRF1	MPTX1	0.019211
BXRF1	STIM1	0.074921
BXRF1	S100B	0.074751
BXRF1	TNNC1	0.15385
BXRF1	CETN2	0.13396
BXRF1	ANXA6	0.15833
BXRF1	ACTN2	0.057188
BXRF1	PRSS2	0.16015
BXRF1	PITPNM3	0.15045
BXRF1	VIL1	0.077504
BXRF1	KCNJ4	0.037221
BXRF1	CETN1	0.16934
BXRF1	C2CD4D	0.012576
BXRF1	VLDLR	0.068984
BXRF1	CDH1	0.033475
BXRF1	MAP1LC3B	0.032363
BXRF1	TTN	0.029299
BXRF1	EGF	0.055069
BXRF1	LTBP4	0.13224
BXRF1	PLSCR4	0.13767
BXRF1	RBM8A	0.011534
BXRF1	SNX9	0.079918
BXRF1	RYR2	0.030929
BXRF1	PRF1	0.068103
BXRF1	IL6ST	0.077968

BXRF1	MYO1E	0.038252
BXRF1	SETD2	0.016586
BXRF1	S100A8	0.064731
BXRF1	NID2	0.17993
BXRF1	NELL1	0.090158
BXRF1	ytzL	0.06549
BXRF1	WDR96	0.017711
BXRF1	DNER	0.10396
BXRF1	PEF1	0.17858
BXRF1	MEGF8	0.029977
BXRF1	C10orf82	0.016812
BXRF1	CHP1	0.070676
BXRF1	CFTR	0.01393
BXRF1	FASN	0.060221
BXRF1	GPR98	0.064399
BXRF1	MICU1	0.14255
BXRF1	CALU	0.14048
BXRF1	PPP3CA	0.02837
BXRF1	BEND2	0.012434
BXRF1	LRP2	0.065634
BXRF1	CAPN2	0.014836
BXRF1	FAM188B	0.063119
BXRF1	NUCB2	0.11537
BXRF1	S100A6	0.11294
BXRF1	LRP8	0.12632
BXRF1	CT55	0.046647
BXRF1	S100P	0.10876
BXRF1	PRDX6	0.019954
BXRF1	SETD1B	0.012579
BXRF1	RAB11FIP3	0.048488
BXRF1	EFCAB4B	0.12267
BXRF1	DTNA	0.081806
BXRF1	REPS2	0.18625
BXRF1	PPEF2	0.07556
BXRF1	EFEMP2	0.17709
BXRF1	ATP2A1	0.0506
BXRF1	WLS	0.020881
BXRF1	CAGE1	0.014997
BXRF1	PKD2L1	0.091495
BXRF1	EHD3	0.065287
BXRF1	THBS2	0.14532
BXRF1	CALM1	0.080237
BXRF1	EIF4E2	0.013273
BXRF1	S100A4	0.17578
BXRF1	S100A12	0.09059
BXRF1	RCN1	0.18396
BXRF1	DPP4	0.020597
BXRF1	ACTN3	0.16867
BXRF1	ATP5F1	0.022355
BXRF1	FBN1	0.16981
BXRF1	F10	0.080716
BXRF1	CAPN1	0.062024
BXRF1	CELSR3	0.097859
BXRF1	N6AMT1	0.02191
BXRF1	SIRT4	0.060609
BXRF1	TESC	0.10677
BXRF1	LSM12	0.012643
BXRF1	C12orf57	0.024274
BXRF1	SETD1A	0.011773
BXRF1	RPH3A	0.075005
BXRF1	SRI	0.085539
BXRF1	LRP4	0.092692
BXRF1	SLIT2	0.034511
BXRF1	MATN2	0.14643
BXRF1	CIB1	0.12634
BXRF1	PPP2R3A	0.1075
BXRF1	DLL1	0.14084
BXRF1	ACTN1	0.080853

BXRF1	EHD4	0.035545
BXRF1	UPF3B	0.015923
BXRF1	PRSS3	0.18734
BXRF1	MYL2	0.18038
BXRF1	EDEM1	0.086633
BXRF1	LRP1	0.04021
BXRF1	SNX19	0.10886
BXRF1	PDGFRB	0.021163
BXRF1	ITSN1	0.030205
BXRF1	DST	0.089965
BXRF1	MYL6	0.16608
BXRF1	COMP	0.12555
BXRF1	CCDC47	0.14246
BXRF1	ANXA7	0.074334
BXRF1	CHP2	0.13335
BXRF1	CDK5R1	0.025903
BXRF1	NECAB2	0.18692
BXRF1	CANX	0.078788
BXRF1	MMP15	0.028462
BXRF1	ANXA11	0.080486
BXRF1	PPP3CB	0.033371
BXRF1	IQGAP1	0.020154
BXRF1	MATN3	0.19248
BXRF1	FAM47C	0.032071
BXRF1	SH3KBP1	0.025256
BXRF1	RET	0.067414
BXRF1	CDH23	0.053446
BXRF1	RAB11FIP4	0.24259
BXRF1	CNRIP1	0.012387
BXRF1	SUV39H2	0.01709
BXRF1	C1orf64	0.03627
BXRF1	PLCE1	0.012319
BXRF1	AC3.5	0.019635
BXRF1	C2orf81	0.013897
BXRF1	CLIC4	0.062198
BXRF1	FAM49B	0.026702
BXRF1	BNIP2	0.073277
BXRF1	JAG2	0.11111
BXRF1	EHD2	0.01245
BXRF1	CADPS	0.19464
BXRF1	CCAR1	0.16154
BXRF1	C1orf195	0.077298
BXRF1	CRELD2	0.17141
BXRF1	YBR056C-B	0.01377
BXRF1	GSN	0.070638
BXRF1	SLC11A2	0.02344
BXRF1	CABP1	0.028153
BXRF1	LETM1	0.1655
BXRF1	FAM231A	0.11527
BXRF1	FAM124A	0.011763
BXRF1	FAM188A	0.13448
BXRF1	AP1B1	0.033163
BXRF1	RCN3	0.18064
BXRF1	PLSCR1	0.075118
BXRF1	SLC6A4	0.01645
BXRF1	NKAPL	0.044452
BXRF1	TLDC2	0.023151
BXRF1	LTBP1	0.17775
BXRF1	F2	0.042142
BXRF1	SYT1	0.062632
BXRF1	NSUN5P1	0.038343
BXRF1	NCS1	0.15549
BXRF1	DAG1	0.040118
BXRF1	NECAB3	0.10827
BXRF1	MLC1	0.014289
BXRF1	SNX6	0.039028
BXRF1	FAM92A1	0.010568
BXRF1	NPC1L1	0.05094

BXRF1	MEGF6	0.17787
BXRF1	CALR	0.019173
BXRF1	KCNIP2	0.061436
BXRF1	HEATR5B	0.037886
BXRF1	CD93	0.12701
BXRF1	GRIN1	0.056835
BXRF1	DHH	0.12347
BXRF1	SPHK2	0.026582
BXRF1	N4BP2L1	0.017617
BXRF1	FAM231D	0.039981
BXRF1	C11orf72	0.015699
BXRF1	MYL6B	0.13985
BXRF1	ATP6V0A1	0.025948
BXRF1	AP1G1	0.029986
BXRF1	CDHR2	0.1509
BXRF1	SNX17	0.010183
BXRF1	CDH5	0.096983
BXRF1	IL6R	0.028665
BXRF1	SYT2	0.15354
BXRF1	CAPNS1	0.040703
BXRF1	TPD52	0.17813
BVRF1	ubl4ab	0.0831
BVRF1	SPTA1	0.056526
BVRF1	S100A7	0.097562
BVRF1	HSPA5	0.052907
BVRF1	LDLR	0.043822
BVRF1	UPF3A	0.043961
BVRF1	RABEP1	0.039302
BVRF1	C12orf61	0.086722
BVRF1	PLSCR3	0.13501
BVRF1	ZC3HAV1L	0.035807
BVRF1	RPUSD4	0.024525
BVRF1	YMR013W-A	0.0539
BVRF1	ATP5B	0.072882
BVRF1	NIN	0.11428
BVRF1	KCNIP1	0.11438
BVRF1	STIM2	0.13514
BVRF1	ANGEL1	0.022138
BVRF1	WIPF1	0.040518
BVRF1	SPTAN1	0.080962
BVRF1	TNFAIP8L3	0.011933
BVRF1	MASP1	0.19395
BVRF1	SCUBE3	0.16784
BVRF1	PROC	0.16264
BVRF1	PDCD6	0.18824
BVRF1	S100A9	0.042355
BVRF1	ANXA2	0.082931
BVRF1	MMP20	0.037045
BVRF1	MATN4	0.17252
BVRF1	NOTCH3	0.038976
BVRF1	RNH1	0.14232
BVRF1	CLIP1	0.06563
BVRF1	AMOT	0.022532
BVRF1	PAGE5	0.012958
BVRF1	MESDC1	0.014693
BVRF1	FAM83F	0.051007
BVRF1	THBD	0.1011
BVRF1	RNMT	0.014896
BVRF1	EML1	0.16226
BVRF1	EPS15	0.058612
BVRF1	PCSK2	0.03505
BVRF1	AP3S1	0.057355
BVRF1	SDF4	0.12128
BVRF1	SPACA3	0.059209
BVRF1	STAT1	0.016609
BVRF1	FAT4	0.15665
BVRF1	DTNBP1	0.018471
BVRF1	PPEF1	0.073969

BVRF1	CXorf67	0.10951
BVRF1	NELL2	0.020121
BVRF1	FAM188B2	0.11167
BVRF1	NUDCD3	0.012524
BVRF1	THBS1	0.016805
BVRF1	PRMT1	0.013313
BVRF1	AP1M1	0.016021
BVRF1	TPPP	0.1112
BVRF1	SYT11	0.21348
BVRF1	CCDC150	0.010319
BVRF1	C1orf105	0.020234
BVRF1	HSP90B1	0.087643
BVRF1	MACF1	0.10773
BVRF1	CCDC176	0.11973
BVRF1	GCH1	0.037939
BVRF1	NKD2	0.17551
BVRF1	ATP2B2	0.011029
BVRF1	C19orf71	0.019794
BVRF1	SNX29P2	0.018119
BVRF1	FAT1	0.080839
BVRF1	S100A11	0.12888
BVRF1	CCDC137	0.079883
BVRF1	NKD1	0.12348
BVRF1	CROCCP3	0.010472
BVRF1	FAM47B	0.031968
BVRF1	PPP2R3B	0.069282
BVRF1	ACTN4	0.069044
BVRF1	EHD1	0.046666
BVRF1	S100A13	0.13661
BVRF1	MELK	0.037899
BVRF1	S100A1	0.084239
BVRF1	ARL14EPL	0.040229
BVRF1	UTRN	0.056107
BVRF1	SLC34A1	0.063819
BVRF1	CALB1	0.12266
BVRF1	NDEL1	0.023026
BVRF1	MASP2	0.17732
BVRF1	PARK7	0.03639
BVRF1	SLAIN1	0.014104
BVRF1	GAS6	0.011111
BVRF1	ANXA2R	0.084737
BVRF1	SPARC	0.072795
BVRF1	NOTCH4	0.037177
BVRF1	PCDH17	0.15665
BVRF1	YJR030C	0.036394
BVRF1	KIAA1841	0.056642
BVRF1	C1orf196	0.015899
BVRF1	REPS1	0.1437
BVRF1	LRCOL1	0.087725
BVRF1	NOTCH2	0.011478
BVRF1	MPTX1	0.02823
BVRF1	STIM1	0.070218
BVRF1	S100B	0.070164
BVRF1	CCDC172	0.010466
BVRF1	TNNC1	0.14762
BVRF1	CETN2	0.12998
BVRF1	ANXA6	0.15105
BVRF1	ACTN2	0.054778
BVRF1	PRSS2	0.15449
BVRF1	PITPNM3	0.1439
BVRF1	VIL1	0.074467
BVRF1	YIR021W-A	0.010675
BVRF1	COMMD9	0.024488
BVRF1	KCNJ4	0.031461
BVRF1	ANGEL2	0.010589
BVRF1	CETN1	0.16373
BVRF1	C2CD4D	0.020162
BVRF1	VLDLR	0.06682

BVRF1	CDH1	0.032208
BVRF1	MAP1LC3B	0.026954
BVRF1	TTN	0.028135
BVRF1	EGF	0.052648
BVRF1	LTBP4	0.12687
BVRF1	PLSCR4	0.13082
BVRF1	WWC2-AS2	0.013655
BVRF1	SNX9	0.075569
BVRF1	FBXL18	0.010775
BVRF1	RYR2	0.029042
BVRF1	PRF1	0.062938
BVRF1	IL6ST	0.076165
BVRF1	MYO1E	0.03534
BVRF1	SETD2	0.012934
BVRF1	S100A8	0.061858
BVRF1	NID2	0.17231
BVRF1	NELL1	0.084488
BVRF1	ytzL	0.12266
BVRF1	WDR96	0.037903
BVRF1	DNER	0.098451
BVRF1	C15orf65	0.011807
BVRF1	PEF1	0.17058
BVRF1	MEGF8	0.022366
BVRF1	C10orf82	0.035874
BVRF1	ERICH1-AS1	0.014858
BVRF1	CHP1	0.068272
BVRF1	CFTR	0.011761
BVRF1	DYDC2	0.013492
BVRF1	FASN	0.058001
BVRF1	GPR98	0.059934
BVRF1	MICU1	0.13592
BVRF1	CALU	0.13384
BVRF1	PPP3CA	0.025659
BVRF1	VWA9	0.015368
BVRF1	BEND2	0.025862
BVRF1	LRP2	0.06295
BVRF1	FAM188B	0.11727
BVRF1	NUCB2	0.10941
BVRF1	UNQ6126/PRO20091	0.021821
BVRF1	S100A6	0.10734
BVRF1	LRP8	0.12229
BVRF1	CT55	0.067929
BVRF1	S100P	0.10099
BVRF1	PRDX6	0.014846
BVRF1	CHAF1A	0.049473
BVRF1	RAB11FIP3	0.043929
BVRF1	EFCAB4B	0.11583
BVRF1	CHGB	0.17324
BVRF1	DTNA	0.075365
BVRF1	REPS2	0.17774
BVRF1	PPEF2	0.070768
BVRF1	ATP2A1	0.04836
BVRF1	WLS	0.014353
BVRF1	CAGE1	0.019761
BVRF1	LINC00205	0.016661
BVRF1	PKD2L1	0.087099
BVRF1	EHD3	0.060375
BVRF1	VWA5B2	0.022893
BVRF1	THBS2	0.1377
BVRF1	CALM1	0.077585
BVRF1	S100A4	0.16847
BVRF1	S100A12	0.08628
BVRF1	RCN1	0.1757
BVRF1	DPP4	0.017617
BVRF1	EIF4E	0.013095
BVRF1	ACTN3	0.16204
BVRF1	ATP5F1	0.023844
BVRF1	FBN1	0.16307

BVRF1	C12orf63	0.016716
BVRF1	F10	0.075731
BVRF1	CAPN1	0.05581
BVRF1	CELSR3	0.092005
BVRF1	N6AMT1	0.014262
BVRF1	SIRT4	0.053711
BVRF1	TESC	0.10298
BVRF1	LSM12	0.037463
BVRF1	C12orf57	0.041221
BVRF1	YAE1D1	0.026507
BVRF1	RPH3A	0.070295
BVRF1	SRI	0.080533
BVRF1	LRP4	0.088409
BVRF1	SLIT2	0.033283
BVRF1	C1orf145	0.010665
BVRF1	MATN2	0.13993
BVRF1	CIB1	0.12049
BVRF1	PPP2R3A	0.10087
BVRF1	DLL1	0.1374
BVRF1	EHD4	0.030632
BVRF1	UPF3B	0.041296
BVRF1	C5orf58	0.027552
BVRF1	PRSS3	0.18092
BVRF1	CCDC169	0.023353
BVRF1	MYL2	0.17502
BVRF1	EDEM1	0.08078
BVRF1	LRP1	0.037544
BVRF1	SNX19	0.10099
BVRF1	PDGFRB	0.020255
BVRF1	ITSN1	0.026429
BVRF1	C15orf37	0.010952
BVRF1	DST	0.087288
BVRF1	MYL6	0.15924
BVRF1	COMP	0.11968
BVRF1	CCDC47	0.13539
BVRF1	ANXA7	0.068297
BVRF1	CHP2	0.12692
BVRF1	CDK5R1	0.043019
BVRF1	CXorf40B	0.010986
BVRF1	NECAB2	0.17786
BVRF1	CANX	0.074934
BVRF1	MMP15	0.023018
BVRF1	ANXA11	0.07548
BVRF1	PPP3CB	0.030821
BVRF1	C5orf34	0.015747
BVRF1	C17orf102	0.01607
BVRF1	IQGAP1	0.017315
BVRF1	MATN3	0.18422
BVRF1	FAM47C	0.061965
BVRF1	SH3KBP1	0.019847
BVRF1	RET	0.065125
BVRF1	MAGED2	0.011091
BVRF1	CDH23	0.04884
BVRF1	LCA10	0.02135
BVRF1	RAB11FIP4	0.23655
BVRF1	CNGC19	0.042941
BVRF1	CNRIP1	0.017058
BVRF1	SUV39H2	0.012417
BVRF1	C1orf64	0.067348
BVRF1	AC3.5	0.052271
BVRF1	C2orf81	0.044603
BVRF1	FAM84A	0.021274
BVRF1	CLIC4	0.059124
BVRF1	FAM49B	0.038071
BVRF1	BNIP2	0.067425
BVRF1	FAM199X	0.020001
BVRF1	JAG2	0.10682
BVRF1	CADPS	0.18875

BVRF1	CCAR1	0.1551
BVRF1	C16orf93	0.019811
BVRF1	CCDC171	0.022122
BVRF1	CCAR2	0.057722
BVRF1	C1orf195	0.14612
BVRF1	CRELD2	0.16315
BVRF1	NPIPA5	0.010434
BVRF1	YBR056C-B	0.036409
BVRF1	GSN	0.067085
BVRF1	SLC11A2	0.022757
BVRF1	CABP1	0.02299
BVRF1	LETM1	0.1575
BVRF1	FAM231A	0.19852
BVRF1	FAM124A	0.065409
BVRF1	FAM188A	0.12622
BVRF1	AP1B1	0.028235
BVRF1	RCN3	0.17185
BVRF1	PLSCR1	0.071459
BVRF1	SLC6A4	0.014474
BVRF1	NKAPL	0.095849
BVRF1	TLDC2	0.050452
BVRF1	LTBP1	0.16999
BVRF1	F2	0.039708
BVRF1	SYT1	0.059892
BVRF1	NSUN5P1	0.058817
BVRF1	NCS1	0.1504
BVRF1	TCAP	0.033792
BVRF1	FBLN5	0.15239
BVRF1	DAG1	0.038051
BVRF1	C5orf48	0.042634
BVRF1	CCDC17	0.011022
BVRF1	NECAB3	0.10121
BVRF1	MLC1	0.014335
BVRF1	SNX6	0.033617
BVRF1	FAM92A1	0.018754
BVRF1	NPC1L1	0.045526
BVRF1	MEGF6	0.16936
BVRF1	CALR	0.018362
BVRF1	C16orf45	0.02134
BVRF1	KCNIP2	0.057019
BVRF1	LRRC14	0.01103
BVRF1	HEATR5B	0.068967
BVRF1	CTAGE15	0.011565
BVRF1	CD93	0.12081
BVRF1	GRIN1	0.054493
BVRF1	C19orf66	0.029804
BVRF1	DHH	0.11863
BVRF1	SPHK2	0.022484
BVRF1	N4BP2L1	0.0211
BVRF1	FAM231D	0.093312
BVRF1	C11orf72	0.024241
BVRF1	IQCF3	0.013213
BVRF1	MYL6B	0.13279
BVRF1	ATP6V0A1	0.021601
BVRF1	AP1G1	0.026148
BVRF1	CDHR2	0.14362
BVRF1	SNX17	0.013856
BVRF1	CDH5	0.091625
BVRF1	IL6R	0.026692
BVRF1	SYT2	0.14805
BVRF1	CAPNS1	0.03344
BVRF1	TPD52	0.17084
BOLF1	ubl4ab	0.04337
BOLF1	SPTA1	0.062468
BOLF1	S100A7	0.10659
BOLF1	HSPA5	0.05767
BOLF1	LDLR	0.047765
BOLF1	UPF3A	0.037738

BOLF1	RABEP1	0.034885
BOLF1	C12orf61	0.042451
BOLF1	PLSCR3	0.14767
BOLF1	ZC3HAV1L	0.027465
BOLF1	RPUSD4	0.015379
BOLF1	YMR013W-A	0.035169
BOLF1	ATP5B	0.078741
BOLF1	NIN	0.12549
BOLF1	KCNIP1	0.12468
BOLF1	STIM2	0.14743
BOLF1	ANGEL1	0.014631
BOLF1	WIPF1	0.029065
BOLF1	SPTAN1	0.088831
BOLF1	MASP1	0.20983
BOLF1	SCUBE3	0.18349
BOLF1	PROC	0.17608
BOLF1	PDCD6	0.20361
BOLF1	S100A9	0.04625
BOLF1	ANXA2	0.090348
BOLF1	MMP20	0.042916
BOLF1	MATN4	0.18879
BOLF1	NOTCH3	0.043797
BOLF1	RNH1	0.15632
BOLF1	UBAP2	0.012097
BOLF1	CLIP1	0.071873
BOLF1	AMOT	0.025705
BOLF1	PAGE5	0.012088
BOLF1	MESDC1	0.011704
BOLF1	FAM83F	0.033216
BOLF1	THBD	0.11045
BOLF1	RNMT	0.019596
BOLF1	EML1	0.17755
BOLF1	EPS15	0.065121
BOLF1	PCSK2	0.040443
BOLF1	AP3S1	0.064286
BOLF1	SDF4	0.13246
BOLF1	SPACA3	0.030054
BOLF1	STAT1	0.018456
BOLF1	FAT4	0.17142
BOLF1	DTNBP1	0.012839
BOLF1	PPEF1	0.082963
BOLF1	CXorf67	0.06466
BOLF1	NELL2	0.026258
BOLF1	FAM188B2	0.066076
BOLF1	NUDCD3	0.012203
BOLF1	THBS1	0.018108
BOLF1	PRMT1	0.017615
BOLF1	AP1M1	0.019781
BOLF1	TPPP	0.12195
BOLF1	SYT11	0.23094
BOLF1	CCDC150	0.011045
BOLF1	C1orf105	0.019186
BOLF1	HSP90B1	0.095462
BOLF1	MACF1	0.11723
BOLF1	CCDC176	0.073942
BOLF1	GCH1	0.041657
BOLF1	NKD2	0.18975
BOLF1	ATP2B2	0.012325
BOLF1	C19orf71	0.013548
BOLF1	SNX29P2	0.013412
BOLF1	FAT1	0.088973
BOLF1	S100A11	0.14114
BOLF1	CCDC137	0.055224
BOLF1	NKD1	0.13502
BOLF1	FAM47B	0.019193
BOLF1	PPP2R3B	0.077836
BOLF1	ACTN4	0.075566
BOLF1	EHD1	0.052146

BOLF1	S100A13	0.14799
BOLF1	MELK	0.04308
BOLF1	S100A1	0.09337
BOLF1	ARL14EPL	0.030007
BOLF1	UTRN	0.06276
BOLF1	SLC34A1	0.039047
BOLF1	CALB1	0.1334
BOLF1	NDEL1	0.017181
BOLF1	MASP2	0.19236
BOLF1	PARK7	0.040243
BOLF1	SLAIN1	0.01816
BOLF1	GAS6	0.011952
BOLF1	ANXA2R	0.058524
BOLF1	EFEMP1	0.17644
BOLF1	SPARC	0.079233
BOLF1	NOTCH4	0.041315
BOLF1	PCDH17	0.17142
BOLF1	YJR030C	0.026455
BOLF1	KIAA1841	0.037232
BOLF1	C1orf196	0.013279
BOLF1	REPS1	0.15778
BOLF1	LRCOL1	0.058221
BOLF1	NOTCH2	0.013469
BOLF1	MPTX1	0.014506
BOLF1	STIM1	0.077383
BOLF1	S100B	0.077247
BOLF1	TNNC1	0.16072
BOLF1	CETN2	0.14051
BOLF1	ANXA6	0.16501
BOLF1	ACTN2	0.059556
BOLF1	PRSS2	0.16761
BOLF1	PITPNM3	0.15713
BOLF1	VIL1	0.080881
BOLF1	COMMD9	0.015683
BOLF1	KCNJ4	0.036613
BOLF1	CETN1	0.17742
BOLF1	C2CD4D	0.013205
BOLF1	VLDLR	0.072209
BOLF1	CDH1	0.034779
BOLF1	MAP1LC3B	0.031714
BOLF1	TTN	0.030604
BOLF1	EGF	0.057292
BOLF1	LTBP4	0.13809
BOLF1	PLSCR4	0.14321
BOLF1	DNAJC9-AS1	0.011491
BOLF1	RBM8A	0.010735
BOLF1	WWC2-AS2	0.012079
BOLF1	SNX9	0.082738
BOLF1	RYR2	0.031868
BOLF1	PRF1	0.069918
BOLF1	IL6ST	0.082234
BOLF1	MYO1E	0.039481
BOLF1	SETD2	0.015996
BOLF1	S100A8	0.067357
BOLF1	NID2	0.18779
BOLF1	NELL1	0.09317
BOLF1	ytzL	0.081174
BOLF1	WDR96	0.023903
BOLF1	DNER	0.10794
BOLF1	PEF1	0.18625
BOLF1	MEGF8	0.02849
BOLF1	C10orf82	0.019194
BOLF1	ERICH1-AS1	0.016195
BOLF1	CHP1	0.073873
BOLF1	CFTR	0.013677
BOLF1	DYDC2	0.011529
BOLF1	FASN	0.062969
BOLF1	GPR98	0.066017

BOLF1	MICU1	0.14851
BOLF1	CALU	0.1463
BOLF1	PPP3CA	0.028818
BOLF1	VWA9	0.010913
BOLF1	BEND2	0.016695
BOLF1	LRP2	0.068406
BOLF1	CAPN2	0.013095
BOLF1	SLC23A1	0.023302
BOLF1	FAM188B	0.075863
BOLF1	NUCB2	0.11982
BOLF1	UNQ6126/PRO20091	0.015744
BOLF1	S100A6	0.1171
BOLF1	LRP8	0.1324
BOLF1	CT55	0.043519
BOLF1	S100P	0.11203
BOLF1	PRDX6	0.018744
BOLF1	CHAF1A	0.038706
BOLF1	SETD1B	0.011545
BOLF1	RAB11FIP3	0.04931
BOLF1	EFCAB4B	0.12725
BOLF1	DTNA	0.083939
BOLF1	REPS2	0.19418
BOLF1	PPEF2	0.077969
BOLF1	EFEMP2	0.18465
BOLF1	ATP2A1	0.052739
BOLF1	WLS	0.018853
BOLF1	CAGE1	0.012614
BOLF1	LINC00205	0.01418
BOLF1	PKD2L1	0.095177
BOLF1	EHD3	0.067199
BOLF1	VWA5B2	0.016317
BOLF1	THBS2	0.15094
BOLF1	CALM1	0.083985
BOLF1	EIF4E2	0.011199
BOLF1	S100A4	0.18356
BOLF1	S100A12	0.094233
BOLF1	RCN1	0.19215
BOLF1	DPP4	0.020306
BOLF1	ACTN3	0.17631
BOLF1	ATP5F1	0.021194
BOLF1	FBN1	0.17742
BOLF1	C12orf63	0.010952
BOLF1	F10	0.083361
BOLF1	CAPN1	0.062999
BOLF1	CELSR3	0.10128
BOLF1	N6AMT1	0.019855
BOLF1	SIRT4	0.061129
BOLF1	TESC	0.11169
BOLF1	LSM12	0.025818
BOLF1	C12orf57	0.018166
BOLF1	SETD1A	0.010736
BOLF1	YAE1D1	0.022541
BOLF1	RPH3A	0.077477
BOLF1	SRI	0.088549
BOLF1	LRP4	0.096489
BOLF1	SLIT2	0.035975
BOLF1	C1orf145	0.011257
BOLF1	MATN2	0.15265
BOLF1	CIB1	0.13161
BOLF1	PPP2R3A	0.11119
BOLF1	DLL1	0.14812
BOLF1	ACTN1	0.084214
BOLF1	EHD4	0.035539
BOLF1	UPF3B	0.038067
BOLF1	C5orf58	0.023676
BOLF1	PRSS3	0.1962
BOLF1	CCDC169	0.017179
BOLF1	MYL2	0.18933

BOLF1	EDEM1	0.089333
BOLF1	LRP1	0.041357
BOLF1	SNX19	0.11187
BOLF1	PDGFRB	0.02191
BOLF1	ITSN1	0.030201
BOLF1	DST	0.094214
BOLF1	MYL6	0.17346
BOLF1	COMP	0.13075
BOLF1	CCDC47	0.14821
BOLF1	ANXA7	0.076159
BOLF1	CHP2	0.1388
BOLF1	CDK5R1	0.029992
BOLF1	NECAB2	0.19465
BOLF1	CANX	0.081841
BOLF1	MMP15	0.027749
BOLF1	ANXA11	0.083172
BOLF1	PPP3CB	0.034211
BOLF1	C5orf34	0.011816
BOLF1	C17orf102	0.014274
BOLF1	IQGAP1	0.02
BOLF1	MATN3	0.20092
BOLF1	FAM47C	0.034177
BOLF1	SH3KBP1	0.024047
BOLF1	RET	0.070574
BOLF1	CDH23	0.054285
BOLF1	LCA10	0.016726
BOLF1	RAB11FIP4	0.25526
BOLF1	CNGC19	0.033558
BOLF1	CNRIP1	0.012253
BOLF1	SUV39H2	0.015972
BOLF1	C1orf64	0.042522
BOLF1	PLCE1	0.01169
BOLF1	AC3.5	0.036006
BOLF1	C2orf81	0.036268
BOLF1	FAM84A	0.010655
BOLF1	CLIC4	0.06449
BOLF1	FAM49B	0.024023
BOLF1	BNIP2	0.075117
BOLF1	FAM199X	0.014514
BOLF1	JAG2	0.11625
BOLF1	EHD2	0.010962
BOLF1	CADPS	0.20416
BOLF1	FAM161B	0.014687
BOLF1	CCAR1	0.16881
BOLF1	C16orf93	0.014832
BOLF1	CCDC171	0.017889
BOLF1	C1orf195	0.085902
BOLF1	CRELD2	0.1785
BOLF1	YBR056C-B	0.023067
BOLF1	GSN	0.073344
BOLF1	SLC11A2	0.024392
BOLF1	CABP1	0.027394
BOLF1	LETM1	0.17233
BOLF1	FAM231A	0.14705
BOLF1	FAM124A	0.058297
BOLF1	FAM188A	0.13919
BOLF1	AP1B1	0.032692
BOLF1	RCN3	0.18808
BOLF1	PLSCR1	0.078064
BOLF1	SLC6A4	0.016358
BOLF1	NKAPL	0.063085
BOLF1	TLDC2	0.040062
BOLF1	LTBP1	0.18547
BOLF1	F2	0.043479
BOLF1	SYT1	0.065258
BOLF1	NSUN5P1	0.032501
BOLF1	PI4K2A	0.033647
BOLF1	NCS1	0.16292

51001	BOLF1	TCAP	0.024778
51002	BOLF1	FBLN5	0.16568
51003	BOLF1	DAG1	0.041501
51004	BOLF1	C5orf48	0.029971
51005	BOLF1	NECAB3	0.11183
51006	BOLF1	MLC1	0.013222
51007	BOLF1	SNX6	0.038704
51008	BOLF1	SECISBP2L	0.013389
51009	BOLF1	FAM92A1	0.011511
51010	BOLF1	NPC1L1	0.051483
51011	BOLF1	MEGF6	0.18526
51012	BOLF1	CALR	0.019814
51013	BOLF1	C16orf45	0.013208
51014	BOLF1	KCNIP2	0.063156
51015	BOLF1	HEATR5B	0.0449
51016	BOLF1	CD93	0.13218
51017	BOLF1	GRIN1	0.059351
51018	BOLF1	C19orf66	0.023
51019	BOLF1	DHH	0.12913
51020	BOLF1	SPHK2	0.026322
51021	BOLF1	N4BP2L1	0.012946
51022	BOLF1	FAM231D	0.051572
51023	BOLF1	C11orf72	0.018301
51024	BOLF1	IQCF3	0.010388
51025	BOLF1	MYL6B	0.14544
51026	BOLF1	ATP6V0A1	0.025289
51027	BOLF1	AP1G1	0.029831
51028	BOLF1	CDHR2	0.1571
51029	BOLF1	CDH5	0.10055
51030	BOLF1	IL6R	0.029759
51031	BOLF1	SYT2	0.16072
51032	BOLF1	CAPNS1	0.039836
51033	BOLF1	TPD52	0.18609
51034	BILF1	ubl4ab	0.12232
51035	BILF1	PIK3CD	0.014317
51036	BILF1	SPTA1	0.073582
51037	BILF1	S100A7	0.12592
51038	BILF1	HSPA5	0.067979
51039	BILF1	LDLR	0.050338
51040	BILF1	UPF3A	0.029812
51041	BILF1	RABEP1	0.04697
51042	BILF1	C12orf61	0.12014
51043	BILF1	PLSCR3	0.15481
51044	BILF1	ZC3HAV1L	0.010341
51045	BILF1	RPUSD4	0.035125
51046	BILF1	MYH7	0.010165
51047	BILF1	SNAPIN	0.015156
51048	BILF1	YMR013W-A	0.046766
51049	BILF1	ATP5B	0.083573
51050	BILF1	NIN	0.15131
51051	BILF1	KCNIP1	0.13704
51052	BILF1	STIM2	0.1528
51053	BILF1	ANGEL1	0.018763
51054	BILF1	C15orf40	0.011321
51055	BILF1	WIPF1	0.046183
51056	BILF1	SPTAN1	0.10085
51057	BILF1	MASP1	0.23575
51058	BILF1	SCUBE3	0.21653
51059	BILF1	TH	0.010488
51060	BILF1	PROC	0.19865
51061	BILF1	PDCD6	0.21512
51062	BILF1	S100A9	0.052081
51063	BILF1	ANXA2	0.10561
51064	BILF1	MMP20	0.064154
51065	BILF1	MATN4	0.22376
51066	BILF1	NOTCH3	0.052363
51067	BILF1	RNH1	0.18849
51068	BILF1	CLIP1	0.081181

BILF1	AMOT	0.034379
BILF1	FAM83F	0.043555
BILF1	THBD	0.11555
BILF1	MAGOH	0.025488
BILF1	EML1	0.2104
BILF1	EPS15	0.078337
BILF1	PCSK2	0.05643
BILF1	PRMT8	0.020832
BILF1	AP3S1	0.080082
BILF1	SDF4	0.15592
BILF1	SPACA3	0.081264
BILF1	STAT1	0.024471
BILF1	FAT4	0.17982
BILF1	DTNBP1	0.022569
BILF1	PPEF1	0.1096
BILF1	CXorf67	0.12983
BILF1	NELL2	0.054306
BILF1	FAM188B2	0.11996
BILF1	KAT2A	0.015636
BILF1	MAP1LC3C	0.016973
BILF1	THBS1	0.0211
BILF1	BICD1	0.014792
BILF1	PRMT1	0.035292
BILF1	AP1M1	0.034394
BILF1	SYT11	0.22942
BILF1	HSP90B1	0.11128
BILF1	MACF1	0.12793
BILF1	CCDC176	0.15491
BILF1	GCH1	0.051214
BILF1	NKD2	0.20011
BILF1	ATP2B2	0.015516
BILF1	FNBP1L	0.025228
BILF1	SNX29P2	0.010075
BILF1	FAT1	0.096464
BILF1	S100A11	0.16816
BILF1	CCDC137	0.079415
BILF1	NKD1	0.15037
BILF1	FAM47B	0.012986
BILF1	PPP2R3B	0.10357
BILF1	ACTN4	0.090356
BILF1	EHD1	0.064413
BILF1	S100A13	0.16766
BILF1	MELK	0.055905
BILF1	S100A1	0.1175
BILF1	UTRN	0.077534
BILF1	SLC34A1	0.060997
BILF1	AQP1	0.01091
BILF1	STAT6	0.021471
BILF1	CALB1	0.15409
BILF1	NDEL1	0.028441
BILF1	MASP2	0.21898
BILF1	PARK7	0.050842
BILF1	GAS6	0.01425
BILF1	ANXA2R	0.091771
BILF1	EFEMP1	0.20321
BILF1	SPARC	0.092328
BILF1	NOTCH4	0.04742
BILF1	PCDH17	0.17982
BILF1	PRDX4	0.01241
BILF1	EIF3B	0.011404
BILF1	YJR030C	0.016718
BILF1	KIAA1841	0.06368
BILF1	REPS1	0.17873
BILF1	LRCOL1	0.1026
BILF1	NOTCH2	0.01975
BILF1	MPTX1	0.036183
BILF1	STIM1	0.08456
BILF1	S100B	0.094672

BILF1	TNNC1	0.18633
BILF1	TMED2	0.015084
BILF1	CETN2	0.15776
BILF1	ANXA6	0.19425
BILF1	ACTN2	0.069402
BILF1	PRSS2	0.19102
BILF1	STAT2	0.022659
BILF1	PITPNM3	0.16343
BILF1	VIL1	0.093381
BILF1	KCNJ4	0.049765
BILF1	CETN1	0.20114
BILF1	C2CD4D	0.025457
BILF1	VLDLR	0.072395
BILF1	CDH1	0.035353
BILF1	ADAMTS13	0.013825
BILF1	MAP1LC3B	0.047442
BILF1	TTN	0.035677
BILF1	ZDHHC17	0.013648
BILF1	EGF	0.059651
BILF1	LTBP4	0.15999
BILF1	PRMT2	0.020859
BILF1	PLSCR4	0.15075
BILF1	RBM8A	0.024274
BILF1	RHOT1	0.013107
BILF1	SNX9	0.09323
BILF1	RYR2	0.035102
BILF1	PRF1	0.079114
BILF1	IL6ST	0.081007
BILF1	MYO1E	0.051198
BILF1	SETD2	0.028756
BILF1	SNCA	0.010422
BILF1	S100A8	0.074282
BILF1	NID2	0.21852
BILF1	NELL1	0.11479
BILF1	ytzL	0.13646
BILF1	WDR96	0.044312
BILF1	DNER	0.11471
BILF1	EIF6	0.012221
BILF1	PEF1	0.20553
BILF1	FBXL8	0.17086
BILF1	MEGF8	0.049681
BILF1	C10orf82	0.041532
BILF1	CHP1	0.079081
BILF1	CFTR	0.019509
BILF1	FASN	0.072675
BILF1	GPR98	0.072222
BILF1	MICU1	0.15496
BILF1	CALU	0.17278
BILF1	PPP3CA	0.03693
BILF1	BEND2	0.028807
BILF1	LRP2	0.070472
BILF1	CAPN2	0.029923
BILF1	FAM188B	0.14307
BILF1	NUCB2	0.13447
BILF1	UNQ6126/PRO20091	0.012269
BILF1	S100A6	0.1292
BILF1	LRP8	0.13267
BILF1	CT55	0.096956
BILF1	S100P	0.14114
BILF1	PRDX6	0.036322
BILF1	SETD1B	0.029795
BILF1	RAB11FIP3	0.062098
BILF1	EFCAB4B	0.15383
BILF1	ALKBH8	0.020022
BILF1	CHGB	0.22367
BILF1	DTNA	0.10148
BILF1	REPS2	0.22843
BILF1	PPEF2	0.095896

BILF1	EFEMP2	0.20322
BILF1	ATP2A1	0.055507
BILF1	WLS	0.035734
BILF1	CAGE1	0.02808
BILF1	PKD2L1	0.099929
BILF1	EHD3	0.081329
BILF1	THBS2	0.1805
BILF1	CALM1	0.095401
BILF1	EIF4E2	0.033395
BILF1	S100A4	0.21339
BILF1	S100A12	0.10478
BILF1	RCN1	0.22516
BILF1	KCNJ6	0.010769
BILF1	DPP4	0.027447
BILF1	EIF4E	0.019481
BILF1	DNMT3A	0.013617
BILF1	ACTN3	0.20375
BILF1	ATP5F1	0.036735
BILF1	FBN1	0.20489
BILF1	C12orf63	0.011551
BILF1	F10	0.10195
BILF1	CAPN1	0.080569
BILF1	UCKL1	0.01859
BILF1	CELSR3	0.10968
BILF1	N6AMT1	0.044973
BILF1	SETD8	0.012465
BILF1	SIRT4	0.085606
BILF1	TESC	0.12014
BILF1	MICAL1	0.024947
BILF1	LSM12	0.034278
BILF1	C12orf57	0.053004
BILF1	SETD1A	0.028406
BILF1	RPH3A	0.089848
BILF1	SRI	0.10152
BILF1	LRP4	0.10073
BILF1	MATN2	0.17866
BILF1	CIB1	0.14589
BILF1	PPP2R3A	0.13656
BILF1	DLL1	0.14528
BILF1	ACTN1	0.093142
BILF1	EHD4	0.050642
BILF1	UPF3B	0.031568
BILF1	MYO6	0.013618
BILF1	PRSS3	0.22294
BILF1	CCDC169	0.0193
BILF1	MYL2	0.21287
BILF1	EDEM1	0.098801
BILF1	LRP1	0.045843
BILF1	ADAR	0.016474
BILF1	SNX19	0.13193
BILF1	PDGFRB	0.022609
BILF1	ITSN1	0.041154
BILF1	C15orf37	0.012633
BILF1	DST	0.093495
BILF1	MYL6	0.2015
BILF1	COMP	0.15417
BILF1	ANXA7	0.098453
BILF1	CHP2	0.15466
BILF1	MUC1	0.089121
BILF1	CDK5R1	0.054159
BILF1	NECAB2	0.23072
BILF1	CANX	0.085918
BILF1	RHOT2	0.013398
BILF1	MMP15	0.042202
BILF1	ANXA11	0.10227
BILF1	PPP3CB	0.044047
BILF1	IQGAP1	0.028665
BILF1	MATN3	0.23451

BILF1	FAM47C	0.072007
BILF1	SH3KBP1	0.039502
BILF1	RET	0.071746
BILF1	MAGED2	0.012292
BILF1	CDH23	0.061914
BILF1	RAB11FIP4	0.26632
BILF1	CNRIP1	0.025483
BILF1	SUV39H2	0.031282
BILF1	C1orf64	0.075942
BILF1	PLCE1	0.019534
BILF1	AC3.5	0.048938
BILF1	C2orf81	0.042878
BILF1	FAM84A	0.018815
BILF1	CLIC4	0.067678
BILF1	EIF4G1	0.021049
BILF1	FAM49B	0.052823
BILF1	BNIP2	0.096762
BILF1	FAM199X	0.0156
BILF1	JAG2	0.11857
BILF1	HDGFL1	0.011182
BILF1	EHD2	0.027333
BILF1	CADPS	0.21565
BILF1	CCAR1	0.19534
BILF1	CCDC171	0.013984
BILF1	CCAR2	0.079254
BILF1	C1orf195	0.16725
BILF1	CRELD2	0.21138
BILF1	NPIPA5	0.010046
BILF1	YBR056C-B	0.03482
BILF1	NBR1	0.021762
BILF1	GSN	0.087306
BILF1	SLC11A2	0.024544
BILF1	EIF4A1	0.019508
BILF1	CABP1	0.042746
BILF1	LETM1	0.18064
BILF1	PIK3CB	0.011643
BILF1	FAM231A	0.23236
BILF1	FAM124A	0.049073
BILF1	FAM188A	0.17082
BILF1	AP1B1	0.046443
BILF1	RCN3	0.22303
BILF1	PLSCR1	0.082139
BILF1	SLC6A4	0.020979
BILF1	NKAPL	0.10575
BILF1	TLDC2	0.059904
BILF1	LTBP1	0.21691
BILF1	F2	0.052568
BILF1	SYT1	0.068113
BILF1	NSUN5P1	0.078102
BILF1	NCS1	0.17361
BILF1	TCAP	0.018399
BILF1	FBLN5	0.19082
BILF1	DAG1	0.044054
BILF1	DOC2A	0.011885
BILF1	C5orf48	0.011409
BILF1	NECAB3	0.13863
BILF1	MLC1	0.02344
BILF1	SNX6	0.053708
BILF1	FAM92A1	0.020522
BILF1	NPC1L1	0.062808
BILF1	MEGF6	0.21922
BILF1	CALR	0.02311
BILF1	C16orf45	0.016888
BILF1	KCNIP2	0.075083
BILF1	LRRC14	0.010992
BILF1	PLCG1	0.011606
BILF1	HEATR5B	0.084497
BILF1	CD93	0.13892

BILF1	GRIN1	0.061447
BILF1	CLINT1	0.028715
BILF1	DHH	0.14085
BILF1	SPHK2	0.038958
BILF1	N4BP2L1	0.033284
BILF1	AP1G2	0.010045
BILF1	FAM231D	0.092206
BILF1	C11orf72	0.032224
BILF1	IQCF3	0.011605
BILF1	MYL6B	0.17331
BILF1	ATP6V0A1	0.035514
BILF1	AP1G1	0.040432
BILF1	CDHR2	0.16469
BILF1	SNX17	0.01773
BILF1	CDH5	0.10749
BILF1	IL6R	0.033996
BILF1	SYT2	0.16276
BILF1	CAPNS1	0.061398
BILF1	TPD52	0.21599
BDLF1	ubl4ab	0.026719
BDLF1	S100A7	0.014305
BDLF1	C12orf61	0.025101
BDLF1	PLSCR3	0.018604
BDLF1	ATP5B	0.034634
BDLF1	NIN	0.023213
BDLF1	KCNIP1	0.026723
BDLF1	STIM2	0.029433
BDLF1	MASP1	0.098299
BDLF1	SCUBE3	0.027638
BDLF1	PROC	0.07806
BDLF1	PDCD6	0.094189
BDLF1	ANXA2	0.019008
BDLF1	MATN4	0.023078
BDLF1	THBD	0.016076
BDLF1	EML1	0.021876
BDLF1	SDF4	0.021645
BDLF1	FAT4	0.020512
BDLF1	CXorf67	0.028468
BDLF1	FAM188B2	0.022459
BDLF1	SYT11	0.10887
BDLF1	HSP90B1	0.01939
BDLF1	MACF1	0.033183
BDLF1	CCDC176	0.040187
BDLF1	GCH1	0.013391
BDLF1	NKD2	0.090324
BDLF1	S100A11	0.012102
BDLF1	CCDC137	0.014798
BDLF1	NKD1	0.017366
BDLF1	S100A13	0.060069
BDLF1	CALB1	0.038288
BDLF1	MASP2	0.074854
BDLF1	ANXA2R	0.018694
BDLF1	EFEMP1	0.054895
BDLF1	SPARC	0.018643
BDLF1	PCDH17	0.020512
BDLF1	KIAA1841	0.01293
BDLF1	LRCOL1	0.022648
BDLF1	MPTX1	0.01008
BDLF1	TNNC1	0.042134
BDLF1	CETN2	0.064212
BDLF1	ANXA6	0.027589
BDLF1	ACTN2	0.013577
BDLF1	PRSS2	0.064138
BDLF1	PITPNM3	0.026682
BDLF1	VIL1	0.02295
BDLF1	CETN1	0.070504
BDLF1	VLDLR	0.029238
BDLF1	CDH1	0.01303

BDLF1	EGF	0.011323
BDLF1	LTBP4	0.038586
BDLF1	PLSCR4	0.014005
BDLF1	IL6ST	0.042704
BDLF1	S100A8	0.013332
BDLF1	NID2	0.046074
BDLF1	ytzL	0.024925
BDLF1	PEF1	0.034789
BDLF1	CHP1	0.029299
BDLF1	FASN	0.017489
BDLF1	MICU1	0.023308
BDLF1	CALU	0.021078
BDLF1	LRP2	0.017325
BDLF1	FAM188B	0.029648
BDLF1	NUCB2	0.010036
BDLF1	S100A6	0.021258
BDLF1	LRP8	0.053573
BDLF1	CT55	0.029143
BDLF1	REPS2	0.033227
BDLF1	EFEMP2	0.037874
BDLF1	PKD2L1	0.011149
BDLF1	CALM1	0.033523
BDLF1	S100A4	0.045561
BDLF1	S100A12	0.013435
BDLF1	RCN1	0.035658
BDLF1	ACTN3	0.05065
BDLF1	FBN1	0.052528
BDLF1	TESC	0.039981
BDLF1	C12orf57	0.011256
BDLF1	LRP4	0.014532
BDLF1	SLIT2	0.012533
BDLF1	MATN2	0.031957
BDLF1	CIB1	0.020903
BDLF1	DLL1	0.081421
BDLF1	ACTN1	0.015344
BDLF1	PRSS3	0.078657
BDLF1	MYL2	0.087599
BDLF1	DST	0.046026
BDLF1	MYL6	0.043908
BDLF1	COMP	0.020818
BDLF1	CHP2	0.017614
BDLF1	NECAB2	0.023673
BDLF1	CANX	0.01125
BDLF1	MATN3	0.045677
BDLF1	FAM47C	0.013402
BDLF1	RET	0.021349
BDLF1	RAB11FIP4	0.13719
BDLF1	C1orf64	0.01668
BDLF1	FAM49B	0.015554
BDLF1	JAG2	0.034357
BDLF1	CADPS	0.097486
BDLF1	CCAR1	0.046934
BDLF1	C1orf195	0.03266
BDLF1	CRELD2	0.022755
BDLF1	SLC11A2	0.011977
BDLF1	LETM1	0.021446
BDLF1	FAM231A	0.054203
BDLF1	RCN3	0.022361
BDLF1	PLSCR1	0.011594
BDLF1	NKAPL	0.024549
BDLF1	TLDC2	0.013636
BDLF1	LTBP1	0.039644
BDLF1	SYT1	0.013664
BDLF1	NSUN5P1	0.015141
BDLF1	NCS1	0.06697
BDLF1	FBLN5	0.0529
BDLF1	DAG1	0.010006
BDLF1	MEGF6	0.024715

BDLF1	HEATR5B	0.020893
BDLF1	CD93	0.014318
BDLF1	GRIN1	0.01443
BDLF1	DHH	0.031731
BDLF1	FAM231D	0.013105
BDLF1	MYL6B	0.012539
BDLF1	CDHR2	0.01967
BDLF1	SYT2	0.057667
BDLF1	TPD52	0.0474

gN	MACF1	0.075807
BZLF2	MMP15	0.020745
BLLF1	LRP2	0.041066
gB	CELSR3	0.023516
gM	SYT11	0.13403
gM	PDGFRB	0.013226
BILF1	PLSCR4	0.15075
BZLF2	LRP8	0.10553
gL	PDCD6	0.10738
BZLF2	DST	0.075447
gB	PLSCR1	0.02527
gN	THBD	0.064702
gL	CDH1	0.017097
gL	DPP4	0.01851
BZLF2	NPC1L1	0.039973
gL	PCDH17	0.058111
gL	DNER	0.03186
BZLF2	PEF1	0.15638
BLLF1	FAT1	0.045891
BILF1	PLCE1	0.019534
gB	EFEMP2	0.069518
LMP1	DNER	0.041096
gM	PKD2L1	0.050287
gB	PEF1	0.06838
gB	PKD2L1	0.030994
BILF1	CLINT1	0.028715
gB	PDCD6	0.10124
BHRF1	NUCB2	0.10042
BLLF1	THBD	0.062799
BZLF2	UTRN	0.051924
BARF1	MEGF6	0.21261
gM	NPC1L1	0.01801
BHRF1	CLINT1	0.015779
BLLF1	CDH23	0.02408
LMP1	LRP8	0.063919
BHRF1	SPTAN1	0.070321
gB	RAB11FIP4	0.13579
gB	RET	0.030269
gB	EDEM1	0.015289
LMP1	SPTAN1	0.035362
BARF1	DLL1	0.15917
LMP2	PLSCR4	0.07492
BHRF1	DPP4	0.01595
gB	CD93	0.041699
LMP2	NOTCH4	0.017944
gN	NUCB2	0.07258
gM	AP3S1	0.026848
BILF1	AP1M1	0.034394
LMP2	RAB11FIP4	0.15475
BZLF2	CELSR3	0.07977
BILF1	STIM2	0.1528
gL	DST	0.051166
LMP1	DLL1	0.076892
BLLF1	ATP5B	0.053498
gB	SNX9	0.025824
BLLF1	DST	0.061246
gL	MACF1	0.049095
gM	EGF	0.031456
LMP2	NOTCH3	0.016329
gM	LDLR	0.025838
BHRF1	PPP3CA	0.023935
gN	RPH3A	0.043364
LMP2	CDK5R1	0.027009
BHRF1	NPC1L1	0.039375
gB	ATP5B	0.03899
BLLF1	PLSCR3	0.082972
BILF1	EPS15	0.078337
BILF1	EHD1	0.064413

69	BILF1	PPP3CA	0.03693
70	LMP1	RET	0.033236
71	BZLF2	SLIT2	0.030771
72	gM	NOTCH4	0.018208
73	BILF1	CDH5	0.10749
74	BILF1	REPS1	0.17873
75	LMP2	PPP3CA	0.011237
76	gB	CDH1	0.015798
77	gN	SNX19	0.059051
78	BZLF2	MICU1	0.11738
79	BZLF2	SLC6A4	0.013127
80	BLLF1	ATP2A1	0.03029
81	LMP2	CAPN1	0.023186
82	BLLF1	IL6R	0.014169
83	gN	SLIT2	0.024516
84	LMP1	RPH3A	0.027497
85	gM	SYT1	0.03529
86	BARF1	PROC	0.19267
87	BLLF1	CHP2	0.082846
88	gB	SPTA1	0.010505
89	BHRF1	MEGF8	0.02054
90	BARF1	ACTN4	0.087703
91	gL	CDH5	0.028026
92	BZLF2	CHP1	0.062734
93	BHRF1	RAB11FIP4	0.2164
94	BZLF2	DTNA	0.069558
95	LMP2	IL6R	0.014423
96	LMP1	EHD3	0.018247
97	BARF1	SLIT2	0.039871
98	BARF1	NELL2	0.052769
99	BZLF2	SYT11	0.1837
100	BLLF1	AP3S1	0.026312
101	gB	FAT4	0.054338
102	gM	DST	0.055905
103	BHRF1	ATP2A1	0.042194
104	BZLF2	GRIN1	0.047393
105	gB	STIM2	0.052164
106	BZLF2	PPP3CA	0.023973
107	gB	CDH5	0.027253
108	BILF1	DPP4	0.027447
109	gN	PPP3CA	0.011928
110	LMP2	GRIN1	0.033191
111	BZLF2	LRP4	0.07655
112	BILF1	KCNIP1	0.13704
113	gM	EFEMP2	0.1051
114	BILF1	DNER	0.11471
115	BHRF1	MICU1	0.11752
116	gM	PPP3CA	0.0115
117	BLLF1	SNX19	0.056062
118	BHRF1	PKD2L1	0.075532
119	BLLF1	PCDH17	0.09592
120	BILF1	LRP4	0.10073
121	BZLF2	VLDLR	0.057915
122	gM	PRF1	0.030927
123	gB	SNX19	0.018131
124	gB	RPH3A	0.016898
125	LMP2	NPC1L1	0.01712
126	LMP2	TESC	0.068235
127	gM	PDCD6	0.12575
128	BILF1	NPC1L1	0.062808
129	LMP2	SRI	0.046549
130	LMP2	CDHR2	0.082866
131	BILF1	KCNIP2	0.075083
132	BLLF1	PRF1	0.031508
133	gL	SRI	0.021626
134	BLLF1	EHD3	0.031118
135	BILF1	FNBP1L	0.025228
136	gL	PLSCR1	0.10069

gN	EHD1	0.024367
BARF1	THBS1	0.020574
BILF1	STIM1	0.08456
gM	DLL1	0.088679
BLLF1	PLSCR1	0.044135
BILF1	MAP1LC3B	0.047442
gL	CDHR2	0.053794
gB	SYT1	0.022709
BHRF1	SYT11	0.18384
BILF1	CLIC4	0.067678
BILF1	CD93	0.13892
BHRF1	CIB1	0.10404
BHRF1	AP1M1	0.015483
BZLF2	CLIC4	0.05141
BHRF1	PDCD6	0.16198
gN	ACTN1	0.053129
LMP1	MICU1	0.0611
BARF1	MATN3	0.22743
BILF1	PRMT8	0.020832
LMP1	IL6ST	0.042827
BILF1	MICU1	0.15496
BZLF2	TESC	0.094458
BARF1	ANXA2	0.10249
BZLF2	DLL1	0.11839
BZLF2	EHD3	0.055815
gB	IL6ST	0.041779
gB	REPS1	0.045423
BHRF1	STIM1	0.061123
gB	VLDLR	0.033357
gN	KCNIP2	0.033051
LMP1	LRP2	0.030663
gN	ATP5B	0.054397
BZLF2	AP3S1	0.053102
BZLF2	EDEM1	0.070155
gM	DNER	0.055174
gM	CHP2	0.077095
gL	MICU1	0.053447
LMP1	EDEM1	0.027985
BHRF1	ATP6V0A1	0.01919
BILF1	AP1G1	0.040432
BILF1	SNX6	0.053708
BARF1	PRSS3	0.21621
BHRF1	ATP2B2	0.010006
gN	IL6R	0.01508
BILF1	CDH1	0.035353
LMP2	DTNA	0.03772
LMP1	DST	0.047744
gM	SYT2	0.090407
gN	RAB11FIP4	0.18063
BILF1	PCDH17	0.17982
gN	CDH1	0.02266
BARF1	NUCB2	0.13862
LMP1	SRI	0.033516
gM	RET	0.040386
gN	CLIC4	0.038022
LMP1	CLIC4	0.027066
BILF1	LDLR	0.050338
gN	NOTCH4	0.019468
gM	FAT4	0.089228
LMP1	JAG2	0.052621
BLLF1	JAG2	0.070176
BHRF1	EFEMP2	0.15529
LMP2	EPS15	0.029949
LMP2	MICU1	0.079321
LMP2	CHP1	0.046003
BHRF1	DTNA	0.069701
BHRF1	MMP15	0.020862
BILF1	CDH23	0.061914

BILF1	ATP2B2	0.015516
gM	PLSCR3	0.077207
BHRF1	SYT1	0.051799
BZLF2	NOTCH3	0.034265
LMP1	GPR98	0.022129
BLLF1	SYT11	0.14762
BZLF2	NOTCH2	0.010598
LMP2	CIB1	0.074814
gM	EDEM1	0.0416
BHRF1	SLIT2	0.030797
BLLF1	GRIN1	0.035199
BILF1	UTRN	0.077534
BHRF1	CAPNS1	0.031618
BARF1	GSN	0.084747
BHRF1	SNX6	0.031352
LMP1	LRP4	0.040335
BLLF1	NOTCH4	0.018239
BILF1	PLSCR1	0.082139
gB	ATP2A1	0.018089
gN	REPS1	0.093044
LMP2	PKD2L1	0.050675
BILF1	DLL1	0.14528
BLLF1	CIB1	0.079696
LMP1	CDH5	0.037613
BZLF2	DTNBP1	0.015092
LMP2	SYT1	0.035647
gL	FAT1	0.020353
gB	LRP2	0.026395
LMP1	EGF	0.025077
BILF1	PEF1	0.20553
BLLF1	PDGFRB	0.013912
gL	SYT1	0.024349
LMP2	MACF1	0.069305
BZLF2	RAB11FIP4	0.20341
LMP1	SYT2	0.074436
gM	SPTA1	0.030712
BLLF1	STIM1	0.039133
LMP2	AP3S1	0.0264
LMP1	THBD	0.045448
LMP2	ATP5B	0.049903
gL	FAT4	0.058111
BHRF1	CD93	0.10456
BLLF1	KCNIP1	0.077286
BILF1	ATP2A1	0.055507
BILF1	AP3S1	0.080082
BILF1	CAPN1	0.080569
BILF1	CIB1	0.14589
gM	MACF1	0.068245
BLLF1	RAB11FIP4	0.16765
gL	CD93	0.044365
gN	LRP1	0.021669
gL	ACTN1	0.031632
gN	LRP2	0.042069
LMP1	EPS15	0.01873
BZLF2	PLSCR1	0.062011
gL	LDLR	0.016675
BZLF2	DNER	0.085268
gN	CAPN1	0.025343
BLLF1	LRP4	0.055172
gM	SPTAN1	0.047596
gN	MICU1	0.087015
BILF1	SYT11	0.22942
BZLF2	IL6ST	0.065907
gB	EGF	0.020861
gN	CDH5	0.055955
BARF1	MATN4	0.21701
gB	MICU1	0.049654
LMP2	RET	0.041209

LMP2	ATP2A1	0.028941
LMP2	LRP1	0.019765
LMP1	REPS1	0.062293
gM	CAPN1	0.02394
BHRF1	PCDH17	0.1354
LMP2	KCNIP1	0.072051
gM	SRI	0.046449
gB	PLSCR4	0.04435
BZLF2	CDH5	0.079404
gM	EHD3	0.030897
gB	ACTN1	0.031135
BZLF2	EHD1	0.043263
BARF1	PRSS2	0.18527
LMP1	LRP1	0.013737
LMP2	EHD3	0.03077
gM	CDHR2	0.082062
BLLF1	SYT2	0.098615
BZLF2	ATP2B2	0.010141
gB	LDLR	0.01596
BZLF2	RYR2	0.025579
BILF1	DTNBP1	0.022569
BHRF1	SNX17	0.0131
gM	CELSR3	0.049575
BHRF1	CHP1	0.062693
gN	CHP2	0.085427
gN	RET	0.044735
gB	DNER	0.030615
gB	KCNIP1	0.047457
gM	IL6R	0.014218
gN	NPC1L1	0.01867
BHRF1	MAP1LC3B	0.025074
gN	TESC	0.074713
LMP1	ATP2A1	0.021908
BLLF1	PLSCR4	0.079606
BZLF2	MAP1LC3B	0.025373
BARF1	CALU	0.1676
LMP2	FAT4	0.090123
LMP2	KCNIP2	0.029968
BILF1	ZDHHC17	0.013648
gM	CHP1	0.045271
LMP2	CELSR3	0.049682
LMP2	STIM1	0.037415
BHRF1	CLIC4	0.051353
BILF1	PDCD6	0.21512
BHRF1	PDGFRB	0.017921
BILF1	PLSCR3	0.15481
BLLF1	GPR98	0.033493
gN	PDCD6	0.14066
gN	SYT1	0.038926
BHRF1	CABP1	0.02127
BHRF1	GPR98	0.052118
LMP2	SLIT2	0.022627
LMP2	RPH3A	0.03932
BARF1	MMP20	0.062311
gM	NUCB2	0.065647
LMP2	JAG2	0.065541
BILF1	SPACA3	0.081264
LMP2	PDCD6	0.12819
gM	KCNIP2	0.030485
BLLF1	SNX9	0.048163
gB	SYT11	0.10795
BZLF2	MEGF8	0.020415
LMP1	KCNIP1	0.056443
gN	SYT11	0.15004
BZLF2	DPP4	0.015916
BLLF1	EDEM1	0.043202
BHRF1	IQGAP1	0.016351
BARF1	PRF1	0.086507

341	BHRF1	CHP2	0.1164
342	BILF1	EHD2	0.027333
343	LMP1	SLIT2	0.018697
344	gN	RYR2	0.017262
345	gN	EPS15	0.032813
346	BHRF1	CAPN1	0.051868
347	gB	CDHR2	0.050305
348	gL	NUCB2	0.038228
349	BHRF1	SYT2	0.12778
350	gM	RAB11FIP3	0.02025
351	LMP1	SNX19	0.035689
352	BLLF1	RAB11FIP3	0.019505
353	gN	SNX9	0.049801
354	BZLF2	THBD	0.082419
355	BILF1	NOTCH3	0.052363
356	LMP1	CDH1	0.017421
357	BILF1	SH3KBP1	0.039502
358	BZLF2	LRP2	0.054641
359	gL	RET	0.033207
360	LMP2	SYT2	0.09181
361	BARF1	LRP8	0.14532
362	BARF1	SCUBE3	0.21
363	BZLF2	ITSN1	0.024753
364	BHRF1	LRP4	0.076639
365	BHRF1	FAT1	0.07008
366	BLLF1	SPTA1	0.0317
367	gL	CLIC4	0.022992
368	BZLF2	IQGAP1	0.016386
369	gM	CD93	0.068917
370	LMP1	EHD1	0.012247
371	BARF1	NOTCH2	0.021305
372	BZLF2	PCDH17	0.13523
373	gB	FAT1	0.020596
374	BILF1	IL6ST	0.081007
375	BZLF2	SPTAN1	0.074494
376	gM	DTNA	0.038148
377	BLLF1	EPS15	0.030878
378	BILF1	EHD4	0.050642
379	LMP1	KCNIP2	0.019503
380	BLLF1	UTRN	0.026781
381	BLLF1	CDHR2	0.088191
382	gM	RYR2	0.016047
383	BILF1	AQP1	0.01091
384	BZLF2	CAPN1	0.051735
385	LMP2	VLDLR	0.042966
386	gL	SNX9	0.025514
387	gB	JAG2	0.046462
388	BZLF2	SNX6	0.031441
389	BZLF2	MELK	0.035345
390	BHRF1	IL6ST	0.065326
391	LMP2	EHD1	0.022571
392	gL	DLL1	0.085216
393	BHRF1	DNER	0.084962
394	gL	IL6ST	0.046992
395	gN	PRF1	0.033456
396	gN	ATP2A1	0.03116
397	LMP2	PLSCR1	0.041593
398	gM	EHD1	0.022812
399	gN	DNER	0.060863
400	BHRF1	LRP8	0.10507
401	gL	STIM1	0.015688
402	LMP1	ATP5B	0.041277
403	LMP1	SYT11	0.11371
404	BILF1	GPR98	0.072222
405	BZLF2	CAPNS1	0.031463
406	BLLF1	DTNA	0.03876
407	LMP1	CD93	0.049935
408	LMP2	CDH5	0.051072

BILF1	RAB11FIP4	0.26632
BZLF2	AGTRAP	0.046254
BZLF2	CDH23	0.042696
BILF1	MAP1LC3C	0.016973
BARF1	CRELD2	0.20501
BILF1	SNX19	0.13193
gB	SRI	0.022752
BZLF2	CD93	0.092618
LMP2	DTNBP1	0.011712
LMP1	TESC	0.05552
gM	NOTCH3	0.016852
gN	AP3S1	0.028569
BHRF1	CDH5	0.079498
gN	CDHR2	0.090939
gM	GPR98	0.032141
BILF1	VLDLR	0.072395
BARF1	F2	0.051076
BHRF1	SLC6A4	0.013092
gL	TESC	0.052573
BHRF1	EGF	0.045803
BILF1	CABP1	0.042746
BHRF1	EHD4	0.028633
BLLF1	NPC1L1	0.016776
gM	ATP2A1	0.028421
gN	JAG2	0.071791
BILF1	JAG2	0.11857
gB	STIM1	0.016372
BILF1	SLC6A4	0.020979
gB	MACF1	0.04752
LMP2	PLSCR3	0.077982
BZLF2	EHD4	0.028735
LMP1	EFEMP2	0.082858
gL	SLIT2	0.017324
LMP2	SNX9	0.045705
BZLF2	SH3KBP1	0.018903
gL	ATP2A1	0.019076
gN	PLSCR1	0.04554
gL	PLSCR3	0.095618
BZLF2	CDH1	0.028221
BILF1	EFEMP2	0.20322
BHRF1	CDH1	0.026734
LMP1	PRF1	0.019246
BILF1	SYT2	0.16276
BHRF1	SPACA3	0.048758
gN	UTRN	0.028921
BARF1	COMP	0.14955
BHRF1	KCNIP1	0.10467
BZLF2	SYT2	0.12766
BZLF2	PDCD6	0.17225
gB	LRP4	0.032737
BZLF2	ACTN1	0.071004
BZLF2	CABP1	0.021746
BARF1	TTN	0.034704
BLLF1	SYT1	0.037947
BHRF1	RYR2	0.025621
BILF1	RPH3A	0.089848
BARF1	NOTCH4	0.051696
BHRF1	THBD	0.087572
BHRF1	PRAME	0.11577
BHRF1	EHD3	0.055515
LMP1	UTRN	0.01463
BARF1	ACTN2	0.067393
BLLF1	LRP8	0.083607
BHRF1	PEF1	0.15656
gN	LRP4	0.056787
BLLF1	MICU1	0.084513
gN	CIB1	0.082046
BHRF1	PLSCR1	0.063154

477	BARF1	DPP4	0.02976
478	LMP1	NOTCH4	0.011099
479	gL	LRP2	0.028597
480	gB	GPR98	0.014131
481	BILF1	SPTAN1	0.10085
482	gL	RAB11FIP4	0.14517
483	BILF1	CAPN2	0.029923
484	BILF1	FAT1	0.096464
485	gM	RAB11FIP4	0.16104
486	BARF1	MASP2	0.21237
487	LMP1	PDGFRB	0.01055
488	gM	ATP5B	0.048908
489	BZLF2	EGF	0.045811
490	gN	STIM2	0.08778
491	BILF1	MELK	0.055905
492	BILF1	THBD	0.11555
493	BZLF2	PLSCR4	0.11304
494	gL	CELSR3	0.023361
495	LMP2	SYT11	0.13671
496	BLLF1	ACTN1	0.051658
497	gL	AP3S1	0.026496
498	BLLF1	IL6ST	0.054445
499	BZLF2	EFEMP2	0.15515
500	BILF1	AP1B1	0.046443
501	BHRF1	AGTRAP	0.078733
502	gM	LRP4	0.051356
503	LMP1	MACF1	0.055014
504	BHRF1	IL6R	0.022261
505	BILF1	RHOT1	0.013107
506	LMP2	STIM2	0.080034
507	LMP2	PDGFRB	0.013018
508	LMP1	GRIN1	0.026319
509	BILF1	DST	0.093495
510	BARF1	THBS2	0.17508
511	BILF1	AP1G2	0.010045
512	BHRF1	DTNBP1	0.020993
513	gN	DTNA	0.041342
514	BILF1	EGF	0.059651
515	gL	PLSCR4	0.092134
516	LMP1	PLSCR1	0.03192
517	BHRF1	AP1B1	0.026588
518	gN	PCDH17	0.098937
519	LMP2	LDLR	0.02455
520	BHRF1	VLDLR	0.0576
521	gM	PEF1	0.10519
522	BHRF1	RPH3A	0.064526
523	BLLF1	RPH3A	0.041475
524	BILF1	PDGFRB	0.022609
525	gM	EPS15	0.030441
526	BARF1	GAS6	0.013934
527	gB	CIB1	0.046962
528	BZLF2	MACF1	0.098848
529	BZLF2	SNX9	0.069559
530	BARF1	FBLN5	0.18508
531	gN	PLSCR4	0.082232
532	gL	GRIN1	0.023894
533	BLLF1	DTNBP1	0.011568
534	gB	CHP2	0.047257
535	BLLF1	KCNIP2	0.031276
536	BHRF1	CDHR2	0.12417
537	BILF1	IQGAP1	0.028665
538	gL	CHP2	0.04771
539	BHRF1	TESC	0.094405
540	BHRF1	DLL1	0.11846
541	LMP2	PEF1	0.10653
542	BZLF2	PDGFRB	0.017985
543	LMP2	PCDH17	0.090123
544	gN	SRI	0.051011

gN	EGF	0.034616
BHRF1	JAG2	0.087033
gL	REPS1	0.044601
BZLF2	ATP2A1	0.04217
gM	SNX19	0.054102
BLLF1	CAPN1	0.022842
BILF1	DOC2A	0.011885
gL	STIM2	0.05662
BHRF1	UTRN	0.05185
gB	SLIT2	0.016783
gL	THBD	0.039219
BZLF2	CIB1	0.11058
BZLF2	PLSCR3	0.11662
BZLF2	KCNJ4	0.027961
BARF1	NID2	0.21193
gL	SYT2	0.074653
LMP1	CIB1	0.057692
gM	STIM2	0.079061
gN	VLDLR	0.047021
BILF1	ATP6V0A1	0.035514
BHRF1	ITSN1	0.023444
BHRF1	PLSCR3	0.11676
BILF1	SYT1	0.068113
BZLF2	FAT4	0.13523
LMP2	DST	0.056846
BARF1	F10	0.098947
LMP2	UTRN	0.026458
BARF1	NOTCH3	0.057127
gB	PLSCR3	0.047373
gN	SPTAN1	0.052329
LMP1	RYR2	0.011583
BHRF1	AP1G1	0.024571
BHRF1	COL17A1	0.12213
BHRF1	SRI	0.074235
BHRF1	RET	0.056448
BHRF1	LRP2	0.054279
BZLF2	ATP5B	0.066928
gN	SNX6	0.010149
gB	SPTAN1	0.025468
LMP1	AP3S1	0.013836
BLLF1	EHD1	0.022444
BARF1	SPARC	0.089614
gB	DST	0.045702
gL	LRP8	0.0665
LMP2	EFEMP2	0.10629
BHRF1	EPS15	0.054259
gM	GRIN1	0.0327
gL	CDK5R1	0.068043
LMP1	PLSCR3	0.059517
BARF1	ADAMTS13	0.013528
BILF1	PRF1	0.079114
LMP2	THBD	0.05904
gN	PKD2L1	0.055468
BILF1	TMED2	0.015084
BLLF1	REPS1	0.08973
BZLF2	SNX19	0.093003
gM	SLIT2	0.022337
BILF1	LRP1	0.045843
BHRF1	MAP1LC3C	0.012131
gL	RPH3A	0.015423
BZLF2	SRI	0.074141
LMP2	NUCB2	0.06619
BZLF2	STIM2	0.11668
gB	DLL1	0.075198
gL	CHP1	0.036382
gL	COL17A1	0.14854
gM	STIM1	0.037412
LMP1	PCDH17	0.068568

613	gN	EFEMP2	0.11673
614	BZLF2	AP1M1	0.014698
615	BLLF1	LDLR	0.026036
616	gB	PCDH17	0.054338
617	BZLF2	FAT1	0.070148
618	LMP1	FAT1	0.031213
619	BZLF2	JAG2	0.092315
620	LMP1	STIM2	0.062444
621	LMP2	PRF1	0.030463
622	gN	CD93	0.076286
623	BLLF1	RYR2	0.016584
624	BHRF1	NOTCH4	0.032605
625	gM	KCNIP1	0.071497
626	gM	ACTN1	0.048059
627	BILF1	NOTCH2	0.01975
628	BHRF1	KCNIP2	0.052418
629	gM	THBD	0.058465
630	LMP2	SPACA3	0.044303
631	BHRF1	CDK5R1	0.048113
632	gN	EHD3	0.033367
633	gM	UTRN	0.026981
634	LMP2	ACTN1	0.048539
635	BHRF1	DST	0.075412
636	LMP2	EGF	0.031789
637	BARF1	LTBP4	0.1552
638	gM	PLSCR1	0.041314
639	BILF1	EHD3	0.081329
640	BZLF2	SYT1	0.052042
641	LMP1	RAB11FIP4	0.13086
642	BLLF1	VLDLR	0.046208
643	BLLF1	CLIC4	0.036928
644	gL	SPTAN1	0.024908
645	BZLF2	CDHR2	0.12402
646	gM	LRP2	0.038086
647	LMP1	SYT1	0.027837
648	BILF1	KCNJ6	0.010769
649	gB	TESC	0.050203
650	BILF1	IL6R	0.033996
651	BILF1	SPTA1	0.073582
652	LMP2	SNX19	0.054165
653	BLLF1	PDCD6	0.13838
654	BLLF1	STIM2	0.08543
655	BARF1	EFEMP1	0.19709
656	BLLF1	SRI	0.048998
657	LMP2	FAT1	0.043709
658	BARF1	LTBP1	0.21037
659	gN	GPR98	0.034993
660	BHRF1	SH3KBP1	0.01809
661	gL	EDEM1	0.015254
662	BARF1	EFEMP2	0.20955
663	gL	PKD2L1	0.032855
664	BLLF1	NUCB2	0.07023
665	BILF1	NOTCH4	0.04742
666	BHRF1	NOTCH3	0.03411
667	BLLF1	RET	0.04375
668	LMP1	CELSR3	0.034796
669	gM	CDH5	0.050812
670	BHRF1	CDH23	0.042638
671	BLLF1	PEF1	0.11376
672	gB	THBD	0.036666
673	gM	CDH23	0.02381
674	BZLF2	CDK5R1	0.025072
675	BLLF1	EFEMP2	0.11363
676	BLLF1	MELK	0.014412
677	gB	CHP1	0.03475
678	BARF1	FBN1	0.19872
679	gM	JAG2	0.064659
680	BILF1	MACF1	0.12793

681	LMP1	SPTA1	0.020268
682	gL	JAG2	0.051034
683	LMP2	DLL1	0.090599
684	LMP1	PKD2L1	0.03885
685	BZLF2	LDLR	0.036167
686	BILF1	CDHR2	0.16469
687	BZLF2	CLINT1	0.010472
688	BLLF1	TESC	0.073207
689	BZLF2	IL6R	0.023618
690	LMP2	SPTA1	0.030226
691	BHRF1	SPTA1	0.051695
692	gN	PDGFRB	0.014267
693	gL	PRAME	0.17682
694	BILF1	SRI	0.10152
695	BARF1	MASP1	0.22863
696	LMP2	IL6ST	0.050514
697	BLLF1	CELSR3	0.052238
698	gM	FAT1	0.043613
699	BHRF1	PLSCR4	0.11318
700	BLLF1	PKD2L1	0.053789
701	BZLF2	GPR98	0.052181
702	gM	CDH1	0.020694
703	gM	TESC	0.067104
704	gL	PEF1	0.070024
705	BHRF1	CELSR3	0.079878
706	BZLF2	KCNIP2	0.052662
707	LMP2	RAB11FIP3	0.019519
708	BHRF1	MACF1	0.098949
709	LMP2	CLIC4	0.034985
710	BZLF2	PKD2L1	0.075441
711	BLLF1	CDH5	0.053942
712	BZLF2	ATP6V0A1	0.01941
713	BLLF1	CD93	0.069679
714	BLLF1	EGF	0.033712
715	LMP2	EDEM1	0.04147
716	BLLF1	MACF1	0.073975
717	gB	CLIC4	0.021732
718	BARF1	MATN2	0.1733
719	gM	MICU1	0.078458
720	gB	SYT2	0.067308
721	BILF1	PKD2L1	0.099929
722	gB	NUCB2	0.038323
723	BILF1	LRP8	0.13267
724	BHRF1	RAB11FIP3	0.040692
725	BILF1	RET	0.071746
726	LMP2	CDH23	0.023537
727	BILF1	MMP15	0.042202
728	gM	VLDLR	0.042352
729	BZLF2	RAB11FIP3	0.040753
730	BZLF2	LRP1	0.032917
731	gL	SNX19	0.015036
732	BILF1	GRIN1	0.061447
733	LMP2	RYR2	0.016089
734	LMP2	MELK	0.015873
735	BILF1	DTNA	0.10148
736	BLLF1	CDH1	0.022233
737	BLLF1	PPP3CA	0.010857
738	BZLF2	CHP2	0.11649
739	gL	LRP4	0.03507
740	BHRF1	MELK	0.033476
741	gN	DLL1	0.099263
742	BARF1	IL6R	0.036951
743	gM	CIB1	0.074019
744	BILF1	CHP1	0.079081
745	BZLF2	EPS15	0.054167
746	LMP2	GPR98	0.032063
747	gN	KCNIP1	0.07936
748	BHRF1	SNX9	0.069624

749	gM	MELK	0.015724
750	BILF1	SNX17	0.01773
751	BILF1	SNX9	0.09323
752	LMP2	LRP8	0.077459
753	BILF1	ATP5B	0.083573
754	BARF1	IL6ST	0.088582
755	BILF1	TESC	0.12014
756	gM	SNX9	0.045258
757	LMP2	CD93	0.065576
758	gN	LRP8	0.085125
759	LMP1	DTNA	0.02286
760	LMP2	CHP2	0.077866
761	gL	KCNIP1	0.048685
762	BHRF1	SNX19	0.093145
763	BLLF1	LRP1	0.020658
764	gN	DST	0.062191
765	LMP1	ACTN1	0.037835
766	LMP1	FAT4	0.068568
767	BHRF1	EHD1	0.043176
768	gL	EFEMP2	0.071243
769	gM	LRP8	0.076317
770	BLLF1	DLL1	0.097966
771	BZLF2	PRF1	0.054833
772	LMP2	SPTAN1	0.048043
773	BHRF1	ATP5B	0.066645
774	gM	RPH3A	0.039611
775	BZLF2	RPH3A	0.064785
776	BILF1	RAB11FIP3	0.062098
777	LMP2	LRP4	0.052069
778	gL	SYT11	0.12173
779	gN	FAT1	0.047839
780	gM	SNX6	0.010557
781	gM	PLSCR4	0.074276
782	gN	MELK	0.01624
783	gL	CIB1	0.047694
784	gN	PEF1	0.11698
785	LMP1	CHP1	0.037886
786	gN	SYT2	0.10069
787	gN	LDLR	0.028298
788	BZLF2	AP1B1	0.026499
789	gL	ATP5B	0.041103
790	gB	GRIN1	0.022153
791	BZLF2	RET	0.056502
792	gN	FAT4	0.098937
793	BHRF1	LDLR	0.037921
794	gB	LRP8	0.059496
795	BZLF2	REPS1	0.13192
796	gN	CHP1	0.050255
797	BZLF2	NUCB2	0.1005
798	gM	IL6ST	0.049555
799	BLLF1	DNER	0.058789
800	BHRF1	LRP1	0.032612
801	BILF1	MEGF8	0.049681
802	BILF1	MYO6	0.013618
803	LMP1	VLDLR	0.035748
804	BZLF2	NOTCH4	0.032648
805	LMP1	LDLR	0.019115
806	LMP1	PEF1	0.082612
807	gM	PCDH17	0.089228
808	BLLF1	FAT4	0.09592
809	gN	GRIN1	0.03605
810	BZLF2	KCNIP1	0.10496
811	BILF1	ACTN1	0.093142
812	BLLF1	SLIT2	0.024027
813	LMP2	LRP2	0.038354
814	BARF1	NELL1	0.11139
815	gL	EGF	0.022289
816	BHRF1	REPS1	0.13211

BHRF1	EDEM1	0.070276
gN	NOTCH3	0.017751
BILF1	RHOT2	0.013398
LMP1	CDHR2	0.063202
LMP2	CDH1	0.0209
LMP2	DNER	0.055195
BILF1	CDK5R1	0.054159
BHRF1	NOTCH2	0.010566
BILF1	NUCB2	0.13447
BHRF1	AP3S1	0.05321
LMP1	SNX9	0.034263
gN	EDEM1	0.045409
LMP1	STIM1	0.026283
BZLF2	STIM1	0.061026
LMP1	NUCB2	0.049842
BHRF1	STIM2	0.11681
BLLF1	NOTCH3	0.016158
gN	IL6ST	0.055188
BILF1	EDEM1	0.098801
gN	SPTA1	0.033367
BILF1	CHP2	0.15466
BHRF1	FAT4	0.1354
LMP2	REPS1	0.084725
BILF1	ITSN1	0.041154
LMP1	CHP2	0.059411
gM	CLIC4	0.03458
BHRF1	KCNJ4	0.02723
LMP1	CDH23	0.014603
BHRF1	PRF1	0.051856
gN	RAB11FIP3	0.021335
gL	DTNBP1	0.037458
BILF1	KCNJ4	0.049765
BARF1	EGF	0.065128
gN	CELSR3	0.054446
BZLF2	SPTA1	0.05219
BILF1	RYR2	0.035102
BHRF1	GRIN1	0.047447
BLLF1	SPTAN1	0.050597
BZLF2	AP1G1	0.024514
gM	REPS1	0.084169
LMP1	PDCD6	0.10662
gL	VLDLR	0.03705
LMP1	PLSCR4	0.056729
BILF1	LRP2	0.070472
BILF1	CAPNS1	0.061398
BLLF1	CHP1	0.049305
gM	LRP1	0.020071
BILF1	FAT4	0.17982
BILF1	CELSR3	0.10968
gL	GPR98	0.01341
gN	STIM1	0.040907
gN	PLSCR3	0.085553
gN	CDH23	0.025628

BMLF1	EPHB4	0.23216
EBNA1	PCBD2	0.09319
EBNA3	BRIP1	0.013262
BZLF1	USP11	0.1463
EBNA3	CLIC4	0.23639
BZLF1	CALML3	0.057416
EBNA1	STX12	0.12661
BMLF1	PALB2	0.32761
EBNA3	OLAH	0.070961
EBNA3	CTNND2	0.24169
LMP2	SLC11A2	0.015378
gN	MACF1	0.075807
EBNA4	FAM101A	0.15792
BBRF2	BIN2	0.011895
EBNA1	IFIT3	0.16129
EBNA-LP	CADPS	0.52394
BGLF4	OIP5	0.14775
EBNA3	YTHDC2	0.33562
BMLF1	NPHP4	0.20892
EBNA-LP	UBE2Q1	0.28278
BILF1	SCUBE3	0.21653
BGLF4	TMED1	0.13162
BMLF1	PARP12	0.029105
EBNA-LP	USP10	0.21349
EBNA-LP	COMP	0.47324
EBNA-LP	CDON	0.17263
EBNA3	AJUBA	0.079524
BZLF1	EDA	0.04922
EBNA3	C17orf59	0.18409
BXRF1	CCDC47	0.14246
EBNA1	HLA-A	0.086093
BXRF1	PPP2R3A	0.1075
EBNA6	UCKL1	0.069969
EBNA3	MYLPF	0.1743
EBNA4	CCDC122	0.013106
BZLF1	RAPGEF1	0.14314
BZLF1	CACNA1A	0.033855
BALF2	ATP5B	0.063193
BZLF1	MAP3K14	0.035502
BMLF1	DNASE1L2	0.20553
BVRF1	SLIT2	0.033283
EBNA1	RHOC	0.054058
EBNA3	INO80E	0.31322
BZLF1	RPS6KA4	0.024416
BZLF1	EXO1	0.066655
BZLF1	GCH1	0.12268
BMLF1	PPEF1	0.52231
EBNA6	C1orf196	0.075054
BGLF4	HSD17B10	0.09365
BZLF2	C17orf105	0.036458
EBNA1	TUBG1	0.085228
BGLF4	HSPB8	0.084253
EBNA-LP	ZFPM2	0.11869
EBNA-LP	TSSK2	0.16098
EBNA3	TPD52	0.62531
EBNA3	DOHH	0.25177
EBNA4	CCDC94	0.23173
EBNA1	PCDHB6	0.033158
BMLF1	MMS19	0.21105
BLLF2	RPL10	0.044083
EBNA3	TFG	0.34509
BMLF1	NR4A1	0.096679
EBNA3	ASF1A	0.2276
BMLF1	UNC119B	0.361
BGLF4	SOS1	0.066393
BLLF2	HIST1H2BL	0.019924
EBNA-LP	C1R	0.22257
EBNA3	CAPN14	0.089042

EBNA3	RAD51C	0.2219
EBNA-LP	PXDN	0.010764
EBNA-LP	SRRT	0.2552
EBNA3	CAMK1	0.10318
EBNA3	SMUG1	0.26477
EBNA3	SAMHD1	0.26802
gM	ACTN4	0.043942
BBRF2	GRAMD1C	0.01526
BNLF2b	IWS1	0.059313
EBNA6	SYT2	0.15339
EBNA3	LRRFIP1	0.22984
BLLF1	CCDC7	0.021545
EBNA1	RANBP3	0.1376
EBNA1	GAD2	0.093835
EBNA4	ATXN7L3B	0.12074
BMLF1	CDK13	0.1127
BMLF1	SNX17	0.269
EBNA3	CHD1L	0.28155
BBRF2	OR2M7	0.030153
BZLF1	PSRC1	0.12467
BMLF1	DHH	0.4177
gN	CCDC176	0.050406
EBNA4	LTBP1	0.22078
EBNA4	NPHP4	0.30339
EBNA3	PEX10	0.29607
BGLF4	MST1R	0.061445
EBNA3	ANGPTL4	0.22481
EBNA1	GSN	0.18207
BZLF1	PARVA	0.088598
BMLF1	CLVS1	0.091118
EBNA-LP	EIF2S3	0.31921
EBNA4	TESC	0.1279
BZLF1	FAF1	0.087546
EBNA3	PITPNM2	0.14421
BZLF1	AGR2	0.25227
EBNA3	NPC1	0.14409
gL	ZNRD1-AS1	0.032259
gL	WHAMMP3	0.019354
EBNA-LP	RPLP0	0.23771
BBRF2	MSMB	0.016134
EBNA1	STX18	0.13281
BZLF1	SERPINA3	0.14058
EBNA-LP	ABCD3	0.22965
BBRF2	RSBN1	0.041327
BZLF1	HSPB8	0.078376
BALF2	CALU	0.11015
BMLF1	BET1	0.2623
EBNA4	EIF4A1	0.027489
EBNA3	HRK	0.30531
BMLF1	RBM8A	0.23644
EBNA3	GAGE4	0.074468
EBNA-LP	GOLPH3	0.28823
EBNA-LP	ALDH3A1	0.019375
BMLF1	TBC1D25	0.40051
EBNA3	MBIP	0.33455
BGLF4	TAF4	0.044261
BHRF1	DEHA2C16280g	0.066556
EBNA3	ENG	0.074926
BLLF1	FAM117A	0.033944
EBNA-LP	ATCAY	0.26754
EBNA1	CHRD	0.10564
EBNA-LP	CREB3	0.049862
EBNA-LP	ADRA2B	0.1653
BZLF1	NOS3	0.020445
BGLF4	SBF2	0.12644
BZLF1	BTN3A1	0.18606
BGLF4	SEPT4	0.026121
BMLF1	EPB41	0.16316

137	EBNA6	FAM166B	0.04982
138	EBNA1	PEX5	0.080489
139	BALF2	SNX9	0.061327
140	BGLF4	UBQLN1	0.14292
141	EBNA3	CLP1	0.016547
142	BDLF1	CHP2	0.017614
143	EBNA-LP	MICALL1	0.3429
144	EBNA-LP	DCP2	0.26199
145	BXRF1	PCSK2	0.040602
146	BMLF1	MORF4L2	0.30426
147	BPLF1	C2orf88	0.016765
148	BMLF1	PRKD3	0.13683
149	EBNA-LP	RB1	0.014096
150	EBNA-LP	CCT2	0.23444
151	BHRF1	DRC1	0.050446
152	EBNA1	CALML3	0.044917
153	BMLF1	HTT	0.054334
154	BZLF1	SPEN	0.060168
155	BXRF1	MACF1	0.11227
156	EBNA4	WRNIP1	0.098723
157	EBNA1	CD80	0.12052
158	EBNA6	SPBC1539.02	0.091922
159	BLLF2	ZNF142	0.013435
160	BZLF1	MCF2	0.08397
161	BMLF1	PCLO	0.15667
162	BZLF1	IKBIP	0.1849
163	gL	TSGA10IP	0.1126
164	BHRF1	NCOA6	0.11008
165	BBRF2	TBC1D3F	0.01312
166	BZLF1	SYNJ2	0.12827
167	LMP2	PRSS3	0.12838
168	LMP2	CALR	0.012827
169	EBNA1	SPC25	0.12747
170	BZLF1	DAPK1	0.11097
171	BGLF4	IL7R	0.10973
172	gL	SPBC1539.02	0.078383
173	EBNA-LP	SRC	0.044819
174	BZLF2	C9orf153	0.025234
175	EBNA-LP	GUCA1C	0.20973
176	BZLF1	RAB11FIP2	0.10579
177	EBNA-LP	VDAC1	0.17043
178	EBNA6	CCDC74B	0.13547
179	EBNA3	CCDC94	0.10401
180	EBNA-LP	RYR3	0.092905
181	EBNA3	AFG3L2	0.11906
182	gL	FAM151B	0.04952
183	BMLF1	CBX1	0.24086
184	EBNA-LP	GNA12	0.1681
185	BGLF4	UHRF1	0.059495
186	EBNA2	PPP2R3B	0.050319
187	BILF1	SIRT4	0.085606
188	BLLF1	CHAF1A	0.06508
189	EBNA-LP	FOXO3	0.018356
190	EBNA3	ATN1	0.23083
191	EBNA3	FAM221B	0.097409
192	EBNA3	TARBP1	0.096116
193	EBNA1	MFI2	0.12147
194	EBNA-LP	RIOK3	0.31317
195	EBNA-LP	GTF3C4	0.30796
196	EBNA3	STAM	0.28591
197	EBNA1	RCN2	0.041908
198	EBNA-LP	CCNT1	0.12521
199	EBNA-LP	HID1	0.29367
200	EBNA-LP	RAMP1	0.1789
201	BZLF1	GH1	0.12133
202	EBNA-LP	AATK	0.23783
203	BZLF1	SULT2A1	0.16948
204	EBNA3	ILF3	0.23455

gL	RCN1	0.076524
BLLF1	MATN4	0.11919
BMLF1	PES1	0.25373
BGLF4	EIF3A	0.12216
BHRF1	C19orf47	0.040981
BGLF4	ADAR	0.15024
BGLF4	RPS19	0.12843
EBNA6	TEX26	0.013307
BPLF1	LRP2	0.055012
BZLF1	SP3	0.052045
BLLF2	ZNF776	0.025248
BLLF2	ETNK2	0.06001
BVRF1	MASP1	0.19395
BGLF4	TBC1D25	0.22556
BGLF4	DR1	0.12138
EBNA3	PCNT	0.27558
BZLF1	LCK	0.031351
BHRF1	NBEAP1	0.010121
BMLF1	MYEF2	0.014128
BZLF1	LRP8	0.24801
BZLF1	TIRAP	0.059663
BMLF1	SYT5	0.023462
BGLF4	BRAF	0.063972
BLLF1	ERICH1	0.024313
EBNA3	MSLN	0.32967
BLLF2	EFNA5	0.010815
EBNA3	RABEP1	0.387
BZLF1	BLOC1S6	0.089007
EBNA1	PLXDC1	0.1454
BRRF1	EHD1	0.071734
BGLF4	MKKS	0.05563
EBNA-LP	ACTL6A	0.16102
EBNA3	KREMEN1	0.37612
BMLF1	MAPK12	0.12298
BGLF4	LITAF	0.15706
BZLF2	MMP15	0.020745
EBNA1	HMOX2	0.10263
BOLF1	MICU1	0.14851
BGLF4	ASAP1	0.035445
EBNA6	TTC29	0.018143
BGLF4	IER3	0.14814
gM	WDR96	0.012692
EBNA-LP	HDAC3	0.059285
BGRF1/BDRF1	PPP3CA	0.037976
BGLF4	NOTCH1	0.013758
BGLF4	ORAI2	0.22976
BMLF1	RIPK3	0.13207
BOLF1	CHP1	0.073873
EBNA1	SLC34A1	0.17842
BLLF2	DARS	0.063524
EBNA6	LINC00523	0.2505
EBNA1	RUNX1	0.043262
BZLF1	IDE	0.02048
EBNA1	PPP1R16A	0.2091
EBNA3	CDH19	0.17459
BZLF1	PLCB4	0.010043
BMLF1	JMY	0.27719
BMLF1	ANXA4	0.22648
EBNA1	GEMIN8	0.16688
gL	C1orf140	0.13078
BZLF1	TRAF7	0.12647
BMLF1	CHRNB4	0.015934
BZLF1	ARHGEF16	0.057568
BGLF4	SON	0.15779
BLLF1	LRP2	0.041066
EBNA-LP	MAGOH	0.30692
BXRF1	KCNIP1	0.11955
EBNA-LP	DYRK1B	0.17417

BGLF4	PIAS2	0.093363
BZLF1	SCIMP	0.18026
BMLF1	METTL6	0.059751
EBNA-LP	DKK1	0.14391
EBNA4	CDKN1A	0.016214
BMLF1	PTK2	0.028832
EBNA3	IQCK	0.08945
BZLF1	GPAA1	0.10587
BMLF1	OGG1	0.13037
BMLF1	DCAF11	0.4511
BZLF1	KPNA5	0.17921
BGLF4	CLIC5	0.10675
BOLF1	ANGEL1	0.014631
BZLF1	LEO1	0.13101
EBNA6	TTC34	0.010156
BZLF1	DIRAS3	0.13271
BZLF1	SAV1	0.13423
BGLF4	ZNF675	0.093789
EBNA3	XKRX	0.14319
BHRF1	AKAP3	0.051663
BMLF1	THBD	0.39966
EBNA1	CASQ1	0.031429
EBNA3	SPATA31E1	0.27845
BBRF2	ZNF649	0.010214
BZLF1	DLC1	0.06877
BNLF2b	CNTLN	0.017723
EBNA3	RAD51	0.11318
BZLF1	SMOC2	0.034924
EBNA-LP	PPID	0.38346
BZLF1	EED	0.12333
EBNA6	MTUS2	0.095664
EBNA1	LILRA4	0.20909
BGLF4	H2AFY	0.12666
EBNA3	SLC25A4	0.15913
EBNA3	LIN37	0.4116
BBRF2	MAN1B1	0.043765
EBNA-LP	TNFRSF21	0.34062
EBNA1	KAT8	0.07966
BGLF4	DCTN1	0.13379
BLLF2	ZKSCAN5	0.020139
BMLF1	GHRHR	0.12436
EBNA1	VPS13A	0.17241
BMLF1	ACTL6A	0.18876
BZLF1	BTNL8	0.2221
BZLF1	DOCK4	0.12053
BMLF1	SUZ12	0.22374
BLLF2	CDK5R2	0.050133
EBNA1	EMILIN1	0.16215
EBNA1	ABT1	0.14554
EBNA3	EIF2B4	0.25646
BMLF1	NMNAT1	0.31771
EBNA3	GLRA1	0.11244
EBNA3	AK1	0.049973
BBRF2	CACNG5	0.012749
BMLF1	SPHKAP	0.44386
EBNA1	PKN3	0.073543
BMLF1	KCNK1	0.024444
EBNA1	VAMP1	0.19909
EBNA-LP	NHLRC1	0.23439
EBNA-LP	BRAF	0.19167
EBNA6	REPS1	0.15825
gB	CELSR3	0.023516
BGLF4	KRT6A	0.15747
EBNA3	EIF4E	0.27331
BZLF1	USP28	0.14007
EBNA3	C22orf26	0.05036
BMLF1	AK8	0.11312
gB	BEND2	0.010153

EBNA-LP	PSMB9	0.10457
BRRF1	IL6ST	0.092933
EBNA-LP	CEBPB	0.077906
EBNA3	KCND2	0.17108
EBNA4	SAP18	0.12045
EBNA3	FOXO1	0.02294
EBNA3	PIK3CG	0.10285
BVRF1	CELSR3	0.092005
BMLF1	SQSTM1	0.07271
EBNA3	CDHR1	0.13851
EBNA1	SNTA1	0.10024
EBNA-LP	NTNG1	0.34172
BLLF1	C5orf34	0.018882
EBNA-LP	EGLN3	0.20979
BZLF1	CBL	0.13905
BBRF2	RPP21	0.055462
BMLF1	HOXA10	0.18563
EBNA3	ZC3HAV1L	0.10657
EBNA2	EPS15	0.044083
BZLF1	SPAST	0.079262
EBNA-LP	TNFRSF10B	0.25799
EBNA-LP	ATP5B	0.21683
BZLF1	ACR	0.1423
EBNA-LP	F11	0.30622
EBNA-LP	BPTF	0.16162
BZLF1	GEMIN2	0.15043
BGLF4	FBXW2	0.21444
gL	HSPA5	0.022494
EBNA1	ID1	0.031865
BZLF1	SUV39H2	0.16366
EBNA-LP	RRM1	0.25736
EBNA-LP	PECAM1	0.26126
gM	SYT11	0.13403
EBNA1	BTBD1	0.18928
EBNA1	CD164	0.12703
EBNA3	ASUN	0.34037
BPLF1	PNMA6A	0.011117
EBNA-LP	BAHD1	0.25902
BZLF1	NR0B2	0.053839
BVRF1	PRDX6	0.014846
BMLF1	JAM3	0.20105
BGLF4	PRKX	0.041817
BZLF1	FEM1B	0.14855
BOLF1	LRCOL1	0.058221
BMLF1	COPS2	0.3169
gL	C1orf64	0.11416
EBNA1	CRYGC	0.16392
BZLF1	FBN1	0.3753
gM	PDGFRB	0.013226
BGLF4	HEATR1	0.15131
BMLF1	PRSS55	0.047697
EBNA3	S100A12	0.3564
EBNA3	C14orf166	0.20636
EBNA3	PUS7	0.054505
BLLF2	ZNF222	0.024306
BILF1	PLSCR4	0.15075
BLLF2	TRAPPC1	0.054557
EBNA3	ADAM9	0.079544
BBRF2	SLC39A13	0.011053
EBNA1	TRIM27	0.047059
EBNA6	NKD1	0.13321
BGLF4	TARBP1	0.014841
EBNA4	TCP10L2	0.15577
EBNA-LP	SRRM1	0.014794
EBNA1	NCOA1	0.063416
BMLF1	SMARCD1	0.29612
BZLF1	MATN3	0.45266
BOLF1	THBS1	0.018108

409	BGLF4	ABCG2	0.14334
410	BMLF1	UBA6	0.27025
411	EBNA1	SAV1	0.12558
412	BGLF4	SRSF5	0.15909
413	BMLF1	PFKFB1	0.17981
414	BGLF4	SPOCK2	0.075498
415	BMLF1	HMGB2	0.070052
416	EBNA4	ATXN1	0.1169
417	BMLF1	APEX1	0.11543
418	EBNA-LP	FAM49B	0.047777
419	EBNA3	SKAP1	0.036323
420	BMLF1	E2F3	0.17698
421	BPLF1	MAP1LC3B	0.02414
422	BMLF1	VIM	0.12755
423	EBNA6	PRR19	0.12517
424	BMLF1	MATN3	0.67106
425	BMLF1	SERPINE2	0.08684
426	BGRF1/BDRF1	GRIN1	0.067097
427	EBNA3	SLC25A5	0.24754
428	EBNA4	FAM45B	0.21358
429	EBNA4	LTBP4	0.16179
430	EBNA1	MC5R	0.14493
431	BMLF1	OPRM1	0.13074
432	BZLF1	MGP	0.042839
433	EBNA3	FST	0.21039
434	BGLF4	SLBP	0.14781
435	EBNA3	SPRED1	0.13407
436	BGLF4	FBXO4	0.16757
437	EBNA1	EXOC3	0.20107
438	EBNA1	PCDH11Y	0.038413
439	EBNA3	COA7	0.025662
440	BARF1	SETD1A	0.029191
441	LMP2	ACTN2	0.037233
442	BGLF4	FBXO44	0.23502
443	BMLF1	TRMT10B	0.059751
444	EBNA3	VAMP1	0.35036
445	EBNA1	AP3M1	0.16782
446	EBNA6	CLUAP1	0.25445
447	BBRF2	HPS6	0.02105
448	BGLF4	ABLIM1	0.14839
449	EBNA-LP	MARK4	0.18852
450	BMLF1	FES	0.032162
451	EBNA3	MAK	0.088654
452	EBNA1	ATP11A	0.10025
453	BZLF2	LRP8	0.10553
454	EBNA3	TRIP11	0.26605
455	BLLF2	GK2	0.097643
456	EBNA-LP	F2RL2	0.24137
457	EBNA3	NDUFAF3	0.33232
458	BMLF1	MPLKIP	0.35995
459	EBNA-LP	NPHP3	0.21356
460	BMLF1	SDF4	0.47313
461	gL	PDCD6	0.10738
462	EBNA-LP	SLC2A8	0.02182
463	EBNA1	MOCS3	0.092139
464	BZLF1	TGM2	0.15464
465	BMLF1	PRKD1	0.029572
466	BMLF1	EDC3	0.37066
467	BOLF1	LRP8	0.1324
468	BILF1	CHGB	0.22367
469	BLLF2	PLSCR2	0.072413
470	EBNA-LP	TFAP2B	0.051271
471	BXRF1	S100A8	0.064731
472	EBNA6	C6orf118	0.11454
473	EBNA1	IL5RA	0.19076
474	BMLF1	SEPT6	0.23257
475	EBNA1	SLC25A12	0.021398
476	BZLF1	TEAD2	0.060824

BZLF1	EWSR1	0.17622
BLLF2	SAMM50	0.031241
EBNA1	EIF2S1	0.16134
EBNA1	CARTPT	0.10217
BZLF1	CCL3	0.032906
BBRF2	KRTAP13-4	0.037638
BMLF1	TAF15	0.34089
BTRF1	MTO1	0.030616
BILF1	C16orf45	0.016888
BILF1	C2orf81	0.042878
BMLF1	TAGLN2	0.42297
BZLF2	DST	0.075447
EBNA4	MAGEB10	0.016831
BGRF1/BDRF1	GSN	0.084741
gB	PLSCR1	0.02527
EBNA3	JMJD1C	0.33313
BZLF1	ISG20L2	0.13162
BGLF4	SPARCL1	0.071853
EBNA3	NEK11	0.17911
EBNA1	H2AFY	0.098248
EBNA3	PSMC4	0.12219
EBNA3	C7orf57	0.027514
BGLF4	IRS1	0.013614
EBNA6	RABEP1	0.19779
BLLF2	MTCP1	0.02712
BMLF1	C10orf82	0.040339
EBNA-LP	ACAN	0.22985
BZLF2	C7orf57	0.018616
EBNA-LP	USF2	0.1351
EBNA-LP	MTA2	0.065711
EBNA1	FLNA	0.036183
EBNA3	DSCC1	0.28949
BLLF1	EFCAB4B	0.076031
BMLF1	MMP8	0.10509
EBNA-LP	GRM7	0.064436
EBNA3	PCDHA12	0.18006
BGLF4	SLC22A11	0.18303
EBNA1	MITD1	0.14514
BZLF1	MRPL48	0.18495
BMLF1	PKN2	0.11227
BZLF1	MAPK14	0.013104
BOLF1	CFTR	0.013677
BGLF4	DERL3	0.18334
EBNA-LP	TRIM41	0.2633
EBNA-LP	STAT5B	0.044833
EBNA3	MMP13	0.043642
BZLF1	MT3	0.033242
EBNA4	CTAGE15	0.050462
BZLF1	WWC1	0.11547
EBNA3	HOOK1	0.23066
EBNA-LP	SGTA	0.36999
EBNA-LP	ACTR8	0.29677
EBNA1	GAN	0.18262
BZLF1	ADAM17	0.02832
BMLF1	SMG8	0.3765
EBNA3	PVRL2	0.14634
EBNA3	MAPK3	0.037658
EBNA3	PGK2	0.033461
gL	SHANK2-AS3	0.040535
EBNA-LP	MAST2	0.15466
BGRF1/BDRF1	SPTAN1	0.10393
BHRF1	PAGE5	0.078876
EBNA-LP	CAPN9	0.087369
BZLF2	ATP5A1	0.017097
BZLF1	EYS	0.083093
EBNA1	SETD6	0.21853
BGLF4	DERL1	0.12817
EBNA2	CD93	0.088502

EBNA1	ARRDC3	0.20312
BMLF1	PPIP5K1	0.029278
BZLF1	ATP2C2	0.1125
BMLF1	PSMA6	0.11013
BZLF1	JMY	0.13582
BLLF2	ALKBH2	0.074084
gN	THBD	0.064702
EBNA3	ARHGAP1	0.21014
BLLF2	ZNF853	0.01517
BMLF1	USF1	0.076983
EBNA3	ECSIT	0.37024
BZLF1	TRAF2	0.0339
EBNA-LP	ZXDC	0.12714
BLLF2	NKAIN2	0.12482
EBNA6	DERL1	0.083711
BMLF1	TNKS	0.11648
EBNA-LP	ACVR1	0.047085
BZLF1	DDX17	0.13003
BMLF1	ATP6V1C1	0.26892
EBNA6	C10orf62	0.16833
BLLF1	ANXA2R	0.063102
BZLF1	EXOC8	0.06043
EBNA3	HDDC2	0.30851
BZLF1	RAB4A	0.094997
BMLF1	ENO1	0.086435
EBNA1	EIF4EBP1	0.10174
BGLF4	ZDHHC8	0.022104
BGLF4	ATG16L1	0.1897
gL	YPR022C	0.15976
BGLF4	FOXRED2	0.25993
BLLF2	MAB21L2	0.04784
EBNA3	CREB3	0.049704
EBNA1	IGHMBP2	0.022124
EBNA-LP	ADRBK1	0.065828
BHRF1	L1TD1	0.012005
EBNA2	THBS1	0.013763
EBNA3	MBD2	0.19287
BHRF1	C9orf106	0.022526
EBNA-LP	MAPK9	0.11708
BZLF1	USHBP1	0.21274
EBNA6	S100A9	0.046442
BMLF1	RGS9BP	0.010999
BGLF4	YEATS2	0.17888
BZLF1	ADAR	0.14695
EBNA3	NCF1	0.074179
BPLF1	LRP4	0.07714
EBNA3	DVL3	0.18431
EBNA3	C19orf44	0.047033
EBNA6	LRP1	0.043324
BGLF4	SMARCC1	0.13717
BHRF1	FAM127B	0.076021
BZLF1	NDUFA13	0.1013
EBNA3	CSNK2A1	0.13078
BGLF4	LTBP1	0.39648
EBNA-LP	NEUROD1	0.035154
BHRF1	CCDC28A	0.17536
EBNA-LP	IRAK2	0.085751
EBNA-LP	CCM2	0.35958
EBNA3	SENP6	0.27322
BGLF4	E2F4	0.03046
gL	CDH1	0.017097
BZLF1	COMMD8	0.23617
gL	DPP4	0.01851
EBNA-LP	PNRC2	0.2831
BPLF1	CHP1	0.059491
BMLF1	BYSL	0.32183
BMLF1	SFTPD	0.31674
EBNA1	WIBG	0.23686

613	BZLF1	KIT	0.025204
614	BMLF1	ESD	0.05379
615	EBNA1	PODXL2	0.18421
616	BBRF2	HEPH	0.020733
617	BMLF1	NOP56	0.36759
618	EBNA3	PABPN1	0.22662
619	BMLF1	CBX2	0.25652
620	EBNA-LP	EMR3	0.12231
621	BZLF1	CRKL	0.15741
622	BBRF2	CCAR1	0.10367
623	BGLF4	TXNRD2	0.21888
624	BMLF1	SUMO2	0.39136
625	EBNA6	TRAF3IP1	0.22023
626	EBNA3	YPL025C	0.048831
627	EBNA-LP	LRCH4	0.40199
628	BZLF2	C22orf26	0.073051
629	BMLF1	CAPN8	0.13733
630	EBNA1	RAD23A	0.12284
631	BBRF2	EVA1C	0.021348
632	BLLF2	HEPH	0.026573
633	BMLF1	BCL2L10	0.30211
634	BPLF1	SYT1	0.052345
635	EBNA3	CT45A2	0.038676
636	BZLF2	PNMAL1	0.031682
637	EBNA4	NUP50	0.072915
638	BZLF1	WAPAL	0.097965
639	EBNA1	P4HB	0.039279
640	EBNA1	ROR1	0.14733
641	BMLF1	SMNDC1	0.38064
642	EBNA3	NDUFS7	0.12039
643	EBNA1	SLC23A1	0.13953
644	EBNA-LP	AIMP1	0.2256
645	BZLF2	FAM106A	0.011878
646	BVRF1	DNER	0.098451
647	EBNA1	ZNF205	0.10783
648	EBNA1	ATG2A	0.22232
649	BZLF1	PRKAA2	0.015139
650	EBNA6	MZT1	0.13979
651	EBNA1	KLF13	0.080555
652	EBNA3	TYMP	0.047674
653	EBNA-LP	RCOR3	0.30542
654	EBNA-LP	DLC1	0.12831
655	BZLF1	ZNF365	0.19177
656	BXRF1	DST	0.089965
657	BMLF1	TNNC2	0.21923
658	BMLF1	ZNF136	0.25992
659	EBNA1	DLGAP3	0.14194
660	EBNA-LP	CABP4	0.15762
661	EBNA6	TCAP	0.11283
662	BLLF2	GIF	0.10729
663	EBNA6	CXorf40B	0.051135
664	BRRF1	PIK3CB	0.01326
665	BZLF1	MLST8	0.16388
666	BZLF1	DYNLRB1	0.1502
667	EBNA-LP	PATL2	0.34009
668	BGLF4	COL6A2	0.14426
669	BMLF1	CCP110	0.34639
670	EBNA-LP	TRIM16	0.21497
671	BLLF2	ZNF334	0.024343
672	EBNA1	DLX4	0.051056
673	BHRF1	WDR96	0.054969
674	BMLF1	ARHGAP10	0.18392
675	EBNA-LP	MEGF8	0.52654
676	EBNA1	SETD1B	0.19756
677	BMLF1	NAIP	0.29802
678	BGLF4	SNX8	0.18473
679	EBNA3	TIMM9	0.23026
680	EBNA-LP	YOD1	0.25251

681	EBNA3	FAT2	0.1413
682	EBNA3	C18orf25	0.05625
683	EBNA1	C1QB	0.17538
684	EBNA4	LSM12	0.18144
685	EBNA-LP	ANGPT2	0.1141
686	BZLF1	PCDHGA1	0.049103
687	BMLF1	MCM10	0.31041
688	gL	CHGB	0.26062
689	EBNA-LP	SLC1A4	0.04496
690	EBNA1	RUVBL2	0.048799
691	BBRF2	EMP3	0.020417
692	BMLF1	TMEM120B	0.025567
693	BMLF1	CLTA	0.051858
694	BGLF4	MTDH	0.08567
695	EBNA3	DLGAP5	0.24629
696	BZLF1	PPP3R2	0.057416
697	EBNA3	USHBP1	0.40373
698	BMLF1	CASP1	0.12609
699	BZLF1	FBXL5	0.16134
700	EBNA3	UBQLN4	0.29965
701	EBNA4	GOLGA6L19	0.23368
702	EBNA1	STXBP4	0.014942
703	EBNA-LP	S100Z	0.2099
704	EBNA-LP	MC1R	0.17241
705	BHRF1	FBN1	0.15897
706	BZLF2	NPC1L1	0.039973
707	BZLF2	TIFAB	0.095871
708	EBNA3	FCHO2	0.29003
709	BLLF2	PMM1	0.017753
710	EBNA-LP	NUP43	0.21047
711	EBNA3	RBM4	0.15611
712	EBNA-LP	IL7R	0.19322
713	BZLF1	EPB41L3	0.083063
714	gM	ACTN2	0.036825
715	BHRF1	FAM194B	0.013968
716	BHRF1	C22orf26	0.092804
717	BHRF1	LDOC1L	0.036388
718	EBNA3	YWHAQ	0.36228
719	EBNA1	DCAF7	0.17027
720	EBNA3	CACNA1A	0.058978
721	BMLF1	TRDMT1	0.078211
722	BZLF1	PRMT8	0.19409
723	BGLF4	ELOVL5	0.13396
724	BMLF1	EIF6	0.4105
725	BMLF1	NAALAD2	0.025792
726	EBNA1	DSG1	0.021224
727	BLLF1	KIAA1958	0.022071
728	EBNA3	SPRR1A	0.23533
729	BZLF1	WASL	0.051539
730	gL	PCDH17	0.058111
731	EBNA3	SMN1	0.33496
732	EBNA3	MDC1	0.29798
733	BZLF2	C3orf22	0.053038
734	EBNA-LP	SDHAF2	0.37141
735	EBNA1	CBX2	0.11254
736	BMLF1	ATP6V0A2	0.24497
737	EBNA-LP	CDC14A	0.079569
738	BMLF1	PRPS1L1	0.010114
739	BZLF1	EIF4E2	0.27193
740	BGLF4	IPO13	0.18243
741	BGLF4	SSX2	0.19341
742	EBNA1	CSNK1D	0.042875
743	BGRF1/BDRF1	AC3.5	0.016223
744	BLLF2	ZMYM6	0.031932
745	EBNA3	ZNF274	0.13047
746	EBNA6	TLDC2	0.14339
747	BMLF1	ZHX3	0.1742
748	EBNA-LP	MCF2	0.19323

749	BGLF4	SS18	0.14347
750	BGLF4	ROBO2	0.097611
751	gL	DNER	0.03186
752	BXRF1	FAM83F	0.016025
753	EBNA3	NCLN	0.26142
754	EBNA1	AP5B1	0.20073
755	EBNA3	TNFAIP3	0.037348
756	BMLF1	TADA3	0.14382
757	BZLF1	PCDHGA7	0.049103
758	BDLF1	CETN2	0.064212
759	BMLF1	CACNA2D4	0.051319
760	EBNA1	SIPA1	0.1046
761	BZLF1	DCAF11	0.2289
762	EBNA3	PHACTR4	0.042301
763	EBNA3	MELK	0.34273
764	BZLF1	HNRNPLL	0.19531
765	BVRF1	PPP3CA	0.025659
766	BLLF1	FAM110D	0.034294
767	EBNA3	TMEM132D	0.37523
768	BMLF1	RASA1	0.1349
769	BGLF4	CHEK2	0.01862
770	EBNA-LP	GYG1	0.30233
771	EBNA3	ETNK2	0.05804
772	EBNA1	HECTD3	0.10498
773	BGLF4	STAMBP	0.10332
774	EBNA-LP	SIRT7	0.19657
775	EBNA1	CCDC130	0.21056
776	BOLF1	S100B	0.077247
777	LMP1	C11orf72	0.013254
778	BVRF1	TNNC1	0.14762
779	BLLF1	EFEMP1	0.12013
780	EBNA-LP	RFC4	0.17202
781	EBNA1	CTSL	0.11256
782	BRRF1	EGF	0.068842
783	BMLF1	RNASET2	0.080492
784	BLLF1	ERICH1-AS1	0.038495
785	EBNA3	DGKZ	0.21239
786	BZLF2	PEF1	0.15638
787	BGLF4	RAP1B	0.093899
788	EBNA3	PARP1	0.11827
789	EBNA-LP	PLEKHA1	0.13062
790	EBNA3	SLC25A23	0.18081
791	EBNA4	CTU1	0.1668
792	BGLF4	DUOX1	0.10154
793	BMLF1	DUSP26	0.15579
794	BZLF1	EDA2R	0.13273
795	EBNA3	PPFIA4	0.38703
796	LMP1	CCDC176	0.069195
797	EBNA1	LYST	0.097439
798	EBNA4	CLINT1	0.045197
799	EBNA3	CD27	0.24871
800	EBNA1	SLC4A1AP	0.14201
801	EBNA-LP	POLR3F	0.22221
802	BZLF1	SLC9A1	0.077618
803	BMLF1	GRB2	0.064881
804	BZLF1	MED23	0.11748
805	EBNA1	IL1R2	0.21846
806	EBNA3	CCNB1IP1	0.27437
807	BHRF1	FAM124A	0.16327
808	BZLF1	SKA1	0.11495
809	EBNA3	STRN	0.27109
810	BNLF2b	EPB42	0.014575
811	EBNA3	LINC00083	0.011516
812	EBNA1	HIST3H3	0.072933
813	EBNA4	BRAF	0.016524
814	EBNA4	PSMD2	0.049918
815	BZLF1	TP53BP1	0.074795
816	BLLF2	DTNBP1	0.032059

817	BLLF1	PPP2R3A	0.063157
818	EBNA1	ECHS1	0.084837
819	BMLF1	PAFAH1B3	0.28759
820	BZLF1	SUMO3	0.20889
821	BMLF1	GTF3C4	0.31692
822	BALF2	PLSCR4	0.10682
823	EBNA-LP	ZXDA	0.13382
824	EBNA4	CALR	0.022682
825	EBNA3	TERT	0.20464
826	EBNA3	CD70	0.24234
827	BZLF1	NOTCH4	0.13641
828	BZLF2	LURAP1L	0.087373
829	BZLF1	PTPRK	0.022869
830	BMLF1	INSIG1	0.22938
831	BMLF1	SNX1	0.24795
832	BTRF1	CHMP6	0.010805
833	BZLF1	USE1	0.14743
834	BZLF1	CEP97	0.21195
835	BZLF1	SDHA	0.19415
836	EBNA3	P4HTM	0.15446
837	BZLF1	LMX1B	0.037026
838	BGRF1/BDRF1	NECAB2	0.22375
839	BDLF1	JAG2	0.034357
840	EBNA3	RILP	0.33753
841	BGLF4	MITF	0.04891
842	BMLF1	TLR4	0.020589
843	BGLF4	EFNA4	0.24325
844	EBNA-LP	RBM17	0.39293
845	EBNA3	NSG2	0.085477
846	EBNA1	CTIF	0.20825
847	EBNA-LP	SLC25A12	0.11965
848	EBNA3	HUS1	0.25724
849	BLLF2	SLC18A1	0.014047
850	BLLF1	FAT1	0.045891
851	EBNA-LP	FMR1	0.39057
852	BMLF1	PBXIP1	0.2957
853	BZLF1	DDX42	0.19117
854	BMLF1	SNX13	0.019167
855	BMLF1	USP25	0.2957
856	BGLF4	TCF3	0.011078
857	EBNA6	ATP6V1E1	0.055973
858	EBNA4	CRELD2	0.21723
859	EBNA1	CD200	0.16279
860	BZLF1	CSNK2A2	0.062471
861	EBNA3	CTBP1	0.059812
862	EBNA1	PRSS8	0.20037
863	EBNA3	CALN1	0.1702
864	BMLF1	DRD3	0.080241
865	BGLF4	SNAP23	0.16991
866	BGLF4	ARL2BP	0.18137
867	EBNA3	RELB	0.16126
868	EBNA3	SERINC1	0.25758
869	EBNA3	SYNE1	0.22061
870	BILF1	ubl4ab	0.12232
871	EBNA3	TREX2	0.051626
872	EBNA3	PRRG3	0.18648
873	EBNA1	MIS12	0.13182
874	EBNA4	PRPF3	0.13573
875	BMLF1	LITAF	0.27198
876	BMLF1	PLXNC1	0.29935
877	EBNA3	TLN2	0.16069
878	EBNA1	N4BP2	0.15371
879	BZLF2	KIAA0040	0.030918
880	BZLF1	CDH4	0.032162
881	EBNA-LP	C1orf195	0.15232
882	LMP2	GCH1	0.027809
883	BPLF1	ZK1128.3	0.10678
884	LMP1	RCN1	0.090347

885	BMLF1	MPP6	0.43034
886	BMLF1	MAN1C1	0.119
887	EBNA3	NECAP1	0.10706
888	EBNA1	UPF3A	0.21128
889	BMLF1	RLBP1	0.068114
890	BGRF1/BDRF1	CFTR	0.021037
891	BGLF4	RHOG	0.094834
892	EBNA3	SOX10	0.049987
893	BZLF1	RBCK1	0.024615
894	EBNA3	CNOT6	0.24949
895	BMLF1	FAM124A	0.093418
896	EBNA3	KIAA0101	0.32628
897	BZLF1	POP5	0.14242
898	EBNA3	EIF1AY	0.54659
899	EBNA3	RHOH	0.26819
900	EBNA3	KRTAP2-2	0.05706
901	EBNA6	NUDT21	0.05634
902	EBNA-LP	DTNB	0.1401
903	BZLF1	SYNPO	0.14335
904	BILF1	PLCE1	0.019534
905	EBNA3	RCHY1	0.17139
906	EBNA-LP	NOL8	0.37125
907	EBNA3	MOV10	0.29077
908	EBNA1	CAND1	0.13781
909	BHRF1	MBD3L5	0.014925
910	BVRF1	KCNIP2	0.057019
911	BZLF1	MPZL2	0.17972
912	BGLF4	CPSF6	0.16637
913	gN	PITPNM3	0.092698
914	EBNA-LP	RBM8A	0.27128
915	BMLF1	CDR2	0.4034
916	BGLF4	IL13RA1	0.21267
917	BLLF2	ZNF18	0.018562
918	BGLF4	LMX1B	0.070363
919	BZLF1	TBC1D2B	0.086597
920	BZLF1	ZBTB14	0.11335
921	BGLF4	ITGAX	0.16592
922	BBRF2	CHMP4C	0.030358
923	BMLF1	SPON1	0.41705
924	BMLF1	ATP11C	0.24724
925	EBNA-LP	SH3GL2	0.2164
926	EBNA3	CHMP1A	0.17985
927	EBNA3	YIPF5	0.090986
928	BMLF1	EFHB	0.26298
929	BMLF1	PLCH2	0.044039
930	BGLF4	CREB3	0.025364
931	EBNA-LP	TGFB1I1	0.15596
932	EBNA3	NEDD9	0.23153
933	BZLF1	COMMD1	0.12622
934	BLLF1	CCDC159	0.027736
935	EBNA-LP	IFITM1	0.23171
936	EBNA3	EDC4	0.23419
937	BMLF1	TRIO	0.13954
938	EBNA1	SNRNP40	0.1451
939	EBNA3	GAGE6	0.031848
940	BGLF4	MERTK	0.050932
941	BGLF4	LRP1	0.12358
942	BGLF4	GAPDH	0.10758
943	EBNA1	PDE9A	0.083584
944	BMLF1	CNTROB	0.34403
945	BGLF4	ESR2	0.045135
946	BZLF1	RNF19B	0.14826
947	BALF2	S100A11	0.10498
948	EBNA-LP	ADARB1	0.014486
949	BMLF1	TMOD3	0.014375
950	EBNA4	GAS1	0.09861
951	EBNA4	CDK5R1	0.12456
952	EBNA3	RET	0.2266

953	gB	EFEMP2	0.069518
954	BLLF2	CASP8AP2	0.093599
955	EBNA1	CASP10	0.15416
956	BZLF1	PAXIP1	0.12952
957	BLLF2	OR2L3	0.09263
958	BTRF1	GOLGA8I	0.018923
959	EBNA1	LDLR	0.10847
960	EBNA6	NUDCD3	0.11414
961	BGLF4	OTX2	0.022935
962	BZLF1	PRKG1	0.067618
963	BMLF1	ERCC1	0.088013
964	BZLF2	CCDC171	0.025135
965	EBNA1	DEDD	0.11565
966	EBNA1	FBL	0.22926
967	BGLF4	POLD3	0.073988
968	EBNA3	FGFR1OP	0.18799
969	EBNA1	PSMA7	0.037888
970	BZLF1	PLK1S1	0.11143
971	BLLF2	CHRD	0.010771
972	LMP1	DNER	0.041096
973	EBNA-LP	DPY30	0.24304
974	BPLF1	RNH1	0.12352
975	BGLF4	CD93	0.29126
976	BGLF4	HS3ST3A1	0.0317
977	BMLF1	TRMT5	0.017817
978	EBNA-LP	PLIN3	0.29266
979	BGLF4	SYT2	0.29937
980	EBNA1	CIB4	0.044917
981	BGLF4	UTP20	0.15465
982	BZLF2	UPF3A	0.059304
983	BZLF1	EGLN3	0.11472
984	BLLF2	GNPTG	0.024487
985	EBNA3	BICD1	0.21114
986	BZLF1	DDX23	0.10563
987	BZLF1	ITGB4	0.098765
988	BGLF4	SEMG1	0.12143
989	BZLF1	TPM4	0.027682
990	BZLF1	LIN28B	0.14428
991	BMLF1	SLCO3A1	0.010833
992	BGLF4	SEC61B	0.11676
993	BMLF1	UTF1	0.28774
994	BMLF1	NCAPG	0.33234
995	BGLF4	TFG	0.18938
996	BMLF1	TCAP	0.22799
997	BHRF1	C16orf95	0.019814
998	BMLF1	MAP2K5	0.15057
999	EBNA3	PECAM1	0.2649
1000	EBNA1	LRP6	0.012735
1001	BVRF1	ATP2B2	0.011029
1002	EBNA3	MED9	0.26309
1003	EBNA3	TGFA	0.17194
1004	EBNA-LP	FBXO11	0.20588
1005	EBNA-LP	MTDH	0.14865
1006	BPLF1	FAT1	0.070859
1007	BLLF2	IWS1	0.098175
1008	BGLF4	GTF2A1	0.12304
1009	BALF2	FAM231A	0.08451
1010	BMLF1	EDEM2	0.15995
1011	BLLF2	PCF11	0.024595
1012	BZLF1	NCOA1	0.066922
1013	BMLF1	TAF6L	0.2065
1014	BGLF4	FAT2	0.039817
1015	BMLF1	RAMP1	0.18919
1016	BMLF1	BCKDK	0.25012
1017	BZLF1	MAPK6	0.052424
1018	EBNA1	REEP5	0.1967
1019	BVRF1	PRSS3	0.18092
1020	BXRF1	PRDX6	0.019954

1021	EBNA1	RABAC1	0.18914
1022	BZLF1	COPS6	0.16575
1023	BGLF4	LZTS1	0.055642
1024	BMLF1	REEP5	0.41725
1025	EBNA3	TYR	0.24387
1026	BGLF4	RBM17	0.21536
1027	EBNA3	AATK	0.23942
1028	BMLF1	GPR98	0.30458
1029	EBNA1	HHLA3	0.22232
1030	EBNA3	RAPGEF1	0.27041
1031	BMLF1	TRADD	0.18328
1032	BPLF1	MAGEB4	0.01671
1033	BZLF1	RBMS1	0.19727
1034	BBRF2	MGAT5	0.015599
1035	BMLF1	CAPNS2	0.23981
1036	BMLF1	PRKX	0.17174
1037	BGLF4	PARK7	0.13505
1038	BHRF1	GOLGA6L20	0.066907
1039	EBNA2	RABEP1	0.012527
1040	BGLF4	CDK8	0.012478
1041	BGLF4	CSK	0.049171
1042	EBNA-LP	NECAB1	0.11139
1043	EBNA1	DGKQ	0.13432
1044	BLLF2	POLR3D	0.043167
1045	EBNA3	DCHS2	0.18006
1046	BBRF2	PGAM4	0.018246
1047	BZLF1	BIK	0.1296
1048	BOLF1	CAPNS1	0.039836
1049	BMLF1	USH1G	0.30198
1050	EBNA1	CIB3	0.044917
1051	BZLF1	MCU	0.15013
1052	BZLF1	NFATC1	0.032686
1053	BXRF1	SPHK2	0.026582
1054	EBNA3	BRD7	0.14177
1055	BZLF1	RNF31	0.096036
1056	gM	PKD2L1	0.050287
1057	EBNA3	VIMP	0.15047
1058	EBNA-LP	HOXA9	0.17279
1059	EBNA1	NCF2	0.038553
1060	BMLF1	STAM2	0.23874
1061	EBNA3	PKN2	0.094567
1062	EBNA-LP	ARL14EP	0.41868
1063	EBNA1	MAGI2	0.062964
1064	BGLF4	CALR3	0.029174
1065	BGLF4	MYL9	0.044354
1066	BTRF1	FAM21B	0.018857
1067	BGLF4	ASPN	0.072738
1068	gN	MYO1E	0.019857
1069	BMLF1	ZNF143	0.22901
1070	BLLF2	TAS2R5	0.028776
1071	BGLF4	EPS15	0.21954
1072	EBNA4	EID1	0.14359
1073	EBNA1	S100A7L2	0.02299
1074	BGLF4	RINT1	0.18065
1075	EBNA-LP	SLC7A8	0.11405
1076	BZLF1	LOR	0.065814
1077	BGLF4	LSM1	0.16126
1078	BZLF1	ZNF646	0.11682
1079	EBNA3	EFEMP1	0.53144
1080	BMLF1	PMF1	0.21541
1081	EBNA3	CENPW	0.27525
1082	BGLF4	FBXO17	0.22789
1083	BMLF1	PTGIS	0.10938
1084	EBNA-LP	SH3BP2	0.26508
1085	BOLF1	MPTX1	0.014506
1086	BBRF2	FAM188A	0.01209
1087	EBNA-LP	HLA-DRB1	0.082559
1088	BTRF1	AP3S1	0.010195

1089	BTRF1	PRPH2	0.012877
1090	gB	PEF1	0.06838
1091	gB	PKD2L1	0.030994
1092	EBNA1	NCBP1	0.09151
1093	BMLF1	UBAP2L	0.44386
1094	BZLF1	NCKIPSD	0.21642
1095	BZLF2	XAGE3	0.10168
1096	EBNA-LP	SERGEF	0.30768
1097	BILF1	CLINT1	0.028715
1098	BGLF4	DARS2	0.070666
1099	EBNA3	SOS1	0.13164
1100	EBNA1	GNMT	0.16847
1101	BZLF2	FAM221B	0.054283
1102	EBNA1	EFHB	0.044917
1103	BBRF2	MED13	0.02066
1104	EBNA3	CTH	0.11531
1105	LMP2	S100A12	0.053625
1106	LMP2	FBLN5	0.1051
1107	EBNA-LP	GPR37L1	0.31975
1108	EBNA3	SSR4	0.22051
1109	BPLF1	CDHR2	0.12503
1110	EBNA1	MPP7	0.17486
1111	EBNA-LP	ARHGAP26	0.15502
1112	BZLF1	CIB1	0.31435
1113	BZLF1	KSR1	0.012
1114	BZLF1	ESPL1	0.097629
1115	BGLF4	ARHGAP10	0.076756
1116	EBNA1	ATR	0.075431
1117	BZLF2	CBY3	0.042929
1118	EBNA6	GOLGA6L19	0.17856
1119	BZLF1	DYNC1H1	0.11463
1120	BZLF1	BTG2	0.10878
1121	BMLF1	FAM161A	0.47289
1122	BZLF1	APOB	0.01773
1123	BGLF4	SETDB1	0.18211
1124	EBNA-LP	B2M	0.18803
1125	BLLF2	HPS6	0.086822
1126	EBNA-LP	KMT2E	0.18928
1127	BNLF2b	GYG2	0.023701
1128	EBNA3	ATRAID	0.24299
1129	EBNA-LP	RFC1	0.12996
1130	BMLF1	IKZF3	0.13663
1131	BMLF1	ERCC3	0.040532
1132	BMLF1	PYDC1	0.31509
1133	EBNA3	CDKN1A	0.083996
1134	BZLF1	TUBB2B	0.034943
1135	BZLF1	ZDHHC17	0.179
1136	BLLF2	G4	0.014549
1137	BMLF1	GNE	0.050591
1138	BMLF1	CRELD1	0.23596
1139	BGLF4	G3BP1	0.12874
1140	BMLF1	TRIM24	0.07342
1141	gM	C1orf64	0.0282
1142	BMLF1	MICAL3	0.03219
1143	BMLF1	RBPJ	0.071608
1144	BGLF4	RHOA	0.020989
1145	BMLF1	GLMN	0.37148
1146	BMLF1	PIK3CD	0.15238
1147	gL	CCDC102B	0.016378
1148	EBNA-LP	MCTP2	0.18708
1149	EBNA3	CCDC153	0.015073
1150	EBNA6	ATXN7L1	0.072672
1151	BLLF1	RGAG4	0.020903
1152	EBNA3	PPP1R1A	0.38333
1153	BZLF1	TCEA1	0.077302
1154	BMLF1	MPZL1	0.28983
1155	BZLF1	C2CD5	0.053341
1156	EBNA-LP	NFYA	0.16738

1157	BMLF1	DYNLT1	0.18987
1158	EBNA3	APAF1	0.19137
1159	EBNA1	FNTA	0.1607
1160	EBNA3	NPAT	0.15027
1161	BMLF1	RAB11FIP1	0.017781
1162	EBNA1	GEMIN7	0.14629
1163	BGLF4	USP47	0.13614
1164	BZLF1	MED11	0.1292
1165	BGLF4	AMOTL2	0.23098
1166	EBNA-LP	KREMEN1	0.37087
1167	EBNA3	FBN1	0.57599
1168	BGLF4	NSMCE1	0.12963
1169	BMLF1	EFCAB3	0.26298
1170	BZLF1	CAND2	0.15802
1171	BLLF2	DNASE1L1	0.1022
1172	BLLF1	ANXA6	0.10617
1173	BLLF2	VPS25	0.01716
1174	BZLF1	PDPK1	0.015732
1175	BMLF1	MAPK9	0.13389
1176	BZLF1	GNAI1	0.052829
1177	BLLF2	EARS2	0.029197
1178	BMLF1	TNFSF13B	0.21114
1179	BMLF1	TFAP2C	0.094806
1180	EBNA3	SHC3	0.31319
1181	EBNA1	CDC42EP2	0.12355
1182	BGLF4	DHX36	0.10748
1183	EBNA-LP	HIVEP1	0.2271
1184	EBNA-LP	SNX27	0.271
1185	BGLF4	NLRP3	0.093765
1186	EBNA1	EIF2B2	0.14359
1187	BZLF1	WEE1	0.050419
1188	EBNA3	ZP3	0.083089
1189	BLLF2	GGTLC2	0.029477
1190	BZLF1	EPO	0.070696
1191	BMLF1	CATSPER2	0.21553
1192	BZLF1	PARD6G	0.17803
1193	BZLF1	WDR82	0.16738
1194	BZLF1	RBBP7	0.099278
1195	EBNA1	ATP5F1	0.20613
1196	EBNA-LP	CMKLR1	0.23292
1197	BGLF4	SORBS3	0.10464
1198	BZLF1	FBXO46	0.23617
1199	EBNA3	MRPS27	0.34367
1200	BZLF1	MRE11A	0.073766
1201	BGLF4	EIF2AK2	0.032699
1202	BGLF4	PCDHGA1	0.058173
1203	BMLF1	DCT	0.016391
1204	BLLF2	IFNE	0.011237
1205	BGLF4	RMDN3	0.090483
1206	BMLF1	NUMA1	0.17255
1207	EBNA6	C4orf22	0.048546
1208	BMLF1	ELP4	0.26219
1209	EBNA-LP	MAD2L1BP	0.32286
1210	gB	PDCD6	0.10124
1211	EBNA1	SLC9A3R2	0.086583
1212	BTRF1	SEC11C	0.012984
1213	EBNA3	EZH2	0.19025
1214	BMLF1	GSTA2	0.041163
1215	EBNA4	FAM49B	0.09668
1216	BLLF2	MCOLN1	0.011666
1217	BGLF4	PCDHB7	0.056436
1218	BMLF1	MOB3B	0.43446
1219	EBNA3	ALOX5	0.17876
1220	BGLF4	NAP1L1	0.15944
1221	EBNA-LP	GNAI1	0.127
1222	EBNA-LP	FLG2	0.05766
1223	BZLF1	MED28	0.13749
1224	BGLF4	PODXL2	0.2024

1225	BZLF1	RFC1	0.0533
1226	EBNA3	RARS	0.14534
1227	BGLF4	NPC1L1	0.22418
1228	EBNA3	EID1	0.23815
1229	BMLF1	HIRA	0.20462
1230	EBNA-LP	MYL6B	0.56118
1231	EBNA-LP	CDH3	0.18238
1232	BGLF4	MCRS1	0.1543
1233	EBNA3	MAP2K3	0.091983
1234	BILF1	F10	0.10195
1235	BMLF1	TNFAIP1	0.26252
1236	BZLF2	NIN	0.11155
1237	EBNA-LP	SPDL1	0.20114
1238	BZLF1	LSM3	0.16958
1239	BZLF1	MICALL1	0.17775
1240	EBNA3	PPP2R2A	0.27104
1241	EBNA2	DTNA	0.056072
1242	BHRF1	NUCB2	0.10042
1243	BMLF1	NT5C3B	0.35622
1244	EBNA3	ITGAX	0.29757
1245	BGLF4	TYRP1	0.14732
1246	BMLF1	MT1H	0.31612
1247	EBNA3	HNRNPC	0.29716
1248	BBRF2	NPIPA1	0.045329
1249	BALF2	HSPA5	0.043699
1250	EBNA3	ATP6V0C	0.22101
1251	EBNA1	NUAK2	0.069257
1252	EBNA3	PEAK1	0.24634
1253	EBNA1	NME2	0.044828
1254	BGLF4	LONP2	0.10567
1255	EBNA3	NUP160	0.22679
1256	BZLF1	CCBE1	0.040177
1257	BGLF4	THAP3	0.20471
1258	BGLF4	ZNF423	0.12842
1259	BMLF1	ZFYVE26	0.33161
1260	BGLF4	AMBRA1	0.24263
1261	EBNA6	PP632	0.072543
1262	gL	CCDC54	0.026865
1263	EBNA1	MAST1	0.065258
1264	EBNA1	SNAP47	0.18989
1265	EBNA6	IQGAP1	0.027145
1266	BGLF4	SPTLC1	0.11442
1267	BMLF1	PPM1G	0.23063
1268	EBNA3	LDHAL6B	0.35042
1269	BMLF1	PIP5K1A	0.16096
1270	EBNA-LP	PDE4DIP	0.31006
1271	BZLF1	PRRG3	0.051176
1272	BLLF2	ZNF169	0.016611
1273	BMLF1	CRNKL1	0.032363
1274	EBNA1	TLDC1	0.22232
1275	BZLF1	BRCA2	0.039569
1276	BZLF1	TRPC1	0.10438
1277	BGLF4	CCAR2	0.20225
1278	BPLF1	C16orf72	0.10508
1279	LMP2	MYO1E	0.018533
1280	EBNA3	RABGAP1L	0.024566
1281	BZLF1	TIMP3	0.15469
1282	BBRF2	C5orf55	0.044878
1283	EBNA6	SHCBP1L	0.039842
1284	EBNA3	DHX58	0.18001
1285	BBRF2	GPI	0.016818
1286	EBNA-LP	KANSL1	0.32796
1287	BRRF1	VIL1	0.09559
1288	BZLF1	MYO5B	0.19086
1289	BGLF4	STUB1	0.077762
1290	BZLF1	NEDD4	0.041017
1291	LMP1	BNIP2	0.021015
1292	EBNA4	HTT	0.017402

1293	EBNA1	TRIP12	0.15648
1294	BZLF1	RPP38	0.16251
1295	BZLF1	DSC1	0.058018
1296	BZLF1	ORMDL3	0.18681
1297	BZLF1	INCENP	0.10511
1298	EBNA-LP	OTOF	0.15409
1299	BGLF4	LPP	0.14024
1300	EBNA3	CROCC	0.19802
1301	BMLF1	PEX19	0.24117
1302	BMLF1	RAD54L	0.24665
1303	EBNA4	CCDC74A	0.15466
1304	BLLF2	TBC1D3B	0.010407
1305	BMLF1	MALT1	0.13069
1306	BZLF1	CDK14	0.0443
1307	EBNA6	PARP10	0.085582
1308	EBNA3	ADRBK1	0.065904
1309	EBNA-LP	TTC1	0.37818
1310	EBNA1	DPY30	0.12232
1311	BMLF1	FOSL1	0.11824
1312	EBNA-LP	PRDX3	0.14105
1313	EBNA3	FGF21	0.336
1314	BGLF4	ATG12	0.19233
1315	BGLF4	RABGGTB	0.20742
1316	BGLF4	ATP5B	0.12314
1317	BMLF1	EID2	0.24882
1318	BMLF1	TRIM71	0.019518
1319	BLLF1	FBRSL1	0.022927
1320	EBNA-LP	ITGA3	0.29547
1321	EBNA3	PCDHB7	0.17455
1322	EBNA-LP	CDHR2	0.58491
1323	BPLF1	TPPP	0.097066
1324	BGLF4	SH3GL1	0.21334
1325	BXRF1	LETM1	0.1655
1326	EBNA1	PICALM	0.043629
1327	BMLF1	NXT1	0.30739
1328	BLLF2	PSME2	0.050072
1329	BMLF1	TERF2	0.16827
1330	EBNA3	RMND5B	0.014738
1331	BLLF1	THBD	0.062799
1332	gL	C19orf35	0.11827
1333	BARF1	SPHK2	0.042405
1334	BTRF1	CASP8AP2	0.013512
1335	EBNA3	SPIDR	0.11913
1336	EBNA4	PRSS2	0.1902
1337	BLLF2	SAC3D1	0.074039
1338	BARF1	ADAR	0.016878
1339	EBNA-LP	SIAH2	0.17019
1340	BGLF4	CDK5RAP3	0.12035
1341	BMLF1	CEACAM1	0.33715
1342	EBNA1	GRK5	0.02833
1343	BVRF1	FASN	0.058001
1344	BLLF2	LGALS12	0.027737
1345	BZLF2	UTRN	0.051924
1346	EBNA3	ZNF143	0.2017
1347	EBNA4	GAS6	0.015151
1348	EBNA1	FSTL4	0.043666
1349	BHRF1	MISP	0.14454
1350	BVRF1	SYT1	0.059892
1351	BMLF1	UBD	0.2721
1352	BARF1	TMED2	0.016972
1353	BOLF1	IL6ST	0.082234
1354	EBNA3	RAP1A	0.20698
1355	BPLF1	PPP2R3A	0.088267
1356	BZLF1	AIF1	0.014346
1357	BGLF4	PCDHB16	0.053256
1358	BMLF1	ERCC8	0.26376
1359	EBNA1	EIF2S2	0.22395
1360	EBNA6	MED9	0.18514

1361	BPLF1	UTRN	0.04917
1362	EBNA-LP	GEMIN5	0.32957
1363	EBNA3	UBE2V1	0.14301
1364	EBNA-LP	LSM6	0.26842
1365	BPLF1	INTS4L2	0.017242
1366	BZLF1	CORO1B	0.19517
1367	EBNA3	MAN1A2	0.070013
1368	BZLF1	ARFGEF1	0.073316
1369	BZLF1	SBF2	0.11529
1370	BMLF1	PIK3C2A	0.063105
1371	EBNA4	SPTAN1	0.11372
1372	BGLF4	ITPR1	0.074541
1373	BHRF1	INTS4L1	0.015317
1374	BZLF1	E2F1	0.015955
1375	BZLF1	S100Z	0.057416
1376	EBNA1	COMMD7	0.18486
1377	LMP2	LETM1	0.090745
1378	BLLF2	AKAP8	0.068673
1379	BBRF2	SAP130	0.032933
1380	BHRF1	FAM181A	0.046603
1381	BZLF1	SCG5	0.16385
1382	EBNA1	EXOSC5	0.13988
1383	BBRF2	NKAIN3	0.038317
1384	BMLF1	POLA1	0.024052
1385	EBNA-LP	CCNA1	0.1774
1386	EBNA-LP	ERBB2IP	0.20671
1387	BARF1	MEGF6	0.21261
1388	EBNA1	FBXO7	0.15357
1389	EBNA-LP	PLA2G2D	0.075881
1390	EBNA-LP	RXRB	0.10536
1391	BMLF1	IFIT1	0.30505
1392	BGLF4	EFCAB4B	0.3298
1393	EBNA-LP	BMX	0.1271
1394	BLLF2	NAP1L4	0.011867
1395	BGLF4	SUN1	0.13026
1396	BZLF1	RGR	0.16496
1397	BMLF1	KIF23	0.19649
1398	BGLF4	NUP133	0.10489
1399	BALF2	CADPS	0.15332
1400	EBNA4	GABARAP	0.032667
1401	BMLF1	ZFAND6	0.26123
1402	EBNA-LP	DCAF11	0.41438
1403	EBNA6	FAM178A	0.15112
1404	gN	ACTN2	0.040652
1405	BMLF1	USP8	0.18546
1406	BZLF1	EIF2S2	0.23494
1407	BMLF1	OPN4	0.024031
1408	gM	NPC1L1	0.01801
1409	BGLF4	PARD6A	0.094393
1410	BTRF1	EMID1	0.014747
1411	BBRF2	IWS1	0.076725
1412	EBNA6	ZNF326	0.13043
1413	BHRF1	WLS	0.043945
1414	BPLF1	PLSCR3	0.11757
1415	BLLF2	SMIM19	0.051963
1416	EBNA3	MB21D2	0.04609
1417	gL	LINC00597	0.097749
1418	BLLF2	TECTB	0.01551
1419	EBNA1	PRKDC	0.025629
1420	BZLF1	PRPF19	0.11961
1421	EBNA6	C12orf36	0.063832
1422	BMLF1	SNRPG	0.24652
1423	EBNA1	ADAMTSL4	0.11698
1424	gB	SPARC	0.033177
1425	EBNA-LP	WRAP53	0.42304
1426	BGLF4	UBE2M	0.10364
1427	BGLF4	EFCAB7	0.068073
1428	BOLF1	RBM8A	0.010735

1429	EBNA3	TP53BP2	0.25145
1430	BHRF1	CLINT1	0.015779
1431	EBNA1	EXOSC8	0.16584
1432	BGLF4	RPL14	0.16495
1433	gL	STAG3L4	0.040382
1434	BMLF1	DPM3	0.24942
1435	BMLF1	CTCFL	0.21002
1436	BGLF4	HSPB9	0.20235
1437	BPLF1	C19orf71	0.051816
1438	BGLF4	FYCO1	0.23762
1439	EBNA3	VAV1	0.051178
1440	gL	CNBD1	0.010944
1441	EBNA6	SH3BP5L	0.15296
1442	BGLF4	TAX1BP1	0.16452
1443	BGRF1/BDRF1	ACTN2	0.067389
1444	EBNA2	MMP20	0.02474
1445	BGLF4	CPS1	0.035429
1446	EBNA3	CXorf67	0.14467
1447	BZLF1	SPRR1A	0.094778
1448	BMLF1	TSHR	0.29564
1449	BGLF4	LTBP4	0.28119
1450	EBNA3	C2CD4D	0.030504
1451	EBNA1	TRIM5	0.073799
1452	BGLF4	SCAMP1	0.18
1453	EBNA1	FCHO2	0.14453
1454	EBNA6	EXOC3L4	0.11612
1455	EBNA3	SNRPD3	0.19909
1456	BLLF1	S100A13	0.10491
1457	BGLF4	VPRBP	0.18197
1458	BMLF1	PPP2R3C	0.21335
1459	EBNA1	GSTZ1	0.19798
1460	BMLF1	HOXA5	0.07387
1461	EBNA3	CELSR2	0.092581
1462	BHRF1	XAGE2	0.13356
1463	gN	RNH1	0.097508
1464	EBNA3	GAPDH	0.20997
1465	EBNA3	ATXN7	0.24423
1466	BMLF1	FBXO7	0.3401
1467	BZLF1	PICALM	0.051309
1468	EBNA3	RAB13	0.01975
1469	BPLF1	CD93	0.099187
1470	EBNA1	ADAMTSL2	0.1307
1471	BZLF1	PUF60	0.13202
1472	BMLF1	HS3ST1	0.020489
1473	EBNA1	NCKIPSD	0.20544
1474	EBNA1	EGF	0.11534
1475	EBNA1	USH1C	0.096441
1476	BMLF1	PABPC3	0.041775
1477	BZLF1	PRRC2A	0.19741
1478	EBNA1	MINK1	0.061807
1479	BMLF1	ZNF346	0.32521
1480	EBNA-LP	CYBA	0.088801
1481	EBNA-LP	MEIS1	0.12583
1482	EBNA1	MYCN	0.031043
1483	BLLF2	G4	0.035306
1484	EBNA-LP	NSUN3	0.0171
1485	EBNA3	HINFP	0.084959
1486	BLLF2	H2BFWT	0.016553
1487	BMLF1	KXD1	0.44262
1488	EBNA3	RPS6	0.086684
1489	EBNA1	PRDM16	0.064946
1490	EBNA-LP	MAP3K7CL	0.37575
1491	EBNA3	VMP1	0.25742
1492	BMLF1	TONSL	0.30083
1493	gL	SH3BP5L	0.089547
1494	EBNA3	SGSM3	0.29728
1495	BZLF1	FAAP20	0.14597
1496	gL	DNAJC9-AS1	0.1399

1497	EBNA4	SPHK2	0.048975
1498	EBNA-LP	LAMTOR3	0.30244
1499	EBNA3	TNNI2	0.31493
1500	EBNA1	MACROD1	0.18305
1501	BMLF1	MC5R	0.31637
1502	EBNA-LP	PEX11A	0.29219
1503	EBNA1	RNF2	0.06778
1504	BMLF1	HIC1	0.12186
1505	BMLF1	TBC1D9B	0.1868
1506	EBNA-LP	RPRD1B	0.26619
1507	BZLF1	WIBG	0.24801
1508	BZLF1	HERC2	0.078536
1509	BGLF4	ADAM22	0.12481
1510	BZLF2	FAM195B	0.033756
1511	BLLF1	FAM127B	0.022457
1512	gM	VIL1	0.050716
1513	EBNA3	ADCY6	0.061675
1514	EBNA-LP	MYL3	0.13611
1515	BGLF4	ZC3H7B	0.14911
1516	EBNA-LP	HERC2	0.16654
1517	BZLF1	CREB5	0.043344
1518	EBNA-LP	SPOCK3	0.16209
1519	EBNA-LP	A1CF	0.25973
1520	BMLF1	SMAD1	0.035133
1521	BLLF2	ZNF121	0.013743
1522	BNLF2b	DNASE1L2	0.11147
1523	BGLF4	OLA1	0.1299
1524	EBNA1	DDX39B	0.11377
1525	EBNA1	PPP3R2	0.044917
1526	EBNA-LP	MAP4K4	0.23035
1527	EBNA-LP	WDR12	0.26578
1528	EBNA1	TYRO3	0.075866
1529	BZLF1	MYCN	0.037379
1530	BMLF1	ASB16	0.43457
1531	EBNA-LP	PTRF	0.20252
1532	EBNA1	LRRFIP1	0.094334
1533	BBRF2	RNASE9	0.022754
1534	EBNA-LP	ACTN4	0.3004
1535	EBNA-LP	TRIM17	0.28409
1536	EBNA3	ARHGAP32	0.15833
1537	BMLF1	ALX1	0.16444
1538	EBNA-LP	PDIA2	0.23611
1539	EBNA3	EPHA7	0.1593
1540	BZLF1	DEDD2	0.10409
1541	EBNA-LP	INTS10	0.34308
1542	EBNA3	CCDC127	0.028257
1543	EBNA3	TDGF1	0.091578
1544	BPLF1	GAGE12H	0.037608
1545	EBNA3	PLA2G12A	0.054483
1546	EBNA1	RAB3A	0.050035
1547	EBNA4	CCDC176	0.28363
1548	BBRF2	P4HA1	0.029995
1549	EBNA3	OPRK1	0.22977
1550	BMLF1	PRRG2	0.23236
1551	BNLF2b	TREML1	0.026058
1552	EBNA3	ANK2	0.059417
1553	BMLF1	FGGY	0.12856
1554	EBNA1	DCAF11	0.21589
1555	EBNA-LP	SENP6	0.26976
1556	BMLF1	TMEM102	0.35726
1557	BZLF1	JUNB	0.016659
1558	EBNA1	IMP3	0.15192
1559	BZLF1	MSX2	0.015224
1560	EBNA3	SMC3	0.15448
1561	BMLF1	TRAF3	0.1113
1562	EBNA6	FAM179A	0.013701
1563	BRRF1	KCNJ4	0.059865
1564	BMLF1	ZC3H11A	0.39591

1565	EBNA3	VPRBP	0.32989
1566	EBNA1	PDZD7	0.1789
1567	BZLF1	RPS27	0.077055
1568	BZLF1	UTP14A	0.17484
1569	BGRF1/BDRF1	FBN1	0.19871
1570	EBNA6	MT2A	0.10556
1571	BLLF1	CDH23	0.02408
1572	EBNA1	IQGAP1	0.1258
1573	BALF2	HSP90B1	0.073232
1574	BMLF1	A2M	0.22859
1575	BVRF1	SYT11	0.21348
1576	BBRF2	TBC1D3B	0.010188
1577	EBNA-LP	CCDC171	0.014189
1578	EBNA3	CTSL	0.23348
1579	EBNA1	CDYL2	0.1446
1580	BGLF4	C4BPA	0.20578
1581	BGLF4	COMMD4	0.23693
1582	BZLF1	RHOH	0.14923
1583	EBNA1	ITGB1BP2	0.012769
1584	BGLF4	TAF9B	0.1167
1585	BGLF4	CNTNAP1	0.097641
1586	BARF1	CCDC47	0.18165
1587	BLLF2	INTS4	0.069792
1588	EBNA-LP	SCAMP5	0.2539
1589	EBNA3	GADD45A	0.26784
1590	EBNA3	MED20	0.21099
1591	BGLF4	CTSL	0.12615
1592	EBNA-LP	PSMA5	0.10835
1593	BGLF4	CD40	0.055111
1594	BMLF1	C5orf48	0.056993
1595	BMLF1	ELOVL1	0.27226
1596	EBNA-LP	PROZ	0.12584
1597	BMLF1	MEX3B	0.19588
1598	EBNA3	FLOT1	0.27251
1599	BGLF4	ADAMTS13	0.15775
1600	EBNA-LP	ZSWIM7	0.29405
1601	BLLF2	CREBZF	0.017699
1602	BMLF1	PEG10	0.3061
1603	EBNA1	SCIN	0.028558
1604	EBNA-LP	ORAI1	0.26062
1605	EBNA1	EIF4G1	0.16783
1606	BXRF1	ACTN2	0.057188
1607	BMLF1	EFTUD2	0.27184
1608	BZLF1	ABCA1	0.024629
1609	LMP1	LRP8	0.063919
1610	BVRF1	KCNJ4	0.031461
1611	EBNA1	ERN1	0.01717
1612	EBNA-LP	BIRC6	0.17847
1613	EBNA3	COL4A3BP	0.096136
1614	EBNA3	ANXA4	0.18188
1615	BPLF1	SCUBE3	0.14596
1616	BZLF1	DDN	0.15594
1617	EBNA1	HOXB7	0.036814
1618	BNLF2b	DCT	0.01998
1619	LMP1	HSP90B1	0.046711
1620	BBRF2	PGAM1	0.048159
1621	BGLF4	STRN4	0.22284
1622	EBNA3	ITM2A	0.41166
1623	BMLF1	CALB2	0.2656
1624	BPLF1	C19orf67	0.028413
1625	BGLF4	EAF1	0.20912
1626	BMLF1	ACTN2	0.20053
1627	BMLF1	NECAP2	0.044185
1628	EBNA-LP	RTN1	0.32515
1629	EBNA1	HNF1B	0.018715
1630	BZLF1	ERBB3	0.055574
1631	BGLF4	DTNB	0.02812
1632	BZLF1	GK	0.17623

1633	BBRF2	G4	0.014018
1634	EBNA4	AP1B1	0.054694
1635	BZLF1	RPP25	0.2114
1636	BVRF1	MYL6	0.15924
1637	BMLF1	ACP1	0.17261
1638	EBNA1	PTGFRN	0.17533
1639	BMLF1	TAF2	0.2373
1640	BGLF4	GREM1	0.034098
1641	BZLF1	VPS28	0.16772
1642	EBNA1	MTMR2	0.024172
1643	EBNA1	SDPR	0.12443
1644	EBNA3	RP9	0.29831
1645	BGLF4	OCLN	0.06141
1646	BMLF1	PSMD10	0.097051
1647	EBNA3	FKBP4	0.14002
1648	BZLF1	IRS2	0.025488
1649	BLLF2	TMEM233	0.053901
1650	EBNA1	MAGEA3	0.22232
1651	EBNA-LP	LONP2	0.21411
1652	BZLF1	UBE2C	0.082828
1653	BHRF1	SPTAN1	0.070321
1654	BZLF1	CIRH1A	0.15902
1655	BGLF4	NOD1	0.040064
1656	gB	RAB11FIP4	0.13579
1657	BZLF1	HDAC5	0.021241
1658	EBNA6	smf	0.047489
1659	BMLF1	PURA	0.10045
1660	EBNA3	MIF4GD	0.38426
1661	BMLF1	MAP3K14	0.14882
1662	BMLF1	CEP70	0.3222
1663	BLLF2	C1QBP	0.02973
1664	BMLF1	CDH2	0.070263
1665	BGLF4	SPACA3	0.18409
1666	EBNA1	PLD2	0.019784
1667	BLLF2	CLDN16	0.016703
1668	BXRF1	EIF4E2	0.013273
1669	BGLF4	APBA1	0.12733
1670	BLLF1	FAM229B	0.025573
1671	EBNA-LP	ABI1	0.15603
1672	BZLF1	SUSD1	0.054178
1673	BMLF1	SPAG5	0.21535
1674	BGLF4	UBE2I	0.029515
1675	BMLF1	PLK1S1	0.30323
1676	EBNA3	CDKN1B	0.099072
1677	EBNA-LP	ASIC1	0.22803
1678	BZLF1	THOC2	0.23687
1679	BMLF1	FNIP2	0.35226
1680	BZLF1	CASP1	0.068445
1681	BBRF2	SLC16A7	0.019868
1682	EBNA3	AKAP17A	0.33161
1683	EBNA1	ELANE	0.1165
1684	EBNA-LP	NFKB2	0.082837
1685	BBRF2	LHPP	0.025531
1686	BGRF1/BDRF1	STAT1	0.02384
1687	BMLF1	DPP10	0.08038
1688	EBNA3	AP2A2	0.21814
1689	BZLF1	HAX1	0.17926
1690	BBRF2	ITIH1	0.012023
1691	BZLF2	SAMD13	0.010877
1692	BZLF1	PKD1L2	0.050218
1693	gN	S100A8	0.042967
1694	gB	RET	0.030269
1695	BGLF4	MZT2B	0.19678
1696	BZLF1	BAIAP2	0.084187
1697	BLLF2	ZNF805	0.020718
1698	BMLF1	LY6G6F	0.040492
1699	EBNA-LP	TRAPPC4	0.36126
1700	EBNA3	ATG2A	0.43795

1701	BZLF1	AP1B1	0.20095
1702	BZLF1	MED18	0.1292
1703	EBNA1	KMT2C	0.14645
1704	BGLF4	HBA1	0.15992
1705	BMLF1	SYNE3	0.30441
1706	EBNA3	DHH	0.44068
1707	EBNA3	RPL27A	0.2912
1708	BZLF1	MYCBPAP	0.15498
1709	EBNA3	GPC3	0.20251
1710	EBNA1	XRCC1	0.13844
1711	BLLF2	ILF2	0.011021
1712	BGLF4	RABL6	0.16361
1713	EBNA-LP	PCSK7	0.01826
1714	BMLF1	TRAF7	0.25262
1715	BTRF1	IP6K2	0.014315
1716	BGLF4	AKAP13	0.06458
1717	BZLF2	FASN	0.05664
1718	BGLF4	EFEMP2	0.39626
1719	BGLF4	CTCF	0.035254
1720	EBNA-LP	PCDHB2	0.15367
1721	EBNA-LP	GTF2H1	0.11198
1722	BGLF4	RAD51D	0.14558
1723	gB	EDEM1	0.015289
1724	BGLF4	CDC42EP2	0.13898
1725	BMLF1	SUB1	0.2856
1726	BGLF4	ICAM3	0.14916
1727	BBRF2	ZNF749	0.010833
1728	BLLF2	ZKSCAN4	0.014835
1729	BZLF1	CIB4	0.057416
1730	BZLF1	LEPR	0.18271
1731	EBNA3	CPSF2	0.33597
1732	EBNA1	NAE1	0.10344
1733	BZLF1	STBD1	0.19622
1734	BGLF4	LIMS1	0.14837
1735	BMLF1	SELP	0.13318
1736	EBNA3	THAP3	0.37207
1737	EBNA1	FBXW9	0.22232
1738	BGLF4	ETS1	0.016927
1739	BZLF1	MEF2BNB	0.23593
1740	BZLF1	YWHAQ	0.19532
1741	BMLF1	LIG4	0.084946
1742	BGLF4	TNPO3	0.15133
1743	BGRF1/BDRF1	NOTCH3	0.05712
1744	EBNA-LP	PARD6A	0.17584
1745	BMLF1	SSR3	0.035295
1746	EBNA1	HOOK1	0.11443
1747	BPLF1	CRYGN	0.044772
1748	BGLF4	MFF	0.10086
1749	EBNA3	UTP20	0.28046
1750	BZLF1	AATK	0.12135
1751	EBNA3	MPZL1	0.24115
1752	BVRF1	S100A4	0.16847
1753	BZLF2	LCHN	0.028742
1754	EBNA3	KLHL2	0.39066
1755	EBNA3	JAM2	0.32387
1756	BZLF1	KAT6A	0.055783
1757	BILF1	FAM92A1	0.020522
1758	BGLF4	DSC2	0.053342
1759	BMLF1	SLC15A4	0.024963
1760	EBNA1	ATP6V0D2	0.1335
1761	BMLF1	ARL4A	0.32598
1762	BZLF2	CASC3	0.075634
1763	EBNA-LP	MAN1C1	0.076695
1764	EBNA2	NSUN5P1	0.038075
1765	BLLF2	GG1	0.076717
1766	BGLF4	HRNR	0.048542
1767	EBNA3	SNX24	0.12183
1768	BGLF4	FEZ1	0.14624

1769	EBNA3	NDUFS3	0.25949
1770	EBNA3	MAP1LC3C	0.43298
1771	BMLF1	PPIL1	0.015481
1772	EBNA1	SLAMF1	0.11479
1773	BGLF4	CD5	0.16027
1774	EBNA-LP	FZD3	0.15977
1775	EBNA-LP	OLAH	0.07509
1776	EBNA-LP	EFCAB4B	0.53245
1777	EBNA3	UBQLNL	0.012212
1778	EBNA4	MMS19	0.086767
1779	BMLF1	ECHS1	0.25462
1780	EBNA3	CDC5L	0.29222
1781	EBNA-LP	CNKSR1	0.22822
1782	BGLF4	GPS2	0.18732
1783	BGLF4	NCKAP1	0.13582
1784	EBNA1	SETD2	0.1251
1785	BMLF1	ZNF24	0.19342
1786	BMLF1	EMX2	0.15092
1787	EBNA3	LYRM9	0.11288
1788	BMLF1	EPRS	0.20406
1789	BMLF1	LRP2BP	0.45783
1790	BMLF1	P4HA3	0.014083
1791	EBNA3	BORA	0.26394
1792	BGLF4	SCG5	0.16863
1793	EBNA3	EIF4G2	0.40194
1794	LMP1	SPTAN1	0.035362
1795	EBNA4	EIF2S1	0.037687
1796	BZLF1	SETDB1	0.16056
1797	EBNA3	DCTN3	0.14016
1798	EBNA1	PXN	0.07858
1799	BRRF1	DLL1	0.1666
1800	BMLF1	NAGK	0.44148
1801	EBNA3	CEP97	0.38292
1802	BZLF1	RAP2A	0.095508
1803	BGLF4	TIMM23	0.16233
1804	EBNA3	MED8	0.24332
1805	BLLF2	ZNF236	0.022049
1806	BMLF1	RXRA	0.032436
1807	EBNA3	ASGR2	0.31665
1808	EBNA3	KLRF2	0.37084
1809	EBNA3	ATG4B	0.3258
1810	BGLF4	EIF3J	0.2373
1811	EBNA1	SLX1A	0.13934
1812	BMLF1	SKP1	0.28609
1813	EBNA-LP	COMMD3	0.43184
1814	BPLF1	FAM46B	0.07408
1815	BGLF4	CWC15	0.24985
1816	EBNA1	PSMB1	0.049299
1817	BZLF1	ZNF335	0.11682
1818	EBNA1	DAZL	0.13411
1819	BLLF2	TUBGCP4	0.018949
1820	BVRF1	LDLR	0.043822
1821	EBNA3	MOB3B	0.39642
1822	BVRF1	ATP6V0A1	0.021601
1823	BZLF2	OTUD6B	0.013103
1824	EBNA1	COL7A1	0.15522
1825	BGLF4	TAF9	0.080551
1826	BGLF4	NR3C2	0.086968
1827	BMLF1	PHACTR4	0.029988
1828	BHRF1	C16orf72	0.13836
1829	EBNA1	PCDHGB7	0.038413
1830	gL	MAGEC3	0.0106
1831	BZLF1	MLLT6	0.14543
1832	BZLF1	CD4	0.049904
1833	BZLF1	PIAS1	0.072693
1834	BTRF1	LYZ	0.029627
1835	EBNA-LP	FBL	0.38847
1836	BZLF1	STK40	0.096921

1837	gM	S100A13	0.095666
1838	BALF2	SCUBE3	0.13825
1839	EBNA3	MS4A3	0.37525
1840	BMLF1	EWSR1	0.30984
1841	BLLF2	PPP1R14B	0.048158
1842	BZLF1	SRGN	0.11147
1843	EBNA1	PYDC1	0.14028
1844	BZLF2	LSM12	0.040069
1845	EBNA1	BOP1	0.10556
1846	EBNA6	CCDC127	0.10005
1847	EBNA1	ASF1A	0.10342
1848	BLLF1	GOLGA6L1	0.013414
1849	BZLF1	INTS3	0.18835
1850	BBRF2	EDEM2	0.011942
1851	BZLF1	NBR1	0.21235
1852	BZLF1	RPS26	0.11377
1853	BMLF1	PAGE5	0.021888
1854	BARF1	DLL1	0.15917
1855	EBNA3	TNFSF10	0.17573
1856	BLLF2	RARRES2	0.03376
1857	BLLF2	PGLYRP1	0.015651
1858	EBNA1	KCNG3	0.14557
1859	BMLF1	GJB1	0.014893
1860	EBNA3	NOA1	0.24999
1861	EBNA3	RTN4	0.10855
1862	BMLF1	AGRN	0.1349
1863	BGLF4	STK38L	0.023388
1864	BBRF2	MIS18BP1	0.037047
1865	BHRF1	PPEF2	0.069267
1866	BMLF1	ASCC1	0.043406
1867	BMLF1	AK1	0.098958
1868	EBNA-LP	PPP1R3C	0.26664
1869	BZLF1	FKBP9L	0.046456
1870	LMP2	PLSCR4	0.07492
1871	EBNA3	CENPT	0.24208
1872	BGLF4	PPP1R16B	0.18065
1873	EBNA3	GNA12	0.16995
1874	EBNA1	CAV2	0.065157
1875	EBNA-LP	PNPT1	0.13431
1876	BHRF1	DPP4	0.01595
1877	BGLF4	RFC5	0.07718
1878	BMLF1	KIAA1045	0.23305
1879	EBNA3	STK16	0.16616
1880	BMLF1	WASH1	0.29412
1881	BMLF1	ATP5H	0.084946
1882	gL	GOLGA6L3	0.13843
1883	BGLF4	SPC24	0.14553
1884	EBNA-LP	ZNF133	0.14304
1885	BMLF1	DMXL2	0.048365
1886	BZLF2	PRDX6	0.015043
1887	EBNA3	C2orf62	0.025925
1888	EBNA1	MADD	0.14829
1889	EBNA-LP	USP28	0.25001
1890	BLLF2	MS4A2	0.038843
1891	BZLF1	SGCA	0.053822
1892	BGLF4	PLXNC1	0.13714
1893	BMLF1	RYBP	0.23062
1894	EBNA3	EVI5L	0.33822
1895	EBNA1	PRDX3	0.064956
1896	BMLF1	MAP4K1	0.24604
1897	LMP2	NKAPL	0.047868
1898	BZLF1	TRIAP1	0.17993
1899	BRRF1	THBD	0.13405
1900	EBNA-LP	NOP2	0.47306
1901	EBNA3	GABBR1	0.10574
1902	EBNA1	EDC4	0.10101
1903	EBNA1	HCK	0.051689
1904	EBNA-LP	F12	0.24027

EBNA6	VWA5B2	0.093225
BGLF4	AQP5	0.17253
EBNA1	CETN1	0.32368
BZLF1	ABI3	0.19278
BMLF1	TBL1XR1	0.18368
EBNA3	TARBP2	0.20867
EBNA-LP	SNX17	0.27544
EBNA-LP	STX11	0.3617
BLLF2	LRP2BP	0.016127
EBNA1	APTX	0.046157
BMLF1	PSMC3	0.12198
BGLF4	ASB16	0.22645
BGLF4	CRHR2	0.12467
BMLF1	KISS1	0.43056
BMLF1	TRAF6	0.022837
BARF1	RPH3A	0.098297
BMLF1	ESRRG	0.17424
EBNA-LP	PEX3	0.25069
BVRF1	LINC00205	0.016661
BMLF1	SMG1	0.21154
EBNA3	ATM	0.076098
EBNA3	C19orf35	0.10026
EBNA1	IRF7	0.010439
EBNA-LP	CUL4A	0.2734
BZLF1	HTRA3	0.23477
BRRF1	EPS15	0.086892
EBNA4	MB21D2	0.11293
BGLF4	HSD17B12	0.097129
BGLF4	HMGB1	0.044855
BGLF4	FSHB	0.14103
EBNA3	KCNG3	0.27608
BLLF2	ZNF624	0.011494
BMLF1	TRIM44	0.3959
EBNA1	CCDC53	0.21197
BGLF4	ANXA10	0.065373
BMLF1	ITPR1	0.14217
BPLF1	NBPF24	0.023693
BZLF1	PLS3	0.054177
BGLF4	MYOT	0.17865
BZLF1	DPH3	0.15227
EBNA1	CSTF2	0.17403
EBNA1	NFKBIL1	0.16059
gM	PPEF1	0.036476
BXRF1	RAB11FIP3	0.048488
EBNA3	ZNRF1	0.30143
BGLF4	EIF4EBP2	0.1812
EBNA-LP	RRM2	0.23384
BZLF1	TCHP	0.17047
EBNA1	MTUS2	0.19562
BMLF1	GAPDHS	0.27191
gB	CD93	0.041699
BZLF1	TAF6L	0.072213
BRRF1	SYT1	0.079007
EBNA3	PPP2R1A	0.093107
EBNA-LP	KAT7	0.16755
BLLF2	ZNF566	0.012782
EBNA1	SH3RF1	0.098755
EBNA3	MYLK2	0.075735
BMLF1	PDS5A	0.3611
EBNA3	ADAD2	0.023361
EBNA1	PTK7	0.075192
EBNA4	PEX11B	0.069847
BMLF1	ETF1	0.33215
EBNA3	UPF1	0.2036
EBNA3	C16orf72	0.12008
BGLF4	ELOVL3	0.14532
BZLF1	DPY30	0.13046
EBNA3	E2F4	0.076664

BGRF1/BDRF1	ANXA6	0.1884
EBNA-LP	SLIT1	0.075176
BZLF1	NXT1	0.15151
BBRF2	SLPI	0.048162
BGLF4	MPP6	0.22282
BILF1	S100A8	0.074282
EBNA3	FSHR	0.19031
EBNA-LP	SLC18A1	0.074339
BMLF1	ANTXR1	0.28866
BZLF1	APH1A	0.082296
gL	CNGC19	0.16989
BPLF1	ANGEL1	0.032586
EBNA1	STRN4	0.21219
EBNA1	PCDHB11	0.033158
BDLF1	MICU1	0.023308
BGLF4	FBXL14	0.22492
BMLF1	PEX5	0.15041
BMLF1	KRT1	0.27112
LMP2	NOTCH4	0.017944
BMLF1	EFCAB4A	0.2398
EBNA-LP	FCGBP	0.40593
EBNA1	MZF1	0.032811
EBNA1	TIRAP	0.056749
BHRF1	ACTN4	0.063502
EBNA1	PIP5K1B	0.19323
EBNA-LP	NOXA1	0.30535
EBNA1	SEMA7A	0.13278
EBNA3	SMAD5	0.077908
EBNA1	EHD2	0.20811
BMLF1	SEMA4D	0.12697
EBNA-LP	KHDRBS2	0.34319
BGLF4	CDC73	0.11535
BMLF1	USE1	0.27763
EBNA3	COL18A1	0.15803
BMLF1	EFCAB8	0.26298
gL	C15orf37	0.02466
EBNA3	DDX58	0.09653
BLLF2	CNOT2	0.030229
EBNA-LP	HSPB7	0.32042
EBNA6	CROCCP3	0.045492
EBNA1	UCHL5	0.079714
BRRF1	SETD1A	0.028956
EBNA1	TECPR2	0.22232
BALF2	FAM84A	0.010755
EBNA3	SAYSD1	0.082049
EBNA1	FLT3	0.090042
EBNA6	MED30	0.031709
EBNA3	STAU1	0.32131
BMLF1	STS	0.011281
BLLF2	EIF3CL	0.028101
EBNA3	TNFAIP8L1	0.12611
BLLF2	PIN4	0.010106
BGRF1/BDRF1	LRP1	0.04996
gN	NUCB2	0.07258
BMLF1	MAP3K12	0.14132
EBNA-LP	BCL3	0.033332
EBNA3	PDLIM7	0.25095
EBNA4	EXOSC1	0.11578
EBNA1	INPP5K	0.026221
BMLF1	RHBDL1	0.30109
EBNA3	GYG1	0.3065
EBNA-LP	SLC25A24	0.17996
EBNA3	STK24	0.14273
EBNA3	APLP1	0.099493
BMLF1	GCK	0.097322
BHRF1	NACAP1	0.028606
EBNA-LP	GUCA1A	0.19669
BDLF1	C12orf57	0.011256

2041	EBNA3	ATP1B2	0.27955
2042	EBNA3	PASK	0.18786
2043	BARF1	PRMT2	0.021402
2044	BGLF4	LPCAT2	0.02933
2045	BARF1	EHD3	0.088941
2046	BALF2	PRF1	0.043561
2047	BVRF1	CAPN1	0.05581
2048	BMLF1	CELF5	0.011251
2049	BMLF1	TEFM	0.25728
2050	BZLF1	AMPH	0.1799
2051	EBNA3	PDIA4	0.19927
2052	gM	AP3S1	0.026848
2053	BMLF1	STX1A	0.13583
2054	BZLF1	TNNI3K	0.074527
2055	BGLF4	ITGA3	0.16844
2056	BMLF1	ZNF496	0.17165
2057	EBNA-LP	TOX4	0.34369
2058	BGLF4	ARFIP2	0.16298
2059	BMLF1	DTX3L	0.29634
2060	BMLF1	ANXA11	0.37828
2061	BMLF1	PIK3C2G	0.047059
2062	EBNA3	GBE1	0.075896
2063	EBNA-LP	LLGL2	0.29319
2064	EBNA-LP	SOCS2	0.22112
2065	BBRF2	CASP8AP2	0.035389
2066	EBNA-LP	IPO9	0.31202
2067	EBNA3	PSMC2	0.11456
2068	EBNA3	PCDHB18	0.18006
2069	EBNA3	STRA13	0.27114
2070	BGLF4	CBX4	0.10125
2071	EBNA1	GTF2E2	0.12988
2072	EBNA3	MCFD2	0.14796
2073	EBNA-LP	TGM1	0.35561
2074	BMLF1	PCDHB8	0.22489
2075	EBNA4	CCDC28A	0.35232
2076	BZLF1	CD24	0.051841
2077	BLLF2	TRAPPC4	0.040034
2078	BMLF1	SSTR2	0.18877
2079	BLLF1	LINC00615	0.025266
2080	EBNA3	SERPINC1	0.27076
2081	EBNA-LP	NSMCE1	0.25005
2082	BZLF1	CUL2	0.14362
2083	EBNA6	CAPNS1	0.054699
2084	EBNA1	KHDRBS2	0.19278
2085	BGLF4	BOC	0.14356
2086	BHRF1	YDL022C-A	0.021684
2087	BLLF2	TIMM17B	0.015618
2088	EBNA-LP	PUS7L	0.061464
2089	BILF1	UCKL1	0.01859
2090	BILF1	AP1M1	0.034394
2091	BMLF1	EIF3A	0.27008
2092	BGLF4	NUDT18	0.19641
2093	EBNA3	SOX1	0.18274
2094	BZLF1	FTH1	0.13183
2095	EBNA3	INTS4L2	0.018631
2096	BGLF4	SKI	0.028521
2097	BGLF4	TMEM97	0.15605
2098	EBNA3	PMAIP1	0.10854
2099	BZLF1	CLIC4	0.15292
2100	BBRF2	ATG12	0.01246
2101	BGLF4	NT5C3B	0.16171
2102	EBNA6	GOLGA6L6	0.19157
2103	EBNA-LP	PNLIPRP1	0.068524
2104	BLLF2	TCEANC	0.010546
2105	EBNA4	C9orf171	0.235
2106	BGLF4	CADM1	0.074206
2107	EBNA-LP	DZIP1	0.24093
2108	LMP2	RAB11FIP4	0.15475

2109	BZLF1	MAP1LC3B	0.22235
2110	EBNA1	KRT14	0.078713
2111	EBNA1	MYL3	0.036953
2112	BZLF1	RASSF4	0.22187
2113	EBNA3	SIRT4	0.48424
2114	EBNA2	PLSCR1	0.055875
2115	BZLF1	PCDHB4	0.038452
2116	BXRF1	SLC34A1	0.026972
2117	BGLF4	ZNF133	0.065177
2118	BMLF1	TCF3	0.04656
2119	EBNA3	CAND2	0.30116
2120	BGLF4	ITIH1	0.057215
2121	EBNA1	ACVR1	0.020508
2122	BZLF1	GPR143	0.10068
2123	BLLF2	MIDN	0.033904
2124	EBNA-LP	CHRD	0.21155
2125	BILF1	VIL1	0.093381
2126	BHRF1	SPATA2L	0.067568
2127	BPLF1	EIF4E2	0.01217
2128	EBNA1	PCDHB10	0.035257
2129	BLLF2	TCEB3C	0.054818
2130	EBNA1	NOS1	0.082
2131	BMLF1	SOX6	0.10027
2132	BGLF4	MARK4	0.053347
2133	BPLF1	WIPF1	0.047391
2134	BMLF1	TTLL3	0.044569
2135	BMLF1	GJA8	0.010077
2136	EBNA3	BIRC5	0.065294
2137	BZLF1	FKBP4	0.070721
2138	BMLF1	MESDC1	0.01537
2139	BZLF2	TTC22	0.010112
2140	EBNA6	NOTCH2	0.017149
2141	EBNA3	DEHA2C16280g	0.038073
2142	gL	ATXN7L2	0.048433
2143	EBNA1	PPP6R2	0.17034
2144	BMLF1	RRAD	0.34562
2145	BZLF1	BNIP3L	0.056733
2146	BGRF1/BDRF1	SLC23A1	0.034007
2147	BMLF1	CD164	0.26934
2148	BZLF1	BAZ2A	0.10947
2149	BZLF1	PSIP1	0.12404
2150	BLLF2	ICOS	0.066514
2151	BHRF1	TEX36	0.030751
2152	BZLF1	ATP10D	0.10817
2153	BGLF4	KIFAP3	0.14602
2154	EBNA4	GAGE4	0.1731
2155	EBNA1	PEA15	0.1723
2156	BMLF1	SIGIRR	0.33135
2157	EBNA3	GTF3C1	0.26304
2158	EBNA1	PAF1	0.10588
2159	BGLF4	EPO	0.072015
2160	EBNA-LP	UBXN6	0.31452
2161	BLLF2	PGLYRP2	0.014994
2162	BGLF4	PTRF	0.13227
2163	EBNA-LP	FAM92A1	0.019069
2164	BZLF1	CD22	0.18978
2165	BLLF2	CLINT1	0.010624
2166	BLLF2	STXBP6	0.021182
2167	BLLF1	SH3BP5L	0.023112
2168	BBRF2	G4	0.013628
2169	BLLF2	PPP2R3B	0.012941
2170	EBNA1	SPAST	0.074577
2171	EBNA-LP	TRAM1	0.27028
2172	EBNA1	ZBTB21	0.10154
2173	BZLF1	PCDHB1	0.049103
2174	EBNA-LP	DAG1	0.15134
2175	BXRF1	GRIN1	0.056835
2176	BZLF1	KISS1R	0.10976

2177	BMLF1	EFEMP2	0.62818
2178	BGLF4	C1D	0.17811
2179	BMLF1	PPP1R10	0.022144
2180	BZLF1	CRYAA	0.090159
2181	BLLF2	DCST1	0.053557
2182	EBNA3	CAV2	0.13241
2183	BBRF2	ORC5	0.016065
2184	EBNA6	LRRC14	0.026244
2185	EBNA1	RBPJ	0.012547
2186	EBNA1	USP22	0.070201
2187	BARF1	CFTR	0.022142
2188	BMLF1	RBKS	0.091293
2189	EBNA-LP	UGP2	0.20619
2190	EBNA-LP	SMG1	0.19207
2191	EBNA3	TBC1D8	0.13728
2192	EBNA-LP	UCK1	0.051751
2193	EBNA-LP	AKTIP	0.24962
2194	EBNA6	CALU	0.14396
2195	BHRF1	PRO1854	0.045283
2196	BNLF2b	TBC1D3H	0.012574
2197	EBNA3	CCNG1	0.26444
2198	EBNA1	NEK11	0.063676
2199	BMLF1	PIN1	0.22593
2200	BZLF2	C9orf106	0.011716
2201	BBRF2	CKMT2	0.011923
2202	EBNA1	CRLF1	0.18711
2203	BGLF4	CASQ2	0.053544
2204	EBNA6	STIM2	0.14407
2205	BZLF1	HS3ST3A1	0.035519
2206	BLLF1	NCS1	0.10874
2207	BLLF2	ZNF517	0.010245
2208	BBRF2	ZNF212	0.013928
2209	EBNA-LP	PLP2	0.32372
2210	BMLF1	SOD1	0.039733
2211	EBNA4	HUS1	0.064275
2212	EBNA3	AIFM1	0.1809
2213	BMLF1	GH1	0.23642
2214	EBNA1	EXOSC1	0.17626
2215	BGLF4	CHMP3	0.13562
2216	BMLF1	CHEK1	0.12278
2217	EBNA3	AKIP1	0.37995
2218	BMLF1	NFS1	0.25155
2219	BPLF1	FAM231D	0.13128
2220	BGLF4	IGBP1	0.123
2221	EBNA4	SERTAD1	0.25811
2222	BGLF4	FLOT1	0.15191
2223	BOLF1	PPP2R3A	0.11119
2224	EBNA-LP	FBXO17	0.40094
2225	BMLF1	FTSJ1	0.056358
2226	EBNA-LP	MICAL1	0.33912
2227	BLLF2	OR2M7	0.077525
2228	BZLF1	SH3KBP1	0.20921
2229	EBNA1	TSN	0.2043
2230	BZLF1	ACTR3	0.037902
2231	BZLF1	STK10	0.11503
2232	BGLF4	FCGR2B	0.19002
2233	BVRF1	PROC	0.16264
2234	EBNA3	AP5S1	0.29682
2235	EBNA1	EIF6	0.22897
2236	EBNA1	HEATR5B	0.010923
2237	BZLF2	CELSR3	0.07977
2238	EBNA-LP	PPAP2B	0.10168
2239	BARF1	ANXA11	0.1054
2240	BGLF4	PRAME	0.14685
2241	BGLF4	TOM1	0.18324
2242	BHRF1	SLC23A1	0.018203
2243	BARF1	CDH1	0.040664
2244	EBNA1	GDF7	0.10935

2245	LMP2	PPP3CB	0.01661
2246	EBNA1	BCLAF1	0.1143
2247	BZLF2	INTS4L2	0.016824
2248	EBNA3	AIM2	0.15104
2249	EBNA-LP	PDE6D	0.44329
2250	EBNA1	MED28	0.12855
2251	BLLF2	ZNF233	0.018646
2252	BNLF2b	MCUR1	0.010402
2253	EBNA3	HOXB9	0.13843
2254	gL	pkmyt1	0.090571
2255	BZLF2	GLTSCR1L	0.097571
2256	EBNA3	DUSP26	0.10056
2257	EBNA3	IKBKG	0.096413
2258	EBNA3	NSRP1	0.34349
2259	BHRF1	TAF11	0.083628
2260	BBRF2	CACNA2D1	0.013787
2261	BGLF4	C17orf62	0.22976
2262	BPLF1	PAGE2B	0.16083
2263	BILF1	STIM2	0.1528
2264	EBNA1	CDK5RAP3	0.087936
2265	EBNA1	PCDHAC2	0.036102
2266	EBNA-LP	UBE2E1	0.16428
2267	BMLF1	SCG3	0.066627
2268	EBNA-LP	RNMTL1	0.14844
2269	EBNA3	CEP70	0.29338
2270	EBNA1	ITPR1	0.063124
2271	BMLF1	BARD1	0.21917
2272	gM	CRELD2	0.10498
2273	EBNA-LP	RBM15B	0.27357
2274	BGLF4	RNF20	0.080287
2275	EBNA3	SNX27	0.27435
2276	LMP2	C12orf57	0.025002
2277	EBNA3	DCAF7	0.34485
2278	BZLF2	KIAA1751	0.023816
2279	BZLF1	PCDH12	0.039246
2280	BMLF1	GNMT	0.30969
2281	EBNA-LP	WT1	0.0326
2282	BMLF1	ARNT	0.094838
2283	gN	CANX	0.047903
2284	EBNA-LP	PIN1	0.21216
2285	BGLF4	ECH1	0.13141
2286	EBNA1	PIFO	0.11028
2287	EBNA3	PRKAA1	0.02748
2288	BZLF1	TNNI3	0.060258
2289	BMLF1	FKBP7	0.21661
2290	BGLF4	WDTC1	0.1242
2291	BGLF4	NF2	0.062696
2292	BGLF4	S100A7	0.24533
2293	gL	S100A11	0.048096
2294	EBNA3	PDGFRB	0.074213
2295	BZLF1	SPRED1	0.045942
2296	BMLF1	SYNGR3	0.046121
2297	BLLF2	KCNJ12	0.020594
2298	EBNA3	FAM228A	0.014867
2299	EBNA2	CETN2	0.10413
2300	EBNA3	PTRF	0.2045
2301	EBNA3	PNLIPRP1	0.065986
2302	BGLF4	SLC25A24	0.060535
2303	BBRF2	CLPX	0.017983
2304	gL	DST	0.051166
2305	EBNA3	COMMD7	0.36763
2306	BBRF2	OR4K13	0.037163
2307	BGLF4	SNX1	0.14072
2308	BZLF1	IGFLR1	0.2154
2309	EBNA1	UBE2V1	0.057354
2310	BMLF1	IFNGR2	0.036182
2311	BMLF1	DAZAP1	0.012258
2312	EBNA1	SPPL2B	0.094988

BPLF1	CTU1	0.070081
BMLF1	PCDHGA4	0.21863
EBNA-LP	U2AF1	0.2702
BMLF1	PCDHGA2	0.22489
EBNA-LP	FBXW7	0.10619
BMLF1	MMP24	0.093553
BVRF1	RPUSD4	0.024525
BZLF1	CPSF6	0.1682
EBNA3	ACIN1	0.28452
BPLF1	PRR21	0.039106
EBNA1	DDX17	0.12281
EBNA6	RBBP8	0.081658
EBNA3	EXOSC3	0.25872
BGLF4	GOLGA4	0.15879
EBNA-LP	AP5S1	0.291
BZLF1	WDR61	0.12941
EBNA-LP	DVL2	0.14271
BRRF1	PRF1	0.093121
BMLF1	UBE2L6	0.30875
EBNA4	RNH1	0.19114
EBNA-LP	DLG2	0.22611
BMLF1	CSDE1	0.043078
BLLF1	FAM195A	0.052548
BGLF4	FBN1	0.35201
BARF1	PPEF1	0.11296
BGLF4	NR3C1	0.029415
BMLF1	TFPT	0.30056
BBRF2	PAGE4	0.072818
EBNA-LP	USP4	0.24587
EBNA-LP	SGSM3	0.29323
EBNA-LP	CPNE6	0.19078
BZLF1	PSMC4	0.068776
BLLF2	ST3GAL1	0.013035
BBRF2	ZC3H18	0.058585
EBNA-LP	PCDHGB6	0.17939
BGLF4	SLAIN2	0.22382
BZLF1	KHDRBS2	0.20277
EBNA3	CALML3	0.21072
BGLF4	TAF3	0.12173
BZLF1	ZNF711	0.10686
EBNA-LP	NFKB1	0.031568
BMLF1	TRAPPC2	0.21663
EBNA3	SIX5	0.1652
EBNA4	SIRT4	0.096187
BHRF1	EIF4E	0.018193
EBNA1	FNBP1	0.21059
BMLF1	POLD2	0.2242
EBNA3	MAP4K5	0.22305
EBNA1	CPSF1	0.18158
EBNA3	SYNCRIP	0.21498
BZLF1	GOPC	0.096482
BZLF2	CT45A5	0.028819
BGLF4	HIPK2	0.019013
EBNA1	ANTXR2	0.14356
BMLF1	NELL2	0.54984
BMLF1	EFCAB9	0.26298
EBNA3	CNTNAP1	0.18919
EBNA3	ZNF622	0.24156
EBNA3	NFKBIA	0.067466
EBNA1	ATP8B1	0.13404
EBNA3	PDK3	0.29092
EBNA4	C17orf105	0.13641
EBNA4	ZC3HAV1L	0.22877
BLLF2	NSMCE4A	0.018971
EBNA1	CIB2	0.044917
EBNA1	MBD2	0.090611
BMLF1	ABCA1	0.059401
EBNA4	ELP4	0.14791

2381	EBNA3	TIGIT	0.29082
2382	EBNA3	HOXC13	0.15959
2383	EBNA1	GRB2	0.012679
2384	EBNA3	ZAP70	0.13605
2385	EBNA-LP	MMP8	0.066593
2386	BOLF1	C5orf58	0.023676
2387	EBNA3	PANK4	0.01596
2388	BMLF1	MKL2	0.28536
2389	EBNA1	PRCP	0.20843
2390	EBNA-LP	MED31	0.25139
2391	EBNA4	ARMC5	0.019844
2392	BGLF4	LAPTM4B	0.2194
2393	EBNA4	C11orf96	0.21207
2394	EBNA3	FTH1	0.24351
2395	BLLF2	BRINP3	0.039011
2396	EBNA3	RAD23B	0.2353
2397	BMLF1	MYOZ2	0.37297
2398	EBNA3	MYO9B	0.22461
2399	BNLF2b	ARSD	0.011859
2400	EBNA3	CYTH1	0.24124
2401	BGLF4	BTN3A1	0.19772
2402	BGRF1/BDRF1	CANX	0.093973
2403	EBNA1	AXIN1	0.017253
2404	BMLF1	SVOPL	0.018323
2405	EBNA-LP	CSNK1E	0.13781
2406	EBNA1	CIRH1A	0.14888
2407	BLLF2	TULP3	0.010538
2408	BGLF4	C19orf60	0.24432
2409	BGLF4	TELO2	0.17607
2410	BGLF4	SORBS1	0.07677
2411	BMLF1	RAD18	0.20405
2412	BGLF4	RRAD	0.16848
2413	BZLF1	ZCCHC8	0.14433
2414	EBNA6	MAP7D2	0.064144
2415	EBNA1	C1orf195	0.01723
2416	BGLF4	MYL7	0.075976
2417	EBNA3	C15orf40	0.011532
2418	BMLF1	LAMTOR2	0.29368
2419	BHRF1	GCH1	0.037098
2420	BALF2	C10orf82	0.014942
2421	EBNA-LP	NUP133	0.19071
2422	EBNA1	SAT1	0.18389
2423	EBNA1	PPP2R1B	0.21564
2424	BMLF1	FSTL4	0.25502
2425	BGLF4	LAMTOR5	0.15595
2426	EBNA-LP	VWF	0.20377
2427	EBNA4	FAM195B	0.098413
2428	EBNA1	MS4A3	0.19079
2429	BGLF4	CLPTM1	0.19688
2430	EBNA6	CDH5	0.10117
2431	EBNA-LP	FBXW5	0.19514
2432	EBNA1	VAMP2	0.13035
2433	BLLF1	LETM1	0.096603
2434	BGLF4	CBX2	0.14882
2435	EBNA3	ANXA7	0.44911
2436	BMLF1	VAMP2	0.24617
2437	EBNA-LP	EFNA4	0.42491
2438	BMLF1	ST14	0.02866
2439	BZLF1	RNF43	0.08115
2440	BZLF2	CANX	0.061284
2441	gB	PROC	0.090876
2442	BMLF1	SMOC1	0.19508
2443	EBNA-LP	CIB3	0.2099
2444	EBNA-LP	IGHG1	0.29525
2445	EBNA3	DCHS1	0.17455
2446	BRRF1	MASP2	0.22351
2447	EBNA3	PLA2G2D	0.074695
2448	EBNA3	DDB2	0.25157

2449	BPLF1	C9orf106	0.015916
2450	EBNA-LP	LMX1B	0.14344
2451	EBNA3	ARID1A	0.1756
2452	BZLF1	RBBP4	0.085316
2453	EBNA-LP	KCNJ9	0.35809
2454	BGLF4	ARHGAP26	0.079365
2455	EBNA3	TRIM17	0.28704
2456	BMLF1	KITLG	0.19271
2457	EBNA3	PIK3CA	0.12942
2458	BZLF1	ARHGEF2	0.022611
2459	EBNA3	NCK2	0.21418
2460	EBNA3	STMN2	0.26541
2461	BMLF1	ESPL1	0.22284
2462	BZLF1	IFITM1	0.12915
2463	BMLF1	PARD6B	0.25216
2464	BLLF2	ERVK6	0.016256
2465	EBNA4	TBCD	0.071095
2466	BLLF1	BNIP2	0.036267
2467	BLLF2	ADCK2	0.020499
2468	EBNA3	ELL	0.23975
2469	BRRF1	EIF4G2	0.022292
2470	BGLF4	NR4A1	0.041524
2471	EBNA3	PRSS2	0.49894
2472	EBNA3	CYFIP1	0.21776
2473	EBNA3	KIFC3	0.069687
2474	BLLF2	MS4A7	0.02686
2475	EBNA1	NPNT	0.045466
2476	EBNA3	STAP1	0.15136
2477	EBNA-LP	TIMM23	0.28675
2478	BZLF1	CHMP1A	0.098157
2479	EBNA-LP	PEX11G	0.32817
2480	EBNA3	MARCH8	0.037659
2481	EBNA3	JAM3	0.19462
2482	EBNA2	NELL1	0.064457
2483	BLLF2	Enho	0.01255
2484	BTRF1	LINC00116	0.015948
2485	BGLF4	AFG3L2	0.051011
2486	EBNA3	NLRC5	0.21382
2487	BLLF2	ZNF605	0.017414
2488	BMLF1	ENG	0.079179
2489	EBNA3	FAM20B	0.040978
2490	BMLF1	KAT2B	0.067411
2491	EBNA1	KLRK1	0.10969
2492	BGLF4	GRB7	0.077933
2493	BMLF1	SCAF1	0.019954
2494	BZLF1	RPS9	0.10212
2495	BMLF1	TH	0.035619
2496	BPLF1	BNIP2	0.059539
2497	EBNA3	VWF	0.207
2498	BNLF2b	AP3S1	0.012913
2499	BZLF1	MSN	0.055154
2500	BMLF1	TXNDC9	0.43141
2501	BZLF1	UXT	0.096091
2502	BMLF1	MAPK6	0.19057
2503	EBNA3	COL1A2	0.17807
2504	EBNA3	KBTBD6	0.40098
2505	BLLF2	ZNF501	0.015587
2506	LMP1	DLL1	0.076892
2507	EBNA4	MNCb-2875	0.09007
2508	EBNA1	HOOK3	0.11022
2509	EBNA1	IGSF8	0.17252
2510	BALF2	CIB1	0.09948
2511	BPLF1	C19orf44	0.033646
2512	EBNA-LP	ATXN7L3	0.23675
2513	EBNA3	CAPS2	0.21072
2514	BMLF1	SGCE	0.18524
2515	EBNA1	FCGR2B	0.17603
2516	BMLF1	WWC1	0.24023

2517	BLLF2	ZNF680	0.013452
2518	EBNA3	CASP7	0.17514
2519	BMLF1	THBS2	0.58898
2520	BMLF1	ZC3HC1	0.23719
2521	EBNA3	LCAT	0.25082
2522	EBNA1	OS9	0.1691
2523	EBNA3	SSR3	0.049131
2524	BHRF1	PLN	0.1123
2525	BZLF1	ZNF256	0.03069
2526	BLLF2	MAD2L1	0.012912
2527	EBNA3	GDF2	0.15691
2528	EBNA3	SMAGP	0.045842
2529	BGLF4	EFCAB6	0.045351
2530	EBNA1	ZNF212	0.088082
2531	EBNA3	ARF6	0.13002
2532	BZLF1	RFC2	0.072302
2533	EBNA3	UCKL1	0.3846
2534	EBNA3	DPH3	0.30354
2535	BXRF1	PRMT1	0.018719
2536	BMLF1	PIP4K2A	0.020071
2537	BMLF1	CDH23	0.30401
2538	BMLF1	SLC17A3	0.028768
2539	EBNA3	UBA6	0.23007
2540	BLLF2	SMYD5	0.063499
2541	BGLF4	LALBA	0.046075
2542	BMLF1	PRSS22	0.049094
2543	BGLF4	NCSTN	0.07939
2544	EBNA1	SSBP1	0.15297
2545	BZLF1	NLRP12	0.12132
2546	BPLF1	UHRF1BP1L	0.081889
2547	EBNA-LP	TAOK3	0.20968
2548	EBNA1	B2M	0.099432
2549	EBNA-LP	FAS	0.053148
2550	EBNA-LP	CHORDC1	0.29723
2551	EBNA-LP	DIAPH1	0.1428
2552	BPLF1	TCERG1L	0.042925
2553	BLLF1	CCDC132	0.012542
2554	BLLF1	ATP5B	0.053498
2555	BGLF4	NEFL	0.12171
2556	BMLF1	OXNAD1	0.019312
2557	EBNA1	ANXA1	0.047609
2558	BZLF1	CCNG1	0.14063
2559	EBNA-LP	MICU1	0.53755
2560	EBNA-LP	CREB5	0.14315
2561	BBRF2	SEC11C	0.043779
2562	BMLF1	FGFR3	0.060507
2563	BTRF1	PRAME	0.040956
2564	BZLF1	PELP1	0.176
2565	BMLF1	SETD6	0.405
2566	EBNA-LP	EHMT2	0.23727
2567	EBNA3	CIB4	0.21072
2568	EBNA-LP	ATP7B	0.096429
2569	EBNA6	CCDC122	0.011577
2570	EBNA1	SEPT7	0.015057
2571	BMLF1	TEAD2	0.18356
2572	EBNA3	AGTRAP	0.3693
2573	EBNA3	OGG1	0.11499
2574	EBNA1	DNAJC21	0.16309
2575	BARF1	MAGOH	0.026178
2576	BGLF4	NDRG2	0.10685
2577	BMLF1	EDF1	0.16837
2578	gB	SNX9	0.025824
2579	EBNA4	C6orf165	0.025661
2580	EBNA-LP	DDX39A	0.26889
2581	EBNA4	MAB21L3	0.050262
2582	EBNA1	TOX3	0.12707
2583	EBNA-LP	SYT8	0.020182
2584	BALF2	LSM12	0.016988

2585	BMLF1	EPHA3	0.16666
2586	BZLF1	AIG1	0.2221
2587	EBNA3	PFKM	0.17366
2588	BMLF1	ZDHHC8	0.14365
2589	EBNA-LP	BMF	0.2365
2590	EBNA-LP	ETV1	0.15072
2591	BZLF1	MYLK2	0.028728
2592	BZLF1	REEP1	0.17484
2593	BZLF1	TPCN1	0.096928
2594	gL	PIH1D2	0.12585
2595	BHRF1	C1orf140	0.11432
2596	BZLF1	TAX1BP3	0.15383
2597	BZLF1	USP2	0.075027
2598	BLLF1	C22orf43	0.012469
2599	BZLF1	AQP5	0.17274
2600	EBNA1	ITGA3	0.15459
2601	EBNA3	CAMKK2	0.062245
2602	BGLF4	RFX6	0.13793
2603	BMLF1	TCIRG1	0.026705
2604	EBNA3	KLHL3	0.22481
2605	BPLF1	PTGES3L	0.032997
2606	BLLF2	ZNF679	0.013157
2607	BMLF1	PARP14	0.032664
2608	EBNA1	THOC5	0.15383
2609	BMLF1	EIF5A	0.13752
2610	BZLF1	WARS	0.092206
2611	BLLF1	DST	0.061246
2612	gL	PRR21	0.060066
2613	BMLF1	RPP25	0.37633
2614	BALF2	C1orf64	0.031099
2615	BZLF1	EHD3	0.24812
2616	BLLF2	POLG	0.060417
2617	BILF1	C1orf195	0.16725
2618	BMLF1	SF3B5	0.038072
2619	BZLF1	LDHAL6B	0.18965
2620	EBNA1	RASGRP2	0.029322
2621	EBNA3	FRS2	0.1681
2622	EBNA1	MATN2	0.34199
2623	LMP1	S100A13	0.080304
2624	BVRF1	PPEF1	0.073969
2625	EBNA1	SLC4A4	0.18348
2626	BLLF2	ZFP37	0.016196
2627	BGLF4	ITGB1BP1	0.19053
2628	EBNA1	SEC13	0.13369
2629	BGRF1/BDRF1	PIK3CD	0.013998
2630	EBNA3	KIAA1751	0.018255
2631	BBRF2	TBC1D3G	0.01421
2632	BMLF1	TNFSF14	0.22488
2633	EBNA3	EEF2K	0.081289
2634	BZLF1	FKBP9	0.061867
2635	EBNA1	RERE	0.04241
2636	BMLF1	GTF2F1	0.14705
2637	EBNA3	CRIPAK	0.32385
2638	BMLF1	EPS15	0.33482
2639	EBNA-LP	ZFYVE26	0.31238
2640	BMLF1	LONRF3	0.37965
2641	BVRF1	C1orf196	0.015899
2642	BMLF1	SPACA5	0.048305
2643	EBNA2	SDF4	0.095196
2644	BZLF2	YML009W-B	0.091747
2645	EBNA3	GORASP2	0.32506
2646	BHRF1	SSUH2	0.012027
2647	BLLF2	GRHL1	0.016789
2648	BPLF1	C9orf169	0.028259
2649	EBNA1	INVS	0.13681
2650	BXRF1	SPTAN1	0.08549
2651	BGLF4	DHX8	0.1528
2652	BMLF1	NAE1	0.26999

2653	EBNA-LP	PDIA6	0.16437
2654	BGLF4	AQP2	0.012277
2655	BMLF1	DROSHA	0.24249
2656	EBNA-LP	PPARGC1A	0.094892
2657	BGLF4	EMR4P	0.032001
2658	BPLF1	LYRM9	0.10431
2659	BZLF1	FANCI	0.17662
2660	BGLF4	ZNF219	0.048066
2661	BGLF4	CCDC53	0.23222
2662	EBNA3	MSR1	0.32945
2663	BGLF4	CENPH	0.099326
2664	EBNA-LP	LATS1	0.071774
2665	BARF1	TESC	0.13156
2666	BMLF1	RHO	0.19427
2667	EBNA-LP	CHMP4A	0.10456
2668	EBNA6	WIPF2	0.12547
2669	EBNA-LP	EEF1E1	0.28402
2670	EBNA-LP	PPP1R27	0.38588
2671	BMLF1	IRAK2	0.10756
2672	BMLF1	PACSIN2	0.080214
2673	BMLF1	TAAR3	0.043938
2674	BZLF2	NCS1	0.13774
2675	EBNA1	VCL	0.076113
2676	BHRF1	SECISBP2L	0.10405
2677	BILF1	EML1	0.2104
2678	EBNA-LP	CDH22	0.17939
2679	BGLF4	CCL18	0.18397
2680	BZLF1	GTF2A1	0.097904
2681	BLLF2	SAMD4B	0.042054
2682	BLLF2	NUFIP1	0.011259
2683	BGLF4	TPP1	0.10933
2684	EBNA3	TAF9B	0.20142
2685	gL	MACF1	0.049095
2686	gL	PRSS2	0.085398
2687	BMLF1	SNW1	0.23761
2688	EBNA1	SELL	0.13122
2689	EBNA1	HDAC9	0.031902
2690	EBNA6	LYRM9	0.19779
2691	BGLF4	SCGN	0.12582
2692	EBNA3	C3orf65	0.068957
2693	BGLF4	MMP2	0.059439
2694	BBRF2	ZNF354A	0.01139
2695	BGLF4	PPP2R2B	0.11115
2696	BGLF4	PXN	0.089071
2697	BBRF2	MAGEL2	0.013015
2698	EBNA3	SERTAD1	0.33216
2699	EBNA3	SPDL1	0.20352
2700	BZLF1	GTF2E1	0.11091
2701	EBNA3	TIRAP	0.104
2702	EBNA3	THOC5	0.28057
2703	BGLF4	TNNC2	0.057678
2704	EBNA-LP	PDCD1	0.31636
2705	BMLF1	VCPKMT	0.05621
2706	EBNA6	CELSR3	0.10332
2707	BMLF1	HSF1	0.15867
2708	BZLF1	SLC4A1	0.13214
2709	EBNA1	IRAK1	0.033918
2710	BILF1	ADAR	0.016474
2711	BLLF2	KCNJ16	0.018168
2712	gM	PPP3CB	0.016708
2713	EBNA3	FNTA	0.29004
2714	EBNA1	TOM1	0.16781
2715	BGLF4	SOS2	0.086673
2716	gM	EGF	0.031456
2717	EBNA3	CAPNS2	0.19223
2718	BPLF1	C9orf153	0.028038
2719	EBNA3	SUZ12	0.20411
2720	BZLF1	OCIAD1	0.21121

BGLF4	CALN1	0.055551
BZLF1	RAC1	0.024267
BMLF1	GMIP	0.3068
BPLF1	EIF6	0.033352
EBNA4	HEATR5B	0.19034
BMLF1	RIPK2	0.042042
BMLF1	PARN	0.21986
LMP2	LTBP4	0.086601
BMLF1	AES	0.21248
BGLF4	MYCBPAP	0.17229
EBNA3	RNF146	0.21865
BMLF1	MAFB	0.16477
EBNA3	MMS22L	0.34104
BGRF1/BDRF1	EFEMP2	0.20954
EBNA1	PITPNC1	0.21541
EBNA-LP	TLN2	0.16004
EBNA-LP	SDC4	0.22021
EBNA3	SAT1	0.33232
EBNA-LP	ATP5A1	0.19562
BGLF4	BAG6	0.10244
BZLF1	TPO	0.085997
BZLF1	FAM175A	0.18244
BZLF1	HSPA1A	0.05326
BMLF1	EME1	0.31914
BMLF1	BRAP	0.28796
BZLF1	ARL3	0.036304
EBNA-LP	SETD1A	0.31885
EBNA3	NMRK2	0.39249
EBNA-LP	PFN1	0.18989
gL	GAGE4	0.092213
EBNA3	PCSK1	0.021199
EBNA3	CYBA	0.089827
BMLF1	PACSIN3	0.35488
EBNA1	SPARCL1	0.052509
EBNA1	PIK3R4	0.083544
BZLF1	DCPS	0.084124
EBNA3	GCKR	0.22865
EBNA1	KRT15	0.15171
EBNA-LP	PPP1R16B	0.3273
BGLF4	KAT6A	0.076368
EBNA-LP	NAA50	0.44093
EBNA1	FCN3	0.14426
BZLF1	ASUN	0.18357
EBNA3	YAF2	0.22658
LMP1	F2	0.018336
BBRF2	FAM210A	0.09568
BMLF1	FAN1	0.27843
EBNA3	HSPA8	0.058284
BGLF4	STAP1	0.069804
EBNA3	LRRK2	0.0456
EBNA3	CCDC26	0.081707
BZLF2	FAM117A	0.063207
EBNA-LP	KCNJ4	0.36331
BZLF1	GLA	0.074097
BZLF1	ELF2	0.042722
EBNA3	DCUN1D1	0.4117
EBNA3	NME2	0.10624
BMLF1	F9	0.20617
EBNA1	PRRG4	0.037952
EBNA-LP	RAD23A	0.25216
BLLF1	RNF216P1	0.020939
EBNA6	CCDC85B	0.099121
EBNA-LP	SLC9A3R1	0.10403
BZLF1	ARRB2	0.053594
EBNA1	SIRPG	0.14703
EBNA1	AMER2	0.21447
BMLF1	MED26	0.26108
BZLF1	CTTN	0.19532

2789	EBNA6	CLIC4	0.062888
2790	BZLF1	APOH	0.094885
2791	BMLF1	FAM231A	0.2152
2792	LMP2	NOTCH3	0.016329
2793	BMLF1	DLG2	0.25199
2794	EBNA1	CCP110	0.16367
2795	EBNA4	SETD1A	0.034895
2796	EBNA3	TBK1	0.10783
2797	EBNA1	ZAK	0.037617
2798	EBNA3	SLX1A	0.27989
2799	BZLF1	RPSA	0.062468
2800	BPLF1	RPUSD4	0.053393
2801	BZLF1	SPATA13	0.050515
2802	EBNA-LP	DNASE1L2	0.16649
2803	EBNA-LP	PTPN4	0.1472
2804	EBNA1	ADAMTS13	0.15919
2805	EBNA6	MUC1	0.18999
2806	BLLF2	LVP1a	0.034644
2807	BGLF4	ATP1A1	0.066038
2808	BMLF1	AP2A1	0.18276
2809	EBNA3	CCDC162P	0.014484
2810	BZLF1	KCNH1	0.1076
2811	EBNA1	PINK1	0.013571
2812	BNLF2b	RABEP1	0.011122
2813	BTRF1	DEFB129	0.047948
2814	EBNA-LP	AK8	0.059704
2815	BHRF1	GAGE3	0.033027
2816	BGLF4	KIAA1524	0.21609
2817	EBNA-LP	PRDX1	0.25697
2818	EBNA3	RAD21	0.21484
2819	EBNA1	SPI1	0.013375
2820	BLLF2	NBPF4	0.03035
2821	BZLF1	GAPDH	0.11786
2822	BGLF4	NOM1	0.23589
2823	BGLF4	DERL2	0.15196
2824	BGLF4	TNFRSF21	0.19611
2825	EBNA4	PDCD6	0.2254
2826	gN	VIL1	0.056235
2827	EBNA4	FAM181A	0.088806
2828	EBNA1	LENG8	0.22232
2829	BGLF4	PHF2	0.13128
2830	EBNA-LP	ROCK2	0.024862
2831	EBNA3	SHCBP1	0.2588
2832	BMLF1	UFD1L	0.35691
2833	EBNA3	RAD51B	0.28286
2834	BNLF2b	ELOVL4	0.010836
2835	BGLF4	SPTSSB	0.15482
2836	BMLF1	RNF111	0.18163
2837	BXRF1	KIAA1841	0.024102
2838	gM	LDLR	0.025838
2839	EBNA3	ATPAF2	0.3992
2840	EBNA3	SCAF11	0.28174
2841	BPLF1	SNX29P2	0.036087
2842	EBNA6	C22orf26	0.16835
2843	BMLF1	CUEDC2	0.40475
2844	EBNA-LP	ACVR1B	0.066975
2845	EBNA3	CELF1	0.2695
2846	EBNA3	PROCR	0.31235
2847	BMLF1	HTN1	0.40527
2848	BARF1	PDCD6	0.23588
2849	EBNA3	CIB3	0.21072
2850	EBNA-LP	KSR1	0.10601
2851	EBNA3	STAT5A	0.057682
2852	EBNA1	LIN28B	0.13569
2853	BGLF4	CENPI	0.11162
2854	BMLF1	ITGA3	0.32167
2855	EBNA2	CNRIP1	0.011118
2856	EBNA3	CLN5	0.282

2857	EBNA3	SGOL1	0.19463
2858	BZLF1	FLT1	0.027895
2859	BGLF4	HPN	0.10021
2860	EBNA1	RABGEF1	0.12001
2861	BMLF1	GOLM1	0.41701
2862	BZLF1	NMNAT1	0.16142
2863	BZLF1	RASSF3	0.17431
2864	BMLF1	SNRNP35	0.011854
2865	BLLF2	DMRTC2	0.027308
2866	EBNA6	TSGA13	0.010057
2867	EBNA3	ADAMTS10	0.20644
2868	BGLF4	SMURF1	0.075079
2869	BLLF2	POM121B	0.082521
2870	BZLF2	KIAA1407	0.036402
2871	BZLF1	CMKLR1	0.12428
2872	BZLF1	EPB42	0.12553
2873	BGLF4	PA2G4	0.073046
2874	EBNA6	C22orf43	0.12302
2875	BLLF2	SAMD4A	0.034243
2876	BLLF1	FAM101A	0.025889
2877	EBNA4	SLIT2	0.041832
2878	EBNA1	TPD52L2	0.22099
2879	EBNA1	VRK2	0.050394
2880	BOLF1	ANXA11	0.083172
2881	BGLF4	PIGA	0.15929
2882	EBNA1	KCNH1	0.10178
2883	EBNA1	SLC25A23	0.042153
2884	EBNA3	SERTAD3	0.31066
2885	EBNA2	SLIT2	0.026725
2886	EBNA1	DTL	0.11626
2887	EBNA1	CGREF1	0.03581
2888	EBNA-LP	FBXO3	0.35937
2889	EBNA6	KHDC1L	0.030325
2890	EBNA-LP	OLA1	0.27754
2891	EBNA3	HMGA2	0.015707
2892	EBNA1	ABCD2	0.14212
2893	BMLF1	EIF4E	0.26438
2894	EBNA4	ZNF728	0.030849
2895	EBNA3	FGF3	0.25148
2896	EBNA3	APOO	0.089971
2897	EBNA3	SIN3B	0.23193
2898	BXRF1	C10orf82	0.016812
2899	BBRF2	OAZ2	0.034248
2900	BMLF1	SNX11	0.019801
2901	BXRF1	TPD52	0.17813
2902	EBNA3	PMS2P1	0.019847
2903	BZLF1	CHP1	0.14306
2904	BGLF4	EDC4	0.12472
2905	BGLF4	BAG1	0.11316
2906	BGLF4	LTBR	0.1481
2907	EBNA3	VSNL1	0.21072
2908	EBNA4	TPPP	0.15202
2909	BGLF4	COMMD2	0.24432
2910	EBNA-LP	PDGFRA	0.037477
2911	BHRF1	AMOT	0.027299
2912	BGLF4	CTSB	0.048487
2913	BGLF4	NUTF2	0.20372
2914	BVRF1	C1orf64	0.067348
2915	BGLF4	AURKB	0.018856
2916	EBNA3	LEP	0.012193
2917	EBNA4	COMMD2	0.23223
2918	EBNA1	HFE	0.15016
2919	BZLF1	RUSC1	0.15536
2920	EBNA-LP	ITGA2	0.28082
2921	EBNA3	PTGES3L	0.03991
2922	EBNA-LP	PRMT8	0.31201
2923	EBNA1	PI4KA	0.19756
2924	BTRF1	ETNK1	0.01968

2925	EBNA1	COMP	0.30496
2926	EBNA1	MAP2K2	0.022026
2927	BZLF1	NOTCH2NL	0.049691
2928	BGLF4	FAP	0.13669
2929	BTRF1	ELOVL4	0.023581
2930	BGLF4	ILKAP	0.11465
2931	EBNA-LP	ERAP1	0.17401
2932	EBNA3	DHX8	0.30581
2933	EBNA1	TESK1	0.10328
2934	BRRF1	SPTAN1	0.11053
2935	BGLF4	ELOVL6	0.14563
2936	EBNA-LP	PCDHB4	0.14275
2937	EBNA1	LRRK2	0.018844
2938	BLLF2	CHMP2A	0.024531
2939	EBNA1	FGF2	0.018053
2940	BLLF2	PHYH	0.084971
2941	BMLF1	GID4	0.017481
2942	EBNA1	ITCH	0.072151
2943	EBNA-LP	TERF2IP	0.14185
2944	gM	COMP	0.078207
2945	BMLF1	QKI	0.29076
2946	EBNA3	PPP2R5B	0.24207
2947	EBNA-LP	UBE2D1	0.14318
2948	BHRF1	PPP3CA	0.023935
2949	BGLF4	EIF4B	0.21004
2950	BZLF1	SLC4A4	0.19183
2951	EBNA-LP	POLI	0.21509
2952	BMLF1	IRAK3	0.12156
2953	BMLF1	HGF	0.17304
2954	EBNA-LP	ZNF350	0.24636
2955	EBNA1	MEGF8	0.31258
2956	BGLF4	SHANK2	0.20255
2957	EBNA3	PIGT	0.25528
2958	EBNA1	AIM2	0.064708
2959	EBNA1	RTN1	0.16432
2960	BZLF2	BNIP2	0.06602
2961	BGLF4	RPP38	0.1717
2962	BGLF4	ZFAND6	0.13809
2963	EBNA-LP	TMPO	0.32076
2964	EBNA1	EGFR	0.014162
2965	EBNA1	NOM1	0.20949
2966	BGLF4	ACVR1B	0.020409
2967	gL	CNRIP1	0.034193
2968	BMLF1	SF3A3	0.26488
2969	EBNA1	BAG3	0.16264
2970	BMLF1	TPD52L2	0.44306
2971	BPLF1	CCDC120	0.073205
2972	BZLF1	MASP1	0.37056
2973	BZLF1	HSPG2	0.079365
2974	BZLF1	MCTP1	0.052239
2975	BMLF1	ELOVL3	0.28786
2976	EBNA-LP	CABS1	0.17471
2977	BHRF1	NPIPA5	0.013348
2978	BZLF1	KDM1A	0.012583
2979	BZLF1	DMC1	0.088519
2980	EBNA4	smf	0.067553
2981	BLLF2	ZBTB24	0.015662
2982	BLLF2	ZFP28	0.012549
2983	EBNA-LP	KEL	0.23009
2984	EBNA3	MED12	0.037242
2985	EBNA2	C2orf81	0.017973
2986	BMLF1	RPS23	0.23306
2987	BMLF1	YWHAB	0.15265
2988	BGLF4	UPF3A	0.20577
2989	BBRF2	ALKBH2	0.041019
2990	BMLF1	TRIM5	0.21572
2991	EBNA3	CTNND1	0.14304
2992	BPLF1	SH3KBP1	0.018109

2993	EBNA-LP	BCAS2	0.33412
2994	EBNA1	MYO1E	0.15032
2995	EBNA-LP	CRMP1	0.13921
2996	EBNA-LP	TEAD2	0.15651
2997	EBNA3	SLC14A2	0.3369
2998	BZLF2	C8orf74	0.03072
2999	BZLF1	CETN1	0.3321
3000	BZLF1	TTC8	0.18007
3001	BLLF1	MSANTD1	0.010725
3002	EBNA3	KIAA0319L	0.39912
3003	EBNA1	MYH7	0.14355
3004	BMLF1	USP37	0.2121
3005	gL	ITFG2	0.02055
3006	EBNA1	RAD54L	0.098322
3007	BGLF4	TULP3	0.15901
3008	EBNA3	CD2BP2	0.32781
3009	EBNA3	KANK2	0.29017
3010	BZLF1	SETMAR	0.12803
3011	BLLF2	OR2M5	0.07986
3012	BALF2	PPEF1	0.048182
3013	EBNA4	C6orf52	0.028737
3014	BZLF1	EID1	0.12211
3015	EBNA3	CDK6	0.032225
3016	EBNA3	FBXL14	0.40178
3017	EBNA4	N4BP2L1	0.05374
3018	EBNA3	ZFP36L1	0.10784
3019	BBRF2	ZNF350	0.012756
3020	BZLF1	TPR	0.094462
3021	EBNA3	CDK5R1	0.088998
3022	EBNA1	SPARC	0.17459
3023	EBNA4	PNAS-138	0.2117
3024	EBNA1	CATSPER1	0.11114
3025	EBNA1	CPS1	0.036016
3026	BRRF1	CRELD2	0.21739
3027	BMLF1	ZNF256	0.17833
3028	EBNA4	LINC00303	0.062425
3029	BMLF1	ALDH9A1	0.065128
3030	BZLF1	SLPI	0.17079
3031	BBRF2	SLC12A3	0.014484
3032	EBNA3	BBS7	0.2932
3033	BGLF4	RRM2	0.1327
3034	BZLF1	EHD4	0.22676
3035	BMLF1	SLC6A11	0.022762
3036	BZLF2	EP400NL	0.072281
3037	BZLF1	UMOD	0.066802
3038	BLLF1	IQCK	0.030662
3039	EBNA1	ZNF326	0.15414
3040	BHRF1	ytzL	0.1574
3041	EBNA3	GAGE2A	0.061456
3042	BPLF1	CT45A4	0.025984
3043	gM	CETN2	0.092477
3044	EBNA3	TNFSF13B	0.18407
3045	EBNA-LP	FIG4	0.17107
3046	BZLF2	FAM83E	0.010867
3047	BGLF4	CDC16	0.12134
3048	BMLF1	IRAK4	0.18064
3049	gL	MAGEB4	0.020545
3050	BZLF1	ACTR8	0.16947
3051	BGLF4	EFCAB13	0.042785
3052	gN	RPH3A	0.043364
3053	EBNA-LP	TFAP2A	0.019761
3054	EBNA-LP	AFF4	0.17759
3055	EBNA1	MCM5	0.081734
3056	BLLF2	VPREB3	0.050086
3057	EBNA-LP	NR0B2	0.12907
3058	EBNA-LP	PRNP	0.10421
3059	EBNA3	MCL1	0.18529
3060	BPLF1	CROCCP3	0.023126

3061	EBNA-LP	CHAF1B	0.21313
3062	BMLF1	ARHGAP22	0.17745
3063	EBNA3	WAC	0.25848
3064	EBNA3	EFCAB12	0.21072
3065	BPLF1	FAM188A	0.11036
3066	EBNA1	SATB1	0.03363
3067	EBNA-LP	CLVS1	0.045167
3068	BLLF2	CD300LF	0.013377
3069	BZLF1	MAPK10	0.05683
3070	EBNA-LP	KIAA0226	0.31587
3071	BMLF1	SDR42E2	0.021214
3072	BGLF4	SLC11A2	0.0415
3073	EBNA3	FUS	0.31027
3074	BGLF4	THBS4	0.042737
3075	BMLF1	HSP90AA1	0.1938
3076	BZLF1	RHOB	0.041405
3077	EBNA3	SUN1	0.24478
3078	EBNA-LP	SCIMP	0.33154
3079	EBNA-LP	APH1A	0.17763
3080	EBNA3	UBE2B	0.11159
3081	BLLF2	ZNF425	0.017329
3082	BGLF4	ARHGAP1	0.11422
3083	EBNA-LP	SHANK2	0.34013
3084	BLLF2	XPO5	0.021053
3085	BGLF4	ABCB1	0.10975
3086	BZLF2	XAGE5	0.06143
3087	BZLF1	AKAP17A	0.18643
3088	BGLF4	RCN2	0.063812
3089	BMLF1	CRHBP	0.13549
3090	BMLF1	CCDC150	0.015575
3091	EBNA3	MADD	0.29165
3092	BMLF1	SLC4A4	0.32001
3093	EBNA3	CHMP1B	0.31316
3094	EBNA6	MSANTD1	0.050284
3095	BPLF1	CNPY1	0.01472
3096	BMLF1	SCARB1	0.010474
3097	BMLF1	NF2	0.12722
3098	BZLF1	UBE2L3	0.057949
3099	EBNA3	ODF2	0.15961
3100	BLLF2	GNS	0.014603
3101	EBNA3	UBE2D2	0.22062
3102	BLLF1	BEND2	0.017261
3103	BMLF1	OLAH	0.12882
3104	BLLF2	SLC16A6	0.027933
3105	BGLF4	KCNA4	0.15294
3106	BMLF1	ATP6V0A4	0.18011
3107	BLLF1	GOLGA6L20	0.015308
3108	EBNA4	CLPX	0.18648
3109	BZLF1	MKKS	0.058392
3110	BGLF4	FSCB	0.058136
3111	BMLF1	ATG12	0.37416
3112	BILF1	CALR	0.02311
3113	BGLF4	LCP2	0.18829
3114	EBNA1	PRSS3	0.36715
3115	BGLF4	SORCS1	0.20687
3116	BZLF1	MRPS31	0.16698
3117	BZLF1	MMRN2	0.21643
3118	BLLF1	UNQ6126/PRO20091	0.022215
3119	BVRF1	ANXA2	0.082931
3120	BZLF1	TRAPPC2	0.095703
3121	EBNA4	EIF1AY	0.041158
3122	EBNA3	ATP11B	0.14526
3123	EBNA3	HSCB	0.3524
3124	EBNA-LP	DRD2	0.020188
3125	BMLF1	KLHL2	0.41504
3126	BGLF4	IGFBP2	0.15554
3127	EBNA-LP	EFCAB7	0.2099
3128	EBNA4	CCDC17	0.050755

EBNA4	GOLGB1	0.14793
EBNA1	CHMP1B	0.15724
BMLF1	NOVA1	0.048327
BXRF1	LRP4	0.092692
BLLF2	MACC1	0.067694
EBNA-LP	NOSIP	0.25835
EBNA3	SMO	0.016122
LMP2	CDK5R1	0.027009
BBRF2	GLTP	0.015538
BGLF4	AMOTL1	0.20935
EBNA3	UBE3A	0.15908
EBNA-LP	ARNT	0.073006
EBNA3	FBXO3	0.36406
BGLF4	S100A8	0.14738
BZLF2	PITPNM3	0.12423
BLLF1	ubl4ab	0.034653
EBNA3	CST3	0.094652
BZLF1	DSG4	0.037964
EBNA4	WLS	0.10118
EBNA-LP	GPD2	0.12815
BGLF4	LASP1	0.14036
BGLF4	SNX33	0.22975
BGLF4	BUB3	0.10319
EBNA3	BNIP3L	0.14016
BZLF1	DEDD	0.12354
BALF2	NKD1	0.1014
EBNA6	SSSCA1	0.13029
BGLF4	TP53RK	0.075877
BHRF1	NPC1L1	0.039375
EBNA-LP	PCBD1	0.14742
BOLF1	TCAP	0.024778
EBNA-LP	ARFGEF1	0.14642
EBNA-LP	ZNHIT6	0.31769
BZLF1	NOTCH1	0.012155
BZLF1	TNFRSF10A	0.13355
BGRF1/BDRF1	S100A8	0.076514
BRRF1	AMBRA1	0.012975
gB	ATP5B	0.03899
gM	MASP2	0.12287
BMLF1	PLA2G2D	0.11501
BMLF1	CATSPER1	0.2159
EBNA3	CRP	0.26209
BMLF1	TGM6	0.0243
EBNA-LP	FNTA	0.28524
EBNA4	UXT	0.10413
BLLF2	ZSCAN10	0.015997
BGLF4	FBXO5	0.051703
EBNA-LP	PTPRO	0.087895
EBNA3	PRMT7	0.028011
BGLF4	LY96	0.12207
EBNA-LP	GH1	0.21817
BMLF1	GNA13	0.15721
BMLF1	S100A1	0.47526
BGLF4	KAT6B	0.088653
BMLF1	BMP10	0.16534
BLLF1	HSP90B1	0.063219
EBNA1	CLIC1	0.10773
BZLF1	LPHN1	0.12936
EBNA-LP	LCMT1	0.45024
EBNA1	KLC1	0.17228
EBNA-LP	CGREF1	0.17207
BGLF4	CIRBP	0.15006
gL	EFEMP1	0.082709
EBNA1	INHBA	0.03275
EBNA3	DTL	0.24074
BMLF1	STRA13	0.28684
BMLF1	CST1	0.034289
BMLF1	GPX8	0.094397

3197	BLLF1	PLSCR3	0.082972
3198	EBNA1	CACNA1C	0.098484
3199	BGLF4	PSMA3	0.053606
3200	BMLF1	BDKRB2	0.16828
3201	EBNA4	SHANK2-AS3	0.086981
3202	BZLF1	ADAMTS13	0.16527
3203	BZLF1	TBC1D25	0.22903
3204	BBRF2	SLC16A6	0.019122
3205	BGLF4	MMP20	0.24916
3206	EBNA6	C5orf34	0.079614
3207	EBNA3	MYH7	0.24872
3208	EBNA6	MBD3L5	0.026783
3209	EBNA6	ZNF767	0.093633
3210	BZLF2	ATP5SL	0.011173
3211	EBNA3	NSUN5P1	0.070407
3212	BGLF4	REL	0.081037
3213	BMLF1	NHEJ1	0.31093
3214	EBNA1	IGFLR1	0.20279
3215	EBNA3	SLC25A12	0.11986
3216	BGLF4	ADRB1	0.041559
3217	BMLF1	TOMM22	0.2569
3218	BMLF1	MMS22L	0.36442
3219	BGLF4	SLC23A1	0.13931
3220	EBNA1	MARK3	0.10759
3221	BZLF1	CD81	0.11754
3222	EBNA1	KAT6A	0.047537
3223	BZLF1	ARL4A	0.14688
3224	BMLF1	SDSL	0.041529
3225	BMLF1	CEP250	0.19685
3226	BZLF2	MPTX1	0.011473
3227	EBNA1	SYPL1	0.075803
3228	EBNA-LP	PLSCR2	0.18012
3229	BMLF1	COBL	0.045807
3230	BGLF4	CLTCL1	0.16692
3231	EBNA-LP	ARRDC3	0.39393
3232	BOLF1	TTN	0.030604
3233	BZLF2	GAGE2A	0.042935
3234	BGLF4	SRR	0.038136
3235	BLLF1	FAM184B	0.013821
3236	BGLF4	SERPINB8	0.19767
3237	BILF1	EPS15	0.078337
3238	BARF1	EIF1AY	0.022972
3239	BHRF1	S100A8	0.053789
3240	BZLF1	ROR1	0.15552
3241	BMLF1	CITED2	0.045731
3242	EBNA1	NCOA2	0.031699
3243	BGLF4	ZMYND10	0.19427
3244	BALF2	ANXA11	0.05924
3245	EBNA1	COL1A2	0.088112
3246	BARF1	VIL1	0.096224
3247	BGLF4	TMSB4X	0.14088
3248	BZLF1	ARHGAP26	0.065606
3249	EBNA1	ATP1A1	0.06264
3250	BILF1	EHD1	0.064413
3251	BMLF1	ARF6	0.13425
3252	EBNA3	HHAT	0.1143
3253	BVRF1	STAT1	0.016609
3254	BMLF1	DDX10	0.01449
3255	BTRF1	AHSA1	0.016114
3256	EBNA-LP	SPOCK1	0.16665
3257	EBNA3	SNAP47	0.37409
3258	EBNA3	RRAD	0.32431
3259	BMLF1	DBNDD2	0.44386
3260	BDLF1	NSUN5P1	0.015141
3261	BZLF1	ANXA7	0.283
3262	BLLF2	TRAPPC8	0.031387
3263	BGLF4	NGLY1	0.17918
3264	BRRF1	PLSCR4	0.17528

3265	BGRF1/BDRF1	SPACA3	0.022259
3266	EBNA1	SCNN1G	0.14379
3267	EBNA3	WRN	0.033209
3268	BZLF1	TTI1	0.23617
3269	BMLF1	CXCR4	0.11443
3270	EBNA-LP	SH3GLB1	0.16578
3271	BZLF1	TAF2	0.097748
3272	BALF2	NECAB2	0.14572
3273	BPLF1	LETM1	0.13706
3274	BBRF2	NBPF16	0.039537
3275	BLLF1	CC2D2B	0.014238
3276	BLLF2	POLR2L	0.10067
3277	EBNA-LP	CRY2	0.1369
3278	BHRF1	FAM78A	0.023437
3279	BMLF1	CGREF1	0.21645
3280	EBNA1	INHBB	0.12918
3281	BLLF2	ZNF408	0.017213
3282	BLLF1	CCDC137	0.060958
3283	BMLF1	FNBP1	0.38092
3284	BMLF1	TTLL8	0.047735
3285	EBNA-LP	PRSS12	0.045824
3286	EBNA1	SPOCK1	0.070081
3287	BPLF1	CCDC176	0.089774
3288	BMLF1	BABAM1	0.33345
3289	EBNA-LP	JUND	0.13297
3290	EBNA-LP	C12orf57	0.048624
3291	BMLF1	KLHL9	0.011801
3292	BMLF1	CABP7	0.21218
3293	EBNA3	DHFR	0.028838
3294	BZLF1	PA2G4	0.04479
3295	BMLF1	MYLIP	0.21927
3296	EBNA-LP	CCAR2	0.3461
3297	BMLF1	RABL6	0.35391
3298	EBNA-LP	ADAM17	0.058467
3299	EBNA3	DDX17	0.23633
3300	BILF1	PPP3CA	0.03693
3301	BLLF2	ACOT6	0.01021
3302	EBNA3	CCDC65	0.016529
3303	LMP2	PPP2R3B	0.03256
3304	LMP1	MYL6B	0.064096
3305	EBNA3	SCG5	0.30327
3306	EBNA3	RPS7	0.20429
3307	BMLF1	CDHR4	0.22489
3308	EBNA1	RALA	0.090609
3309	EBNA2	GCH1	0.028825
3310	BLLF1	MB21D2	0.019837
3311	EBNA3	ELOVL3	0.26816
3312	gL	GAGE12G	0.070023
3313	EBNA6	TMED2	0.013039
3314	BLLF2	ZNF649	0.021434
3315	BXRF1	ITSN1	0.030205
3316	EBNA3	STX6	0.22569
3317	EBNA2	CCDC137	0.033151
3318	BMLF1	TAF1D	0.34933
3319	BLLF2	ZNF774	0.015841
3320	BLLF2	ZNF879	0.020718
3321	BARF1	EIF4E	0.01998
3322	BMLF1	UBXN1	0.28061
3323	EBNA-LP	LONRF3	0.3383
3324	BMLF1	ZNF165	0.19408
3325	BMLF1	APIP	0.22459
3326	EBNA3	KIR2DL3	0.28848
3327	BMLF1	ARL3	0.11002
3328	EBNA-LP	DLGAP5	0.24381
3329	BMLF1	CDK8	0.10777
3330	BZLF1	IL15	0.087045
3331	LMP1	RET	0.033236
3332	BMLF1	SBF2	0.26631

3333	BZLF2	LTBP4	0.1236
3334	EBNA3	USP44	0.24134
3335	BXRF1	BNIP2	0.073277
3336	EBNA6	C20orf195	0.084234
3337	BMLF1	SLC6A6	0.023473
3338	BGLF4	ERCC1	0.040182
3339	EBNA3	C21orf67	0.15263
3340	EBNA2	CCDC176	0.078105
3341	BPLF1	F10	0.055141
3342	EBNA-LP	CAV1	0.010744
3343	BMLF1	FAM21A	0.033858
3344	EBNA1	IGFBP5	0.099693
3345	BZLF1	KRT17	0.10765
3346	BMLF1	CBFA2T3	0.12265
3347	EBNA3	TEAD4	0.12281
3348	BZLF2	C18orf25	0.040023
3349	BZLF1	MAU2	0.17355
3350	BZLF2	SLIT2	0.030771
3351	EBNA3	REL	0.15719
3352	BMLF1	RAD51AP1	0.40774
3353	BGLF4	HPRT1	0.062131
3354	BGLF4	RFX5	0.097266
3355	BMLF1	KLK10	0.047742
3356	EBNA3	RUFY1	0.30459
3357	EBNA1	YTHDC2	0.15573
3358	BLLF2	ZNF135	0.011262
3359	EBNA-LP	BRCC3	0.19032
3360	EBNA-LP	ACP1	0.12641
3361	EBNA-LP	CTTNBP2NL	0.41724
3362	EBNA1	NGFR	0.042926
3363	BLLF1	SS18L2	0.011466
3364	BGRF1/BDRF1	WIPF1	0.017466
3365	EBNA3	MYB	0.11971
3366	BMLF1	CALML3	0.26298
3367	BLLF2	PPP1R3D	0.01406
3368	EBNA3	PEX2	0.21713
3369	BZLF1	PDZD3	0.11388
3370	BZLF2	LINC00114	0.012597
3371	EBNA-LP	SYTL5	0.33556
3372	BZLF1	STAT5A	0.029739
3373	EBNA4	MMP15	0.060192
3374	BGLF4	GPAA1	0.11472
3375	EBNA2	CDK5R1	0.028035
3376	BPLF1	CYCB3-1	0.10727
3377	BZLF1	BORA	0.12251
3378	BZLF1	CD37	0.20276
3379	BZLF2	C5orf48	0.062663
3380	BLLF2	G4	0.029436
3381	EBNA-LP	EFHC1	0.11566
3382	BMLF1	VAPB	0.17062
3383	gM	NOTCH4	0.018208
3384	BMLF1	CNOT3	0.27469
3385	BZLF1	GPATCH8	0.16351
3386	BGLF4	CDT1	0.14411
3387	EBNA4	CNST	0.021858
3388	BILF1	CDH5	0.10749
3389	BMLF1	RPS2	0.24911
3390	EBNA6	UNC119	0.11203
3391	EBNA3	RYR3	0.094826
3392	EBNA1	SYT1	0.1534
3393	EBNA1	CNKSR1	0.092449
3394	EBNA3	CASP3	0.09959
3395	EBNA1	DCP1B	0.09621
3396	BGLF4	PCDHB3	0.050061
3397	BMLF1	PLSCR2	0.22552
3398	EBNA6	BLACE	0.024358
3399	BARF1	GRIN1	0.071173
3400	BLLF2	TDRD3	0.018279

EBNA3	GCC1	0.31946
BPLF1	MAGEB16	0.013146
EBNA1	AGR2	0.24042
EBNA4	STIM1	0.10123
EBNA3	MBD1	0.14831
BZLF1	APTX	0.051214
EBNA2	NID2	0.13523
BGLF4	GATA4	0.011047
EBNA1	MYL1	0.038417
EBNA-LP	SLC9A1	0.14042
EBNA1	OPTN	0.14814
EBNA-LP	MED20	0.21026
BDLF1	MASP1	0.098299
BMLF1	CDK20	0.18768
EBNA1	GDF2	0.0732
BZLF1	TYR	0.14009
BZLF2	MLC1	0.016944
EBNA3	SLC16A11	0.07036
EBNA2	IL6ST	0.062043
EBNA3	STX16	0.22295
EBNA1	FAM96B	0.1375
gL	FAM92B	0.023975
BZLF1	AJUBA	0.039161
BZLF1	F2RL2	0.12912
gL	SPARC	0.033762
EBNA3	UBE2Q1	0.28523
EBNA-LP	RHOA	0.043107
EBNA3	GGT1	0.24924
BMLF1	PRSS38	0.049094
EBNA-LP	ALDOC	0.1784
EBNA-LP	OGT	0.12718
BGLF4	IL1RAPL1	0.11092
BGLF4	PARN	0.10892
EBNA3	ZFC3H1	0.37119
BZLF1	ALB	0.05963
BOLF1	C12orf57	0.018166
BGLF4	DNMBP	0.16748
EBNA-LP	KITLG	0.17638
BTRF1	PDCD4	0.011427
EBNA3	SLC4A4	0.3277
BGLF4	DRD4	0.053771
BZLF2	WLS	0.022182
EBNA-LP	PTK6	0.14162
BZLF1	PTPRN	0.10542
EBNA-LP	ZNF148	0.098077
BLLF2	RNASE2	0.014837
BHRF1	SPARC	0.070944
BMLF1	TRIM17	0.32288
EBNA3	DTX2	0.24521
BHRF1	EFCAB4B	0.11293
BNLF2b	TECTB	0.017579
BLLF2	TSPY3	0.018872
EBNA-LP	COPB1	0.16407
BZLF1	CCDC59	0.17318
EBNA-LP	PIAS3	0.16287
EBNA1	SNRNP200	0.11028
BILF1	S100A1	0.1175
EBNA6	BICD1	0.011843
BPLF1	YMR013W-A	0.072113
EBNA-LP	ZP3	0.082173
EBNA3	CCDC28A	0.16676
EBNA1	BMI1	0.051029
BBRF2	SLC16A11	0.038964
BMLF1	NPM3	0.37927
BLLF2	XRCC4	0.01322
EBNA3	MUC1	0.20363
EBNA3	RXRB	0.11249
EBNA3	LDLR	0.17194

3469	EBNA1	PDGFB	0.016832
3470	BGLF4	RUVBL2	0.057839
3471	BGLF4	RAET1E	0.19672
3472	BZLF1	NELFB	0.14121
3473	EBNA-LP	USP19	0.19822
3474	EBNA3	EXOSC10	0.17403
3475	EBNA1	TBCB	0.16937
3476	BGLF4	GEMIN6	0.16041
3477	BGLF4	KIF14	0.12762
3478	EBNA-LP	SCRIB	0.16915
3479	EBNA1	KCNS1	0.13553
3480	EBNA-LP	UBE2B	0.10955
3481	EBNA1	WNK3	0.098281
3482	BMLF1	NPBWR2	0.34294
3483	EBNA-LP	L3MBTL1	0.080948
3484	BGLF4	MED30	0.18572
3485	EBNA3	DRC1	0.029286
3486	BMLF1	AKIRIN2	0.060209
3487	gL	YIR021W-A	0.043183
3488	BMLF1	CELF1	0.27668
3489	EBNA-LP	EPHA2	0.085018
3490	EBNA1	BTG2	0.10284
3491	BVRF1	C5orf58	0.027552
3492	BZLF1	MASP2	0.37304
3493	BMLF1	CYGB	0.019544
3494	BGLF4	CPSF2	0.193
3495	BLLF2	IL26	0.015934
3496	BGLF4	ZNF366	0.14053
3497	EBNA3	MAST1	0.17745
3498	BZLF1	PODXL2	0.1957
3499	BZLF1	ATP2B4	0.12562
3500	BGLF4	DZIP1	0.13294
3501	BMLF1	ATP6V1E2	0.039393
3502	EBNA-LP	HES1	0.019004
3503	gM	F2	0.024954
3504	BHRF1	THBS1	0.016439
3505	EBNA1	ESRRA	0.02221
3506	BMLF1	MMP25	0.10154
3507	gL	FAM184B	0.054804
3508	EBNA-LP	CRYGC	0.32846
3509	EBNA-LP	BAP1	0.17752
3510	BLLF2	NPAP1	0.082521
3511	BMLF1	RPS20	0.015525
3512	BZLF1	DAZAP2	0.19146
3513	gL	FAM74A4	0.042871
3514	EBNA1	TTBK2	0.10734
3515	BZLF1	KIF23	0.099359
3516	BOLF1	LDLR	0.047765
3517	BGLF4	PML	0.025164
3518	BGLF4	SLC9A7	0.12594
3519	EBNA-LP	VSNL1	0.2099
3520	EBNA3	CIITA	0.19683
3521	BARF1	HSP90B1	0.11469
3522	BZLF2	CALM1	0.075574
3523	BMLF1	MYH10	0.12368
3524	EBNA3	CD2AP	0.2886
3525	EBNA3	ATP6V0D2	0.25251
3526	EBNA1	IL1RL1	0.12349
3527	BVRF1	JAG2	0.10682
3528	BGLF4	HSPBP1	0.20184
3529	EBNA-LP	EPPIN	0.36149
3530	BPLF1	XAGE3	0.16083
3531	BZLF1	CEP72	0.15978
3532	BGLF4	CLASP2	0.065087
3533	BGLF4	BCL2L11	0.034965
3534	EBNA3	SLC18A1	0.071768
3535	BZLF2	N6AMT1	0.014692
3536	BMLF1	POMT2	0.047406

3537	BLLF2	GOLGA2P5	0.11989
3538	BMLF1	PRSS16	0.1438
3539	EBNA6	PRRC2B	0.25028
3540	BZLF1	SUDS3	0.17525
3541	BZLF1	TNNI2	0.16376
3542	EBNA6	FAM47C	0.11648
3543	EBNA3	SPACA3	0.31519
3544	BGLF4	ALX1	0.079509
3545	BMLF1	DDX23	0.23192
3546	BZLF1	PROZ	0.02232
3547	EBNA6	C19orf44	0.067222
3548	BALF2	C12orf57	0.023637
3549	BZLF1	KCND2	0.090004
3550	BZLF2	CXorf67	0.064616
3551	EBNA-LP	EEA1	0.24798
3552	BGLF4	NLK	0.030147
3553	EBNA3	HNRNPUL1	0.28233
3554	EBNA1	IFNLR1	0.18563
3555	EBNA1	RCVRN	0.064872
3556	BGLF4	GK	0.13539
3557	EBNA3	RUNX1	0.11095
3558	BLLF1	MATN2	0.099776
3559	EBNA1	POP4	0.18118
3560	BGLF4	APC2	0.14777
3561	BLLF1	SLAIN1	0.057086
3562	BMLF1	HMCN1	0.25023
3563	EBNA3	SMARCA2	0.10337
3564	EBNA3	YGL014C-A	0.10964
3565	BZLF1	GNB2	0.11325
3566	BGLF4	RIPK1	0.014878
3567	EBNA6	SAMD9L	0.016786
3568	BGLF4	IL1R1	0.15519
3569	BZLF1	ITIH1	0.048316
3570	BGLF4	PVR	0.088952
3571	EBNA-LP	EPHB4	0.21396
3572	BGLF4	FGFR1	0.011357
3573	gL	ARMC5	0.010656
3574	EBNA1	ZC3H10	0.1706
3575	BBRF2	OR2M5	0.030984
3576	BLLF2	ZNF25	0.012311
3577	EBNA3	NR1H2	0.068574
3578	EBNA4	NELL2	0.074133
3579	EBNA4	MT2A	0.12498
3580	BMLF1	MC4R	0.24477
3581	EBNA3	MEIS1	0.12582
3582	BZLF1	STX12	0.13451
3583	BARF1	NKD2	0.21939
3584	EBNA1	TNFRSF10A	0.12465
3585	BZLF1	GDF7	0.1223
3586	EBNA-LP	CDC73	0.1782
3587	BPLF1	MYL6B	0.11571
3588	EBNA3	PYDC1	0.28793
3589	EBNA-LP	SKIL	0.15575
3590	EBNA4	LAMTOR1	0.091606
3591	BPLF1	DHH	0.085527
3592	BGLF4	USP5	0.11267
3593	EBNA-LP	EXOSC7	0.29646
3594	EBNA3	HELZ2	0.1962
3595	EBNA3	CHST3	0.030574
3596	EBNA-LP	SLC6A17	0.032149
3597	EBNA3	SYT4	0.22209
3598	EBNA1	MPZL3	0.19024
3599	BZLF1	SNCB	0.027098
3600	BGLF4	TMBIM4	0.16418
3601	BGLF4	PIGK	0.095272
3602	BHRF1	LETM1	0.13613
3603	BZLF1	WSB1	0.21277
3604	BGLF4	APBB2	0.089288

EBNA1	SNRPD3	0.098006
BDLF1	S100A12	0.013435
EBNA1	AP2A2	0.12452
EBNA-LP	PIGK	0.18631
BGRF1/BDRF1	S100P	0.13692
BILF1	REPS1	0.17873
BBRF2	MFAP5	0.019975
BGLF4	SNAP25	0.053696
BMLF1	IL7R	0.21243
BGLF4	SCAI	0.17608
EBNA-LP	RUSC1	0.26909
BGLF4	PPP4R4	0.23255
BZLF1	PCDHA1	0.044854
BPLF1	smf	0.022208
EBNA1	SYNE3	0.12529
BGLF4	UCHL5	0.097735
BGLF4	MED1	0.019145
BMLF1	ILK	0.04682
EBNA1	MMP2	0.045668
LMP2	THBS2	0.08762
BZLF1	AKT3	0.021938
BBRF2	IFNK	0.029063
BZLF1	PVALB	0.061221
EBNA-LP	CFL2	0.36667
EBNA1	CENPT	0.1119
BLLF2	IL17RD	0.076422
BDLF1	CDH1	0.01303
BLLF1	C19orf71	0.024273
EBNA4	ELOVL7	0.1197
BGLF4	DDN	0.16374
BMLF1	IFI35	0.39293
BLLF1	VWA5B2	0.022272
BZLF1	CD200R1	0.18472
EBNA-LP	ZNF622	0.23908
BGLF4	ARF1	0.062504
BMLF1	FOXK1	0.087459
EBNA3	PKM	0.15627
BLLF2	SCAMP2	0.034587
EBNA-LP	CCR1	0.156
EBNA3	CD151	0.32112
BZLF1	VPS18	0.1277
EBNA3	RBBP8NL	0.030401
EBNA1	RWDD3	0.1844
BMLF1	MOB1A	0.40884
EBNA1	MYLIP	0.087545
BZLF1	ATP10B	0.21618
BLLF2	SGSH	0.015933
EBNA3	WASH1	0.28911
BZLF1	RNF6	0.085552
EBNA4	DAG1	0.11791
EBNA1	CDH1	0.063631
BZLF1	SH3PXD2B	0.11572
EBNA1	FAS	0.027538
BZLF1	TXNRD2	0.21979
BLLF2	MAGEA11	0.01617
BMLF1	TGOLN2	0.34535
EBNA1	POLG	0.12985
BALF2	PPP3CB	0.020716
LMP1	CALB1	0.066925
EBNA1	CABP7	0.036955
BMLF1	RIMS1	0.20532
EBNA1	TOX4	0.15957
BGLF4	SERGEF	0.17004
BMLF1	TGFB2	0.012452
EBNA-LP	STAT2	0.27688
EBNA3	HNRNPF	0.28464
BLLF2	ZNF84	0.014967
EBNA3	DYNC1H1	0.21338

BMLF1	GCC1	0.35551
BZLF1	SLC7A8	0.041219
BZLF1	NSF	0.087786
BGLF4	JAK1	0.059196
EBNA3	STRN4	0.39381
BZLF1	TMEM97	0.14722
EBNA3	CACYBP	0.29339
EBNA-LP	SPARCL1	0.20114
BZLF1	USP13	0.080677
BGLF4	CACYBP	0.16695
EBNA-LP	MINK1	0.13185
BGLF4	NAPG	0.18196
EBNA3	NAE1	0.24255
EBNA3	PPM1E	0.17323
BHRF1	C1orf192	0.053102
EBNA3	JAK1	0.18799
EBNA3	MORF4L2	0.28687
EBNA6	EAF1	0.15729
BZLF1	SIRT6	0.13682
BZLF1	TAGLN2	0.20858
EBNA4	CFTR	0.026108
EBNA3	BIN2	0.4246
EBNA1	GLMN	0.18205
BZLF1	PSMA2	0.05351
BLLF2	PLAGL1	0.020942
BGLF4	CRYAB	0.072923
EBNA3	CCDC104	0.035976
BLLF1	CCDC69	0.011526
EBNA4	NUP98	0.062115
EBNA3	GAGE5	0.031848
EBNA3	UBE2D4	0.23929
BZLF1	ITGAV	0.054988
BXRF1	EFEMP1	0.1687
EBNA1	CD2	0.13803
EBNA-LP	PRDX6	0.33155
EBNA-LP	ADAP1	0.10972
BZLF1	AKIP1	0.19741
EBNA4	MSANTD1	0.066256
EBNA-LP	MAP4K3	0.23647
BMLF1	METTL3	0.082315
BMLF1	SDR42E1	0.020169
gL	WLS	0.056892
BGLF4	VPS18	0.13501
BHRF1	PIR	0.073459
BGLF4	GPD2	0.038879
EBNA1	WAS	0.12268
EBNA1	PPFIA3	0.19672
EBNA1	QKI	0.19606
BMLF1	CYTH1	0.27146
BGLF4	NCBP1	0.10162
gB	MATN2	0.061286
BMLF1	RNPS1	0.28176
EBNA-LP	EVI5	0.24212
BGLF4	C1R	0.097833
BLLF2	PRDM14	0.013906
BXRF1	MMP20	0.042921
EBNA4	BSDC1	0.037565
BPLF1	S100A12	0.07488
BLLF2	CAMLG	0.071771
EBNA-LP	VAMP1	0.34444
BPLF1	IQGAP1	0.015977
BMLF1	CBS	0.08527
EBNA-LP	MGP	0.12242
BHRF1	SPHK1	0.010377
EBNA3	PCDHGA12	0.18006
EBNA1	VPS72	0.13476
EBNA1	CRY2	0.056651
EBNA-LP	SMOC1	0.15994

3741	EBNA3	ARR3	0.2986
3742	BGLF4	ZZEF1	0.035943
3743	EBNA-LP	INTS9	0.36347
3744	EBNA3	FBXW8	0.39916
3745	EBNA1	RSF1	0.069955
3746	BMLF1	NSF	0.18143
3747	BZLF1	RPL28	0.18101
3748	BMLF1	WHSC1L1	0.028491
3749	EBNA4	FLT3	0.014543
3750	EBNA-LP	RPL15	0.30443
3751	EBNA3	PAM	0.16429
3752	EBNA3	PLCB4	0.092979
3753	EBNA6	MEN1	0.047754
3754	EBNA-LP	AGO4	0.30732
3755	EBNA-LP	CDK12	0.093011
3756	BZLF1	PPP2R2A	0.13685
3757	BGLF4	JADE1	0.13987
3758	EBNA3	MC4R	0.2263
3759	EBNA3	SETDB1	0.2881
3760	EBNA1	TAB2	0.061961
3761	BZLF1	SOX5	0.027851
3762	BLLF2	ZNF628	0.014943
3763	BGLF4	TMEM67	0.14761
3764	EBNA1	TAPBP	0.045141
3765	BPLF1	SLAIN1	0.13651
3766	BGLF4	TPSAB1	0.2183
3767	BMLF1	MBOAT1	0.028482
3768	EBNA-LP	MAN1A1	0.084633
3769	EBNA-LP	CCDC53	0.4095
3770	BZLF2	NIPSNAP3B	0.0123
3771	BGLF4	EFCAB2	0.068073
3772	EBNA3	TULP3	0.27145
3773	BMLF1	CENPE	0.15171
3774	BMLF1	TRPC1	0.19633
3775	EBNA3	IL10RB	0.34208
3776	BMLF1	RARA	0.020802
3777	EBNA3	CDC42	0.013992
3778	BGLF4	SESTD1	0.17186
3779	BMLF1	SSR4	0.24788
3780	BHRF1	GOLGA6L6	0.11277
3781	BMLF1	CTU1	0.39339
3782	EBNA-LP	ULK2	0.29751
3783	EBNA3	FAM188A	0.64536
3784	EBNA-LP	YWHAZ	0.15769
3785	EBNA3	TNFRSF12A	0.25601
3786	gN	S100A1	0.05182
3787	EBNA-LP	CAPN14	0.09102
3788	EBNA1	NSL1	0.12461
3789	EBNA1	THBS2	0.38016
3790	BVRF1	SLC11A2	0.022757
3791	BGLF4	RNF14	0.085438
3792	EBNA3	TIMM50	0.16
3793	EBNA1	DNAJB4	0.16116
3794	EBNA-LP	EPB41L3	0.20553
3795	BZLF1	ACIN1	0.15293
3796	BLLF2	ZNF235	0.019274
3797	BZLF1	RAB11FIP4	0.37909
3798	EBNA-LP	IGF2BP3	0.28607
3799	EBNA3	DDX23	0.21105
3800	EBNA3	PEX14	0.17366
3801	BMLF1	FABP3	0.32241
3802	BGLF4	AMELX	0.12352
3803	BZLF1	CYTH2	0.10763
3804	BMLF1	DNAJC8	0.024169
3805	EBNA1	POLD2	0.082034
3806	BMLF1	ARPC5	0.3157
3807	EBNA3	ARPC2	0.26055
3808	BZLF1	RNF41	0.078082

3809	BMLF1	TNPO2	0.33966
3810	EBNA3	PICALM	0.11514
3811	BLLF2	NUDT13	0.019043
3812	BGLF4	SNRPD3	0.11022
3813	gN	MASP1	0.15399
3814	BLLF2	SNRPD2	0.016566
3815	EBNA6	OTUD1	0.050482
3816	EBNA-LP	KIF21A	0.32149
3817	BLLF1	CCDC94	0.03189
3818	EBNA-LP	RNF115	0.28304
3819	BMLF1	FBXO25	0.37314
3820	BLLF2	ZNF600	0.013362
3821	BLLF2	APOL6	0.068424
3822	EBNA3	GTF3C6	0.35777
3823	BPLF1	MIR7-3HG	0.016077
3824	EBNA1	IRAK3	0.04521
3825	BMLF1	SETD2	0.22036
3826	BLLF2	FBXO44	0.017122
3827	BGLF4	SEPT12	0.12675
3828	EBNA3	TEAD3	0.13944
3829	EBNA-LP	EIF2AK3	0.078384
3830	EBNA3	TIMP3	0.26291
3831	BGLF4	CDC27	0.12033
3832	BNLF2b	SLC18B1	0.024656
3833	BZLF1	MUL1	0.01641
3834	EBNA6	PSMG3	0.022303
3835	BALF2	ACTN1	0.064056
3836	EBNA-LP	AP1G1	0.25063
3837	BZLF1	ERBB2IP	0.11247
3838	BZLF2	C1orf222	0.021648
3839	BPLF1	FBXO34	0.010062
3840	BMLF1	PRSS33	0.047938
3841	gL	SDE2	0.012639
3842	BGLF4	SPOCK1	0.074754
3843	BMLF1	PPM1B	0.25804
3844	BLLF1	PPEF1	0.035995
3845	BHRF1	TCTEX1D1	0.032101
3846	LMP2	PPP3CA	0.011237
3847	EBNA-LP	ZZEF1	0.158
3848	BGLF4	OR2L13	0.16625
3849	EBNA-LP	EPCAM	0.32957
3850	BLLF1	GAREML	0.057505
3851	BILF1	PPEF2	0.095896
3852	BGLF4	VPS33A	0.14141
3853	BGLF4	NR4A2	0.037732
3854	gB	CDH1	0.015798
3855	EBNA-LP	ITGA2B	0.23129
3856	BZLF1	USP5	0.10179
3857	EBNA1	SNRPB2	0.14664
3858	EBNA3	AK4	0.014265
3859	BMLF1	NEGR1	0.38189
3860	BLLF1	LINC00597	0.026831
3861	EBNA1	ALOX5AP	0.079995
3862	BMLF1	IQGAP1	0.20412
3863	EBNA-LP	HDAC4	0.026017
3864	BZLF1	GHR	0.021838
3865	BMLF1	MFAP1	0.023253
3866	gN	SNX19	0.059051
3867	EBNA3	FAM166B	0.016091
3868	EBNA-LP	RAD21	0.21084
3869	EBNA1	ANXA11	0.24178
3870	BGRF1/BDRF1	S100A12	0.10798
3871	EBNA3	ARL4D	0.23606
3872	EBNA-LP	EMILIN1	0.31868
3873	BZLF1	GMCL1P1	0.20688
3874	BZLF2	MICU1	0.11738
3875	BLLF2	ZNF732	0.012942
3876	BMLF1	ANKHD1	0.45452

3877	BTRF1	RPRD1A	0.013271
3878	LMP1	FAM231D	0.021873
3879	EBNA3	SH3PXD2A	0.34074
3880	BRRF1	EIF4A1	0.022624
3881	BGLF4	WDR4	0.18363
3882	BMLF1	HRNR	0.20276
3883	BZLF1	GLMN	0.19251
3884	BZLF1	FCHO2	0.15408
3885	BZLF1	PTGIS	0.029036
3886	EBNA1	WNT1	0.032065
3887	BZLF1	CNNM3	0.20684
3888	BMLF1	FAM47C	0.062073
3889	BMLF1	PVRIG	0.034023
3890	BZLF1	EFHC2	0.057416
3891	EBNA1	MTPAP	0.12601
3892	EBNA3	ST13	0.25573
3893	EBNA3	EIF5A	0.14696
3894	BGRF1/BDRF1	ADAR	0.016089
3895	EBNA6	BCORP1	0.11243
3896	BMLF1	LAPTM5	0.047688
3897	EBNA4	STX1A	0.01227
3898	EBNA1	S100A9	0.11808
3899	BXRF1	AP3S1	0.063299
3900	EBNA3	ASB13	0.40422
3901	BZLF1	RIN3	0.22169
3902	EBNA3	CALB1	0.43841
3903	BZLF1	DLGAP5	0.11954
3904	BGLF4	ILF2	0.1643
3905	LMP2	ANXA6	0.099533
3906	BPLF1	ANXA6	0.13138
3907	EBNA4	FAM47B	0.20804
3908	EBNA1	SLC6A3	0.092913
3909	EBNA-LP	YEATS2	0.30223
3910	EBNA3	SCEL	0.3061
3911	BGLF4	RBM26	0.21384
3912	BMLF1	MRPL20	0.32765
3913	EBNA-LP	HAP1	0.27357
3914	EBNA3	SLC5A6	0.011552
3915	EBNA-LP	BRCA1	0.036554
3916	EBNA1	FGFR1OP	0.08193
3917	BMLF1	PDE3B	0.15283
3918	BPLF1	DPP4	0.01386
3919	BBRF2	OR2J2	0.030153
3920	EBNA-LP	NSG2	0.089611
3921	EBNA1	MYL10	0.043725
3922	EBNA-LP	MED18	0.2617
3923	BZLF1	ASS1	0.016751
3924	BGLF4	RCAN1	0.071235
3925	BNLF2b	NIPBL	0.020128
3926	EBNA3	PEX16	0.24828
3927	EBNA1	DLG2	0.11094
3928	EBNA-LP	DYNLL1	0.1596
3929	BZLF1	CECR2	0.16836
3930	BXRF1	PLSCR3	0.14185
3931	BGLF4	FNBP1L	0.21394
3932	EBNA2	THBD	0.078967
3933	EBNA-LP	TRIP12	0.28568
3934	EBNA3	DNAJC10	0.16195
3935	BZLF1	PXMP2	0.18496
3936	BGLF4	NLRC4	0.076453
3937	EBNA1	NOXO1	0.10799
3938	EBNA1	RORA	0.023336
3939	BMLF1	PRAM1	0.47413
3940	EBNA1	PCDHA5	0.036102
3941	EBNA1	CTNNA3	0.073522
3942	EBNA3	WLS	0.40363
3943	BLLF2	MACROD2	0.014224
3944	BGLF4	PDGFB	0.018758

EBNA1	SPG7	0.044636
BMLF1	TPSD1	0.049094
EBNA-LP	BOK	0.24282
BTRF1	DCT	0.010332
BLLF2	ZNF761	0.013032
BMLF1	OSBPL1A	0.22692
BZLF2	SLC6A4	0.013127
EBNA3	KCNJ6	0.29161
EBNA3	UBE2I	0.079361
BMLF1	KHK	0.030946
EBNA-LP	ACTN2	0.20327
BLLF2	ZNF485	0.010709
BMLF1	KIF18A	0.15713
EBNA1	MOB1B	0.1133
BOLF1	C19orf71	0.013548
BGLF4	NDUFS7	0.049752
BLLF2	KARS	0.052603
gN	C11orf72	0.010811
BMLF1	COG3	0.22887
BZLF1	KIAA1109	0.18092
EBNA-LP	PPP4C	0.20426
BTRF1	PLSCR3	0.031932
EBNA3	MMP25	0.060929
BPLF1	ACTN2	0.047924
EBNA1	EEF1B2	0.15438
BXRF1	FAM49B	0.026702
EBNA-LP	PCSK2	0.37151
BLLF2	TAS2R9	0.07066
BGLF4	MPZL1	0.11304
BMLF1	GPRASP1	0.45783
EBNA1	ABI1	0.066864
EBNA3	POLE4	0.30303
BMLF1	C2orf40	0.060064
BMLF1	COMMD3	0.47224
BGLF4	UBQLN4	0.16498
EBNA3	KIAA0141	0.3992
EBNA4	LINC00472	0.22525
BPLF1	YGL006W-A	0.014059
BMLF1	ADH1A	0.30289
EBNA2	CIB1	0.094105
BGLF4	MARK2	0.038
BMLF1	FXR2	0.49427
BMLF1	CABP5	0.22719
BPLF1	C1orf195	0.17947
gM	NID2	0.11528
EBNA3	TTYH1	0.17933
BZLF1	SNCA	0.023725
EBNA4	SNX19	0.14756
EBNA4	NPIPB11	0.015125
LMP2	FAM188B	0.072846
EBNA3	CD163	0.34725
BGLF4	ATP6V1F	0.13958
BGLF4	GTF2E1	0.13901
EBNA3	SRSF5	0.29025
EBNA-LP	NDE1	0.12959
BGLF4	STK16	0.039692
gL	LINC00205	0.10237
BZLF2	C16orf72	0.078686
EBNA-LP	CD72	0.27088
EBNA3	CLN6	0.28791
BMLF1	RASGRP3	0.10795
BZLF1	SNX7	0.0118
EBNA3	DSN1	0.26422
BTRF1	RABEP1	0.036285
EBNA3	EMR2	0.10813
BMLF1	OR2L13	0.32351
BMLF1	HIP1	0.15182
BZLF1	ANKHD1	0.26087

4013	BMLF1	NOX1	0.11858
4014	BMLF1	TGFBR3	0.07721
4015	BMLF1	PFDN4	0.31555
4016	BMLF1	IFNA16	0.010526
4017	EBNA3	FAM175A	0.33412
4018	BGLF4	COPB1	0.086957
4019	EBNA-LP	PJA1	0.29801
4020	BMLF1	WNT1	0.061434
4021	BLLF1	ATP2A1	0.03029
4022	BTRF1	IWS1	0.034806
4023	BLLF2	ZSCAN32	0.018319
4024	BLLF2	KCNJ13	0.012123
4025	BRRF1	LDLR	0.058345
4026	gB	C1orf195	0.043126
4027	BGLF4	PPARGC1A	0.061496
4028	EBNA2	CALB1	0.096842
4029	BMLF1	DTL	0.25639
4030	EBNA3	SMAD6	0.093091
4031	BMLF1	SCAP	0.2464
4032	EBNA-LP	BNIPL	0.29306
4033	LMP1	F10	0.032283
4034	BMLF1	TRIM9	0.021642
4035	EBNA1	ZP3	0.038258
4036	EBNA3	CCL18	0.3265
4037	BZLF1	EDC4	0.11055
4038	BMLF1	UQCRC2	0.20182
4039	BGLF4	KXD1	0.22862
4040	BGLF4	PIGT	0.14147
4041	EBNA3	DDAH2	0.27814
4042	BZLF1	PDCD6	0.33409
4043	EBNA-LP	IPO5	0.26785
4044	BLLF1	SZRD1	0.01131
4045	BGLF4	FAN1	0.14239
4046	BLLF2	MLF1	0.026113
4047	LMP2	CAPN1	0.023186
4048	EBNA3	TRPM6	0.2514
4049	EBNA-LP	THBD	0.4043
4050	BLLF2	SLCO1B7	0.015671
4051	gB	DHH	0.051341
4052	BMLF1	SERPINC1	0.29512
4053	BZLF1	CBFB	0.045954
4054	EBNA-LP	CD40	0.10438
4055	EBNA2	PDGFRB	0.016178
4056	EBNA1	ITK	0.039361
4057	EBNA1	EPOR	0.13371
4058	BMLF1	TGM5	0.015082
4059	BGLF4	ING4	0.11546
4060	EBNA1	NOTCH4	0.12461
4061	EBNA3	TDP2	0.21812
4062	BMLF1	AKAP7	0.12607
4063	BGLF4	EXOC2	0.20346
4064	BGLF4	ARHGEF1	0.085325
4065	BZLF1	MT-RNR2	0.1199
4066	BMLF1	CARD8	0.33611
4067	BGLF4	CORO1A	0.081788
4068	EBNA1	HNRNPU	0.1187
4069	EBNA6	RBBP8NL	0.067169
4070	EBNA3	TTC37	0.35718
4071	BZLF1	TEN1	0.1577
4072	BGLF4	CASP6	0.071404
4073	BARF1	NOP2	0.030797
4074	BGLF4	INSIG2	0.096863
4075	EBNA1	MKNK1	0.069574
4076	BHRF1	N4BP2L1	0.018008
4077	BGLF4	KPNA5	0.18458
4078	EBNA6	LY96	0.11219
4079	EBNA-LP	TRAK2	0.20018
4080	EBNA3	FOS	0.039511

4081	EBNA3	PVR	0.16437
4082	BGLF4	FAM160A2	0.20361
4083	BMLF1	BBS4	0.072607
4084	EBNA3	PPP3R1	0.21486
4085	BZLF1	LRIF1	0.18862
4086	EBNA3	DHDDS	0.42747
4087	EBNA6	PRMT8	0.017044
4088	EBNA-LP	RAD51	0.11171
4089	BZLF1	TNK1	0.13952
4090	BMLF1	ARRB1	0.069085
4091	gN	NID2	0.1282
4092	BZLF1	IGBP1	0.11544
4093	BGLF4	WIPF2	0.22281
4094	BMLF1	ADI1	0.25153
4095	EBNA1	DNM1L	0.014971
4096	BLLF1	IL6R	0.014169
4097	BGLF4	PKM	0.069699
4098	BMLF1	NOTCH3	0.26335
4099	EBNA-LP	ATP6V0D1	0.20958
4100	EBNA3	TLL1	0.046703
4101	BXRF1	ANXA2R	0.04147
4102	EBNA-LP	SH3D19	0.25142
4103	EBNA4	LINC00523	0.3346
4104	BBRF2	MED7	0.014881
4105	EBNA4	SRI	0.11338
4106	BZLF1	MRPL51	0.18431
4107	BMLF1	SELENBP1	0.30579
4108	BMLF1	TRIM27	0.13644
4109	BGLF4	DNAJC1	0.1132
4110	EBNA6	CRELD2	0.17598
4111	EBNA1	FCGR1A	0.15181
4112	BZLF1	HTRA2	0.096813
4113	BGLF4	DEPDC1	0.16235
4114	BMLF1	AC3.5	0.064825
4115	EBNA6	FNBP1L	0.020923
4116	EBNA2	ANXA7	0.05063
4117	EBNA1	SLIT3	0.016426
4118	gL	C9orf169	0.042578
4119	BMLF1	CAMSAP3	0.046028
4120	BMLF1	SCIMP	0.36381
4121	EBNA-LP	ARFIP2	0.28759
4122	BMLF1	PODXL2	0.39135
4123	BLLF2	MFAP2	0.1302
4124	EBNA-LP	BATF2	0.21534
4125	EBNA1	HNRNPUL1	0.15094
4126	BZLF1	JAK1	0.098006
4127	EBNA1	MT1H	0.12595
4128	BZLF1	GTF2F2	0.075393
4129	EBNA-LP	BNIP3L	0.13961
4130	EBNA-LP	DDX11	0.039844
4131	EBNA2	VIL1	0.05906
4132	BLLF2	CKM	0.017671
4133	gL	C14orf178	0.069442
4134	BNLF2b	TCEAL5	0.045759
4135	BZLF1	HM13	0.1162
4136	EBNA-LP	OOEP	0.034904
4137	BMLF1	RELB	0.19318
4138	EBNA3	WBP2	0.41358
4139	EBNA3	NRG1	0.052777
4140	EBNA3	RCOR3	0.30935
4141	EBNA3	PCDHA7	0.16911
4142	BGLF4	VPS4B	0.087427
4143	EBNA1	HINFP	0.018443
4144	BZLF1	C1orf87	0.057416
4145	BGLF4	NDUFA9	0.13662
4146	EBNA-LP	SERPINC1	0.26722
4147	BMLF1	MTR	0.012229
4148	EBNA4	NAGK	0.030395

BZLF1	IFNLR1	0.19642
EBNA3	FAM203B	0.080765
BGLF4	NFS1	0.1289
EBNA1	SMURF1	0.06754
BZLF1	BHLHE40	0.031462
BPLF1	C12orf61	0.070379
BRRF1	HSP90B1	0.11412
BBRF2	RABEP1	0.089126
BMLF1	AP5B1	0.39651
BLLF2	RHCG	0.051981
gL	POM121L2	0.093013
EBNA4	KIAA0226L	0.05946
EBNA-LP	PLA2G2C	0.14352
EBNA-LP	KAL1	0.26824
BZLF1	SH3RF1	0.10732
BLLF2	HIST1H2BB	0.015213
EBNA-LP	CUL4B	0.30017
BLLF2	MYADM	0.036958
BGLF4	SLC24A1	0.18812
BZLF1	PLAT	0.13662
BGLF4	NDOR1	0.13614
gN	SLIT2	0.024516
EBNA3	FAM86B2	0.017188
EBNA3	USP34	0.30759
BMLF1	TNFSF10	0.20263
EBNA-LP	IGFBP5	0.18425
BMLF1	MACF1	0.37414
EBNA-LP	ULK3	0.19747
EBNA3	PPP2R5C	0.24988
BGLF4	ANXA7	0.26885
BGLF4	SPARC	0.16899
BGLF4	TMEM173	0.030798
LMP1	RPH3A	0.027497
EBNA3	COG4	0.29591
EBNA-LP	ABCC2	0.30678
EBNA-LP	PITPNC1	0.39286
BZLF1	ITK	0.045746
BHRF1	YMR013W-A	0.076123
EBNA-LP	RARG	0.051651
BZLF1	EIF4G2	0.23232
BMLF1	ZEB1	0.11986
gM	SYT1	0.03529
EBNA3	DDC	0.17869
BZLF1	EIF3A	0.10634
EBNA3	ASS1	0.049328
BGLF4	EVI5	0.13013
BMLF1	PUS10	0.12827
EBNA3	SPRYD7	0.027475
EBNA3	SCAF1	0.13144
BMLF1	PIR	0.37196
BXRF1	SDF4	0.12714
BMLF1	TSR2	0.45782
LMP2	C2CD4D	0.012761
BVRF1	NID2	0.17231
BGLF4	MYL5	0.058125
BMLF1	IL6R	0.13993
EBNA-LP	EFCAB13	0.16306
BLLF2	SRRT	0.010443
EBNA-LP	MAPK11	0.10102
BMLF1	IFITM1	0.25199
BARF1	PROC	0.19267
BMLF1	RAB3A	0.12214
EBNA1	EDEM1	0.27796
BMLF1	ZNF148	0.13038
BLLF1	CHP2	0.082846
EBNA-LP	ACAA2	0.31843
EBNA6	SUMO4	0.019843
EBNA6	CXorf67	0.21544

4217	BGLF4	RPGRIP1	0.1838
4218	BGRF1/BDRF1	KCNIP1	0.14126
4219	BGLF4	IMP4	0.15593
4220	BZLF1	SUV420H2	0.20557
4221	BLLF2	MOCS3	0.010602
4222	BVRF1	FAM199X	0.020001
4223	BGLF4	CD1B	0.18659
4224	EBNA1	HPGDS	0.050722
4225	BZLF1	PCDHGA2	0.049103
4226	BBRF2	BTBD17	0.010792
4227	BGRF1/BDRF1	DNMT3A	0.01332
4228	EBNA2	GPR98	0.045352
4229	BLLF2	OR2T29	0.084698
4230	BGLF4	LRRC26	0.18387
4231	EBNA-LP	ERCC5	0.17407
4232	EBNA3	COMMD10	0.43795
4233	BMLF1	DLEU1	0.47224
4234	BMLF1	CACNA1I	0.29019
4235	EBNA3	INHBB	0.23496
4236	BGLF4	SSR4	0.0946
4237	EBNA-LP	PITPNM3	0.55968
4238	BZLF1	FOXN3	0.010935
4239	EBNA2	SLC6A4	0.010582
4240	BGLF4	RING1	0.085725
4241	EBNA-LP	FBN2	0.1693
4242	EBNA-LP	DLGAP1	0.30905
4243	EBNA-LP	SLC22A5	0.23793
4244	BLLF2	HCST	0.1856
4245	BLLF1	C1orf111	0.027448
4246	EBNA-LP	RITA1	0.2822
4247	BMLF1	IL17RA	0.32621
4248	LMP1	LRCOL1	0.037431
4249	EBNA-LP	S100A14	0.13313
4250	EBNA-LP	SCAP	0.22722
4251	BZLF1	RANBP2	0.078656
4252	BGLF4	PLXDC1	0.15957
4253	gB	SPTA1	0.010505
4254	EBNA-LP	XPO1	0.17551
4255	EBNA3	SAE1	0.15452
4256	EBNA3	JUNB	0.057086
4257	BGLF4	SAMHD1	0.14747
4258	BGLF4	PRUNE	0.16998
4259	BZLF1	PLEK	0.059609
4260	BHRF1	LRCOL1	0.10778
4261	BTRF1	MFAP2	0.034387
4262	EBNA3	MOB1B	0.26032
4263	BDLF1	S100A8	0.013332
4264	EBNA3	AIG1	0.41166
4265	BGLF4	NLGN4Y	0.15919
4266	BGLF4	RABEP1	0.23248
4267	BGLF4	TRIP11	0.1422
4268	EBNA3	KDM2B	0.17012
4269	BVRF1	LCA10	0.02135
4270	BGLF4	NF1	0.027704
4271	BZLF1	MX1	0.08382
4272	BPLF1	TP53INP1	0.092737
4273	EBNA1	AJUBA	0.036027
4274	EBNA-LP	PXN	0.1562
4275	BZLF1	SPTA1	0.20259
4276	BGLF4	EFCAB11	0.068073
4277	EBNA3	TPP1	0.20289
4278	BZLF1	C19orf80	0.22904
4279	BMLF1	TUBA3C	0.17278
4280	EBNA3	CTAGE15	0.022666
4281	EBNA1	UBXN7	0.15714
4282	BLLF1	CCDC104	0.013182
4283	BHRF1	FAM84A	0.032688
4284	BZLF1	PPP2R5D	0.14162

4285	BGLF4	SNX17	0.16377
4286	EBNA-LP	ARPC3	0.27969
4287	EBNA1	EXOC2	0.18731
4288	BZLF1	EPHA2	0.047483
4289	BMLF1	DMBT1	0.16439
4290	BGRF1/BDRF1	N6AMT1	0.043711
4291	BMLF1	CASP3	0.10863
4292	BHRF1	MEGF8	0.02054
4293	BZLF1	RNPS1	0.17125
4294	BLLF2	ZNF433	0.012141
4295	EBNA3	CCDC59	0.33039
4296	BZLF1	USP47	0.13567
4297	EBNA3	SLX4	0.28305
4298	BGLF4	FANCD2	0.18772
4299	BMLF1	MAP4K3	0.2709
4300	EBNA1	BPTF	0.065434
4301	BMLF1	UVRAG	0.34778
4302	EBNA3	RGAG1	0.036924
4303	EBNA1	JADE1	0.11166
4304	BTRF1	CNTLN	0.014209
4305	EBNA3	TULP1	0.23562
4306	BZLF1	CCDC50	0.22903
4307	EBNA4	DBNDD2	0.15528
4308	EBNA3	SNX9	0.33083
4309	gL	FAM221B	0.11346
4310	BZLF1	NPC2	0.11598
4311	BBRF2	CLUH	0.018689
4312	BMLF1	FBLN2	0.23528
4313	EBNA4	WIPF1	0.11705
4314	BOLF1	TPPP	0.12195
4315	BGLF4	KIF2A	0.080323
4316	EBNA-LP	TPSAB1	0.37151
4317	EBNA2	EFCAB4B	0.08939
4318	BZLF1	ZRANB1	0.095615
4319	EBNA-LP	FZD10	0.16018
4320	BLLF2	DNAJA2	0.016386
4321	BZLF1	CHGB	0.22236
4322	EBNA3	ARHGEF16	0.15081
4323	BGLF4	PAF1	0.13177
4324	EBNA3	DLG1	0.026419
4325	EBNA3	NCOA5	0.33039
4326	BZLF1	DLL4	0.026797
4327	BLLF2	ZNF284	0.020075
4328	BGLF4	THAP11	0.15707
4329	EBNA3	PDZD11	0.38479
4330	BGLF4	STRADB	0.0313
4331	BPLF1	KIAA1958	0.052675
4332	gN	REPS2	0.12876
4333	EBNA1	MUL1	0.011209
4334	EBNA-LP	SERPINE1	0.067594
4335	BMLF1	ATP8B1	0.255
4336	BGLF4	PSMA7	0.045792
4337	EBNA-LP	SYNE3	0.2692
4338	BMLF1	YBR056C-B	0.040313
4339	EBNA3	ytzL	0.1972
4340	EBNA3	EDEM2	0.11731
4341	EBNA4	SKP1	0.056393
4342	BGLF4	AP5S1	0.16668
4343	BZLF1	UACA	0.11496
4344	BLLF2	BEX5	0.067097
4345	BZLF1	SRPX2	0.13
4346	EBNA3	AK6	0.42138
4347	EBNA-LP	CDH7	0.16032
4348	BZLF1	SLC25A23	0.052515
4349	BGLF4	EXOSC8	0.17652
4350	EBNA3	STAT2	0.28336
4351	BZLF1	SPARCL1	0.063567
4352	BZLF1	FBXO4	0.1598

BARF1	EPS15	0.085654
gM	C2CD4D	0.010345
EBNA-LP	KIF5B	0.21971
EBNA-LP	SIK1	0.060127
BPLF1	MED1	0.010966
BARF1	ACTN4	0.087703
BMLF1	ATP2B1	0.24619
BALF2	NPC1L1	0.030292
BMLF1	THOC1	0.22743
BGLF4	KCNJ4	0.22307
EBNA-LP	PCBP1	0.33621
BZLF1	ZNF24	0.04997
BGLF4	ATG13	0.12
EBNA1	POU2F1	0.039509
EBNA-LP	SLC26A8	0.27462
BGLF4	ADCYAP1R1	0.05543
gM	CALR	0.012828
BZLF1	TRA2B	0.22552
EBNA3	DNAJC9-AS1	0.1051
EBNA-LP	ARHGEF2	0.065973
BHRF1	CCDC127	0.051863
BNLF2b	GOLGA8CP	0.12252
BGLF4	LRP2	0.13956
BGLF4	NPHP4	0.088127
BGLF4	LMTK2	0.052874
EBNA1	FMR1	0.22794
BZLF1	TECPR2	0.23617
BNLF2b	SLC38A9	0.022585
BLLF2	TMED9	0.014897
BMLF1	SCNN1B	0.25878
BMLF1	HPCAL4	0.24248
gL	CDH5	0.028026
BZLF1	TOMM20	0.098711
BPLF1	FAM83F	0.080334
BGLF4	SERTAD1	0.20979
BMLF1	CETN2	0.34984
gN	FAM47C	0.020266
EBNA-LP	TICRR	0.30164
EBNA3	CSNK1A1	0.11099
BZLF2	CCDC17	0.014946
BGLF4	KIR2DL3	0.15553
BMLF1	SERPINB9	0.26923
BZLF1	SIGLEC8	0.19651
EBNA3	CAST	0.42332
BGLF4	ZNF711	0.14648
EBNA1	IGF2BP3	0.14375
gL	CCDC57	0.013804
EBNA-LP	ARAF	0.16676
BGLF4	METTL1	0.18532
EBNA3	ATP5G2	0.015708
EBNA3	ALKBH2	0.07173
BMLF1	PRKAG1	0.092401
EBNA1	RPH3A	0.22765
BGLF4	CDK20	0.034751
EBNA-LP	UNC119	0.35897
BZLF1	IPO5	0.14616
BGLF4	IFIH1	0.072662
BMLF1	ETV6	0.22481
BZLF1	NPC1	0.078791
EBNA-LP	MMP12	0.019462
BLLF2	PGAM1	0.08885
EBNA-LP	SLC2A4	0.18168
BMLF1	SRSF8	0.011243
EBNA-LP	ELOVL6	0.25952
EBNA-LP	PPP1R13B	0.32071
EBNA4	HBA1	0.15392
BGLF4	MCM10	0.15181
EBNA4	MBD3L2	0.020113

4421	BLLF2	LIPT1	0.016272
4422	BZLF1	ADRB3	0.063514
4423	EBNA6	KCNJ4	0.045254
4424	EBNA1	OCM2	0.044917
4425	BZLF1	DDC	0.098324
4426	EBNA6	LINC00615	0.13977
4427	EBNA-LP	MARK3	0.2513
4428	BLLF2	DKK1	0.016687
4429	BZLF1	NOC2L	0.1093
4430	EBNA3	FZD8	0.13023
4431	EBNA6	NOP2	0.021661
4432	EBNA6	C2CD4D	0.047871
4433	BZLF2	CDRT4	0.030609
4434	BMLF1	LDHD	0.38134
4435	BMLF1	IQCB1	0.41995
4436	EBNA-LP	NEDD4L	0.14672
4437	BMLF1	PIAS4	0.19369
4438	BZLF2	CHP1	0.062734
4439	BMLF1	RAD21	0.22771
4440	BMLF1	POLE3	0.31927
4441	BVRF1	RPH3A	0.070295
4442	EBNA1	PIK3CB	0.093134
4443	LMP2	F10	0.046179
4444	BZLF1	PCDHA9	0.049103
4445	BMLF1	SLC1A7	0.028857
4446	BMLF1	MAGI3	0.26238
4447	gL	KIAA1841	0.1171
4448	EBNA3	CCDC53	0.41536
4449	EBNA3	LRRC47	0.40246
4450	EBNA-LP	RAB4A	0.19472
4451	EBNA3	GNPNAT1	0.097087
4452	BMLF1	KCNG3	0.27614
4453	BMLF1	CRHR2	0.24582
4454	EBNA4	TNFRSF12A	0.11316
4455	EBNA1	ACTN2	0.1324
4456	EBNA1	CTR9	0.099776
4457	BNLF2b	B4GALT3	0.011916
4458	EBNA3	THOC2	0.39736
4459	EBNA1	ECT2	0.024218
4460	BGLF4	TMEM30A	0.20986
4461	EBNA-LP	BCKDK	0.21699
4462	BZLF1	RUNX1T1	0.035843
4463	BLLF2	RPA4	0.042416
4464	BGLF4	PSMF1	0.096814
4465	BMLF1	SMARCAD1	0.22008
4466	BZLF1	SNX3	0.19114
4467	BZLF1	MEA1	0.20275
4468	EBNA3	PCDHA3	0.16911
4469	EBNA3	STX12	0.24898
4470	EBNA4	CAGE1	0.047247
4471	BMLF1	KIAA0408	0.47224
4472	BLLF2	G4	0.010335
4473	BBRF2	FAM203A	0.04241
4474	BZLF1	PTPRD	0.01641
4475	EBNA3	TTC23L	0.026138
4476	EBNA4	ELOVL4	0.1679
4477	BHRF1	RAB11FIP4	0.2164
4478	EBNA1	ATP7B	0.039809
4479	BGLF4	HNRNPD	0.12313
4480	EBNA3	FCN1	0.04927
4481	EBNA6	C1orf192	0.098238
4482	BGLF4	RPL28	0.17554
4483	EBNA-LP	SNAI1	0.11117
4484	BGLF4	PDIA6	0.090951
4485	BMLF1	CRADD	0.27068
4486	EBNA-LP	UNC13D	0.26157
4487	BGLF4	ELK4	0.095706
4488	BGLF4	USP1	0.1419

BGLF4	MYO5B	0.16773
EBNA3	FAM231A	0.33319
BGLF4	HNRNPA2B1	0.15745
BGLF4	RNMTL1	0.043747
BGLF4	ARHGEF15	0.1614
BMLF1	DUX4L9	0.20458
EBNA4	DYDC1	0.040536
BBRF2	OR2T1	0.033291
BLLF2	RPS5	0.015785
BMLF1	TNNT1	0.013246
BZLF2	DTNA	0.069558
EBNA6	SART1	0.10223
BLLF2	G4	0.083419
EBNA-LP	GPATCH8	0.31419
EBNA1	ZMIZ2	0.10357
LMP2	IL6R	0.014423
BZLF1	VPS33B	0.13229
EBNA3	NAP1L1	0.29823
EBNA4	KIAA1841	0.17511
BZLF1	TNIP2	0.041778
BMLF1	SRBD1	0.076036
EBNA3	OPRM1	0.12044
BLLF2	PCCB	0.013966
EBNA3	SEC11C	0.07098
BHRF1	PNMA6A	0.023108
BMLF1	MYH11	0.014975
BMLF1	CD8A	0.18976
BZLF1	NDUFS7	0.036177
BLLF1	TCP10L2	0.022153
EBNA3	ZHX3	0.14034
BGLF4	GAB1	0.090532
BZLF1	RFX6	0.10965
EBNA1	RPS9	0.095047
BMLF1	LGALS3	0.28403
EBNA4	CCDC66	0.055215
EBNA3	PPP2R3B	0.51801
gL	PNAS-138	0.098442
EBNA4	PRO1854	0.1124
EBNA-LP	CDT1	0.25863
EBNA3	FBXL12	0.39911
EBNA3	CCDC114	0.010537
BGLF4	RTN1	0.18275
BLLF1	FAM45A	0.044533
EBNA3	CARS	0.17517
BMLF1	SLC22A13	0.012696
BZLF1	PPP1R13B	0.1688
BHRF1	FAM203B	0.076286
BARF1	MYO6	0.01457
BMLF1	DHX8	0.31015
EBNA-LP	ATP6V0D2	0.24873
BZLF1	SYTL5	0.17798
EBNA-LP	S100A12	0.34761
BILF1	LTBP1	0.21691
BMLF1	LYZL6	0.068224
BMLF1	CSNK2A1	0.14552
EBNA1	PRICKLE3	0.16687
BMLF1	NTHL1	0.042306
BZLF2	UHRF1BP1L	0.060044
BNLF2b	NKAIN2	0.054728
BLLF2	SLC39A6	0.023428
EBNA3	SERPINB5	0.30748
BMLF1	KIF6	0.35982
EBNA-LP	RAD18	0.1737
EBNA3	PPM1D	0.17777
EBNA3	CD37	0.3754
BARF1	SDF4	0.16075
BZLF2	FAM106B	0.011878
BGLF4	RNF168	0.074768

4557	EBNA-LP	ELF2	0.15478
4558	EBNA4	NPIPA5	0.038189
4559	BMLF1	SMYD2	0.23817
4560	BMLF1	SIGLEC8	0.39221
4561	BGLF4	MZB1	0.18618
4562	EBNA3	SHFM1	0.39118
4563	BGLF4	RAB11A	0.098117
4564	EBNA-LP	TEAD1	0.17952
4565	BZLF1	GYS1	0.073833
4566	BBRF2	ARSH	0.010487
4567	EBNA-LP	ANKLE2	0.26381
4568	LMP1	EHD3	0.018247
4569	EBNA3	NR2F6	0.11348
4570	EBNA3	CRH	0.16746
4571	BMLF1	KIF5B	0.24444
4572	EBNA1	ACTR8	0.1608
4573	EBNA-LP	CREB3L3	0.15492
4574	BGLF4	TCERG1	0.17637
4575	EBNA3	FBXW9	0.43795
4576	BGLF4	NLRP12	0.11676
4577	BARF1	SLIT2	0.039871
4578	BMLF1	STK24	0.17204
4579	BLLF2	STIM1	0.0103
4580	BGLF4	MUC1	0.11506
4581	EBNA-LP	THBS2	0.59374
4582	BALF2	SLC11A2	0.019887
4583	BOLF1	RAB11FIP3	0.04931
4584	EBNA-LP	FAM47B	0.014277
4585	EBNA1	PCDHB8	0.038413
4586	gL	C22orf26	0.071708
4587	BGLF4	FASTK	0.097573
4588	BGLF4	CCDC28B	0.22953
4589	BZLF1	NUDT21	0.18414
4590	BGLF4	PCDHA13	0.058173
4591	BMLF1	ATP6AP2	0.34227
4592	EBNA3	RPUSD4	0.54675
4593	EBNA4	TERT	0.13721
4594	EBNA1	HRNR	0.028406
4595	BMLF1	EIF2B5	0.26045
4596	BZLF2	C3orf65	0.048289
4597	EBNA4	RABGAP1L	0.053888
4598	BGLF4	ACD	0.10395
4599	EBNA-LP	KPNA1	0.23626
4600	EBNA3	ZXDC	0.12652
4601	BMLF1	SLX1A	0.30543
4602	EBNA-LP	MVP	0.29375
4603	EBNA-LP	PCDHA13	0.17939
4604	BBRF2	CRTAP	0.016014
4605	BMLF1	HNRNPA1	0.014157
4606	EBNA4	ATN1	0.10696
4607	BGLF4	TNK2	0.045843
4608	BMLF1	PI4KA	0.35435
4609	BZLF1	DTNB	0.014414
4610	BHRF1	LINC00472	0.081893
4611	BMLF1	CCDC59	0.36073
4612	BMLF1	UHRF1BP1L	0.010157
4613	BHRF1	TXNIP	0.075004
4614	BZLF1	ATRX	0.093893
4615	BMLF1	RPL27A	0.30654
4616	EBNA-LP	RAB21	0.28568
4617	BALF2	LTBP4	0.10673
4618	BDLF1	HEATR5B	0.020893
4619	BMLF1	FNTA	0.30098
4620	BZLF1	PROC	0.32139
4621	BMLF1	EFCAB2	0.26298
4622	EBNA3	LRP12	0.32499
4623	EBNA3	MT2A	0.26487
4624	BARF1	NELL2	0.052769

4625	BBRF2	BZW1	0.011665
4626	EBNA6	CCDC152	0.028758
4627	EBNA3	SPAST	0.15184
4628	BZLF1	STIM2	0.34028
4629	BMLF1	APBA2	0.32063
4630	EBNA3	SIK1	0.057851
4631	BGLF4	IARS	0.056786
4632	EBNA-LP	ASGR1	0.31655
4633	BZLF1	RCAN1	0.045795
4634	BGLF4	STEAP3	0.12912
4635	EBNA3	CDC37	0.24077
4636	BGLF4	PIM3	0.061591
4637	EBNA-LP	RPUSD2	0.035092
4638	EBNA-LP	RRAGC	0.17264
4639	EBNA3	THY1	0.056867
4640	BGLF4	ARID5B	0.10252
4641	BGLF4	CTIF	0.2316
4642	EBNA-LP	ARL6IP1	0.40812
4643	EBNA1	DHPS	0.21537
4644	BMLF1	FST	0.23377
4645	BMLF1	CRHR1	0.11296
4646	BLLF1	KIAA1407	0.018092
4647	EBNA-LP	C2CD5	0.18845
4648	EBNA4	GTF3C6	0.17962
4649	BLLF2	LEMD1	0.027073
4650	EBNA3	EFCAB14	0.21072
4651	BMLF1	ETNK1	0.040978
4652	EBNA-LP	COPS5	0.22186
4653	EBNA1	CTNND1	0.065404
4654	EBNA-LP	ZC3H13	0.35368
4655	LMP1	NKD2	0.1001
4656	BBRF2	POLR3D	0.014922
4657	EBNA4	FAM195A	0.34344
4658	BZLF1	TNS4	0.19111
4659	BGLF4	NAE1	0.12508
4660	gM	NKD2	0.11742
4661	BGLF4	ZFYVE20	0.117
4662	EBNA1	TAF11	0.14148
4663	BGLF4	SUV420H2	0.21804
4664	BLLF2	RABEP1	0.13753
4665	EBNA-LP	CASP1	0.11829
4666	BTRF1	CKB	0.010464
4667	EBNA-LP	UBXN7	0.33246
4668	EBNA1	WIPI2	0.12944
4669	BVRF1	CCDC171	0.022122
4670	BXRF1	GCH1	0.040353
4671	BMLF1	STXBP2	0.23014
4672	BLLF1	CCDC169	0.017235
4673	BBRF2	BNIPL	0.011898
4674	EBNA1	PTPRO	0.027871
4675	BMLF1	TRA2A	0.027445
4676	EBNA3	EIF4E2	0.4394
4677	EBNA1	SUN1	0.10662
4678	BGLF4	MDM4	0.10804
4679	EBNA3	PCDHGB7	0.18006
4680	BLLF1	YJR030C	0.033659
4681	EBNA4	ARL6IP4	0.16004
4682	BMLF1	TSC1	0.11931
4683	BMLF1	MCM7	0.19046
4684	EBNA3	CLASP2	0.12652
4685	BZLF1	VDR	0.028948
4686	BBRF2	GPR123	0.012641
4687	EBNA-LP	NGLY1	0.32932
4688	BGLF4	PROS1	0.015646
4689	EBNA4	MICAL1	0.03728
4690	BMLF1	CBR4	0.35981
4691	EBNA4	STATH	0.32002
4692	BGLF4	PSMG2	0.18468

EBNA3	RUSC1	0.27338
EBNA3	ZBTB8OS	0.010264
EBNA3	CARD8	0.32076
BGLF4	DDX42	0.17157
EBNA1	SIGIRR	0.15931
EBNA3	CENPE	0.13605
EBNA-LP	MLLT10	0.13507
EBNA1	LOXL3	0.12073
BGLF4	PPP3CA	0.12467
EBNA-LP	CDC25A	0.047514
gM	MPTX1	0.013871
EBNA3	INO80B	0.26735
BGLF4	CBFB	0.072365
BLLF2	OR4F15	0.079663
BMLF1	METTL2B	0.059751
BZLF1	UCKL1	0.23085
BZLF1	DDB2	0.1225
BGLF4	ADIPOQ	0.018949
EBNA3	CDK5RAP2	0.13305
BZLF1	TAF10	0.019782
EBNA3	SLC4A11	0.011444
BGRF1/BDRF1	F10	0.098939
BMLF1	WDFY3	0.35558
LMP1	HEATR5B	0.035535
EBNA1	MAT2B	0.090073
BMLF1	ANKRD27	0.38052
BMLF1	PORCN	0.023759
EBNA-LP	CDK10	0.19807
EBNA1	FLOT1	0.13825
BLLF2	FYB	0.081855
EBNA-LP	LDLRAP1	0.14799
BGLF4	RPLP1	0.17554
EBNA1	SLC26A8	0.14532
gM	MYL2	0.12438
EBNA3	C3	0.17221
BLLF2	HIST1H2BJ	0.016318
BVRF1	YJR030C	0.036394
EBNA3	MAN1A1	0.083615
BZLF2	SYT11	0.1837
BLLF2	LARP6	0.024385
EBNA1	DAZ1	0.19758
BMLF1	PIP5K1C	0.016486
EBNA3	IL13	0.17678
BGLF4	CD24	0.058694
EBNA-LP	UBD	0.24601
BLLF2	OR2J2	0.077525
BLLF2	ERLEC1	0.032394
EBNA-LP	BBS7	0.28698
BZLF1	HAND2	0.031403
BMLF1	NOXA1	0.33439
EBNA-LP	WWP1	0.17998
gL	GOLGA6L20	0.063692
BZLF1	YAF2	0.11305
BHRF1	GAS6	0.010965
BGLF4	TNPO1	0.14055
BGLF4	POR	0.029104
BMLF1	IREB2	0.10337
BILF1	RBM8A	0.024274
EBNA-LP	NR2F2	0.059914
BGLF4	FXR2	0.28916
BGLF4	ZFC3H1	0.20393
BMLF1	PIK3R1	0.01989
EBNA1	TRIM17	0.1312
BMLF1	SCAF8	0.030655
EBNA3	CEACAM6	0.36087
EBNA-LP	PKN1	0.072443
EBNA-LP	PIKFYVE	0.21702
EBNA1	EYS	0.071713

4761	BLLF2	CD8A	0.018866
4762	gB	EFEMP1	0.079727
4763	BPLF1	EIF4E	0.020835
4764	BARF1	RHOT1	0.014662
4765	BLLF1	AP3S1	0.026312
4766	BVRF1	UNQ6126/PRO20091	0.021821
4767	BGLF4	EIF3L	0.23386
4768	gL	GAGE12I	0.070023
4769	gB	NCS1	0.07723
4770	EBNA3	EXOC2	0.36281
4771	EBNA-LP	RFX1	0.29679
4772	EBNA-LP	PIAS4	0.17174
4773	EBNA3	OXA1L	0.24917
4774	BGLF4	LCK	0.020572
4775	BMLF1	PIH1D2	0.013295
4776	EBNA1	HFE2	0.1439
4777	BZLF1	DHX9	0.08206
4778	EBNA3	GOLGA6L20	0.051129
4779	EBNA3	SETD8	0.28279
4780	EBNA3	GNS	0.12983
4781	EBNA1	SLC22A4	0.13446
4782	BGLF4	ERAP1	0.086966
4783	EBNA3	MIR17HG	0.15684
4784	EBNA-LP	AGO3	0.30732
4785	EBNA3	NBN	0.088058
4786	EBNA3	RANGAP1	0.17134
4787	EBNA3	ATG16L1	0.33752
4788	EBNA3	LRP6	0.020773
4789	BNLF2b	GOLGA8I	0.031534
4790	gB	FAT4	0.054338
4791	BMLF1	CD9	0.24751
4792	BBRF2	ZDHHC11	0.012988
4793	BZLF1	RWDD3	0.19741
4794	BGLF4	CPSF3	0.12705
4795	EBNA6	FAM149B1	0.13179
4796	BZLF1	GOLGA4	0.14782
4797	BVRF1	TLDC2	0.050452
4798	BGLF4	GSE1	0.24432
4799	EBNA-LP	HSP90AB1	0.164
4800	BMLF1	DCPS	0.20864
4801	EBNA4	FAM83C	0.15719
4802	BZLF1	ZSWIM7	0.15284
4803	EBNA3	HEATR5B	0.1006
4804	EBNA4	BCKDHA	0.025565
4805	EBNA3	AIMP2	0.28336
4806	EBNA1	AIFM1	0.077231
4807	BZLF1	SERPINB8	0.19105
4808	EBNA3	AOC1	0.10248
4809	BZLF1	CALM1	0.16116
4810	EBNA4	RWDD4	0.089687
4811	EBNA1	SIAH2	0.070267
4812	BMLF1	PPP2R5B	0.26309
4813	BMLF1	CALU	0.52897
4814	BZLF2	MAGEB17	0.011695
4815	EBNA1	TNKS	0.060386
4816	EBNA1	TFG	0.16728
4817	gM	DST	0.055905
4818	BMLF1	DPPA4	0.34975
4819	BILF1	AC3.5	0.048938
4820	EBNA-LP	POLR2B	0.15005
4821	BZLF1	MBD1	0.046
4822	EBNA6	NCS1	0.15364
4823	BMLF1	PPP2R2B	0.26317
4824	EBNA-LP	TAB3	0.16936
4825	BMLF1	ELOVL2	0.26558
4826	BMLF1	PRSS12	0.092978
4827	EBNA-LP	ARHGEF12	0.14673
4828	EBNA3	MAPK7	0.10745

4829	BBRF2	SGSH	0.012391
4830	BMLF1	RHOBTB3	0.32709
4831	EBNA-LP	STXBP5	0.063633
4832	BXRF1	RABEP1	0.027991
4833	BMLF1	CDH8	0.20658
4834	EBNA3	ERC1	0.18816
4835	BVRF1	CCDC169	0.023353
4836	BZLF1	PVRL3	0.15622
4837	EBNA3	NUAK1	0.20076
4838	BMLF1	SCEL	0.33607
4839	EBNA3	UHMK1	0.24933
4840	BGLF4	SGPL1	0.040699
4841	BHRF1	C10orf68	0.068698
4842	EBNA1	CHD4	0.024188
4843	BZLF1	SRSF5	0.16667
4844	BZLF1	SH3GLB1	0.081267
4845	BZLF2	NSUN5P1	0.025152
4846	EBNA3	UBA5	0.18579
4847	EBNA3	KLHL12	0.41081
4848	BOLF1	F2	0.043479
4849	EBNA1	STAT5A	0.03026
4850	BMLF1	DAG1	0.15038
4851	BVRF1	UPF3B	0.041296
4852	EBNA-LP	LETMD1	0.30769
4853	BALF2	FAM92A1	0.010071
4854	EBNA3	FSIP2	0.18917
4855	BPLF1	CT55	0.081525
4856	EBNA4	CITED2	0.015438
4857	EBNA6	COL17A1	0.19308
4858	EBNA-LP	EFCAB2	0.2099
4859	EBNA3	CCP110	0.3213
4860	EBNA3	ESRRA	0.10158
4861	BGLF4	JMJD1C	0.21649
4862	BGLF4	FBXO25	0.18918
4863	BMLF1	ATF4	0.13117
4864	EBNA-LP	STX4	0.17211
4865	EBNA3	LSM1	0.28537
4866	EBNA1	FAM109A	0.15336
4867	EBNA1	NDEL1	0.07776
4868	EBNA-LP	CADM1	0.13326
4869	BGLF4	NINL	0.055831
4870	EBNA-LP	DNAJB9	0.2844
4871	EBNA3	CENPF	0.10491
4872	LMP2	ACTN4	0.04424
4873	BZLF2	C12orf61	0.039033
4874	BMLF1	MICAL2	0.029328
4875	BGLF4	IRS2	0.039197
4876	BMLF1	STARD13	0.32217
4877	EBNA3	FNIP2	0.32561
4878	BGLF4	DNAJB2	0.16555
4879	BGLF4	SERPINA1	0.16243
4880	EBNA-LP	LILRB4	0.29422
4881	EBNA-LP	FBXW8	0.3936
4882	BMLF1	AGL	0.17662
4883	EBNA3	USP4	0.24901
4884	BZLF1	SOS1	0.051464
4885	BMLF1	RUVBL1	0.13275
4886	EBNA-LP	RALA	0.18535
4887	BMLF1	FMR1	0.34555
4888	EBNA1	DSC2	0.037494
4889	EBNA-LP	TPD52L1	0.29749
4890	BGLF4	VCAN	0.036598
4891	EBNA3	PLCZ1	0.072965
4892	EBNA3	CASP10	0.30234
4893	BPLF1	PNMAL1	0.04871
4894	BGLF4	DRD2	0.01351
4895	BMLF1	RTN1	0.35838
4896	EBNA-LP	DAXX	0.11272

4897	EBNA3	ITGA2	0.28521
4898	BLLF1	CT45A1	0.013839
4899	BLLF2	VWA3A	0.043778
4900	BMLF1	PCDHA7	0.21127
4901	EBNA3	PPP1R13L	0.18033
4902	BMLF1	E2F1	0.086213
4903	EBNA3	SREBF2	0.072059
4904	EBNA1	PCDHA1	0.035121
4905	LMP1	ACTN2	0.029494
4906	EBNA6	WWC2-AS2	0.1102
4907	EBNA3	ZK1128.3	0.12171
4908	EBNA-LP	TRAPPC1	0.045346
4909	EBNA-LP	LCA5	0.32948
4910	EBNA-LP	HNRNPL	0.28547
4911	EBNA-LP	ZC3HAV1L	0.012018
4912	EBNA1	CIB1	0.3061
4913	BLLF1	C12orf61	0.03677
4914	BBRF2	BAHD1	0.014035
4915	BMLF1	KIRREL3	0.38016
4916	BGLF4	ANXA6	0.36866
4917	BMLF1	LVP1a	0.080977
4918	BZLF1	SNAP25	0.050656
4919	EBNA6	MYO1E	0.043268
4920	EBNA1	EGFL8	0.053038
4921	EBNA-LP	PSMA6	0.10655
4922	EBNA3	POGZ	0.2452
4923	EBNA1	GNB2L1	0.019703
4924	BZLF1	MYL2	0.33548
4925	BNLF2b	NCR3	0.036496
4926	BGLF4	DARS	0.11283
4927	EBNA2	PDCD6	0.15047
4928	BOLF1	IL6R	0.029759
4929	EBNA-LP	DCAF8	0.41438
4930	BHRF1	ATP2A1	0.042194
4931	BPLF1	DDX26B	0.11678
4932	BGLF4	ADRB3	0.068304
4933	EBNA3	LYST	0.19432
4934	BZLF1	NUP85	0.09266
4935	EBNA-LP	CACYBP	0.28916
4936	EBNA3	RPL5	0.19676
4937	EBNA1	TBKBP1	0.22192
4938	BMLF1	THOC2	0.34818
4939	EBNA-LP	CLIC5	0.18494
4940	BBRF2	PMM1	0.01099
4941	gN	FAM188B2	0.030177
4942	BZLF1	CETN3	0.083018
4943	BGLF4	EED	0.14621
4944	BMLF1	SLC16A13	0.031311
4945	BZLF1	ITGAM	0.10488
4946	BMLF1	FZD3	0.17077
4947	BZLF1	STK24	0.057331
4948	BLLF2	HIC1	0.012755
4949	BZLF1	ARHGAP21	0.15282
4950	BGLF4	INSIG1	0.1183
4951	EBNA3	MAGEA1	0.28814
4952	BGLF4	FGF1	0.07834
4953	EBNA1	TLN1	0.016171
4954	BZLF1	ZC3HC1	0.066583
4955	BGLF4	LMAN1	0.069072
4956	EBNA1	SERPINB8	0.17992
4957	BZLF1	LRP1B	0.048402
4958	BLLF2	LVP1a	0.10059
4959	BBRF2	PRAME	0.078998
4960	BGLF4	CREB5	0.078034
4961	EBNA1	CD4	0.046855
4962	BLLF1	CCDC26	0.0225
4963	EBNA4	FAM74A4	0.085839
4964	BZLF1	DFNB31	0.11228

4965	BGLF4	SYNM	0.14914
4966	gL	HRASLS5	0.011491
4967	EBNA-LP	POLG	0.26764
4968	EBNA1	CENPI	0.095451
4969	BMLF1	PROCA1	0.24175
4970	EBNA3	SLC15A2	0.26997
4971	BGLF4	CDC20	0.084703
4972	BMLF1	IGF1	0.035633
4973	EBNA-LP	ATP1B1	0.24553
4974	EBNA1	USP33	0.073455
4975	LMP2	F2	0.025165
4976	BMLF1	MBNL1	0.26052
4977	BLLF2	BNIPL	0.010799
4978	EBNA3	LAMTOR1	0.22858
4979	BMLF1	PRF1	0.37865
4980	EBNA1	UBE2L6	0.14281
4981	BLLF2	ZNF569	0.024153
4982	EBNA6	C4orf17	0.049741
4983	EBNA-LP	MSN	0.12042
4984	EBNA4	INSIG1	0.092026
4985	EBNA-LP	DUOX2	0.185
4986	BMLF1	NANOS1	0.35581
4987	EBNA-LP	ATP7A	0.030794
4988	EBNA4	ERICH1	0.19937
4989	BOLF1	C5orf34	0.011816
4990	EBNA-LP	CDH1	0.10159
4991	EBNA1	HSPB1	0.01868
4992	BZLF2	GRIN1	0.047393
4993	EBNA3	RTF1	0.19216
4994	EBNA3	FAM127B	0.078192
4995	BZLF1	BRCC3	0.10263
4996	gB	STIM2	0.052164
4997	EBNA1	TMPO	0.14821
4998	BMLF1	PFKFB3	0.011351
4999	EBNA1	DISC1	0.09659
5000	BLLF2	RNASE4	0.022259
5001	EBNA-LP	CYTIP	0.38969
5002	EBNA1	KLKB1	0.17238
5003	BZLF1	PCDHGB6	0.049103
5004	BZLF1	SETD1B	0.20536
5005	BGLF4	PPFIA1	0.21543
5006	BZLF1	NCK2	0.11105
5007	BHRF1	CDR2L	0.029957
5008	BMLF1	ANXA8L2	0.22732
5009	EBNA3	ELTD1	0.13789
5010	EBNA3	NPB	0.36287
5011	BZLF1	RP9	0.14544
5012	EBNA-LP	HBA1	0.27475
5013	EBNA4	KCNJ9	0.025629
5014	BGLF4	ZNF408	0.13003
5015	BMLF1	RAD9A	0.19236
5016	EBNA1	SALL1	0.01778
5017	BMLF1	ADAD2	0.10268
5018	EBNA3	OCRL	0.1742
5019	BZLF1	AGRN	0.065711
5020	BMLF1	EFCAB6	0.19286
5021	EBNA-LP	COBL	0.037225
5022	BZLF1	MYB	0.041766
5023	EBNA-LP	ATXN1	0.21844
5024	BZLF2	PPP3CA	0.023973
5025	EBNA3	F12	0.24421
5026	BMLF1	ELAVL2	0.43363
5027	EBNA4	PARK7	0.053536
5028	EBNA-LP	ITPKB	0.029545
5029	BMLF1	KRT16	0.3366
5030	EBNA-LP	ALKBH8	0.35257
5031	EBNA-LP	PRMT5	0.20387
5032	BLLF1	C17orf102	0.027511

5033	BMLF1	ATP11B	0.16656
5034	BGLF4	WAPAL	0.10619
5035	LMP1	MASP1	0.11601
5036	EBNA-LP	KCNJ3	0.29857
5037	BLLF2	VPREB1	0.10059
5038	BMLF1	DPPA5	0.050543
5039	BZLF1	PTPRM	0.036152
5040	BZLF1	CHORDC1	0.15532
5041	BVRF1	CXorf40B	0.010986
5042	BLLF2	TIGD1	0.062166
5043	EBNA1	CCL3	0.031115
5044	BALF2	MICU1	0.11212
5045	BALF2	DHH	0.099774
5046	BPLF1	AC3.5	0.058848
5047	BBRF2	CDK5R2	0.028411
5048	BGLF4	TSG101	0.072414
5049	BMLF1	CTRL	0.047945
5050	BALF2	CD93	0.098888
5051	EBNA6	CTAGE15	0.031639
5052	EBNA-LP	ERN1	0.070823
5053	EBNA3	PABPC1	0.18824
5054	BMLF1	MED31	0.2807
5055	EBNA3	DLGAP4	0.33442
5056	BGLF4	KLK5	0.21997
5057	EBNA-LP	UCHL5	0.15385
5058	EBNA3	PPP2R5E	0.28637
5059	BMLF1	CHST2	0.048567
5060	BZLF1	SYNCRIP	0.11167
5061	BMLF1	SETDB2	0.045193
5062	gL	CHAF1A	0.20205
5063	EBNA1	PPEF1	0.32413
5064	EBNA6	C6orf222	0.069246
5065	EBNA3	EDAR	0.25066
5066	EBNA3	PRMT10	0.049276
5067	EBNA3	KIN	0.071546
5068	EBNA-LP	PAK2	0.032994
5069	EBNA1	ALKBH8	0.20994
5070	BZLF1	DLG2	0.11886
5071	BPLF1	FASN	0.046334
5072	gB	CDH5	0.027253
5073	BRRF1	EHD3	0.09032
5074	BXRF1	FAM188B2	0.052839
5075	gL	COA7	0.035165
5076	EBNA-LP	RNF40	0.19539
5077	BLLF2	HIST2H2BE	0.015213
5078	BGLF4	NFYB	0.086301
5079	EBNA3	TJP2	0.30693
5080	BZLF1	TCF7	0.024577
5081	BGLF4	PAIP1	0.19362
5082	EBNA3	KIAA0226L	0.013944
5083	EBNA4	EIF2B4	0.03849
5084	BILF1	ACTN4	0.090356
5085	BZLF1	STAMBPL1	0.1818
5086	EBNA6	MAGOH	0.019676
5087	EBNA-LP	SYT11	0.59029
5088	EBNA3	POLR1A	0.22889
5089	EBNA-LP	RABGEF1	0.26858
5090	BALF2	KCNJ4	0.016634
5091	EBNA1	CHMP1A	0.092537
5092	BGLF4	MAP1S	0.083579
5093	BGLF4	CTNND2	0.1518
5094	BGRF1/BDRF1	PRDX6	0.035326
5095	EBNA3	UBXN7	0.33629
5096	EBNA1	MDC1	0.15216
5097	EBNA-LP	SNX15	0.10955
5098	EBNA3	GPR179	0.11235
5099	BLLF2	KIAA0513	0.020922
5100	BGLF4	NPM1	0.029225

5101	BZLF1	EIF4A2	0.18277
5102	gM	CALB1	0.083024
5103	EBNA-LP	EXOSC5	0.26222
5104	EBNA3	KRT81	0.34309
5105	BMLF1	FUNDC1	0.31023
5106	BZLF1	UBE2Q1	0.12811
5107	BGLF4	SCEL	0.16823
5108	BNLF2b	CHIT1	0.013583
5109	EBNA1	PSMA3	0.046542
5110	BMLF1	SLC23A3	0.017366
5111	BMLF1	YTHDF3	0.47224
5112	EBNA-LP	ZNF165	0.15176
5113	EBNA-LP	TBC1D10A	0.31408
5114	EBNA3	IGFLR1	0.39913
5115	EBNA1	PCDHB2	0.033158
5116	BMLF1	RPS6KB1	0.039925
5117	BZLF1	UBE2N	0.050619
5118	BMLF1	GMFG	0.016158
5119	BMLF1	LIMK1	0.15969
5120	EBNA3	MTF1	0.090645
5121	EBNA-LP	ATG5	0.2575
5122	BZLF1	UNC5B	0.1597
5123	BILF1	CXorf67	0.12983
5124	BMLF1	PITX2	0.029839
5125	EBNA-LP	CAPNS1	0.48474
5126	EBNA1	OTUD7B	0.102
5127	BBRF2	IP6K2	0.030404
5128	BMLF1	NABP2	0.33863
5129	BHRF1	FBRSL1	0.075407
5130	BZLF1	PTBP1	0.19228
5131	BZLF1	IRAK1	0.038499
5132	BGLF4	PDZD7	0.20224
5133	EBNA-LP	DSG3	0.15291
5134	EBNA3	SUMO1	0.14311
5135	BBRF2	MUC20	0.034942
5136	EBNA6	ZNRD1-AS1	0.044622
5137	BARF1	SNX6	0.058606
5138	EBNA-LP	NOTCH3	0.26206
5139	BHRF1	PNMAL2	0.085848
5140	EBNA3	P4HB	0.12852
5141	EBNA3	BCCIP	0.34458
5142	EBNA-LP	NPBWR1	0.29146
5143	EBNA3	SLC17A7	0.018288
5144	EBNA4	NUDCD3	0.15253
5145	BGLF4	RHO	0.091988
5146	BGLF4	CRIPAK	0.18018
5147	BGLF4	SLC25A17	0.11519
5148	EBNA1	RFX1	0.13875
5149	EBNA-LP	ANXA5	0.054812
5150	EBNA-LP	CST3	0.092953
5151	BGLF4	DYNLT1	0.10035
5152	EBNA6	PPEF1	0.091586
5153	BMLF1	NOA1	0.28215
5154	EBNA3	KMT2E	0.20449
5155	EBNA3	CHIC2	0.40426
5156	BPLF1	FAM178B	0.060132
5157	EBNA1	MICU3	0.042436
5158	gL	C6orf118	0.07084
5159	EBNA1	ZNF224	0.1011
5160	BMLF1	SNX8	0.32838
5161	EBNA3	HEYL	0.080959
5162	BGLF4	MAPKAPK3	0.028171
5163	EBNA-LP	PEF1	0.6549
5164	BMLF1	ILDR2	0.064562
5165	BZLF1	SPC24	0.14439
5166	EBNA1	PPM1F	0.044917
5167	BGLF4	RABGAP1	0.1492
5168	BZLF1	DPM1	0.12237

5169	BZLF1	VPRBP	0.1728
5170	EBNA4	GAGE12B	0.1692
5171	EBNA-LP	RAP2A	0.20293
5172	EBNA3	UBE3C	0.24741
5173	BZLF1	MZT2B	0.18902
5174	BGLF4	RTKN	0.11925
5175	EBNA-LP	RIPK1	0.052352
5176	BHRF1	KIAA0907	0.017524
5177	BMLF1	FAM58A	0.30247
5178	BMLF1	B3GNTL1	0.021703
5179	EBNA1	PPM1B	0.089159
5180	BLLF1	GAGE12G	0.014025
5181	BZLF1	SNTN	0.054233
5182	BZLF1	POLG	0.1455
5183	EBNA3	AK3	0.013266
5184	BGLF4	ATF5	0.078355
5185	BGLF4	TPO	0.083048
5186	EBNA-LP	TBX5	0.032325
5187	BOLF1	ARL14EPL	0.030007
5188	BILF1	DPP4	0.027447
5189	BGLF4	CDHR4	0.058173
5190	EBNA6	TECPR1	0.13676
5191	EBNA1	GSTP1	0.075075
5192	EBNA3	CITED1	0.048896
5193	BGLF4	COPS5	0.13538
5194	BXRF1	LRP8	0.12632
5195	BZLF1	EIF4B	0.22273
5196	BGLF4	IL5RA	0.20226
5197	BGLF4	MEIS1	0.06186
5198	EBNA3	ZFP36	0.10294
5199	EBNA1	CDH16	0.038413
5200	gN	PPP3CA	0.011928
5201	BMLF1	EIF3I	0.42145
5202	BGLF4	LAMB3	0.09893
5203	BMLF1	VPS33B	0.25099
5204	BZLF1	RBMY1B	0.2213
5205	BHRF1	SHCBP1L	0.015986
5206	BZLF1	S100A12	0.23087
5207	BOLF1	NOTCH2	0.013469
5208	BHRF1	LAMTOR1	0.038943
5209	EBNA-LP	SMYD2	0.22816
5210	BMLF1	CAPN14	0.14594
5211	BBRF2	CDC42EP4	0.012277
5212	BZLF1	KCNMA1	0.083713
5213	BGLF4	PKP2	0.11197
5214	EBNA1	EIF4A1	0.16937
5215	BGLF4	BOLL	0.20402
5216	BLLF2	MT2A	0.050936
5217	EBNA-LP	NOP58	0.29723
5218	BBRF2	GK3P	0.039527
5219	BGLF4	CAPN1	0.26788
5220	EBNA1	ZNF136	0.10036
5221	BZLF1	ACTN2	0.1281
5222	BBRF2	KCNK13	0.014689
5223	BBRF2	DCST1	0.014911
5224	EBNA1	LTBP1	0.41075
5225	BTRF1	RDM1	0.024163
5226	BXRF1	TTN	0.029299
5227	BGLF4	CD86	0.10035
5228	EBNA4	TPD52	0.21856
5229	EBNA6	ATP2B2	0.015341
5230	BGLF4	GEMIN8	0.18546
5231	EBNA3	DYNLRB1	0.28576
5232	EBNA6	DHPS	0.10441
5233	BGLF4	CYTH1	0.13049
5234	LMP2	GRIN1	0.033191
5235	BMLF1	IL2RA	0.026436
5236	EBNA3	RNF187	0.23077

5237	BMLF1	FNDC3A	0.017662
5238	BZLF1	THEG	0.1806
5239	BMLF1	PCDHB15	0.21127
5240	BZLF1	AIFM1	0.083677
5241	BMLF1	SLC22A15	0.018129
5242	BMLF1	AFF1	0.27067
5243	BLLF2	IGBP1	0.068173
5244	BBRF2	ETNK2	0.038895
5245	BBRF2	CKLF	0.023886
5246	EBNA1	TNFRSF14	0.13191
5247	BMLF1	TAS2R16	0.30832
5248	EBNA3	ZC3H14	0.36318
5249	EBNA1	IL17RA	0.1395
5250	EBNA1	PCGF3	0.1229
5251	BMLF1	ITGAX	0.32223
5252	EBNA3	GABPA	0.1231
5253	BZLF2	CT45A2	0.020639
5254	EBNA3	PRKDC	0.075273
5255	gL	MLC1	0.041973
5256	EBNA3	MTMR2	0.083225
5257	EBNA-LP	ETNK2	0.026937
5258	BILF1	S100A9	0.052081
5259	BGLF4	INTS4	0.20196
5260	EBNA1	MAPRE1	0.099596
5261	BGLF4	TSPYL5	0.15203
5262	BMLF1	GJD3	0.35982
5263	BZLF1	FAM84B	0.22211
5264	BZLF1	HPSE	0.081382
5265	EBNA6	HSP90B1	0.092246
5266	BHRF1	FAM151B	0.036204
5267	EBNA3	LY96	0.21706
5268	BHRF1	UPF3B	0.11533
5269	EBNA1	RNF139	0.088634
5270	BLLF2	ZNF100	0.02076
5271	BMLF1	GPC3	0.22467
5272	BMLF1	RALA	0.20574
5273	BZLF1	CELSR2	0.020198
5274	BGLF4	ARL4D	0.11769
5275	BMLF1	PLEKHA1	0.15377
5276	EBNA1	MAGEA2	0.13476
5277	EBNA-LP	HSD17B14	0.30329
5278	EBNA-LP	C6	0.29431
5279	EBNA-LP	EID1	0.2352
5280	BZLF1	ADAM23	0.10568
5281	BGLF4	TBK1	0.030974
5282	BMLF1	CTSH	0.13641
5283	BGLF4	PTPN1	0.046721
5284	BMLF1	GLT8D1	0.014742
5285	BLLF2	GOLGA8F	0.074477
5286	BGLF4	MCL1	0.10073
5287	BMLF1	GPR179	0.086481
5288	BGLF4	SAP30	0.1633
5289	BVRF1	CDH23	0.04884
5290	BZLF1	NCBP1	0.096385
5291	BMLF1	SLC6A18	0.021934
5292	BZLF1	CRH	0.085992
5293	BILF1	CAGE1	0.02808
5294	BMLF1	KCNJ4	0.34919
5295	EBNA-LP	EIF3D	0.38652
5296	BGLF4	MAL	0.18379
5297	BGLF4	CCNE2	0.084621
5298	BGLF4	INVS	0.14773
5299	gL	YJEFN3	0.061699
5300	BMLF1	CAMSAP2	0.4172
5301	BBRF2	TSPAN15	0.011502
5302	EBNA6	PRMT1	0.027791
5303	BMLF1	SPHK1	0.1594
5304	EBNA4	C2orf62	0.093585

5305	EBNA3	FAM184B	0.042479
5306	BGLF4	SAT1	0.18916
5307	BGLF4	PRICKLE3	0.18971
5308	BPLF1	CCDC152	0.015231
5309	EBNA6	ATP5SL	0.028063
5310	EBNA3	TUT1	0.24981
5311	BMLF1	NOS2	0.1195
5312	BGLF4	USP19	0.10758
5313	EBNA3	TGM2	0.26427
5314	EBNA1	NUAK1	0.086519
5315	BBRF2	POM121B	0.033738
5316	BGLF4	NRXN1	0.12891
5317	BZLF1	PPFIA3	0.20894
5318	BARF1	REPS1	0.19592
5319	BMLF1	POMT1	0.033926
5320	BHRF1	LINC00588	0.094276
5321	BGLF4	PLK4	0.047713
5322	EBNA3	PAX9	0.27223
5323	BLLF2	DENND1C	0.014078
5324	BZLF1	PIDD	0.18895
5325	BILF1	IQCF3	0.011605
5326	BOLF1	MYO1E	0.039481
5327	BGLF4	RNF182	0.11367
5328	BGLF4	CIB3	0.068073
5329	BGLF4	SGOL1	0.10287
5330	EBNA6	RSRC2	0.21966
5331	EBNA1	RTF1	0.085134
5332	BMLF1	SLC6A4	0.13145
5333	BLLF2	ZNF621	0.024354
5334	BGLF4	PCSK9	0.055907
5335	EBNA1	TNNI2	0.14395
5336	BZLF2	CCDC94	0.068043
5337	EBNA3	RASSF2	0.35689
5338	BGLF4	CCM2	0.20401
5339	BPLF1	SELO	0.095529
5340	BGLF4	RICTOR	0.14861
5341	BZLF1	POLR3F	0.10711
5342	EBNA1	RPS6KC1	0.095205
5343	BMLF1	PRDX6	0.33639
5344	BZLF2	LINS	0.01968
5345	EBNA4	CCDC150	0.11889
5346	EBNA1	EIF5A	0.075628
5347	BXRF1	RNH1	0.15028
5348	BGLF4	MKL2	0.16116
5349	BALF2	TLDC2	0.021649
5350	EBNA-LP	KIAA0319	0.30945
5351	BGLF4	ZFYVE28	0.15924
5352	gN	ubl4ab	0.037184
5353	EBNA1	ERRFI1	0.035142
5354	BZLF1	ASGR2	0.17009
5355	gB	LSM12	0.014846
5356	BZLF1	TADA3	0.029617
5357	BGLF4	TFAP2C	0.030826
5358	EBNA3	PHB2	0.23654
5359	BZLF2	MBD3L5	0.012307
5360	EBNA4	MRFAP1L1	0.31715
5361	BZLF1	NCOA6	0.079625
5362	BMLF1	PPP3CA	0.20433
5363	EBNA3	CDC45	0.25648
5364	BMLF1	SLC16A3	0.01633
5365	EBNA4	SAMD13	0.029108
5366	BZLF1	KPNA2	0.14009
5367	EBNA3	SGCA	0.16281
5368	EBNA3	RPL11	0.19676
5369	BMLF1	CENPA	0.1653
5370	EBNA-LP	PASK	0.18704
5371	EBNA3	SLC30A3	0.010762
5372	EBNA6	MMP20	0.053904

5373	BOLF1	MYL6	0.17346
5374	BMLF1	PLA2G2A	0.022487
5375	BGLF4	HINFP	0.041203
5376	BGLF4	CDH26	0.058173
5377	BMLF1	TOP3A	0.26237
5378	gL	LINS	0.048205
5379	BGLF4	ANP32A	0.1536
5380	EBNA1	TBRG1	0.14737
5381	BMLF1	SLX4IP	0.47224
5382	BGLF4	MELK	0.11144
5383	BGLF4	KDM2B	0.1111
5384	BGLF4	SPRR3	0.098148
5385	EBNA1	SPDL1	0.097102
5386	EBNA3	BAHD1	0.2623
5387	BGLF4	PSENEN	0.12872
5388	EBNA-LP	CYLD	0.086891
5389	EBNA3	PSMC6	0.12437
5390	EBNA3	ARHGDIA	0.24437
5391	BMLF1	ADCYAP1R1	0.11522
5392	BZLF1	N4BP2	0.16524
5393	BMLF1	ARID1A	0.19109
5394	EBNA4	FAM178B	0.17724
5395	BGLF4	MYCBP	0.17733
5396	gL	MIMI	0.12923
5397	EBNA-LP	INADL	0.30907
5398	EBNA-LP	LRCOL1	0.093215
5399	EBNA-LP	NLRC4	0.16136
5400	BPLF1	EHD4	0.027261
5401	EBNA3	PPP1R16A	0.41167
5402	EBNA6	EME1	0.12541
5403	BMLF1	RBP7	0.041125
5404	BARF1	SETD2	0.029549
5405	BMLF1	ARHGAP30	0.054603
5406	EBNA4	SMEK3P	0.14143
5407	BBRF2	NCOA5	0.01954
5408	BGLF4	CD97	0.030973
5409	BGLF4	TAF7	0.022597
5410	BZLF1	TAF4	0.025699
5411	EBNA3	TMED9	0.27088
5412	EBNA1	MGP	0.036538
5413	BTRF1	GGN	0.026018
5414	EBNA-LP	TBL1XR1	0.16509
5415	BMLF1	CD22	0.37941
5416	EBNA3	DAXX	0.11396
5417	BZLF2	LRP4	0.07655
5418	EBNA4	C9orf169	0.085441
5419	EBNA4	NDEL1	0.063009
5420	BZLF1	CRY1	0.039877
5421	BMLF1	HEXIM1	0.29232
5422	gL	GID4	0.064932
5423	EBNA-LP	AJUBA	0.078769
5424	EBNA3	POLD2	0.19975
5425	BILF1	KCNIP1	0.13704
5426	BMLF1	ACTB	0.13396
5427	EBNA-LP	ADAMTSL2	0.28345
5428	EBNA3	EHD2	0.36086
5429	BLLF1	SGD1	0.020277
5430	BMLF1	FBXW5	0.24362
5431	EBNA1	STAT3	0.015775
5432	EBNA3	N4BP1	0.34403
5433	BBRF2	ST8SIA3	0.01623
5434	EBNA1	VANGL1	0.20278
5435	BGLF4	ZXDA	0.059449
5436	gM	EFEMP2	0.1051
5437	gB	S100A11	0.048071
5438	EBNA3	RRAS2	0.23229
5439	BMLF1	KIAA1524	0.41722
5440	EBNA3	ANKRD28	0.37995

5441	EBNA6	TCERG1L	0.086951
5442	EBNA3	SPHKAP	0.41166
5443	EBNA1	YWHAG	0.14382
5444	BZLF1	PLSCR2	0.049691
5445	EBNA6	GAGE4	0.11453
5446	BXRF1	NECAB2	0.18692
5447	BGLF4	VPS26A	0.15222
5448	EBNA-LP	BLOC1S5	0.32462
5449	BPLF1	GAREML	0.15494
5450	BLLF2	ADA	0.011052
5451	BMLF1	PHF8	0.1786
5452	BPLF1	EDEM1	0.070962
5453	EBNA1	CX3CR1	0.081159
5454	BILF1	DNER	0.11471
5455	BGLF4	RAD51AP1	0.23831
5456	BPLF1	ANXA2R	0.12048
5457	BGLF4	MAST1	0.04568
5458	EBNA1	LCMT1	0.26186
5459	BZLF1	HSPE1	0.14701
5460	BRRF1	CLINT1	0.034845
5461	EBNA1	BAZ1B	0.10246
5462	BZLF1	FRS2	0.087577
5463	EBNA1	ASPH	0.099083
5464	EBNA-LP	SSSCA1	0.37965
5465	EBNA3	ARIH1	0.27751
5466	BMLF1	TARBP1	0.14961
5467	EBNA6	PPP2R3A	0.11394
5468	EBNA-LP	RAB3IL1	0.36402
5469	BZLF2	CALB1	0.11948
5470	EBNA4	RASSF4	0.14072
5471	BMLF1	ANKIB1	0.40834
5472	EBNA-LP	COL3A1	0.17022
5473	EBNA3	JUP	0.02331
5474	EBNA3	ZSWIM7	0.2993
5475	BGLF4	ARID1A	0.10985
5476	BPLF1	C19orf47	0.034107
5477	BLLF2	GOLGA6D	0.052161
5478	BLLF2	LUZP6	0.017699
5479	BGLF4	EFHB	0.068073
5480	BVRF1	THBD	0.1011
5481	EBNA3	GALK2	0.041709
5482	BVRF1	SNX9	0.075569
5483	BMLF1	C12orf57	0.044236
5484	BZLF1	HSD17B8	0.13534
5485	BGRF1/BDRF1	PPP3CB	0.042814
5486	BGLF4	CHMP4A	0.018169
5487	BZLF2	C2orf62	0.028654
5488	BGLF4	MED25	0.11235
5489	BLLF2	XBP1	0.023898
5490	BGLF4	HIRA	0.089468
5491	EBNA4	SAMD14	0.02951
5492	EBNA-LP	TRMT61B	0.43287
5493	LMP2	S100P	0.056227
5494	BZLF1	EEF1B2	0.1655
5495	EBNA4	SYT11	0.25586
5496	gL	SMEK3P	0.066102
5497	EBNA1	PRPF8	0.17205
5498	BZLF2	PPP2R3B	0.067939
5499	BGLF4	DACT1	0.094444
5500	BGLF4	CDHR1	0.037876
5501	EBNA2	FAM47C	0.035695
5502	LMP2	DHH	0.075093
5503	EBNA-LP	CSTF2	0.31408
5504	BOLF1	YAE1D1	0.022541
5505	EBNA1	ELOVL2	0.13167
5506	EBNA-LP	NUP98	0.17551
5507	EBNA-LP	IPO7	0.2441
5508	EBNA1	MEIS1	0.029426

EBNA-LP	PPFIA2	0.39353
gL	CCDC159	0.1048
EBNA3	PNRC2	0.28666
EBNA3	USP54	0.41089
BMLF1	DPYSL2	0.089265
EBNA-LP	MCM4	0.16888
BLLF1	YMR013W-A	0.038079
BGLF4	WRNIP1	0.12731
BZLF1	YWHAG	0.15276
EBNA3	UBE2M	0.23752
BLLF2	IFNW1	0.03465
EBNA-LP	RTP4	0.36413
EBNA2	FAT1	0.061992
EBNA6	SRI	0.090138
BGLF4	RAMP2	0.070866
BLLF2	TCEB3B	0.044696
BMLF1	FCHO2	0.31569
BZLF2	C16orf52	0.010617
BMLF1	SYNE2	0.1746
BXRF1	TESC	0.10677
EBNA4	SYBU	0.088697
EBNA3	YY1AP1	0.30228
EBNA3	OCA2	0.21989
BMLF1	RTN4	0.12035
BHRF1	MICU1	0.11752
gL	SNX29P2	0.057863
EBNA3	SLAIN1	0.15727
BGLF4	PCDHB12	0.05466
BZLF1	COMMD5	0.19547
BZLF1	TAB3	0.082516
BALF2	FAT4	0.12834
EBNA3	MAPT	0.074502
BZLF1	TICAM1	0.031521
EBNA3	GLUD1	0.2181
EBNA3	CEBPA	0.052413
EBNA1	COL4A3BP	0.037732
gL	MNCb-2875	0.045278
EBNA6	NCOA6	0.15732
BHRF1	FAM160A1	0.15486
BGLF4	PEX3	0.14241
BTRF1	SNX25	0.019066
EBNA3	RRAGC	0.17395
BGRF1/BDRF1	MYL6	0.19543
EBNA3	EIF4ENIF1	0.26812
BBRF2	ZSWIM1	0.019912
EBNA-LP	STAM	0.28191
BZLF1	ZMYND11	0.087267
BMLF1	PCDHGA3	0.22489
BMLF1	YAP1	0.12057
EBNA3	ACVRL1	0.019065
BNLF2b	YIPF5	0.05388
BMLF1	DNAJC15	0.015703
EBNA6	OFD1	0.10985
EBNA-LP	PSMA4	0.10156
BXRF1	DTNA	0.081806
EBNA3	DLG4	0.047681
EBNA-LP	ATP8B1	0.24185
BGLF4	KLHL3	0.10681
EBNA1	RBM15B	0.14856
EBNA3	PRPF40A	0.28779
BGLF4	TNIP1	0.15158
EBNA6	MELK	0.048618
EBNA4	S100A11	0.1737
gL	EFCAB4B	0.036204
EBNA-LP	ARF1	0.12707
EBNA-LP	SMAGP	0.051507
BMLF1	SCAMP2	0.25505
EBNA-LP	WIPF2	0.39355

5577	BGLF4	SELL	0.14724
5578	EBNA3	MMP15	0.38568
5579	EBNA1	ADAM12	0.056009
5580	BZLF2	PPEF2	0.069157
5581	BZLF1	CLEC2A	0.21919
5582	BGLF4	IL6R	0.083645
5583	BGLF4	TF	0.13018
5584	BZLF2	SMEK3P	0.035008
5585	BLLF2	CTIF	0.030549
5586	BMLF1	CCNC	0.17587
5587	BGLF4	NUP153	0.10334
5588	BMLF1	TMPRSS5	0.030365
5589	EBNA3	HOXA10	0.14725
5590	EBNA3	S100G	0.19298
5591	EBNA1	PPP2R5D	0.13014
5592	BMLF1	ABCD2	0.27075
5593	BMLF1	UTP20	0.30674
5594	BLLF1	DAG1	0.023409
5595	EBNA1	UBR5	0.10335
5596	BBRF2	RHD	0.053219
5597	BGLF4	SSBP1	0.1899
5598	EBNA3	NUP43	0.20164
5599	BMLF1	ARF1	0.14228
5600	EBNA-LP	GLYCTK	0.42023
5601	BZLF1	BCAS3	0.18943
5602	BMLF1	FBXW7	0.12598
5603	EBNA6	EIF3J	0.12376
5604	BMLF1	BTNL8	0.44386
5605	BLLF2	XPO7	0.066465
5606	EBNA-LP	NMRK1	0.02529
5607	BLLF2	HILS1	0.044492
5608	BMLF1	TMOD4	0.014055
5609	EBNA1	MYLK	0.065788
5610	EBNA6	S100A7	0.1051
5611	BGLF4	CLPP	0.21375
5612	BZLF1	MAL	0.18751
5613	BHRF1	TCERG1L	0.061679
5614	BLLF1	FAM188A	0.079353
5615	BMLF1	POM121C	0.29692
5616	EBNA3	FANCE	0.37075
5617	BVRF1	YMR013W-A	0.0539
5618	EBNA3	UBQLN2	0.35718
5619	EBNA3	FLNC	0.32681
5620	BZLF1	NDC80	0.11683
5621	BGLF4	UXT	0.11483
5622	EBNA3	PIFO	0.23552
5623	BMLF1	CALM1	0.23247
5624	EBNA-LP	AKAP9	0.19592
5625	EBNA3	L1TD1	0.013726
5626	BMLF1	NPC1L1	0.37215
5627	BZLF2	S100A7	0.095153
5628	EBNA-LP	CRTAC1	0.21388
5629	BMLF1	SUV39H1	0.19951
5630	BPLF1	EIF4G2	0.025156
5631	EBNA3	MAP3K1	0.034219
5632	BGRF1/BDRF1	EIF4E	0.019003
5633	BLLF2	OR2D3	0.084698
5634	BMLF1	MAP2K3	0.11721
5635	EBNA3	ARHGAP26	0.15644
5636	BZLF1	FAT1	0.25883
5637	EBNA3	FOXRED2	0.44521
5638	EBNA3	ZNF639	0.092936
5639	BMLF1	MAD2L2	0.2393
5640	EBNA-LP	VEGFA	0.012704
5641	BMLF1	SHBG	0.063184
5642	EBNA3	ZGPAT	0.13847
5643	BZLF1	NDOR1	0.12488
5644	EBNA-LP	POLE4	0.29882

5645	EBNA3	SPATA18	0.25299
5646	BXRF1	HSP90B1	0.091084
5647	BMLF1	SRSF2	0.31964
5648	gL	NUDCD3	0.087799
5649	BLLF2	EPC2	0.024588
5650	BLLF2	UCKL1	0.045116
5651	BMLF1	AKT2	0.037669
5652	BGLF4	EEF1A1	0.039511
5653	EBNA3	BICD2	0.40458
5654	BNLF2b	ANAPC10	0.030934
5655	EBNA-LP	FASN	0.20525
5656	BMLF1	SPTSSB	0.31925
5657	EBNA-LP	LCP1	0.11603
5658	BLLF2	DNAJA1	0.013486
5659	BMLF1	VSIG4	0.39725
5660	BLLF2	NUCB2	0.012419
5661	EBNA4	TP53INP1	0.19995
5662	BBRF2	AKAP8L	0.055272
5663	EBNA3	RPS10	0.19321
5664	BZLF1	RAD9B	0.2044
5665	BGRF1/BDRF1	EFEMP1	0.19708
5666	BLLF2	SLC16A9	0.019793
5667	BGLF4	KIF7	0.13869
5668	EBNA1	ITGA1	0.12953
5669	EBNA4	FAM178A	0.2218
5670	BGRF1/BDRF1	PRSS2	0.18526
5671	EBNA1	GREM1	0.027084
5672	EBNA3	ATXN1L	0.078603
5673	EBNA3	SYNGR3	0.13319
5674	BGLF4	BMPR2	0.017579
5675	EBNA-LP	APTX	0.12257
5676	BVRF1	COMMD9	0.024488
5677	EBNA3	BAX	0.014
5678	EBNA-LP	ECHS1	0.21511
5679	EBNA6	WIBG	0.20405
5680	BBRF2	SLAMF7	0.02861
5681	BLLF1	ARMC12	0.014016
5682	BMLF1	SCHIP1	0.40456
5683	EBNA2	REPS2	0.13862
5684	LMP2	SPARC	0.049328
5685	BGLF4	CEP63	0.17897
5686	EBNA-LP	PCDH18	0.17399
5687	EBNA1	CHMP3	0.12359
5688	BZLF1	ZNF408	0.0968
5689	BGLF4	LONRF3	0.18483
5690	EBNA1	INS	0.025271
5691	EBNA3	NOXO1	0.24081
5692	BRRF1	NELL1	0.11892
5693	EBNA1	TRIM32	0.083098
5694	BZLF1	COTL1	0.18267
5695	EBNA-LP	REL	0.15687
5696	EBNA1	EVI5L	0.16509
5697	EBNA-LP	SCYL3	0.33347
5698	BMLF1	GGA3	0.29188
5699	BGLF4	PCDHA4	0.05466
5700	EBNA3	CHAF1B	0.21707
5701	BGLF4	GABARAP	0.14456
5702	EBNA6	TTN	0.030379
5703	EBNA3	RHOBTB3	0.32524
5704	EBNA2	CDH1	0.024803
5705	BZLF1	EFEMP2	0.42247
5706	BMLF1	STAT2	0.28349
5707	EBNA3	PEX13	0.29249
5708	EBNA3	TRAF3IP1	0.41166
5709	BMLF1	MAL	0.32712
5710	BRRF1	ALKBH8	0.023665
5711	BMLF1	ASPH	0.19112
5712	BGLF4	BRD4	0.12849

5713	gM	PPP3CA	0.0115
5714	EBNA3	WWP2	0.12231
5715	BMLF1	NPC1	0.15528
5716	BZLF1	MICAL1	0.21015
5717	BMLF1	SPG11	0.4079
5718	BZLF1	MTF1	0.016204
5719	BGLF4	NID2	0.39033
5720	BMLF1	SECTM1	0.082456
5721	BZLF1	FNTA	0.16833
5722	EBNA4	ARMC12	0.14854
5723	BMLF1	NOS3	0.040988
5724	EBNA3	ATF3	0.13806
5725	EBNA3	UPF3A	0.36633
5726	EBNA3	SNTB2	0.31869
5727	BMLF1	ELN	0.30559
5728	BZLF2	THBS1	0.016485
5729	BGLF4	EIF3C	0.2373
5730	EBNA6	GUCD1	0.085035
5731	EBNA6	SPAC5H10.02c	0.017544
5732	EBNA-LP	REST	0.017178
5733	EBNA3	GDI1	0.29894
5734	BBRF2	EIF2A	0.010648
5735	EBNA-LP	MORF4L1	0.25126
5736	BLLF1	SNX19	0.056062
5737	EBNA-LP	PPP1R12A	0.17207
5738	EBNA3	DMD	0.14189
5739	EBNA-LP	SNX2	0.28796
5740	EBNA-LP	P4HTM	0.15364
5741	EBNA-LP	LALBA	0.15379
5742	EBNA6	GADD45A	0.096934
5743	EBNA3	FSCB	0.17987
5744	BMLF1	HSPA2	0.33738
5745	BALF2	ANXA7	0.050011
5746	EBNA1	ARHGEF12	0.049357
5747	EBNA2	ANXA2R	0.044038
5748	EBNA-LP	RNF32	0.31943
5749	BGLF4	PPP1R12A	0.091478
5750	BLLF2	SPINK6	0.013369
5751	EBNA3	MYCBPAP	0.30654
5752	EBNA1	DNASE1L3	0.021862
5753	gL	S100A12	0.033601
5754	EBNA-LP	GUCY2C	0.1179
5755	EBNA3	LIMK1	0.13609
5756	EBNA1	MAP2K5	0.06812
5757	BZLF1	TESC	0.223
5758	EBNA6	GEMIN7	0.11806
5759	BZLF1	TRIM29	0.0539
5760	EBNA1	DYRK1A	0.033968
5761	BGLF4	DPPA4	0.19941
5762	BPLF1	FBLN5	0.1322
5763	EBNA3	RAB24	0.32065
5764	EBNA-LP	NDUFAF1	0.35403
5765	BZLF1	PSMG2	0.17497
5766	EBNA-LP	KLK13	0.33948
5767	BGLF4	MEA1	0.20982
5768	EBNA6	YDL022C-A	0.033603
5769	BGLF4	GP6	0.17086
5770	BRRF1	PPP3CA	0.041132
5771	BGLF4	DTX3L	0.1334
5772	EBNA1	SPACA3	0.1782
5773	EBNA6	XAGE3	0.2897
5774	EBNA-LP	STK10	0.22996
5775	EBNA4	C16orf93	0.11987
5776	BGLF4	BECN1	0.11445
5777	BMLF1	HERC2	0.19314
5778	EBNA6	TTC22	0.023197
5779	EBNA1	CLN3	0.027274
5780	EBNA6	CCDC7	0.094569

5781	BGLF4	STXBP2	0.12979
5782	BGLF4	GOLPH3	0.16517
5783	BZLF1	FAM231D	0.011322
5784	BLLF2	G4	0.056009
5785	EBNA3	BAZ2A	0.20686
5786	EBNA1	PUM2	0.24571
5787	EBNA-LP	DUSP2	0.11499
5788	EBNA1	FBXO27	0.20872
5789	BMLF1	PRAMEF13	0.096914
5790	BBRF2	KCNJ18	0.013023
5791	LMP2	HSP90B1	0.060012
5792	BGLF4	PCDHAC2	0.05466
5793	EBNA-LP	SNX5	0.096567
5794	BZLF1	SYNE1	0.11937
5795	EBNA-LP	ANXA1	0.085563
5796	BZLF1	YY1	0.044096
5797	BOLF1	KCNIP1	0.12468
5798	BHRF1	PPP3CB	0.03032
5799	BMLF1	SLC4A9	0.013281
5800	EBNA3	PCM1	0.19354
5801	BZLF1	C1QTNF3	0.13626
5802	BGLF4	HIVEP1	0.13844
5803	EBNA6	C22orf42	0.035422
5804	BGLF4	PSPH	0.025965
5805	BLLF2	ZNF267	0.020413
5806	EBNA-LP	BLOC1S6	0.16595
5807	BGLF4	UBE2A	0.10062
5808	BZLF1	EIF3D	0.22706
5809	EBNA3	EME1	0.2933
5810	EBNA-LP	VAPA	0.13935
5811	BZLF1	ATP6V0A1	0.17415
5812	BMLF1	HRAS	0.062937
5813	EBNA3	HAND2	0.065625
5814	BZLF1	EEF1G	0.15571
5815	BGLF4	KIRREL3	0.20217
5816	EBNA3	IFT20	0.29941
5817	BMLF1	COL4A3	0.15467
5818	EBNA-LP	RDX	0.26038
5819	EBNA-LP	NCOA2	0.084184
5820	BZLF1	KIF14	0.12565
5821	gM	FAM47C	0.025073
5822	EBNA3	CD72	0.27359
5823	BNLF2b	MMRN2	0.011268
5824	BMLF1	AGR3	0.50141
5825	BZLF1	CTNNA3	0.08388
5826	EBNA1	USP10	0.10726
5827	EBNA-LP	KLK3	0.42019
5828	BVRF1	THBS2	0.1377
5829	EBNA2	FASN	0.045018
5830	BMLF1	FBXL3	0.3322
5831	gL	S100A6	0.044651
5832	gN	SPARC	0.05414
5833	BZLF1	DLGAP1	0.16747
5834	BHRF1	PKD2L1	0.075532
5835	BGLF4	ERCC4	0.058866
5836	EBNA1	RAP2A	0.087945
5837	EBNA3	TXNDC9	0.40042
5838	BALF2	YJR030C	0.012303
5839	BGLF4	DGCR8	0.18324
5840	BGLF4	ZNRF1	0.16239
5841	EBNA1	CYFIP2	0.16664
5842	BRRF1	STIM1	0.098935
5843	EBNA3	IFRD2	0.41354
5844	BZLF1	AQP1	0.047509
5845	BZLF2	NACA	0.021073
5846	BMLF1	REST	0.036579
5847	EBNA3	PCGF3	0.26919
5848	EBNA1	CXXC1	0.09203

5849	BZLF1	SLX4	0.15803
5850	BGLF4	MARK3	0.069254
5851	BMLF1	TUBB4A	0.12692
5852	BGLF4	NPM3	0.19023
5853	BARF1	DTNBP1	0.019997
5854	EBNA-LP	DYNC1H1	0.21029
5855	EBNA-LP	ZW10	0.13136
5856	EBNA-LP	EXOC8	0.17583
5857	BZLF1	DOCK3	0.20243
5858	BMLF1	NME1	0.19104
5859	EBNA-LP	ERCC6	0.1023
5860	gL	NDEL1	0.041403
5861	EBNA-LP	CNOT6	0.24639
5862	BMLF1	GLTP	0.013846
5863	EBNA-LP	CAGE1	0.023073
5864	EBNA4	CCDC42	0.03077
5865	BMLF1	RCOR3	0.33802
5866	BMLF1	MIEF1	0.34327
5867	BMLF1	KCNJ3	0.29734
5868	EBNA1	KIF7	0.12795
5869	EBNA3	AURKAIP1	0.30396
5870	EBNA1	SLC9A7	0.11134
5871	BGLF4	POLR2E	0.063138
5872	EBNA3	OGN	0.28403
5873	BZLF1	LMO2	0.074039
5874	BZLF1	UHMK1	0.14142
5875	BZLF2	TCP10L2	0.046541
5876	EBNA3	RPL6	0.063047
5877	EBNA3	PRAME	0.23532
5878	BBRF2	OR10AD1	0.031196
5879	BGLF4	PDCD4	0.20663
5880	BZLF1	CASR	0.13768
5881	gL	MASP1	0.11775
5882	BGLF4	GMNN	0.13559
5883	BLLF2	SLC39A9	0.033565
5884	EBNA-LP	SPATA31E1	0.18933
5885	BPLF1	CBY3	0.051144
5886	BMLF1	RAE1	0.023066
5887	BGLF4	AATK	0.066024
5888	BMLF1	GYG1	0.33094
5889	EBNA1	ELF1	0.04966
5890	EBNA3	STT3A	0.34188
5891	BZLF1	TUBB4A	0.01348
5892	BMLF1	SPC25	0.27938
5893	EBNA-LP	SH3KBP1	0.33349
5894	BMLF1	RPL10A	0.039281
5895	EBNA3	SPOP	0.36193
5896	EBNA3	MAPKAPK2	0.082139
5897	BBRF2	TSPAN32	0.017195
5898	BMLF1	CDCA8	0.2424
5899	gB	HEATR5B	0.022553
5900	EBNA3	BAG3	0.32366
5901	BLLF1	PCDH17	0.09592
5902	EBNA3	PNMA1	0.31934
5903	BGLF4	IFITM1	0.13343
5904	BGLF4	NUCB1	0.065213
5905	BZLF1	OCM2	0.057416
5906	BZLF2	SLC11A2	0.020101
5907	BMLF1	TNFRSF1B	0.28718
5908	BZLF1	MAPT	0.027891
5909	BGLF4	PHYH	0.091299
5910	BMLF1	PTPRE	0.16534
5911	EBNA-LP	CIC	0.31576
5912	EBNA3	PLK4	0.17647
5913	BZLF1	PARP9	0.26643
5914	BGLF4	SMNDC1	0.22232
5915	EBNA3	NPRL2	0.29141
5916	EBNA4	RITA1	0.16294

5917	BNLF2b	TBC1D28	0.013968
5918	BOLF1	C11orf72	0.018301
5919	BOLF1	ERICH1-AS1	0.016195
5920	BRRF1	MAP1LC3C	0.022638
5921	EBNA3	VAMP3	0.2428
5922	BGLF4	TULP1	0.13029
5923	BGLF4	SPSB2	0.20775
5924	BZLF1	NEK2	0.032865
5925	BMLF1	NSUN7	0.077134
5926	BGLF4	HSPA1A	0.05846
5927	BGLF4	CNTROB	0.17801
5928	BZLF1	FBXO3	0.1911
5929	BMLF1	SLC9A1	0.14299
5930	BLLF1	HSPA5	0.037238
5931	BLLF2	ZNF362	0.01242
5932	BNLF2b	FAM19A3	0.16762
5933	BARF1	FAT4	0.20957
5934	EBNA6	SNURFL	0.13734
5935	EBNA-LP	DUOX1	0.20819
5936	BGLF4	LPXN	0.11869
5937	EBNA-LP	ENG	0.073853
5938	BZLF1	PLA2G16	0.088537
5939	BGLF4	PLCG2	0.04483
5940	BMLF1	CDH19	0.21805
5941	EBNA1	VDAC1	0.076408
5942	EBNA1	PHB	0.06403
5943	BLLF2	FIP1L1	0.196
5944	EBNA2	LSM12	0.015991
5945	BMLF1	ZC3H15	0.35143
5946	EBNA-LP	RNF146	0.21682
5947	BGLF4	UBA6	0.096506
5948	BGLF4	PSMC2	0.060205
5949	BMLF1	STRN	0.27896
5950	EBNA1	ZCCHC8	0.13413
5951	BLLF2	ZNF660	0.023238
5952	EBNA3	PTPN3	0.12977
5953	BMLF1	PLRG1	0.34239
5954	EBNA-LP	CAPS2	0.2099
5955	EBNA4	KIAA0087	0.21412
5956	BOLF1	PPEF1	0.082963
5957	BHRF1	XAGE3	0.18173
5958	BZLF1	RARRES2	0.15911
5959	BMLF1	PCYT1A	0.012738
5960	EBNA3	NR1H4	0.045874
5961	BGLF4	JAG2	0.22919
5962	BZLF1	NDUFAB1	0.032141
5963	BILF1	LRP4	0.10073
5964	EBNA1	MTCH1	0.16269
5965	EBNA4	F2	0.055026
5966	BLLF2	ATG10	0.060522
5967	EBNA3	SRGAP2	0.16668
5968	EBNA1	TCAP	0.12754
5969	BMLF1	HPN	0.17949
5970	EBNA-LP	CACNA1I	0.288
5971	BZLF1	MAPK8IP1	0.054444
5972	BZLF2	VLDLR	0.057915
5973	BMLF1	C1R	0.2496
5974	EBNA-LP	PAX3	0.11913
5975	BPLF1	JAG2	0.093014
5976	BMLF1	SULF1	0.05971
5977	BGLF4	TOMM20	0.1119
5978	gL	ZNF767	0.061467
5979	BZLF1	POP7	0.16251
5980	EBNA6	UNQ6125/PRO20090	0.20862
5981	BZLF1	LIN37	0.22205
5982	EBNA-LP	GNAI2	0.096788
5983	BZLF1	BCKDK	0.10005
5984	BDLF1	VIL1	0.02295

5985	BMLF1	GTF3C1	0.28608
5986	BLLF1	MEGF6	0.11749
5987	EBNA1	TRIM33	0.047992
5988	BZLF1	AKTIP	0.13776
5989	BZLF1	GP1BA	0.12183
5990	EBNA3	HDAC5	0.050671
5991	BZLF1	SUN2	0.096513
5992	gL	UPF3A	0.15042
5993	BHRF1	CLIP1	0.06053
5994	BZLF1	MEF2D	0.050748
5995	EBNA1	SFN	0.055288
5996	BBRF2	ZNF256	0.011822
5997	EBNA3	NDOR1	0.25024
5998	BLLF2	SDSL	0.058014
5999	EBNA-LP	STAMBPL1	0.34414
6000	BZLF1	BBS12	0.18564
6001	EBNA1	TCERG1	0.13913
6002	BGRF1/BDRF1	DOC2A	0.012136
6003	BLLF2	SCAMP5	0.036502
6004	BZLF1	BEGAIN	0.2221
6005	EBNA1	SAMHD1	0.13103
6006	BMLF1	EIF2B4	0.26223
6007	BLLF2	BAHD1	0.022638
6008	BBRF2	CDC42EP3	0.016663
6009	EBNA6	NXF3	0.093677
6010	BZLF1	MAGEB18	0.23617
6011	BZLF1	BMPR1B	0.03498
6012	BXRF1	FAM47C	0.032071
6013	BMLF1	RWDD3	0.41608
6014	EBNA-LP	DLG4	0.048258
6015	BZLF1	ELMO1	0.089411
6016	BGLF4	DMAP1	0.1464
6017	BMLF1	RAF1	0.048836
6018	EBNA-LP	SPTAN1	0.37413
6019	EBNA3	ELOVL1	0.25409
6020	BLLF1	LURAP1L	0.047952
6021	EBNA-LP	LIN28A	0.19631
6022	EBNA3	DNM1	0.11511
6023	EBNA3	ATCAY	0.26993
6024	EBNA6	C16orf11	0.041635
6025	EBNA3	CSRP2BP	0.2616
6026	BVRF1	EFCAB4B	0.11583
6027	BLLF2	OR9K2	0.081986
6028	BGLF4	PCDH1	0.049259
6029	BGLF4	APEX1	0.071827
6030	EBNA-LP	ZNHIT1	0.39136
6031	EBNA3	KAT6A	0.13048
6032	BBRF2	OR2T4	0.032661
6033	BGLF4	CITED2	0.029834
6034	EBNA-LP	MED30	0.27658
6035	EBNA3	PSPH	0.09901
6036	BZLF1	YBX1	0.038552
6037	EBNA-LP	MLST8	0.2955
6038	BGLF4	LTF	0.12348
6039	BMLF1	SECISBP2L	0.025416
6040	BLLF2	PRDM5	0.017942
6041	BMLF1	TARBP2	0.21597
6042	EBNA1	CALCOCO2	0.14723
6043	EBNA3	OGDHL	0.36723
6044	EBNA-LP	SATB1	0.10488
6045	EBNA-LP	MUC1	0.2009
6046	BGLF4	DFNB31	0.12307
6047	BDLF1	DHH	0.031731
6048	EBNA4	PRMT1	0.045853
6049	BZLF1	ETV7	0.054087
6050	BMLF1	SNX20	0.33196
6051	BLLF2	NTMT1	0.012773
6052	BBRF2	PSMD2	0.015126

6053	EBNA-LP	ASAP1	0.10031
6054	EBNA1	UACA	0.10733
6055	EBNA1	CSRP2BP	0.11689
6056	BMLF1	TRIP6	0.24323
6057	EBNA1	SOX1	0.047164
6058	EBNA6	LINC00469	0.012465
6059	EBNA-LP	KCNN4	0.25023
6060	BOLF1	MYL2	0.18933
6061	EBNA1	PCDHGA4	0.037832
6062	BZLF1	NLGN4Y	0.15433
6063	EBNA3	TERF2IP	0.14302
6064	BGLF4	DIABLO	0.15186
6065	EBNA1	ZXDA	0.020114
6066	BZLF2	RUNDC1	0.016888
6067	BLLF2	RTN3	0.010062
6068	EBNA3	ZNF3	0.10405
6069	BGRF1/BDRF1	SETD2	0.027993
6070	BZLF2	CDKN2AIPNL	0.08172
6071	BMLF1	MSLN	0.35589
6072	EBNA-LP	TRIM44	0.35366
6073	EBNA4	CCDC54	0.050433
6074	BGLF4	TNNI3	0.07174
6075	BZLF1	PARK7	0.14322
6076	BILF1	RCN3	0.22303
6077	EBNA-LP	SNRPE	0.23587
6078	EBNA-LP	MBD1	0.14787
6079	BMLF1	DDX58	0.10632
6080	EBNA3	SPHK2	0.30623
6081	EBNA1	AGTR1	0.058053
6082	BZLF1	DTX3L	0.13333
6083	BGLF4	RNF6	0.098214
6084	BMLF1	CLIC4	0.21657
6085	BLLF2	DMRTA1	0.024694
6086	BGLF4	HAND1	0.014923
6087	EBNA4	ANGEL2	0.056986
6088	gB	PITPNM3	0.053879
6089	EBNA3	DGKQ	0.24317
6090	EBNA1	USP26	0.15461
6091	EBNA1	STMN2	0.13273
6092	BMLF1	VPS52	0.37317
6093	EBNA6	MYOZ3	0.12811
6094	BGLF4	KIAA2018	0.038448
6095	BLLF2	EMID1	0.037069
6096	EBNA3	CCDC141	0.43795
6097	EBNA3	IRAK1	0.074909
6098	gL	ANXA2R	0.17323
6099	BNLF2b	KCNJ18	0.01217
6100	EBNA-LP	CDC27	0.2145
6101	EBNA4	SNX6	0.065623
6102	EBNA3	HRG	0.11012
6103	EBNA3	LATS1	0.069606
6104	EBNA-LP	AHI1	0.12423
6105	BPLF1	FAM228A	0.012955
6106	EBNA-LP	NCOA7	0.27043
6107	EBNA3	MAGEA3	0.43795
6108	BZLF1	PLCB1	0.012255
6109	gL	KHDC1L	0.016747
6110	EBNA3	ACOT8	0.17771
6111	EBNA3	PSMD2	0.16298
6112	BMLF1	ETNK2	0.040508
6113	BVRF1	MELK	0.037899
6114	EBNA1	CDHR2	0.37615
6115	BMLF1	DLX4	0.24357
6116	EBNA6	BEND4	0.037569
6117	BGLF4	RNF103	0.12405
6118	EBNA3	SULF2	0.041168
6119	EBNA-LP	RXRA	0.022674
6120	BMLF1	E2F4	0.10816

6121	EBNA4	C9orf153	0.084437
6122	EBNA3	GTF2E1	0.21229
6123	EBNA1	UNC119B	0.19375
6124	EBNA4	PLCE1	0.026797
6125	BZLF1	RASSF5	0.17225
6126	BGLF4	NKX2-1	0.018905
6127	BGLF4	MTPAP	0.12437
6128	BMLF1	SEC31A	0.21177
6129	EBNA3	GOLPH3	0.29249
6130	BMLF1	AGO2	0.14548
6131	BBRF2	TAS2R10	0.043074
6132	BZLF1	PBRM1	0.14179
6133	BDLF1	PRSS2	0.064138
6134	gM	HSP90B1	0.058456
6135	EBNA3	HIF1AN	0.16341
6136	EBNA3	MFAP2	0.12635
6137	BGRF1/BDRF1	EHD4	0.052124
6138	EBNA-LP	SPTBN4	0.091993
6139	EBNA1	NUTF2	0.18803
6140	BMLF1	TRIM37	0.22717
6141	EBNA3	SETD1A	0.32414
6142	BMLF1	PCDHB11	0.19264
6143	gM	PRF1	0.030927
6144	BGLF4	TJP2	0.17289
6145	BGLF4	STARD13	0.16542
6146	EBNA6	C5orf48	0.17602
6147	BBRF2	SERTAD2	0.032467
6148	EBNA-LP	SVOP	0.023362
6149	EBNA4	KHNYN	0.14327
6150	BGLF4	HUS1	0.13524
6151	BGLF4	F2R	0.027941
6152	BZLF1	PEX16	0.13207
6153	EBNA-LP	NSUN5P2	0.087967
6154	BBRF2	ANO4	0.021152
6155	BGRF1/BDRF1	EGF	0.065124
6156	gL	FAM219B	0.018494
6157	BLLF2	GPR89C	0.01054
6158	BMLF1	SLC2A1	0.049295
6159	EBNA3	RPL26	0.037287
6160	BBRF2	BEX4	0.021144
6161	BMLF1	TTYH1	0.14623
6162	EBNA1	PALLD	0.14517
6163	BGLF4	SLC4A1	0.13448
6164	EBNA1	PTPRM	0.030365
6165	BZLF1	TNFRSF14	0.14031
6166	EBNA3	DOCK9	0.20905
6167	BGLF4	SEPT8	0.18614
6168	EBNA1	UBA1	0.094375
6169	BPLF1	RAB11FIP4	0.1921
6170	gB	SNX19	0.018131
6171	EBNA3	ING2	0.14539
6172	BMLF1	PIP5K1B	0.35538
6173	BMLF1	PCDHB4	0.17963
6174	EBNA3	MAGEC1	0.43795
6175	EBNA1	TPD52	0.39603
6176	BZLF1	USH2A	0.12385
6177	BGLF4	LSM6	0.15245
6178	BMLF1	TRPC6	0.22365
6179	EBNA-LP	PCDHGB4	0.1739
6180	EBNA4	UPF3B	0.27226
6181	EBNA1	SYTL2	0.10011
6182	BZLF2	KIAA1908	0.030494
6183	BLLF2	CDCA4	0.023863
6184	EBNA-LP	PSG5	0.40584
6185	BZLF1	SAFB2	0.18667
6186	BLLF2	RABEP2	0.17848
6187	BBRF2	TNNI3	0.010116
6188	BMLF1	LBP	0.13151

EBNA4	CENPF	0.044002
EBNA2	S100A12	0.067486
EBNA-LP	MRPL12	0.3904
EBNA4	FNIP2	0.40281
BZLF1	SPINK1	0.1966
BLLF2	SLC39A8	0.013295
EBNA-LP	VAPB	0.14176
BZLF1	DNASE1	0.13998
BALF2	AP1B1	0.015522
EBNA1	CBLB	0.13581
BZLF1	FEZ1	0.14634
EBNA1	WASF1	0.1117
BZLF1	EIF3L	0.23073
BGLF4	LILRB2	0.1103
EBNA3	HNRNPR	0.31133
EBNA1	LRRC4C	0.20278
BLLF1	LINS	0.015366
BMLF1	RBMS2	0.010182
BBRF2	ZNF763	0.013084
BGLF4	DSG3	0.049477
BGLF4	CSRP2BP	0.13869
BGLF4	PKDREJ	0.055134
EBNA-LP	PFN2	0.2977
EBNA3	FEZ2	0.38428
EBNA3	FAM21C	0.33803
BGLF4	FZD1	0.043291
BZLF2	GOLGA6L18	0.064501
gL	THBS2	0.049285
EBNA1	INHA	0.068247
EBNA-LP	STXBP4	0.14221
EBNA3	SLC16A8	0.077125
EBNA6	NAF1	0.19192
BLLF2	PLXDC2	0.046883
BPLF1	EHD1	0.04076
BMLF1	UAP1L1	0.018345
BGLF4	SERPINE2	0.040368
BLLF2	ZNF876P	0.011129
EBNA1	SEPT12	0.11813
BMLF1	PLXNB3	0.34736
EBNA-LP	UBE2I	0.079329
EBNA3	COL4A2	0.27145
BZLF1	SIKE1	0.17492
BHRF1	KIAA1751	0.035298
EBNA1	ANXA8	0.022372
BXRF1	DPP4	0.020597
BGLF4	WASH1	0.16128
BMLF1	ASB13	0.43457
EBNA-LP	MBOAT7	0.33189
BZLF1	DOHH	0.11899
gB	RPH3A	0.016898
EBNA3	L3MBTL1	0.080056
BZLF1	ATP11A	0.10841
BZLF1	PPP2R3B	0.32512
EBNA-LP	CYFIP2	0.32801
EBNA1	CALR	0.041195
BGLF4	HADHB	0.079345
BMLF1	COG1	0.31727
EBNA3	TAF8	0.2246
BZLF1	PPP1R9B	0.092244
BLLF2	SERTAD2	0.10879
BGLF4	TRAF3IP2	0.19817
BMLF1	C1QTNF3	0.26311
BGLF4	ATP7B	0.045466
BLLF2	OR2T10	0.080961
BMLF1	CENPT	0.26143
BGLF4	DLG1	0.014147
gL	GR6	0.041338
BMLF1	SLC17A4	0.02815

6257	EBNA1	BARD1	0.11601
6258	BMLF1	PTPRC	0.020223
6259	BZLF1	UBL4A	0.19506
6260	EBNA1	C1orf94	0.22232
6261	EBNA3	KNTC1	0.20433
6262	EBNA1	FPR1	0.12236
6263	EBNA1	KIF24	0.15112
6264	EBNA4	C1orf228	0.084731
6265	EBNA4	CCDC65	0.049041
6266	BTRF1	CGREF1	0.01153
6267	EBNA-LP	RAB11A	0.18121
6268	BOLF1	DST	0.094214
6269	BGLF4	HMG20A	0.096494
6270	BMLF1	RFC1	0.15346
6271	BMLF1	CLGN	0.17183
6272	EBNA-LP	RHBDD1	0.21706
6273	BZLF1	MALL	0.16996
6274	BXRF1	REPS2	0.18625
6275	BGLF4	MAT2B	0.10479
6276	EBNA1	HOXB9	0.034345
6277	BGLF4	ARHGAP44	0.18795
6278	BGLF4	SHANK3	0.080443
6279	BZLF1	GIPC1	0.07492
6280	BGLF4	BMX	0.034995
6281	BLLF2	ZNF705D	0.019705
6282	EBNA4	DMAP1	0.1296
6283	BGLF4	ADAM9	0.039916
6284	BLLF2	NAP1L3	0.013502
6285	BMLF1	ZGPAT	0.18219
6286	BGLF4	CEP76	0.18566
6287	BGLF4	S100A9	0.11413
6288	BMLF1	RPUSD4	0.52663
6289	gN	ANXA7	0.038813
6290	BGRF1/BDRF1	EHD2	0.028074
6291	EBNA6	C17orf59	0.30813
6292	EBNA3	C6orf229	0.060313
6293	EBNA3	IGBP1	0.2296
6294	EBNA3	PEF1	0.67161
6295	EBNA1	TAGLN2	0.19597
6296	BGLF4	CCR5	0.074572
6297	EBNA3	MAPK14	0.026928
6298	BMLF1	NELFCD	0.29556
6299	EBNA4	FAT1	0.11467
6300	BVRF1	CLIP1	0.06563
6301	BZLF2	MSANTD2	0.014962
6302	EBNA-LP	KCNH2	0.14833
6303	BZLF1	CDK11B	0.059526
6304	BGLF4	THBS3	0.042815
6305	BGLF4	C16orf70	0.20864
6306	EBNA1	PCDHGA11	0.038413
6307	BLLF2	ZNF850	0.012401
6308	EBNA3	FAM178B	0.065733
6309	BMLF1	ADORA1	0.05537
6310	EBNA1	N4BP1	0.16648
6311	BZLF1	RAD18	0.07455
6312	EBNA6	CFTR	0.017964
6313	BGLF4	BLM	0.029448
6314	EBNA-LP	FSTL5	0.20358
6315	BGLF4	ORAI1	0.149
6316	BZLF1	KRT81	0.16448
6317	EBNA1	NUMBL	0.21105
6318	LMP2	NPC1L1	0.01712
6319	BMLF1	DOT1L	0.34607
6320	EBNA3	ZWILCH	0.30693
6321	BMLF1	ITK	0.15376
6322	BZLF1	RNF167	0.12874
6323	BZLF1	HLA-DRB1	0.042526
6324	BTRF1	ABRA	0.021066

6325	EBNA-LP	AIRE	0.12078	
6326	EBNA1	KIF21A	0.15112	
6327	BOLF1	S100P	0.11203	
6328	BGLF4	FAM109A	0.17206	
6329	BGLF4	PIK3CG	0.03323	
6330	gL	C11orf72		0.054975
6331	gL	BEND3	0.011976	
6332	BZLF1	AP1G1	0.15994	
6333	BZLF1	HECTD3	0.11462	
6334	BGLF4	ACTA1	0.0898	
6335	BGLF4	NLRP2	0.13208	
6336	EBNA6	CETN1	0.1673	
6337	BZLF1	TPD52L1	0.16312	
6338	BHRF1	COA7	0.027758	
6339	BZLF1	PPP3CB	0.12214	
6340	BGLF4	SUCLA2	0.070282	
6341	BMLF1	CASP7	0.19903	
6342	gL	LSM12	0.086109	
6343	EBNA-LP	FGF2	0.034679	
6344	EBNA1	MED15	0.10786	
6345	EBNA1	UBXN6	0.15579	
6346	LMP2	TESC	0.068235	
6347	BGLF4	VAMP2	0.13705	
6348	BZLF1	CNEP1R1	0.11931	
6349	BBRF2	EID2B	0.03006	
6350	BMLF1	ADRB1	0.088905	
6351	BLLF2	ZC3H12D	0.029917	
6352	EBNA3	CRKL	0.2915	
6353	BBRF2	MT1E	0.011195	
6354	BGLF4	TPPP3	0.066538	
6355	BGLF4	LCAT	0.1436	
6356	gL	CXorf67	0.17574	
6357	BMLF1	F7	0.092312	
6358	EBNA4	PRDM11	0.019044	
6359	EBNA1	SEC23A	0.083893	
6360	BLLF2	MAGEC2	0.02086	
6361	BMLF1	TTC14	0.053548	
6362	EBNA3	EIF2A	0.25403	
6363	BZLF1	TAP1	0.065487	
6364	BZLF1	DYRK1B	0.07899	
6365	BZLF1	NTNG1	0.18844	
6366	EBNA3	SLC24A1	0.33004	
6367	BGLF4	ANXA4	0.059596	
6368	BMLF1	PLK3	0.11897	
6369	BMLF1	NCBP1	0.15787	
6370	EBNA3	ZZEF1	0.15751	
6371	BZLF1	HAP1	0.14866	
6372	BZLF1	ARL11	0.19542	
6373	gM	PDCD6	0.12575	
6374	BGLF4	PICALM	0.061345	
6375	BMLF1	PHIP	0.26168	
6376	EBNA1	FTSJ3	0.2337	
6377	BMLF1	USP44	0.25901	
6378	EBNA-LP	TERF2	0.15179	
6379	BMLF1	RORA	0.13846	
6380	BZLF1	SH3RF2	0.15017	
6381	BBRF2	POLDIP2	0.038228	
6382	BMLF1	YEATS4	0.20993	
6383	EBNA-LP	COLQ	0.22823	
6384	BILF1	CLIP1	0.081181	
6385	BMLF1	ASCC3	0.22698	
6386	EBNA3	SMURF1	0.14207	
6387	EBNA3	BEND2	0.044498	
6388	BMLF1	RBMY1B	0.40188	
6389	EBNA3	PSMA2	0.10297	
6390	EBNA1	ENPP1	0.042435	
6391	BLLF2	TMED5	0.017106	
6392	BGLF4	PMAIP1	0.055379	

6393	BZLF1	NAA38	0.18142
6394	EBNA1	CPSF3	0.11984
6395	EBNA1	COMMD1	0.11697
6396	BMLF1	TMEM8B	0.30546
6397	BZLF1	NUMA1	0.058198
6398	BILF1	NPC1L1	0.062808
6399	BZLF1	PDZD11	0.20714
6400	EBNA3	PI4KA	0.34955
6401	EBNA-LP	SPINK7	0.38174
6402	BMLF1	FOS	0.052065
6403	EBNA3	MSANTD1	0.02438
6404	EBNA3	SLC32A1	0.041639
6405	BZLF1	TRPM6	0.14186
6406	EBNA6	CAMLG	0.10785
6407	EBNA3	EIF3G	0.39493
6408	BOLF1	UTRN	0.06276
6409	EBNA4	DYM	0.23025
6410	BXRF1	EDEM1	0.086633
6411	EBNA-LP	GORAB	0.35412
6412	BMLF1	U2SURP	0.42743
6413	BZLF2	ubl4ab	0.049115
6414	EBNA1	RIPK4	0.098338
6415	BPLF1	METTL1	0.097571
6416	BMLF1	DOK1	0.23132
6417	EBNA-LP	HPX	0.21606
6418	EBNA3	SPRY2	0.08021
6419	BZLF1	ADAM15	0.11502
6420	EBNA3	RCAN1	0.15052
6421	BLLF2	TAS2R3	0.064691
6422	BMLF1	AGO3	0.32109
6423	BZLF2	RRP7B	0.013711
6424	BZLF1	ANXA10	0.055404
6425	EBNA6	EFEMP1	0.15999
6426	EBNA3	RBMX	0.20348
6427	EBNA-LP	NCOR1	0.064484
6428	BMLF1	HNRNPM	0.29847
6429	EBNA-LP	PRKAR1A	0.18637
6430	BGLF4	CIB1	0.29484
6431	BLLF2	ZNF117	0.015884
6432	BMLF1	PATL2	0.36132
6433	BMLF1	THAP1	0.24413
6434	EBNA-LP	NDRG2	0.1959
6435	EBNA3	ALDH3A1	0.015119
6436	BILF1	MATN4	0.22376
6437	EBNA-LP	WAS	0.23793
6438	EBNA-LP	PARP4	0.27861
6439	EBNA6	FAM74A4	0.049
6440	EBNA3	PCDHGA4	0.17529
6441	EBNA1	SERPINF2	0.10972
6442	EBNA-LP	ODF2	0.15921
6443	BMLF1	TMPRSS11A	0.042436
6444	EBNA1	CIRBP	0.14486
6445	BZLF1	HNRNPL	0.15251
6446	BZLF1	DAB2	0.081939
6447	EBNA-LP	PUF60	0.26124
6448	BZLF1	ATPAF1	0.22217
6449	EBNA1	YAF2	0.098483
6450	EBNA-LP	ARHGEF1	0.16886
6451	EBNA-LP	NID1	0.15345
6452	EBNA3	MLF1	0.27984
6453	EBNA1	TNK1	0.13079
6454	BGRF1/BDRF1	HSPA5	0.066009
6455	BGLF4	TRIM44	0.19427
6456	BZLF1	TRIM41	0.12269
6457	EBNA-LP	HGF	0.1564
6458	EBNA-LP	TAP2	0.10302
6459	BZLF1	PCDHA10	0.044854
6460	BLLF2	MED26	0.03451

6461	BMLF1	MFF	0.22751
6462	BLLF2	SLCO2B1	0.02916
6463	BZLF1	SNRPC	0.18765
6464	gL	CANX	0.028391
6465	EBNA-LP	TMSB4X	0.25207
6466	EBNA4	TBC1D7	0.049337
6467	BTRF1	PAGE4	0.01506
6468	EBNA3	IL2RG	0.26308
6469	EBNA1	F2	0.11898
6470	EBNA1	MAPK8	0.048142
6471	EBNA-LP	RPGR	0.29549
6472	EBNA3	FBXO44	0.41955
6473	EBNA1	TMEM30A	0.19114
6474	EBNA2	ATP6V0A1	0.014003
6475	BMLF1	INCENP	0.21629
6476	BMLF1	CSNK1E	0.17066
6477	BMLF1	SLC8A1	0.010037
6478	EBNA1	YBX1	0.033836
6479	BMLF1	BRD7	0.16986
6480	BZLF1	PDK3	0.15577
6481	BMLF1	ADRB2	0.057957
6482	BMLF1	CYB5RL	0.019466
6483	EBNA6	PRSS3	0.18519
6484	EBNA1	GLRB	0.09155
6485	BZLF1	NEK9	0.018542
6486	EBNA6	PPP3CB	0.038274
6487	BZLF1	COIL	0.16235
6488	LMP2	SRI	0.046549
6489	EBNA-LP	PEG10	0.26864
6490	EBNA-LP	EGFL8	0.20614
6491	EBNA1	TBC1D8B	0.018813
6492	BZLF1	NDUFAF4	0.24141
6493	BMLF1	WIPF2	0.43033
6494	BGLF4	ATP6V1H	0.1391
6495	BMLF1	PFKL	0.21444
6496	EBNA1	RBL2	0.10787
6497	BZLF1	SCHIP1	0.19134
6498	BLLF2	PRAF2	0.090505
6499	BHRF1	S100B	0.068119
6500	BZLF1	PRUNE	0.16373
6501	BMLF1	HSPB9	0.4036
6502	BZLF1	PCDHGA4	0.048194
6503	BLLF2	TMEM132D	0.089763
6504	BMLF1	BBS2	0.19539
6505	EBNA3	CDON	0.17486
6506	EBNA1	SH3PXD2A	0.17308
6507	EBNA3	MUL1	0.069828
6508	BBRF2	LVP1a	0.033086
6509	EBNA-LP	ZYX	0.21656
6510	BMLF1	PAK3	0.14065
6511	BLLF1	PPP2R3B	0.032704
6512	BGLF4	KIF5B	0.10827
6513	EBNA3	CTSH	0.12569
6514	EBNA3	MME	0.094716
6515	BZLF1	HPRT1	0.062944
6516	BZLF2	RABEP1	0.063583
6517	EBNA3	SS18L2	0.031345
6518	EBNA4	DDOST	0.13677
6519	BILF1	C5orf48	0.011409
6520	EBNA3	TAF15	0.32821
6521	EBNA3	GAGE12F	0.061456
6522	BZLF1	CBX4	0.089305
6523	EBNA3	ECT2	0.061518
6524	EBNA1	HIRA	0.051614
6525	BMLF1	PRKD2	0.037979
6526	BBRF2	KYNU	0.01922
6527	EBNA-LP	COG2	0.25858
6528	BMLF1	CAMLG	0.3878

EBNA3	BIRC3	0.20282
BMLF1	IL23R	0.010275
EBNA-LP	DCHS1	0.1739
BZLF1	UBA52	0.020537
EBNA-LP	RAD9B	0.3686
BLLF1	RHPN1-AS1	0.041155
EBNA1	PRRC2A	0.1844
EBNA3	PCLO	0.13321
BZLF1	PLSCR4	0.36004
EBNA3	GLS	0.23327
BZLF1	C19orf68	0.23617
EBNA-LP	TLDC2	0.049479
BMLF1	CCS	0.22857
BGLF4	EDC3	0.21015
BMLF1	MARCH5	0.19115
EBNA1	ARHGEF1	0.06246
BZLF1	DDX3X	0.0184
BGLF4	INTS8	0.20196
BZLF1	CHCHD3	0.14606
BHRF1	ATXN7L3B	0.051103
BGLF4	HMG20B	0.086159
BMLF1	CTRC	0.050518
BGLF4	ADM	0.06213
BLLF2	ZNF419	0.017439
BRRF1	RNH1	0.19414
BMLF1	INIP	0.39591
BGLF4	BUB1B	0.026984
BBRF2	SLC39A7	0.011026
BNLF2b	PPP2R3C	0.039957
EBNA3	NCAPD2	0.27141
EBNA1	FSTL5	0.043666
BMLF1	SLC17A2	0.02815
EBNA3	POP7	0.32014
BZLF1	MYLK	0.071399
EBNA3	PPP1R32	0.41727
BLLF2	RTF1	0.022145
EBNA-LP	MKS1	0.26799
BARF1	PCDH17	0.20957
BHRF1	THBS2	0.13447
BGRF1/BDRF1	SYT1	0.074418
EBNA1	ARRB2	0.050252
EBNA-LP	APOA1BP	0.333
BZLF1	STAT1	0.053819
BOLF1	AC3.5	0.036006
BZLF2	RNH1	0.13884
BZLF1	MSR1	0.19718
BGLF4	GOLGA2	0.21724
BLLF2	ZNF75D	0.011529
EBNA-LP	MAPK14	0.026824
BGLF4	ELOVL7	0.14988
EBNA-LP	TPR	0.1792
EBNA-LP	RNF14	0.1502
BMLF1	DEFB129	0.080219
BMLF1	CARD18	0.044047
BXRF1	ATP2A1	0.0506
BMLF1	LOX	0.24493
BTRF1	PANK2	0.02293
BZLF1	C1QBP	0.13642
EBNA-LP	SULF2	0.042159
BGLF4	IFIT2	0.17489
LMP2	CDHR2	0.082866
EBNA-LP	HEATR5B	0.06835
BLLF2	BHLHB9	0.035913
EBNA3	PLCB3	0.11601
BLLF2	ZNF14	0.01787
BMLF1	ARPC3	0.31462
BLLF1	NUTM2A	0.010598
BZLF1	MAP3K4	0.077479

6597	BLLF2	LGALS9	0.021753
6598	BGLF4	PLXNB2	0.1502
6599	BLLF2	CEP57	0.040199
6600	BZLF1	C19orf57	0.21682
6601	BZLF1	C14orf166	0.10317
6602	EBNA-LP	MC5R	0.28673
6603	BLLF2	ZNF282	0.026912
6604	BBRF2	TICRR	0.035177
6605	gL	OTUD1	0.032371
6606	BZLF2	SRRM3	0.015484
6607	EBNA-LP	SGIP1	0.18023
6608	EBNA-LP	GTF2I	0.16171
6609	BLLF2	CLDN5	0.02677
6610	BMLF1	NEK3	0.24385
6611	BGLF4	PIK3R4	0.054939
6612	BLLF2	COPZ1	0.010854
6613	BGRF1/BDRF1	C11orf72	0.011796
6614	EBNA4	C1orf111	0.18622
6615	EBNA6	SZRD1	0.09214
6616	EBNA4	FAM45A	0.28647
6617	EBNA1	ALAS2	0.10582
6618	EBNA1	DERL1	0.11725
6619	BGLF4	MYL12A	0.068128
6620	EBNA1	YTHDC1	0.17872
6621	BZLF1	RFX5	0.058062
6622	BMLF1	SH3KBP1	0.34223
6623	EBNA1	S100A12	0.22473
6624	BGLF4	INTS12	0.16313
6625	EBNA1	COMMD5	0.18246
6626	BMLF1	UBE2D3	0.15674
6627	BZLF1	CBX2	0.11972
6628	EBNA3	ubl4ab	0.11268
6629	BMLF1	LCN2	0.028302
6630	BMLF1	HOXC13	0.19992
6631	EBNA-LP	IKBKAP	0.1359
6632	BZLF1	MRFAP1	0.19151
6633	gN	CT55	0.034171
6634	BLLF2	EFCAB4A	0.02149
6635	BGLF4	NHLRC1	0.11498
6636	BZLF1	SUCLA2	0.064752
6637	BMLF1	SEL1L	0.38499
6638	EBNA3	FOXK2	0.020725
6639	BGRF1/BDRF1	SLC11A2	0.026567
6640	EBNA1	DR1	0.084663
6641	BHRF1	MASP2	0.17273
6642	gL	CCDC152	0.01987
6643	BMLF1	NECAB1	0.16765
6644	BZLF1	TBC1D8B	0.029191
6645	BMLF1	PSMA1	0.12232
6646	BBRF2	PHYH	0.029456
6647	BMLF1	TOM1	0.33014
6648	BMLF1	ZNF408	0.25531
6649	BARF1	CELSR3	0.12757
6650	EBNA4	THBS2	0.18681
6651	EBNA2	EHD1	0.034018
6652	EBNA-LP	RPA2	0.14074
6653	BMLF1	BCCIP	0.36793
6654	BLLF2	OR1K1	0.083407
6655	EBNA1	UBE2C	0.077206
6656	EBNA-LP	CDH8	0.16534
6657	BZLF2	FAM231A	0.11568
6658	BBRF2	ZNF641	0.012348
6659	BGRF1/BDRF1	RABEP1	0.045647
6660	EBNA3	MAB21L3	0.017968
6661	EBNA6	SERTAD1	0.20743
6662	BZLF1	TAF9	0.067269
6663	EBNA3	MALT1	0.11776
6664	BZLF1	MOCS3	0.099331

6665	EBNA-LP	LCP2	0.33069
6666	BMLF1	CST5	0.47077
6667	BLLF2	H2AFY2	0.010406
6668	BGLF4	MAPK10	0.034417
6669	EBNA3	CACTIN	0.24722
6670	BHRF1	MAP7D2	0.036316
6671	BZLF1	MED29	0.15012
6672	EBNA3	GALNT3	0.081943
6673	gB	FAM188B2	0.031679
6674	EBNA6	TGM2	0.04118
6675	EBNA-LP	OXA1L	0.2455
6676	BMLF1	PPP1R2	0.38673
6677	EBNA1	BCKDHB	0.17372
6678	EBNA-LP	IL10RB	0.33715
6679	EBNA4	LRP1	0.055097
6680	BHRF1	MATN2	0.1365
6681	BILF1	KCNIP2	0.075083
6682	LMP1	CALM1	0.045068
6683	BZLF1	PAGR1	0.19426
6684	EBNA-LP	HDAC10	0.13704
6685	BPLF1	MISP	0.12726
6686	BGLF4	TOX4	0.19156
6687	BGLF4	DMPK	0.011965
6688	EBNA3	AFF1	0.21603
6689	BDLF1	CXorf67	0.028468
6690	BZLF1	TOPBP1	0.13128
6691	BMLF1	GML	0.061575
6692	BMLF1	HDAC6	0.023801
6693	EBNA-LP	GPS2	0.3297
6694	EBNA3	NT5C2	0.18306
6695	BMLF1	NYX	0.013743
6696	EBNA-LP	CEP152	0.18964
6697	BLLF2	THEG	0.052449
6698	EBNA3	SRRM5	0.029457
6699	EBNA3	SMURF2	0.14268
6700	BTRF1	ALKBH2	0.02575
6701	EBNA3	MUC17	0.27936
6702	BMLF1	TNPO1	0.26237
6703	EBNA3	ATP5A1	0.19918
6704	BMLF1	SIAH2	0.1993
6705	BZLF1	CDH3	0.070337
6706	BGLF4	TRIP13	0.097727
6707	BGLF4	MT1G	0.10888
6708	BZLF2	FAM92B	0.014079
6709	BALF2	SNX6	0.01946
6710	BMLF1	SKA2	0.24179
6711	EBNA-LP	SCNN1G	0.25892
6712	EBNA4	SPATA2L	0.15082
6713	BMLF1	ARHGAP17	0.31325
6714	BMLF1	ADRM1	0.29646
6715	EBNA-LP	PPM1D	0.1776
6716	EBNA3	CLSTN1	0.061259
6717	BZLF2	CCDC120	0.047244
6718	BGLF4	TLX3	0.080111
6719	EBNA3	WAS	0.24123
6720	gL	GOLGA6L9	0.11631
6721	BMLF1	SLC6A17	0.075466
6722	BZLF1	TRIM39	0.10402
6723	BZLF1	RBBP8	0.099505
6724	BZLF2	C2orf91	0.01148
6725	BZLF1	IRAK3	0.049552
6726	BHRF1	PITPNM3	0.12437
6727	EBNA4	PXMP4	0.17449
6728	BTRF1	TBC1D20	0.010554
6729	BLLF1	PRF1	0.031508
6730	BZLF1	LOX	0.12007
6731	BLLF2	ELOVL7	0.05608
6732	EBNA-LP	HTT	0.050985

6733	BMLF1	RHBDD1	0.22869
6734	BTRF1	TRAPPC4	0.010317
6735	BMLF1	PCDHGC3	0.21802
6736	BZLF1	PPP1CA	0.042568
6737	BZLF2	LINC00615	0.050971
6738	BPLF1	PARK7	0.026889
6739	BLLF1	LINC00588	0.035251
6740	BMLF1	NUP43	0.19672
6741	BMLF1	TOX4	0.38081
6742	gM	NKD1	0.074797
6743	BZLF1	NF2	0.058999
6744	BMLF1	FRG1	0.018642
6745	BBRF2	MED28	0.013678
6746	gL	CT45A5	0.047797
6747	BMLF1	SVEP1	0.22424
6748	EBNA1	POLE3	0.13068
6749	EBNA3	VWA5B2	0.064597
6750	EBNA6	PDCD6	0.19035
6751	EBNA6	SCIMP	0.23866
6752	EBNA3	OLFM1	0.3664
6753	EBNA1	PKD2L1	0.22803
6754	EBNA1	REPS2	0.44674
6755	EBNA4	CNOT1	0.088389
6756	EBNA6	MAGED4	0.019073
6757	BMLF1	WIPF3	0.09008
6758	BMLF1	ZWILCH	0.32653
6759	BMLF1	RILP	0.32777
6760	BZLF1	HLTF	0.055429
6761	BDLF1	S100A7	0.014305
6762	BMLF1	MAN1B1	0.12753
6763	EBNA1	HERC2	0.071435
6764	EBNA3	TRIM25	0.075988
6765	BLLF2	OR51S1	0.087398
6766	BGLF4	SCUBE1	0.025306
6767	EBNA-LP	ADI1	0.2293
6768	EBNA3	CRYAA	0.17066
6769	EBNA3	IL6	0.011723
6770	EBNA3	ASPSCR1	0.28696
6771	BBRF2	TCEAL5	0.039329
6772	EBNA4	CCDC69	0.13298
6773	BMLF1	TMPRSS2	0.035552
6774	EBNA-LP	AMY1A	0.24475
6775	EBNA4	CETN2	0.15635
6776	EBNA3	TPD52L2	0.41819
6777	BZLF1	STARD13	0.15998
6778	BZLF1	MAP3K3	0.082993
6779	BGLF4	RAB5A	0.11075
6780	BGLF4	UNC119B	0.20689
6781	BMLF1	PEX11B	0.33971
6782	BMLF1	STXBP3	0.21064
6783	BBRF2	MKS1	0.016762
6784	EBNA-LP	ATPAF2	0.39363
6785	BGLF4	MAP1LC3B	0.21603
6786	EBNA3	AKAP5	0.066787
6787	BMLF1	CDCP1	0.027736
6788	EBNA1	MAGEA4	0.20423
6789	EBNA3	ATP7B	0.09704
6790	BTRF1	TMEM120B	0.016326
6791	EBNA-LP	SNTB2	0.31065
6792	EBNA3	GPSM2	0.23296
6793	BMLF1	HSPA5	0.21204
6794	BTRF1	GPR179	0.049604
6795	EBNA3	ZNF496	0.127
6796	BZLF1	MRVI1	0.14752
6797	BMLF1	DNAJC7	0.36912
6798	EBNA1	CD9	0.11777
6799	BLLF2	ZNF300P1	0.011551
6800	EBNA6	KIAA2026	0.012467

6801	EBNA-LP	CBX6	0.28686
6802	BZLF1	FGFR1OP	0.089593
6803	EBNA3	DNAAF2	0.1645
6804	EBNA1	DFNB31	0.10487
6805	EBNA3	SPPL2B	0.20569
6806	BMLF1	RHOXF2	0.25902
6807	BGLF4	TOP1	0.07709
6808	BZLF1	DNAJC21	0.17361
6809	BZLF2	FAM78B	0.01931
6810	BZLF1	CD58	0.17798
6811	BVRF1	NCS1	0.1504
6812	EBNA-LP	ATP6V0A1	0.27527
6813	BZLF1	RNF135	0.14936
6814	BLLF2	ZNF8	0.018265
6815	BRRF1	GAS6	0.014698
6816	BZLF1	PVRL4	0.17871
6817	EBNA1	BIN3	0.15722
6818	BZLF1	SREBF2	0.023858
6819	BGLF4	MAK	0.024809
6820	EBNA-LP	EGLN1	0.17277
6821	EBNA3	IMMP2L	0.022374
6822	BZLF1	SRC	0.022812
6823	EBNA-LP	C12orf61	0.10826
6824	BZLF1	PDS5A	0.17873
6825	BZLF1	GRIN3A	0.075269
6826	BMLF1	TNS4	0.38072
6827	EBNA1	CDH7	0.03561
6828	BRRF1	HSPA5	0.069991
6829	EBNA6	ANXA11	0.085495
6830	BBRF2	OR4F4	0.030153
6831	EBNA6	C9orf153	0.062143
6832	BMLF1	TRABD2B	0.23155
6833	EBNA3	HIST3H3	0.21778
6834	BGLF4	NCBP2	0.099316
6835	BLLF2	ZNF304	0.016158
6836	BMLF1	GDF2	0.17215
6837	BMLF1	SHQ1	0.35506
6838	EBNA3	YDL022C-A	0.020265
6839	EBNA1	VPS33A	0.12839
6840	EBNA2	GSN	0.052378
6841	BZLF1	EIF3J	0.23414
6842	BZLF1	USP7	0.070906
6843	gL	MYL6	0.075558
6844	EBNA1	TJP3	0.1762
6845	BARF1	SNX9	0.10201
6846	BMLF1	ANXA2P2	0.23671
6847	EBNA1	RMI1	0.17354
6848	BBRF2	LYZ	0.058734
6849	BZLF1	CAMSAP2	0.20887
6850	EBNA4	CCDC154	0.067531
6851	EBNA3	TIPRL	0.40743
6852	BGLF4	ARFGEF2	0.082624
6853	BGLF4	PROC	0.2977
6854	BMLF1	TUBA1B	0.14243
6855	EBNA6	CNGC19	0.21675
6856	BHRF1	FAM45B	0.12166
6857	BLLF1	ACTN2	0.039609
6858	BZLF1	EIF3K	0.18926
6859	BZLF1	FBXO7	0.16403
6860	EBNA-LP	CPSF6	0.31148
6861	BMLF1	LYZL2	0.066586
6862	BZLF2	FAM160B1	0.058594
6863	EBNA1	EPS15L1	0.010082
6864	BBRF2	CCAR2	0.076161
6865	BZLF1	DLAT	0.18099
6866	BBRF2	OR2H1	0.039625
6867	BILF1	CALB1	0.15409
6868	EBNA-LP	ZBTB21	0.23418

6869	EBNA-LP	GFI1B	0.12363
6870	BGLF4	SYNJ2	0.12407
6871	BMLF1	CDK10	0.24021
6872	EBNA1	NOS3	0.019212
6873	EBNA1	LRP4	0.22478
6874	EBNA-LP	FGFR1	0.024739
6875	BZLF1	DNAJB4	0.17095
6876	BLLF2	CEP290	0.018815
6877	BZLF1	PKD1	0.020315
6878	EBNA3	ZNF703	0.16715
6879	BGLF4	CUL4B	0.17131
6880	BZLF1	MED4	0.14149
6881	BGLF4	BRE	0.15099
6882	BZLF1	CD47	0.13647
6883	BLLF2	ZKSCAN8	0.01458
6884	EBNA4	FAM160B2	0.096076
6885	BHRF1	UBAP2	0.12603
6886	BMLF1	POP7	0.35397
6887	EBNA-LP	ITGAM	0.20164
6888	EBNA3	PRKAB2	0.11078
6889	BZLF2	MB21D2	0.027172
6890	EBNA-LP	NXF3	0.40085
6891	EBNA3	IL1A	0.14443
6892	BLLF2	EPC1	0.027089
6893	EBNA3	NUFIP1	0.2625
6894	EBNA-LP	FBXO46	0.43184
6895	EBNA-LP	RAET1G	0.35833
6896	BGLF4	KCNMB4	0.15545
6897	BMLF1	CELA3B	0.047979
6898	EBNA1	DDX39A	0.12448
6899	gL	SRI	0.021626
6900	BMLF1	CHCHD3	0.31064
6901	BMLF1	TELO2	0.35295
6902	EBNA-LP	BIN2	0.41868
6903	BGRF1/BDRF1	LTBP1	0.21036
6904	BMLF1	RAB5C	0.033773
6905	EBNA4	RILP	0.21167
6906	BZLF1	LSM10	0.15364
6907	EBNA-LP	DVL1	0.069985
6908	EBNA-LP	CTCFL	0.18918
6909	BMLF1	WWP2	0.14762
6910	BGLF4	HFE2	0.16138
6911	BZLF2	C3orf84	0.010353
6912	EBNA4	RPL7A	0.012212
6913	EBNA3	CD93	0.48056
6914	EBNA3	MYL4	0.15247
6915	BILF1	CCAR1	0.19534
6916	EBNA1	RABGAP1	0.13373
6917	BGLF4	FUNDC1	0.14433
6918	EBNA6	FAM199X	0.07039
6919	EBNA2	AP3S1	0.041963
6920	BGLF4	PGRMC1	0.17304
6921	EBNA3	PLA2G5	0.037359
6922	EBNA-LP	MAP3K4	0.17796
6923	EBNA1	SRR	0.029909
6924	EBNA3	VPS72	0.28835
6925	EBNA1	CUL5	0.086732
6926	BLLF1	EHD3	0.031118
6927	EBNA6	HEATR2	0.013733
6928	BMLF1	ZNF217	0.15282
6929	EBNA3	BCAS2	0.3384
6930	EBNA3	DSG4	0.13961
6931	BGLF4	ADORA2A	0.029898
6932	EBNA3	MAP4K2	0.14117
6933	BRRF1	SETD8	0.014782
6934	gM	PRSS2	0.10693
6935	EBNA-LP	CTU1	0.38808
6936	EBNA1	FIG4	0.077096

BMLF1	ILKAP	0.26768
EBNA-LP	CCNB1	0.068815
EBNA3	NR5A2	0.045305
BDLF1	ACTN1	0.015344
BGLF4	NDFIP2	0.11756
BMLF1	NDE1	0.14371
BTRF1	CACNA2D4	0.03128
EBNA-LP	LY96	0.21404
EBNA4	BCLAF1	0.10633
EBNA1	BRCC3	0.096277
EBNA6	ZC3HAV1L	0.16516
BZLF1	CLUAP1	0.20355
BLLF2	TOR1A	0.013788
BILF1	FNBP1L	0.025228
BMLF1	OXA1L	0.2652
BLLF2	NIPSNAP1	0.077728
EBNA-LP	TAF11	0.25137
BGRF1/BDRF1	PLSCR1	0.089822
gN	EFCAB4B	0.078956
EBNA1	CREBZF	0.046398
BZLF1	GLB1	0.084962
BGLF4	CASP7	0.092179
EBNA3	UBA1	0.24782
EBNA1	NAP1L4	0.13157
BZLF1	CD28	0.098876
BLLF2	OR4F17	0.077525
EBNA-LP	GTF2E1	0.22218
EBNA-LP	CD44	0.1507
gL	PLSCR1	0.10069
BMLF1	RYR1	0.059142
BMLF1	RBM45	0.010743
BARF1	CAPN1	0.088108
BOLF1	AP3S1	0.064286
BHRF1	CADPS	0.17264
EBNA3	SOS2	0.17646
EBNA3	GEM	0.23387
BMLF1	EFHC1	0.17038
BGLF4	ZFP36L1	0.046364
EBNA3	PCDHGB5	0.18006
BMLF1	TTYH3	0.11655
EBNA-LP	RPS6KA5	0.059008
EBNA1	C10orf62	0.22232
EBNA-LP	BTBD1	0.36814
BGLF4	MRPL20	0.15412
EBNA-LP	SNCAIP	0.28719
EBNA3	PPM1F	0.12468
EBNA1	IFI35	0.18689
BZLF1	CAPNS1	0.30579
EBNA3	CTTNBP2NL	0.42312
EBNA4	CYBA	0.067664
gN	EHD1	0.024367
EBNA1	KHDRBS1	0.13834
EBNA6	RAB11FIP4	0.22172
BMLF1	GUCA1B	0.21098
BLLF1	PP632	0.011376
EBNA3	KIAA1279	0.37133
BARF1	THBS1	0.020574
EBNA4	HFE	0.017336
EBNA1	ZFPM1	0.023921
BLLF2	NBPF14	0.14725
BZLF1	DLX4	0.057083
EBNA1	PIDD	0.17814
BGLF4	EIF4EBP1	0.11692
EBNA3	C11orf35	0.019796
BZLF1	BIN3	0.16737
BMLF1	KDM2B	0.17057
BMLF1	MRPL23	0.032261
BZLF1	MBD2	0.096395

BMLF1	HSP90B1	0.32046
EBNA4	C1orf145	0.20429
BZLF1	AMELX	0.12018
EBNA3	CD80	0.24551
EBNA-LP	MATN2	0.5286
EBNA-LP	CCNG1	0.26096
EBNA-LP	PPP2R3C	0.16219
EBNA4	FAM160A1	0.31181
EBNA6	CD93	0.1231
EBNA3	NCK1	0.15219
EBNA3	CBS	0.067708
EBNA3	GDF7	0.21619
BLLF2	KRTAP16-1	0.10635
BMLF1	KCTD5	0.36114
EBNA6	TIFAB	0.25556
BLLF2	INPP4B	0.024297
BMLF1	RPL5	0.21479
EBNA3	EXOC3L4	0.061267
gL	C17orf59	0.21579
BGLF4	HTRA2	0.096891
gL	PAGE5	0.10211
EBNA6	EIF4G2	0.054778
EBNA-LP	LRP2BP	0.41868
BMLF1	MMP19	0.079856
BLLF2	LYZL1	0.071427
BGLF4	PAM	0.090036
EBNA-LP	LDHAL6B	0.34552
EBNA-LP	WASF2	0.25583
BVRF1	CANX	0.074934
BMLF1	GK	0.30723
BLLF2	FOPNL	0.033012
BGLF4	ASS1	0.0198
BMLF1	DIABLO	0.29872
BMLF1	ATG9A	0.35039
EBNA2	CDH23	0.036584
EBNA-LP	FNBP1	0.36511
EBNA6	GAGE13	0.061919
EBNA3	LETM1	0.65846
BGLF4	YLPM1	0.18759
BMLF1	NFKB1	0.041175
EBNA-LP	MIF4GD	0.37968
BZLF1	WBP2	0.22361
BLLF2	ATG12	0.023656
BBRF2	TBC1D3	0.011181
BZLF1	ATP6V0D2	0.14096
EBNA-LP	ARAP1	0.048877
BZLF1	TAS2R16	0.1524
BLLF2	IFNA5	0.04109
EBNA3	PLA2G2C	0.14321
BRRF1	FAT1	0.11254
BZLF1	CUL9	0.13054
EBNA-LP	FBLN2	0.19403
BBRF2	FAM168B	0.018059
BZLF1	KRT7	0.094185
BZLF1	THOC5	0.16212
EBNA-LP	MTTP	0.012178
BZLF1	BSG	0.10256
BMLF1	CYFIP1	0.24103
EBNA1	RAB5B	0.1228
BPLF1	YJR030C	0.065353
BGLF4	AVEN	0.20956
EBNA-LP	TBXA2R	0.16847
BZLF1	FPR1	0.13103
EBNA6	EIF4E	0.034536
BOLF1	UNQ6126/PRO20091	0.015744
BMLF1	NLK	0.13012
BZLF1	PLEKHG6	0.06088
BGLF4	STIP1	0.16043

7073	EBNA1	CADM1	0.068286
7074	EBNA-LP	ZNRF1	0.29721
7075	EBNA6	HEATR5A	0.091221
7076	BZLF1	POLR2A	0.066431
7077	EBNA-LP	RBCK1	0.10091
7078	BZLF1	GCHFR	0.12128
7079	BGLF4	FRS2	0.094721
7080	EBNA1	CLK2	0.086145
7081	EBNA1	TAOK3	0.10594
7082	BBRF2	PLXDC2	0.014957
7083	EBNA-LP	CHEK2	0.065037
7084	EBNA4	TLX1NB	0.063341
7085	EBNA-LP	JMY	0.23717
7086	BZLF1	ELAVL2	0.24605
7087	BLLF2	PRUNE2	0.01458
7088	BMLF1	RIN3	0.39268
7089	BMLF1	NUP98	0.16428
7090	BMLF1	RTP4	0.39952
7091	BGLF4	NUP37	0.11759
7092	EBNA1	VPS51	0.16727
7093	BZLF1	ZC3H7B	0.13687
7094	BGLF4	TGFB3	0.032537
7095	EBNA1	DNMT3A	0.12771
7096	BZLF1	S100A11	0.35883
7097	LMP2	GSN	0.043449
7098	BGRF1/BDRF1	PPP2R3B	0.1005
7099	LMP2	LRCOL1	0.046847
7100	BMLF1	GPATCH8	0.35123
7101	EBNA-LP	PIK3R2	0.20247
7102	EBNA3	AGXT	0.22469
7103	EBNA3	MAVS	0.058484
7104	BMLF1	PEX14	0.18229
7105	BZLF1	LCMT1	0.2737
7106	EBNA-LP	XIAP	0.20801
7107	BLLF2	TAS2R50	0.039451
7108	EBNA4	C19orf47	0.10717
7109	BMLF1	CD248	0.2241
7110	BGLF4	EPHB4	0.069959
7111	BGLF4	HSP90AB1	0.082154
7112	BZLF1	BCS1L	0.13348
7113	EBNA-LP	DAPK1	0.21207
7114	BPLF1	OTUD6B	0.014131
7115	BGLF4	MRPL49	0.19035
7116	BGLF4	DNASE1L2	0.053915
7117	EBNA-LP	GABPB1	0.13741
7118	EBNA3	PLK1S1	0.25313
7119	BLLF2	LOXL1	0.030278
7120	BMLF1	CSTB	0.014383
7121	BZLF1	INS	0.026801
7122	EBNA-LP	TMBIM4	0.29743
7123	BMLF1	DAZ3	0.011338
7124	EBNA-LP	CAMKK2	0.062999
7125	BZLF1	CD151	0.17357
7126	BGLF4	GDI1	0.16589
7127	BGLF4	STX2	0.11434
7128	BMLF1	CSRNP2	0.20659
7129	EBNA-LP	WTIP	0.20094
7130	BMLF1	SP1	0.040903
7131	EBNA1	HSP90B1	0.22039
7132	EBNA1	KMT2B	0.062753
7133	BZLF1	FLCN	0.18548
7134	BZLF1	SFN	0.062502
7135	EBNA3	ERCC8	0.25375
7136	EBNA-LP	STON2	0.31746
7137	EBNA-LP	TNK1	0.26656
7138	EBNA1	KXD1	0.20793
7139	BGLF4	EIF3K	0.19445
7140	EBNA3	MGST1	0.14215

7141	BZLF1	TUBG1	0.092328
7142	EBNA-LP	STXBP2	0.22679
7143	EBNA4	GTF2A1	0.13119
7144	BGLF4	LCMT2	0.27592
7145	BMLF1	GOLGA8I	0.034933
7146	BMLF1	S1PR3	0.01083
7147	BHRF1	MIR7-3HG	0.023061
7148	BPLF1	PIH1D2	0.083672
7149	BMLF1	BNIPL	0.332
7150	EBNA4	PLAC8	0.029097
7151	BPLF1	GPR98	0.052727
7152	BVRF1	PAGE5	0.012958
7153	EBNA3	POP1	0.27369
7154	BMLF1	PBX1	0.073466
7155	EBNA-LP	PARD6G	0.31908
7156	gB	COMP	0.049395
7157	BMLF1	SLC1A6	0.024584
7158	BBRF2	ZNF717	0.010726
7159	BZLF1	RTN4R	0.16845
7160	BGRF1/BDRF1	TH	0.010287
7161	EBNA3	NCF4	0.25783
7162	EBNA-LP	DOT1L	0.33037
7163	EBNA4	PHYH	0.18926
7164	BMLF1	DMAP1	0.24776
7165	BMLF1	LCA5	0.33829
7166	EBNA4	MYO1E	0.056681
7167	EBNA3	ZNF224	0.23607
7168	BMLF1	FGF3	0.27097
7169	BMLF1	HSPA1A	0.15
7170	BZLF1	TNS3	0.20333
7171	BMLF1	CEBPG	0.12212
7172	EBNA-LP	ARIH1	0.27418
7173	BMLF1	NKAP	0.24937
7174	EBNA-LP	ELF1	0.1725
7175	EBNA1	ARHGAP21	0.14451
7176	BILF1	REPS2	0.22843
7177	BGLF4	SEPT2	0.070385
7178	EBNA1	TAX1BP3	0.14459
7179	BLLF2	SRL	0.052636
7180	BLLF2	RIF1	0.10896
7181	EBNA-LP	ZFYVE27	0.24162
7182	EBNA-LP	ZNF205	0.24903
7183	BMLF1	LRPPRC	0.21173
7184	EBNA-LP	CHMP1B	0.30895
7185	BGRF1/BDRF1	EIF4G2	0.0176
7186	BGLF4	BNIPL	0.15917
7187	BMLF1	IGFL4	0.030621
7188	EBNA3	DLG2	0.2289
7189	EBNA3	LTF	0.1925
7190	EBNA1	BTK	0.013044
7191	BLLF2	HIST1H1D	0.01681
7192	BZLF1	PPAP2B	0.041267
7193	EBNA-LP	PRCP	0.37266
7194	EBNA3	KRAS	0.12128
7195	EBNA3	IL1RAPL1	0.20476
7196	BLLF2	ZNF492	0.022475
7197	BGRF1/BDRF1	NELL1	0.11138
7198	BMLF1	MAP1LC3B	0.35906
7199	EBNA3	NKD1	0.51348
7200	BGLF4	CARTPT	0.11235
7201	BZLF1	LDHD	0.20262
7202	EBNA3	MGMT	0.1023
7203	EBNA3	SEMG1	0.25368
7204	BMLF1	GFI1	0.15711
7205	BZLF1	SYP	0.0629
7206	EBNA3	MARK2	0.13581
7207	BMLF1	VHL	0.16568
7208	EBNA2	TESC	0.077287

7209	EBNA3	STAP2	0.18178
7210	EBNA3	NBR1	0.35159
7211	EBNA-LP	PLCZ1	0.074204
7212	EBNA3	LRAT	0.015852
7213	EBNA1	NRXN1	0.12172
7214	BMLF1	XPA	0.25025
7215	BHRF1	ZBTB8OS	0.015793
7216	BGRF1/BDRF1	YMR013W-A	0.017571
7217	BALF2	PITPNM3	0.11097
7218	BZLF1	MAP3K12	0.031208
7219	BMLF1	TNFAIP8L3	0.015318
7220	EBNA3	STXBP3	0.20444
7221	BGLF4	DVL3	0.10032
7222	BGLF4	C3	0.093867
7223	EBNA3	EIF5A2	0.23183
7224	BGLF4	HSFX1	0.098524
7225	EBNA3	TLX1NB	0.023791
7226	BBRF2	MPV17L2	0.015907
7227	EBNA-LP	BTG2	0.1941
7228	BMLF1	UCN	0.20192
7229	BZLF1	SLC6A4	0.088156
7230	BZLF1	VMP1	0.13536
7231	EBNA1	CD5	0.14548
7232	EBNA3	RAPGEF4	0.12899
7233	BZLF1	F2RL1	0.027539
7234	EBNA1	RCC1	0.1007
7235	BMLF1	KEL	0.27094
7236	BARF1	WLS	0.041118
7237	EBNA4	PPP2R3B	0.11553
7238	BMLF1	ESR1	0.029161
7239	EBNA3	SP3	0.10986
7240	EBNA3	BIRC2	0.11244
7241	BMLF1	IL17RE	0.41722
7242	EBNA1	CROCC	0.069642
7243	BTRF1	RHCG	0.014646
7244	BLLF2	TNMD	0.067316
7245	BZLF1	KBTBD6	0.21685
7246	BGLF4	BBS12	0.18346
7247	BGLF4	ATP6V0A4	0.1037
7248	EBNA3	ANXA10	0.19977
7249	EBNA1	AGO2	0.075422
7250	BMLF1	CDT1	0.28678
7251	EBNA3	SNRNP200	0.23453
7252	BMLF1	PRMT5	0.21219
7253	BMLF1	MSH3	0.076912
7254	EBNA3	NOTCH1	0.019004
7255	BLLF1	C14orf178	0.01899
7256	BMLF1	IL10RB	0.36625
7257	BMLF1	JUN	0.017169
7258	EBNA3	PAIP2	0.37405
7259	gM	S100P	0.056612
7260	BLLF2	TBATA	0.13466
7261	EBNA4	IQGAP1	0.03867
7262	EBNA4	RPS19	0.13528
7263	EBNA3	AKT3	0.10985
7264	BMLF1	PRMT6	0.22198
7265	EBNA3	PDE3B	0.13702
7266	BMLF1	TXNRD2	0.37732
7267	BOLF1	NKAPL	0.063085
7268	BMLF1	TBC1D8	0.18076
7269	BILF1	STIM1	0.08456
7270	EBNA1	NLGN3	0.082325
7271	EBNA3	FHIT	0.19205
7272	BILF1	FASN	0.072675
7273	BMLF1	TBC1D24	0.34871
7274	BMLF1	GLI3	0.010731
7275	EBNA1	PSMA2	0.049289
7276	BMLF1	GGA1	0.31028

7277	BMLF1	LETMD1	0.35403
7278	BMLF1	CTAG1A	0.45783
7279	BMLF1	SFR1	0.41185
7280	EBNA-LP	SIK3	0.20716
7281	EBNA1	DLG3	0.083938
7282	LMP2	MATN4	0.1119
7283	BGLF4	CDKN1B	0.058052
7284	BMLF1	TMPRSS7	0.046359
7285	EBNA3	PGR	0.078609
7286	BZLF1	MUC17	0.14795
7287	BZLF1	INIP	0.20153
7288	BMLF1	TTN	0.11098
7289	BNLF2b	CACNA2D3	0.058677
7290	BGLF4	PRKAB2	0.050316
7291	EBNA3	PSMC5	0.099443
7292	BMLF1	SPSB4	0.43457
7293	EBNA3	LCMT2	0.49132
7294	BALF2	SIRT4	0.031499
7295	BZLF2	DPH6	0.010854
7296	EBNA-LP	GTF2A2	0.16768
7297	BMLF1	COL3A1	0.18472
7298	BMLF1	TLL1	0.096358
7299	EBNA4	C20orf196	0.032442
7300	EBNA3	ERBB2IP	0.20944
7301	BGLF4	GRIA1	0.082854
7302	BZLF2	RHPN1-AS1	0.059664
7303	EBNA3	RAP1B	0.18479
7304	BZLF1	SPRTN	0.14121
7305	BLLF2	KLC3	0.01132
7306	EBNA1	PPFIA4	0.19672
7307	EBNA-LP	AMOTL1	0.35364
7308	BBRF2	PSME2	0.030062
7309	EBNA-LP	YWHAQ	0.35726
7310	BMLF1	THBS1	0.063274
7311	EBNA-LP	POLR1A	0.22585
7312	BBRF2	SLCO2B1	0.017318
7313	EBNA-LP	EFCAB14	0.2099
7314	BHRF1	YAE1D1	0.065318
7315	EBNA3	TBC1D7	0.36693
7316	EBNA3	SCAMP1	0.32388
7317	BZLF1	CDR2	0.19146
7318	EBNA2	KIAA1841	0.026684
7319	BZLF1	PCDH9	0.045167
7320	EBNA3	ENTPD7	0.06194
7321	BMLF1	SPC24	0.29685
7322	BZLF1	BCR	0.082221
7323	EBNA3	PROM1	0.23457
7324	BGLF4	MGARP	0.12594
7325	BGLF4	TFDP1	0.0274
7326	BVRF1	HSP90B1	0.087643
7327	BGLF4	STATH	0.1858
7328	EBNA4	PI4K2A	0.049416
7329	EBNA1	IL7R	0.098598
7330	EBNA1	IFNA2	0.13089
7331	BBRF2	DTX2	0.011892
7332	BGLF4	ASF1A	0.13711
7333	EBNA1	SSX2IP	0.18708
7334	EBNA1	MLST8	0.15465
7335	EBNA3	PRKCD	0.041448
7336	BMLF1	APLP1	0.13289
7337	EBNA1	ATP7A	0.01584
7338	BGLF4	SNTB1	0.11497
7339	EBNA3	AMOT	0.21066
7340	EBNA-LP	MMP25	0.062435
7341	BZLF1	RABGGTB	0.21132
7342	LMP2	COMP	0.078722
7343	EBNA-LP	ATP6V1F	0.24259
7344	EBNA-LP	PRKCB	0.071403

7345	BGLF4	AGTR2	0.075435
7346	BZLF1	NOA1	0.11706
7347	gL	FAM83F	0.12039
7348	EBNA-LP	CCNH	0.050776
7349	BGLF4	INTS3	0.19331
7350	EBNA1	MBNL1	0.12709
7351	EBNA1	PPM1D	0.060027
7352	BHRF1	GPRIN3	0.054548
7353	EBNA1	KAT5	0.080953
7354	BMLF1	SKAP1	0.10489
7355	EBNA-LP	PSMC5	0.10393
7356	BLLF2	CLCA2	0.020154
7357	EBNA3	RMI1	0.38004
7358	EBNA-LP	NDC80	0.21671
7359	BZLF1	YTHDC1	0.1902
7360	EBNA3	ANXA11	0.3906
7361	EBNA3	E2F3	0.14174
7362	BHRF1	DYDC1	0.019285
7363	gM	DLL1	0.088679
7364	EBNA3	DAO	0.20739
7365	BZLF1	C12orf50	0.010062
7366	BZLF1	ATRAID	0.12393
7367	BMLF1	CD72	0.30882
7368	BLLF1	CETN1	0.1255
7369	EBNA1	RAC3	0.059222
7370	EBNA-LP	IFIT3	0.30057
7371	EBNA-LP	DUT	0.24216
7372	BMLF1	SLC31A2	0.011785
7373	BZLF2	MYL6B	0.12949
7374	EBNA4	SRRM5	0.07413
7375	EBNA3	RSRC2	0.11886
7376	BZLF1	HSPA5	0.14114
7377	EBNA3	NEFM	0.27427
7378	EBNA1	CNOT6	0.13552
7379	EBNA-LP	STX18	0.27157
7380	EBNA3	SAP30	0.26561
7381	BGLF4	GLB1	0.093279
7382	BZLF1	PFN2	0.16328
7383	BGLF4	CUL4A	0.15806
7384	BMLF1	PXMP4	0.40462
7385	EBNA3	C9orf40	0.014113
7386	BGLF4	ANGPTL4	0.11881
7387	BZLF1	TCEB1	0.11435
7388	BGLF4	PHB	0.088186
7389	EBNA1	SRRT	0.11979
7390	BZLF1	KCNIP3	0.018861
7391	EBNA3	DNMT3A	0.23123
7392	gB	CRELD2	0.064233
7393	BMLF1	VPS28	0.30121
7394	EBNA3	MMP12	0.016262
7395	BLLF1	PLSCR1	0.044135
7396	EBNA-LP	CBX7	0.27592
7397	BMLF1	VPS41	0.23972
7398	EBNA-LP	VPS33B	0.24297
7399	EBNA1	APOBEC1	0.081185
7400	BILF1	MAP1LC3B	0.047442
7401	LMP1	HSPA5	0.027169
7402	BOLF1	C10orf82	0.019194
7403	BMLF1	KPNA5	0.33034
7404	BZLF1	TERF1	0.059908
7405	BMLF1	NEK9	0.15458
7406	EBNA3	RUNX2	0.058192
7407	BXRF1	ACTN1	0.080853
7408	EBNA-LP	APP	0.028098
7409	BZLF1	SH3GLB2	0.16619
7410	BLLF2	ZNF688	0.013856
7411	BBRF2	PPP1R14B	0.015541
7412	BMLF1	TMPRSS15	0.016911

7413	EBNA-LP	CENPA	0.14126
7414	EBNA3	IGF2BP2	0.28767
7415	BGLF4	CACNA1I	0.16819
7416	EBNA1	SIRT2	0.02708
7417	BMLF1	RNF6	0.20735
7418	BZLF2	NCKAP5L	0.075845
7419	BLLF2	OR4F6	0.092406
7420	EBNA1	SART1	0.1411
7421	BMLF1	DZIP3	0.35173
7422	EBNA3	USE1	0.27316
7423	BLLF2	PYDC2	0.011543
7424	BMLF1	APOB	0.043999
7425	BMLF1	EGFL7	0.19619
7426	EBNA-LP	NAPA	0.24256
7427	EBNA-LP	ATXN2	0.19646
7428	BTRF1	TNRC6C	0.013765
7429	BGLF4	SCUBE3	0.415
7430	BVRF1	C19orf66	0.029804
7431	EBNA-LP	MOB4	0.32369
7432	EBNA-LP	PRPF19	0.22742
7433	EBNA-LP	MEPCE	0.09501
7434	EBNA3	MYL10	0.20444
7435	BPLF1	GOLGA6L1	0.039054
7436	BPLF1	C21orf58	0.042578
7437	BLLF2	ELP4	0.083298
7438	EBNA1	PEX11B	0.1716
7439	BZLF1	SRSF3	0.19339
7440	EBNA1	ARF4	0.10989
7441	BZLF1	GABPA	0.028856
7442	BLLF2	DPY19L2P1	0.015516
7443	EBNA3	CTDSP1	0.2131
7444	EBNA4	WIPF2	0.1613
7445	BHRF1	C5orf52	0.013333
7446	BGLF4	FBXL5	0.16934
7447	BGLF4	ATG7	0.091707
7448	BGLF4	KRT20	0.1582
7449	BZLF1	DGKZ	0.11533
7450	BGLF4	GOLM1	0.21589
7451	EBNA-LP	CLUAP1	0.38697
7452	EBNA-LP	SGCE	0.14729
7453	BRRF1	PRSS3	0.22747
7454	EBNA-LP	WIBG	0.40828
7455	BZLF1	RABL6	0.15749
7456	BZLF1	IL13RA1	0.20858
7457	BGLF4	MAP3K4	0.047441
7458	EBNA3	ZFYVE28	0.29252
7459	EBNA3	FAT3	0.17465
7460	BBRF2	SMIM19	0.019167
7461	BBRF2	GOLGA8F	0.016357
7462	BGLF4	EREG	0.071454
7463	BMLF1	HPRT1	0.13294
7464	BZLF2	UCKL1	0.023046
7465	EBNA6	ODF3L2	0.012559
7466	EBNA3	LRCOL1	0.12936
7467	EBNA6	CCDC159	0.12621
7468	EBNA-LP	MAP4K1	0.21458
7469	BGLF4	NOP56	0.21142
7470	EBNA-LP	PSMA1	0.11805
7471	BMLF1	EVI5L	0.37122
7472	EBNA3	CDK10	0.19741
7473	EBNA-LP	IQGAP1	0.20293
7474	EBNA3	NELFB	0.24529
7475	BZLF1	ZC3H15	0.16478
7476	BZLF1	PSMD10	0.041095
7477	EBNA-LP	NOP56	0.36052
7478	EBNA1	RAB11FIP5	0.071064
7479	BMLF1	PAX6	0.010258
7480	BMLF1	ADAM22	0.27063

7481	EBNA-LP	ARID4B	0.27198
7482	EBNA3	C1R	0.22507
7483	BZLF1	TRABD2B	0.092517
7484	BGLF4	FLNA	0.045651
7485	EBNA3	FAM199X	0.040174
7486	BZLF1	KCNJ9	0.21183
7487	gL	CDHR2	0.053794
7488	BMLF1	WIPI2	0.28868
7489	BMLF1	PCDHA8	0.21127
7490	EBNA-LP	MAPK3	0.037146
7491	LMP2	CALB1	0.083893
7492	EBNA1	PCDHGB1	0.038413
7493	EBNA-LP	ERCC1	0.076168
7494	EBNA-LP	PPP1R12C	0.41868
7495	EBNA-LP	EXOC7	0.31178
7496	EBNA6	FAM86B1	0.011687
7497	BLLF2	TMEM136	0.058753
7498	BHRF1	NID2	0.16802
7499	EBNA3	MAD2L1BP	0.32679
7500	BZLF2	ZBTB8OS	0.015428
7501	BZLF1	PRPF40A	0.1523
7502	BGLF4	EPHA4	0.035512
7503	BMLF1	DND1	0.010206
7504	BMLF1	AKAP17A	0.33619
7505	BMLF1	HM13	0.24888
7506	EBNA6	LINC00205	0.12386
7507	EBNA-LP	IGHMBP2	0.077451
7508	BLLF2	GGT2	0.015156
7509	BZLF2	CCDC74A	0.048564
7510	BMLF1	IRF6	0.10403
7511	EBNA3	KAL1	0.2718
7512	EBNA1	SH3PXD2B	0.10867
7513	EBNA-LP	COPS6	0.31348
7514	EBNA1	SIRT3	0.11592
7515	BLLF2	PGLS	0.012825
7516	gL	FAM231D	0.19935
7517	BZLF1	TSHR	0.13954
7518	EBNA-LP	TCEA1	0.16283
7519	BGLF4	ORC5	0.10798
7520	BMLF1	GLT1D1	0.014682
7521	BMLF1	MSI2	0.038568
7522	BZLF1	ORC2	0.12942
7523	BLLF1	COMP	0.08386
7524	BLLF2	WFDC9	0.029436
7525	BLLF1	MESDC1	0.014417
7526	EBNA-LP	GSPT2	0.2498
7527	BLLF2	WTAP	0.070208
7528	EBNA1	DHX8	0.16168
7529	BZLF1	NOD1	0.039855
7530	BGLF4	SNTN	0.064239
7531	EBNA-LP	WDR66	0.2099
7532	EBNA-LP	ACTN1	0.29946
7533	BGLF4	PRICKLE1	0.11271
7534	BGLF4	DSN1	0.14071
7535	EBNA3	CEP63	0.31449
7536	gB	SYT1	0.022709
7537	EBNA1	PRPF19	0.11325
7538	EBNA1	CENPF	0.048319
7539	EBNA3	APBA2	0.31481
7540	EBNA-LP	ANK1	0.11569
7541	EBNA1	CABP1	0.21009
7542	EBNA1	SMARCC1	0.10087
7543	BZLF1	ERC1	0.087187
7544	EBNA-LP	GZMA	0.28907
7545	BZLF1	METTL17	0.22041
7546	BLLF2	PMP22	0.023076
7547	BGRF1/BDRF1	STAT2	0.022084
7548	BHRF1	RMND5B	0.013382

7549	BGRF1/BDRF1	RAB11FIP3	0.063943
7550	EBNA2	STIM1	0.053861
7551	EBNA-LP	PHC2	0.27345
7552	BGLF4	RECQL	0.092546
7553	BZLF2	FAM46A	0.047604
7554	EBNA3	TMCC2	0.41166
7555	EBNA1	MEAF6	0.1418
7556	BGLF4	ZAK	0.028713
7557	BGRF1/BDRF1	RHOT1	0.014006
7558	BGLF4	PAFAH1B3	0.13402
7559	BVRF1	EGF	0.052648
7560	EBNA1	ZNRF1	0.1559
7561	BGLF4	UCN	0.10532
7562	EBNA-LP	FOXN3	0.084441
7563	EBNA3	NLGN4X	0.16962
7564	EBNA2	MICU1	0.10627
7565	BLLF2	ZFP3	0.017247
7566	EBNA3	CALCOCO2	0.28331
7567	BMLF1	PANK1	0.060658
7568	EBNA3	RASSF1	0.16802
7569	BZLF1	DDX58	0.047913
7570	EBNA3	VPS33A	0.25402
7571	EBNA3	CDK14	0.12824
7572	BZLF1	MAD1L1	0.091997
7573	EBNA-LP	MYRIP	0.28628
7574	BLLF1	MLC1	0.012164
7575	BMLF1	SLC16A6	0.028609
7576	gB	TPD52	0.07903
7577	EBNA3	CABS1	0.17712
7578	BMLF1	PIWIL3	0.02771
7579	BMLF1	PIK3C3	0.19856
7580	BHRF1	SGD1	0.073914
7581	EBNA3	CPS1	0.073234
7582	BZLF1	PRMT1	0.21422
7583	BZLF1	TBC1D5	0.15185
7584	BLLF2	FAM3B	0.030634
7585	BBRF2	ELP4	0.03055
7586	BZLF1	JPH2	0.12856
7587	BMLF1	NUP85	0.1596
7588	EBNA1	DRD1	0.016153
7589	EBNA3	ARHGEF7	0.067004
7590	BGLF4	PEX16	0.13774
7591	EBNA6	CCDC158	0.010405
7592	BXRF1	RCN1	0.18396
7593	BGLF4	IGFBP7	0.19073
7594	BLLF1	POM121L2	0.025611
7595	BMLF1	SCARF2	0.39724
7596	BGRF1/BDRF1	SLIT2	0.039869
7597	BZLF1	SNX17	0.16958
7598	BGLF4	CELSR3	0.27638
7599	EBNA4	OCEL1	0.02671
7600	BMLF1	IL5RA	0.35995
7601	LMP1	SPARC	0.038934
7602	BMLF1	NKD2	0.43755
7603	gL	NKD2	0.10108
7604	EBNA3	SMYD2	0.23178
7605	EBNA1	LATS2	0.053325
7606	EBNA3	APC	0.02235
7607	BZLF1	INVS	0.14488
7608	BMLF1	STT3B	0.022056
7609	EBNA1	ORC1	0.080522
7610	BGRF1/BDRF1	CCAR1	0.18945
7611	BLLF1	UHRF1BP1L	0.036726
7612	gL	CADPS	0.1077
7613	EBNA-LP	ubl4ab	0.11042
7614	BPLF1	PRR23A	0.051151
7615	EBNA1	CCAR1	0.311
7616	BGLF4	HSF1	0.067654

7617	BBRF2	RPL23	0.016088
7618	BOLF1	RPH3A	0.077477
7619	BGRF1/BDRF1	CRELD2	0.205
7620	BZLF1	GFI1	0.070341
7621	BMLF1	SOST	0.24693
7622	BMLF1	CHD7	0.10189
7623	BZLF1	ADAMTSL2	0.1415
7624	BMLF1	SMARCB1	0.1555
7625	BMLF1	SPG7	0.17879
7626	BXRF1	PLCE1	0.012319
7627	BMLF1	KDM1A	0.051573
7628	EBNA-LP	EEF1A1	0.093306
7629	EBNA3	UHRF1	0.10858
7630	EBNA-LP	IER3	0.26561
7631	BZLF1	ALOX5	0.09464
7632	EBNA-LP	ARID1B	0.2082
7633	EBNA-LP	PPP2R2B	0.21966
7634	EBNA-LP	SLC34A1	0.30469
7635	BLLF2	ZSCAN30	0.018145
7636	BGLF4	MED7	0.13938
7637	EBNA3	MLLT1	0.2838
7638	BMLF1	TNNI2	0.34495
7639	EBNA4	SLC4A4	0.064223
7640	EBNA3	CTNNA3	0.21781
7641	BHRF1	MATN3	0.17965
7642	EBNA6	C18orf25	0.11453
7643	EBNA3	MYH9	0.088641
7644	BZLF1	CD53	0.19631
7645	EBNA3	PAFAH1B1	0.036051
7646	BHRF1	SYT11	0.18384
7647	BZLF1	PGRMC1	0.16358
7648	BMLF1	FBXO17	0.43665
7649	EBNA-LP	GNL3L	0.26431
7650	EBNA3	SLC14A1	0.11817
7651	EBNA-LP	RAPGEF4	0.12711
7652	EBNA-LP	DDX20	0.16665
7653	BLLF2	ZNF260	0.024171
7654	EBNA1	NPM3	0.16831
7655	BILF1	CLIC4	0.067678
7656	EBNA3	ISOC2	0.29512
7657	BLLF2	ZNF491	0.017246
7658	EBNA-LP	KAT2A	0.15987
7659	EBNA-LP	RACGAP1	0.079944
7660	EBNA3	CEP164	0.2917
7661	EBNA3	PCDHB6	0.15424
7662	BGLF4	SERPINF2	0.11421
7663	EBNA1	AURKAIP1	0.13739
7664	BILF1	CD93	0.13892
7665	EBNA3	PPP2R3C	0.16201
7666	EBNA-LP	SCIN	0.12568
7667	BTRF1	GOLGA6D	0.012976
7668	EBNA3	PRCP	0.37859
7669	EBNA-LP	SHOX	0.17143
7670	EBNA3	PILRB	0.37481
7671	BGLF4	BRCA2	0.04375
7672	BZLF1	ATF4	0.029845
7673	BZLF1	NPHP3	0.10238
7674	EBNA3	CCDC150	0.051937
7675	EBNA-LP	RBPMS	0.29419
7676	BHRF1	C6orf222	0.037291
7677	BMLF1	CLN3	0.065519
7678	EBNA3	MT-ND1	0.30076
7679	EBNA-LP	NR2C1	0.12838
7680	EBNA1	RWDD1	0.22232
7681	BZLF1	MRPS16	0.16273
7682	BLLF2	SLC4A9	0.033657
7683	EBNA3	LRP5	0.13023
7684	BMLF1	CPSF2	0.33681

7685	BGLF4	IK	0.19622
7686	BLLF2	LZTS1	0.036739
7687	EBNA3	MLKL	0.23252
7688	EBNA-LP	VPS26A	0.27328
7689	BGRF1/BDRF1	PKD2L1	0.10936
7690	BHRF1	CIB1	0.10404
7691	EBNA1	PDE4A	0.071665
7692	EBNA-LP	MPDZ	0.27429
7693	BZLF1	AXIN1	0.019059
7694	EBNA3	SPAG5	0.1967
7695	EBNA-LP	TLK2	0.21543
7696	BHRF1	AP1M1	0.015483
7697	EBNA1	ANK2	0.030089
7698	BALF2	CDHR2	0.1178
7699	BMLF1	ERBB2IP	0.22696
7700	BVRF1	SRI	0.080533
7701	EBNA4	FAM43B	0.022703
7702	BGLF4	EMD	0.11716
7703	BZLF2	PNMAL2	0.049902
7704	BZLF2	CLIC4	0.05141
7705	BGLF4	TRAF7	0.14168
7706	EBNA1	MCC	0.14317
7707	EBNA1	AKTIP	0.12987
7708	BGLF4	KRT7	0.10669
7709	gL	GOLGA6L10	0.11631
7710	EBNA-LP	MED11	0.2617
7711	EBNA4	C9orf40	0.042715
7712	BLLF2	ZSCAN22	0.010646
7713	EBNA6	LINC00083	0.013394
7714	EBNA1	CGN	0.18838
7715	EBNA3	KIAA0087	0.095589
7716	EBNA3	PDPK1	0.044667
7717	EBNA3	NCKAP5L	0.12454
7718	BMLF1	THAP11	0.28431
7719	BLLF2	HNRNPF	0.011144
7720	BMLF1	SH3PXD2B	0.23404
7721	EBNA4	ACTN3	0.20548
7722	BGLF4	SYBU	0.2163
7723	EBNA3	SMNDC1	0.38093
7724	BGLF4	SET	0.076107
7725	BGLF4	SGOL2	0.13167
7726	EBNA4	ULK2	0.01213
7727	gL	COMMD9	0.071434
7728	BGLF4	HSPD1	0.030959
7729	EBNA1	SFI1	0.16914
7730	BMLF1	WNT3A	0.033746
7731	BMLF1	BAP1	0.20229
7732	BGLF4	SAV1	0.14152
7733	BGLF4	UNC13D	0.14772
7734	BMLF1	INVS	0.28024
7735	EBNA-LP	DYSF	0.079386
7736	BVRF1	FAM83F	0.051007
7737	BMLF1	DSCAM	0.28861
7738	BZLF2	HUS1B	0.047269
7739	BTRF1	ETNK2	0.021707
7740	BOLF1	TLDC2	0.040062
7741	EBNA6	GID4	0.075861
7742	EBNA1	UBB	0.027046
7743	BZLF1	PVRL1	0.11549
7744	BGLF4	PTPN9	0.07893
7745	BZLF1	XPO4	0.18424
7746	BILF1	S100A13	0.16766
7747	EBNA6	C19orf48	0.034578
7748	gL	MAGEB17	0.019389
7749	EBNA-LP	TFDP1	0.073983
7750	EBNA3	GRB2	0.049158
7751	BMLF1	FAM109B	0.32426
7752	BZLF1	MAGEC2	0.1819

7753	EBNA1	CDH24	0.044226
7754	BMLF1	SNX4	0.33502
7755	BLLF1	AC3.5	0.018661
7756	EBNA-LP	GLRA1	0.11075
7757	BMLF1	NR5A2	0.07685
7758	BZLF1	FHL2	0.033304
7759	EBNA3	CTNNA2	0.15876
7760	BGLF4	ELMO2	0.078528
7761	BMLF1	FBXL14	0.43266
7762	gL	TCP10L2	0.086654
7763	BMLF1	DHX9	0.1895
7764	gB	ubl4ab	0.034648
7765	BHRF1	PDCD6	0.16198
7766	EBNA3	XK	0.40885
7767	EBNA1	KPNB1	0.09357
7768	BGLF4	PEX6	0.14443
7769	EBNA3	ARL6	0.15254
7770	EBNA1	SYT11	0.39152
7771	BARF1	GCH1	0.05243
7772	BNLF2b	KCNK13	0.014569
7773	EBNA3	AP2B1	0.17503
7774	BGLF4	SUFU	0.060452
7775	EBNA-LP	LAMTOR2	0.26551
7776	BGLF4	MORF4L1	0.16202
7777	BMLF1	PSMD2	0.18563
7778	EBNA6	ACTN3	0.16964
7779	EBNA3	SMARCC2	0.20747
7780	EBNA3	RAC3	0.15473
7781	EBNA1	TIFA	0.19142
7782	gL	YML009W-B	0.075338
7783	EBNA1	RBM25	0.14051
7784	gN	ACTN1	0.053129
7785	gL	ERICH1-AS1	0.13147
7786	EBNA-LP	PRKG1	0.15002
7787	EBNA1	SF3A3	0.11543
7788	BPLF1	CANX	0.065489
7789	BZLF1	ASXL1	0.10211
7790	BZLF1	ARNT	0.024408
7791	BMLF1	OXTR	0.015702
7792	EBNA-LP	HEYL	0.080481
7793	EBNA3	OCIAD1	0.39767
7794	BZLF1	DACT1	0.089442
7795	EBNA3	C11orf72	0.048437
7796	BXRF1	RET	0.067414
7797	EBNA-LP	NOC4L	0.32693
7798	EBNA4	PEX19	0.12789
7799	BLLF2	ZFP64	0.014046
7800	LMP1	MICU1	0.0611
7801	EBNA3	FANCI	0.34739
7802	EBNA-LP	TJP2	0.30244
7803	EBNA1	WNK1	0.10505
7804	EBNA1	CLDN7	0.066608
7805	BZLF2	FAM231B	0.051947
7806	EBNA6	FAM195A	0.27243
7807	EBNA-LP	ORAI3	0.40592
7808	BLLF2	MT1X	0.065501
7809	BMLF1	CCDC159	0.015438
7810	EBNA3	PLXNC1	0.26512
7811	EBNA1	IFIT1	0.17879
7812	BPLF1	LINC00472	0.094936
7813	BLLF2	NUDT1	0.01632
7814	EBNA3	KMT2C	0.27851
7815	BMLF1	SYNPO	0.28215
7816	BGLF4	UBE2F	0.11833
7817	BGLF4	PRSS3	0.34865
7818	BRRF1	FBN1	0.20972
7819	BLLF2	SLC16A11	0.067898
7820	EBNA3	NELL2	0.55342

7821	EBNA-LP	CD8A	0.16028
7822	BGRF1/BDRF1	TNNC1	0.18072
7823	BZLF1	ELOVL1	0.13847
7824	BOLF1	WIPF1	0.029065
7825	EBNA6	PRDM11	0.015152
7826	BMLF1	FAM131A	0.033611
7827	BGLF4	TCTEX1D4	0.24432
7828	BGLF4	RAB33A	0.14831
7829	BMLF1	GRSF1	0.040784
7830	EBNA3	PTPN12	0.14835
7831	BZLF1	ASPH	0.10952
7832	BRRF1	RET	0.082547
7833	BMLF1	LTBP3	0.19347
7834	EBNA3	STRN3	0.16278
7835	BZLF1	MTUS2	0.20676
7836	EBNA3	RNF103	0.24973
7837	EBNA1	JAM3	0.10652
7838	BGRF1/BDRF1	ITSN1	0.042334
7839	BGLF4	CTNNA1	0.057898
7840	EBNA1	CBX7	0.14399
7841	EBNA-LP	FOXH1	0.023661
7842	BGLF4	KIAA1377	0.24432
7843	EBNA1	FBXW11	0.10674
7844	EBNA-LP	RBX1	0.14498
7845	EBNA6	L1TD1	0.030862
7846	gN	EFEMP1	0.12328
7847	BMLF1	RARG	0.065141
7848	BLLF2	CITED4	0.01098
7849	BGLF4	MC3R	0.14268
7850	BMLF1	OOEP	0.063456
7851	gL	CCDC120	0.10976
7852	BZLF1	ATP2A1	0.13576
7853	EBNA1	TAF3	0.089585
7854	EBNA1	ZFAND6	0.11529
7855	BGLF4	SDPR	0.149
7856	EBNA-LP	EDA2R	0.25963
7857	EBNA1	SNRPE	0.12503
7858	BGLF4	ANXA8L2	0.048161
7859	BZLF2	CCDC96	0.010201
7860	EBNA-LP	ADAM15	0.20996
7861	EBNA3	GORAB	0.35864
7862	EBNA1	PCDHB7	0.037277
7863	BALF2	FAM231D	0.036182
7864	BZLF1	ALKBH3	0.15503
7865	BZLF1	CIITA	0.09932
7866	BMLF1	ATXN7	0.25403
7867	EBNA1	ARFIP2	0.14827
7868	BARF1	CALM1	0.092592
7869	BZLF1	COL7A1	0.16446
7870	BMLF1	TJAP1	0.39309
7871	EBNA3	SSTR3	0.21574
7872	EBNA1	ARID4B	0.12775
7873	EBNA3	GGA1	0.30698
7874	EBNA6	FAM166A	0.19657
7875	BLLF2	SMARCD3	0.010234
7876	EBNA4	GCSAML	0.20615
7877	EBNA-LP	TCF3	0.029997
7878	EBNA3	SOCS7	0.30292
7879	gL	PAGE2B	0.20296
7880	BMLF1	IPO8	0.32172
7881	BLLF1	C9orf169	0.010065
7882	EBNA3	FUNDC1	0.27172
7883	EBNA3	STAT4	0.058429
7884	BZLF1	VCL	0.080088
7885	BOLF1	ANXA2R	0.058524
7886	BLLF2	LGALS4	0.020781
7887	gL	TNNC1	0.071335
7888	BGLF4	COMMD6	0.18968

EBNA-LP	MAN1A2	0.07124
EBNA3	MMP8	0.065219
BPLF1	AMER2	0.10264
BPLF1	LURAP1L	0.12954
BMLF1	ADAM17	0.066028
BGLF4	STXBP3	0.11232
BGLF4	ING2	0.08882
EBNA-LP	HMCN2	0.22578
EBNA1	TRADD	0.051859
BMLF1	DPH5	0.054554
BZLF1	EIF6	0.23898
BZLF1	KAT7	0.076345
EBNA4	FSCN1	0.093997
EBNA3	NOS1	0.15375
BMLF1	SETD8	0.28347
EBNA4	YTHDF1	0.13804
BZLF1	IL12A	0.065344
BMLF1	EPB41L3	0.24611
BMLF1	AMOTL2	0.41268
EBNA3	TTC8	0.3075
BXRF1	EPS15	0.063465
BMLF1	HIST2H3A	0.29272
gL	S100B	0.017735
BGLF4	HRC	0.057633
EBNA3	SEPN1	0.3514
BZLF1	HGS	0.091813
BMLF1	RGS16	0.40797
BILF1	CCDC176	0.15491
BPLF1	UPF3B	0.10852
EBNA-LP	NKX3-1	0.021914
BHRF1	PHYHIP	0.020129
BLLF2	DIS3	0.010221
BMLF1	MYO9B	0.22603
BMLF1	ADARB1	0.058885
EBNA2	CAPNS1	0.02037
gL	ZNF728	0.01494
BLLF1	MYL6	0.11538
EBNA3	ERG	0.1197
BGLF4	ARL6	0.076454
EBNA4	C22orf42	0.046455
EBNA3	SAMD14	0.013326
EBNA3	RALBP1	0.29249
EBNA1	PAG1	0.17009
EBNA3	RAP2A	0.20507
EBNA1	LPL	0.034348
BMLF1	CD28	0.18829
BZLF1	CXADR	0.040974
BARF1	MATN3	0.22743
BBRF2	SCAMP5	0.011256
EBNA-LP	SMC1A	0.12882
EBNA1	S100B	0.22364
EBNA3	PCDHGB2	0.18006
EBNA1	PCDHB16	0.035257
BZLF1	BICD1	0.13647
BMLF1	NKAPL	0.091624
BARF1	MELK	0.061018
BGLF4	WDYHV1	0.16549
BGLF4	WASF1	0.13498
BGLF4	MATN4	0.43782
BZLF1	HOXC13	0.045757
BMLF1	RNF146	0.25101
BZLF1	MMP15	0.23762
BMLF1	HMG20B	0.20484
EBNA1	LIN7A	0.12726
BZLF1	MEGF6	0.45263
EBNA-LP	SMG5	0.28717
BZLF1	CDK10	0.077473
BMLF1	RWDD1	0.47224

7957	BGLF4	UBE4A	0.19408
7958	BGLF4	NMRK2	0.17567
7959	BLLF1	PHYHIP	0.010129
7960	EBNA1	APBB1	0.029427
7961	EBNA3	ATP1B1	0.2492
7962	BMLF1	SLC15A1	0.037225
7963	EBNA-LP	TLL1	0.04908
7964	BGLF4	SLPI	0.17682
7965	EBNA6	C16orf95	0.051613
7966	EBNA1	SLC22A6	0.13942
7967	BARF1	N6AMT1	0.046287
7968	BMLF1	NEFL	0.26394
7969	gL	FAM43B	0.010754
7970	EBNA3	MMP27	0.060454
7971	BZLF1	RASSF9	0.18259
7972	LMP1	MATN3	0.096643
7973	BLLF2	ZNF772	0.012673
7974	BGLF4	PBRM1	0.17405
7975	EBNA-LP	TCF4	0.11461
7976	BGLF4	PIP5K1B	0.15596
7977	EBNA-LP	NUP160	0.23686
7978	EBNA3	TRABD2B	0.20012
7979	BZLF1	SEC24B	0.10355
7980	EBNA1	OGDHL	0.19925
7981	BZLF1	CDC6	0.058374
7982	BLLF2	ZNF264	0.013144
7983	EBNA3	FOXL2	0.036723
7984	BMLF1	EDEM3	0.15282
7985	EBNA1	S100A16	0.021529
7986	BGLF4	RUVBL1	0.06902
7987	EBNA4	NOP56	0.074864
7988	BZLF1	KCNA4	0.1487
7989	EBNA-LP	ANTXR1	0.25956
7990	BZLF1	TF	0.12891
7991	EBNA-LP	CDHR3	0.17939
7992	EBNA3	DDX1	0.14183
7993	EBNA4	SDHAF2	0.12789
7994	BBRF2	RPL18	0.022099
7995	BGLF4	TRAPPC10	0.20357
7996	BGLF4	EIF5A	0.08279
7997	EBNA1	STK38	0.021826
7998	BXRF1	F2	0.042142
7999	BLLF2	TCRA	0.023207
8000	BGLF4	MCU	0.15743
8001	BLLF2	ZNF302	0.010489
8002	BZLF2	SCUBE3	0.16358
8003	EBNA3	CASP8	0.065164
8004	BMLF1	PIFO	0.26455
8005	BTRF1	UBXN11	0.021977
8006	BVRF1	TCAP	0.033792
8007	EBNA6	GCH1	0.071247
8008	gL	PSMG3	0.012806
8009	EBNA6	LINC00303	0.049441
8010	EBNA3	LZTFL1	0.39169
8011	BHRF1	CC2D2B	0.041283
8012	BZLF1	CD164	0.13493
8013	EBNA3	SNX4	0.33884
8014	BGLF4	GTF2F2	0.087885
8015	EBNA1	DLL1	0.22865
8016	BZLF1	CDC42EP1	0.15527
8017	gL	TPD52	0.081821
8018	BMLF1	DEPTOR	0.39265
8019	EBNA6	S100P	0.11774
8020	BGLF4	PNMA1	0.17632
8021	EBNA4	EAF2	0.12599
8022	BLLF2	OLFML1	0.015701
8023	BGLF4	SNAPIN	0.13769
8024	EBNA3	HERC5	0.21117

8025	EBNA3	PRDX6	0.33766
8026	EBNA-LP	PSMB5	0.10279
8027	BPLF1	ATP5B	0.059095
8028	EBNA-LP	IRF7	0.046255
8029	BZLF2	FAM217B	0.013122
8030	EBNA-LP	ARHGDIA	0.2411
8031	BLLF2	SPINK13	0.014778
8032	BMLF1	CFI	0.038022
8033	BTRF1	CCRN4L	0.019
8034	EBNA1	SNRNP70	0.16668
8035	BMLF1	RBM28	0.012762
8036	EBNA1	VCAN	0.025183
8037	BGLF4	TMEM11	0.18701
8038	BZLF1	AKAP5	0.029796
8039	BZLF1	XRCC6	0.012856
8040	BLLF2	ZNF510	0.016481
8041	BILF1	PRMT8	0.020832
8042	EBNA3	MDFIC	0.1724
8043	BHRF1	S100A11	0.12557
8044	BMLF1	LDHAL6B	0.37
8045	gN	S100A12	0.058578
8046	BZLF2	C16orf86	0.02376
8047	LMP1	IL6ST	0.042827
8048	EBNA3	DDX11	0.038395
8049	EBNA1	RHBDD1	0.12316
8050	BZLF1	PFKFB1	0.090706
8051	EBNA-LP	EDIL3	0.17947
8052	BMLF1	SYT1	0.21777
8053	EBNA1	GZMB	0.14183
8054	BMLF1	MYB	0.14435
8055	BMLF1	C19orf12	0.042181
8056	BMLF1	EVL	0.28463
8057	BMLF1	LIN7A	0.25381
8058	BMLF1	ALG1L2	0.015725
8059	EBNA-LP	IFIH1	0.14845
8060	gL	KIAA0087	0.11264
8061	BZLF1	TMED9	0.14564
8062	BGLF4	NR0B2	0.075676
8063	BGLF4	EIF3M	0.2556
8064	EBNA1	HSPA2	0.1418
8065	BGLF4	MED15	0.14295
8066	BVRF1	MATN3	0.18422
8067	EBNA-LP	AK6	0.4139
8068	BZLF1	GCSAM	0.19061
8069	BVRF1	MESDC1	0.014693
8070	EBNA3	TGFBR1	0.016696
8071	EBNA3	LDB3	0.314
8072	BTRF1	TIGD1	0.016836
8073	BGLF4	LSM5	0.17718
8074	EBNA3	WNK3	0.2017
8075	BARF1	MLC1	0.02674
8076	BZLF2	C12orf71	0.017029
8077	EBNA4	EIF4A3	0.01755
8078	BZLF1	HNF1A	0.017649
8079	BLLF2	TSPAN33	0.017055
8080	EBNA4	METTL1	0.24048
8081	EBNA3	FXYD4	0.12137
8082	BPLF1	MELK	0.032247
8083	EBNA3	BTRC	0.13652
8084	BLLF2	NGDN	0.037069
8085	BMLF1	RPLP0	0.24177
8086	BBRF2	CPSF2	0.013975
8087	BLLF2	OR2T7	0.087045
8088	BLLF2	NRSN2	0.017149
8089	EBNA1	HRAS	0.018837
8090	EBNA6	CCDC57	0.019305
8091	EBNA-LP	PLA2G2E	0.075881
8092	EBNA1	PLAU	0.1238

8093	BZLF1	CABS1	0.048272
8094	BZLF1	ALDOC	0.091317
8095	BMLF1	KIAA0101	0.34773
8096	EBNA4	COA7	0.067984
8097	BMLF1	RINT1	0.33101
8098	EBNA6	UPF3A	0.14576
8099	LMP1	CALU	0.067248
8100	EBNA-LP	SF3A1	0.35741
8101	BZLF1	GAD2	0.099628
8102	BZLF1	EFCAB10	0.053924
8103	EBNA3	TNFRSF13B	0.34419
8104	BLLF2	TBC1D5	0.013329
8105	EBNA3	NOL3	0.30144
8106	LMP1	S100A4	0.090105
8107	BGLF4	SNAP47	0.20873
8108	BNLF2b	SLCO4A1	0.012484
8109	BGLF4	SIRT6	0.14186
8110	EBNA4	NAF1	0.24169
8111	BMLF1	UNC5B	0.32593
8112	BMLF1	STMN3	0.020455
8113	EBNA-LP	PLK1	0.027047
8114	EBNA1	YTHDF3	0.22232
8115	EBNA3	VPS54	0.35529
8116	BMLF1	PCDHGB6	0.22489
8117	EBNA-LP	ADAMTS5	0.18431
8118	EBNA4	C19orf57	0.19787
8119	BGLF4	NCOA1	0.084176
8120	EBNA1	C19orf68	0.22232
8121	BMLF1	LRRC26	0.32997
8122	EBNA6	CAGE1	0.030292
8123	BPLF1	SLC34A1	0.097098
8124	BXRF1	FAM231A	0.11527
8125	EBNA6	C21orf58	0.10041
8126	EBNA3	CUL9	0.28133
8127	BBRF2	CCDC22	0.071798
8128	EBNA-LP	WASH1	0.28516
8129	BGLF4	CXADR	0.04424
8130	EBNA3	GAGE3	0.031848
8131	BILF1	MICU1	0.15496
8132	BMLF1	FASTK	0.32409
8133	EBNA-LP	LPAR1	0.10982
8134	EBNA1	PDIA2	0.10917
8135	EBNA2	CALM1	0.061662
8136	BZLF1	TMEM30B	0.21539
8137	BGRF1/BDRF1	CCDC176	0.042326
8138	BZLF2	TESC	0.094458
8139	BZLF2	THEGL	0.02331
8140	EBNA3	DLGAP3	0.29009
8141	BGLF4	SDC2	0.10781
8142	BLLF1	CALB1	0.090246
8143	EBNA3	TXNIP	0.16163
8144	gM	ANXA2	0.055442
8145	BMLF1	FABP5	0.34715
8146	EBNA1	SNTB2	0.16692
8147	BMLF1	ADAM23	0.26354
8148	EBNA3	DUSP1	0.064349
8149	EBNA1	TNFSF10	0.075653
8150	BHRF1	LURAP1L	0.14649
8151	EBNA-LP	CACNB4	0.14181
8152	BMLF1	PRSS8	0.35613
8153	EBNA3	PUSL1	0.054505
8154	BLLF2	NIPSNAP3A	0.062206
8155	EBNA-LP	UXT	0.18418
8156	BVRF1	LETM1	0.1575
8157	BVRF1	IL6ST	0.076165
8158	EBNA1	KRT1	0.10589
8159	EBNA-LP	PLD3	0.1899
8160	EBNA1	SEPT14	0.16635

8161	BLLF2	SPACA5	0.060149
8162	gL	GCSAML	0.10904
8163	EBNA3	COMP	0.48524
8164	BVRF1	RYR2	0.029042
8165	EBNA1	IL13	0.091585
8166	gL	PNMA6A	0.014492
8167	BOLF1	SIRT4	0.061129
8168	BZLF1	HIPK1	0.051364
8169	BMLF1	ACVRL1	0.028758
8170	EBNA4	LCHN	0.10115
8171	EBNA1	DIAPH1	0.052617
8172	EBNA6	CCDC114	0.028233
8173	EBNA1	BCL2L2	0.16853
8174	EBNA-LP	MARCH11	0.019548
8175	EBNA-LP	APH1B	0.3089
8176	BGLF4	TRPC5	0.14267
8177	BZLF1	SRSF7	0.16132
8178	EBNA4	SCIMP	0.34829
8179	EBNA-LP	HMG20B	0.16544
8180	BMLF1	CCDC50	0.45783
8181	BGLF4	MAP3K5	0.015202
8182	BZLF2	C11orf86	0.021331
8183	BHRF1	RGAG1	0.033185
8184	BLLF2	NMNAT1	0.011194
8185	BMLF1	STATH	0.36258
8186	gL	CASC3	0.16282
8187	EBNA1	NUDT21	0.17634
8188	BMLF1	WNK3	0.21999
8189	EBNA1	RACGAP1	0.035101
8190	BLLF2	MGAT2	0.020892
8191	BMLF1	TAPBP	0.16275
8192	EBNA1	TMEM30B	0.20278
8193	BLLF2	GPRIN1	0.014202
8194	BGLF4	WSB1	0.21963
8195	BZLF1	SIGIRR	0.16896
8196	BLLF2	ZNF248	0.017597
8197	BZLF1	PPP2R5A	0.090877
8198	BZLF1	SRSF2	0.18303
8199	BGLF4	SLC18A1	0.011374
8200	BGLF4	NPHS2	0.18675
8201	EBNA-LP	NF1	0.047029
8202	EBNA4	GEMIN7	0.16873
8203	EBNA3	NIPSNAP3B	0.020668
8204	EBNA3	LSM3	0.30255
8205	BZLF1	CTSB	0.043871
8206	BLLF1	YJEFN3	0.015839
8207	BBRF2	RIF1	0.024798
8208	EBNA3	CTCF	0.073247
8209	EBNA-LP	PIDD	0.34221
8210	BMLF1	C11orf54	0.36557
8211	EBNA-LP	LRRK2	0.045601
8212	EBNA-LP	MYLPF	0.1738
8213	EBNA3	ACSS1	0.14981
8214	BALF2	LRP1	0.02664
8215	EBNA-LP	KRT1	0.23821
8216	LMP2	S100A1	0.047246
8217	BZLF1	MYO1E	0.155
8218	EBNA3	PARD6A	0.16783
8219	EBNA1	MYH9	0.050374
8220	BGLF4	NPLOC4	0.13263
8221	EBNA-LP	SEPSECS	0.25423
8222	EBNA6	SECISBP2L	0.17551
8223	EBNA-LP	LONP1	0.18507
8224	EBNA6	C9orf40	0.036462
8225	EBNA-LP	MEAF6	0.26172
8226	BGLF4	ORC2	0.13612
8227	BMLF1	FLNB	0.2578
8228	EBNA3	BTG2	0.19788

8229	EBNA1	MRE11A	0.070238
8230	BGLF4	RARS	0.052421
8231	BGLF4	LRP4	0.21669
8232	BGLF4	YOD1	0.1375
8233	BMLF1	HEY2	0.025791
8234	EBNA-LP	S100A5	0.14549
8235	EBNA3	TRIM21	0.18112
8236	gL	S100A1	0.01167
8237	EBNA-LP	IL15	0.15257
8238	BARF1	ANXA2	0.10249
8239	BZLF1	CDHR3	0.049103
8240	BXRF1	RCN3	0.18064
8241	EBNA3	CDYL2	0.32365
8242	BGLF4	GAPDHS	0.13171
8243	BMLF1	SLC1A1	0.027576
8244	BZLF2	SECISBP2L	0.06565
8245	BMLF1	TNFRSF14	0.2829
8246	EBNA-LP	GAD1	0.19581
8247	BZLF1	NCAPG	0.16627
8248	EBNA-LP	LCMT2	0.48307
8249	BGLF4	USP37	0.087573
8250	gL	SUMO4	0.011587
8251	BZLF1	NUMBL	0.22027
8252	BGLF4	POU2F1	0.077004
8253	EBNA3	FRAT2	0.12956
8254	BZLF1	SMG7	0.14383
8255	EBNA-LP	NMRK2	0.38573
8256	EBNA4	ATG9A	0.027876
8257	BZLF2	C9orf170	0.01791
8258	EBNA-LP	TLL2	0.053497
8259	BMLF1	EPS8	0.37716
8260	EBNA1	INPP5D	0.06366
8261	gL	C10orf91	0.17948
8262	EBNA-LP	ICAM3	0.28139
8263	BMLF1	ARSA	0.093781
8264	EBNA-LP	CSF2RB	0.28565
8265	BMLF1	MTSS1	0.19309
8266	EBNA-LP	NBN	0.08701
8267	EBNA-LP	KIF3A	0.1695
8268	EBNA3	KIAA1958	0.050283
8269	BMLF1	GSTO1	0.067007
8270	EBNA-LP	SLC1A3	0.0185
8271	BZLF1	CD80	0.13545
8272	BZLF1	MZF1	0.040265
8273	EBNA-LP	NPHP1	0.19095
8274	EBNA-LP	C11orf72	0.029667
8275	BLLF2	ZNF615	0.022901
8276	BGLF4	CDKN3	0.038977
8277	BGLF4	USP32	0.05154
8278	EBNA-LP	TRIM27	0.11686
8279	LMP2	MATN2	0.093406
8280	EBNA1	DMD	0.076186
8281	BLLF2	ZIK1	0.018108
8282	BZLF2	DLL1	0.11839
8283	EBNA3	PIK3R6	0.20731
8284	BALF2	MPTX1	0.01344
8285	BZLF1	AES	0.099076
8286	BILF1	LETM1	0.18064
8287	EBNA3	STK3	0.10536
8288	BZLF2	C5orf58	0.062236
8289	BMLF1	LRRFIP2	0.4198
8290	EBNA-LP	RPL27A	0.30443
8291	BOLF1	EHD1	0.052146
8292	BZLF1	DYX1C1	0.1721
8293	BMLF1	NDC80	0.23601
8294	EBNA1	PTK2B	0.011958
8295	EBNA3	ZHX1	0.12757
8296	EBNA3	PLCG1	0.12973

8297	EBNA3	ASCL1	0.033124
8298	BGLF4	CIR1	0.078877
8299	EBNA3	ELK3	0.11317
8300	EBNA1	PSMC4	0.064836
8301	EBNA-LP	TH	0.035481
8302	BZLF1	BAK1	0.030168
8303	BMLF1	ANG	0.071798
8304	BMLF1	RNF144B	0.27494
8305	EBNA-LP	TBC1D9B	0.14203
8306	BMLF1	SMAGP	0.11239
8307	EBNA6	SLC4A4	0.042084
8308	BHRF1	GOLGA6L9	0.10513
8309	BMLF1	ITGB5	0.2429
8310	BMLF1	IGF2	0.16863
8311	BTRF1	RHD	0.018325
8312	BGLF4	TCEB1	0.13413
8313	EBNA-LP	FAM47C	0.065732
8314	EBNA3	PLSCR2	0.18082
8315	EBNA1	NPHS1	0.11796
8316	BHRF1	TNFAIP8L3	0.02237
8317	EBNA3	CDH3	0.18601
8318	BHRF1	YFL013W-A	0.081886
8319	EBNA3	CCDC74B	0.052333
8320	BMLF1	RPA3	0.19619
8321	BMLF1	LPAR1	0.13014
8322	EBNA3	TRPC6	0.21843
8323	EBNA3	MTDH	0.15956
8324	BMLF1	RCAN1	0.18791
8325	BMLF1	CST4	0.034289
8326	BZLF2	EHD3	0.055815
8327	BZLF1	PCDHGA10	0.049103
8328	EBNA-LP	SHCBP1	0.21541
8329	EBNA4	PXMP2	0.12972
8330	BMLF1	NAP1L1	0.33654
8331	BMLF1	SLAIN2	0.40515
8332	BHRF1	BSDC1	0.01612
8333	BALF2	ZC3HAV1L	0.011743
8334	BGLF4	USP12	0.17033
8335	BMLF1	KLC2	0.31344
8336	BZLF1	ASGR1	0.17348
8337	EBNA6	POP4	0.1923
8338	EBNA-LP	GSK3A	0.069595
8339	EBNA3	LRP2	0.22729
8340	BZLF1	EXD3	0.23617
8341	BLLF2	ERVK6	0.01255
8342	EBNA6	ADAMTS13	0.012168
8343	BMLF1	CHRNA7	0.025146
8344	EBNA4	C12orf71	0.054113
8345	BGLF4	OCIAD1	0.22044
8346	BHRF1	PRDM11	0.010567
8347	gB	SDF4	0.050378
8348	BZLF1	MYO9A	0.11188
8349	BGLF4	CRYGD	0.19284
8350	BMLF1	NDFIP1	0.17712
8351	BLLF1	PNMAL2	0.028418
8352	BLLF1	FAM160A1	0.051905
8353	BZLF1	DCTN3	0.055527
8354	BMLF1	KIF24	0.35982
8355	BLLF2	TIGD7	0.012435
8356	EBNA-LP	PTPN13	0.12707
8357	EBNA3	ELP3	0.06536
8358	EBNA3	CCND3	0.10352
8359	EBNA-LP	KLKB1	0.29699
8360	BRRF1	SDF4	0.16075
8361	EBNA1	DCTN1	0.12318
8362	EBNA-LP	FASLG	0.10486
8363	EBNA-LP	CSNK2A1	0.13015
8364	gB	IL6ST	0.041779

EBNA1	AP2A1	0.091141
BZLF1	FAM21C	0.18175
BZLF1	IRF2	0.016516
EBNA-LP	WHAMM	0.094428
EBNA-LP	FCN1	0.052047
BZLF1	FLVCR1	0.13918
EBNA3	RPS6KB1	0.031672
BGLF4	HHEX	0.011517
BZLF2	C1orf168	0.041548
BGLF4	CLASP1	0.065823
BGLF4	MYH10	0.06274
BMLF1	KCNK13	0.012189
BBRF2	TAS2R39	0.025432
BGLF4	PRRG4	0.057144
BGLF4	MED26	0.1426
BZLF1	MAPK12	0.048853
BMLF1	PTPRT	0.14071
gB	REPS1	0.045423
BLLF1	C10orf82	0.023204
BZLF1	RAB5A	0.10938
EBNA-LP	CDCA5	0.25704
EBNA3	EFNA4	0.43365
BTRF1	POLR2L	0.013898
BLLF1	CAGE1	0.010865
EBNA-LP	EIF2B2	0.2541
EBNA1	RHOA	0.014258
EBNA4	HUS1B	0.16928
BZLF1	RPL13	0.18101
EBNA1	DOCK1	0.11713
BMLF1	RECQL	0.23103
BZLF1	PPP1R32	0.22359
BZLF2	PTGES3L	0.026064
BZLF1	DSG1	0.028357
EBNA6	MYO6	0.012674
EBNA3	DSC3	0.15013
BBRF2	NDRG3	0.017579
EBNA3	SCAI	0.27806
EBNA1	ATN1	0.12409
BZLF1	A1CF	0.14298
EBNA1	SMN1	0.18748
BPLF1	MUC1	0.099477
BMLF1	PDCD4	0.36688
BMLF1	HUS1	0.27353
BZLF1	MCIDAS	0.11577
BMLF1	POLD1	0.033414
EBNA3	APOE	0.020187
EBNA1	TRAPPC4	0.20458
BZLF2	GAGE10	0.050047
BGLF4	PDGFRA	0.015014
BZLF1	GAREML	0.011528
BXRF1	S100A6	0.11294
BMLF1	PDE6D	0.46478
BHRF1	LYRM9	0.12427
BGLF4	S100A13	0.26091
BLLF2	ARSB	0.015933
EBNA3	CDK4	0.067527
BHRF1	C22orf42	0.018462
BZLF1	SPTBN4	0.042964
BGRF1/BDRF1	REPS2	0.22152
BBRF2	DMRTB1	0.012772
EBNA3	EIF3F	0.35979
gB	REPS2	0.074038
EBNA6	BTBD16	0.036414
BBRF2	ZDHHC11B	0.012988
BBRF2	EXOC1	0.036809
BGLF4	MAP3K7CL	0.20895
EBNA3	USF1	0.047564
EBNA3	NMNAT1	0.29788

8433	BGLF4	PPM1F	0.062442
8434	BMLF1	KEAP1	0.27642
8435	BMLF1	SOCS7	0.32761
8436	BMLF1	ARFIP2	0.31465
8437	EBNA6	ATXN7L3B	0.098649
8438	BOLF1	CABP1	0.027394
8439	BGLF4	RFWD2	0.10814
8440	EBNA3	TTYH2	0.3444
8441	EBNA1	PMEL	0.13904
8442	BLLF2	TSPAN16	0.027282
8443	EBNA3	IGFL2	0.16873
8444	BGLF4	PTPN12	0.066248
8445	BHRF1	STIM1	0.061123
8446	BMLF1	FSTL3	0.1773
8447	EBNA3	GRIA1	0.14955
8448	BGLF4	SH3RF1	0.10947
8449	EBNA1	INADL	0.16293
8450	BGLF4	DDX23	0.10109
8451	BGLF4	RUNX3	0.039326
8452	BMLF1	EIF2S3	0.34831
8453	gM	S100B	0.04227
8454	EBNA3	CKS1B	0.31528
8455	BMLF1	EFNB3	0.037149
8456	BMLF1	NPM1	0.078053
8457	EBNA1	AMOT	0.12728
8458	BGLF4	PSMC6	0.064746
8459	EBNA3	SLC2A1	0.026538
8460	BLLF2	PPP4R2	0.11523
8461	BGLF4	PRRG1	0.060357
8462	EBNA4	MATN4	0.23007
8463	EBNA1	TRIP6	0.11509
8464	EBNA3	IPO13	0.33108
8465	EBNA3	NKX2-1	0.0372
8466	BZLF1	STAU1	0.17532
8467	BZLF2	S100A11	0.12568
8468	EBNA3	RQCD1	0.25051
8469	BGLF4	HPGDS	0.063844
8470	BMLF1	OTUB1	0.28124
8471	EBNA3	EPS15L1	0.1357
8472	BBRF2	NBPF14	0.039537
8473	EBNA-LP	HTN1	0.37089
8474	EBNA1	LIG3	0.051607
8475	EBNA-LP	CDC25C	0.043617
8476	EBNA3	VCP	0.11255
8477	EBNA3	HJURP	0.20511
8478	EBNA1	NUCB1	0.044991
8479	BZLF1	MED22	0.12535
8480	EBNA1	PSMD9	0.079172
8481	EBNA3	KIAA1109	0.34736
8482	EBNA3	BOC	0.26511
8483	BZLF1	TRAF3IP2	0.19174
8484	BZLF1	ORAI1	0.14249
8485	EBNA1	MTF1	0.010021
8486	EBNA3	PAK3	0.10846
8487	BGLF4	GP1BB	0.17421
8488	EBNA3	PLA2G10	0.034336
8489	EBNA-LP	DNMT1	0.19576
8490	LMP2	NECAB3	0.059033
8491	BPLF1	WHAMMP3	0.012874
8492	EBNA-LP	AQP2	0.038296
8493	EBNA4	FBXO25	0.26408
8494	BLLF2	MS4A4A	0.02686
8495	BRRF1	MATN4	0.23015
8496	EBNA1	STAT6	0.13603
8497	BMLF1	CDHR3	0.22489
8498	EBNA-LP	ACTR1B	0.30695
8499	EBNA-LP	ROPN1L	0.36794
8500	EBNA1	GPD2	0.025755

8501	BMLF1	RRM1	0.27615
8502	EBNA-LP	EIF4E3	0.037699
8503	EBNA3	SIAH2	0.17149
8504	BPLF1	FAM127C	0.037791
8505	BLLF2	NBPF6	0.03035
8506	BOLF1	CCDC47	0.14821
8507	BMLF1	RELN	0.014271
8508	EBNA1	CASQ2	0.042665
8509	EBNA1	PAFAH1B3	0.11421
8510	EBNA4	C3orf22	0.21522
8511	BZLF1	WTIP	0.10433
8512	BOLF1	NDEL1	0.017181
8513	EBNA-LP	ZEB1	0.083736
8514	gB	VLDLR	0.033357
8515	EBNA1	ATP2C2	0.10467
8516	EBNA1	SNCAIP	0.13976
8517	EBNA3	MAPK8	0.095785
8518	BLLF2	SPINK5	0.019421
8519	EBNA6	NUP160	0.066615
8520	BGLF4	RASSF1	0.085896
8521	BLLF2	ECU09	0.021684
8522	EBNA1	TAF15	0.14696
8523	BHRF1	LINS	0.038769
8524	BZLF1	CASP8	0.030817
8525	BZLF1	UBXN7	0.16933
8526	BZLF1	GAP43	0.15126
8527	EBNA6	PRAME	0.22592
8528	EBNA-LP	IGSF8	0.33535
8529	EBNA-LP	OCM	0.17789
8530	BGLF4	CALR	0.0449
8531	BZLF1	STRAP	0.10987
8532	EBNA1	HGF	0.077387
8533	BMLF1	NCF1	0.096627
8534	EBNA-LP	TCEB1	0.21118
8535	EBNA-LP	NCK1	0.15146
8536	BMLF1	CSN2	0.23182
8537	gN	KCNIP2	0.033051
8538	EBNA3	EIF2AK2	0.13634
8539	BMLF1	DNM1L	0.10505
8540	EBNA3	PTPRJ	0.061561
8541	EBNA-LP	CBS	0.067847
8542	BGLF4	ZNF622	0.12888
8543	BMLF1	SUV39H2	0.26261
8544	EBNA1	ADORA2A	0.027329
8545	BLLF2	TNNT1	0.067451
8546	EBNA1	NR1I2	0.032098
8547	BGLF4	ASXL1	0.12199
8548	LMP1	LRP2	0.030663
8549	BZLF2	ZC3HAV1L	0.045974
8550	EBNA3	FAT4	0.65766
8551	BZLF1	ADRBK1	0.027718
8552	EBNA-LP	TPD52L2	0.41184
8553	EBNA4	C11orf72	0.094262
8554	BZLF1	GUCA1B	0.065721
8555	EBNA3	ITGAM	0.20404
8556	BMLF1	ZWINT	0.25078
8557	BMLF1	SOX2	0.015982
8558	BMLF1	TLX3	0.19939
8559	BGRF1/BDRF1	EIF4E2	0.032489
8560	BXRF1	NKD2	0.18068
8561	EBNA6	C9orf62	0.032859
8562	EBNA4	ATP5B	0.088312
8563	BZLF1	PKD2	0.027671
8564	EBNA-LP	EFNA1	0.18365
8565	EBNA-LP	AUNIP	0.34817
8566	BZLF1	HIRA	0.059405
8567	BGLF4	CCNC	0.072887
8568	EBNA1	EIF3D	0.21661

8569	EBNA1	SPRR3	0.066455
8570	EBNA3	ADAM23	0.22801
8571	BZLF2	FAM92A1	0.010301
8572	BZLF1	CLPTM1	0.19033
8573	BLLF2	SDS	0.022697
8574	BLLF2	BCKDHA	0.012611
8575	BMLF1	BECN1	0.22389
8576	BGLF4	MMS19	0.1222
8577	BLLF2	GPR111	0.011988
8578	EBNA1	RPGRIP1	0.16698
8579	BZLF2	BEND4	0.015553
8580	EBNA1	EZH2	0.10261
8581	EBNA6	EIF4A1	0.013856
8582	EBNA1	APLP1	0.028103
8583	BZLF1	NKRF	0.16145
8584	BMLF1	WTIP	0.2241
8585	BLLF2	CHMP1A	0.018184
8586	EBNA-LP	SPC24	0.27154
8587	BMLF1	FBP1	0.14763
8588	BLLF1	EP400NL	0.027099
8589	EBNA1	CCBE1	0.030134
8590	BILF1	NKAPL	0.10575
8591	BZLF1	CREB1	0.011857
8592	BVRF1	CALB1	0.12266
8593	EBNA-LP	TRAF3IP3	0.40592
8594	EBNA3	HPS1	0.30642
8595	EBNA1	RCOR3	0.14253
8596	BGLF4	RAC3	0.076282
8597	BMLF1	PIAS1	0.17054
8598	BBRF2	MLF1	0.018483
8599	EBNA-LP	TMF1	0.24543
8600	BZLF1	SYTL1	0.16926
8601	BBRF2	LFNG	0.011304
8602	BZLF1	PCDH18	0.047714
8603	BZLF1	RNF111	0.076338
8604	EBNA3	GRB7	0.14894
8605	EBNA6	LINC00471	0.12649
8606	BILF1	UPF3B	0.031568
8607	BMLF1	PMEL	0.30508
8608	EBNA-LP	FAN1	0.25538
8609	BMLF1	ORC2	0.27067
8610	gN	ATP5B	0.054397
8611	BGLF4	PAICS	0.11009
8612	EBNA3	XRCC5	0.08183
8613	BGLF4	TSSK4	0.047081
8614	EBNA3	CIRBP	0.28104
8615	BGLF4	SRY	0.077169
8616	EBNA3	FCGR1A	0.2872
8617	BZLF1	NUP98	0.10189
8618	EBNA-LP	SUPT4H1	0.098597
8619	BMLF1	PPP1R8	0.25177
8620	BMLF1	HPCA	0.24729
8621	BGLF4	S100A7A	0.047037
8622	BZLF1	CAPSL	0.055771
8623	EBNA4	WHAMMP3	0.036762
8624	EBNA3	AP1S2	0.016395
8625	BZLF1	MET	0.032048
8626	EBNA-LP	BCKDHA	0.32467
8627	BGLF4	SNRPD2	0.11541
8628	BLLF2	PXMP2	0.043081
8629	BGLF4	EXOC7	0.17694
8630	EBNA-LP	KAT6B	0.14972
8631	BZLF1	SPRY2	0.031635
8632	BMLF1	RAP2A	0.2322
8633	BLLF2	PDE6G	0.039553
8634	BLLF2	ZNF7	0.015317
8635	BMLF1	RASSF1	0.19165
8636	BMLF1	CELSR3	0.44531

BMLF1	SLURP1	0.032274
EBNA1	NELFA	0.13179
EBNA2	S100A11	0.099965
BHRF1	LINC00471	0.080167
BMLF1	CREBBP	0.039142
EBNA3	PES1	0.22941
BMLF1	ZNF655	0.24331
EBNA-LP	ESRRA	0.10106
EBNA1	SEPT9	0.10629
BMLF1	CD2AP	0.30593
EBNA-LP	UBE2V1	0.14184
EBNA3	PTPN6	0.035599
BMLF1	COL6A2	0.27671
BLLF2	OR10G2	0.091156
EBNA6	NUCB2	0.11917
EBNA-LP	TRIB3	0.099203
BMLF1	C5AR2	0.32379
EBNA1	RNF111	0.065894
BMLF1	DYRK1B	0.21778
EBNA3	ALS2CR11	0.032402
BGLF4	CREM	0.074998
BZLF1	FAM188A	0.41104
BMLF1	FLG2	0.1291
BZLF1	IFIH1	0.070317
BGLF4	RNF167	0.13115
gL	KIAA0226L	0.01851
EBNA-LP	CD2BP2	0.32282
EBNA4	CCDC153	0.033785
EBNA-LP	MLC1	0.2416
LMP1	PRSS3	0.10425
BGLF4	NR1H2	0.034531
BGLF4	ZNF274	0.068707
BZLF1	ATP6V0C	0.12475
BGLF4	CLCN3	0.1002
EBNA1	NLRP1	0.08129
BLLF2	NOL6	0.054544
BMLF1	DDX17	0.24208
BZLF2	PIH1D2	0.058222
BGLF4	USP26	0.17221
BILF1	C2CD4D	0.025457
BZLF1	RBX1	0.077561
BMLF1	TGFBRAP1	0.32577
EBNA6	CCDC144A	0.015778
BLLF2	NANOGNB	0.014803
BOLF1	PCDH17	0.17142
BZLF1	CTNNBIP1	0.12253
BMLF1	NQO1	0.31415
EBNA1	APBA1	0.11086
BLLF2	TKTL2	0.016868
BMLF1	PSMG2	0.36797
BZLF2	AP3S1	0.053102
BMLF1	FOXO1	0.039297
BZLF1	MCM5	0.094323
BMLF1	KLHL12	0.39577
BZLF1	CDC34	0.077963
EBNA3	MYO6	0.11125
EBNA3	PLG	0.1428
BZLF1	HMCN2	0.0802
BARF1	AP1B1	0.050626
BZLF1	TRADD	0.059513
EBNA4	RAD1	0.11443
EBNA1	TSSK2	0.058773
BGLF4	LIN28A	0.11875
EBNA-LP	STAG1	0.26344
BZLF1	TWIST2	0.023761
BZLF1	MYL12A	0.058208
BLLF2	RNASE8	0.013274
BMLF1	EHMT1	0.28239

8705	BMLF1	FZD4	0.10568
8706	BGLF4	C1S	0.08139
8707	EBNA3	ERC2	0.31154
8708	EBNA-LP	SULT2B1	0.24464
8709	BLLF1	C5orf48	0.042307
8710	EBNA4	FAM231B	0.20956
8711	EBNA1	RBM5	0.13126
8712	BZLF1	RBM26	0.22126
8713	BZLF1	ULK1	0.04613
8714	EBNA-LP	POLR1B	0.20134
8715	EBNA3	PANK3	0.13127
8716	EBNA4	CCDC159	0.19348
8717	EBNA3	SLC25A10	0.20089
8718	BZLF1	SPON1	0.21435
8719	BZLF1	RAC3	0.06569
8720	EBNA1	VSNL1	0.044917
8721	BBRF2	APOL3	0.012387
8722	EBNA4	MYOZ2	0.29756
8723	BARF1	MYL6B	0.1787
8724	BGLF4	SYNE3	0.14661
8725	BMLF1	NCS1	0.43558
8726	BMLF1	ECSIT	0.3935
8727	EBNA3	RPA3	0.17856
8728	EBNA3	ARL6IP1	0.41513
8729	BPLF1	N6AMT1	0.013794
8730	BLLF2	ZNF10	0.018272
8731	BTRF1	MAN1A2	0.013857
8732	BNLF2b	TBC1D3F	0.016574
8733	BZLF1	PCDHGB1	0.049103
8734	gL	F10	0.020307
8735	EBNA3	TNKS2	0.18589
8736	BMLF1	RALB	0.25225
8737	BARF1	RYR2	0.040372
8738	BXRF1	NSUN5P1	0.038343
8739	BGLF4	NKX3-1	0.010153
8740	EBNA-LP	INTS12	0.30419
8741	BRRF1	SIRT4	0.090284
8742	EBNA-LP	CCDC137	0.06539
8743	BGLF4	SIKE1	0.18701
8744	BZLF2	ZK1128.3	0.082696
8745	BMLF1	ELK4	0.20571
8746	EBNA3	RBKS	0.027307
8747	BLLF2	GRHL3	0.015086
8748	EBNA3	COMMD6	0.34762
8749	BGLF4	CDC6	0.060719
8750	BGLF4	USH2A	0.12983
8751	BMLF1	PLAUR	0.19327
8752	EBNA-LP	SERPINF2	0.19126
8753	EBNA3	STUB1	0.14743
8754	EBNA3	ARL11	0.37979
8755	BLLF2	ZNF444	0.032272
8756	BTRF1	PLSCR4	0.030578
8757	EBNA1	BMX	0.039372
8758	EBNA-LP	EIF3H	0.38652
8759	EBNA1	NDE1	0.062662
8760	EBNA3	TENC1	0.21412
8761	BMLF1	CNOT4	0.22356
8762	EBNA1	ATG4B	0.16713
8763	BMLF1	PLCD1	0.037698
8764	BRRF1	MEGF8	0.06159
8765	BMLF1	TNFRSF21	0.36739
8766	EBNA3	FAM127A	0.043741
8767	BMLF1	STX11	0.3672
8768	BXRF1	NOTCH2	0.013912
8769	BZLF1	AXL	0.056064
8770	EBNA1	SRSF10	0.148
8771	BZLF1	DARS2	0.070205
8772	BGLF4	RBM4	0.081871

gL	LINC00083	0.012612
EBNA3	MAGEA6	0.40098
BGRF1/BDRF1	WDR96	0.013656
BGLF4	TPX2	0.060787
BMLF1	C16orf93	0.02413
BZLF1	OR2L13	0.16229
gL	CXorf21	0.065689
BZLF1	ZNF652	0.11682
BZLF1	IFT122	0.20565
BZLF2	ATXN7L3B	0.038353
BZLF2	EDEM1	0.070155
EBNA3	RHBDL2	0.012771
BDLF1	GRIN1	0.01443
BZLF1	SBDS	0.07682
EBNA3	CLSTN3	0.10397
BXRF1	MYL6	0.16608
EBNA6	CCDC42	0.022566
EBNA4	FBXW12	0.010778
BMLF1	NEK2	0.096086
BLLF2	ZNF584	0.012734
BGLF4	UHMK1	0.077683
EBNA1	GABARAP	0.13769
BZLF2	RNMT	0.017437
EBNA4	FAM211A	0.016488
BMLF1	TFAP4	0.17225
EBNA1	PPP4R1	0.16421
EBNA-LP	PRKAA1	0.027651
BLLF2	MIS18BP1	0.078967
BGLF4	FLT3	0.054376
BGLF4	FBXL8	0.24432
BMLF1	SLC6A19	0.024798
BPLF1	GRIN1	0.047759
BMLF1	GSTO2	0.067002
BMLF1	CALCOCO2	0.3269
BZLF1	ACAN	0.12189
BMLF1	PABPC4L	0.039667
BGLF4	ACE	0.024561
EBNA1	TAF9B	0.08731
EBNA-LP	PDZD11	0.37946
BPLF1	DLL1	0.11886
BGLF4	OLFM1	0.20525
BMLF1	ATXN1L	0.033738
BZLF2	PROC	0.1583
EBNA-LP	VPRBP	0.32568
EBNA3	MAP3K12	0.10559
EBNA-LP	EFCAB10	0.19711
EBNA1	CIITA	0.088507
BZLF1	UBE2I	0.02658
EBNA6	CCAR2	0.11069
LMP1	RNH1	0.065198
EBNA3	SLC16A3	0.053777
EBNA3	KIF2C	0.14262
EBNA-LP	RASSF2	0.35242
BBRF2	DEDD2	0.024874
BGRF1/BDRF1	GCH1	0.04949
EBNA3	EIF3I	0.40527
EBNA1	PDK3	0.14708
BGRF1/BDRF1	S100A6	0.13317
BZLF2	CCDC137	0.077538
EBNA-LP	HEY2	0.018732
BMLF1	INPP5B	0.25045
BMLF1	ALDH7A1	0.29175
EBNA-LP	N4BP2L1	0.027148
gM	DNER	0.055174
EBNA3	SIT1	0.30023
EBNA-LP	ATG4B	0.3212
EBNA-LP	RANBP3	0.29187
BGRF1/BDRF1	VIL1	0.090629

8841	EBNA-LP	BRIP1	0.014772
8842	EBNA3	LIMS1	0.27226
8843	BBRF2	TNRC6A	0.020273
8844	BGRF1/BDRF1	CELSR3	0.12007
8845	EBNA6	C12orf57	0.072528
8846	EBNA4	JMJD7	0.020682
8847	EBNA3	EYA2	0.098599
8848	EBNA3	TRIM28	0.049456
8849	EBNA3	STX2	0.20246
8850	EBNA3	SAMD13	0.01004
8851	BLLF1	C9orf153	0.011053
8852	EBNA-LP	SPRED1	0.1352
8853	EBNA4	ASPSCR1	0.1754
8854	EBNA4	AC3.5	0.20167
8855	EBNA3	CEP72	0.30341
8856	BZLF1	RIOK3	0.16446
8857	BMLF1	CEP76	0.35726
8858	BLLF1	CNRIP1	0.01176
8859	BMLF1	CSDC2	0.018846
8860	EBNA3	TBC1D5	0.303
8861	BGLF4	MADD	0.16299
8862	BGLF4	DAZL	0.14167
8863	BGLF4	CEACAM1	0.17177
8864	EBNA1	GIT1	0.10831
8865	BHRF1	C7orf57	0.032775
8866	BLLF2	XRCC5	0.011968
8867	BGLF4	IL8	0.10105
8868	BZLF1	CAMTA2	0.085252
8869	BZLF1	CENPT	0.11955
8870	BMLF1	GNS	0.16643
8871	BZLF1	FSBP	0.17318
8872	BHRF1	KHNYN	0.042391
8873	EBNA6	GOLGA6L3	0.20339
8874	EBNA3	UBQLN1	0.26623
8875	BGLF4	HOOK1	0.12795
8876	BGLF4	TMEM8B	0.15342
8877	gM	CHP2	0.077095
8878	gL	GPRIN3	0.042846
8879	EBNA-LP	PARK7	0.21833
8880	EBNA3	COPS3	0.33761
8881	BGLF4	PRPF6	0.16757
8882	BGLF4	CALML4	0.068073
8883	EBNA6	RCN3	0.18563
8884	EBNA3	HID1	0.29723
8885	BMLF1	PCDHA6	0.2049
8886	EBNA2	CLIP1	0.051054
8887	BMLF1	SLC13A1	0.044838
8888	gL	SLC34A1	0.13847
8889	BZLF1	COMMD3	0.23617
8890	BGLF4	DOCK9	0.10758
8891	BMLF1	CDC42EP2	0.2743
8892	BGLF4	SHOX	0.08999
8893	BHRF1	FBXL18	0.028301
8894	BDLF1	SCUBE3	0.027638
8895	BGLF4	TCF12	0.057412
8896	EBNA3	XPO6	0.31253
8897	BBRF2	BEX2	0.091856
8898	BZLF1	ITGA6	0.1484
8899	EBNA1	RIPK1	0.018815
8900	EBNA3	NACA	0.27413
8901	EBNA6	LINC00476	0.039263
8902	BVRF1	PLSCR4	0.13082
8903	BGLF4	PTGIS	0.044883
8904	BMLF1	SNG1	0.032578
8905	EBNA1	TNFRSF10B	0.13436
8906	BZLF1	GORAB	0.18683
8907	BGLF4	SMARCC2	0.12945
8908	EBNA-LP	FRS2	0.16607

8909	EBNA3	RBBP8	0.19791
8910	EBNA-LP	BSG	0.19649
8911	EBNA3	TSN	0.3844
8912	BXRF1	WIPF1	0.021731
8913	BZLF1	MOB1A	0.19521
8914	EBNA6	MSANTD2	0.033297
8915	BMLF1	TNFRSF10A	0.28389
8916	BMLF1	ANXA13	0.23379
8917	BMLF1	PDIA3	0.15803
8918	gM	BNIP2	0.035986
8919	BGLF4	KMT2B	0.091767
8920	BHRF1	SNURFL	0.065186
8921	EBNA-LP	MPZL1	0.23992
8922	EBNA3	BRAP	0.2543
8923	BRRF1	TESC	0.13056
8924	EBNA6	PAGE2B	0.2897
8925	BZLF1	PDCL3	0.20258
8926	EBNA-LP	HNF4A	0.019961
8927	EBNA3	CLPX	0.23047
8928	BMLF1	SLC16A14	0.031311
8929	gN	MMP20	0.01061
8930	BMLF1	SEC61B	0.22816
8931	EBNA6	PIR	0.13413
8932	BLLF2	MIA2	0.020213
8933	BMLF1	PPP4R1	0.37636
8934	EBNA3	SERPINA1	0.28682
8935	BZLF1	DSC3	0.046158
8936	BMLF1	ECT2	0.068553
8937	EBNA-LP	TDRD7	0.30661
8938	EBNA1	TRIM29	0.044029
8939	BZLF1	C17orf59	0.012352
8940	BMLF1	ORC6	0.31502
8941	BGLF4	SULT2A1	0.16578
8942	EBNA3	STEAP1	0.050686
8943	BMLF1	RPL11	0.21479
8944	BGLF4	CAND2	0.18755
8945	EBNA3	ERICH1	0.089325
8946	EBNA6	JAG2	0.11166
8947	EBNA-LP	CREBBP	0.027599
8948	BMLF1	PEX26	0.30691
8949	BZLF1	IARS	0.049478
8950	BGLF4	ID1	0.0535
8951	BZLF1	ELMO2	0.059716
8952	BMLF1	NSRP1	0.35322
8953	EBNA-LP	SRI	0.38646
8954	BOLF1	EHD3	0.067199
8955	BPLF1	TPD52	0.14832
8956	BZLF1	TRIM16	0.10156
8957	BLLF2	P4HA3	0.030584
8958	BPLF1	C6orf118	0.054151
8959	BGLF4	GGA3	0.16008
8960	EBNA-LP	BGLAP	0.17892
8961	EBNA-LP	CTNNA3	0.21676
8962	BGLF4	RYK	0.077998
8963	EBNA4	TPD52L2	0.13165
8964	BGLF4	RNASE1	0.19538
8965	EBNA3	NLRP12	0.23603
8966	BGLF4	CCBE1	0.049259
8967	EBNA1	EFNB1	0.14396
8968	BBRF2	MT1F	0.011195
8969	BMLF1	ZNF335	0.28277
8970	BMLF1	HSD17B14	0.33153
8971	EBNA3	ELAVL2	0.43092
8972	BMLF1	TDP2	0.24034
8973	gN	KIAA1841	0.015868
8974	EBNA-LP	NPBWR2	0.31
8975	BTRF1	LYZL2	0.039564
8976	EBNA3	RBMY1B	0.39563

8977	EBNA1	PPP1R12C	0.21561
8978	BGLF4	FN1	0.097918
8979	BHRF1	C2orf62	0.035858
8980	BMLF1	SLC36A1	0.055977
8981	BGLF4	MCFD2	0.0511
8982	BLLF2	DCTN2	0.013864
8983	gL	MICU1	0.053447
8984	BLLF2	CACNA2D3	0.059466
8985	BLLF2	GCLC	0.017071
8986	BZLF2	COA7	0.013651
8987	EBNA-LP	CENPJ	0.16783
8988	BGLF4	HDAC5	0.027469
8989	EBNA3	GAD2	0.18505
8990	EBNA3	BUB3	0.19452
8991	EBNA3	BPTF	0.16299
8992	BGLF4	ATP6V1E1	0.13844
8993	EBNA3	WHAMMP3	0.017197
8994	EBNA1	LRRFIP2	0.19886
8995	EBNA-LP	LEPR	0.32184
8996	BLLF1	PACRGL	0.011176
8997	BLLF1	GOLGA6L18	0.032573
8998	EBNA3	HTRA3	0.39659
8999	BGLF4	NUP214	0.13907
9000	EBNA-LP	SRSF11	0.34463
9001	BLLF2	ZNF497	0.026069
9002	EBNA1	TSPYL5	0.12692
9003	BMLF1	SYNGR1	0.038961
9004	BOLF1	CCAR1	0.16881
9005	BMLF1	ARRB2	0.1027
9006	EBNA1	E2F3	0.034629
9007	BMLF1	BRK1	0.029187
9008	BLLF2	MT1G	0.087702
9009	BZLF1	CHAMP1	0.088498
9010	EBNA1	VAV3	0.033358
9011	BMLF1	CDH24	0.19044
9012	EBNA-LP	PKM	0.1543
9013	BMLF1	MBD2	0.20981
9014	EBNA-LP	ID2	0.083651
9015	EBNA3	TLN1	0.078465
9016	BMLF1	CDC23	0.014343
9017	EBNA2	CHP1	0.05475
9018	BMLF1	PSPC1	0.33278
9019	BMLF1	MAP2	0.23259
9020	EBNA-LP	ZAK	0.11288
9021	EBNA3	IKBIP	0.3489
9022	EBNA3	SEPT7	0.087691
9023	LMP1	EDEM1	0.027985
9024	BZLF1	KIF2A	0.085656
9025	EBNA-LP	GALK2	0.033054
9026	EBNA4	STX6	0.1479
9027	BZLF1	RRAD	0.16527
9028	EBNA1	NOTCH3	0.16376
9029	BGLF4	ING5	0.12377
9030	gL	CCDC85A	0.013116
9031	EBNA-LP	DLX4	0.19544
9032	BGLF4	THOC1	0.176
9033	BOLF1	SUV39H2	0.015972
9034	EBNA-LP	SMAD4	0.025288
9035	BZLF1	ARID5B	0.081594
9036	EBNA-LP	DCTN1	0.2359
9037	BZLF1	PCDHGC4	0.049103
9038	EBNA3	HSPE1	0.28702
9039	EBNA1	ATG5	0.13621
9040	BGLF4	SPSB4	0.22645
9041	EBNA-LP	ECM29	0.015554
9042	BGLF4	UMOD	0.066526
9043	EBNA1	ELL	0.13491
9044	BNLF2b	CCAR2	0.079254

EBNA3	TK2	0.024796
EBNA1	PLOD3	0.12746
EBNA4	SUV39H2	0.036354
EBNA-LP	DYNLRB1	0.28207
BZLF1	CLK2	0.093733
BMLF1	CEACAM6	0.38978
BZLF1	MADD	0.15754
EBNA-LP	TRIM24	0.049913
BMLF1	POLI	0.2526
BVRF1	CFTR	0.011761
BMLF1	UBTF	0.26927
EBNA1	ECM1	0.099136
BGLF4	EIF4A3	0.1224
gL	MYL6B	0.049259
LMP2	C1orf195	0.072065
BLLF1	C15orf65	0.015699
BLLF2	HYAL2	0.01079
BZLF1	SCN5A	0.061226
BHRF1	ACTN2	0.050501
BRRF1	FAT4	0.20906
EBNA-LP	SMARCA4	0.10612
BZLF1	SLC9A3R2	0.095246
EBNA-LP	ATG9A	0.35786
BXRF1	COMP	0.12555
BGLF4	TAF11	0.17397
EBNA-LP	IL6R	0.14021
EBNA3	PCDHGC4	0.18006
BGLF4	MYL10	0.066162
BMLF1	HIPK1	0.13346
BLLF1	S100A8	0.041828
EBNA6	CASC3	0.20417
BLLF2	CLDN8	0.011729
BLLF2	ZNF182	0.016033
BMLF1	TMEM173	0.11469
EBNA6	TRMT61B	0.10108
BHRF1	GAGE12H	0.061401
BMLF1	PEX6	0.30308
BMLF1	PRDX2	0.053784
BZLF1	RMDN3	0.074808
BMLF1	DDN	0.32679
EBNA-LP	RHOH	0.26255
BGLF4	TRAPPC4	0.2138
BGLF4	CELSR1	0.02832
EBNA-LP	VAMP3	0.23867
BZLF1	NTM	0.17644
EBNA1	MALL	0.15915
EBNA3	TMPO	0.32481
BPLF1	UPF3A	0.091945
EBNA1	CBX1	0.085772
EBNA1	TCF4	0.03294
BZLF1	COMMD7	0.19672
BMLF1	EFCAB10	0.24702
BGLF4	PGS1	0.03713
BGLF4	CD151	0.1795
EBNA2	STIM2	0.10605
BZLF1	ZIC3	0.01847
EBNA-LP	SLA2	0.32874
EBNA-LP	SLC25A17	0.19778
BMLF1	STXBP4	0.18759
EBNA-LP	AGGF1	0.29804
EBNA1	MAPK9	0.049904
BZLF1	SDIM1	0.22173
EBNA3	RB1CC1	0.13357
EBNA-LP	MAGEC2	0.34421
BGLF4	CTNNBIP1	0.12931
BHRF1	CCDC69	0.061393
BMLF1	CHORDC1	0.33176
EBNA3	SYMPK	0.2921

9113	EBNA4	MASP1	0.23196
9114	EBNA-LP	POLR2J	0.15924
9115	BPLF1	THBS2	0.12001
9116	EBNA-LP	SLC4A1AP	0.31355
9117	EBNA1	SCAP	0.12058
9118	BPLF1	CALU	0.11652
9119	BZLF1	NPC1L1	0.23341
9120	EBNA3	UMODL1	0.16647
9121	BVRF1	ANXA7	0.068297
9122	EBNA-LP	E2F5	0.10647
9123	EBNA-LP	PPEF1	0.52025
9124	BMLF1	MIB1	0.24012
9125	BGLF4	PSMC5	0.051868
9126	EBNA1	TGFB1I1	0.078404
9127	EBNA3	PLA2G4B	0.039817
9128	BGLF4	TGM1	0.20962
9129	BMLF1	PCSK5	0.023679
9130	EBNA3	TRIP12	0.29144
9131	EBNA3	EHMT1	0.27999
9132	BXRF1	LRCOL1	0.049472
9133	BHRF1	C7orf62	0.074454
9134	EBNA-LP	CCNC	0.13687
9135	EBNA1	EEF1G	0.14527
9136	BMLF1	SUSD1	0.24729
9137	BMLF1	RGR	0.32502
9138	BZLF1	BLMH	0.13037
9139	EBNA3	C14orf178	0.055925
9140	gN	FAM188A	0.082881
9141	BGLF4	PCDHGB3	0.058173
9142	EBNA-LP	MEPE	0.38288
9143	BBRF2	LYZL1	0.070543
9144	EBNA-LP	HIC1	0.086042
9145	EBNA3	MINK1	0.13258
9146	BMLF1	TAF9	0.14656
9147	EBNA4	LRP2	0.080507
9148	BZLF1	FBLN2	0.06383
9149	EBNA-LP	AATF	0.13667
9150	EBNA-LP	ACIN1	0.2802
9151	EBNA3	SGTA	0.37521
9152	EBNA1	RNF31	0.087359
9153	BGLF4	LAT	0.092381
9154	BNLF2b	STIM2	0.012218
9155	BZLF2	GOLGA6L4	0.072534
9156	BZLF1	CYLD	0.033214
9157	EBNA1	CITED1	0.017097
9158	BZLF1	MYL6	0.3813
9159	EBNA3	KRTAP13-4	0.053578
9160	BZLF2	FAM178A	0.066087
9161	BHRF1	ATP6V0A1	0.01919
9162	EBNA3	PICK1	0.14088
9163	BMLF1	STAT4	0.095224
9164	BZLF2	C16orf45	0.013883
9165	BZLF1	RNF40	0.099925
9166	BHRF1	ubl4ab	0.067228
9167	BZLF1	SCUBE1	0.017195
9168	EBNA3	HGS	0.18299
9169	EBNA-LP	SLC24A1	0.3241
9170	EBNA1	PRKAB2	0.025819
9171	BZLF2	CCDC67	0.010356
9172	EBNA6	SMN1	0.060136
9173	EBNA1	SREBF2	0.022047
9174	BMLF1	MED20	0.24347
9175	EBNA1	HNRNPA2B1	0.15154
9176	BPLF1	CCDC150	0.035489
9177	EBNA1	NIFK	0.17855
9178	EBNA3	NUP37	0.20164
9179	EBNA3	PDE9A	0.19327
9180	BDLF1	CCAR1	0.046934

9181	EBNA3	FEM1B	0.27724
9182	BVRF1	CDH1	0.032208
9183	BGLF4	EDA2R	0.14159
9184	BMLF1	MIF4GD	0.39296
9185	BLLF2	ZGPAT	0.011089
9186	EBNA-LP	SLC5A2	0.04072
9187	BLLF2	HAUS5	0.044414
9188	EBNA3	TRIM39	0.22564
9189	BPLF1	C17orf104	0.032612
9190	BGLF4	FZR1	0.12495
9191	BZLF1	CGN	0.19916
9192	LMP1	ANXA2	0.043681
9193	BILF1	AP1G1	0.040432
9194	EBNA-LP	YEATS4	0.18204
9195	EBNA3	CNGC19	0.16422
9196	BZLF1	KHDRBS1	0.14565
9197	BZLF1	EPS15L1	0.020124
9198	BGLF4	SF3B4	0.20362
9199	EBNA2	TNNC1	0.11618
9200	LMP2	FAM231A	0.11632
9201	EBNA-LP	TRAF1	0.27804
9202	EBNA-LP	TNIK	0.14291
9203	EBNA6	HIN1L	0.017218
9204	BLLF2	ZBTB38	0.012096
9205	BMLF1	APH1A	0.20646
9206	EBNA-LP	MMP10	0.055871
9207	EBNA1	ELOVL7	0.14151
9208	EBNA-LP	NME1	0.17496
9209	BMLF1	UBE2E1	0.18855
9210	EBNA-LP	SPTBN1	0.14139
9211	BGLF4	EPPIN	0.20345
9212	BMLF1	BLOC1S4	0.35673
9213	EBNA3	RASSF3	0.3353
9214	LMP1	FAM231A	0.091531
9215	BBRF2	C6orf10	0.079406
9216	BGLF4	TLR2	0.028247
9217	BGLF4	USP2	0.08291
9218	BMLF1	CETN1	0.47863
9219	BZLF1	PML	0.020376
9220	EBNA3	GOLGA6L18	0.093196
9221	BZLF1	GEMIN7	0.15495
9222	EBNA3	PAGR1	0.3759
9223	EBNA3	SLC1A3	0.02296
9224	EBNA3	RAB14	0.08662
9225	gL	CT45A3	0.047071
9226	BHRF1	PRMT1	0.013472
9227	BMLF1	DBT	0.012542
9228	BILF1	SNX6	0.053708
9229	EBNA3	S100A1	0.48846
9230	BZLF1	HRG	0.056287
9231	BGLF4	RRP8	0.1608
9232	EBNA1	LRPPRC	0.12947
9233	BNLF2b	CCRN4L	0.036745
9234	EBNA3	TFB1M	0.36718
9235	EBNA1	GCK	0.052647
9236	EBNA3	SLC9A3R2	0.2032
9237	EBNA1	PRSS2	0.32047
9238	BLLF2	FXYD6P3	0.065641
9239	BMLF1	PCDHGA1	0.22489
9240	BMLF1	PCDHGA9	0.22489
9241	BBRF2	OR4F6	0.029347
9242	BLLF2	SLC24A4	0.012659
9243	BMLF1	NDFIP2	0.23778
9244	BLLF2	ARMC10	0.013764
9245	EBNA1	MED26	0.10125
9246	EBNA3	PSMC1	0.13323
9247	BGLF4	MAP3K3	0.050703
9248	EBNA6	CCDC150	0.078974

9249	EBNA3	NCKAP1L	0.11999
9250	BMLF1	EMID1	0.021576
9251	EBNA3	FAM101A	0.07203
9252	BLLF2	ZNF727	0.013112
9253	BHRF1	C19orf82	0.088999
9254	BBRF2	CHMP1A	0.01041
9255	BMLF1	CHMP4A	0.14411
9256	EBNA3	NLK	0.10705
9257	BPLF1	SPIDR	0.097669
9258	BMLF1	SHARPIN	0.21431
9259	EBNA3	DLAT	0.31723
9260	EBNA3	HOXA5	0.055907
9261	gL	NECAB3	0.020685
9262	EBNA-LP	RAP1GAP	0.2245
9263	EBNA6	UBQLNL	0.021215
9264	BGRF1/BDRF1	S100A13	0.16262
9265	BZLF1	FAM188B2	0.011596
9266	BZLF1	SOD3	0.09401
9267	BMLF1	MAPT	0.095104
9268	BALF2	AP1G1	0.016048
9269	BZLF1	FMR1	0.23797
9270	BZLF1	CD97	0.024897
9271	EBNA3	ZNF350	0.24952
9272	BZLF1	VAMP3	0.14301
9273	EBNA3	EIF2B2	0.25898
9274	EBNA3	MAGEF1	0.019165
9275	EBNA4	DST	0.1037
9276	BGLF4	CRYAA	0.092867
9277	EBNA-LP	DDR1	0.1341
9278	BLLF2	GOLGA8DP	0.060478
9279	BGLF4	ARVCF	0.18391
9280	BZLF1	TCHH	0.038461
9281	BZLF1	MME	0.032101
9282	BGLF4	AMOT	0.12757
9283	BMLF1	SLC17A6	0.072493
9284	BMLF1	SNX24	0.16649
9285	BGLF4	PRNP	0.056315
9286	BZLF1	TECPR1	0.18461
9287	EBNA-LP	PARVA	0.1729
9288	gM	ATP5F1	0.012344
9289	gL	ARMCX5	0.06186
9290	BBRF2	GPR179	0.097943
9291	EBNA6	C6orf223	0.27871
9292	EBNA3	RHOU	0.13348
9293	BLLF2	ATP1B4	0.024166
9294	BMLF1	FBN1	0.55207
9295	EBNA6	CDKN2AIPNL	0.21801
9296	BMLF1	BTBD2	0.43806
9297	EBNA-LP	PTTG1	0.20426
9298	EBNA3	PCDH12	0.1442
9299	BMLF1	LRAT	0.038413
9300	BGLF4	CLEC1B	0.19769
9301	BHRF1	GOLGA6L18	0.10513
9302	EBNA3	ZNF256	0.13409
9303	BZLF2	C16orf11	0.019415
9304	EBNA6	FAM178B	0.12777
9305	BGLF4	TXLNB	0.22975
9306	BZLF1	CAV2	0.069654
9307	BZLF1	HMOX2	0.11085
9308	BZLF1	PTPN13	0.03271
9309	EBNA3	MCU	0.28213
9310	BMLF1	DPEP1	0.16405
9311	BMLF1	NFYB	0.20466
9312	BZLF1	ARHGAP32	0.077244
9313	BPLF1	THBS1	0.015111
9314	BGLF4	RAD51C	0.1081
9315	EBNA-LP	PTPN1	0.095918
9316	BMLF1	SIK1	0.086992

9317	BMLF1	GTF2A2	0.19667
9318	EBNA4	CLPP	0.16714
9319	BGLF4	RAD17	0.1211
9320	BHRF1	S100A7	0.095078
9321	EBNA3	NFU1	0.056864
9322	BGLF4	DUSP15	0.047426
9323	BGLF4	SDIM1	0.22642
9324	EBNA1	NUDT18	0.17322
9325	BGRF1/BDRF1	UTRN	0.079869
9326	EBNA-LP	MMP1	0.05205
9327	EBNA-LP	ZNF655	0.21453
9328	EBNA1	NAPA	0.1264
9329	BLLF2	ZNF561	0.013776
9330	EBNA4	NBP35	0.010892
9331	EBNA1	SPINK9	0.19084
9332	BGLF4	HJURP	0.10782
9333	EBNA3	ACTL6A	0.17427
9334	BZLF1	APPBP2	0.19476
9335	EBNA-LP	DMC1	0.1939
9336	BGLF4	MFI2	0.14009
9337	BGLF4	CHD4	0.043157
9338	EBNA3	GRIP1	0.014394
9339	BLLF2	MS4A12	0.01528
9340	BMLF1	CHGA	0.093778
9341	BZLF1	C1R	0.10007
9342	BGLF4	SH3GL2	0.12149
9343	EBNA-LP	RBM12B	0.48723
9344	BZLF1	SFR1	0.2118
9345	EBNA3	HSPB1	0.077151
9346	EBNA-LP	SEC31A	0.21041
9347	EBNA4	CLHC1	0.012553
9348	EBNA-LP	APIP	0.1926
9349	EBNA1	NOP56	0.2013
9350	BMLF1	YOD1	0.28164
9351	BPLF1	FAM47B	0.051713
9352	EBNA4	AMER1	0.27946
9353	BTRF1	UBLCP1	0.010473
9354	EBNA1	COPS6	0.15497
9355	BGLF4	NOX1	0.060435
9356	BMLF1	DEPDC1	0.28754
9357	EBNA6	PLSCR4	0.14205
9358	BZLF1	RHBDL3	0.14662
9359	BGLF4	IL15RA	0.19855
9360	BLLF2	ZDHHC23	0.010168
9361	EBNA3	PPIB	0.33952
9362	EBNA3	FAM83F	0.10548
9363	BMLF1	ARFGEF2	0.14398
9364	BMLF1	TENC1	0.24341
9365	BZLF1	TRAF3IP1	0.2221
9366	BZLF1	KNSTRN	0.11963
9367	EBNA1	AES	0.092308
9368	BVRF1	MAP1LC3B	0.026954
9369	BZLF1	ATF6	0.010686
9370	EBNA-LP	PALM	0.18244
9371	EBNA1	RPL5	0.11211
9372	EBNA3	NCOA2	0.08959
9373	EBNA1	ADAM15	0.10824
9374	EBNA-LP	ASH2L	0.21174
9375	BZLF1	PSPC1	0.19189
9376	EBNA3	EDN1	0.012324
9377	EBNA1	RHBDL3	0.13671
9378	BBRF2	OR2T34	0.030153
9379	EBNA3	PORCN	0.067982
9380	BARF1	PRSS3	0.21621
9381	BGLF4	PEG10	0.14422
9382	BRRF1	CDH23	0.072651
9383	BZLF1	DLG4	0.016454
9384	BHRF1	ANGEL1	0.029744

9385	BHRF1	ATP2B2	0.010006
9386	EBNA3	RAB5B	0.25584
9387	BMLF1	HDAC2	0.031041
9388	EBNA3	FAM131C	0.023068
9389	BZLF1	ROBO1	0.077207
9390	BLLF1	KIAA1841	0.032403
9391	gN	IL6R	0.01508
9392	BGRF1/BDRF1	FNBP1L	0.025903
9393	EBNA-LP	TBP	0.04956
9394	EBNA-LP	CD2AP	0.28424
9395	BDLF1	CADPS	0.097486
9396	BLLF2	TNFRSF18	0.01651
9397	BGRF1/BDRF1	NOTCH2	0.021301
9398	EBNA-LP	CASK	0.13885
9399	BHRF1	NKAPL	0.12937
9400	EBNA-LP	TIFA	0.38858
9401	BGLF4	LAX1	0.10536
9402	EBNA-LP	RERE	0.14679
9403	BMLF1	TXNIP	0.17405
9404	BZLF1	PGR	0.020247
9405	BGLF4	PCDH17	0.39838
9406	BVRF1	WLS	0.014353
9407	BBRF2	CTIF	0.018511
9408	BMLF1	BUB1	0.12871
9409	BZLF1	BAP1	0.087264
9410	EBNA3	GLB1	0.17628
9411	BZLF1	SFTPC	0.13931
9412	EBNA-LP	PPP2R5D	0.29059
9413	BARF1	DHH	0.14521
9414	BMLF1	IL5	0.29072
9415	EBNA-LP	NELFA	0.25484
9416	EBNA1	CELSR3	0.28499
9417	EBNA-LP	SMARCC2	0.19268
9418	BGLF4	SNW1	0.1223
9419	EBNA-LP	CIR1	0.15049
9420	BMLF1	TGM4	0.025124
9421	EBNA-LP	TYRO3	0.13851
9422	BDLF1	S100A6	0.021258
9423	BGLF4	TADA3	0.052056
9424	EBNA1	EGLN1	0.085491
9425	BBRF2	LYZL2	0.072652
9426	BMLF1	ATP5G2	0.068194
9427	EBNA1	SKP1	0.14338
9428	BZLF1	MAP4K1	0.10042
9429	BVRF1	NPIPA5	0.010434
9430	EBNA-LP	MYO9A	0.22382
9431	EBNA1	USP8	0.080231
9432	BGLF4	DAB2	0.082127
9433	EBNA3	FBN2	0.17049
9434	EBNA-LP	HIP1	0.1311
9435	BMLF1	WDYHV1	0.3418
9436	BLLF2	SOGA1	0.062016
9437	BMLF1	RBBP7	0.22302
9438	gL	DAG1	0.075037
9439	EBNA3	HRNR	0.15926
9440	BMLF1	CRLF1	0.34645
9441	BGLF4	ATP6AP2	0.16658
9442	BMLF1	NEU4	0.24366
9443	BZLF1	HMG20B	0.052594
9444	BZLF1	DDX39A	0.13388
9445	EBNA1	EIF3C	0.22336
9446	BALF2	CANX	0.057493
9447	BRRF1	SRI	0.1117
9448	BLLF2	GKN2	0.043579
9449	BGLF4	ORM1	0.21523
9450	BMLF1	SEPT7	0.11556
9451	BMLF1	ANXA1	0.089947
9452	EBNA-LP	TRMT112	0.47251

EBNA-LP	SLX1A	0.27633
EBNA-LP	BBS1	0.30454
EBNA1	GAD1	0.084522
BZLF1	PITPNM3	0.37101
BGLF4	NCAN	0.025992
BMLF1	PEX1	0.18866
BZLF1	SMC5	0.13264
BMLF1	MEGF8	0.53519
BZLF2	CCDC146	0.048483
BZLF1	BTK	0.016007
EBNA3	PCDHB16	0.16421
EBNA2	PRF1	0.047335
EBNA-LP	FBXL8	0.43184
BGLF4	GNB2L1	0.026912
BBRF2	MT1L	0.011424
EBNA3	ZFYVE9	0.35833
EBNA1	CCAR2	0.19533
BILF1	CDH1	0.035353
BARF1	MYL6	0.20779
EBNA-LP	PHYH	0.1657
BZLF1	RNF115	0.14136
LMP2	DTNA	0.03772
EBNA3	TAF4	0.091271
BMLF1	GALK2	0.085831
EBNA3	DRD2	0.020493
BMLF1	ZC3H7B	0.31618
BPLF1	F2	0.029461
EBNA6	C1orf111	0.13898
EBNA4	FAM78A	0.037043
EBNA3	AP1G2	0.30607
BPLF1	SECISBP2L	0.09792
EBNA3	TCL1B	0.01021
BBRF2	OR5L2	0.03752
EBNA1	USP6	0.11342
BXRF1	CDK5R1	0.025903
BHRF1	CTAGE15	0.021352
BZLF1	SMUG1	0.13161
BDLF1	MPTX1	0.01008
BLLF1	CTAGE15	0.010761
BMLF1	DNAJC19	0.018009
BMLF1	A1CF	0.27118
EBNA6	CCDC28A	0.27966
BZLF1	TLN1	0.021079
BGLF4	PPP6C	0.13979
EBNA3	NAA15	0.22165
BGLF4	DYRK1B	0.046599
BMLF1	SLC6A12	0.022568
BMLF1	ASB9	0.36411
EBNA1	EDIL3	0.038476
BGLF4	RPS27L	0.13643
EBNA1	AIRE	0.03587
EBNA1	SLC7A11	0.087436
EBNA3	TOMM7	0.24864
BPLF1	MATN4	0.15008
EBNA-LP	GTF2B	0.18954
BGLF4	TOMM70A	0.14729
BMLF1	PDGFRA	0.03932
BLLF2	ZNF32	0.021782
BGRF1/BDRF1	CLIC4	0.07394
EBNA3	MCTP2	0.18784
BALF2	S100A4	0.14115
EBNA1	MAPK11	0.046968
BBRF2	ZWINT	0.016488
BLLF2	TRMT5	0.013457
EBNA3	RNASE1	0.35477
BMLF1	CDK4	0.09538
BALF2	KCNIP1	0.095393
BZLF1	SNRPE	0.13281

9521	BGLF4	RAD51	0.050892
9522	BZLF1	POLRMT	0.16664
9523	BNLF2b	SPPL2A	0.019076
9524	BARF1	CLIP1	0.088777
9525	EBNA4	S100A8	0.080554
9526	BGLF4	CACTIN	0.13935
9527	EBNA-LP	FNIP2	0.31968
9528	EBNA3	NCOA3	0.16239
9529	EBNA4	C10orf82	0.16114
9530	BLLF2	RPL8	0.071576
9531	BZLF1	NRD1	0.012979
9532	EBNA4	CXorf30	0.20611
9533	BGLF4	KIT	0.016217
9534	BBRF2	NCR3	0.036831
9535	BZLF1	MECOM	0.022456
9536	EBNA-LP	TPCN2	0.11353
9537	LMP1	DST	0.047744
9538	EBNA3	PCDH20	0.18006
9539	EBNA1	PPM1G	0.063364
9540	BZLF1	SLBP	0.14547
9541	EBNA-LP	HIF1AN	0.16086
9542	BZLF1	FBF1	0.16357
9543	EBNA3	PCDHA5	0.16911
9544	BZLF1	CBX8	0.14131
9545	BOLF1	CHAF1A	0.038706
9546	BLLF2	ZNF44	0.011301
9547	BZLF1	BRE	0.14787
9548	EBNA-LP	UBTF	0.24437
9549	EBNA-LP	PEX12	0.27547
9550	BPLF1	FAM49B	0.034276
9551	EBNA3	RNF43	0.17796
9552	BOLF1	ANXA6	0.16501
9553	BBRF2	TMEM98	0.073004
9554	BOLF1	CAPN2	0.013095
9555	BMLF1	GIT2	0.28549
9556	EBNA1	FBXO46	0.22232
9557	BALF2	EFEMP2	0.14074
9558	EBNA-LP	STRN3	0.15982
9559	BBRF2	ZER1	0.013177
9560	BMLF1	ARHGEF6	0.20753
9561	EBNA3	RPS6KA5	0.05847
9562	BLLF2	TAF11	0.073167
9563	EBNA3	STON2	0.32194
9564	BOLF1	NELL2	0.026258
9565	EBNA1	UPF2	0.2084
9566	BZLF1	ZNF496	0.036356
9567	BZLF1	KEL	0.10395
9568	BMLF1	SERGEF	0.34204
9569	EBNA3	AFF4	0.17797
9570	BHRF1	C11orf65	0.025332
9571	BOLF1	PLSCR3	0.14767
9572	BMLF1	GSTM2	0.04854
9573	EBNA-LP	GPX8	0.030447
9574	EBNA-LP	APLF	0.24313
9575	BGLF4	CTNND1	0.084343
9576	EBNA-LP	WWTR1	0.14386
9577	BGLF4	PLXNB3	0.17725
9578	BMLF1	TAF7	0.093709
9579	EBNA6	CCDC172	0.030833
9580	BHRF1	C14orf178	0.052743
9581	EBNA3	TRMT10B	0.059675
9582	BMLF1	CDKN1C	0.28829
9583	EBNA3	PLEKHA5	0.26993
9584	BZLF1	KCNMB4	0.15173
9585	EBNA-LP	MGARP	0.23363
9586	BMLF1	SLC17A1	0.058692
9587	BRRF1	JAG2	0.13695
9588	BMLF1	PI4KAP2	0.11592

9589	BMLF1	MINK1	0.1479
9590	BPLF1	CUEDC1	0.052338
9591	BGLF4	IKZF3	0.051776
9592	BLLF1	CALR	0.013409
9593	EBNA1	BRD4	0.11617
9594	BPLF1	MBD3L5	0.011827
9595	EBNA3	PLCG2	0.0939
9596	BMLF1	SAA2	0.029317
9597	BZLF1	URM1	0.19729
9598	EBNA3	EPHA3	0.15607
9599	BLLF2	CHML	0.010042
9600	gM	SYT2	0.090407
9601	BMLF1	ALDH5A1	0.03637
9602	BGLF4	DBNL	0.13422
9603	BMLF1	MMP9	0.07879
9604	BZLF1	MRPL28	0.18495
9605	EBNA3	RPTOR	0.1344
9606	BZLF1	NOP2	0.29812
9607	EBNA4	SZRD1	0.12086
9608	EBNA3	GSTO1	0.011374
9609	BZLF1	GCC2	0.10546
9610	EBNA3	TOX4	0.34808
9611	EBNA3	DVL2	0.14534
9612	BPLF1	C5orf58	0.08206
9613	BMLF1	DSCC1	0.31892
9614	BMLF1	ERLIN2	0.3481
9615	EBNA4	GCK	0.011176
9616	EBNA1	MAP4K5	0.095502
9617	EBNA-LP	MOV10	0.2872
9618	BZLF1	HPX	0.11277
9619	EBNA4	ADM	0.040345
9620	EBNA1	TLE1	0.068333
9621	EBNA-LP	PKDREJ	0.16409
9622	BDLF1	LRCOL1	0.022648
9623	BMLF1	CUL3	0.14865
9624	EBNA1	DGKG	0.058029
9625	EBNA-LP	PPP3CB	0.19903
9626	EBNA1	PIK3CG	0.056962
9627	BLLF2	OR4F3	0.07986
9628	EBNA-LP	RBP4	0.15345
9629	EBNA1	CDH22	0.038413
9630	BPLF1	FAM229B	0.0599
9631	EBNA-LP	UBB	0.050245
9632	EBNA1	GH1	0.11474
9633	BGLF4	RHOT2	0.19152
9634	EBNA-LP	ZWILCH	0.30259
9635	EBNA3	STK38	0.11059
9636	EBNA3	PLCB1	0.04198
9637	EBNA1	TPSAB1	0.21321
9638	EBNA-LP	NUAK1	0.19883
9639	gL	PGBD4	0.047106
9640	EBNA1	C19orf57	0.2042
9641	BMLF1	PARK7	0.20851
9642	EBNA1	HID1	0.14206
9643	BARF1	PLCE1	0.021068
9644	EBNA6	GAGE5	0.061919
9645	BGLF4	VSNL1	0.068073
9646	BGLF4	TGFBR2	0.012712
9647	BMLF1	DRG1	0.26904
9648	BLLF2	ZNF808	0.012141
9649	BGLF4	ARL6IP4	0.20848
9650	EBNA3	NPHP3	0.21554
9651	EBNA1	TEAD4	0.030946
9652	EBNA3	CUBN	0.064092
9653	BHRF1	PRDX6	0.014369
9654	BZLF1	POLR2J	0.070067
9655	EBNA1	HNRNPD	0.09269
9656	EBNA4	ELN	0.28557

9657	EBNA1	MLLT1	0.13143
9658	BHRF1	GTF2E2	0.1344
9659	BMLF1	CABP1	0.34867
9660	EBNA3	NR1I2	0.11167
9661	BZLF1	RAB11A	0.096241
9662	BZLF1	PRCP	0.21845
9663	BBRF2	CTAGE3P	0.010068
9664	EBNA6	SPANXA2-OT1	0.11536
9665	BGLF4	PDZK1	0.20105
9666	BZLF1	DHX30	0.20506
9667	EBNA1	CRHR2	0.11249
9668	BGLF4	DLK2	0.080107
9669	EBNA6	GRIN1	0.058991
9670	EBNA4	MUC1	0.28996
9671	EBNA3	BCORP1	0.056087
9672	BMLF1	PCSK4	0.076158
9673	BMLF1	ANXA3	0.14206
9674	BGLF4	MYB	0.064552
9675	EBNA3	PSMA7	0.085252
9676	BMLF1	CHD1L	0.29596
9677	BMLF1	NFYC	0.1767
9678	BZLF1	SELE	0.099407
9679	EBNA1	RSPO1	0.11069
9680	EBNA3	RCOR1	0.20346
9681	BMLF1	MT-ND3	0.3231
9682	EBNA-LP	HNRNPC	0.2941
9683	EBNA-LP	SHANK1	0.15834
9684	EBNA3	EFCAB7	0.21072
9685	EBNA3	GZMB	0.27665
9686	EBNA1	NECAB3	0.33728
9687	BZLF1	TSKS	0.10834
9688	EBNA-LP	TBRG1	0.3194
9689	EBNA1	TSHR	0.13031
9690	EBNA1	TBC1D24	0.15509
9691	BGLF4	ACP1	0.049028
9692	BGRF1/BDRF1	N4BP2L1	0.010534
9693	EBNA6	GPRIN3	0.099856
9694	BZLF1	KLF13	0.088234
9695	EBNA-LP	SMAD1	0.016622
9696	BZLF1	CDH20	0.047672
9697	BMLF1	STRIP1	0.37904
9698	BZLF1	TBK1	0.05062
9699	BLLF2	NXPE4	0.024982
9700	BZLF1	ABCD3	0.12841
9701	EBNA3	NPIPA5	0.018436
9702	EBNA3	BOP1	0.24683
9703	EBNA3	COMMD5	0.37746
9704	EBNA3	HMGB1	0.082634
9705	BMLF1	CIR1	0.18653
9706	EBNA-LP	NCSTN	0.15379
9707	BGLF4	NAA15	0.13407
9708	EBNA-LP	IRF6	0.065815
9709	EBNA3	PRAM1	0.45195
9710	BZLF1	SRPK1	0.051026
9711	BZLF1	CLIC1	0.11562
9712	BMLF1	MAU2	0.34512
9713	EBNA6	PP632	0.042723
9714	BLLF2	KCNJ4	0.011702
9715	BGLF4	CEP97	0.21441
9716	BGLF4	INPP5D	0.069391
9717	EBNA6	CCDC54	0.038047
9718	BMLF1	PSEN2	0.036013
9719	EBNA1	LAMB3	0.074374
9720	BZLF1	RWDD1	0.23617
9721	EBNA3	NELFA	0.24373
9722	BZLF2	IQCF3	0.013405
9723	gB	ACTN3	0.077391
9724	BZLF1	AATF	0.054482

9725	EBNA3	STK38L	0.10918
9726	BMLF1	BUB1B	0.11335
9727	BGLF4	PAX9	0.17451
9728	BGLF4	DAO	0.10477
9729	BMLF1	FOXP3	0.01518
9730	BGLF4	BBS4	0.03941
9731	EBNA4	ATP6V0A1	0.047321
9732	BGLF4	MYL1	0.053773
9733	EBNA-LP	NEDD9	0.2284
9734	EBNA2	RPH3A	0.053937
9735	EBNA4	BLACE	0.011718
9736	EBNA1	GTF2H1	0.043039
9737	BMLF1	RRAS2	0.26751
9738	BZLF1	CSF2	0.12714
9739	LMP1	FAM188A	0.052866
9740	BGLF4	SLC9A3R1	0.061252
9741	EBNA1	PCDHGB4	0.037277
9742	EBNA4	FEZ1	0.068493
9743	BZLF1	EEA1	0.14037
9744	EBNA-LP	SETD1B	0.32929
9745	BZLF1	BIRC7	0.11583
9746	BHRF1	MB21D2	0.049364
9747	BPLF1	CAPNS1	0.029111
9748	BMLF1	TLR1	0.037803
9749	EBNA2	IQGAP1	0.01133
9750	BGLF4	PTPRD	0.031406
9751	EBNA1	ADI1	0.11565
9752	BALF2	S100A9	0.032343
9753	BZLF1	PSMC3	0.05295
9754	gB	FAM47C	0.01769
9755	EBNA4	PDE6D	0.069924
9756	EBNA1	ARPC5	0.13475
9757	EBNA3	WAPAL	0.19517
9758	BLLF2	TAF4	0.014955
9759	BPLF1	NCOA6	0.094891
9760	BGLF4	LILRB4	0.16029
9761	BZLF2	C1orf140	0.065371
9762	EBNA1	SRSF3	0.18412
9763	BZLF2	NPCDR1	0.010184
9764	BMLF1	ZNF639	0.13424
9765	EBNA3	PIP5K1A	0.15384
9766	EBNA-LP	DDIT3	0.092433
9767	BHRF1	C19orf66	0.095977
9768	BMLF1	MAPK8	0.1036
9769	EBNA3	KHDRBS3	0.3354
9770	BILF1	BICD1	0.014792
9771	gN	RAB11FIP4	0.18063
9772	EBNA-LP	TAF1	0.080027
9773	BILF1	PCDH17	0.17982
9774	BGLF4	MRPS12	0.15295
9775	EBNA-LP	RAF1	0.034947
9776	gN	CCDC137	0.015797
9777	EBNA-LP	UNQ6126/PRO20091	0.011222
9778	BMLF1	SLC16A9	0.03047
9779	EBNA1	RFC4	0.059689
9780	EBNA3	STRAP	0.23033
9781	LMP2	FAM188B2	0.0474
9782	EBNA-LP	MYL9	0.14581
9783	BBRF2	DNASE1L2	0.088344
9784	EBNA3	VAPB	0.14256
9785	BGLF4	IGSF8	0.1898
9786	EBNA-LP	CABP2	0.17448
9787	EBNA-LP	SLC3A2	0.1913
9788	EBNA2	FAM83F	0.019793
9789	EBNA-LP	ATP10D	0.21833
9790	EBNA1	PKN2	0.035934
9791	BZLF1	ITM2C	0.14917
9792	BGLF4	ATP6V0C	0.12701

9793	BMLF1	SMC6	0.30966
9794	EBNA1	TDGF1	0.04552
9795	EBNA3	ATG9A	0.36427
9796	BZLF1	NR0B1	0.032925
9797	BGLF4	PDCD1	0.17931
9798	BGLF4	MED31	0.15748
9799	BMLF1	BRAF	0.19519
9800	EBNA3	SLC51A	0.14511
9801	EBNA-LP	MAP1S	0.15417
9802	EBNA3	GSN	0.29022
9803	BGLF4	CRY1	0.060123
9804	BGLF4	SERPINC1	0.1505
9805	BLLF1	SHANK2-AS3	0.010281
9806	BMLF1	IRGM	0.011535
9807	EBNA1	ADM	0.058795
9808	BMLF1	PRMT1	0.34553
9809	EBNA-LP	AQP6	0.053508
9810	EBNA4	RNMT	0.070404
9811	BGLF4	RAMP1	0.098313
9812	BZLF2	MAGEF1	0.013409
9813	BBRF2	FXYD6P3	0.021276
9814	EBNA-LP	TAX1BP3	0.28302
9815	EBNA3	LMNA	0.17378
9816	BZLF1	PRTN3	0.20614
9817	BZLF1	MYH2	0.12288
9818	EBNA1	GZMA	0.1639
9819	BMLF1	RBP4	0.15109
9820	EBNA3	BBS1	0.31104
9821	gL	RBBP8NL	0.03826
9822	BPLF1	CCDC153	0.01098
9823	BGLF4	SRC	0.014906
9824	BZLF1	ARHGEF1	0.070361
9825	EBNA3	RAB5A	0.2044
9826	BGLF4	MCTP1	0.061609
9827	BGLF4	SPPL2B	0.11194
9828	BMLF1	CHGB	0.43778
9829	BMLF1	RBM38	0.015281
9830	BGLF4	BTK	0.016164
9831	BLLF2	ZNF738	0.017539
9832	BMLF1	EDAR	0.27431
9833	BZLF1	CAST	0.24285
9834	EBNA3	AMER3	0.1121
9835	BMLF1	RASEF	0.20536
9836	BZLF1	EPAS1	0.012126
9837	BZLF1	UBE2H	0.11844
9838	BGRF1/BDRF1	MLC1	0.025354
9839	EBNA3	REEP5	0.387
9840	BZLF2	C9orf171	0.06708
9841	BGLF4	GLS2	0.14252
9842	BZLF1	DPP4	0.12761
9843	BZLF1	PRC1	0.03046
9844	BBRF2	CGREF1	0.022697
9845	BMLF1	SSRP1	0.27141
9846	EBNA3	NR2C2	0.093063
9847	EBNA-LP	CALB1	0.42744
9848	BPLF1	C22orf43	0.042858
9849	BLLF2	ZNF493	0.013082
9850	BMLF1	CMA1	0.042552
9851	BZLF1	YAP1	0.058227
9852	BGLF4	TRIM37	0.096711
9853	BMLF1	SDHA	0.34201
9854	BLLF2	ZNF878	0.01476
9855	EBNA-LP	PIK3CB	0.15592
9856	BMLF1	RET	0.21548
9857	BLLF2	TSPAN18	0.015222
9858	LMP2	THBS1	0.011777
9859	EBNA3	ERCC4	0.11582
9860	BLLF2	KCNJ18	0.015531

9861	BMLF1	TMPRSS4	0.039928
9862	BMLF1	ATP5O	0.086257
9863	EBNA-LP	SCUBE2	0.20358
9864	EBNA3	EQTN	0.091053
9865	EBNA3	CCDC130	0.41354
9866	BRRF1	CDH1	0.040425
9867	BGLF4	CDCA5	0.14699
9868	EBNA-LP	MED23	0.22115
9869	EBNA6	TP53INP1	0.15514
9870	EBNA3	BAIAP2	0.1677
9871	BVRF1	TESC	0.10298
9872	BGLF4	GADD45G	0.16078
9873	BGLF4	LIMD1	0.077343
9874	EBNA-LP	CCBE1	0.16105
9875	BMLF1	KLF13	0.22855
9876	BMLF1	LRP6	0.021143
9877	EBNA4	YMR013W-A	0.21004
9878	EBNA3	KANSL1	0.33278
9879	BLLF1	C14orf80	0.032736
9880	EBNA1	CD27	0.12637
9881	BILF1	CNRIP1	0.025483
9882	EBNA-LP	IKBKB	0.038881
9883	EBNA4	TPD52L1	0.2321
9884	EBNA1	TNFRSF1A	0.067266
9885	EBNA-LP	ARSA	0.056937
9886	EBNA1	MUC5B	0.17841
9887	BPLF1	ATP2A1	0.039082
9888	BGRF1/BDRF1	SPTA1	0.075792
9889	BZLF1	ECH1	0.1181
9890	EBNA1	PEX12	0.13306
9891	EBNA1	CARD11	0.08975
9892	EBNA-LP	RUNX1	0.10898
9893	EBNA1	CD72	0.12366
9894	BZLF1	MT-CO1	0.076129
9895	BGLF4	PPP6R3	0.1923
9896	EBNA3	CALML4	0.21072
9897	BTRF1	SPACA3	0.037135
9898	BMLF1	KREMEN1	0.40552
9899	BMLF1	AP1S3	0.060597
9900	BPLF1	MYL2	0.1515
9901	EBNA1	PRF1	0.23714
9902	BHRF1	RNF216P1	0.081379
9903	BMLF1	CDC73	0.19387
9904	EBNA1	POLI	0.080372
9905	BMLF1	NHLRC1	0.27217
9906	BGLF4	CFTR	0.083806
9907	BMLF1	USHBP1	0.44049
9908	BTRF1	OAS3	0.013328
9909	BZLF1	PPM1E	0.067625
9910	BZLF1	VEGFC	0.091889
9911	EBNA3	FBXW5	0.19378
9912	BPLF1	S100A1	0.074047
9913	BILF1	MUC1	0.089121
9914	BMLF1	TSSK6	0.22609
9915	BMLF1	RASSF5	0.36075
9916	BMLF1	BRCA2	0.084854
9917	BMLF1	NR4A2	0.080166
9918	gL	YBR056C-B	0.092977
9919	EBNA-LP	BRD3	0.27103
9920	EBNA3	EHMT2	0.2415
9921	EBNA-LP	SDF4	0.47976
9922	BVRF1	DYDC2	0.013492
9923	BHRF1	CAGE1	0.014594
9924	EBNA4	HSP90B1	0.11245
9925	EBNA-LP	UFL1	0.2779
9926	EBNA1	PCDHA10	0.035121
9927	EBNA3	MPP7	0.32258
9928	EBNA3	GJA1	0.05127

9929	BGLF4	PRKACB	0.017674
9930	EBNA3	CORO1B	0.36207
9931	EBNA-LP	FXN	0.014458
9932	BBRF2	YIPF5	0.068299
9933	BGLF4	CD244	0.19251
9934	BALF2	CNRIP1	0.010788
9935	BLLF2	SLC39A10	0.012764
9936	EBNA-LP	ECH1	0.25086
9937	EBNA3	CD1A	0.33124
9938	BZLF1	LRSAM1	0.1119
9939	EBNA-LP	PABPC1	0.18619
9940	EBNA1	KIF1B	0.16363
9941	BHRF1	ARMCX5	0.033367
9942	EBNA3	FYN	0.076325
9943	BBRF2	AGTPBP1	0.017338
9944	BMLF1	ITPKB	0.077715
9945	gM	CAGE1	0.012812
9946	EBNA1	GPKOW	0.16941
9947	BMLF1	XPO4	0.36087
9948	BGLF4	BEND7	0.24432
9949	EBNA3	GNMT	0.30242
9950	BZLF1	ARF4	0.11846
9951	BLLF2	TAS2R42	0.05756
9952	EBNA3	A2M	0.20926
9953	BGLF4	COL13A1	0.13309
9954	EBNA1	DNASE1	0.13064
9955	EBNA6	AXDND1	0.01177
9956	BZLF1	PEX19	0.12642
9957	EBNA-LP	CDH6	0.12877
9958	EBNA-LP	MYL7	0.19917
9959	EBNA4	BLOC1S1	0.23727
9960	EBNA3	PKDREJ	0.16485
9961	BGLF4	MALL	0.17904
9962	EBNA3	YTHDC1	0.35571
9963	BGLF4	EXOC3	0.21895
9964	BLLF2	HIST2H2BC	0.023068
9965	BLLF2	H2BFS	0.016318
9966	BTRF1	ZNF326	0.018394
9967	BZLF1	PDGFRL	0.015051
9968	EBNA-LP	PFDN4	0.28244
9969	BZLF1	PRKCQ	0.018271
9970	gN	CDH1	0.02266
9971	BZLF1	MAD2L1BP	0.16716
9972	BGLF4	ELOVL1	0.13809
9973	BMLF1	CYLD	0.10899
9974	EBNA3	TNFSF12	0.18918
9975	BZLF1	EIF4EBP1	0.10938
9976	BGLF4	PLSCR1	0.18325
9977	BZLF1	TYROBP	0.097037
9978	EBNA-LP	ZNF711	0.23855
9979	EBNA3	MEF2A	0.068089
9980	EBNA1	DLAT	0.17214
9981	BPLF1	FAM160B2	0.026482
9982	BNLF2b	MFSD7	0.020201
9983	EBNA6	MYOZ2	0.23599
9984	EBNA3	CCDC92	0.39788
9985	EBNA3	CSN2	0.20787
9986	EBNA3	AASDHPPT	0.33279
9987	EBNA1	SORCS1	0.18715
9988	EBNA1	SETDB1	0.15348
9989	EBNA-LP	UBE2M	0.23548
9990	EBNA4	LAMTOR2	0.10117
9991	EBNA3	ZFYVE1	0.24538
9992	BBRF2	TSPAN33	0.012614
9993	EBNA-LP	EIF4G2	0.39551
9994	EBNA1	HBA1	0.15227
9995	BMLF1	PPL	0.31651
9996	BZLF2	TFB1M	0.026085

9997	EBNA3	CXCR4	0.10286
9998	EBNA3	MAP4K3	0.23724
9999	BMLF1	ATR	0.17626
10000	EBNA-LP	ZWINT	0.23019
10001	BOLF1	VWA9	0.010913
10002	EBNA3	GTF2F1	0.11724
10003	BZLF1	PSMD9	0.088595
10004	BARF1	NUCB2	0.13862
10005	BZLF1	SOD1	0.021806
10006	BGLF4	PEF1	0.40779
10007	BZLF1	TPPP3	0.056619
10008	BLLF2	TRAPPC2	0.017591
10009	gL	WWC2-AS2	0.095946
10010	EBNA3	CCDC120	0.088797
10011	LMP1	SRI	0.033516
10012	EBNA-LP	PODXL2	0.35772
10013	BZLF1	AGXT	0.12915
10014	EBNA-LP	BBS10	0.31433
10015	BMLF1	NR1I2	0.13512
10016	BZLF2	FAM117B	0.10168
10017	EBNA3	OBSCN	0.12896
10018	BLLF2	ZNF317	0.02154
10019	LMP2	ACTN3	0.11101
10020	BMLF1	MAGEC1	0.47224
10021	BMLF1	NUTF2	0.35602
10022	EBNA3	PA2G4	0.13989
10023	EBNA3	CD244	0.34382
10024	BGLF4	PPARG	0.01243
10025	BMLF1	TIFA	0.43209
10026	BLLF2	MTX2	0.014585
10027	BOLF1	TESC	0.11169
10028	EBNA3	EFCAB4B	0.54585
10029	EBNA-LP	ACACA	0.18792
10030	EBNA3	KDM4A	0.20035
10031	BTRF1	PLD4	0.015773
10032	BHRF1	CCDC150	0.042642
10033	BGLF4	COMMD1	0.13723
10034	EBNA-LP	DCC	0.18322
10035	EBNA3	MEAF6	0.26577
10036	BZLF1	ABT1	0.154
10037	BGLF4	SNX9	0.20305
10038	EBNA-LP	CRELD2	0.6631
10039	BHRF1	RSRC1	0.13954
10040	BZLF1	SERINC1	0.12858
10041	BGLF4	PRPF19	0.13018
10042	EBNA3	JMJD6	0.13718
10043	EBNA1	NPHS2	0.17314
10044	BALF2	PPP3CA	0.015731
10045	BZLF1	SLX4IP	0.23617
10046	BZLF1	APBA1	0.11929
10047	EBNA-LP	PTGIS	0.08426
10048	EBNA-LP	UACA	0.21881
10049	BZLF1	AASDHPPT	0.18568
10050	BZLF2	PP632	0.011336
10051	BMLF1	BOC	0.29276
10052	EBNA3	IFT52	0.089096
10053	BLLF2	ZNF224	0.012084
10054	BMLF1	RBMY1D	0.011243
10055	EBNA3	FZD4	0.094702
10056	BZLF1	RASA1	0.038114
10057	BGLF4	NOTCH3	0.18253
10058	EBNA-LP	NIPBL	0.14527
10059	EBNA-LP	BAZ2A	0.20455
10060	BZLF1	DDO	0.13685
10061	EBNA-LP	NUP50	0.2616
10062	BGLF4	MSR1	0.19363
10063	EBNA3	JMJD8	0.010026
10064	BLLF2	ZNF107	0.011897

EBNA-LP	XPO6	0.3085
EBNA-LP	KCNIP1	0.42621
BMLF1	RALBP1	0.28638
EBNA3	FDXR	0.2613
BGLF4	HEG1	0.033632
BGLF4	VWF	0.11976
EBNA1	EPHB4	0.10918
gL	MAB21L3	0.022988
gL	MEGF6	0.067922
EBNA4	PLAUR	0.16154
EBNA3	MITD1	0.31263
BZLF1	ITGAX	0.15997
BGLF4	RBM12B	0.2695
EBNA1	GGA2	0.13976
BMLF1	FN3K	0.056235
BMLF1	NME6	0.025496
BGLF4	ZNF652	0.15166
BZLF1	ZFYVE16	0.06938
BGLF4	PRAM1	0.25804
BMLF1	PPP2R1A	0.10756
BMLF1	PSRC1	0.25413
BHRF1	GAGE12G	0.061401
EBNA3	TSPYL5	0.28528
EBNA3	PCDHAC2	0.16911
BTRF1	OAZ3	0.02145
BMLF1	RPUSD3	0.11174
BZLF1	KIF5B	0.11278
BMLF1	ULK3	0.23146
BNLF2b	G4	0.011625
BMLF1	LEO1	0.26074
EBNA6	LINC00574	0.011513
BPLF1	C1orf196	0.042145
EBNA3	TBC1D25	0.38928
EBNA3	ORC1	0.2038
EBNA3	EID2	0.22784
BGLF4	ASB13	0.22645
BMLF1	AKAP14	0.093072
EBNA1	PROCR	0.15936
BILF1	HDGFL1	0.011182
EBNA3	NDC80	0.21959
LMP2	PITPNM3	0.084277
EBNA-LP	ERGIC1	0.26581
EBNA3	ATP5I	0.042511
BGLF4	DCC	0.11187
BZLF1	MELK	0.21368
BMLF1	FANCM	0.25469
EBNA4	FAM27D1	0.21502
BMLF1	COL4A2	0.29193
EBNA4	C4orf17	0.048482
BLLF2	ZNF398	0.01845
EBNA6	FAM188B	0.22769
BMLF1	TEAD3	0.17917
BGLF4	ERRFI1	0.059167
EBNA4	NUP205	0.13405
BLLF2	TMEM98	0.12486
EBNA-LP	TXNDC17	0.34938
EBNA1	NHP2L1	0.1737
EBNA4	SWT1	0.10854
BZLF1	SPOCK3	0.051603
BGLF4	ZNHIT6	0.17358
BZLF1	GAD1	0.09244
BMLF1	NEK11	0.2152
gL	C10orf82	0.085914
BMLF1	STARD6	0.021189
BLLF2	SLC16A5	0.035936
BGLF4	FGF19	0.18678
BMLF1	ATXN1	0.22623
BLLF2	STX6	0.066604

10133	EBNA1	CTDSP1	0.078928
10134	BZLF1	LTN1	0.14982
10135	BBRF2	SUMF2	0.084062
10136	BLLF2	ARSG	0.011875
10137	EBNA3	PEX11G	0.33282
10138	EBNA-LP	IRS2	0.080438
10139	BGLF4	ROR1	0.092779
10140	EBNA-LP	RAB7A	0.205
10141	BNLF2b	EIF2A	0.012677
10142	EBNA3	TXNDC17	0.35415
10143	BGLF4	MIF	0.080599
10144	EBNA3	RPL30	0.044923
10145	EBNA3	GATA1	0.023364
10146	gL	CC2D2B	0.055753
10147	BVRF1	TPPP	0.1112
10148	BGRF1/BDRF1	ANXA2R	0.02844
10149	BMLF1	SOX5	0.14036
10150	EBNA3	NSUN7	0.16527
10151	EBNA3	OTUD7B	0.24041
10152	BMLF1	DTX2	0.28437
10153	EBNA1	SIRT7	0.091563
10154	BLLF2	OR5L2	0.078589
10155	BLLF2	HIC2	0.010067
10156	BPLF1	CCDC17	0.022122
10157	BLLF1	TNFAIP8L1	0.044015
10158	BGLF4	PPP2R5A	0.10356
10159	BMLF1	TCHP	0.34298
10160	BMLF1	SOCS5	0.34492
10161	BGLF4	HTATIP2	0.11361
10162	EBNA-LP	BLOC1S3	0.21239
10163	EBNA3	TRUB1	0.051547
10164	BZLF1	CDK5RAP3	0.099404
10165	BGLF4	UBA52	0.022147
10166	BZLF2	CCDC151	0.012069
10167	BLLF2	ZNF611	0.012141
10168	BMLF1	MMP27	0.10959
10169	BMLF1	IFT122	0.36142
10170	EBNA-LP	CELF1	0.26627
10171	BLLF1	YTHDF1	0.022636
10172	EBNA3	ABCG2	0.27566
10173	BZLF1	MAGEA4	0.21685
10174	BLLF1	FAM46A	0.023769
10175	gL	GAGE2E	0.037989
10176	EBNA1	RRM1	0.12559
10177	EBNA2	MASP1	0.15419
10178	BMLF1	GOLGA4	0.324
10179	EBNA1	ATF3	0.035905
10180	BHRF1	SHANK2-AS3	0.037932
10181	BMLF1	PSMG1	0.43078
10182	BVRF1	SIRT4	0.053711
10183	EBNA1	DNAJB2	0.15504
10184	BZLF1	TTC1	0.20307
10185	BMLF1	EIF2AK2	0.14402
10186	EBNA1	PSME3	0.067005
10187	BMLF1	MYL1	0.19331
10188	EBNA-LP	CCNF	0.29014
10189	BZLF1	ELF3	0.03531
10190	EBNA-LP	DOCK1	0.23382
10191	gN	GCH1	0.025407
10192	gL	SWT1	0.054717
10193	BGLF4	RSPO1	0.1279
10194	EBNA2	HSP90B1	0.068488
10195	BMLF1	RPL18A	0.043889
10196	EBNA1	NF1	0.02532
10197	BZLF1	RGS16	0.2353
10198	BZLF1	GIT1	0.1182
10199	BMLF1	DIAPH1	0.17403
10200	BMLF1	GYS1	0.21107

10201	EBNA4	YML009W-B	0.1337
10202	BARF1	FASN	0.074861
10203	BBRF2	ZNF500	0.010393
10204	BZLF1	PLK3	0.04688
10205	BGLF4	NCOA5	0.20561
10206	BMLF1	ATP1B2	0.28439
10207	EBNA1	ADAM23	0.096112
10208	EBNA1	PTPN23	0.053142
10209	BLLF2	SSBP2	0.060506
10210	EBNA3	ATG13	0.23587
10211	BGLF4	SP3	0.06678
10212	BMLF1	MYCN	0.16462
10213	BNLF2b	TM2D3	0.099448
10214	BZLF1	LPL	0.03922
10215	BMLF1	EFNA1	0.20058
10216	EBNA6	SAMD14	0.022688
10217	BHRF1	HBA1	0.071792
10218	EBNA3	ESPL1	0.19906
10219	EBNA1	ITGA2B	0.11286
10220	BZLF2	PPP2R3A	0.09851
10221	BZLF1	DUSP7	0.020638
10222	EBNA3	ACACA	0.19041
10223	EBNA3	CANT1	0.087541
10224	BMLF1	SYNM	0.30512
10225	EBNA-LP	KAT5	0.14889
10226	gM	RET	0.040386
10227	BZLF1	PRKAR1A	0.095328
10228	LMP2	CT55	0.05754
10229	gN	CLIC4	0.038022
10230	EBNA3	RANBP2	0.14614
10231	BGLF4	PPP1R13B	0.17829
10232	BZLF1	DHX8	0.17067
10233	BOLF1	CAPN1	0.062999
10234	BZLF1	KHDRBS3	0.19516
10235	EBNA-LP	CASQ2	0.13775
10236	EBNA3	OTUD1	0.033168
10237	EBNA-LP	SFTPC	0.24223
10238	BGLF4	ZNF473	0.13487
10239	BILF1	PRMT1	0.035292
10240	BILF1	TNNC1	0.18633
10241	EBNA1	FSTL1	0.025921
10242	BRRF1	NOTCH2	0.021418
10243	BLLF2	BCAS3	0.014532
10244	BLLF2	TSPY4	0.018872
10245	EBNA6	SFI1	0.14602
10246	EBNA-LP	CDKN2A	0.028604
10247	EBNA4	CCDC151	0.045157
10248	BLLF2	TCEAL2	0.016062
10249	BZLF1	SLC25A4	0.086576
10250	BDLF1	REPS2	0.033227
10251	EBNA4	C2orf78	0.14576
10252	BMLF1	SUMF2	0.059338
10253	EBNA-LP	PEX6	0.28219
10254	BZLF1	ADAMTSL4	0.12678
10255	EBNA-LP	ERC1	0.18662
10256	gL	SZRD1	0.055656
10257	EBNA3	DDOST	0.24689
10258	BTRF1	FIP1L1	0.031498
10259	BGLF4	KCNJ9	0.21212
10260	BLLF2	UBLCP1	0.031354
10261	BGLF4	UFL1	0.14244
10262	LMP1	CLIC4	0.027066
10263	BZLF1	SNRPB	0.10746
10264	BGLF4	TIPIN	0.14725
10265	EBNA4	COMMD5	0.24091
10266	BZLF1	MAST1	0.073689
10267	BMLF1	CSTF2	0.31927
10268	BMLF1	BRE	0.28591

10269	EBNA-LP	JPH2	0.24067
10270	BZLF1	POLB	0.053329
10271	BTRF1	ATCAY	0.01012
10272	BGLF4	SLX4	0.15948
10273	BMLF1	OBP2B	0.011448
10274	BMLF1	LARP4	0.037432
10275	EBNA3	TRPC3	0.21964
10276	EBNA1	PTGER4	0.07028
10277	BZLF1	RTF1	0.09167
10278	EBNA6	OCEL1	0.024665
10279	BMLF1	NLRP3	0.21033
10280	EBNA1	ATRX	0.087707
10281	BVRF1	S100A1	0.084239
10282	LMP2	S100A4	0.11429
10283	BGLF4	THRB	0.026957
10284	EBNA3	SYTL1	0.32188
10285	BALF2	ANXA2R	0.033194
10286	EBNA-LP	DCTN3	0.13943
10287	EBNA-LP	PPP2CB	0.12446
10288	BGRF1/BDRF1	RET	0.078408
10289	EBNA3	ANKLE2	0.26752
10290	EBNA4	PGBD4	0.10261
10291	EBNA3	BYSL	0.28369
10292	BZLF1	UBXN1	0.13486
10293	BGRF1/BDRF1	MEGF6	0.2126
10294	EBNA1	PARPBP	0.1685
10295	BOLF1	FAM47B	0.019193
10296	EBNA-LP	NSUN5P1	0.066347
10297	BRRF1	MACF1	0.13945
10298	EBNA-LP	IL4	0.058798
10299	EBNA1	EEA1	0.13284
10300	gL	CCDC171	0.065781
10301	EBNA-LP	FAAP20	0.27608
10302	BMLF1	SMARCA2	0.12003
10303	BMLF1	ZCCHC11	0.25011
10304	EBNA3	SPC25	0.25895
10305	BHRF1	LCHN	0.032704
10306	EBNA-LP	RORA	0.10721
10307	EBNA1	YWHAH	0.090963
10308	BZLF2	FAM219B	0.01739
10309	BVRF1	NUCB2	0.10941
10310	BMLF1	DCSTAMP	0.22111
10311	BMLF1	NUDCD3	0.019892
10312	gN	PRSS2	0.11945
10313	BARF1	PLSCR3	0.18033
10314	BPLF1	GAGE12B	0.037608
10315	EBNA3	EIF4H	0.3683
10316	BTRF1	C6orf10	0.026628
10317	BHRF1	KIAA1841	0.081951
10318	BGLF4	AP2A1	0.1002
10319	BMLF1	HOXA1	0.043529
10320	EBNA2	PPP2R3A	0.076975
10321	BGLF4	EIF4A2	0.16009
10322	EBNA3	HSPA14	0.33875
10323	BMLF1	VDR	0.11703
10324	EBNA6	C11orf65	0.051229
10325	BZLF1	PPM1B	0.098826
10326	EBNA-LP	PROX1	0.025749
10327	EBNA-LP	MMP20	0.4123
10328	EBNA-LP	NELFE	0.27882
10329	BZLF1	MYL4	0.048053
10330	BLLF1	CT45A5	0.010012
10331	EBNA-LP	WNK3	0.20016
10332	EBNA3	TRAF5	0.21338
10333	EBNA6	MEGF6	0.18248
10334	EBNA-LP	TGFB3	0.054343
10335	EBNA6	KAT2A	0.01128
10336	BMLF1	RPGRIP1	0.36477

10337	BILF1	LDLR	0.050338
10338	BMLF1	SLC6A8	0.01557
10339	EBNA3	INPP5K	0.05651
10340	BMLF1	NDUFB10	0.36665
10341	EBNA1	PGR	0.017136
10342	EBNA-LP	RRAGD	0.29514
10343	BMLF1	FLNA	0.097241
10344	BBRF2	GALNT2	0.021481
10345	EBNA3	TLE3	0.28371
10346	EBNA3	RUNX1T1	0.14449
10347	EBNA2	SPHK2	0.013842
10348	BMLF1	RBM18	0.039667
10349	EBNA4	CEP57	0.10055
10350	BGLF4	NUFIP2	0.25913
10351	BVRF1	C2CD4D	0.020162
10352	BLLF1	C1orf64	0.043447
10353	EBNA6	SLAIN1	0.25929
10354	BMLF1	STT3A	0.35635
10355	EBNA3	CTNNBIP1	0.22893
10356	BZLF1	GADD45A	0.14433
10357	BGLF4	USP7	0.075099
10358	BGLF4	ANKRD27	0.19659
10359	BGLF4	VPS53	0.20143
10360	gN	NOTCH4	0.019468
10361	EBNA6	C16orf93	0.082286
10362	EBNA1	EMR2	0.016446
10363	EBNA-LP	FAM83F	0.041183
10364	BZLF1	OGG1	0.056605
10365	BZLF1	UPF1	0.10599
10366	BZLF2	MAB21L3	0.015577
10367	EBNA1	FCGBP	0.2091
10368	BLLF2	SF1	0.011842
10369	BZLF1	CDC73	0.09886
10370	BPLF1	C10orf91	0.13409
10371	BBRF2	ARSB	0.012391
10372	EBNA3	XRCC6	0.050982
10373	BGLF4	BIRC3	0.10636
10374	BMLF1	OGDH	0.010687
10375	EBNA6	MNCb-2875	0.063092
10376	BGLF4	PTPRN	0.11408
10377	BZLF1	MIF4GD	0.20396
10378	EBNA-LP	RABGAP1	0.26627
10379	BZLF1	TFAP2C	0.013545
10380	BARF1	TFB1M	0.047438
10381	BGLF4	RCVRN	0.077134
10382	BLLF2	GALNT15	0.019731
10383	BGLF4	AGR2	0.25349
10384	BGLF4	ABCD2	0.13919
10385	gM	FAT4	0.089228
10386	EBNA-LP	LENG8	0.43184
10387	BGLF4	RRM1	0.13052
10388	EBNA3	APITD1	0.19488
10389	EBNA3	CETN1	0.50359
10390	EBNA1	SOS1	0.045495
10391	LMP1	JAG2	0.052621
10392	BMLF1	CELA3A	0.047985
10393	EBNA3	CHRD	0.21426
10394	EBNA-LP	NUP107	0.20957
10395	EBNA4	EXOC3L4	0.14422
10396	EBNA3	NELFCD	0.25987
10397	EBNA3	RORA	0.10707
10398	BLLF1	JAG2	0.070176
10399	BBRF2	TBRG4	0.038362
10400	gL	C16orf45	0.040328
10401	EBNA3	USP13	0.17083
10402	BGLF4	SLAMF1	0.13458
10403	EBNA1	CSE1L	0.16357
10404	BZLF1	EPHA10	0.15083

BMLF1	FKBP1A	0.17654
EBNA-LP	VWCE	0.20358
BMLF1	VPS13A	0.33987
BMLF1	S100A12	0.34275
BMLF1	SLC17A8	0.049567
BTRF1	TRAPPC1	0.022825
BMLF1	KMT2D	0.24124
BZLF1	AIRE	0.043948
BGLF4	ERLIN1	0.1949
BPLF1	ANXA7	0.060324
EBNA-LP	KRT20	0.29783
EBNA3	KLK6	0.25751
EBNA1	RNASE1	0.18256
BGLF4	TOX3	0.17796
EBNA3	PLEKHA1	0.13202
BLLF2	TMEM132A	0.066796
BGLF4	HIF1AN	0.10562
BPLF1	PAGE3	0.054555
BMLF1	CELSR2	0.11965
BLLF2	GVINP1	0.010561
BGLF4	BCL2L1	0.013372
BZLF2	TEX36	0.030194
BGLF4	CTNNA2	0.081615
BMLF1	BRD3	0.29953
BXRF1	STAT1	0.01824
BGLF4	NLGN3	0.087351
BZLF1	MORC4	0.16567
BZLF1	RCHY1	0.072412
EBNA1	RND2	0.17235
BLLF2	AATF	0.01779
BGLF4	DZIP3	0.18431
BMLF1	EMG1	0.049507
EBNA-LP	PSMD10	0.084429
BLLF2	PAPSS2	0.010328
BGLF4	WNK3	0.059946
EBNA3	PIK3R1	0.016644
EBNA3	CASR	0.26067
BGLF4	PIAS4	0.099354
BMLF1	INPP5D	0.13266
EBNA-LP	SOX4	0.043505
EBNA3	RBBP7	0.1991
BZLF1	ADAM12	0.063928
BLLF2	MKI67	0.050296
BMLF1	RHCG	0.04299
BMLF1	NUCB2	0.45939
EBNA1	PEX10	0.14104
EBNA4	PIR	0.19303
BZLF1	KRT6A	0.14562
BGLF4	NECAB2	0.45159
EBNA3	PALM	0.18477
EBNA3	LAT2	0.33285
EBNA6	CCDC67	0.031238
EBNA1	OAS3	0.11858
gM	S100A1	0.047517
EBNA6	ATXN7L2	0.072672
BLLF2	EDAR	0.03449
EBNA6	SMEK3P	0.10643
BGLF4	PPP2R5E	0.14904
BGLF4	PCDHA9	0.058173
EBNA4	PDCD4	0.11792
BLLF2	CLDN23	0.02374
EBNA1	MKL1	0.11399
EBNA3	CDH1	0.10432
EBNA1	CALML5	0.033943
BMLF1	KIAA1279	0.39845
BILF1	PIK3CB	0.011643
BGLF4	SMG1	0.058744
BHRF1	EFEMP2	0.15529

10473	EBNA1	BCL6	0.015462
10474	BMLF1	HSD17B12	0.21184
10475	BMLF1	SEC13	0.2145
10476	EBNA3	RAB11A	0.18365
10477	BMLF1	TTC3	0.2473
10478	EBNA1	PYCARD	0.019909
10479	EBNA-LP	MYH10	0.11856
10480	EBNA4	S100A13	0.16668
10481	EBNA1	PEX11G	0.1698
10482	EBNA6	GTF2E1	0.14888
10483	EBNA1	DROSHA	0.11572
10484	EBNA2	CRELD2	0.12683
10485	EBNA-LP	RBFOX2	0.25094
10486	EBNA3	TUBGCP2	0.23627
10487	BLLF2	ZNF791	0.011346
10488	BZLF1	RAB7A	0.10693
10489	BMLF1	DCC	0.21112
10490	BMLF1	CLIC6	0.064251
10491	EBNA-LP	TREML1	0.33273
10492	EBNA4	MIR7-3HG	0.057795
10493	LMP2	EPS15	0.029949
10494	BNLF2b	SLC18A1	0.015603
10495	EBNA3	MSN	0.12041
10496	BGLF4	MYC	0.018984
10497	BNLF2b	KCNJ11	0.013183
10498	EBNA3	SERPINE1	0.068328
10499	BZLF2	CCSER1	0.071082
10500	EBNA6	CCDC174	0.02368
10501	BMLF1	CCT2	0.26983
10502	EBNA1	NPBWR2	0.15515
10503	BZLF1	ARHGAP17	0.16037
10504	BZLF1	PEX26	0.16425
10505	BARF1	AP1M1	0.037393
10506	BZLF2	GOLGA6L19	0.064501
10507	BGLF4	SNRNP40	0.15952
10508	EBNA1	SEC31A	0.11585
10509	EBNA4	YAL042C-A	0.063481
10510	EBNA3	KIF1B	0.2896
10511	BGLF4	COL1A1	0.043108
10512	EBNA1	SSTR2	0.082703
10513	BZLF1	ZFYVE26	0.18206
10514	BHRF1	C12orf57	0.039323
10515	BALF2	PLSCR3	0.11075
10516	EBNA6	S100A4	0.17763
10517	BZLF1	CDC5L	0.16587
10518	BZLF1	MST1R	0.10216
10519	BMLF1	PSMC5	0.11215
10520	LMP2	MICU1	0.079321
10521	EBNA1	DSN1	0.13158
10522	BMLF1	PACS1	0.32969
10523	EBNA3	GBAS	0.36858
10524	EBNA6	FAM195B	0.089305
10525	EBNA3	TRIM44	0.35796
10526	BRRF1	PPEF2	0.099318
10527	BMLF1	ALDH3A1	0.062188
10528	EBNA3	MAPK1	0.015437
10529	BLLF1	LYRM9	0.04433
10530	BZLF1	IFRD2	0.22358
10531	EBNA3	SPOCK2	0.1873
10532	BILF1	ATP5F1	0.036735
10533	EBNA1	MPZL2	0.16926
10534	BVRF1	S100A13	0.13661
10535	EBNA-LP	RNF6	0.18185
10536	BGLF4	OCRL	0.09983
10537	EBNA-LP	PLK3	0.10285
10538	BBRF2	OR10H5	0.030153
10539	EBNA3	ARPC4	0.3012
10540	gL	CXorf30	0.11051

10541	BMLF1	BAZ2B	0.30074
10542	BMLF1	CAPS	0.20661
10543	EBNA3	CCDC87	0.016873
10544	BMLF1	ABHD17B	0.045183
10545	BMLF1	TUBG1	0.22093
10546	EBNA-LP	SEPT5	0.12834
10547	EBNA-LP	PSIP1	0.22468
10548	EBNA-LP	FANCG	0.26029
10549	EBNA-LP	COG3	0.2189
10550	EBNA1	IKBKE	0.060227
10551	EBNA1	ASPSCR1	0.14463
10552	EBNA-LP	CRH	0.16574
10553	EBNA3	F2RL1	0.052528
10554	EBNA1	KCNMA1	0.078841
10555	BLLF2	ZNF76	0.010662
10556	BLLF2	FBXO5	0.010782
10557	EBNA-LP	AGR2	0.43178
10558	BZLF1	CINP	0.19957
10559	BARF1	EDEM1	0.11485
10560	BOLF1	LINC00205	0.01418
10561	EBNA1	SMAD2	0.013744
10562	EBNA3	SLA2	0.33466
10563	BALF2	LRCOL1	0.037951
10564	BMLF1	PRRC2A	0.41608
10565	BBRF2	OR2T7	0.033291
10566	BGLF4	F3	0.054299
10567	EBNA1	XRCC4	0.076839
10568	BGLF4	PMS2	0.10753
10569	EBNA1	AP1G2	0.17734
10570	BALF2	DTNA	0.051482
10571	EBNA3	E2F5	0.10547
10572	BBRF2	G4	0.047365
10573	EBNA3	PRPF8	0.32128
10574	EBNA4	IFIT2	0.13878
10575	EBNA3	PRPF3	0.33568
10576	BBRF2	SCGB3A1	0.058536
10577	EBNA1	TJP1	0.12867
10578	BMLF1	KLK5	0.39469
10579	EBNA6	DAZAP2	0.16014
10580	BGLF4	MYL6B	0.3487
10581	BHRF1	PLAC8L1	0.035027
10582	BGLF4	NCOA2	0.04695
10583	EBNA4	ZWINT	0.088813
10584	EBNA3	TBC1D8B	0.15086
10585	BZLF1	APH1B	0.17225
10586	BZLF1	ZBTB25	0.033876
10587	EBNA6	FAM127B	0.14561
10588	BMLF1	MATN4	0.69673
10589	BALF2	N4BP2L1	0.011138
10590	BLLF2	DBF4	0.017907
10591	BALF2	FBN1	0.13764
10592	BMLF1	TRAK2	0.22467
10593	EBNA6	ZDHHC17	0.012692
10594	EBNA1	SULT2A1	0.16084
10595	EBNA4	C7orf57	0.080476
10596	EBNA1	ITM2C	0.13964
10597	BLLF2	ZNF355P	0.019687
10598	BMLF1	SMG9	0.35321
10599	BLLF1	RNH1	0.094001
10600	EBNA-LP	TGFBR1	0.016714
10601	BLLF2	IFRD1	0.058764
10602	BLLF2	HIST1H1C	0.01566
10603	BGLF4	CUL2	0.14809
10604	EBNA1	SYNE1	0.11241
10605	BLLF2	IP6K3	0.042011
10606	BMLF1	PIWIL1	0.30147
10607	BMLF1	XPO7	0.018779
10608	EBNA1	UBQLN2	0.17347

10609	BGLF4	BTRC	0.079225
10610	gB	CCAR1	0.073375
10611	BGLF4	YWHAE	0.12562
10612	EBNA3	MMP2	0.11969
10613	EBNA3	MMP17	0.049865
10614	EBNA1	SETD1A	0.19126
10615	EBNA1	SMYD2	0.12855
10616	BMLF1	VAMP7	0.15948
10617	EBNA6	RPS2	0.042392
10618	BGLF4	CDON	0.095972
10619	EBNA-LP	SYNM	0.27583
10620	BBRF2	CRTC3	0.028742
10621	EBNA4	PEF1	0.22299
10622	LMP2	CHP1	0.046003
10623	EBNA6	PPP4R2	0.19736
10624	EBNA-LP	IGF2R	0.23165
10625	EBNA-LP	NOSTRIN	0.37823
10626	BBRF2	MEN1	0.017637
10627	BHRF1	DTNA	0.069701
10628	BMLF1	MBD3	0.25634
10629	EBNA-LP	DGKB	0.1864
10630	BALF2	CETN2	0.11301
10631	BGLF4	STAT6	0.17573
10632	BZLF1	NIFK	0.18844
10633	BBRF2	EMID1	0.02927
10634	BZLF1	PTGES3	0.09678
10635	BGRF1/BDRF1	FASN	0.070561
10636	BPLF1	C20orf195	0.032553
10637	EBNA-LP	TRPC6	0.21559
10638	EBNA-LP	ANXA2R	0.084037
10639	EBNA3	LCP1	0.11643
10640	EBNA1	HSPA9	0.098328
10641	BZLF1	DES	0.10938
10642	BPLF1	PNPT1	0.038132
10643	BMLF1	CALCOCO1	0.22861
10644	BMLF1	ARHGEF11	0.1432
10645	EBNA6	EID2	0.14155
10646	EBNA3	MBL2	0.20833
10647	LMP1	LTBP4	0.068735
10648	BGLF4	AURKAIP1	0.1633
10649	BBRF2	SMYD5	0.038808
10650	BMLF1	BACE1	0.10775
10651	EBNA1	CBFA2T2	0.021123
10652	EBNA4	CT45A2	0.097421
10653	BLLF2	OR2M3	0.07986
10654	gL	CCDC138	0.013255
10655	BGLF4	NOX5	0.04823
10656	BGRF1/BDRF1	NUCB2	0.13861
10657	BMLF1	MARK3	0.28797
10658	EBNA3	KRT17	0.22863
10659	EBNA3	SORBS3	0.20239
10660	BXRF1	CDH23	0.053446
10661	BNLF2b	KCNK1	0.013951
10662	BMLF1	PUS7L	0.12827
10663	BMLF1	RBM11	0.010387
10664	BXRF1	N6AMT1	0.02191
10665	BMLF1	ANTXR2	0.33067
10666	EBNA-LP	RTKN	0.23678
10667	EBNA-LP	KIR2DL3	0.28537
10668	EBNA2	WDR96	0.018729
10669	BMLF1	EIF3H	0.4086
10670	BLLF2	SUPT6H	0.01409
10671	EBNA3	RHOA	0.043191
10672	BGLF4	AMER2	0.22694
10673	BHRF1	MMP20	0.03676
10674	BMLF1	VCAN	0.13137
10675	BLLF1	ANGEL1	0.016307
10676	EBNA6	TNFRSF12A	0.093368

10677	EBNA-LP	LAMP5	0.053428
10678	EBNA3	TNIP2	0.11203
10679	EBNA3	WDR6	0.33982
10680	EBNA1	SPTBN4	0.038938
10681	EBNA1	PKD1L2	0.040498
10682	BGLF4	TIA1	0.18418
10683	EBNA-LP	IFIT1	0.30528
10684	BGLF4	EGR2	0.063109
10685	BMLF1	SSH1	0.11786
10686	BZLF1	CTU2	0.19692
10687	EBNA-LP	ADRM1	0.26739
10688	BGLF4	SFTPD	0.18281
10689	EBNA3	LIG4	0.072443
10690	BLLF2	TCP10	0.078998
10691	BLLF2	ZNF165	0.014013
10692	BGLF4	PDZD3	0.12173
10693	BGLF4	GZMA	0.16997
10694	gL	PPP2R3A	0.024557
10695	EBNA-LP	NR5A2	0.046277
10696	BGRF1/BDRF1	EPS15	0.080698
10697	EBNA3	GTF2H2	0.032579
10698	BMLF1	BLOC1S3	0.23367
10699	EBNA-LP	DMBT1	0.16215
10700	EBNA3	ANGEL1	0.039888
10701	BGLF4	PSMD4	0.10394
10702	EBNA3	VIL1	0.26254
10703	BZLF2	CCDC7	0.030799
10704	BZLF1	NT5C2	0.071829
10705	BGLF4	PPP2R5D	0.15432
10706	EBNA1	TOMM20	0.08977
10707	BMLF1	SYT12	0.054739
10708	BZLF1	CCS	0.096746
10709	EBNA6	CKB	0.081341
10710	EBNA3	PCDHA6	0.16404
10711	BMLF1	DCTN2	0.23871
10712	BBRF2	SBSPON	0.010588
10713	BMLF1	SLC25A13	0.14468
10714	EBNA1	CACNB4	0.071355
10715	EBNA4	PNMAL2	0.20356
10716	EBNA6	BOP	0.021556
10717	BZLF1	UBA2	0.014423
10718	BLLF2	KDM3A	0.013323
10719	BGLF4	NDUFAF1	0.20007
10720	BILF1	LRRC14	0.010992
10721	BPLF1	FAM195B	0.028833
10722	BMLF1	CROCC	0.2364
10723	BGLF4	PROCA1	0.058753
10724	EBNA4	GOLGA6L20	0.14208
10725	EBNA3	NCOR1P1	0.011672
10726	BMLF1	ZHX2	0.14254
10727	BLLF2	TNFSF18	0.088453
10728	BMLF1	PMP22	0.35406
10729	BGLF4	SMOC2	0.04442
10730	BGLF4	TJP1	0.13542
10731	BGLF4	PREX1	0.060408
10732	EBNA3	IL8	0.17508
10733	EBNA-LP	EXOSC3	0.25563
10734	BGLF4	SPRY2	0.04235
10735	BZLF1	CDK5RAP2	0.06237
10736	BGLF4	PPIH	0.17536
10737	EBNA3	BACE1	0.063766
10738	BZLF1	GAS6	0.033144
10739	EBNA-LP	DEAF1	0.19421
10740	BMLF1	SUPT20H	0.385
10741	BGLF4	IL13	0.10125
10742	BGLF4	BICD2	0.23597
10743	BMLF1	TERT	0.20931
10744	BLLF2	ARMCX6	0.074472

10745	BMLF1	FPR1	0.27761
10746	BLLF2	DPP6	0.012016
10747	BMLF1	TAL1	0.048242
10748	BZLF1	ATP6V0A2	0.14057
10749	BMLF1	MYO1D	0.01601
10750	BGLF4	RELB	0.087729
10751	BMLF1	MAP3K8	0.19744
10752	EBNA4	NELL1	0.12165
10753	BZLF1	MLKL	0.088753
10754	BGLF4	TRAM1	0.15352
10755	BHRF1	PCSK2	0.032462
10756	BMLF1	STK25	0.22731
10757	BMLF1	IKBKE	0.15904
10758	BLLF2	ZNF324	0.014585
10759	EBNA-LP	AGTRAP	0.36326
10760	EBNA3	PATL2	0.34341
10761	BMLF1	ZNF133	0.18958
10762	EBNA4	ADA	0.022778
10763	EBNA1	CCNB1	0.032786
10764	EBNA3	NACA	0.034461
10765	EBNA1	ASCL1	0.010998
10766	EBNA1	TESC	0.20467
10767	BZLF1	DYNLT1	0.094937
10768	EBNA3	SETMAR	0.22949
10769	BVRF1	CCDC47	0.13539
10770	EBNA1	RAPGEF4	0.066743
10771	BDLF1	VLDLR	0.029238
10772	gL	C16orf93	0.070099
10773	BTRF1	FAM210A	0.04222
10774	BMLF1	USH1C	0.23205
10775	BMLF1	PIM1	0.13705
10776	EBNA6	EPSTI1	0.017298
10777	BMLF1	RFX6	0.2454
10778	EBNA1	ARPC2	0.1241
10779	BLLF2	CRYGB	0.020753
10780	BVRF1	REPS2	0.17774
10781	EBNA1	ZNF703	0.074464
10782	EBNA-LP	PTPRT	0.10495
10783	BMLF1	SMAD4	0.031278
10784	EBNA3	TGFBR2	0.038627
10785	EBNA3	PDE4DIP	0.31419
10786	BGLF4	TBC1D7	0.21054
10787	EBNA-LP	ASXL1	0.18245
10788	BHRF1	RILP	0.09377
10789	EBNA2	S100B	0.053821
10790	BPLF1	C20orf96	0.017383
10791	BGLF4	AKT1S1	0.12043
10792	BMLF1	PGM3	0.031874
10793	EBNA3	SMARCA4	0.11425
10794	BMLF1	EXOC2	0.36582
10795	BLLF2	ELOVL3	0.05608
10796	BBRF2	MAGEB6	0.014725
10797	BMLF1	FHL2	0.11536
10798	EBNA3	LPL	0.097464
10799	BZLF1	WWP2	0.050706
10800	BHRF1	KIAA0087	0.083708
10801	BMLF1	RAD51C	0.24072
10802	BLLF2	TMPO	0.064947
10803	BZLF1	NFKBIB	0.099484
10804	EBNA3	SNRNP70	0.32192
10805	BHRF1	MMP15	0.020862
10806	EBNA3	ARRDC3	0.3995
10807	BLLF2	EDEM1	0.012645
10808	BZLF1	UTF1	0.12862
10809	BZLF1	WRNIP1	0.12835
10810	BGLF4	ATG5	0.14751
10811	BILF1	CDH23	0.061914
10812	EBNA-LP	RAB14	0.086283

10813	EBNA-LP	SCAMP1	0.31951
10814	EBNA1	ORC4	0.084151
10815	EBNA1	PCDHGA12	0.038413
10816	EBNA1	F9	0.075093
10817	BHRF1	HUS1B	0.078618
10818	BLLF2	TRO	0.025747
10819	BGLF4	HIST1H3A	0.12599
10820	BMLF1	TLK2	0.23309
10821	EBNA-LP	DLL1	0.33925
10822	gL	FAM188B	0.20972
10823	EBNA6	AHSA1	0.13166
10824	EBNA1	PTPN9	0.049768
10825	EBNA3	MAN1B1	0.091369
10826	BBRF2	PBOV1	0.021356
10827	gL	CLIP1	0.020798
10828	EBNA3	COPS5	0.2392
10829	EBNA4	LINC00478	0.082437
10830	BMLF1	PSMA7	0.10317
10831	EBNA3	SPARCL1	0.20236
10832	EBNA-LP	KCTD5	0.32665
10833	BDLF1	SYT11	0.10887
10834	BGLF4	HFE	0.15352
10835	EBNA-LP	DSCAM	0.26336
10836	EBNA1	DLC1	0.064392
10837	BZLF1	HYAL2	0.041115
10838	EBNA1	CAPN2	0.21173
10839	BLLF2	LYRM1	0.011036
10840	EBNA-LP	ELF4	0.1592
10841	BNLF2b	SDS	0.023142
10842	BPLF1	CETN2	0.11216
10843	BZLF1	HS3ST5	0.15398
10844	EBNA4	FAM166B	0.05565
10845	BMLF1	GPRC5C	0.11016
10846	BGRF1/BDRF1	DLL1	0.15917
10847	BGLF4	MRM1	0.043747
10848	EBNA3	TRIM23	0.078889
10849	BMLF1	AGTR1	0.13349
10850	BGLF4	FZD3	0.094567
10851	EBNA1	BAIAP2	0.078205
10852	EBNA1	LAMTOR3	0.15197
10853	BMLF1	PTAR1	0.042745
10854	BMLF1	NKX3-1	0.029803
10855	BZLF1	VCAN	0.031294
10856	BMLF1	ALPK1	0.018992
10857	BZLF1	CEP290	0.10329
10858	BARF1	STAT6	0.022034
10859	EBNA-LP	CNIH2	0.22644
10860	BGLF4	LONP1	0.095888
10861	EBNA3	MRPS31	0.32493
10862	EBNA1	PLK2	0.060951
10863	EBNA3	SNRPB2	0.29309
10864	EBNA3	RHNO1	0.3432
10865	BBRF2	SPATA31A6	0.012426
10866	EBNA1	TF	0.12302
10867	EBNA1	IL1A	0.08115
10868	BDLF1	NKD1	0.017366
10869	BGLF4	DOK1	0.09503
10870	BZLF1	RCC1	0.10697
10871	BLLF2	CC2D1B	0.012058
10872	EBNA1	UTRN	0.23879
10873	BBRF2	ALKBH4	0.027206
10874	BILF1	ATP2B2	0.015516
10875	EBNA6	KIAA1841	0.12985
10876	EBNA-LP	USE1	0.26941
10877	EBNA3	TIMP1	0.2016
10878	EBNA-LP	MMP14	0.12426
10879	BZLF1	NSMCE2	0.1069
10880	BZLF1	SLC25A25	0.052515

10881	EBNA3	KIF24	0.3255
10882	BLLF2	TRAPPC2L	0.019348
10883	BLLF2	GOLGA6B	0.061779
10884	BGRF1/BDRF1	NID2	0.21192
10885	BZLF1	FLT3	0.093354
10886	BZLF1	CRY2	0.061528
10887	EBNA6	YTHDF1	0.10991
10888	BHRF1	C2CD4D	0.025069
10889	BZLF1	MPHOSPH6	0.2162
10890	BZLF1	TARDBP	0.067751
10891	EBNA1	CXorf67	0.025675
10892	EBNA-LP	PKN2	0.094714
10893	BXRF1	EML1	0.17052
10894	EBNA-LP	LSM3	0.29909
10895	EBNA-LP	DNA2	0.010828
10896	EBNA1	LRP8	0.24232
10897	EBNA1	PPEF2	0.22857
10898	BLLF2	ZNF75CP	0.01533
10899	BMLF1	CISD2	0.03038
10900	EBNA-LP	ZHX3	0.14002
10901	BMLF1	FOXG1	0.078561
10902	BARF1	DTNA	0.11107
10903	EBNA-LP	ARHGEF11	0.11459
10904	BLLF2	HSPA9	0.029269
10905	BMLF1	C11orf72	0.027007
10906	EBNA1	NPB	0.17753
10907	BZLF1	VPS35	0.14333
10908	BBRF2	TMED9	0.012237
10909	EBNA3	FCHSD2	0.43795
10910	BGLF4	ESRRA	0.054355
10911	EBNA4	WDR63	0.010729
10912	BGLF4	RCOR1	0.1233
10913	BGRF1/BDRF1	CNGC19	0.011582
10914	EBNA-LP	TFG	0.34078
10915	EBNA1	PROZ	0.01374
10916	BLLF2	DAD1	0.071155
10917	BMLF1	BAD	0.056855
10918	gL	SDF4	0.051243
10919	EBNA1	EPB41L3	0.073634
10920	BALF2	IL6R	0.02075
10921	BLLF2	LVP1a	0.11612
10922	BGLF4	ELAVL1	0.11366
10923	BGLF4	ZFYVE9	0.20751
10924	BMLF1	ARPP21	0.010482
10925	BGLF4	CLK2	0.052324
10926	EBNA6	NANOS1	0.098223
10927	BMLF1	RPS3A	0.24903
10928	BZLF1	CASP7	0.084082
10929	BGLF4	EPAS1	0.024022
10930	EBNA-LP	DCAF7	0.34037
10931	EBNA1	MOB1A	0.18257
10932	BGLF4	CDHR2	0.36346
10933	BGLF4	RYBP	0.12535
10934	gL	ANKRD26P1	0.010385
10935	BZLF1	MKL2	0.13505
10936	BZLF1	GABARAP	0.1443
10937	EBNA3	MARK4	0.18923
10938	BZLF1	LSM2	0.068096
10939	BHRF1	C17orf66	0.024814
10940	EBNA-LP	IFT20	0.29539
10941	BHRF1	C18orf25	0.062588
10942	EBNA3	XAGE3	0.17318
10943	BPLF1	FAM84A	0.036733
10944	BTRF1	EXOC1	0.010991
10945	BMLF1	NOD2	0.018517
10946	BMLF1	UTP14A	0.37372
10947	BMLF1	FKBP14	0.20367
10948	BMLF1	PCGF1	0.27527

10949	EBNA4	C16orf11	0.034629
10950	EBNA3	PRICKLE1	0.21414
10951	BHRF1	ERICH1-AS1	0.10145
10952	BRRF1	LRP1	0.05337
10953	BMLF1	HSPB2	0.35667
10954	EBNA3	LTBP3	0.15541
10955	EBNA3	DOCK4	0.23603
10956	BGLF4	MAPK9	0.033129
10957	BMLF1	PDLIM7	0.27648
10958	BPLF1	GAGE12G	0.037608
10959	EBNA1	RNF103	0.11049
10960	BZLF1	STAP1	0.045407
10961	BGLF4	CNOT3	0.15476
10962	BZLF1	GRB2	0.015688
10963	EBNA-LP	ARHGAP32	0.15679
10964	EBNA3	THBS1	0.06533
10965	BGLF4	GDF2	0.087822
10966	EBNA1	SERBP1	0.22042
10967	BMLF1	SHROOM2	0.17527
10968	EBNA4	EIF2B2	0.037439
10969	EBNA-LP	RANBP9	0.27804
10970	EBNA3	PARP9	0.45012
10971	gL	HEATR5A	0.067656
10972	BZLF1	RHBDD1	0.12885
10973	EBNA3	NFATC1	0.10942
10974	BGLF4	EGFL6	0.060076
10975	BOLF1	LTBP4	0.13809
10976	BLLF2	MAP2	0.01105
10977	BZLF1	ZXDC	0.028952
10978	EBNA2	S100A13	0.10908
10979	BGLF4	RASGRP1	0.021712
10980	BZLF1	PCDHB15	0.046144
10981	BMLF1	ITGA1	0.24531
10982	EBNA1	PDLIM7	0.12136
10983	EBNA-LP	KIF24	0.32149
10984	LMP2	NSUN5P1	0.044887
10985	BMLF1	CLCF1	0.26093
10986	BZLF2	C1orf111	0.053408
10987	BBRF2	OR2J1	0.030153
10988	BMLF1	OCA2	0.22587
10989	EBNA-LP	VPS41	0.22701
10990	EBNA4	SLC22A5	0.019423
10991	BGLF4	ASGR1	0.18072
10992	EBNA1	DGKI	0.15834
10993	EBNA1	ULK3	0.078111
10994	EBNA3	GOLGA8CP	0.17213
10995	BZLF1	NOS1	0.086312
10996	BLLF2	PMS2	0.021595
10997	BBRF2	LGALS9	0.014359
10998	EBNA3	YJR030C	0.091133
10999	BGLF4	PSMC1	0.069699
11000	EBNA-LP	MERTK	0.1539
11001	BZLF2	CT45A4	0.01869
11002	BDLF1	TNNC1	0.042134
11003	EBNA-LP	PFKL	0.19944
11004	BGLF4	MRPL12	0.22951
11005	BRRF1	CETN1	0.20514
11006	BMLF1	SLC12A5	0.01693
11007	BMLF1	S100A11	0.5377
11008	EBNA3	SELP	0.12371
11009	BLLF2	IFNA17	0.013659
11010	BGLF4	RYR3	0.030258
11011	EBNA1	RRAS2	0.094163
11012	LMP2	NKD1	0.075861
11013	BMLF1	MARK2	0.15565
11014	BMLF1	DCK	0.086258
11015	EBNA4	PSRC1	0.16597
11016	BPLF1	C19orf35	0.085104

11017	BLLF1	PAGE2B	0.058996
11018	EBNA3	STBD1	0.36343
11019	BGLF4	SKIV2L2	0.10592
11020	BZLF1	AP2A2	0.13106
11021	EBNA-LP	PIM1	0.10484
11022	BLLF1	CCDC17	0.011177
11023	BZLF1	PTRF	0.11115
11024	BMLF1	OVCH2	0.02953
11025	BPLF1	PACRGL	0.023825
11026	EBNA3	PITPNC1	0.3992
11027	BZLF1	ZNF224	0.10955
11028	EBNA-LP	TRPC3	0.21676
11029	EBNA4	TRAF3IP1	0.29174
11030	BLLF2	ESM1	0.018563
11031	EBNA1	PRKX	0.063249
11032	BZLF1	FAM160A1	0.010711
11033	BGLF4	SPON1	0.22019
11034	EBNA-LP	ABCB1	0.21636
11035	BLLF2	G4	0.035696
11036	gL	GAGE12H	0.070023
11037	EBNA3	MMP24	0.051434
11038	BNLF2b	TNNI1	0.017666
11039	BMLF1	SEPT8	0.38931
11040	gM	PLSCR3	0.077207
11041	EBNA-LP	C10orf62	0.43184
11042	EBNA1	PDE3B	0.063737
11043	BZLF1	PPP4R4	0.22922
11044	EBNA3	NRXN1	0.2257
11045	EBNA-LP	ITGB4	0.1917
11046	EBNA1	LZTS1	0.028702
11047	BHRF1	CCDC159	0.079361
11048	EBNA-LP	WWC1	0.20417
11049	BLLF2	PEG3	0.013852
11050	BGLF4	CTBP1	0.022328
11051	EBNA3	E2F1	0.065605
11052	BZLF2	WIBG	0.069698
11053	BZLF2	FAM110D	0.076756
11054	EBNA-LP	PCDH12	0.14369
11055	EBNA3	ZSCAN21	0.1392
11056	BZLF1	ITGA3	0.16382
11057	BHRF1	SYT1	0.051799
11058	BZLF1	MBD4	0.093571
11059	BZLF1	POLR1B	0.10213
11060	BZLF1	POLDIP3	0.20572
11061	BMLF1	SEC23IP	0.02633
11062	BZLF1	PPP2R3A	0.33227
11063	BGLF4	IST1	0.28609
11064	BGLF4	CBLC	0.012014
11065	BMLF1	RBP1	0.035315
11066	EBNA1	ZZEF1	0.010597
11067	BGLF4	SRSF3	0.1846
11068	EBNA3	EGFR	0.027962
11069	BMLF1	LOXL2	0.11653
11070	EBNA1	MORC4	0.15296
11071	BZLF1	THBS2	0.39077
11072	EBNA1	PDS5B	0.13211
11073	EBNA3	NUDT14	0.38233
11074	BZLF2	CCDC152	0.011046
11075	EBNA-LP	PIK3R4	0.20113
11076	EBNA3	KCNN4	0.25623
11077	gM	NSUN5P1	0.035347
11078	EBNA1	PTPRS	0.036554
11079	EBNA4	SELO	0.25015
11080	EBNA1	COMMD2	0.22232
11081	BGLF4	NPBWR2	0.17298
11082	EBNA1	DNER	0.27613
11083	BMLF1	PXDN	0.059379
11084	BOLF1	VLDLR	0.072209

11085	BZLF1	FKBP2	0.18659
11086	EBNA-LP	LLGL1	0.091209
11087	EBNA3	FTSJ3	0.42193
11088	BBRF2	ZNF782	0.011579
11089	EBNA3	SLAIN2	0.38908
11090	EBNA3	CCS	0.1996
11091	BMLF1	MNS1	0.057292
11092	EBNA2	AP1G1	0.018311
11093	EBNA3	PADI6	0.12099
11094	EBNA1	DHH	0.28013
11095	BRRF1	ATP6V0A1	0.042735
11096	BGLF4	MCM7	0.081787
11097	EBNA1	TPCN2	0.026865
11098	BZLF1	NHP2L1	0.18368
11099	BRRF1	SETD2	0.028269
11100	EBNA-LP	DPP7	0.088575
11101	LMP1	S100A8	0.030993
11102	BZLF2	C19orf48	0.026981
11103	EBNA3	HIPK1	0.11463
11104	BLLF2	OR4N2	0.087261
11105	BLLF2	NIPBL	0.019115
11106	BMLF1	USP1	0.27957
11107	EBNA6	THBD	0.10882
11108	BMLF1	SLC25A23	0.21019
11109	BMLF1	ENDOU	0.095457
11110	gL	SYT14L	0.011006
11111	BGRF1/BDRF1	SH3KBP1	0.040629
11112	EBNA1	HDHD3	0.18304
11113	BGLF4	EFCAB14	0.068073
11114	EBNA1	RPS27L	0.12154
11115	EBNA1	ARHGAP10	0.055267
11116	BMLF1	ACADS	0.024509
11117	EBNA-LP	KIF7	0.28853
11118	BZLF1	TNS1	0.18531
11119	EBNA-LP	ARL6	0.14941
11120	BZLF1	HSPD1	0.030127
11121	BGRF1/BDRF1	ZC3HAV1L	0.012199
11122	BMLF1	MYH9	0.088904
11123	BGLF4	TPM4	0.036049
11124	EBNA6	C18orf42	0.064092
11125	BGLF4	BRD7	0.078074
11126	EBNA1	PLEKHA1	0.053456
11127	BARF1	RCN3	0.23
11128	BGLF4	KDR	0.016464
11129	BZLF2	NOTCH3	0.034265
11130	LMP1	GPR98	0.022129
11131	EBNA1	NR2F1	0.024485
11132	BMLF1	NUP37	0.19672
11133	BGLF4	GLI1	0.046561
11134	EBNA-LP	PACSIN2	0.017128
11135	BMLF1	DIS3L	0.33572
11136	EBNA-LP	KIFC3	0.071711
11137	BDLF1	S100A11	0.012102
11138	BZLF2	FAM74A4	0.019904
11139	BZLF1	RBM15B	0.15651
11140	BMLF1	PRKDC	0.088905
11141	BGLF4	TUBG1	0.10035
11142	EBNA-LP	ATP6V0C	0.21783
11143	EBNA-LP	TCHHL1	0.2099
11144	BLLF2	ZNF416	0.016813
11145	EBNA3	AGR2	0.43897
11146	BPLF1	MAGOH	0.010048
11147	LMP2	TLDC2	0.023998
11148	EBNA3	ATP5SL	0.017483
11149	EBNA1	CSRP3	0.069919
11150	BMLF1	DUS2	0.034331
11151	BGLF4	GOPC	0.10136
11152	BBRF2	EID1	0.034411

11153	EBNA1	GRN	0.12982
11154	EBNA3	CABP2	0.17515
11155	EBNA3	IKBKAP	0.13648
11156	BZLF1	AFG3L2	0.048051
11157	BGLF4	MAP4K4	0.063931
11158	EBNA-LP	ANXA8	0.16367
11159	BGLF4	NOS3	0.021904
11160	BGLF4	TAP1	0.062691
11161	BMLF1	CHST5	0.043467
11162	BGLF4	BIK	0.1374
11163	EBNA6	PPEF2	0.081518
11164	BGLF4	RBMY1B	0.21373
11165	EBNA1	ZNF148	0.016853
11166	BMLF1	BCAS3	0.4013
11167	EBNA-LP	FKBP9L	0.19197
11168	BGLF4	ZFP36	0.050708
11169	EBNA-LP	PRKD1	0.01567
11170	BLLF2	ZNF26	0.018272
11171	EBNA3	NLRP1	0.1863
11172	EBNA1	CDK5RAP2	0.053674
11173	BMLF1	PRSS42	0.049094
11174	EBNA-LP	THBS3	0.14799
11175	BLLF2	ETNK1	0.059758
11176	BZLF1	CHFR	0.096217
11177	BLLF1	CRELD2	0.11298
11178	BGLF4	ITGA5	0.084147
11179	BLLF2	IPO8	0.01907
11180	BMLF1	MMP11	0.10015
11181	BGLF4	IGF2BP1	0.078405
11182	EBNA-LP	SVEP1	0.17868
11183	EBNA3	TOMM22	0.24122
11184	EBNA3	UACA	0.2215
11185	EBNA-LP	NSMAF	0.35612
11186	EBNA4	CCDC104	0.086918
11187	EBNA-LP	ARHGEF16	0.14977
11188	EBNA3	FHL1	0.21047
11189	BLLF2	SPINK7	0.01137
11190	BGLF4	CCDC33	0.24432
11191	EBNA-LP	CLCN3	0.19
11192	EBNA-LP	DACH1	0.097245
11193	BMLF1	DNM3	0.16954
11194	BMLF1	SON	0.28429
11195	EBNA3	EFHC1	0.11432
11196	BMLF1	MAPK7	0.11644
11197	EBNA1	RNF6	0.074313
11198	EBNA1	USP32	0.036456
11199	BMLF1	MTERF	0.28932
11200	EBNA-LP	PARP1	0.11635
11201	EBNA4	GTF2E1	0.1946
11202	BLLF2	PROM2	0.014283
11203	EBNA3	SELPLG	0.25978
11204	EBNA3	CDKN3	0.10826
11205	EBNA6	FAM46D	0.024661
11206	EBNA1	CD48	0.12822
11207	EBNA1	DNASE1L2	0.041082
11208	BMLF1	LENG8	0.47224
11209	EBNA1	EWSR1	0.16735
11210	gL	CCSER1	0.15281
11211	EBNA3	MEN1	0.057102
11212	BLLF1	SYT11	0.14762
11213	BZLF1	PSMB5	0.051495
11214	gL	C12orf50	0.13676
11215	EBNA1	ZNF655	0.093521
11216	BMLF1	CNPY4	0.095066
11217	BZLF1	PIFO	0.11902
11218	EBNA3	IL12B	0.046318
11219	BPLF1	CLIP1	0.056582
11220	BZLF1	DSG3	0.041703

11221	BMLF1	CHMP1A	0.18164
11222	BHRF1	UNQ6125/PRO20090	0.11348
11223	BZLF1	GGA3	0.15329
11224	BZLF1	SSR4	0.093875
11225	EBNA-LP	TBC1D15	0.31086
11226	EBNA1	FBXW7	0.037956
11227	BMLF1	RASSF6	0.43005
11228	EBNA3	ZNF217	0.10823
11229	EBNA1	ATP8A1	0.13979
11230	BMLF1	PADI4	0.12779
11231	BGLF4	USP14	0.12489
11232	gL	COMP	0.049795
11233	gL	RSRC2	0.1484
11234	EBNA3	CINP	0.36946
11235	BOLF1	RNMT	0.019596
11236	gN	FAM188B	0.044482
11237	EBNA-LP	TRIM2	0.28262
11238	BZLF2	NOTCH2	0.010598
11239	BZLF1	TEK	0.039069
11240	EBNA3	CUL7	0.25864
11241	EBNA1	SOD3	0.086936
11242	BBRF2	BRINP3	0.027756
11243	BHRF1	CCDC66	0.018583
11244	BGLF4	CDK5RAP2	0.069633
11245	EBNA-LP	AKIP1	0.37519
11246	EBNA1	MED8	0.11387
11247	EBNA-LP	BCDIN3D	0.082323
11248	EBNA3	NF2	0.11474
11249	BZLF1	CD200	0.17428
11250	BGLF4	PDE2A	0.047446
11251	EBNA6	POLG	0.084561
11252	EBNA3	APC2	0.26062
11253	BZLF1	LILRB3	0.18038
11254	EBNA-LP	ATP10A	0.19512
11255	EBNA1	C2CD5	0.042312
11256	BMLF1	WDR77	0.26581
11257	EBNA-LP	MIA3	0.29626
11258	EBNA3	MAGI1	0.27321
11259	BMLF1	UBE3A	0.18158
11260	BMLF1	UMODL1	0.20889
11261	BZLF1	SDHAF2	0.20375
11262	BMLF1	ERICH1-AS1	0.028141
11263	BHRF1	REPS2	0.1734
11264	BMLF1	GEMIN2	0.29407
11265	EBNA-LP	ANXA2	0.3118
11266	BZLF1	UFD1L	0.16981
11267	BZLF1	CSNK1D	0.047651
11268	BMLF1	RBCK1	0.13344
11269	BGLF4	POLDIP3	0.19984
11270	EBNA3	SCARF2	0.36643
11271	BGLF4	VEGFC	0.095165
11272	BGLF4	PTGER4	0.084808
11273	EBNA-LP	TUBA3C	0.12741
11274	EBNA3	TTN	0.11695
11275	BZLF1	CCDC28A	0.01109
11276	BMLF1	BLOC1S6	0.18379
11277	gL	ZK1128.3	0.059584
11278	EBNA3	RNF111	0.15896
11279	BMLF1	RDX	0.28131
11280	EBNA3	XYLB	0.031153
11281	BMLF1	AP2S1	0.010825
11282	BZLF1	UBA5	0.069264
11283	BGLF4	EZH2	0.12123
11284	EBNA3	RBBP4	0.17034
11285	EBNA-LP	NOD1	0.080073
11286	BILF1	HEATR5B	0.084497
11287	EBNA4	GPRIN3	0.11589
11288	BBRF2	GNPTG	0.014436

11289	BGLF4	MBL2	0.10874
11290	BGLF4	GIPC1	0.081429
11291	BZLF2	YPR022C	0.083631
11292	BGLF4	NFKBIL1	0.1811
11293	EBNA3	CDK20	0.14843
11294	BMLF1	TFAP2B	0.062182
11295	BZLF1	S100A16	0.031292
11296	BZLF1	FIS1	0.042223
11297	EBNA3	BIN3	0.31315
11298	BZLF1	PRPF6	0.17053
11299	EBNA1	DSP	0.068299
11300	EBNA3	NCF2	0.10472
11301	BGLF4	DTX1	0.092636
11302	LMP2	CIB1	0.074814
11303	BLLF2	TMEM78	0.010792
11304	BMLF1	NRIP1	0.14507
11305	EBNA6	S100A12	0.092962
11306	BLLF1	SPBC1539.02	0.018858
11307	BLLF2	OAS3	0.043622
11308	gM	ANXA7	0.035959
11309	EBNA3	PLSCR4	0.55973
11310	EBNA-LP	VANGL1	0.39355
11311	BGLF4	ROPN1L	0.20789
11312	gL	SPRYD7	0.03517
11313	EBNA3	MEX3B	0.15889
11314	EBNA-LP	PTPN12	0.14844
11315	EBNA3	ARL13B	0.35702
11316	EBNA4	CDH23	0.077296
11317	EBNA1	CUL7	0.12974
11318	EBNA-LP	SPC25	0.25564
11319	BGLF4	LILRB3	0.18648
11320	EBNA3	CCNE2	0.1727
11321	BZLF1	STAT5B	0.022813
11322	BMLF1	MSR1	0.33556
11323	EBNA1	ZNF622	0.10923
11324	BMLF1	NCOR1	0.073703
11325	EBNA3	NOL8	0.37594
11326	BARF1	SYT1	0.078967
11327	BMLF1	ATN1	0.2432
11328	BXRF1	CFTR	0.01393
11329	BMLF1	PTK6	0.16476
11330	EBNA3	PTPN11	0.061645
11331	BZLF1	CASP10	0.16375
11332	BMLF1	SMC3	0.16453
11333	BZLF1	SWSAP1	0.19415
11334	BMLF1	NXF1	0.26669
11335	EBNA-LP	KIAA0319L	0.39355
11336	BMLF1	MGST3	0.037347
11337	BMLF1	P4HB	0.1644
11338	BMLF1	FAT1	0.39294
11339	EBNA3	PRMT2	0.23962
11340	EBNA3	MC1R	0.17464
11341	EBNA1	RHO	0.088775
11342	BBRF2	CMTM4	0.011143
11343	BLLF2	SUMF1	0.043879
11344	EBNA1	LOX	0.11281
11345	BMLF1	INO80B	0.28811
11346	BMLF1	HMCN2	0.26791
11347	EBNA3	NSMCE2	0.20554
11348	EBNA3	F9	0.18546
11349	BZLF1	ACVR2B	0.026405
11350	BGLF4	ERBB4	0.018626
11351	BMLF1	SIRT3	0.26991
11352	BMLF1	SEC24C	0.14425
11353	EBNA-LP	SLC1A5	0.018442
11354	EBNA-LP	HPCAL1	0.2099
11355	EBNA1	ULK2	0.17243
11356	EBNA-LP	AKAP5	0.066077

11357	EBNA-LP	ARHGAP24	0.1833
11358	EBNA-LP	BCCIP	0.33806
11359	EBNA1	KIRREL3	0.18926
11360	BHRF1	CCDC154	0.025697
11361	BMLF1	SPAG9	0.074231
11362	BMLF1	MEF2A	0.077716
11363	EBNA4	ACTN2	0.070761
11364	EBNA-LP	S100A2	0.19934
11365	EBNA4	ELOVL3	0.1197
11366	BZLF1	EIF4A3	0.14138
11367	BMLF1	OLA1	0.31809
11368	BZLF1	KLRF2	0.2073
11369	BLLF1	RGAG1	0.010596
11370	BMLF1	KIAA0020	0.024139
11371	EBNA3	EDEM1	0.45054
11372	EBNA3	NGFRAP1	0.26056
11373	EBNA3	FAM120C	0.026393
11374	EBNA3	ASIC2	0.3057
11375	EBNA1	AGRN	0.061607
11376	BZLF1	RPS27L	0.13096
11377	EBNA4	OFD1	0.14346
11378	BMLF1	RUVBL2	0.12573
11379	EBNA-LP	SOCS1	0.18678
11380	BLLF2	TREX2	0.062878
11381	BZLF1	BMX	0.04596
11382	EBNA3	CASD1	0.16974
11383	EBNA3	MAGOH	0.31208
11384	EBNA3	RYK	0.20914
11385	BLLF2	CLPP	0.080171
11386	EBNA3	CDCA5	0.26081
11387	BLLF2	OAS2	0.02444
11388	EBNA6	DKC1	0.086919
11389	BLLF1	BVES-AS1	0.022907
11390	EBNA-LP	PTPN22	0.083733
11391	BMLF1	METTL21C	0.059751
11392	EBNA3	TIMM44	0.26248
11393	EBNA1	SERPINB5	0.15551
11394	BLLF2	ELOVL1	0.05608
11395	EBNA-LP	NAP1L1	0.29518
11396	EBNA3	NDFIP2	0.21362
11397	BALF2	THBS2	0.11169
11398	BGLF4	ITPR3	0.033134
11399	EBNA-LP	KIT	0.040292
11400	BZLF1	OTUB1	0.13923
11401	BLLF2	SOSTDC1	0.01831
11402	BMLF1	RAB8A	0.24276
11403	EBNA-LP	SYNPR	0.011429
11404	EBNA3	RTKN	0.24151
11405	BMLF1	DAZ2	0.011111
11406	BMLF1	TSC22D4	0.22434
11407	EBNA3	SHANK3	0.14071
11408	BLLF2	HR	0.018039
11409	EBNA3	MYOD1	0.042174
11410	EBNA1	XPO1	0.10336
11411	EBNA4	BVES-AS1	0.12668
11412	BZLF2	CHAF1A	0.10663
11413	EBNA1	STAM2	0.10074
11414	BHRF1	PPP2R3B	0.067833
11415	EBNA6	C19orf67	0.068969
11416	EBNA3	NCOA6	0.14204
11417	BGLF4	SOCS7	0.16963
11418	BZLF1	CCNK	0.10573
11419	BMLF1	ATIC	0.018644
11420	BMLF1	THBS3	0.19168
11421	BNLF2b	ASTL	0.014117
11422	EBNA3	C19orf80	0.42461
11423	BZLF1	HDDC2	0.15088
11424	EBNA1	MMP14	0.064091

11425	EBNA1	PRMT6	0.12721
11426	BZLF1	TIMM10	0.11387
11427	BLLF2	TBC1D16	0.010476
11428	BGLF4	SPC25	0.1372
11429	BMLF1	SYP	0.1422
11430	BRRF1	IGFBP2	0.012518
11431	EBNA1	CTU1	0.22057
11432	BXRF1	FAM124A	0.011763
11433	BZLF1	SUFU	0.042897
11434	gM	NELL1	0.05018
11435	EBNA2	RAB11FIP4	0.17904
11436	EBNA1	ITGB3	0.03368
11437	BMLF1	RGL4	0.064395
11438	EBNA4	TLDC2	0.20049
11439	BPLF1	MEGF8	0.020167
11440	EBNA3	ANKZF1	0.35796
11441	BRRF1	PKD2	0.010281
11442	BGLF4	ABCG5	0.095228
11443	BMLF1	PYGO1	0.30702
11444	EBNA1	RNF32	0.15001
11445	EBNA1	EFNA1	0.096003
11446	BZLF1	FMNL1	0.13806
11447	BOLF1	PI4K2A	0.033647
11448	BZLF1	CREBRF	0.052323
11449	BZLF1	NGLY1	0.16994
11450	EBNA6	AKAP3	0.1052
11451	BZLF1	IFNAR2	0.082902
11452	EBNA-LP	STX1A	0.13926
11453	BGLF4	IL10RA	0.19561
11454	EBNA3	MRE11A	0.13592
11455	BHRF1	PRR15L	0.024887
11456	BHRF1	ZK1128.3	0.045054
11457	BBRF2	SPINK5	0.01879
11458	BBRF2	POLR2H	0.017267
11459	BVRF1	CDH5	0.091625
11460	BMLF1	MED16	0.012675
11461	EBNA1	CALB2	0.059635
11462	BBRF2	ITIH5	0.011393
11463	BPLF1	C1orf168	0.060248
11464	BHRF1	POM121L2	0.076024
11465	BGLF4	RIPK2	0.010463
11466	EBNA1	DDX1	0.068263
11467	EBNA3	CBR4	0.35293
11468	BARF1	RAB11FIP4	0.29212
11469	EBNA1	ZFYVE27	0.12116
11470	BZLF1	CUBN	0.023641
11471	EBNA-LP	EAF2	0.25434
11472	EBNA-LP	PBX1	0.049116
11473	BZLF1	WDR4	0.17502
11474	BHRF1	MATN4	0.16837
11475	BMLF1	PLSCR1	0.2904
11476	EBNA-LP	YKT6	0.067689
11477	EBNA3	PTRH2	0.17177
11478	BZLF1	VLDLR	0.13584
11479	EBNA4	EDAR	0.10216
11480	BZLF1	XRN1	0.13392
11481	BOLF1	EIF4E2	0.011199
11482	BGLF4	PLA2G12B	0.039329
11483	BMLF1	NIF3L1	0.363
11484	BZLF2	C15orf65	0.020547
11485	EBNA3	PLRG1	0.31803
11486	EBNA2	FAM188A	0.096208
11487	gL	S100P	0.013392
11488	EBNA-LP	C1QA	0.35232
11489	EBNA1	EPB41	0.047778
11490	EBNA6	GOLGA6L20	0.11347
11491	EBNA3	GAS1	0.10821
11492	BMLF1	HDAC1	0.04113

11493	BLLF2	APOL5	0.021191
11494	BLLF2	PRAME	0.13851
11495	BZLF1	STXBP3	0.10688
11496	BGLF4	WWC1	0.13702
11497	EBNA3	CYBB	0.15928
11498	EBNA1	PKMYT1	0.032308
11499	BALF2	SPHK2	0.01114
11500	EBNA1	DVL1	0.035711
11501	EBNA1	SMPDL3A	0.16225
11502	BMLF1	CCDC172	0.012591
11503	EBNA-LP	EDAR	0.24746
11504	BLLF2	RITA1	0.070398
11505	BZLF2	MUM1L1	0.062127
11506	EBNA-LP	AKAP7	0.087038
11507	BGLF4	HRAS	0.026124
11508	EBNA-LP	GIT2	0.24004
11509	EBNA1	ZDHHC17	0.17217
11510	BMLF1	TBC1D7	0.38177
11511	BGLF4	SDF4	0.30046
11512	BGLF4	APLP2	0.088852
11513	BZLF1	RAD23A	0.13182
11514	BGLF4	LTC4S	0.081563
11515	EBNA1	PCDH19	0.038413
11516	BMLF1	CASK	0.15178
11517	BMLF1	AHSG	0.053146
11518	gL	MYL2	0.10546
11519	EBNA3	BEND7	0.43795
11520	EBNA3	NXT1	0.29595
11521	BMLF1	EIF4H	0.35896
11522	BMLF1	TMPRSS6	0.34599
11523	EBNA-LP	SYVN1	0.17694
11524	BZLF1	RHOG	0.085928
11525	EBNA-LP	CANX	0.30358
11526	BMLF1	MRPL28	0.37763
11527	BZLF2	CROCCP3	0.01729
11528	EBNA3	SLC38A1	0.033004
11529	BGLF4	BCOR	0.1069
11530	BZLF1	PAGE2B	0.011844
11531	EBNA3	CBFA2T3	0.080595
11532	gL	LURAP1L	0.17349
11533	EBNA-LP	CDH18	0.16534
11534	BLLF2	POM121L1P	0.066477
11535	BRRF1	NBR1	0.02589
11536	EBNA-LP	APOH	0.17152
11537	EBNA3	PARD6G	0.32379
11538	BMLF1	RAPGEF4	0.13785
11539	EBNA3	MORF4L1	0.25613
11540	EBNA4	MYL6	0.20406
11541	BGLF4	SRCIN1	0.09261
11542	BMLF1	ADAM15	0.22867
11543	BHRF1	YGL014C-A	0.12492
11544	EBNA3	EIF2S2	0.39731
11545	BGRF1/BDRF1	DTNBP1	0.019021
11546	BHRF1	SS18L2	0.033622
11547	EBNA1	CMKLR1	0.11645
11548	BGLF4	EIF4E	0.16525
11549	EBNA-LP	MYOZ3	0.41355
11550	BMLF1	BNIP2	0.42773
11551	EBNA3	PLEKHF2	0.24926
11552	EBNA6	FAM13C	0.015509
11553	BLLF2	MCCC2	0.019866
11554	EBNA3	DYDC2	0.032865
11555	BZLF1	FCGBP	0.22211
11556	EBNA1	TRMT61B	0.2479
11557	EBNA-LP	BLMH	0.25099
11558	BGLF4	IFI6	0.17588
11559	EBNA-LP	MPHOSPH10	0.24086
11560	BMLF1	MKS1	0.29745

EBNA-LP	RLF	0.20927
BLLF2	AP5M1	0.017318
BLLF2	ZNF354A	0.02459
EBNA4	FAM161B	0.33782
BBRF2	TAS2R46	0.023843
EBNA3	PLCL2	0.022985
BGLF4	PPM1D	0.086685
EBNA-LP	MYC	0.039341
BMLF1	ACADM	0.026849
EBNA1	ERCC4	0.048591
BMLF1	PRAMEF14	0.073446
BLLF2	ZNF837	0.014966
EBNA3	ITGB1BP1	0.34108
BMLF1	IL1RL1	0.23281
BMLF1	EIF2S2	0.39595
EBNA-LP	DRD1	0.030556
BGLF4	STRN	0.15296
EBNA1	RASA1	0.032085
EBNA3	SH3RF1	0.21899
BBRF2	NRDE2	0.019017
BGLF4	CFL1	0.086157
gB	WDR96	0.012272
gB	C1orf64	0.019639
BGLF4	C10orf62	0.24432
EBNA3	RPGR	0.3018
BGLF4	TLN2	0.074478
EBNA1	E2F1	0.012487
BXRF1	AMOT	0.025578
BMLF1	BIN3	0.33962
BALF2	NID2	0.14421
EBNA1	PDLIM4	0.17587
BZLF1	TRIM5	0.082105
EBNA1	FN1	0.090943
BLLF2	FBXO17	0.036078
BLLF2	FSIP2	0.20692
BZLF2	C5orf52	0.017947
BZLF2	UPF3B	0.067421
EBNA3	CCAR1	0.49005
BLLF1	FSIP1	0.036268
BGLF4	ATRX	0.10141
EBNA3	RAB21	0.29013
BMLF1	APPL1	0.17882
BMLF1	MORC4	0.37699
EBNA-LP	GEMIN2	0.27055
EBNA1	LONRF3	0.16176
EBNA1	USP37	0.065853
EBNA1	TCF25	0.048659
EBNA1	MORF4L2	0.14843
BILF1	FAM199X	0.0156
BMLF1	BCL7C	0.080543
EBNA1	ETV7	0.048981
EBNA1	DZIP3	0.1794
BOLF1	DTNBP1	0.012839
EBNA6	TLX1NB	0.045372
BGLF4	DDX39A	0.12837
EBNA1	GABPA	0.023563
EBNA-LP	PIR	0.35155
EBNA3	RAD17	0.24872
BZLF1	TP53RK	0.14496
BGLF4	INTS10	0.19104
BPLF1	SCIMP	0.1335
BGLF4	ISCU	0.020117
BMLF1	IGFLR1	0.43034
BLLF2	OR10Q1	0.076296
BMLF1	NOD1	0.091051
BGLF4	HGF	0.088178
BGLF4	TMED2	0.15445
EBNA3	CCDC67	0.01313

11629	BZLF1	NPBWR1	0.15582
11630	EBNA3	RAC1	0.045196
11631	BMLF1	CLN6	0.30169
11632	BGLF4	RASSF4	0.22951
11633	BZLF1	CUL4A	0.15694
11634	BZLF1	LINC00523	0.011602
11635	BGLF4	INSR	0.012414
11636	BZLF1	RUFY1	0.16533
11637	BGLF4	PRMT2	0.13696
11638	EBNA-LP	NAA15	0.21921
11639	EBNA1	DST	0.15826
11640	BLLF2	TBC1D3	0.011597
11641	BZLF1	GSK3A	0.03234
11642	BOLF1	SPTAN1	0.088831
11643	BMLF1	TUBGCP2	0.26301
11644	BGLF4	RAB22A	0.12322
11645	BZLF1	SNAPIN	0.14038
11646	EBNA-LP	ECM1	0.19391
11647	EBNA4	C1orf64	0.20239
11648	BBRF2	DGCR14	0.011941
11649	EBNA1	MAP1LC3A	0.14558
11650	EBNA1	F12	0.13827
11651	BLLF2	ENTPD6	0.012917
11652	BMLF1	HAX1	0.3323
11653	EBNA-LP	PCDHB14	0.1636
11654	BZLF1	MAP1S	0.070554
11655	BXRF1	MAP1LC3B	0.032363
11656	EBNA3	PELI2	0.27882
11657	BGLF4	MTCH1	0.1776
11658	EBNA1	KIAA0319	0.15765
11659	EBNA3	ILKAP	0.22625
11660	BZLF1	NR3C1	0.019831
11661	EBNA-LP	PPP2R3A	0.50834
11662	EBNA3	RPUSD2	0.052937
11663	BZLF1	MZT1	0.18333
11664	BGLF4	MED28	0.16373
11665	BMLF1	UFC1	0.44387
11666	BGLF4	VSTM2L	0.24432
11667	BMLF1	SIRT1	0.014439
11668	BZLF2	CCDC114	0.011201
11669	EBNA4	SUPT20HL2	0.19624
11670	EBNA3	INTS4	0.36808
11671	BMLF1	ATL2	0.23664
11672	EBNA3	MEF2BNB	0.43347
11673	BMLF1	AIP	0.17514
11674	EBNA1	POLR2B	0.063508
11675	BZLF1	ELP4	0.12132
11676	BXRF1	PITPNM3	0.15045
11677	BOLF1	PRDX6	0.018744
11678	BLLF2	RPS10	0.034761
11679	BGLF4	PXMP4	0.20964
11680	EBNA1	ZFYVE16	0.060949
11681	EBNA3	TUBGCP3	0.14849
11682	EBNA1	RAB25	0.20342
11683	EBNA3	DYRK1A	0.11681
11684	BGLF4	ARFRP1	0.12741
11685	EBNA-LP	ABCD1	0.18349
11686	BZLF1	CALN1	0.046998
11687	BLLF1	THBS1	0.012316
11688	BMLF1	CDK14	0.16182
11689	BZLF1	IMMT	0.18578
11690	BMLF1	CASR	0.28465
11691	EBNA3	PIK3CB	0.1589
11692	EBNA-LP	KAT8	0.16169
11693	BXRF1	MATN3	0.19248
11694	EBNA-LP	NHP2L1	0.31949
11695	BGLF4	FMR1	0.23223
11696	BMLF1	TCEB2	0.24244

11697	EBNA3	TRAK2	0.20146
11698	EBNA3	PIK3CD	0.15281
11699	EBNA6	DDX26B	0.23298
11700	EBNA1	RAB4A	0.088107
11701	BZLF1	FBN3	0.069724
11702	EBNA1	F2RL2	0.12096
11703	BMLF1	ARHGEF7	0.10035
11704	BZLF2	FAM166B	0.016686
11705	BMLF1	TTLL6	0.28661
11706	EBNA3	ESR1	0.023638
11707	EBNA-LP	NACA	0.27064
11708	EBNA3	PGS1	0.11589
11709	BZLF1	INHBB	0.13552
11710	EBNA6	SLC14A2	0.10575
11711	BPLF1	FAM199X	0.0365
11712	BHRF1	C11orf35	0.02649
11713	BMLF1	GRIP1	0.060676
11714	EBNA1	GRB7	0.057465
11715	EBNA-LP	LDOC1	0.37504
11716	BZLF1	ERCC6	0.047704
11717	EBNA-LP	TLN1	0.078784
11718	BGLF4	PLEKHG6	0.081881
11719	gL	SPANXA2-OT1	0.060491
11720	BZLF1	PRDM16	0.07004
11721	BZLF1	TRIO	0.02384
11722	BMLF1	SPRTN	0.31483
11723	EBNA3	BTN3A1	0.36145
11724	BMLF1	WAPAL	0.21802
11725	EBNA6	BNIP2	0.080935
11726	EBNA3	MRPS7	0.06538
11727	EBNA6	PPP3CA	0.034206
11728	EBNA-LP	MAGI1	0.26966
11729	BDLF1	MATN2	0.031957
11730	BGLF4	GH1	0.12643
11731	EBNA1	SPINK7	0.1968
11732	gM	EDEM1	0.0416
11733	BGLF4	MED23	0.14389
11734	BVRF1	ATP2A1	0.04836
11735	BMLF1	HDAC4	0.03549
11736	BMLF1	KIF1B	0.29507
11737	BRRF1	MASP1	0.24001
11738	EBNA-LP	MAP4K5	0.22233
11739	BZLF1	PDGFRB	0.048993
11740	BZLF1	GTF2A2	0.09435
11741	BMLF1	ATG2A	0.47224
11742	BZLF1	MAT2B	0.098369
11743	BPLF1	ZK1128.3	0.042048
11744	EBNA-LP	TOMM20	0.21152
11745	EBNA1	APOH	0.089458
11746	EBNA1	APC	0.012497
11747	EBNA3	MICU1	0.55121
11748	BMLF1	ADAMTS10	0.24044
11749	BGLF4	EXD3	0.24432
11750	EBNA-LP	CCL18	0.32171
11751	BLLF2	RPL23	0.073418
11752	BGLF4	STK25	0.052583
11753	EBNA3	FAM151B	0.050726
11754	BMLF1	ZNF274	0.1731
11755	EBNA-LP	PMS2	0.21016
11756	BZLF1	EYA2	0.027367
11757	EBNA1	COMMD3	0.22232
11758	EBNA4	SPHK1	0.025945
11759	EBNA4	TICRR	0.24295
11760	EBNA3	SLC2A4	0.18494
11761	EBNA2	FBN1	0.12855
11762	BMLF1	MAP3K7	0.042872
11763	BZLF2	CETN2	0.12648
11764	EBNA-LP	PRPF31	0.29672

11765	BMLF1	NKX2-5	0.014963
11766	EBNA1	STT3A	0.18743
11767	BMLF1	GNL3L	0.30739
11768	EBNA1	MCTP2	0.041209
11769	EBNA4	TTYH1	0.35401
11770	BLLF2	LVP1a	0.026485
11771	BGLF4	CDH1	0.065917
11772	BZLF2	ZK1128.3	0.026431
11773	BMLF1	ERC1	0.20574
11774	BMLF1	NEFH	0.06325
11775	EBNA3	IL4	0.059356
11776	EBNA-LP	CHMP5	0.34486
11777	EBNA-LP	BHLHE40	0.097794
11778	BZLF1	SERPINF2	0.11466
11779	EBNA1	MMP15	0.2296
11780	EBNA3	SPTSSA	0.28596
11781	BMLF1	EXTL1	0.018808
11782	BHRF1	ATP5F1	0.020977
11783	EBNA3	RBFOX2	0.25349
11784	BGLF4	HNRNPC	0.15744
11785	BPLF1	NOTCH3	0.034836
11786	EBNA1	CLTB	0.16248
11787	BGLF4	ADAM23	0.11836
11788	EBNA3	PLEKHG6	0.17476
11789	BZLF1	STX4	0.096741
11790	BGLF4	ZNF326	0.20239
11791	BVRF1	LTBP1	0.16999
11792	BMLF1	SNAPIN	0.23429
11793	BVRF1	DPP4	0.017617
11794	BBRF2	NIPBL	0.016914
11795	BZLF2	GAGE3	0.021152
11796	BMLF1	PMVK	0.030459
11797	BTRF1	FBXO10	0.011226
11798	BZLF2	LINC00299	0.011058
11799	BZLF1	PCDHA11	0.046144
11800	EBNA6	C15orf54	0.10697
11801	EBNA3	SCGB3A1	0.16307
11802	BMLF1	LRP4	0.34385
11803	BZLF1	RAD1	0.10854
11804	BGRF1/BDRF1	PRSS3	0.2162
11805	EBNA4	FAM199X	0.097262
11806	EBNA3	PRDX4	0.42023
11807	EBNA-LP	LRP5	0.12842
11808	BGLF4	TIMM10	0.1273
11809	BLLF2	CTAGE6	0.018636
11810	BMLF1	MOB4	0.33795
11811	BHRF1	SLIT2	0.030797
11812	EBNA6	RSRC1	0.23721
11813	BBRF2	SLURP1	0.047341
11814	BHRF1	UBQLNL	0.012485
11815	BGLF4	ARHGAP4	0.12713
11816	BZLF1	DEPTOR	0.19691
11817	BZLF1	EIF2S1	0.16962
11818	BGLF4	MEOX2	0.072894
11819	EBNA-LP	SUCLA2	0.17663
11820	BPLF1	PNAS-138	0.075559
11821	BZLF1	RIMS2	0.15365
11822	EBNA-LP	TRAPPC2	0.19987
11823	EBNA1	SMC1A	0.065698
11824	BGLF4	PRF1	0.23258
11825	EBNA-LP	MAP3K5	0.070434
11826	BGLF4	DNM1L	0.033391
11827	EBNA3	COL17A1	0.27076
11828	EBNA3	PCSK2	0.37892
11829	EBNA3	HSPA1A	0.12676
11830	BLLF1	TPPP	0.074482
11831	EBNA-LP	ZNF256	0.13462
11832	BGLF4	HCK	0.031826

BNLF2b	FAM19A4	0.023829
BZLF1	HEXIM1	0.14558
BZLF1	KIF3B	0.10238
EBNA-LP	DST	0.23654
BZLF1	CALCA	0.065787
BGLF4	KLC1	0.18612
EBNA-LP	SDPR	0.2717
BZLF1	UMODL1	0.044094
BMLF1	CDH3	0.21159
BZLF1	ACACB	0.11988
BGLF4	APOE	0.012175
BGLF4	TSPY1	0.13833
EBNA3	SMARCAD1	0.20076
EBNA-LP	SNX7	0.12516
EBNA1	IGFBP7	0.17432
BGLF4	RPL27A	0.18098
BGLF4	ELP5	0.20349
EBNA-LP	SLC23A2	0.034041
EBNA3	LCMT1	0.45808
gL	TCAP	0.096786
EBNA3	LIN28B	0.27654
BPLF1	SETD2	0.012016
EBNA6	ERLIN1	0.19379
BZLF1	GNB2L1	0.02263
BLLF1	GRIN1	0.035199
BMLF1	COMMD8	0.47224
EBNA-LP	GRIN1	0.2055
BMLF1	IL18R1	0.032129
BMLF1	TJP2	0.32591
gB	ANXA2	0.036922
EBNA3	ADI1	0.23233
BBRF2	TAS2R38	0.026913
EBNA4	KAT2A	0.019912
BZLF1	ADNP2	0.024047
EBNA3	SUV39H1	0.19736
BZLF1	SCAMP2	0.11304
BGLF4	ZMYND11	0.11329
BLLF2	CD53	0.011442
BHRF1	C10orf91	0.1556
EBNA-LP	MAN1B1	0.091939
BGLF4	MPP1	0.17425
EBNA3	LOXL4	0.31107
BVRF1	PPEF2	0.070768
BGLF4	ESPL1	0.10629
EBNA3	CRY1	0.11628
BGLF4	ZGPAT	0.065129
BLLF2	ZNF548	0.017271
EBNA3	UQCRC2	0.16336
EBNA1	FEZ2	0.19123
BMLF1	ORC1	0.22835
BMLF1	RPN1	0.023945
BZLF2	UBQLNL	0.010089
BZLF1	STRN4	0.2231
BOLF1	CLIC4	0.06449
EBNA6	SNW1	0.078814
BZLF2	NECAB2	0.17336
BZLF1	MBD3	0.11948
BMLF1	TRAIP	0.30834
EBNA1	PKDREJ	0.038361
EBNA-LP	CDH24	0.15733
EBNA1	HIP1	0.05697
BLLF2	ZNF136	0.014073
EBNA3	ZMYND11	0.18838
BPLF1	KIAA1751	0.016992
EBNA-LP	FKBP10	0.178
EBNA3	VEGFC	0.16839
BZLF2	PRR19	0.043323
BMLF1	HNRNPR	0.32065

11901	BGLF4	UGP2	0.10141
11902	EBNA3	FNTB	0.22431
11903	BGLF4	EDIL3	0.058233
11904	EBNA1	PLXDC2	0.20909
11905	EBNA-LP	TGM3	0.076687
11906	BLLF2	FAXC	0.013561
11907	EBNA1	PLK3	0.042554
11908	EBNA3	TAOK3	0.21257
11909	BRRF1	ATP2B2	0.018165
11910	EBNA3	MMP10	0.05405
11911	EBNA3	NOTCH2NL	0.18082
11912	BZLF1	IGSF1	0.17412
11913	BMLF1	CHRNA3	0.013717
11914	EBNA3	MAP3K11	0.13316
11915	BMLF1	SLC25A18	0.030525
11916	BMLF1	CD200	0.36839
11917	BMLF1	GGN	0.048096
11918	BZLF1	CHD8	0.061687
11919	EBNA-LP	CTR9	0.19248
11920	BGLF4	PEX1	0.077846
11921	EBNA3	PIH1D2	0.10183
11922	BBRF2	OR2AG1	0.032661
11923	BLLF2	OR2H2	0.078589
11924	EBNA-LP	APC	0.022115
11925	BMLF1	CD226	0.21117
11926	BZLF1	EIF3G	0.23351
11927	BDLF1	FAM231D	0.013105
11928	BZLF1	KMT2B	0.068275
11929	BGLF4	HMCN1	0.088281
11930	EBNA3	AP2M1	0.22973
11931	EBNA2	PRSS2	0.12192
11932	BZLF1	NAA50	0.26017
11933	BGLF4	SPOP	0.19946
11934	BMLF1	MPDZ	0.29002
11935	BLLF2	IFNA21	0.020126
11936	BLLF1	PIH1D2	0.035655
11937	BMLF1	NPIPA1	0.032041
11938	EBNA6	CDRT4	0.065562
11939	EBNA-LP	KPNA2	0.25008
11940	BGLF4	SPRR2E	0.1142
11941	BZLF1	NCL	0.14005
11942	EBNA-LP	TAB1	0.14543
11943	EBNA3	PADI2	0.15357
11944	BGLF4	PTGES3	0.10846
11945	EBNA3	TSC2	0.08244
11946	EBNA3	SPC24	0.27505
11947	BZLF1	ECT2	0.02699
11948	BZLF1	ABI1	0.073412
11949	BMLF1	SLC22A8	0.035702
11950	EBNA3	AKAP3	0.4169
11951	EBNA3	CMPK2	0.13466
11952	EBNA1	PSMB5	0.04753
11953	BMLF1	IL1A	0.14951
11954	BZLF1	BRAP	0.12022
11955	BGLF4	ARRB1	0.044607
11956	EBNA-LP	PDK3	0.28678
11957	EBNA1	RXRB	0.035598
11958	BGLF4	SCHIP1	0.20223
11959	BZLF1	PSG5	0.22204
11960	BZLF1	GTF2B	0.092717
11961	BMLF1	ULK2	0.30814
11962	EBNA3	ECM29	0.024911
11963	EBNA3	TCOF1	0.35854
11964	EBNA3	S100A10	0.1104
11965	gB	GCH1	0.014596
11966	BGLF4	PASK	0.053218
11967	EBNA-LP	TLR2	0.048032
11968	EBNA3	NFKBIL1	0.32861

11969	BMLF1	DCAF7	0.37526
11970	EBNA1	RPS10	0.096245
11971	BBRF2	ANAPC10	0.024948
11972	gL	FAM46B	0.090659
11973	BMLF1	VPS4B	0.17721
11974	BZLF1	HIST1H1E	0.1247
11975	EBNA2	CCDC47	0.10513
11976	EBNA-LP	EIF4EBP3	0.33586
11977	BMLF1	PLA2G2C	0.19202
11978	BZLF1	SNRNP200	0.11806
11979	EBNA1	HMCN1	0.079161
11980	BGLF4	UBE2E1	0.074559
11981	gB	CLIP1	0.021274
11982	BHRF1	PNMAL1	0.056466
11983	EBNA3	MAGED4	0.012942
11984	EBNA1	NEGR1	0.17552
11985	BLLF2	GGT7	0.034697
11986	BZLF1	DUSP2	0.025217
11987	BZLF1	COPB1	0.077387
11988	EBNA1	TRPS1	0.022798
11989	BMLF1	IPO5	0.27546
11990	BBRF2	SLC39A6	0.01372
11991	BZLF1	PABPC1	0.10197
11992	BLLF1	FAM188B2	0.06742
11993	BGLF4	TXNDC17	0.20754
11994	EBNA4	CCDC87	0.07302
11995	BZLF1	RIT1	0.16523
11996	EBNA3	CCDC57	0.011157
11997	BZLF1	SMG1	0.098514
11998	BZLF1	CDC45	0.13433
11999	BPLF1	CHAF1A	0.16798
12000	BMLF1	OCLN	0.17513
12001	BGLF4	CAMK1	0.021295
12002	gL	ASAH2B	0.0126
12003	EBNA3	CDC25C	0.041093
12004	BZLF1	LILRA4	0.2221
12005	BGLF4	CCDC92	0.22233
12006	BGLF4	CTU2	0.20364
12007	BMLF1	PLCG1	0.12908
12008	BGLF4	ARL11	0.19777
12009	BMLF1	NCAPH	0.34337
12010	EBNA3	GPATCH8	0.31803
12011	BGLF4	ROBO1	0.080729
12012	BMLF1	RPL28	0.29723
12013	EBNA-LP	CD93	0.4687
12014	BMLF1	SMURF2	0.16102
12015	EBNA1	MEX3B	0.034608
12016	EBNA-LP	OTUD7B	0.23796
12017	EBNA-LP	BCL6	0.0529
12018	BZLF1	SEC31A	0.12185
12019	BZLF1	IGF2BP3	0.15334
12020	EBNA1	TMCC2	0.20909
12021	BLLF2	ZNF814	0.018774
12022	BMLF1	LTBP4	0.43916
12023	BGLF4	VPS4A	0.094743
12024	BMLF1	PAG1	0.33795
12025	BZLF1	ANXA8L1	0.035342
12026	BZLF1	KCNG3	0.15369
12027	EBNA1	RRAS	0.096468
12028	BLLF2	MAB21L1	0.032244
12029	BBRF2	CCRN4L	0.034371
12030	EBNA2	LETM1	0.12242
12031	BZLF1	SNX27	0.14183
12032	BILF1	UTRN	0.077534
12033	EBNA3	TOMM70A	0.276
12034	BLLF2	CRYBA4	0.041667
12035	BMLF1	SCAI	0.30033
12036	EBNA1	SNCB	0.018398

12037	BMLF1	HSD17B8	0.27669
12038	EBNA3	KIF7	0.29168
12039	BZLF1	DCSTAMP	0.10668
12040	BLLF2	DKK4	0.037676
12041	BZLF1	RAB3IP	0.14395
12042	BMLF1	FBXW2	0.41015
12043	BGLF4	APPBP2	0.19644
12044	EBNA3	TRMT112	0.48058
12045	EBNA1	PDCL3	0.19074
12046	EBNA3	LINC00597	0.078515
12047	BMLF1	CDH15	0.18224
12048	BLLF2	YTHDC1	0.10348
12049	BZLF1	DUOX2	0.097959
12050	BGLF4	SYP	0.064825
12051	EBNA-LP	CARD8	0.3141
12052	BZLF1	NCOA2	0.034591
12053	EBNA4	TBC1D25	0.019396
12054	BLLF2	THAP7	0.023488
12055	BVRF1	DLL1	0.1374
12056	gB	S100A6	0.043675
12057	BMLF1	PRPF4B	0.23391
12058	EBNA3	POP4	0.33617
12059	EBNA-LP	ADORA1	0.050976
12060	EBNA3	UCN	0.18848
12061	EBNA3	RNF41	0.18197
12062	EBNA1	PRKCQ	0.016022
12063	BMLF1	FBXL15	0.36221
12064	EBNA-LP	INO80	0.16885
12065	EBNA1	BTRC	0.071544
12066	EBNA-LP	RPS6KC1	0.22198
12067	EBNA-LP	PSMB1	0.10787
12068	BNLF2b	SPINK13	0.015208
12069	EBNA3	PRKCQ	0.045045
12070	BGLF4	XPA	0.1109
12071	EBNA3	LLGL2	0.29636
12072	EBNA1	MYD88	0.03766
12073	BGLF4	SPRED1	0.062771
12074	BMLF1	SLA2	0.34196
12075	BGLF4	PFKM	0.079049
12076	EBNA3	TOPBP1	0.2643
12077	BMLF1	GINS2	0.36767
12078	BALF2	PRSS3	0.14506
12079	EBNA1	PCDHGC5	0.038413
12080	BZLF1	ITM2A	0.2221
12081	EBNA3	EXOC8	0.17668
12082	EBNA2	CELSR3	0.06966
12083	EBNA6	SNX9	0.08143
12084	EBNA1	CCNA2	0.062578
12085	BZLF1	HSPB2	0.16968
12086	BHRF1	CAPNS1	0.031618
12087	EBNA3	CASQ1	0.11396
12088	EBNA1	PPP1R13L	0.077221
12089	LMP2	S100A11	0.082776
12090	EBNA-LP	RCOR1	0.2012
12091	BXRF1	VIL1	0.077504
12092	BLLF1	CANX	0.046477
12093	EBNA2	TPD52	0.13427
12094	BZLF2	SLAIN1	0.090075
12095	BMLF1	ATG3	0.25972
12096	BARF1	CHP1	0.086468
12097	EBNA3	SYT8	0.014604
12098	BGLF4	RFC1	0.067323
12099	gL	SLAIN1	0.19495
12100	EBNA1	FAP	0.1318
12101	BLLF2	SRM	0.020507
12102	EBNA3	APP	0.028257
12103	BLLF2	CLDN24	0.028093
12104	BBRF2	TMEM2	0.017265

12105	BGLF4	KIF1B	0.15432
12106	BMLF1	KIAA1377	0.47224
12107	EBNA3	ATP6V0A2	0.24762
12108	EBNA3	LINC00478	0.027819
12109	EBNA4	CEP63	0.034598
12110	BVRF1	PRSS2	0.15449
12111	EBNA1	FKBP1A	0.095605
12112	EBNA3	POP5	0.28752
12113	EBNA3	pkmyt1	0.069132
12114	BMLF1	ADAMTSL4	0.29122
12115	BLLF2	TIMD4	0.017336
12116	EBNA-LP	INSIG1	0.20843
12117	EBNA3	AHR	0.030762
12118	EBNA1	UBE4A	0.17618
12119	BXRF1	CXorf67	0.059011
12120	BMLF1	NGFRAP1	0.29022
12121	BZLF2	DHH	0.10883
12122	BZLF1	BIRC2	0.056249
12123	BNLF2b	DEFB129	0.095261
12124	BGLF4	KIR2DL1	0.19146
12125	EBNA-LP	LSM12	0.032332
12126	BGLF4	G0S2	0.18911
12127	BMLF1	PRSS3P2	0.028743
12128	BMLF1	NCOA7	0.30108
12129	EBNA1	PTPRB	0.033851
12130	EBNA-LP	MAP3K7	0.03235
12131	BZLF2	RPUSD4	0.034334
12132	BZLF1	LRRC26	0.1778
12133	BMLF1	SPAST	0.16379
12134	BBRF2	G4	0.01209
12135	EBNA3	DCP1B	0.23138
12136	BBRF2	YIPF6	0.023612
12137	BGLF4	BDKRB2	0.085399
12138	BGLF4	TESC	0.20861
12139	BGLF4	POLB	0.06682
12140	EBNA-LP	GAREM	0.43184
12141	BZLF1	HERC5	0.10531
12142	EBNA3	CAPN2	0.35988
12143	BGLF4	GSK3A	0.020905
12144	BGLF4	IFT122	0.20719
12145	BZLF1	DNASE1L2	0.04979
12146	BZLF1	BOP1	0.1157
12147	BMLF1	BCL2A1	0.41721
12148	EBNA3	FIP1L1	0.17902
12149	BGLF4	MSX2	0.024406
12150	EBNA1	ODF2	0.04974
12151	BMLF1	CNN2	0.023181
12152	BZLF1	UBQLN1	0.1332
12153	BMLF1	DRD4	0.099666
12154	EBNA-LP	DPM1	0.22357
12155	EBNA-LP	DAZAP2	0.36377
12156	BLLF1	LINC00205	0.03026
12157	EBNA3	AKAP1	0.14015
12158	EBNA-LP	ADAD2	0.027299
12159	BZLF1	MEIS1	0.033964
12160	BMLF1	ARPC2	0.28892
12161	BZLF1	LSM1	0.15624
12162	BGLF4	PCDHGB6	0.058173
12163	BHRF1	C12orf50	0.12154
12164	EBNA3	BID	0.13086
12165	BZLF1	PRDX3	0.070252
12166	BGLF4	SEPT5	0.049882
12167	EBNA-LP	SLC14A2	0.33212
12168	BGLF4	PCGF1	0.14822
12169	BGLF4	TRPM6	0.087983
12170	BMLF1	DAO	0.22754
12171	BGLF4	CYFIP1	0.11916
12172	BZLF1	TNFSF12	0.093423

12173	BBRF2	ZNF33B	0.011463
12174	EBNA3	WDR82	0.32294
12175	EBNA3	ELOVL4	0.26256
12176	EBNA-LP	MYLK2	0.07519
12177	BZLF1	BABAM1	0.17204
12178	EBNA1	AATK	0.11302
12179	BMLF1	EIF4E2	0.42369
12180	EBNA3	RECQL	0.20319
12181	EBNA1	AKT3	0.013897
12182	BZLF1	MICU1	0.35589
12183	LMP2	S100B	0.042203
12184	BPLF1	UCKL1	0.0424
12185	BGRF1/BDRF1	S100A4	0.20694
12186	EBNA1	MYO6	0.066099
12187	BARF1	GSN	0.084747
12188	BGLF4	ARF4	0.12665
12189	BLLF2	CPSF1	0.011646
12190	BNLF2b	VWA3A	0.049586
12191	EBNA3	FBXO4	0.30022
12192	BLLF2	OR5L1	0.075258
12193	BGLF4	FGF2	0.022457
12194	BOLF1	S100A13	0.14799
12195	BMLF1	GRB10	0.23182
12196	BZLF2	MIR7-3HG	0.017539
12197	EBNA4	MAD1L1	0.17066
12198	BZLF1	PIGS	0.15757
12199	BLLF2	ZNF682	0.013755
12200	BMLF1	NNMT	0.045821
12201	EBNA1	MAGEA1	0.14306
12202	BBRF2	ARMCX4	0.064168
12203	BGLF4	FBXL12	0.22281
12204	BMLF1	C19orf25	0.47224
12205	BZLF1	C6orf223	0.011257
12206	gL	FASN	0.02932
12207	BILF1	C15orf40	0.011321
12208	BMLF1	ARL4D	0.27195
12209	EBNA-LP	CTTN	0.35732
12210	EBNA4	ATP10B	0.023387
12211	BARF1	STAT1	0.025124
12212	BGLF4	CSNK2A2	0.043168
12213	BLLF2	ZNF737	0.014568
12214	BZLF1	PSMA4	0.050508
12215	BGLF4	OPTN	0.16277
12216	BGLF4	APOBEC1	0.089151
12217	EBNA-LP	KCNIP2	0.32762
12218	EBNA3	EPHA10	0.27512
12219	BHRF1	RABGAP1L	0.017278
12220	BLLF2	IPO13	0.011343
12221	BLLF1	ZNF767	0.022975
12222	BMLF1	RAET1E	0.37983
12223	EBNA-LP	FCRL4	0.39356
12224	BMLF1	AKAP1	0.16345
12225	EBNA-LP	KDR	0.044006
12226	BGLF4	GRB10	0.10859
12227	EBNA1	SLC6A4	0.08533
12228	BLLF2	RFX1	0.011414
12229	BZLF1	TAF3	0.098577
12230	BMLF1	STRN3	0.18848
12231	BPLF1	FAM46A	0.061074
12232	BLLF2	VPS29	0.022694
12233	BHRF1	SNX6	0.031352
12234	BGLF4	SCUBE2	0.066109
12235	BGLF4	ADAMTSL2	0.15332
12236	EBNA-LP	CD274	0.30613
12237	EBNA1	MICALL1	0.16545
12238	EBNA3	RNF31	0.20427
12239	BHRF1	HIN1L	0.010307
12240	EBNA1	HAX1	0.17032

12241	EBNA3	ARL4A	0.29523
12242	EBNA3	CENPH	0.20053
12243	EBNA3	PHYHIP	0.024369
12244	EBNA1	SNAPIN	0.13501
12245	BGLF4	KAT2B	0.033007
12246	EBNA-LP	AIP	0.1319
12247	BZLF1	RAMP1	0.092214
12248	BGRF1/BDRF1	SNX17	0.011257
12249	EBNA-LP	HADHA	0.14304
12250	LMP1	LRP4	0.040335
12251	EBNA6	YTHDF3	0.16443
12252	EBNA3	FAM69C	0.21448
12253	gN	CALR	0.013823
12254	EBNA6	KRI1	0.23179
12255	EBNA1	IGSF1	0.16367
12256	EBNA-LP	MFI2	0.25421
12257	EBNA3	FOXC1	0.019705
12258	EBNA-LP	GLRX3	0.012817
12259	EBNA3	MAZ	0.28775
12260	BMLF1	RPL30	0.04147
12261	EBNA1	SHANK2	0.19839
12262	EBNA-LP	EIF4E	0.2689
12263	BGLF4	BMI1	0.06746
12264	EBNA-LP	NOS2	0.10854
12265	BLLF1	CXorf67	0.06555
12266	BARF1	RNH1	0.19436
12267	BMLF1	FBXO11	0.24364
12268	BZLF1	KCNS1	0.1434
12269	EBNA3	FAR2	0.010042
12270	BZLF1	STXBP4	0.024985
12271	EBNA-LP	SOX13	0.18804
12272	BMLF1	BIN2	0.45783
12273	BRRF1	PDCD6	0.23287
12274	BMLF1	IQGAP3	0.031711
12275	BGLF4	PADI1	0.038208
12276	BMLF1	MC3R	0.28014
12277	EBNA3	RPL14	0.26519
12278	BZLF1	SLC34A1	0.18581
12279	BZLF1	MST4	0.031629
12280	BZLF2	C19orf66	0.04443
12281	EBNA4	IL12A	0.11222
12282	EBNA3	SUPT20H	0.35329
12283	BGLF4	SMC6	0.13881
12284	BGLF4	RASSF2	0.19585
12285	gL	UHRF1BP1L	0.12428
12286	EBNA-LP	RPS19	0.21747
12287	EBNA1	PRRG2	0.039928
12288	BGLF4	SH3RF2	0.14805
12289	EBNA6	PAGE5	0.125
12290	BGLF4	C19orf57	0.22411
12291	EBNA3	XPO4	0.35162
12292	BMLF1	PTPRJ	0.081851
12293	BMLF1	MED4	0.25776
12294	EBNA-LP	SUSD1	0.19742
12295	BPLF1	TIFAB	0.13603
12296	BGLF4	AKR1B10	0.20278
12297	BGLF4	PCDHGA10	0.058173
12298	EBNA-LP	PTPRS	0.13668
12299	EBNA-LP	RNF11	0.24274
12300	EBNA1	ASCC3	0.097791
12301	EBNA3	DPPA4	0.32035
12302	BGLF4	LEMD3	0.12332
12303	BXRF1	GAS6	0.011729
12304	EBNA3	MYO18A	0.28098
12305	EBNA-LP	FOXD3	0.052152
12306	BLLF2	NCR3	0.04106
12307	EBNA1	ZYX	0.10094
12308	BLLF1	NOTCH4	0.018239

BILF1	PLSCR1	0.082139
EBNA3	NOSIP	0.26078
BZLF1	HOMER3	0.1565
BGLF4	SEC24C	0.065602
BZLF1	CD82	0.20871
BLLF1	C9orf171	0.030334
BGLF4	RNF144B	0.12285
EBNA6	YMR001C-A	0.016254
EBNA1	HSPB2	0.15859
EBNA-LP	PRF1	0.37776
BNLF2b	DTNBP1	0.022569
EBNA1	ZNF646	0.10783
BZLF1	SELPLG	0.13063
BMLF1	TRAF1	0.31298
EBNA-LP	DBNDD2	0.40592
EBNA6	GRAMD3	0.011669
BLLF2	TSTD1	0.02067
BGLF4	TNKS	0.064318
LMP1	NID2	0.091531
EBNA-LP	PVRL3	0.27505
gB	ATP2A1	0.018089
BZLF1	ANK3	0.079291
BZLF1	SPG7	0.052012
EBNA1	ARF1	0.047359
BGLF4	SLC25A12	0.035877
EBNA3	MYLIP	0.19388
EBNA3	NAA50	0.45042
BVRF1	PITPNM3	0.1439
BGLF4	LRPPRC	0.15936
BGLF4	ARHGAP32	0.08528
EBNA1	CASK	0.067436
BMLF1	SLC5A1	0.23953
gN	REPS1	0.093044
EBNA1	DDO	0.1284
BGLF4	BET1	0.13377
EBNA2	RAB11FIP3	0.032225
EBNA3	S100A4	0.62787
BLLF2	ZSCAN5C	0.028125
BZLF1	PRMT2	0.14773
BGLF4	GPR37L1	0.17836
EBNA1	LMNA	0.079581
BMLF1	CLVS2	0.091118
BOLF1	ANXA2	0.090348
BMLF1	CALR3	0.17531
EBNA6	RQCD1	0.1414
BGLF4	CDC26	0.15498
EBNA3	ARHGAP22	0.143
BLLF1	DYDC2	0.012591
EBNA-LP	PRKDC	0.074907
BRRF1	RAB11FIP4	0.28792
BGLF4	TAF13	0.056334
EBNA1	PLS1	0.039716
BMLF1	CDK5RAP3	0.29005
BLLF2	CD2	0.012507
EBNA3	SMARCA1	0.16006
EBNA3	RNF7	0.25117
BGLF4	PCDHA7	0.05466
EBNA4	ZNF326	0.16644
BGLF4	CNEP1R1	0.13304
BMLF1	SLC40A1	0.22556
EBNA-LP	KBTBD7	0.39536
BMLF1	SAP18	0.29147
BZLF1	ARHGAP10	0.06269
BZLF1	ATP6V1D	0.13839
BGLF4	XRCC1	0.15613
EBNA6	FAM229B	0.15347
EBNA-LP	PCDHGA10	0.17939
BZLF1	TRAK1	0.13006

12377	BHRF1	BTBD16	0.014271
12378	BDLF1	NID2	0.046074
12379	EBNA-LP	PLAUR	0.17687
12380	BGLF4	SNX7	0.026854
12381	EBNA3	MOCS3	0.20238
12382	BZLF1	PILRB	0.2023
12383	BMLF1	INSR	0.030271
12384	BLLF1	FBN1	0.11997
12385	EBNA3	SLC6A17	0.027831
12386	LMP2	PKD2L1	0.050675
12387	BLLF2	SLC16A13	0.060205
12388	BLLF2	RINT1	0.052792
12389	gN	THBS1	0.012641
12390	BMLF1	AIMP1	0.22491
12391	EBNA4	ZNRD1-AS1	0.056025
12392	BZLF1	PRKRA	0.16738
12393	EBNA4	AXDND1	0.013376
12394	BZLF1	SPINK7	0.20902
12395	EBNA3	RASA1	0.10663
12396	BLLF2	ZNF665	0.013362
12397	BLLF2	NHEJ1	0.018932
12398	EBNA3	PACSIN2	0.010853
12399	BHRF1	IQCF3	0.020702
12400	EBNA3	MIMI	0.10939
12401	BMLF1	EFCAB1	0.26298
12402	EBNA1	NLRX1	0.094987
12403	BMLF1	WSB1	0.42135
12404	BMLF1	DTX1	0.20291
12405	BLLF2	DMRT1	0.016701
12406	EBNA1	KLHL12	0.23329
12407	BGLF4	GNAI2	0.048646
12408	BMLF1	C2orf71	0.011469
12409	EBNA-LP	GEMIN7	0.27883
12410	BTRF1	N4BP3	0.010793
12411	BMLF1	RMI1	0.41616
12412	EBNA6	ATG4B	0.12954
12413	BLLF2	ZBTB40	0.015498
12414	BLLF2	NXT1	0.030814
12415	EBNA3	CATSPER1	0.21417
12416	EBNA3	MACF1	0.3904
12417	EBNA1	SSX2	0.15249
12418	BLLF1	C6orf222	0.012723
12419	BZLF1	AOC1	0.041002
12420	EBNA1	DAB2	0.073817
12421	EBNA3	CALU	0.54894
12422	BLLF1	UPF3B	0.048875
12423	EBNA-LP	VCP	0.11064
12424	EBNA3	NOP58	0.3014
12425	EBNA1	RPS6KA1	0.019816
12426	BZLF1	NKD2	0.32562
12427	BGLF4	KRT81	0.17791
12428	EBNA3	LCP2	0.33542
12429	BOLF1	PPEF2	0.077969
12430	BARF1	UTRN	0.084772
12431	EBNA1	ATP6V1D	0.12917
12432	BMLF1	SUV420H1	0.056088
12433	EBNA-LP	RNF111	0.15758
12434	EBNA-LP	CTU2	0.35924
12435	EBNA-LP	LILRA4	0.40592
12436	EBNA-LP	FGF21	0.33092
12437	BMLF1	APOA4	0.011807
12438	EBNA-LP	CENPE	0.13468
12439	EBNA6	TTYH1	0.20483
12440	EBNA3	DYNLT1	0.18333
12441	EBNA-LP	SNRPB	0.19998
12442	BGLF4	PSMB1	0.05682
12443	BLLF2	TNRC6C	0.046136
12444	EBNA6	SHANK2-AS3	0.071066

12445	BOLF1	C1orf145		0.011257
12446	BMLF1	IGFBP7	0.3634	
12447	EBNA4	C1orf196		0.11553
12448	EBNA3	KIRREL3	0.35784	
12449	BZLF1	ERCC5	0.089962	
12450	EBNA6	AP5B1	0.11963	
12451	EBNA4	MSANTD2	0.035045	
12452	EBNA3	PCBP1	0.34027	
12453	EBNA3	SNTB1	0.22093	
12454	BGLF4	PITPNM3	0.34682	
12455	BMLF1	SRY	0.17766	
12456	BMLF1	DCAF8	0.4511	
12457	BZLF1	CENPJ	0.072007	
12458	EBNA3	RP1	0.21006	
12459	EBNA4	SOGA2	0.010757	
12460	EBNA3	SCIMP	0.33612	
12461	BILF1	PITPNM3	0.16343	
12462	BLLF1	PITPNM3	0.090117	
12463	BGLF4	BLOC1S6	0.094175	
12464	EBNA-LP	HRAS	0.052285	
12465	BMLF1	ANXA2R	0.09211	
12466	BMLF1	MOCS3	0.22579	
12467	EBNA6	EHD1	0.057457	
12468	BRRF1	PPP2R3B	0.1087	
12469	EBNA4	CLUAP1	0.31649	
12470	EBNA-LP	GMIP	0.27562	
12471	EBNA-LP	RTN2	0.32525	
12472	EBNA1	ACTL6A	0.086023	
12473	BGLF4	PRMT6	0.13906	
12474	EBNA-LP	CDHR1	0.13856	
12475	BMLF1	CD302	0.018918	
12476	BPLF1	CDKN2AIPNL		0.11424
12477	BLLF1	REPS2	0.12512	
12478	BMLF1	MTO1	0.045982	
12479	EBNA4	TTI1	0.19005	
12480	EBNA3	RPL13	0.28237	
12481	BGRF1/BDRF1		CDH1	0.038438
12482	BMLF1	NLGN3	0.1577	
12483	BMLF1	ATP5F1	0.32582	
12484	EBNA3	OTUD6A	0.033529	
12485	BZLF1	TNPO3	0.1377	
12486	BZLF1	CEBPA	0.019404	
12487	EBNA3	VOPP1	0.055112	
12488	BGLF4	BRAP	0.12069	
12489	BGLF4	NAPA	0.13805	
12490	BILF1	DLL1	0.14528	
12491	EBNA4	CTNNBIP1		0.12061
12492	BPLF1	CDH1	0.02839	
12493	BMLF1	PLN	0.28194	
12494	gL	RRP7B	0.011553	
12495	EBNA1	GAB2	0.046488	
12496	EBNA-LP	PELI2	0.27529	
12497	BDLF1	DST	0.046026	
12498	BZLF1	PALM3	0.15847	
12499	EBNA-LP	FAM199X	0.015276	
12500	EBNA3	OTOF	0.15528	
12501	BGLF4	DAZ1	0.20127	
12502	EBNA6	FBRSL1	0.12683	
12503	EBNA1	HM13	0.10832	
12504	BMLF1	AP3M1	0.33016	
12505	EBNA3	TP53BP1	0.15609	
12506	EBNA-LP	DGKZ	0.20922	
12507	BGLF4	EHMT1	0.15757	
12508	BZLF1	ITGB1BP2		0.022877
12509	BLLF2	CRTC2	0.10458	
12510	BGLF4	DNAJB5	0.19799	
12511	EBNA1	CCNC	0.039085	
12512	EBNA3	PTCRA	0.39907	

12513	BMLF1	MAGEE1	0.33845
12514	BRRF1	KAT2A	0.01634
12515	BGLF4	BMPR1B	0.022748
12516	BXRF1	SETD1B	0.012579
12517	BGLF4	GRB2	0.022198
12518	BZLF2	C1orf105	0.063613
12519	EBNA6	TIPRL	0.13275
12520	BZLF1	GCKR	0.12053
12521	BZLF1	CCDC28B	0.22191
12522	BPLF1	YIR021W-A	0.025752
12523	EBNA1	FZD8	0.056858
12524	BZLF1	LYL1	0.13638
12525	BMLF1	SEC11C	0.025687
12526	BGRF1/BDRF1	PIK3CB	0.011407
12527	BMLF1	DPP9	0.071044
12528	BLLF1	CIB1	0.079696
12529	EBNA3	SENP5	0.31765
12530	BILF1	S100B	0.094672
12531	EBNA1	PSMD4	0.095488
12532	BPLF1	C12orf63	0.033663
12533	EBNA3	MMP1	0.050419
12534	BLLF2	ZNF77	0.02203
12535	EBNA1	RFC3	0.084581
12536	EBNA-LP	CEACAM6	0.35589
12537	BMLF1	COCH	0.36512
12538	BZLF1	JAM3	0.11179
12539	EBNA4	FAM149B1	0.16763
12540	BZLF1	PINX1	0.081211
12541	EBNA3	ATP2B4	0.22842
12542	BMLF1	TNRC6B	0.019043
12543	EBNA-LP	DLGAP3	0.28642
12544	BZLF1	PCDHB18	0.049103
12545	BZLF1	EHD1	0.20874
12546	BMLF1	PLAA	0.41711
12547	BGLF4	RNF126	0.20031
12548	EBNA6	LOH12CR1	0.012146
12549	BGLF4	SNRPA	0.18132
12550	BMLF1	ZFYVE9	0.37273
12551	EBNA1	SPATA13	0.042882
12552	EBNA3	TRIM24	0.052211
12553	EBNA3	PDZD3	0.22486
12554	BMLF1	CCPG1	0.046662
12555	EBNA4	NECAB3	0.14746
12556	BNLF2b	ARSB	0.012368
12557	BGLF4	PCDH18	0.056505
12558	BZLF1	MRPL13	0.18431
12559	BMLF1	PRG2	0.038518
12560	BLLF2	PPFIA3	0.01419
12561	BGLF4	OPRD1	0.10896
12562	EBNA3	STX11	0.36724
12563	gL	C20orf62	0.032228
12564	EBNA3	TLE1	0.15491
12565	BPLF1	C22orf26	0.058321
12566	BLLF2	B3GALTL	0.0162
12567	BHRF1	PTGES3L	0.032489
12568	EBNA6	C17orf102	0.098548
12569	EBNA3	BIN1	0.22647
12570	BOLF1	C1orf64	0.042522
12571	gL	TTC23L	0.032739
12572	BMLF1	SCAMP1	0.33024
12573	EBNA-LP	TEN1	0.32281
12574	BZLF1	CANX	0.20092
12575	BZLF1	CDC42EP2	0.13195
12576	BMLF1	EIF3C	0.42145
12577	EBNA3	PAK2	0.029187
12578	EBNA3	SSBP1	0.34043
12579	BZLF1	RAP1B	0.085518
12580	BZLF1	NLRC4	0.073994

12581	BPLF1	LINC00476	0.015768
12582	EBNA-LP	MMP19	0.042126
12583	EBNA-LP	TARDBP	0.17656
12584	EBNA1	ALG2	0.12319
12585	EBNA1	CCDC176	0.050439
12586	BGLF4	POLD4	0.10977
12587	BLLF2	ZNF213	0.028797
12588	BMLF1	TDRKH	0.050618
12589	EBNA3	FOXD3	0.05118
12590	BMLF1	TNFRSF6B	0.40563
12591	EBNA3	SMARCE1	0.21012
12592	EBNA1	NPHP1	0.09253
12593	EBNA-LP	SDC1	0.12582
12594	EBNA6	MATN3	0.19515
12595	EBNA1	CYTIP	0.2066
12596	LMP1	CDH5	0.037613
12597	BMLF1	H2AFX	0.18741
12598	EBNA4	C22orf26	0.1921
12599	EBNA3	SYNJ2	0.22958
12600	BZLF1	ARVCF	0.17419
12601	BZLF2	DTNBP1	0.015092
12602	BLLF2	BEX4	0.05754
12603	EBNA1	SPHKAP	0.20909
12604	BGLF4	FOS	0.018306
12605	EBNA1	ARF5	0.11102
12606	EBNA2	NOTCH3	0.025671
12607	EBNA3	REST	0.015982
12608	BGLF4	SLC6A2	0.2165
12609	BMLF1	BCL2L11	0.081188
12610	BMLF1	PCDHGA6	0.22489
12611	BGLF4	PTPRJ	0.028965
12612	BDLF1	CT55	0.029143
12613	EBNA3	MAP3K5	0.068611
12614	BGLF4	PKD2L2	0.068841
12615	BMLF1	AKR1E2	0.018668
12616	EBNA-LP	ARHGEF4	0.15253
12617	BGLF4	DLG3	0.10101
12618	BMLF1	CCDC28B	0.44364
12619	BARF1	CDHR2	0.19188
12620	BBRF2	NHP2L1	0.017754
12621	EBNA3	TRIAP1	0.33244
12622	EBNA3	KIR2DL4	0.34233
12623	BZLF1	PRPF4	0.17153
12624	LMP2	SIRT4	0.020575
12625	BBRF2	SSBP3	0.010509
12626	EBNA-LP	DVL3	0.18215
12627	BGLF4	SMG5	0.1595
12628	BLLF1	C20orf195	0.015421
12629	BZLF1	MC1R	0.092783
12630	LMP2	SYT1	0.035647
12631	EBNA6	INTS4L2	0.045556
12632	BMLF1	TMEM184C	0.028205
12633	BLLF2	LVP1a	0.010887
12634	BZLF1	PCLO	0.037285
12635	BZLF1	RHNO1	0.18707
12636	BZLF1	SNRNP40	0.15403
12637	BGLF4	TNFRSF12A	0.14169
12638	BARF1	PPP2R3B	0.10674
12639	BGLF4	PPP1R8	0.1632
12640	EBNA1	IST1	0.27783
12641	BMLF1	FDXR	0.293
12642	EBNA3	RFC3	0.20156
12643	BHRF1	CCDC152	0.020648
12644	gB	LTBP1	0.074685
12645	EBNA3	RIPK1	0.052152
12646	BVRF1	SNX17	0.013856
12647	EBNA-LP	STRN	0.2648
12648	BZLF1	MYO18A	0.13702

12649	BMLF1	GSE1	0.47224
12650	EBNA4	YTHDF3	0.20935
12651	EBNA4	TXLNA	0.095438
12652	BZLF1	SAE1	0.054099
12653	EBNA3	SH3GL3	0.33822
12654	EBNA3	TBC1D24	0.31766
12655	BLLF2	PDCD4	0.058195
12656	BZLF1	VIPAS39	0.17539
12657	EBNA1	CDH8	0.036654
12658	EBNA-LP	EPHA7	0.15673
12659	EBNA-LP	PCDHA7	0.16848
12660	EBNA-LP	BAZ1B	0.17571
12661	BMLF1	MCIDAS	0.26045
12662	BLLF2	CDKN2AIP	0.056943
12663	BMLF1	SKI	0.063316
12664	BBRF2	BAG1	0.017542
12665	BMLF1	PRSS21	0.043385
12666	BZLF1	SUN1	0.11645
12667	EBNA3	RPS6KC1	0.22268
12668	gL	FAT1	0.020353
12669	BPLF1	C9orf171	0.085466
12670	EBNA1	MCIDAS	0.10087
12671	BGLF4	SPTA1	0.19227
12672	BMLF1	SCAF11	0.31729
12673	BRRF1	KCNIP1	0.14962
12674	EBNA3	RAD23A	0.25646
12675	EBNA4	CALM1	0.095142
12676	BZLF2	FAM161B	0.097965
12677	EBNA-LP	ZNF326	0.30484
12678	BMLF1	MOB1B	0.29636
12679	BMLF1	RAD23B	0.25743
12680	EBNA3	CHMP3	0.24239
12681	EBNA1	TRAIP	0.12279
12682	EBNA2	EDEM1	0.06118
12683	EBNA1	MUC2	0.202
12684	EBNA3	NR0B1	0.078513
12685	BGLF4	PAX8	0.023287
12686	EBNA-LP	WWP2	0.12185
12687	BGLF4	TSHR	0.14777
12688	BGLF4	PLD2	0.032848
12689	BZLF1	TXLNB	0.22209
12690	EBNA4	NOL3	0.23384
12691	BLLF2	POLR2H	0.051531
12692	EBNA-LP	LCAT	0.24543
12693	EBNA-LP	DSP	0.14524
12694	gN	F10	0.050265
12695	EBNA3	WSB1	0.39195
12696	BOLF1	EFCAB4B	0.12725
12697	EBNA3	COL3A1	0.17267
12698	BMLF1	RP1L1	0.36001
12699	EBNA3	PPEF1	0.53303
12700	gL	FAM27D1	0.093262
12701	BZLF1	CADM1	0.072721
12702	gL	MAGEB2	0.010737
12703	BGLF4	HNRNPL	0.15159
12704	EBNA6	C6orf52	0.019339
12705	BZLF1	VAPA	0.050027
12706	BGRF1/BDRF1	C1orf64	0.025418
12707	EBNA3	SNX2	0.29215
12708	BMLF1	FHIT	0.23114
12709	BGRF1/BDRF1	FAM47C	0.019611
12710	BMLF1	EFCAB7	0.26298
12711	EBNA-LP	CDH19	0.17394
12712	BMLF1	MED15	0.26141
12713	BLLF2	ZNF343	0.015559
12714	BGLF4	UBE2B	0.054248
12715	BGLF4	NFATC1	0.056712
12716	BMLF1	PPP2R5E	0.33074

12717	BHRF1	FAM229B	0.085326
12718	EBNA3	NCKAP1	0.23018
12719	BMLF1	CAMKK2	0.096681
12720	EBNA1	PSMB9	0.047824
12721	BPLF1	FAM161B	0.1532
12722	BILF1	EIF3B	0.011404
12723	BMLF1	SH3GL2	0.23995
12724	gM	CALM1	0.054385
12725	BMLF1	PCDH15	0.12479
12726	EBNA3	VEGFA	0.012842
12727	BNLF2b	ABHD17B	0.057992
12728	EBNA3	RPS6KA4	0.08115
12729	gM	ANXA6	0.098431
12730	BGLF4	NTRK1	0.030472
12731	EBNA1	CDCA8	0.10894
12732	BMLF1	PARP15	0.034508
12733	BZLF1	ARL13B	0.18382
12734	EBNA-LP	LCK	0.05836
12735	BVRF1	YAE1D1	0.026507
12736	EBNA6	EIF4G1	0.020846
12737	gB	LRP2	0.026395
12738	BGLF4	PRDM5	0.10877
12739	EBNA-LP	S100G	0.19221
12740	EBNA3	SNX22	0.12183
12741	EBNA3	PLAT	0.23088
12742	EBNA3	CAMLG	0.36178
12743	BOLF1	S100A11	0.14114
12744	BZLF2	AMOT	0.021141
12745	EBNA-LP	INTS2	0.31545
12746	BLLF2	SLC16A7	0.034067
12747	BMLF1	KRTAP12-4	0.045197
12748	EBNA1	TRA2B	0.2153
12749	BGLF4	SREBF2	0.04056
12750	BMLF1	NSUN5P2	0.065329
12751	BGLF4	TOLLIP	0.16463
12752	BMLF1	ACO1	0.066908
12753	BMLF1	IGHMBP2	0.095519
12754	EBNA1	MAGEC2	0.17005
12755	BGLF4	GUCA1B	0.067297
12756	EBNA1	GJD3	0.18124
12757	EBNA4	SUV420H2	0.022728
12758	BRRF1	MYL2	0.21675
12759	EBNA-LP	LAMTOR1	0.22489
12760	BGLF4	EDAR	0.13852
12761	EBNA3	VHL	0.14439
12762	BMLF1	POLR1A	0.25071
12763	BZLF1	CCDC92	0.21453
12764	LMP2	VIL1	0.051423
12765	BMLF1	BBS12	0.38634
12766	BMLF1	STAT6	0.26748
12767	BLLF2	ZBTB41	0.02013
12768	LMP2	PRSS2	0.10908
12769	BGLF4	ZP3	0.045772
12770	BPLF1	CT45A5	0.033653
12771	BLLF2	SLC18B1	0.028627
12772	BGLF4	FBXL2	0.20589
12773	BMLF1	EFCAB11	0.26298
12774	BZLF1	ELK1	0.039775
12775	EBNA4	EIF3J	0.13544
12776	EBNA3	FAM188B	0.16057
12777	BOLF1	PPP3CB	0.034211
12778	EBNA-LP	HSP90B1	0.34098
12779	BGLF4	PCDH10	0.058173
12780	EBNA6	MPTX1	0.044754
12781	EBNA3	BUB1B	0.099862
12782	BMLF1	EXOSC3	0.2638
12783	BZLF1	ATF3	0.038466
12784	BMLF1	MICU1	0.49911

12785	EBNA6	CCDC162P	0.036833
12786	BZLF1	CX3CL1	0.056084
12787	BZLF2	WDR96	0.035243
12788	BALF2	MASP2	0.14186
12789	BVRF1	VLDLR	0.06682
12790	BMLF1	ASIP	0.056389
12791	EBNA3	PCDHB14	0.16421
12792	BGLF4	MYLPF	0.054336
12793	BLLF2	TICRR	0.1216
12794	BMLF1	CSTF2T	0.011252
12795	gM	FAM188A	0.075303
12796	BMLF1	SERINC1	0.28843
12797	BXRF1	MAGOH	0.011161
12798	BGLF4	OC90	0.052072
12799	BLLF2	CRLF2	0.031921
12800	EBNA1	PTPN2	0.043045
12801	EBNA3	COPS6	0.31749
12802	gN	NKD2	0.1314
12803	EBNA4	IL17REL	0.069222
12804	BZLF1	RAB33A	0.13625
12805	BGLF4	FBLN5	0.32578
12806	EBNA-LP	SNX8	0.32262
12807	EBNA3	ASAP1	0.099424
12808	BZLF1	BRPF3	0.14704
12809	EBNA3	PGRMC1	0.30374
12810	BZLF2	CNRIP1	0.011304
12811	BHRF1	MASP1	0.18881
12812	BGLF4	BCR	0.055752
12813	LMP2	C10orf82	0.016002
12814	BHRF1	C4orf33	0.022061
12815	BMLF1	WWTR1	0.16753
12816	BZLF1	NOL3	0.1708
12817	EBNA3	MCC	0.29505
12818	BGLF4	PPP6R1	0.18758
12819	BMLF1	SGIP1	0.2181
12820	BMLF1	FCN3	0.33695
12821	LMP1	EGF	0.025077
12822	EBNA4	LINC00299	0.019208
12823	EBNA3	PSMA3	0.096971
12824	BOLF1	N4BP2L1	0.012946
12825	EBNA3	PCBD1	0.15765
12826	EBNA3	MECOM	0.073926
12827	BLLF2	RASIP1	0.011062
12828	BPLF1	PRSS2	0.11107
12829	BLLF2	PSME1	0.060622
12830	BGLF4	RWDD1	0.24432
12831	EBNA4	TMED10	0.011798
12832	BGLF4	COL1A2	0.097604
12833	BHRF1	FAM117B	0.17096
12834	BZLF1	PLEKHA5	0.11694
12835	EBNA1	SNX8	0.17105
12836	gL	C8orf88	0.059697
12837	EBNA-LP	MDFI	0.20923
12838	EBNA4	EID2	0.17408
12839	BARF1	MMP15	0.048678
12840	BGLF4	LYL1	0.18001
12841	EBNA4	CCDC27	0.011488
12842	BPLF1	C10orf67	0.11034
12843	EBNA-LP	ADAM22	0.23439
12844	BBRF2	OAZ3	0.037055
12845	EBNA-LP	DERL2	0.27213
12846	BGLF4	VPS33B	0.13717
12847	EBNA-LP	PXMP4	0.37008
12848	BMLF1	GTF2E2	0.28832
12849	BZLF1	CACNG2	0.12034
12850	BBRF2	CRTC2	0.028742
12851	BMLF1	SLC9A3R2	0.23401
12852	BMLF1	APOD	0.16399

12853	EBNA3	AMFR	0.12575
12854	EBNA-LP	FHOD1	0.27838
12855	BZLF1	TRIM44	0.18327
12856	BILF1	PEF1	0.20553
12857	BVRF1	C10orf82	0.035874
12858	BHRF1	GOLGA6L19	0.10513
12859	EBNA-LP	LOXL2	0.098771
12860	BZLF1	IL1RAPL1	0.10898
12861	EBNA4	FAM188B	0.32663
12862	EBNA3	BEND3	0.011816
12863	BMLF1	ANKEF1	0.26298
12864	EBNA-LP	FZR1	0.21381
12865	EBNA1	RAB24	0.15665
12866	EBNA1	SESTD1	0.15562
12867	EBNA-LP	APOE	0.020021
12868	EBNA-LP	CLPP	0.41489
12869	EBNA3	TXNRD2	0.36903
12870	BPLF1	DHPS	0.053086
12871	BTRF1	IP6K3	0.012896
12872	EBNA3	NR3C2	0.15103
12873	BMLF1	SNIP1	0.30969
12874	EBNA1	PCDHB4	0.029863
12875	EBNA1	IL1RAPL1	0.10205
12876	BGLF4	ERC1	0.10458
12877	BXRF1	SYT11	0.2199
12878	BGLF4	LDHD	0.20779
12879	EBNA-LP	INPP5D	0.12161
12880	BMLF1	PRICKLE3	0.38535
12881	BGLF4	MAPK7	0.033712
12882	EBNA6	SPATA1	0.038522
12883	BLLF2	DAO	0.020745
12884	BLLF2	STARD7	0.020728
12885	BMLF1	VEGFC	0.18167
12886	BMLF1	PADI1	0.20209
12887	EBNA3	GGA2	0.2775
12888	EBNA-LP	CD70	0.23978
12889	EBNA-LP	NUP85	0.16752
12890	EBNA1	PRX	0.1501
12891	EBNA1	GPSM2	0.10262
12892	BZLF1	OPRD1	0.10521
12893	BZLF1	TBC1D10A	0.17396
12894	BLLF2	SEC11C	0.077012
12895	EBNA1	HSF1	0.040137
12896	EBNA3	HDAC6	0.018922
12897	BGLF4	EIF2B1	0.11113
12898	BMLF1	CALN1	0.21218
12899	BGLF4	SGCE	0.047318
12900	BMLF1	PABPC1L2A	0.039667
12901	EBNA-LP	CD53	0.35848
12902	EBNA-LP	LIG3	0.14787
12903	BGRF1/BDRF1	ACTN3	0.1976
12904	BARF1	FAM188A	0.17613
12905	BGLF4	FARSA	0.018734
12906	BRRF1	CELSR3	0.12802
12907	BGLF4	RANGAP1	0.085118
12908	BGRF1/BDRF1	EHD3	0.083784
12909	BGRF1/BDRF1	NBR1	0.021214
12910	BMLF1	SLC25A4	0.17568
12911	EBNA3	ATXN7L3	0.2548
12912	BLLF1	PDGFRB	0.013912
12913	EBNA3	CLDN7	0.20885
12914	BZLF1	POR	0.014626
12915	BMLF1	KISS1R	0.23453
12916	EBNA3	PIAS1	0.1529
12917	EBNA1	BAZ1A	0.089199
12918	EBNA-LP	TICAM1	0.091104
12919	EBNA3	S100A7A	0.17252
12920	BMLF1	KLC3	0.025153

12921	BMLF1	CORIN	0.028055
12922	BLLF1	MATN3	0.13216
12923	BZLF1	SAMHD1	0.14089
12924	EBNA-LP	KRT6A	0.30003
12925	gL	SYT1	0.024349
12926	BLLF2	MORF4L1	0.012463
12927	BMLF1	CYBB	0.1735
12928	BZLF1	BBS7	0.1658
12929	BZLF1	DDOST	0.13831
12930	EBNA-LP	LPL	0.097416
12931	EBNA3	RAD51AP1	0.41246
12932	BZLF1	VCP	0.057312
12933	BLLF2	EFNB1	0.015049
12934	BGLF4	KIF5A	0.11771
12935	EBNA-LP	IRF9	0.091631
12936	BMLF1	GP6	0.32778
12937	BGLF4	MME	0.041065
12938	EBNA-LP	RGS19	0.20738
12939	EBNA-LP	CXorf40A	0.43184
12940	EBNA3	COMMD1	0.25143
12941	BOLF1	JAG2	0.11625
12942	EBNA4	URM1	0.16928
12943	EBNA-LP	DROSHA	0.22678
12944	BLLF2	ZNF829	0.01091
12945	EBNA1	CD200R1	0.17393
12946	BGLF4	ERCC5	0.09689
12947	EBNA3	FAM92A1	0.028692
12948	BZLF1	RYR3	0.024226
12949	BGLF4	CAMK2A	0.034063
12950	BLLF1	TNFAIP8L3	0.011737
12951	BMLF1	CEP152	0.23525
12952	BZLF1	CAPN1	0.28261
12953	EBNA-LP	FBXO25	0.33784
12954	BLLF2	OR2T8	0.07986
12955	BMLF1	CALCA	0.15894
12956	BGLF4	SIN3B	0.14723
12957	EBNA-LP	BCS1L	0.26834
12958	BVRF1	RAB11FIP4	0.23655
12959	EBNA6	CETN2	0.13224
12960	EBNA-LP	NCALD	0.17038
12961	BZLF1	STRN3	0.061766
12962	BGLF4	GYG1	0.16318
12963	BBRF2	ZNF793	0.01038
12964	BGLF4	TRIM29	0.084604
12965	EBNA-LP	WDR48	0.27219
12966	EBNA3	CDH24	0.15838
12967	EBNA-LP	ATR	0.16426
12968	BLLF2	PIGL	0.10527
12969	BGLF4	FADD	0.082019
12970	BXRF1	C1orf195	0.077298
12971	BMLF1	S100A4	0.60064
12972	EBNA-LP	AQP1	0.07321
12973	EBNA1	KANK1	0.10296
12974	EBNA3	STAM2	0.2244
12975	BZLF1	SIRT2	0.031619
12976	BZLF1	PSMA5	0.053906
12977	EBNA6	MRPL11	0.16329
12978	BPLF1	MRFAP1L1	0.11294
12979	EBNA1	PPP6R3	0.17007
12980	BZLF1	NELFA	0.13992
12981	EBNA-LP	MEF2C	0.012095
12982	BBRF2	RABEP2	0.15886
12983	EBNA-LP	MAGEA3	0.43184
12984	EBNA1	ZNRF3	0.10017
12985	EBNA1	TIMM23	0.1476
12986	BGLF4	FAM64A	0.23815
12987	EBNA3	CASQ2	0.13884
12988	BXRF1	FAM188A	0.13448

12989	BMLF1	TWIST2	0.1124
12990	EBNA1	KISS1R	0.10253
12991	BZLF1	PCSK9	0.056464
12992	EBNA1	ICAM3	0.13209
12993	EBNA3	PDLIM4	0.36919
12994	EBNA3	YPR022C	0.12046
12995	EBNA1	UHMK1	0.13396
12996	BHRF1	ENTHD1	0.01414
12997	EBNA3	CT45A5	0.040327
12998	BLLF2	MT1A	0.065486
12999	BMLF1	ACIN1	0.3025
13000	BMLF1	ARHGAP24	0.21484
13001	BGLF4	TPCN1	0.10928
13002	BLLF2	FLOT2	0.012205
13003	EBNA1	PABPC1	0.095961
13004	BMLF1	SHMT1	0.010744
13005	EBNA1	WIPF2	0.20278
13006	EBNA1	BCAS3	0.17682
13007	BZLF1	SPARC	0.17918
13008	BMLF1	GC	0.013607
13009	BNLF2b	SLCO3A1	0.01013
13010	EBNA-LP	BMPR1B	0.084054
13011	BZLF1	HINFP	0.02379
13012	BMLF1	GLS2	0.28771
13013	BVRF1	NKD2	0.17551
13014	BGLF4	RPS6KA1	0.01678
13015	BGLF4	TUBB4A	0.032796
13016	BGLF4	DCAF11	0.23579
13017	BLLF2	ZSCAN2	0.016896
[truncated: 755,599 more chars]
